# A high-quality genome provides insights into the new taxonomic status and genomic characteristics of *Cladopus chinensis* (Podostemaceae)

**DOI:** 10.1038/s41438-020-0269-5

**Published:** 2020-04-01

**Authors:** Ting Xue, Xuehai Zheng, Duo Chen, Limin Liang, Nan Chen, Zhen Huang, Wenfang Fan, Jiannan Chen, Wan Cen, Shuai Chen, Jinmao Zhu, Binghua Chen, Xingtan Zhang, Youqiang Chen

**Affiliations:** 1 Public Service Platform for Industrialization Development Technology of Marine Biological Medicine and Products of the State Oceanic Administration, Fujian Key Laboratory of Special Marine Bioresource Sustainable Utilization, Key Laboratory of Developmental and Neural Biology, College of Life Sciences, Fujian Normal University, Fuzhou, China; 2 Center of Engineering Technology Research for Microalga Germplasm Improvement of Fujian, Southern Institute of Oceanography, Fujian Normal University, Fuzhou, China; 3 College of Fine Arts, Fujian Normal University, Fuzhou, China; 4 FAFU and UIUC-SIB Joint Center for Genomics and Biotechnology, Fujian Provincial Key Laboratory of Haixia Applied Plant Systems Biology, Key Laboratory of Genetics, Breeding and Multiple Utilization of Crops, Ministry of Education, Fujian Agriculture and Forestry University, Fuzhou, China

**Keywords:** High-throughput screening, Bioinformatics, Genomic analysis

## Abstract

The Podostemaceae are ecologically and morphologically unusual aquatic angiosperms that survive only in rivers with pristine hydrology and high water quality and are at a relatively high risk of extinction. The taxonomic status of Podostemaceae has always been controversial. Here, we report the first high-quality genome assembly for *Cladopus chinensis* of Podostemaceae, obtained by incorporating Hi-C, Illumina and PacBio sequencing. We generated an 827.92 Mb genome with a contig N50 of 1.42 Mb and 27,370 annotated protein-coding genes. The assembled genome size was close to the estimated size, and 659.42 Mb of the assembly was assigned to 29 superscaffolds (scaffold N50 21.22 Mb). A total of 59.20% repetitive sequences were identified, among which long terminal repeats (LTRs) were the most abundant class (28.97% of the genome). Genome evolution analysis suggested that the divergence time of *Cladopus chinensis* (106 Mya) was earlier than that of Malpighiales (82 Mya) and that this taxon diverged into an independent branch of Podestemales. A recent whole-genome duplication (WGD) event occurred 4.43 million years ago. Comparative genomic analysis revealed that the expansion and contraction of oxidative phosphorylation, photosynthesis and isoflavonoid metabolism genes in *Cladopus chinensis* are probably related to the genomic characteristics of this growing submerged species. Transcriptome analysis revealed that upregulated genes in the shoot group compared to the root group were enriched in the NAC gene family and transcription factors associated with shoot development and defense responses, including WUSCHEL (WUS), ASYMMETRIC LEAVES (ASL), SHOOT MERISTEMLESS (STM), NAC2, NAC8, NAC29, NAC47, NAC73, NAC83 and NAC102. These findings provide new insights into the genomic diversity of unusual aquatic angiosperms and serve as a valuable reference for the taxonomic status and unusual shoot apical meristem of Podostemaceae.

## Introduction

Podostemaceae are freshwater hydrophytes whose center of diversity is the Neotropics. In the Amazon, these species form subaquatic meadows producing autochthonous carbon, which serve as a source of food for the associated fauna^1^. The Amazon is the richest region of Podostemaceae occurrence^2^. These plants require rivers with pristine hydrology and good water quality, which are very important characteristics for their productivity and for maintaining the associated fauna, and their destruction can lead to ecological and economic losses as well as the loss of cultural and hedonic value^3,4^.

Podostemaceae is a morphologically and esthetically aquatic angiosperm family distributed in the rivers of tropical, subtropical and temperate regions^5,6^. Angiosperms normally exhibit a common system of gravitropism (the growth movement of organs in response to gravity). The roots grow downward into the soil, and the shoots upward in this gravitropism system. The plant meristems, including the root apical meristem (RAM) and shoot apical meristem (SAM), are unique structures of undifferentiated pluripotent stem cells^7^. However, Podostemaceae, Streptocarpus and Lemnaceae exhibit unusual modifications of the SAM that may reflect adaptation to new habitats and can subsequently diversify in the evolution of plants^8^.

Several studies have focused on the establishment of the SAM, including the auxin-related gene activities, transcription factors and morphological characteristics involved^9–13^. A low auxin level contributes to the initial establishment of the apical-basal axis that extends to form the apical meristems of the shoots^9^. The monopteros (MP) gene encodes an auxin response factor whose activity as a transcriptional activator facilitates auxin flow by promoting vascular development. MP expression is relatively weak in auxin-deficient conditions^10^. WUSCHEL (WUS) is a homeodomain transcription factor whose expression establishes a de novo stem cell population^11^. SHOOT MERISTEMLESS (STM) encodes a MEINOX/three-amino acid loop extension (TALE)-HD-type transcription factor required for the initiation and maintenance of the SAM^12^. Hamada et al. reported that mutations of the WUS gene in *Arabidopsis thaliana* halt the entire process of SAM formation and result in no production of juvenile leaves^13^. Based on these studies, the SAM is deemed vital for the vegetative growth of the stem and produces determinate lateral leaves with shoot branches at their axils, which is associated with the expression of key regulatory genes in the evolution of plant organization.

Podostemaceae species grow submerged on rock surfaces in rapids and waterfalls during the rainy season and bear flowers above the water during the dry season, when the water level decreases^14^. Studies involving large amounts of data have mainly been concentrated in certain geographical regions and focused on the molecular phylogenetic analysis of Podostemaceae among other angiosperms, including the evaluation of the phylogeny, classification, and biogeography of Podostemaceae as a whole^15,16^. However, little is known about the evolution of the distinct shoot and root systems of Podostemaceae. Katayama et al. examined the expression patterns of the STM, WUS and asymmetric leaves (ASL) genes related to shoot development in Tristichoideae and Podostemoideae^8^. The subfamily Tristichoideae shows typical shoot organogenesis with a tunica-corpus SAM that produced leaves, but Podostemoideae is devoid of SAM, and new leaves in these plants develop below the older leaves without a SAM structure. Katayama et al. found that the leaves or bracts of Podostemoideae were involved in SAM initiation and maintenance and differentiated into single apical leaves or bracts, resulting in the evolution of mixed shoot-leaf organs in Podostemaceae according to the analysis of phylogenetic and expression patterns^8^. However, questions remain regarding how the unusual modification of the SAM arose during the evolution of Podostemaceae and what key regulatory genes are involved in the evolution of adaptation to the environment. The reasons for these phenomena are not clear. Furthermore, genomic information for Podostemaceae species has not been previously reported.

**Fig. 1:**
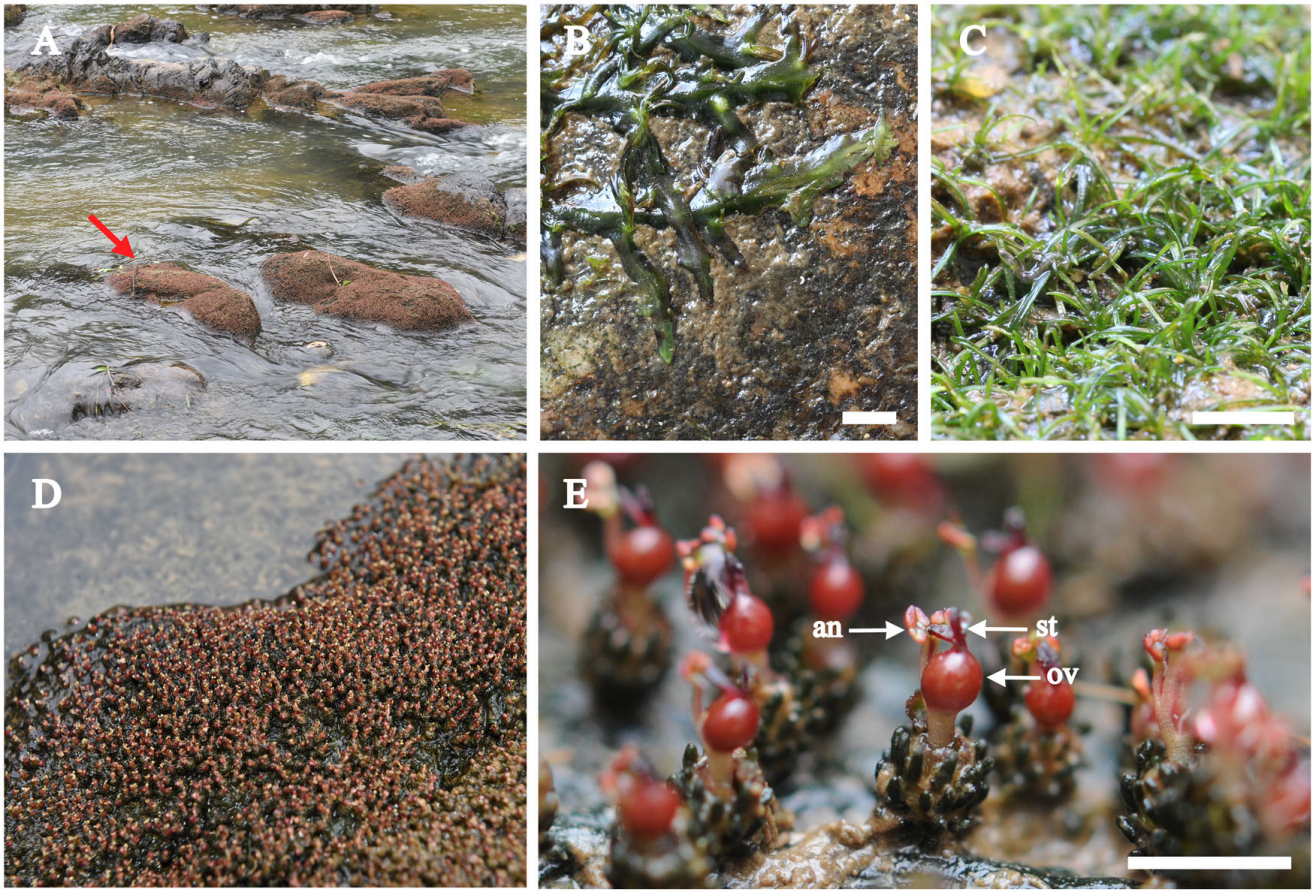
*Cladopus chinensis*. **a** Flowering plants in the habitat of the species. **b** Roots. **c** Leaves on sterile stems. **d** Flowering plants. **e** Flowering shoots (an-anther; ov-ovary; st-style). Bar = 3 mm

*Cladopus chinensis* (*C. chinensis*) is a species of the genus *Cladopus* that exhibits flattened roots creeping on rock surfaces, and leaves and flowers are produced from the roots (Fig. 1). *C. chinensis* can be used as an excellent material for studying the evolution of angiosperm morphology due to its extraordinary aerial shoots and underground root system^17^. Because of the limited genomic information available, an in-depth investigation of the genetic basis of *C. chinensis* is still lacking.

In this study, to systematically understand the evolution of organogenesis in *C. chinensis*, we performed third-generation sequencing (TGS) on the PacBio SEQUEL platform and Hi-C technology to generate a high-quality genome assembly and annotation of *C. chinensis*. We also performed transcriptome sequencing to identify the genes that are involved in the diversity of SAM establishment. These results provide insight into the evolutionary model underlying shoot novelty in Podostemaceae.

## Results

### Genome sequencing and quality assembly

**Fig. 2:**
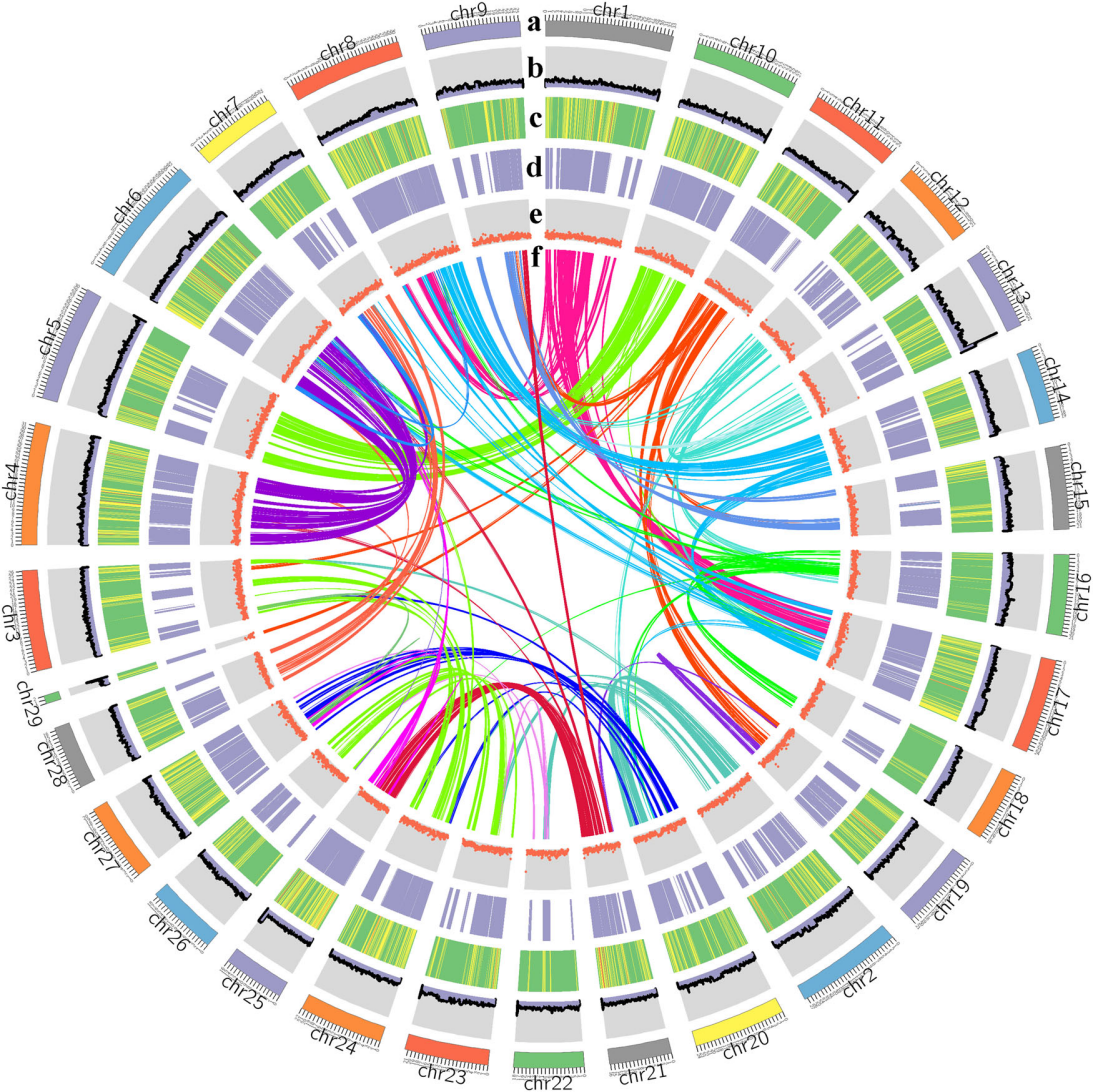
Characterization of the elements in the superscaffold of the *C. chinensis* genome. **a** The 29 assembled superscaffolds of the genome. **b** Distribution of GC content in the genome. **c** Distribution of gene density within sliding windows of 1 Mb. Higher density is shown in green. **d** Expression values of root- and shoot-expressed genes. **e** Percent coverage of TEs in nonoverlapping windows. **f** Schematic presentation of major interchromosomal relationships in the *C. chinensis* genome. Each line represents a syntenic block; block size = 3 kb

**Fig. 3:**
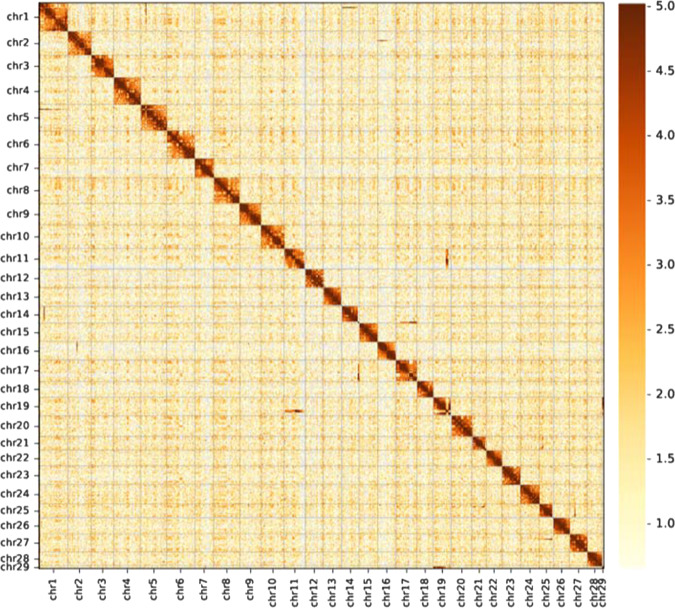
*C. chinensis* genome-wide all-by-all Hi-C interaction heat map. The map shows high-resolution individual superscaffolds, which were scaffolded and assembled independently

We estimated a genome size of 820 Mb for *C. chinensis*, with heterozygosity of 0.73% and a repeat content of 51.0% (Fig. S1). A total of 95.36 Gb of PacBio long reads (~105X coverage of the genome) and 106.2 Gb of Illumina clean reads (~118X coverage of the genome) were generated, resulting in approximately 224.2-fold coverage of the *C. chinensis* genome (Table 1). The total size of all reads assembled from the *C. chinensis* genome was 827.92 Mb, consisting of 5629 contigs. The contig N50 was 1.42 Mb, and the longest contig was 8.89 Mb (Fig. 2 and Table 2). The genome size was close to the results based on flow cytometry (Fig. S2) and genome surveys (Fig. S1). The assembly quality was assessed through BUSCO analysis and the alignment of Illumina short reads to the genome. The NGS short reads from the Illumina sequencing platform were mapped to the contigs by using Bowtie2 software, and approximately 99.78% of the Illumina resequencing reads were mapped to the assembly (Table S1). BUSCO analysis revealed that the assembly completeness was 90.7% in the *C. chinensis* genome (Table S2). Using the contact matrix and the agglomerative hierarchical clustering method in LACHESIS^18^, the 538 contigs were successfully clustered into 29 superscaffolds (Fig. 3; Table S3). The scaffold N50 reached 1.42 and 21.22 Mb (Table 2), providing the first high-quality genome assembly for *C. chinensis*. Moreover, a total of 836 syntenic blocks were detected in the *C. chinensis* genome, which involved 15,410 genes. Synteny analysis of the 29 superscaffolds of *C. chinensis* confirmed that superscaffold no. 4 shared the most syntenic blocks with superscaffold no. 6 (Fig. 2).

**Table 1:** Sequencing data used for *C. chinensis* genome construction

Library resource	Sequencing platform	Insert size (bp)	Clean data (Gb)	Sequence coverage (X)	Use of the data
Genome	Illumina HiSeq X Ten	250 bp	106.2	118	Genome estimation and polishing
Genome	PacBio SEQUEL	20 kb	95.36	105	Genome assembly
Hi-C	Illumina HiSeq X Ten	250 bp	96.4	107	Chromosome construction
Transcriptome	PacBio SEQUEL	0.6–3 kb	77.7	—	Difference analysis and annotation

Note that the sequence coverage was calculated using the K-mer-based estimate of genome size

**Table 2:** Assembly statistics for the *C. chinensis* genome

Items	Canu		Hi-C	
	Contig_len(Mb)	Contig_number	Scaffold_len(Mb)	Scaffold_number
Total	827.92	5629	827.92	4929
Max	8.89	—	33.49	—
Number ≥ 2 kb	—	5387	—	4737
N50	1.42	184	21.22	18

### Gene prediction and functional annotation

A combination of reference plant protein homology support, transcriptome data, and *ab initio* gene prediction was used to generate all gene models. All gene models were merged, and redundancy was removed with MAKER, leading to a total of 27,370 protein-coding genes. The average transcript length was 1465 bp, and the average CDS length was 868 bp (Table 3). We functionally annotated 22,564, 14,193, 22,564 and 11,950 genes to the eggNOG, GO, COG and KEGG databases, respectively, leading to 23,163 (84.63% of the total) genes showing at least one hit to the public databases (Table 3). In total, 2156 transcription factors were identified in the *C. chinensis* genome, and these genes were classified into 28 families, including 705 protein kinase family, 459 PPR, 155 MYB and 123 bHLH superfamily proteins (Table S4).

**Table 3:** Annotation statistics for the *C. chinensis* genome

Annotation statistics for the genome	Number	Percent (%)
Total protein	27,370	
eggNOG	22,564	82.44
GO	14,193	51.86
COG	22,564	82.44
KEGG	11,950	43.66
In all databases	8549	31.23
In at least one database	23,163	84.63

### Annotation of noncoding RNAs (ncRNAs)

We identified snRNA, miRNA and rRNA genes in the *C. chinensis* genome from the Rfam database using BLASTN software (E-value ≤ 1e-5), and we used tRNAscan-SE and RNAmmer to predict tRNAs and rRNAs, resulting in a *C. chinensis* genome with 79 miRNAs, 1997 tRNAs, 397 rRNAs, 116 sRNAs, and 128 snRNAs (Table S5).

### Repeat element (TE) annotation and burst analysis

The *C. chinensis* genome contained 490.13 Mb of repetitive sequences, accounting for 59.20% of the genome (Table S6). Long terminal repeat (LTR) retrotransposons accounted for 28.97% of the genome, with 22.30% Ty1/copia and 0.72% Ty3/gypsy sequences. Kimura distance analysis indicated an LTR burst (Fig. S3) involving the Ty1/copia and Ty3/gypsy superfamilies. Tandem Repeats Finder identified over 50,071 tandem repeats, accounting for 7.40% of the *C. chinensis* genome (Table S6). Telomeric and centromeric repeats were identified by searching the ends of contigs, and 8 telomeric repeat sequences and 572 centromeric repeat sequences were identified (Tables S7, S8).

### Evolution of the *C. chinensis* genome

**Fig. 4:**
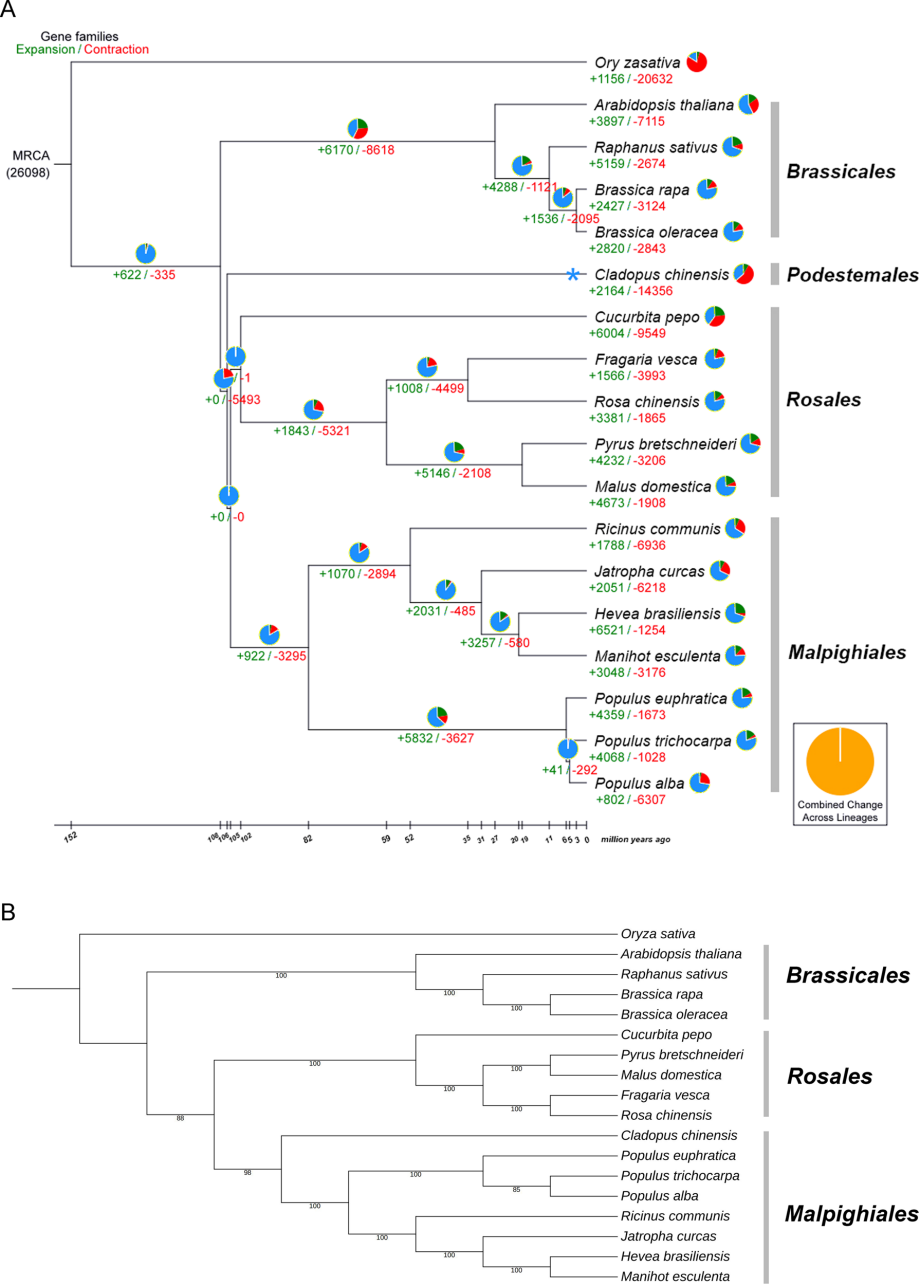
Phylogenetic tree and evolution analysis of *C. chinensis*. **a** Phylogenetic tree showing the number of gene families displaying expansion (green) and contraction (red) among 18 plant species. The pie charts show the proportions of expanded (green), contracted (red) and conserved (blue) gene family among all gene families. The estimated divergence time (million years ago) is displayed below the phylogenetic tree in black. *The time of the occurrence of a recent whole-genome duplication (WGD) event in *C. chinensis*. MRCA, most recent common ancestor. **b** The species tree of 18 species inferred from the complete dataset of single-copy homologous gene using concatenation (RAxML) and gene-tree-based coalescent (ASTRAL) methods

**Fig. 5:**
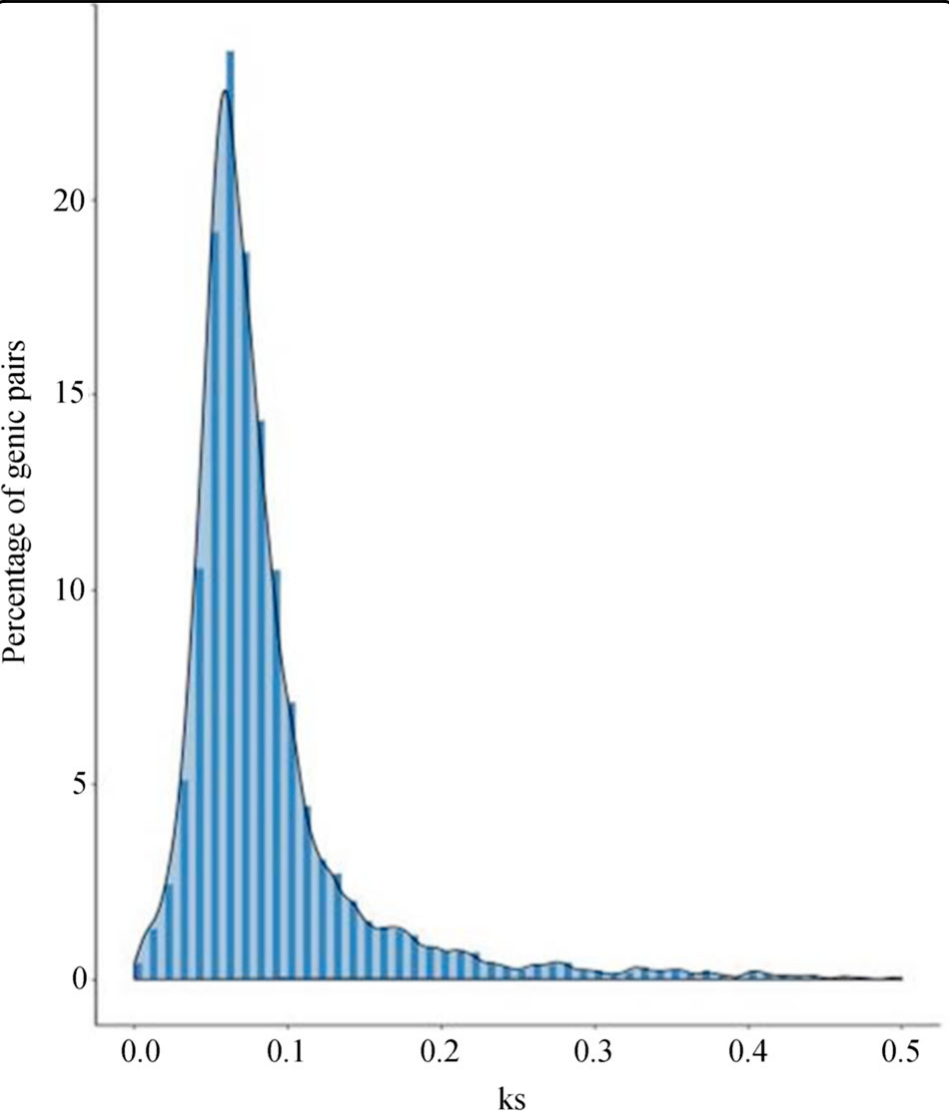
*K*s distributions for duplicated gene pairs in *C. chinensis*

We collected the genome sequences of representative plant species and performed comparative genomic analysis with *C. chinensis* to reveal the genome evolution and divergence time of *C. chinensis*. The results suggested that gene family contractions outnumbered expansions in *C. chinensis*, *R. communis*, *O. sativa*, *P. alba*, *B. oleracea*, *B. rapa*, *J. curcas*, *M. esculenta*, *A. thaliana*, *C. pepo* and *F. vesca*, in contrast to the other seven species (Fig. 4a). The results support the view that Malpighiales and Rosales share a common Rosidae ancestor, which is the basal taxon of dicotyledons. The phylogenetic tree showed that *C. chinensis* phylogenetically diverged into an independent branch approximately 106 million years ago (Mya), without clustering into Malpighiales after the divergence of the *A. thaliana* (Brassicales) lineage 108 Mya. The data further confirmed that the divergence time of *C. chinensis* (106 Mya) was earlier than those of Malpighiales (82 Mya) and Rosales (102 Mya) and later than that of Brassicales (108 Mya). We also found that the internal branches separating Brassicales, Podestemales, Rosales, and Malpighiales were very short, as shown in Fig. 4a, suggesting the rapid diversification of these major clades. To obtain a highly reliable phylogenetic tree, we identified 91 single-copy homologous genes and performed coalescent analysis with RAxML and ASTRAL to estimate the species tree from gene trees. This result showed that *C. chinensis* phylogenetically clusters into Malpighiales, which is consistent with the results from NCBI Taxonomy (Fig. 4b). On the basis of the number of transversions at four-fold degenerate sites, we calculated an age distribution for all duplicate gene pairs. Using 8,074 paralogous gene pairs of similar ages and excluding tandem or local duplications, a large peak centered on a synonymous substitution rate (*K*_s_) of approximately 0.14 was observed in the *C. chinensis* genome (Fig. 5), indicating only one recent whole-genome duplication (WGD) event and one ancient WGD that occurred 4.43 million years ago.

### Gene family analysis

We performed comparative genomic analyses among 18 plant species and detected 81,585 families of homologous genes. A total of 17,964 gene families were identified in *C. chinensis*, among which 2164 and 14,356 gene families showed expansion and contraction, respectively (Fig. 4). A total of 2164 expanded gene families were annotated to KEGG pathways (Table S9) and GO terms (Table S10). KEGG analysis showed that most of the expanded genes were clustered in the categories of energy metabolism, signal transduction and aging. GO analysis showed that the expanded orthogroups were related to metabolic process and cellular component categories and the stimulus response, cysteine synthase, phototropism and sugar carrier terms. A total of 14,356 of the contracted gene families were involved in carbohydrate metabolism, signal transduction, secondary metabolism biosynthesis, amino acid metabolism, the nervous system, the metabolism of terpenoids and polyketides, the immune system, environmental adaptation and aging (Table S11). The GO terms of the contracted genes were related to the cellular component, flavonoid biosynthetic process, developmental process, stimulus response, catalytic activity, biological regulation, transcription regulator activity, immune system process, signaling, growth and reproduction process categories (Table S12). When we sought to investigate the environmental adaptation of *C. chinensis*, we found that a series of photosynthesis and energy metabolism-associated gene families exhibit significant expansion, including the NADPH oxidase (NDHF, NDHB1, GPSA), NADH dehydrogenase (NAD1, NDHD, NAD4L, NAD7, NDHG), chlorophyll a-b binding protein (LHCB) and cytochrome P450 (CYP86) families (Table S13). Notably, CYP450 genes are involved in the regulation of basic developmental processes such as cell differentiation and growth, indicating effects on the expression of developmental genes^19^. Additionally, approximately half of the encoded proteins catalyze specific biochemical reactions in various metabolic pathways. To obtain insights into the evolutionary relationships among *C. chinensis* CYP450 family proteins, we constructed a phylogenetic trees based on the sequences of full-length CYP450 proteins from 216 amino acid sequences of *C. chinensis* (121) and *A. thaliana* (95) using the neighbor-joining method. The numbers of most CYP450 types, including CYP71, CYP86 and CYP90, were higher than in *A. thaliana* indicating a close relationship of the development and metabolism of *C. chinensis* plants (Fig. S4).

**Fig. 6:**
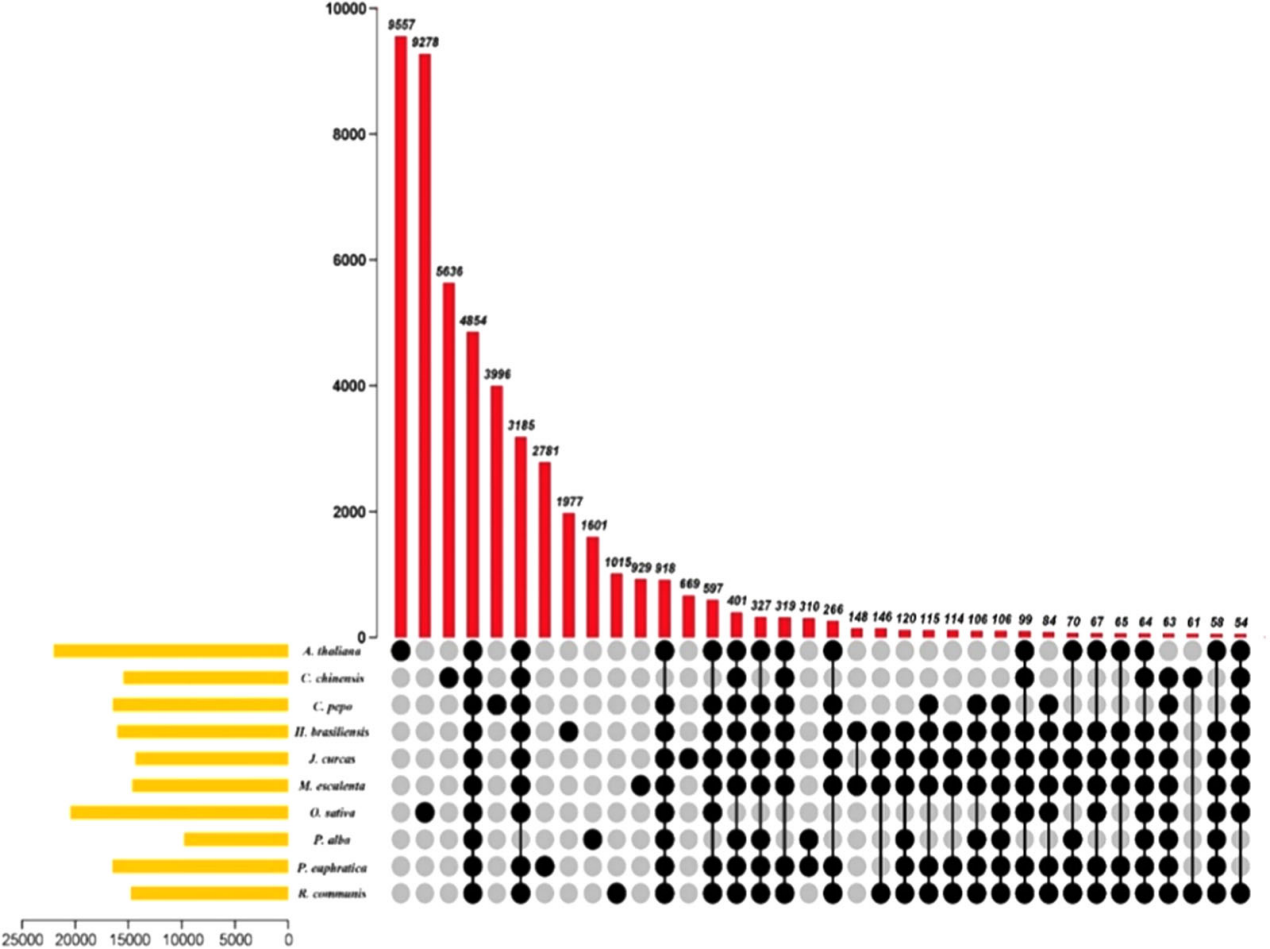
UpSet plot of the intersection of gene families in *C. chinensis, A. thaliana, O. sativa, P. alba, M. esculenta, P. euphratica, J. curcas, R. communis, C. pepo and H. brasiliensis*. The numbers of gene families (clusters) are indicated for each species and species intersection

The comparison of *C. chinensis*, *A. thaliana*, *O. sativa*, *P. alba*, *M. esculenta*, *P. euphratica*, *J. curca*s, *R. communis*, *C. pepo* and *H. brasiliensis* revealed that 4858 (31.37%) of the 15,485 *C. chinensis* gene families were shared by the other nine species, whereas 5,636 gene families were unique to *C. chinensis* (Fig. 6; Fig. S5). These 5,636 unique families have been specific to *C. chinensis* during its long history of evolution. Some of them may have been lost in other species, although we believe that some gene families originated de novo in Podostemaceae. Functional analysis performed via GO analysis revealed that these 5,636 unique families were enriched in suberin biosynthesis, cationic antimicrobial peptides, folate biosynthesis and ABC transporters (Table S14). KEGG analysis showed that most of the 5636 unique families were clustered in the signal transduction, lipid metabolism, energy metabolism and environmental adaptation categories (Table S15).

### Phylogenetic analysis of the WRKY, NAC and resistance gene families

**Fig. 7:**
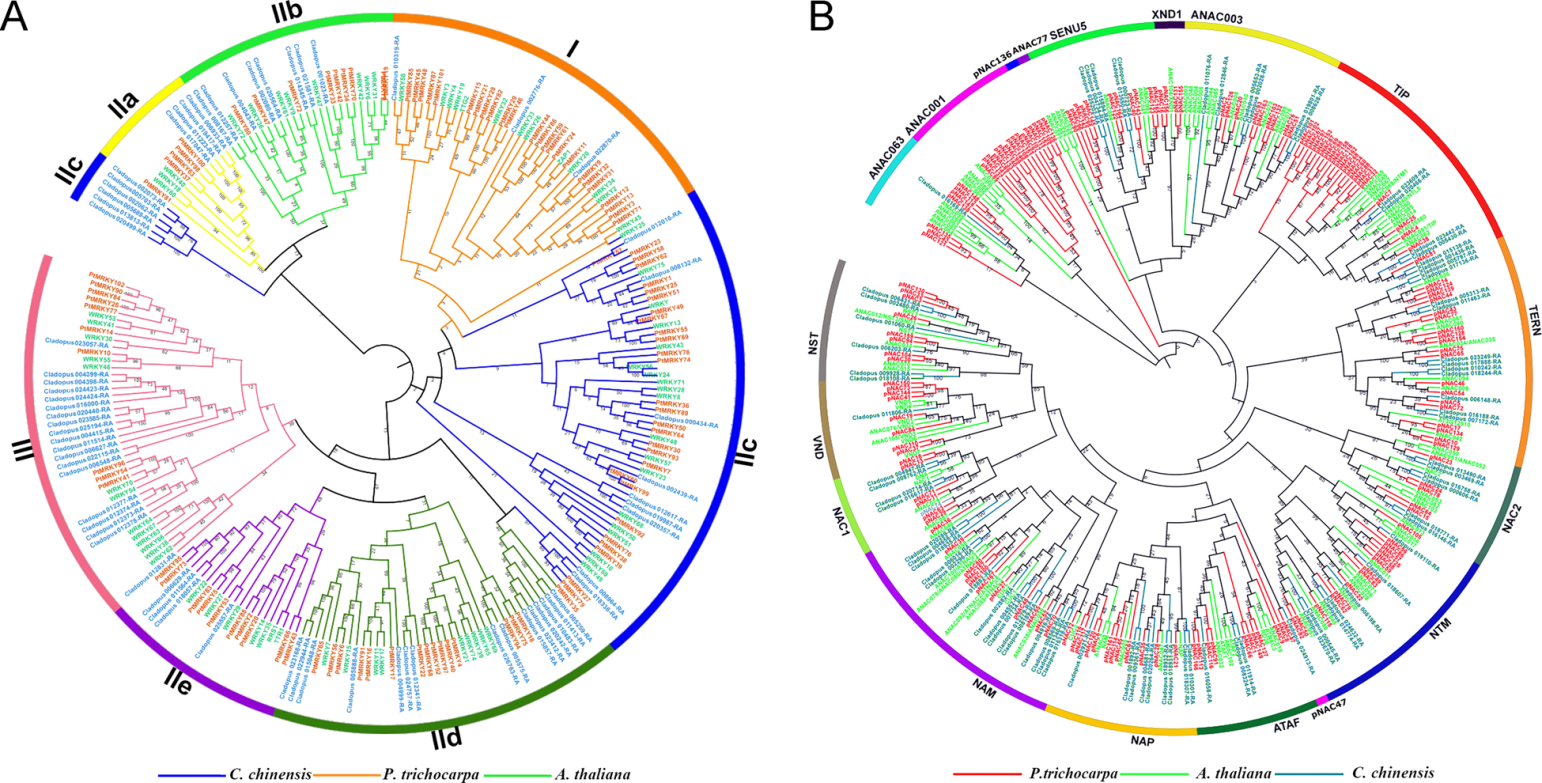
The evolutionary tree of WRKY and NAC gene families in *C. chinensis*. **a** The evolutionary tree and expression values of WRKY box genes in *C. chinensis*, *P. trichocarpa* and *A. thaliana*. **b** The evolutionary tree and expression values of NAC box genes in *C. chinensis*, *P. trichocarpa* and *A. thaliana*. Genes in the WRKY and NAC families were clustered separately using MEGA7 software via the neighbor-joining method

A phylogenetic tree was constructed based on the WRKY gene family members from *A. thaliana* (72), *P. trichocarpa* (102) and *C. chinensis* (68) to examine the evolutionary relationships among them. According to our results, a total of 68 WRKY genes were identified in the *C. chinensis* genome and classified into 7 groups: I, IIa, IIb, IIc, IId, IIe and III. The subfamily III group was one of the most ancient WRKY types, while subfamily I had fewer members, with no more than six members in each species. The identified WRKY genes of *C. chinensis* were only classified into the IIc subfamily (Fig. 7a).

To obtain insights into the evolutionary relationships among *C. chinensis* NAC family proteins, phylogenetic trees were constructed based on the sequences of full-length NAC-box proteins from 350 amino acid sequences of *C. chinensis* (81), *P. trichocarpa* (165) and *A. thaliana* (104) using the neighbor-joining method. The 81 NAC genes of *C. chinensis* were classified into 18 categories, including NAC1, NAP, NAM and TIP subfamily genes. The number of NAM and NAP subfamily genes from the NAC family was higher than in *A. thaliana* and *P. trichocarpa*. We speculate that the members of the NAM and NAP subfamilies could play roles in the development and defense responses of *C. chinensis* (Fig. 7b).

Furthermore, we analyzed the evolutionary relationships among resistance (R) gene proteins from *C. chinensis* (26) and *A. thaliana* (423) via phylogenetic tree analysis. The results showed that the R gene proteins could be classified into three groups, including TNL, CNL and RLP/RLK subfamily genes. Interestingly, the number of R gene proteins from *A. thaliana* (423) was 16.26-fold higher than that in *C. chinensis* (26), and the RLP/RLK subfamily genes of *C. chinensis* were identified and clustered. It is speculated that the presence of most R gene proteins reduces resistance and environmental adaptability, resulting in the survival of *C. chinensis* only in good-quality water (Fig. S6).

### Positively selected genes in *C. chinensis*

The *K*a*/K*s ratios of the single-copy genes were evaluated, and positively selected genes were identified in *C. chinensis*. A total of 490 candidate genes in *C. chinensis* underwent positive selection (*P* < 0.05). Most of which were enriched in GO terms related to the biological regulation, cellular process, developmental process, growth, metabolic process, stimulus response, catalytic activity, binding, positive regulation of biological process and cell part categories (Table S16; Fig. S7). KEGG enrichment analyses showed that the positively selected genes were enriched in the categories of environmental adaptation, endocrine system, metabolism of terpenoids and polyketides, lipid metabolism, energy metabolism, carbohydrate metabolism, biosynthesis of other secondary metabolites, amino acid metabolism, translation, signal transduction and cell growth (Table S17; Fig. S8).

### Comparative analysis of gene expression

**Fig. 8:**
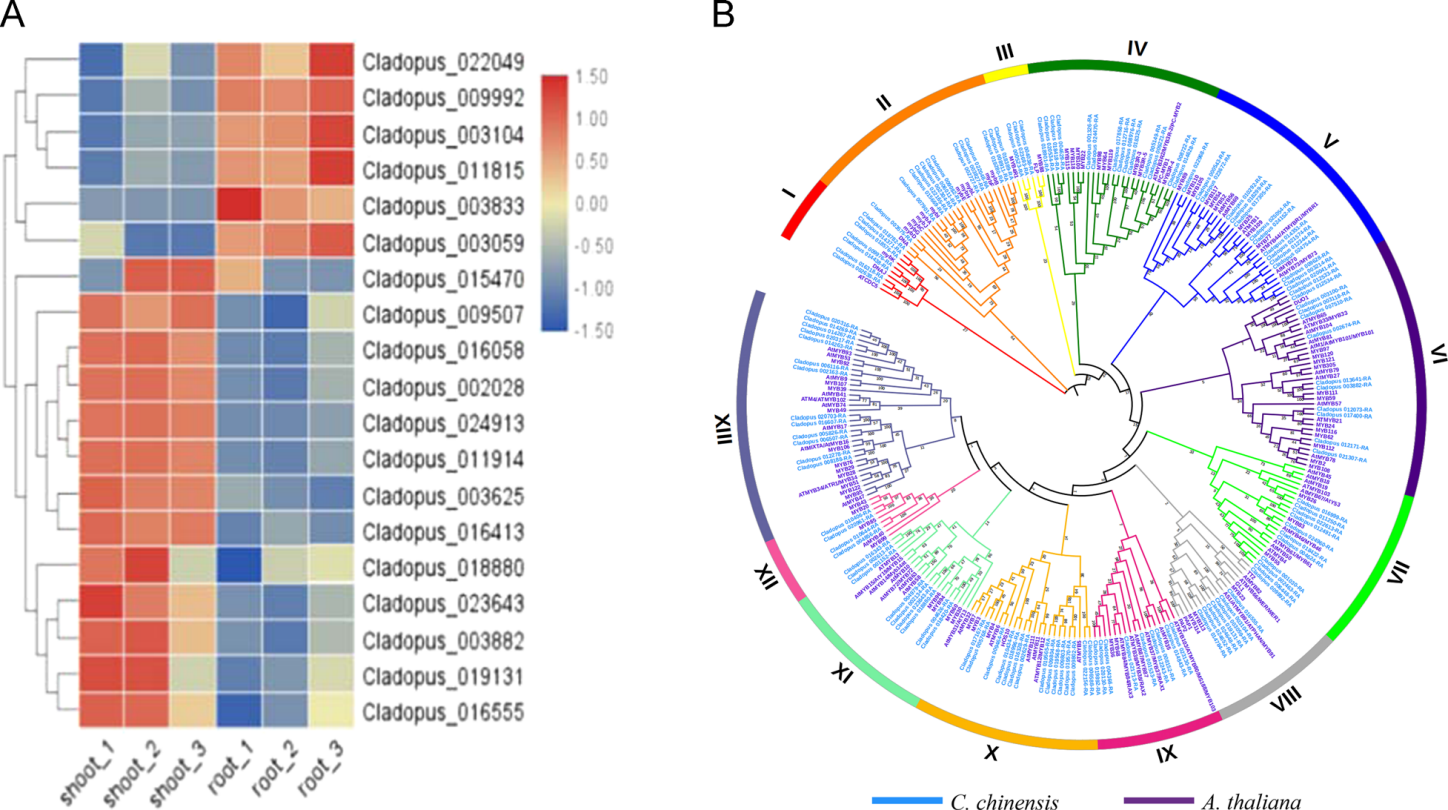
Expression patterns of the root- and shoot-specific expression and evolutionary tree of MYB gene family in *C. chinensis*. **a** Heatmap showing the root- and shoot-specific expression of the members of WUS, STM, ASL and NAC. Each box represents an individual gene, and the red and blue boxes represent relatively high levels and low levels of gene expression, respectively. **b** Evolutionary tree and expression values of MYB box genes in *C. chinensis* and *A. thaliana*. Genes in the MYB family were separately clustered using MEGA7 software via the neighbor-joining method. The genes of *C. chinensis* and *A. thaliana* are indicated by blue and purple, respectively

To obtain an overview of the transcriptome profile, principal component analysis (PCA) was performed according to normalized log10 (FPKM + 1) values. The first principal component (PC1), which explained 99.66% of the total variance, showed clearly different gene expression profiles between the root group and the shoot group (Fig. S9). We identified the specific genes responsible for shoot development and defense responses in *C. chinensis*. In comparison with the root group, we identified 3376 DEGs (log2 FC ≥ 2 and *p*-value ≤ 0.05) in the shoot group, including 2002 downregulated and 1374 upregulated genes (Table S18). Some of these DEGs were associated with shoot development, including WUS, ASL and STM. Among these genes, CcWUS10, CcASL4, CcASL9, CcSTM3 and CcSTM4 were expressed at low levels in the shoots, whereas CcWUS1, CcASL1, CcASL2 and CcASL7 were highly expressed in the shoots (Fig. 8a, Tables S18–S21). Podostemaceae species have extraordinary body plans showing unusual shoot organogenesis involving the modification of the SAM, making *C. chinensis* an ideal system for studying the development of roots and shoots in plants. To identify the potential key genes or transcription factors involved in shoot formation in *C. chinensis*, we identified 609 genes that were highly expressed in shoots (log2 FC ≥ 1.5 and *p*-value ≤ 0.01) (Table S22; Fig. S10), and these highly expressed genes were significantly enriched in the metabolic process, stimulus response, developmental process, environmental adaptation, signal transduction, energy metabolism, carbohydrate metabolism and secondary metabolite synthesis were identified according to KEGG and GO enrichment analyses (Figs. S11, S12; Tables S23, S24). Additionally, we found that the 609 highly expressed genes in the shoots included hormone biosynthesis and signaling genes, including 11 auxin, 1 cytokinin, 2 abscisic acid, 5 gibberellin and 1 brassinosteroid gene. The development of the plant shoot is dependent on the SAM, MYB and bHLH transcription factors, which could regulate meristem initiation, meristem function and leaf patterning. Notably, 107 transcription factors were identified from the 609 highly expressed genes in the shoots, including 11 MYB, 11 bHLH and HD-ZIP family proteins (Table S25). In addition, we observed that the CcASL1, CcASL2, CcASL7, CcASL8 and CcSTM1 genes were involved in shoot development and were significantly expressed in specific shoot tissues (Fig. S13). Furthermore, we identified 132 and 146 MYB proteins in *C. chinensis* and *A. thaliana*. These MYB genes are divided into 13 subfamilies, and the numbers of genes in subfamilies I, III, and X are greater than those in the other subfamilies of *A. thaliana* (Fig. 8b). MYB transcription factors are widely involved in the regulation of various physiological responses and phenylpropanoid metabolic pathways, indicating a close relationship with the growth of *C. chinensis* on submerged rock surfaces in rapids and waterfalls during the rainy season.

The expression of WUS, STM and ASL is involved in the initiation and maintenance of shoot development in Tristichoideae and Podostemoideae, respectively^8^. To confirm the accuracy of the obtained unigene expression levels, nine target genes (CcWUS1, CcWUS10, CcASL1, CcASL2, CcASL4, CcASL7, CcASL9, CcSTM3, CcSTM4) associated with shoot development were analyzed in the present study (Fig. S13). The relative levels of the amplified mRNAs were evaluated according to the 2^−ΔΔCt^ method. Compared with the FPKM values, the relative expression levels of all nine unigenes determined by qRT-PCR were consistent with the RNA-seq data.

## Discussion

The Podostemaceae family comprises ~300 species classified into ~50 genera, among which 3 genera, *Cladopus*, *Terniopsis* and *Hydrobryum*, have been confirmed from Fujian, Hainan, Guangdong and Yunnan in China (Fig. S14). Recently, a set of studies assessing the phylogeny, classification, biogeography and morphological evolution of the Podostemaceae family were reported. Katayama et al. examined the expression patterns of the STM, WUS and ARP genes during shoot development in Tristichoideae and Podostemoideae and tried to reveal the genetic basis for the evolution of shoots in Podostemaceae^8^. Mutations in the WUS gene of *A. thaliana* reported by Hamada et al. halted the entire process of SAM formation and resulted in the production of no juvenile leaves^13^. Here, we assembled the genome of *C. chinensis* to the superscaffold scale using the Hi-C technique. The *C. chinensis* genome assembly has been vastly improved by Hi-C analysis, producing an N50 scaffold size for the assembly of 21.22 Mb, compared to the N50 contig size of 1.42 Mb for the de novo-assembled genome obtained with Canu software. These results demonstrated that a high-quality *C. chinensis* genome was produced and suggested that the numbers of repetitive sequences and ncRNA sequences were relatively high in *C. chinensis* compared with other dicotyledon species. The present genome provides a good reference for understanding the distinct aerial shoot and underground root system traits of *C. chinensis* and related species.

According to the NCBI taxonomy database, *C. chinensis* is classified into Podostemonaceae of Malpighiales based on morphological characteristics and partial nucleotide alignment (*rbc* L, *atp*B, 18 S rRNA), which is consistent with the integrated system of the angiosperm phylogeny group (APG) classification (APG, 1998; APG II, 2003; APG III, 2009; APG IV, 2016)^20^. However, Podostemonaceae was removed from Malpighiales and classified into Podestemales in the integrated system of the classification of flowering plants by Cronquist^21^ and Wu^22^. Based on a concatenated sequence alignment of *C. chinensis* and 17 other plant species, a phylogenetic tree was reconstructed (Fig. 4a). The bootstrap values were >99% for all the relationships. *P. alba*, *J. curcas*, *M. esculenta*, *R. communis*, *P. euphratica*, *H. brasiliensis* and *P. trichocarpa* were grouped together in Malpighiales*. C. chinensis* phylogenetically diverged into an independent branch approximately 106 million years ago (Mya), after the divergence of the Brassicales lineage 108 Mya but before the divergence of Malpighiales (82 Mya) and *Rosales* (102 Mya). We found that the internal branches separating Brassicales, Podestemales, Rosales, and Malpighiales were very short, as shown in Fig. 4a, suggesting the rapid diversification of these major clades. We performed coalescent analysis with RAxML and ASTRAL to estimate the species tree from gene trees. This result showed that *C. chinensis* phylogenetically clustered into Malpighiales, which is inconsistent with the findings of Cronquist^21^ and Wu^22^ (Fig. 4b). Based on our data, we speculated that *C. chinensis* may belong to the order Malpighiales, which is consistent with the conclusions from the APG and NCBI Taxonomy.

KEGG and GO analyses showed that the cytochrome P450 (CYP) gene and serine/threonine protein kinase families were significantly contracted in the *C. chinensis* genome (Tables S10, S11). The cytochrome P450 gene family (mostly the CYP81, CYP90, CYP85, CYP4 and CYP7 families) is related to isoflavonoid metabolic processes that regulate secondary metabolite synthesis and affect broad environmental adaptability. Previous research suggests that the cytochrome P450 gene family is important for the regulation of basic developmental processes. For example, a previous study showed that high levels of CYP85 family gene expression alleviate the jasmonate response, resulting in longer primary roots and more lateral roots and enhanced drought tolerance in tobacco^19^. The ROTUNDIFOLIA3 (ROT3) locus of *A. thaliana*, which is involved in the regulation of leaf morphogenesis, harbors a CYP90 sequence^23^. Serine/threonine protein kinases are involved in the response to environmental stresses (high salt, drought and low temperature) and regulate multiple life processes in cells^24^. It is speculated that the contraction of these types of gene families affects the accumulation of secondary metabolites and reduces environmental adaptability, resulting in the survival of *C. chinensis* only in good-quality water.

ATP is an energy carrier and is conserved in the form of NADH, and FADH_2_ must be converted to ATP (oxidative phosphorylation). NADH and NADPH undergo spontaneous enzymatic reactions that participate in photosynthesis induced by light via the phytochrome system and respiratory metabolism^25^. The chlorophyll a-b-binding proteins are located in the chloroplast thylakoid membrane, where Chl a and Chl b bind to form protein complexes that transfer absorbed light energy to photosystem reactions^26^. KEGG and GO analysis showed that most of the expanded genes in the *C. chinensis* genome are involved in plant energy metabolism, especially oxidative phosphorylation (NADH and NADPH) and photosynthesis (chlorophyll a-b-binding proteins) (Tables S8, S9). Thus, it is likely that the significant expansion of energy metabolism-associated genes in *C. chinensis* could increase oxidative phosphorylation and photosynthesis, which would be beneficial for the growth of the plants on submerged rock surfaces and for bearing flowers exposed to the air when the water level decreases.

Katayama et al. analyzed the expression patterns of key regulatory genes related to shoot development (i.e., STM, WUS and ARP orthologs) in Tristichoideae and Podostemoideae. They found that STM and WUS were expressed in the floral meristem, similar to the pattern in model plants. Because of limited genome information, it was impossible for these authors to annotate all members of the STM, WUS and ASL gene families. The probes used for in situ hybridization in their studies could only indicate the expression profiles of these genes according to the coexpression of all gene family members^8^. Based on the available genome information and transcriptome data, we annotated all members of these gene families and analyzed their gene expression patterns. Additionally, we found that the expression levels of different members of these gene families were upregulated or downregulated to very different extents other in shoots. In this study, we performed transcriptome sequencing to identify the genes that are involved in the diversity of SAM establishment in whole shoots and roots. In future work, we will perform single-cell transcriptome analysis to evaluate the expression patterns of these genes in fine fractions of different development stages (e.g., meristem, young shoot, and mature shoot) by laser capture microdissection to reveal the roles of STM, WUS and ASL gene family members in the shoot development of *C. chinensis*.

The NAC proteins are one of the largest families of transcription factors in plants; these proteins regulate gene expression during the life of plants and exhibit important biological functions in response to both stress and plant growth^27^. We annotated 81 members of the NAC gene family in *C. chinensis*, and the numbers belonging to the NAM and NAP subfamilies were much higher than those of other subfamilies. NAC102 is an important gene of the NAM subfamily that is thought to participate in the regulation of seed germination in flooded environments^27^. Our transcriptome analysis showed that some genes exhibiting significant expression in shoots were members of the NAC gene family, including NAC2, NAC5, NAC8, NAC29, NAC47, NAC73, NAC83 and NAC102. We also found that among these highly expressed NAC genes, NAC29 (Cladopus_020264), NAC47 (Cladopus_004681) and NAC83 (Cladopus_004734) were included in the genes that were duplicated via a WGD event in *C. chinensis* superscaffold no. 4 and superscaffold no. 6 (Fig. S15). Additionally, the gene pairs (Cladopus_020264-Cladopus_004681, Cladopus_004734-Cladopus_025202) exhibited 97.8 and 97.0% identity scores, respectively (Table S26), suggesting that they were functionally redundant and were likely duplicated during the evolution of shoot development in *C. chinensis*. It is speculated that the presence and expression of these genes play roles in the shoot development and environmental adaptation of *C. chinensis*. However, which of these genes are beneficial to the environmental adaptation and shoot development of *C. chinensis* remains to be further studied and analyzed.

## Materials and methods

### Genome sequencing and de novo assembly

The root, leaf and shoot samples of *C. chinensis* (sample number CBH03085, Fig. S16) used for genome sequencing and assembly were collected from the Tingjiang River in Changting, Fujian, China (E116^。^25'10", N25^。^51'03", Alt. 281.3 m)^28^. The voucher specimens of *C. chinensis* were deposited in the Plant Herbaria of the College of Life Science, Fujian Normal University (collection number FNU0039809). To generate enough short and long reads for the genome assembly of *C. chinensis*, next-generation sequencing (NGS) on the Illumina HiSeq X Ten platform and third-generation sequencing (TGS) on the PacBio SEQUEL platform were applied for genome sequencing. Genomic DNA was produced via the CTAB method, and the integrity of the DNA was checked via agarose gel electrophoresis. The purity and concentration of the DNA were analyzed by using a NanoDrop 2000 spectrophotometer (Thermo Scientific, USA). For Illumina X Ten sequencing, we constructed a paired-end library with 250 base pair (bp) sequences using the method indicated by the manufacturer. As a result, 125.95 Gb (~140X coverage of the estimated genome size, Table 1) of short reads were generated from the Illumina platform, which were further cleaned by using Trimmomatic (version 0.36) with the default parameters, resulting in 106.2 Gb (~118×, Table 1) of cleaned data for the following analysis. For PacBio library construction and sequencing, SMRTbell libraries (~20 Kbp) were obtained according to the PacBio protocol. After the removal of adapters, 95.36 Gb of subreads (~105×, Table 1) were corrected, trimmed and assembled using CANU (version 1.6) with the parameters corOutCoverage = 80 and corMinCoverage = 0. To improve accuracy, the primary contigs were further filtered with the Pilon program^29^ using 106.2 Gb (118×) of Illumina paired-end reads. We summed the statistics of the assemblies in Table 1. Genome completeness was assessed using the BUSCO databases^30^ with embryophyta_odb10 models.

### Estimation of genome size and heterozygosity

Weused NGS short reads and a K-mer-based method to estimate the genome size, heterozygosity and repeat content of *C. chinensis*. Approximately 106.2 Gb of reads (250 bp) were generated and used to determine the abundance of 21-K-mers in the Illumina data. The frequency of 21-K-mers was counted with Jellyfish software^31^.

Root, leaf and shoot samples of *C. chinensis* were prepared according to a previously reported protocol^32^ and stained with propidium iodide (50 mg/ml). Quantification was performed using flow cytometry in a BD FACSCalibur cytometer (Becton Dickinson, San Jose, CA), and the results were calculated as the ratio of the mean fluorescence of *C. chinensis* to that of *Zea mays* B73. The estimated genome size of *C. chinensis* was 835 ± 5.52 Mb.

### High-quality assembly using Hi-C technology

Samples were examined for the integrity of nuclei by DAPI staining to ensure the quality of the Hi-C library. Samples with confirmed high-quality nuclei were subjected to the Hi-C procedure^33,34^. Chromatin was digested with the restriction enzyme *Mbo* I or *Hin*d III and ligated together in situ after biotinylation. DNA fragments were enriched via the interaction of biotin and blunt-end ligation and then subjected to HiSeq sequencing. From Hi-C library sequencing, approximately 96.4 Gb of data were generated (Table 1). The sequencing reads were mapped to the *C. chinensis* genome with Bowtie software. We applied an iterative alignment method to increase the read mapping ratio. We aligned the two read ends to the genome independently, and only the read pairs in which both ends were uniquely aligned to the reference genome were used for the detection and filtering of valid interaction products by using HiC-Pro (version 2.7.8)^35^. The order and direction of scaffolds/contigs were clustered into superscaffolds by using LACHESIS^36^ based on the relationships among valid reads.

### RNA extraction and sequencing

Total RNA was prepared from *C. chinensis* roots and shoots (Fig. S17) using TRIzol reagent (Invitrogen, California, USA). A NanoDrop 2000 spectrophotometer (Waltham, MA, USA) and an Agilent 2100 Bioanalyzer (Agilent Technologies, USA) were applied to check RNA quality; the absorbance at 260 nm/280 nm was 1.8, and the RIN value was 9.1. Equal amounts of RNA from each tissue were used for cDNA library construction. Approximately 77.7 Gb of transcript data were produced for *C. chinensis* from the Illumina HiSeq X Ten sequencing platform and processed using Trimmomatic (version 0.36) with the default parameters. Reads originating from RNA-seq were aligned to the reference genome using HISAT2^37^. FPKM values and read counts were estimated using Stringtie^38^ and Ballgown^39^. The differential expression of genes was analyzed using edgeR^40^, for which the criteria were a log2-fold change (FC) ≥ 1 and a false discovery rate (FDR) ≤ 0.05.

### Genome annotation

In the *C. chinensis* genome, de novo- and homology-based approaches were combined to search TEs and other repetitive sequences. We identified repeat sequences using Tandem Repeats Finder^41^, LTR_FINDER^42^, RepeatProteinMask and RepeatMasker^43^ and used Tandem Repeats Finder to search for tandem repeats in the genome assembly with the following parameters: Mismatch = 7, Match = 2, Delta = 7, PI = 10, Minscore = 50, PM = 100, and MaxPerid = 2,000. Using LTR_FINDER (version 1.0.6), a de novo repeat library was built. Subsequently, we aligned the genome sequences to Repbase TE (version 3.2.9)^44^ by using RepeatMasker for the searching of known repeat sequences and mapping onto the de novo repeat libraries to identify novel types of repeat sequences. tRNAscan-SE (version 2.0.3)^45^ was used to detect reliable tRNA positions, and noncoding RNA (ncRNA) was predicted by searching the RFAM (version 12.0)^46^ databases by using Infernal software (version 1.0)^47^ with the default parameters. Centromere and telomere repeats were identified with Tandem Repeats Finder (version 4.07b)^41^. We transformed the resulting ‘dat_dir file’ into a GFF3 file, which was used to identify centromeric and telomeric repeats.

The *C. chinensis* genome assembly was annotated via the following approaches: homology-based, transcriptome-based, and *ab initio* annotation. Thirteen representative species were selected to perform homology annotation, including *Manihot esculenta* (*M. esculenta*)^48^, *Populus euphratica* (*P. euphratica*)^49^, *Populus alba* (*P. alba*)^50^, *Jatropha curcas* (*J. curca*s)^51^, *Ricinus communis* (*R. communis*)^52^, *Hevea brasiliensis* (*H. brasiliensis*)^53^, *Arabidopsis thaliana* (*A. thaliana*)^54^, *Cucurbita pepo* (*C. pepo*)^55^, *Fragaria vesca* (*F. vesca*)^56^, *Malus domestica* (*M. domestica*)^57^, *Populus trichocarpa* (*P. trichocarpa*)^52^, *Pyrus x bretschneideri* (*P. bretschneideri*)^58^ and *Rosa chinensis* (*R. chinensis*)^59^. The protein sequences of these species were aligned to *C. chinensis* genome sequences using TBLASTN software^60^ with an E-value ≤ 1e-5. Genewise (version 2.2.0)^61^ was utilized to predict the exact gene structures based on all TBLASTN results. We used Cufflinks (version 2.2.1)^62^ to preliminarily identify gene structures. Augustus^63^ was used for *ab initio* annotation with the repeat-masked genome sequences. We integrated all genes predicted from the three annotation procedures with MAKER software^64^.

Functional annotation of the protein-coding genes was carried out by using BLASTP with an E-value ≤ 1e-5 in four integrated protein sequence databases: eggNOG, GO, COG and KEGG. We used InterProScan (version 4.8)^65^ and HMMER (version 3.1)^66^ to annotate protein domains by searching the INTERPRO (version 32.0)^67^ and Pfam (version 27.0)^68^ databases. Gene Ontology (GO) terms were produced from the InterPro or Pfam entry^69^. The pathways were assigned through BLAST searches in the Kyoto Encyclopedia of Genes and Genomes (KEGG) database (release 53) with an E-value ≤ 1e-5^70^. The functions of the genes were predicted and classified using the Clusters of Orthologous Groups (COG) database^71^.

### Phylogenetic analysis and divergence time estimation

We investigated the relationships of *C. chinensis* with 17 other species and performed a phylogenetic analysis based on protein-coding genes from the *C. chinensis* genome and 17 other species. We extracted and downloaded the protein sequences of single-copy genes from 18 species from the NCBI database, including *M. esculenta*, *P. euphratica*, *P. alba*, *J. curca*s, *R. communis*, *H. brasiliensis*, *A. thaliana*, *C. pepo*, *F. vesca*, *M. domestica*, *P. trichocarpa*, *P. bretschneideri*, *R. chinensis*, *Brassica oleracea* (*B. oleracea*), *Brassica rapa* (*B. rapa*), *Raphanus sativus* (*R. sativus*) and *Oryza sativa* (*O. sativa*). The similarities among the proteins from all species were searched in an all-to-all manner by using BLASTP software with an E-value ≤ 1e-5. By using OrthoFinder software (version 2.27)^72^, we generated multiple sequence alignments for the protein sequences in each single-copy family with the default parameters as well as for phylogenetic tree construction. We designated *O. sativa* as the outgroup of the phylogenetic tree. The phylogenetic relationships were constructed through the superalignment of the coding DNA sequences (CDSs) using the maximum likelihood (ML) method, and the divergence time between species was estimated using the MCMCtree program of PAML (http://abacus.gene.ucl.ac.uk/software/paml.html). The TimeTree database (http://www.time.org/) was used for the divergence time recalibration of these plant species. The CDSs were aligned with the guidance of the protein alignments and then concatenated into the superalignment matrix of each family. We compared the cluster size differences between the ancestor and each species and analyzed the expansion and contraction of the gene families by using CAFE software (version 2.1)^73^. For coalescent analysis, the concatenated alignment of single-copy homologous genes for 18 species was used as the input for ML inference with RAxML (version 7.2.8)^74^. The alignment was partitioned by the genes, and ProtTest^75^ was used to select the appropriate model of amino acid substitution for each partition. We used the -f a option of RAxML to generate 200 rapid bootstrap replicates, followed by a search for the best-scoring ML tree, and then used ASTRAL^76^ to estimate the species tree from the gene trees.

### Synteny analysis

The conserved paralogs of the protein sequences of *C. chinensis* were obtained with BLASTP (E-value ≤ 1E-5). By using MCScanX (http://chibba.pgml.uga.edu/mcscab2), we identified collinearity blocks in the genome. The Circos tool (http://www.circos.ca) was used to map gene density, GC content and repeat content as well as gene synteny on individual pseudochromosomes.

### Whole-genome duplication

We took advantage of the high-quality genome of *C. chinensis*, analyzed WGD events and determined the source of the high number of genes in *C. chinensis*. First, the protein sequences from *C. chinensis* were searched to identify syntenic blocks by using BLASTP with an E-value ≤ 1e-5. We identified gene synteny and collinearity by using MCScanX software^77^ and calculated the synonymous substitution rate (*K*s) for syntenic gene pairs using *K*a*K*s_Calculator software^78^ and the Nei-Gojobori method^79^.

### Quantitative real-time PCR (qRT-PCR) validation

Total RNA was extracted from the three replicates using the TransZol Up Plus RNA Kit (Transgen Biotech, Beijing). Primers were designed using Primer Premier 5.0, and the sequences are listed in Table S27. qRT-PCR was performed using the ABI 7300 Real-time PCR System (Framingham, MA, USA) with SYBR Green PCR Master Mix (TaKaRa) following procedures described previously^80^. All PCR assays were performed in triplicate. The reference gene was glyceraldehyde-3-phosphate dehydrogenase (GAPDH). The relative expression level was quantified via the 2^−ΔΔCt^ method^81^.

## Supplementary information

Table S25 Transcription factors identified from 609 specific expressed genes in shoots

Supplementary Figure S1-S17

Table S14 Statistics of GO enrichment of the 5636 gene families in the C. chinensis

Table S15 Statistics of KEGG enrichment of the 5636 gene families in the C. chinensis

Supplementary Table S1-S7,S26-27

Table S8 Size and location of centromere satellite repeat arrays

Table S9. KEGG enrichment of the expansion familes genes identified in the C. chinensis

Table S10. GO enrichment of the expansion familes genes identified in the C. chinensis

Table S11. KEGG enrichment of the contraction familes genes identified in the C. chinensis

Table S12. GO enrichment of the contraction familes genes identified in the C. chinensis

Table S13 expand_annotation

Table S16 GO enrichment of the positively genes identified in the C. chinensis

Table S16 GO enrichment of the positively genes identified in the C. chinensis

Table S18 my.gene.counts.matrix.root_vs_shoot.DESeq2

Table S19 CcWUS_sequence

Table S20 CcALS1_sequence

Table S21 CcSTM4_sequence

Table S22 609 specific expressed genes in shoots

Table S23 GO enrichment of the 609 specific expressed genes in the shoot of C. chinensis

Table S24 KEGG enrichment of the 609 specific expressed genes in the shoot of C. chinensis

## Data Availability

The genome sequence data that support the findings of this study have been deposited in the BIG Sub system under BioProject accession number CRA002215 (http://bigd.big.ac.cn/gsa/s/a118ohEn). Raw sequencing data for RNA-Seq were used for annotation and biological analyses and have been deposited in the BIG Sub system under BioProject accession number CRA002218 (http://bigd.big.ac.cn/gsa/s/0VZ87KD1).
