# Supplementary material for: A high-quality genome provides insights into the new taxonomic status and genomic characteristics of Cladopus chinensis (Podostemaceae)
Source: Hortic Res. 2020 Apr 1;7:46. doi: 10.1038/s41438-020-0269-5 (PMC7109043; doi:10.1038/s41438-020-0269-5)
Supplement: Supplementary file 1 — Table S25 Transcription factors identified from 609 specific expressed genes in shoots [file 41438_2020_269_MOESM1_ESM.pdf]

Number	Classify
11	MYB family protein
11	basic helix-loop-helix (bHLH) DNA-binding superfamily protein
8	HD-ZIP family protein
7	Ethylene-responsivefactor
6	G2-like
6	NAC domain containing protein
6	TCP family transcription factor
5	Dof family protein
5	Trihelix
5	zinc finger-HD
4	Auxin response factor (ARF)
4	LBD transcription factor family protein
4	WRKY family transcription factor
3	B3 transcription factor family protein
3	basic region/leucine zipper transcription factor
3	GRF family transcription factor
2	DBB transcription factor family protein
2	GATA transcription factor family protein
2	plant-specific transcription factor YABBY family protein
2	AP2 transcription factor family protein
1	C2H2-type zinc finger family protein
1	C2H2 transcription factor family protein
1	CPP transcription factor family protein
1	M-type_MADS family protein
1	TALE
1	WOX
1	Nin-like
1	SAP
