# Supplementary material for: A high-quality genome provides insights into the new taxonomic status and genomic characteristics of Cladopus chinensis (Podostemaceae)
Source: Hortic Res. 2020 Apr 1;7:46. doi: 10.1038/s41438-020-0269-5 (PMC7109043; doi:10.1038/s41438-020-0269-5)
Supplement: Supplementary file 2 — Supplementary Figure S1-S17 [file 41438_2020_269_MOESM2_ESM.docx]

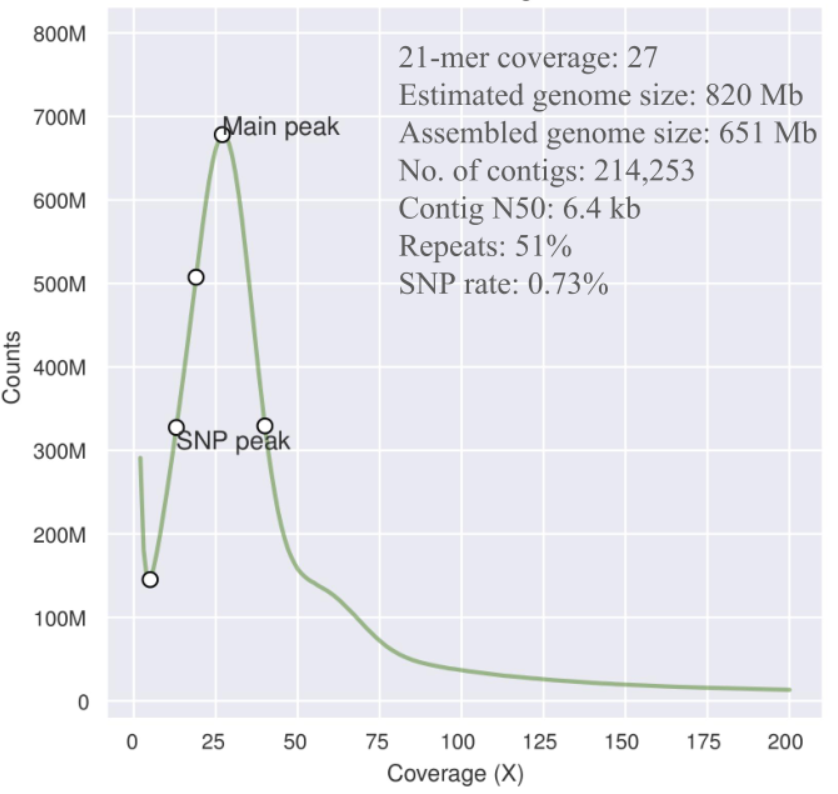


Fig. S1 21-K-mer count distribution for the genome size estimation.


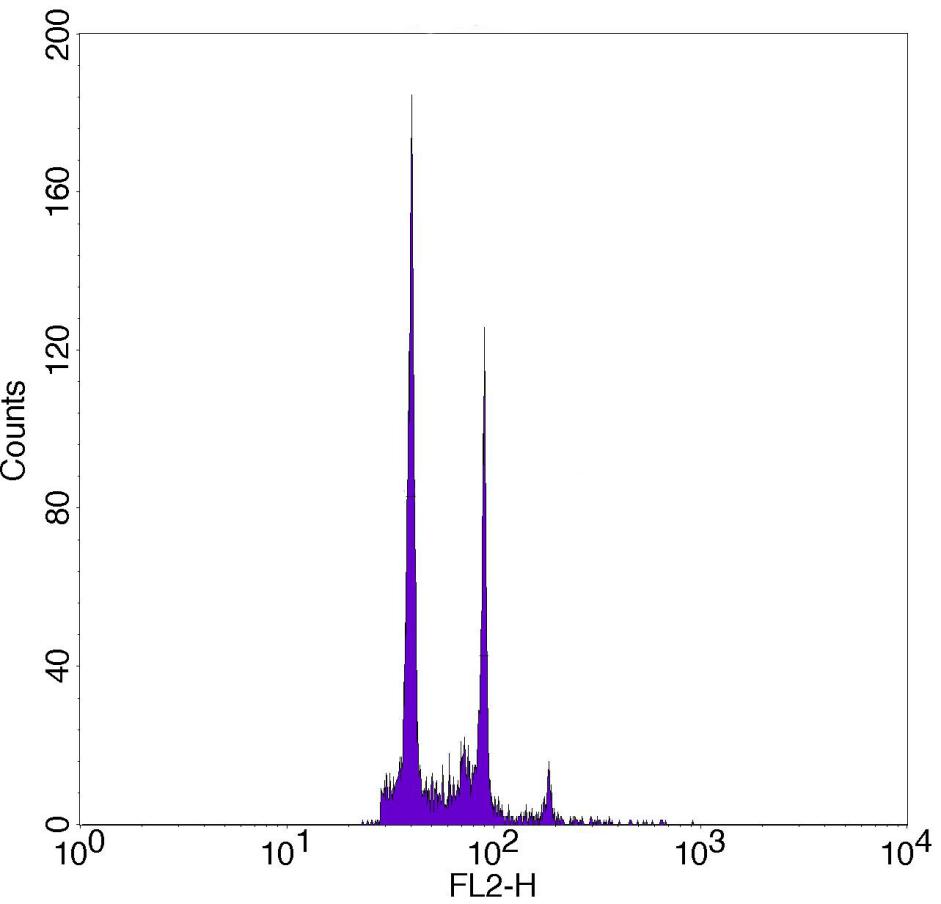


Fig. S2 The size estimation of the *C. chinensis* genome by flow cytometry. The genome size of *C. chinensis* was estimated to be approximately 36.30±0.24% of *Zea mays* B73 (~2.3 G) as internal reference. We validated the result by flow cytometry, with the *C. chinensis* genome size identified as 835±5.52 Mb.


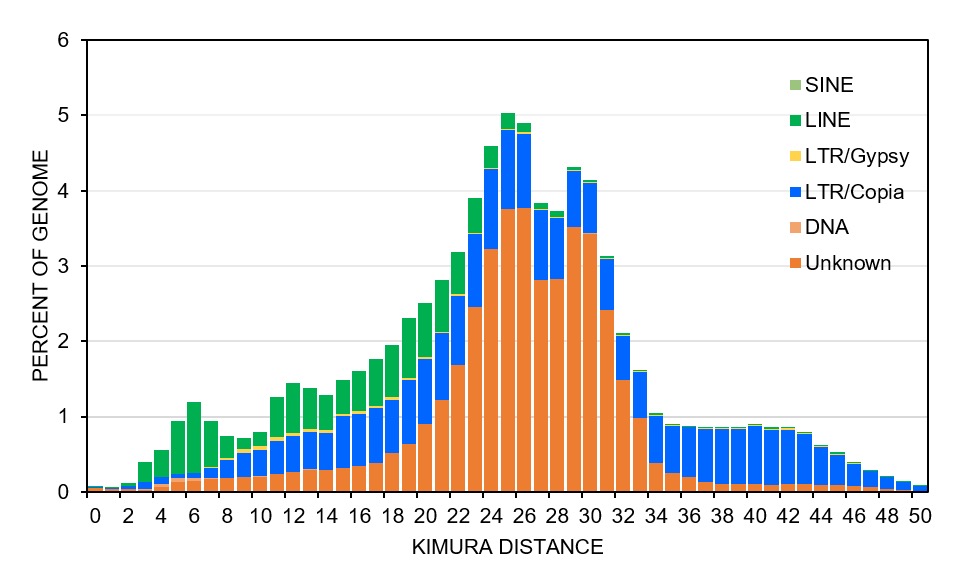


Fig. S3 Kimura distance-based copy divergence analysis of transposable elements in *C. chinensis* genome. The graph represents percentage of genome (y-axis) for each type of TEs (SINE,LINE, LTR/Gypsy, LTR/Copia and DNAtransposons), clustered according to Kimura distances to their corresponding consensus sequences (x-axis, K-value from 0 to 50).


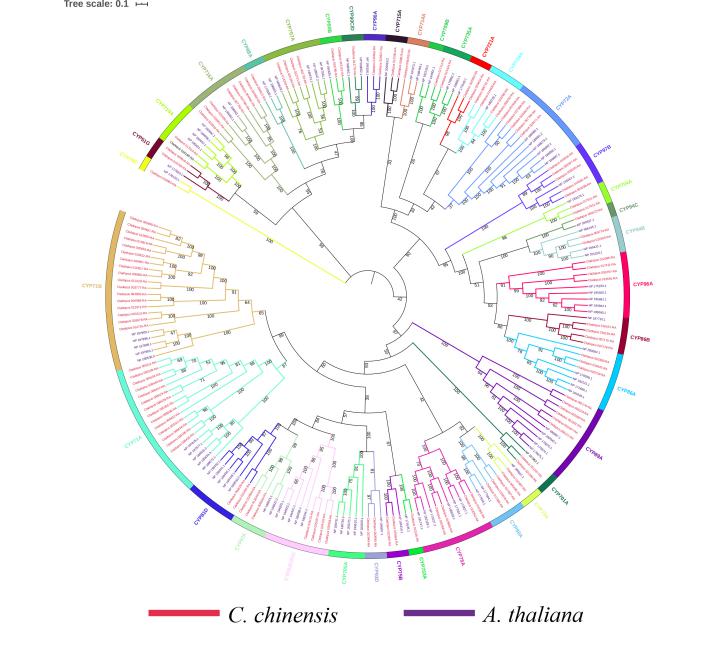


Fig. S4 The evolutionary tree and expression values of CYP450 box genes in *C. chinensis*, *P. trichocarpa* and *A. thaliana*.


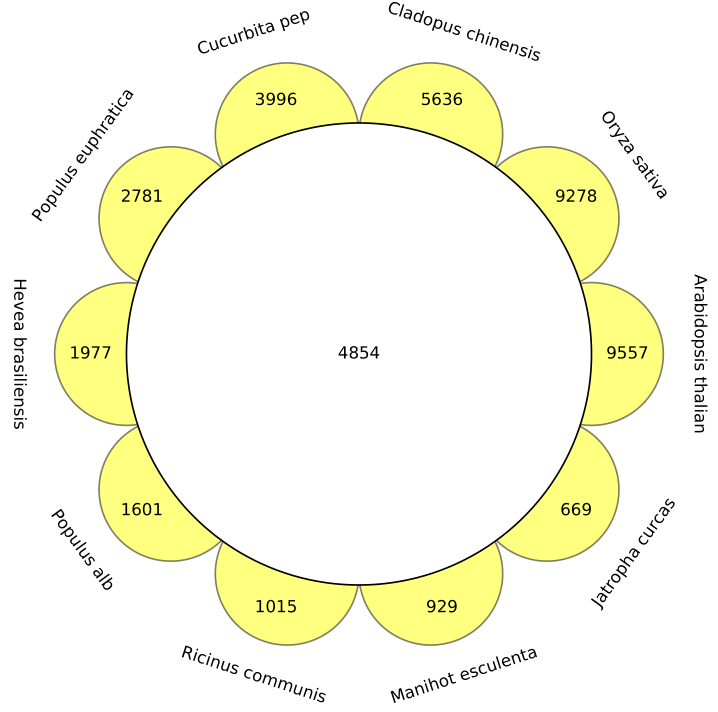


Fig.S5 Venn diagram of the number of gene families in *C. chinensis*,*A. thaliana*, *O. sativa*, *P. alba*, *M. esculenta*, *P. euphratica*, *J. curca*s, *R. communis*, *C. pepo* and *H. brasiliensis.* The numbers of gene families (clusters) are indicated for each species and species intersection.


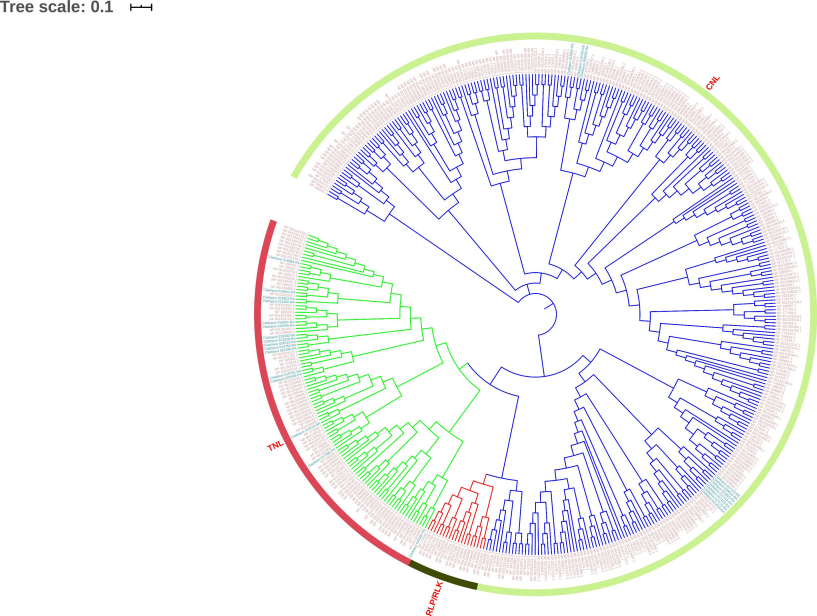


Fig. S6 The evolutionary tree and expression values of [resistance](http://xueshu.baidu.com/usercenter/paper/show?paperid=28c86e6a270c860cf56f3d04241087f3&site=xueshu_se) (R) genes in *C. chinensis*, *P. trichocarpa* and *A. thaliana*.


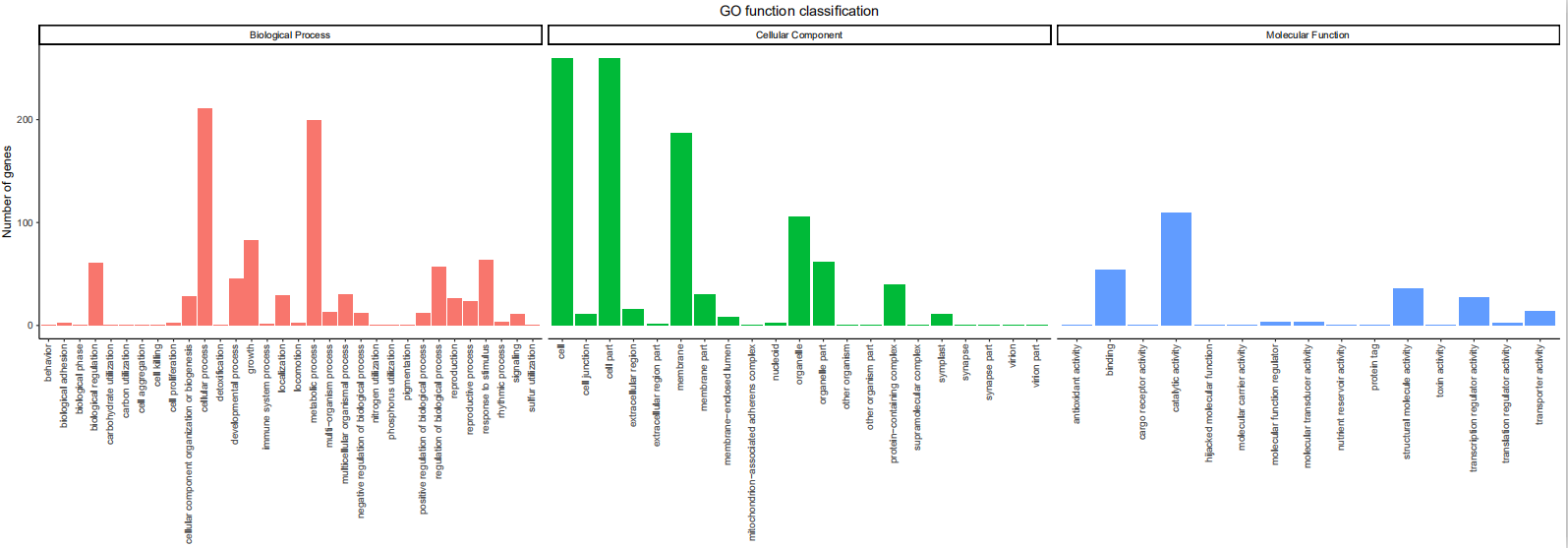


Fig. S7 GO enrichment of the 490 positively selected genes in *C. chinensis*


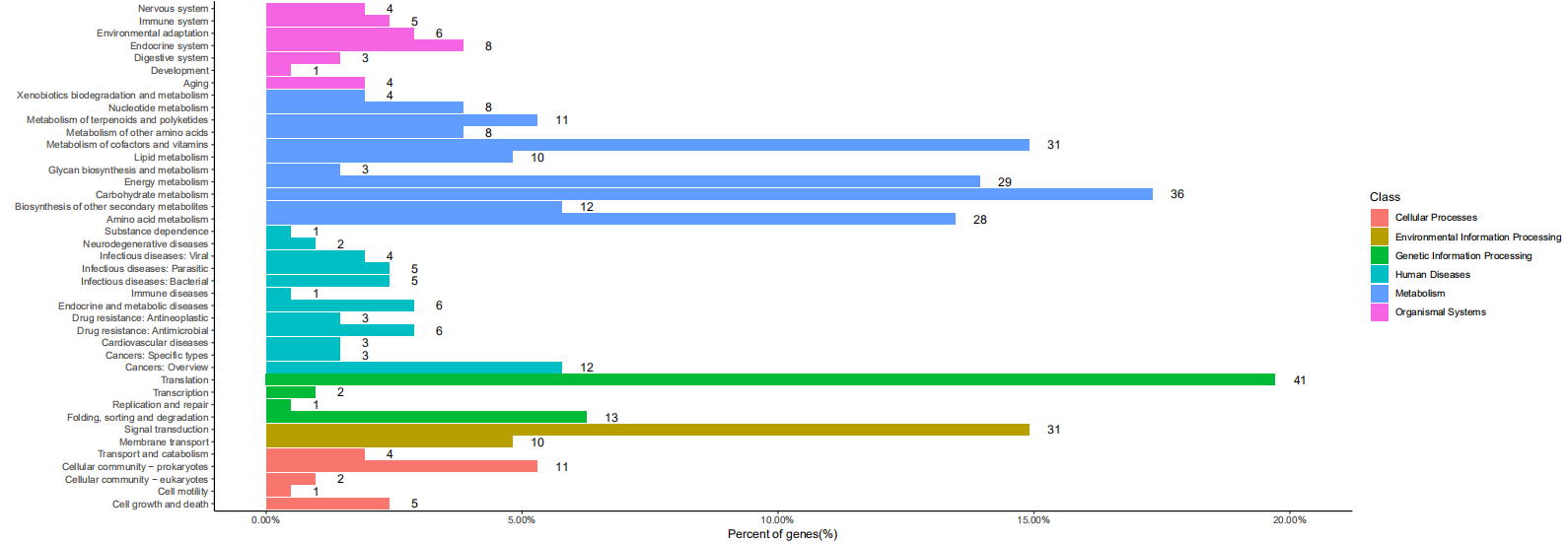


Fig. S8 KEGG enrichment of the 490 positively selected genes in *C. chinensis*


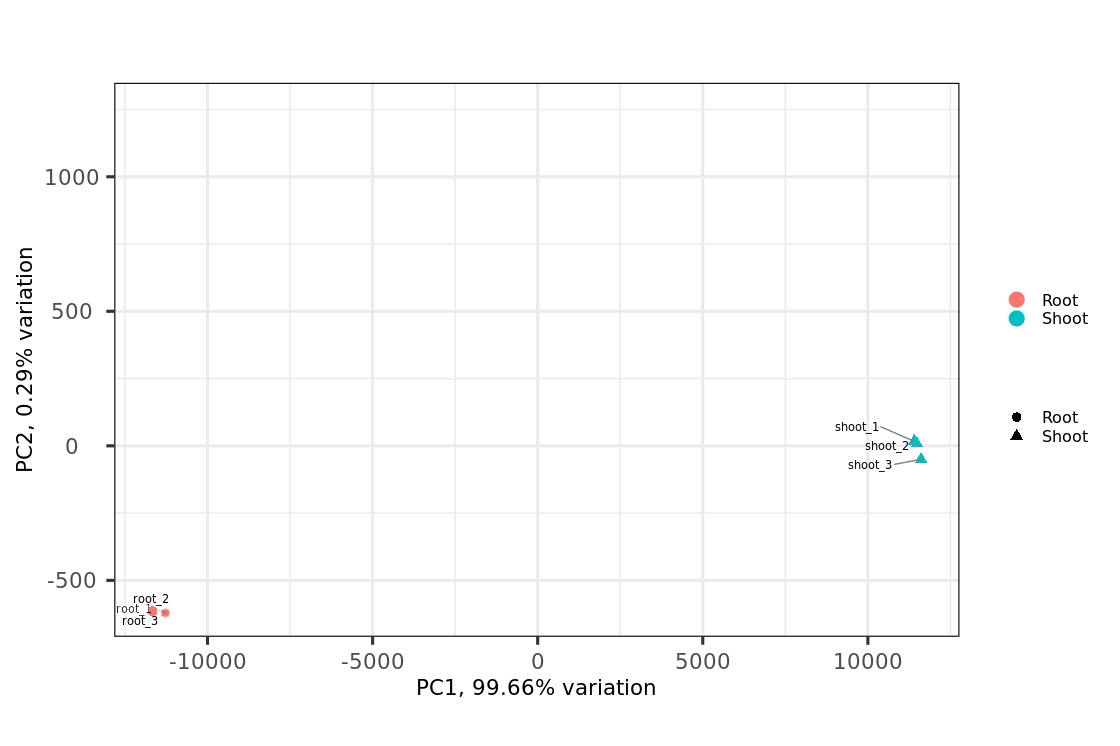


Fig. S9 Principal component analysis for root and shoot samples. Root (root_1,root_2,root_3) and shoot (shoot_1,shoot_2,shoot_3) samples were performed in triplicate.


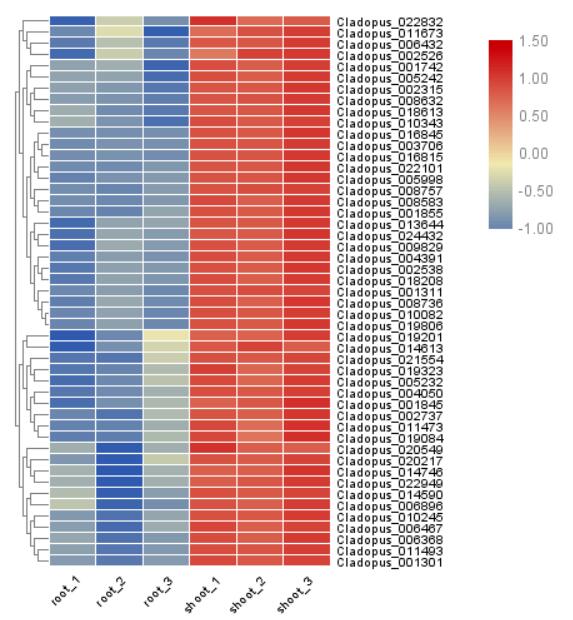


Fig. S10 Heatmap showing the top 50 shoot-specific expressed genes.


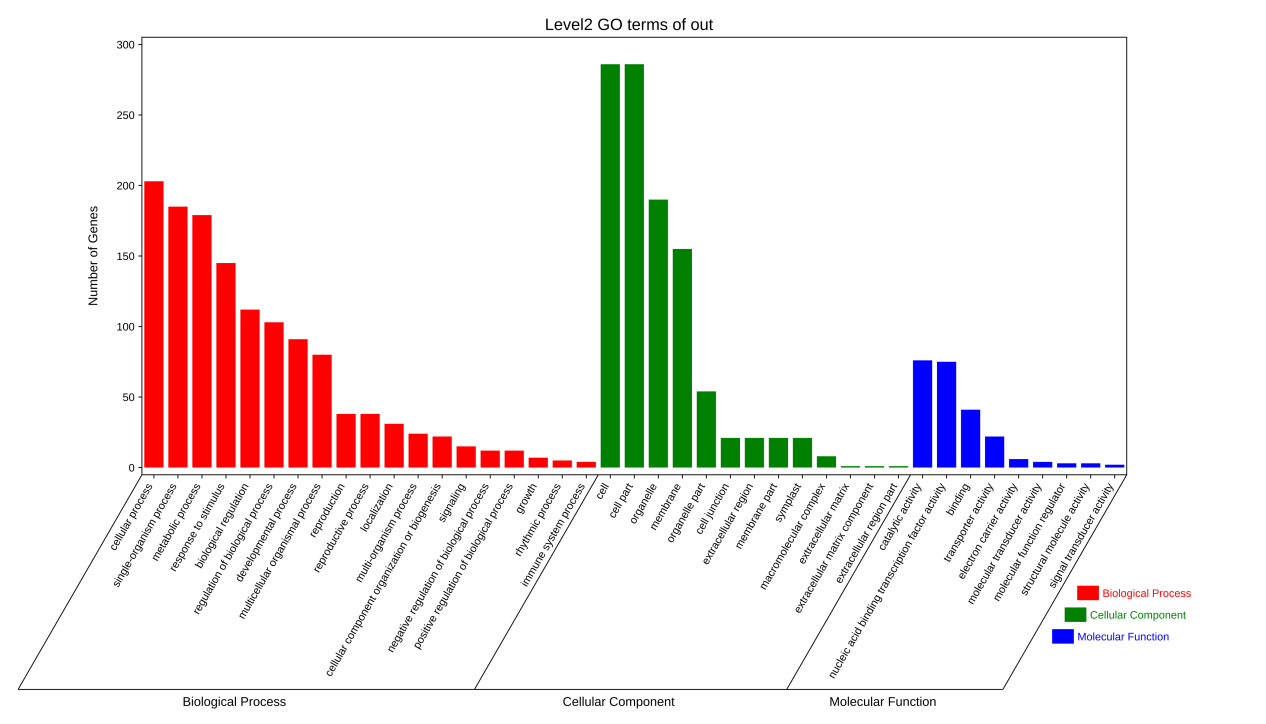


Fig. S11 GO enrichment of the 609 specific expressed genes in the shoot of *C. chinensis*


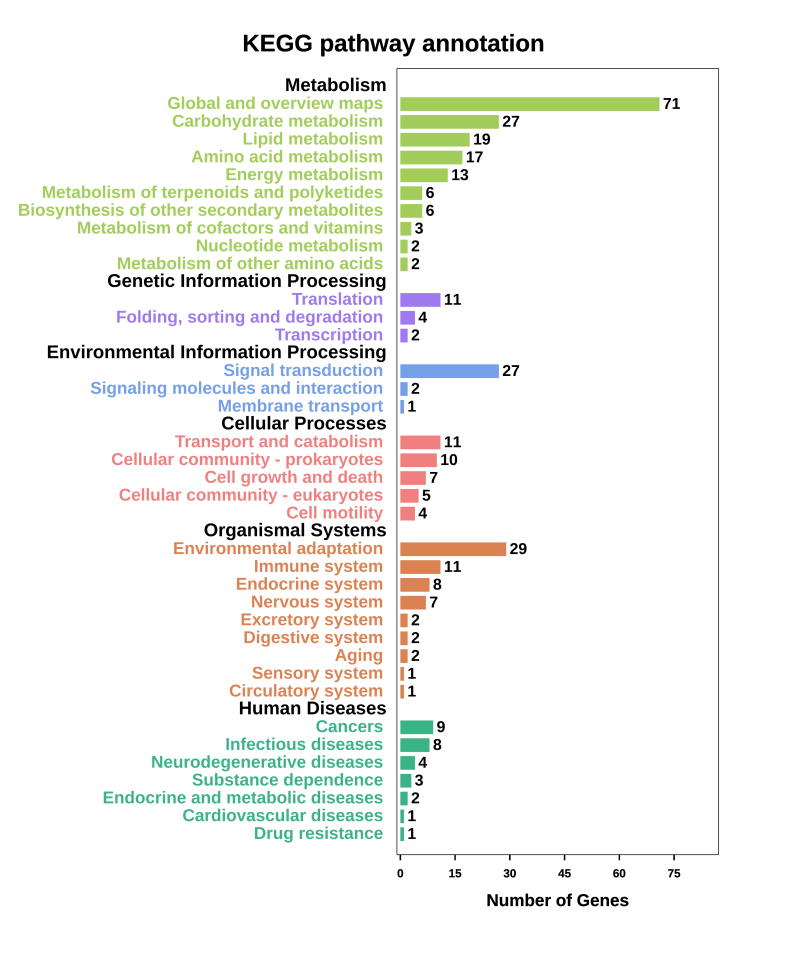


Fig. S12 KEGG enrichment of the 609 specific expressed genes in the shoot of *C. chinensis*

Fig. S13 Real-time quantitative RT-qPCR confirmation of 9 genes at the root and shoot. Relative gene expressions were analyzed using the 2^−ΔΔCt^ method. The expression values were adjusted by setting the expression of root to be 1 for each gene.


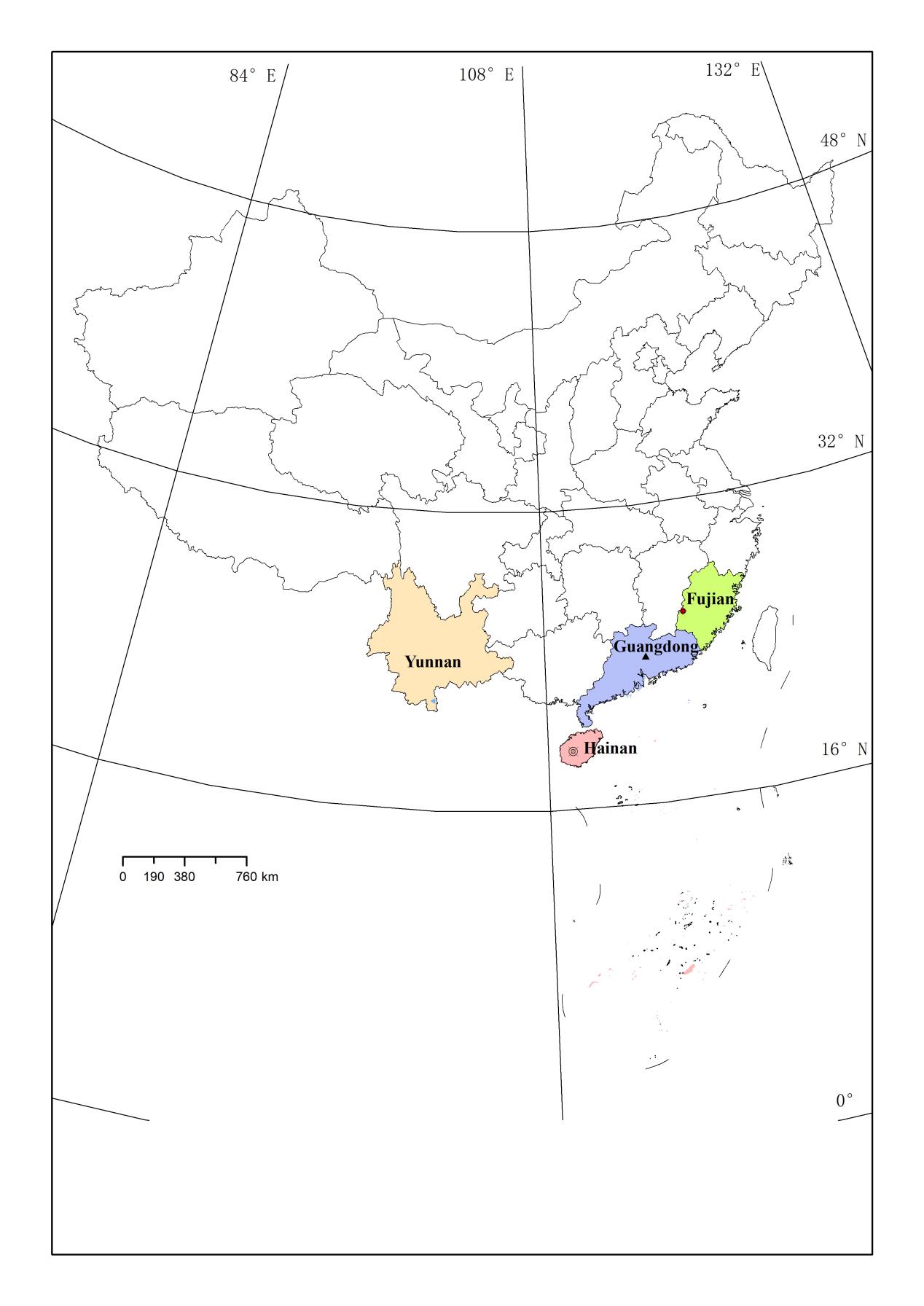


Fig. S14 Distribution of 6 currently known species of *Cladopus, Terniopsis* and *Hydrobryum* in China*.* ●*Cladopus chinensis*;▲*Cladopus nymanii*;◎*Cladopus yinggelingensis*;╋*Terniopsis sessilis*;╳*Terniopsis daoyinensis*; ◄*Hydrobryum griffithii*


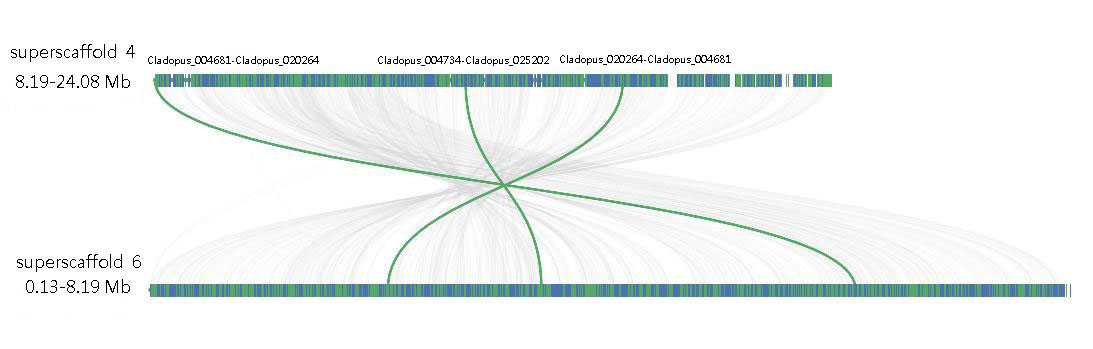


Fig. S15 Syntenic analysis of NAC29 (Cladopus_020264), NAC47 (Cladopus_004681) and NAC83 (Cladopus_004734) flanking region. NAC gene paris were marked by green line.


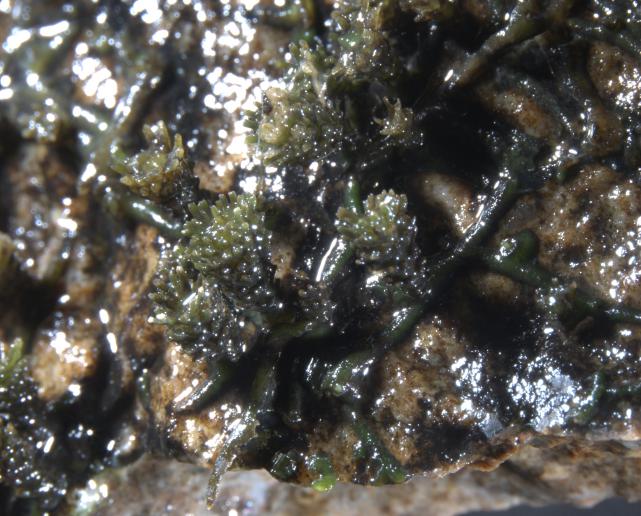


Fig. S16 Whole plant of *Cladopus chinensis* (Podostemaceae) grow submerged on rock surfaces.


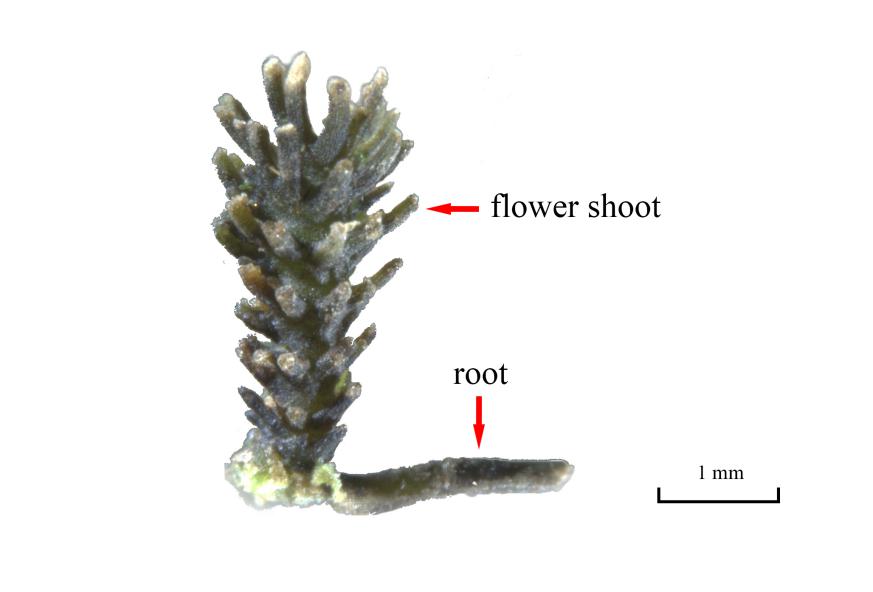


Fig. S17 Root and shoot of *Cladopus chinensis* (Podostemaceae)
