# Supplementary material for: A high-quality genome provides insights into the new taxonomic status and genomic characteristics of Cladopus chinensis (Podostemaceae)
Source: Hortic Res. 2020 Apr 1;7:46. doi: 10.1038/s41438-020-0269-5 (PMC7109043; doi:10.1038/s41438-020-0269-5)
Supplement: Supplementary file 4 — Table S15 Statistics of KEGG enrichment of the 5636 gene families in the C. chinensis [file 41438_2020_269_MOESM4_ESM.pdf]

OG	GO class	GO description	Number of genes
OG0004207	Biological Process	multi-organism process(GO:0051704)	1
OG0004207	Biological Process	response to stimulus(GO:0050896)	1
OG0005149	Biological Process	biological regulation(GO:0065007)	1
OG0005149	Biological Process	cellular process(GO:0009987)	1
OG0005149	Biological Process	metabolic process(GO:0008152)	1
OG0005149	Biological Process	multi-organism process(GO:0051704)	1
OG0005149	Biological Process	positive regulation of biological process(GO:0048518)	1
OG0005149	Biological Process	regulation of biological process(GO:0050789)	1
OG0005149	Biological Process	response to stimulus(GO:0050896)	1
OG0005149	Biological Process	signaling(GO:0023052)	1
OG0011836	Biological Process	cellular component organization or biogenesis(GO:0071840)	5
OG0011836	Biological Process	cellular process(GO:0009987)	5
OG0011836	Biological Process	metabolic process(GO:0008152)	5
OG0011855	Biological Process	cellular process(GO:0009987)	1
OG0011855	Biological Process	metabolic process(GO:0008152)	1
OG0012548	Biological Process	biological regulation(GO:0065007)	1
OG0012548	Biological Process	cellular process(GO:0009987)	1
OG0012548	Biological Process	metabolic process(GO:0008152)	1
OG0012548	Biological Process	regulation of biological process(GO:0050789)	1
OG0013086	Biological Process	biological regulation(GO:0065007)	3
OG0013086	Biological Process	cellular process(GO:0009987)	3
OG0013086	Biological Process	metabolic process(GO:0008152)	3
OG0013086	Biological Process	negative regulation of biological process(GO:0048519)	3
OG0013086	Biological Process	regulation of biological process(GO:0050789)	3
OG0013086	Biological Process	response to stimulus(GO:0050896)	3
OG0013092	Biological Process	biological regulation(GO:0065007)	3
OG0013092	Biological Process	cellular process(GO:0009987)	3
OG0013092	Biological Process	metabolic process(GO:0008152)	3
OG0013092	Biological Process	regulation of biological process(GO:0050789)	3
OG0013101	Biological Process	biological adhesion(GO:0022610)	2
OG0013101	Biological Process	biological regulation(GO:0065007)	2
OG0013101	Biological Process	cell proliferation(GO:0008283)	2
OG0013101	Biological Process	cellular component organization or biogenesis(GO:0071840)	2
OG0013101	Biological Process	cellular process(GO:0009987)	2
OG0013101	Biological Process	developmental process(GO:0032502)	2
OG0013101	Biological Process	growth(GO:0040007)	2
OG0013101	Biological Process	immune system process(GO:0002376)	2
OG0013101	Biological Process	localization(GO:0051179)	2
OG0013101	Biological Process	locomotion(GO:0040011)	2
OG0013101	Biological Process	metabolic process(GO:0008152)	2
OG0013101	Biological Process	multi-organism process(GO:0051704)	2
OG0013101	Biological Process	multicellular organismal process(GO:0032501)	2
OG0013101	Biological Process	negative regulation of biological process(GO:0048519)	2
OG0013101	Biological Process	positive regulation of biological process(GO:0048518)	2

OG0013101	Biological Process	regulation of biological process(GO:0050789)	2
OG0013101	Biological Process	reproduction(GO:0000003)	2
OG0013101	Biological Process	reproductive process(GO:0022414)	2
OG0013101	Biological Process	response to stimulus(GO:0050896)	2
OG0013101	Biological Process	signaling(GO:0023052)	2
OG0013110	Biological Process	metabolic process(GO:0008152)	2
OG0013555	Biological Process	cellular process(GO:0009987)	1
OG0013555	Biological Process	metabolic process(GO:0008152)	1
OG0013563	Biological Process	cellular component organization or biogenesis(GO:0071840)	2
OG0013563	Biological Process	cellular process(GO:0009987)	2
OG0013567	Biological Process	biological regulation(GO:0065007)	2
OG0013567	Biological Process	cellular process(GO:0009987)	2
OG0013567	Biological Process	metabolic process(GO:0008152)	2
OG0013567	Biological Process	regulation of biological process(GO:0050789)	2
OG0013576	Biological Process	biological regulation(GO:0065007)	1
OG0013576	Biological Process	cellular process(GO:0009987)	1
OG0013576	Biological Process	developmental process(GO:0032502)	1
OG0013576	Biological Process	metabolic process(GO:0008152)	1
OG0013576	Biological Process	multicellular organismal process(GO:0032501)	1
OG0013576	Biological Process	regulation of biological process(GO:0050789)	1
OG0013576	Biological Process	reproduction(GO:0000003)	1
OG0013576	Biological Process	reproductive process(GO:0022414)	1
OG0013579	Biological Process	biological regulation(GO:0065007)	2
OG0013579	Biological Process	cellular component organization or biogenesis(GO:0071840)	2
OG0013579	Biological Process	cellular process(GO:0009987)	2
OG0013579	Biological Process	developmental process(GO:0032502)	2
OG0013579	Biological Process	metabolic process(GO:0008152)	2
OG0013579	Biological Process	multicellular organismal process(GO:0032501)	2
OG0013579	Biological Process	negative regulation of biological process(GO:0048519)	2
OG0013579	Biological Process	regulation of biological process(GO:0050789)	2
OG0013589	Biological Process	developmental process(GO:0032502)	2
OG0013589	Biological Process	multicellular organismal process(GO:0032501)	2
OG0013589	Biological Process	reproduction(GO:0000003)	2
OG0013589	Biological Process	reproductive process(GO:0022414)	2
OG0013594	Biological Process	developmental process(GO:0032502)	2
OG0013594	Biological Process	multicellular organismal process(GO:0032501)	2
OG0013594	Biological Process	reproduction(GO:0000003)	2
OG0013594	Biological Process	reproductive process(GO:0022414)	2
OG0013595	Biological Process	cellular component organization or biogenesis(GO:0071840)	2
OG0013595	Biological Process	cellular process(GO:0009987)	2
OG0013595	Biological Process	metabolic process(GO:0008152)	2
OG0013595	Biological Process	reproduction(GO:0000003)	2
OG0013595	Biological Process	reproductive process(GO:0022414)	2
OG0013596	Biological Process	cellular component organization or biogenesis(GO:0071840)	2

OG0013596	Biological Process	cellular process(GO:0009987)	2
OG0013600	Biological Process	biological regulation(GO:0065007)	1
OG0013600	Biological Process	cellular process(GO:0009987)	1
OG0013600	Biological Process	metabolic process(GO:0008152)	1
OG0013600	Biological Process	regulation of biological process(GO:0050789)	1
OG0013609	Biological Process	biological regulation(GO:0065007)	1
OG0013609	Biological Process	developmental process(GO:0032502)	1
OG0013609	Biological Process	growth(GO:0040007)	1
OG0013609	Biological Process	multicellular organismal process(GO:0032501)	1
OG0013609	Biological Process	regulation of biological process(GO:0050789)	1
OG0013612	Biological Process	biological regulation(GO:0065007)	2
OG0013612	Biological Process	cellular process(GO:0009987)	2
OG0013612	Biological Process	developmental process(GO:0032502)	2
OG0013612	Biological Process	metabolic process(GO:0008152)	2
OG0013612	Biological Process	multicellular organismal process(GO:0032501)	2
OG0013612	Biological Process	negative regulation of biological process(GO:0048519)	2
OG0013612	Biological Process	regulation of biological process(GO:0050789)	2
OG0013612	Biological Process	response to stimulus(GO:0050896)	2
OG0013612	Biological Process	signaling(GO:0023052)	2
OG0013622	Biological Process	biological regulation(GO:0065007)	2
OG0013622	Biological Process	cellular process(GO:0009987)	2
OG0013622	Biological Process	developmental process(GO:0032502)	2
OG0013622	Biological Process	metabolic process(GO:0008152)	2
OG0013622	Biological Process	multicellular organismal process(GO:0032501)	2
OG0013622	Biological Process	regulation of biological process(GO:0050789)	2
OG0013627	Biological Process	response to stimulus(GO:0050896)	2
OG0013632	Biological Process	biological regulation(GO:0065007)	2
OG0013632	Biological Process	cellular process(GO:0009987)	2
OG0013632	Biological Process	metabolic process(GO:0008152)	2
OG0013632	Biological Process	regulation of biological process(GO:0050789)	2
OG0013633	Biological Process	biological regulation(GO:0065007)	2
OG0013633	Biological Process	cellular component organization or biogenesis(GO:0071840)	2
OG0013633	Biological Process	cellular process(GO:0009987)	2
OG0013633	Biological Process	developmental process(GO:0032502)	2
OG0013633	Biological Process	metabolic process(GO:0008152)	2
OG0013633	Biological Process	multicellular organismal process(GO:0032501)	2
OG0013633	Biological Process	negative regulation of biological process(GO:0048519)	2
OG0013633	Biological Process	regulation of biological process(GO:0050789)	2
OG0013633	Biological Process	reproduction(GO:0000003)	2
OG0013633	Biological Process	reproductive process(GO:0022414)	2
OG0013636	Biological Process	biological regulation(GO:0065007)	1
OG0013636	Biological Process	cellular process(GO:0009987)	1
OG0013636	Biological Process	developmental process(GO:0032502)	1
OG0013636	Biological Process	immune system process(GO:0002376)	1

OG0013636	Biological Process	metabolic process(GO:0008152)	1
OG0013636	Biological Process	multi-organism process(GO:0051704)	1
OG0013636	Biological Process	multicellular organismal process(GO:0032501)	1
OG0013636	Biological Process	negative regulation of biological process(GO:0048519)	1
OG0013636	Biological Process	regulation of biological process(GO:0050789)	1
OG0013636	Biological Process	reproduction(GO:0000003)	1
OG0013636	Biological Process	reproductive process(GO:0022414)	1
OG0013636	Biological Process	response to stimulus(GO:0050896)	1
OG0013637	Biological Process	developmental process(GO:0032502)	1
OG0013637	Biological Process	locomotion(GO:0040011)	1
OG0013637	Biological Process	multi-organism process(GO:0051704)	1
OG0013637	Biological Process	multicellular organismal process(GO:0032501)	1
OG0013637	Biological Process	reproduction(GO:0000003)	1
OG0013637	Biological Process	reproductive process(GO:0022414)	1
OG0013637	Biological Process	response to stimulus(GO:0050896)	1
OG0013638	Biological Process	biological adhesion(GO:0022610)	1
OG0013638	Biological Process	biological regulation(GO:0065007)	1
OG0013638	Biological Process	cell proliferation(GO:0008283)	1
OG0013638	Biological Process	cellular component organization or biogenesis(GO:0071840)	1
OG0013638	Biological Process	cellular process(GO:0009987)	1
OG0013638	Biological Process	developmental process(GO:0032502)	1
OG0013638	Biological Process	growth(GO:0040007)	1
OG0013638	Biological Process	localization(GO:0051179)	1
OG0013638	Biological Process	locomotion(GO:0040011)	1
OG0013638	Biological Process	metabolic process(GO:0008152)	1
OG0013638	Biological Process	multi-organism process(GO:0051704)	1
OG0013638	Biological Process	multicellular organismal process(GO:0032501)	1
OG0013638	Biological Process	negative regulation of biological process(GO:0048519)	1
OG0013638	Biological Process	positive regulation of biological process(GO:0048518)	1
OG0013638	Biological Process	regulation of biological process(GO:0050789)	1
OG0013638	Biological Process	reproduction(GO:0000003)	1
OG0013638	Biological Process	reproductive process(GO:0022414)	1
OG0013638	Biological Process	response to stimulus(GO:0050896)	1
OG0013638	Biological Process	signaling(GO:0023052)	1
OG0013656	Biological Process	biological regulation(GO:0065007)	2
OG0013656	Biological Process	cellular process(GO:0009987)	2
OG0013656	Biological Process	growth(GO:0040007)	2
OG0013656	Biological Process	metabolic process(GO:0008152)	2
OG0013656	Biological Process	regulation of biological process(GO:0050789)	2
OG0027162	Biological Process	cellular component organization or biogenesis(GO:0071840)	1
OG0027162	Biological Process	cellular process(GO:0009987)	1
OG0027162	Biological Process	growth(GO:0040007)	1
OG0027162	Biological Process	metabolic process(GO:0008152)	1
OG0027163	Biological Process	cellular process(GO:0009987)	1
OG0027163	Biological Process	metabolic process(GO:0008152)	1

OG0027167	Biological Process	growth(GO:0040007)	1
OG0027176	Biological Process	cellular process(GO:0009987)	1
OG0027176	Biological Process	metabolic process(GO:0008152)	1
OG0027184	Biological Process	biological regulation(GO:0065007)	1
OG0027184	Biological Process	cellular component organization or biogenesis(GO:0071840)	1
OG0027184	Biological Process	cellular process(GO:0009987)	1
OG0027184	Biological Process	developmental process(GO:0032502)	1
OG0027184	Biological Process	immune system process(GO:0002376)	1
OG0027184	Biological Process	metabolic process(GO:0008152)	1
OG0027184	Biological Process	multi-organism process(GO:0051704)	1
OG0027184	Biological Process	multicellular organismal process(GO:0032501)	1
OG0027184	Biological Process	regulation of biological process(GO:0050789)	1
OG0027184	Biological Process	reproduction(GO:0000003)	1
OG0027184	Biological Process	reproductive process(GO:0022414)	1
OG0027184	Biological Process	response to stimulus(GO:0050896)	1
OG0027184	Biological Process	signaling(GO:0023052)	1
OG0027185	Biological Process	biological regulation(GO:0065007)	1
OG0027185	Biological Process	cellular component organization or biogenesis(GO:0071840)	1
OG0027185	Biological Process	cellular process(GO:0009987)	1
OG0027185	Biological Process	metabolic process(GO:0008152)	1
OG0027185	Biological Process	positive regulation of biological process(GO:0048518)	1
OG0027185	Biological Process	regulation of biological process(GO:0050789)	1
OG0027186	Biological Process	cellular process(GO:0009987)	1
OG0027186	Biological Process	developmental process(GO:0032502)	1
OG0027186	Biological Process	metabolic process(GO:0008152)	1
OG0027186	Biological Process	multicellular organismal process(GO:0032501)	1
OG0027186	Biological Process	reproduction(GO:0000003)	1
OG0027186	Biological Process	reproductive process(GO:0022414)	1
OG0027199	Biological Process	cellular process(GO:0009987)	1
OG0027199	Biological Process	localization(GO:0051179)	1
OG0027200	Biological Process	cellular process(GO:0009987)	1
OG0027200	Biological Process	localization(GO:0051179)	1
OG0027201	Biological Process	cellular process(GO:0009987)	1
OG0027201	Biological Process	metabolic process(GO:0008152)	1
OG0027207	Biological Process	cellular component organization or biogenesis(GO:0071840)	1
OG0027207	Biological Process	cellular process(GO:0009987)	1
OG0027207	Biological Process	developmental process(GO:0032502)	1
OG0027207	Biological Process	multicellular organismal process(GO:0032501)	1
OG0027210	Biological Process	cellular process(GO:0009987)	1
OG0027210	Biological Process	metabolic process(GO:0008152)	1
OG0027213	Biological Process	cellular process(GO:0009987)	1
OG0027213	Biological Process	metabolic process(GO:0008152)	1
OG0027214	Biological Process	cellular process(GO:0009987)	1
OG0027214	Biological Process	localization(GO:0051179)	1
OG0027218	Biological Process	biological regulation(GO:0065007)	1

OG0027218	Biological Process	cellular component organization or biogenesis(GO:0071840)	1
OG0027218	Biological Process	cellular process(GO:0009987)	1
OG0027218	Biological Process	developmental process(GO:0032502)	1
OG0027218	Biological Process	growth(GO:0040007)	1
OG0027218	Biological Process	immune system process(GO:0002376)	1
OG0027218	Biological Process	localization(GO:0051179)	1
OG0027218	Biological Process	locomotion(GO:0040011)	1
OG0027218	Biological Process	metabolic process(GO:0008152)	1
OG0027218	Biological Process	multi-organism process(GO:0051704)	1
OG0027218	Biological Process	multicellular organismal process(GO:0032501)	1
OG0027218	Biological Process	negative regulation of biological process(GO:0048519)	1
OG0027218	Biological Process	positive regulation of biological process(GO:0048518)	1
OG0027218	Biological Process	regulation of biological process(GO:0050789)	1
OG0027218	Biological Process	reproduction(GO:0000003)	1
OG0027218	Biological Process	reproductive process(GO:0022414)	1
OG0027218	Biological Process	response to stimulus(GO:0050896)	1
OG0027218	Biological Process	signaling(GO:0023052)	1
OG0027219	Biological Process	biological regulation(GO:0065007)	1
OG0027219	Biological Process	cellular process(GO:0009987)	1
OG0027219	Biological Process	metabolic process(GO:0008152)	1
OG0027219	Biological Process	regulation of biological process(GO:0050789)	1
OG0027225	Biological Process	cellular component organization or biogenesis(GO:0071840)	1
OG0027225	Biological Process	cellular process(GO:0009987)	1
OG0027225	Biological Process	metabolic process(GO:0008152)	1
OG0027226	Biological Process	cellular process(GO:0009987)	1
OG0027226	Biological Process	growth(GO:0040007)	1
OG0027226	Biological Process	metabolic process(GO:0008152)	1
OG0027226	Biological Process	response to stimulus(GO:0050896)	1
OG0027227	Biological Process	cellular process(GO:0009987)	1
OG0027227	Biological Process	growth(GO:0040007)	1
OG0027227	Biological Process	metabolic process(GO:0008152)	1
OG0027237	Biological Process	cellular process(GO:0009987)	1
OG0027237	Biological Process	metabolic process(GO:0008152)	1
OG0027238	Biological Process	cellular process(GO:0009987)	1
OG0027238	Biological Process	metabolic process(GO:0008152)	1
OG0027240	Biological Process	cellular process(GO:0009987)	1
OG0027240	Biological Process	metabolic process(GO:0008152)	1
OG0027242	Biological Process	cellular process(GO:0009987)	1
OG0027242	Biological Process	metabolic process(GO:0008152)	1
OG0027243	Biological Process	biological regulation(GO:0065007)	1
OG0027243	Biological Process	cellular process(GO:0009987)	1
OG0027243	Biological Process	metabolic process(GO:0008152)	1
OG0027243	Biological Process	negative regulation of biological process(GO:0048519)	1
OG0027243	Biological Process	regulation of biological process(GO:0050789)	1
OG0027246	Biological Process	cellular component organization or biogenesis(GO:0071840)	1

OG0027246	Biological Process	cellular process(GO:0009987)	1
OG0027246	Biological Process	growth(GO:0040007)	1
OG0027246	Biological Process	metabolic process(GO:0008152)	1
OG0027251	Biological Process	cellular process(GO:0009987)	1
OG0027251	Biological Process	metabolic process(GO:0008152)	1
OG0027252	Biological Process	growth(GO:0040007)	1
OG0027253	Biological Process	cellular component organization or biogenesis(GO:0071840)	1
OG0027253	Biological Process	cellular process(GO:0009987)	1
OG0027253	Biological Process	growth(GO:0040007)	1
OG0027253	Biological Process	metabolic process(GO:0008152)	1
OG0027253	Biological Process	multi-organism process(GO:0051704)	1
OG0027253	Biological Process	response to stimulus(GO:0050896)	1
OG0027254	Biological Process	growth(GO:0040007)	1
OG0027260	Biological Process	cellular process(GO:0009987)	1
OG0027260	Biological Process	metabolic process(GO:0008152)	1
OG0027262	Biological Process	cellular process(GO:0009987)	1
OG0027262	Biological Process	metabolic process(GO:0008152)	1
OG0027292	Biological Process	biological regulation(GO:0065007)	1
OG0027292	Biological Process	cell proliferation(GO:0008283)	1
OG0027292	Biological Process	cellular process(GO:0009987)	1
OG0027292	Biological Process	developmental process(GO:0032502)	1
OG0027292	Biological Process	metabolic process(GO:0008152)	1
OG0027292	Biological Process	multicellular organismal process(GO:0032501)	1
OG0027292	Biological Process	positive regulation of biological process(GO:0048518)	1
OG0027292	Biological Process	regulation of biological process(GO:0050789)	1
OG0027303	Biological Process	cellular process(GO:0009987)	1
OG0027303	Biological Process	metabolic process(GO:0008152)	1
OG0027303	Biological Process	response to stimulus(GO:0050896)	1
OG0027309	Biological Process	response to stimulus(GO:0050896)	1
OG0027312	Biological Process	biological regulation(GO:0065007)	1
OG0027312	Biological Process	cellular process(GO:0009987)	1
OG0027312	Biological Process	metabolic process(GO:0008152)	1
OG0027312	Biological Process	regulation of biological process(GO:0050789)	1
OG0027312	Biological Process	response to stimulus(GO:0050896)	1
OG0027316	Biological Process	cellular process(GO:0009987)	1
OG0027316	Biological Process	growth(GO:0040007)	1
OG0027316	Biological Process	localization(GO:0051179)	1
OG0027318	Biological Process	cellular component organization or biogenesis(GO:0071840)	1
OG0027318	Biological Process	cellular process(GO:0009987)	1
OG0027318	Biological Process	growth(GO:0040007)	1
OG0027318	Biological Process	localization(GO:0051179)	1
OG0027318	Biological Process	metabolic process(GO:0008152)	1
OG0027322	Biological Process	cellular component organization or biogenesis(GO:0071840)	1
OG0027322	Biological Process	cellular process(GO:0009987)	1
OG0027322	Biological Process	metabolic process(GO:0008152)	1
OG0027323	Biological Process	cellular process(GO:0009987)	1

OG0027323	Biological Process	growth(GO:0040007)	1
OG0027323	Biological Process	metabolic process(GO:0008152)	1
OG0027328	Biological Process	cellular process(GO:0009987)	1
OG0027328	Biological Process	growth(GO:0040007)	1
OG0027328	Biological Process	metabolic process(GO:0008152)	1
OG0027329	Biological Process	metabolic process(GO:0008152)	1
OG0027330	Biological Process	cellular process(GO:0009987)	1
OG0027330	Biological Process	growth(GO:0040007)	1
OG0027330	Biological Process	metabolic process(GO:0008152)	1
OG0027340	Biological Process	cellular process(GO:0009987)	1
OG0027340	Biological Process	metabolic process(GO:0008152)	1
OG0027345	Biological Process	biological regulation(GO:0065007)	1
OG0027345	Biological Process	cellular component organization or biogenesis(GO:0071840)	1
OG0027345	Biological Process	cellular process(GO:0009987)	1
OG0027345	Biological Process	immune system process(GO:0002376)	1
OG0027345	Biological Process	localization(GO:0051179)	1
OG0027345	Biological Process	regulation of biological process(GO:0050789)	1
OG0027345	Biological Process	response to stimulus(GO:0050896)	1
OG0027348	Biological Process	biological regulation(GO:0065007)	1
OG0027348	Biological Process	metabolic process(GO:0008152)	1
OG0027348	Biological Process	response to stimulus(GO:0050896)	1
OG0027352	Biological Process	biological regulation(GO:0065007)	1
OG0027352	Biological Process	cellular process(GO:0009987)	1
OG0027352	Biological Process	metabolic process(GO:0008152)	1
OG0027352	Biological Process	regulation of biological process(GO:0050789)	1
OG0027353	Biological Process	developmental process(GO:0032502)	1
OG0027353	Biological Process	multicellular organismal process(GO:0032501)	1
OG0027353	Biological Process	reproduction(GO:0000003)	1
OG0027353	Biological Process	reproductive process(GO:0022414)	1
OG0027369	Biological Process	response to stimulus(GO:0050896)	1
OG0027376	Biological Process	biological regulation(GO:0065007)	1
OG0027376	Biological Process	cellular process(GO:0009987)	1
OG0027376	Biological Process	developmental process(GO:0032502)	1
OG0027376	Biological Process	growth(GO:0040007)	1
OG0027376	Biological Process	immune system process(GO:0002376)	1
OG0027376	Biological Process	localization(GO:0051179)	1
OG0027376	Biological Process	metabolic process(GO:0008152)	1
OG0027376	Biological Process	multi-organism process(GO:0051704)	1
OG0027376	Biological Process	multicellular organismal process(GO:0032501)	1
OG0027376	Biological Process	negative regulation of biological process(GO:0048519)	1
OG0027376	Biological Process	regulation of biological process(GO:0050789)	1
OG0027376	Biological Process	reproduction(GO:0000003)	1
OG0027376	Biological Process	reproductive process(GO:0022414)	1
OG0027379	Biological Process	biological regulation(GO:0065007)	1
OG0027379	Biological Process	cell proliferation(GO:0008283)	1
OG0027379	Biological Process	cellular process(GO:0009987)	1

OG0027379	Biological Process	developmental process(GO:0032502)	1
OG0027379	Biological Process	metabolic process(GO:0008152)	1
OG0027379	Biological Process	multicellular organismal process(GO:0032501)	1
OG0027379	Biological Process	negative regulation of biological process(GO:0048519)	1
OG0027379	Biological Process	positive regulation of biological process(GO:0048518)	1
OG0027379	Biological Process	regulation of biological process(GO:0050789)	1
OG0027379	Biological Process	reproduction(GO:0000003)	1
OG0027379	Biological Process	reproductive process(GO:0022414)	1
OG0027379	Biological Process	response to stimulus(GO:0050896)	1
OG0027380	Biological Process	biological regulation(GO:0065007)	1
OG0027380	Biological Process	cellular process(GO:0009987)	1
OG0027380	Biological Process	developmental process(GO:0032502)	1
OG0027380	Biological Process	localization(GO:0051179)	1
OG0027380	Biological Process	metabolic process(GO:0008152)	1
OG0027380	Biological Process	multicellular organismal process(GO:0032501)	1
OG0027380	Biological Process	regulation of biological process(GO:0050789)	1
OG0027380	Biological Process	response to stimulus(GO:0050896)	1
OG0027389	Biological Process	cellular component organization or biogenesis(GO:0071840)	1
OG0027389	Biological Process	cellular process(GO:0009987)	1
OG0027389	Biological Process	metabolic process(GO:0008152)	1
OG0027396	Biological Process	biological regulation(GO:0065007)	1
OG0027396	Biological Process	cellular process(GO:0009987)	1
OG0027396	Biological Process	metabolic process(GO:0008152)	1
OG0027396	Biological Process	regulation of biological process(GO:0050789)	1
OG0027400	Biological Process	cellular process(GO:0009987)	1
OG0027400	Biological Process	metabolic process(GO:0008152)	1
OG0027400	Biological Process	response to stimulus(GO:0050896)	1
OG0027402	Biological Process	cellular process(GO:0009987)	1
OG0027402	Biological Process	metabolic process(GO:0008152)	1
OG0027410	Biological Process	cellular process(GO:0009987)	1
OG0027410	Biological Process	growth(GO:0040007)	1
OG0027410	Biological Process	metabolic process(GO:0008152)	1
OG0027411	Biological Process	cellular process(GO:0009987)	1
OG0027411	Biological Process	metabolic process(GO:0008152)	1
OG0027417	Biological Process	cellular process(GO:0009987)	1
OG0027417	Biological Process	metabolic process(GO:0008152)	1
OG0027418	Biological Process	cellular process(GO:0009987)	1
OG0027418	Biological Process	growth(GO:0040007)	1
OG0027418	Biological Process	metabolic process(GO:0008152)	1
OG0027419	Biological Process	cellular process(GO:0009987)	1
OG0027419	Biological Process	metabolic process(GO:0008152)	1
OG0027432	Biological Process	developmental process(GO:0032502)	1
OG0027432	Biological Process	multicellular organismal process(GO:0032501)	1
OG0027432	Biological Process	response to stimulus(GO:0050896)	1
OG0027433	Biological Process	cellular process(GO:0009987)	1

OG0027433	Biological Process	metabolic process(GO:0008152)	1
OG0027439	Biological Process	response to stimulus(GO:0050896)	1
OG0027441	Biological Process	biological regulation(GO:0065007)	1
OG0027441	Biological Process	cellular process(GO:0009987)	1
OG0027441	Biological Process	regulation of biological process(GO:0050789)	1
OG0027441	Biological Process	response to stimulus(GO:0050896)	1
OG0027441	Biological Process	signaling(GO:0023052)	1
OG0027442	Biological Process	cellular process(GO:0009987)	1
OG0027442	Biological Process	metabolic process(GO:0008152)	1
OG0027452	Biological Process	biological regulation(GO:0065007)	1
OG0027452	Biological Process	cellular process(GO:0009987)	1
OG0027452	Biological Process	metabolic process(GO:0008152)	1
OG0027452	Biological Process	regulation of biological process(GO:0050789)	1
OG0027455	Biological Process	cellular process(GO:0009987)	1
OG0027455	Biological Process	metabolic process(GO:0008152)	1
OG0027458	Biological Process	biological regulation(GO:0065007)	1
OG0027458	Biological Process	cellular process(GO:0009987)	1
OG0027458	Biological Process	regulation of biological process(GO:0050789)	1
OG0027458	Biological Process	response to stimulus(GO:0050896)	1
OG0027458	Biological Process	signaling(GO:0023052)	1
OG0027461	Biological Process	biological regulation(GO:0065007)	1
OG0027461	Biological Process	cellular process(GO:0009987)	1
OG0027461	Biological Process	metabolic process(GO:0008152)	1
OG0027461	Biological Process	regulation of biological process(GO:0050789)	1
OG0027461	Biological Process	response to stimulus(GO:0050896)	1
OG0027461	Biological Process	signaling(GO:0023052)	1
OG0027474	Biological Process	cellular process(GO:0009987)	1
OG0027474	Biological Process	metabolic process(GO:0008152)	1
OG0027475	Biological Process	cellular process(GO:0009987)	1
OG0027475	Biological Process	metabolic process(GO:0008152)	1
OG0027475	Biological Process	response to stimulus(GO:0050896)	1
OG0027479	Biological Process	biological regulation(GO:0065007)	1
OG0027479	Biological Process	cellular process(GO:0009987)	1
OG0027479	Biological Process	developmental process(GO:0032502)	1
OG0027479	Biological Process	metabolic process(GO:0008152)	1
OG0027479	Biological Process	multicellular organismal process(GO:0032501)	1
OG0027479	Biological Process	regulation of biological process(GO:0050789)	1
OG0027479	Biological Process	response to stimulus(GO:0050896)	1
OG0027483	Biological Process	cellular component organization or biogenesis(GO:0071840)	1
OG0027483	Biological Process	cellular process(GO:0009987)	1
OG0027483	Biological Process	multicellular organismal process(GO:0032501)	1
OG0027483	Biological Process	response to stimulus(GO:0050896)	1
OG0027486	Biological Process	biological regulation(GO:0065007)	1
OG0027486	Biological Process	cellular process(GO:0009987)	1
OG0027486	Biological Process	metabolic process(GO:0008152)	1

OG0027486	Biological Process	regulation of biological process(GO:0050789)	1
OG0027486	Biological Process	response to stimulus(GO:0050896)	1
OG0027486	Biological Process	signaling(GO:0023052)	1
OG0027488	Biological Process	biological regulation(GO:0065007)	1
OG0027488	Biological Process	cellular process(GO:0009987)	1
OG0027488	Biological Process	metabolic process(GO:0008152)	1
OG0027488	Biological Process	regulation of biological process(GO:0050789)	1
OG0027492	Biological Process	cellular component organization or biogenesis(GO:0071840)	1
OG0027492	Biological Process	cellular process(GO:0009987)	1
OG0027492	Biological Process	metabolic process(GO:0008152)	1
OG0027507	Biological Process	cellular component organization or biogenesis(GO:0071840)	1
OG0027507	Biological Process	cellular process(GO:0009987)	1
OG0027507	Biological Process	metabolic process(GO:0008152)	1
OG0027507	Biological Process	response to stimulus(GO:0050896)	1
OG0027510	Biological Process	biological regulation(GO:0065007)	1
OG0027510	Biological Process	cellular component organization or biogenesis(GO:0071840)	1
OG0027510	Biological Process	cellular process(GO:0009987)	1
OG0027510	Biological Process	developmental process(GO:0032502)	1
OG0027510	Biological Process	regulation of biological process(GO:0050789)	1
OG0027510	Biological Process	response to stimulus(GO:0050896)	1
OG0027513	Biological Process	developmental process(GO:0032502)	1
OG0027514	Biological Process	biological regulation(GO:0065007)	1
OG0027514	Biological Process	localization(GO:0051179)	1
OG0027516	Biological Process	biological regulation(GO:0065007)	1
OG0027516	Biological Process	cellular process(GO:0009987)	1
OG0027516	Biological Process	developmental process(GO:0032502)	1
OG0027516	Biological Process	metabolic process(GO:0008152)	1
OG0027516	Biological Process	multicellular organismal process(GO:0032501)	1
OG0027516	Biological Process	regulation of biological process(GO:0050789)	1
OG0027523	Biological Process	cellular component organization or biogenesis(GO:0071840)	1
OG0027523	Biological Process	cellular process(GO:0009987)	1
OG0027523	Biological Process	localization(GO:0051179)	1
OG0027523	Biological Process	metabolic process(GO:0008152)	1
OG0027523	Biological Process	response to stimulus(GO:0050896)	1
OG0027529	Biological Process	cellular process(GO:0009987)	1
OG0027529	Biological Process	metabolic process(GO:0008152)	1
OG0027530	Biological Process	biological regulation(GO:0065007)	1
OG0027530	Biological Process	cellular process(GO:0009987)	1
OG0027530	Biological Process	developmental process(GO:0032502)	1
OG0027530	Biological Process	metabolic process(GO:0008152)	1
OG0027530	Biological Process	multicellular organismal process(GO:0032501)	1
OG0027530	Biological Process	positive regulation of biological process(GO:0048518)	1
OG0027530	Biological Process	regulation of biological process(GO:0050789)	1
OG0027530	Biological Process	reproduction(GO:0000003)	1

OG0027530	Biological Process	reproductive process(GO:0022414)	1
OG0027530	Biological Process	response to stimulus(GO:0050896)	1
OG0027530	Biological Process	signaling(GO:0023052)	1
OG0027532	Biological Process	cellular process(GO:0009987)	1
OG0027532	Biological Process	metabolic process(GO:0008152)	1
OG0027535	Biological Process	biological regulation(GO:0065007)	1
OG0027535	Biological Process	cellular process(GO:0009987)	1
OG0027535	Biological Process	localization(GO:0051179)	1
OG0027535	Biological Process	metabolic process(GO:0008152)	1
OG0027535	Biological Process	negative regulation of biological process(GO:0048519)	1
OG0027535	Biological Process	regulation of biological process(GO:0050789)	1
OG0027537	Biological Process	cellular process(GO:0009987)	1
OG0027537	Biological Process	localization(GO:0051179)	1
OG0027538	Biological Process	cellular process(GO:0009987)	1
OG0027538	Biological Process	metabolic process(GO:0008152)	1
OG0027540	Biological Process	metabolic process(GO:0008152)	1
OG0027540	Biological Process	response to stimulus(GO:0050896)	1
OG0027546	Biological Process	multi-organism process(GO:0051704)	1
OG0027546	Biological Process	response to stimulus(GO:0050896)	1
OG0027565	Biological Process	biological regulation(GO:0065007)	1
OG0027565	Biological Process	cellular component organization or biogenesis(GO:0071840)	1
OG0027565	Biological Process	cellular process(GO:0009987)	1
OG0027565	Biological Process	metabolic process(GO:0008152)	1
OG0027565	Biological Process	positive regulation of biological process(GO:0048518)	1
OG0027565	Biological Process	regulation of biological process(GO:0050789)	1
OG0027565	Biological Process	response to stimulus(GO:0050896)	1
OG0027565	Biological Process	signaling(GO:0023052)	1
OG0027567	Biological Process	cellular process(GO:0009987)	1
OG0027567	Biological Process	metabolic process(GO:0008152)	1
OG0027567	Biological Process	response to stimulus(GO:0050896)	1
OG0027569	Biological Process	cellular process(GO:0009987)	1
OG0027569	Biological Process	metabolic process(GO:0008152)	1
OG0027569	Biological Process	response to stimulus(GO:0050896)	1
OG0027570	Biological Process	cellular process(GO:0009987)	1
OG0027570	Biological Process	metabolic process(GO:0008152)	1
OG0027570	Biological Process	response to stimulus(GO:0050896)	1
OG0027571	Biological Process	cellular process(GO:0009987)	1
OG0027571	Biological Process	metabolic process(GO:0008152)	1
OG0027572	Biological Process	cellular process(GO:0009987)	1
OG0027572	Biological Process	growth(GO:0040007)	1
OG0027572	Biological Process	metabolic process(GO:0008152)	1
OG0027573	Biological Process	cellular process(GO:0009987)	1
OG0027573	Biological Process	metabolic process(GO:0008152)	1
OG0027605	Biological Process	metabolic process(GO:0008152)	1
OG0027606	Biological Process	cellular process(GO:0009987)	1
OG0027606	Biological Process	localization(GO:0051179)	1

OG0027609	Biological Process	cellular process(GO:0009987)	1
OG0027609	Biological Process	metabolic process(GO:0008152)	1
OG0027609	Biological Process	response to stimulus(GO:0050896)	1
OG0027613	Biological Process	cellular process(GO:0009987)	1
OG0027613	Biological Process	metabolic process(GO:0008152)	1
OG0027613	Biological Process	response to stimulus(GO:0050896)	1
OG0027614	Biological Process	biological regulation(GO:0065007)	1
OG0027614	Biological Process	cellular process(GO:0009987)	1
OG0027614	Biological Process	localization(GO:0051179)	1
OG0027614	Biological Process	regulation of biological process(GO:0050789)	1
OG0027614	Biological Process	response to stimulus(GO:0050896)	1
OG0027615	Biological Process	biological regulation(GO:0065007)	1
OG0027615	Biological Process	cellular process(GO:0009987)	1
OG0027615	Biological Process	developmental process(GO:0032502)	1
OG0027615	Biological Process	metabolic process(GO:0008152)	1
OG0027615	Biological Process	multicellular organismal process(GO:0032501)	1
OG0027615	Biological Process	pigmentation(GO:0043473)	1
OG0027615	Biological Process	regulation of biological process(GO:0050789)	1
OG0027615	Biological Process	response to stimulus(GO:0050896)	1
OG0027626	Biological Process	localization(GO:0051179)	1
OG0027626	Biological Process	response to stimulus(GO:0050896)	1
OG0027634	Biological Process	cellular process(GO:0009987)	1
OG0027634	Biological Process	growth(GO:0040007)	1
OG0027634	Biological Process	metabolic process(GO:0008152)	1
OG0027636	Biological Process	cellular process(GO:0009987)	1
OG0027636	Biological Process	metabolic process(GO:0008152)	1
OG0027640	Biological Process	biological regulation(GO:0065007)	1
OG0027640	Biological Process	cellular process(GO:0009987)	1
OG0027640	Biological Process	growth(GO:0040007)	1
OG0027640	Biological Process	metabolic process(GO:0008152)	1
OG0027640	Biological Process	negative regulation of biological process(GO:0048519)	1
OG0027640	Biological Process	regulation of biological process(GO:0050789)	1
OG0027643	Biological Process	cellular process(GO:0009987)	1
OG0027643	Biological Process	growth(GO:0040007)	1
OG0027643	Biological Process	metabolic process(GO:0008152)	1
OG0027644	Biological Process	cellular process(GO:0009987)	1
OG0027644	Biological Process	metabolic process(GO:0008152)	1
OG0027669	Biological Process	cellular component organization or biogenesis(GO:0071840)	1
OG0027669	Biological Process	cellular process(GO:0009987)	1
OG0027669	Biological Process	metabolic process(GO:0008152)	1
OG0027704	Biological Process	biological regulation(GO:0065007)	1
OG0027704	Biological Process	cellular process(GO:0009987)	1
OG0027704	Biological Process	metabolic process(GO:0008152)	1
OG0027704	Biological Process	regulation of biological process(GO:0050789)	1
OG0027704	Biological Process	response to stimulus(GO:0050896)	1
OG0027708	Biological Process	cellular process(GO:0009987)	1

OG0027708	Biological Process	metabolic process(GO:0008152)	1
OG0027708	Biological Process	response to stimulus(GO:0050896)	1
OG0027711	Biological Process	biological regulation(GO:0065007)	1
OG0027711	Biological Process	cellular process(GO:0009987)	1
OG0027711	Biological Process	developmental process(GO:0032502)	1
OG0027711	Biological Process	metabolic process(GO:0008152)	1
OG0027711	Biological Process	multicellular organismal process(GO:0032501)	1
OG0027711	Biological Process	regulation of biological process(GO:0050789)	1
OG0027711	Biological Process	response to stimulus(GO:0050896)	1
OG0027714	Biological Process	cellular process(GO:0009987)	1
OG0027714	Biological Process	metabolic process(GO:0008152)	1
OG0027718	Biological Process	biological regulation(GO:0065007)	1
OG0027718	Biological Process	cellular process(GO:0009987)	1
OG0027718	Biological Process	metabolic process(GO:0008152)	1
OG0027718	Biological Process	regulation of biological process(GO:0050789)	1
OG0027738	Biological Process	cellular process(GO:0009987)	1
OG0027738	Biological Process	developmental process(GO:0032502)	1
OG0027738	Biological Process	localization(GO:0051179)	1
OG0027738	Biological Process	metabolic process(GO:0008152)	1
OG0027738	Biological Process	signaling(GO:0023052)	1
OG0027761	Biological Process	biological regulation(GO:0065007)	1
OG0027761	Biological Process	cellular process(GO:0009987)	1
OG0027761	Biological Process	developmental process(GO:0032502)	1
OG0027761	Biological Process	metabolic process(GO:0008152)	1
OG0027761	Biological Process	multicellular organismal process(GO:0032501)	1
OG0027761	Biological Process	negative regulation of biological process(GO:0048519)	1
OG0027761	Biological Process	regulation of biological process(GO:0050789)	1
OG0027773	Biological Process	cellular process(GO:0009987)	1
OG0027773	Biological Process	metabolic process(GO:0008152)	1
OG0027773	Biological Process	response to stimulus(GO:0050896)	1
OG0027774	Biological Process	cellular process(GO:0009987)	1
OG0027774	Biological Process	metabolic process(GO:0008152)	1
OG0027775	Biological Process	biological regulation(GO:0065007)	1
OG0027775	Biological Process	cellular component organization or biogenesis(GO:0071840)	1
OG0027775	Biological Process	cellular process(GO:0009987)	1
OG0027775	Biological Process	developmental process(GO:0032502)	1
OG0027775	Biological Process	immune system process(GO:0002376)	1
OG0027775	Biological Process	metabolic process(GO:0008152)	1
OG0027775	Biological Process	multi-organism process(GO:0051704)	1
OG0027775	Biological Process	multicellular organismal process(GO:0032501)	1
OG0027775	Biological Process	regulation of biological process(GO:0050789)	1
OG0027775	Biological Process	reproduction(GO:0000003)	1
OG0027775	Biological Process	reproductive process(GO:0022414)	1
OG0027775	Biological Process	response to stimulus(GO:0050896)	1
OG0027775	Biological Process	signaling(GO:0023052)	1

OG0027783	Biological Process	biological regulation(GO:0065007)	1
OG0027783	Biological Process	cellular component organization or biogenesis(GO:0071840)	1
OG0027783	Biological Process	cellular process(GO:0009987)	1
OG0027793	Biological Process	metabolic process(GO:0008152)	1
OG0027793	Biological Process	response to stimulus(GO:0050896)	1
OG0027794	Biological Process	behavior(GO:0007610)	1
OG0027794	Biological Process	biological regulation(GO:0065007)	1
OG0027794	Biological Process	cell proliferation(GO:0008283)	1
OG0027794	Biological Process	cellular component organization or biogenesis(GO:0071840)	1
OG0027794	Biological Process	cellular process(GO:0009987)	1
OG0027794	Biological Process	developmental process(GO:0032502)	1
OG0027794	Biological Process	immune system process(GO:0002376)	1
OG0027794	Biological Process	localization(GO:0051179)	1
OG0027794	Biological Process	locomotion(GO:0040011)	1
OG0027794	Biological Process	metabolic process(GO:0008152)	1
OG0027794	Biological Process	multi-organism process(GO:0051704)	1
OG0027794	Biological Process	multicellular organismal process(GO:0032501)	1
OG0027794	Biological Process	negative regulation of biological process(GO:0048519)	1
OG0027794	Biological Process	positive regulation of biological process(GO:0048518)	1
OG0027794	Biological Process	regulation of biological process(GO:0050789)	1
OG0027794	Biological Process	reproduction(GO:0000003)	1
OG0027794	Biological Process	reproductive process(GO:0022414)	1
OG0027794	Biological Process	response to stimulus(GO:0050896)	1
OG0027794	Biological Process	signaling(GO:0023052)	1
OG0027795	Biological Process	cellular process(GO:0009987)	1
OG0027795	Biological Process	metabolic process(GO:0008152)	1
OG0027795	Biological Process	response to stimulus(GO:0050896)	1
OG0027800	Biological Process	cellular process(GO:0009987)	1
OG0027800	Biological Process	growth(GO:0040007)	1
OG0027800	Biological Process	metabolic process(GO:0008152)	1
OG0027803	Biological Process	biological regulation(GO:0065007)	1
OG0027803	Biological Process	cell killing(GO:0001906)	1
OG0027803	Biological Process	cellular component organization or biogenesis(GO:0071840)	1
OG0027803	Biological Process	cellular process(GO:0009987)	1
OG0027803	Biological Process	metabolic process(GO:0008152)	1
OG0027803	Biological Process	multi-organism process(GO:0051704)	1
OG0027809	Biological Process	growth(GO:0040007)	1
OG0027811	Biological Process	cellular process(GO:0009987)	1
OG0027811	Biological Process	localization(GO:0051179)	1
OG0027811	Biological Process	metabolic process(GO:0008152)	1
OG0027811	Biological Process	response to stimulus(GO:0050896)	1
OG0027812	Biological Process	cellular process(GO:0009987)	1
OG0027812	Biological Process	metabolic process(GO:0008152)	1
OG0027814	Biological Process	cellular process(GO:0009987)	1
OG0027814	Biological Process	metabolic process(GO:0008152)	1
OG0027815	Biological Process	localization(GO:0051179)	1

OG0027818	Biological Process	biological regulation(GO:0065007)	1
OG0027818	Biological Process	cellular process(GO:0009987)	1
OG0027818	Biological Process	metabolic process(GO:0008152)	1
OG0027818	Biological Process	regulation of biological process(GO:0050789)	1
OG0027821	Biological Process	response to stimulus(GO:0050896)	1
OG0027829	Biological Process	response to stimulus(GO:0050896)	1
OG0027837	Biological Process	biological regulation(GO:0065007)	1
OG0027837	Biological Process	cellular process(GO:0009987)	1
OG0027837	Biological Process	metabolic process(GO:0008152)	1
OG0027837	Biological Process	regulation of biological process(GO:0050789)	1
OG0027840	Biological Process	cellular process(GO:0009987)	1
OG0027840	Biological Process	metabolic process(GO:0008152)	1
OG0027840	Biological Process	response to stimulus(GO:0050896)	1
OG0027843	Biological Process	cellular process(GO:0009987)	1
OG0027843	Biological Process	localization(GO:0051179)	1
OG0027843	Biological Process	locomotion(GO:0040011)	1
OG0027843	Biological Process	metabolic process(GO:0008152)	1
OG0027843	Biological Process	response to stimulus(GO:0050896)	1
OG0027844	Biological Process	biological regulation(GO:0065007)	1
OG0027844	Biological Process	cellular component organization or biogenesis(GO:0071840)	1
OG0027844	Biological Process	cellular process(GO:0009987)	1
OG0027844	Biological Process	growth(GO:0040007)	1
OG0027844	Biological Process	metabolic process(GO:0008152)	1
OG0027844	Biological Process	negative regulation of biological process(GO:0048519)	1
OG0027844	Biological Process	regulation of biological process(GO:0050789)	1
OG0027848	Biological Process	cellular process(GO:0009987)	1
OG0027848	Biological Process	metabolic process(GO:0008152)	1
OG0027850	Biological Process	cellular component organization or biogenesis(GO:0071840)	1
OG0027850	Biological Process	cellular process(GO:0009987)	1
OG0027850	Biological Process	metabolic process(GO:0008152)	1
OG0027852	Biological Process	cellular process(GO:0009987)	1
OG0027852	Biological Process	growth(GO:0040007)	1
OG0027852	Biological Process	metabolic process(GO:0008152)	1
OG0027854	Biological Process	cellular process(GO:0009987)	1
OG0027854	Biological Process	metabolic process(GO:0008152)	1
OG0027863	Biological Process	cellular component organization or biogenesis(GO:0071840)	1
OG0027863	Biological Process	cellular process(GO:0009987)	1
OG0027863	Biological Process	developmental process(GO:0032502)	1
OG0027863	Biological Process	multicellular organismal process(GO:0032501)	1
OG0027866	Biological Process	cellular component organization or biogenesis(GO:0071840)	1
OG0027866	Biological Process	cellular process(GO:0009987)	1
OG0027866	Biological Process	metabolic process(GO:0008152)	1
OG0027868	Biological Process	cellular process(GO:0009987)	1
OG0027868	Biological Process	metabolic process(GO:0008152)	1
OG0027868	Biological Process	response to stimulus(GO:0050896)	1

OG0027875	Biological Process	biological regulation(GO:0065007)	1
OG0027875	Biological Process	cellular component organization or biogenesis(GO:0071840)	1
OG0027875	Biological Process	cellular process(GO:0009987)	1
OG0027875	Biological Process	developmental process(GO:0032502)	1
OG0027875	Biological Process	metabolic process(GO:0008152)	1
OG0027875	Biological Process	multicellular organismal process(GO:0032501)	1
OG0027875	Biological Process	negative regulation of biological process(GO:0048519)	1
OG0027875	Biological Process	regulation of biological process(GO:0050789)	1
OG0027875	Biological Process	reproduction(GO:0000003)	1
OG0027875	Biological Process	reproductive process(GO:0022414)	1
OG0027875	Biological Process	response to stimulus(GO:0050896)	1
OG0027876	Biological Process	cellular process(GO:0009987)	1
OG0027876	Biological Process	metabolic process(GO:0008152)	1
OG0027876	Biological Process	multi-organism process(GO:0051704)	1
OG0027876	Biological Process	response to stimulus(GO:0050896)	1
OG0027883	Biological Process	biological regulation(GO:0065007)	1
OG0027883	Biological Process	cellular component organization or biogenesis(GO:0071840)	1
OG0027883	Biological Process	cellular process(GO:0009987)	1
OG0027883	Biological Process	developmental process(GO:0032502)	1
OG0027883	Biological Process	growth(GO:0040007)	1
OG0027883	Biological Process	metabolic process(GO:0008152)	1
OG0027883	Biological Process	multicellular organismal process(GO:0032501)	1
OG0027883	Biological Process	negative regulation of biological process(GO:0048519)	1
OG0027883	Biological Process	positive regulation of biological process(GO:0048518)	1
OG0027883	Biological Process	regulation of biological process(GO:0050789)	1
OG0027883	Biological Process	reproduction(GO:0000003)	1
OG0027883	Biological Process	reproductive process(GO:0022414)	1
OG0027884	Biological Process	cellular process(GO:0009987)	1
OG0027884	Biological Process	localization(GO:0051179)	1
OG0027887	Biological Process	biological regulation(GO:0065007)	1
OG0027887	Biological Process	cellular component organization or biogenesis(GO:0071840)	1
OG0027887	Biological Process	cellular process(GO:0009987)	1
OG0027887	Biological Process	developmental process(GO:0032502)	1
OG0027887	Biological Process	growth(GO:0040007)	1
OG0027887	Biological Process	immune system process(GO:0002376)	1
OG0027887	Biological Process	localization(GO:0051179)	1
OG0027887	Biological Process	locomotion(GO:0040011)	1
OG0027887	Biological Process	metabolic process(GO:0008152)	1
OG0027887	Biological Process	multi-organism process(GO:0051704)	1
OG0027887	Biological Process	multicellular organismal process(GO:0032501)	1
OG0027887	Biological Process	negative regulation of biological process(GO:0048519)	1
OG0027887	Biological Process	positive regulation of biological process(GO:0048518)	1
OG0027887	Biological Process	regulation of biological process(GO:0050789)	1
OG0027887	Biological Process	reproduction(GO:0000003)	1

OG0027887	Biological Process	reproductive process(GO:0022414)	1
OG0027887	Biological Process	response to stimulus(GO:0050896)	1
OG0027887	Biological Process	signaling(GO:0023052)	1
OG0027889	Biological Process	biological regulation(GO:0065007)	1
OG0027889	Biological Process	cellular process(GO:0009987)	1
OG0027889	Biological Process	metabolic process(GO:0008152)	1
OG0027889	Biological Process	regulation of biological process(GO:0050789)	1
OG0027893	Biological Process	response to stimulus(GO:0050896)	1
OG0027898	Biological Process	biological regulation(GO:0065007)	1
OG0027898	Biological Process	localization(GO:0051179)	1
OG0027898	Biological Process	metabolic process(GO:0008152)	1
OG0027898	Biological Process	positive regulation of biological process(GO:0048518)	1
OG0027898	Biological Process	regulation of biological process(GO:0050789)	1
OG0027898	Biological Process	response to stimulus(GO:0050896)	1
OG0027899	Biological Process	cellular process(GO:0009987)	1
OG0027899	Biological Process	metabolic process(GO:0008152)	1
OG0027899	Biological Process	response to stimulus(GO:0050896)	1
OG0027902	Biological Process	cellular process(GO:0009987)	1
OG0027902	Biological Process	metabolic process(GO:0008152)	1
OG0027902	Biological Process	response to stimulus(GO:0050896)	1
OG0027904	Biological Process	response to stimulus(GO:0050896)	1
OG0027905	Biological Process	cellular process(GO:0009987)	1
OG0027905	Biological Process	metabolic process(GO:0008152)	1
OG0027907	Biological Process	metabolic process(GO:0008152)	1
OG0027908	Biological Process	metabolic process(GO:0008152)	1
OG0027910	Biological Process	cellular process(GO:0009987)	1
OG0027910	Biological Process	metabolic process(GO:0008152)	1
OG0027910	Biological Process	response to stimulus(GO:0050896)	1
OG0027916	Biological Process	biological regulation(GO:0065007)	1
OG0027916	Biological Process	cellular process(GO:0009987)	1
OG0027916	Biological Process	developmental process(GO:0032502)	1
OG0027916	Biological Process	growth(GO:0040007)	1
OG0027916	Biological Process	localization(GO:0051179)	1
OG0027916	Biological Process	metabolic process(GO:0008152)	1
OG0027916	Biological Process	multicellular organismal process(GO:0032501)	1
OG0027916	Biological Process	positive regulation of biological process(GO:0048518)	1
OG0027916	Biological Process	regulation of biological process(GO:0050789)	1
OG0027916	Biological Process	response to stimulus(GO:0050896)	1
OG0027916	Biological Process	signaling(GO:0023052)	1
OG0027932	Biological Process	biological regulation(GO:0065007)	1
OG0027932	Biological Process	developmental process(GO:0032502)	1
OG0027932	Biological Process	multi-organism process(GO:0051704)	1
OG0027932	Biological Process	multicellular organismal process(GO:0032501)	1
OG0027932	Biological Process	negative regulation of biological process(GO:0048519)	1
OG0027932	Biological Process	regulation of biological process(GO:0050789)	1

OG0027932	Biological Process	reproduction(GO:0000003)	1
OG0027932	Biological Process	reproductive process(GO:0022414)	1
OG0027932	Biological Process	response to stimulus(GO:0050896)	1
OG0027938	Biological Process	biological regulation(GO:0065007)	1
OG0027938	Biological Process	cellular process(GO:0009987)	1
OG0027938	Biological Process	metabolic process(GO:0008152)	1
OG0027938	Biological Process	multi-organism process(GO:0051704)	1
OG0027938	Biological Process	response to stimulus(GO:0050896)	1
OG0027939	Biological Process	cellular component organization or biogenesis(GO:0071840)	1
OG0027939	Biological Process	cellular process(GO:0009987)	1
OG0027939	Biological Process	developmental process(GO:0032502)	1
OG0027939	Biological Process	locomotion(GO:0040011)	1
OG0027939	Biological Process	multicellular organismal process(GO:0032501)	1
OG0027939	Biological Process	response to stimulus(GO:0050896)	1
OG0027939	Biological Process	signaling(GO:0023052)	1
OG0027940	Biological Process	biological regulation(GO:0065007)	1
OG0027940	Biological Process	cellular process(GO:0009987)	1
OG0027940	Biological Process	developmental process(GO:0032502)	1
OG0027940	Biological Process	metabolic process(GO:0008152)	1
OG0027940	Biological Process	multicellular organismal process(GO:0032501)	1
OG0027940	Biological Process	negative regulation of biological process(GO:0048519)	1
OG0027940	Biological Process	regulation of biological process(GO:0050789)	1
OG0027940	Biological Process	reproduction(GO:0000003)	1
OG0027940	Biological Process	reproductive process(GO:0022414)	1
OG0027941	Biological Process	metabolic process(GO:0008152)	1
OG0027943	Biological Process	biological regulation(GO:0065007)	1
OG0027943	Biological Process	cell proliferation(GO:0008283)	1
OG0027943	Biological Process	cellular component organization or biogenesis(GO:0071840)	1
OG0027943	Biological Process	cellular process(GO:0009987)	1
OG0027943	Biological Process	developmental process(GO:0032502)	1
OG0027943	Biological Process	growth(GO:0040007)	1
OG0027943	Biological Process	localization(GO:0051179)	1
OG0027943	Biological Process	locomotion(GO:0040011)	1
OG0027943	Biological Process	metabolic process(GO:0008152)	1
OG0027943	Biological Process	multi-organism process(GO:0051704)	1
OG0027943	Biological Process	multicellular organismal process(GO:0032501)	1
OG0027943	Biological Process	positive regulation of biological process(GO:0048518)	1
OG0027943	Biological Process	regulation of biological process(GO:0050789)	1
OG0027943	Biological Process	reproduction(GO:0000003)	1
OG0027943	Biological Process	reproductive process(GO:0022414)	1
OG0027943	Biological Process	response to stimulus(GO:0050896)	1
OG0027943	Biological Process	signaling(GO:0023052)	1
OG0027951	Biological Process	cellular process(GO:0009987)	1
OG0027951	Biological Process	metabolic process(GO:0008152)	1
OG0027952	Biological Process	biological regulation(GO:0065007)	1

OG0027952	Biological Process	cellular process(GO:0009987)	1
OG0027952	Biological Process	metabolic process(GO:0008152)	1
OG0027952	Biological Process	regulation of biological process(GO:0050789)	1
OG0027953	Biological Process	biological regulation(GO:0065007)	1
OG0027953	Biological Process	cellular process(GO:0009987)	1
OG0027953	Biological Process	metabolic process(GO:0008152)	1
OG0027953	Biological Process	regulation of biological process(GO:0050789)	1
OG0027962	Biological Process	biological regulation(GO:0065007)	1
OG0027962	Biological Process	cellular process(GO:0009987)	1
OG0027962	Biological Process	metabolic process(GO:0008152)	1
OG0027962	Biological Process	regulation of biological process(GO:0050789)	1
OG0027962	Biological Process	response to stimulus(GO:0050896)	1
OG0027962	Biological Process	signaling(GO:0023052)	1
OG0027964	Biological Process	cellular process(GO:0009987)	1
OG0027964	Biological Process	localization(GO:0051179)	1
OG0027964	Biological Process	metabolic process(GO:0008152)	1
OG0027966	Biological Process	metabolic process(GO:0008152)	1
OG0027968	Biological Process	cellular process(GO:0009987)	1
OG0027968	Biological Process	metabolic process(GO:0008152)	1
OG0027981	Biological Process	biological regulation(GO:0065007)	1
OG0027981	Biological Process	cellular process(GO:0009987)	1
OG0027981	Biological Process	developmental process(GO:0032502)	1
OG0027981	Biological Process	metabolic process(GO:0008152)	1
OG0027981	Biological Process	multi-organism process(GO:0051704)	1
OG0027981	Biological Process	multicellular organismal process(GO:0032501)	1
OG0027981	Biological Process	regulation of biological process(GO:0050789)	1
OG0027981	Biological Process	reproduction(GO:0000003)	1
OG0027981	Biological Process	reproductive process(GO:0022414)	1
OG0027981	Biological Process	response to stimulus(GO:0050896)	1
OG0027984	Biological Process	cellular process(GO:0009987)	1
OG0027984	Biological Process	metabolic process(GO:0008152)	1
OG0027986	Biological Process	cellular process(GO:0009987)	1
OG0027986	Biological Process	metabolic process(GO:0008152)	1
OG0027986	Biological Process	response to stimulus(GO:0050896)	1
OG0027987	Biological Process	cellular process(GO:0009987)	1
OG0027987	Biological Process	metabolic process(GO:0008152)	1
OG0027987	Biological Process	response to stimulus(GO:0050896)	1
OG0027993	Biological Process	biological regulation(GO:0065007)	1
OG0027993	Biological Process	cellular process(GO:0009987)	1
OG0027993	Biological Process	metabolic process(GO:0008152)	1
OG0027993	Biological Process	regulation of biological process(GO:0050789)	1
OG0027993	Biological Process	response to stimulus(GO:0050896)	1
OG0027993	Biological Process	rhythmic process(GO:0048511)	1
OG0027994	Biological Process	cellular process(GO:0009987)	1
OG0027994	Biological Process	developmental process(GO:0032502)	1
OG0027994	Biological Process	multicellular organismal process(GO:0032501)	1

OG0027995	Biological Process	cellular process(GO:0009987)	1
OG0027995	Biological Process	developmental process(GO:0032502)	1
OG0027995	Biological Process	multicellular organismal process(GO:0032501)	1
OG0027997	Biological Process	cellular process(GO:0009987)	1
OG0027997	Biological Process	developmental process(GO:0032502)	1
OG0027997	Biological Process	multicellular organismal process(GO:0032501)	1
OG0028002	Biological Process	metabolic process(GO:0008152)	1
OG0028003	Biological Process	metabolic process(GO:0008152)	1
OG0028012	Biological Process	response to stimulus(GO:0050896)	1
OG0028016	Biological Process	cellular process(GO:0009987)	1
OG0028016	Biological Process	metabolic process(GO:0008152)	1
OG0028018	Biological Process	developmental process(GO:0032502)	1
OG0028018	Biological Process	multicellular organismal process(GO:0032501)	1
OG0028021	Biological Process	response to stimulus(GO:0050896)	1
OG0028022	Biological Process	response to stimulus(GO:0050896)	1
OG0028025	Biological Process	response to stimulus(GO:0050896)	1
OG0028031	Biological Process	response to stimulus(GO:0050896)	1
OG0028038	Biological Process	cellular process(GO:0009987)	1
OG0028038	Biological Process	localization(GO:0051179)	1
OG0028038	Biological Process	metabolic process(GO:0008152)	1
OG0028041	Biological Process	response to stimulus(GO:0050896)	1
OG0028043	Biological Process	cellular process(GO:0009987)	1
OG0028043	Biological Process	metabolic process(GO:0008152)	1
OG0028043	Biological Process	response to stimulus(GO:0050896)	1
OG0028048	Biological Process	biological regulation(GO:0065007)	1
OG0028048	Biological Process	cellular process(GO:0009987)	1
OG0028048	Biological Process	localization(GO:0051179)	1
OG0028048	Biological Process	metabolic process(GO:0008152)	1
OG0028048	Biological Process	regulation of biological process(GO:0050789)	1
OG0028048	Biological Process	response to stimulus(GO:0050896)	1
OG0028048	Biological Process	signaling(GO:0023052)	1
OG0028052	Biological Process	biological regulation(GO:0065007)	1
OG0028052	Biological Process	cellular component organization or biogenesis(GO:0071840)	1
OG0028052	Biological Process	cellular process(GO:0009987)	1
OG0028052	Biological Process	metabolic process(GO:0008152)	1
OG0028052	Biological Process	regulation of biological process(GO:0050789)	1
OG0028057	Biological Process	biological regulation(GO:0065007)	1
OG0028057	Biological Process	cellular component organization or biogenesis(GO:0071840)	1
OG0028057	Biological Process	cellular process(GO:0009987)	1
OG0028057	Biological Process	developmental process(GO:0032502)	1
OG0028057	Biological Process	regulation of biological process(GO:0050789)	1
OG0028057	Biological Process	response to stimulus(GO:0050896)	1
OG0028059	Biological Process	cellular process(GO:0009987)	1
OG0028059	Biological Process	metabolic process(GO:0008152)	1
OG0028063	Biological Process	growth(GO:0040007)	1

OG0028063	Biological Process	metabolic process(GO:0008152)	1
OG0028065	Biological Process	cellular process(GO:0009987)	1
OG0028065	Biological Process	metabolic process(GO:0008152)	1
OG0028068	Biological Process	cellular process(GO:0009987)	1
OG0028068	Biological Process	metabolic process(GO:0008152)	1
OG0028071	Biological Process	cellular process(GO:0009987)	1
OG0028071	Biological Process	growth(GO:0040007)	1
OG0028071	Biological Process	metabolic process(GO:0008152)	1
OG0028071	Biological Process	response to stimulus(GO:0050896)	1
OG0028073	Biological Process	cellular process(GO:0009987)	1
OG0028073	Biological Process	metabolic process(GO:0008152)	1
OG0028074	Biological Process	cellular component organization or biogenesis(GO:0071840)	1
OG0028074	Biological Process	cellular process(GO:0009987)	1
OG0028074	Biological Process	metabolic process(GO:0008152)	1
OG0028076	Biological Process	cellular process(GO:0009987)	1
OG0028076	Biological Process	metabolic process(GO:0008152)	1
OG0028078	Biological Process	biological regulation(GO:0065007)	1
OG0028078	Biological Process	cellular component organization or biogenesis(GO:0071840)	1
OG0028078	Biological Process	cellular process(GO:0009987)	1
OG0028078	Biological Process	metabolic process(GO:0008152)	1
OG0028078	Biological Process	regulation of biological process(GO:0050789)	1
OG0028078	Biological Process	reproduction(GO:0000003)	1
OG0028078	Biological Process	reproductive process(GO:0022414)	1
OG0028081	Biological Process	cellular process(GO:0009987)	1
OG0028081	Biological Process	growth(GO:0040007)	1
OG0028081	Biological Process	metabolic process(GO:0008152)	1
OG0028083	Biological Process	cellular process(GO:0009987)	1
OG0028083	Biological Process	localization(GO:0051179)	1
OG0028083	Biological Process	metabolic process(GO:0008152)	1
OG0028087	Biological Process	cellular component organization or biogenesis(GO:0071840)	1
OG0028087	Biological Process	cellular process(GO:0009987)	1
OG0028087	Biological Process	metabolic process(GO:0008152)	1
OG0028089	Biological Process	cellular component organization or biogenesis(GO:0071840)	1
OG0028089	Biological Process	cellular process(GO:0009987)	1
OG0028089	Biological Process	metabolic process(GO:0008152)	1
OG0028089	Biological Process	response to stimulus(GO:0050896)	1
OG0028091	Biological Process	cellular process(GO:0009987)	1
OG0028091	Biological Process	growth(GO:0040007)	1
OG0028091	Biological Process	localization(GO:0051179)	1
OG0028091	Biological Process	metabolic process(GO:0008152)	1
OG0028098	Biological Process	cellular process(GO:0009987)	1
OG0028098	Biological Process	metabolic process(GO:0008152)	1
OG0028099	Biological Process	biological regulation(GO:0065007)	1
OG0028099	Biological Process	cellular process(GO:0009987)	1
OG0028099	Biological Process	metabolic process(GO:0008152)	1
OG0028099	Biological Process	multi-organism process(GO:0051704)	1

OG0028099	Biological Process	positive regulation of biological process(GO:0048518)	1
OG0028099	Biological Process	regulation of biological process(GO:0050789)	1
OG0028099	Biological Process	response to stimulus(GO:0050896)	1
OG0028099	Biological Process	signaling(GO:0023052)	1
OG0028116	Biological Process	biological regulation(GO:0065007)	1
OG0028116	Biological Process	cellular process(GO:0009987)	1
OG0028116	Biological Process	metabolic process(GO:0008152)	1
OG0028116	Biological Process	regulation of biological process(GO:0050789)	1
OG0028116	Biological Process	response to stimulus(GO:0050896)	1
OG0028119	Biological Process	biological regulation(GO:0065007)	1
OG0028119	Biological Process	cellular component organization or biogenesis(GO:0071840)	1
OG0028119	Biological Process	cellular process(GO:0009987)	1
OG0028119	Biological Process	localization(GO:0051179)	1
OG0028119	Biological Process	multi-organism process(GO:0051704)	1
OG0028119	Biological Process	multicellular organismal process(GO:0032501)	1
OG0028119	Biological Process	reproduction(GO:0000003)	1
OG0028119	Biological Process	reproductive process(GO:0022414)	1
OG0028119	Biological Process	signaling(GO:0023052)	1
OG0028124	Biological Process	cellular process(GO:0009987)	1
OG0028124	Biological Process	metabolic process(GO:0008152)	1
OG0028128	Biological Process	cellular process(GO:0009987)	1
OG0028128	Biological Process	growth(GO:0040007)	1
OG0028128	Biological Process	localization(GO:0051179)	1
OG0028128	Biological Process	metabolic process(GO:0008152)	1
OG0028132	Biological Process	cellular process(GO:0009987)	1
OG0028132	Biological Process	growth(GO:0040007)	1
OG0028132	Biological Process	metabolic process(GO:0008152)	1
OG0028138	Biological Process	developmental process(GO:0032502)	1
OG0028138	Biological Process	multicellular organismal process(GO:0032501)	1
OG0028139	Biological Process	cellular process(GO:0009987)	1
OG0028139	Biological Process	metabolic process(GO:0008152)	1
OG0028152	Biological Process	cellular process(GO:0009987)	1
OG0028152	Biological Process	metabolic process(GO:0008152)	1
OG0028152	Biological Process	response to stimulus(GO:0050896)	1
OG0028153	Biological Process	biological regulation(GO:0065007)	1
OG0028153	Biological Process	cellular component organization or biogenesis(GO:0071840)	1
OG0028153	Biological Process	cellular process(GO:0009987)	1
OG0028153	Biological Process	developmental process(GO:0032502)	1
OG0028153	Biological Process	metabolic process(GO:0008152)	1
OG0028153	Biological Process	multicellular organismal process(GO:0032501)	1
OG0028153	Biological Process	regulation of biological process(GO:0050789)	1
OG0028153	Biological Process	reproduction(GO:0000003)	1
OG0028153	Biological Process	reproductive process(GO:0022414)	1
OG0028160	Biological Process	biological regulation(GO:0065007)	1
OG0028160	Biological Process	cellular process(GO:0009987)	1

OG0028160	Biological Process	immune system process(GO:0002376)	1
OG0028160	Biological Process	metabolic process(GO:0008152)	1
OG0028160	Biological Process	multi-organism process(GO:0051704)	1
OG0028160	Biological Process	regulation of biological process(GO:0050789)	1
OG0028160	Biological Process	response to stimulus(GO:0050896)	1
OG0028160	Biological Process	signaling(GO:0023052)	1
OG0028171	Biological Process	cellular component organization or biogenesis(GO:0071840)	1
OG0028171	Biological Process	cellular process(GO:0009987)	1
OG0028171	Biological Process	growth(GO:0040007)	1
OG0028171	Biological Process	metabolic process(GO:0008152)	1
OG0028172	Biological Process	cellular process(GO:0009987)	1
OG0028172	Biological Process	metabolic process(GO:0008152)	1
OG0028173	Biological Process	growth(GO:0040007)	1
OG0028175	Biological Process	cellular process(GO:0009987)	1
OG0028175	Biological Process	metabolic process(GO:0008152)	1
OG0028176	Biological Process	cellular process(GO:0009987)	1
OG0028176	Biological Process	metabolic process(GO:0008152)	1
OG0028177	Biological Process	cellular process(GO:0009987)	1
OG0028177	Biological Process	metabolic process(GO:0008152)	1
OG0028180	Biological Process	cellular process(GO:0009987)	1
OG0028180	Biological Process	metabolic process(GO:0008152)	1
OG0028181	Biological Process	cellular process(GO:0009987)	1
OG0028181	Biological Process	growth(GO:0040007)	1
OG0028181	Biological Process	metabolic process(GO:0008152)	1
OG0028184	Biological Process	biological regulation(GO:0065007)	1
OG0028184	Biological Process	cellular process(GO:0009987)	1
OG0028184	Biological Process	developmental process(GO:0032502)	1
OG0028184	Biological Process	metabolic process(GO:0008152)	1
OG0028184	Biological Process	multicellular organismal process(GO:0032501)	1
OG0028184	Biological Process	positive regulation of biological process(GO:0048518)	1
OG0028184	Biological Process	regulation of biological process(GO:0050789)	1
OG0028184	Biological Process	response to stimulus(GO:0050896)	1
OG0028184	Biological Process	signaling(GO:0023052)	1
OG0028185	Biological Process	biological regulation(GO:0065007)	1
OG0028185	Biological Process	cellular process(GO:0009987)	1
OG0028185	Biological Process	metabolic process(GO:0008152)	1
OG0028185	Biological Process	regulation of biological process(GO:0050789)	1
OG0028185	Biological Process	response to stimulus(GO:0050896)	1
OG0028186	Biological Process	biological regulation(GO:0065007)	1
OG0028186	Biological Process	cellular process(GO:0009987)	1
OG0028186	Biological Process	metabolic process(GO:0008152)	1
OG0028186	Biological Process	regulation of biological process(GO:0050789)	1
OG0028188	Biological Process	biological regulation(GO:0065007)	1
OG0028188	Biological Process	cellular process(GO:0009987)	1
OG0028188	Biological Process	developmental process(GO:0032502)	1
OG0028188	Biological Process	metabolic process(GO:0008152)	1

OG0028188	Biological Process	multicellular organismal process(GO:0032501)	1
OG0028188	Biological Process	regulation of biological process(GO:0050789)	1
OG0028188	Biological Process	reproduction(GO:0000003)	1
OG0028188	Biological Process	reproductive process(GO:0022414)	1
OG0028193	Biological Process	biological regulation(GO:0065007)	1
OG0028193	Biological Process	cellular process(GO:0009987)	1
OG0028193	Biological Process	immune system process(GO:0002376)	1
OG0028193	Biological Process	metabolic process(GO:0008152)	1
OG0028193	Biological Process	multi-organism process(GO:0051704)	1
OG0028193	Biological Process	regulation of biological process(GO:0050789)	1
OG0028193	Biological Process	response to stimulus(GO:0050896)	1
OG0028195	Biological Process	biological regulation(GO:0065007)	1
OG0028195	Biological Process	cellular process(GO:0009987)	1
OG0028195	Biological Process	metabolic process(GO:0008152)	1
OG0028195	Biological Process	negative regulation of biological process(GO:0048519)	1
OG0028195	Biological Process	regulation of biological process(GO:0050789)	1
OG0028196	Biological Process	biological regulation(GO:0065007)	1
OG0028196	Biological Process	cellular process(GO:0009987)	1
OG0028196	Biological Process	metabolic process(GO:0008152)	1
OG0028196	Biological Process	regulation of biological process(GO:0050789)	1
OG0028201	Biological Process	cellular component organization or biogenesis(GO:0071840)	1
OG0028201	Biological Process	cellular process(GO:0009987)	1
OG0028201	Biological Process	metabolic process(GO:0008152)	1
OG0028202	Biological Process	biological regulation(GO:0065007)	1
OG0028202	Biological Process	cellular process(GO:0009987)	1
OG0028202	Biological Process	metabolic process(GO:0008152)	1
OG0028202	Biological Process	regulation of biological process(GO:0050789)	1
OG0028202	Biological Process	response to stimulus(GO:0050896)	1
OG0028202	Biological Process	signaling(GO:0023052)	1
OG0028209	Biological Process	cellular process(GO:0009987)	1
OG0028209	Biological Process	metabolic process(GO:0008152)	1
OG0028209	Biological Process	response to stimulus(GO:0050896)	1
OG0028210	Biological Process	cell proliferation(GO:0008283)	1
OG0028210	Biological Process	cellular process(GO:0009987)	1
OG0028210	Biological Process	developmental process(GO:0032502)	1
OG0028210	Biological Process	localization(GO:0051179)	1
OG0028210	Biological Process	multicellular organismal process(GO:0032501)	1
OG0028216	Biological Process	cellular process(GO:0009987)	1
OG0028216	Biological Process	metabolic process(GO:0008152)	1
OG0028216	Biological Process	response to stimulus(GO:0050896)	1
OG0028222	Biological Process	biological regulation(GO:0065007)	1
OG0028222	Biological Process	cellular process(GO:0009987)	1
OG0028222	Biological Process	developmental process(GO:0032502)	1
OG0028222	Biological Process	multi-organism process(GO:0051704)	1
OG0028222	Biological Process	multicellular organismal process(GO:0032501)	1

OG0028222	Biological Process	negative regulation of biological process(GO:0048519)	1
OG0028222	Biological Process	regulation of biological process(GO:0050789)	1
OG0028222	Biological Process	response to stimulus(GO:0050896)	1
OG0028224	Biological Process	biological regulation(GO:0065007)	1
OG0028224	Biological Process	cellular process(GO:0009987)	1
OG0028224	Biological Process	developmental process(GO:0032502)	1
OG0028224	Biological Process	multi-organism process(GO:0051704)	1
OG0028224	Biological Process	multicellular organismal process(GO:0032501)	1
OG0028224	Biological Process	negative regulation of biological process(GO:0048519)	1
OG0028224	Biological Process	regulation of biological process(GO:0050789)	1
OG0028224	Biological Process	response to stimulus(GO:0050896)	1
OG0028229	Biological Process	response to stimulus(GO:0050896)	1
OG0028231	Biological Process	metabolic process(GO:0008152)	1
OG0028232	Biological Process	metabolic process(GO:0008152)	1
OG0028233	Biological Process	metabolic process(GO:0008152)	1
OG0028241	Biological Process	cellular component organization or biogenesis(GO:0071840)	1
OG0028241	Biological Process	cellular process(GO:0009987)	1
OG0028241	Biological Process	localization(GO:0051179)	1
OG0028241	Biological Process	metabolic process(GO:0008152)	1
OG0028241	Biological Process	response to stimulus(GO:0050896)	1
OG0028249	Biological Process	biological regulation(GO:0065007)	1
OG0028249	Biological Process	cellular process(GO:0009987)	1
OG0028249	Biological Process	developmental process(GO:0032502)	1
OG0028249	Biological Process	metabolic process(GO:0008152)	1
OG0028249	Biological Process	multicellular organismal process(GO:0032501)	1
OG0028249	Biological Process	regulation of biological process(GO:0050789)	1
OG0028249	Biological Process	reproduction(GO:0000003)	1
OG0028249	Biological Process	reproductive process(GO:0022414)	1
OG0028249	Biological Process	response to stimulus(GO:0050896)	1
OG0028249	Biological Process	signaling(GO:0023052)	1
OG0028255	Biological Process	biological regulation(GO:0065007)	1
OG0028255	Biological Process	cellular component organization or biogenesis(GO:0071840)	1
OG0028255	Biological Process	cellular process(GO:0009987)	1
OG0028255	Biological Process	localization(GO:0051179)	1
OG0028255	Biological Process	metabolic process(GO:0008152)	1
OG0028255	Biological Process	regulation of biological process(GO:0050789)	1
OG0028259	Biological Process	biological regulation(GO:0065007)	1
OG0028259	Biological Process	cell proliferation(GO:0008283)	1
OG0028259	Biological Process	cellular component organization or biogenesis(GO:0071840)	1
OG0028259	Biological Process	cellular process(GO:0009987)	1
OG0028259	Biological Process	developmental process(GO:0032502)	1
OG0028259	Biological Process	growth(GO:0040007)	1
OG0028259	Biological Process	localization(GO:0051179)	1
OG0028259	Biological Process	locomotion(GO:0040011)	1
OG0028259	Biological Process	metabolic process(GO:0008152)	1

OG0028259	Biological Process	multicellular organismal process(GO:0032501)	1
OG0028259	Biological Process	negative regulation of biological process(GO:0048519)	1
OG0028259	Biological Process	positive regulation of biological process(GO:0048518)	1
OG0028259	Biological Process	regulation of biological process(GO:0050789)	1
OG0028259	Biological Process	reproduction(GO:0000003)	1
OG0028259	Biological Process	reproductive process(GO:0022414)	1
OG0028259	Biological Process	response to stimulus(GO:0050896)	1
OG0028299	Biological Process	cellular component organization or biogenesis(GO:0071840)	1
OG0028299	Biological Process	cellular process(GO:0009987)	1
OG0028299	Biological Process	localization(GO:0051179)	1
OG0028305	Biological Process	biological regulation(GO:0065007)	1
OG0028305	Biological Process	cellular process(GO:0009987)	1
OG0028305	Biological Process	developmental process(GO:0032502)	1
OG0028305	Biological Process	metabolic process(GO:0008152)	1
OG0028305	Biological Process	multicellular organismal process(GO:0032501)	1
OG0028305	Biological Process	negative regulation of biological process(GO:0048519)	1
OG0028305	Biological Process	regulation of biological process(GO:0050789)	1
OG0028305	Biological Process	reproduction(GO:0000003)	1
OG0028305	Biological Process	reproductive process(GO:0022414)	1
OG0028305	Biological Process	response to stimulus(GO:0050896)	1
OG0028308	Biological Process	biological regulation(GO:0065007)	1
OG0028308	Biological Process	cellular process(GO:0009987)	1
OG0028308	Biological Process	metabolic process(GO:0008152)	1
OG0028323	Biological Process	biological regulation(GO:0065007)	1
OG0028323	Biological Process	cellular process(GO:0009987)	1
OG0028323	Biological Process	metabolic process(GO:0008152)	1
OG0028323	Biological Process	regulation of biological process(GO:0050789)	1
OG0028330	Biological Process	response to stimulus(GO:0050896)	1
OG0028331	Biological Process	developmental process(GO:0032502)	1
OG0028331	Biological Process	multicellular organismal process(GO:0032501)	1
OG0028331	Biological Process	reproduction(GO:0000003)	1
OG0028331	Biological Process	reproductive process(GO:0022414)	1
OG0028334	Biological Process	biological regulation(GO:0065007)	1
OG0028334	Biological Process	cellular process(GO:0009987)	1
OG0028334	Biological Process	metabolic process(GO:0008152)	1
OG0028334	Biological Process	regulation of biological process(GO:0050789)	1
OG0028334	Biological Process	response to stimulus(GO:0050896)	1
OG0028334	Biological Process	signaling(GO:0023052)	1
OG0028341	Biological Process	multi-organism process(GO:0051704)	1
OG0028341	Biological Process	response to stimulus(GO:0050896)	1
OG0028345	Biological Process	biological regulation(GO:0065007)	1
OG0028345	Biological Process	cellular process(GO:0009987)	1
OG0028345	Biological Process	metabolic process(GO:0008152)	1
OG0028345	Biological Process	regulation of biological process(GO:0050789)	1

OG0028373	Biological Process	cellular process(GO:0009987)	1
OG0028373	Biological Process	metabolic process(GO:0008152)	1
OG0028373	Biological Process	response to stimulus(GO:0050896)	1
OG0028388	Biological Process	biological regulation(GO:0065007)	1
OG0028388	Biological Process	cellular process(GO:0009987)	1
OG0028388	Biological Process	developmental process(GO:0032502)	1
OG0028388	Biological Process	metabolic process(GO:0008152)	1
OG0028388	Biological Process	multicellular organismal process(GO:0032501)	1
OG0028388	Biological Process	regulation of biological process(GO:0050789)	1
OG0028388	Biological Process	response to stimulus(GO:0050896)	1
OG0028390	Biological Process	cellular process(GO:0009987)	1
OG0028390	Biological Process	metabolic process(GO:0008152)	1
OG0028390	Biological Process	response to stimulus(GO:0050896)	1
OG0028399	Biological Process	cellular process(GO:0009987)	1
OG0028399	Biological Process	metabolic process(GO:0008152)	1
OG0028405	Biological Process	cellular process(GO:0009987)	1
OG0028405	Biological Process	metabolic process(GO:0008152)	1
OG0028405	Biological Process	response to stimulus(GO:0050896)	1
OG0028410	Biological Process	cellular component organization or biogenesis(GO:0071840)	1
OG0028410	Biological Process	cellular process(GO:0009987)	1
OG0028410	Biological Process	developmental process(GO:0032502)	1
OG0028410	Biological Process	metabolic process(GO:0008152)	1
OG0028410	Biological Process	multicellular organismal process(GO:0032501)	1
OG0028410	Biological Process	reproduction(GO:0000003)	1
OG0028410	Biological Process	reproductive process(GO:0022414)	1
OG0028410	Biological Process	response to stimulus(GO:0050896)	1
OG0028415	Biological Process	cellular process(GO:0009987)	1
OG0028415	Biological Process	metabolic process(GO:0008152)	1
OG0028415	Biological Process	response to stimulus(GO:0050896)	1
OG0028416	Biological Process	cellular process(GO:0009987)	1
OG0028416	Biological Process	metabolic process(GO:0008152)	1
OG0028416	Biological Process	response to stimulus(GO:0050896)	1
OG0028417	Biological Process	biological regulation(GO:0065007)	1
OG0028417	Biological Process	cellular process(GO:0009987)	1
OG0028417	Biological Process	metabolic process(GO:0008152)	1
OG0028417	Biological Process	regulation of biological process(GO:0050789)	1
OG0028419	Biological Process	biological regulation(GO:0065007)	1
OG0028419	Biological Process	cell proliferation(GO:0008283)	1
OG0028419	Biological Process	cellular component organization or biogenesis(GO:0071840)	1
OG0028419	Biological Process	cellular process(GO:0009987)	1
OG0028419	Biological Process	developmental process(GO:0032502)	1
OG0028419	Biological Process	metabolic process(GO:0008152)	1
OG0028419	Biological Process	multicellular organismal process(GO:0032501)	1
OG0028419	Biological Process	positive regulation of biological process(GO:0048518)	1
OG0028419	Biological Process	regulation of biological process(GO:0050789)	1

OG0028419	Biological Process	reproduction(GO:0000003)	1
OG0028419	Biological Process	reproductive process(GO:0022414)	1
OG0028419	Biological Process	response to stimulus(GO:0050896)	1
OG0028433	Biological Process	biological regulation(GO:0065007)	1
OG0028433	Biological Process	cellular process(GO:0009987)	1
OG0028433	Biological Process	metabolic process(GO:0008152)	1
OG0028433	Biological Process	regulation of biological process(GO:0050789)	1
OG0028440	Biological Process	cellular component organization or biogenesis(GO:0071840)	1
OG0028440	Biological Process	cellular process(GO:0009987)	1
OG0028440	Biological Process	metabolic process(GO:0008152)	1
OG0028440	Biological Process	reproduction(GO:0000003)	1
OG0028440	Biological Process	reproductive process(GO:0022414)	1
OG0028440	Biological Process	response to stimulus(GO:0050896)	1
OG0028446	Biological Process	cellular process(GO:0009987)	1
OG0028446	Biological Process	developmental process(GO:0032502)	1
OG0028446	Biological Process	metabolic process(GO:0008152)	1
OG0028446	Biological Process	multicellular organismal process(GO:0032501)	1
OG0028446	Biological Process	reproduction(GO:0000003)	1
OG0028446	Biological Process	reproductive process(GO:0022414)	1
OG0028446	Biological Process	response to stimulus(GO:0050896)	1
OG0028447	Biological Process	biological regulation(GO:0065007)	1
OG0028447	Biological Process	cellular component organization or biogenesis(GO:0071840)	1
OG0028447	Biological Process	cellular process(GO:0009987)	1
OG0028447	Biological Process	detoxification(GO:0098754)	1
OG0028447	Biological Process	localization(GO:0051179)	1
OG0028447	Biological Process	metabolic process(GO:0008152)	1
OG0028447	Biological Process	multi-organism process(GO:0051704)	1
OG0028447	Biological Process	negative regulation of biological process(GO:0048519)	1
OG0028447	Biological Process	positive regulation of biological process(GO:0048518)	1
OG0028447	Biological Process	regulation of biological process(GO:0050789)	1
OG0028447	Biological Process	reproduction(GO:0000003)	1
OG0028447	Biological Process	reproductive process(GO:0022414)	1
OG0028447	Biological Process	response to stimulus(GO:0050896)	1
OG0028448	Biological Process	biological regulation(GO:0065007)	1
OG0028448	Biological Process	cellular component organization or biogenesis(GO:0071840)	1
OG0028448	Biological Process	cellular process(GO:0009987)	1
OG0028448	Biological Process	detoxification(GO:0098754)	1
OG0028448	Biological Process	localization(GO:0051179)	1
OG0028448	Biological Process	metabolic process(GO:0008152)	1
OG0028448	Biological Process	multi-organism process(GO:0051704)	1
OG0028448	Biological Process	negative regulation of biological process(GO:0048519)	1
OG0028448	Biological Process	positive regulation of biological process(GO:0048518)	1
OG0028448	Biological Process	regulation of biological process(GO:0050789)	1
OG0028448	Biological Process	reproduction(GO:0000003)	1
OG0028448	Biological Process	reproductive process(GO:0022414)	1

OG0028448	Biological Process	response to stimulus(GO:0050896)	1
OG0028470	Biological Process	cellular component organization or biogenesis(GO:0071840)	1
OG0028470	Biological Process	cellular process(GO:0009987)	1
OG0028470	Biological Process	metabolic process(GO:0008152)	1
OG0028470	Biological Process	response to stimulus(GO:0050896)	1
OG0028484	Biological Process	biological regulation(GO:0065007)	1
OG0028484	Biological Process	cellular process(GO:0009987)	1
OG0028484	Biological Process	metabolic process(GO:0008152)	1
OG0028484	Biological Process	regulation of biological process(GO:0050789)	1
OG0028484	Biological Process	response to stimulus(GO:0050896)	1
OG0028485	Biological Process	multicellular organismal process(GO:0032501)	1
OG0028485	Biological Process	response to stimulus(GO:0050896)	1
OG0028500	Biological Process	biological regulation(GO:0065007)	1
OG0028500	Biological Process	cellular process(GO:0009987)	1
OG0028500	Biological Process	regulation of biological process(GO:0050789)	1
OG0028500	Biological Process	response to stimulus(GO:0050896)	1
OG0028500	Biological Process	signaling(GO:0023052)	1
OG0028504	Biological Process	biological regulation(GO:0065007)	1
OG0028504	Biological Process	cellular process(GO:0009987)	1
OG0028504	Biological Process	developmental process(GO:0032502)	1
OG0028504	Biological Process	metabolic process(GO:0008152)	1
OG0028504	Biological Process	multicellular organismal process(GO:0032501)	1
OG0028504	Biological Process	regulation of biological process(GO:0050789)	1
OG0028506	Biological Process	cellular process(GO:0009987)	1
OG0028506	Biological Process	developmental process(GO:0032502)	1
OG0028506	Biological Process	metabolic process(GO:0008152)	1
OG0028506	Biological Process	multicellular organismal process(GO:0032501)	1
OG0028506	Biological Process	reproduction(GO:0000003)	1
OG0028506	Biological Process	reproductive process(GO:0022414)	1
OG0028506	Biological Process	response to stimulus(GO:0050896)	1
OG0028528	Biological Process	biological regulation(GO:0065007)	1
OG0028528	Biological Process	cellular process(GO:0009987)	1
OG0028528	Biological Process	metabolic process(GO:0008152)	1
OG0028528	Biological Process	regulation of biological process(GO:0050789)	1
OG0028530	Biological Process	biological regulation(GO:0065007)	1
OG0028530	Biological Process	cellular process(GO:0009987)	1
OG0028530	Biological Process	metabolic process(GO:0008152)	1
OG0028530	Biological Process	regulation of biological process(GO:0050789)	1
OG0028530	Biological Process	response to stimulus(GO:0050896)	1
OG0028532	Biological Process	developmental process(GO:0032502)	1
OG0028532	Biological Process	multicellular organismal process(GO:0032501)	1
OG0028533	Biological Process	cellular process(GO:0009987)	1
OG0028533	Biological Process	detoxification(GO:0098754)	1
OG0028533	Biological Process	metabolic process(GO:0008152)	1
OG0028533	Biological Process	response to stimulus(GO:0050896)	1

OG0028536	Biological Process	response to stimulus(GO:0050896)	1
OG0028538	Biological Process	biological regulation(GO:0065007)	1
OG0028538	Biological Process	cellular process(GO:0009987)	1
OG0028538	Biological Process	developmental process(GO:0032502)	1
OG0028538	Biological Process	metabolic process(GO:0008152)	1
OG0028538	Biological Process	multicellular organismal process(GO:0032501)	1
OG0028538	Biological Process	regulation of biological process(GO:0050789)	1
OG0028539	Biological Process	cellular process(GO:0009987)	1
OG0028539	Biological Process	metabolic process(GO:0008152)	1
OG0028543	Biological Process	response to stimulus(GO:0050896)	1
OG0028545	Biological Process	cellular process(GO:0009987)	1
OG0028545	Biological Process	localization(GO:0051179)	1
OG0028545	Biological Process	locomotion(GO:0040011)	1
OG0028545	Biological Process	metabolic process(GO:0008152)	1
OG0028545	Biological Process	response to stimulus(GO:0050896)	1
OG0028546	Biological Process	cellular component organization or biogenesis(GO:0071840)	1
OG0028546	Biological Process	cellular process(GO:0009987)	1
OG0028546	Biological Process	growth(GO:0040007)	1
OG0028546	Biological Process	metabolic process(GO:0008152)	1
OG0028546	Biological Process	multi-organism process(GO:0051704)	1
OG0028546	Biological Process	response to stimulus(GO:0050896)	1
OG0028547	Biological Process	growth(GO:0040007)	1
OG0028548	Biological Process	cellular process(GO:0009987)	1
OG0028548	Biological Process	growth(GO:0040007)	1
OG0028548	Biological Process	metabolic process(GO:0008152)	1
OG0028549	Biological Process	biological regulation(GO:0065007)	1
OG0028549	Biological Process	cellular process(GO:0009987)	1
OG0028549	Biological Process	metabolic process(GO:0008152)	1
OG0028549	Biological Process	positive regulation of biological process(GO:0048518)	1
OG0028549	Biological Process	regulation of biological process(GO:0050789)	1
OG0028549	Biological Process	response to stimulus(GO:0050896)	1
OG0028551	Biological Process	biological regulation(GO:0065007)	1
OG0028551	Biological Process	cellular process(GO:0009987)	1
OG0028551	Biological Process	metabolic process(GO:0008152)	1
OG0028551	Biological Process	positive regulation of biological process(GO:0048518)	1
OG0028551	Biological Process	regulation of biological process(GO:0050789)	1
OG0028551	Biological Process	response to stimulus(GO:0050896)	1
OG0028556	Biological Process	biological regulation(GO:0065007)	1
OG0028556	Biological Process	cellular process(GO:0009987)	1
OG0028556	Biological Process	metabolic process(GO:0008152)	1
OG0028556	Biological Process	regulation of biological process(GO:0050789)	1
OG0028556	Biological Process	response to stimulus(GO:0050896)	1
OG0028556	Biological Process	signaling(GO:0023052)	1
OG0028566	Biological Process	cellular process(GO:0009987)	1
OG0028566	Biological Process	metabolic process(GO:0008152)	1

OG0028571	Biological Process	cellular component organization or biogenesis(GO:0071840)	1
OG0028571	Biological Process	cellular process(GO:0009987)	1
OG0028571	Biological Process	metabolic process(GO:0008152)	1
OG0028572	Biological Process	cellular process(GO:0009987)	1
OG0028572	Biological Process	metabolic process(GO:0008152)	1
OG0028593	Biological Process	cellular process(GO:0009987)	1
OG0028593	Biological Process	metabolic process(GO:0008152)	1
OG0028595	Biological Process	biological regulation(GO:0065007)	1
OG0028595	Biological Process	cellular process(GO:0009987)	1
OG0028595	Biological Process	metabolic process(GO:0008152)	1
OG0028595	Biological Process	regulation of biological process(GO:0050789)	1
OG0028595	Biological Process	response to stimulus(GO:0050896)	1
OG0028597	Biological Process	cellular component organization or biogenesis(GO:0071840)	1
OG0028597	Biological Process	cellular process(GO:0009987)	1
OG0028597	Biological Process	metabolic process(GO:0008152)	1
OG0028599	Biological Process	cellular process(GO:0009987)	1
OG0028599	Biological Process	localization(GO:0051179)	1
OG0028599	Biological Process	response to stimulus(GO:0050896)	1
OG0028610	Biological Process	biological regulation(GO:0065007)	1
OG0028610	Biological Process	cellular process(GO:0009987)	1
OG0028610	Biological Process	metabolic process(GO:0008152)	1
OG0028610	Biological Process	positive regulation of biological process(GO:0048518)	1
OG0028610	Biological Process	regulation of biological process(GO:0050789)	1
OG0028610	Biological Process	response to stimulus(GO:0050896)	1
OG0028612	Biological Process	cellular component organization or biogenesis(GO:0071840)	1
OG0028612	Biological Process	cellular process(GO:0009987)	1
OG0028612	Biological Process	developmental process(GO:0032502)	1
OG0028612	Biological Process	metabolic process(GO:0008152)	1
OG0028612	Biological Process	multicellular organismal process(GO:0032501)	1
OG0028612	Biological Process	response to stimulus(GO:0050896)	1
OG0028624	Biological Process	localization(GO:0051179)	1
OG0028627	Biological Process	biological regulation(GO:0065007)	1
OG0028627	Biological Process	cellular process(GO:0009987)	1
OG0028627	Biological Process	metabolic process(GO:0008152)	1
OG0028627	Biological Process	regulation of biological process(GO:0050789)	1
OG0028630	Biological Process	cellular process(GO:0009987)	1
OG0028630	Biological Process	developmental process(GO:0032502)	1
OG0028630	Biological Process	metabolic process(GO:0008152)	1
OG0028630	Biological Process	multicellular organismal process(GO:0032501)	1
OG0028630	Biological Process	reproduction(GO:0000003)	1
OG0028630	Biological Process	reproductive process(GO:0022414)	1
OG0028656	Biological Process	cellular component organization or biogenesis(GO:0071840)	1
OG0028656	Biological Process	cellular process(GO:0009987)	1
OG0028656	Biological Process	growth(GO:0040007)	1
OG0028656	Biological Process	metabolic process(GO:0008152)	1

OG0028657	Biological Process	growth(GO:0040007)	1
OG0028657	Biological Process	metabolic process(GO:0008152)	1
OG0028657	Biological Process	multi-organism process(GO:0051704)	1
OG0028657	Biological Process	response to stimulus(GO:0050896)	1
OG0028660	Biological Process	cellular process(GO:0009987)	1
OG0028660	Biological Process	metabolic process(GO:0008152)	1
OG0028661	Biological Process	cellular process(GO:0009987)	1
OG0028661	Biological Process	metabolic process(GO:0008152)	1
OG0028662	Biological Process	cellular process(GO:0009987)	1
OG0028662	Biological Process	metabolic process(GO:0008152)	1
OG0028663	Biological Process	cellular process(GO:0009987)	1
OG0028663	Biological Process	metabolic process(GO:0008152)	1
OG0028664	Biological Process	cellular process(GO:0009987)	1
OG0028664	Biological Process	metabolic process(GO:0008152)	1
OG0028666	Biological Process	cellular process(GO:0009987)	1
OG0028666	Biological Process	metabolic process(GO:0008152)	1
OG0028672	Biological Process	biological regulation(GO:0065007)	1
OG0028672	Biological Process	cellular component organization or biogenesis(GO:0071840)	1
OG0028672	Biological Process	cellular process(GO:0009987)	1
OG0028672	Biological Process	metabolic process(GO:0008152)	1
OG0028672	Biological Process	regulation of biological process(GO:0050789)	1
OG0028689	Biological Process	biological regulation(GO:0065007)	1
OG0028689	Biological Process	cellular process(GO:0009987)	1
OG0028689	Biological Process	developmental process(GO:0032502)	1
OG0028689	Biological Process	metabolic process(GO:0008152)	1
OG0028689	Biological Process	multicellular organismal process(GO:0032501)	1
OG0028689	Biological Process	negative regulation of biological process(GO:0048519)	1
OG0028689	Biological Process	regulation of biological process(GO:0050789)	1
OG0028689	Biological Process	reproduction(GO:0000003)	1
OG0028689	Biological Process	reproductive process(GO:0022414)	1
OG0028689	Biological Process	response to stimulus(GO:0050896)	1
OG0028713	Biological Process	biological regulation(GO:0065007)	1
OG0028713	Biological Process	cellular process(GO:0009987)	1
OG0028713	Biological Process	metabolic process(GO:0008152)	1
OG0028713	Biological Process	multi-organism process(GO:0051704)	1
OG0028713	Biological Process	positive regulation of biological process(GO:0048518)	1
OG0028713	Biological Process	regulation of biological process(GO:0050789)	1
OG0028713	Biological Process	response to stimulus(GO:0050896)	1
OG0028719	Biological Process	cellular process(GO:0009987)	1
OG0028719	Biological Process	metabolic process(GO:0008152)	1
OG0028722	Biological Process	biological regulation(GO:0065007)	1
OG0028722	Biological Process	cellular process(GO:0009987)	1
OG0028722	Biological Process	developmental process(GO:0032502)	1
OG0028722	Biological Process	growth(GO:0040007)	1
OG0028722	Biological Process	localization(GO:0051179)	1

OG0028722	Biological Process	multicellular organismal process(GO:0032501)	1
OG0028722	Biological Process	regulation of biological process(GO:0050789)	1
OG0028722	Biological Process	response to stimulus(GO:0050896)	1
OG0028732	Biological Process	metabolic process(GO:0008152)	1
OG0028745	Biological Process	cellular process(GO:0009987)	1
OG0028745	Biological Process	metabolic process(GO:0008152)	1
OG0028746	Biological Process	cellular process(GO:0009987)	1
OG0028746	Biological Process	growth(GO:0040007)	1
OG0028746	Biological Process	metabolic process(GO:0008152)	1
OG0028747	Biological Process	cellular process(GO:0009987)	1
OG0028747	Biological Process	metabolic process(GO:0008152)	1
OG0028749	Biological Process	cellular process(GO:0009987)	1
OG0028749	Biological Process	growth(GO:0040007)	1
OG0028749	Biological Process	metabolic process(GO:0008152)	1
OG0028749	Biological Process	multi-organism process(GO:0051704)	1
OG0028754	Biological Process	developmental process(GO:0032502)	1
OG0028754	Biological Process	immune system process(GO:0002376)	1
OG0028754	Biological Process	multi-organism process(GO:0051704)	1
OG0028754	Biological Process	multicellular organismal process(GO:0032501)	1
OG0028754	Biological Process	response to stimulus(GO:0050896)	1
OG0028758	Biological Process	biological regulation(GO:0065007)	1
OG0028758	Biological Process	cellular process(GO:0009987)	1
OG0028758	Biological Process	developmental process(GO:0032502)	1
OG0028758	Biological Process	multicellular organismal process(GO:0032501)	1
OG0028758	Biological Process	regulation of biological process(GO:0050789)	1
OG0028758	Biological Process	response to stimulus(GO:0050896)	1
OG0028758	Biological Process	rhythmic process(GO:0048511)	1
OG0028758	Biological Process	signaling(GO:0023052)	1
OG0028761	Biological Process	response to stimulus(GO:0050896)	1
OG0028762	Biological Process	developmental process(GO:0032502)	1
OG0028762	Biological Process	multicellular organismal process(GO:0032501)	1
OG0028762	Biological Process	reproduction(GO:0000003)	1
OG0028762	Biological Process	reproductive process(GO:0022414)	1
OG0028762	Biological Process	response to stimulus(GO:0050896)	1
OG0028766	Biological Process	developmental process(GO:0032502)	1
OG0028766	Biological Process	multicellular organismal process(GO:0032501)	1
OG0028766	Biological Process	reproduction(GO:0000003)	1
OG0028766	Biological Process	reproductive process(GO:0022414)	1
OG0028769	Biological Process	biological regulation(GO:0065007)	1
OG0028769	Biological Process	cellular process(GO:0009987)	1
OG0028769	Biological Process	developmental process(GO:0032502)	1
OG0028769	Biological Process	metabolic process(GO:0008152)	1
OG0028769	Biological Process	multicellular organismal process(GO:0032501)	1
OG0028769	Biological Process	negative regulation of biological process(GO:0048519)	1
OG0028769	Biological Process	regulation of biological process(GO:0050789)	1

OG0028769	Biological Process	reproduction(GO:0000003)	1
OG0028769	Biological Process	reproductive process(GO:0022414)	1
OG0028771	Biological Process	biological regulation(GO:0065007)	1
OG0028771	Biological Process	cellular process(GO:0009987)	1
OG0028771	Biological Process	metabolic process(GO:0008152)	1
OG0028771	Biological Process	response to stimulus(GO:0050896)	1
OG0028820	Biological Process	biological regulation(GO:0065007)	1
OG0028820	Biological Process	cellular component organization or biogenesis(GO:0071840)	1
OG0028820	Biological Process	cellular process(GO:0009987)	1
OG0028820	Biological Process	developmental process(GO:0032502)	1
OG0028820	Biological Process	growth(GO:0040007)	1
OG0028820	Biological Process	multicellular organismal process(GO:0032501)	1
OG0028820	Biological Process	positive regulation of biological process(GO:0048518)	1
OG0028820	Biological Process	regulation of biological process(GO:0050789)	1
OG0028820	Biological Process	reproduction(GO:0000003)	1
OG0028820	Biological Process	reproductive process(GO:0022414)	1
OG0028822	Biological Process	biological regulation(GO:0065007)	1
OG0028822	Biological Process	cellular component organization or biogenesis(GO:0071840)	1
OG0028822	Biological Process	cellular process(GO:0009987)	1
OG0028822	Biological Process	metabolic process(GO:0008152)	1
OG0028822	Biological Process	negative regulation of biological process(GO:0048519)	1
OG0028822	Biological Process	regulation of biological process(GO:0050789)	1
OG0028843	Biological Process	cellular process(GO:0009987)	1
OG0028843	Biological Process	localization(GO:0051179)	1
OG0028845	Biological Process	biological regulation(GO:0065007)	1
OG0028845	Biological Process	cellular process(GO:0009987)	1
OG0028845	Biological Process	developmental process(GO:0032502)	1
OG0028845	Biological Process	metabolic process(GO:0008152)	1
OG0028845	Biological Process	multicellular organismal process(GO:0032501)	1
OG0028845	Biological Process	regulation of biological process(GO:0050789)	1
OG0028845	Biological Process	reproduction(GO:0000003)	1
OG0028845	Biological Process	reproductive process(GO:0022414)	1
OG0028849	Biological Process	biological regulation(GO:0065007)	1
OG0028849	Biological Process	cellular process(GO:0009987)	1
OG0028849	Biological Process	developmental process(GO:0032502)	1
OG0028849	Biological Process	metabolic process(GO:0008152)	1
OG0028849	Biological Process	multicellular organismal process(GO:0032501)	1
OG0028849	Biological Process	negative regulation of biological process(GO:0048519)	1
OG0028849	Biological Process	regulation of biological process(GO:0050789)	1
OG0028849	Biological Process	reproduction(GO:0000003)	1
OG0028849	Biological Process	reproductive process(GO:0022414)	1
OG0028849	Biological Process	response to stimulus(GO:0050896)	1
OG0028849	Biological Process	signaling(GO:0023052)	1
OG0028859	Biological Process	biological regulation(GO:0065007)	1

OG0028859	Biological Process	cellular process(GO:0009987)	1
OG0028859	Biological Process	metabolic process(GO:0008152)	1
OG0028859	Biological Process	regulation of biological process(GO:0050789)	1
OG0028859	Biological Process	response to stimulus(GO:0050896)	1
OG0028859	Biological Process	signaling(GO:0023052)	1
OG0028869	Biological Process	response to stimulus(GO:0050896)	1
OG0028870	Biological Process	response to stimulus(GO:0050896)	1
OG0028872	Biological Process	response to stimulus(GO:0050896)	1
OG0028874	Biological Process	cellular process(GO:0009987)	1
OG0028874	Biological Process	metabolic process(GO:0008152)	1
OG0028878	Biological Process	cellular process(GO:0009987)	1
OG0028878	Biological Process	metabolic process(GO:0008152)	1
OG0028879	Biological Process	cellular component organization or biogenesis(GO:0071840)	1
OG0028879	Biological Process	cellular process(GO:0009987)	1
OG0028879	Biological Process	metabolic process(GO:0008152)	1
OG0028879	Biological Process	response to stimulus(GO:0050896)	1
OG0028903	Biological Process	cellular component organization or biogenesis(GO:0071840)	1
OG0028903	Biological Process	cellular process(GO:0009987)	1
OG0028903	Biological Process	growth(GO:0040007)	1
OG0028903	Biological Process	metabolic process(GO:0008152)	1
OG0028904	Biological Process	growth(GO:0040007)	1
OG0028907	Biological Process	cellular process(GO:0009987)	1
OG0028907	Biological Process	localization(GO:0051179)	1
OG0028907	Biological Process	metabolic process(GO:0008152)	1
OG0028908	Biological Process	biological regulation(GO:0065007)	1
OG0028908	Biological Process	cellular component organization or biogenesis(GO:0071840)	1
OG0028908	Biological Process	cellular process(GO:0009987)	1
OG0028908	Biological Process	developmental process(GO:0032502)	1
OG0028908	Biological Process	metabolic process(GO:0008152)	1
OG0028908	Biological Process	regulation of biological process(GO:0050789)	1
OG0028909	Biological Process	growth(GO:0040007)	1
OG0028910	Biological Process	cellular process(GO:0009987)	1
OG0028910	Biological Process	metabolic process(GO:0008152)	1
OG0028913	Biological Process	cellular process(GO:0009987)	1
OG0028913	Biological Process	growth(GO:0040007)	1
OG0028913	Biological Process	metabolic process(GO:0008152)	1
OG0028925	Biological Process	cellular process(GO:0009987)	1
OG0028925	Biological Process	localization(GO:0051179)	1
OG0028934	Biological Process	response to stimulus(GO:0050896)	1
OG0028941	Biological Process	biological regulation(GO:0065007)	1
OG0028941	Biological Process	cellular process(GO:0009987)	1
OG0028941	Biological Process	metabolic process(GO:0008152)	1
OG0028941	Biological Process	regulation of biological process(GO:0050789)	1
OG0028946	Biological Process	biological regulation(GO:0065007)	1
OG0028946	Biological Process	cellular process(GO:0009987)	1
OG0028946	Biological Process	metabolic process(GO:0008152)	1

OG0028946	Biological Process	regulation of biological process(GO:0050789)	1
OG0028991	Biological Process	biological regulation(GO:0065007)	1
OG0028991	Biological Process	cellular process(GO:0009987)	1
OG0028991	Biological Process	localization(GO:0051179)	1
OG0028991	Biological Process	regulation of biological process(GO:0050789)	1
OG0028991	Biological Process	response to stimulus(GO:0050896)	1
OG0028991	Biological Process	signaling(GO:0023052)	1
OG0029004	Biological Process	developmental process(GO:0032502)	1
OG0029012	Biological Process	cellular process(GO:0009987)	1
OG0029012	Biological Process	metabolic process(GO:0008152)	1
OG0029015	Biological Process	biological regulation(GO:0065007)	1
OG0029015	Biological Process	cellular component organization or biogenesis(GO:0071840)	1
OG0029015	Biological Process	cellular process(GO:0009987)	1
OG0029015	Biological Process	developmental process(GO:0032502)	1
OG0029015	Biological Process	metabolic process(GO:0008152)	1
OG0029015	Biological Process	multicellular organismal process(GO:0032501)	1
OG0029015	Biological Process	regulation of biological process(GO:0050789)	1
OG0029015	Biological Process	response to stimulus(GO:0050896)	1
OG0029015	Biological Process	signaling(GO:0023052)	1
OG0029029	Biological Process	cellular process(GO:0009987)	1
OG0029029	Biological Process	metabolic process(GO:0008152)	1
OG0029033	Biological Process	biological regulation(GO:0065007)	1
OG0029033	Biological Process	cellular process(GO:0009987)	1
OG0029033	Biological Process	metabolic process(GO:0008152)	1
OG0029033	Biological Process	regulation of biological process(GO:0050789)	1
OG0029035	Biological Process	biological regulation(GO:0065007)	1
OG0029035	Biological Process	cellular process(GO:0009987)	1
OG0029035	Biological Process	metabolic process(GO:0008152)	1
OG0029035	Biological Process	multi-organism process(GO:0051704)	1
OG0029035	Biological Process	response to stimulus(GO:0050896)	1
OG0029037	Biological Process	biological regulation(GO:0065007)	1
OG0029037	Biological Process	cellular process(GO:0009987)	1
OG0029037	Biological Process	regulation of biological process(GO:0050789)	1
OG0029037	Biological Process	response to stimulus(GO:0050896)	1
OG0029037	Biological Process	signaling(GO:0023052)	1
OG0029047	Biological Process	metabolic process(GO:0008152)	1
OG0029058	Biological Process	response to stimulus(GO:0050896)	1
OG0029059	Biological Process	response to stimulus(GO:0050896)	1
OG0029060	Biological Process	biological regulation(GO:0065007)	1
OG0029060	Biological Process	cellular process(GO:0009987)	1
OG0029060	Biological Process	metabolic process(GO:0008152)	1
OG0029060	Biological Process	regulation of biological process(GO:0050789)	1
OG0029072	Biological Process	biological adhesion(GO:0022610)	1
OG0029072	Biological Process	biological regulation(GO:0065007)	1
OG0029072	Biological Process	cellular component organization or biogenesis(GO:0071840)	1

OG0029072	Biological Process	cellular process(GO:0009987)	1
OG0029072	Biological Process	developmental process(GO:0032502)	1
OG0029072	Biological Process	localization(GO:0051179)	1
OG0029072	Biological Process	locomotion(GO:0040011)	1
OG0029072	Biological Process	metabolic process(GO:0008152)	1
OG0029072	Biological Process	multicellular organismal process(GO:0032501)	1
OG0029072	Biological Process	regulation of biological process(GO:0050789)	1
OG0029072	Biological Process	response to stimulus(GO:0050896)	1
OG0029099	Biological Process	response to stimulus(GO:0050896)	1
OG0029102	Biological Process	biological regulation(GO:0065007)	1
OG0029102	Biological Process	cellular process(GO:0009987)	1
OG0029102	Biological Process	developmental process(GO:0032502)	1
OG0029102	Biological Process	multicellular organismal process(GO:0032501)	1
OG0029102	Biological Process	negative regulation of biological process(GO:0048519)	1
OG0029102	Biological Process	regulation of biological process(GO:0050789)	1
OG0029110	Biological Process	biological regulation(GO:0065007)	1
OG0029110	Biological Process	cellular process(GO:0009987)	1
OG0029110	Biological Process	regulation of biological process(GO:0050789)	1
OG0029110	Biological Process	response to stimulus(GO:0050896)	1
OG0029110	Biological Process	sulfur utilization(GO:0006791)	1
OG0029111	Biological Process	biological regulation(GO:0065007)	1
OG0029111	Biological Process	cellular process(GO:0009987)	1
OG0029111	Biological Process	regulation of biological process(GO:0050789)	1
OG0029111	Biological Process	response to stimulus(GO:0050896)	1
OG0029111	Biological Process	sulfur utilization(GO:0006791)	1
OG0029112	Biological Process	cellular process(GO:0009987)	1
OG0029112	Biological Process	metabolic process(GO:0008152)	1
OG0029112	Biological Process	multi-organism process(GO:0051704)	1
OG0029112	Biological Process	response to stimulus(GO:0050896)	1
OG0029113	Biological Process	biological regulation(GO:0065007)	1
OG0029113	Biological Process	cellular process(GO:0009987)	1
OG0029113	Biological Process	immune system process(GO:0002376)	1
OG0029113	Biological Process	positive regulation of biological process(GO:0048518)	1
OG0029113	Biological Process	regulation of biological process(GO:0050789)	1
OG0029113	Biological Process	response to stimulus(GO:0050896)	1
OG0029113	Biological Process	signaling(GO:0023052)	1
OG0029117	Biological Process	metabolic process(GO:0008152)	1
OG0029118	Biological Process	response to stimulus(GO:0050896)	1
OG0029120	Biological Process	response to stimulus(GO:0050896)	1
OG0029122	Biological Process	cellular process(GO:0009987)	1
OG0029122	Biological Process	metabolic process(GO:0008152)	1
OG0029122	Biological Process	response to stimulus(GO:0050896)	1
OG0029124	Biological Process	biological regulation(GO:0065007)	1
OG0029124	Biological Process	cellular component organization or biogenesis(GO:0071840)	1
OG0029124	Biological Process	cellular process(GO:0009987)	1

OG0029124	Biological Process	localization(GO:0051179)	1
OG0029124	Biological Process	metabolic process(GO:0008152)	1
OG0029125	Biological Process	biological regulation(GO:0065007)	1
OG0029125	Biological Process	cellular process(GO:0009987)	1
OG0029125	Biological Process	developmental process(GO:0032502)	1
OG0029125	Biological Process	multicellular organismal process(GO:0032501)	1
OG0029125	Biological Process	positive regulation of biological process(GO:0048518)	1
OG0029125	Biological Process	regulation of biological process(GO:0050789)	1
OG0029125	Biological Process	reproduction(GO:0000003)	1
OG0029125	Biological Process	reproductive process(GO:0022414)	1
OG0029125	Biological Process	response to stimulus(GO:0050896)	1
OG0029141	Biological Process	biological regulation(GO:0065007)	1
OG0029141	Biological Process	cellular process(GO:0009987)	1
OG0029141	Biological Process	localization(GO:0051179)	1
OG0029141	Biological Process	metabolic process(GO:0008152)	1
OG0029141	Biological Process	regulation of biological process(GO:0050789)	1
OG0029141	Biological Process	response to stimulus(GO:0050896)	1
OG0029141	Biological Process	signaling(GO:0023052)	1
OG0029142	Biological Process	cellular process(GO:0009987)	1
OG0029142	Biological Process	developmental process(GO:0032502)	1
OG0029142	Biological Process	metabolic process(GO:0008152)	1
OG0029142	Biological Process	multicellular organismal process(GO:0032501)	1
OG0029142	Biological Process	response to stimulus(GO:0050896)	1
OG0029143	Biological Process	biological regulation(GO:0065007)	1
OG0029143	Biological Process	cell proliferation(GO:0008283)	1
OG0029143	Biological Process	cellular process(GO:0009987)	1
OG0029143	Biological Process	developmental process(GO:0032502)	1
OG0029143	Biological Process	metabolic process(GO:0008152)	1
OG0029143	Biological Process	multicellular organismal process(GO:0032501)	1
OG0029143	Biological Process	positive regulation of biological process(GO:0048518)	1
OG0029143	Biological Process	regulation of biological process(GO:0050789)	1
OG0029143	Biological Process	reproduction(GO:0000003)	1
OG0029143	Biological Process	reproductive process(GO:0022414)	1
OG0029143	Biological Process	response to stimulus(GO:0050896)	1
OG0029159	Biological Process	growth(GO:0040007)	1
OG0029161	Biological Process	cellular process(GO:0009987)	1
OG0029161	Biological Process	metabolic process(GO:0008152)	1
OG0029163	Biological Process	cellular process(GO:0009987)	1
OG0029163	Biological Process	metabolic process(GO:0008152)	1
OG0029165	Biological Process	biological regulation(GO:0065007)	1
OG0029165	Biological Process	cellular process(GO:0009987)	1
OG0029165	Biological Process	developmental process(GO:0032502)	1
OG0029165	Biological Process	metabolic process(GO:0008152)	1
OG0029165	Biological Process	multi-organism process(GO:0051704)	1
OG0029165	Biological Process	multicellular organismal process(GO:0032501)	1

OG0029165	Biological Process	regulation of biological process(GO:0050789)	1
OG0029165	Biological Process	reproduction(GO:0000003)	1
OG0029165	Biological Process	reproductive process(GO:0022414)	1
OG0029165	Biological Process	response to stimulus(GO:0050896)	1
OG0029168	Biological Process	biological regulation(GO:0065007)	1
OG0029168	Biological Process	cellular process(GO:0009987)	1
OG0029168	Biological Process	metabolic process(GO:0008152)	1
OG0029168	Biological Process	multi-organism process(GO:0051704)	1
OG0029168	Biological Process	negative regulation of biological process(GO:0048519)	1
OG0029168	Biological Process	regulation of biological process(GO:0050789)	1
OG0029168	Biological Process	response to stimulus(GO:0050896)	1
OG0029168	Biological Process	signaling(GO:0023052)	1
OG0029172	Biological Process	biological regulation(GO:0065007)	1
OG0029172	Biological Process	cellular process(GO:0009987)	1
OG0029172	Biological Process	localization(GO:0051179)	1
OG0029194	Biological Process	biological regulation(GO:0065007)	1
OG0029194	Biological Process	cellular process(GO:0009987)	1
OG0029194	Biological Process	metabolic process(GO:0008152)	1
OG0029194	Biological Process	regulation of biological process(GO:0050789)	1
OG0029194	Biological Process	response to stimulus(GO:0050896)	1
OG0029203	Biological Process	biological regulation(GO:0065007)	1
OG0029203	Biological Process	cellular process(GO:0009987)	1
OG0029203	Biological Process	regulation of biological process(GO:0050789)	1
OG0029203	Biological Process	response to stimulus(GO:0050896)	1
OG0029203	Biological Process	signaling(GO:0023052)	1
OG0029204	Biological Process	immune system process(GO:0002376)	1
OG0029204	Biological Process	multi-organism process(GO:0051704)	1
OG0029204	Biological Process	response to stimulus(GO:0050896)	1
OG0029213	Biological Process	cellular process(GO:0009987)	1
OG0029213	Biological Process	localization(GO:0051179)	1
OG0029223	Biological Process	biological regulation(GO:0065007)	1
OG0029223	Biological Process	cellular component organization or biogenesis(GO:0071840)	1
OG0029223	Biological Process	cellular process(GO:0009987)	1
OG0029223	Biological Process	developmental process(GO:0032502)	1
OG0029223	Biological Process	metabolic process(GO:0008152)	1
OG0029223	Biological Process	multicellular organismal process(GO:0032501)	1
OG0029223	Biological Process	negative regulation of biological process(GO:0048519)	1
OG0029223	Biological Process	regulation of biological process(GO:0050789)	1
OG0029223	Biological Process	reproduction(GO:0000003)	1
OG0029223	Biological Process	reproductive process(GO:0022414)	1
OG0029223	Biological Process	response to stimulus(GO:0050896)	1
OG0029225	Biological Process	biological regulation(GO:0065007)	1
OG0029225	Biological Process	cellular component organization or biogenesis(GO:0071840)	1
OG0029225	Biological Process	cellular process(GO:0009987)	1
OG0029225	Biological Process	developmental process(GO:0032502)	1

OG0029225	Biological Process	metabolic process(GO:0008152)	1
OG0029225	Biological Process	multicellular organismal process(GO:0032501)	1
OG0029225	Biological Process	negative regulation of biological process(GO:0048519)	1
OG0029225	Biological Process	regulation of biological process(GO:0050789)	1
OG0029225	Biological Process	reproduction(GO:0000003)	1
OG0029225	Biological Process	reproductive process(GO:0022414)	1
OG0029225	Biological Process	response to stimulus(GO:0050896)	1
OG0029226	Biological Process	biological regulation(GO:0065007)	1
OG0029226	Biological Process	cellular component organization or biogenesis(GO:0071840)	1
OG0029226	Biological Process	cellular process(GO:0009987)	1
OG0029226	Biological Process	developmental process(GO:0032502)	1
OG0029226	Biological Process	metabolic process(GO:0008152)	1
OG0029226	Biological Process	multicellular organismal process(GO:0032501)	1
OG0029226	Biological Process	negative regulation of biological process(GO:0048519)	1
OG0029226	Biological Process	regulation of biological process(GO:0050789)	1
OG0029226	Biological Process	reproduction(GO:0000003)	1
OG0029226	Biological Process	reproductive process(GO:0022414)	1
OG0029226	Biological Process	response to stimulus(GO:0050896)	1
OG0029230	Biological Process	cellular process(GO:0009987)	1
OG0029230	Biological Process	metabolic process(GO:0008152)	1
OG0029241	Biological Process	cellular process(GO:0009987)	1
OG0029241	Biological Process	metabolic process(GO:0008152)	1
OG0029254	Biological Process	cellular process(GO:0009987)	1
OG0029254	Biological Process	localization(GO:0051179)	1
OG0029254	Biological Process	response to stimulus(GO:0050896)	1
OG0029294	Biological Process	cellular process(GO:0009987)	1
OG0029294	Biological Process	metabolic process(GO:0008152)	1
OG0029295	Biological Process	cellular process(GO:0009987)	1
OG0029295	Biological Process	metabolic process(GO:0008152)	1
OG0029309	Biological Process	developmental process(GO:0032502)	1
OG0029309	Biological Process	multicellular organismal process(GO:0032501)	1
OG0029311	Biological Process	developmental process(GO:0032502)	1
OG0029311	Biological Process	multicellular organismal process(GO:0032501)	1
OG0029315	Biological Process	biological regulation(GO:0065007)	1
OG0029315	Biological Process	cellular process(GO:0009987)	1
OG0029315	Biological Process	metabolic process(GO:0008152)	1
OG0029315	Biological Process	regulation of biological process(GO:0050789)	1
OG0029316	Biological Process	biological regulation(GO:0065007)	1
OG0029316	Biological Process	cellular process(GO:0009987)	1
OG0029316	Biological Process	developmental process(GO:0032502)	1
OG0029316	Biological Process	metabolic process(GO:0008152)	1
OG0029316	Biological Process	multicellular organismal process(GO:0032501)	1
OG0029316	Biological Process	negative regulation of biological process(GO:0048519)	1
OG0029316	Biological Process	regulation of biological process(GO:0050789)	1

OG0029316	Biological Process	reproduction(GO:0000003)	1
OG0029316	Biological Process	reproductive process(GO:0022414)	1
OG0029316	Biological Process	response to stimulus(GO:0050896)	1
OG0029319	Biological Process	cellular process(GO:0009987)	1
OG0029319	Biological Process	localization(GO:0051179)	1
OG0029319	Biological Process	metabolic process(GO:0008152)	1
OG0029321	Biological Process	response to stimulus(GO:0050896)	1
OG0029323	Biological Process	biological regulation(GO:0065007)	1
OG0029323	Biological Process	cellular component organization or biogenesis(GO:0071840)	1
OG0029323	Biological Process	cellular process(GO:0009987)	1
OG0029323	Biological Process	immune system process(GO:0002376)	1
OG0029323	Biological Process	localization(GO:0051179)	1
OG0029323	Biological Process	multi-organism process(GO:0051704)	1
OG0029323	Biological Process	negative regulation of biological process(GO:0048519)	1
OG0029323	Biological Process	regulation of biological process(GO:0050789)	1
OG0029323	Biological Process	response to stimulus(GO:0050896)	1
OG0029323	Biological Process	signaling(GO:0023052)	1
OG0029327	Biological Process	cellular component organization or biogenesis(GO:0071840)	1
OG0029327	Biological Process	cellular process(GO:0009987)	1
OG0029327	Biological Process	metabolic process(GO:0008152)	1
OG0029327	Biological Process	response to stimulus(GO:0050896)	1
OG0029337	Biological Process	cellular process(GO:0009987)	1
OG0029337	Biological Process	metabolic process(GO:0008152)	1
OG0029338	Biological Process	cellular component organization or biogenesis(GO:0071840)	1
OG0029338	Biological Process	cellular process(GO:0009987)	1
OG0029338	Biological Process	growth(GO:0040007)	1
OG0029338	Biological Process	metabolic process(GO:0008152)	1
OG0029341	Biological Process	cellular process(GO:0009987)	1
OG0029341	Biological Process	growth(GO:0040007)	1
OG0029341	Biological Process	metabolic process(GO:0008152)	1
OG0029341	Biological Process	multi-organism process(GO:0051704)	1
OG0029341	Biological Process	response to stimulus(GO:0050896)	1
OG0029348	Biological Process	cellular process(GO:0009987)	1
OG0029348	Biological Process	metabolic process(GO:0008152)	1
OG0029348	Biological Process	multi-organism process(GO:0051704)	1
OG0029348	Biological Process	response to stimulus(GO:0050896)	1
OG0029355	Biological Process	cellular component organization or biogenesis(GO:0071840)	1
OG0029355	Biological Process	cellular process(GO:0009987)	1
OG0029355	Biological Process	growth(GO:0040007)	1
OG0029355	Biological Process	metabolic process(GO:0008152)	1
OG0029356	Biological Process	cellular process(GO:0009987)	1
OG0029356	Biological Process	metabolic process(GO:0008152)	1
OG0029359	Biological Process	biological regulation(GO:0065007)	1
OG0029359	Biological Process	cellular process(GO:0009987)	1
OG0029359	Biological Process	metabolic process(GO:0008152)	1
OG0029359	Biological Process	regulation of biological process(GO:0050789)	1

OG0029362	Biological Process	cellular process(GO:0009987)	1
OG0029362	Biological Process	metabolic process(GO:0008152)	1
OG0029366	Biological Process	growth(GO:0040007)	1
OG0029366	Biological Process	metabolic process(GO:0008152)	1
OG0029375	Biological Process	cellular process(GO:0009987)	1
OG0029375	Biological Process	developmental process(GO:0032502)	1
OG0029375	Biological Process	metabolic process(GO:0008152)	1
OG0029375	Biological Process	multicellular organismal process(GO:0032501)	1
OG0029375	Biological Process	reproduction(GO:0000003)	1
OG0029375	Biological Process	reproductive process(GO:0022414)	1
OG0029376	Biological Process	biological regulation(GO:0065007)	1
OG0029376	Biological Process	cellular process(GO:0009987)	1
OG0029376	Biological Process	metabolic process(GO:0008152)	1
OG0029376	Biological Process	regulation of biological process(GO:0050789)	1
OG0029376	Biological Process	response to stimulus(GO:0050896)	1
OG0029384	Biological Process	biological regulation(GO:0065007)	1
OG0029384	Biological Process	metabolic process(GO:0008152)	1
OG0029384	Biological Process	regulation of biological process(GO:0050789)	1
OG0029387	Biological Process	biological regulation(GO:0065007)	1
OG0029387	Biological Process	cellular process(GO:0009987)	1
OG0029387	Biological Process	developmental process(GO:0032502)	1
OG0029387	Biological Process	multicellular organismal process(GO:0032501)	1
OG0029387	Biological Process	positive regulation of biological process(GO:0048518)	1
OG0029387	Biological Process	regulation of biological process(GO:0050789)	1
OG0029387	Biological Process	reproduction(GO:0000003)	1
OG0029387	Biological Process	reproductive process(GO:0022414)	1
OG0029387	Biological Process	response to stimulus(GO:0050896)	1
OG0029389	Biological Process	biological regulation(GO:0065007)	1
OG0029389	Biological Process	cellular component organization or biogenesis(GO:0071840)	1
OG0029389	Biological Process	cellular process(GO:0009987)	1
OG0029389	Biological Process	developmental process(GO:0032502)	1
OG0029389	Biological Process	growth(GO:0040007)	1
OG0029389	Biological Process	metabolic process(GO:0008152)	1
OG0029389	Biological Process	multicellular organismal process(GO:0032501)	1
OG0029389	Biological Process	regulation of biological process(GO:0050789)	1
OG0029389	Biological Process	reproduction(GO:0000003)	1
OG0029389	Biological Process	reproductive process(GO:0022414)	1
OG0029389	Biological Process	response to stimulus(GO:0050896)	1
OG0029389	Biological Process	signaling(GO:0023052)	1
OG0029391	Biological Process	biological regulation(GO:0065007)	1
OG0029391	Biological Process	cellular process(GO:0009987)	1
OG0029391	Biological Process	metabolic process(GO:0008152)	1
OG0029391	Biological Process	multi-organism process(GO:0051704)	1
OG0029391	Biological Process	regulation of biological process(GO:0050789)	1
OG0029391	Biological Process	response to stimulus(GO:0050896)	1

OG0029392	Biological Process	cellular process(GO:0009987)	1
OG0029392	Biological Process	signaling(GO:0023052)	1
OG0029412	Biological Process	response to stimulus(GO:0050896)	1
OG0029420	Biological Process	developmental process(GO:0032502)	1
OG0029420	Biological Process	multicellular organismal process(GO:0032501)	1
OG0029421	Biological Process	metabolic process(GO:0008152)	1
OG0029424	Biological Process	biological regulation(GO:0065007)	1
OG0029424	Biological Process	cellular process(GO:0009987)	1
OG0029424	Biological Process	metabolic process(GO:0008152)	1
OG0029424	Biological Process	negative regulation of biological process(GO:0048519)	1
OG0029424	Biological Process	regulation of biological process(GO:0050789)	1
OG0029424	Biological Process	response to stimulus(GO:0050896)	1
OG0029426	Biological Process	biological regulation(GO:0065007)	1
OG0029426	Biological Process	cellular process(GO:0009987)	1
OG0029426	Biological Process	metabolic process(GO:0008152)	1
OG0029426	Biological Process	regulation of biological process(GO:0050789)	1
OG0029426	Biological Process	response to stimulus(GO:0050896)	1
OG0029429	Biological Process	biological regulation(GO:0065007)	1
OG0029429	Biological Process	cellular process(GO:0009987)	1
OG0029429	Biological Process	metabolic process(GO:0008152)	1
OG0029429	Biological Process	multi-organism process(GO:0051704)	1
OG0029429	Biological Process	negative regulation of biological process(GO:0048519)	1
OG0029429	Biological Process	regulation of biological process(GO:0050789)	1
OG0029429	Biological Process	response to stimulus(GO:0050896)	1
OG0029430	Biological Process	biological regulation(GO:0065007)	1
OG0029430	Biological Process	cellular process(GO:0009987)	1
OG0029430	Biological Process	metabolic process(GO:0008152)	1
OG0029430	Biological Process	multi-organism process(GO:0051704)	1
OG0029430	Biological Process	negative regulation of biological process(GO:0048519)	1
OG0029430	Biological Process	regulation of biological process(GO:0050789)	1
OG0029430	Biological Process	response to stimulus(GO:0050896)	1
OG0029431	Biological Process	biological regulation(GO:0065007)	1
OG0029431	Biological Process	cellular process(GO:0009987)	1
OG0029431	Biological Process	metabolic process(GO:0008152)	1
OG0029431	Biological Process	multi-organism process(GO:0051704)	1
OG0029431	Biological Process	negative regulation of biological process(GO:0048519)	1
OG0029431	Biological Process	regulation of biological process(GO:0050789)	1
OG0029431	Biological Process	response to stimulus(GO:0050896)	1
OG0029453	Biological Process	biological regulation(GO:0065007)	1
OG0029453	Biological Process	cellular process(GO:0009987)	1
OG0029453	Biological Process	metabolic process(GO:0008152)	1
OG0029453	Biological Process	regulation of biological process(GO:0050789)	1
OG0029457	Biological Process	cellular component organization or biogenesis(GO:0071840)	1
OG0029457	Biological Process	cellular process(GO:0009987)	1

OG0029457	Biological Process	growth(GO:0040007)	1
OG0029457	Biological Process	metabolic process(GO:0008152)	1
OG0029458	Biological Process	cellular process(GO:0009987)	1
OG0029458	Biological Process	growth(GO:0040007)	1
OG0029458	Biological Process	metabolic process(GO:0008152)	1
OG0029459	Biological Process	cellular process(GO:0009987)	1
OG0029459	Biological Process	metabolic process(GO:0008152)	1
OG0029461	Biological Process	cellular process(GO:0009987)	1
OG0029461	Biological Process	metabolic process(GO:0008152)	1
OG0029463	Biological Process	growth(GO:0040007)	1
OG0029466	Biological Process	cellular process(GO:0009987)	1
OG0029466	Biological Process	metabolic process(GO:0008152)	1
OG0029467	Biological Process	cellular process(GO:0009987)	1
OG0029467	Biological Process	metabolic process(GO:0008152)	1
OG0029469	Biological Process	cellular process(GO:0009987)	1
OG0029469	Biological Process	metabolic process(GO:0008152)	1
OG0029470	Biological Process	biological regulation(GO:0065007)	1
OG0029470	Biological Process	cellular component organization or biogenesis(GO:0071840)	1
OG0029470	Biological Process	cellular process(GO:0009987)	1
OG0029470	Biological Process	growth(GO:0040007)	1
OG0029470	Biological Process	metabolic process(GO:0008152)	1
OG0029470	Biological Process	negative regulation of biological process(GO:0048519)	1
OG0029470	Biological Process	regulation of biological process(GO:0050789)	1
OG0029471	Biological Process	biological regulation(GO:0065007)	1
OG0029471	Biological Process	cellular process(GO:0009987)	1
OG0029471	Biological Process	growth(GO:0040007)	1
OG0029471	Biological Process	metabolic process(GO:0008152)	1
OG0029471	Biological Process	positive regulation of biological process(GO:0048518)	1
OG0029471	Biological Process	regulation of biological process(GO:0050789)	1
OG0029472	Biological Process	cellular process(GO:0009987)	1
OG0029472	Biological Process	growth(GO:0040007)	1
OG0029472	Biological Process	metabolic process(GO:0008152)	1
OG0029473	Biological Process	growth(GO:0040007)	1
OG0029479	Biological Process	cellular process(GO:0009987)	1
OG0029479	Biological Process	metabolic process(GO:0008152)	1
OG0029482	Biological Process	cellular component organization or biogenesis(GO:0071840)	1
OG0029482	Biological Process	cellular process(GO:0009987)	1
OG0029482	Biological Process	growth(GO:0040007)	1
OG0029482	Biological Process	metabolic process(GO:0008152)	1
OG0029485	Biological Process	growth(GO:0040007)	1
OG0029486	Biological Process	biological regulation(GO:0065007)	1
OG0029486	Biological Process	cellular process(GO:0009987)	1
OG0029486	Biological Process	metabolic process(GO:0008152)	1
OG0029486	Biological Process	multi-organism process(GO:0051704)	1
OG0029486	Biological Process	positive regulation of biological process(GO:0048518)	1

OG0029486	Biological Process	regulation of biological process(GO:0050789)	1
OG0029486	Biological Process	response to stimulus(GO:0050896)	1
OG0029486	Biological Process	signaling(GO:0023052)	1
OG0029489	Biological Process	cellular process(GO:0009987)	1
OG0029489	Biological Process	metabolic process(GO:0008152)	1
OG0029490	Biological Process	cellular component organization or biogenesis(GO:0071840)	1
OG0029490	Biological Process	cellular process(GO:0009987)	1
OG0029490	Biological Process	growth(GO:0040007)	1
OG0029490	Biological Process	metabolic process(GO:0008152)	1
OG0029493	Biological Process	cellular process(GO:0009987)	1
OG0029493	Biological Process	metabolic process(GO:0008152)	1
OG0029497	Biological Process	growth(GO:0040007)	1
OG0029497	Biological Process	metabolic process(GO:0008152)	1
OG0029500	Biological Process	growth(GO:0040007)	1
OG0029502	Biological Process	cellular process(GO:0009987)	1
OG0029502	Biological Process	metabolic process(GO:0008152)	1
OG0029504	Biological Process	cellular process(GO:0009987)	1
OG0029504	Biological Process	metabolic process(GO:0008152)	1
OG0029504	Biological Process	response to stimulus(GO:0050896)	1
OG0029509	Biological Process	cellular component organization or biogenesis(GO:0071840)	1
OG0029509	Biological Process	cellular process(GO:0009987)	1
OG0029509	Biological Process	localization(GO:0051179)	1
OG0029517	Biological Process	cellular process(GO:0009987)	1
OG0029517	Biological Process	metabolic process(GO:0008152)	1
OG0029522	Biological Process	cellular component organization or biogenesis(GO:0071840)	1
OG0029522	Biological Process	cellular process(GO:0009987)	1
OG0029522	Biological Process	growth(GO:0040007)	1
OG0029522	Biological Process	metabolic process(GO:0008152)	1
OG0029522	Biological Process	response to stimulus(GO:0050896)	1
OG0029523	Biological Process	growth(GO:0040007)	1
OG0029527	Biological Process	cellular process(GO:0009987)	1
OG0029527	Biological Process	growth(GO:0040007)	1
OG0029527	Biological Process	metabolic process(GO:0008152)	1
OG0029528	Biological Process	cellular process(GO:0009987)	1
OG0029528	Biological Process	metabolic process(GO:0008152)	1
OG0029536	Biological Process	growth(GO:0040007)	1
OG0029539	Biological Process	cellular process(GO:0009987)	1
OG0029539	Biological Process	growth(GO:0040007)	1
OG0029539	Biological Process	metabolic process(GO:0008152)	1
OG0029540	Biological Process	cellular process(GO:0009987)	1
OG0029540	Biological Process	growth(GO:0040007)	1
OG0029540	Biological Process	metabolic process(GO:0008152)	1
OG0029542	Biological Process	cellular process(GO:0009987)	1
OG0029542	Biological Process	metabolic process(GO:0008152)	1
OG0029543	Biological Process	biological regulation(GO:0065007)	1
OG0029543	Biological Process	cellular component organization or biogenesis(GO:0071840)	1

OG0029543	Biological Process	cellular process(GO:0009987)	1
OG0029543	Biological Process	metabolic process(GO:0008152)	1
OG0029543	Biological Process	regulation of biological process(GO:0050789)	1
OG0029543	Biological Process	response to stimulus(GO:0050896)	1
OG0029548	Biological Process	cellular process(GO:0009987)	1
OG0029548	Biological Process	metabolic process(GO:0008152)	1
OG0029548	Biological Process	response to stimulus(GO:0050896)	1
OG0029549	Biological Process	metabolic process(GO:0008152)	1
OG0029549	Biological Process	response to stimulus(GO:0050896)	1
OG0029550	Biological Process	cellular process(GO:0009987)	1
OG0029550	Biological Process	metabolic process(GO:0008152)	1
OG0029553	Biological Process	cellular process(GO:0009987)	1
OG0029553	Biological Process	metabolic process(GO:0008152)	1
OG0029554	Biological Process	cellular process(GO:0009987)	1
OG0029554	Biological Process	localization(GO:0051179)	1
OG0029554	Biological Process	metabolic process(GO:0008152)	1
OG0029560	Biological Process	growth(GO:0040007)	1
OG0029562	Biological Process	growth(GO:0040007)	1
OG0029567	Biological Process	biological regulation(GO:0065007)	1
OG0029567	Biological Process	cellular process(GO:0009987)	1
OG0029567	Biological Process	metabolic process(GO:0008152)	1
OG0029567	Biological Process	regulation of biological process(GO:0050789)	1
OG0029568	Biological Process	cellular process(GO:0009987)	1
OG0029568	Biological Process	metabolic process(GO:0008152)	1
OG0029568	Biological Process	response to stimulus(GO:0050896)	1
OG0029569	Biological Process	cellular process(GO:0009987)	1
OG0029569	Biological Process	metabolic process(GO:0008152)	1
OG0029571	Biological Process	cellular process(GO:0009987)	1
OG0029571	Biological Process	metabolic process(GO:0008152)	1
OG0029576	Biological Process	cellular process(GO:0009987)	1
OG0029576	Biological Process	localization(GO:0051179)	1
OG0029576	Biological Process	metabolic process(GO:0008152)	1
OG0029577	Biological Process	cellular process(GO:0009987)	1
OG0029577	Biological Process	metabolic process(GO:0008152)	1
OG0029578	Biological Process	cellular process(GO:0009987)	1
OG0029578	Biological Process	growth(GO:0040007)	1
OG0029578	Biological Process	metabolic process(GO:0008152)	1
OG0029581	Biological Process	metabolic process(GO:0008152)	1
OG0029584	Biological Process	developmental process(GO:0032502)	1
OG0029584	Biological Process	multicellular organismal process(GO:0032501)	1
OG0029584	Biological Process	reproduction(GO:0000003)	1
OG0029584	Biological Process	reproductive process(GO:0022414)	1
OG0029585	Biological Process	metabolic process(GO:0008152)	1
OG0029586	Biological Process	biological regulation(GO:0065007)	1
OG0029586	Biological Process	cellular process(GO:0009987)	1
OG0029586	Biological Process	developmental process(GO:0032502)	1

OG0029586	Biological Process	multicellular organismal process(GO:0032501)	1
OG0029586	Biological Process	regulation of biological process(GO:0050789)	1
OG0029586	Biological Process	response to stimulus(GO:0050896)	1
OG0029586	Biological Process	signaling(GO:0023052)	1
OG0029588	Biological Process	biological regulation(GO:0065007)	1
OG0029588	Biological Process	cellular process(GO:0009987)	1
OG0029588	Biological Process	metabolic process(GO:0008152)	1
OG0029588	Biological Process	regulation of biological process(GO:0050789)	1
OG0029590	Biological Process	cellular process(GO:0009987)	1
OG0029590	Biological Process	metabolic process(GO:0008152)	1
OG0029610	Biological Process	biological regulation(GO:0065007)	1
OG0029610	Biological Process	cellular process(GO:0009987)	1
OG0029610	Biological Process	developmental process(GO:0032502)	1
OG0029610	Biological Process	metabolic process(GO:0008152)	1
OG0029610	Biological Process	multicellular organismal process(GO:0032501)	1
OG0029610	Biological Process	regulation of biological process(GO:0050789)	1
OG0029610	Biological Process	reproduction(GO:0000003)	1
OG0029610	Biological Process	reproductive process(GO:0022414)	1
OG0029610	Biological Process	response to stimulus(GO:0050896)	1
OG0029610	Biological Process	signaling(GO:0023052)	1
OG0029621	Biological Process	biological regulation(GO:0065007)	1
OG0029621	Biological Process	cellular process(GO:0009987)	1
OG0029621	Biological Process	regulation of biological process(GO:0050789)	1
OG0029621	Biological Process	response to stimulus(GO:0050896)	1
OG0029621	Biological Process	signaling(GO:0023052)	1
OG0029626	Biological Process	biological regulation(GO:0065007)	1
OG0029626	Biological Process	cellular process(GO:0009987)	1
OG0029626	Biological Process	localization(GO:0051179)	1
OG0029626	Biological Process	multi-organism process(GO:0051704)	1
OG0029626	Biological Process	regulation of biological process(GO:0050789)	1
OG0029626	Biological Process	response to stimulus(GO:0050896)	1
OG0029632	Biological Process	biological regulation(GO:0065007)	1
OG0029632	Biological Process	cellular process(GO:0009987)	1
OG0029632	Biological Process	metabolic process(GO:0008152)	1
OG0029632	Biological Process	regulation of biological process(GO:0050789)	1
OG0029636	Biological Process	biological regulation(GO:0065007)	1
OG0029636	Biological Process	cellular component organization or biogenesis(GO:0071840)	1
OG0029636	Biological Process	cellular process(GO:0009987)	1
OG0029636	Biological Process	developmental process(GO:0032502)	1
OG0029636	Biological Process	regulation of biological process(GO:0050789)	1
OG0029636	Biological Process	response to stimulus(GO:0050896)	1
OG0029637	Biological Process	biological regulation(GO:0065007)	1
OG0029637	Biological Process	cellular component organization or biogenesis(GO:0071840)	1
OG0029637	Biological Process	cellular process(GO:0009987)	1
OG0029637	Biological Process	developmental process(GO:0032502)	1

OG0029637	Biological Process	regulation of biological process(GO:0050789)	1
OG0029637	Biological Process	response to stimulus(GO:0050896)	1
OG0029640	Biological Process	metabolic process(GO:0008152)	1
OG0029640	Biological Process	response to stimulus(GO:0050896)	1
OG0029645	Biological Process	biological regulation(GO:0065007)	1
OG0029645	Biological Process	cellular process(GO:0009987)	1
OG0029645	Biological Process	metabolic process(GO:0008152)	1
OG0029645	Biological Process	regulation of biological process(GO:0050789)	1
OG0029645	Biological Process	response to stimulus(GO:0050896)	1
OG0029645	Biological Process	signaling(GO:0023052)	1
OG0029648	Biological Process	biological regulation(GO:0065007)	1
OG0029648	Biological Process	cellular process(GO:0009987)	1
OG0029648	Biological Process	metabolic process(GO:0008152)	1
OG0029648	Biological Process	regulation of biological process(GO:0050789)	1
OG0029650	Biological Process	metabolic process(GO:0008152)	1
OG0029651	Biological Process	cellular process(GO:0009987)	1
OG0029651	Biological Process	metabolic process(GO:0008152)	1
OG0029651	Biological Process	rhythmic process(GO:0048511)	1
OG0029656	Biological Process	cellular component organization or biogenesis(GO:0071840)	1
OG0029656	Biological Process	cellular process(GO:0009987)	1
OG0029656	Biological Process	metabolic process(GO:0008152)	1
OG0029660	Biological Process	biological regulation(GO:0065007)	1
OG0029660	Biological Process	cellular process(GO:0009987)	1
OG0029660	Biological Process	metabolic process(GO:0008152)	1
OG0029665	Biological Process	biological regulation(GO:0065007)	1
OG0029665	Biological Process	cellular process(GO:0009987)	1
OG0029665	Biological Process	metabolic process(GO:0008152)	1
OG0029665	Biological Process	negative regulation of biological process(GO:0048519)	1
OG0029665	Biological Process	regulation of biological process(GO:0050789)	1
OG0029679	Biological Process	biological regulation(GO:0065007)	1
OG0029679	Biological Process	cellular process(GO:0009987)	1
OG0029679	Biological Process	developmental process(GO:0032502)	1
OG0029679	Biological Process	metabolic process(GO:0008152)	1
OG0029679	Biological Process	multicellular organismal process(GO:0032501)	1
OG0029679	Biological Process	regulation of biological process(GO:0050789)	1
OG0029679	Biological Process	reproduction(GO:0000003)	1
OG0029679	Biological Process	reproductive process(GO:0022414)	1
OG0029683	Biological Process	response to stimulus(GO:0050896)	1
OG0029690	Biological Process	cellular process(GO:0009987)	1
OG0029690	Biological Process	growth(GO:0040007)	1
OG0029690	Biological Process	metabolic process(GO:0008152)	1
OG0029691	Biological Process	cellular process(GO:0009987)	1
OG0029691	Biological Process	metabolic process(GO:0008152)	1
OG0029692	Biological Process	cellular process(GO:0009987)	1
OG0029692	Biological Process	metabolic process(GO:0008152)	1

OG0029741	Biological Process	response to stimulus(GO:0050896)	1
OG0029744	Biological Process	cellular process(GO:0009987)	1
OG0029744	Biological Process	detoxification(GO:0098754)	1
OG0029744	Biological Process	metabolic process(GO:0008152)	1
OG0029744	Biological Process	response to stimulus(GO:0050896)	1
OG0029750	Biological Process	cellular process(GO:0009987)	1
OG0029750	Biological Process	multi-organism process(GO:0051704)	1
OG0029753	Biological Process	biological regulation(GO:0065007)	1
OG0029753	Biological Process	cellular process(GO:0009987)	1
OG0029753	Biological Process	metabolic process(GO:0008152)	1
OG0029753	Biological Process	regulation of biological process(GO:0050789)	1
OG0029757	Biological Process	cellular component organization or biogenesis(GO:0071840)	1
OG0029757	Biological Process	cellular process(GO:0009987)	1
OG0029757	Biological Process	metabolic process(GO:0008152)	1
OG0029758	Biological Process	biological regulation(GO:0065007)	1
OG0029758	Biological Process	cellular process(GO:0009987)	1
OG0029758	Biological Process	developmental process(GO:0032502)	1
OG0029758	Biological Process	metabolic process(GO:0008152)	1
OG0029758	Biological Process	multi-organism process(GO:0051704)	1
OG0029758	Biological Process	multicellular organismal process(GO:0032501)	1
OG0029758	Biological Process	regulation of biological process(GO:0050789)	1
OG0029758	Biological Process	reproduction(GO:0000003)	1
OG0029758	Biological Process	reproductive process(GO:0022414)	1
OG0029758	Biological Process	response to stimulus(GO:0050896)	1
OG0029768	Biological Process	cellular process(GO:0009987)	1
OG0029768	Biological Process	developmental process(GO:0032502)	1
OG0029768	Biological Process	metabolic process(GO:0008152)	1
OG0029768	Biological Process	multicellular organismal process(GO:0032501)	1
OG0029777	Biological Process	metabolic process(GO:0008152)	1
OG0029788	Biological Process	cellular component organization or biogenesis(GO:0071840)	1
OG0029788	Biological Process	cellular process(GO:0009987)	1
OG0029788	Biological Process	metabolic process(GO:0008152)	1
OG0029788	Biological Process	response to stimulus(GO:0050896)	1
OG0029791	Biological Process	metabolic process(GO:0008152)	1
OG0029807	Biological Process	cellular process(GO:0009987)	1
OG0029807	Biological Process	metabolic process(GO:0008152)	1
OG0029810	Biological Process	biological regulation(GO:0065007)	1
OG0029810	Biological Process	multi-organism process(GO:0051704)	1
OG0029810	Biological Process	response to stimulus(GO:0050896)	1
OG0029812	Biological Process	cellular component organization or biogenesis(GO:0071840)	1
OG0029812	Biological Process	cellular process(GO:0009987)	1
OG0029812	Biological Process	metabolic process(GO:0008152)	1
OG0029813	Biological Process	biological regulation(GO:0065007)	1
OG0029813	Biological Process	cellular process(GO:0009987)	1
OG0029813	Biological Process	metabolic process(GO:0008152)	1

OG0029813	Biological Process	negative regulation of biological process(GO:0048519)	1
OG0029813	Biological Process	regulation of biological process(GO:0050789)	1
OG0029813	Biological Process	response to stimulus(GO:0050896)	1
OG0029813	Biological Process	signaling(GO:0023052)	1
OG0029820	Biological Process	biological regulation(GO:0065007)	1
OG0029820	Biological Process	cellular process(GO:0009987)	1
OG0029820	Biological Process	developmental process(GO:0032502)	1
OG0029820	Biological Process	metabolic process(GO:0008152)	1
OG0029820	Biological Process	multicellular organismal process(GO:0032501)	1
OG0029820	Biological Process	negative regulation of biological process(GO:0048519)	1
OG0029820	Biological Process	regulation of biological process(GO:0050789)	1
OG0029820	Biological Process	reproduction(GO:0000003)	1
OG0029820	Biological Process	reproductive process(GO:0022414)	1
OG0029820	Biological Process	response to stimulus(GO:0050896)	1
OG0029828	Biological Process	response to stimulus(GO:0050896)	1
OG0029837	Biological Process	biological regulation(GO:0065007)	1
OG0029837	Biological Process	cellular process(GO:0009987)	1
OG0029837	Biological Process	metabolic process(GO:0008152)	1
OG0029837	Biological Process	regulation of biological process(GO:0050789)	1
OG0029837	Biological Process	response to stimulus(GO:0050896)	1
OG0029837	Biological Process	signaling(GO:0023052)	1
OG0029839	Biological Process	cellular process(GO:0009987)	1
OG0029839	Biological Process	metabolic process(GO:0008152)	1
OG0029840	Biological Process	cellular process(GO:0009987)	1
OG0029840	Biological Process	metabolic process(GO:0008152)	1
OG0029841	Biological Process	growth(GO:0040007)	1
OG0029842	Biological Process	biological regulation(GO:0065007)	1
OG0029842	Biological Process	cellular component organization or biogenesis(GO:0071840)	1
OG0029842	Biological Process	cellular process(GO:0009987)	1
OG0029842	Biological Process	developmental process(GO:0032502)	1
OG0029842	Biological Process	localization(GO:0051179)	1
OG0029842	Biological Process	metabolic process(GO:0008152)	1
OG0029842	Biological Process	positive regulation of biological process(GO:0048518)	1
OG0029842	Biological Process	regulation of biological process(GO:0050789)	1
OG0029842	Biological Process	reproduction(GO:0000003)	1
OG0029842	Biological Process	reproductive process(GO:0022414)	1
OG0029842	Biological Process	response to stimulus(GO:0050896)	1
OG0029842	Biological Process	signaling(GO:0023052)	1
OG0029845	Biological Process	cellular process(GO:0009987)	1
OG0029845	Biological Process	metabolic process(GO:0008152)	1
OG0029847	Biological Process	cellular process(GO:0009987)	1
OG0029847	Biological Process	localization(GO:0051179)	1
OG0029847	Biological Process	metabolic process(GO:0008152)	1
OG0029847	Biological Process	response to stimulus(GO:0050896)	1
OG0029848	Biological Process	cellular process(GO:0009987)	1

OG0029848	Biological Process	metabolic process(GO:0008152)	1
OG0029848	Biological Process	response to stimulus(GO:0050896)	1
OG0029853	Biological Process	cellular process(GO:0009987)	1
OG0029853	Biological Process	metabolic process(GO:0008152)	1
OG0029854	Biological Process	cellular component organization or biogenesis(GO:0071840)	1
OG0029854	Biological Process	cellular process(GO:0009987)	1
OG0029854	Biological Process	metabolic process(GO:0008152)	1
OG0029860	Biological Process	cellular component organization or biogenesis(GO:0071840)	1
OG0029860	Biological Process	cellular process(GO:0009987)	1
OG0029860	Biological Process	growth(GO:0040007)	1
OG0029860	Biological Process	localization(GO:0051179)	1
OG0029860	Biological Process	metabolic process(GO:0008152)	1
OG0029861	Biological Process	cellular process(GO:0009987)	1
OG0029861	Biological Process	metabolic process(GO:0008152)	1
OG0029862	Biological Process	cellular process(GO:0009987)	1
OG0029862	Biological Process	metabolic process(GO:0008152)	1
OG0029865	Biological Process	biological regulation(GO:0065007)	1
OG0029865	Biological Process	cellular process(GO:0009987)	1
OG0029865	Biological Process	metabolic process(GO:0008152)	1
OG0029869	Biological Process	cellular process(GO:0009987)	1
OG0029869	Biological Process	metabolic process(GO:0008152)	1
OG0029876	Biological Process	cellular process(GO:0009987)	1
OG0029876	Biological Process	metabolic process(GO:0008152)	1
OG0029878	Biological Process	cellular component organization or biogenesis(GO:0071840)	1
OG0029878	Biological Process	cellular process(GO:0009987)	1
OG0029878	Biological Process	metabolic process(GO:0008152)	1
OG0029881	Biological Process	cellular process(GO:0009987)	1
OG0029881	Biological Process	metabolic process(GO:0008152)	1
OG0029882	Biological Process	cellular process(GO:0009987)	1
OG0029882	Biological Process	metabolic process(GO:0008152)	1
OG0029884	Biological Process	cellular process(GO:0009987)	1
OG0029884	Biological Process	growth(GO:0040007)	1
OG0029884	Biological Process	metabolic process(GO:0008152)	1
OG0029886	Biological Process	cellular process(GO:0009987)	1
OG0029886	Biological Process	growth(GO:0040007)	1
OG0029886	Biological Process	metabolic process(GO:0008152)	1
OG0029893	Biological Process	cellular process(GO:0009987)	1
OG0029893	Biological Process	metabolic process(GO:0008152)	1
OG0029894	Biological Process	cellular process(GO:0009987)	1
OG0029894	Biological Process	growth(GO:0040007)	1
OG0029894	Biological Process	metabolic process(GO:0008152)	1
OG0029895	Biological Process	cellular process(GO:0009987)	1
OG0029895	Biological Process	metabolic process(GO:0008152)	1
OG0029895	Biological Process	response to stimulus(GO:0050896)	1
OG0029904	Biological Process	cellular process(GO:0009987)	1
OG0029904	Biological Process	metabolic process(GO:0008152)	1

OG0029906	Biological Process	cellular process(GO:0009987)	1
OG0029906	Biological Process	metabolic process(GO:0008152)	1
OG0029907	Biological Process	localization(GO:0051179)	1
OG0029907	Biological Process	response to stimulus(GO:0050896)	1
OG0029908	Biological Process	cellular process(GO:0009987)	1
OG0029908	Biological Process	metabolic process(GO:0008152)	1
OG0029909	Biological Process	biological regulation(GO:0065007)	1
OG0029909	Biological Process	cellular component organization or biogenesis(GO:0071840)	1
OG0029909	Biological Process	cellular process(GO:0009987)	1
OG0029909	Biological Process	localization(GO:0051179)	1
OG0029909	Biological Process	response to stimulus(GO:0050896)	1
OG0029910	Biological Process	cellular process(GO:0009987)	1
OG0029910	Biological Process	growth(GO:0040007)	1
OG0029910	Biological Process	metabolic process(GO:0008152)	1
OG0029913	Biological Process	biological regulation(GO:0065007)	1
OG0029913	Biological Process	cellular process(GO:0009987)	1
OG0029913	Biological Process	metabolic process(GO:0008152)	1
OG0029913	Biological Process	negative regulation of biological process(GO:0048519)	1
OG0029913	Biological Process	regulation of biological process(GO:0050789)	1
OG0029914	Biological Process	cellular process(GO:0009987)	1
OG0029914	Biological Process	growth(GO:0040007)	1
OG0029914	Biological Process	metabolic process(GO:0008152)	1
OG0029921	Biological Process	biological regulation(GO:0065007)	1
OG0029921	Biological Process	cellular process(GO:0009987)	1
OG0029921	Biological Process	metabolic process(GO:0008152)	1
OG0029921	Biological Process	regulation of biological process(GO:0050789)	1
OG0029926	Biological Process	growth(GO:0040007)	1
OG0029928	Biological Process	cellular component organization or biogenesis(GO:0071840)	1
OG0029928	Biological Process	cellular process(GO:0009987)	1
OG0029928	Biological Process	metabolic process(GO:0008152)	1
OG0029932	Biological Process	growth(GO:0040007)	1
OG0029932	Biological Process	metabolic process(GO:0008152)	1
OG0029936	Biological Process	cellular process(GO:0009987)	1
OG0029936	Biological Process	metabolic process(GO:0008152)	1
OG0029936	Biological Process	response to stimulus(GO:0050896)	1
OG0029937	Biological Process	cellular process(GO:0009987)	1
OG0029937	Biological Process	localization(GO:0051179)	1
OG0029937	Biological Process	metabolic process(GO:0008152)	1
OG0029937	Biological Process	response to stimulus(GO:0050896)	1
OG0029941	Biological Process	cellular component organization or biogenesis(GO:0071840)	1
OG0029941	Biological Process	cellular process(GO:0009987)	1
OG0029941	Biological Process	growth(GO:0040007)	1
OG0029941	Biological Process	metabolic process(GO:0008152)	1
OG0029951	Biological Process	cellular process(GO:0009987)	1
OG0029951	Biological Process	metabolic process(GO:0008152)	1
OG0029952	Biological Process	cellular process(GO:0009987)	1

OG0029952	Biological Process	metabolic process(GO:0008152)	1
OG0029957	Biological Process	biological regulation(GO:0065007)	1
OG0029957	Biological Process	cellular process(GO:0009987)	1
OG0029957	Biological Process	developmental process(GO:0032502)	1
OG0029957	Biological Process	metabolic process(GO:0008152)	1
OG0029957	Biological Process	multicellular organismal process(GO:0032501)	1
OG0029957	Biological Process	regulation of biological process(GO:0050789)	1
OG0029957	Biological Process	reproduction(GO:0000003)	1
OG0029957	Biological Process	reproductive process(GO:0022414)	1
OG0029957	Biological Process	response to stimulus(GO:0050896)	1
OG0029959	Biological Process	growth(GO:0040007)	1
OG0029961	Biological Process	metabolic process(GO:0008152)	1
OG0029962	Biological Process	cellular process(GO:0009987)	1
OG0029962	Biological Process	metabolic process(GO:0008152)	1
OG0029964	Biological Process	cellular process(GO:0009987)	1
OG0029964	Biological Process	growth(GO:0040007)	1
OG0029964	Biological Process	metabolic process(GO:0008152)	1
OG0029964	Biological Process	response to stimulus(GO:0050896)	1
OG0029965	Biological Process	cellular component organization or biogenesis(GO:0071840)	1
OG0029965	Biological Process	cellular process(GO:0009987)	1
OG0029965	Biological Process	growth(GO:0040007)	1
OG0029965	Biological Process	metabolic process(GO:0008152)	1
OG0029967	Biological Process	cellular process(GO:0009987)	1
OG0029967	Biological Process	metabolic process(GO:0008152)	1
OG0029968	Biological Process	cellular process(GO:0009987)	1
OG0029968	Biological Process	growth(GO:0040007)	1
OG0029968	Biological Process	metabolic process(GO:0008152)	1
OG0029971	Biological Process	cellular process(GO:0009987)	1
OG0029971	Biological Process	growth(GO:0040007)	1
OG0029971	Biological Process	metabolic process(GO:0008152)	1
OG0029972	Biological Process	cellular process(GO:0009987)	1
OG0029972	Biological Process	growth(GO:0040007)	1
OG0029972	Biological Process	metabolic process(GO:0008152)	1
OG0029973	Biological Process	cellular component organization or biogenesis(GO:0071840)	1
OG0029973	Biological Process	cellular process(GO:0009987)	1
OG0029973	Biological Process	growth(GO:0040007)	1
OG0029973	Biological Process	metabolic process(GO:0008152)	1
OG0029974	Biological Process	metabolic process(GO:0008152)	1
OG0029975	Biological Process	cellular process(GO:0009987)	1
OG0029975	Biological Process	metabolic process(GO:0008152)	1
OG0029983	Biological Process	biological regulation(GO:0065007)	1
OG0029983	Biological Process	cellular process(GO:0009987)	1
OG0029983	Biological Process	metabolic process(GO:0008152)	1
OG0029983	Biological Process	regulation of biological process(GO:0050789)	1
OG0029986	Biological Process	response to stimulus(GO:0050896)	1
OG0029994	Biological Process	cellular process(GO:0009987)	1

OG0029994	Biological Process	developmental process(GO:0032502)	1
OG0029994	Biological Process	growth(GO:0040007)	1
OG0029994	Biological Process	localization(GO:0051179)	1
OG0029994	Biological Process	multicellular organismal process(GO:0032501)	1
OG0029994	Biological Process	reproduction(GO:0000003)	1
OG0029994	Biological Process	reproductive process(GO:0022414)	1
OG0029999	Biological Process	multi-organism process(GO:0051704)	1
OG0029999	Biological Process	response to stimulus(GO:0050896)	1
OG0030001	Biological Process	cellular process(GO:0009987)	1
OG0030001	Biological Process	multi-organism process(GO:0051704)	1
OG0030001	Biological Process	response to stimulus(GO:0050896)	1
OG0030002	Biological Process	developmental process(GO:0032502)	1
OG0030002	Biological Process	multicellular organismal process(GO:0032501)	1
OG0030002	Biological Process	reproduction(GO:0000003)	1
OG0030002	Biological Process	reproductive process(GO:0022414)	1
OG0030004	Biological Process	developmental process(GO:0032502)	1
OG0030004	Biological Process	multicellular organismal process(GO:0032501)	1
OG0030004	Biological Process	reproduction(GO:0000003)	1
OG0030004	Biological Process	reproductive process(GO:0022414)	1
OG0030006	Biological Process	biological regulation(GO:0065007)	1
OG0030006	Biological Process	cellular process(GO:0009987)	1
OG0030006	Biological Process	metabolic process(GO:0008152)	1
OG0030006	Biological Process	regulation of biological process(GO:0050789)	1
OG0030006	Biological Process	response to stimulus(GO:0050896)	1
OG0030006	Biological Process	signaling(GO:0023052)	1
OG0030014	Biological Process	cellular process(GO:0009987)	1
OG0030014	Biological Process	metabolic process(GO:0008152)	1
OG0030014	Biological Process	response to stimulus(GO:0050896)	1
OG0030016	Biological Process	biological regulation(GO:0065007)	1
OG0030016	Biological Process	cellular process(GO:0009987)	1
OG0030016	Biological Process	regulation of biological process(GO:0050789)	1
OG0030016	Biological Process	response to stimulus(GO:0050896)	1
OG0030016	Biological Process	signaling(GO:0023052)	1
OG0030019	Biological Process	cellular process(GO:0009987)	1
OG0030019	Biological Process	metabolic process(GO:0008152)	1
OG0030019	Biological Process	response to stimulus(GO:0050896)	1
OG0030021	Biological Process	cellular process(GO:0009987)	1
OG0030021	Biological Process	metabolic process(GO:0008152)	1
OG0030027	Biological Process	cellular component organization or biogenesis(GO:0071840)	1
OG0030027	Biological Process	cellular process(GO:0009987)	1
OG0030027	Biological Process	developmental process(GO:0032502)	1
OG0030027	Biological Process	growth(GO:0040007)	1
OG0030027	Biological Process	multi-organism process(GO:0051704)	1
OG0030027	Biological Process	multicellular organismal process(GO:0032501)	1
OG0030027	Biological Process	reproduction(GO:0000003)	1
OG0030027	Biological Process	reproductive process(GO:0022414)	1

OG0030028	Biological Process	cellular process(GO:0009987)	1
OG0030028	Biological Process	metabolic process(GO:0008152)	1
OG0030028	Biological Process	response to stimulus(GO:0050896)	1
OG0030032	Biological Process	cellular process(GO:0009987)	1
OG0030032	Biological Process	growth(GO:0040007)	1
OG0030032	Biological Process	metabolic process(GO:0008152)	1
OG0030033	Biological Process	cellular process(GO:0009987)	1
OG0030033	Biological Process	growth(GO:0040007)	1
OG0030033	Biological Process	metabolic process(GO:0008152)	1
OG0030035	Biological Process	cellular process(GO:0009987)	1
OG0030035	Biological Process	metabolic process(GO:0008152)	1
OG0030036	Biological Process	cellular process(GO:0009987)	1
OG0030036	Biological Process	localization(GO:0051179)	1
OG0030036	Biological Process	metabolic process(GO:0008152)	1
OG0030037	Biological Process	cellular process(GO:0009987)	1
OG0030037	Biological Process	metabolic process(GO:0008152)	1
OG0030045	Biological Process	biological regulation(GO:0065007)	1
OG0030045	Biological Process	cellular process(GO:0009987)	1
OG0030045	Biological Process	metabolic process(GO:0008152)	1
OG0030045	Biological Process	regulation of biological process(GO:0050789)	1
OG0030045	Biological Process	response to stimulus(GO:0050896)	1
OG0030046	Biological Process	biological regulation(GO:0065007)	1
OG0030046	Biological Process	cellular process(GO:0009987)	1
OG0030046	Biological Process	metabolic process(GO:0008152)	1
OG0030046	Biological Process	regulation of biological process(GO:0050789)	1
OG0030046	Biological Process	response to stimulus(GO:0050896)	1
OG0030049	Biological Process	biological regulation(GO:0065007)	1
OG0030049	Biological Process	cellular process(GO:0009987)	1
OG0030049	Biological Process	metabolic process(GO:0008152)	1
OG0030049	Biological Process	positive regulation of biological process(GO:0048518)	1
OG0030049	Biological Process	regulation of biological process(GO:0050789)	1
OG0030049	Biological Process	response to stimulus(GO:0050896)	1
OG0030051	Biological Process	cellular component organization or biogenesis(GO:0071840)	1
OG0030051	Biological Process	cellular process(GO:0009987)	1
OG0030051	Biological Process	multicellular organismal process(GO:0032501)	1
OG0030051	Biological Process	response to stimulus(GO:0050896)	1
OG0030057	Biological Process	biological regulation(GO:0065007)	1
OG0030057	Biological Process	cellular process(GO:0009987)	1
OG0030057	Biological Process	developmental process(GO:0032502)	1
OG0030057	Biological Process	metabolic process(GO:0008152)	1
OG0030057	Biological Process	multicellular organismal process(GO:0032501)	1
OG0030057	Biological Process	positive regulation of biological process(GO:0048518)	1
OG0030057	Biological Process	regulation of biological process(GO:0050789)	1
OG0030057	Biological Process	response to stimulus(GO:0050896)	1
OG0030057	Biological Process	signaling(GO:0023052)	1

OG0030061	Biological Process	localization(GO:0051179)	1
OG0030062	Biological Process	biological regulation(GO:0065007)	1
OG0030062	Biological Process	cellular component organization or biogenesis(GO:0071840)	1
OG0030062	Biological Process	cellular process(GO:0009987)	1
OG0030062	Biological Process	developmental process(GO:0032502)	1
OG0030062	Biological Process	localization(GO:0051179)	1
OG0030062	Biological Process	metabolic process(GO:0008152)	1
OG0030062	Biological Process	multicellular organismal process(GO:0032501)	1
OG0030062	Biological Process	negative regulation of biological process(GO:0048519)	1
OG0030062	Biological Process	regulation of biological process(GO:0050789)	1
OG0030062	Biological Process	reproduction(GO:0000003)	1
OG0030062	Biological Process	reproductive process(GO:0022414)	1
OG0030062	Biological Process	response to stimulus(GO:0050896)	1
OG0030063	Biological Process	biological regulation(GO:0065007)	1
OG0030063	Biological Process	cellular process(GO:0009987)	1
OG0030063	Biological Process	metabolic process(GO:0008152)	1
OG0030063	Biological Process	positive regulation of biological process(GO:0048518)	1
OG0030063	Biological Process	regulation of biological process(GO:0050789)	1
OG0030063	Biological Process	response to stimulus(GO:0050896)	1
OG0030065	Biological Process	cellular process(GO:0009987)	1
OG0030065	Biological Process	developmental process(GO:0032502)	1
OG0030065	Biological Process	metabolic process(GO:0008152)	1
OG0030065	Biological Process	multicellular organismal process(GO:0032501)	1
OG0030065	Biological Process	reproduction(GO:0000003)	1
OG0030065	Biological Process	reproductive process(GO:0022414)	1
OG0030065	Biological Process	response to stimulus(GO:0050896)	1
OG0030071	Biological Process	biological regulation(GO:0065007)	1
OG0030071	Biological Process	cellular process(GO:0009987)	1
OG0030071	Biological Process	developmental process(GO:0032502)	1
OG0030071	Biological Process	metabolic process(GO:0008152)	1
OG0030071	Biological Process	multi-organism process(GO:0051704)	1
OG0030071	Biological Process	multicellular organismal process(GO:0032501)	1
OG0030071	Biological Process	regulation of biological process(GO:0050789)	1
OG0030071	Biological Process	reproduction(GO:0000003)	1
OG0030071	Biological Process	reproductive process(GO:0022414)	1
OG0030071	Biological Process	response to stimulus(GO:0050896)	1
OG0030072	Biological Process	biological regulation(GO:0065007)	1
OG0030072	Biological Process	cellular process(GO:0009987)	1
OG0030072	Biological Process	metabolic process(GO:0008152)	1
OG0030072	Biological Process	regulation of biological process(GO:0050789)	1
OG0030072	Biological Process	response to stimulus(GO:0050896)	1
OG0030072	Biological Process	signaling(GO:0023052)	1
OG0030075	Biological Process	multi-organism process(GO:0051704)	1
OG0030075	Biological Process	response to stimulus(GO:0050896)	1
OG0030077	Biological Process	metabolic process(GO:0008152)	1

OG0030077	Biological Process	multi-organism process(GO:0051704)	1
OG0030077	Biological Process	response to stimulus(GO:0050896)	1
OG0030080	Biological Process	cellular process(GO:0009987)	1
OG0030080	Biological Process	metabolic process(GO:0008152)	1
OG0030083	Biological Process	cellular process(GO:0009987)	1
OG0030083	Biological Process	metabolic process(GO:0008152)	1
OG0030087	Biological Process	biological regulation(GO:0065007)	1
OG0030087	Biological Process	cellular process(GO:0009987)	1
OG0030087	Biological Process	metabolic process(GO:0008152)	1
OG0030087	Biological Process	multi-organism process(GO:0051704)	1
OG0030087	Biological Process	negative regulation of biological process(GO:0048519)	1
OG0030087	Biological Process	regulation of biological process(GO:0050789)	1
OG0030087	Biological Process	response to stimulus(GO:0050896)	1
OG0030088	Biological Process	biological regulation(GO:0065007)	1
OG0030088	Biological Process	cellular process(GO:0009987)	1
OG0030088	Biological Process	metabolic process(GO:0008152)	1
OG0030088	Biological Process	regulation of biological process(GO:0050789)	1
OG0030088	Biological Process	response to stimulus(GO:0050896)	1
OG0030089	Biological Process	cellular process(GO:0009987)	1
OG0030089	Biological Process	developmental process(GO:0032502)	1
OG0030089	Biological Process	localization(GO:0051179)	1
OG0030089	Biological Process	multi-organism process(GO:0051704)	1
OG0030089	Biological Process	multicellular organismal process(GO:0032501)	1
OG0030089	Biological Process	reproduction(GO:0000003)	1
OG0030089	Biological Process	reproductive process(GO:0022414)	1
OG0030095	Biological Process	cellular process(GO:0009987)	1
OG0030095	Biological Process	metabolic process(GO:0008152)	1
OG0030100	Biological Process	growth(GO:0040007)	1
OG0030100	Biological Process	metabolic process(GO:0008152)	1
OG0030110	Biological Process	cellular process(GO:0009987)	1
OG0030110	Biological Process	metabolic process(GO:0008152)	1
OG0030111	Biological Process	biological regulation(GO:0065007)	1
OG0030111	Biological Process	cellular process(GO:0009987)	1
OG0030111	Biological Process	metabolic process(GO:0008152)	1
OG0030111	Biological Process	regulation of biological process(GO:0050789)	1
OG0030112	Biological Process	localization(GO:0051179)	1
OG0030112	Biological Process	response to stimulus(GO:0050896)	1
OG0030116	Biological Process	cellular process(GO:0009987)	1
OG0030116	Biological Process	metabolic process(GO:0008152)	1
OG0030135	Biological Process	cellular component organization or biogenesis(GO:0071840)	1
OG0030135	Biological Process	cellular process(GO:0009987)	1
OG0030135	Biological Process	developmental process(GO:0032502)	1
OG0030135	Biological Process	growth(GO:0040007)	1
OG0030135	Biological Process	localization(GO:0051179)	1
OG0030135	Biological Process	metabolic process(GO:0008152)	1
OG0030135	Biological Process	multicellular organismal process(GO:0032501)	1

OG0030139	Biological Process	biological regulation(GO:0065007)	1
OG0030139	Biological Process	cellular process(GO:0009987)	1
OG0030139	Biological Process	developmental process(GO:0032502)	1
OG0030139	Biological Process	metabolic process(GO:0008152)	1
OG0030139	Biological Process	multicellular organismal process(GO:0032501)	1
OG0030139	Biological Process	negative regulation of biological process(GO:0048519)	1
OG0030139	Biological Process	regulation of biological process(GO:0050789)	1
OG0030139	Biological Process	reproduction(GO:0000003)	1
OG0030139	Biological Process	reproductive process(GO:0022414)	1
OG0030139	Biological Process	response to stimulus(GO:0050896)	1
OG0030141	Biological Process	biological regulation(GO:0065007)	1
OG0030141	Biological Process	cellular process(GO:0009987)	1
OG0030141	Biological Process	metabolic process(GO:0008152)	1
OG0030141	Biological Process	negative regulation of biological process(GO:0048519)	1
OG0030141	Biological Process	regulation of biological process(GO:0050789)	1
OG0030141	Biological Process	response to stimulus(GO:0050896)	1
OG0030141	Biological Process	signaling(GO:0023052)	1
OG0030147	Biological Process	cellular process(GO:0009987)	1
OG0030147	Biological Process	metabolic process(GO:0008152)	1
OG0030150	Biological Process	biological regulation(GO:0065007)	1
OG0030150	Biological Process	cellular component organization or biogenesis(GO:0071840)	1
OG0030150	Biological Process	cellular process(GO:0009987)	1
OG0030150	Biological Process	metabolic process(GO:0008152)	1
OG0030150	Biological Process	regulation of biological process(GO:0050789)	1
OG0030155	Biological Process	cellular process(GO:0009987)	1
OG0030155	Biological Process	localization(GO:0051179)	1
OG0030155	Biological Process	metabolic process(GO:0008152)	1
OG0030160	Biological Process	response to stimulus(GO:0050896)	1
OG0030162	Biological Process	biological regulation(GO:0065007)	1
OG0030162	Biological Process	cellular component organization or biogenesis(GO:0071840)	1
OG0030162	Biological Process	cellular process(GO:0009987)	1
OG0030162	Biological Process	developmental process(GO:0032502)	1
OG0030162	Biological Process	growth(GO:0040007)	1
OG0030162	Biological Process	localization(GO:0051179)	1
OG0030162	Biological Process	metabolic process(GO:0008152)	1
OG0030162	Biological Process	multi-organism process(GO:0051704)	1
OG0030162	Biological Process	multicellular organismal process(GO:0032501)	1
OG0030162	Biological Process	negative regulation of biological process(GO:0048519)	1
OG0030162	Biological Process	regulation of biological process(GO:0050789)	1
OG0030162	Biological Process	reproduction(GO:0000003)	1
OG0030162	Biological Process	reproductive process(GO:0022414)	1
OG0030166	Biological Process	biological regulation(GO:0065007)	1
OG0030166	Biological Process	cellular process(GO:0009987)	1
OG0030166	Biological Process	developmental process(GO:0032502)	1
OG0030166	Biological Process	metabolic process(GO:0008152)	1

OG0030166	Biological Process	multi-organism process(GO:0051704)	1
OG0030166	Biological Process	multicellular organismal process(GO:0032501)	1
OG0030166	Biological Process	regulation of biological process(GO:0050789)	1
OG0030166	Biological Process	reproduction(GO:0000003)	1
OG0030166	Biological Process	reproductive process(GO:0022414)	1
OG0030166	Biological Process	response to stimulus(GO:0050896)	1
OG0030175	Biological Process	cellular process(GO:0009987)	1
OG0030175	Biological Process	metabolic process(GO:0008152)	1
OG0030180	Biological Process	biological regulation(GO:0065007)	1
OG0030180	Biological Process	cellular process(GO:0009987)	1
OG0030180	Biological Process	metabolic process(GO:0008152)	1
OG0030180	Biological Process	negative regulation of biological process(GO:0048519)	1
OG0030180	Biological Process	regulation of biological process(GO:0050789)	1
OG0030180	Biological Process	response to stimulus(GO:0050896)	1
OG0030180	Biological Process	signaling(GO:0023052)	1
OG0030182	Biological Process	cellular process(GO:0009987)	1
OG0030182	Biological Process	metabolic process(GO:0008152)	1
OG0030182	Biological Process	response to stimulus(GO:0050896)	1
OG0030184	Biological Process	cellular process(GO:0009987)	1
OG0030184	Biological Process	metabolic process(GO:0008152)	1
OG0030191	Biological Process	biological regulation(GO:0065007)	1
OG0030191	Biological Process	cellular process(GO:0009987)	1
OG0030191	Biological Process	developmental process(GO:0032502)	1
OG0030191	Biological Process	metabolic process(GO:0008152)	1
OG0030191	Biological Process	multicellular organismal process(GO:0032501)	1
OG0030191	Biological Process	regulation of biological process(GO:0050789)	1
OG0030191	Biological Process	response to stimulus(GO:0050896)	1
OG0030195	Biological Process	developmental process(GO:0032502)	1
OG0030195	Biological Process	metabolic process(GO:0008152)	1
OG0030195	Biological Process	multicellular organismal process(GO:0032501)	1
OG0030195	Biological Process	reproduction(GO:0000003)	1
OG0030195	Biological Process	reproductive process(GO:0022414)	1
OG0030195	Biological Process	response to stimulus(GO:0050896)	1
OG0030196	Biological Process	biological regulation(GO:0065007)	1
OG0030196	Biological Process	cellular process(GO:0009987)	1
OG0030196	Biological Process	metabolic process(GO:0008152)	1
OG0030196	Biological Process	negative regulation of biological process(GO:0048519)	1
OG0030196	Biological Process	regulation of biological process(GO:0050789)	1
OG0030196	Biological Process	response to stimulus(GO:0050896)	1
OG0030200	Biological Process	cellular process(GO:0009987)	1
OG0030200	Biological Process	metabolic process(GO:0008152)	1
OG0030202	Biological Process	biological regulation(GO:0065007)	1
OG0030202	Biological Process	localization(GO:0051179)	1
OG0030202	Biological Process	metabolic process(GO:0008152)	1
OG0030202	Biological Process	regulation of biological process(GO:0050789)	1

OG0030202	Biological Process	response to stimulus(GO:0050896)	1
OG0030203	Biological Process	biological regulation(GO:0065007)	1
OG0030203	Biological Process	localization(GO:0051179)	1
OG0030203	Biological Process	metabolic process(GO:0008152)	1
OG0030203	Biological Process	regulation of biological process(GO:0050789)	1
OG0030203	Biological Process	response to stimulus(GO:0050896)	1
OG0030204	Biological Process	biological regulation(GO:0065007)	1
OG0030204	Biological Process	localization(GO:0051179)	1
OG0030204	Biological Process	metabolic process(GO:0008152)	1
OG0030204	Biological Process	regulation of biological process(GO:0050789)	1
OG0030204	Biological Process	response to stimulus(GO:0050896)	1
OG0030205	Biological Process	biological regulation(GO:0065007)	1
OG0030205	Biological Process	localization(GO:0051179)	1
OG0030205	Biological Process	metabolic process(GO:0008152)	1
OG0030205	Biological Process	regulation of biological process(GO:0050789)	1
OG0030205	Biological Process	response to stimulus(GO:0050896)	1
OG0030209	Biological Process	biological regulation(GO:0065007)	1
OG0030209	Biological Process	cellular process(GO:0009987)	1
OG0030209	Biological Process	developmental process(GO:0032502)	1
OG0030209	Biological Process	metabolic process(GO:0008152)	1
OG0030209	Biological Process	multicellular organismal process(GO:0032501)	1
OG0030209	Biological Process	regulation of biological process(GO:0050789)	1
OG0030209	Biological Process	reproduction(GO:0000003)	1
OG0030209	Biological Process	reproductive process(GO:0022414)	1
OG0030213	Biological Process	cellular process(GO:0009987)	1
OG0030213	Biological Process	metabolic process(GO:0008152)	1
OG0030216	Biological Process	cellular process(GO:0009987)	1
OG0030216	Biological Process	metabolic process(GO:0008152)	1
OG0030218	Biological Process	cellular process(GO:0009987)	1
OG0030218	Biological Process	metabolic process(GO:0008152)	1
OG0030219	Biological Process	metabolic process(GO:0008152)	1
OG0030224	Biological Process	cellular process(GO:0009987)	1
OG0030224	Biological Process	metabolic process(GO:0008152)	1
OG0030225	Biological Process	cellular process(GO:0009987)	1
OG0030225	Biological Process	metabolic process(GO:0008152)	1
OG0030230	Biological Process	cellular process(GO:0009987)	1
OG0030230	Biological Process	growth(GO:0040007)	1
OG0030230	Biological Process	metabolic process(GO:0008152)	1
OG0030231	Biological Process	metabolic process(GO:0008152)	1
OG0030236	Biological Process	cellular process(GO:0009987)	1
OG0030236	Biological Process	metabolic process(GO:0008152)	1
OG0030239	Biological Process	cellular process(GO:0009987)	1
OG0030239	Biological Process	growth(GO:0040007)	1
OG0030239	Biological Process	metabolic process(GO:0008152)	1
OG0030239	Biological Process	response to stimulus(GO:0050896)	1
OG0030241	Biological Process	cellular component organization or biogenesis(GO:0071840)	1

OG0030241	Biological Process	cellular process(GO:0009987)	1
OG0030241	Biological Process	localization(GO:0051179)	1
OG0030241	Biological Process	metabolic process(GO:0008152)	1
OG0030244	Biological Process	metabolic process(GO:0008152)	1
OG0030246	Biological Process	cellular process(GO:0009987)	1
OG0030246	Biological Process	growth(GO:0040007)	1
OG0030246	Biological Process	metabolic process(GO:0008152)	1
OG0030247	Biological Process	cellular component organization or biogenesis(GO:0071840)	1
OG0030247	Biological Process	cellular process(GO:0009987)	1
OG0030247	Biological Process	metabolic process(GO:0008152)	1
OG0030248	Biological Process	cellular component organization or biogenesis(GO:0071840)	1
OG0030248	Biological Process	cellular process(GO:0009987)	1
OG0030248	Biological Process	growth(GO:0040007)	1
OG0030248	Biological Process	metabolic process(GO:0008152)	1
OG0030249	Biological Process	cellular component organization or biogenesis(GO:0071840)	1
OG0030249	Biological Process	cellular process(GO:0009987)	1
OG0030249	Biological Process	growth(GO:0040007)	1
OG0030249	Biological Process	metabolic process(GO:0008152)	1
OG0030250	Biological Process	cellular process(GO:0009987)	1
OG0030250	Biological Process	growth(GO:0040007)	1
OG0030250	Biological Process	metabolic process(GO:0008152)	1
OG0030251	Biological Process	cellular process(GO:0009987)	1
OG0030251	Biological Process	growth(GO:0040007)	1
OG0030251	Biological Process	localization(GO:0051179)	1
OG0030251	Biological Process	multi-organism process(GO:0051704)	1
OG0030251	Biological Process	response to stimulus(GO:0050896)	1
OG0030252	Biological Process	cellular process(GO:0009987)	1
OG0030252	Biological Process	growth(GO:0040007)	1
OG0030252	Biological Process	metabolic process(GO:0008152)	1
OG0030253	Biological Process	cellular process(GO:0009987)	1
OG0030253	Biological Process	growth(GO:0040007)	1
OG0030253	Biological Process	metabolic process(GO:0008152)	1
OG0030254	Biological Process	cellular process(GO:0009987)	1
OG0030254	Biological Process	growth(GO:0040007)	1
OG0030254	Biological Process	metabolic process(GO:0008152)	1
OG0030255	Biological Process	cellular process(GO:0009987)	1
OG0030255	Biological Process	metabolic process(GO:0008152)	1
OG0030257	Biological Process	cellular process(GO:0009987)	1
OG0030257	Biological Process	metabolic process(GO:0008152)	1
OG0030259	Biological Process	cellular process(GO:0009987)	1
OG0030259	Biological Process	growth(GO:0040007)	1
OG0030259	Biological Process	metabolic process(GO:0008152)	1
OG0030260	Biological Process	cellular component organization or biogenesis(GO:0071840)	1
OG0030260	Biological Process	cellular process(GO:0009987)	1
OG0030260	Biological Process	growth(GO:0040007)	1
OG0030260	Biological Process	metabolic process(GO:0008152)	1

OG0030261	Biological Process	biological regulation(GO:0065007)	1
OG0030261	Biological Process	cellular component organization or biogenesis(GO:0071840)	1
OG0030261	Biological Process	cellular process(GO:0009987)	1
OG0030261	Biological Process	growth(GO:0040007)	1
OG0030261	Biological Process	metabolic process(GO:0008152)	1
OG0030261	Biological Process	positive regulation of biological process(GO:0048518)	1
OG0030261	Biological Process	regulation of biological process(GO:0050789)	1
OG0030263	Biological Process	cellular process(GO:0009987)	1
OG0030263	Biological Process	growth(GO:0040007)	1
OG0030263	Biological Process	metabolic process(GO:0008152)	1
OG0030263	Biological Process	response to stimulus(GO:0050896)	1
OG0030266	Biological Process	cellular component organization or biogenesis(GO:0071840)	1
OG0030266	Biological Process	cellular process(GO:0009987)	1
OG0030266	Biological Process	growth(GO:0040007)	1
OG0030266	Biological Process	metabolic process(GO:0008152)	1
OG0030267	Biological Process	cellular process(GO:0009987)	1
OG0030267	Biological Process	metabolic process(GO:0008152)	1
OG0030269	Biological Process	cellular process(GO:0009987)	1
OG0030269	Biological Process	growth(GO:0040007)	1
OG0030269	Biological Process	metabolic process(GO:0008152)	1
OG0030272	Biological Process	biological regulation(GO:0065007)	1
OG0030272	Biological Process	cellular process(GO:0009987)	1
OG0030272	Biological Process	metabolic process(GO:0008152)	1
OG0030272	Biological Process	negative regulation of biological process(GO:0048519)	1
OG0030272	Biological Process	regulation of biological process(GO:0050789)	1
OG0030273	Biological Process	cellular process(GO:0009987)	1
OG0030273	Biological Process	growth(GO:0040007)	1
OG0030273	Biological Process	metabolic process(GO:0008152)	1
OG0030275	Biological Process	cellular process(GO:0009987)	1
OG0030275	Biological Process	growth(GO:0040007)	1
OG0030275	Biological Process	metabolic process(GO:0008152)	1
OG0030278	Biological Process	biological regulation(GO:0065007)	1
OG0030278	Biological Process	cellular process(GO:0009987)	1
OG0030278	Biological Process	metabolic process(GO:0008152)	1
OG0030278	Biological Process	negative regulation of biological process(GO:0048519)	1
OG0030278	Biological Process	regulation of biological process(GO:0050789)	1
OG0030280	Biological Process	biological regulation(GO:0065007)	1
OG0030280	Biological Process	cellular component organization or biogenesis(GO:0071840)	1
OG0030280	Biological Process	cellular process(GO:0009987)	1
OG0030280	Biological Process	metabolic process(GO:0008152)	1
OG0030280	Biological Process	regulation of biological process(GO:0050789)	1
OG0030281	Biological Process	cellular process(GO:0009987)	1
OG0030281	Biological Process	metabolic process(GO:0008152)	1
OG0030282	Biological Process	biological regulation(GO:0065007)	1
OG0030282	Biological Process	cellular component organization or biogenesis(GO:0071840)	1

OG0030282	Biological Process	cellular process(GO:0009987)	1
OG0030282	Biological Process	metabolic process(GO:0008152)	1
OG0030282	Biological Process	regulation of biological process(GO:0050789)	1
OG0030282	Biological Process	response to stimulus(GO:0050896)	1
OG0030283	Biological Process	localization(GO:0051179)	1
OG0030286	Biological Process	growth(GO:0040007)	1
OG0030287	Biological Process	biological regulation(GO:0065007)	1
OG0030287	Biological Process	cellular component organization or biogenesis(GO:0071840)	1
OG0030287	Biological Process	cellular process(GO:0009987)	1
OG0030287	Biological Process	growth(GO:0040007)	1
OG0030287	Biological Process	metabolic process(GO:0008152)	1
OG0030287	Biological Process	positive regulation of biological process(GO:0048518)	1
OG0030287	Biological Process	regulation of biological process(GO:0050789)	1
OG0030289	Biological Process	cellular process(GO:0009987)	1
OG0030289	Biological Process	localization(GO:0051179)	1
OG0030289	Biological Process	metabolic process(GO:0008152)	1
OG0030289	Biological Process	response to stimulus(GO:0050896)	1
OG0030291	Biological Process	cellular component organization or biogenesis(GO:0071840)	1
OG0030291	Biological Process	cellular process(GO:0009987)	1
OG0030291	Biological Process	metabolic process(GO:0008152)	1
OG0030291	Biological Process	response to stimulus(GO:0050896)	1
OG0030293	Biological Process	cellular component organization or biogenesis(GO:0071840)	1
OG0030293	Biological Process	cellular process(GO:0009987)	1
OG0030293	Biological Process	localization(GO:0051179)	1
OG0030293	Biological Process	locomotion(GO:0040011)	1
OG0030298	Biological Process	cellular component organization or biogenesis(GO:0071840)	1
OG0030298	Biological Process	cellular process(GO:0009987)	1
OG0030298	Biological Process	growth(GO:0040007)	1
OG0030298	Biological Process	metabolic process(GO:0008152)	1
OG0030300	Biological Process	cellular process(GO:0009987)	1
OG0030300	Biological Process	growth(GO:0040007)	1
OG0030300	Biological Process	metabolic process(GO:0008152)	1
OG0030301	Biological Process	cellular process(GO:0009987)	1
OG0030301	Biological Process	metabolic process(GO:0008152)	1
OG0030302	Biological Process	cellular process(GO:0009987)	1
OG0030302	Biological Process	metabolic process(GO:0008152)	1
OG0030303	Biological Process	growth(GO:0040007)	1
OG0030305	Biological Process	cellular process(GO:0009987)	1
OG0030305	Biological Process	metabolic process(GO:0008152)	1
OG0030309	Biological Process	cellular component organization or biogenesis(GO:0071840)	1
OG0030309	Biological Process	cellular process(GO:0009987)	1
OG0030309	Biological Process	metabolic process(GO:0008152)	1
OG0030310	Biological Process	cellular component organization or biogenesis(GO:0071840)	1
OG0030310	Biological Process	cellular process(GO:0009987)	1
OG0030310	Biological Process	growth(GO:0040007)	1

OG0030310	Biological Process	metabolic process(GO:0008152)	1
OG0030314	Biological Process	cellular process(GO:0009987)	1
OG0030314	Biological Process	metabolic process(GO:0008152)	1
OG0030315	Biological Process	cellular process(GO:0009987)	1
OG0030315	Biological Process	metabolic process(GO:0008152)	1
OG0030317	Biological Process	cellular process(GO:0009987)	1
OG0030317	Biological Process	metabolic process(GO:0008152)	1
OG0030318	Biological Process	cellular process(GO:0009987)	1
OG0030318	Biological Process	metabolic process(GO:0008152)	1
OG0030320	Biological Process	growth(GO:0040007)	1
OG0030320	Biological Process	metabolic process(GO:0008152)	1
OG0030320	Biological Process	multi-organism process(GO:0051704)	1
OG0030320	Biological Process	response to stimulus(GO:0050896)	1
OG0030322	Biological Process	cellular process(GO:0009987)	1
OG0030322	Biological Process	growth(GO:0040007)	1
OG0030322	Biological Process	metabolic process(GO:0008152)	1
OG0030326	Biological Process	cellular process(GO:0009987)	1
OG0030326	Biological Process	metabolic process(GO:0008152)	1
OG0030327	Biological Process	cellular process(GO:0009987)	1
OG0030327	Biological Process	metabolic process(GO:0008152)	1
OG0030327	Biological Process	response to stimulus(GO:0050896)	1
OG0030330	Biological Process	cellular process(GO:0009987)	1
OG0030330	Biological Process	growth(GO:0040007)	1
OG0030330	Biological Process	metabolic process(GO:0008152)	1
OG0030336	Biological Process	cellular component organization or biogenesis(GO:0071840)	1
OG0030336	Biological Process	cellular process(GO:0009987)	1
OG0030336	Biological Process	growth(GO:0040007)	1
OG0030336	Biological Process	immune system process(GO:0002376)	1
OG0030336	Biological Process	metabolic process(GO:0008152)	1
OG0030336	Biological Process	multi-organism process(GO:0051704)	1
OG0030336	Biological Process	response to stimulus(GO:0050896)	1
OG0030348	Biological Process	developmental process(GO:0032502)	1
OG0030348	Biological Process	metabolic process(GO:0008152)	1
OG0030348	Biological Process	multicellular organismal process(GO:0032501)	1
OG0030348	Biological Process	reproduction(GO:0000003)	1
OG0030348	Biological Process	reproductive process(GO:0022414)	1
OG0030348	Biological Process	response to stimulus(GO:0050896)	1
OG0030350	Biological Process	developmental process(GO:0032502)	1
OG0030350	Biological Process	multicellular organismal process(GO:0032501)	1
OG0030350	Biological Process	reproduction(GO:0000003)	1
OG0030350	Biological Process	reproductive process(GO:0022414)	1
OG0030355	Biological Process	cellular process(GO:0009987)	1
OG0030355	Biological Process	localization(GO:0051179)	1
OG0030361	Biological Process	biological regulation(GO:0065007)	1
OG0030361	Biological Process	cellular component organization or biogenesis(GO:0071840)	1
OG0030361	Biological Process	cellular process(GO:0009987)	1

OG0030361	Biological Process	developmental process(GO:0032502)	1
OG0030361	Biological Process	metabolic process(GO:0008152)	1
OG0030361	Biological Process	multi-organism process(GO:0051704)	1
OG0030361	Biological Process	multicellular organismal process(GO:0032501)	1
OG0030361	Biological Process	regulation of biological process(GO:0050789)	1
OG0030361	Biological Process	reproduction(GO:0000003)	1
OG0030361	Biological Process	reproductive process(GO:0022414)	1
OG0030361	Biological Process	response to stimulus(GO:0050896)	1
OG0030361	Biological Process	signaling(GO:0023052)	1
OG0030367	Biological Process	cellular process(GO:0009987)	1
OG0030367	Biological Process	localization(GO:0051179)	1
OG0030371	Biological Process	cellular process(GO:0009987)	1
OG0030371	Biological Process	metabolic process(GO:0008152)	1
OG0030376	Biological Process	biological regulation(GO:0065007)	1
OG0030376	Biological Process	cellular process(GO:0009987)	1
OG0030376	Biological Process	developmental process(GO:0032502)	1
OG0030376	Biological Process	metabolic process(GO:0008152)	1
OG0030376	Biological Process	multicellular organismal process(GO:0032501)	1
OG0030376	Biological Process	regulation of biological process(GO:0050789)	1
OG0030376	Biological Process	reproduction(GO:0000003)	1
OG0030376	Biological Process	reproductive process(GO:0022414)	1
OG0030376	Biological Process	response to stimulus(GO:0050896)	1
OG0030384	Biological Process	response to stimulus(GO:0050896)	1
OG0030388	Biological Process	biological regulation(GO:0065007)	1
OG0030388	Biological Process	cellular process(GO:0009987)	1
OG0030388	Biological Process	metabolic process(GO:0008152)	1
OG0030388	Biological Process	regulation of biological process(GO:0050789)	1
OG0030389	Biological Process	biological regulation(GO:0065007)	1
OG0030389	Biological Process	cellular process(GO:0009987)	1
OG0030389	Biological Process	metabolic process(GO:0008152)	1
OG0030389	Biological Process	regulation of biological process(GO:0050789)	1
OG0030412	Biological Process	cellular process(GO:0009987)	1
OG0030412	Biological Process	metabolic process(GO:0008152)	1
OG0030412	Biological Process	response to stimulus(GO:0050896)	1
OG0030423	Biological Process	metabolic process(GO:0008152)	1
OG0030435	Biological Process	cellular process(GO:0009987)	1
OG0030435	Biological Process	developmental process(GO:0032502)	1
OG0030435	Biological Process	growth(GO:0040007)	1
OG0030435	Biological Process	metabolic process(GO:0008152)	1
OG0030435	Biological Process	multicellular organismal process(GO:0032501)	1
OG0030435	Biological Process	reproduction(GO:0000003)	1
OG0030435	Biological Process	reproductive process(GO:0022414)	1
OG0030435	Biological Process	response to stimulus(GO:0050896)	1
OG0030436	Biological Process	cellular process(GO:0009987)	1
OG0030436	Biological Process	developmental process(GO:0032502)	1
OG0030436	Biological Process	growth(GO:0040007)	1

OG0030436	Biological Process	metabolic process(GO:0008152)	1
OG0030436	Biological Process	multicellular organismal process(GO:0032501)	1
OG0030436	Biological Process	reproduction(GO:0000003)	1
OG0030436	Biological Process	reproductive process(GO:0022414)	1
OG0030436	Biological Process	response to stimulus(GO:0050896)	1
OG0030441	Biological Process	cellular component organization or biogenesis(GO:0071840)	1
OG0030441	Biological Process	cellular process(GO:0009987)	1
OG0030441	Biological Process	metabolic process(GO:0008152)	1
OG0030446	Biological Process	cellular process(GO:0009987)	1
OG0030446	Biological Process	developmental process(GO:0032502)	1
OG0030446	Biological Process	metabolic process(GO:0008152)	1
OG0030446	Biological Process	multicellular organismal process(GO:0032501)	1
OG0030446	Biological Process	reproduction(GO:0000003)	1
OG0030446	Biological Process	reproductive process(GO:0022414)	1
OG0030450	Biological Process	response to stimulus(GO:0050896)	1
OG0030458	Biological Process	metabolic process(GO:0008152)	1
OG0030490	Biological Process	developmental process(GO:0032502)	1
OG0030490	Biological Process	multi-organism process(GO:0051704)	1
OG0030490	Biological Process	multicellular organismal process(GO:0032501)	1
OG0030490	Biological Process	reproduction(GO:0000003)	1
OG0030490	Biological Process	reproductive process(GO:0022414)	1
OG0030496	Biological Process	response to stimulus(GO:0050896)	1
OG0030524	Biological Process	biological regulation(GO:0065007)	1
OG0030524	Biological Process	cellular component organization or biogenesis(GO:0071840)	1
OG0030524	Biological Process	cellular process(GO:0009987)	1
OG0030524	Biological Process	developmental process(GO:0032502)	1
OG0030524	Biological Process	metabolic process(GO:0008152)	1
OG0030524	Biological Process	multicellular organismal process(GO:0032501)	1
OG0030524	Biological Process	regulation of biological process(GO:0050789)	1
OG0030524	Biological Process	reproduction(GO:0000003)	1
OG0030524	Biological Process	reproductive process(GO:0022414)	1
OG0030542	Biological Process	cellular process(GO:0009987)	1
OG0030542	Biological Process	metabolic process(GO:0008152)	1
OG0030551	Biological Process	biological regulation(GO:0065007)	1
OG0030551	Biological Process	cellular process(GO:0009987)	1
OG0030551	Biological Process	developmental process(GO:0032502)	1
OG0030551	Biological Process	immune system process(GO:0002376)	1
OG0030551	Biological Process	metabolic process(GO:0008152)	1
OG0030551	Biological Process	multi-organism process(GO:0051704)	1
OG0030551	Biological Process	multicellular organismal process(GO:0032501)	1
OG0030551	Biological Process	regulation of biological process(GO:0050789)	1
OG0030551	Biological Process	response to stimulus(GO:0050896)	1
OG0030551	Biological Process	signaling(GO:0023052)	1
OG0030552	Biological Process	biological regulation(GO:0065007)	1
OG0030552	Biological Process	cellular process(GO:0009987)	1

OG0030552	Biological Process	metabolic process(GO:0008152)	1
OG0030552	Biological Process	regulation of biological process(GO:0050789)	1
OG0030552	Biological Process	response to stimulus(GO:0050896)	1
OG0030555	Biological Process	cellular process(GO:0009987)	1
OG0030555	Biological Process	developmental process(GO:0032502)	1
OG0030555	Biological Process	metabolic process(GO:0008152)	1
OG0030555	Biological Process	multicellular organismal process(GO:0032501)	1
OG0030555	Biological Process	reproduction(GO:0000003)	1
OG0030555	Biological Process	reproductive process(GO:0022414)	1
OG0030568	Biological Process	response to stimulus(GO:0050896)	1
OG0030578	Biological Process	cellular process(GO:0009987)	1
OG0030578	Biological Process	localization(GO:0051179)	1
OG0030584	Biological Process	biological regulation(GO:0065007)	1
OG0030584	Biological Process	cellular process(GO:0009987)	1
OG0030584	Biological Process	developmental process(GO:0032502)	1
OG0030584	Biological Process	localization(GO:0051179)	1
OG0030584	Biological Process	metabolic process(GO:0008152)	1
OG0030584	Biological Process	multicellular organismal process(GO:0032501)	1
OG0030584	Biological Process	regulation of biological process(GO:0050789)	1
OG0030584	Biological Process	response to stimulus(GO:0050896)	1
OG0030585	Biological Process	biological regulation(GO:0065007)	1
OG0030585	Biological Process	cellular component organization or biogenesis(GO:0071840)	1
OG0030585	Biological Process	cellular process(GO:0009987)	1
OG0030585	Biological Process	regulation of biological process(GO:0050789)	1
OG0030585	Biological Process	response to stimulus(GO:0050896)	1
OG0030586	Biological Process	biological regulation(GO:0065007)	1
OG0030586	Biological Process	cellular component organization or biogenesis(GO:0071840)	1
OG0030586	Biological Process	cellular process(GO:0009987)	1
OG0030586	Biological Process	metabolic process(GO:0008152)	1
OG0030586	Biological Process	negative regulation of biological process(GO:0048519)	1
OG0030586	Biological Process	regulation of biological process(GO:0050789)	1
OG0030587	Biological Process	biological regulation(GO:0065007)	1
OG0030587	Biological Process	cell proliferation(GO:0008283)	1
OG0030587	Biological Process	cellular process(GO:0009987)	1
OG0030587	Biological Process	developmental process(GO:0032502)	1
OG0030587	Biological Process	metabolic process(GO:0008152)	1
OG0030587	Biological Process	multicellular organismal process(GO:0032501)	1
OG0030587	Biological Process	negative regulation of biological process(GO:0048519)	1
OG0030587	Biological Process	positive regulation of biological process(GO:0048518)	1
OG0030587	Biological Process	regulation of biological process(GO:0050789)	1
OG0030587	Biological Process	reproduction(GO:0000003)	1
OG0030587	Biological Process	reproductive process(GO:0022414)	1
OG0030587	Biological Process	response to stimulus(GO:0050896)	1
OG0030590	Biological Process	metabolic process(GO:0008152)	1

OG0030593	Biological Process	metabolic process(GO:0008152)	1
OG0030594	Biological Process	metabolic process(GO:0008152)	1
OG0030596	Biological Process	biological regulation(GO:0065007)	1
OG0030596	Biological Process	cellular component organization or biogenesis(GO:0071840)	1
OG0030596	Biological Process	cellular process(GO:0009987)	1
OG0030596	Biological Process	developmental process(GO:0032502)	1
OG0030596	Biological Process	growth(GO:0040007)	1
OG0030596	Biological Process	immune system process(GO:0002376)	1
OG0030596	Biological Process	localization(GO:0051179)	1
OG0030596	Biological Process	metabolic process(GO:0008152)	1
OG0030596	Biological Process	multi-organism process(GO:0051704)	1
OG0030596	Biological Process	multicellular organismal process(GO:0032501)	1
OG0030596	Biological Process	negative regulation of biological process(GO:0048519)	1
OG0030596	Biological Process	regulation of biological process(GO:0050789)	1
OG0030596	Biological Process	response to stimulus(GO:0050896)	1
OG0030596	Biological Process	signaling(GO:0023052)	1
OG0030604	Biological Process	response to stimulus(GO:0050896)	1
OG0030605	Biological Process	cellular component organization or biogenesis(GO:0071840)	1
OG0030605	Biological Process	cellular process(GO:0009987)	1
OG0030605	Biological Process	localization(GO:0051179)	1
OG0030607	Biological Process	response to stimulus(GO:0050896)	1
OG0030608	Biological Process	response to stimulus(GO:0050896)	1
OG0030609	Biological Process	cellular process(GO:0009987)	1
OG0030609	Biological Process	developmental process(GO:0032502)	1
OG0030609	Biological Process	multi-organism process(GO:0051704)	1
OG0030609	Biological Process	multicellular organismal process(GO:0032501)	1
OG0030609	Biological Process	reproduction(GO:0000003)	1
OG0030609	Biological Process	reproductive process(GO:0022414)	1
OG0030614	Biological Process	response to stimulus(GO:0050896)	1
OG0030620	Biological Process	biological regulation(GO:0065007)	1
OG0030620	Biological Process	cellular process(GO:0009987)	1
OG0030620	Biological Process	developmental process(GO:0032502)	1
OG0030620	Biological Process	localization(GO:0051179)	1
OG0030620	Biological Process	metabolic process(GO:0008152)	1
OG0030620	Biological Process	multi-organism process(GO:0051704)	1
OG0030620	Biological Process	positive regulation of biological process(GO:0048518)	1
OG0030620	Biological Process	regulation of biological process(GO:0050789)	1
OG0030620	Biological Process	reproduction(GO:0000003)	1
OG0030620	Biological Process	reproductive process(GO:0022414)	1
OG0030621	Biological Process	biological regulation(GO:0065007)	1
OG0030621	Biological Process	cellular process(GO:0009987)	1
OG0030621	Biological Process	immune system process(GO:0002376)	1
OG0030621	Biological Process	metabolic process(GO:0008152)	1
OG0030621	Biological Process	multi-organism process(GO:0051704)	1
OG0030621	Biological Process	regulation of biological process(GO:0050789)	1

OG0030621	Biological Process	response to stimulus(GO:0050896)	1
OG0030621	Biological Process	signaling(GO:0023052)	1
OG0030623	Biological Process	biological regulation(GO:0065007)	1
OG0030623	Biological Process	cellular process(GO:0009987)	1
OG0030623	Biological Process	developmental process(GO:0032502)	1
OG0030623	Biological Process	localization(GO:0051179)	1
OG0030623	Biological Process	locomotion(GO:0040011)	1
OG0030623	Biological Process	metabolic process(GO:0008152)	1
OG0030623	Biological Process	multicellular organismal process(GO:0032501)	1
OG0030623	Biological Process	regulation of biological process(GO:0050789)	1
OG0030623	Biological Process	response to stimulus(GO:0050896)	1
OG0030623	Biological Process	signaling(GO:0023052)	1
OG0030624	Biological Process	biological regulation(GO:0065007)	1
OG0030624	Biological Process	cellular process(GO:0009987)	1
OG0030624	Biological Process	metabolic process(GO:0008152)	1
OG0030624	Biological Process	regulation of biological process(GO:0050789)	1
OG0030625	Biological Process	cellular process(GO:0009987)	1
OG0030625	Biological Process	metabolic process(GO:0008152)	1
OG0030629	Biological Process	biological regulation(GO:0065007)	1
OG0030629	Biological Process	multi-organism process(GO:0051704)	1
OG0030629	Biological Process	response to stimulus(GO:0050896)	1
OG0030630	Biological Process	biological regulation(GO:0065007)	1
OG0030630	Biological Process	cellular process(GO:0009987)	1
OG0030630	Biological Process	metabolic process(GO:0008152)	1
OG0030630	Biological Process	negative regulation of biological process(GO:0048519)	1
OG0030630	Biological Process	regulation of biological process(GO:0050789)	1
OG0030630	Biological Process	response to stimulus(GO:0050896)	1
OG0030630	Biological Process	signaling(GO:0023052)	1
OG0030642	Biological Process	biological regulation(GO:0065007)	1
OG0030642	Biological Process	cellular process(GO:0009987)	1
OG0030642	Biological Process	developmental process(GO:0032502)	1
OG0030642	Biological Process	metabolic process(GO:0008152)	1
OG0030642	Biological Process	multicellular organismal process(GO:0032501)	1
OG0030642	Biological Process	negative regulation of biological process(GO:0048519)	1
OG0030642	Biological Process	regulation of biological process(GO:0050789)	1
OG0030642	Biological Process	reproduction(GO:0000003)	1
OG0030642	Biological Process	reproductive process(GO:0022414)	1
OG0030642	Biological Process	response to stimulus(GO:0050896)	1
OG0030643	Biological Process	biological regulation(GO:0065007)	1
OG0030643	Biological Process	cellular process(GO:0009987)	1
OG0030643	Biological Process	metabolic process(GO:0008152)	1
OG0030643	Biological Process	regulation of biological process(GO:0050789)	1
OG0030656	Biological Process	developmental process(GO:0032502)	1
OG0030656	Biological Process	multicellular organismal process(GO:0032501)	1
OG0030658	Biological Process	biological regulation(GO:0065007)	1

OG0030658	Biological Process	cellular process(GO:0009987)	1
OG0030658	Biological Process	metabolic process(GO:0008152)	1
OG0030658	Biological Process	regulation of biological process(GO:0050789)	1
OG0030658	Biological Process	response to stimulus(GO:0050896)	1
OG0030660	Biological Process	cellular process(GO:0009987)	1
OG0030660	Biological Process	metabolic process(GO:0008152)	1
OG0030660	Biological Process	reproduction(GO:0000003)	1
OG0030660	Biological Process	reproductive process(GO:0022414)	1
OG0030660	Biological Process	response to stimulus(GO:0050896)	1
OG0030661	Biological Process	cellular process(GO:0009987)	1
OG0030661	Biological Process	metabolic process(GO:0008152)	1
OG0030661	Biological Process	reproduction(GO:0000003)	1
OG0030661	Biological Process	reproductive process(GO:0022414)	1
OG0030661	Biological Process	response to stimulus(GO:0050896)	1
OG0030666	Biological Process	response to stimulus(GO:0050896)	1
OG0030667	Biological Process	response to stimulus(GO:0050896)	1
OG0030669	Biological Process	biological regulation(GO:0065007)	1
OG0030669	Biological Process	cellular process(GO:0009987)	1
OG0030669	Biological Process	developmental process(GO:0032502)	1
OG0030669	Biological Process	metabolic process(GO:0008152)	1
OG0030669	Biological Process	multicellular organismal process(GO:0032501)	1
OG0030669	Biological Process	positive regulation of biological process(GO:0048518)	1
OG0030669	Biological Process	regulation of biological process(GO:0050789)	1
OG0030669	Biological Process	reproduction(GO:0000003)	1
OG0030669	Biological Process	reproductive process(GO:0022414)	1
OG0030670	Biological Process	biological regulation(GO:0065007)	1
OG0030670	Biological Process	cellular process(GO:0009987)	1
OG0030670	Biological Process	metabolic process(GO:0008152)	1
OG0030670	Biological Process	regulation of biological process(GO:0050789)	1
OG0030682	Biological Process	cellular process(GO:0009987)	1
OG0030682	Biological Process	metabolic process(GO:0008152)	1
OG0030691	Biological Process	response to stimulus(GO:0050896)	1
OG0030700	Biological Process	metabolic process(GO:0008152)	1
OG0030700	Biological Process	multi-organism process(GO:0051704)	1
OG0030700	Biological Process	response to stimulus(GO:0050896)	1
OG0030702	Biological Process	cellular process(GO:0009987)	1
OG0030702	Biological Process	metabolic process(GO:0008152)	1
OG0030702	Biological Process	response to stimulus(GO:0050896)	1
OG0030704	Biological Process	biological regulation(GO:0065007)	1
OG0030704	Biological Process	cellular component organization or biogenesis(GO:0071840)	1
OG0030704	Biological Process	cellular process(GO:0009987)	1
OG0030704	Biological Process	developmental process(GO:0032502)	1
OG0030704	Biological Process	metabolic process(GO:0008152)	1
OG0030704	Biological Process	multicellular organismal process(GO:0032501)	1
OG0030704	Biological Process	regulation of biological process(GO:0050789)	1

OG0030704	Biological Process	response to stimulus(GO:0050896)	1
OG0030706	Biological Process	cellular process(GO:0009987)	1
OG0030706	Biological Process	developmental process(GO:0032502)	1
OG0030706	Biological Process	metabolic process(GO:0008152)	1
OG0030706	Biological Process	multicellular organismal process(GO:0032501)	1
OG0030706	Biological Process	reproduction(GO:0000003)	1
OG0030706	Biological Process	reproductive process(GO:0022414)	1
OG0030706	Biological Process	response to stimulus(GO:0050896)	1
OG0030708	Biological Process	cellular process(GO:0009987)	1
OG0030708	Biological Process	developmental process(GO:0032502)	1
OG0030708	Biological Process	metabolic process(GO:0008152)	1
OG0030708	Biological Process	multicellular organismal process(GO:0032501)	1
OG0030708	Biological Process	reproduction(GO:0000003)	1
OG0030708	Biological Process	reproductive process(GO:0022414)	1
OG0030708	Biological Process	response to stimulus(GO:0050896)	1
OG0030709	Biological Process	biological regulation(GO:0065007)	1
OG0030709	Biological Process	cellular process(GO:0009987)	1
OG0030709	Biological Process	developmental process(GO:0032502)	1
OG0030709	Biological Process	localization(GO:0051179)	1
OG0030709	Biological Process	metabolic process(GO:0008152)	1
OG0030709	Biological Process	multicellular organismal process(GO:0032501)	1
OG0030709	Biological Process	regulation of biological process(GO:0050789)	1
OG0030710	Biological Process	biological regulation(GO:0065007)	1
OG0030710	Biological Process	cellular process(GO:0009987)	1
OG0030710	Biological Process	developmental process(GO:0032502)	1
OG0030710	Biological Process	metabolic process(GO:0008152)	1
OG0030710	Biological Process	multicellular organismal process(GO:0032501)	1
OG0030710	Biological Process	negative regulation of biological process(GO:0048519)	1
OG0030710	Biological Process	positive regulation of biological process(GO:0048518)	1
OG0030710	Biological Process	regulation of biological process(GO:0050789)	1
OG0030710	Biological Process	reproduction(GO:0000003)	1
OG0030710	Biological Process	reproductive process(GO:0022414)	1
OG0030710	Biological Process	response to stimulus(GO:0050896)	1
OG0030715	Biological Process	biological regulation(GO:0065007)	1
OG0030715	Biological Process	cellular process(GO:0009987)	1
OG0030715	Biological Process	metabolic process(GO:0008152)	1
OG0030715	Biological Process	regulation of biological process(GO:0050789)	1
OG0030730	Biological Process	localization(GO:0051179)	1
OG0030730	Biological Process	response to stimulus(GO:0050896)	1
OG0030732	Biological Process	developmental process(GO:0032502)	1
OG0030732	Biological Process	metabolic process(GO:0008152)	1
OG0030743	Biological Process	cellular component organization or biogenesis(GO:0071840)	1
OG0030743	Biological Process	cellular process(GO:0009987)	1
OG0030743	Biological Process	growth(GO:0040007)	1
OG0030743	Biological Process	response to stimulus(GO:0050896)	1

OG0030746	Biological Process	cellular process(GO:0009987)	1
OG0030746	Biological Process	metabolic process(GO:0008152)	1
OG0030753	Biological Process	cellular process(GO:0009987)	1
OG0030753	Biological Process	metabolic process(GO:0008152)	1
OG0030757	Biological Process	cellular component organization or biogenesis(GO:0071840)	1
OG0030757	Biological Process	cellular process(GO:0009987)	1
OG0030757	Biological Process	metabolic process(GO:0008152)	1
OG0030763	Biological Process	cellular process(GO:0009987)	1
OG0030763	Biological Process	metabolic process(GO:0008152)	1
OG0030763	Biological Process	multi-organism process(GO:0051704)	1
OG0030763	Biological Process	response to stimulus(GO:0050896)	1
OG0030766	Biological Process	biological regulation(GO:0065007)	1
OG0030766	Biological Process	cellular process(GO:0009987)	1
OG0030766	Biological Process	regulation of biological process(GO:0050789)	1
OG0030766	Biological Process	response to stimulus(GO:0050896)	1
OG0030767	Biological Process	biological regulation(GO:0065007)	1
OG0030767	Biological Process	cellular process(GO:0009987)	1
OG0030767	Biological Process	metabolic process(GO:0008152)	1
OG0030767	Biological Process	positive regulation of biological process(GO:0048518)	1
OG0030767	Biological Process	regulation of biological process(GO:0050789)	1
OG0030773	Biological Process	developmental process(GO:0032502)	1
OG0030776	Biological Process	biological regulation(GO:0065007)	1
OG0030776	Biological Process	cellular process(GO:0009987)	1
OG0030776	Biological Process	developmental process(GO:0032502)	1
OG0030776	Biological Process	localization(GO:0051179)	1
OG0030776	Biological Process	multi-organism process(GO:0051704)	1
OG0030776	Biological Process	multicellular organismal process(GO:0032501)	1
OG0030776	Biological Process	regulation of biological process(GO:0050789)	1
OG0030776	Biological Process	response to stimulus(GO:0050896)	1
OG0030778	Biological Process	cellular process(GO:0009987)	1
OG0030778	Biological Process	metabolic process(GO:0008152)	1
OG0030791	Biological Process	cellular component organization or biogenesis(GO:0071840)	1
OG0030791	Biological Process	cellular process(GO:0009987)	1
OG0030791	Biological Process	metabolic process(GO:0008152)	1
OG0030791	Biological Process	response to stimulus(GO:0050896)	1
OG0030794	Biological Process	response to stimulus(GO:0050896)	1
OG0030796	Biological Process	cellular component organization or biogenesis(GO:0071840)	1
OG0030796	Biological Process	cellular process(GO:0009987)	1
OG0030796	Biological Process	metabolic process(GO:0008152)	1
OG0030796	Biological Process	response to stimulus(GO:0050896)	1
OG0030814	Biological Process	biological regulation(GO:0065007)	1
OG0030814	Biological Process	cellular process(GO:0009987)	1
OG0030814	Biological Process	developmental process(GO:0032502)	1
OG0030814	Biological Process	metabolic process(GO:0008152)	1
OG0030814	Biological Process	multicellular organismal process(GO:0032501)	1

OG0030814	Biological Process	regulation of biological process(GO:0050789)	1
OG0030814	Biological Process	reproduction(GO:0000003)	1
OG0030814	Biological Process	reproductive process(GO:0022414)	1
OG0030815	Biological Process	biological regulation(GO:0065007)	1
OG0030815	Biological Process	cellular process(GO:0009987)	1
OG0030815	Biological Process	metabolic process(GO:0008152)	1
OG0030815	Biological Process	regulation of biological process(GO:0050789)	1
OG0030815	Biological Process	response to stimulus(GO:0050896)	1
OG0030816	Biological Process	biological regulation(GO:0065007)	1
OG0030816	Biological Process	cellular process(GO:0009987)	1
OG0030816	Biological Process	metabolic process(GO:0008152)	1
OG0030816	Biological Process	regulation of biological process(GO:0050789)	1
OG0030818	Biological Process	cellular process(GO:0009987)	1
OG0030818	Biological Process	localization(GO:0051179)	1
OG0030818	Biological Process	metabolic process(GO:0008152)	1
OG0030820	Biological Process	cellular component organization or biogenesis(GO:0071840)	1
OG0030820	Biological Process	cellular process(GO:0009987)	1
OG0030820	Biological Process	growth(GO:0040007)	1
OG0030820	Biological Process	metabolic process(GO:0008152)	1
OG0030852	Biological Process	biological adhesion(GO:0022610)	1
OG0030852	Biological Process	biological regulation(GO:0065007)	1
OG0030852	Biological Process	cellular component organization or biogenesis(GO:0071840)	1
OG0030852	Biological Process	cellular process(GO:0009987)	1
OG0030852	Biological Process	developmental process(GO:0032502)	1
OG0030852	Biological Process	localization(GO:0051179)	1
OG0030852	Biological Process	locomotion(GO:0040011)	1
OG0030852	Biological Process	metabolic process(GO:0008152)	1
OG0030852	Biological Process	multicellular organismal process(GO:0032501)	1
OG0030852	Biological Process	regulation of biological process(GO:0050789)	1
OG0030852	Biological Process	response to stimulus(GO:0050896)	1
OG0030853	Biological Process	cellular process(GO:0009987)	1
OG0030853	Biological Process	metabolic process(GO:0008152)	1
OG0030856	Biological Process	cellular process(GO:0009987)	1
OG0030856	Biological Process	metabolic process(GO:0008152)	1
OG0030861	Biological Process	cellular process(GO:0009987)	1
OG0030861	Biological Process	growth(GO:0040007)	1
OG0030861	Biological Process	metabolic process(GO:0008152)	1
OG0030864	Biological Process	cellular process(GO:0009987)	1
OG0030864	Biological Process	metabolic process(GO:0008152)	1
OG0030868	Biological Process	biological regulation(GO:0065007)	1
OG0030868	Biological Process	cell killing(GO:0001906)	1
OG0030868	Biological Process	cellular component organization or biogenesis(GO:0071840)	1
OG0030868	Biological Process	cellular process(GO:0009987)	1
OG0030868	Biological Process	metabolic process(GO:0008152)	1
OG0030868	Biological Process	multi-organism process(GO:0051704)	1

OG0030869	Biological Process	cellular component organization or biogenesis(GO:0071840)	1
OG0030869	Biological Process	cellular process(GO:0009987)	1
OG0030869	Biological Process	growth(GO:0040007)	1
OG0030869	Biological Process	metabolic process(GO:0008152)	1
OG0030870	Biological Process	growth(GO:0040007)	1
OG0030879	Biological Process	biological adhesion(GO:0022610)	1
OG0030879	Biological Process	cellular component organization or biogenesis(GO:0071840)	1
OG0030879	Biological Process	cellular process(GO:0009987)	1
OG0030879	Biological Process	growth(GO:0040007)	1
OG0030879	Biological Process	metabolic process(GO:0008152)	1
OG0030879	Biological Process	multi-organism process(GO:0051704)	1
OG0030879	Biological Process	reproduction(GO:0000003)	1
OG0030879	Biological Process	response to stimulus(GO:0050896)	1
OG0030882	Biological Process	cellular process(GO:0009987)	1
OG0030882	Biological Process	growth(GO:0040007)	1
OG0030882	Biological Process	metabolic process(GO:0008152)	1
OG0030884	Biological Process	cellular process(GO:0009987)	1
OG0030884	Biological Process	growth(GO:0040007)	1
OG0030884	Biological Process	metabolic process(GO:0008152)	1
OG0030885	Biological Process	growth(GO:0040007)	1
OG0030887	Biological Process	cellular process(GO:0009987)	1
OG0030887	Biological Process	growth(GO:0040007)	1
OG0030887	Biological Process	metabolic process(GO:0008152)	1
OG0030893	Biological Process	growth(GO:0040007)	1
OG0030895	Biological Process	cellular process(GO:0009987)	1
OG0030895	Biological Process	metabolic process(GO:0008152)	1
OG0030896	Biological Process	biological regulation(GO:0065007)	1
OG0030896	Biological Process	cellular component organization or biogenesis(GO:0071840)	1
OG0030896	Biological Process	cellular process(GO:0009987)	1
OG0030896	Biological Process	metabolic process(GO:0008152)	1
OG0030896	Biological Process	regulation of biological process(GO:0050789)	1
OG0030896	Biological Process	response to stimulus(GO:0050896)	1
OG0030897	Biological Process	growth(GO:0040007)	1
OG0030905	Biological Process	multicellular organismal process(GO:0032501)	1
OG0030905	Biological Process	response to stimulus(GO:0050896)	1
OG0030907	Biological Process	biological regulation(GO:0065007)	1
OG0030907	Biological Process	cellular process(GO:0009987)	1
OG0030907	Biological Process	developmental process(GO:0032502)	1
OG0030907	Biological Process	immune system process(GO:0002376)	1
OG0030907	Biological Process	metabolic process(GO:0008152)	1
OG0030907	Biological Process	multi-organism process(GO:0051704)	1
OG0030907	Biological Process	multicellular organismal process(GO:0032501)	1
OG0030907	Biological Process	regulation of biological process(GO:0050789)	1
OG0030907	Biological Process	response to stimulus(GO:0050896)	1
OG0030907	Biological Process	signaling(GO:0023052)	1
OG0030908	Biological Process	biological regulation(GO:0065007)	1

OG0030908	Biological Process	cellular process(GO:0009987)	1
OG0030908	Biological Process	developmental process(GO:0032502)	1
OG0030908	Biological Process	immune system process(GO:0002376)	1
OG0030908	Biological Process	metabolic process(GO:0008152)	1
OG0030908	Biological Process	multi-organism process(GO:0051704)	1
OG0030908	Biological Process	multicellular organismal process(GO:0032501)	1
OG0030908	Biological Process	regulation of biological process(GO:0050789)	1
OG0030908	Biological Process	response to stimulus(GO:0050896)	1
OG0030908	Biological Process	signaling(GO:0023052)	1
OG0030909	Biological Process	biological regulation(GO:0065007)	1
OG0030909	Biological Process	cellular process(GO:0009987)	1
OG0030909	Biological Process	developmental process(GO:0032502)	1
OG0030909	Biological Process	immune system process(GO:0002376)	1
OG0030909	Biological Process	metabolic process(GO:0008152)	1
OG0030909	Biological Process	multi-organism process(GO:0051704)	1
OG0030909	Biological Process	multicellular organismal process(GO:0032501)	1
OG0030909	Biological Process	regulation of biological process(GO:0050789)	1
OG0030909	Biological Process	response to stimulus(GO:0050896)	1
OG0030909	Biological Process	signaling(GO:0023052)	1
OG0030913	Biological Process	biological regulation(GO:0065007)	1
OG0030913	Biological Process	cellular process(GO:0009987)	1
OG0030913	Biological Process	developmental process(GO:0032502)	1
OG0030913	Biological Process	growth(GO:0040007)	1
OG0030913	Biological Process	metabolic process(GO:0008152)	1
OG0030913	Biological Process	regulation of biological process(GO:0050789)	1
OG0030913	Biological Process	response to stimulus(GO:0050896)	1
OG0030927	Biological Process	cellular process(GO:0009987)	1
OG0030927	Biological Process	detoxification(GO:0098754)	1
OG0030927	Biological Process	metabolic process(GO:0008152)	1
OG0030927	Biological Process	response to stimulus(GO:0050896)	1
OG0030928	Biological Process	cellular process(GO:0009987)	1
OG0030928	Biological Process	localization(GO:0051179)	1
OG0030928	Biological Process	metabolic process(GO:0008152)	1
OG0030929	Biological Process	cellular process(GO:0009987)	1
OG0030929	Biological Process	metabolic process(GO:0008152)	1
OG0030931	Biological Process	cellular process(GO:0009987)	1
OG0030931	Biological Process	metabolic process(GO:0008152)	1
OG0030934	Biological Process	growth(GO:0040007)	1
OG0030936	Biological Process	biological regulation(GO:0065007)	1
OG0030936	Biological Process	cellular process(GO:0009987)	1
OG0030936	Biological Process	metabolic process(GO:0008152)	1
OG0030936	Biological Process	regulation of biological process(GO:0050789)	1
OG0030937	Biological Process	biological regulation(GO:0065007)	1
OG0030937	Biological Process	cellular process(GO:0009987)	1
OG0030937	Biological Process	metabolic process(GO:0008152)	1
OG0030937	Biological Process	regulation of biological process(GO:0050789)	1

OG0030940	Biological Process	cellular process(GO:0009987)	1
OG0030940	Biological Process	metabolic process(GO:0008152)	1
OG0030941	Biological Process	cellular process(GO:0009987)	1
OG0030941	Biological Process	metabolic process(GO:0008152)	1
OG0030942	Biological Process	growth(GO:0040007)	1
OG0030945	Biological Process	cellular process(GO:0009987)	1
OG0030945	Biological Process	growth(GO:0040007)	1
OG0030945	Biological Process	metabolic process(GO:0008152)	1
OG0030950	Biological Process	cellular component organization or biogenesis(GO:0071840)	1
OG0030950	Biological Process	cellular process(GO:0009987)	1
OG0030950	Biological Process	metabolic process(GO:0008152)	1
OG0030951	Biological Process	cellular process(GO:0009987)	1
OG0030951	Biological Process	metabolic process(GO:0008152)	1
OG0030952	Biological Process	cellular process(GO:0009987)	1
OG0030952	Biological Process	metabolic process(GO:0008152)	1
OG0030955	Biological Process	cellular process(GO:0009987)	1
OG0030955	Biological Process	metabolic process(GO:0008152)	1
OG0030962	Biological Process	cellular process(GO:0009987)	1
OG0030962	Biological Process	metabolic process(GO:0008152)	1
OG0030969	Biological Process	cellular process(GO:0009987)	1
OG0030969	Biological Process	metabolic process(GO:0008152)	1
OG0030969	Biological Process	response to stimulus(GO:0050896)	1
OG0030971	Biological Process	cellular process(GO:0009987)	1
OG0030971	Biological Process	metabolic process(GO:0008152)	1
OG0030971	Biological Process	response to stimulus(GO:0050896)	1
OG0030972	Biological Process	cellular process(GO:0009987)	1
OG0030972	Biological Process	growth(GO:0040007)	1
OG0030972	Biological Process	metabolic process(GO:0008152)	1
OG0030973	Biological Process	cellular process(GO:0009987)	1
OG0030973	Biological Process	metabolic process(GO:0008152)	1
OG0030975	Biological Process	cellular process(GO:0009987)	1
OG0030975	Biological Process	localization(GO:0051179)	1
OG0030975	Biological Process	metabolic process(GO:0008152)	1
OG0030979	Biological Process	growth(GO:0040007)	1
OG0030980	Biological Process	cellular process(GO:0009987)	1
OG0030980	Biological Process	metabolic process(GO:0008152)	1
OG0030984	Biological Process	cellular component organization or biogenesis(GO:0071840)	1
OG0030984	Biological Process	cellular process(GO:0009987)	1
OG0030984	Biological Process	localization(GO:0051179)	1
OG0030984	Biological Process	locomotion(GO:0040011)	1
OG0030987	Biological Process	cellular component organization or biogenesis(GO:0071840)	1
OG0030987	Biological Process	cellular process(GO:0009987)	1
OG0030987	Biological Process	response to stimulus(GO:0050896)	1
OG0031011	Biological Process	metabolic process(GO:0008152)	1
OG0031012	Biological Process	metabolic process(GO:0008152)	1
OG0031015	Biological Process	cellular process(GO:0009987)	1

OG0031015	Biological Process	metabolic process(GO:0008152)	1
OG0031015	Biological Process	multi-organism process(GO:0051704)	1
OG0031015	Biological Process	response to stimulus(GO:0050896)	1
OG0031016	Biological Process	metabolic process(GO:0008152)	1
OG0031017	Biological Process	multicellular organismal process(GO:0032501)	1
OG0031017	Biological Process	response to stimulus(GO:0050896)	1
OG0031019	Biological Process	cell proliferation(GO:0008283)	1
OG0031019	Biological Process	cellular process(GO:0009987)	1
OG0031019	Biological Process	developmental process(GO:0032502)	1
OG0031019	Biological Process	multicellular organismal process(GO:0032501)	1
OG0031027	Biological Process	response to stimulus(GO:0050896)	1
OG0031031	Biological Process	cellular process(GO:0009987)	1
OG0031031	Biological Process	developmental process(GO:0032502)	1
OG0031031	Biological Process	metabolic process(GO:0008152)	1
OG0031031	Biological Process	multicellular organismal process(GO:0032501)	1
OG0031031	Biological Process	reproduction(GO:0000003)	1
OG0031031	Biological Process	reproductive process(GO:0022414)	1
OG0031031	Biological Process	response to stimulus(GO:0050896)	1
OG0031059	Biological Process	cellular process(GO:0009987)	1
OG0031059	Biological Process	localization(GO:0051179)	1
OG0031060	Biological Process	biological regulation(GO:0065007)	1
OG0031060	Biological Process	cellular process(GO:0009987)	1
OG0031060	Biological Process	developmental process(GO:0032502)	1
OG0031060	Biological Process	metabolic process(GO:0008152)	1
OG0031060	Biological Process	multi-organism process(GO:0051704)	1
OG0031060	Biological Process	multicellular organismal process(GO:0032501)	1
OG0031060	Biological Process	regulation of biological process(GO:0050789)	1
OG0031060	Biological Process	reproduction(GO:0000003)	1
OG0031060	Biological Process	reproductive process(GO:0022414)	1
OG0031060	Biological Process	response to stimulus(GO:0050896)	1
OG0031063	Biological Process	metabolic process(GO:0008152)	1
OG0031080	Biological Process	biological regulation(GO:0065007)	1
OG0031080	Biological Process	cellular process(GO:0009987)	1
OG0031080	Biological Process	metabolic process(GO:0008152)	1
OG0031080	Biological Process	positive regulation of biological process(GO:0048518)	1
OG0031080	Biological Process	regulation of biological process(GO:0050789)	1
OG0031080	Biological Process	response to stimulus(GO:0050896)	1
OG0031086	Biological Process	developmental process(GO:0032502)	1
OG0031086	Biological Process	multicellular organismal process(GO:0032501)	1
OG0031088	Biological Process	metabolic process(GO:0008152)	1
OG0031096	Biological Process	response to stimulus(GO:0050896)	1
OG0031101	Biological Process	biological regulation(GO:0065007)	1
OG0031101	Biological Process	cellular process(GO:0009987)	1
OG0031101	Biological Process	developmental process(GO:0032502)	1
OG0031101	Biological Process	multicellular organismal process(GO:0032501)	1

OG0031101	Biological Process	regulation of biological process(GO:0050789)	1
OG0031101	Biological Process	response to stimulus(GO:0050896)	1
OG0031101	Biological Process	signaling(GO:0023052)	1
OG0031106	Biological Process	developmental process(GO:0032502)	1
OG0031106	Biological Process	multicellular organismal process(GO:0032501)	1
OG0031107	Biological Process	biological regulation(GO:0065007)	1
OG0031107	Biological Process	cellular process(GO:0009987)	1
OG0031107	Biological Process	developmental process(GO:0032502)	1
OG0031107	Biological Process	localization(GO:0051179)	1
OG0031107	Biological Process	multicellular organismal process(GO:0032501)	1
OG0031107	Biological Process	regulation of biological process(GO:0050789)	1
OG0031107	Biological Process	reproduction(GO:0000003)	1
OG0031107	Biological Process	reproductive process(GO:0022414)	1
OG0031107	Biological Process	response to stimulus(GO:0050896)	1
OG0031107	Biological Process	signaling(GO:0023052)	1
OG0031111	Biological Process	developmental process(GO:0032502)	1
OG0031111	Biological Process	multicellular organismal process(GO:0032501)	1
OG0031111	Biological Process	reproduction(GO:0000003)	1
OG0031111	Biological Process	reproductive process(GO:0022414)	1
OG0031120	Biological Process	biological regulation(GO:0065007)	1
OG0031120	Biological Process	cell aggregation(GO:0098743)	1
OG0031120	Biological Process	cell proliferation(GO:0008283)	1
OG0031120	Biological Process	cellular component organization or biogenesis(GO:0071840)	1
OG0031120	Biological Process	cellular process(GO:0009987)	1
OG0031120	Biological Process	developmental process(GO:0032502)	1
OG0031120	Biological Process	growth(GO:0040007)	1
OG0031120	Biological Process	metabolic process(GO:0008152)	1
OG0031120	Biological Process	multi-organism process(GO:0051704)	1
OG0031120	Biological Process	negative regulation of biological process(GO:0048519)	1
OG0031120	Biological Process	positive regulation of biological process(GO:0048518)	1
OG0031120	Biological Process	regulation of biological process(GO:0050789)	1
OG0031120	Biological Process	response to stimulus(GO:0050896)	1
OG0031120	Biological Process	signaling(GO:0023052)	1
OG0031144	Biological Process	cellular process(GO:0009987)	1
OG0031144	Biological Process	metabolic process(GO:0008152)	1
OG0031145	Biological Process	metabolic process(GO:0008152)	1
OG0031148	Biological Process	cellular process(GO:0009987)	1
OG0031148	Biological Process	metabolic process(GO:0008152)	1
OG0031149	Biological Process	cellular process(GO:0009987)	1
OG0031149	Biological Process	metabolic process(GO:0008152)	1
OG0031152	Biological Process	cellular process(GO:0009987)	1
OG0031152	Biological Process	growth(GO:0040007)	1
OG0031152	Biological Process	metabolic process(GO:0008152)	1
OG0031155	Biological Process	cellular process(GO:0009987)	1
OG0031155	Biological Process	metabolic process(GO:0008152)	1

OG0031157	Biological Process	metabolic process(GO:0008152)	1
OG0031160	Biological Process	growth(GO:0040007)	1
OG0031172	Biological Process	cellular process(GO:0009987)	1
OG0031172	Biological Process	localization(GO:0051179)	1
OG0031172	Biological Process	metabolic process(GO:0008152)	1
OG0031174	Biological Process	biological adhesion(GO:0022610)	1
OG0031174	Biological Process	cellular component organization or biogenesis(GO:0071840)	1
OG0031174	Biological Process	cellular process(GO:0009987)	1
OG0031174	Biological Process	growth(GO:0040007)	1
OG0031174	Biological Process	metabolic process(GO:0008152)	1
OG0031174	Biological Process	multi-organism process(GO:0051704)	1
OG0031174	Biological Process	reproduction(GO:0000003)	1
OG0031174	Biological Process	response to stimulus(GO:0050896)	1
OG0031175	Biological Process	growth(GO:0040007)	1
OG0031177	Biological Process	metabolic process(GO:0008152)	1
OG0031185	Biological Process	metabolic process(GO:0008152)	1
OG0031189	Biological Process	cellular component organization or biogenesis(GO:0071840)	1
OG0031189	Biological Process	cellular process(GO:0009987)	1
OG0031189	Biological Process	growth(GO:0040007)	1
OG0031189	Biological Process	metabolic process(GO:0008152)	1
OG0031190	Biological Process	biological regulation(GO:0065007)	1
OG0031190	Biological Process	cellular process(GO:0009987)	1
OG0031190	Biological Process	metabolic process(GO:0008152)	1
OG0031190	Biological Process	negative regulation of biological process(GO:0048519)	1
OG0031190	Biological Process	regulation of biological process(GO:0050789)	1
OG0031190	Biological Process	response to stimulus(GO:0050896)	1
OG0031191	Biological Process	biological regulation(GO:0065007)	1
OG0031191	Biological Process	cellular process(GO:0009987)	1
OG0031191	Biological Process	metabolic process(GO:0008152)	1
OG0031191	Biological Process	regulation of biological process(GO:0050789)	1
OG0031194	Biological Process	growth(GO:0040007)	1
OG0031194	Biological Process	metabolic process(GO:0008152)	1
OG0031194	Biological Process	multi-organism process(GO:0051704)	1
OG0031194	Biological Process	response to stimulus(GO:0050896)	1
OG0031195	Biological Process	cellular process(GO:0009987)	1
OG0031195	Biological Process	growth(GO:0040007)	1
OG0031195	Biological Process	metabolic process(GO:0008152)	1
OG0031197	Biological Process	cellular component organization or biogenesis(GO:0071840)	1
OG0031197	Biological Process	cellular process(GO:0009987)	1
OG0031197	Biological Process	growth(GO:0040007)	1
OG0031197	Biological Process	localization(GO:0051179)	1
OG0031197	Biological Process	metabolic process(GO:0008152)	1
OG0031199	Biological Process	cellular process(GO:0009987)	1
OG0031199	Biological Process	localization(GO:0051179)	1
OG0031199	Biological Process	metabolic process(GO:0008152)	1
OG0031199	Biological Process	response to stimulus(GO:0050896)	1

OG0031200	Biological Process	growth(GO:0040007)	1
OG0031200	Biological Process	metabolic process(GO:0008152)	1
OG0031203	Biological Process	cellular process(GO:0009987)	1
OG0031203	Biological Process	metabolic process(GO:0008152)	1
OG0031203	Biological Process	response to stimulus(GO:0050896)	1
OG0031204	Biological Process	cellular process(GO:0009987)	1
OG0031204	Biological Process	growth(GO:0040007)	1
OG0031204	Biological Process	metabolic process(GO:0008152)	1
OG0031208	Biological Process	cellular process(GO:0009987)	1
OG0031208	Biological Process	growth(GO:0040007)	1
OG0031208	Biological Process	metabolic process(GO:0008152)	1
OG0031209	Biological Process	cellular process(GO:0009987)	1
OG0031209	Biological Process	growth(GO:0040007)	1
OG0031209	Biological Process	metabolic process(GO:0008152)	1
OG0031212	Biological Process	cellular process(GO:0009987)	1
OG0031212	Biological Process	growth(GO:0040007)	1
OG0031212	Biological Process	localization(GO:0051179)	1
OG0031212	Biological Process	metabolic process(GO:0008152)	1
OG0031214	Biological Process	cellular process(GO:0009987)	1
OG0031214	Biological Process	growth(GO:0040007)	1
OG0031214	Biological Process	metabolic process(GO:0008152)	1
OG0031217	Biological Process	cellular process(GO:0009987)	1
OG0031217	Biological Process	detoxification(GO:0098754)	1
OG0031217	Biological Process	metabolic process(GO:0008152)	1
OG0031217	Biological Process	response to stimulus(GO:0050896)	1
OG0031223	Biological Process	cellular process(GO:0009987)	1
OG0031223	Biological Process	localization(GO:0051179)	1
OG0031223	Biological Process	metabolic process(GO:0008152)	1
OG0031223	Biological Process	response to stimulus(GO:0050896)	1
OG0031228	Biological Process	cellular process(GO:0009987)	1
OG0031228	Biological Process	growth(GO:0040007)	1
OG0031228	Biological Process	metabolic process(GO:0008152)	1
OG0031231	Biological Process	growth(GO:0040007)	1
OG0031232	Biological Process	metabolic process(GO:0008152)	1
OG0031233	Biological Process	cellular process(GO:0009987)	1
OG0031233	Biological Process	metabolic process(GO:0008152)	1
OG0031235	Biological Process	cellular process(GO:0009987)	1
OG0031235	Biological Process	growth(GO:0040007)	1
OG0031235	Biological Process	metabolic process(GO:0008152)	1
OG0031235	Biological Process	response to stimulus(GO:0050896)	1
OG0031241	Biological Process	cellular process(GO:0009987)	1
OG0031241	Biological Process	metabolic process(GO:0008152)	1
OG0031241	Biological Process	response to stimulus(GO:0050896)	1
OG0031248	Biological Process	cellular process(GO:0009987)	1
OG0031248	Biological Process	metabolic process(GO:0008152)	1
OG0031249	Biological Process	cellular process(GO:0009987)	1
OG0031249	Biological Process	metabolic process(GO:0008152)	1

OG0031250	Biological Process	cellular process(GO:0009987)	1
OG0031250	Biological Process	localization(GO:0051179)	1
OG0031250	Biological Process	metabolic process(GO:0008152)	1
OG0031252	Biological Process	cellular process(GO:0009987)	1
OG0031252	Biological Process	metabolic process(GO:0008152)	1
OG0031254	Biological Process	cellular process(GO:0009987)	1
OG0031254	Biological Process	growth(GO:0040007)	1
OG0031254	Biological Process	metabolic process(GO:0008152)	1
OG0031256	Biological Process	cellular process(GO:0009987)	1
OG0031256	Biological Process	metabolic process(GO:0008152)	1
OG0031258	Biological Process	biological regulation(GO:0065007)	1
OG0031258	Biological Process	cellular process(GO:0009987)	1
OG0031258	Biological Process	developmental process(GO:0032502)	1
OG0031258	Biological Process	growth(GO:0040007)	1
OG0031258	Biological Process	metabolic process(GO:0008152)	1
OG0031258	Biological Process	regulation of biological process(GO:0050789)	1
OG0031258	Biological Process	response to stimulus(GO:0050896)	1
OG0031259	Biological Process	cellular process(GO:0009987)	1
OG0031259	Biological Process	metabolic process(GO:0008152)	1
OG0031273	Biological Process	biological regulation(GO:0065007)	1
OG0031273	Biological Process	cellular component organization or biogenesis(GO:0071840)	1
OG0031273	Biological Process	cellular process(GO:0009987)	1
OG0031273	Biological Process	developmental process(GO:0032502)	1
OG0031273	Biological Process	regulation of biological process(GO:0050789)	1
OG0031273	Biological Process	response to stimulus(GO:0050896)	1
OG0031277	Biological Process	cellular component organization or biogenesis(GO:0071840)	1
OG0031277	Biological Process	cellular process(GO:0009987)	1
OG0031277	Biological Process	metabolic process(GO:0008152)	1
OG0031277	Biological Process	response to stimulus(GO:0050896)	1
OG0031282	Biological Process	cellular component organization or biogenesis(GO:0071840)	1
OG0031282	Biological Process	cellular process(GO:0009987)	1
OG0031282	Biological Process	developmental process(GO:0032502)	1
OG0031282	Biological Process	growth(GO:0040007)	1
OG0031282	Biological Process	metabolic process(GO:0008152)	1
OG0031283	Biological Process	cellular process(GO:0009987)	1
OG0031283	Biological Process	developmental process(GO:0032502)	1
OG0031292	Biological Process	biological regulation(GO:0065007)	1
OG0031292	Biological Process	cellular component organization or biogenesis(GO:0071840)	1
OG0031292	Biological Process	cellular process(GO:0009987)	1
OG0031292	Biological Process	developmental process(GO:0032502)	1
OG0031292	Biological Process	regulation of biological process(GO:0050789)	1
OG0031292	Biological Process	response to stimulus(GO:0050896)	1
OG0031318	Biological Process	biological regulation(GO:0065007)	1
OG0031318	Biological Process	cellular process(GO:0009987)	1
OG0031318	Biological Process	metabolic process(GO:0008152)	1

OG0031318	Biological Process	regulation of biological process(GO:0050789)	1
OG0031319	Biological Process	cellular process(GO:0009987)	1
OG0031319	Biological Process	immune system process(GO:0002376)	1
OG0031319	Biological Process	metabolic process(GO:0008152)	1
OG0031319	Biological Process	multi-organism process(GO:0051704)	1
OG0031319	Biological Process	response to stimulus(GO:0050896)	1
OG0031324	Biological Process	multi-organism process(GO:0051704)	1
OG0031324	Biological Process	response to stimulus(GO:0050896)	1
OG0031330	Biological Process	biological regulation(GO:0065007)	1
OG0031330	Biological Process	cellular process(GO:0009987)	1
OG0031330	Biological Process	metabolic process(GO:0008152)	1
OG0031330	Biological Process	negative regulation of biological process(GO:0048519)	1
OG0031330	Biological Process	regulation of biological process(GO:0050789)	1
OG0031331	Biological Process	biological regulation(GO:0065007)	1
OG0031331	Biological Process	cellular component organization or biogenesis(GO:0071840)	1
OG0031331	Biological Process	cellular process(GO:0009987)	1
OG0031331	Biological Process	developmental process(GO:0032502)	1
OG0031331	Biological Process	growth(GO:0040007)	1
OG0031331	Biological Process	immune system process(GO:0002376)	1
OG0031331	Biological Process	localization(GO:0051179)	1
OG0031331	Biological Process	locomotion(GO:0040011)	1
OG0031331	Biological Process	metabolic process(GO:0008152)	1
OG0031331	Biological Process	multi-organism process(GO:0051704)	1
OG0031331	Biological Process	multicellular organismal process(GO:0032501)	1
OG0031331	Biological Process	negative regulation of biological process(GO:0048519)	1
OG0031331	Biological Process	positive regulation of biological process(GO:0048518)	1
OG0031331	Biological Process	regulation of biological process(GO:0050789)	1
OG0031331	Biological Process	reproduction(GO:0000003)	1
OG0031331	Biological Process	reproductive process(GO:0022414)	1
OG0031337	Biological Process	cellular process(GO:0009987)	1
OG0031337	Biological Process	metabolic process(GO:0008152)	1
OG0031337	Biological Process	multi-organism process(GO:0051704)	1
OG0031337	Biological Process	response to stimulus(GO:0050896)	1
OG0031340	Biological Process	biological regulation(GO:0065007)	1
OG0031340	Biological Process	cellular process(GO:0009987)	1
OG0031340	Biological Process	metabolic process(GO:0008152)	1
OG0031340	Biological Process	regulation of biological process(GO:0050789)	1
OG0031341	Biological Process	biological regulation(GO:0065007)	1
OG0031349	Biological Process	metabolic process(GO:0008152)	1
OG0031352	Biological Process	cellular process(GO:0009987)	1
OG0031352	Biological Process	metabolic process(GO:0008152)	1
OG0031352	Biological Process	response to stimulus(GO:0050896)	1
OG0031356	Biological Process	cellular process(GO:0009987)	1
OG0031356	Biological Process	metabolic process(GO:0008152)	1
OG0031377	Biological Process	cellular process(GO:0009987)	1

OG0031377	Biological Process	developmental process(GO:0032502)	1
OG0031377	Biological Process	immune system process(GO:0002376)	1
OG0031377	Biological Process	metabolic process(GO:0008152)	1
OG0031377	Biological Process	multi-organism process(GO:0051704)	1
OG0031377	Biological Process	multicellular organismal process(GO:0032501)	1
OG0031377	Biological Process	reproduction(GO:0000003)	1
OG0031377	Biological Process	reproductive process(GO:0022414)	1
OG0031377	Biological Process	response to stimulus(GO:0050896)	1
OG0031382	Biological Process	cellular component organization or biogenesis(GO:0071840)	1
OG0031382	Biological Process	cellular process(GO:0009987)	1
OG0031382	Biological Process	localization(GO:0051179)	1
OG0031382	Biological Process	metabolic process(GO:0008152)	1
OG0031382	Biological Process	multi-organism process(GO:0051704)	1
OG0031382	Biological Process	response to stimulus(GO:0050896)	1
OG0031383	Biological Process	growth(GO:0040007)	1
OG0031383	Biological Process	metabolic process(GO:0008152)	1
OG0031383	Biological Process	multi-organism process(GO:0051704)	1
OG0031383	Biological Process	response to stimulus(GO:0050896)	1
OG0031384	Biological Process	cellular component organization or biogenesis(GO:0071840)	1
OG0031384	Biological Process	cellular process(GO:0009987)	1
OG0031384	Biological Process	growth(GO:0040007)	1
OG0031384	Biological Process	metabolic process(GO:0008152)	1
OG0031386	Biological Process	cellular process(GO:0009987)	1
OG0031386	Biological Process	growth(GO:0040007)	1
OG0031386	Biological Process	metabolic process(GO:0008152)	1
OG0031386	Biological Process	multi-organism process(GO:0051704)	1
OG0031386	Biological Process	response to stimulus(GO:0050896)	1
OG0031396	Biological Process	cellular process(GO:0009987)	1
OG0031396	Biological Process	detoxification(GO:0098754)	1
OG0031396	Biological Process	metabolic process(GO:0008152)	1
OG0031396	Biological Process	response to stimulus(GO:0050896)	1
OG0031401	Biological Process	cellular component organization or biogenesis(GO:0071840)	1
OG0031401	Biological Process	cellular process(GO:0009987)	1
OG0031401	Biological Process	growth(GO:0040007)	1
OG0031401	Biological Process	metabolic process(GO:0008152)	1
OG0031402	Biological Process	cellular process(GO:0009987)	1
OG0031402	Biological Process	metabolic process(GO:0008152)	1
OG0031415	Biological Process	biological regulation(GO:0065007)	1
OG0031415	Biological Process	cellular component organization or biogenesis(GO:0071840)	1
OG0031415	Biological Process	cellular process(GO:0009987)	1
OG0031415	Biological Process	developmental process(GO:0032502)	1
OG0031415	Biological Process	immune system process(GO:0002376)	1
OG0031415	Biological Process	metabolic process(GO:0008152)	1
OG0031415	Biological Process	multi-organism process(GO:0051704)	1
OG0031415	Biological Process	multicellular organismal process(GO:0032501)	1
OG0031415	Biological Process	negative regulation of biological process(GO:0048519)	1

OG0031415	Biological Process	positive regulation of biological process(GO:0048518)	1
OG0031415	Biological Process	regulation of biological process(GO:0050789)	1
OG0031415	Biological Process	reproduction(GO:0000003)	1
OG0031415	Biological Process	reproductive process(GO:0022414)	1
OG0031415	Biological Process	response to stimulus(GO:0050896)	1
OG0031415	Biological Process	signaling(GO:0023052)	1
OG0031416	Biological Process	biological regulation(GO:0065007)	1
OG0031416	Biological Process	cellular process(GO:0009987)	1
OG0031416	Biological Process	developmental process(GO:0032502)	1
OG0031416	Biological Process	metabolic process(GO:0008152)	1
OG0031416	Biological Process	multicellular organismal process(GO:0032501)	1
OG0031416	Biological Process	regulation of biological process(GO:0050789)	1
OG0031416	Biological Process	response to stimulus(GO:0050896)	1
OG0031419	Biological Process	biological regulation(GO:0065007)	1
OG0031419	Biological Process	cellular process(GO:0009987)	1
OG0031419	Biological Process	developmental process(GO:0032502)	1
OG0031419	Biological Process	metabolic process(GO:0008152)	1
OG0031419	Biological Process	multicellular organismal process(GO:0032501)	1
OG0031419	Biological Process	negative regulation of biological process(GO:0048519)	1
OG0031419	Biological Process	regulation of biological process(GO:0050789)	1
OG0031419	Biological Process	reproduction(GO:0000003)	1
OG0031419	Biological Process	reproductive process(GO:0022414)	1
OG0031421	Biological Process	cellular component organization or biogenesis(GO:0071840)	1
OG0031421	Biological Process	cellular process(GO:0009987)	1
OG0031421	Biological Process	developmental process(GO:0032502)	1
OG0031421	Biological Process	metabolic process(GO:0008152)	1
OG0031421	Biological Process	multicellular organismal process(GO:0032501)	1
OG0031423	Biological Process	cellular process(GO:0009987)	1
OG0031423	Biological Process	metabolic process(GO:0008152)	1
OG0031425	Biological Process	biological regulation(GO:0065007)	1
OG0031425	Biological Process	cellular process(GO:0009987)	1
OG0031425	Biological Process	metabolic process(GO:0008152)	1
OG0031425	Biological Process	regulation of biological process(GO:0050789)	1
OG0031434	Biological Process	cellular component organization or biogenesis(GO:0071840)	1
OG0031434	Biological Process	cellular process(GO:0009987)	1
OG0031434	Biological Process	metabolic process(GO:0008152)	1
OG0031435	Biological Process	cellular component organization or biogenesis(GO:0071840)	1
OG0031435	Biological Process	cellular process(GO:0009987)	1
OG0031435	Biological Process	metabolic process(GO:0008152)	1
OG0031437	Biological Process	biological regulation(GO:0065007)	1
OG0031437	Biological Process	cellular component organization or biogenesis(GO:0071840)	1
OG0031437	Biological Process	cellular process(GO:0009987)	1
OG0031437	Biological Process	metabolic process(GO:0008152)	1
OG0031437	Biological Process	positive regulation of biological process(GO:0048518)	1

OG0031437	Biological Process	regulation of biological process(GO:0050789)	1
OG0031442	Biological Process	metabolic process(GO:0008152)	1
OG0031448	Biological Process	cellular process(GO:0009987)	1
OG0031448	Biological Process	metabolic process(GO:0008152)	1
OG0031460	Biological Process	cellular process(GO:0009987)	1
OG0031460	Biological Process	developmental process(GO:0032502)	1
OG0031460	Biological Process	localization(GO:0051179)	1
OG0031460	Biological Process	metabolic process(GO:0008152)	1
OG0031460	Biological Process	signaling(GO:0023052)	1
OG0031466	Biological Process	cellular process(GO:0009987)	1
OG0031466	Biological Process	metabolic process(GO:0008152)	1
OG0031474	Biological Process	cellular component organization or biogenesis(GO:0071840)	1
OG0031474	Biological Process	cellular process(GO:0009987)	1
OG0031474	Biological Process	metabolic process(GO:0008152)	1
OG0031487	Biological Process	cellular process(GO:0009987)	1
OG0031487	Biological Process	metabolic process(GO:0008152)	1
OG0031489	Biological Process	metabolic process(GO:0008152)	1
OG0031502	Biological Process	cellular process(GO:0009987)	1
OG0031502	Biological Process	growth(GO:0040007)	1
OG0031502	Biological Process	localization(GO:0051179)	1
OG0031503	Biological Process	cellular process(GO:0009987)	1
OG0031503	Biological Process	metabolic process(GO:0008152)	1
OG0031505	Biological Process	cellular component organization or biogenesis(GO:0071840)	1
OG0031505	Biological Process	cellular process(GO:0009987)	1
OG0031505	Biological Process	growth(GO:0040007)	1
OG0031505	Biological Process	localization(GO:0051179)	1
OG0031505	Biological Process	metabolic process(GO:0008152)	1
OG0031524	Biological Process	developmental process(GO:0032502)	1
OG0031524	Biological Process	metabolic process(GO:0008152)	1
OG0031524	Biological Process	multi-organism process(GO:0051704)	1
OG0031524	Biological Process	multicellular organismal process(GO:0032501)	1
OG0031524	Biological Process	response to stimulus(GO:0050896)	1
OG0031526	Biological Process	cellular process(GO:0009987)	1
OG0031526	Biological Process	metabolic process(GO:0008152)	1
OG0031526	Biological Process	response to stimulus(GO:0050896)	1
OG0031528	Biological Process	cellular component organization or biogenesis(GO:0071840)	1
OG0031528	Biological Process	cellular process(GO:0009987)	1
OG0031528	Biological Process	developmental process(GO:0032502)	1
OG0031528	Biological Process	immune system process(GO:0002376)	1
OG0031528	Biological Process	metabolic process(GO:0008152)	1
OG0031528	Biological Process	multi-organism process(GO:0051704)	1
OG0031528	Biological Process	multicellular organismal process(GO:0032501)	1
OG0031528	Biological Process	response to stimulus(GO:0050896)	1
OG0031532	Biological Process	biological regulation(GO:0065007)	1
OG0031532	Biological Process	cellular process(GO:0009987)	1
OG0031532	Biological Process	developmental process(GO:0032502)	1

OG0031532	Biological Process	metabolic process(GO:0008152)	1
OG0031532	Biological Process	multicellular organismal process(GO:0032501)	1
OG0031532	Biological Process	negative regulation of biological process(GO:0048519)	1
OG0031532	Biological Process	regulation of biological process(GO:0050789)	1
OG0031532	Biological Process	reproduction(GO:0000003)	1
OG0031532	Biological Process	reproductive process(GO:0022414)	1
OG0031532	Biological Process	response to stimulus(GO:0050896)	1
OG0031532	Biological Process	rhythmic process(GO:0048511)	1
OG0031538	Biological Process	response to stimulus(GO:0050896)	1
OG0031555	Biological Process	cellular process(GO:0009987)	1
OG0031555	Biological Process	localization(GO:0051179)	1
OG0031555	Biological Process	response to stimulus(GO:0050896)	1
OG0031557	Biological Process	biological regulation(GO:0065007)	1
OG0031557	Biological Process	cellular process(GO:0009987)	1
OG0031557	Biological Process	metabolic process(GO:0008152)	1
OG0031557	Biological Process	multi-organism process(GO:0051704)	1
OG0031557	Biological Process	response to stimulus(GO:0050896)	1
OG0031560	Biological Process	cellular process(GO:0009987)	1
OG0031560	Biological Process	metabolic process(GO:0008152)	1
OG0031564	Biological Process	cellular process(GO:0009987)	1
OG0031564	Biological Process	metabolic process(GO:0008152)	1
OG0031565	Biological Process	biological regulation(GO:0065007)	1
OG0031565	Biological Process	cellular process(GO:0009987)	1
OG0031565	Biological Process	developmental process(GO:0032502)	1
OG0031565	Biological Process	metabolic process(GO:0008152)	1
OG0031565	Biological Process	multicellular organismal process(GO:0032501)	1
OG0031565	Biological Process	regulation of biological process(GO:0050789)	1
OG0031565	Biological Process	reproduction(GO:0000003)	1
OG0031565	Biological Process	reproductive process(GO:0022414)	1
OG0031580	Biological Process	response to stimulus(GO:0050896)	1
OG0031582	Biological Process	response to stimulus(GO:0050896)	1
OG0031583	Biological Process	developmental process(GO:0032502)	1
OG0031583	Biological Process	growth(GO:0040007)	1
OG0031583	Biological Process	response to stimulus(GO:0050896)	1
OG0031588	Biological Process	metabolic process(GO:0008152)	1
OG0031594	Biological Process	cellular process(GO:0009987)	1
OG0031594	Biological Process	developmental process(GO:0032502)	1
OG0031594	Biological Process	multicellular organismal process(GO:0032501)	1
OG0031595	Biological Process	biological regulation(GO:0065007)	1
OG0031595	Biological Process	cellular component organization or biogenesis(GO:0071840)	1
OG0031595	Biological Process	cellular process(GO:0009987)	1
OG0031595	Biological Process	developmental process(GO:0032502)	1
OG0031595	Biological Process	localization(GO:0051179)	1
OG0031595	Biological Process	metabolic process(GO:0008152)	1
OG0031595	Biological Process	multicellular organismal process(GO:0032501)	1

OG0031595	Biological Process	negative regulation of biological process(GO:0048519)	1
OG0031595	Biological Process	regulation of biological process(GO:0050789)	1
OG0031595	Biological Process	response to stimulus(GO:0050896)	1
OG0031602	Biological Process	cellular process(GO:0009987)	1
OG0031602	Biological Process	metabolic process(GO:0008152)	1
OG0031605	Biological Process	biological regulation(GO:0065007)	1
OG0031605	Biological Process	cellular process(GO:0009987)	1
OG0031605	Biological Process	developmental process(GO:0032502)	1
OG0031605	Biological Process	immune system process(GO:0002376)	1
OG0031605	Biological Process	metabolic process(GO:0008152)	1
OG0031605	Biological Process	multi-organism process(GO:0051704)	1
OG0031605	Biological Process	multicellular organismal process(GO:0032501)	1
OG0031605	Biological Process	negative regulation of biological process(GO:0048519)	1
OG0031605	Biological Process	regulation of biological process(GO:0050789)	1
OG0031605	Biological Process	reproduction(GO:0000003)	1
OG0031605	Biological Process	reproductive process(GO:0022414)	1
OG0031605	Biological Process	response to stimulus(GO:0050896)	1
OG0031605	Biological Process	signaling(GO:0023052)	1
OG0031607	Biological Process	cellular process(GO:0009987)	1
OG0031607	Biological Process	localization(GO:0051179)	1
OG0031630	Biological Process	cellular component organization or biogenesis(GO:0071840)	1
OG0031630	Biological Process	cellular process(GO:0009987)	1
OG0031630	Biological Process	developmental process(GO:0032502)	1
OG0031630	Biological Process	metabolic process(GO:0008152)	1
OG0031630	Biological Process	multicellular organismal process(GO:0032501)	1
OG0031639	Biological Process	biological regulation(GO:0065007)	1
OG0031639	Biological Process	cellular process(GO:0009987)	1
OG0031639	Biological Process	metabolic process(GO:0008152)	1
OG0031639	Biological Process	positive regulation of biological process(GO:0048518)	1
OG0031639	Biological Process	regulation of biological process(GO:0050789)	1
OG0031646	Biological Process	biological regulation(GO:0065007)	1
OG0031646	Biological Process	cellular process(GO:0009987)	1
OG0031646	Biological Process	metabolic process(GO:0008152)	1
OG0031646	Biological Process	regulation of biological process(GO:0050789)	1
OG0031648	Biological Process	metabolic process(GO:0008152)	1
OG0031648	Biological Process	response to stimulus(GO:0050896)	1
OG0031665	Biological Process	biological regulation(GO:0065007)	1
OG0031665	Biological Process	cellular process(GO:0009987)	1
OG0031665	Biological Process	metabolic process(GO:0008152)	1
OG0031665	Biological Process	positive regulation of biological process(GO:0048518)	1
OG0031665	Biological Process	regulation of biological process(GO:0050789)	1
OG0031680	Biological Process	response to stimulus(GO:0050896)	1
OG0031681	Biological Process	cellular process(GO:0009987)	1
OG0031681	Biological Process	metabolic process(GO:0008152)	1

OG0031681	Biological Process	response to stimulus(GO:0050896)	1
OG0031685	Biological Process	response to stimulus(GO:0050896)	1
OG0031689	Biological Process	biological regulation(GO:0065007)	1
OG0031689	Biological Process	cellular process(GO:0009987)	1
OG0031689	Biological Process	metabolic process(GO:0008152)	1
OG0031689	Biological Process	regulation of biological process(GO:0050789)	1
OG0031695	Biological Process	response to stimulus(GO:0050896)	1
OG0031703	Biological Process	cellular component organization or biogenesis(GO:0071840)	1
OG0031703	Biological Process	cellular process(GO:0009987)	1
OG0031703	Biological Process	metabolic process(GO:0008152)	1
OG0031704	Biological Process	cellular process(GO:0009987)	1
OG0031704	Biological Process	growth(GO:0040007)	1
OG0031704	Biological Process	metabolic process(GO:0008152)	1
OG0031706	Biological Process	growth(GO:0040007)	1
OG0031710	Biological Process	cellular process(GO:0009987)	1
OG0031710	Biological Process	metabolic process(GO:0008152)	1
OG0031711	Biological Process	cellular process(GO:0009987)	1
OG0031711	Biological Process	localization(GO:0051179)	1
OG0031711	Biological Process	metabolic process(GO:0008152)	1
OG0031711	Biological Process	response to stimulus(GO:0050896)	1
OG0031718	Biological Process	cellular process(GO:0009987)	1
OG0031718	Biological Process	metabolic process(GO:0008152)	1
OG0031722	Biological Process	cellular process(GO:0009987)	1
OG0031722	Biological Process	growth(GO:0040007)	1
OG0031722	Biological Process	localization(GO:0051179)	1
OG0031722	Biological Process	metabolic process(GO:0008152)	1
OG0031724	Biological Process	cellular process(GO:0009987)	1
OG0031724	Biological Process	localization(GO:0051179)	1
OG0031724	Biological Process	metabolic process(GO:0008152)	1
OG0031726	Biological Process	cellular process(GO:0009987)	1
OG0031726	Biological Process	growth(GO:0040007)	1
OG0031726	Biological Process	metabolic process(GO:0008152)	1
OG0031733	Biological Process	cellular process(GO:0009987)	1
OG0031733	Biological Process	localization(GO:0051179)	1
OG0031733	Biological Process	metabolic process(GO:0008152)	1
OG0031736	Biological Process	cellular process(GO:0009987)	1
OG0031736	Biological Process	localization(GO:0051179)	1
OG0031736	Biological Process	metabolic process(GO:0008152)	1
OG0031739	Biological Process	cellular process(GO:0009987)	1
OG0031739	Biological Process	metabolic process(GO:0008152)	1
OG0031743	Biological Process	growth(GO:0040007)	1
OG0031745	Biological Process	cellular process(GO:0009987)	1
OG0031745	Biological Process	metabolic process(GO:0008152)	1
OG0031747	Biological Process	cellular process(GO:0009987)	1
OG0031747	Biological Process	localization(GO:0051179)	1
OG0031747	Biological Process	metabolic process(GO:0008152)	1
OG0031750	Biological Process	cellular process(GO:0009987)	1

OG0031750	Biological Process	metabolic process(GO:0008152)	1
OG0031757	Biological Process	cellular process(GO:0009987)	1
OG0031757	Biological Process	metabolic process(GO:0008152)	1
OG0031758	Biological Process	growth(GO:0040007)	1
OG0031759	Biological Process	cellular process(GO:0009987)	1
OG0031759	Biological Process	metabolic process(GO:0008152)	1
OG0031762	Biological Process	cellular process(GO:0009987)	1
OG0031762	Biological Process	detoxification(GO:0098754)	1
OG0031762	Biological Process	metabolic process(GO:0008152)	1
OG0031762	Biological Process	response to stimulus(GO:0050896)	1
OG0031766	Biological Process	cellular process(GO:0009987)	1
OG0031766	Biological Process	developmental process(GO:0032502)	1
OG0031771	Biological Process	cellular process(GO:0009987)	1
OG0031771	Biological Process	developmental process(GO:0032502)	1
OG0031773	Biological Process	cellular process(GO:0009987)	1
OG0031773	Biological Process	developmental process(GO:0032502)	1
OG0031776	Biological Process	cellular process(GO:0009987)	1
OG0031776	Biological Process	metabolic process(GO:0008152)	1
OG0031777	Biological Process	cellular process(GO:0009987)	1
OG0031777	Biological Process	metabolic process(GO:0008152)	1
OG0031780	Biological Process	cellular process(GO:0009987)	1
OG0031780	Biological Process	localization(GO:0051179)	1
OG0031784	Biological Process	biological regulation(GO:0065007)	1
OG0031784	Biological Process	cellular component organization or biogenesis(GO:0071840)	1
OG0031784	Biological Process	cellular process(GO:0009987)	1
OG0031784	Biological Process	developmental process(GO:0032502)	1
OG0031784	Biological Process	growth(GO:0040007)	1
OG0031784	Biological Process	localization(GO:0051179)	1
OG0031784	Biological Process	metabolic process(GO:0008152)	1
OG0031784	Biological Process	multi-organism process(GO:0051704)	1
OG0031784	Biological Process	multicellular organismal process(GO:0032501)	1
OG0031784	Biological Process	negative regulation of biological process(GO:0048519)	1
OG0031784	Biological Process	regulation of biological process(GO:0050789)	1
OG0031784	Biological Process	reproduction(GO:0000003)	1
OG0031784	Biological Process	reproductive process(GO:0022414)	1
OG0031785	Biological Process	biological regulation(GO:0065007)	1
OG0031785	Biological Process	cellular process(GO:0009987)	1
OG0031785	Biological Process	immune system process(GO:0002376)	1
OG0031785	Biological Process	localization(GO:0051179)	1
OG0031785	Biological Process	metabolic process(GO:0008152)	1
OG0031785	Biological Process	multi-organism process(GO:0051704)	1
OG0031785	Biological Process	positive regulation of biological process(GO:0048518)	1
OG0031785	Biological Process	regulation of biological process(GO:0050789)	1
OG0031785	Biological Process	response to stimulus(GO:0050896)	1
OG0031785	Biological Process	signaling(GO:0023052)	1
OG0031787	Biological Process	biological regulation(GO:0065007)	1

OG0031787	Biological Process	cellular process(GO:0009987)	1
OG0031787	Biological Process	localization(GO:0051179)	1
OG0031787	Biological Process	metabolic process(GO:0008152)	1
OG0031787	Biological Process	regulation of biological process(GO:0050789)	1
OG0031787	Biological Process	response to stimulus(GO:0050896)	1
OG0031789	Biological Process	cellular process(GO:0009987)	1
OG0031789	Biological Process	metabolic process(GO:0008152)	1
OG0031790	Biological Process	metabolic process(GO:0008152)	1
OG0031793	Biological Process	cellular process(GO:0009987)	1
OG0031793	Biological Process	metabolic process(GO:0008152)	1
OG0031795	Biological Process	cellular process(GO:0009987)	1
OG0031795	Biological Process	metabolic process(GO:0008152)	1
OG0031805	Biological Process	biological regulation(GO:0065007)	1
OG0031805	Biological Process	cellular process(GO:0009987)	1
OG0031805	Biological Process	metabolic process(GO:0008152)	1
OG0031805	Biological Process	regulation of biological process(GO:0050789)	1
OG0031806	Biological Process	cellular process(GO:0009987)	1
OG0031806	Biological Process	metabolic process(GO:0008152)	1
OG0031807	Biological Process	biological regulation(GO:0065007)	1
OG0031807	Biological Process	cellular process(GO:0009987)	1
OG0031807	Biological Process	developmental process(GO:0032502)	1
OG0031807	Biological Process	metabolic process(GO:0008152)	1
OG0031807	Biological Process	multi-organism process(GO:0051704)	1
OG0031807	Biological Process	multicellular organismal process(GO:0032501)	1
OG0031807	Biological Process	positive regulation of biological process(GO:0048518)	1
OG0031807	Biological Process	regulation of biological process(GO:0050789)	1
OG0031807	Biological Process	reproduction(GO:0000003)	1
OG0031807	Biological Process	reproductive process(GO:0022414)	1
OG0031807	Biological Process	response to stimulus(GO:0050896)	1
OG0031807	Biological Process	signaling(GO:0023052)	1
OG0031810	Biological Process	biological regulation(GO:0065007)	1
OG0031810	Biological Process	cell proliferation(GO:0008283)	1
OG0031810	Biological Process	cellular component organization or biogenesis(GO:0071840)	1
OG0031810	Biological Process	cellular process(GO:0009987)	1
OG0031810	Biological Process	developmental process(GO:0032502)	1
OG0031810	Biological Process	multicellular organismal process(GO:0032501)	1
OG0031810	Biological Process	regulation of biological process(GO:0050789)	1
OG0031813	Biological Process	cellular component organization or biogenesis(GO:0071840)	1
OG0031813	Biological Process	cellular process(GO:0009987)	1
OG0031813	Biological Process	metabolic process(GO:0008152)	1
OG0031818	Biological Process	cellular component organization or biogenesis(GO:0071840)	1
OG0031818	Biological Process	cellular process(GO:0009987)	1
OG0031818	Biological Process	localization(GO:0051179)	1
OG0031818	Biological Process	metabolic process(GO:0008152)	1
OG0031822	Biological Process	cellular process(GO:0009987)	1

OG0031822	Biological Process	localization(GO:0051179)	1
OG0031822	Biological Process	metabolic process(GO:0008152)	1
OG0031827	Biological Process	biological regulation(GO:0065007)	1
OG0031827	Biological Process	cellular process(GO:0009987)	1
OG0031827	Biological Process	developmental process(GO:0032502)	1
OG0031827	Biological Process	multicellular organismal process(GO:0032501)	1
OG0031827	Biological Process	positive regulation of biological process(GO:0048518)	1
OG0031827	Biological Process	regulation of biological process(GO:0050789)	1
OG0031827	Biological Process	reproduction(GO:0000003)	1
OG0031827	Biological Process	reproductive process(GO:0022414)	1
OG0031827	Biological Process	response to stimulus(GO:0050896)	1
OG0031842	Biological Process	cellular process(GO:0009987)	1
OG0031842	Biological Process	growth(GO:0040007)	1
OG0031842	Biological Process	metabolic process(GO:0008152)	1
OG0031843	Biological Process	cellular process(GO:0009987)	1
OG0031843	Biological Process	metabolic process(GO:0008152)	1
OG0031844	Biological Process	cellular process(GO:0009987)	1
OG0031844	Biological Process	metabolic process(GO:0008152)	1
OG0031849	Biological Process	cellular process(GO:0009987)	1
OG0031849	Biological Process	metabolic process(GO:0008152)	1
OG0031851	Biological Process	cellular process(GO:0009987)	1
OG0031851	Biological Process	growth(GO:0040007)	1
OG0031851	Biological Process	metabolic process(GO:0008152)	1
OG0031855	Biological Process	biological adhesion(GO:0022610)	1
OG0031855	Biological Process	cellular component organization or biogenesis(GO:0071840)	1
OG0031855	Biological Process	cellular process(GO:0009987)	1
OG0031855	Biological Process	growth(GO:0040007)	1
OG0031855	Biological Process	metabolic process(GO:0008152)	1
OG0031855	Biological Process	multi-organism process(GO:0051704)	1
OG0031855	Biological Process	reproduction(GO:0000003)	1
OG0031855	Biological Process	response to stimulus(GO:0050896)	1
OG0031862	Biological Process	cellular process(GO:0009987)	1
OG0031862	Biological Process	metabolic process(GO:0008152)	1
OG0031863	Biological Process	cellular component organization or biogenesis(GO:0071840)	1
OG0031863	Biological Process	cellular process(GO:0009987)	1
OG0031863	Biological Process	metabolic process(GO:0008152)	1
OG0031864	Biological Process	cellular process(GO:0009987)	1
OG0031864	Biological Process	localization(GO:0051179)	1
OG0031864	Biological Process	metabolic process(GO:0008152)	1
OG0031865	Biological Process	cellular component organization or biogenesis(GO:0071840)	1
OG0031865	Biological Process	cellular process(GO:0009987)	1
OG0031865	Biological Process	metabolic process(GO:0008152)	1
OG0031865	Biological Process	response to stimulus(GO:0050896)	1
OG0031870	Biological Process	biological regulation(GO:0065007)	1
OG0031870	Biological Process	cellular process(GO:0009987)	1
OG0031870	Biological Process	metabolic process(GO:0008152)	1

OG0031870	Biological Process	regulation of biological process(GO:0050789)	1
OG0031888	Biological Process	metabolic process(GO:0008152)	1
OG0031889	Biological Process	biological regulation(GO:0065007)	1
OG0031889	Biological Process	cellular component organization or biogenesis(GO:0071840)	1
OG0031889	Biological Process	cellular process(GO:0009987)	1
OG0031889	Biological Process	metabolic process(GO:0008152)	1
OG0031889	Biological Process	multi-organism process(GO:0051704)	1
OG0031889	Biological Process	negative regulation of biological process(GO:0048519)	1
OG0031889	Biological Process	positive regulation of biological process(GO:0048518)	1
OG0031889	Biological Process	regulation of biological process(GO:0050789)	1
OG0031911	Biological Process	response to stimulus(GO:0050896)	1
OG0031919	Biological Process	cellular process(GO:0009987)	1
OG0031919	Biological Process	metabolic process(GO:0008152)	1
OG0031925	Biological Process	cellular process(GO:0009987)	1
OG0031925	Biological Process	metabolic process(GO:0008152)	1
OG0031928	Biological Process	cellular process(GO:0009987)	1
OG0031928	Biological Process	developmental process(GO:0032502)	1
OG0031928	Biological Process	metabolic process(GO:0008152)	1
OG0031928	Biological Process	multicellular organismal process(GO:0032501)	1
OG0031928	Biological Process	response to stimulus(GO:0050896)	1
OG0031929	Biological Process	biological regulation(GO:0065007)	1
OG0031929	Biological Process	cellular process(GO:0009987)	1
OG0031929	Biological Process	metabolic process(GO:0008152)	1
OG0031929	Biological Process	regulation of biological process(GO:0050789)	1
OG0031929	Biological Process	response to stimulus(GO:0050896)	1
OG0031929	Biological Process	signaling(GO:0023052)	1
OG0031931	Biological Process	biological regulation(GO:0065007)	1
OG0031931	Biological Process	cellular process(GO:0009987)	1
OG0031931	Biological Process	developmental process(GO:0032502)	1
OG0031931	Biological Process	metabolic process(GO:0008152)	1
OG0031931	Biological Process	multi-organism process(GO:0051704)	1
OG0031931	Biological Process	multicellular organismal process(GO:0032501)	1
OG0031931	Biological Process	regulation of biological process(GO:0050789)	1
OG0031931	Biological Process	reproduction(GO:0000003)	1
OG0031931	Biological Process	reproductive process(GO:0022414)	1
OG0031931	Biological Process	response to stimulus(GO:0050896)	1
OG0031933	Biological Process	multi-organism process(GO:0051704)	1
OG0031933	Biological Process	response to stimulus(GO:0050896)	1
OG0031937	Biological Process	cellular process(GO:0009987)	1
OG0031937	Biological Process	metabolic process(GO:0008152)	1
OG0031938	Biological Process	biological regulation(GO:0065007)	1
OG0031938	Biological Process	cellular process(GO:0009987)	1
OG0031938	Biological Process	metabolic process(GO:0008152)	1
OG0031938	Biological Process	positive regulation of biological process(GO:0048518)	1
OG0031938	Biological Process	regulation of biological process(GO:0050789)	1

OG0031940	Biological Process	biological regulation(GO:0065007)	1
OG0031940	Biological Process	cellular process(GO:0009987)	1
OG0031940	Biological Process	metabolic process(GO:0008152)	1
OG0031940	Biological Process	positive regulation of biological process(GO:0048518)	1
OG0031940	Biological Process	regulation of biological process(GO:0050789)	1
OG0031943	Biological Process	biological regulation(GO:0065007)	1
OG0031943	Biological Process	cellular process(GO:0009987)	1
OG0031943	Biological Process	metabolic process(GO:0008152)	1
OG0031943	Biological Process	regulation of biological process(GO:0050789)	1
OG0031943	Biological Process	response to stimulus(GO:0050896)	1
OG0031950	Biological Process	cellular component organization or biogenesis(GO:0071840)	1
OG0031950	Biological Process	cellular process(GO:0009987)	1
OG0031950	Biological Process	growth(GO:0040007)	1
OG0031950	Biological Process	metabolic process(GO:0008152)	1
OG0031951	Biological Process	cellular process(GO:0009987)	1
OG0031951	Biological Process	growth(GO:0040007)	1
OG0031951	Biological Process	metabolic process(GO:0008152)	1
OG0031952	Biological Process	cellular process(GO:0009987)	1
OG0031952	Biological Process	growth(GO:0040007)	1
OG0031952	Biological Process	localization(GO:0051179)	1
OG0031952	Biological Process	metabolic process(GO:0008152)	1
OG0031953	Biological Process	cellular component organization or biogenesis(GO:0071840)	1
OG0031953	Biological Process	cellular process(GO:0009987)	1
OG0031953	Biological Process	growth(GO:0040007)	1
OG0031953	Biological Process	metabolic process(GO:0008152)	1
OG0031958	Biological Process	biological regulation(GO:0065007)	1
OG0031958	Biological Process	cellular process(GO:0009987)	1
OG0031958	Biological Process	localization(GO:0051179)	1
OG0031958	Biological Process	metabolic process(GO:0008152)	1
OG0031958	Biological Process	regulation of biological process(GO:0050789)	1
OG0031958	Biological Process	response to stimulus(GO:0050896)	1
OG0031958	Biological Process	signaling(GO:0023052)	1
OG0031959	Biological Process	cellular process(GO:0009987)	1
OG0031959	Biological Process	metabolic process(GO:0008152)	1
OG0031960	Biological Process	cellular process(GO:0009987)	1
OG0031960	Biological Process	metabolic process(GO:0008152)	1
OG0031962	Biological Process	cellular process(GO:0009987)	1
OG0031962	Biological Process	metabolic process(GO:0008152)	1
OG0031963	Biological Process	cellular component organization or biogenesis(GO:0071840)	1
OG0031963	Biological Process	cellular process(GO:0009987)	1
OG0031967	Biological Process	biological regulation(GO:0065007)	1
OG0031967	Biological Process	cellular component organization or biogenesis(GO:0071840)	1
OG0031967	Biological Process	cellular process(GO:0009987)	1
OG0031967	Biological Process	metabolic process(GO:0008152)	1
OG0031967	Biological Process	negative regulation of biological process(GO:0048519)	1

OG0031967	Biological Process	regulation of biological process(GO:0050789)	1
OG0031972	Biological Process	cellular process(GO:0009987)	1
OG0031972	Biological Process	metabolic process(GO:0008152)	1
OG0031972	Biological Process	response to stimulus(GO:0050896)	1
OG0032001	Biological Process	metabolic process(GO:0008152)	1
OG0032021	Biological Process	metabolic process(GO:0008152)	1
OG0032031	Biological Process	metabolic process(GO:0008152)	1
OG0032036	Biological Process	metabolic process(GO:0008152)	1
OG0032036	Biological Process	response to stimulus(GO:0050896)	1
OG0032044	Biological Process	cellular process(GO:0009987)	1
OG0032044	Biological Process	metabolic process(GO:0008152)	1
OG0032045	Biological Process	cellular process(GO:0009987)	1
OG0032045	Biological Process	metabolic process(GO:0008152)	1
OG0032046	Biological Process	cellular process(GO:0009987)	1
OG0032046	Biological Process	metabolic process(GO:0008152)	1
OG0032049	Biological Process	growth(GO:0040007)	1
OG0032050	Biological Process	cellular component organization or biogenesis(GO:0071840)	1
OG0032050	Biological Process	cellular process(GO:0009987)	1
OG0032050	Biological Process	growth(GO:0040007)	1
OG0032050	Biological Process	metabolic process(GO:0008152)	1
OG0032051	Biological Process	cellular process(GO:0009987)	1
OG0032051	Biological Process	metabolic process(GO:0008152)	1
OG0032054	Biological Process	response to stimulus(GO:0050896)	1
OG0032070	Biological Process	metabolic process(GO:0008152)	1
OG0032073	Biological Process	metabolic process(GO:0008152)	1
OG0032074	Biological Process	metabolic process(GO:0008152)	1
OG0032086	Biological Process	cellular process(GO:0009987)	1
OG0032086	Biological Process	developmental process(GO:0032502)	1
OG0032086	Biological Process	multi-organism process(GO:0051704)	1
OG0032086	Biological Process	multicellular organismal process(GO:0032501)	1
OG0032086	Biological Process	reproduction(GO:0000003)	1
OG0032086	Biological Process	reproductive process(GO:0022414)	1
OG0032087	Biological Process	response to stimulus(GO:0050896)	1
OG0032088	Biological Process	response to stimulus(GO:0050896)	1
OG0032092	Biological Process	metabolic process(GO:0008152)	1
OG0032093	Biological Process	response to stimulus(GO:0050896)	1
OG0032095	Biological Process	biological regulation(GO:0065007)	1
OG0032095	Biological Process	cell proliferation(GO:0008283)	1
OG0032095	Biological Process	cellular component organization or biogenesis(GO:0071840)	1
OG0032095	Biological Process	cellular process(GO:0009987)	1
OG0032095	Biological Process	developmental process(GO:0032502)	1
OG0032095	Biological Process	metabolic process(GO:0008152)	1
OG0032095	Biological Process	multicellular organismal process(GO:0032501)	1
OG0032095	Biological Process	positive regulation of biological process(GO:0048518)	1
OG0032095	Biological Process	regulation of biological process(GO:0050789)	1
OG0032095	Biological Process	reproduction(GO:0000003)	1

OG0032095	Biological Process	reproductive process(GO:0022414)	1
OG0032095	Biological Process	response to stimulus(GO:0050896)	1
OG0032096	Biological Process	biological regulation(GO:0065007)	1
OG0032096	Biological Process	cellular process(GO:0009987)	1
OG0032096	Biological Process	metabolic process(GO:0008152)	1
OG0032096	Biological Process	regulation of biological process(GO:0050789)	1
OG0032101	Biological Process	cellular process(GO:0009987)	1
OG0032101	Biological Process	metabolic process(GO:0008152)	1
OG0032102	Biological Process	response to stimulus(GO:0050896)	1
OG0032119	Biological Process	cellular process(GO:0009987)	1
OG0032119	Biological Process	multi-organism process(GO:0051704)	1
OG0032124	Biological Process	response to stimulus(GO:0050896)	1
OG0032126	Biological Process	biological regulation(GO:0065007)	1
OG0032126	Biological Process	cellular process(GO:0009987)	1
OG0032126	Biological Process	developmental process(GO:0032502)	1
OG0032126	Biological Process	metabolic process(GO:0008152)	1
OG0032126	Biological Process	multicellular organismal process(GO:0032501)	1
OG0032126	Biological Process	regulation of biological process(GO:0050789)	1
OG0032126	Biological Process	reproduction(GO:0000003)	1
OG0032126	Biological Process	reproductive process(GO:0022414)	1
OG0032126	Biological Process	response to stimulus(GO:0050896)	1
OG0032134	Biological Process	cellular process(GO:0009987)	1
OG0032134	Biological Process	metabolic process(GO:0008152)	1
OG0032134	Biological Process	response to stimulus(GO:0050896)	1
OG0032142	Biological Process	metabolic process(GO:0008152)	1
OG0032148	Biological Process	biological regulation(GO:0065007)	1
OG0032148	Biological Process	cellular process(GO:0009987)	1
OG0032148	Biological Process	metabolic process(GO:0008152)	1
OG0032148	Biological Process	regulation of biological process(GO:0050789)	1
OG0032148	Biological Process	response to stimulus(GO:0050896)	1
OG0032151	Biological Process	response to stimulus(GO:0050896)	1
OG0032157	Biological Process	cellular process(GO:0009987)	1
OG0032157	Biological Process	developmental process(GO:0032502)	1
OG0032157	Biological Process	growth(GO:0040007)	1
OG0032157	Biological Process	localization(GO:0051179)	1
OG0032157	Biological Process	multicellular organismal process(GO:0032501)	1
OG0032157	Biological Process	reproduction(GO:0000003)	1
OG0032157	Biological Process	reproductive process(GO:0022414)	1
OG0032159	Biological Process	cellular component organization or biogenesis(GO:0071840)	1
OG0032159	Biological Process	cellular process(GO:0009987)	1
OG0032159	Biological Process	metabolic process(GO:0008152)	1
OG0032159	Biological Process	response to stimulus(GO:0050896)	1
OG0032161	Biological Process	cellular process(GO:0009987)	1
OG0032161	Biological Process	metabolic process(GO:0008152)	1
OG0032161	Biological Process	response to stimulus(GO:0050896)	1
OG0032170	Biological Process	response to stimulus(GO:0050896)	1

OG0032183	Biological Process	metabolic process(GO:0008152)	1
OG0032183	Biological Process	multi-organism process(GO:0051704)	1
OG0032183	Biological Process	response to stimulus(GO:0050896)	1
OG0032185	Biological Process	cellular component organization or biogenesis(GO:0071840)	1
OG0032185	Biological Process	cellular process(GO:0009987)	1
OG0032185	Biological Process	growth(GO:0040007)	1
OG0032185	Biological Process	metabolic process(GO:0008152)	1
OG0032186	Biological Process	biological regulation(GO:0065007)	1
OG0032186	Biological Process	cellular component organization or biogenesis(GO:0071840)	1
OG0032186	Biological Process	cellular process(GO:0009987)	1
OG0032186	Biological Process	metabolic process(GO:0008152)	1
OG0032186	Biological Process	regulation of biological process(GO:0050789)	1
OG0032186	Biological Process	response to stimulus(GO:0050896)	1
OG0032191	Biological Process	cellular process(GO:0009987)	1
OG0032191	Biological Process	localization(GO:0051179)	1
OG0032191	Biological Process	metabolic process(GO:0008152)	1
OG0032191	Biological Process	multi-organism process(GO:0051704)	1
OG0032196	Biological Process	cellular component organization or biogenesis(GO:0071840)	1
OG0032196	Biological Process	cellular process(GO:0009987)	1
OG0032196	Biological Process	developmental process(GO:0032502)	1
OG0032196	Biological Process	metabolic process(GO:0008152)	1
OG0032196	Biological Process	multicellular organismal process(GO:0032501)	1
OG0032201	Biological Process	response to stimulus(GO:0050896)	1
OG0032220	Biological Process	biological regulation(GO:0065007)	1
OG0032220	Biological Process	cellular process(GO:0009987)	1
OG0032220	Biological Process	metabolic process(GO:0008152)	1
OG0032220	Biological Process	positive regulation of biological process(GO:0048518)	1
OG0032220	Biological Process	regulation of biological process(GO:0050789)	1
OG0032220	Biological Process	response to stimulus(GO:0050896)	1
OG0032220	Biological Process	rhythmic process(GO:0048511)	1
OG0032220	Biological Process	signaling(GO:0023052)	1
OG0032222	Biological Process	biological regulation(GO:0065007)	1
OG0032222	Biological Process	cellular component organization or biogenesis(GO:0071840)	1
OG0032222	Biological Process	cellular process(GO:0009987)	1
OG0032222	Biological Process	metabolic process(GO:0008152)	1
OG0032222	Biological Process	negative regulation of biological process(GO:0048519)	1
OG0032222	Biological Process	regulation of biological process(GO:0050789)	1
OG0032223	Biological Process	cellular process(GO:0009987)	1
OG0032223	Biological Process	localization(GO:0051179)	1
OG0032223	Biological Process	response to stimulus(GO:0050896)	1
OG0032224	Biological Process	biological regulation(GO:0065007)	1
OG0032224	Biological Process	cellular process(GO:0009987)	1
OG0032224	Biological Process	developmental process(GO:0032502)	1
OG0032224	Biological Process	metabolic process(GO:0008152)	1
OG0032224	Biological Process	multicellular organismal process(GO:0032501)	1

OG0032224	Biological Process	negative regulation of biological process(GO:0048519)	1
OG0032224	Biological Process	regulation of biological process(GO:0050789)	1
OG0032224	Biological Process	reproduction(GO:0000003)	1
OG0032224	Biological Process	reproductive process(GO:0022414)	1
OG0032224	Biological Process	response to stimulus(GO:0050896)	1
OG0032230	Biological Process	biological regulation(GO:0065007)	1
OG0032230	Biological Process	cellular process(GO:0009987)	1
OG0032230	Biological Process	developmental process(GO:0032502)	1
OG0032230	Biological Process	metabolic process(GO:0008152)	1
OG0032230	Biological Process	negative regulation of biological process(GO:0048519)	1
OG0032230	Biological Process	regulation of biological process(GO:0050789)	1
OG0032232	Biological Process	biological regulation(GO:0065007)	1
OG0032232	Biological Process	cellular process(GO:0009987)	1
OG0032232	Biological Process	metabolic process(GO:0008152)	1
OG0032232	Biological Process	positive regulation of biological process(GO:0048518)	1
OG0032232	Biological Process	regulation of biological process(GO:0050789)	1
OG0032232	Biological Process	response to stimulus(GO:0050896)	1
OG0032234	Biological Process	biological regulation(GO:0065007)	1
OG0032234	Biological Process	cellular process(GO:0009987)	1
OG0032234	Biological Process	metabolic process(GO:0008152)	1
OG0032234	Biological Process	positive regulation of biological process(GO:0048518)	1
OG0032234	Biological Process	regulation of biological process(GO:0050789)	1
OG0032234	Biological Process	response to stimulus(GO:0050896)	1
OG0032236	Biological Process	biological regulation(GO:0065007)	1
OG0032236	Biological Process	cellular component organization or biogenesis(GO:0071840)	1
OG0032236	Biological Process	cellular process(GO:0009987)	1
OG0032236	Biological Process	metabolic process(GO:0008152)	1
OG0032236	Biological Process	regulation of biological process(GO:0050789)	1
OG0032245	Biological Process	biological regulation(GO:0065007)	1
OG0032245	Biological Process	cellular process(GO:0009987)	1
OG0032245	Biological Process	metabolic process(GO:0008152)	1
OG0032245	Biological Process	regulation of biological process(GO:0050789)	1
OG0032252	Biological Process	biological regulation(GO:0065007)	1
OG0032252	Biological Process	cellular process(GO:0009987)	1
OG0032252	Biological Process	developmental process(GO:0032502)	1
OG0032252	Biological Process	metabolic process(GO:0008152)	1
OG0032252	Biological Process	multicellular organismal process(GO:0032501)	1
OG0032252	Biological Process	regulation of biological process(GO:0050789)	1
OG0032252	Biological Process	response to stimulus(GO:0050896)	1
OG0032253	Biological Process	cellular process(GO:0009987)	1
OG0032253	Biological Process	metabolic process(GO:0008152)	1
OG0032254	Biological Process	cellular process(GO:0009987)	1
OG0032254	Biological Process	metabolic process(GO:0008152)	1
OG0032256	Biological Process	cellular process(GO:0009987)	1

OG0032256	Biological Process	metabolic process(GO:0008152)	1
OG0032256	Biological Process	response to stimulus(GO:0050896)	1
OG0032262	Biological Process	cellular process(GO:0009987)	1
OG0032262	Biological Process	metabolic process(GO:0008152)	1
OG0032264	Biological Process	cellular process(GO:0009987)	1
OG0032264	Biological Process	developmental process(GO:0032502)	1
OG0032264	Biological Process	growth(GO:0040007)	1
OG0032264	Biological Process	metabolic process(GO:0008152)	1
OG0032264	Biological Process	multicellular organismal process(GO:0032501)	1
OG0032270	Biological Process	developmental process(GO:0032502)	1
OG0032273	Biological Process	cellular process(GO:0009987)	1
OG0032273	Biological Process	metabolic process(GO:0008152)	1
OG0032278	Biological Process	biological regulation(GO:0065007)	1
OG0032278	Biological Process	cellular process(GO:0009987)	1
OG0032278	Biological Process	metabolic process(GO:0008152)	1
OG0032278	Biological Process	positive regulation of biological process(GO:0048518)	1
OG0032278	Biological Process	regulation of biological process(GO:0050789)	1
OG0032278	Biological Process	response to stimulus(GO:0050896)	1
OG0032289	Biological Process	cellular component organization or biogenesis(GO:0071840)	1
OG0032289	Biological Process	cellular process(GO:0009987)	1
OG0032289	Biological Process	metabolic process(GO:0008152)	1
OG0032290	Biological Process	cellular component organization or biogenesis(GO:0071840)	1
OG0032290	Biological Process	cellular process(GO:0009987)	1
OG0032290	Biological Process	metabolic process(GO:0008152)	1
OG0032297	Biological Process	biological regulation(GO:0065007)	1
OG0032297	Biological Process	cellular component organization or biogenesis(GO:0071840)	1
OG0032297	Biological Process	cellular process(GO:0009987)	1
OG0032297	Biological Process	developmental process(GO:0032502)	1
OG0032297	Biological Process	immune system process(GO:0002376)	1
OG0032297	Biological Process	metabolic process(GO:0008152)	1
OG0032297	Biological Process	multi-organism process(GO:0051704)	1
OG0032297	Biological Process	multicellular organismal process(GO:0032501)	1
OG0032297	Biological Process	negative regulation of biological process(GO:0048519)	1
OG0032297	Biological Process	positive regulation of biological process(GO:0048518)	1
OG0032297	Biological Process	regulation of biological process(GO:0050789)	1
OG0032297	Biological Process	reproduction(GO:0000003)	1
OG0032297	Biological Process	reproductive process(GO:0022414)	1
OG0032297	Biological Process	response to stimulus(GO:0050896)	1
OG0032297	Biological Process	signaling(GO:0023052)	1
OG0032305	Biological Process	biological regulation(GO:0065007)	1
OG0032305	Biological Process	cell proliferation(GO:0008283)	1
OG0032305	Biological Process	cellular component organization or biogenesis(GO:0071840)	1
OG0032305	Biological Process	cellular process(GO:0009987)	1
OG0032305	Biological Process	developmental process(GO:0032502)	1

OG0032305	Biological Process	multicellular organismal process(GO:0032501)	1
OG0032305	Biological Process	regulation of biological process(GO:0050789)	1
OG0032315	Biological Process	biological regulation(GO:0065007)	1
OG0032315	Biological Process	cellular process(GO:0009987)	1
OG0032315	Biological Process	metabolic process(GO:0008152)	1
OG0032315	Biological Process	regulation of biological process(GO:0050789)	1
OG0032321	Biological Process	biological regulation(GO:0065007)	1
OG0032321	Biological Process	cellular process(GO:0009987)	1
OG0032321	Biological Process	immune system process(GO:0002376)	1
OG0032321	Biological Process	multi-organism process(GO:0051704)	1
OG0032321	Biological Process	positive regulation of biological process(GO:0048518)	1
OG0032321	Biological Process	regulation of biological process(GO:0050789)	1
OG0032321	Biological Process	response to stimulus(GO:0050896)	1
OG0032321	Biological Process	signaling(GO:0023052)	1
OG0032328	Biological Process	cellular process(GO:0009987)	1
OG0032328	Biological Process	metabolic process(GO:0008152)	1
OG0032328	Biological Process	response to stimulus(GO:0050896)	1
OG0032335	Biological Process	response to stimulus(GO:0050896)	1
OG0032342	Biological Process	biological regulation(GO:0065007)	1
OG0032342	Biological Process	cellular process(GO:0009987)	1
OG0032342	Biological Process	metabolic process(GO:0008152)	1
OG0032342	Biological Process	negative regulation of biological process(GO:0048519)	1
OG0032342	Biological Process	positive regulation of biological process(GO:0048518)	1
OG0032342	Biological Process	regulation of biological process(GO:0050789)	1
OG0032342	Biological Process	response to stimulus(GO:0050896)	1
OG0032344	Biological Process	biological regulation(GO:0065007)	1
OG0032344	Biological Process	cellular process(GO:0009987)	1
OG0032344	Biological Process	metabolic process(GO:0008152)	1
OG0032344	Biological Process	regulation of biological process(GO:0050789)	1
OG0032344	Biological Process	response to stimulus(GO:0050896)	1
OG0032345	Biological Process	cellular process(GO:0009987)	1
OG0032345	Biological Process	metabolic process(GO:0008152)	1
OG0032345	Biological Process	response to stimulus(GO:0050896)	1
OG0032350	Biological Process	biological regulation(GO:0065007)	1
OG0032350	Biological Process	cellular process(GO:0009987)	1
OG0032350	Biological Process	metabolic process(GO:0008152)	1
OG0032350	Biological Process	negative regulation of biological process(GO:0048519)	1
OG0032350	Biological Process	regulation of biological process(GO:0050789)	1
OG0032372	Biological Process	cellular process(GO:0009987)	1
OG0032372	Biological Process	localization(GO:0051179)	1
OG0032372	Biological Process	response to stimulus(GO:0050896)	1
OG0032381	Biological Process	response to stimulus(GO:0050896)	1
OG0032384	Biological Process	metabolic process(GO:0008152)	1
OG0032388	Biological Process	cellular process(GO:0009987)	1
OG0032388	Biological Process	metabolic process(GO:0008152)	1

OG0032388	Biological Process	multi-organism process(GO:0051704)	1
OG0032388	Biological Process	response to stimulus(GO:0050896)	1
OG0032403	Biological Process	biological regulation(GO:0065007)	1
OG0032403	Biological Process	cellular component organization or biogenesis(GO:0071840)	1
OG0032403	Biological Process	cellular process(GO:0009987)	1
OG0032403	Biological Process	developmental process(GO:0032502)	1
OG0032403	Biological Process	growth(GO:0040007)	1
OG0032403	Biological Process	immune system process(GO:0002376)	1
OG0032403	Biological Process	localization(GO:0051179)	1
OG0032403	Biological Process	locomotion(GO:0040011)	1
OG0032403	Biological Process	metabolic process(GO:0008152)	1
OG0032403	Biological Process	multi-organism process(GO:0051704)	1
OG0032403	Biological Process	multicellular organismal process(GO:0032501)	1
OG0032403	Biological Process	negative regulation of biological process(GO:0048519)	1
OG0032403	Biological Process	positive regulation of biological process(GO:0048518)	1
OG0032403	Biological Process	regulation of biological process(GO:0050789)	1
OG0032403	Biological Process	reproduction(GO:0000003)	1
OG0032403	Biological Process	reproductive process(GO:0022414)	1
OG0032408	Biological Process	cellular process(GO:0009987)	1
OG0032408	Biological Process	metabolic process(GO:0008152)	1
OG0032408	Biological Process	response to stimulus(GO:0050896)	1
OG0032413	Biological Process	biological regulation(GO:0065007)	1
OG0032413	Biological Process	cellular process(GO:0009987)	1
OG0032413	Biological Process	developmental process(GO:0032502)	1
OG0032413	Biological Process	metabolic process(GO:0008152)	1
OG0032413	Biological Process	multicellular organismal process(GO:0032501)	1
OG0032413	Biological Process	regulation of biological process(GO:0050789)	1
OG0032413	Biological Process	response to stimulus(GO:0050896)	1
OG0032414	Biological Process	biological regulation(GO:0065007)	1
OG0032414	Biological Process	cellular process(GO:0009987)	1
OG0032414	Biological Process	metabolic process(GO:0008152)	1
OG0032415	Biological Process	cellular process(GO:0009987)	1
OG0032415	Biological Process	localization(GO:0051179)	1
OG0032415	Biological Process	response to stimulus(GO:0050896)	1
OG0032426	Biological Process	cellular process(GO:0009987)	1
OG0032426	Biological Process	metabolic process(GO:0008152)	1
OG0032426	Biological Process	response to stimulus(GO:0050896)	1
OG0032431	Biological Process	biological regulation(GO:0065007)	1
OG0032431	Biological Process	cellular process(GO:0009987)	1
OG0032431	Biological Process	developmental process(GO:0032502)	1
OG0032431	Biological Process	metabolic process(GO:0008152)	1
OG0032431	Biological Process	multicellular organismal process(GO:0032501)	1
OG0032433	Biological Process	biological regulation(GO:0065007)	1
OG0032433	Biological Process	cellular process(GO:0009987)	1
OG0032433	Biological Process	metabolic process(GO:0008152)	1

OG0032433	Biological Process	positive regulation of biological process(GO:0048518)	1
OG0032433	Biological Process	regulation of biological process(GO:0050789)	1
OG0032433	Biological Process	response to stimulus(GO:0050896)	1
OG0032433	Biological Process	signaling(GO:0023052)	1
OG0032447	Biological Process	cellular component organization or biogenesis(GO:0071840)	1
OG0032447	Biological Process	cellular process(GO:0009987)	1
OG0032447	Biological Process	metabolic process(GO:0008152)	1
OG0032449	Biological Process	cellular process(GO:0009987)	1
OG0032449	Biological Process	metabolic process(GO:0008152)	1
OG0032452	Biological Process	cellular process(GO:0009987)	1
OG0032452	Biological Process	localization(GO:0051179)	1
OG0032452	Biological Process	metabolic process(GO:0008152)	1
OG0032453	Biological Process	cellular process(GO:0009987)	1
OG0032453	Biological Process	metabolic process(GO:0008152)	1
OG0032459	Biological Process	cellular process(GO:0009987)	1
OG0032459	Biological Process	metabolic process(GO:0008152)	1
OG0032464	Biological Process	biological regulation(GO:0065007)	1
OG0032464	Biological Process	cellular component organization or biogenesis(GO:0071840)	1
OG0032464	Biological Process	cellular process(GO:0009987)	1
OG0032464	Biological Process	localization(GO:0051179)	1
OG0032464	Biological Process	metabolic process(GO:0008152)	1
OG0032464	Biological Process	multicellular organismal process(GO:0032501)	1
OG0032464	Biological Process	negative regulation of biological process(GO:0048519)	1
OG0032464	Biological Process	regulation of biological process(GO:0050789)	1
OG0032464	Biological Process	response to stimulus(GO:0050896)	1
OG0032464	Biological Process	signaling(GO:0023052)	1
OG0032466	Biological Process	cellular component organization or biogenesis(GO:0071840)	1
OG0032466	Biological Process	cellular process(GO:0009987)	1
OG0032466	Biological Process	metabolic process(GO:0008152)	1
OG0032473	Biological Process	biological regulation(GO:0065007)	1
OG0032473	Biological Process	cellular process(GO:0009987)	1
OG0032473	Biological Process	metabolic process(GO:0008152)	1
OG0032473	Biological Process	negative regulation of biological process(GO:0048519)	1
OG0032473	Biological Process	regulation of biological process(GO:0050789)	1
OG0032473	Biological Process	response to stimulus(GO:0050896)	1
OG0032474	Biological Process	cellular component organization or biogenesis(GO:0071840)	1
OG0032474	Biological Process	cellular process(GO:0009987)	1
OG0032474	Biological Process	developmental process(GO:0032502)	1
OG0032474	Biological Process	metabolic process(GO:0008152)	1
OG0032474	Biological Process	multicellular organismal process(GO:0032501)	1
OG0032474	Biological Process	reproduction(GO:0000003)	1
OG0032491	Biological Process	cellular component organization or biogenesis(GO:0071840)	1
OG0032491	Biological Process	cellular process(GO:0009987)	1
OG0032491	Biological Process	growth(GO:0040007)	1

OG0032491	Biological Process	metabolic process(GO:0008152)	1
OG0032492	Biological Process	cellular process(GO:0009987)	1
OG0032492	Biological Process	growth(GO:0040007)	1
OG0032492	Biological Process	metabolic process(GO:0008152)	1
OG0032498	Biological Process	cellular process(GO:0009987)	1
OG0032498	Biological Process	detoxification(GO:0098754)	1
OG0032498	Biological Process	metabolic process(GO:0008152)	1
OG0032498	Biological Process	response to stimulus(GO:0050896)	1
OG0032501	Biological Process	cellular process(GO:0009987)	1
OG0032501	Biological Process	metabolic process(GO:0008152)	1
OG0032503	Biological Process	biological regulation(GO:0065007)	1
OG0032503	Biological Process	cellular process(GO:0009987)	1
OG0032503	Biological Process	regulation of biological process(GO:0050789)	1
OG0032503	Biological Process	response to stimulus(GO:0050896)	1
OG0032503	Biological Process	signaling(GO:0023052)	1
OG0032507	Biological Process	biological regulation(GO:0065007)	1
OG0032507	Biological Process	cellular process(GO:0009987)	1
OG0032507	Biological Process	developmental process(GO:0032502)	1
OG0032507	Biological Process	metabolic process(GO:0008152)	1
OG0032507	Biological Process	multi-organism process(GO:0051704)	1
OG0032507	Biological Process	multicellular organismal process(GO:0032501)	1
OG0032507	Biological Process	regulation of biological process(GO:0050789)	1
OG0032507	Biological Process	reproduction(GO:0000003)	1
OG0032507	Biological Process	reproductive process(GO:0022414)	1
OG0032507	Biological Process	response to stimulus(GO:0050896)	1
OG0032530	Biological Process	cellular process(GO:0009987)	1
OG0032530	Biological Process	localization(GO:0051179)	1
OG0032530	Biological Process	metabolic process(GO:0008152)	1
OG0032548	Biological Process	multi-organism process(GO:0051704)	1
OG0032548	Biological Process	multicellular organismal process(GO:0032501)	1
OG0032548	Biological Process	reproduction(GO:0000003)	1
OG0032548	Biological Process	reproductive process(GO:0022414)	1
OG0032551	Biological Process	biological regulation(GO:0065007)	1
OG0032551	Biological Process	cellular component organization or biogenesis(GO:0071840)	1
OG0032551	Biological Process	cellular process(GO:0009987)	1
OG0032551	Biological Process	developmental process(GO:0032502)	1
OG0032551	Biological Process	regulation of biological process(GO:0050789)	1
OG0032551	Biological Process	response to stimulus(GO:0050896)	1
OG0032561	Biological Process	cellular process(GO:0009987)	1
OG0032561	Biological Process	developmental process(GO:0032502)	1
OG0032561	Biological Process	metabolic process(GO:0008152)	1
OG0032561	Biological Process	multicellular organismal process(GO:0032501)	1
OG0032561	Biological Process	reproduction(GO:0000003)	1
OG0032561	Biological Process	reproductive process(GO:0022414)	1
OG0032562	Biological Process	cellular component organization or biogenesis(GO:0071840)	1

OG0032562	Biological Process	cellular process(GO:0009987)	1
OG0032562	Biological Process	metabolic process(GO:0008152)	1
OG0032572	Biological Process	growth(GO:0040007)	1
OG0032574	Biological Process	metabolic process(GO:0008152)	1
OG0032588	Biological Process	developmental process(GO:0032502)	1
OG0032588	Biological Process	multicellular organismal process(GO:0032501)	1
OG0032588	Biological Process	reproduction(GO:0000003)	1
OG0032588	Biological Process	reproductive process(GO:0022414)	1
OG0032588	Biological Process	response to stimulus(GO:0050896)	1
OG0032590	Biological Process	cellular process(GO:0009987)	1
OG0032590	Biological Process	developmental process(GO:0032502)	1
OG0032590	Biological Process	multi-organism process(GO:0051704)	1
OG0032590	Biological Process	multicellular organismal process(GO:0032501)	1
OG0032590	Biological Process	reproduction(GO:0000003)	1
OG0032590	Biological Process	reproductive process(GO:0022414)	1
OG0032596	Biological Process	cellular component organization or biogenesis(GO:0071840)	1
OG0032596	Biological Process	cellular process(GO:0009987)	1
OG0032596	Biological Process	developmental process(GO:0032502)	1
OG0032596	Biological Process	growth(GO:0040007)	1
OG0032596	Biological Process	metabolic process(GO:0008152)	1
OG0032596	Biological Process	response to stimulus(GO:0050896)	1
OG0032598	Biological Process	cellular process(GO:0009987)	1
OG0032598	Biological Process	metabolic process(GO:0008152)	1
OG0032616	Biological Process	response to stimulus(GO:0050896)	1
OG0032626	Biological Process	cellular process(GO:0009987)	1
OG0032626	Biological Process	metabolic process(GO:0008152)	1
OG0032626	Biological Process	response to stimulus(GO:0050896)	1
OG0032627	Biological Process	biological regulation(GO:0065007)	1
OG0032627	Biological Process	cellular process(GO:0009987)	1
OG0032627	Biological Process	metabolic process(GO:0008152)	1
OG0032627	Biological Process	negative regulation of biological process(GO:0048519)	1
OG0032627	Biological Process	regulation of biological process(GO:0050789)	1
OG0032637	Biological Process	growth(GO:0040007)	1
OG0032638	Biological Process	metabolic process(GO:0008152)	1
OG0032639	Biological Process	growth(GO:0040007)	1
OG0032656	Biological Process	cellular process(GO:0009987)	1
OG0032656	Biological Process	metabolic process(GO:0008152)	1
OG0032662	Biological Process	cellular process(GO:0009987)	1
OG0032662	Biological Process	metabolic process(GO:0008152)	1
OG0032666	Biological Process	cellular process(GO:0009987)	1
OG0032666	Biological Process	growth(GO:0040007)	1
OG0032666	Biological Process	metabolic process(GO:0008152)	1
OG0032671	Biological Process	cellular process(GO:0009987)	1
OG0032671	Biological Process	metabolic process(GO:0008152)	1
OG0032673	Biological Process	cellular process(GO:0009987)	1
OG0032673	Biological Process	metabolic process(GO:0008152)	1

OG0032674	Biological Process	cellular process(GO:0009987)	1
OG0032674	Biological Process	metabolic process(GO:0008152)	1
OG0032681	Biological Process	cellular process(GO:0009987)	1
OG0032681	Biological Process	metabolic process(GO:0008152)	1
OG0032681	Biological Process	response to stimulus(GO:0050896)	1
OG0032686	Biological Process	cellular component organization or biogenesis(GO:0071840)	1
OG0032686	Biological Process	cellular process(GO:0009987)	1
OG0032693	Biological Process	metabolic process(GO:0008152)	1
OG0032694	Biological Process	metabolic process(GO:0008152)	1
OG0032700	Biological Process	cellular process(GO:0009987)	1
OG0032700	Biological Process	metabolic process(GO:0008152)	1
OG0032701	Biological Process	cellular process(GO:0009987)	1
OG0032701	Biological Process	localization(GO:0051179)	1
OG0032701	Biological Process	metabolic process(GO:0008152)	1
OG0032702	Biological Process	cellular process(GO:0009987)	1
OG0032702	Biological Process	localization(GO:0051179)	1
OG0032702	Biological Process	metabolic process(GO:0008152)	1
OG0032702	Biological Process	multi-organism process(GO:0051704)	1
OG0032704	Biological Process	cellular process(GO:0009987)	1
OG0032704	Biological Process	metabolic process(GO:0008152)	1
OG0032709	Biological Process	cellular process(GO:0009987)	1
OG0032709	Biological Process	metabolic process(GO:0008152)	1
OG0032710	Biological Process	biological regulation(GO:0065007)	1
OG0032710	Biological Process	cellular component organization or biogenesis(GO:0071840)	1
OG0032710	Biological Process	cellular process(GO:0009987)	1
OG0032710	Biological Process	metabolic process(GO:0008152)	1
OG0032710	Biological Process	regulation of biological process(GO:0050789)	1
OG0032710	Biological Process	response to stimulus(GO:0050896)	1
OG0032711	Biological Process	cellular process(GO:0009987)	1
OG0032711	Biological Process	metabolic process(GO:0008152)	1
OG0032714	Biological Process	cellular process(GO:0009987)	1
OG0032714	Biological Process	metabolic process(GO:0008152)	1
OG0032719	Biological Process	cellular process(GO:0009987)	1
OG0032719	Biological Process	metabolic process(GO:0008152)	1
OG0032719	Biological Process	response to stimulus(GO:0050896)	1
OG0032722	Biological Process	cellular process(GO:0009987)	1
OG0032722	Biological Process	growth(GO:0040007)	1
OG0032722	Biological Process	metabolic process(GO:0008152)	1
OG0032725	Biological Process	cellular process(GO:0009987)	1
OG0032725	Biological Process	metabolic process(GO:0008152)	1
OG0032727	Biological Process	cellular process(GO:0009987)	1
OG0032727	Biological Process	metabolic process(GO:0008152)	1
OG0032730	Biological Process	cellular process(GO:0009987)	1
OG0032730	Biological Process	metabolic process(GO:0008152)	1
OG0032745	Biological Process	cellular process(GO:0009987)	1
OG0032745	Biological Process	growth(GO:0040007)	1

OG0032745	Biological Process	metabolic process(GO:0008152)	1
OG0032745	Biological Process	response to stimulus(GO:0050896)	1
OG0032747	Biological Process	cellular process(GO:0009987)	1
OG0032747	Biological Process	metabolic process(GO:0008152)	1
OG0032751	Biological Process	biological regulation(GO:0065007)	1
OG0032751	Biological Process	cellular process(GO:0009987)	1
OG0032751	Biological Process	metabolic process(GO:0008152)	1
OG0032751	Biological Process	negative regulation of biological process(GO:0048519)	1
OG0032751	Biological Process	regulation of biological process(GO:0050789)	1
OG0032752	Biological Process	growth(GO:0040007)	1
OG0032756	Biological Process	growth(GO:0040007)	1
OG0032757	Biological Process	cellular process(GO:0009987)	1
OG0032757	Biological Process	growth(GO:0040007)	1
OG0032757	Biological Process	metabolic process(GO:0008152)	1
OG0032759	Biological Process	cellular process(GO:0009987)	1
OG0032759	Biological Process	growth(GO:0040007)	1
OG0032759	Biological Process	metabolic process(GO:0008152)	1
OG0032760	Biological Process	cellular component organization or biogenesis(GO:0071840)	1
OG0032760	Biological Process	cellular process(GO:0009987)	1
OG0032766	Biological Process	cellular process(GO:0009987)	1
OG0032766	Biological Process	growth(GO:0040007)	1
OG0032766	Biological Process	metabolic process(GO:0008152)	1
OG0032778	Biological Process	biological regulation(GO:0065007)	1
OG0032778	Biological Process	cellular process(GO:0009987)	1
OG0032778	Biological Process	regulation of biological process(GO:0050789)	1
OG0032779	Biological Process	biological regulation(GO:0065007)	1
OG0032779	Biological Process	cellular process(GO:0009987)	1
OG0032779	Biological Process	metabolic process(GO:0008152)	1
OG0032779	Biological Process	regulation of biological process(GO:0050789)	1
OG0032780	Biological Process	biological regulation(GO:0065007)	1
OG0032780	Biological Process	cellular process(GO:0009987)	1
OG0032780	Biological Process	metabolic process(GO:0008152)	1
OG0032780	Biological Process	multi-organism process(GO:0051704)	1
OG0032780	Biological Process	regulation of biological process(GO:0050789)	1
OG0032780	Biological Process	response to stimulus(GO:0050896)	1
OG0032780	Biological Process	signaling(GO:0023052)	1
OG0032787	Biological Process	response to stimulus(GO:0050896)	1
OG0032794	Biological Process	cellular process(GO:0009987)	1
OG0032794	Biological Process	metabolic process(GO:0008152)	1
OG0032795	Biological Process	cellular process(GO:0009987)	1
OG0032795	Biological Process	metabolic process(GO:0008152)	1
OG0032799	Biological Process	response to stimulus(GO:0050896)	1
OG0032800	Biological Process	cellular process(GO:0009987)	1
OG0032800	Biological Process	signaling(GO:0023052)	1
OG0032801	Biological Process	cellular process(GO:0009987)	1
OG0032801	Biological Process	localization(GO:0051179)	1

OG0032801	Biological Process	locomotion(GO:0040011)	1
OG0032802	Biological Process	cellular component organization or biogenesis(GO:0071840)	1
OG0032802	Biological Process	cellular process(GO:0009987)	1
OG0032802	Biological Process	growth(GO:0040007)	1
OG0032802	Biological Process	metabolic process(GO:0008152)	1
OG0032803	Biological Process	biological regulation(GO:0065007)	1
OG0032803	Biological Process	cellular process(GO:0009987)	1
OG0032803	Biological Process	metabolic process(GO:0008152)	1
OG0032803	Biological Process	regulation of biological process(GO:0050789)	1
OG0032806	Biological Process	cellular process(GO:0009987)	1
OG0032806	Biological Process	growth(GO:0040007)	1
OG0032806	Biological Process	metabolic process(GO:0008152)	1
OG0032806	Biological Process	response to stimulus(GO:0050896)	1
OG0032807	Biological Process	cellular process(GO:0009987)	1
OG0032807	Biological Process	metabolic process(GO:0008152)	1
OG0032808	Biological Process	cellular component organization or biogenesis(GO:0071840)	1
OG0032808	Biological Process	cellular process(GO:0009987)	1
OG0032808	Biological Process	growth(GO:0040007)	1
OG0032808	Biological Process	metabolic process(GO:0008152)	1
OG0032819	Biological Process	biological regulation(GO:0065007)	1
OG0032819	Biological Process	cellular process(GO:0009987)	1
OG0032819	Biological Process	developmental process(GO:0032502)	1
OG0032819	Biological Process	immune system process(GO:0002376)	1
OG0032819	Biological Process	metabolic process(GO:0008152)	1
OG0032819	Biological Process	multi-organism process(GO:0051704)	1
OG0032819	Biological Process	multicellular organismal process(GO:0032501)	1
OG0032819	Biological Process	regulation of biological process(GO:0050789)	1
OG0032819	Biological Process	response to stimulus(GO:0050896)	1
OG0032819	Biological Process	signaling(GO:0023052)	1
OG0032822	Biological Process	cellular process(GO:0009987)	1
OG0032822	Biological Process	metabolic process(GO:0008152)	1
OG0032824	Biological Process	biological regulation(GO:0065007)	1
OG0032824	Biological Process	cellular process(GO:0009987)	1
OG0032824	Biological Process	metabolic process(GO:0008152)	1
OG0032824	Biological Process	multicellular organismal process(GO:0032501)	1
OG0032824	Biological Process	regulation of biological process(GO:0050789)	1
OG0032824	Biological Process	response to stimulus(GO:0050896)	1
OG0032829	Biological Process	metabolic process(GO:0008152)	1
OG0032830	Biological Process	biological regulation(GO:0065007)	1
OG0032830	Biological Process	cellular component organization or biogenesis(GO:0071840)	1
OG0032830	Biological Process	cellular process(GO:0009987)	1
OG0032830	Biological Process	metabolic process(GO:0008152)	1
OG0032830	Biological Process	negative regulation of biological process(GO:0048519)	1
OG0032830	Biological Process	regulation of biological process(GO:0050789)	1
OG0032830	Biological Process	response to stimulus(GO:0050896)	1

OG0032830	Biological Process	signaling(GO:0023052)	1
OG0032847	Biological Process	response to stimulus(GO:0050896)	1
OG0032848	Biological Process	multi-organism process(GO:0051704)	1
OG0032848	Biological Process	response to stimulus(GO:0050896)	1
OG0032850	Biological Process	response to stimulus(GO:0050896)	1
OG0032852	Biological Process	biological regulation(GO:0065007)	1
OG0032852	Biological Process	cellular process(GO:0009987)	1
OG0032852	Biological Process	metabolic process(GO:0008152)	1
OG0032852	Biological Process	regulation of biological process(GO:0050789)	1
OG0032855	Biological Process	cellular process(GO:0009987)	1
OG0032855	Biological Process	metabolic process(GO:0008152)	1
OG0032856	Biological Process	localization(GO:0051179)	1
OG0032857	Biological Process	cellular process(GO:0009987)	1
OG0032857	Biological Process	metabolic process(GO:0008152)	1
OG0032857	Biological Process	multi-organism process(GO:0051704)	1
OG0032857	Biological Process	response to stimulus(GO:0050896)	1
OG0032863	Biological Process	cellular process(GO:0009987)	1
OG0032863	Biological Process	localization(GO:0051179)	1
OG0032868	Biological Process	biological regulation(GO:0065007)	1
OG0032868	Biological Process	cellular process(GO:0009987)	1
OG0032868	Biological Process	developmental process(GO:0032502)	1
OG0032868	Biological Process	metabolic process(GO:0008152)	1
OG0032868	Biological Process	multicellular organismal process(GO:0032501)	1
OG0032868	Biological Process	regulation of biological process(GO:0050789)	1
OG0032868	Biological Process	response to stimulus(GO:0050896)	1
OG0032871	Biological Process	developmental process(GO:0032502)	1
OG0032881	Biological Process	biological regulation(GO:0065007)	1
OG0032881	Biological Process	cellular process(GO:0009987)	1
OG0032881	Biological Process	immune system process(GO:0002376)	1
OG0032881	Biological Process	multi-organism process(GO:0051704)	1
OG0032881	Biological Process	positive regulation of biological process(GO:0048518)	1
OG0032881	Biological Process	regulation of biological process(GO:0050789)	1
OG0032881	Biological Process	response to stimulus(GO:0050896)	1
OG0032881	Biological Process	signaling(GO:0023052)	1
OG0032885	Biological Process	cellular process(GO:0009987)	1
OG0032885	Biological Process	metabolic process(GO:0008152)	1
OG0032886	Biological Process	developmental process(GO:0032502)	1
OG0032886	Biological Process	immune system process(GO:0002376)	1
OG0032886	Biological Process	multi-organism process(GO:0051704)	1
OG0032886	Biological Process	multicellular organismal process(GO:0032501)	1
OG0032886	Biological Process	response to stimulus(GO:0050896)	1
OG0032887	Biological Process	cellular process(GO:0009987)	1
OG0032887	Biological Process	metabolic process(GO:0008152)	1
OG0032889	Biological Process	cellular process(GO:0009987)	1
OG0032889	Biological Process	localization(GO:0051179)	1
OG0032891	Biological Process	metabolic process(GO:0008152)	1

OG0032896	Biological Process	cellular process(GO:0009987)	1
OG0032896	Biological Process	growth(GO:0040007)	1
OG0032896	Biological Process	metabolic process(GO:0008152)	1
OG0032896	Biological Process	response to stimulus(GO:0050896)	1
OG0032909	Biological Process	metabolic process(GO:0008152)	1
OG0032910	Biological Process	cellular process(GO:0009987)	1
OG0032910	Biological Process	localization(GO:0051179)	1
OG0032913	Biological Process	biological regulation(GO:0065007)	1
OG0032913	Biological Process	localization(GO:0051179)	1
OG0032913	Biological Process	metabolic process(GO:0008152)	1
OG0032913	Biological Process	regulation of biological process(GO:0050789)	1
OG0032913	Biological Process	response to stimulus(GO:0050896)	1
OG0032920	Biological Process	biological regulation(GO:0065007)	1
OG0032920	Biological Process	cellular process(GO:0009987)	1
OG0032920	Biological Process	metabolic process(GO:0008152)	1
OG0032920	Biological Process	regulation of biological process(GO:0050789)	1
OG0032920	Biological Process	response to stimulus(GO:0050896)	1
OG0032920	Biological Process	rhythmic process(GO:0048511)	1
OG0032922	Biological Process	biological regulation(GO:0065007)	1
OG0032922	Biological Process	cellular process(GO:0009987)	1
OG0032922	Biological Process	metabolic process(GO:0008152)	1
OG0032922	Biological Process	regulation of biological process(GO:0050789)	1
OG0032922	Biological Process	response to stimulus(GO:0050896)	1
OG0032922	Biological Process	rhythmic process(GO:0048511)	1
OG0032926	Biological Process	biological regulation(GO:0065007)	1
OG0032926	Biological Process	cellular process(GO:0009987)	1
OG0032926	Biological Process	developmental process(GO:0032502)	1
OG0032926	Biological Process	multicellular organismal process(GO:0032501)	1
OG0032926	Biological Process	positive regulation of biological process(GO:0048518)	1
OG0032926	Biological Process	regulation of biological process(GO:0050789)	1
OG0032926	Biological Process	reproduction(GO:0000003)	1
OG0032926	Biological Process	reproductive process(GO:0022414)	1
OG0032926	Biological Process	response to stimulus(GO:0050896)	1
OG0032928	Biological Process	biological regulation(GO:0065007)	1
OG0032928	Biological Process	cellular component organization or biogenesis(GO:0071840)	1
OG0032928	Biological Process	cellular process(GO:0009987)	1
OG0032928	Biological Process	developmental process(GO:0032502)	1
OG0032928	Biological Process	growth(GO:0040007)	1
OG0032928	Biological Process	metabolic process(GO:0008152)	1
OG0032928	Biological Process	multicellular organismal process(GO:0032501)	1
OG0032928	Biological Process	regulation of biological process(GO:0050789)	1
OG0032928	Biological Process	reproduction(GO:0000003)	1
OG0032928	Biological Process	reproductive process(GO:0022414)	1
OG0032928	Biological Process	response to stimulus(GO:0050896)	1
OG0032928	Biological Process	signaling(GO:0023052)	1

OG0032932	Biological Process	cellular process(GO:0009987)	1
OG0032932	Biological Process	signaling(GO:0023052)	1
OG0032942	Biological Process	cellular process(GO:0009987)	1
OG0032942	Biological Process	metabolic process(GO:0008152)	1
OG0032942	Biological Process	response to stimulus(GO:0050896)	1
OG0032956	Biological Process	metabolic process(GO:0008152)	1
OG0032956	Biological Process	response to stimulus(GO:0050896)	1
OG0032970	Biological Process	cellular process(GO:0009987)	1
OG0032970	Biological Process	metabolic process(GO:0008152)	1
OG0032970	Biological Process	rhythmic process(GO:0048511)	1
OG0032974	Biological Process	biological regulation(GO:0065007)	1
OG0032974	Biological Process	cellular process(GO:0009987)	1
OG0032974	Biological Process	metabolic process(GO:0008152)	1
OG0032975	Biological Process	cellular process(GO:0009987)	1
OG0032975	Biological Process	developmental process(GO:0032502)	1
OG0032975	Biological Process	metabolic process(GO:0008152)	1
OG0032975	Biological Process	response to stimulus(GO:0050896)	1
OG0032983	Biological Process	biological regulation(GO:0065007)	1
OG0032983	Biological Process	cellular process(GO:0009987)	1
OG0032983	Biological Process	metabolic process(GO:0008152)	1
OG0032983	Biological Process	regulation of biological process(GO:0050789)	1
OG0032984	Biological Process	cellular process(GO:0009987)	1
OG0032984	Biological Process	metabolic process(GO:0008152)	1
OG0032986	Biological Process	biological regulation(GO:0065007)	1
OG0032986	Biological Process	cellular component organization or biogenesis(GO:0071840)	1
OG0032986	Biological Process	cellular process(GO:0009987)	1
OG0032986	Biological Process	metabolic process(GO:0008152)	1
OG0032986	Biological Process	positive regulation of biological process(GO:0048518)	1
OG0032986	Biological Process	regulation of biological process(GO:0050789)	1
OG0032987	Biological Process	response to stimulus(GO:0050896)	1
OG0032988	Biological Process	cellular process(GO:0009987)	1
OG0032988	Biological Process	localization(GO:0051179)	1
OG0033002	Biological Process	cellular process(GO:0009987)	1
OG0033002	Biological Process	localization(GO:0051179)	1
OG0033002	Biological Process	metabolic process(GO:0008152)	1
OG0033002	Biological Process	multi-organism process(GO:0051704)	1
OG0033003	Biological Process	cellular process(GO:0009987)	1
OG0033003	Biological Process	metabolic process(GO:0008152)	1
OG0033006	Biological Process	cellular process(GO:0009987)	1
OG0033006	Biological Process	growth(GO:0040007)	1
OG0033006	Biological Process	metabolic process(GO:0008152)	1
OG0033014	Biological Process	cellular process(GO:0009987)	1
OG0033014	Biological Process	growth(GO:0040007)	1
OG0033014	Biological Process	metabolic process(GO:0008152)	1
OG0033016	Biological Process	cellular process(GO:0009987)	1
OG0033016	Biological Process	growth(GO:0040007)	1

OG0033016	Biological Process	metabolic process(GO:0008152)	1
OG0033017	Biological Process	cellular process(GO:0009987)	1
OG0033017	Biological Process	growth(GO:0040007)	1
OG0033017	Biological Process	metabolic process(GO:0008152)	1
OG0033018	Biological Process	biological regulation(GO:0065007)	1
OG0033018	Biological Process	cellular component organization or biogenesis(GO:0071840)	1
OG0033018	Biological Process	cellular process(GO:0009987)	1
OG0033018	Biological Process	growth(GO:0040007)	1
OG0033018	Biological Process	metabolic process(GO:0008152)	1
OG0033018	Biological Process	positive regulation of biological process(GO:0048518)	1
OG0033018	Biological Process	regulation of biological process(GO:0050789)	1
OG0033019	Biological Process	biological regulation(GO:0065007)	1
OG0033019	Biological Process	cellular component organization or biogenesis(GO:0071840)	1
OG0033019	Biological Process	cellular process(GO:0009987)	1
OG0033019	Biological Process	developmental process(GO:0032502)	1
OG0033019	Biological Process	localization(GO:0051179)	1
OG0033019	Biological Process	metabolic process(GO:0008152)	1
OG0033019	Biological Process	positive regulation of biological process(GO:0048518)	1
OG0033019	Biological Process	regulation of biological process(GO:0050789)	1
OG0033019	Biological Process	reproduction(GO:0000003)	1
OG0033019	Biological Process	reproductive process(GO:0022414)	1
OG0033019	Biological Process	response to stimulus(GO:0050896)	1
OG0033019	Biological Process	signaling(GO:0023052)	1
OG0033021	Biological Process	cellular process(GO:0009987)	1
OG0033021	Biological Process	metabolic process(GO:0008152)	1
OG0033022	Biological Process	cellular process(GO:0009987)	1
OG0033022	Biological Process	metabolic process(GO:0008152)	1
OG0033024	Biological Process	cellular process(GO:0009987)	1
OG0033024	Biological Process	metabolic process(GO:0008152)	1
OG0033026	Biological Process	cellular process(GO:0009987)	1
OG0033026	Biological Process	growth(GO:0040007)	1
OG0033026	Biological Process	metabolic process(GO:0008152)	1
OG0033027	Biological Process	cellular process(GO:0009987)	1
OG0033027	Biological Process	metabolic process(GO:0008152)	1
OG0033028	Biological Process	growth(GO:0040007)	1
OG0033028	Biological Process	metabolic process(GO:0008152)	1
OG0033031	Biological Process	cellular component organization or biogenesis(GO:0071840)	1
OG0033031	Biological Process	cellular process(GO:0009987)	1
OG0033031	Biological Process	metabolic process(GO:0008152)	1
OG0033033	Biological Process	growth(GO:0040007)	1
OG0033034	Biological Process	cellular component organization or biogenesis(GO:0071840)	1
OG0033034	Biological Process	cellular process(GO:0009987)	1
OG0033034	Biological Process	metabolic process(GO:0008152)	1
OG0033037	Biological Process	cellular component organization or biogenesis(GO:0071840)	1
OG0033037	Biological Process	cellular process(GO:0009987)	1

OG0033037	Biological Process	metabolic process(GO:0008152)	1
OG0033040	Biological Process	cellular process(GO:0009987)	1
OG0033040	Biological Process	metabolic process(GO:0008152)	1
OG0033040	Biological Process	response to stimulus(GO:0050896)	1
OG0033041	Biological Process	growth(GO:0040007)	1
OG0033042	Biological Process	cellular process(GO:0009987)	1
OG0033042	Biological Process	localization(GO:0051179)	1
OG0033042	Biological Process	metabolic process(GO:0008152)	1
OG0033042	Biological Process	response to stimulus(GO:0050896)	1
OG0033048	Biological Process	cellular process(GO:0009987)	1
OG0033048	Biological Process	metabolic process(GO:0008152)	1
OG0033051	Biological Process	cellular process(GO:0009987)	1
OG0033051	Biological Process	growth(GO:0040007)	1
OG0033051	Biological Process	metabolic process(GO:0008152)	1
OG0033052	Biological Process	biological regulation(GO:0065007)	1
OG0033052	Biological Process	cellular process(GO:0009987)	1
OG0033052	Biological Process	developmental process(GO:0032502)	1
OG0033052	Biological Process	growth(GO:0040007)	1
OG0033052	Biological Process	metabolic process(GO:0008152)	1
OG0033052	Biological Process	regulation of biological process(GO:0050789)	1
OG0033052	Biological Process	response to stimulus(GO:0050896)	1
OG0033053	Biological Process	biological regulation(GO:0065007)	1
OG0033053	Biological Process	cellular component organization or biogenesis(GO:0071840)	1
OG0033053	Biological Process	cellular process(GO:0009987)	1
OG0033053	Biological Process	growth(GO:0040007)	1
OG0033053	Biological Process	metabolic process(GO:0008152)	1
OG0033053	Biological Process	regulation of biological process(GO:0050789)	1
OG0033054	Biological Process	cellular process(GO:0009987)	1
OG0033054	Biological Process	growth(GO:0040007)	1
OG0033054	Biological Process	metabolic process(GO:0008152)	1
OG0033055	Biological Process	biological regulation(GO:0065007)	1
OG0033055	Biological Process	cellular process(GO:0009987)	1
OG0033055	Biological Process	developmental process(GO:0032502)	1
OG0033055	Biological Process	growth(GO:0040007)	1
OG0033055	Biological Process	metabolic process(GO:0008152)	1
OG0033055	Biological Process	regulation of biological process(GO:0050789)	1
OG0033055	Biological Process	response to stimulus(GO:0050896)	1
OG0033059	Biological Process	cellular process(GO:0009987)	1
OG0033059	Biological Process	metabolic process(GO:0008152)	1
OG0033059	Biological Process	response to stimulus(GO:0050896)	1
OG0033062	Biological Process	cellular process(GO:0009987)	1
OG0033062	Biological Process	growth(GO:0040007)	1
OG0033062	Biological Process	metabolic process(GO:0008152)	1
OG0033062	Biological Process	response to stimulus(GO:0050896)	1
OG0033065	Biological Process	cellular process(GO:0009987)	1
OG0033065	Biological Process	metabolic process(GO:0008152)	1

OG0033065	Biological Process	response to stimulus(GO:0050896)	1
OG0033068	Biological Process	cellular process(GO:0009987)	1
OG0033068	Biological Process	growth(GO:0040007)	1
OG0033068	Biological Process	metabolic process(GO:0008152)	1
OG0033069	Biological Process	cellular process(GO:0009987)	1
OG0033069	Biological Process	growth(GO:0040007)	1
OG0033069	Biological Process	metabolic process(GO:0008152)	1
OG0033070	Biological Process	cellular process(GO:0009987)	1
OG0033070	Biological Process	metabolic process(GO:0008152)	1
OG0033085	Biological Process	cellular process(GO:0009987)	1
OG0033085	Biological Process	metabolic process(GO:0008152)	1
OG0033095	Biological Process	biological regulation(GO:0065007)	1
OG0033095	Biological Process	cellular process(GO:0009987)	1
OG0033095	Biological Process	developmental process(GO:0032502)	1
OG0033095	Biological Process	metabolic process(GO:0008152)	1
OG0033095	Biological Process	multicellular organismal process(GO:0032501)	1
OG0033095	Biological Process	negative regulation of biological process(GO:0048519)	1
OG0033095	Biological Process	regulation of biological process(GO:0050789)	1
OG0033095	Biological Process	reproduction(GO:0000003)	1
OG0033095	Biological Process	reproductive process(GO:0022414)	1
OG0033095	Biological Process	response to stimulus(GO:0050896)	1
OG0033101	Biological Process	cellular process(GO:0009987)	1
OG0033101	Biological Process	metabolic process(GO:0008152)	1
OG0033105	Biological Process	cellular process(GO:0009987)	1
OG0033105	Biological Process	metabolic process(GO:0008152)	1
OG0033107	Biological Process	cellular process(GO:0009987)	1
OG0033107	Biological Process	metabolic process(GO:0008152)	1
OG0033107	Biological Process	response to stimulus(GO:0050896)	1
OG0033109	Biological Process	cellular component organization or biogenesis(GO:0071840)	1
OG0033109	Biological Process	cellular process(GO:0009987)	1
OG0033109	Biological Process	metabolic process(GO:0008152)	1
OG0033123	Biological Process	biological regulation(GO:0065007)	1
OG0033123	Biological Process	cellular process(GO:0009987)	1
OG0033123	Biological Process	developmental process(GO:0032502)	1
OG0033123	Biological Process	metabolic process(GO:0008152)	1
OG0033123	Biological Process	multicellular organismal process(GO:0032501)	1
OG0033123	Biological Process	regulation of biological process(GO:0050789)	1
OG0033123	Biological Process	reproduction(GO:0000003)	1
OG0033123	Biological Process	reproductive process(GO:0022414)	1
OG0033124	Biological Process	metabolic process(GO:0008152)	1
OG0033124	Biological Process	response to stimulus(GO:0050896)	1
OG0033128	Biological Process	cellular process(GO:0009987)	1
OG0033128	Biological Process	metabolic process(GO:0008152)	1
OG0033130	Biological Process	cellular component organization or biogenesis(GO:0071840)	1
OG0033130	Biological Process	cellular process(GO:0009987)	1
OG0033130	Biological Process	metabolic process(GO:0008152)	1

OG0033136	Biological Process	biological regulation(GO:0065007)	1
OG0033136	Biological Process	cellular component organization or biogenesis(GO:0071840)	1
OG0033136	Biological Process	cellular process(GO:0009987)	1
OG0033136	Biological Process	metabolic process(GO:0008152)	1
OG0033136	Biological Process	regulation of biological process(GO:0050789)	1
OG0033136	Biological Process	response to stimulus(GO:0050896)	1
OG0033139	Biological Process	biological regulation(GO:0065007)	1
OG0033139	Biological Process	localization(GO:0051179)	1
OG0033139	Biological Process	metabolic process(GO:0008152)	1
OG0033139	Biological Process	regulation of biological process(GO:0050789)	1
OG0033139	Biological Process	response to stimulus(GO:0050896)	1
OG0033140	Biological Process	biological regulation(GO:0065007)	1
OG0033140	Biological Process	localization(GO:0051179)	1
OG0033140	Biological Process	metabolic process(GO:0008152)	1
OG0033140	Biological Process	regulation of biological process(GO:0050789)	1
OG0033140	Biological Process	response to stimulus(GO:0050896)	1
OG0033141	Biological Process	response to stimulus(GO:0050896)	1
OG0033145	Biological Process	cellular process(GO:0009987)	1
OG0033145	Biological Process	developmental process(GO:0032502)	1
OG0033145	Biological Process	metabolic process(GO:0008152)	1
OG0033145	Biological Process	multicellular organismal process(GO:0032501)	1
OG0033147	Biological Process	cellular process(GO:0009987)	1
OG0033147	Biological Process	developmental process(GO:0032502)	1
OG0033147	Biological Process	metabolic process(GO:0008152)	1
OG0033147	Biological Process	multi-organism process(GO:0051704)	1
OG0033147	Biological Process	multicellular organismal process(GO:0032501)	1
OG0033147	Biological Process	reproduction(GO:0000003)	1
OG0033147	Biological Process	reproductive process(GO:0022414)	1
OG0033153	Biological Process	biological regulation(GO:0065007)	1
OG0033153	Biological Process	cellular process(GO:0009987)	1
OG0033153	Biological Process	metabolic process(GO:0008152)	1
OG0033153	Biological Process	multi-organism process(GO:0051704)	1
OG0033153	Biological Process	regulation of biological process(GO:0050789)	1
OG0033155	Biological Process	biological regulation(GO:0065007)	1
OG0033155	Biological Process	cellular process(GO:0009987)	1
OG0033155	Biological Process	detoxification(GO:0098754)	1
OG0033155	Biological Process	metabolic process(GO:0008152)	1
OG0033155	Biological Process	regulation of biological process(GO:0050789)	1
OG0033155	Biological Process	response to stimulus(GO:0050896)	1
OG0033157	Biological Process	cellular process(GO:0009987)	1
OG0033157	Biological Process	localization(GO:0051179)	1
OG0033176	Biological Process	response to stimulus(GO:0050896)	1
OG0033177	Biological Process	response to stimulus(GO:0050896)	1
OG0033184	Biological Process	cellular component organization or biogenesis(GO:0071840)	1
OG0033184	Biological Process	cellular process(GO:0009987)	1

OG0033184	Biological Process	developmental process(GO:0032502)	1
OG0033184	Biological Process	growth(GO:0040007)	1
OG0033184	Biological Process	metabolic process(GO:0008152)	1
OG0033184	Biological Process	multicellular organismal process(GO:0032501)	1
OG0033184	Biological Process	response to stimulus(GO:0050896)	1
OG0033191	Biological Process	response to stimulus(GO:0050896)	1
OG0033192	Biological Process	cellular process(GO:0009987)	1
OG0033192	Biological Process	metabolic process(GO:0008152)	1
OG0033192	Biological Process	response to stimulus(GO:0050896)	1
OG0033218	Biological Process	cellular component organization or biogenesis(GO:0071840)	1
OG0033218	Biological Process	cellular process(GO:0009987)	1
OG0033218	Biological Process	metabolic process(GO:0008152)	1
OG0033231	Biological Process	biological regulation(GO:0065007)	1
OG0033231	Biological Process	localization(GO:0051179)	1
OG0033231	Biological Process	metabolic process(GO:0008152)	1
OG0033231	Biological Process	regulation of biological process(GO:0050789)	1
OG0033231	Biological Process	response to stimulus(GO:0050896)	1
OG0033242	Biological Process	response to stimulus(GO:0050896)	1
OG0033243	Biological Process	cellular process(GO:0009987)	1
OG0033243	Biological Process	developmental process(GO:0032502)	1
OG0033243	Biological Process	metabolic process(GO:0008152)	1
OG0033243	Biological Process	multicellular organismal process(GO:0032501)	1
OG0033243	Biological Process	response to stimulus(GO:0050896)	1
OG0033247	Biological Process	response to stimulus(GO:0050896)	1
OG0033252	Biological Process	developmental process(GO:0032502)	1
OG0033252	Biological Process	multicellular organismal process(GO:0032501)	1
OG0033252	Biological Process	reproduction(GO:0000003)	1
OG0033252	Biological Process	reproductive process(GO:0022414)	1
OG0033252	Biological Process	response to stimulus(GO:0050896)	1
OG0033265	Biological Process	cellular process(GO:0009987)	1
OG0033265	Biological Process	metabolic process(GO:0008152)	1
OG0033267	Biological Process	biological regulation(GO:0065007)	1
OG0033267	Biological Process	cellular process(GO:0009987)	1
OG0033267	Biological Process	developmental process(GO:0032502)	1
OG0033267	Biological Process	metabolic process(GO:0008152)	1
OG0033267	Biological Process	multicellular organismal process(GO:0032501)	1
OG0033267	Biological Process	pigmentation(GO:0043473)	1
OG0033267	Biological Process	regulation of biological process(GO:0050789)	1
OG0033267	Biological Process	response to stimulus(GO:0050896)	1
OG0033268	Biological Process	cellular process(GO:0009987)	1
OG0033268	Biological Process	metabolic process(GO:0008152)	1
OG0033268	Biological Process	response to stimulus(GO:0050896)	1
OG0033291	Biological Process	cellular process(GO:0009987)	1
OG0033291	Biological Process	immune system process(GO:0002376)	1
OG0033291	Biological Process	metabolic process(GO:0008152)	1
OG0033291	Biological Process	response to stimulus(GO:0050896)	1

OG0033294	Biological Process	cellular process(GO:0009987)	1
OG0033294	Biological Process	metabolic process(GO:0008152)	1
OG0033294	Biological Process	response to stimulus(GO:0050896)	1
OG0033299	Biological Process	biological regulation(GO:0065007)	1
OG0033299	Biological Process	cellular process(GO:0009987)	1
OG0033299	Biological Process	metabolic process(GO:0008152)	1
OG0033299	Biological Process	multi-organism process(GO:0051704)	1
OG0033299	Biological Process	regulation of biological process(GO:0050789)	1
OG0033299	Biological Process	response to stimulus(GO:0050896)	1
OG0033300	Biological Process	developmental process(GO:0032502)	1
OG0033300	Biological Process	multi-organism process(GO:0051704)	1
OG0033300	Biological Process	multicellular organismal process(GO:0032501)	1
OG0033300	Biological Process	response to stimulus(GO:0050896)	1
OG0033303	Biological Process	biological regulation(GO:0065007)	1
OG0033303	Biological Process	cellular process(GO:0009987)	1
OG0033303	Biological Process	developmental process(GO:0032502)	1
OG0033303	Biological Process	metabolic process(GO:0008152)	1
OG0033303	Biological Process	multicellular organismal process(GO:0032501)	1
OG0033303	Biological Process	positive regulation of biological process(GO:0048518)	1
OG0033303	Biological Process	regulation of biological process(GO:0050789)	1
OG0033303	Biological Process	reproduction(GO:0000003)	1
OG0033303	Biological Process	reproductive process(GO:0022414)	1
OG0033307	Biological Process	biological regulation(GO:0065007)	1
OG0033307	Biological Process	cellular process(GO:0009987)	1
OG0033307	Biological Process	metabolic process(GO:0008152)	1
OG0033307	Biological Process	response to stimulus(GO:0050896)	1
OG0033308	Biological Process	cellular process(GO:0009987)	1
OG0033308	Biological Process	developmental process(GO:0032502)	1
OG0033308	Biological Process	metabolic process(GO:0008152)	1
OG0033308	Biological Process	multicellular organismal process(GO:0032501)	1
OG0033308	Biological Process	response to stimulus(GO:0050896)	1
OG0033310	Biological Process	cellular process(GO:0009987)	1
OG0033310	Biological Process	localization(GO:0051179)	1
OG0033310	Biological Process	response to stimulus(GO:0050896)	1
OG0033311	Biological Process	biological regulation(GO:0065007)	1
OG0033311	Biological Process	metabolic process(GO:0008152)	1
OG0033311	Biological Process	regulation of biological process(GO:0050789)	1
OG0033318	Biological Process	cellular component organization or biogenesis(GO:0071840)	1
OG0033318	Biological Process	cellular process(GO:0009987)	1
OG0033318	Biological Process	metabolic process(GO:0008152)	1
OG0033321	Biological Process	cellular process(GO:0009987)	1
OG0033321	Biological Process	response to stimulus(GO:0050896)	1
OG0033324	Biological Process	cellular process(GO:0009987)	1
OG0033324	Biological Process	metabolic process(GO:0008152)	1
OG0033327	Biological Process	cellular process(GO:0009987)	1

OG0033327	Biological Process	metabolic process(GO:0008152)	1
OG0033327	Biological Process	multi-organism process(GO:0051704)	1
OG0033327	Biological Process	response to stimulus(GO:0050896)	1
OG0033332	Biological Process	biological regulation(GO:0065007)	1
OG0033332	Biological Process	cellular process(GO:0009987)	1
OG0033332	Biological Process	metabolic process(GO:0008152)	1
OG0033332	Biological Process	multi-organism process(GO:0051704)	1
OG0033332	Biological Process	negative regulation of biological process(GO:0048519)	1
OG0033332	Biological Process	regulation of biological process(GO:0050789)	1
OG0033332	Biological Process	response to stimulus(GO:0050896)	1
OG0033332	Biological Process	signaling(GO:0023052)	1
OG0033335	Biological Process	biological regulation(GO:0065007)	1
OG0033335	Biological Process	cellular component organization or biogenesis(GO:0071840)	1
OG0033335	Biological Process	cellular process(GO:0009987)	1
OG0033335	Biological Process	developmental process(GO:0032502)	1
OG0033335	Biological Process	localization(GO:0051179)	1
OG0033335	Biological Process	metabolic process(GO:0008152)	1
OG0033335	Biological Process	multicellular organismal process(GO:0032501)	1
OG0033335	Biological Process	regulation of biological process(GO:0050789)	1
OG0033335	Biological Process	reproduction(GO:0000003)	1
OG0033335	Biological Process	reproductive process(GO:0022414)	1
OG0033335	Biological Process	response to stimulus(GO:0050896)	1
OG0033335	Biological Process	signaling(GO:0023052)	1
OG0033340	Biological Process	response to stimulus(GO:0050896)	1
OG0033341	Biological Process	biological regulation(GO:0065007)	1
OG0033341	Biological Process	cellular process(GO:0009987)	1
OG0033341	Biological Process	growth(GO:0040007)	1
OG0033341	Biological Process	localization(GO:0051179)	1
OG0033341	Biological Process	metabolic process(GO:0008152)	1
OG0033341	Biological Process	regulation of biological process(GO:0050789)	1
OG0033341	Biological Process	response to stimulus(GO:0050896)	1
OG0033343	Biological Process	biological regulation(GO:0065007)	1
OG0033343	Biological Process	cellular process(GO:0009987)	1
OG0033343	Biological Process	metabolic process(GO:0008152)	1
OG0033343	Biological Process	regulation of biological process(GO:0050789)	1
OG0033344	Biological Process	cellular process(GO:0009987)	1
OG0033344	Biological Process	metabolic process(GO:0008152)	1
OG0033347	Biological Process	cellular process(GO:0009987)	1
OG0033347	Biological Process	growth(GO:0040007)	1
OG0033347	Biological Process	metabolic process(GO:0008152)	1
OG0033348	Biological Process	cellular process(GO:0009987)	1
OG0033348	Biological Process	metabolic process(GO:0008152)	1
OG0033354	Biological Process	cellular process(GO:0009987)	1
OG0033354	Biological Process	metabolic process(GO:0008152)	1
OG0033355	Biological Process	cellular process(GO:0009987)	1
OG0033355	Biological Process	metabolic process(GO:0008152)	1

OG0033359	Biological Process	cellular component organization or biogenesis(GO:0071840)	1
OG0033359	Biological Process	cellular process(GO:0009987)	1
OG0033359	Biological Process	developmental process(GO:0032502)	1
OG0033359	Biological Process	metabolic process(GO:0008152)	1
OG0033359	Biological Process	multi-organism process(GO:0051704)	1
OG0033359	Biological Process	multicellular organismal process(GO:0032501)	1
OG0033359	Biological Process	response to stimulus(GO:0050896)	1
OG0033361	Biological Process	cellular process(GO:0009987)	1
OG0033361	Biological Process	metabolic process(GO:0008152)	1
OG0033374	Biological Process	response to stimulus(GO:0050896)	1
OG0033388	Biological Process	cellular process(GO:0009987)	1
OG0033388	Biological Process	localization(GO:0051179)	1
OG0033388	Biological Process	metabolic process(GO:0008152)	1
OG0033413	Biological Process	biological regulation(GO:0065007)	1
OG0033413	Biological Process	cellular process(GO:0009987)	1
OG0033413	Biological Process	metabolic process(GO:0008152)	1
OG0033413	Biological Process	negative regulation of biological process(GO:0048519)	1
OG0033413	Biological Process	regulation of biological process(GO:0050789)	1
OG0033413	Biological Process	response to stimulus(GO:0050896)	1
OG0033414	Biological Process	cellular process(GO:0009987)	1
OG0033414	Biological Process	localization(GO:0051179)	1
OG0033414	Biological Process	metabolic process(GO:0008152)	1
OG0033415	Biological Process	growth(GO:0040007)	1
OG0033419	Biological Process	cellular process(GO:0009987)	1
OG0033419	Biological Process	metabolic process(GO:0008152)	1
OG0033422	Biological Process	cellular process(GO:0009987)	1
OG0033422	Biological Process	metabolic process(GO:0008152)	1
OG0033428	Biological Process	cellular component organization or biogenesis(GO:0071840)	1
OG0033428	Biological Process	cellular process(GO:0009987)	1
OG0033428	Biological Process	localization(GO:0051179)	1
OG0033428	Biological Process	metabolic process(GO:0008152)	1
OG0033429	Biological Process	biological regulation(GO:0065007)	1
OG0033429	Biological Process	cellular component organization or biogenesis(GO:0071840)	1
OG0033429	Biological Process	cellular process(GO:0009987)	1
OG0033429	Biological Process	growth(GO:0040007)	1
OG0033429	Biological Process	metabolic process(GO:0008152)	1
OG0033429	Biological Process	regulation of biological process(GO:0050789)	1
OG0033433	Biological Process	biological regulation(GO:0065007)	1
OG0033433	Biological Process	cellular component organization or biogenesis(GO:0071840)	1
OG0033433	Biological Process	cellular process(GO:0009987)	1
OG0033433	Biological Process	growth(GO:0040007)	1
OG0033433	Biological Process	metabolic process(GO:0008152)	1
OG0033433	Biological Process	regulation of biological process(GO:0050789)	1
OG0033435	Biological Process	cellular process(GO:0009987)	1
OG0033435	Biological Process	metabolic process(GO:0008152)	1

OG0033441	Biological Process	cellular component organization or biogenesis(GO:0071840)	1
OG0033441	Biological Process	cellular process(GO:0009987)	1
OG0033441	Biological Process	growth(GO:0040007)	1
OG0033441	Biological Process	metabolic process(GO:0008152)	1
OG0033444	Biological Process	cellular process(GO:0009987)	1
OG0033444	Biological Process	metabolic process(GO:0008152)	1
OG0033446	Biological Process	cellular process(GO:0009987)	1
OG0033446	Biological Process	growth(GO:0040007)	1
OG0033446	Biological Process	metabolic process(GO:0008152)	1
OG0033450	Biological Process	cellular process(GO:0009987)	1
OG0033450	Biological Process	metabolic process(GO:0008152)	1
OG0033450	Biological Process	response to stimulus(GO:0050896)	1
OG0033453	Biological Process	cellular process(GO:0009987)	1
OG0033453	Biological Process	metabolic process(GO:0008152)	1
OG0033458	Biological Process	cellular process(GO:0009987)	1
OG0033458	Biological Process	growth(GO:0040007)	1
OG0033458	Biological Process	metabolic process(GO:0008152)	1
OG0033459	Biological Process	cellular process(GO:0009987)	1
OG0033459	Biological Process	metabolic process(GO:0008152)	1
OG0033459	Biological Process	response to stimulus(GO:0050896)	1
OG0033460	Biological Process	biological regulation(GO:0065007)	1
OG0033460	Biological Process	cellular process(GO:0009987)	1
OG0033460	Biological Process	metabolic process(GO:0008152)	1
OG0033460	Biological Process	negative regulation of biological process(GO:0048519)	1
OG0033460	Biological Process	regulation of biological process(GO:0050789)	1
OG0033460	Biological Process	response to stimulus(GO:0050896)	1
OG0033462	Biological Process	cellular process(GO:0009987)	1
OG0033462	Biological Process	metabolic process(GO:0008152)	1
OG0033463	Biological Process	cellular process(GO:0009987)	1
OG0033463	Biological Process	growth(GO:0040007)	1
OG0033463	Biological Process	metabolic process(GO:0008152)	1
OG0033464	Biological Process	cellular process(GO:0009987)	1
OG0033464	Biological Process	metabolic process(GO:0008152)	1
OG0033467	Biological Process	growth(GO:0040007)	1
OG0033469	Biological Process	cellular process(GO:0009987)	1
OG0033469	Biological Process	metabolic process(GO:0008152)	1
OG0033470	Biological Process	cellular process(GO:0009987)	1
OG0033470	Biological Process	metabolic process(GO:0008152)	1
OG0033484	Biological Process	cellular process(GO:0009987)	1
OG0033484	Biological Process	growth(GO:0040007)	1
OG0033484	Biological Process	metabolic process(GO:0008152)	1
OG0033489	Biological Process	biological regulation(GO:0065007)	1
OG0033489	Biological Process	cellular component organization or biogenesis(GO:0071840)	1
OG0033489	Biological Process	cellular process(GO:0009987)	1
OG0033489	Biological Process	metabolic process(GO:0008152)	1
OG0033489	Biological Process	negative regulation of biological process(GO:0048519)	1

OG0033489	Biological Process	regulation of biological process(GO:0050789)	1
OG0033489	Biological Process	response to stimulus(GO:0050896)	1
OG0033490	Biological Process	cellular process(GO:0009987)	1
OG0033490	Biological Process	localization(GO:0051179)	1
OG0033490	Biological Process	metabolic process(GO:0008152)	1
OG0033491	Biological Process	cellular component organization or biogenesis(GO:0071840)	1
OG0033491	Biological Process	cellular process(GO:0009987)	1
OG0033491	Biological Process	growth(GO:0040007)	1
OG0033491	Biological Process	metabolic process(GO:0008152)	1
OG0033492	Biological Process	cellular process(GO:0009987)	1
OG0033492	Biological Process	localization(GO:0051179)	1
OG0033492	Biological Process	locomotion(GO:0040011)	1
OG0033504	Biological Process	cellular process(GO:0009987)	1
OG0033504	Biological Process	metabolic process(GO:0008152)	1
OG0033510	Biological Process	cellular process(GO:0009987)	1
OG0033510	Biological Process	metabolic process(GO:0008152)	1
OG0033514	Biological Process	cellular process(GO:0009987)	1
OG0033514	Biological Process	metabolic process(GO:0008152)	1
OG0033517	Biological Process	cellular process(GO:0009987)	1
OG0033517	Biological Process	metabolic process(GO:0008152)	1
OG0033518	Biological Process	biological regulation(GO:0065007)	1
OG0033518	Biological Process	cellular process(GO:0009987)	1
OG0033518	Biological Process	developmental process(GO:0032502)	1
OG0033518	Biological Process	growth(GO:0040007)	1
OG0033518	Biological Process	metabolic process(GO:0008152)	1
OG0033518	Biological Process	regulation of biological process(GO:0050789)	1
OG0033518	Biological Process	response to stimulus(GO:0050896)	1
OG0033520	Biological Process	biological regulation(GO:0065007)	1
OG0033520	Biological Process	cellular process(GO:0009987)	1
OG0033520	Biological Process	developmental process(GO:0032502)	1
OG0033520	Biological Process	growth(GO:0040007)	1
OG0033520	Biological Process	metabolic process(GO:0008152)	1
OG0033520	Biological Process	regulation of biological process(GO:0050789)	1
OG0033520	Biological Process	response to stimulus(GO:0050896)	1
OG0033521	Biological Process	biological regulation(GO:0065007)	1
OG0033521	Biological Process	cellular process(GO:0009987)	1
OG0033521	Biological Process	developmental process(GO:0032502)	1
OG0033521	Biological Process	growth(GO:0040007)	1
OG0033521	Biological Process	metabolic process(GO:0008152)	1
OG0033521	Biological Process	regulation of biological process(GO:0050789)	1
OG0033521	Biological Process	response to stimulus(GO:0050896)	1
OG0033522	Biological Process	growth(GO:0040007)	1
OG0033522	Biological Process	localization(GO:0051179)	1
OG0033522	Biological Process	multi-organism process(GO:0051704)	1
OG0033523	Biological Process	cellular component organization or biogenesis(GO:0071840)	1
OG0033523	Biological Process	cellular process(GO:0009987)	1

OG0033523	Biological Process	growth(GO:0040007)	1
OG0033523	Biological Process	metabolic process(GO:0008152)	1
OG0033533	Biological Process	cellular process(GO:0009987)	1
OG0033533	Biological Process	metabolic process(GO:0008152)	1
OG0033534	Biological Process	cellular process(GO:0009987)	1
OG0033534	Biological Process	metabolic process(GO:0008152)	1
OG0033539	Biological Process	response to stimulus(GO:0050896)	1
OG0033551	Biological Process	response to stimulus(GO:0050896)	1
OG0033552	Biological Process	biological regulation(GO:0065007)	1
OG0033552	Biological Process	cellular process(GO:0009987)	1
OG0033552	Biological Process	metabolic process(GO:0008152)	1
OG0033552	Biological Process	regulation of biological process(GO:0050789)	1
OG0033554	Biological Process	cellular process(GO:0009987)	1
OG0033554	Biological Process	metabolic process(GO:0008152)	1
OG0033556	Biological Process	cellular process(GO:0009987)	1
OG0033556	Biological Process	metabolic process(GO:0008152)	1
OG0033568	Biological Process	biological regulation(GO:0065007)	1
OG0033568	Biological Process	cellular process(GO:0009987)	1
OG0033568	Biological Process	metabolic process(GO:0008152)	1
OG0033568	Biological Process	positive regulation of biological process(GO:0048518)	1
OG0033568	Biological Process	regulation of biological process(GO:0050789)	1
OG0033571	Biological Process	cellular process(GO:0009987)	1
OG0033571	Biological Process	metabolic process(GO:0008152)	1
OG0033571	Biological Process	multi-organism process(GO:0051704)	1
OG0033571	Biological Process	response to stimulus(GO:0050896)	1
OG0033582	Biological Process	biological regulation(GO:0065007)	1
OG0033582	Biological Process	cellular process(GO:0009987)	1
OG0033582	Biological Process	metabolic process(GO:0008152)	1
OG0033582	Biological Process	positive regulation of biological process(GO:0048518)	1
OG0033582	Biological Process	regulation of biological process(GO:0050789)	1
OG0033582	Biological Process	response to stimulus(GO:0050896)	1
OG0033599	Biological Process	biological regulation(GO:0065007)	1
OG0033599	Biological Process	cellular process(GO:0009987)	1
OG0033599	Biological Process	metabolic process(GO:0008152)	1
OG0033599	Biological Process	positive regulation of biological process(GO:0048518)	1
OG0033599	Biological Process	regulation of biological process(GO:0050789)	1
OG0033599	Biological Process	response to stimulus(GO:0050896)	1
OG0033599	Biological Process	rhythmic process(GO:0048511)	1
OG0033599	Biological Process	signaling(GO:0023052)	1
OG0033604	Biological Process	biological regulation(GO:0065007)	1
OG0033604	Biological Process	cellular process(GO:0009987)	1
OG0033604	Biological Process	developmental process(GO:0032502)	1
OG0033604	Biological Process	metabolic process(GO:0008152)	1
OG0033604	Biological Process	multicellular organismal process(GO:0032501)	1
OG0033604	Biological Process	regulation of biological process(GO:0050789)	1

OG0033604	Biological Process	response to stimulus(GO:0050896)	1
OG0033608	Biological Process	developmental process(GO:0032502)	1
OG0033608	Biological Process	multicellular organismal process(GO:0032501)	1
OG0033608	Biological Process	reproduction(GO:0000003)	1
OG0033608	Biological Process	reproductive process(GO:0022414)	1
OG0033608	Biological Process	response to stimulus(GO:0050896)	1
OG0033609	Biological Process	cellular process(GO:0009987)	1
OG0033609	Biological Process	developmental process(GO:0032502)	1
OG0033609	Biological Process	multi-organism process(GO:0051704)	1
OG0033609	Biological Process	multicellular organismal process(GO:0032501)	1
OG0033609	Biological Process	reproduction(GO:0000003)	1
OG0033609	Biological Process	reproductive process(GO:0022414)	1
OG0033611	Biological Process	cellular process(GO:0009987)	1
OG0033611	Biological Process	developmental process(GO:0032502)	1
OG0033611	Biological Process	multi-organism process(GO:0051704)	1
OG0033611	Biological Process	multicellular organismal process(GO:0032501)	1
OG0033611	Biological Process	reproduction(GO:0000003)	1
OG0033611	Biological Process	reproductive process(GO:0022414)	1
OG0033617	Biological Process	cellular component organization or biogenesis(GO:0071840)	1
OG0033617	Biological Process	cellular process(GO:0009987)	1
OG0033617	Biological Process	developmental process(GO:0032502)	1
OG0033617	Biological Process	growth(GO:0040007)	1
OG0033617	Biological Process	metabolic process(GO:0008152)	1
OG0033617	Biological Process	response to stimulus(GO:0050896)	1
OG0033618	Biological Process	biological regulation(GO:0065007)	1
OG0033618	Biological Process	cellular process(GO:0009987)	1
OG0033618	Biological Process	metabolic process(GO:0008152)	1
OG0033618	Biological Process	regulation of biological process(GO:0050789)	1
OG0033624	Biological Process	biological regulation(GO:0065007)	1
OG0033624	Biological Process	cellular process(GO:0009987)	1
OG0033624	Biological Process	localization(GO:0051179)	1
OG0033624	Biological Process	metabolic process(GO:0008152)	1
OG0033624	Biological Process	regulation of biological process(GO:0050789)	1
OG0033624	Biological Process	response to stimulus(GO:0050896)	1
OG0033624	Biological Process	signaling(GO:0023052)	1
OG0033625	Biological Process	cellular process(GO:0009987)	1
OG0033625	Biological Process	metabolic process(GO:0008152)	1
OG0033630	Biological Process	cellular process(GO:0009987)	1
OG0033630	Biological Process	metabolic process(GO:0008152)	1
OG0033631	Biological Process	cellular process(GO:0009987)	1
OG0033631	Biological Process	localization(GO:0051179)	1
OG0033631	Biological Process	metabolic process(GO:0008152)	1
OG0033650	Biological Process	cellular component organization or biogenesis(GO:0071840)	1
OG0033650	Biological Process	cellular process(GO:0009987)	1
OG0033650	Biological Process	metabolic process(GO:0008152)	1
OG0033653	Biological Process	response to stimulus(GO:0050896)	1

OG0033655	Biological Process	cellular component organization or biogenesis(GO:0071840)	1
OG0033655	Biological Process	cellular process(GO:0009987)	1
OG0033657	Biological Process	biological regulation(GO:0065007)	1
OG0033657	Biological Process	cellular process(GO:0009987)	1
OG0033657	Biological Process	metabolic process(GO:0008152)	1
OG0033657	Biological Process	regulation of biological process(GO:0050789)	1
OG0033657	Biological Process	response to stimulus(GO:0050896)	1
OG0033661	Biological Process	metabolic process(GO:0008152)	1
OG0033667	Biological Process	biological regulation(GO:0065007)	1
OG0033667	Biological Process	growth(GO:0040007)	1
OG0033667	Biological Process	immune system process(GO:0002376)	1
OG0033667	Biological Process	localization(GO:0051179)	1
OG0033667	Biological Process	metabolic process(GO:0008152)	1
OG0033667	Biological Process	multi-organism process(GO:0051704)	1
OG0033667	Biological Process	regulation of biological process(GO:0050789)	1
OG0033667	Biological Process	response to stimulus(GO:0050896)	1
OG0033668	Biological Process	biological regulation(GO:0065007)	1
OG0033668	Biological Process	cellular process(GO:0009987)	1
OG0033668	Biological Process	metabolic process(GO:0008152)	1
OG0033668	Biological Process	regulation of biological process(GO:0050789)	1
OG0033668	Biological Process	response to stimulus(GO:0050896)	1
OG0033673	Biological Process	cellular process(GO:0009987)	1
OG0033673	Biological Process	growth(GO:0040007)	1
OG0033673	Biological Process	metabolic process(GO:0008152)	1
OG0033675	Biological Process	cellular process(GO:0009987)	1
OG0033675	Biological Process	metabolic process(GO:0008152)	1
OG0033678	Biological Process	cellular component organization or biogenesis(GO:0071840)	1
OG0033678	Biological Process	cellular process(GO:0009987)	1
OG0033678	Biological Process	localization(GO:0051179)	1
OG0033678	Biological Process	metabolic process(GO:0008152)	1
OG0033679	Biological Process	response to stimulus(GO:0050896)	1
OG0033680	Biological Process	cellular process(GO:0009987)	1
OG0033680	Biological Process	metabolic process(GO:0008152)	1
OG0033694	Biological Process	cellular process(GO:0009987)	1
OG0033694	Biological Process	metabolic process(GO:0008152)	1
OG0033699	Biological Process	cellular process(GO:0009987)	1
OG0033699	Biological Process	developmental process(GO:0032502)	1
OG0033699	Biological Process	metabolic process(GO:0008152)	1
OG0033699	Biological Process	multicellular organismal process(GO:0032501)	1
OG0033703	Biological Process	biological regulation(GO:0065007)	1
OG0033703	Biological Process	cellular process(GO:0009987)	1
OG0033703	Biological Process	developmental process(GO:0032502)	1
OG0033703	Biological Process	metabolic process(GO:0008152)	1
OG0033703	Biological Process	multicellular organismal process(GO:0032501)	1
OG0033703	Biological Process	positive regulation of biological process(GO:0048518)	1

OG0033703	Biological Process	regulation of biological process(GO:0050789)	1
OG0033703	Biological Process	reproduction(GO:0000003)	1
OG0033703	Biological Process	reproductive process(GO:0022414)	1
OG0033715	Biological Process	cellular process(GO:0009987)	1
OG0033715	Biological Process	localization(GO:0051179)	1
OG0033715	Biological Process	response to stimulus(GO:0050896)	1
OG0033716	Biological Process	biological regulation(GO:0065007)	1
OG0033716	Biological Process	cell proliferation(GO:0008283)	1
OG0033716	Biological Process	cellular component organization or biogenesis(GO:0071840)	1
OG0033716	Biological Process	cellular process(GO:0009987)	1
OG0033716	Biological Process	developmental process(GO:0032502)	1
OG0033716	Biological Process	metabolic process(GO:0008152)	1
OG0033716	Biological Process	multicellular organismal process(GO:0032501)	1
OG0033716	Biological Process	negative regulation of biological process(GO:0048519)	1
OG0033716	Biological Process	positive regulation of biological process(GO:0048518)	1
OG0033716	Biological Process	regulation of biological process(GO:0050789)	1
OG0033716	Biological Process	response to stimulus(GO:0050896)	1
OG0033716	Biological Process	signaling(GO:0023052)	1
OG0033726	Biological Process	cellular process(GO:0009987)	1
OG0033726	Biological Process	metabolic process(GO:0008152)	1
OG0033727	Biological Process	cellular component organization or biogenesis(GO:0071840)	1
OG0033727	Biological Process	cellular process(GO:0009987)	1
OG0033727	Biological Process	metabolic process(GO:0008152)	1
OG0033728	Biological Process	cellular process(GO:0009987)	1
OG0033728	Biological Process	metabolic process(GO:0008152)	1
OG0033733	Biological Process	metabolic process(GO:0008152)	1
OG0033738	Biological Process	metabolic process(GO:0008152)	1
OG0033740	Biological Process	cellular process(GO:0009987)	1
OG0033740	Biological Process	metabolic process(GO:0008152)	1
OG0033741	Biological Process	cellular process(GO:0009987)	1
OG0033741	Biological Process	metabolic process(GO:0008152)	1
OG0033749	Biological Process	cellular process(GO:0009987)	1
OG0033749	Biological Process	developmental process(GO:0032502)	1
OG0033749	Biological Process	metabolic process(GO:0008152)	1
OG0033749	Biological Process	multicellular organismal process(GO:0032501)	1
OG0033749	Biological Process	reproduction(GO:0000003)	1
OG0033749	Biological Process	reproductive process(GO:0022414)	1
OG0033750	Biological Process	cellular component organization or biogenesis(GO:0071840)	1
OG0033750	Biological Process	cellular process(GO:0009987)	1
OG0033750	Biological Process	metabolic process(GO:0008152)	1
OG0033751	Biological Process	response to stimulus(GO:0050896)	1
OG0033761	Biological Process	biological adhesion(GO:0022610)	1
OG0033761	Biological Process	biological regulation(GO:0065007)	1
OG0033761	Biological Process	cellular component organization or biogenesis(GO:0071840)	1
OG0033761	Biological Process	cellular process(GO:0009987)	1

OG0033761	Biological Process	developmental process(GO:0032502)	1
OG0033761	Biological Process	metabolic process(GO:0008152)	1
OG0033761	Biological Process	multi-organism process(GO:0051704)	1
OG0033761	Biological Process	multicellular organismal process(GO:0032501)	1
OG0033761	Biological Process	negative regulation of biological process(GO:0048519)	1
OG0033761	Biological Process	regulation of biological process(GO:0050789)	1
OG0033761	Biological Process	reproduction(GO:0000003)	1
OG0033761	Biological Process	reproductive process(GO:0022414)	1
OG0033761	Biological Process	response to stimulus(GO:0050896)	1
OG0033761	Biological Process	signaling(GO:0023052)	1
OG0033763	Biological Process	response to stimulus(GO:0050896)	1
OG0033764	Biological Process	cellular process(GO:0009987)	1
OG0033764	Biological Process	metabolic process(GO:0008152)	1
OG0033768	Biological Process	cellular process(GO:0009987)	1
OG0033768	Biological Process	developmental process(GO:0032502)	1
OG0033768	Biological Process	metabolic process(GO:0008152)	1
OG0033768	Biological Process	multicellular organismal process(GO:0032501)	1
OG0033768	Biological Process	reproduction(GO:0000003)	1
OG0033768	Biological Process	reproductive process(GO:0022414)	1
OG0033768	Biological Process	response to stimulus(GO:0050896)	1
OG0033769	Biological Process	metabolic process(GO:0008152)	1
OG0033772	Biological Process	biological regulation(GO:0065007)	1
OG0033772	Biological Process	cellular process(GO:0009987)	1
OG0033772	Biological Process	developmental process(GO:0032502)	1
OG0033772	Biological Process	metabolic process(GO:0008152)	1
OG0033772	Biological Process	multicellular organismal process(GO:0032501)	1
OG0033772	Biological Process	regulation of biological process(GO:0050789)	1
OG0033782	Biological Process	biological regulation(GO:0065007)	1
OG0033782	Biological Process	cellular component organization or biogenesis(GO:0071840)	1
OG0033782	Biological Process	cellular process(GO:0009987)	1
OG0033782	Biological Process	developmental process(GO:0032502)	1
OG0033782	Biological Process	growth(GO:0040007)	1
OG0033782	Biological Process	localization(GO:0051179)	1
OG0033782	Biological Process	locomotion(GO:0040011)	1
OG0033782	Biological Process	metabolic process(GO:0008152)	1
OG0033782	Biological Process	multi-organism process(GO:0051704)	1
OG0033782	Biological Process	multicellular organismal process(GO:0032501)	1
OG0033782	Biological Process	negative regulation of biological process(GO:0048519)	1
OG0033782	Biological Process	positive regulation of biological process(GO:0048518)	1
OG0033782	Biological Process	regulation of biological process(GO:0050789)	1
OG0033782	Biological Process	reproduction(GO:0000003)	1
OG0033782	Biological Process	response to stimulus(GO:0050896)	1
OG0033782	Biological Process	signaling(GO:0023052)	1
OG0033783	Biological Process	biological regulation(GO:0065007)	1
OG0033783	Biological Process	cellular process(GO:0009987)	1

OG0033783	Biological Process	metabolic process(GO:0008152)	1
OG0033783	Biological Process	regulation of biological process(GO:0050789)	1
OG0033790	Biological Process	cellular process(GO:0009987)	1
OG0033790	Biological Process	localization(GO:0051179)	1
OG0033790	Biological Process	metabolic process(GO:0008152)	1
OG0033791	Biological Process	cellular process(GO:0009987)	1
OG0033791	Biological Process	growth(GO:0040007)	1
OG0033791	Biological Process	metabolic process(GO:0008152)	1
OG0033792	Biological Process	cellular process(GO:0009987)	1
OG0033792	Biological Process	metabolic process(GO:0008152)	1
OG0033802	Biological Process	biological regulation(GO:0065007)	1
OG0033802	Biological Process	cellular process(GO:0009987)	1
OG0033802	Biological Process	developmental process(GO:0032502)	1
OG0033802	Biological Process	multicellular organismal process(GO:0032501)	1
OG0033802	Biological Process	regulation of biological process(GO:0050789)	1
OG0033802	Biological Process	response to stimulus(GO:0050896)	1
OG0033802	Biological Process	signaling(GO:0023052)	1
OG0033803	Biological Process	metabolic process(GO:0008152)	1
OG0033804	Biological Process	cellular process(GO:0009987)	1
OG0033804	Biological Process	metabolic process(GO:0008152)	1
OG0033804	Biological Process	response to stimulus(GO:0050896)	1
OG0033806	Biological Process	response to stimulus(GO:0050896)	1
OG0033810	Biological Process	metabolic process(GO:0008152)	1
OG0033810	Biological Process	multi-organism process(GO:0051704)	1
OG0033810	Biological Process	response to stimulus(GO:0050896)	1
OG0033826	Biological Process	cellular component organization or biogenesis(GO:0071840)	1
OG0033826	Biological Process	cellular process(GO:0009987)	1
OG0033826	Biological Process	metabolic process(GO:0008152)	1
OG0033828	Biological Process	growth(GO:0040007)	1
OG0033830	Biological Process	growth(GO:0040007)	1
OG0033842	Biological Process	cellular component organization or biogenesis(GO:0071840)	1
OG0033842	Biological Process	cellular process(GO:0009987)	1
OG0033842	Biological Process	metabolic process(GO:0008152)	1
OG0033842	Biological Process	response to stimulus(GO:0050896)	1
OG0033844	Biological Process	cellular component organization or biogenesis(GO:0071840)	1
OG0033844	Biological Process	cellular process(GO:0009987)	1
OG0033844	Biological Process	metabolic process(GO:0008152)	1
OG0033844	Biological Process	response to stimulus(GO:0050896)	1
OG0033846	Biological Process	cellular component organization or biogenesis(GO:0071840)	1
OG0033846	Biological Process	cellular process(GO:0009987)	1
OG0033846	Biological Process	metabolic process(GO:0008152)	1
OG0033846	Biological Process	response to stimulus(GO:0050896)	1
OG0033847	Biological Process	cellular component organization or biogenesis(GO:0071840)	1
OG0033847	Biological Process	cellular process(GO:0009987)	1
OG0033847	Biological Process	metabolic process(GO:0008152)	1

OG0033847	Biological Process	response to stimulus(GO:0050896)	1
OG0033856	Biological Process	biological regulation(GO:0065007)	1
OG0033856	Biological Process	cellular process(GO:0009987)	1
OG0033856	Biological Process	localization(GO:0051179)	1
OG0033856	Biological Process	regulation of biological process(GO:0050789)	1
OG0033856	Biological Process	response to stimulus(GO:0050896)	1
OG0033856	Biological Process	signaling(GO:0023052)	1
OG0033865	Biological Process	cellular process(GO:0009987)	1
OG0033865	Biological Process	metabolic process(GO:0008152)	1
OG0033867	Biological Process	cellular process(GO:0009987)	1
OG0033867	Biological Process	metabolic process(GO:0008152)	1
OG0033872	Biological Process	developmental process(GO:0032502)	1
OG0033872	Biological Process	multicellular organismal process(GO:0032501)	1
OG0033872	Biological Process	reproduction(GO:0000003)	1
OG0033872	Biological Process	reproductive process(GO:0022414)	1
OG0033875	Biological Process	cellular component organization or biogenesis(GO:0071840)	1
OG0033875	Biological Process	cellular process(GO:0009987)	1
OG0033875	Biological Process	metabolic process(GO:0008152)	1
OG0033877	Biological Process	cellular process(GO:0009987)	1
OG0033877	Biological Process	metabolic process(GO:0008152)	1
OG0033898	Biological Process	biological regulation(GO:0065007)	1
OG0033898	Biological Process	localization(GO:0051179)	1
OG0033898	Biological Process	metabolic process(GO:0008152)	1
OG0033898	Biological Process	regulation of biological process(GO:0050789)	1
OG0033898	Biological Process	response to stimulus(GO:0050896)	1
OG0033899	Biological Process	biological regulation(GO:0065007)	1
OG0033899	Biological Process	localization(GO:0051179)	1
OG0033899	Biological Process	metabolic process(GO:0008152)	1
OG0033899	Biological Process	regulation of biological process(GO:0050789)	1
OG0033899	Biological Process	response to stimulus(GO:0050896)	1
OG0033900	Biological Process	biological regulation(GO:0065007)	1
OG0033900	Biological Process	localization(GO:0051179)	1
OG0033900	Biological Process	metabolic process(GO:0008152)	1
OG0033900	Biological Process	regulation of biological process(GO:0050789)	1
OG0033900	Biological Process	response to stimulus(GO:0050896)	1
OG0033910	Biological Process	biological regulation(GO:0065007)	1
OG0033910	Biological Process	cellular process(GO:0009987)	1
OG0033910	Biological Process	developmental process(GO:0032502)	1
OG0033910	Biological Process	metabolic process(GO:0008152)	1
OG0033910	Biological Process	multicellular organismal process(GO:0032501)	1
OG0033910	Biological Process	regulation of biological process(GO:0050789)	1
OG0033910	Biological Process	response to stimulus(GO:0050896)	1
OG0033918	Biological Process	cellular process(GO:0009987)	1
OG0033918	Biological Process	metabolic process(GO:0008152)	1
OG0033933	Biological Process	biological regulation(GO:0065007)	1

OG0033933	Biological Process	cellular process(GO:0009987)	1
OG0033933	Biological Process	developmental process(GO:0032502)	1
OG0033933	Biological Process	metabolic process(GO:0008152)	1
OG0033933	Biological Process	multi-organism process(GO:0051704)	1
OG0033933	Biological Process	multicellular organismal process(GO:0032501)	1
OG0033933	Biological Process	regulation of biological process(GO:0050789)	1
OG0033933	Biological Process	reproduction(GO:0000003)	1
OG0033933	Biological Process	reproductive process(GO:0022414)	1
OG0033933	Biological Process	response to stimulus(GO:0050896)	1
OG0033944	Biological Process	biological regulation(GO:0065007)	1
OG0033944	Biological Process	cellular process(GO:0009987)	1
OG0033944	Biological Process	metabolic process(GO:0008152)	1
OG0033944	Biological Process	multi-organism process(GO:0051704)	1
OG0033944	Biological Process	positive regulation of biological process(GO:0048518)	1
OG0033944	Biological Process	regulation of biological process(GO:0050789)	1
OG0033944	Biological Process	response to stimulus(GO:0050896)	1
OG0033947	Biological Process	biological regulation(GO:0065007)	1
OG0033947	Biological Process	cellular component organization or biogenesis(GO:0071840)	1
OG0033947	Biological Process	cellular process(GO:0009987)	1
OG0033947	Biological Process	developmental process(GO:0032502)	1
OG0033947	Biological Process	localization(GO:0051179)	1
OG0033947	Biological Process	metabolic process(GO:0008152)	1
OG0033947	Biological Process	multicellular organismal process(GO:0032501)	1
OG0033947	Biological Process	regulation of biological process(GO:0050789)	1
OG0033947	Biological Process	response to stimulus(GO:0050896)	1
OG0033947	Biological Process	rhythmic process(GO:0048511)	1
OG0033947	Biological Process	signaling(GO:0023052)	1
OG0033956	Biological Process	cellular process(GO:0009987)	1
OG0033956	Biological Process	metabolic process(GO:0008152)	1
OG0033957	Biological Process	growth(GO:0040007)	1
OG0033957	Biological Process	metabolic process(GO:0008152)	1
OG0033957	Biological Process	multi-organism process(GO:0051704)	1
OG0033957	Biological Process	response to stimulus(GO:0050896)	1
OG0033979	Biological Process	metabolic process(GO:0008152)	1
OG0033987	Biological Process	biological regulation(GO:0065007)	1
OG0033987	Biological Process	cellular process(GO:0009987)	1
OG0033987	Biological Process	regulation of biological process(GO:0050789)	1
OG0033987	Biological Process	response to stimulus(GO:0050896)	1
OG0033988	Biological Process	biological regulation(GO:0065007)	1
OG0033988	Biological Process	cellular process(GO:0009987)	1
OG0033988	Biological Process	immune system process(GO:0002376)	1
OG0033988	Biological Process	multi-organism process(GO:0051704)	1
OG0033988	Biological Process	regulation of biological process(GO:0050789)	1
OG0033988	Biological Process	response to stimulus(GO:0050896)	1
OG0033989	Biological Process	metabolic process(GO:0008152)	1

OG0033991	Biological Process	cellular process(GO:0009987)	1
OG0033991	Biological Process	localization(GO:0051179)	1
OG0033991	Biological Process	metabolic process(GO:0008152)	1
OG0034003	Biological Process	cellular process(GO:0009987)	1
OG0034003	Biological Process	metabolic process(GO:0008152)	1
OG0034004	Biological Process	biological regulation(GO:0065007)	1
OG0034004	Biological Process	cellular process(GO:0009987)	1
OG0034004	Biological Process	metabolic process(GO:0008152)	1
OG0034004	Biological Process	regulation of biological process(GO:0050789)	1
OG0034004	Biological Process	response to stimulus(GO:0050896)	1
OG0034004	Biological Process	rhythmic process(GO:0048511)	1
OG0034004	Biological Process	signaling(GO:0023052)	1
OG0034013	Biological Process	response to stimulus(GO:0050896)	1
OG0034030	Biological Process	metabolic process(GO:0008152)	1
OG0034038	Biological Process	cellular process(GO:0009987)	1
OG0034038	Biological Process	developmental process(GO:0032502)	1
OG0034038	Biological Process	multicellular organismal process(GO:0032501)	1
OG0034045	Biological Process	cellular process(GO:0009987)	1
OG0034045	Biological Process	metabolic process(GO:0008152)	1
OG0034051	Biological Process	biological regulation(GO:0065007)	1
OG0034051	Biological Process	cellular process(GO:0009987)	1
OG0034051	Biological Process	developmental process(GO:0032502)	1
OG0034051	Biological Process	immune system process(GO:0002376)	1
OG0034051	Biological Process	metabolic process(GO:0008152)	1
OG0034051	Biological Process	multi-organism process(GO:0051704)	1
OG0034051	Biological Process	multicellular organismal process(GO:0032501)	1
OG0034051	Biological Process	negative regulation of biological process(GO:0048519)	1
OG0034051	Biological Process	regulation of biological process(GO:0050789)	1
OG0034051	Biological Process	reproduction(GO:0000003)	1
OG0034051	Biological Process	reproductive process(GO:0022414)	1
OG0034051	Biological Process	response to stimulus(GO:0050896)	1
OG0034051	Biological Process	signaling(GO:0023052)	1
OG0034055	Biological Process	biological regulation(GO:0065007)	1
OG0034055	Biological Process	cellular process(GO:0009987)	1
OG0034055	Biological Process	metabolic process(GO:0008152)	1
OG0034055	Biological Process	multi-organism process(GO:0051704)	1
OG0034055	Biological Process	regulation of biological process(GO:0050789)	1
OG0034055	Biological Process	response to stimulus(GO:0050896)	1
OG0034055	Biological Process	signaling(GO:0023052)	1
OG0034056	Biological Process	biological regulation(GO:0065007)	1
OG0034056	Biological Process	cellular process(GO:0009987)	1
OG0034056	Biological Process	metabolic process(GO:0008152)	1
OG0034056	Biological Process	multi-organism process(GO:0051704)	1
OG0034056	Biological Process	regulation of biological process(GO:0050789)	1
OG0034056	Biological Process	response to stimulus(GO:0050896)	1

OG0034060	Biological Process	cellular component organization or biogenesis(GO:0071840)	1
OG0034060	Biological Process	cellular process(GO:0009987)	1
OG0034060	Biological Process	developmental process(GO:0032502)	1
OG0034060	Biological Process	multicellular organismal process(GO:0032501)	1
OG0034069	Biological Process	cellular process(GO:0009987)	1
OG0034069	Biological Process	growth(GO:0040007)	1
OG0034069	Biological Process	metabolic process(GO:0008152)	1
OG0034072	Biological Process	cellular process(GO:0009987)	1
OG0034072	Biological Process	growth(GO:0040007)	1
OG0034072	Biological Process	metabolic process(GO:0008152)	1
OG0034076	Biological Process	cellular process(GO:0009987)	1
OG0034076	Biological Process	growth(GO:0040007)	1
OG0034076	Biological Process	metabolic process(GO:0008152)	1
OG0034078	Biological Process	cellular component organization or biogenesis(GO:0071840)	1
OG0034078	Biological Process	cellular process(GO:0009987)	1
OG0034078	Biological Process	growth(GO:0040007)	1
OG0034078	Biological Process	metabolic process(GO:0008152)	1
OG0034078	Biological Process	reproduction(GO:0000003)	1
OG0034078	Biological Process	reproductive process(GO:0022414)	1
OG0034079	Biological Process	cellular process(GO:0009987)	1
OG0034079	Biological Process	localization(GO:0051179)	1
OG0034081	Biological Process	biological adhesion(GO:0022610)	1
OG0034081	Biological Process	cellular component organization or biogenesis(GO:0071840)	1
OG0034081	Biological Process	cellular process(GO:0009987)	1
OG0034081	Biological Process	growth(GO:0040007)	1
OG0034081	Biological Process	metabolic process(GO:0008152)	1
OG0034081	Biological Process	multi-organism process(GO:0051704)	1
OG0034081	Biological Process	reproduction(GO:0000003)	1
OG0034081	Biological Process	response to stimulus(GO:0050896)	1
OG0034089	Biological Process	growth(GO:0040007)	1
OG0034090	Biological Process	cellular component organization or biogenesis(GO:0071840)	1
OG0034090	Biological Process	cellular process(GO:0009987)	1
OG0034090	Biological Process	metabolic process(GO:0008152)	1
OG0034091	Biological Process	cellular process(GO:0009987)	1
OG0034091	Biological Process	growth(GO:0040007)	1
OG0034091	Biological Process	metabolic process(GO:0008152)	1
OG0034092	Biological Process	cellular component organization or biogenesis(GO:0071840)	1
OG0034092	Biological Process	cellular process(GO:0009987)	1
OG0034092	Biological Process	growth(GO:0040007)	1
OG0034092	Biological Process	localization(GO:0051179)	1
OG0034092	Biological Process	metabolic process(GO:0008152)	1
OG0034093	Biological Process	cellular process(GO:0009987)	1
OG0034093	Biological Process	metabolic process(GO:0008152)	1
OG0034096	Biological Process	cellular process(GO:0009987)	1
OG0034096	Biological Process	growth(GO:0040007)	1
OG0034096	Biological Process	metabolic process(GO:0008152)	1

OG0034097	Biological Process	cellular process(GO:0009987)	1
OG0034097	Biological Process	metabolic process(GO:0008152)	1
OG0034098	Biological Process	cellular component organization or biogenesis(GO:0071840)	1
OG0034098	Biological Process	cellular process(GO:0009987)	1
OG0034098	Biological Process	growth(GO:0040007)	1
OG0034098	Biological Process	metabolic process(GO:0008152)	1
OG0034100	Biological Process	cellular process(GO:0009987)	1
OG0034100	Biological Process	growth(GO:0040007)	1
OG0034100	Biological Process	metabolic process(GO:0008152)	1
OG0034101	Biological Process	cellular component organization or biogenesis(GO:0071840)	1
OG0034101	Biological Process	cellular process(GO:0009987)	1
OG0034101	Biological Process	growth(GO:0040007)	1
OG0034101	Biological Process	metabolic process(GO:0008152)	1
OG0034102	Biological Process	cellular component organization or biogenesis(GO:0071840)	1
OG0034102	Biological Process	cellular process(GO:0009987)	1
OG0034102	Biological Process	metabolic process(GO:0008152)	1
OG0034103	Biological Process	cellular process(GO:0009987)	1
OG0034103	Biological Process	metabolic process(GO:0008152)	1
OG0034105	Biological Process	cellular process(GO:0009987)	1
OG0034105	Biological Process	metabolic process(GO:0008152)	1
OG0034106	Biological Process	cellular process(GO:0009987)	1
OG0034106	Biological Process	localization(GO:0051179)	1
OG0034106	Biological Process	locomotion(GO:0040011)	1
OG0034108	Biological Process	cellular process(GO:0009987)	1
OG0034108	Biological Process	metabolic process(GO:0008152)	1
OG0034110	Biological Process	growth(GO:0040007)	1
OG0034113	Biological Process	cellular process(GO:0009987)	1
OG0034113	Biological Process	metabolic process(GO:0008152)	1
OG0034114	Biological Process	cellular process(GO:0009987)	1
OG0034114	Biological Process	metabolic process(GO:0008152)	1
OG0034115	Biological Process	cellular process(GO:0009987)	1
OG0034115	Biological Process	localization(GO:0051179)	1
OG0034115	Biological Process	metabolic process(GO:0008152)	1
OG0034118	Biological Process	biological adhesion(GO:0022610)	1
OG0034118	Biological Process	cellular component organization or biogenesis(GO:0071840)	1
OG0034118	Biological Process	cellular process(GO:0009987)	1
OG0034118	Biological Process	growth(GO:0040007)	1
OG0034118	Biological Process	metabolic process(GO:0008152)	1
OG0034118	Biological Process	multi-organism process(GO:0051704)	1
OG0034118	Biological Process	reproduction(GO:0000003)	1
OG0034118	Biological Process	response to stimulus(GO:0050896)	1
OG0034123	Biological Process	cellular process(GO:0009987)	1
OG0034123	Biological Process	metabolic process(GO:0008152)	1
OG0034131	Biological Process	cellular process(GO:0009987)	1
OG0034131	Biological Process	metabolic process(GO:0008152)	1
OG0034136	Biological Process	cellular process(GO:0009987)	1

OG0034136	Biological Process	growth(GO:0040007)	1
OG0034136	Biological Process	metabolic process(GO:0008152)	1
OG0034137	Biological Process	biological regulation(GO:0065007)	1
OG0034137	Biological Process	cellular process(GO:0009987)	1
OG0034137	Biological Process	metabolic process(GO:0008152)	1
OG0034141	Biological Process	cellular process(GO:0009987)	1
OG0034141	Biological Process	metabolic process(GO:0008152)	1
OG0034142	Biological Process	cellular process(GO:0009987)	1
OG0034142	Biological Process	localization(GO:0051179)	1
OG0034142	Biological Process	locomotion(GO:0040011)	1
OG0034143	Biological Process	biological regulation(GO:0065007)	1
OG0034143	Biological Process	cellular process(GO:0009987)	1
OG0034143	Biological Process	metabolic process(GO:0008152)	1
OG0034144	Biological Process	cellular process(GO:0009987)	1
OG0034144	Biological Process	metabolic process(GO:0008152)	1
OG0034146	Biological Process	biological regulation(GO:0065007)	1
OG0034146	Biological Process	cellular process(GO:0009987)	1
OG0034146	Biological Process	response to stimulus(GO:0050896)	1
OG0034147	Biological Process	cellular process(GO:0009987)	1
OG0034147	Biological Process	metabolic process(GO:0008152)	1
OG0034153	Biological Process	cellular process(GO:0009987)	1
OG0034153	Biological Process	metabolic process(GO:0008152)	1
OG0034155	Biological Process	biological regulation(GO:0065007)	1
OG0034155	Biological Process	cellular process(GO:0009987)	1
OG0034155	Biological Process	growth(GO:0040007)	1
OG0034155	Biological Process	metabolic process(GO:0008152)	1
OG0034155	Biological Process	negative regulation of biological process(GO:0048519)	1
OG0034155	Biological Process	regulation of biological process(GO:0050789)	1
OG0034157	Biological Process	cellular process(GO:0009987)	1
OG0034157	Biological Process	growth(GO:0040007)	1
OG0034157	Biological Process	localization(GO:0051179)	1
OG0034157	Biological Process	metabolic process(GO:0008152)	1
OG0034158	Biological Process	cellular process(GO:0009987)	1
OG0034158	Biological Process	growth(GO:0040007)	1
OG0034158	Biological Process	localization(GO:0051179)	1
OG0034158	Biological Process	metabolic process(GO:0008152)	1
OG0034159	Biological Process	cellular process(GO:0009987)	1
OG0034159	Biological Process	growth(GO:0040007)	1
OG0034159	Biological Process	metabolic process(GO:0008152)	1
OG0034161	Biological Process	cellular process(GO:0009987)	1
OG0034161	Biological Process	metabolic process(GO:0008152)	1
OG0034162	Biological Process	cellular process(GO:0009987)	1
OG0034162	Biological Process	metabolic process(GO:0008152)	1
OG0034163	Biological Process	cellular component organization or biogenesis(GO:0071840)	1
OG0034163	Biological Process	cellular process(GO:0009987)	1
OG0034163	Biological Process	growth(GO:0040007)	1

OG0034163	Biological Process	metabolic process(GO:0008152)	1
OG0034166	Biological Process	cellular process(GO:0009987)	1
OG0034166	Biological Process	metabolic process(GO:0008152)	1
OG0034172	Biological Process	metabolic process(GO:0008152)	1
OG0034173	Biological Process	cellular process(GO:0009987)	1
OG0034173	Biological Process	metabolic process(GO:0008152)	1
OG0034182	Biological Process	cellular process(GO:0009987)	1
OG0034182	Biological Process	growth(GO:0040007)	1
OG0034182	Biological Process	localization(GO:0051179)	1
OG0034182	Biological Process	metabolic process(GO:0008152)	1
OG0034182	Biological Process	multi-organism process(GO:0051704)	1
OG0034182	Biological Process	response to stimulus(GO:0050896)	1
OG0034183	Biological Process	cellular component organization or biogenesis(GO:0071840)	1
OG0034183	Biological Process	cellular process(GO:0009987)	1
OG0034183	Biological Process	metabolic process(GO:0008152)	1
OG0034188	Biological Process	cellular process(GO:0009987)	1
OG0034188	Biological Process	metabolic process(GO:0008152)	1
OG0034189	Biological Process	biological regulation(GO:0065007)	1
OG0034189	Biological Process	cellular process(GO:0009987)	1
OG0034189	Biological Process	developmental process(GO:0032502)	1
OG0034189	Biological Process	growth(GO:0040007)	1
OG0034189	Biological Process	metabolic process(GO:0008152)	1
OG0034189	Biological Process	regulation of biological process(GO:0050789)	1
OG0034189	Biological Process	response to stimulus(GO:0050896)	1
OG0034190	Biological Process	cellular process(GO:0009987)	1
OG0034190	Biological Process	metabolic process(GO:0008152)	1
OG0034192	Biological Process	cellular process(GO:0009987)	1
OG0034192	Biological Process	metabolic process(GO:0008152)	1
OG0034195	Biological Process	cellular process(GO:0009987)	1
OG0034195	Biological Process	growth(GO:0040007)	1
OG0034195	Biological Process	metabolic process(GO:0008152)	1
OG0034197	Biological Process	cellular process(GO:0009987)	1
OG0034197	Biological Process	metabolic process(GO:0008152)	1
OG0034198	Biological Process	biological regulation(GO:0065007)	1
OG0034198	Biological Process	cellular component organization or biogenesis(GO:0071840)	1
OG0034198	Biological Process	cellular process(GO:0009987)	1
OG0034198	Biological Process	developmental process(GO:0032502)	1
OG0034198	Biological Process	metabolic process(GO:0008152)	1
OG0034198	Biological Process	regulation of biological process(GO:0050789)	1
OG0034198	Biological Process	response to stimulus(GO:0050896)	1
OG0034200	Biological Process	growth(GO:0040007)	1
OG0034200	Biological Process	metabolic process(GO:0008152)	1
OG0034201	Biological Process	cellular process(GO:0009987)	1
OG0034201	Biological Process	metabolic process(GO:0008152)	1
OG0034203	Biological Process	cellular process(GO:0009987)	1
OG0034203	Biological Process	localization(GO:0051179)	1

OG0034203	Biological Process	metabolic process(GO:0008152)	1
OG0034209	Biological Process	cellular process(GO:0009987)	1
OG0034209	Biological Process	growth(GO:0040007)	1
OG0034209	Biological Process	metabolic process(GO:0008152)	1
OG0034212	Biological Process	cellular process(GO:0009987)	1
OG0034212	Biological Process	metabolic process(GO:0008152)	1
OG0034213	Biological Process	cellular process(GO:0009987)	1
OG0034213	Biological Process	localization(GO:0051179)	1
OG0034215	Biological Process	cellular process(GO:0009987)	1
OG0034215	Biological Process	growth(GO:0040007)	1
OG0034215	Biological Process	metabolic process(GO:0008152)	1
OG0034216	Biological Process	cellular process(GO:0009987)	1
OG0034216	Biological Process	localization(GO:0051179)	1
OG0034216	Biological Process	locomotion(GO:0040011)	1
OG0034216	Biological Process	metabolic process(GO:0008152)	1
OG0034216	Biological Process	response to stimulus(GO:0050896)	1
OG0034218	Biological Process	biological regulation(GO:0065007)	1
OG0034218	Biological Process	cellular component organization or biogenesis(GO:0071840)	1
OG0034218	Biological Process	cellular process(GO:0009987)	1
OG0034218	Biological Process	growth(GO:0040007)	1
OG0034218	Biological Process	metabolic process(GO:0008152)	1
OG0034218	Biological Process	regulation of biological process(GO:0050789)	1
OG0034219	Biological Process	cellular component organization or biogenesis(GO:0071840)	1
OG0034219	Biological Process	cellular process(GO:0009987)	1
OG0034219	Biological Process	growth(GO:0040007)	1
OG0034219	Biological Process	metabolic process(GO:0008152)	1
OG0034219	Biological Process	multi-organism process(GO:0051704)	1
OG0034219	Biological Process	response to stimulus(GO:0050896)	1
OG0034220	Biological Process	growth(GO:0040007)	1
OG0034223	Biological Process	metabolic process(GO:0008152)	1
OG0034225	Biological Process	biological regulation(GO:0065007)	1
OG0034225	Biological Process	cellular process(GO:0009987)	1
OG0034225	Biological Process	developmental process(GO:0032502)	1
OG0034225	Biological Process	growth(GO:0040007)	1
OG0034225	Biological Process	metabolic process(GO:0008152)	1
OG0034225	Biological Process	regulation of biological process(GO:0050789)	1
OG0034225	Biological Process	response to stimulus(GO:0050896)	1
OG0034227	Biological Process	cellular process(GO:0009987)	1
OG0034227	Biological Process	metabolic process(GO:0008152)	1
OG0034228	Biological Process	cellular process(GO:0009987)	1
OG0034228	Biological Process	metabolic process(GO:0008152)	1
OG0034229	Biological Process	cellular process(GO:0009987)	1
OG0034229	Biological Process	metabolic process(GO:0008152)	1
OG0034233	Biological Process	cellular process(GO:0009987)	1
OG0034233	Biological Process	growth(GO:0040007)	1
OG0034233	Biological Process	metabolic process(GO:0008152)	1

OG0034238	Biological Process	cellular component organization or biogenesis(GO:0071840)	1
OG0034238	Biological Process	cellular process(GO:0009987)	1
OG0034238	Biological Process	metabolic process(GO:0008152)	1
OG0034239	Biological Process	cellular process(GO:0009987)	1
OG0034239	Biological Process	growth(GO:0040007)	1
OG0034239	Biological Process	metabolic process(GO:0008152)	1
OG0034247	Biological Process	growth(GO:0040007)	1
OG0034247	Biological Process	metabolic process(GO:0008152)	1
OG0034247	Biological Process	multi-organism process(GO:0051704)	1
OG0034247	Biological Process	response to stimulus(GO:0050896)	1
OG0034250	Biological Process	biological regulation(GO:0065007)	1
OG0034250	Biological Process	cellular process(GO:0009987)	1
OG0034250	Biological Process	metabolic process(GO:0008152)	1
OG0034250	Biological Process	negative regulation of biological process(GO:0048519)	1
OG0034250	Biological Process	regulation of biological process(GO:0050789)	1
OG0034250	Biological Process	response to stimulus(GO:0050896)	1
OG0034251	Biological Process	cellular process(GO:0009987)	1
OG0034251	Biological Process	metabolic process(GO:0008152)	1
OG0034251	Biological Process	response to stimulus(GO:0050896)	1
OG0034252	Biological Process	cellular process(GO:0009987)	1
OG0034252	Biological Process	growth(GO:0040007)	1
OG0034252	Biological Process	metabolic process(GO:0008152)	1
OG0034252	Biological Process	response to stimulus(GO:0050896)	1
OG0034254	Biological Process	cellular component organization or biogenesis(GO:0071840)	1
OG0034254	Biological Process	cellular process(GO:0009987)	1
OG0034254	Biological Process	metabolic process(GO:0008152)	1
OG0034254	Biological Process	response to stimulus(GO:0050896)	1
OG0034256	Biological Process	cellular process(GO:0009987)	1
OG0034256	Biological Process	signaling(GO:0023052)	1
OG0034258	Biological Process	cellular process(GO:0009987)	1
OG0034258	Biological Process	metabolic process(GO:0008152)	1
OG0034258	Biological Process	response to stimulus(GO:0050896)	1
OG0034261	Biological Process	cellular process(GO:0009987)	1
OG0034261	Biological Process	growth(GO:0040007)	1
OG0034261	Biological Process	metabolic process(GO:0008152)	1
OG0034262	Biological Process	cellular process(GO:0009987)	1
OG0034262	Biological Process	metabolic process(GO:0008152)	1
OG0034264	Biological Process	growth(GO:0040007)	1
OG0034264	Biological Process	metabolic process(GO:0008152)	1
OG0034268	Biological Process	cellular process(GO:0009987)	1
OG0034268	Biological Process	growth(GO:0040007)	1
OG0034268	Biological Process	localization(GO:0051179)	1
OG0034269	Biological Process	cellular component organization or biogenesis(GO:0071840)	1
OG0034269	Biological Process	cellular process(GO:0009987)	1
OG0034269	Biological Process	metabolic process(GO:0008152)	1
OG0034278	Biological Process	cellular component organization or biogenesis(GO:0071840)	1

OG0034278	Biological Process	cellular process(GO:0009987)	1
OG0034278	Biological Process	growth(GO:0040007)	1
OG0034278	Biological Process	metabolic process(GO:0008152)	1
OG0034279	Biological Process	cellular process(GO:0009987)	1
OG0034279	Biological Process	metabolic process(GO:0008152)	1
OG0034281	Biological Process	cellular component organization or biogenesis(GO:0071840)	1
OG0034281	Biological Process	cellular process(GO:0009987)	1
OG0034281	Biological Process	metabolic process(GO:0008152)	1
OG0034283	Biological Process	cellular process(GO:0009987)	1
OG0034283	Biological Process	growth(GO:0040007)	1
OG0034283	Biological Process	metabolic process(GO:0008152)	1
OG0034284	Biological Process	cellular component organization or biogenesis(GO:0071840)	1
OG0034284	Biological Process	cellular process(GO:0009987)	1
OG0034284	Biological Process	growth(GO:0040007)	1
OG0034284	Biological Process	metabolic process(GO:0008152)	1
OG0034285	Biological Process	biological regulation(GO:0065007)	1
OG0034285	Biological Process	cellular process(GO:0009987)	1
OG0034285	Biological Process	metabolic process(GO:0008152)	1
OG0034285	Biological Process	negative regulation of biological process(GO:0048519)	1
OG0034285	Biological Process	regulation of biological process(GO:0050789)	1
OG0034286	Biological Process	cellular process(GO:0009987)	1
OG0034286	Biological Process	growth(GO:0040007)	1
OG0034286	Biological Process	metabolic process(GO:0008152)	1
OG0034286	Biological Process	multi-organism process(GO:0051704)	1
OG0034286	Biological Process	response to stimulus(GO:0050896)	1
OG0034291	Biological Process	cellular component organization or biogenesis(GO:0071840)	1
OG0034291	Biological Process	cellular process(GO:0009987)	1
OG0034291	Biological Process	metabolic process(GO:0008152)	1
OG0034291	Biological Process	response to stimulus(GO:0050896)	1
OG0034295	Biological Process	cellular process(GO:0009987)	1
OG0034295	Biological Process	growth(GO:0040007)	1
OG0034295	Biological Process	metabolic process(GO:0008152)	1
OG0034298	Biological Process	biological regulation(GO:0065007)	1
OG0034298	Biological Process	cellular process(GO:0009987)	1
OG0034298	Biological Process	metabolic process(GO:0008152)	1
OG0034298	Biological Process	regulation of biological process(GO:0050789)	1
OG0034299	Biological Process	localization(GO:0051179)	1
OG0034299	Biological Process	response to stimulus(GO:0050896)	1
OG0034306	Biological Process	cellular process(GO:0009987)	1
OG0034306	Biological Process	metabolic process(GO:0008152)	1
OG0034307	Biological Process	cellular process(GO:0009987)	1
OG0034307	Biological Process	metabolic process(GO:0008152)	1
OG0034308	Biological Process	cellular process(GO:0009987)	1
OG0034308	Biological Process	growth(GO:0040007)	1
OG0034308	Biological Process	metabolic process(GO:0008152)	1
OG0034309	Biological Process	biological regulation(GO:0065007)	1

OG0034309	Biological Process	cellular component organization or biogenesis(GO:0071840)	1
OG0034309	Biological Process	cellular process(GO:0009987)	1
OG0034309	Biological Process	growth(GO:0040007)	1
OG0034309	Biological Process	metabolic process(GO:0008152)	1
OG0034309	Biological Process	positive regulation of biological process(GO:0048518)	1
OG0034309	Biological Process	regulation of biological process(GO:0050789)	1
OG0034310	Biological Process	cellular process(GO:0009987)	1
OG0034310	Biological Process	growth(GO:0040007)	1
OG0034310	Biological Process	metabolic process(GO:0008152)	1
OG0034311	Biological Process	cellular component organization or biogenesis(GO:0071840)	1
OG0034311	Biological Process	cellular process(GO:0009987)	1
OG0034311	Biological Process	growth(GO:0040007)	1
OG0034311	Biological Process	metabolic process(GO:0008152)	1
OG0034312	Biological Process	cellular process(GO:0009987)	1
OG0034312	Biological Process	growth(GO:0040007)	1
OG0034312	Biological Process	metabolic process(GO:0008152)	1
OG0034313	Biological Process	cellular process(GO:0009987)	1
OG0034313	Biological Process	growth(GO:0040007)	1
OG0034313	Biological Process	metabolic process(GO:0008152)	1
OG0034315	Biological Process	cellular process(GO:0009987)	1
OG0034315	Biological Process	metabolic process(GO:0008152)	1
OG0034316	Biological Process	cellular component organization or biogenesis(GO:0071840)	1
OG0034316	Biological Process	cellular process(GO:0009987)	1
OG0034316	Biological Process	growth(GO:0040007)	1
OG0034316	Biological Process	metabolic process(GO:0008152)	1
OG0034317	Biological Process	cellular process(GO:0009987)	1
OG0034317	Biological Process	metabolic process(GO:0008152)	1
OG0034323	Biological Process	cellular process(GO:0009987)	1
OG0034323	Biological Process	growth(GO:0040007)	1
OG0034323	Biological Process	metabolic process(GO:0008152)	1
OG0034326	Biological Process	metabolic process(GO:0008152)	1
OG0034333	Biological Process	cellular component organization or biogenesis(GO:0071840)	1
OG0034333	Biological Process	cellular process(GO:0009987)	1
OG0034333	Biological Process	growth(GO:0040007)	1
OG0034333	Biological Process	metabolic process(GO:0008152)	1
OG0034336	Biological Process	cellular process(GO:0009987)	1
OG0034336	Biological Process	detoxification(GO:0098754)	1
OG0034336	Biological Process	metabolic process(GO:0008152)	1
OG0034336	Biological Process	response to stimulus(GO:0050896)	1
OG0034348	Biological Process	cellular component organization or biogenesis(GO:0071840)	1
OG0034348	Biological Process	cellular process(GO:0009987)	1
OG0034348	Biological Process	metabolic process(GO:0008152)	1
OG0034350	Biological Process	cellular process(GO:0009987)	1
OG0034350	Biological Process	metabolic process(GO:0008152)	1
OG0034350	Biological Process	response to stimulus(GO:0050896)	1
OG0034356	Biological Process	cellular process(GO:0009987)	1

OG0034356	Biological Process	metabolic process(GO:0008152)	1
OG0034356	Biological Process	response to stimulus(GO:0050896)	1
OG0034365	Biological Process	cellular process(GO:0009987)	1
OG0034365	Biological Process	developmental process(GO:0032502)	1
OG0034383	Biological Process	biological regulation(GO:0065007)	1
OG0034383	Biological Process	cellular process(GO:0009987)	1
OG0034383	Biological Process	metabolic process(GO:0008152)	1
OG0034383	Biological Process	positive regulation of biological process(GO:0048518)	1
OG0034383	Biological Process	regulation of biological process(GO:0050789)	1
OG0034386	Biological Process	biological regulation(GO:0065007)	1
OG0034386	Biological Process	cell proliferation(GO:0008283)	1
OG0034386	Biological Process	cellular process(GO:0009987)	1
OG0034386	Biological Process	developmental process(GO:0032502)	1
OG0034386	Biological Process	metabolic process(GO:0008152)	1
OG0034386	Biological Process	multicellular organismal process(GO:0032501)	1
OG0034386	Biological Process	positive regulation of biological process(GO:0048518)	1
OG0034386	Biological Process	regulation of biological process(GO:0050789)	1
OG0034407	Biological Process	biological regulation(GO:0065007)	1
OG0034407	Biological Process	cellular process(GO:0009987)	1
OG0034407	Biological Process	metabolic process(GO:0008152)	1
OG0034407	Biological Process	regulation of biological process(GO:0050789)	1
OG0034410	Biological Process	biological regulation(GO:0065007)	1
OG0034410	Biological Process	cellular process(GO:0009987)	1
OG0034410	Biological Process	metabolic process(GO:0008152)	1
OG0034410	Biological Process	regulation of biological process(GO:0050789)	1
OG0034428	Biological Process	biological regulation(GO:0065007)	1
OG0034428	Biological Process	cellular process(GO:0009987)	1
OG0034428	Biological Process	immune system process(GO:0002376)	1
OG0034428	Biological Process	multi-organism process(GO:0051704)	1
OG0034428	Biological Process	positive regulation of biological process(GO:0048518)	1
OG0034428	Biological Process	regulation of biological process(GO:0050789)	1
OG0034428	Biological Process	response to stimulus(GO:0050896)	1
OG0034428	Biological Process	signaling(GO:0023052)	1
OG0034452	Biological Process	cellular component organization or biogenesis(GO:0071840)	1
OG0034452	Biological Process	cellular process(GO:0009987)	1
OG0034452	Biological Process	metabolic process(GO:0008152)	1
OG0034467	Biological Process	response to stimulus(GO:0050896)	1
OG0034468	Biological Process	response to stimulus(GO:0050896)	1
OG0034484	Biological Process	cellular process(GO:0009987)	1
OG0034484	Biological Process	developmental process(GO:0032502)	1
OG0034486	Biological Process	cellular process(GO:0009987)	1
OG0034486	Biological Process	developmental process(GO:0032502)	1
OG0034487	Biological Process	cellular process(GO:0009987)	1
OG0034487	Biological Process	metabolic process(GO:0008152)	1
OG0034489	Biological Process	cellular process(GO:0009987)	1

OG0034489	Biological Process	localization(GO:0051179)	1
OG0034497	Biological Process	biological regulation(GO:0065007)	1
OG0034497	Biological Process	cellular process(GO:0009987)	1
OG0034497	Biological Process	metabolic process(GO:0008152)	1
OG0034497	Biological Process	multi-organism process(GO:0051704)	1
OG0034497	Biological Process	response to stimulus(GO:0050896)	1
OG0034498	Biological Process	cellular component organization or biogenesis(GO:0071840)	1
OG0034498	Biological Process	cellular process(GO:0009987)	1
OG0034498	Biological Process	metabolic process(GO:0008152)	1
OG0034499	Biological Process	biological regulation(GO:0065007)	1
OG0034499	Biological Process	cellular process(GO:0009987)	1
OG0034499	Biological Process	regulation of biological process(GO:0050789)	1
OG0034499	Biological Process	response to stimulus(GO:0050896)	1
OG0034499	Biological Process	signaling(GO:0023052)	1
OG0034504	Biological Process	cellular process(GO:0009987)	1
OG0034504	Biological Process	growth(GO:0040007)	1
OG0034504	Biological Process	metabolic process(GO:0008152)	1
OG0034505	Biological Process	cellular process(GO:0009987)	1
OG0034505	Biological Process	metabolic process(GO:0008152)	1
OG0034505	Biological Process	response to stimulus(GO:0050896)	1
OG0034507	Biological Process	cellular component organization or biogenesis(GO:0071840)	1
OG0034507	Biological Process	cellular process(GO:0009987)	1
OG0034507	Biological Process	localization(GO:0051179)	1
OG0034507	Biological Process	locomotion(GO:0040011)	1
OG0034517	Biological Process	cellular process(GO:0009987)	1
OG0034517	Biological Process	metabolic process(GO:0008152)	1
OG0034522	Biological Process	cellular component organization or biogenesis(GO:0071840)	1
OG0034522	Biological Process	cellular process(GO:0009987)	1
OG0034525	Biological Process	biological regulation(GO:0065007)	1
OG0034525	Biological Process	cellular process(GO:0009987)	1
OG0034525	Biological Process	metabolic process(GO:0008152)	1
OG0034525	Biological Process	regulation of biological process(GO:0050789)	1
OG0034529	Biological Process	cellular process(GO:0009987)	1
OG0034529	Biological Process	growth(GO:0040007)	1
OG0034529	Biological Process	metabolic process(GO:0008152)	1
OG0034533	Biological Process	cellular process(GO:0009987)	1
OG0034533	Biological Process	metabolic process(GO:0008152)	1
OG0034539	Biological Process	cellular process(GO:0009987)	1
OG0034539	Biological Process	metabolic process(GO:0008152)	1
OG0034543	Biological Process	cellular process(GO:0009987)	1
OG0034543	Biological Process	metabolic process(GO:0008152)	1
OG0034543	Biological Process	response to stimulus(GO:0050896)	1
OG0034548	Biological Process	cellular process(GO:0009987)	1
OG0034548	Biological Process	metabolic process(GO:0008152)	1
OG0034549	Biological Process	cellular process(GO:0009987)	1
OG0034549	Biological Process	growth(GO:0040007)	1

OG0034549	Biological Process	metabolic process(GO:0008152)	1
OG0034553	Biological Process	cellular process(GO:0009987)	1
OG0034553	Biological Process	metabolic process(GO:0008152)	1
OG0034556	Biological Process	biological regulation(GO:0065007)	1
OG0034556	Biological Process	cellular process(GO:0009987)	1
OG0034556	Biological Process	regulation of biological process(GO:0050789)	1
OG0034557	Biological Process	biological regulation(GO:0065007)	1
OG0034557	Biological Process	cellular process(GO:0009987)	1
OG0034557	Biological Process	metabolic process(GO:0008152)	1
OG0034557	Biological Process	regulation of biological process(GO:0050789)	1
OG0034561	Biological Process	cellular process(GO:0009987)	1
OG0034561	Biological Process	metabolic process(GO:0008152)	1
OG0034562	Biological Process	cellular process(GO:0009987)	1
OG0034562	Biological Process	metabolic process(GO:0008152)	1
OG0034567	Biological Process	cellular process(GO:0009987)	1
OG0034567	Biological Process	metabolic process(GO:0008152)	1
OG0034568	Biological Process	cellular component organization or biogenesis(GO:0071840)	1
OG0034568	Biological Process	cellular process(GO:0009987)	1
OG0034568	Biological Process	metabolic process(GO:0008152)	1
OG0034572	Biological Process	biological regulation(GO:0065007)	1
OG0034572	Biological Process	cellular component organization or biogenesis(GO:0071840)	1
OG0034572	Biological Process	cellular process(GO:0009987)	1
OG0034572	Biological Process	metabolic process(GO:0008152)	1
OG0034572	Biological Process	regulation of biological process(GO:0050789)	1
OG0034572	Biological Process	reproduction(GO:0000003)	1
OG0034572	Biological Process	reproductive process(GO:0022414)	1
OG0034574	Biological Process	cellular process(GO:0009987)	1
OG0034574	Biological Process	metabolic process(GO:0008152)	1
OG0034576	Biological Process	cellular process(GO:0009987)	1
OG0034576	Biological Process	growth(GO:0040007)	1
OG0034576	Biological Process	metabolic process(GO:0008152)	1
OG0034577	Biological Process	cellular process(GO:0009987)	1
OG0034577	Biological Process	growth(GO:0040007)	1
OG0034577	Biological Process	metabolic process(GO:0008152)	1
OG0034581	Biological Process	cellular component organization or biogenesis(GO:0071840)	1
OG0034581	Biological Process	cellular process(GO:0009987)	1
OG0034581	Biological Process	growth(GO:0040007)	1
OG0034581	Biological Process	metabolic process(GO:0008152)	1
OG0034582	Biological Process	biological regulation(GO:0065007)	1
OG0034582	Biological Process	cellular process(GO:0009987)	1
OG0034582	Biological Process	metabolic process(GO:0008152)	1
OG0034582	Biological Process	negative regulation of biological process(GO:0048519)	1
OG0034582	Biological Process	regulation of biological process(GO:0050789)	1
OG0034583	Biological Process	cellular process(GO:0009987)	1
OG0034583	Biological Process	metabolic process(GO:0008152)	1

OG0034585	Biological Process	cellular process(GO:0009987)	1
OG0034585	Biological Process	growth(GO:0040007)	1
OG0034585	Biological Process	localization(GO:0051179)	1
OG0034585	Biological Process	multi-organism process(GO:0051704)	1
OG0034585	Biological Process	response to stimulus(GO:0050896)	1
OG0034586	Biological Process	cellular process(GO:0009987)	1
OG0034586	Biological Process	growth(GO:0040007)	1
OG0034586	Biological Process	metabolic process(GO:0008152)	1
OG0034587	Biological Process	cellular process(GO:0009987)	1
OG0034587	Biological Process	growth(GO:0040007)	1
OG0034587	Biological Process	metabolic process(GO:0008152)	1
OG0034588	Biological Process	cellular process(GO:0009987)	1
OG0034588	Biological Process	metabolic process(GO:0008152)	1
OG0034589	Biological Process	cellular process(GO:0009987)	1
OG0034589	Biological Process	metabolic process(GO:0008152)	1
OG0034590	Biological Process	cellular component organization or biogenesis(GO:0071840)	1
OG0034590	Biological Process	cellular process(GO:0009987)	1
OG0034590	Biological Process	growth(GO:0040007)	1
OG0034590	Biological Process	metabolic process(GO:0008152)	1
OG0034591	Biological Process	cellular process(GO:0009987)	1
OG0034591	Biological Process	growth(GO:0040007)	1
OG0034591	Biological Process	metabolic process(GO:0008152)	1
OG0034592	Biological Process	cellular process(GO:0009987)	1
OG0034592	Biological Process	growth(GO:0040007)	1
OG0034592	Biological Process	metabolic process(GO:0008152)	1
OG0034596	Biological Process	growth(GO:0040007)	1
OG0034599	Biological Process	cellular process(GO:0009987)	1
OG0034599	Biological Process	localization(GO:0051179)	1
OG0034599	Biological Process	metabolic process(GO:0008152)	1
OG0034599	Biological Process	response to stimulus(GO:0050896)	1
OG0034600	Biological Process	cellular process(GO:0009987)	1
OG0034600	Biological Process	metabolic process(GO:0008152)	1
OG0034600	Biological Process	response to stimulus(GO:0050896)	1
OG0034601	Biological Process	cellular process(GO:0009987)	1
OG0034601	Biological Process	metabolic process(GO:0008152)	1
OG0034608	Biological Process	biological regulation(GO:0065007)	1
OG0034608	Biological Process	cellular component organization or biogenesis(GO:0071840)	1
OG0034608	Biological Process	cellular process(GO:0009987)	1
OG0034608	Biological Process	growth(GO:0040007)	1
OG0034608	Biological Process	metabolic process(GO:0008152)	1
OG0034608	Biological Process	positive regulation of biological process(GO:0048518)	1
OG0034608	Biological Process	regulation of biological process(GO:0050789)	1
OG0034610	Biological Process	cellular component organization or biogenesis(GO:0071840)	1
OG0034610	Biological Process	cellular process(GO:0009987)	1
OG0034610	Biological Process	metabolic process(GO:0008152)	1
OG0034614	Biological Process	cellular process(GO:0009987)	1

OG0034614	Biological Process	metabolic process(GO:0008152)	1
OG0034614	Biological Process	multi-organism process(GO:0051704)	1
OG0034614	Biological Process	response to stimulus(GO:0050896)	1
OG0034623	Biological Process	cellular component organization or biogenesis(GO:0071840)	1
OG0034623	Biological Process	cellular process(GO:0009987)	1
OG0034623	Biological Process	localization(GO:0051179)	1
OG0034623	Biological Process	metabolic process(GO:0008152)	1
OG0034626	Biological Process	metabolic process(GO:0008152)	1
OG0034627	Biological Process	cellular process(GO:0009987)	1
OG0034627	Biological Process	metabolic process(GO:0008152)	1
OG0034627	Biological Process	response to stimulus(GO:0050896)	1
OG0034629	Biological Process	biological regulation(GO:0065007)	1
OG0034629	Biological Process	cellular component organization or biogenesis(GO:0071840)	1
OG0034629	Biological Process	cellular process(GO:0009987)	1
OG0034629	Biological Process	metabolic process(GO:0008152)	1
OG0034629	Biological Process	negative regulation of biological process(GO:0048519)	1
OG0034629	Biological Process	regulation of biological process(GO:0050789)	1
OG0034630	Biological Process	cellular component organization or biogenesis(GO:0071840)	1
OG0034630	Biological Process	cellular process(GO:0009987)	1
OG0034630	Biological Process	localization(GO:0051179)	1
OG0034630	Biological Process	response to stimulus(GO:0050896)	1
OG0034632	Biological Process	biological regulation(GO:0065007)	1
OG0034632	Biological Process	cellular component organization or biogenesis(GO:0071840)	1
OG0034632	Biological Process	cellular process(GO:0009987)	1
OG0034632	Biological Process	metabolic process(GO:0008152)	1
OG0034632	Biological Process	negative regulation of biological process(GO:0048519)	1
OG0034632	Biological Process	regulation of biological process(GO:0050789)	1
OG0034638	Biological Process	cellular process(GO:0009987)	1
OG0034638	Biological Process	metabolic process(GO:0008152)	1
OG0034643	Biological Process	cellular process(GO:0009987)	1
OG0034643	Biological Process	metabolic process(GO:0008152)	1
OG0034645	Biological Process	cellular process(GO:0009987)	1
OG0034645	Biological Process	localization(GO:0051179)	1
OG0034645	Biological Process	metabolic process(GO:0008152)	1
OG0034646	Biological Process	cellular process(GO:0009987)	1
OG0034646	Biological Process	localization(GO:0051179)	1
OG0034646	Biological Process	metabolic process(GO:0008152)	1
OG0034647	Biological Process	cellular process(GO:0009987)	1
OG0034647	Biological Process	growth(GO:0040007)	1
OG0034647	Biological Process	metabolic process(GO:0008152)	1
OG0034648	Biological Process	cellular process(GO:0009987)	1
OG0034648	Biological Process	metabolic process(GO:0008152)	1
OG0034650	Biological Process	cellular process(GO:0009987)	1
OG0034650	Biological Process	metabolic process(GO:0008152)	1
OG0034652	Biological Process	growth(GO:0040007)	1

OG0034652	Biological Process	metabolic process(GO:0008152)	1
OG0034652	Biological Process	multi-organism process(GO:0051704)	1
OG0034652	Biological Process	response to stimulus(GO:0050896)	1
OG0034658	Biological Process	cellular process(GO:0009987)	1
OG0034658	Biological Process	metabolic process(GO:0008152)	1
OG0034659	Biological Process	cellular process(GO:0009987)	1
OG0034659	Biological Process	metabolic process(GO:0008152)	1
OG0034670	Biological Process	cellular process(GO:0009987)	1
OG0034670	Biological Process	response to stimulus(GO:0050896)	1
OG0034672	Biological Process	biological regulation(GO:0065007)	1
OG0034672	Biological Process	cellular process(GO:0009987)	1
OG0034672	Biological Process	metabolic process(GO:0008152)	1
OG0034672	Biological Process	regulation of biological process(GO:0050789)	1
OG0034672	Biological Process	response to stimulus(GO:0050896)	1
OG0034673	Biological Process	biological regulation(GO:0065007)	1
OG0034673	Biological Process	cellular process(GO:0009987)	1
OG0034673	Biological Process	metabolic process(GO:0008152)	1
OG0034673	Biological Process	regulation of biological process(GO:0050789)	1
OG0034673	Biological Process	response to stimulus(GO:0050896)	1
OG0034675	Biological Process	biological regulation(GO:0065007)	1
OG0034675	Biological Process	cellular process(GO:0009987)	1
OG0034675	Biological Process	metabolic process(GO:0008152)	1
OG0034675	Biological Process	regulation of biological process(GO:0050789)	1
OG0034675	Biological Process	response to stimulus(GO:0050896)	1
OG0034676	Biological Process	biological regulation(GO:0065007)	1
OG0034676	Biological Process	cellular process(GO:0009987)	1
OG0034676	Biological Process	metabolic process(GO:0008152)	1
OG0034676	Biological Process	regulation of biological process(GO:0050789)	1
OG0034676	Biological Process	response to stimulus(GO:0050896)	1
OG0034699	Biological Process	cellular process(GO:0009987)	1
OG0034699	Biological Process	developmental process(GO:0032502)	1
OG0034699	Biological Process	metabolic process(GO:0008152)	1
OG0034699	Biological Process	nitrogen utilization(GO:0019740)	1
OG0034699	Biological Process	response to stimulus(GO:0050896)	1
OG0034705	Biological Process	cellular process(GO:0009987)	1
OG0034705	Biological Process	localization(GO:0051179)	1
OG0034705	Biological Process	response to stimulus(GO:0050896)	1
OG0034707	Biological Process	metabolic process(GO:0008152)	1
OG0034707	Biological Process	multi-organism process(GO:0051704)	1
OG0034707	Biological Process	response to stimulus(GO:0050896)	1
OG0034712	Biological Process	biological regulation(GO:0065007)	1
OG0034712	Biological Process	cellular process(GO:0009987)	1
OG0034712	Biological Process	metabolic process(GO:0008152)	1
OG0034722	Biological Process	biological regulation(GO:0065007)	1
OG0034722	Biological Process	cellular process(GO:0009987)	1
OG0034722	Biological Process	developmental process(GO:0032502)	1

OG0034722	Biological Process	metabolic process(GO:0008152)	1
OG0034722	Biological Process	multicellular organismal process(GO:0032501)	1
OG0034722	Biological Process	positive regulation of biological process(GO:0048518)	1
OG0034722	Biological Process	regulation of biological process(GO:0050789)	1
OG0034722	Biological Process	response to stimulus(GO:0050896)	1
OG0034722	Biological Process	signaling(GO:0023052)	1
OG0034729	Biological Process	developmental process(GO:0032502)	1
OG0034729	Biological Process	multicellular organismal process(GO:0032501)	1
OG0034729	Biological Process	reproduction(GO:0000003)	1
OG0034729	Biological Process	reproductive process(GO:0022414)	1
OG0034732	Biological Process	biological regulation(GO:0065007)	1
OG0034732	Biological Process	cellular process(GO:0009987)	1
OG0034732	Biological Process	metabolic process(GO:0008152)	1
OG0034732	Biological Process	regulation of biological process(GO:0050789)	1
OG0034735	Biological Process	biological regulation(GO:0065007)	1
OG0034735	Biological Process	cellular process(GO:0009987)	1
OG0034735	Biological Process	metabolic process(GO:0008152)	1
OG0034735	Biological Process	regulation of biological process(GO:0050789)	1
OG0034735	Biological Process	response to stimulus(GO:0050896)	1
OG0034735	Biological Process	signaling(GO:0023052)	1
OG0034737	Biological Process	cellular process(GO:0009987)	1
OG0034737	Biological Process	localization(GO:0051179)	1
OG0034737	Biological Process	response to stimulus(GO:0050896)	1
OG0034740	Biological Process	cellular component organization or biogenesis(GO:0071840)	1
OG0034740	Biological Process	cellular process(GO:0009987)	1
OG0034740	Biological Process	metabolic process(GO:0008152)	1
OG0034750	Biological Process	biological regulation(GO:0065007)	1
OG0034750	Biological Process	cellular process(GO:0009987)	1
OG0034750	Biological Process	developmental process(GO:0032502)	1
OG0034750	Biological Process	metabolic process(GO:0008152)	1
OG0034750	Biological Process	multicellular organismal process(GO:0032501)	1
OG0034750	Biological Process	regulation of biological process(GO:0050789)	1
OG0034767	Biological Process	biological regulation(GO:0065007)	1
OG0034767	Biological Process	carbon utilization(GO:0015976)	1
OG0034767	Biological Process	cellular process(GO:0009987)	1
OG0034767	Biological Process	metabolic process(GO:0008152)	1
OG0034767	Biological Process	regulation of biological process(GO:0050789)	1
OG0034769	Biological Process	biological regulation(GO:0065007)	1
OG0034769	Biological Process	cellular process(GO:0009987)	1
OG0034769	Biological Process	developmental process(GO:0032502)	1
OG0034769	Biological Process	metabolic process(GO:0008152)	1
OG0034769	Biological Process	multi-organism process(GO:0051704)	1
OG0034769	Biological Process	multicellular organismal process(GO:0032501)	1
OG0034769	Biological Process	regulation of biological process(GO:0050789)	1

OG0034769	Biological Process	reproduction(GO:0000003)	1
OG0034769	Biological Process	reproductive process(GO:0022414)	1
OG0034769	Biological Process	response to stimulus(GO:0050896)	1
OG0034770	Biological Process	cellular process(GO:0009987)	1
OG0034770	Biological Process	localization(GO:0051179)	1
OG0034780	Biological Process	response to stimulus(GO:0050896)	1
OG0034785	Biological Process	cellular process(GO:0009987)	1
OG0034785	Biological Process	metabolic process(GO:0008152)	1
OG0034792	Biological Process	cellular process(GO:0009987)	1
OG0034792	Biological Process	metabolic process(GO:0008152)	1
OG0034805	Biological Process	response to stimulus(GO:0050896)	1
OG0034812	Biological Process	cellular process(GO:0009987)	1
OG0034812	Biological Process	metabolic process(GO:0008152)	1
OG0034816	Biological Process	response to stimulus(GO:0050896)	1
OG0034825	Biological Process	biological regulation(GO:0065007)	1
OG0034825	Biological Process	cellular process(GO:0009987)	1
OG0034825	Biological Process	metabolic process(GO:0008152)	1
OG0034825	Biological Process	regulation of biological process(GO:0050789)	1
OG0034842	Biological Process	biological regulation(GO:0065007)	1
OG0034842	Biological Process	cellular component organization or biogenesis(GO:0071840)	1
OG0034842	Biological Process	cellular process(GO:0009987)	1
OG0034842	Biological Process	metabolic process(GO:0008152)	1
OG0034842	Biological Process	negative regulation of biological process(GO:0048519)	1
OG0034842	Biological Process	regulation of biological process(GO:0050789)	1
OG0034842	Biological Process	response to stimulus(GO:0050896)	1
OG0034852	Biological Process	biological regulation(GO:0065007)	1
OG0034852	Biological Process	cellular process(GO:0009987)	1
OG0034852	Biological Process	developmental process(GO:0032502)	1
OG0034852	Biological Process	growth(GO:0040007)	1
OG0034852	Biological Process	metabolic process(GO:0008152)	1
OG0034852	Biological Process	multicellular organismal process(GO:0032501)	1
OG0034852	Biological Process	regulation of biological process(GO:0050789)	1
OG0034852	Biological Process	response to stimulus(GO:0050896)	1
OG0034852	Biological Process	signaling(GO:0023052)	1
OG0034853	Biological Process	cellular component organization or biogenesis(GO:0071840)	1
OG0034853	Biological Process	cellular process(GO:0009987)	1
OG0034853	Biological Process	developmental process(GO:0032502)	1
OG0034853	Biological Process	multicellular organismal process(GO:0032501)	1
OG0034854	Biological Process	cellular component organization or biogenesis(GO:0071840)	1
OG0034854	Biological Process	cellular process(GO:0009987)	1
OG0034854	Biological Process	developmental process(GO:0032502)	1
OG0034854	Biological Process	localization(GO:0051179)	1
OG0034854	Biological Process	multicellular organismal process(GO:0032501)	1
OG0034856	Biological Process	cellular process(GO:0009987)	1
OG0034856	Biological Process	metabolic process(GO:0008152)	1

OG0034888	Biological Process	cellular process(GO:0009987)	1
OG0034888	Biological Process	developmental process(GO:0032502)	1
OG0034888	Biological Process	growth(GO:0040007)	1
OG0034888	Biological Process	metabolic process(GO:0008152)	1
OG0034888	Biological Process	multicellular organismal process(GO:0032501)	1
OG0034909	Biological Process	metabolic process(GO:0008152)	1
OG0034918	Biological Process	biological regulation(GO:0065007)	1
OG0034918	Biological Process	cellular component organization or biogenesis(GO:0071840)	1
OG0034918	Biological Process	cellular process(GO:0009987)	1
OG0034918	Biological Process	immune system process(GO:0002376)	1
OG0034918	Biological Process	localization(GO:0051179)	1
OG0034918	Biological Process	regulation of biological process(GO:0050789)	1
OG0034918	Biological Process	response to stimulus(GO:0050896)	1
OG0034926	Biological Process	cellular process(GO:0009987)	1
OG0034926	Biological Process	metabolic process(GO:0008152)	1
OG0034934	Biological Process	cellular component organization or biogenesis(GO:0071840)	1
OG0034934	Biological Process	cellular process(GO:0009987)	1
OG0034934	Biological Process	developmental process(GO:0032502)	1
OG0034934	Biological Process	localization(GO:0051179)	1
OG0034934	Biological Process	multi-organism process(GO:0051704)	1
OG0034934	Biological Process	multicellular organismal process(GO:0032501)	1
OG0034934	Biological Process	response to stimulus(GO:0050896)	1
OG0034935	Biological Process	biological regulation(GO:0065007)	1
OG0034935	Biological Process	cellular process(GO:0009987)	1
OG0034935	Biological Process	metabolic process(GO:0008152)	1
OG0034935	Biological Process	regulation of biological process(GO:0050789)	1
OG0034969	Biological Process	cellular process(GO:0009987)	1
OG0034969	Biological Process	metabolic process(GO:0008152)	1
OG0034970	Biological Process	cellular process(GO:0009987)	1
OG0034970	Biological Process	localization(GO:0051179)	1
OG0034970	Biological Process	metabolic process(GO:0008152)	1
OG0034979	Biological Process	response to stimulus(GO:0050896)	1
OG0034980	Biological Process	biological regulation(GO:0065007)	1
OG0034980	Biological Process	cellular process(GO:0009987)	1
OG0034980	Biological Process	metabolic process(GO:0008152)	1
OG0034980	Biological Process	regulation of biological process(GO:0050789)	1
OG0034980	Biological Process	response to stimulus(GO:0050896)	1
OG0034981	Biological Process	biological regulation(GO:0065007)	1
OG0034981	Biological Process	cellular process(GO:0009987)	1
OG0034981	Biological Process	metabolic process(GO:0008152)	1
OG0034981	Biological Process	regulation of biological process(GO:0050789)	1
OG0034981	Biological Process	response to stimulus(GO:0050896)	1
OG0034982	Biological Process	cellular component organization or biogenesis(GO:0071840)	1
OG0034982	Biological Process	cellular process(GO:0009987)	1
OG0034989	Biological Process	developmental process(GO:0032502)	1

OG0034989	Biological Process	metabolic process(GO:0008152)	1
OG0034989	Biological Process	multicellular organismal process(GO:0032501)	1
OG0034998	Biological Process	cellular process(GO:0009987)	1
OG0034998	Biological Process	metabolic process(GO:0008152)	1
OG0034998	Biological Process	response to stimulus(GO:0050896)	1
OG0035020	Biological Process	biological regulation(GO:0065007)	1
OG0035020	Biological Process	cellular process(GO:0009987)	1
OG0035020	Biological Process	metabolic process(GO:0008152)	1
OG0035020	Biological Process	regulation of biological process(GO:0050789)	1
OG0035020	Biological Process	response to stimulus(GO:0050896)	1
OG0035020	Biological Process	rhythmic process(GO:0048511)	1
OG0035022	Biological Process	metabolic process(GO:0008152)	1
OG0035022	Biological Process	response to stimulus(GO:0050896)	1
OG0035025	Biological Process	cellular process(GO:0009987)	1
OG0035025	Biological Process	metabolic process(GO:0008152)	1
OG0035025	Biological Process	response to stimulus(GO:0050896)	1
OG0035028	Biological Process	cellular component organization or biogenesis(GO:0071840)	1
OG0035028	Biological Process	cellular process(GO:0009987)	1
OG0035028	Biological Process	developmental process(GO:0032502)	1
OG0035028	Biological Process	growth(GO:0040007)	1
OG0035028	Biological Process	localization(GO:0051179)	1
OG0035028	Biological Process	metabolic process(GO:0008152)	1
OG0035028	Biological Process	multicellular organismal process(GO:0032501)	1
OG0035031	Biological Process	biological regulation(GO:0065007)	1
OG0035031	Biological Process	cellular process(GO:0009987)	1
OG0035031	Biological Process	developmental process(GO:0032502)	1
OG0035031	Biological Process	metabolic process(GO:0008152)	1
OG0035031	Biological Process	multi-organism process(GO:0051704)	1
OG0035031	Biological Process	multicellular organismal process(GO:0032501)	1
OG0035031	Biological Process	regulation of biological process(GO:0050789)	1
OG0035031	Biological Process	reproduction(GO:0000003)	1
OG0035031	Biological Process	reproductive process(GO:0022414)	1
OG0035031	Biological Process	response to stimulus(GO:0050896)	1
OG0035033	Biological Process	response to stimulus(GO:0050896)	1
OG0035037	Biological Process	cellular process(GO:0009987)	1
OG0035037	Biological Process	developmental process(GO:0032502)	1
OG0035037	Biological Process	multicellular organismal process(GO:0032501)	1
OG0035039	Biological Process	cellular process(GO:0009987)	1
OG0035039	Biological Process	developmental process(GO:0032502)	1
OG0035039	Biological Process	multicellular organismal process(GO:0032501)	1
OG0035047	Biological Process	biological regulation(GO:0065007)	1
OG0035047	Biological Process	cellular process(GO:0009987)	1
OG0035047	Biological Process	developmental process(GO:0032502)	1
OG0035047	Biological Process	growth(GO:0040007)	1
OG0035047	Biological Process	localization(GO:0051179)	1

OG0035047	Biological Process	metabolic process(GO:0008152)	1
OG0035047	Biological Process	multicellular organismal process(GO:0032501)	1
OG0035047	Biological Process	positive regulation of biological process(GO:0048518)	1
OG0035047	Biological Process	regulation of biological process(GO:0050789)	1
OG0035047	Biological Process	response to stimulus(GO:0050896)	1
OG0035047	Biological Process	signaling(GO:0023052)	1
OG0035063	Biological Process	cellular component organization or biogenesis(GO:0071840)	1
OG0035063	Biological Process	cellular process(GO:0009987)	1
OG0035063	Biological Process	developmental process(GO:0032502)	1
OG0035063	Biological Process	growth(GO:0040007)	1
OG0035063	Biological Process	metabolic process(GO:0008152)	1
OG0035063	Biological Process	multicellular organismal process(GO:0032501)	1
OG0035077	Biological Process	biological regulation(GO:0065007)	1
OG0035077	Biological Process	cellular process(GO:0009987)	1
OG0035077	Biological Process	metabolic process(GO:0008152)	1
OG0035077	Biological Process	regulation of biological process(GO:0050789)	1
OG0035079	Biological Process	cellular component organization or biogenesis(GO:0071840)	1
OG0035079	Biological Process	cellular process(GO:0009987)	1
OG0035079	Biological Process	growth(GO:0040007)	1
OG0035079	Biological Process	metabolic process(GO:0008152)	1
OG0035083	Biological Process	cellular process(GO:0009987)	1
OG0035083	Biological Process	metabolic process(GO:0008152)	1
OG0035091	Biological Process	response to stimulus(GO:0050896)	1
OG0035094	Biological Process	cellular process(GO:0009987)	1
OG0035094	Biological Process	localization(GO:0051179)	1
OG0035094	Biological Process	response to stimulus(GO:0050896)	1
OG0035095	Biological Process	biological regulation(GO:0065007)	1
OG0035095	Biological Process	cellular component organization or biogenesis(GO:0071840)	1
OG0035095	Biological Process	cellular process(GO:0009987)	1
OG0035095	Biological Process	developmental process(GO:0032502)	1
OG0035095	Biological Process	metabolic process(GO:0008152)	1
OG0035095	Biological Process	multicellular organismal process(GO:0032501)	1
OG0035095	Biological Process	regulation of biological process(GO:0050789)	1
OG0035099	Biological Process	metabolic process(GO:0008152)	1
OG0035106	Biological Process	biological regulation(GO:0065007)	1
OG0035106	Biological Process	cellular process(GO:0009987)	1
OG0035106	Biological Process	developmental process(GO:0032502)	1
OG0035106	Biological Process	multicellular organismal process(GO:0032501)	1
OG0035106	Biological Process	negative regulation of biological process(GO:0048519)	1
OG0035106	Biological Process	regulation of biological process(GO:0050789)	1
OG0035163	Biological Process	response to stimulus(GO:0050896)	1
OG0035165	Biological Process	biological regulation(GO:0065007)	1
OG0035165	Biological Process	cellular process(GO:0009987)	1
OG0035165	Biological Process	localization(GO:0051179)	1

OG0035165	Biological Process	metabolic process(GO:0008152)	1
OG0035165	Biological Process	multi-organism process(GO:0051704)	1
OG0035165	Biological Process	negative regulation of biological process(GO:0048519)	1
OG0035165	Biological Process	regulation of biological process(GO:0050789)	1
OG0035165	Biological Process	response to stimulus(GO:0050896)	1
OG0035168	Biological Process	cellular process(GO:0009987)	1
OG0035168	Biological Process	growth(GO:0040007)	1
OG0035168	Biological Process	localization(GO:0051179)	1
OG0035168	Biological Process	metabolic process(GO:0008152)	1
OG0035175	Biological Process	biological regulation(GO:0065007)	1
OG0035175	Biological Process	cellular process(GO:0009987)	1
OG0035175	Biological Process	developmental process(GO:0032502)	1
OG0035175	Biological Process	metabolic process(GO:0008152)	1
OG0035175	Biological Process	multicellular organismal process(GO:0032501)	1
OG0035175	Biological Process	negative regulation of biological process(GO:0048519)	1
OG0035175	Biological Process	regulation of biological process(GO:0050789)	1
OG0035175	Biological Process	reproduction(GO:0000003)	1
OG0035175	Biological Process	reproductive process(GO:0022414)	1
OG0035175	Biological Process	response to stimulus(GO:0050896)	1
OG0035176	Biological Process	cellular process(GO:0009987)	1
OG0035176	Biological Process	developmental process(GO:0032502)	1
OG0035176	Biological Process	multicellular organismal process(GO:0032501)	1
OG0035176	Biological Process	reproduction(GO:0000003)	1
OG0035176	Biological Process	reproductive process(GO:0022414)	1
OG0035177	Biological Process	biological regulation(GO:0065007)	1
OG0035177	Biological Process	cellular process(GO:0009987)	1
OG0035177	Biological Process	developmental process(GO:0032502)	1
OG0035177	Biological Process	metabolic process(GO:0008152)	1
OG0035177	Biological Process	multicellular organismal process(GO:0032501)	1
OG0035177	Biological Process	negative regulation of biological process(GO:0048519)	1
OG0035177	Biological Process	regulation of biological process(GO:0050789)	1
OG0035177	Biological Process	reproduction(GO:0000003)	1
OG0035177	Biological Process	reproductive process(GO:0022414)	1
OG0035177	Biological Process	response to stimulus(GO:0050896)	1
OG0035181	Biological Process	biological regulation(GO:0065007)	1
OG0035181	Biological Process	cellular process(GO:0009987)	1
OG0035181	Biological Process	developmental process(GO:0032502)	1
OG0035181	Biological Process	metabolic process(GO:0008152)	1
OG0035181	Biological Process	multicellular organismal process(GO:0032501)	1
OG0035181	Biological Process	negative regulation of biological process(GO:0048519)	1
OG0035181	Biological Process	regulation of biological process(GO:0050789)	1
OG0035181	Biological Process	reproduction(GO:0000003)	1
OG0035181	Biological Process	reproductive process(GO:0022414)	1
OG0035181	Biological Process	response to stimulus(GO:0050896)	1

OG0035194	Biological Process	cellular component organization or biogenesis(GO:0071840)	1
OG0035194	Biological Process	cellular process(GO:0009987)	1
OG0035194	Biological Process	metabolic process(GO:0008152)	1
OG0035200	Biological Process	cellular process(GO:0009987)	1
OG0035200	Biological Process	metabolic process(GO:0008152)	1
OG0035200	Biological Process	response to stimulus(GO:0050896)	1
OG0035205	Biological Process	cellular component organization or biogenesis(GO:0071840)	1
OG0035205	Biological Process	cellular process(GO:0009987)	1
OG0035205	Biological Process	growth(GO:0040007)	1
OG0035205	Biological Process	metabolic process(GO:0008152)	1
OG0035214	Biological Process	response to stimulus(GO:0050896)	1
OG0035232	Biological Process	cellular process(GO:0009987)	1
OG0035232	Biological Process	metabolic process(GO:0008152)	1
OG0000213	Cellular Component	cell part(GO:0044464)	1
OG0000213	Cellular Component	cell(GO:0005623)	1
OG0000213	Cellular Component	organelle part(GO:0044422)	1
OG0000213	Cellular Component	organelle(GO:0043226)	1
OG0005149	Cellular Component	cell part(GO:0044464)	1
OG0005149	Cellular Component	cell(GO:0005623)	1
OG0005149	Cellular Component	protein-containing complex(GO:0032991)	1
OG0009289	Cellular Component	cell part(GO:0044464)	5
OG0009289	Cellular Component	cell(GO:0005623)	5
OG0009289	Cellular Component	membrane(GO:0016020)	1
OG0009289	Cellular Component	organelle part(GO:0044422)	1
OG0009289	Cellular Component	organelle(GO:0043226)	5
OG0010766	Cellular Component	cell part(GO:0044464)	6
OG0010766	Cellular Component	cell(GO:0005623)	6
OG0010766	Cellular Component	organelle(GO:0043226)	6
OG0010771	Cellular Component	cell part(GO:0044464)	4
OG0010771	Cellular Component	cell(GO:0005623)	4
OG0010771	Cellular Component	membrane(GO:0016020)	4
OG0010771	Cellular Component	organelle part(GO:0044422)	4
OG0010771	Cellular Component	organelle(GO:0043226)	4
OG0010776	Cellular Component	cell part(GO:0044464)	2
OG0010776	Cellular Component	cell(GO:0005623)	2
OG0010776	Cellular Component	membrane(GO:0016020)	1
OG0010776	Cellular Component	organelle(GO:0043226)	1
OG0011829	Cellular Component	cell part(GO:0044464)	1
OG0011829	Cellular Component	cell(GO:0005623)	1
OG0011829	Cellular Component	extracellular region(GO:0005576)	1
OG0011829	Cellular Component	membrane(GO:0016020)	1
OG0011829	Cellular Component	organelle part(GO:0044422)	1
OG0011829	Cellular Component	organelle(GO:0043226)	1
OG0011838	Cellular Component	cell part(GO:0044464)	3
OG0011838	Cellular Component	cell(GO:0005623)	3
OG0011838	Cellular Component	organelle(GO:0043226)	3

OG0011855	Cellular Component	cell part(GO:0044464)	1
OG0011855	Cellular Component	cell(GO:0005623)	1
OG0012548	Cellular Component	cell part(GO:0044464)	1
OG0012548	Cellular Component	cell(GO:0005623)	1
OG0012548	Cellular Component	organelle(GO:0043226)	1
OG0012570	Cellular Component	cell part(GO:0044464)	1
OG0012570	Cellular Component	cell(GO:0005623)	1
OG0012570	Cellular Component	organelle(GO:0043226)	1
OG0012574	Cellular Component	cell part(GO:0044464)	4
OG0012574	Cellular Component	cell(GO:0005623)	4
OG0012574	Cellular Component	membrane(GO:0016020)	4
OG0013086	Cellular Component	cell part(GO:0044464)	3
OG0013086	Cellular Component	cell(GO:0005623)	3
OG0013086	Cellular Component	organelle(GO:0043226)	3
OG0013092	Cellular Component	cell part(GO:0044464)	1
OG0013092	Cellular Component	cell(GO:0005623)	1
OG0013092	Cellular Component	organelle(GO:0043226)	1
OG0013101	Cellular Component	cell part(GO:0044464)	2
OG0013101	Cellular Component	cell(GO:0005623)	2
OG0013101	Cellular Component	membrane(GO:0016020)	2
OG0013101	Cellular Component	membrane-enclosed lumen(GO:0031974)	2
OG0013101	Cellular Component	organelle part(GO:0044422)	2
OG0013101	Cellular Component	organelle(GO:0043226)	2
OG0013101	Cellular Component	protein-containing complex(GO:0032991)	2
OG0013118	Cellular Component	cell part(GO:0044464)	2
OG0013118	Cellular Component	cell(GO:0005623)	2
OG0013118	Cellular Component	organelle(GO:0043226)	2
OG0013555	Cellular Component	cell part(GO:0044464)	1
OG0013555	Cellular Component	cell(GO:0005623)	1
OG0013555	Cellular Component	membrane-enclosed lumen(GO:0031974)	1
OG0013555	Cellular Component	organelle part(GO:0044422)	1
OG0013555	Cellular Component	organelle(GO:0043226)	1
OG0013555	Cellular Component	protein-containing complex(GO:0032991)	1
OG0013556	Cellular Component	cell part(GO:0044464)	1
OG0013556	Cellular Component	cell(GO:0005623)	1
OG0013556	Cellular Component	membrane(GO:0016020)	1
OG0013559	Cellular Component	membrane(GO:0016020)	2
OG0013561	Cellular Component	membrane(GO:0016020)	1
OG0013563	Cellular Component	cell part(GO:0044464)	2
OG0013563	Cellular Component	cell(GO:0005623)	2
OG0013563	Cellular Component	organelle(GO:0043226)	2
OG0013564	Cellular Component	cell part(GO:0044464)	2
OG0013564	Cellular Component	cell(GO:0005623)	2
OG0013564	Cellular Component	membrane(GO:0016020)	2
OG0013567	Cellular Component	cell part(GO:0044464)	2
OG0013567	Cellular Component	cell(GO:0005623)	2
OG0013567	Cellular Component	organelle(GO:0043226)	2

OG0013568	Cellular Component	cell part(GO:0044464)	2
OG0013568	Cellular Component	cell(GO:0005623)	2
OG0013568	Cellular Component	membrane(GO:0016020)	2
OG0013577	Cellular Component	cell part(GO:0044464)	1
OG0013577	Cellular Component	cell(GO:0005623)	1
OG0013577	Cellular Component	membrane(GO:0016020)	1
OG0013577	Cellular Component	organelle part(GO:0044422)	1
OG0013577	Cellular Component	organelle(GO:0043226)	1
OG0013579	Cellular Component	cell part(GO:0044464)	2
OG0013579	Cellular Component	cell(GO:0005623)	2
OG0013579	Cellular Component	organelle(GO:0043226)	2
OG0013582	Cellular Component	cell part(GO:0044464)	2
OG0013582	Cellular Component	cell(GO:0005623)	2
OG0013582	Cellular Component	membrane(GO:0016020)	2
OG0013594	Cellular Component	cell part(GO:0044464)	2
OG0013594	Cellular Component	cell(GO:0005623)	2
OG0013594	Cellular Component	organelle(GO:0043226)	2
OG0013595	Cellular Component	cell junction(GO:0030054)	2
OG0013595	Cellular Component	symplast(GO:0055044)	2
OG0013596	Cellular Component	cell part(GO:0044464)	2
OG0013596	Cellular Component	cell(GO:0005623)	2
OG0013596	Cellular Component	membrane part(GO:0044425)	2
OG0013596	Cellular Component	membrane(GO:0016020)	2
OG0013604	Cellular Component	cell part(GO:0044464)	2
OG0013604	Cellular Component	cell(GO:0005623)	2
OG0013604	Cellular Component	organelle(GO:0043226)	2
OG0013612	Cellular Component	cell part(GO:0044464)	2
OG0013612	Cellular Component	cell(GO:0005623)	2
OG0013612	Cellular Component	organelle(GO:0043226)	2
OG0013622	Cellular Component	cell part(GO:0044464)	2
OG0013622	Cellular Component	cell(GO:0005623)	2
OG0013622	Cellular Component	organelle(GO:0043226)	2
OG0013627	Cellular Component	cell part(GO:0044464)	2
OG0013627	Cellular Component	cell(GO:0005623)	2
OG0013627	Cellular Component	membrane(GO:0016020)	2
OG0013632	Cellular Component	cell part(GO:0044464)	2
OG0013632	Cellular Component	cell(GO:0005623)	2
OG0013632	Cellular Component	organelle(GO:0043226)	2
OG0013633	Cellular Component	cell part(GO:0044464)	2
OG0013633	Cellular Component	cell(GO:0005623)	2
OG0013633	Cellular Component	organelle(GO:0043226)	2
OG0013636	Cellular Component	cell part(GO:0044464)	1
OG0013636	Cellular Component	cell(GO:0005623)	1
OG0013636	Cellular Component	membrane part(GO:0044425)	1
OG0013636	Cellular Component	membrane(GO:0016020)	1
OG0013636	Cellular Component	organelle(GO:0043226)	1
OG0013637	Cellular Component	cell part(GO:0044464)	1

OG0013637	Cellular Component	cell(GO:0005623)	1
OG0013637	Cellular Component	membrane(GO:0016020)	1
OG0013638	Cellular Component	cell part(GO:0044464)	1
OG0013638	Cellular Component	cell(GO:0005623)	1
OG0013638	Cellular Component	membrane part(GO:0044425)	1
OG0013638	Cellular Component	membrane(GO:0016020)	1
OG0013638	Cellular Component	organelle part(GO:0044422)	1
OG0013638	Cellular Component	organelle(GO:0043226)	1
OG0013638	Cellular Component	protein-containing complex(GO:0032991)	1
OG0013638	Cellular Component	synapse part(GO:0044456)	1
OG0013638	Cellular Component	synapse(GO:0045202)	1
OG0013652	Cellular Component	cell part(GO:0044464)	1
OG0013652	Cellular Component	cell(GO:0005623)	1
OG0013652	Cellular Component	organelle(GO:0043226)	1
OG0013655	Cellular Component	cell part(GO:0044464)	2
OG0013655	Cellular Component	cell(GO:0005623)	2
OG0013655	Cellular Component	organelle(GO:0043226)	2
OG0013656	Cellular Component	cell part(GO:0044464)	2
OG0013656	Cellular Component	cell(GO:0005623)	2
OG0013656	Cellular Component	organelle(GO:0043226)	2
OG0027162	Cellular Component	cell part(GO:0044464)	1
OG0027162	Cellular Component	cell(GO:0005623)	1
OG0027162	Cellular Component	extracellular region(GO:0005576)	1
OG0027162	Cellular Component	membrane(GO:0016020)	1
OG0027163	Cellular Component	cell part(GO:0044464)	1
OG0027163	Cellular Component	cell(GO:0005623)	1
OG0027167	Cellular Component	cell part(GO:0044464)	1
OG0027167	Cellular Component	cell(GO:0005623)	1
OG0027167	Cellular Component	membrane(GO:0016020)	1
OG0027171	Cellular Component	cell part(GO:0044464)	1
OG0027171	Cellular Component	cell(GO:0005623)	1
OG0027171	Cellular Component	organelle(GO:0043226)	1
OG0027176	Cellular Component	cell part(GO:0044464)	1
OG0027176	Cellular Component	cell(GO:0005623)	1
OG0027176	Cellular Component	extracellular region(GO:0005576)	1
OG0027179	Cellular Component	cell part(GO:0044464)	1
OG0027179	Cellular Component	cell(GO:0005623)	1
OG0027179	Cellular Component	membrane(GO:0016020)	1
OG0027180	Cellular Component	cell part(GO:0044464)	1
OG0027180	Cellular Component	cell(GO:0005623)	1
OG0027180	Cellular Component	membrane(GO:0016020)	1
OG0027181	Cellular Component	membrane(GO:0016020)	1
OG0027184	Cellular Component	cell part(GO:0044464)	1
OG0027184	Cellular Component	cell(GO:0005623)	1
OG0027184	Cellular Component	organelle(GO:0043226)	1
OG0027185	Cellular Component	cell part(GO:0044464)	1
OG0027185	Cellular Component	cell(GO:0005623)	1

OG0027185	Cellular Component	organelle(GO:0043226)	1
OG0027199	Cellular Component	cell junction(GO:0030054)	1
OG0027199	Cellular Component	cell part(GO:0044464)	1
OG0027199	Cellular Component	cell(GO:0005623)	1
OG0027199	Cellular Component	membrane(GO:0016020)	1
OG0027199	Cellular Component	organelle(GO:0043226)	1
OG0027199	Cellular Component	symplast(GO:0055044)	1
OG0027200	Cellular Component	cell junction(GO:0030054)	1
OG0027200	Cellular Component	cell part(GO:0044464)	1
OG0027200	Cellular Component	cell(GO:0005623)	1
OG0027200	Cellular Component	membrane(GO:0016020)	1
OG0027200	Cellular Component	organelle(GO:0043226)	1
OG0027200	Cellular Component	symplast(GO:0055044)	1
OG0027201	Cellular Component	cell part(GO:0044464)	1
OG0027201	Cellular Component	cell(GO:0005623)	1
OG0027201	Cellular Component	membrane(GO:0016020)	1
OG0027201	Cellular Component	organelle(GO:0043226)	1
OG0027205	Cellular Component	cell part(GO:0044464)	1
OG0027205	Cellular Component	cell(GO:0005623)	1
OG0027205	Cellular Component	membrane(GO:0016020)	1
OG0027205	Cellular Component	organelle part(GO:0044422)	1
OG0027205	Cellular Component	organelle(GO:0043226)	1
OG0027206	Cellular Component	cell part(GO:0044464)	1
OG0027206	Cellular Component	cell(GO:0005623)	1
OG0027206	Cellular Component	organelle(GO:0043226)	1
OG0027208	Cellular Component	cell part(GO:0044464)	1
OG0027208	Cellular Component	cell(GO:0005623)	1
OG0027208	Cellular Component	organelle(GO:0043226)	1
OG0027210	Cellular Component	cell part(GO:0044464)	1
OG0027210	Cellular Component	cell(GO:0005623)	1
OG0027210	Cellular Component	organelle part(GO:0044422)	1
OG0027210	Cellular Component	organelle(GO:0043226)	1
OG0027213	Cellular Component	cell part(GO:0044464)	1
OG0027213	Cellular Component	cell(GO:0005623)	1
OG0027213	Cellular Component	membrane-enclosed lumen(GO:0031974)	1
OG0027213	Cellular Component	organelle part(GO:0044422)	1
OG0027213	Cellular Component	organelle(GO:0043226)	1
OG0027213	Cellular Component	protein-containing complex(GO:0032991)	1
OG0027214	Cellular Component	cell part(GO:0044464)	1
OG0027214	Cellular Component	cell(GO:0005623)	1
OG0027214	Cellular Component	membrane(GO:0016020)	1
OG0027218	Cellular Component	cell part(GO:0044464)	1
OG0027218	Cellular Component	cell(GO:0005623)	1
OG0027218	Cellular Component	extracellular region part(GO:0044421)	1
OG0027218	Cellular Component	extracellular region(GO:0005576)	1
OG0027218	Cellular Component	organelle part(GO:0044422)	1
OG0027218	Cellular Component	organelle(GO:0043226)	1

OG0027218	Cellular Component	protein-containing complex(GO:0032991)	1
OG0027219	Cellular Component	cell part(GO:0044464)	1
OG0027219	Cellular Component	cell(GO:0005623)	1
OG0027219	Cellular Component	membrane-enclosed lumen(GO:0031974)	1
OG0027219	Cellular Component	organelle part(GO:0044422)	1
OG0027219	Cellular Component	organelle(GO:0043226)	1
OG0027226	Cellular Component	cell part(GO:0044464)	1
OG0027226	Cellular Component	cell(GO:0005623)	1
OG0027226	Cellular Component	protein-containing complex(GO:0032991)	1
OG0027227	Cellular Component	cell part(GO:0044464)	1
OG0027227	Cellular Component	cell(GO:0005623)	1
OG0027227	Cellular Component	membrane(GO:0016020)	1
OG0027230	Cellular Component	cell part(GO:0044464)	1
OG0027230	Cellular Component	cell(GO:0005623)	1
OG0027230	Cellular Component	membrane(GO:0016020)	1
OG0027231	Cellular Component	cell part(GO:0044464)	1
OG0027231	Cellular Component	cell(GO:0005623)	1
OG0027234	Cellular Component	cell part(GO:0044464)	1
OG0027234	Cellular Component	cell(GO:0005623)	1
OG0027235	Cellular Component	membrane(GO:0016020)	1
OG0027237	Cellular Component	cell part(GO:0044464)	1
OG0027237	Cellular Component	cell(GO:0005623)	1
OG0027237	Cellular Component	extracellular region(GO:0005576)	1
OG0027238	Cellular Component	cell part(GO:0044464)	1
OG0027238	Cellular Component	cell(GO:0005623)	1
OG0027240	Cellular Component	cell part(GO:0044464)	1
OG0027240	Cellular Component	cell(GO:0005623)	1
OG0027240	Cellular Component	extracellular region(GO:0005576)	1
OG0027243	Cellular Component	cell part(GO:0044464)	1
OG0027243	Cellular Component	cell(GO:0005623)	1
OG0027243	Cellular Component	membrane(GO:0016020)	1
OG0027244	Cellular Component	cell part(GO:0044464)	1
OG0027244	Cellular Component	cell(GO:0005623)	1
OG0027244	Cellular Component	membrane(GO:0016020)	1
OG0027245	Cellular Component	cell part(GO:0044464)	1
OG0027245	Cellular Component	cell(GO:0005623)	1
OG0027245	Cellular Component	membrane part(GO:0044425)	1
OG0027245	Cellular Component	membrane(GO:0016020)	1
OG0027245	Cellular Component	organelle part(GO:0044422)	1
OG0027245	Cellular Component	organelle(GO:0043226)	1
OG0027245	Cellular Component	protein-containing complex(GO:0032991)	1
OG0027246	Cellular Component	cell part(GO:0044464)	1
OG0027246	Cellular Component	cell(GO:0005623)	1
OG0027249	Cellular Component	cell part(GO:0044464)	1
OG0027249	Cellular Component	cell(GO:0005623)	1
OG0027249	Cellular Component	membrane(GO:0016020)	1

OG0027250	Cellular Component	cell part(GO:0044464)	1
OG0027250	Cellular Component	cell(GO:0005623)	1
OG0027250	Cellular Component	membrane(GO:0016020)	1
OG0027252	Cellular Component	cell part(GO:0044464)	1
OG0027252	Cellular Component	cell(GO:0005623)	1
OG0027252	Cellular Component	membrane(GO:0016020)	1
OG0027254	Cellular Component	cell part(GO:0044464)	1
OG0027254	Cellular Component	cell(GO:0005623)	1
OG0027254	Cellular Component	membrane(GO:0016020)	1
OG0027258	Cellular Component	cell part(GO:0044464)	1
OG0027258	Cellular Component	cell(GO:0005623)	1
OG0027258	Cellular Component	membrane(GO:0016020)	1
OG0027260	Cellular Component	cell part(GO:0044464)	1
OG0027260	Cellular Component	cell(GO:0005623)	1
OG0027260	Cellular Component	membrane(GO:0016020)	1
OG0027262	Cellular Component	cell part(GO:0044464)	1
OG0027262	Cellular Component	cell(GO:0005623)	1
OG0027262	Cellular Component	protein-containing complex(GO:0032991)	1
OG0027265	Cellular Component	cell part(GO:0044464)	1
OG0027265	Cellular Component	cell(GO:0005623)	1
OG0027265	Cellular Component	organelle part(GO:0044422)	1
OG0027265	Cellular Component	organelle(GO:0043226)	1
OG0027268	Cellular Component	cell part(GO:0044464)	1
OG0027268	Cellular Component	cell(GO:0005623)	1
OG0027268	Cellular Component	membrane(GO:0016020)	1
OG0027268	Cellular Component	organelle part(GO:0044422)	1
OG0027268	Cellular Component	organelle(GO:0043226)	1
OG0027272	Cellular Component	cell part(GO:0044464)	1
OG0027272	Cellular Component	cell(GO:0005623)	1
OG0027281	Cellular Component	cell part(GO:0044464)	1
OG0027281	Cellular Component	cell(GO:0005623)	1
OG0027281	Cellular Component	organelle(GO:0043226)	1
OG0027292	Cellular Component	cell part(GO:0044464)	1
OG0027292	Cellular Component	cell(GO:0005623)	1
OG0027292	Cellular Component	organelle(GO:0043226)	1
OG0027303	Cellular Component	cell part(GO:0044464)	1
OG0027303	Cellular Component	cell(GO:0005623)	1
OG0027303	Cellular Component	organelle(GO:0043226)	1
OG0027304	Cellular Component	cell part(GO:0044464)	1
OG0027304	Cellular Component	cell(GO:0005623)	1
OG0027304	Cellular Component	organelle(GO:0043226)	1
OG0027309	Cellular Component	cell junction(GO:0030054)	1
OG0027309	Cellular Component	cell part(GO:0044464)	1
OG0027309	Cellular Component	cell(GO:0005623)	1
OG0027309	Cellular Component	membrane(GO:0016020)	1
OG0027309	Cellular Component	organelle part(GO:0044422)	1
OG0027309	Cellular Component	organelle(GO:0043226)	1

OG0027309	Cellular Component	symplast(GO:0055044)	1
OG0027311	Cellular Component	cell part(GO:0044464)	1
OG0027311	Cellular Component	cell(GO:0005623)	1
OG0027311	Cellular Component	organelle(GO:0043226)	1
OG0027312	Cellular Component	cell part(GO:0044464)	1
OG0027312	Cellular Component	cell(GO:0005623)	1
OG0027312	Cellular Component	organelle(GO:0043226)	1
OG0027316	Cellular Component	cell part(GO:0044464)	1
OG0027316	Cellular Component	cell(GO:0005623)	1
OG0027316	Cellular Component	membrane part(GO:0044425)	1
OG0027316	Cellular Component	membrane(GO:0016020)	1
OG0027316	Cellular Component	protein-containing complex(GO:0032991)	1
OG0027317	Cellular Component	cell part(GO:0044464)	1
OG0027317	Cellular Component	cell(GO:0005623)	1
OG0027317	Cellular Component	membrane(GO:0016020)	1
OG0027317	Cellular Component	protein-containing complex(GO:0032991)	1
OG0027318	Cellular Component	cell part(GO:0044464)	1
OG0027318	Cellular Component	cell(GO:0005623)	1
OG0027320	Cellular Component	cell part(GO:0044464)	1
OG0027320	Cellular Component	cell(GO:0005623)	1
OG0027320	Cellular Component	membrane(GO:0016020)	1
OG0027321	Cellular Component	cell part(GO:0044464)	1
OG0027321	Cellular Component	cell(GO:0005623)	1
OG0027321	Cellular Component	membrane(GO:0016020)	1
OG0027322	Cellular Component	cell part(GO:0044464)	1
OG0027322	Cellular Component	cell(GO:0005623)	1
OG0027322	Cellular Component	membrane(GO:0016020)	1
OG0027323	Cellular Component	cell part(GO:0044464)	1
OG0027323	Cellular Component	cell(GO:0005623)	1
OG0027323	Cellular Component	extracellular region(GO:0005576)	1
OG0027323	Cellular Component	membrane(GO:0016020)	1
OG0027326	Cellular Component	cell part(GO:0044464)	1
OG0027326	Cellular Component	cell(GO:0005623)	1
OG0027326	Cellular Component	membrane(GO:0016020)	1
OG0027328	Cellular Component	cell part(GO:0044464)	1
OG0027328	Cellular Component	cell(GO:0005623)	1
OG0027328	Cellular Component	membrane(GO:0016020)	1
OG0027328	Cellular Component	organelle part(GO:0044422)	1
OG0027328	Cellular Component	organelle(GO:0043226)	1
OG0027328	Cellular Component	protein-containing complex(GO:0032991)	1
OG0027330	Cellular Component	cell part(GO:0044464)	1
OG0027330	Cellular Component	cell(GO:0005623)	1
OG0027330	Cellular Component	membrane(GO:0016020)	1
OG0027330	Cellular Component	organelle part(GO:0044422)	1
OG0027330	Cellular Component	organelle(GO:0043226)	1
OG0027330	Cellular Component	protein-containing complex(GO:0032991)	1

OG0027331	Cellular Component	cell part(GO:0044464)	1
OG0027331	Cellular Component	cell(GO:0005623)	1
OG0027333	Cellular Component	cell part(GO:0044464)	1
OG0027333	Cellular Component	cell(GO:0005623)	1
OG0027333	Cellular Component	organelle(GO:0043226)	1
OG0027336	Cellular Component	cell part(GO:0044464)	1
OG0027336	Cellular Component	cell(GO:0005623)	1
OG0027336	Cellular Component	organelle(GO:0043226)	1
OG0027337	Cellular Component	cell part(GO:0044464)	1
OG0027337	Cellular Component	cell(GO:0005623)	1
OG0027337	Cellular Component	organelle(GO:0043226)	1
OG0027339	Cellular Component	cell part(GO:0044464)	1
OG0027339	Cellular Component	cell(GO:0005623)	1
OG0027339	Cellular Component	membrane part(GO:0044425)	1
OG0027339	Cellular Component	membrane(GO:0016020)	1
OG0027339	Cellular Component	organelle part(GO:0044422)	1
OG0027339	Cellular Component	organelle(GO:0043226)	1
OG0027341	Cellular Component	cell part(GO:0044464)	1
OG0027341	Cellular Component	cell(GO:0005623)	1
OG0027341	Cellular Component	membrane(GO:0016020)	1
OG0027341	Cellular Component	organelle part(GO:0044422)	1
OG0027341	Cellular Component	organelle(GO:0043226)	1
OG0027345	Cellular Component	cell part(GO:0044464)	1
OG0027345	Cellular Component	cell(GO:0005623)	1
OG0027345	Cellular Component	membrane part(GO:0044425)	1
OG0027345	Cellular Component	membrane(GO:0016020)	1
OG0027345	Cellular Component	organelle part(GO:0044422)	1
OG0027345	Cellular Component	organelle(GO:0043226)	1
OG0027345	Cellular Component	protein-containing complex(GO:0032991)	1
OG0027353	Cellular Component	cell part(GO:0044464)	1
OG0027353	Cellular Component	cell(GO:0005623)	1
OG0027353	Cellular Component	membrane-enclosed lumen(GO:0031974)	1
OG0027353	Cellular Component	organelle part(GO:0044422)	1
OG0027353	Cellular Component	organelle(GO:0043226)	1
OG0027355	Cellular Component	membrane(GO:0016020)	1
OG0027359	Cellular Component	cell part(GO:0044464)	1
OG0027359	Cellular Component	cell(GO:0005623)	1
OG0027359	Cellular Component	membrane(GO:0016020)	1
OG0027359	Cellular Component	organelle part(GO:0044422)	1
OG0027359	Cellular Component	organelle(GO:0043226)	1
OG0027363	Cellular Component	cell part(GO:0044464)	1
OG0027363	Cellular Component	cell(GO:0005623)	1
OG0027363	Cellular Component	organelle(GO:0043226)	1
OG0027368	Cellular Component	membrane(GO:0016020)	1
OG0027369	Cellular Component	cell part(GO:0044464)	1
OG0027369	Cellular Component	cell(GO:0005623)	1
OG0027369	Cellular Component	membrane(GO:0016020)	1

OG0027369	Cellular Component	organelle part(GO:0044422)	1
OG0027369	Cellular Component	organelle(GO:0043226)	1
OG0027370	Cellular Component	cell part(GO:0044464)	1
OG0027370	Cellular Component	cell(GO:0005623)	1
OG0027370	Cellular Component	membrane(GO:0016020)	1
OG0027374	Cellular Component	cell part(GO:0044464)	1
OG0027374	Cellular Component	cell(GO:0005623)	1
OG0027376	Cellular Component	cell part(GO:0044464)	1
OG0027376	Cellular Component	cell(GO:0005623)	1
OG0027376	Cellular Component	membrane part(GO:0044425)	1
OG0027376	Cellular Component	membrane(GO:0016020)	1
OG0027376	Cellular Component	organelle part(GO:0044422)	1
OG0027376	Cellular Component	organelle(GO:0043226)	1
OG0027376	Cellular Component	protein-containing complex(GO:0032991)	1
OG0027377	Cellular Component	cell part(GO:0044464)	1
OG0027377	Cellular Component	cell(GO:0005623)	1
OG0027377	Cellular Component	membrane-enclosed lumen(GO:0031974)	1
OG0027377	Cellular Component	organelle part(GO:0044422)	1
OG0027377	Cellular Component	organelle(GO:0043226)	1
OG0027379	Cellular Component	cell part(GO:0044464)	1
OG0027379	Cellular Component	cell(GO:0005623)	1
OG0027379	Cellular Component	organelle(GO:0043226)	1
OG0027380	Cellular Component	cell part(GO:0044464)	1
OG0027380	Cellular Component	cell(GO:0005623)	1
OG0027380	Cellular Component	membrane(GO:0016020)	1
OG0027380	Cellular Component	organelle part(GO:0044422)	1
OG0027380	Cellular Component	organelle(GO:0043226)	1
OG0027381	Cellular Component	cell part(GO:0044464)	1
OG0027381	Cellular Component	cell(GO:0005623)	1
OG0027381	Cellular Component	membrane(GO:0016020)	1
OG0027381	Cellular Component	organelle part(GO:0044422)	1
OG0027381	Cellular Component	organelle(GO:0043226)	1
OG0027383	Cellular Component	cell part(GO:0044464)	1
OG0027383	Cellular Component	cell(GO:0005623)	1
OG0027383	Cellular Component	membrane(GO:0016020)	1
OG0027383	Cellular Component	organelle part(GO:0044422)	1
OG0027383	Cellular Component	organelle(GO:0043226)	1
OG0027389	Cellular Component	cell part(GO:0044464)	1
OG0027389	Cellular Component	cell(GO:0005623)	1
OG0027389	Cellular Component	membrane(GO:0016020)	1
OG0027389	Cellular Component	organelle part(GO:0044422)	1
OG0027389	Cellular Component	organelle(GO:0043226)	1
OG0027390	Cellular Component	cell part(GO:0044464)	1
OG0027390	Cellular Component	cell(GO:0005623)	1
OG0027390	Cellular Component	extracellular region(GO:0005576)	1
OG0027396	Cellular Component	cell part(GO:0044464)	1
OG0027396	Cellular Component	cell(GO:0005623)	1

OG0027396	Cellular Component	organelle(GO:0043226)	1
OG0027400	Cellular Component	cell part(GO:0044464)	1
OG0027400	Cellular Component	cell(GO:0005623)	1
OG0027400	Cellular Component	membrane(GO:0016020)	1
OG0027400	Cellular Component	organelle part(GO:0044422)	1
OG0027400	Cellular Component	organelle(GO:0043226)	1
OG0027402	Cellular Component	cell part(GO:0044464)	1
OG0027402	Cellular Component	cell(GO:0005623)	1
OG0027410	Cellular Component	cell part(GO:0044464)	1
OG0027410	Cellular Component	cell(GO:0005623)	1
OG0027410	Cellular Component	membrane(GO:0016020)	1
OG0027417	Cellular Component	cell part(GO:0044464)	1
OG0027417	Cellular Component	cell(GO:0005623)	1
OG0027417	Cellular Component	membrane(GO:0016020)	1
OG0027418	Cellular Component	cell part(GO:0044464)	1
OG0027418	Cellular Component	cell(GO:0005623)	1
OG0027418	Cellular Component	membrane(GO:0016020)	1
OG0027419	Cellular Component	cell part(GO:0044464)	1
OG0027419	Cellular Component	cell(GO:0005623)	1
OG0027422	Cellular Component	cell part(GO:0044464)	1
OG0027422	Cellular Component	cell(GO:0005623)	1
OG0027422	Cellular Component	membrane(GO:0016020)	1
OG0027432	Cellular Component	cell part(GO:0044464)	1
OG0027432	Cellular Component	cell(GO:0005623)	1
OG0027432	Cellular Component	organelle(GO:0043226)	1
OG0027433	Cellular Component	cell part(GO:0044464)	1
OG0027433	Cellular Component	cell(GO:0005623)	1
OG0027433	Cellular Component	membrane part(GO:0044425)	1
OG0027433	Cellular Component	membrane(GO:0016020)	1
OG0027433	Cellular Component	organelle part(GO:0044422)	1
OG0027433	Cellular Component	organelle(GO:0043226)	1
OG0027433	Cellular Component	protein-containing complex(GO:0032991)	1
OG0027437	Cellular Component	cell part(GO:0044464)	1
OG0027437	Cellular Component	cell(GO:0005623)	1
OG0027437	Cellular Component	membrane(GO:0016020)	1
OG0027437	Cellular Component	organelle(GO:0043226)	1
OG0027439	Cellular Component	cell part(GO:0044464)	1
OG0027439	Cellular Component	cell(GO:0005623)	1
OG0027439	Cellular Component	membrane(GO:0016020)	1
OG0027439	Cellular Component	membrane-enclosed lumen(GO:0031974)	1
OG0027439	Cellular Component	organelle part(GO:0044422)	1
OG0027439	Cellular Component	organelle(GO:0043226)	1
OG0027439	Cellular Component	protein-containing complex(GO:0032991)	1
OG0027442	Cellular Component	cell part(GO:0044464)	1
OG0027442	Cellular Component	cell(GO:0005623)	1
OG0027442	Cellular Component	organelle(GO:0043226)	1
OG0027443	Cellular Component	cell part(GO:0044464)	1

OG0027443	Cellular Component	cell(GO:0005623)	1
OG0027443	Cellular Component	membrane(GO:0016020)	1
OG0027445	Cellular Component	cell part(GO:0044464)	1
OG0027445	Cellular Component	cell(GO:0005623)	1
OG0027445	Cellular Component	organelle(GO:0043226)	1
OG0027452	Cellular Component	cell part(GO:0044464)	1
OG0027452	Cellular Component	cell(GO:0005623)	1
OG0027452	Cellular Component	organelle(GO:0043226)	1
OG0027454	Cellular Component	cell part(GO:0044464)	1
OG0027454	Cellular Component	cell(GO:0005623)	1
OG0027454	Cellular Component	membrane(GO:0016020)	1
OG0027454	Cellular Component	organelle(GO:0043226)	1
OG0027454	Cellular Component	protein-containing complex(GO:0032991)	1
OG0027458	Cellular Component	cell part(GO:0044464)	1
OG0027458	Cellular Component	cell(GO:0005623)	1
OG0027458	Cellular Component	membrane(GO:0016020)	1
OG0027458	Cellular Component	organelle part(GO:0044422)	1
OG0027458	Cellular Component	organelle(GO:0043226)	1
OG0027458	Cellular Component	protein-containing complex(GO:0032991)	1
OG0027461	Cellular Component	cell part(GO:0044464)	1
OG0027461	Cellular Component	cell(GO:0005623)	1
OG0027461	Cellular Component	organelle(GO:0043226)	1
OG0027472	Cellular Component	cell part(GO:0044464)	1
OG0027472	Cellular Component	cell(GO:0005623)	1
OG0027472	Cellular Component	membrane(GO:0016020)	1
OG0027472	Cellular Component	organelle part(GO:0044422)	1
OG0027472	Cellular Component	organelle(GO:0043226)	1
OG0027473	Cellular Component	cell part(GO:0044464)	1
OG0027473	Cellular Component	cell(GO:0005623)	1
OG0027473	Cellular Component	organelle(GO:0043226)	1
OG0027474	Cellular Component	cell part(GO:0044464)	1
OG0027474	Cellular Component	cell(GO:0005623)	1
OG0027474	Cellular Component	membrane(GO:0016020)	1
OG0027474	Cellular Component	organelle part(GO:0044422)	1
OG0027474	Cellular Component	organelle(GO:0043226)	1
OG0027475	Cellular Component	cell part(GO:0044464)	1
OG0027475	Cellular Component	cell(GO:0005623)	1
OG0027475	Cellular Component	extracellular region(GO:0005576)	1
OG0027475	Cellular Component	membrane(GO:0016020)	1
OG0027475	Cellular Component	organelle part(GO:0044422)	1
OG0027475	Cellular Component	organelle(GO:0043226)	1
OG0027479	Cellular Component	cell part(GO:0044464)	1
OG0027479	Cellular Component	cell(GO:0005623)	1
OG0027479	Cellular Component	organelle(GO:0043226)	1
OG0027482	Cellular Component	cell part(GO:0044464)	1
OG0027482	Cellular Component	cell(GO:0005623)	1
OG0027482	Cellular Component	membrane(GO:0016020)	1

OG0027482	Cellular Component	organelle part(GO:0044422)	1
OG0027482	Cellular Component	organelle(GO:0043226)	1
OG0027483	Cellular Component	cell part(GO:0044464)	1
OG0027483	Cellular Component	cell(GO:0005623)	1
OG0027483	Cellular Component	membrane(GO:0016020)	1
OG0027483	Cellular Component	organelle part(GO:0044422)	1
OG0027483	Cellular Component	organelle(GO:0043226)	1
OG0027486	Cellular Component	cell part(GO:0044464)	1
OG0027486	Cellular Component	cell(GO:0005623)	1
OG0027486	Cellular Component	organelle(GO:0043226)	1
OG0027488	Cellular Component	cell part(GO:0044464)	1
OG0027488	Cellular Component	cell(GO:0005623)	1
OG0027488	Cellular Component	organelle(GO:0043226)	1
OG0027489	Cellular Component	cell part(GO:0044464)	1
OG0027489	Cellular Component	cell(GO:0005623)	1
OG0027489	Cellular Component	organelle(GO:0043226)	1
OG0027490	Cellular Component	cell part(GO:0044464)	1
OG0027490	Cellular Component	cell(GO:0005623)	1
OG0027490	Cellular Component	membrane(GO:0016020)	1
OG0027491	Cellular Component	cell part(GO:0044464)	1
OG0027491	Cellular Component	cell(GO:0005623)	1
OG0027491	Cellular Component	membrane(GO:0016020)	1
OG0027492	Cellular Component	cell part(GO:0044464)	1
OG0027492	Cellular Component	cell(GO:0005623)	1
OG0027492	Cellular Component	membrane(GO:0016020)	1
OG0027492	Cellular Component	organelle part(GO:0044422)	1
OG0027492	Cellular Component	organelle(GO:0043226)	1
OG0027497	Cellular Component	cell part(GO:0044464)	1
OG0027497	Cellular Component	cell(GO:0005623)	1
OG0027497	Cellular Component	organelle part(GO:0044422)	1
OG0027497	Cellular Component	organelle(GO:0043226)	1
OG0027497	Cellular Component	protein-containing complex(GO:0032991)	1
OG0027498	Cellular Component	cell part(GO:0044464)	1
OG0027498	Cellular Component	cell(GO:0005623)	1
OG0027498	Cellular Component	membrane(GO:0016020)	1
OG0027499	Cellular Component	cell part(GO:0044464)	1
OG0027499	Cellular Component	cell(GO:0005623)	1
OG0027499	Cellular Component	organelle(GO:0043226)	1
OG0027502	Cellular Component	membrane(GO:0016020)	1
OG0027503	Cellular Component	membrane(GO:0016020)	1
OG0027505	Cellular Component	cell part(GO:0044464)	1
OG0027505	Cellular Component	cell(GO:0005623)	1
OG0027505	Cellular Component	membrane(GO:0016020)	1
OG0027506	Cellular Component	cell part(GO:0044464)	1
OG0027506	Cellular Component	cell(GO:0005623)	1
OG0027506	Cellular Component	membrane(GO:0016020)	1
OG0027507	Cellular Component	cell part(GO:0044464)	1

OG0027507	Cellular Component	cell(GO:0005623)	1
OG0027509	Cellular Component	cell part(GO:0044464)	1
OG0027509	Cellular Component	cell(GO:0005623)	1
OG0027509	Cellular Component	membrane(GO:0016020)	1
OG0027510	Cellular Component	cell part(GO:0044464)	1
OG0027510	Cellular Component	cell(GO:0005623)	1
OG0027510	Cellular Component	membrane(GO:0016020)	1
OG0027511	Cellular Component	cell part(GO:0044464)	1
OG0027511	Cellular Component	cell(GO:0005623)	1
OG0027511	Cellular Component	organelle(GO:0043226)	1
OG0027516	Cellular Component	cell part(GO:0044464)	1
OG0027516	Cellular Component	cell(GO:0005623)	1
OG0027516	Cellular Component	organelle(GO:0043226)	1
OG0027523	Cellular Component	cell part(GO:0044464)	1
OG0027523	Cellular Component	cell(GO:0005623)	1
OG0027523	Cellular Component	organelle(GO:0043226)	1
OG0027524	Cellular Component	cell junction(GO:0030054)	1
OG0027524	Cellular Component	cell part(GO:0044464)	1
OG0027524	Cellular Component	cell(GO:0005623)	1
OG0027524	Cellular Component	membrane(GO:0016020)	1
OG0027524	Cellular Component	organelle part(GO:0044422)	1
OG0027524	Cellular Component	organelle(GO:0043226)	1
OG0027524	Cellular Component	protein-containing complex(GO:0032991)	1
OG0027524	Cellular Component	symplast(GO:0055044)	1
OG0027525	Cellular Component	cell part(GO:0044464)	1
OG0027525	Cellular Component	cell(GO:0005623)	1
OG0027525	Cellular Component	organelle(GO:0043226)	1
OG0027528	Cellular Component	cell part(GO:0044464)	1
OG0027528	Cellular Component	cell(GO:0005623)	1
OG0027528	Cellular Component	organelle(GO:0043226)	1
OG0027530	Cellular Component	cell part(GO:0044464)	1
OG0027530	Cellular Component	cell(GO:0005623)	1
OG0027530	Cellular Component	organelle(GO:0043226)	1
OG0027535	Cellular Component	cell part(GO:0044464)	1
OG0027535	Cellular Component	cell(GO:0005623)	1
OG0027535	Cellular Component	membrane-enclosed lumen(GO:0031974)	1
OG0027535	Cellular Component	organelle part(GO:0044422)	1
OG0027535	Cellular Component	organelle(GO:0043226)	1
OG0027537	Cellular Component	cell part(GO:0044464)	1
OG0027537	Cellular Component	cell(GO:0005623)	1
OG0027537	Cellular Component	membrane part(GO:0044425)	1
OG0027537	Cellular Component	membrane(GO:0016020)	1
OG0027537	Cellular Component	organelle part(GO:0044422)	1
OG0027537	Cellular Component	organelle(GO:0043226)	1
OG0027538	Cellular Component	cell part(GO:0044464)	1
OG0027538	Cellular Component	cell(GO:0005623)	1
OG0027538	Cellular Component	protein-containing complex(GO:0032991)	1

OG0027539	Cellular Component	cell part(GO:0044464)	1
OG0027539	Cellular Component	cell(GO:0005623)	1
OG0027539	Cellular Component	organelle(GO:0043226)	1
OG0027540	Cellular Component	cell part(GO:0044464)	1
OG0027540	Cellular Component	cell(GO:0005623)	1
OG0027544	Cellular Component	membrane(GO:0016020)	1
OG0027546	Cellular Component	cell part(GO:0044464)	1
OG0027546	Cellular Component	cell(GO:0005623)	1
OG0027546	Cellular Component	membrane(GO:0016020)	1
OG0027546	Cellular Component	organelle(GO:0043226)	1
OG0027548	Cellular Component	cell part(GO:0044464)	1
OG0027548	Cellular Component	cell(GO:0005623)	1
OG0027548	Cellular Component	membrane(GO:0016020)	1
OG0027548	Cellular Component	organelle part(GO:0044422)	1
OG0027548	Cellular Component	organelle(GO:0043226)	1
OG0027552	Cellular Component	membrane(GO:0016020)	1
OG0027554	Cellular Component	cell junction(GO:0030054)	1
OG0027554	Cellular Component	cell part(GO:0044464)	1
OG0027554	Cellular Component	cell(GO:0005623)	1
OG0027554	Cellular Component	membrane part(GO:0044425)	1
OG0027554	Cellular Component	membrane(GO:0016020)	1
OG0027554	Cellular Component	symplast(GO:0055044)	1
OG0027560	Cellular Component	cell part(GO:0044464)	1
OG0027560	Cellular Component	cell(GO:0005623)	1
OG0027560	Cellular Component	membrane(GO:0016020)	1
OG0027560	Cellular Component	organelle part(GO:0044422)	1
OG0027560	Cellular Component	organelle(GO:0043226)	1
OG0027565	Cellular Component	cell part(GO:0044464)	1
OG0027565	Cellular Component	cell(GO:0005623)	1
OG0027565	Cellular Component	membrane(GO:0016020)	1
OG0027565	Cellular Component	membrane-enclosed lumen(GO:0031974)	1
OG0027565	Cellular Component	organelle part(GO:0044422)	1
OG0027565	Cellular Component	organelle(GO:0043226)	1
OG0027567	Cellular Component	cell part(GO:0044464)	1
OG0027567	Cellular Component	cell(GO:0005623)	1
OG0027569	Cellular Component	cell part(GO:0044464)	1
OG0027569	Cellular Component	cell(GO:0005623)	1
OG0027569	Cellular Component	membrane(GO:0016020)	1
OG0027570	Cellular Component	cell part(GO:0044464)	1
OG0027570	Cellular Component	cell(GO:0005623)	1
OG0027570	Cellular Component	membrane(GO:0016020)	1
OG0027571	Cellular Component	cell part(GO:0044464)	1
OG0027571	Cellular Component	cell(GO:0005623)	1
OG0027572	Cellular Component	cell part(GO:0044464)	1
OG0027572	Cellular Component	cell(GO:0005623)	1
OG0027572	Cellular Component	membrane(GO:0016020)	1
OG0027581	Cellular Component	cell part(GO:0044464)	1

OG0027581	Cellular Component	cell(GO:0005623)	1
OG0027581	Cellular Component	membrane(GO:0016020)	1
OG0027581	Cellular Component	organelle part(GO:0044422)	1
OG0027581	Cellular Component	organelle(GO:0043226)	1
OG0027585	Cellular Component	cell part(GO:0044464)	1
OG0027585	Cellular Component	cell(GO:0005623)	1
OG0027585	Cellular Component	membrane(GO:0016020)	1
OG0027585	Cellular Component	organelle part(GO:0044422)	1
OG0027585	Cellular Component	organelle(GO:0043226)	1
OG0027590	Cellular Component	cell part(GO:0044464)	1
OG0027590	Cellular Component	cell(GO:0005623)	1
OG0027590	Cellular Component	membrane(GO:0016020)	1
OG0027590	Cellular Component	organelle part(GO:0044422)	1
OG0027590	Cellular Component	organelle(GO:0043226)	1
OG0027592	Cellular Component	cell part(GO:0044464)	1
OG0027592	Cellular Component	cell(GO:0005623)	1
OG0027592	Cellular Component	membrane(GO:0016020)	1
OG0027605	Cellular Component	cell part(GO:0044464)	1
OG0027605	Cellular Component	cell(GO:0005623)	1
OG0027605	Cellular Component	membrane(GO:0016020)	1
OG0027605	Cellular Component	organelle part(GO:0044422)	1
OG0027605	Cellular Component	organelle(GO:0043226)	1
OG0027606	Cellular Component	cell junction(GO:0030054)	1
OG0027606	Cellular Component	cell part(GO:0044464)	1
OG0027606	Cellular Component	cell(GO:0005623)	1
OG0027606	Cellular Component	membrane(GO:0016020)	1
OG0027606	Cellular Component	organelle part(GO:0044422)	1
OG0027606	Cellular Component	organelle(GO:0043226)	1
OG0027606	Cellular Component	symplast(GO:0055044)	1
OG0027609	Cellular Component	cell part(GO:0044464)	1
OG0027609	Cellular Component	cell(GO:0005623)	1
OG0027609	Cellular Component	organelle(GO:0043226)	1
OG0027610	Cellular Component	cell part(GO:0044464)	1
OG0027610	Cellular Component	cell(GO:0005623)	1
OG0027610	Cellular Component	membrane(GO:0016020)	1
OG0027610	Cellular Component	organelle part(GO:0044422)	1
OG0027610	Cellular Component	organelle(GO:0043226)	1
OG0027613	Cellular Component	cell part(GO:0044464)	1
OG0027613	Cellular Component	cell(GO:0005623)	1
OG0027613	Cellular Component	organelle(GO:0043226)	1
OG0027614	Cellular Component	cell part(GO:0044464)	1
OG0027614	Cellular Component	cell(GO:0005623)	1
OG0027615	Cellular Component	cell part(GO:0044464)	1
OG0027615	Cellular Component	cell(GO:0005623)	1
OG0027615	Cellular Component	organelle(GO:0043226)	1
OG0027620	Cellular Component	cell junction(GO:0030054)	1
OG0027620	Cellular Component	cell part(GO:0044464)	1

OG0027620	Cellular Component	cell(GO:0005623)	1
OG0027620	Cellular Component	membrane(GO:0016020)	1
OG0027620	Cellular Component	symplast(GO:0055044)	1
OG0027626	Cellular Component	cell part(GO:0044464)	1
OG0027626	Cellular Component	cell(GO:0005623)	1
OG0027626	Cellular Component	membrane(GO:0016020)	1
OG0027628	Cellular Component	cell part(GO:0044464)	1
OG0027628	Cellular Component	cell(GO:0005623)	1
OG0027628	Cellular Component	membrane(GO:0016020)	1
OG0027634	Cellular Component	cell part(GO:0044464)	1
OG0027634	Cellular Component	cell(GO:0005623)	1
OG0027634	Cellular Component	protein-containing complex(GO:0032991)	1
OG0027636	Cellular Component	cell part(GO:0044464)	1
OG0027636	Cellular Component	cell(GO:0005623)	1
OG0027639	Cellular Component	cell part(GO:0044464)	1
OG0027639	Cellular Component	cell(GO:0005623)	1
OG0027640	Cellular Component	cell part(GO:0044464)	1
OG0027640	Cellular Component	cell(GO:0005623)	1
OG0027640	Cellular Component	membrane(GO:0016020)	1
OG0027642	Cellular Component	cell part(GO:0044464)	1
OG0027642	Cellular Component	cell(GO:0005623)	1
OG0027643	Cellular Component	cell part(GO:0044464)	1
OG0027643	Cellular Component	cell(GO:0005623)	1
OG0027649	Cellular Component	cell part(GO:0044464)	1
OG0027649	Cellular Component	cell(GO:0005623)	1
OG0027649	Cellular Component	membrane(GO:0016020)	1
OG0027650	Cellular Component	cell part(GO:0044464)	1
OG0027650	Cellular Component	cell(GO:0005623)	1
OG0027650	Cellular Component	membrane(GO:0016020)	1
OG0027654	Cellular Component	cell part(GO:0044464)	1
OG0027654	Cellular Component	cell(GO:0005623)	1
OG0027654	Cellular Component	membrane part(GO:0044425)	1
OG0027654	Cellular Component	membrane(GO:0016020)	1
OG0027654	Cellular Component	organelle part(GO:0044422)	1
OG0027654	Cellular Component	organelle(GO:0043226)	1
OG0027654	Cellular Component	protein-containing complex(GO:0032991)	1
OG0027655	Cellular Component	cell part(GO:0044464)	1
OG0027655	Cellular Component	cell(GO:0005623)	1
OG0027655	Cellular Component	membrane(GO:0016020)	1
OG0027658	Cellular Component	cell part(GO:0044464)	1
OG0027658	Cellular Component	cell(GO:0005623)	1
OG0027661	Cellular Component	cell part(GO:0044464)	1
OG0027661	Cellular Component	cell(GO:0005623)	1
OG0027661	Cellular Component	membrane part(GO:0044425)	1
OG0027661	Cellular Component	membrane(GO:0016020)	1
OG0027661	Cellular Component	organelle part(GO:0044422)	1
OG0027661	Cellular Component	organelle(GO:0043226)	1

OG0027661	Cellular Component	protein-containing complex(GO:0032991)	1
OG0027662	Cellular Component	cell part(GO:0044464)	1
OG0027662	Cellular Component	cell(GO:0005623)	1
OG0027662	Cellular Component	membrane(GO:0016020)	1
OG0027665	Cellular Component	cell part(GO:0044464)	1
OG0027665	Cellular Component	cell(GO:0005623)	1
OG0027667	Cellular Component	cell part(GO:0044464)	1
OG0027667	Cellular Component	cell(GO:0005623)	1
OG0027667	Cellular Component	membrane(GO:0016020)	1
OG0027670	Cellular Component	cell part(GO:0044464)	1
OG0027670	Cellular Component	cell(GO:0005623)	1
OG0027670	Cellular Component	membrane(GO:0016020)	1
OG0027674	Cellular Component	extracellular region(GO:0005576)	1
OG0027699	Cellular Component	cell part(GO:0044464)	1
OG0027699	Cellular Component	cell(GO:0005623)	1
OG0027699	Cellular Component	organelle(GO:0043226)	1
OG0027704	Cellular Component	cell part(GO:0044464)	1
OG0027704	Cellular Component	cell(GO:0005623)	1
OG0027704	Cellular Component	organelle(GO:0043226)	1
OG0027708	Cellular Component	cell part(GO:0044464)	1
OG0027708	Cellular Component	cell(GO:0005623)	1
OG0027708	Cellular Component	organelle(GO:0043226)	1
OG0027711	Cellular Component	cell part(GO:0044464)	1
OG0027711	Cellular Component	cell(GO:0005623)	1
OG0027711	Cellular Component	organelle(GO:0043226)	1
OG0027721	Cellular Component	cell junction(GO:0030054)	1
OG0027721	Cellular Component	cell part(GO:0044464)	1
OG0027721	Cellular Component	cell(GO:0005623)	1
OG0027721	Cellular Component	membrane(GO:0016020)	1
OG0027721	Cellular Component	membrane-enclosed lumen(GO:0031974)	1
OG0027721	Cellular Component	organelle part(GO:0044422)	1
OG0027721	Cellular Component	organelle(GO:0043226)	1
OG0027721	Cellular Component	protein-containing complex(GO:0032991)	1
OG0027721	Cellular Component	symplast(GO:0055044)	1
OG0027738	Cellular Component	cell part(GO:0044464)	1
OG0027738	Cellular Component	cell(GO:0005623)	1
OG0027738	Cellular Component	membrane part(GO:0044425)	1
OG0027738	Cellular Component	membrane(GO:0016020)	1
OG0027738	Cellular Component	organelle part(GO:0044422)	1
OG0027738	Cellular Component	organelle(GO:0043226)	1
OG0027738	Cellular Component	protein-containing complex(GO:0032991)	1
OG0027755	Cellular Component	cell part(GO:0044464)	1
OG0027755	Cellular Component	cell(GO:0005623)	1
OG0027755	Cellular Component	organelle(GO:0043226)	1
OG0027756	Cellular Component	cell part(GO:0044464)	1
OG0027756	Cellular Component	cell(GO:0005623)	1

OG0027756	Cellular Component	organelle(GO:0043226)	1
OG0027761	Cellular Component	cell part(GO:0044464)	1
OG0027761	Cellular Component	cell(GO:0005623)	1
OG0027761	Cellular Component	organelle(GO:0043226)	1
OG0027764	Cellular Component	cell part(GO:0044464)	1
OG0027764	Cellular Component	cell(GO:0005623)	1
OG0027764	Cellular Component	organelle(GO:0043226)	1
OG0027773	Cellular Component	cell part(GO:0044464)	1
OG0027773	Cellular Component	cell(GO:0005623)	1
OG0027773	Cellular Component	organelle(GO:0043226)	1
OG0027774	Cellular Component	cell part(GO:0044464)	1
OG0027774	Cellular Component	cell(GO:0005623)	1
OG0027774	Cellular Component	membrane part(GO:0044425)	1
OG0027774	Cellular Component	membrane(GO:0016020)	1
OG0027774	Cellular Component	organelle part(GO:0044422)	1
OG0027774	Cellular Component	organelle(GO:0043226)	1
OG0027775	Cellular Component	cell part(GO:0044464)	1
OG0027775	Cellular Component	cell(GO:0005623)	1
OG0027775	Cellular Component	organelle(GO:0043226)	1
OG0027777	Cellular Component	cell part(GO:0044464)	1
OG0027777	Cellular Component	cell(GO:0005623)	1
OG0027778	Cellular Component	cell part(GO:0044464)	1
OG0027778	Cellular Component	cell(GO:0005623)	1
OG0027783	Cellular Component	cell junction(GO:0030054)	1
OG0027783	Cellular Component	cell part(GO:0044464)	1
OG0027783	Cellular Component	cell(GO:0005623)	1
OG0027783	Cellular Component	membrane part(GO:0044425)	1
OG0027783	Cellular Component	membrane(GO:0016020)	1
OG0027783	Cellular Component	organelle part(GO:0044422)	1
OG0027783	Cellular Component	organelle(GO:0043226)	1
OG0027783	Cellular Component	symplast(GO:0055044)	1
OG0027791	Cellular Component	cell part(GO:0044464)	1
OG0027791	Cellular Component	cell(GO:0005623)	1
OG0027791	Cellular Component	membrane-enclosed lumen(GO:0031974)	1
OG0027791	Cellular Component	organelle part(GO:0044422)	1
OG0027791	Cellular Component	organelle(GO:0043226)	1
OG0027793	Cellular Component	cell part(GO:0044464)	1
OG0027793	Cellular Component	cell(GO:0005623)	1
OG0027793	Cellular Component	organelle(GO:0043226)	1
OG0027794	Cellular Component	cell part(GO:0044464)	1
OG0027794	Cellular Component	cell(GO:0005623)	1
OG0027794	Cellular Component	extracellular region part(GO:0044421)	1
OG0027794	Cellular Component	extracellular region(GO:0005576)	1
OG0027794	Cellular Component	membrane part(GO:0044425)	1
OG0027794	Cellular Component	membrane(GO:0016020)	1
OG0027794	Cellular Component	organelle part(GO:0044422)	1
OG0027794	Cellular Component	organelle(GO:0043226)	1

OG0027794	Cellular Component	synapse part(GO:0044456)	1
OG0027794	Cellular Component	synapse(GO:0045202)	1
OG0027796	Cellular Component	cell part(GO:0044464)	1
OG0027796	Cellular Component	cell(GO:0005623)	1
OG0027799	Cellular Component	cell part(GO:0044464)	1
OG0027799	Cellular Component	cell(GO:0005623)	1
OG0027800	Cellular Component	cell part(GO:0044464)	1
OG0027800	Cellular Component	cell(GO:0005623)	1
OG0027800	Cellular Component	extracellular region(GO:0005576)	1
OG0027800	Cellular Component	membrane(GO:0016020)	1
OG0027801	Cellular Component	cell part(GO:0044464)	1
OG0027801	Cellular Component	cell(GO:0005623)	1
OG0027801	Cellular Component	membrane part(GO:0044425)	1
OG0027801	Cellular Component	membrane(GO:0016020)	1
OG0027801	Cellular Component	organelle part(GO:0044422)	1
OG0027801	Cellular Component	organelle(GO:0043226)	1
OG0027801	Cellular Component	protein-containing complex(GO:0032991)	1
OG0027803	Cellular Component	cell part(GO:0044464)	1
OG0027803	Cellular Component	cell(GO:0005623)	1
OG0027803	Cellular Component	membrane(GO:0016020)	1
OG0027805	Cellular Component	cell part(GO:0044464)	1
OG0027805	Cellular Component	cell(GO:0005623)	1
OG0027805	Cellular Component	membrane(GO:0016020)	1
OG0027809	Cellular Component	cell part(GO:0044464)	1
OG0027809	Cellular Component	cell(GO:0005623)	1
OG0027809	Cellular Component	membrane(GO:0016020)	1
OG0027811	Cellular Component	cell part(GO:0044464)	1
OG0027811	Cellular Component	cell(GO:0005623)	1
OG0027811	Cellular Component	membrane(GO:0016020)	1
OG0027814	Cellular Component	cell part(GO:0044464)	1
OG0027814	Cellular Component	cell(GO:0005623)	1
OG0027814	Cellular Component	membrane part(GO:0044425)	1
OG0027814	Cellular Component	membrane(GO:0016020)	1
OG0027814	Cellular Component	organelle part(GO:0044422)	1
OG0027814	Cellular Component	organelle(GO:0043226)	1
OG0027814	Cellular Component	protein-containing complex(GO:0032991)	1
OG0027815	Cellular Component	cell part(GO:0044464)	1
OG0027815	Cellular Component	cell(GO:0005623)	1
OG0027815	Cellular Component	membrane(GO:0016020)	1
OG0027815	Cellular Component	organelle part(GO:0044422)	1
OG0027815	Cellular Component	organelle(GO:0043226)	1
OG0027817	Cellular Component	cell junction(GO:0030054)	1
OG0027817	Cellular Component	cell part(GO:0044464)	1
OG0027817	Cellular Component	cell(GO:0005623)	1
OG0027817	Cellular Component	membrane(GO:0016020)	1
OG0027817	Cellular Component	organelle part(GO:0044422)	1
OG0027817	Cellular Component	organelle(GO:0043226)	1

OG0027817	Cellular Component	symplast(GO:0055044)	1
OG0027820	Cellular Component	cell part(GO:0044464)	1
OG0027820	Cellular Component	cell(GO:0005623)	1
OG0027820	Cellular Component	organelle(GO:0043226)	1
OG0027821	Cellular Component	cell part(GO:0044464)	1
OG0027821	Cellular Component	cell(GO:0005623)	1
OG0027821	Cellular Component	membrane-enclosed lumen(GO:0031974)	1
OG0027821	Cellular Component	organelle part(GO:0044422)	1
OG0027821	Cellular Component	organelle(GO:0043226)	1
OG0027825	Cellular Component	cell part(GO:0044464)	1
OG0027825	Cellular Component	cell(GO:0005623)	1
OG0027825	Cellular Component	organelle(GO:0043226)	1
OG0027826	Cellular Component	cell part(GO:0044464)	1
OG0027826	Cellular Component	cell(GO:0005623)	1
OG0027826	Cellular Component	membrane(GO:0016020)	1
OG0027826	Cellular Component	membrane-enclosed lumen(GO:0031974)	1
OG0027826	Cellular Component	organelle part(GO:0044422)	1
OG0027826	Cellular Component	organelle(GO:0043226)	1
OG0027826	Cellular Component	protein-containing complex(GO:0032991)	1
OG0027828	Cellular Component	cell part(GO:0044464)	1
OG0027828	Cellular Component	cell(GO:0005623)	1
OG0027832	Cellular Component	cell part(GO:0044464)	1
OG0027832	Cellular Component	cell(GO:0005623)	1
OG0027832	Cellular Component	membrane(GO:0016020)	1
OG0027833	Cellular Component	membrane(GO:0016020)	1
OG0027836	Cellular Component	cell junction(GO:0030054)	1
OG0027836	Cellular Component	cell part(GO:0044464)	1
OG0027836	Cellular Component	cell(GO:0005623)	1
OG0027836	Cellular Component	membrane(GO:0016020)	1
OG0027836	Cellular Component	membrane-enclosed lumen(GO:0031974)	1
OG0027836	Cellular Component	organelle part(GO:0044422)	1
OG0027836	Cellular Component	organelle(GO:0043226)	1
OG0027836	Cellular Component	protein-containing complex(GO:0032991)	1
OG0027836	Cellular Component	symplast(GO:0055044)	1
OG0027837	Cellular Component	cell part(GO:0044464)	1
OG0027837	Cellular Component	cell(GO:0005623)	1
OG0027837	Cellular Component	organelle(GO:0043226)	1
OG0027840	Cellular Component	cell part(GO:0044464)	1
OG0027840	Cellular Component	cell(GO:0005623)	1
OG0027840	Cellular Component	extracellular region(GO:0005576)	1
OG0027840	Cellular Component	membrane(GO:0016020)	1
OG0027840	Cellular Component	organelle part(GO:0044422)	1
OG0027840	Cellular Component	organelle(GO:0043226)	1
OG0027843	Cellular Component	cell part(GO:0044464)	1
OG0027843	Cellular Component	cell(GO:0005623)	1
OG0027844	Cellular Component	cell part(GO:0044464)	1
OG0027844	Cellular Component	cell(GO:0005623)	1

OG0027844	Cellular Component	membrane(GO:0016020)	1
OG0027845	Cellular Component	cell part(GO:0044464)	1
OG0027845	Cellular Component	cell(GO:0005623)	1
OG0027845	Cellular Component	membrane(GO:0016020)	1
OG0027852	Cellular Component	cell part(GO:0044464)	1
OG0027852	Cellular Component	cell(GO:0005623)	1
OG0027852	Cellular Component	membrane(GO:0016020)	1
OG0027853	Cellular Component	cell part(GO:0044464)	1
OG0027853	Cellular Component	cell(GO:0005623)	1
OG0027856	Cellular Component	cell part(GO:0044464)	1
OG0027856	Cellular Component	cell(GO:0005623)	1
OG0027856	Cellular Component	membrane part(GO:0044425)	1
OG0027856	Cellular Component	membrane(GO:0016020)	1
OG0027856	Cellular Component	organelle part(GO:0044422)	1
OG0027856	Cellular Component	organelle(GO:0043226)	1
OG0027859	Cellular Component	cell part(GO:0044464)	1
OG0027859	Cellular Component	cell(GO:0005623)	1
OG0027859	Cellular Component	protein-containing complex(GO:0032991)	1
OG0027862	Cellular Component	membrane(GO:0016020)	1
OG0027863	Cellular Component	cell part(GO:0044464)	1
OG0027863	Cellular Component	cell(GO:0005623)	1
OG0027863	Cellular Component	membrane(GO:0016020)	1
OG0027863	Cellular Component	organelle(GO:0043226)	1
OG0027866	Cellular Component	cell part(GO:0044464)	1
OG0027866	Cellular Component	cell(GO:0005623)	1
OG0027866	Cellular Component	organelle(GO:0043226)	1
OG0027868	Cellular Component	cell part(GO:0044464)	1
OG0027868	Cellular Component	cell(GO:0005623)	1
OG0027868	Cellular Component	membrane part(GO:0044425)	1
OG0027868	Cellular Component	membrane(GO:0016020)	1
OG0027868	Cellular Component	organelle part(GO:0044422)	1
OG0027868	Cellular Component	organelle(GO:0043226)	1
OG0027868	Cellular Component	protein-containing complex(GO:0032991)	1
OG0027874	Cellular Component	cell part(GO:0044464)	1
OG0027874	Cellular Component	cell(GO:0005623)	1
OG0027874	Cellular Component	membrane(GO:0016020)	1
OG0027874	Cellular Component	organelle part(GO:0044422)	1
OG0027874	Cellular Component	organelle(GO:0043226)	1
OG0027875	Cellular Component	cell part(GO:0044464)	1
OG0027875	Cellular Component	cell(GO:0005623)	1
OG0027875	Cellular Component	membrane(GO:0016020)	1
OG0027875	Cellular Component	organelle part(GO:0044422)	1
OG0027875	Cellular Component	organelle(GO:0043226)	1
OG0027875	Cellular Component	protein-containing complex(GO:0032991)	1
OG0027876	Cellular Component	cell part(GO:0044464)	1
OG0027876	Cellular Component	cell(GO:0005623)	1

OG0027876	Cellular Component	membrane part(GO:0044425)	1
OG0027876	Cellular Component	membrane(GO:0016020)	1
OG0027876	Cellular Component	organelle part(GO:0044422)	1
OG0027876	Cellular Component	organelle(GO:0043226)	1
OG0027877	Cellular Component	cell part(GO:0044464)	1
OG0027877	Cellular Component	cell(GO:0005623)	1
OG0027877	Cellular Component	membrane(GO:0016020)	1
OG0027877	Cellular Component	organelle part(GO:0044422)	1
OG0027877	Cellular Component	organelle(GO:0043226)	1
OG0027883	Cellular Component	cell part(GO:0044464)	1
OG0027883	Cellular Component	cell(GO:0005623)	1
OG0027883	Cellular Component	organelle(GO:0043226)	1
OG0027884	Cellular Component	cell part(GO:0044464)	1
OG0027884	Cellular Component	cell(GO:0005623)	1
OG0027884	Cellular Component	membrane(GO:0016020)	1
OG0027887	Cellular Component	cell part(GO:0044464)	1
OG0027887	Cellular Component	cell(GO:0005623)	1
OG0027887	Cellular Component	extracellular region part(GO:0044421)	1
OG0027887	Cellular Component	extracellular region(GO:0005576)	1
OG0027887	Cellular Component	organelle part(GO:0044422)	1
OG0027887	Cellular Component	organelle(GO:0043226)	1
OG0027887	Cellular Component	protein-containing complex(GO:0032991)	1
OG0027889	Cellular Component	cell part(GO:0044464)	1
OG0027889	Cellular Component	cell(GO:0005623)	1
OG0027889	Cellular Component	membrane-enclosed lumen(GO:0031974)	1
OG0027889	Cellular Component	organelle part(GO:0044422)	1
OG0027889	Cellular Component	organelle(GO:0043226)	1
OG0027897	Cellular Component	cell part(GO:0044464)	1
OG0027897	Cellular Component	cell(GO:0005623)	1
OG0027897	Cellular Component	organelle(GO:0043226)	1
OG0027898	Cellular Component	cell part(GO:0044464)	1
OG0027898	Cellular Component	cell(GO:0005623)	1
OG0027898	Cellular Component	extracellular region(GO:0005576)	1
OG0027898	Cellular Component	organelle part(GO:0044422)	1
OG0027898	Cellular Component	organelle(GO:0043226)	1
OG0027899	Cellular Component	cell part(GO:0044464)	1
OG0027899	Cellular Component	cell(GO:0005623)	1
OG0027899	Cellular Component	extracellular region(GO:0005576)	1
OG0027899	Cellular Component	membrane(GO:0016020)	1
OG0027899	Cellular Component	organelle part(GO:0044422)	1
OG0027899	Cellular Component	organelle(GO:0043226)	1
OG0027901	Cellular Component	cell part(GO:0044464)	1
OG0027901	Cellular Component	cell(GO:0005623)	1
OG0027902	Cellular Component	cell part(GO:0044464)	1
OG0027902	Cellular Component	cell(GO:0005623)	1
OG0027902	Cellular Component	membrane part(GO:0044425)	1
OG0027902	Cellular Component	membrane(GO:0016020)	1

OG0027902	Cellular Component	organelle part(GO:0044422)	1
OG0027902	Cellular Component	organelle(GO:0043226)	1
OG0027904	Cellular Component	cell junction(GO:0030054)	1
OG0027904	Cellular Component	cell part(GO:0044464)	1
OG0027904	Cellular Component	cell(GO:0005623)	1
OG0027904	Cellular Component	organelle(GO:0043226)	1
OG0027904	Cellular Component	protein-containing complex(GO:0032991)	1
OG0027904	Cellular Component	symplast(GO:0055044)	1
OG0027905	Cellular Component	cell part(GO:0044464)	1
OG0027905	Cellular Component	cell(GO:0005623)	1
OG0027905	Cellular Component	membrane(GO:0016020)	1
OG0027905	Cellular Component	organelle part(GO:0044422)	1
OG0027905	Cellular Component	organelle(GO:0043226)	1
OG0027906	Cellular Component	cell part(GO:0044464)	1
OG0027906	Cellular Component	cell(GO:0005623)	1
OG0027906	Cellular Component	organelle(GO:0043226)	1
OG0027907	Cellular Component	cell part(GO:0044464)	1
OG0027907	Cellular Component	cell(GO:0005623)	1
OG0027907	Cellular Component	membrane-enclosed lumen(GO:0031974)	1
OG0027907	Cellular Component	organelle part(GO:0044422)	1
OG0027907	Cellular Component	organelle(GO:0043226)	1
OG0027908	Cellular Component	cell part(GO:0044464)	1
OG0027908	Cellular Component	cell(GO:0005623)	1
OG0027908	Cellular Component	membrane-enclosed lumen(GO:0031974)	1
OG0027908	Cellular Component	organelle part(GO:0044422)	1
OG0027908	Cellular Component	organelle(GO:0043226)	1
OG0027910	Cellular Component	cell part(GO:0044464)	1
OG0027910	Cellular Component	cell(GO:0005623)	1
OG0027910	Cellular Component	organelle part(GO:0044422)	1
OG0027910	Cellular Component	organelle(GO:0043226)	1
OG0027916	Cellular Component	cell part(GO:0044464)	1
OG0027916	Cellular Component	cell(GO:0005623)	1
OG0027916	Cellular Component	membrane(GO:0016020)	1
OG0027929	Cellular Component	cell part(GO:0044464)	1
OG0027929	Cellular Component	cell(GO:0005623)	1
OG0027929	Cellular Component	membrane(GO:0016020)	1
OG0027932	Cellular Component	cell part(GO:0044464)	1
OG0027932	Cellular Component	cell(GO:0005623)	1
OG0027932	Cellular Component	membrane-enclosed lumen(GO:0031974)	1
OG0027932	Cellular Component	organelle part(GO:0044422)	1
OG0027932	Cellular Component	organelle(GO:0043226)	1
OG0027932	Cellular Component	protein-containing complex(GO:0032991)	1
OG0027934	Cellular Component	cell part(GO:0044464)	1
OG0027934	Cellular Component	cell(GO:0005623)	1
OG0027934	Cellular Component	membrane(GO:0016020)	1
OG0027934	Cellular Component	organelle part(GO:0044422)	1
OG0027934	Cellular Component	organelle(GO:0043226)	1

OG0027935	Cellular Component	cell part(GO:0044464)	1
OG0027935	Cellular Component	cell(GO:0005623)	1
OG0027935	Cellular Component	organelle(GO:0043226)	1
OG0027935	Cellular Component	protein-containing complex(GO:0032991)	1
OG0027938	Cellular Component	cell part(GO:0044464)	1
OG0027938	Cellular Component	cell(GO:0005623)	1
OG0027938	Cellular Component	membrane(GO:0016020)	1
OG0027938	Cellular Component	organelle(GO:0043226)	1
OG0027940	Cellular Component	cell part(GO:0044464)	1
OG0027940	Cellular Component	cell(GO:0005623)	1
OG0027940	Cellular Component	organelle(GO:0043226)	1
OG0027941	Cellular Component	cell part(GO:0044464)	1
OG0027941	Cellular Component	cell(GO:0005623)	1
OG0027943	Cellular Component	cell part(GO:0044464)	1
OG0027943	Cellular Component	cell(GO:0005623)	1
OG0027943	Cellular Component	membrane part(GO:0044425)	1
OG0027943	Cellular Component	membrane(GO:0016020)	1
OG0027943	Cellular Component	organelle part(GO:0044422)	1
OG0027943	Cellular Component	organelle(GO:0043226)	1
OG0027943	Cellular Component	protein-containing complex(GO:0032991)	1
OG0027943	Cellular Component	synapse part(GO:0044456)	1
OG0027943	Cellular Component	synapse(GO:0045202)	1
OG0027945	Cellular Component	cell part(GO:0044464)	1
OG0027945	Cellular Component	cell(GO:0005623)	1
OG0027945	Cellular Component	membrane(GO:0016020)	1
OG0027945	Cellular Component	organelle part(GO:0044422)	1
OG0027945	Cellular Component	organelle(GO:0043226)	1
OG0027951	Cellular Component	cell part(GO:0044464)	1
OG0027951	Cellular Component	cell(GO:0005623)	1
OG0027951	Cellular Component	membrane part(GO:0044425)	1
OG0027951	Cellular Component	membrane(GO:0016020)	1
OG0027951	Cellular Component	organelle part(GO:0044422)	1
OG0027951	Cellular Component	organelle(GO:0043226)	1
OG0027952	Cellular Component	cell part(GO:0044464)	1
OG0027952	Cellular Component	cell(GO:0005623)	1
OG0027952	Cellular Component	organelle(GO:0043226)	1
OG0027953	Cellular Component	cell part(GO:0044464)	1
OG0027953	Cellular Component	cell(GO:0005623)	1
OG0027953	Cellular Component	organelle(GO:0043226)	1
OG0027954	Cellular Component	cell part(GO:0044464)	1
OG0027954	Cellular Component	cell(GO:0005623)	1
OG0027954	Cellular Component	organelle(GO:0043226)	1
OG0027958	Cellular Component	cell part(GO:0044464)	1
OG0027958	Cellular Component	cell(GO:0005623)	1
OG0027958	Cellular Component	membrane(GO:0016020)	1
OG0027962	Cellular Component	cell part(GO:0044464)	1
OG0027962	Cellular Component	cell(GO:0005623)	1

OG0027962	Cellular Component	membrane part(GO:0044425)	1
OG0027962	Cellular Component	membrane(GO:0016020)	1
OG0027964	Cellular Component	cell part(GO:0044464)	1
OG0027964	Cellular Component	cell(GO:0005623)	1
OG0027964	Cellular Component	organelle(GO:0043226)	1
OG0027966	Cellular Component	cell part(GO:0044464)	1
OG0027966	Cellular Component	cell(GO:0005623)	1
OG0027978	Cellular Component	cell part(GO:0044464)	1
OG0027978	Cellular Component	cell(GO:0005623)	1
OG0027978	Cellular Component	membrane(GO:0016020)	1
OG0027981	Cellular Component	cell part(GO:0044464)	1
OG0027981	Cellular Component	cell(GO:0005623)	1
OG0027984	Cellular Component	cell part(GO:0044464)	1
OG0027984	Cellular Component	cell(GO:0005623)	1
OG0027986	Cellular Component	cell part(GO:0044464)	1
OG0027986	Cellular Component	cell(GO:0005623)	1
OG0027987	Cellular Component	cell part(GO:0044464)	1
OG0027987	Cellular Component	cell(GO:0005623)	1
OG0027993	Cellular Component	cell part(GO:0044464)	1
OG0027993	Cellular Component	cell(GO:0005623)	1
OG0027993	Cellular Component	organelle(GO:0043226)	1
OG0027994	Cellular Component	cell part(GO:0044464)	1
OG0027994	Cellular Component	cell(GO:0005623)	1
OG0027994	Cellular Component	organelle part(GO:0044422)	1
OG0027994	Cellular Component	organelle(GO:0043226)	1
OG0027995	Cellular Component	cell part(GO:0044464)	1
OG0027995	Cellular Component	cell(GO:0005623)	1
OG0027995	Cellular Component	organelle part(GO:0044422)	1
OG0027995	Cellular Component	organelle(GO:0043226)	1
OG0027997	Cellular Component	cell part(GO:0044464)	1
OG0027997	Cellular Component	cell(GO:0005623)	1
OG0027997	Cellular Component	organelle part(GO:0044422)	1
OG0027997	Cellular Component	organelle(GO:0043226)	1
OG0028005	Cellular Component	cell part(GO:0044464)	1
OG0028005	Cellular Component	cell(GO:0005623)	1
OG0028005	Cellular Component	membrane(GO:0016020)	1
OG0028009	Cellular Component	cell part(GO:0044464)	1
OG0028009	Cellular Component	cell(GO:0005623)	1
OG0028009	Cellular Component	membrane(GO:0016020)	1
OG0028009	Cellular Component	organelle part(GO:0044422)	1
OG0028009	Cellular Component	organelle(GO:0043226)	1
OG0028012	Cellular Component	cell part(GO:0044464)	1
OG0028012	Cellular Component	cell(GO:0005623)	1
OG0028012	Cellular Component	membrane(GO:0016020)	1
OG0028012	Cellular Component	organelle part(GO:0044422)	1
OG0028012	Cellular Component	organelle(GO:0043226)	1
OG0028014	Cellular Component	cell part(GO:0044464)	1

OG0028014	Cellular Component	cell(GO:0005623)	1
OG0028014	Cellular Component	organelle(GO:0043226)	1
OG0028018	Cellular Component	extracellular region part(GO:0044421)	1
OG0028018	Cellular Component	extracellular region(GO:0005576)	1
OG0028019	Cellular Component	cell junction(GO:0030054)	1
OG0028019	Cellular Component	cell part(GO:0044464)	1
OG0028019	Cellular Component	cell(GO:0005623)	1
OG0028019	Cellular Component	symplast(GO:0055044)	1
OG0028021	Cellular Component	cell junction(GO:0030054)	1
OG0028021	Cellular Component	cell part(GO:0044464)	1
OG0028021	Cellular Component	cell(GO:0005623)	1
OG0028021	Cellular Component	membrane(GO:0016020)	1
OG0028021	Cellular Component	symplast(GO:0055044)	1
OG0028022	Cellular Component	cell junction(GO:0030054)	1
OG0028022	Cellular Component	cell part(GO:0044464)	1
OG0028022	Cellular Component	cell(GO:0005623)	1
OG0028022	Cellular Component	membrane(GO:0016020)	1
OG0028022	Cellular Component	symplast(GO:0055044)	1
OG0028025	Cellular Component	cell junction(GO:0030054)	1
OG0028025	Cellular Component	cell part(GO:0044464)	1
OG0028025	Cellular Component	cell(GO:0005623)	1
OG0028025	Cellular Component	membrane(GO:0016020)	1
OG0028025	Cellular Component	symplast(GO:0055044)	1
OG0028026	Cellular Component	extracellular region(GO:0005576)	1
OG0028027	Cellular Component	cell part(GO:0044464)	1
OG0028027	Cellular Component	cell(GO:0005623)	1
OG0028027	Cellular Component	membrane(GO:0016020)	1
OG0028027	Cellular Component	organelle part(GO:0044422)	1
OG0028027	Cellular Component	organelle(GO:0043226)	1
OG0028031	Cellular Component	cell junction(GO:0030054)	1
OG0028031	Cellular Component	cell part(GO:0044464)	1
OG0028031	Cellular Component	cell(GO:0005623)	1
OG0028031	Cellular Component	membrane(GO:0016020)	1
OG0028031	Cellular Component	symplast(GO:0055044)	1
OG0028033	Cellular Component	cell part(GO:0044464)	1
OG0028033	Cellular Component	cell(GO:0005623)	1
OG0028033	Cellular Component	protein-containing complex(GO:0032991)	1
OG0028038	Cellular Component	cell part(GO:0044464)	1
OG0028038	Cellular Component	cell(GO:0005623)	1
OG0028038	Cellular Component	membrane(GO:0016020)	1
OG0028038	Cellular Component	organelle part(GO:0044422)	1
OG0028038	Cellular Component	organelle(GO:0043226)	1
OG0028041	Cellular Component	cell junction(GO:0030054)	1
OG0028041	Cellular Component	cell part(GO:0044464)	1
OG0028041	Cellular Component	cell(GO:0005623)	1
OG0028041	Cellular Component	membrane(GO:0016020)	1
OG0028041	Cellular Component	symplast(GO:0055044)	1

OG0028042	Cellular Component	cell junction(GO:0030054)	1
OG0028042	Cellular Component	cell part(GO:0044464)	1
OG0028042	Cellular Component	cell(GO:0005623)	1
OG0028042	Cellular Component	membrane(GO:0016020)	1
OG0028042	Cellular Component	membrane-enclosed lumen(GO:0031974)	1
OG0028042	Cellular Component	organelle part(GO:0044422)	1
OG0028042	Cellular Component	organelle(GO:0043226)	1
OG0028042	Cellular Component	protein-containing complex(GO:0032991)	1
OG0028042	Cellular Component	symplast(GO:0055044)	1
OG0028043	Cellular Component	cell part(GO:0044464)	1
OG0028043	Cellular Component	cell(GO:0005623)	1
OG0028043	Cellular Component	organelle(GO:0043226)	1
OG0028048	Cellular Component	cell part(GO:0044464)	1
OG0028048	Cellular Component	cell(GO:0005623)	1
OG0028048	Cellular Component	membrane(GO:0016020)	1
OG0028048	Cellular Component	organelle(GO:0043226)	1
OG0028049	Cellular Component	cell part(GO:0044464)	1
OG0028049	Cellular Component	cell(GO:0005623)	1
OG0028049	Cellular Component	membrane(GO:0016020)	1
OG0028049	Cellular Component	organelle part(GO:0044422)	1
OG0028049	Cellular Component	organelle(GO:0043226)	1
OG0028050	Cellular Component	membrane(GO:0016020)	1
OG0028052	Cellular Component	cell part(GO:0044464)	1
OG0028052	Cellular Component	cell(GO:0005623)	1
OG0028052	Cellular Component	organelle part(GO:0044422)	1
OG0028052	Cellular Component	organelle(GO:0043226)	1
OG0028057	Cellular Component	cell part(GO:0044464)	1
OG0028057	Cellular Component	cell(GO:0005623)	1
OG0028057	Cellular Component	membrane(GO:0016020)	1
OG0028059	Cellular Component	cell part(GO:0044464)	1
OG0028059	Cellular Component	cell(GO:0005623)	1
OG0028064	Cellular Component	cell part(GO:0044464)	1
OG0028064	Cellular Component	cell(GO:0005623)	1
OG0028064	Cellular Component	membrane part(GO:0044425)	1
OG0028064	Cellular Component	membrane(GO:0016020)	1
OG0028064	Cellular Component	organelle part(GO:0044422)	1
OG0028064	Cellular Component	organelle(GO:0043226)	1
OG0028064	Cellular Component	protein-containing complex(GO:0032991)	1
OG0028071	Cellular Component	cell part(GO:0044464)	1
OG0028071	Cellular Component	cell(GO:0005623)	1
OG0028071	Cellular Component	protein-containing complex(GO:0032991)	1
OG0028072	Cellular Component	cell part(GO:0044464)	1
OG0028072	Cellular Component	cell(GO:0005623)	1
OG0028073	Cellular Component	cell part(GO:0044464)	1
OG0028073	Cellular Component	cell(GO:0005623)	1
OG0028074	Cellular Component	cell part(GO:0044464)	1

OG0028074	Cellular Component	cell(GO:0005623)	1
OG0028074	Cellular Component	organelle(GO:0043226)	1
OG0028074	Cellular Component	protein-containing complex(GO:0032991)	1
OG0028076	Cellular Component	cell part(GO:0044464)	1
OG0028076	Cellular Component	cell(GO:0005623)	1
OG0028077	Cellular Component	cell part(GO:0044464)	1
OG0028077	Cellular Component	cell(GO:0005623)	1
OG0028078	Cellular Component	cell part(GO:0044464)	1
OG0028078	Cellular Component	cell(GO:0005623)	1
OG0028078	Cellular Component	membrane(GO:0016020)	1
OG0028079	Cellular Component	cell part(GO:0044464)	1
OG0028079	Cellular Component	cell(GO:0005623)	1
OG0028079	Cellular Component	membrane(GO:0016020)	1
OG0028081	Cellular Component	cell part(GO:0044464)	1
OG0028081	Cellular Component	cell(GO:0005623)	1
OG0028083	Cellular Component	cell part(GO:0044464)	1
OG0028083	Cellular Component	cell(GO:0005623)	1
OG0028083	Cellular Component	membrane part(GO:0044425)	1
OG0028083	Cellular Component	membrane(GO:0016020)	1
OG0028083	Cellular Component	protein-containing complex(GO:0032991)	1
OG0028086	Cellular Component	cell part(GO:0044464)	1
OG0028086	Cellular Component	cell(GO:0005623)	1
OG0028086	Cellular Component	membrane(GO:0016020)	1
OG0028087	Cellular Component	cell part(GO:0044464)	1
OG0028087	Cellular Component	cell(GO:0005623)	1
OG0028087	Cellular Component	membrane(GO:0016020)	1
OG0028089	Cellular Component	cell part(GO:0044464)	1
OG0028089	Cellular Component	cell(GO:0005623)	1
OG0028089	Cellular Component	protein-containing complex(GO:0032991)	1
OG0028090	Cellular Component	cell part(GO:0044464)	1
OG0028090	Cellular Component	cell(GO:0005623)	1
OG0028090	Cellular Component	membrane(GO:0016020)	1
OG0028091	Cellular Component	cell part(GO:0044464)	1
OG0028091	Cellular Component	cell(GO:0005623)	1
OG0028091	Cellular Component	membrane(GO:0016020)	1
OG0028091	Cellular Component	protein-containing complex(GO:0032991)	1
OG0028093	Cellular Component	cell part(GO:0044464)	1
OG0028093	Cellular Component	cell(GO:0005623)	1
OG0028099	Cellular Component	cell part(GO:0044464)	1
OG0028099	Cellular Component	cell(GO:0005623)	1
OG0028099	Cellular Component	protein-containing complex(GO:0032991)	1
OG0028116	Cellular Component	cell part(GO:0044464)	1
OG0028116	Cellular Component	cell(GO:0005623)	1
OG0028116	Cellular Component	organelle(GO:0043226)	1
OG0028119	Cellular Component	cell part(GO:0044464)	1
OG0028119	Cellular Component	cell(GO:0005623)	1

OG0028119	Cellular Component	membrane part(GO:0044425)	1
OG0028119	Cellular Component	membrane(GO:0016020)	1
OG0028119	Cellular Component	organelle part(GO:0044422)	1
OG0028119	Cellular Component	organelle(GO:0043226)	1
OG0028119	Cellular Component	protein-containing complex(GO:0032991)	1
OG0028120	Cellular Component	cell part(GO:0044464)	1
OG0028120	Cellular Component	cell(GO:0005623)	1
OG0028120	Cellular Component	organelle(GO:0043226)	1
OG0028124	Cellular Component	cell part(GO:0044464)	1
OG0028124	Cellular Component	cell(GO:0005623)	1
OG0028125	Cellular Component	cell part(GO:0044464)	1
OG0028125	Cellular Component	cell(GO:0005623)	1
OG0028125	Cellular Component	membrane(GO:0016020)	1
OG0028128	Cellular Component	cell part(GO:0044464)	1
OG0028128	Cellular Component	cell(GO:0005623)	1
OG0028128	Cellular Component	membrane(GO:0016020)	1
OG0028128	Cellular Component	protein-containing complex(GO:0032991)	1
OG0028129	Cellular Component	cell part(GO:0044464)	1
OG0028129	Cellular Component	cell(GO:0005623)	1
OG0028129	Cellular Component	membrane(GO:0016020)	1
OG0028131	Cellular Component	cell part(GO:0044464)	1
OG0028131	Cellular Component	cell(GO:0005623)	1
OG0028131	Cellular Component	membrane(GO:0016020)	1
OG0028132	Cellular Component	cell part(GO:0044464)	1
OG0028132	Cellular Component	cell(GO:0005623)	1
OG0028132	Cellular Component	membrane(GO:0016020)	1
OG0028133	Cellular Component	cell part(GO:0044464)	1
OG0028133	Cellular Component	cell(GO:0005623)	1
OG0028133	Cellular Component	membrane part(GO:0044425)	1
OG0028133	Cellular Component	membrane(GO:0016020)	1
OG0028133	Cellular Component	organelle part(GO:0044422)	1
OG0028133	Cellular Component	organelle(GO:0043226)	1
OG0028133	Cellular Component	protein-containing complex(GO:0032991)	1
OG0028139	Cellular Component	cell part(GO:0044464)	1
OG0028139	Cellular Component	cell(GO:0005623)	1
OG0028139	Cellular Component	organelle(GO:0043226)	1
OG0028145	Cellular Component	cell part(GO:0044464)	1
OG0028145	Cellular Component	cell(GO:0005623)	1
OG0028145	Cellular Component	membrane part(GO:0044425)	1
OG0028145	Cellular Component	membrane(GO:0016020)	1
OG0028145	Cellular Component	membrane-enclosed lumen(GO:0031974)	1
OG0028145	Cellular Component	organelle part(GO:0044422)	1
OG0028145	Cellular Component	organelle(GO:0043226)	1
OG0028145	Cellular Component	protein-containing complex(GO:0032991)	1
OG0028146	Cellular Component	extracellular region(GO:0005576)	1
OG0028147	Cellular Component	membrane(GO:0016020)	1

OG0028148	Cellular Component	membrane(GO:0016020)	1
OG0028152	Cellular Component	cell part(GO:0044464)	1
OG0028152	Cellular Component	cell(GO:0005623)	1
OG0028152	Cellular Component	membrane part(GO:0044425)	1
OG0028152	Cellular Component	membrane(GO:0016020)	1
OG0028152	Cellular Component	organelle part(GO:0044422)	1
OG0028152	Cellular Component	organelle(GO:0043226)	1
OG0028153	Cellular Component	cell part(GO:0044464)	1
OG0028153	Cellular Component	cell(GO:0005623)	1
OG0028153	Cellular Component	membrane(GO:0016020)	1
OG0028153	Cellular Component	organelle(GO:0043226)	1
OG0028160	Cellular Component	cell part(GO:0044464)	1
OG0028160	Cellular Component	cell(GO:0005623)	1
OG0028160	Cellular Component	organelle part(GO:0044422)	1
OG0028160	Cellular Component	organelle(GO:0043226)	1
OG0028170	Cellular Component	cell part(GO:0044464)	1
OG0028170	Cellular Component	cell(GO:0005623)	1
OG0028170	Cellular Component	membrane part(GO:0044425)	1
OG0028170	Cellular Component	membrane(GO:0016020)	1
OG0028170	Cellular Component	organelle part(GO:0044422)	1
OG0028170	Cellular Component	organelle(GO:0043226)	1
OG0028170	Cellular Component	protein-containing complex(GO:0032991)	1
OG0028171	Cellular Component	cell part(GO:0044464)	1
OG0028171	Cellular Component	cell(GO:0005623)	1
OG0028172	Cellular Component	cell part(GO:0044464)	1
OG0028172	Cellular Component	cell(GO:0005623)	1
OG0028172	Cellular Component	membrane(GO:0016020)	1
OG0028173	Cellular Component	cell part(GO:0044464)	1
OG0028173	Cellular Component	cell(GO:0005623)	1
OG0028173	Cellular Component	extracellular region(GO:0005576)	1
OG0028174	Cellular Component	cell part(GO:0044464)	1
OG0028174	Cellular Component	cell(GO:0005623)	1
OG0028174	Cellular Component	membrane(GO:0016020)	1
OG0028175	Cellular Component	cell part(GO:0044464)	1
OG0028175	Cellular Component	cell(GO:0005623)	1
OG0028175	Cellular Component	membrane(GO:0016020)	1
OG0028176	Cellular Component	cell part(GO:0044464)	1
OG0028176	Cellular Component	cell(GO:0005623)	1
OG0028177	Cellular Component	cell part(GO:0044464)	1
OG0028177	Cellular Component	cell(GO:0005623)	1
OG0028177	Cellular Component	protein-containing complex(GO:0032991)	1
OG0028180	Cellular Component	cell part(GO:0044464)	1
OG0028180	Cellular Component	cell(GO:0005623)	1
OG0028181	Cellular Component	cell part(GO:0044464)	1
OG0028181	Cellular Component	cell(GO:0005623)	1
OG0028181	Cellular Component	membrane(GO:0016020)	1
OG0028182	Cellular Component	cell part(GO:0044464)	1

OG0028182	Cellular Component	cell(GO:0005623)	1
OG0028182	Cellular Component	organelle(GO:0043226)	1
OG0028184	Cellular Component	cell part(GO:0044464)	1
OG0028184	Cellular Component	cell(GO:0005623)	1
OG0028184	Cellular Component	organelle(GO:0043226)	1
OG0028193	Cellular Component	cell part(GO:0044464)	1
OG0028193	Cellular Component	cell(GO:0005623)	1
OG0028193	Cellular Component	organelle part(GO:0044422)	1
OG0028193	Cellular Component	organelle(GO:0043226)	1
OG0028194	Cellular Component	cell part(GO:0044464)	1
OG0028194	Cellular Component	cell(GO:0005623)	1
OG0028194	Cellular Component	membrane(GO:0016020)	1
OG0028196	Cellular Component	cell part(GO:0044464)	1
OG0028196	Cellular Component	cell(GO:0005623)	1
OG0028196	Cellular Component	organelle(GO:0043226)	1
OG0028197	Cellular Component	cell part(GO:0044464)	1
OG0028197	Cellular Component	cell(GO:0005623)	1
OG0028197	Cellular Component	organelle(GO:0043226)	1
OG0028201	Cellular Component	cell part(GO:0044464)	1
OG0028201	Cellular Component	cell(GO:0005623)	1
OG0028201	Cellular Component	membrane-enclosed lumen(GO:0031974)	1
OG0028201	Cellular Component	organelle part(GO:0044422)	1
OG0028201	Cellular Component	organelle(GO:0043226)	1
OG0028201	Cellular Component	protein-containing complex(GO:0032991)	1
OG0028202	Cellular Component	cell part(GO:0044464)	1
OG0028202	Cellular Component	cell(GO:0005623)	1
OG0028202	Cellular Component	organelle(GO:0043226)	1
OG0028205	Cellular Component	cell part(GO:0044464)	1
OG0028205	Cellular Component	cell(GO:0005623)	1
OG0028205	Cellular Component	membrane(GO:0016020)	1
OG0028205	Cellular Component	organelle part(GO:0044422)	1
OG0028205	Cellular Component	organelle(GO:0043226)	1
OG0028208	Cellular Component	cell junction(GO:0030054)	1
OG0028208	Cellular Component	cell part(GO:0044464)	1
OG0028208	Cellular Component	cell(GO:0005623)	1
OG0028208	Cellular Component	membrane(GO:0016020)	1
OG0028208	Cellular Component	symplast(GO:0055044)	1
OG0028209	Cellular Component	cell part(GO:0044464)	1
OG0028209	Cellular Component	cell(GO:0005623)	1
OG0028209	Cellular Component	extracellular region(GO:0005576)	1
OG0028209	Cellular Component	membrane(GO:0016020)	1
OG0028209	Cellular Component	organelle part(GO:0044422)	1
OG0028209	Cellular Component	organelle(GO:0043226)	1
OG0028210	Cellular Component	cell part(GO:0044464)	1
OG0028210	Cellular Component	cell(GO:0005623)	1
OG0028210	Cellular Component	membrane(GO:0016020)	1
OG0028210	Cellular Component	membrane-enclosed lumen(GO:0031974)	1

OG0028210	Cellular Component	organelle part(GO:0044422)	1
OG0028210	Cellular Component	organelle(GO:0043226)	1
OG0028210	Cellular Component	protein-containing complex(GO:0032991)	1
OG0028216	Cellular Component	cell part(GO:0044464)	1
OG0028216	Cellular Component	cell(GO:0005623)	1
OG0028216	Cellular Component	extracellular region(GO:0005576)	1
OG0028216	Cellular Component	membrane(GO:0016020)	1
OG0028216	Cellular Component	organelle part(GO:0044422)	1
OG0028216	Cellular Component	organelle(GO:0043226)	1
OG0028222	Cellular Component	cell junction(GO:0030054)	1
OG0028222	Cellular Component	cell part(GO:0044464)	1
OG0028222	Cellular Component	cell(GO:0005623)	1
OG0028222	Cellular Component	extracellular region(GO:0005576)	1
OG0028222	Cellular Component	membrane(GO:0016020)	1
OG0028222	Cellular Component	membrane-enclosed lumen(GO:0031974)	1
OG0028222	Cellular Component	organelle part(GO:0044422)	1
OG0028222	Cellular Component	organelle(GO:0043226)	1
OG0028222	Cellular Component	protein-containing complex(GO:0032991)	1
OG0028222	Cellular Component	symplast(GO:0055044)	1
OG0028224	Cellular Component	cell junction(GO:0030054)	1
OG0028224	Cellular Component	cell part(GO:0044464)	1
OG0028224	Cellular Component	cell(GO:0005623)	1
OG0028224	Cellular Component	extracellular region(GO:0005576)	1
OG0028224	Cellular Component	membrane(GO:0016020)	1
OG0028224	Cellular Component	membrane-enclosed lumen(GO:0031974)	1
OG0028224	Cellular Component	organelle part(GO:0044422)	1
OG0028224	Cellular Component	organelle(GO:0043226)	1
OG0028224	Cellular Component	protein-containing complex(GO:0032991)	1
OG0028224	Cellular Component	symplast(GO:0055044)	1
OG0028228	Cellular Component	cell part(GO:0044464)	1
OG0028228	Cellular Component	cell(GO:0005623)	1
OG0028228	Cellular Component	membrane(GO:0016020)	1
OG0028228	Cellular Component	organelle part(GO:0044422)	1
OG0028228	Cellular Component	organelle(GO:0043226)	1
OG0028229	Cellular Component	cell part(GO:0044464)	1
OG0028229	Cellular Component	cell(GO:0005623)	1
OG0028229	Cellular Component	membrane(GO:0016020)	1
OG0028229	Cellular Component	organelle part(GO:0044422)	1
OG0028229	Cellular Component	organelle(GO:0043226)	1
OG0028241	Cellular Component	cell part(GO:0044464)	1
OG0028241	Cellular Component	cell(GO:0005623)	1
OG0028241	Cellular Component	organelle(GO:0043226)	1
OG0028244	Cellular Component	cell part(GO:0044464)	1
OG0028244	Cellular Component	cell(GO:0005623)	1
OG0028244	Cellular Component	membrane part(GO:0044425)	1
OG0028244	Cellular Component	membrane(GO:0016020)	1

OG0028244	Cellular Component	organelle part(GO:0044422)	1
OG0028244	Cellular Component	organelle(GO:0043226)	1
OG0028244	Cellular Component	protein-containing complex(GO:0032991)	1
OG0028249	Cellular Component	cell part(GO:0044464)	1
OG0028249	Cellular Component	cell(GO:0005623)	1
OG0028249	Cellular Component	organelle part(GO:0044422)	1
OG0028249	Cellular Component	organelle(GO:0043226)	1
OG0028249	Cellular Component	protein-containing complex(GO:0032991)	1
OG0028250	Cellular Component	cell part(GO:0044464)	1
OG0028250	Cellular Component	cell(GO:0005623)	1
OG0028250	Cellular Component	organelle(GO:0043226)	1
OG0028251	Cellular Component	cell part(GO:0044464)	1
OG0028251	Cellular Component	cell(GO:0005623)	1
OG0028251	Cellular Component	organelle(GO:0043226)	1
OG0028255	Cellular Component	cell part(GO:0044464)	1
OG0028255	Cellular Component	cell(GO:0005623)	1
OG0028255	Cellular Component	membrane(GO:0016020)	1
OG0028255	Cellular Component	organelle part(GO:0044422)	1
OG0028255	Cellular Component	organelle(GO:0043226)	1
OG0028256	Cellular Component	cell part(GO:0044464)	1
OG0028256	Cellular Component	cell(GO:0005623)	1
OG0028256	Cellular Component	membrane part(GO:0044425)	1
OG0028256	Cellular Component	membrane(GO:0016020)	1
OG0028256	Cellular Component	organelle part(GO:0044422)	1
OG0028256	Cellular Component	organelle(GO:0043226)	1
OG0028258	Cellular Component	cell part(GO:0044464)	1
OG0028258	Cellular Component	cell(GO:0005623)	1
OG0028258	Cellular Component	organelle(GO:0043226)	1
OG0028259	Cellular Component	cell junction(GO:0030054)	1
OG0028259	Cellular Component	cell part(GO:0044464)	1
OG0028259	Cellular Component	cell(GO:0005623)	1
OG0028259	Cellular Component	membrane(GO:0016020)	1
OG0028259	Cellular Component	organelle part(GO:0044422)	1
OG0028259	Cellular Component	organelle(GO:0043226)	1
OG0028259	Cellular Component	protein-containing complex(GO:0032991)	1
OG0028259	Cellular Component	symplast(GO:0055044)	1
OG0028295	Cellular Component	cell junction(GO:0030054)	1
OG0028295	Cellular Component	cell part(GO:0044464)	1
OG0028295	Cellular Component	cell(GO:0005623)	1
OG0028295	Cellular Component	membrane(GO:0016020)	1
OG0028295	Cellular Component	membrane-enclosed lumen(GO:0031974)	1
OG0028295	Cellular Component	organelle part(GO:0044422)	1
OG0028295	Cellular Component	organelle(GO:0043226)	1
OG0028295	Cellular Component	protein-containing complex(GO:0032991)	1
OG0028295	Cellular Component	symplast(GO:0055044)	1
OG0028299	Cellular Component	cell part(GO:0044464)	1

OG0028299	Cellular Component	cell(GO:0005623)	1
OG0028299	Cellular Component	membrane part(GO:0044425)	1
OG0028299	Cellular Component	membrane(GO:0016020)	1
OG0028299	Cellular Component	organelle part(GO:0044422)	1
OG0028299	Cellular Component	organelle(GO:0043226)	1
OG0028299	Cellular Component	protein-containing complex(GO:0032991)	1
OG0028301	Cellular Component	cell part(GO:0044464)	1
OG0028301	Cellular Component	cell(GO:0005623)	1
OG0028301	Cellular Component	membrane(GO:0016020)	1
OG0028302	Cellular Component	cell part(GO:0044464)	1
OG0028302	Cellular Component	cell(GO:0005623)	1
OG0028304	Cellular Component	cell part(GO:0044464)	1
OG0028304	Cellular Component	cell(GO:0005623)	1
OG0028304	Cellular Component	membrane(GO:0016020)	1
OG0028305	Cellular Component	cell part(GO:0044464)	1
OG0028305	Cellular Component	cell(GO:0005623)	1
OG0028305	Cellular Component	organelle(GO:0043226)	1
OG0028308	Cellular Component	cell part(GO:0044464)	1
OG0028308	Cellular Component	cell(GO:0005623)	1
OG0028308	Cellular Component	organelle(GO:0043226)	1
OG0028323	Cellular Component	cell part(GO:0044464)	1
OG0028323	Cellular Component	cell(GO:0005623)	1
OG0028323	Cellular Component	organelle(GO:0043226)	1
OG0028326	Cellular Component	cell part(GO:0044464)	1
OG0028326	Cellular Component	cell(GO:0005623)	1
OG0028330	Cellular Component	cell part(GO:0044464)	1
OG0028330	Cellular Component	cell(GO:0005623)	1
OG0028330	Cellular Component	organelle(GO:0043226)	1
OG0028331	Cellular Component	cell part(GO:0044464)	1
OG0028331	Cellular Component	cell(GO:0005623)	1
OG0028334	Cellular Component	cell part(GO:0044464)	1
OG0028334	Cellular Component	cell(GO:0005623)	1
OG0028334	Cellular Component	membrane(GO:0016020)	1
OG0028334	Cellular Component	organelle(GO:0043226)	1
OG0028339	Cellular Component	cell part(GO:0044464)	1
OG0028339	Cellular Component	cell(GO:0005623)	1
OG0028339	Cellular Component	membrane(GO:0016020)	1
OG0028341	Cellular Component	cell part(GO:0044464)	1
OG0028341	Cellular Component	cell(GO:0005623)	1
OG0028341	Cellular Component	membrane(GO:0016020)	1
OG0028341	Cellular Component	organelle(GO:0043226)	1
OG0028345	Cellular Component	cell part(GO:0044464)	1
OG0028345	Cellular Component	cell(GO:0005623)	1
OG0028345	Cellular Component	organelle(GO:0043226)	1
OG0028346	Cellular Component	cell part(GO:0044464)	1
OG0028346	Cellular Component	cell(GO:0005623)	1
OG0028346	Cellular Component	organelle(GO:0043226)	1

OG0028353	Cellular Component	cell part(GO:0044464)	1
OG0028353	Cellular Component	cell(GO:0005623)	1
OG0028353	Cellular Component	organelle(GO:0043226)	1
OG0028358	Cellular Component	cell part(GO:0044464)	1
OG0028358	Cellular Component	cell(GO:0005623)	1
OG0028358	Cellular Component	organelle part(GO:0044422)	1
OG0028358	Cellular Component	organelle(GO:0043226)	1
OG0028358	Cellular Component	protein-containing complex(GO:0032991)	1
OG0028360	Cellular Component	cell part(GO:0044464)	1
OG0028360	Cellular Component	cell(GO:0005623)	1
OG0028360	Cellular Component	organelle part(GO:0044422)	1
OG0028360	Cellular Component	organelle(GO:0043226)	1
OG0028360	Cellular Component	protein-containing complex(GO:0032991)	1
OG0028361	Cellular Component	cell part(GO:0044464)	1
OG0028361	Cellular Component	cell(GO:0005623)	1
OG0028361	Cellular Component	organelle part(GO:0044422)	1
OG0028361	Cellular Component	organelle(GO:0043226)	1
OG0028361	Cellular Component	protein-containing complex(GO:0032991)	1
OG0028362	Cellular Component	cell part(GO:0044464)	1
OG0028362	Cellular Component	cell(GO:0005623)	1
OG0028362	Cellular Component	organelle part(GO:0044422)	1
OG0028362	Cellular Component	organelle(GO:0043226)	1
OG0028362	Cellular Component	protein-containing complex(GO:0032991)	1
OG0028365	Cellular Component	cell part(GO:0044464)	1
OG0028365	Cellular Component	cell(GO:0005623)	1
OG0028365	Cellular Component	organelle part(GO:0044422)	1
OG0028365	Cellular Component	organelle(GO:0043226)	1
OG0028365	Cellular Component	protein-containing complex(GO:0032991)	1
OG0028366	Cellular Component	cell part(GO:0044464)	1
OG0028366	Cellular Component	cell(GO:0005623)	1
OG0028366	Cellular Component	organelle part(GO:0044422)	1
OG0028366	Cellular Component	organelle(GO:0043226)	1
OG0028366	Cellular Component	protein-containing complex(GO:0032991)	1
OG0028373	Cellular Component	cell part(GO:0044464)	1
OG0028373	Cellular Component	cell(GO:0005623)	1
OG0028373	Cellular Component	membrane(GO:0016020)	1
OG0028373	Cellular Component	organelle part(GO:0044422)	1
OG0028373	Cellular Component	organelle(GO:0043226)	1
OG0028374	Cellular Component	cell part(GO:0044464)	1
OG0028374	Cellular Component	cell(GO:0005623)	1
OG0028374	Cellular Component	membrane(GO:0016020)	1
OG0028385	Cellular Component	membrane(GO:0016020)	1
OG0028388	Cellular Component	cell part(GO:0044464)	1
OG0028388	Cellular Component	cell(GO:0005623)	1
OG0028388	Cellular Component	membrane-enclosed lumen(GO:0031974)	1
OG0028388	Cellular Component	organelle part(GO:0044422)	1

OG0028388	Cellular Component	organelle(GO:0043226)	1
OG0028390	Cellular Component	cell part(GO:0044464)	1
OG0028390	Cellular Component	cell(GO:0005623)	1
OG0028390	Cellular Component	membrane part(GO:0044425)	1
OG0028390	Cellular Component	membrane(GO:0016020)	1
OG0028390	Cellular Component	nucleoid(GO:0009295)	1
OG0028390	Cellular Component	organelle part(GO:0044422)	1
OG0028390	Cellular Component	organelle(GO:0043226)	1
OG0028390	Cellular Component	protein-containing complex(GO:0032991)	1
OG0028391	Cellular Component	cell part(GO:0044464)	1
OG0028391	Cellular Component	cell(GO:0005623)	1
OG0028391	Cellular Component	organelle(GO:0043226)	1
OG0028391	Cellular Component	protein-containing complex(GO:0032991)	1
OG0028393	Cellular Component	extracellular region(GO:0005576)	1
OG0028394	Cellular Component	cell part(GO:0044464)	1
OG0028394	Cellular Component	cell(GO:0005623)	1
OG0028394	Cellular Component	organelle(GO:0043226)	1
OG0028396	Cellular Component	cell part(GO:0044464)	1
OG0028396	Cellular Component	cell(GO:0005623)	1
OG0028396	Cellular Component	membrane(GO:0016020)	1
OG0028396	Cellular Component	organelle part(GO:0044422)	1
OG0028396	Cellular Component	organelle(GO:0043226)	1
OG0028399	Cellular Component	cell part(GO:0044464)	1
OG0028399	Cellular Component	cell(GO:0005623)	1
OG0028405	Cellular Component	cell part(GO:0044464)	1
OG0028405	Cellular Component	cell(GO:0005623)	1
OG0028405	Cellular Component	membrane part(GO:0044425)	1
OG0028405	Cellular Component	membrane(GO:0016020)	1
OG0028405	Cellular Component	organelle part(GO:0044422)	1
OG0028405	Cellular Component	organelle(GO:0043226)	1
OG0028405	Cellular Component	protein-containing complex(GO:0032991)	1
OG0028410	Cellular Component	cell part(GO:0044464)	1
OG0028410	Cellular Component	cell(GO:0005623)	1
OG0028410	Cellular Component	membrane part(GO:0044425)	1
OG0028410	Cellular Component	membrane(GO:0016020)	1
OG0028410	Cellular Component	organelle(GO:0043226)	1
OG0028412	Cellular Component	cell part(GO:0044464)	1
OG0028412	Cellular Component	cell(GO:0005623)	1
OG0028412	Cellular Component	organelle(GO:0043226)	1
OG0028415	Cellular Component	cell part(GO:0044464)	1
OG0028415	Cellular Component	cell(GO:0005623)	1
OG0028415	Cellular Component	organelle part(GO:0044422)	1
OG0028415	Cellular Component	organelle(GO:0043226)	1
OG0028416	Cellular Component	cell part(GO:0044464)	1
OG0028416	Cellular Component	cell(GO:0005623)	1
OG0028416	Cellular Component	organelle part(GO:0044422)	1

OG0028416	Cellular Component	organelle(GO:0043226)	1
OG0028417	Cellular Component	cell part(GO:0044464)	1
OG0028417	Cellular Component	cell(GO:0005623)	1
OG0028417	Cellular Component	organelle(GO:0043226)	1
OG0028419	Cellular Component	cell part(GO:0044464)	1
OG0028419	Cellular Component	cell(GO:0005623)	1
OG0028419	Cellular Component	membrane(GO:0016020)	1
OG0028419	Cellular Component	organelle part(GO:0044422)	1
OG0028419	Cellular Component	organelle(GO:0043226)	1
OG0028419	Cellular Component	protein-containing complex(GO:0032991)	1
OG0028419	Cellular Component	supramolecular complex(GO:0099080)	1
OG0028420	Cellular Component	cell junction(GO:0030054)	1
OG0028420	Cellular Component	cell part(GO:0044464)	1
OG0028420	Cellular Component	cell(GO:0005623)	1
OG0028420	Cellular Component	symplast(GO:0055044)	1
OG0028421	Cellular Component	cell part(GO:0044464)	1
OG0028421	Cellular Component	cell(GO:0005623)	1
OG0028421	Cellular Component	membrane(GO:0016020)	1
OG0028433	Cellular Component	cell part(GO:0044464)	1
OG0028433	Cellular Component	cell(GO:0005623)	1
OG0028433	Cellular Component	organelle(GO:0043226)	1
OG0028438	Cellular Component	cell part(GO:0044464)	1
OG0028438	Cellular Component	cell(GO:0005623)	1
OG0028438	Cellular Component	membrane(GO:0016020)	1
OG0028438	Cellular Component	organelle part(GO:0044422)	1
OG0028438	Cellular Component	organelle(GO:0043226)	1
OG0028440	Cellular Component	cell part(GO:0044464)	1
OG0028440	Cellular Component	cell(GO:0005623)	1
OG0028440	Cellular Component	membrane-enclosed lumen(GO:0031974)	1
OG0028440	Cellular Component	organelle part(GO:0044422)	1
OG0028440	Cellular Component	organelle(GO:0043226)	1
OG0028440	Cellular Component	protein-containing complex(GO:0032991)	1
OG0028447	Cellular Component	cell part(GO:0044464)	1
OG0028447	Cellular Component	cell(GO:0005623)	1
OG0028447	Cellular Component	membrane-enclosed lumen(GO:0031974)	1
OG0028447	Cellular Component	organelle part(GO:0044422)	1
OG0028447	Cellular Component	organelle(GO:0043226)	1
OG0028447	Cellular Component	protein-containing complex(GO:0032991)	1
OG0028448	Cellular Component	cell part(GO:0044464)	1
OG0028448	Cellular Component	cell(GO:0005623)	1
OG0028448	Cellular Component	membrane-enclosed lumen(GO:0031974)	1
OG0028448	Cellular Component	organelle part(GO:0044422)	1
OG0028448	Cellular Component	organelle(GO:0043226)	1
OG0028448	Cellular Component	protein-containing complex(GO:0032991)	1
OG0028450	Cellular Component	cell part(GO:0044464)	1
OG0028450	Cellular Component	cell(GO:0005623)	1

OG0028468	Cellular Component	cell part(GO:0044464)	1
OG0028468	Cellular Component	cell(GO:0005623)	1
OG0028468	Cellular Component	membrane part(GO:0044425)	1
OG0028468	Cellular Component	membrane(GO:0016020)	1
OG0028468	Cellular Component	organelle(GO:0043226)	1
OG0028469	Cellular Component	cell part(GO:0044464)	1
OG0028469	Cellular Component	cell(GO:0005623)	1
OG0028470	Cellular Component	cell part(GO:0044464)	1
OG0028470	Cellular Component	cell(GO:0005623)	1
OG0028470	Cellular Component	organelle(GO:0043226)	1
OG0028470	Cellular Component	protein-containing complex(GO:0032991)	1
OG0028472	Cellular Component	membrane(GO:0016020)	1
OG0028484	Cellular Component	cell part(GO:0044464)	1
OG0028484	Cellular Component	cell(GO:0005623)	1
OG0028484	Cellular Component	membrane-enclosed lumen(GO:0031974)	1
OG0028484	Cellular Component	organelle part(GO:0044422)	1
OG0028484	Cellular Component	organelle(GO:0043226)	1
OG0028492	Cellular Component	cell part(GO:0044464)	1
OG0028492	Cellular Component	cell(GO:0005623)	1
OG0028492	Cellular Component	organelle(GO:0043226)	1
OG0028500	Cellular Component	cell part(GO:0044464)	1
OG0028500	Cellular Component	cell(GO:0005623)	1
OG0028500	Cellular Component	membrane(GO:0016020)	1
OG0028500	Cellular Component	organelle part(GO:0044422)	1
OG0028500	Cellular Component	organelle(GO:0043226)	1
OG0028500	Cellular Component	protein-containing complex(GO:0032991)	1
OG0028502	Cellular Component	cell part(GO:0044464)	1
OG0028502	Cellular Component	cell(GO:0005623)	1
OG0028502	Cellular Component	membrane(GO:0016020)	1
OG0028502	Cellular Component	organelle part(GO:0044422)	1
OG0028502	Cellular Component	organelle(GO:0043226)	1
OG0028504	Cellular Component	cell part(GO:0044464)	1
OG0028504	Cellular Component	cell(GO:0005623)	1
OG0028504	Cellular Component	organelle(GO:0043226)	1
OG0028506	Cellular Component	cell part(GO:0044464)	1
OG0028506	Cellular Component	cell(GO:0005623)	1
OG0028506	Cellular Component	membrane(GO:0016020)	1
OG0028506	Cellular Component	organelle part(GO:0044422)	1
OG0028506	Cellular Component	organelle(GO:0043226)	1
OG0028508	Cellular Component	cell part(GO:0044464)	1
OG0028508	Cellular Component	cell(GO:0005623)	1
OG0028508	Cellular Component	membrane(GO:0016020)	1
OG0028508	Cellular Component	organelle part(GO:0044422)	1
OG0028508	Cellular Component	organelle(GO:0043226)	1
OG0028508	Cellular Component	protein-containing complex(GO:0032991)	1
OG0028516	Cellular Component	cell part(GO:0044464)	1

OG0028516	Cellular Component	cell(GO:0005623)	1
OG0028516	Cellular Component	extracellular region(GO:0005576)	1
OG0028522	Cellular Component	cell part(GO:0044464)	1
OG0028522	Cellular Component	cell(GO:0005623)	1
OG0028522	Cellular Component	membrane(GO:0016020)	1
OG0028522	Cellular Component	organelle part(GO:0044422)	1
OG0028522	Cellular Component	organelle(GO:0043226)	1
OG0028524	Cellular Component	cell part(GO:0044464)	1
OG0028524	Cellular Component	cell(GO:0005623)	1
OG0028524	Cellular Component	organelle(GO:0043226)	1
OG0028530	Cellular Component	cell part(GO:0044464)	1
OG0028530	Cellular Component	cell(GO:0005623)	1
OG0028530	Cellular Component	organelle(GO:0043226)	1
OG0028533	Cellular Component	cell part(GO:0044464)	1
OG0028533	Cellular Component	cell(GO:0005623)	1
OG0028534	Cellular Component	cell part(GO:0044464)	1
OG0028534	Cellular Component	cell(GO:0005623)	1
OG0028534	Cellular Component	organelle(GO:0043226)	1
OG0028536	Cellular Component	cell part(GO:0044464)	1
OG0028536	Cellular Component	cell(GO:0005623)	1
OG0028537	Cellular Component	cell part(GO:0044464)	1
OG0028537	Cellular Component	cell(GO:0005623)	1
OG0028537	Cellular Component	organelle(GO:0043226)	1
OG0028538	Cellular Component	cell part(GO:0044464)	1
OG0028538	Cellular Component	cell(GO:0005623)	1
OG0028538	Cellular Component	organelle(GO:0043226)	1
OG0028539	Cellular Component	cell part(GO:0044464)	1
OG0028539	Cellular Component	cell(GO:0005623)	1
OG0028539	Cellular Component	membrane part(GO:0044425)	1
OG0028539	Cellular Component	membrane(GO:0016020)	1
OG0028539	Cellular Component	organelle part(GO:0044422)	1
OG0028539	Cellular Component	organelle(GO:0043226)	1
OG0028543	Cellular Component	cell part(GO:0044464)	1
OG0028543	Cellular Component	cell(GO:0005623)	1
OG0028543	Cellular Component	membrane(GO:0016020)	1
OG0028543	Cellular Component	organelle part(GO:0044422)	1
OG0028543	Cellular Component	organelle(GO:0043226)	1
OG0028545	Cellular Component	cell part(GO:0044464)	1
OG0028545	Cellular Component	cell(GO:0005623)	1
OG0028547	Cellular Component	cell part(GO:0044464)	1
OG0028547	Cellular Component	cell(GO:0005623)	1
OG0028547	Cellular Component	membrane(GO:0016020)	1
OG0028548	Cellular Component	cell part(GO:0044464)	1
OG0028548	Cellular Component	cell(GO:0005623)	1
OG0028548	Cellular Component	extracellular region(GO:0005576)	1
OG0028548	Cellular Component	membrane(GO:0016020)	1
OG0028549	Cellular Component	cell part(GO:0044464)	1

OG0028549	Cellular Component	cell(GO:0005623)	1
OG0028549	Cellular Component	organelle(GO:0043226)	1
OG0028550	Cellular Component	cell part(GO:0044464)	1
OG0028550	Cellular Component	cell(GO:0005623)	1
OG0028550	Cellular Component	membrane(GO:0016020)	1
OG0028550	Cellular Component	organelle part(GO:0044422)	1
OG0028550	Cellular Component	organelle(GO:0043226)	1
OG0028551	Cellular Component	cell part(GO:0044464)	1
OG0028551	Cellular Component	cell(GO:0005623)	1
OG0028551	Cellular Component	organelle(GO:0043226)	1
OG0028556	Cellular Component	cell part(GO:0044464)	1
OG0028556	Cellular Component	cell(GO:0005623)	1
OG0028556	Cellular Component	organelle(GO:0043226)	1
OG0028559	Cellular Component	cell part(GO:0044464)	1
OG0028559	Cellular Component	cell(GO:0005623)	1
OG0028559	Cellular Component	membrane(GO:0016020)	1
OG0028559	Cellular Component	organelle part(GO:0044422)	1
OG0028559	Cellular Component	organelle(GO:0043226)	1
OG0028562	Cellular Component	cell junction(GO:0030054)	1
OG0028562	Cellular Component	cell part(GO:0044464)	1
OG0028562	Cellular Component	cell(GO:0005623)	1
OG0028562	Cellular Component	symplast(GO:0055044)	1
OG0028566	Cellular Component	cell part(GO:0044464)	1
OG0028566	Cellular Component	cell(GO:0005623)	1
OG0028571	Cellular Component	cell part(GO:0044464)	1
OG0028571	Cellular Component	cell(GO:0005623)	1
OG0028571	Cellular Component	membrane(GO:0016020)	1
OG0028572	Cellular Component	cell part(GO:0044464)	1
OG0028572	Cellular Component	cell(GO:0005623)	1
OG0028572	Cellular Component	membrane(GO:0016020)	1
OG0028574	Cellular Component	cell part(GO:0044464)	1
OG0028574	Cellular Component	cell(GO:0005623)	1
OG0028574	Cellular Component	membrane(GO:0016020)	1
OG0028582	Cellular Component	cell part(GO:0044464)	1
OG0028582	Cellular Component	cell(GO:0005623)	1
OG0028582	Cellular Component	organelle(GO:0043226)	1
OG0028583	Cellular Component	cell part(GO:0044464)	1
OG0028583	Cellular Component	cell(GO:0005623)	1
OG0028583	Cellular Component	organelle(GO:0043226)	1
OG0028593	Cellular Component	cell junction(GO:0030054)	1
OG0028593	Cellular Component	cell part(GO:0044464)	1
OG0028593	Cellular Component	cell(GO:0005623)	1
OG0028593	Cellular Component	membrane(GO:0016020)	1
OG0028593	Cellular Component	organelle part(GO:0044422)	1
OG0028593	Cellular Component	organelle(GO:0043226)	1
OG0028593	Cellular Component	symplast(GO:0055044)	1
OG0028597	Cellular Component	cell part(GO:0044464)	1

OG0028597	Cellular Component	cell(GO:0005623)	1
OG0028597	Cellular Component	organelle(GO:0043226)	1
OG0028599	Cellular Component	cell part(GO:0044464)	1
OG0028599	Cellular Component	cell(GO:0005623)	1
OG0028599	Cellular Component	membrane(GO:0016020)	1
OG0028600	Cellular Component	cell part(GO:0044464)	1
OG0028600	Cellular Component	cell(GO:0005623)	1
OG0028600	Cellular Component	organelle(GO:0043226)	1
OG0028601	Cellular Component	cell part(GO:0044464)	1
OG0028601	Cellular Component	cell(GO:0005623)	1
OG0028601	Cellular Component	organelle(GO:0043226)	1
OG0028610	Cellular Component	cell part(GO:0044464)	1
OG0028610	Cellular Component	cell(GO:0005623)	1
OG0028610	Cellular Component	organelle(GO:0043226)	1
OG0028611	Cellular Component	cell part(GO:0044464)	1
OG0028611	Cellular Component	cell(GO:0005623)	1
OG0028611	Cellular Component	extracellular region part(GO:0044421)	1
OG0028611	Cellular Component	extracellular region(GO:0005576)	1
OG0028611	Cellular Component	membrane-enclosed lumen(GO:0031974)	1
OG0028611	Cellular Component	organelle part(GO:0044422)	1
OG0028611	Cellular Component	organelle(GO:0043226)	1
OG0028612	Cellular Component	cell junction(GO:0030054)	1
OG0028612	Cellular Component	cell part(GO:0044464)	1
OG0028612	Cellular Component	cell(GO:0005623)	1
OG0028612	Cellular Component	membrane(GO:0016020)	1
OG0028612	Cellular Component	membrane-enclosed lumen(GO:0031974)	1
OG0028612	Cellular Component	organelle part(GO:0044422)	1
OG0028612	Cellular Component	organelle(GO:0043226)	1
OG0028612	Cellular Component	protein-containing complex(GO:0032991)	1
OG0028612	Cellular Component	symplast(GO:0055044)	1
OG0028614	Cellular Component	cell part(GO:0044464)	1
OG0028614	Cellular Component	cell(GO:0005623)	1
OG0028614	Cellular Component	membrane(GO:0016020)	1
OG0028614	Cellular Component	organelle part(GO:0044422)	1
OG0028614	Cellular Component	organelle(GO:0043226)	1
OG0028624	Cellular Component	cell part(GO:0044464)	1
OG0028624	Cellular Component	cell(GO:0005623)	1
OG0028624	Cellular Component	membrane(GO:0016020)	1
OG0028624	Cellular Component	organelle part(GO:0044422)	1
OG0028624	Cellular Component	organelle(GO:0043226)	1
OG0028630	Cellular Component	cell part(GO:0044464)	1
OG0028630	Cellular Component	cell(GO:0005623)	1
OG0028630	Cellular Component	membrane(GO:0016020)	1
OG0028639	Cellular Component	cell part(GO:0044464)	1
OG0028639	Cellular Component	cell(GO:0005623)	1
OG0028639	Cellular Component	organelle(GO:0043226)	1
OG0028640	Cellular Component	cell part(GO:0044464)	1

OG0028640	Cellular Component	cell(GO:0005623)	1
OG0028640	Cellular Component	organelle(GO:0043226)	1
OG0028645	Cellular Component	cell part(GO:0044464)	1
OG0028645	Cellular Component	cell(GO:0005623)	1
OG0028645	Cellular Component	membrane(GO:0016020)	1
OG0028645	Cellular Component	organelle part(GO:0044422)	1
OG0028645	Cellular Component	organelle(GO:0043226)	1
OG0028646	Cellular Component	cell part(GO:0044464)	1
OG0028646	Cellular Component	cell(GO:0005623)	1
OG0028646	Cellular Component	organelle(GO:0043226)	1
OG0028655	Cellular Component	cell part(GO:0044464)	1
OG0028655	Cellular Component	cell(GO:0005623)	1
OG0028656	Cellular Component	cell part(GO:0044464)	1
OG0028656	Cellular Component	cell(GO:0005623)	1
OG0028656	Cellular Component	extracellular region(GO:0005576)	1
OG0028656	Cellular Component	membrane(GO:0016020)	1
OG0028657	Cellular Component	cell part(GO:0044464)	1
OG0028657	Cellular Component	cell(GO:0005623)	1
OG0028657	Cellular Component	extracellular region(GO:0005576)	1
OG0028657	Cellular Component	membrane part(GO:0044425)	1
OG0028657	Cellular Component	membrane(GO:0016020)	1
OG0028660	Cellular Component	cell part(GO:0044464)	1
OG0028660	Cellular Component	cell(GO:0005623)	1
OG0028661	Cellular Component	cell part(GO:0044464)	1
OG0028661	Cellular Component	cell(GO:0005623)	1
OG0028663	Cellular Component	cell part(GO:0044464)	1
OG0028663	Cellular Component	cell(GO:0005623)	1
OG0028664	Cellular Component	cell part(GO:0044464)	1
OG0028664	Cellular Component	cell(GO:0005623)	1
OG0028667	Cellular Component	cell part(GO:0044464)	1
OG0028667	Cellular Component	cell(GO:0005623)	1
OG0028670	Cellular Component	cell part(GO:0044464)	1
OG0028670	Cellular Component	cell(GO:0005623)	1
OG0028670	Cellular Component	membrane(GO:0016020)	1
OG0028672	Cellular Component	cell part(GO:0044464)	1
OG0028672	Cellular Component	cell(GO:0005623)	1
OG0028672	Cellular Component	organelle part(GO:0044422)	1
OG0028672	Cellular Component	organelle(GO:0043226)	1
OG0028674	Cellular Component	cell part(GO:0044464)	1
OG0028674	Cellular Component	cell(GO:0005623)	1
OG0028674	Cellular Component	membrane(GO:0016020)	1
OG0028674	Cellular Component	organelle part(GO:0044422)	1
OG0028674	Cellular Component	organelle(GO:0043226)	1
OG0028677	Cellular Component	cell part(GO:0044464)	1
OG0028677	Cellular Component	cell(GO:0005623)	1
OG0028677	Cellular Component	organelle(GO:0043226)	1
OG0028687	Cellular Component	cell part(GO:0044464)	1

OG0028687	Cellular Component	cell(GO:0005623)	1
OG0028687	Cellular Component	organelle(GO:0043226)	1
OG0028689	Cellular Component	cell part(GO:0044464)	1
OG0028689	Cellular Component	cell(GO:0005623)	1
OG0028689	Cellular Component	organelle(GO:0043226)	1
OG0028713	Cellular Component	cell part(GO:0044464)	1
OG0028713	Cellular Component	cell(GO:0005623)	1
OG0028713	Cellular Component	organelle(GO:0043226)	1
OG0028714	Cellular Component	cell part(GO:0044464)	1
OG0028714	Cellular Component	cell(GO:0005623)	1
OG0028714	Cellular Component	organelle(GO:0043226)	1
OG0028722	Cellular Component	cell part(GO:0044464)	1
OG0028722	Cellular Component	cell(GO:0005623)	1
OG0028722	Cellular Component	membrane(GO:0016020)	1
OG0028724	Cellular Component	cell part(GO:0044464)	1
OG0028724	Cellular Component	cell(GO:0005623)	1
OG0028724	Cellular Component	membrane(GO:0016020)	1
OG0028724	Cellular Component	organelle part(GO:0044422)	1
OG0028724	Cellular Component	organelle(GO:0043226)	1
OG0028730	Cellular Component	cell part(GO:0044464)	1
OG0028730	Cellular Component	cell(GO:0005623)	1
OG0028730	Cellular Component	organelle(GO:0043226)	1
OG0028731	Cellular Component	cell part(GO:0044464)	1
OG0028731	Cellular Component	cell(GO:0005623)	1
OG0028731	Cellular Component	organelle(GO:0043226)	1
OG0028732	Cellular Component	cell part(GO:0044464)	1
OG0028732	Cellular Component	cell(GO:0005623)	1
OG0028737	Cellular Component	cell part(GO:0044464)	1
OG0028737	Cellular Component	cell(GO:0005623)	1
OG0028737	Cellular Component	organelle(GO:0043226)	1
OG0028745	Cellular Component	cell part(GO:0044464)	1
OG0028745	Cellular Component	cell(GO:0005623)	1
OG0028747	Cellular Component	cell part(GO:0044464)	1
OG0028747	Cellular Component	cell(GO:0005623)	1
OG0028747	Cellular Component	membrane(GO:0016020)	1
OG0028749	Cellular Component	cell part(GO:0044464)	1
OG0028749	Cellular Component	cell(GO:0005623)	1
OG0028749	Cellular Component	membrane(GO:0016020)	1
OG0028754	Cellular Component	cell part(GO:0044464)	1
OG0028754	Cellular Component	cell(GO:0005623)	1
OG0028754	Cellular Component	organelle(GO:0043226)	1
OG0028758	Cellular Component	cell part(GO:0044464)	1
OG0028758	Cellular Component	cell(GO:0005623)	1
OG0028758	Cellular Component	organelle(GO:0043226)	1
OG0028759	Cellular Component	cell part(GO:0044464)	1
OG0028759	Cellular Component	cell(GO:0005623)	1
OG0028759	Cellular Component	membrane part(GO:0044425)	1

OG0028759	Cellular Component	membrane(GO:0016020)	1
OG0028759	Cellular Component	organelle part(GO:0044422)	1
OG0028759	Cellular Component	organelle(GO:0043226)	1
OG0028762	Cellular Component	cell part(GO:0044464)	1
OG0028762	Cellular Component	cell(GO:0005623)	1
OG0028762	Cellular Component	membrane-enclosed lumen(GO:0031974)	1
OG0028762	Cellular Component	organelle part(GO:0044422)	1
OG0028762	Cellular Component	organelle(GO:0043226)	1
OG0028764	Cellular Component	cell part(GO:0044464)	1
OG0028764	Cellular Component	cell(GO:0005623)	1
OG0028764	Cellular Component	membrane part(GO:0044425)	1
OG0028764	Cellular Component	membrane(GO:0016020)	1
OG0028764	Cellular Component	organelle part(GO:0044422)	1
OG0028764	Cellular Component	organelle(GO:0043226)	1
OG0028764	Cellular Component	protein-containing complex(GO:0032991)	1
OG0028766	Cellular Component	cell part(GO:0044464)	1
OG0028766	Cellular Component	cell(GO:0005623)	1
OG0028766	Cellular Component	membrane(GO:0016020)	1
OG0028769	Cellular Component	cell part(GO:0044464)	1
OG0028769	Cellular Component	cell(GO:0005623)	1
OG0028769	Cellular Component	organelle(GO:0043226)	1
OG0028771	Cellular Component	cell part(GO:0044464)	1
OG0028771	Cellular Component	cell(GO:0005623)	1
OG0028799	Cellular Component	membrane(GO:0016020)	1
OG0028800	Cellular Component	cell part(GO:0044464)	1
OG0028800	Cellular Component	cell(GO:0005623)	1
OG0028800	Cellular Component	membrane part(GO:0044425)	1
OG0028800	Cellular Component	membrane(GO:0016020)	1
OG0028800	Cellular Component	organelle part(GO:0044422)	1
OG0028800	Cellular Component	organelle(GO:0043226)	1
OG0028802	Cellular Component	cell part(GO:0044464)	1
OG0028802	Cellular Component	cell(GO:0005623)	1
OG0028802	Cellular Component	organelle(GO:0043226)	1
OG0028807	Cellular Component	cell part(GO:0044464)	1
OG0028807	Cellular Component	cell(GO:0005623)	1
OG0028807	Cellular Component	membrane(GO:0016020)	1
OG0028807	Cellular Component	organelle part(GO:0044422)	1
OG0028807	Cellular Component	organelle(GO:0043226)	1
OG0028810	Cellular Component	cell part(GO:0044464)	1
OG0028810	Cellular Component	cell(GO:0005623)	1
OG0028810	Cellular Component	organelle(GO:0043226)	1
OG0028812	Cellular Component	cell part(GO:0044464)	1
OG0028812	Cellular Component	cell(GO:0005623)	1
OG0028812	Cellular Component	organelle(GO:0043226)	1
OG0028818	Cellular Component	cell part(GO:0044464)	1
OG0028818	Cellular Component	cell(GO:0005623)	1
OG0028818	Cellular Component	organelle(GO:0043226)	1

OG0028819	Cellular Component	cell part(GO:0044464)	1
OG0028819	Cellular Component	cell(GO:0005623)	1
OG0028819	Cellular Component	membrane(GO:0016020)	1
OG0028819	Cellular Component	organelle part(GO:0044422)	1
OG0028819	Cellular Component	organelle(GO:0043226)	1
OG0028821	Cellular Component	cell part(GO:0044464)	1
OG0028821	Cellular Component	cell(GO:0005623)	1
OG0028821	Cellular Component	organelle part(GO:0044422)	1
OG0028821	Cellular Component	organelle(GO:0043226)	1
OG0028822	Cellular Component	cell part(GO:0044464)	1
OG0028822	Cellular Component	cell(GO:0005623)	1
OG0028822	Cellular Component	membrane-enclosed lumen(GO:0031974)	1
OG0028822	Cellular Component	organelle part(GO:0044422)	1
OG0028822	Cellular Component	organelle(GO:0043226)	1
OG0028822	Cellular Component	protein-containing complex(GO:0032991)	1
OG0028823	Cellular Component	cell part(GO:0044464)	1
OG0028823	Cellular Component	cell(GO:0005623)	1
OG0028823	Cellular Component	membrane(GO:0016020)	1
OG0028823	Cellular Component	organelle part(GO:0044422)	1
OG0028823	Cellular Component	organelle(GO:0043226)	1
OG0028824	Cellular Component	cell part(GO:0044464)	1
OG0028824	Cellular Component	cell(GO:0005623)	1
OG0028824	Cellular Component	organelle(GO:0043226)	1
OG0028838	Cellular Component	cell part(GO:0044464)	1
OG0028838	Cellular Component	cell(GO:0005623)	1
OG0028838	Cellular Component	membrane(GO:0016020)	1
OG0028840	Cellular Component	cell part(GO:0044464)	1
OG0028840	Cellular Component	cell(GO:0005623)	1
OG0028840	Cellular Component	organelle(GO:0043226)	1
OG0028843	Cellular Component	cell part(GO:0044464)	1
OG0028843	Cellular Component	cell(GO:0005623)	1
OG0028843	Cellular Component	membrane part(GO:0044425)	1
OG0028843	Cellular Component	membrane(GO:0016020)	1
OG0028843	Cellular Component	organelle(GO:0043226)	1
OG0028849	Cellular Component	cell part(GO:0044464)	1
OG0028849	Cellular Component	cell(GO:0005623)	1
OG0028849	Cellular Component	organelle(GO:0043226)	1
OG0028850	Cellular Component	cell part(GO:0044464)	1
OG0028850	Cellular Component	cell(GO:0005623)	1
OG0028850	Cellular Component	organelle(GO:0043226)	1
OG0028853	Cellular Component	cell part(GO:0044464)	1
OG0028853	Cellular Component	cell(GO:0005623)	1
OG0028863	Cellular Component	cell junction(GO:0030054)	1
OG0028863	Cellular Component	cell part(GO:0044464)	1
OG0028863	Cellular Component	cell(GO:0005623)	1
OG0028863	Cellular Component	membrane(GO:0016020)	1
OG0028863	Cellular Component	organelle part(GO:0044422)	1

OG0028863	Cellular Component	organelle(GO:0043226)	1
OG0028863	Cellular Component	protein-containing complex(GO:0032991)	1
OG0028863	Cellular Component	symplast(GO:0055044)	1
OG0028864	Cellular Component	membrane(GO:0016020)	1
OG0028865	Cellular Component	membrane(GO:0016020)	1
OG0028866	Cellular Component	extracellular region(GO:0005576)	1
OG0028867	Cellular Component	cell part(GO:0044464)	1
OG0028867	Cellular Component	cell(GO:0005623)	1
OG0028867	Cellular Component	membrane part(GO:0044425)	1
OG0028867	Cellular Component	membrane(GO:0016020)	1
OG0028867	Cellular Component	membrane-enclosed lumen(GO:0031974)	1
OG0028867	Cellular Component	organelle part(GO:0044422)	1
OG0028867	Cellular Component	organelle(GO:0043226)	1
OG0028867	Cellular Component	protein-containing complex(GO:0032991)	1
OG0028869	Cellular Component	cell part(GO:0044464)	1
OG0028869	Cellular Component	cell(GO:0005623)	1
OG0028869	Cellular Component	membrane(GO:0016020)	1
OG0028869	Cellular Component	organelle(GO:0043226)	1
OG0028870	Cellular Component	cell part(GO:0044464)	1
OG0028870	Cellular Component	cell(GO:0005623)	1
OG0028870	Cellular Component	membrane(GO:0016020)	1
OG0028870	Cellular Component	organelle(GO:0043226)	1
OG0028872	Cellular Component	cell part(GO:0044464)	1
OG0028872	Cellular Component	cell(GO:0005623)	1
OG0028872	Cellular Component	membrane(GO:0016020)	1
OG0028872	Cellular Component	organelle(GO:0043226)	1
OG0028874	Cellular Component	cell part(GO:0044464)	1
OG0028874	Cellular Component	cell(GO:0005623)	1
OG0028874	Cellular Component	organelle(GO:0043226)	1
OG0028879	Cellular Component	cell part(GO:0044464)	1
OG0028879	Cellular Component	cell(GO:0005623)	1
OG0028879	Cellular Component	membrane part(GO:0044425)	1
OG0028879	Cellular Component	membrane(GO:0016020)	1
OG0028879	Cellular Component	organelle part(GO:0044422)	1
OG0028879	Cellular Component	organelle(GO:0043226)	1
OG0028881	Cellular Component	cell part(GO:0044464)	1
OG0028881	Cellular Component	cell(GO:0005623)	1
OG0028881	Cellular Component	extracellular region(GO:0005576)	1
OG0028881	Cellular Component	organelle(GO:0043226)	1
OG0028900	Cellular Component	cell part(GO:0044464)	1
OG0028900	Cellular Component	cell(GO:0005623)	1
OG0028903	Cellular Component	cell part(GO:0044464)	1
OG0028903	Cellular Component	cell(GO:0005623)	1
OG0028903	Cellular Component	membrane(GO:0016020)	1
OG0028903	Cellular Component	organelle(GO:0043226)	1
OG0028903	Cellular Component	protein-containing complex(GO:0032991)	1

OG0028904	Cellular Component	cell part(GO:0044464)	1
OG0028904	Cellular Component	cell(GO:0005623)	1
OG0028904	Cellular Component	membrane(GO:0016020)	1
OG0028906	Cellular Component	cell part(GO:0044464)	1
OG0028906	Cellular Component	cell(GO:0005623)	1
OG0028908	Cellular Component	cell part(GO:0044464)	1
OG0028908	Cellular Component	cell(GO:0005623)	1
OG0028913	Cellular Component	cell part(GO:0044464)	1
OG0028913	Cellular Component	cell(GO:0005623)	1
OG0028913	Cellular Component	protein-containing complex(GO:0032991)	1
OG0028919	Cellular Component	cell part(GO:0044464)	1
OG0028919	Cellular Component	cell(GO:0005623)	1
OG0028919	Cellular Component	membrane(GO:0016020)	1
OG0028919	Cellular Component	organelle part(GO:0044422)	1
OG0028919	Cellular Component	organelle(GO:0043226)	1
OG0028925	Cellular Component	cell part(GO:0044464)	1
OG0028925	Cellular Component	cell(GO:0005623)	1
OG0028925	Cellular Component	membrane(GO:0016020)	1
OG0028925	Cellular Component	organelle part(GO:0044422)	1
OG0028925	Cellular Component	organelle(GO:0043226)	1
OG0028928	Cellular Component	membrane(GO:0016020)	1
OG0028930	Cellular Component	cell part(GO:0044464)	1
OG0028930	Cellular Component	cell(GO:0005623)	1
OG0028930	Cellular Component	organelle(GO:0043226)	1
OG0028934	Cellular Component	cell part(GO:0044464)	1
OG0028934	Cellular Component	cell(GO:0005623)	1
OG0028934	Cellular Component	organelle(GO:0043226)	1
OG0028949	Cellular Component	cell part(GO:0044464)	1
OG0028949	Cellular Component	cell(GO:0005623)	1
OG0028949	Cellular Component	membrane part(GO:0044425)	1
OG0028949	Cellular Component	membrane(GO:0016020)	1
OG0028954	Cellular Component	cell part(GO:0044464)	1
OG0028954	Cellular Component	cell(GO:0005623)	1
OG0028954	Cellular Component	membrane(GO:0016020)	1
OG0028959	Cellular Component	cell part(GO:0044464)	1
OG0028959	Cellular Component	cell(GO:0005623)	1
OG0028959	Cellular Component	membrane(GO:0016020)	1
OG0028959	Cellular Component	organelle(GO:0043226)	1
OG0028963	Cellular Component	cell part(GO:0044464)	1
OG0028963	Cellular Component	cell(GO:0005623)	1
OG0028963	Cellular Component	membrane(GO:0016020)	1
OG0028963	Cellular Component	organelle(GO:0043226)	1
OG0028964	Cellular Component	cell part(GO:0044464)	1
OG0028964	Cellular Component	cell(GO:0005623)	1
OG0028964	Cellular Component	membrane(GO:0016020)	1
OG0028964	Cellular Component	organelle(GO:0043226)	1
OG0028965	Cellular Component	cell part(GO:0044464)	1

OG0028965	Cellular Component	cell(GO:0005623)	1
OG0028965	Cellular Component	membrane(GO:0016020)	1
OG0028965	Cellular Component	organelle(GO:0043226)	1
OG0028969	Cellular Component	cell part(GO:0044464)	1
OG0028969	Cellular Component	cell(GO:0005623)	1
OG0028969	Cellular Component	membrane(GO:0016020)	1
OG0028969	Cellular Component	organelle(GO:0043226)	1
OG0028970	Cellular Component	cell part(GO:0044464)	1
OG0028970	Cellular Component	cell(GO:0005623)	1
OG0028970	Cellular Component	membrane(GO:0016020)	1
OG0028970	Cellular Component	organelle(GO:0043226)	1
OG0028974	Cellular Component	membrane(GO:0016020)	1
OG0028991	Cellular Component	cell part(GO:0044464)	1
OG0028991	Cellular Component	cell(GO:0005623)	1
OG0028991	Cellular Component	membrane(GO:0016020)	1
OG0028991	Cellular Component	organelle(GO:0043226)	1
OG0028992	Cellular Component	cell part(GO:0044464)	1
OG0028992	Cellular Component	cell(GO:0005623)	1
OG0028992	Cellular Component	membrane(GO:0016020)	1
OG0028992	Cellular Component	organelle part(GO:0044422)	1
OG0028992	Cellular Component	organelle(GO:0043226)	1
OG0029004	Cellular Component	cell part(GO:0044464)	1
OG0029004	Cellular Component	cell(GO:0005623)	1
OG0029004	Cellular Component	membrane(GO:0016020)	1
OG0029007	Cellular Component	cell part(GO:0044464)	1
OG0029007	Cellular Component	cell(GO:0005623)	1
OG0029007	Cellular Component	organelle(GO:0043226)	1
OG0029011	Cellular Component	membrane(GO:0016020)	1
OG0029013	Cellular Component	cell part(GO:0044464)	1
OG0029013	Cellular Component	cell(GO:0005623)	1
OG0029013	Cellular Component	membrane(GO:0016020)	1
OG0029013	Cellular Component	organelle part(GO:0044422)	1
OG0029013	Cellular Component	organelle(GO:0043226)	1
OG0029015	Cellular Component	cell part(GO:0044464)	1
OG0029015	Cellular Component	cell(GO:0005623)	1
OG0029015	Cellular Component	organelle part(GO:0044422)	1
OG0029015	Cellular Component	organelle(GO:0043226)	1
OG0029015	Cellular Component	protein-containing complex(GO:0032991)	1
OG0029016	Cellular Component	cell part(GO:0044464)	1
OG0029016	Cellular Component	cell(GO:0005623)	1
OG0029016	Cellular Component	organelle part(GO:0044422)	1
OG0029016	Cellular Component	organelle(GO:0043226)	1
OG0029018	Cellular Component	cell part(GO:0044464)	1
OG0029018	Cellular Component	cell(GO:0005623)	1
OG0029018	Cellular Component	organelle(GO:0043226)	1
OG0029021	Cellular Component	cell part(GO:0044464)	1
OG0029021	Cellular Component	cell(GO:0005623)	1

OG0029021	Cellular Component	organelle(GO:0043226)	1
OG0029022	Cellular Component	cell part(GO:0044464)	1
OG0029022	Cellular Component	cell(GO:0005623)	1
OG0029022	Cellular Component	organelle(GO:0043226)	1
OG0029026	Cellular Component	cell part(GO:0044464)	1
OG0029026	Cellular Component	cell(GO:0005623)	1
OG0029026	Cellular Component	membrane(GO:0016020)	1
OG0029027	Cellular Component	cell part(GO:0044464)	1
OG0029027	Cellular Component	cell(GO:0005623)	1
OG0029027	Cellular Component	organelle(GO:0043226)	1
OG0029029	Cellular Component	cell part(GO:0044464)	1
OG0029029	Cellular Component	cell(GO:0005623)	1
OG0029029	Cellular Component	membrane(GO:0016020)	1
OG0029035	Cellular Component	cell part(GO:0044464)	1
OG0029035	Cellular Component	cell(GO:0005623)	1
OG0029035	Cellular Component	membrane(GO:0016020)	1
OG0029035	Cellular Component	organelle(GO:0043226)	1
OG0029037	Cellular Component	cell junction(GO:0030054)	1
OG0029037	Cellular Component	cell part(GO:0044464)	1
OG0029037	Cellular Component	cell(GO:0005623)	1
OG0029037	Cellular Component	membrane(GO:0016020)	1
OG0029037	Cellular Component	organelle(GO:0043226)	1
OG0029037	Cellular Component	symplast(GO:0055044)	1
OG0029047	Cellular Component	cell part(GO:0044464)	1
OG0029047	Cellular Component	cell(GO:0005623)	1
OG0029047	Cellular Component	membrane(GO:0016020)	1
OG0029047	Cellular Component	organelle part(GO:0044422)	1
OG0029047	Cellular Component	organelle(GO:0043226)	1
OG0029058	Cellular Component	cell part(GO:0044464)	1
OG0029058	Cellular Component	cell(GO:0005623)	1
OG0029058	Cellular Component	membrane(GO:0016020)	1
OG0029058	Cellular Component	organelle part(GO:0044422)	1
OG0029058	Cellular Component	organelle(GO:0043226)	1
OG0029059	Cellular Component	cell part(GO:0044464)	1
OG0029059	Cellular Component	cell(GO:0005623)	1
OG0029059	Cellular Component	organelle(GO:0043226)	1
OG0029060	Cellular Component	cell part(GO:0044464)	1
OG0029060	Cellular Component	cell(GO:0005623)	1
OG0029060	Cellular Component	organelle(GO:0043226)	1
OG0029072	Cellular Component	cell part(GO:0044464)	1
OG0029072	Cellular Component	cell(GO:0005623)	1
OG0029072	Cellular Component	extracellular region part(GO:0044421)	1
OG0029072	Cellular Component	extracellular region(GO:0005576)	1
OG0029072	Cellular Component	membrane-enclosed lumen(GO:0031974)	1
OG0029072	Cellular Component	organelle part(GO:0044422)	1
OG0029072	Cellular Component	organelle(GO:0043226)	1
OG0029072	Cellular Component	protein-containing complex(GO:0032991)	1

OG0029072	Cellular Component	supramolecular complex(GO:0099080)	1
OG0029113	Cellular Component	cell part(GO:0044464)	1
OG0029113	Cellular Component	cell(GO:0005623)	1
OG0029113	Cellular Component	membrane(GO:0016020)	1
OG0029113	Cellular Component	organelle(GO:0043226)	1
OG0029115	Cellular Component	cell part(GO:0044464)	1
OG0029115	Cellular Component	cell(GO:0005623)	1
OG0029115	Cellular Component	membrane part(GO:0044425)	1
OG0029115	Cellular Component	membrane(GO:0016020)	1
OG0029115	Cellular Component	organelle part(GO:0044422)	1
OG0029115	Cellular Component	organelle(GO:0043226)	1
OG0029117	Cellular Component	cell part(GO:0044464)	1
OG0029117	Cellular Component	cell(GO:0005623)	1
OG0029117	Cellular Component	membrane(GO:0016020)	1
OG0029117	Cellular Component	organelle(GO:0043226)	1
OG0029118	Cellular Component	cell part(GO:0044464)	1
OG0029118	Cellular Component	cell(GO:0005623)	1
OG0029118	Cellular Component	organelle(GO:0043226)	1
OG0029120	Cellular Component	cell part(GO:0044464)	1
OG0029120	Cellular Component	cell(GO:0005623)	1
OG0029120	Cellular Component	organelle(GO:0043226)	1
OG0029122	Cellular Component	cell part(GO:0044464)	1
OG0029122	Cellular Component	cell(GO:0005623)	1
OG0029124	Cellular Component	cell junction(GO:0030054)	1
OG0029124	Cellular Component	cell part(GO:0044464)	1
OG0029124	Cellular Component	cell(GO:0005623)	1
OG0029124	Cellular Component	membrane(GO:0016020)	1
OG0029124	Cellular Component	organelle(GO:0043226)	1
OG0029124	Cellular Component	symplast(GO:0055044)	1
OG0029125	Cellular Component	cell part(GO:0044464)	1
OG0029125	Cellular Component	cell(GO:0005623)	1
OG0029125	Cellular Component	organelle(GO:0043226)	1
OG0029131	Cellular Component	cell junction(GO:0030054)	1
OG0029131	Cellular Component	cell part(GO:0044464)	1
OG0029131	Cellular Component	cell(GO:0005623)	1
OG0029131	Cellular Component	symplast(GO:0055044)	1
OG0029134	Cellular Component	cell part(GO:0044464)	1
OG0029134	Cellular Component	cell(GO:0005623)	1
OG0029134	Cellular Component	organelle(GO:0043226)	1
OG0029141	Cellular Component	cell part(GO:0044464)	1
OG0029141	Cellular Component	cell(GO:0005623)	1
OG0029141	Cellular Component	membrane part(GO:0044425)	1
OG0029141	Cellular Component	membrane(GO:0016020)	1
OG0029141	Cellular Component	organelle part(GO:0044422)	1
OG0029141	Cellular Component	organelle(GO:0043226)	1
OG0029142	Cellular Component	cell part(GO:0044464)	1
OG0029142	Cellular Component	cell(GO:0005623)	1

OG0029142	Cellular Component	membrane-enclosed lumen(GO:0031974)	1
OG0029142	Cellular Component	organelle part(GO:0044422)	1
OG0029142	Cellular Component	organelle(GO:0043226)	1
OG0029143	Cellular Component	cell part(GO:0044464)	1
OG0029143	Cellular Component	cell(GO:0005623)	1
OG0029143	Cellular Component	organelle(GO:0043226)	1
OG0029145	Cellular Component	cell part(GO:0044464)	1
OG0029145	Cellular Component	cell(GO:0005623)	1
OG0029145	Cellular Component	organelle(GO:0043226)	1
OG0029149	Cellular Component	cell part(GO:0044464)	1
OG0029149	Cellular Component	cell(GO:0005623)	1
OG0029149	Cellular Component	membrane-enclosed lumen(GO:0031974)	1
OG0029149	Cellular Component	organelle part(GO:0044422)	1
OG0029149	Cellular Component	organelle(GO:0043226)	1
OG0029159	Cellular Component	cell part(GO:0044464)	1
OG0029159	Cellular Component	cell(GO:0005623)	1
OG0029159	Cellular Component	membrane(GO:0016020)	1
OG0029160	Cellular Component	cell part(GO:0044464)	1
OG0029160	Cellular Component	cell(GO:0005623)	1
OG0029160	Cellular Component	membrane(GO:0016020)	1
OG0029161	Cellular Component	cell part(GO:0044464)	1
OG0029161	Cellular Component	cell(GO:0005623)	1
OG0029161	Cellular Component	organelle part(GO:0044422)	1
OG0029161	Cellular Component	organelle(GO:0043226)	1
OG0029163	Cellular Component	cell part(GO:0044464)	1
OG0029163	Cellular Component	cell(GO:0005623)	1
OG0029163	Cellular Component	membrane(GO:0016020)	1
OG0029163	Cellular Component	organelle part(GO:0044422)	1
OG0029163	Cellular Component	organelle(GO:0043226)	1
OG0029163	Cellular Component	synapse part(GO:0044456)	1
OG0029163	Cellular Component	synapse(GO:0045202)	1
OG0029165	Cellular Component	cell part(GO:0044464)	1
OG0029165	Cellular Component	cell(GO:0005623)	1
OG0029168	Cellular Component	cell part(GO:0044464)	1
OG0029168	Cellular Component	cell(GO:0005623)	1
OG0029168	Cellular Component	membrane-enclosed lumen(GO:0031974)	1
OG0029168	Cellular Component	organelle part(GO:0044422)	1
OG0029168	Cellular Component	organelle(GO:0043226)	1
OG0029172	Cellular Component	cell part(GO:0044464)	1
OG0029172	Cellular Component	cell(GO:0005623)	1
OG0029172	Cellular Component	membrane(GO:0016020)	1
OG0029173	Cellular Component	membrane(GO:0016020)	1
OG0029193	Cellular Component	membrane(GO:0016020)	1
OG0029194	Cellular Component	cell part(GO:0044464)	1
OG0029194	Cellular Component	cell(GO:0005623)	1
OG0029194	Cellular Component	organelle(GO:0043226)	1
OG0029198	Cellular Component	cell part(GO:0044464)	1

OG0029198	Cellular Component	cell(GO:0005623)	1
OG0029198	Cellular Component	membrane(GO:0016020)	1
OG0029198	Cellular Component	organelle(GO:0043226)	1
OG0029200	Cellular Component	cell part(GO:0044464)	1
OG0029200	Cellular Component	cell(GO:0005623)	1
OG0029200	Cellular Component	membrane(GO:0016020)	1
OG0029201	Cellular Component	cell part(GO:0044464)	1
OG0029201	Cellular Component	cell(GO:0005623)	1
OG0029201	Cellular Component	membrane(GO:0016020)	1
OG0029201	Cellular Component	organelle part(GO:0044422)	1
OG0029201	Cellular Component	organelle(GO:0043226)	1
OG0029203	Cellular Component	cell junction(GO:0030054)	1
OG0029203	Cellular Component	cell part(GO:0044464)	1
OG0029203	Cellular Component	cell(GO:0005623)	1
OG0029203	Cellular Component	membrane(GO:0016020)	1
OG0029203	Cellular Component	organelle(GO:0043226)	1
OG0029203	Cellular Component	symplast(GO:0055044)	1
OG0029204	Cellular Component	cell junction(GO:0030054)	1
OG0029204	Cellular Component	cell part(GO:0044464)	1
OG0029204	Cellular Component	cell(GO:0005623)	1
OG0029204	Cellular Component	membrane(GO:0016020)	1
OG0029204	Cellular Component	organelle part(GO:0044422)	1
OG0029204	Cellular Component	organelle(GO:0043226)	1
OG0029204	Cellular Component	symplast(GO:0055044)	1
OG0029213	Cellular Component	cell part(GO:0044464)	1
OG0029213	Cellular Component	cell(GO:0005623)	1
OG0029213	Cellular Component	membrane(GO:0016020)	1
OG0029213	Cellular Component	organelle part(GO:0044422)	1
OG0029213	Cellular Component	organelle(GO:0043226)	1
OG0029223	Cellular Component	cell part(GO:0044464)	1
OG0029223	Cellular Component	cell(GO:0005623)	1
OG0029223	Cellular Component	membrane(GO:0016020)	1
OG0029223	Cellular Component	organelle part(GO:0044422)	1
OG0029223	Cellular Component	organelle(GO:0043226)	1
OG0029223	Cellular Component	protein-containing complex(GO:0032991)	1
OG0029225	Cellular Component	cell part(GO:0044464)	1
OG0029225	Cellular Component	cell(GO:0005623)	1
OG0029225	Cellular Component	membrane(GO:0016020)	1
OG0029225	Cellular Component	organelle part(GO:0044422)	1
OG0029225	Cellular Component	organelle(GO:0043226)	1
OG0029225	Cellular Component	protein-containing complex(GO:0032991)	1
OG0029226	Cellular Component	cell part(GO:0044464)	1
OG0029226	Cellular Component	cell(GO:0005623)	1
OG0029226	Cellular Component	membrane(GO:0016020)	1
OG0029226	Cellular Component	organelle part(GO:0044422)	1
OG0029226	Cellular Component	organelle(GO:0043226)	1

OG0029226	Cellular Component	protein-containing complex(GO:0032991)	1
OG0029227	Cellular Component	cell part(GO:0044464)	1
OG0029227	Cellular Component	cell(GO:0005623)	1
OG0029227	Cellular Component	membrane part(GO:0044425)	1
OG0029227	Cellular Component	membrane(GO:0016020)	1
OG0029227	Cellular Component	organelle part(GO:0044422)	1
OG0029227	Cellular Component	organelle(GO:0043226)	1
OG0029227	Cellular Component	protein-containing complex(GO:0032991)	1
OG0029230	Cellular Component	cell part(GO:0044464)	1
OG0029230	Cellular Component	cell(GO:0005623)	1
OG0029230	Cellular Component	membrane(GO:0016020)	1
OG0029233	Cellular Component	membrane(GO:0016020)	1
OG0029241	Cellular Component	cell part(GO:0044464)	1
OG0029241	Cellular Component	cell(GO:0005623)	1
OG0029241	Cellular Component	membrane(GO:0016020)	1
OG0029241	Cellular Component	organelle part(GO:0044422)	1
OG0029241	Cellular Component	organelle(GO:0043226)	1
OG0029241	Cellular Component	synapse part(GO:0044456)	1
OG0029241	Cellular Component	synapse(GO:0045202)	1
OG0029243	Cellular Component	cell part(GO:0044464)	1
OG0029243	Cellular Component	cell(GO:0005623)	1
OG0029243	Cellular Component	membrane(GO:0016020)	1
OG0029243	Cellular Component	organelle part(GO:0044422)	1
OG0029243	Cellular Component	organelle(GO:0043226)	1
OG0029254	Cellular Component	cell junction(GO:0030054)	1
OG0029254	Cellular Component	cell part(GO:0044464)	1
OG0029254	Cellular Component	cell(GO:0005623)	1
OG0029254	Cellular Component	membrane part(GO:0044425)	1
OG0029254	Cellular Component	membrane(GO:0016020)	1
OG0029254	Cellular Component	organelle(GO:0043226)	1
OG0029254	Cellular Component	symplast(GO:0055044)	1
OG0029256	Cellular Component	cell part(GO:0044464)	1
OG0029256	Cellular Component	cell(GO:0005623)	1
OG0029256	Cellular Component	organelle(GO:0043226)	1
OG0029257	Cellular Component	membrane(GO:0016020)	1
OG0029261	Cellular Component	cell part(GO:0044464)	1
OG0029261	Cellular Component	cell(GO:0005623)	1
OG0029261	Cellular Component	membrane(GO:0016020)	1
OG0029266	Cellular Component	cell part(GO:0044464)	1
OG0029266	Cellular Component	cell(GO:0005623)	1
OG0029266	Cellular Component	extracellular region(GO:0005576)	1
OG0029266	Cellular Component	membrane(GO:0016020)	1
OG0029268	Cellular Component	cell part(GO:0044464)	1
OG0029268	Cellular Component	cell(GO:0005623)	1
OG0029268	Cellular Component	extracellular region(GO:0005576)	1
OG0029268	Cellular Component	membrane(GO:0016020)	1
OG0029277	Cellular Component	cell part(GO:0044464)	1

OG0029277	Cellular Component	cell(GO:0005623)	1
OG0029277	Cellular Component	organelle(GO:0043226)	1
OG0029288	Cellular Component	cell part(GO:0044464)	1
OG0029288	Cellular Component	cell(GO:0005623)	1
OG0029292	Cellular Component	cell part(GO:0044464)	1
OG0029292	Cellular Component	cell(GO:0005623)	1
OG0029292	Cellular Component	membrane(GO:0016020)	1
OG0029295	Cellular Component	cell part(GO:0044464)	1
OG0029295	Cellular Component	cell(GO:0005623)	1
OG0029295	Cellular Component	membrane(GO:0016020)	1
OG0029300	Cellular Component	cell part(GO:0044464)	1
OG0029300	Cellular Component	cell(GO:0005623)	1
OG0029300	Cellular Component	membrane(GO:0016020)	1
OG0029303	Cellular Component	cell part(GO:0044464)	1
OG0029303	Cellular Component	cell(GO:0005623)	1
OG0029305	Cellular Component	cell part(GO:0044464)	1
OG0029305	Cellular Component	cell(GO:0005623)	1
OG0029305	Cellular Component	organelle(GO:0043226)	1
OG0029308	Cellular Component	cell part(GO:0044464)	1
OG0029308	Cellular Component	cell(GO:0005623)	1
OG0029308	Cellular Component	membrane(GO:0016020)	1
OG0029316	Cellular Component	cell part(GO:0044464)	1
OG0029316	Cellular Component	cell(GO:0005623)	1
OG0029316	Cellular Component	organelle(GO:0043226)	1
OG0029319	Cellular Component	cell part(GO:0044464)	1
OG0029319	Cellular Component	cell(GO:0005623)	1
OG0029319	Cellular Component	membrane(GO:0016020)	1
OG0029319	Cellular Component	organelle(GO:0043226)	1
OG0029320	Cellular Component	cell part(GO:0044464)	1
OG0029320	Cellular Component	cell(GO:0005623)	1
OG0029320	Cellular Component	extracellular region(GO:0005576)	1
OG0029320	Cellular Component	organelle(GO:0043226)	1
OG0029321	Cellular Component	membrane(GO:0016020)	1
OG0029323	Cellular Component	cell junction(GO:0030054)	1
OG0029323	Cellular Component	cell part(GO:0044464)	1
OG0029323	Cellular Component	cell(GO:0005623)	1
OG0029323	Cellular Component	membrane part(GO:0044425)	1
OG0029323	Cellular Component	membrane(GO:0016020)	1
OG0029323	Cellular Component	organelle part(GO:0044422)	1
OG0029323	Cellular Component	organelle(GO:0043226)	1
OG0029323	Cellular Component	protein-containing complex(GO:0032991)	1
OG0029323	Cellular Component	symplast(GO:0055044)	1
OG0029324	Cellular Component	cell junction(GO:0030054)	1
OG0029324	Cellular Component	symplast(GO:0055044)	1
OG0029327	Cellular Component	cell junction(GO:0030054)	1
OG0029327	Cellular Component	cell part(GO:0044464)	1
OG0029327	Cellular Component	cell(GO:0005623)	1

OG0029327	Cellular Component	extracellular region(GO:0005576)	1
OG0029327	Cellular Component	organelle(GO:0043226)	1
OG0029327	Cellular Component	symplast(GO:0055044)	1
OG0029331	Cellular Component	cell part(GO:0044464)	1
OG0029331	Cellular Component	cell(GO:0005623)	1
OG0029331	Cellular Component	organelle(GO:0043226)	1
OG0029337	Cellular Component	cell part(GO:0044464)	1
OG0029337	Cellular Component	cell(GO:0005623)	1
OG0029338	Cellular Component	cell part(GO:0044464)	1
OG0029338	Cellular Component	cell(GO:0005623)	1
OG0029338	Cellular Component	membrane(GO:0016020)	1
OG0029341	Cellular Component	cell part(GO:0044464)	1
OG0029341	Cellular Component	cell(GO:0005623)	1
OG0029341	Cellular Component	membrane(GO:0016020)	1
OG0029341	Cellular Component	nucleoid(GO:0009295)	1
OG0029342	Cellular Component	cell part(GO:0044464)	1
OG0029342	Cellular Component	cell(GO:0005623)	1
OG0029343	Cellular Component	cell part(GO:0044464)	1
OG0029343	Cellular Component	cell(GO:0005623)	1
OG0029343	Cellular Component	membrane(GO:0016020)	1
OG0029346	Cellular Component	cell part(GO:0044464)	1
OG0029346	Cellular Component	cell(GO:0005623)	1
OG0029348	Cellular Component	cell part(GO:0044464)	1
OG0029348	Cellular Component	cell(GO:0005623)	1
OG0029348	Cellular Component	organelle(GO:0043226)	1
OG0029355	Cellular Component	cell part(GO:0044464)	1
OG0029355	Cellular Component	cell(GO:0005623)	1
OG0029355	Cellular Component	membrane(GO:0016020)	1
OG0029356	Cellular Component	cell part(GO:0044464)	1
OG0029356	Cellular Component	cell(GO:0005623)	1
OG0029359	Cellular Component	cell part(GO:0044464)	1
OG0029359	Cellular Component	cell(GO:0005623)	1
OG0029362	Cellular Component	cell part(GO:0044464)	1
OG0029362	Cellular Component	cell(GO:0005623)	1
OG0029362	Cellular Component	membrane(GO:0016020)	1
OG0029363	Cellular Component	membrane(GO:0016020)	1
OG0029366	Cellular Component	cell part(GO:0044464)	1
OG0029366	Cellular Component	cell(GO:0005623)	1
OG0029366	Cellular Component	membrane(GO:0016020)	1
OG0029368	Cellular Component	cell part(GO:0044464)	1
OG0029368	Cellular Component	cell(GO:0005623)	1
OG0029375	Cellular Component	cell part(GO:0044464)	1
OG0029375	Cellular Component	cell(GO:0005623)	1
OG0029375	Cellular Component	organelle(GO:0043226)	1
OG0029376	Cellular Component	cell part(GO:0044464)	1
OG0029376	Cellular Component	cell(GO:0005623)	1
OG0029376	Cellular Component	organelle(GO:0043226)	1

OG0029383	Cellular Component	cell junction(GO:0030054)	1
OG0029383	Cellular Component	cell part(GO:0044464)	1
OG0029383	Cellular Component	cell(GO:0005623)	1
OG0029383	Cellular Component	membrane part(GO:0044425)	1
OG0029383	Cellular Component	membrane(GO:0016020)	1
OG0029383	Cellular Component	symplast(GO:0055044)	1
OG0029384	Cellular Component	cell part(GO:0044464)	1
OG0029384	Cellular Component	cell(GO:0005623)	1
OG0029384	Cellular Component	organelle part(GO:0044422)	1
OG0029384	Cellular Component	organelle(GO:0043226)	1
OG0029387	Cellular Component	cell part(GO:0044464)	1
OG0029387	Cellular Component	cell(GO:0005623)	1
OG0029387	Cellular Component	organelle(GO:0043226)	1
OG0029388	Cellular Component	cell part(GO:0044464)	1
OG0029388	Cellular Component	cell(GO:0005623)	1
OG0029388	Cellular Component	organelle(GO:0043226)	1
OG0029389	Cellular Component	cell part(GO:0044464)	1
OG0029389	Cellular Component	cell(GO:0005623)	1
OG0029391	Cellular Component	cell part(GO:0044464)	1
OG0029391	Cellular Component	cell(GO:0005623)	1
OG0029391	Cellular Component	organelle(GO:0043226)	1
OG0029392	Cellular Component	cell part(GO:0044464)	1
OG0029392	Cellular Component	cell(GO:0005623)	1
OG0029392	Cellular Component	membrane(GO:0016020)	1
OG0029408	Cellular Component	cell part(GO:0044464)	1
OG0029408	Cellular Component	cell(GO:0005623)	1
OG0029408	Cellular Component	organelle(GO:0043226)	1
OG0029412	Cellular Component	cell junction(GO:0030054)	1
OG0029412	Cellular Component	cell part(GO:0044464)	1
OG0029412	Cellular Component	cell(GO:0005623)	1
OG0029412	Cellular Component	membrane(GO:0016020)	1
OG0029412	Cellular Component	membrane-enclosed lumen(GO:0031974)	1
OG0029412	Cellular Component	organelle part(GO:0044422)	1
OG0029412	Cellular Component	organelle(GO:0043226)	1
OG0029412	Cellular Component	protein-containing complex(GO:0032991)	1
OG0029412	Cellular Component	symplast(GO:0055044)	1
OG0029414	Cellular Component	cell junction(GO:0030054)	1
OG0029414	Cellular Component	cell part(GO:0044464)	1
OG0029414	Cellular Component	cell(GO:0005623)	1
OG0029414	Cellular Component	symplast(GO:0055044)	1
OG0029416	Cellular Component	cell part(GO:0044464)	1
OG0029416	Cellular Component	cell(GO:0005623)	1
OG0029420	Cellular Component	cell part(GO:0044464)	1
OG0029420	Cellular Component	cell(GO:0005623)	1
OG0029420	Cellular Component	membrane(GO:0016020)	1
OG0029420	Cellular Component	organelle(GO:0043226)	1
OG0029424	Cellular Component	cell part(GO:0044464)	1

OG0029424	Cellular Component	cell(GO:0005623)	1
OG0029424	Cellular Component	organelle part(GO:0044422)	1
OG0029424	Cellular Component	organelle(GO:0043226)	1
OG0029424	Cellular Component	protein-containing complex(GO:0032991)	1
OG0029426	Cellular Component	cell part(GO:0044464)	1
OG0029426	Cellular Component	cell(GO:0005623)	1
OG0029426	Cellular Component	organelle(GO:0043226)	1
OG0029429	Cellular Component	cell part(GO:0044464)	1
OG0029429	Cellular Component	cell(GO:0005623)	1
OG0029429	Cellular Component	organelle(GO:0043226)	1
OG0029430	Cellular Component	cell part(GO:0044464)	1
OG0029430	Cellular Component	cell(GO:0005623)	1
OG0029430	Cellular Component	organelle(GO:0043226)	1
OG0029431	Cellular Component	cell part(GO:0044464)	1
OG0029431	Cellular Component	cell(GO:0005623)	1
OG0029431	Cellular Component	organelle(GO:0043226)	1
OG0029449	Cellular Component	membrane(GO:0016020)	1
OG0029453	Cellular Component	cell part(GO:0044464)	1
OG0029453	Cellular Component	cell(GO:0005623)	1
OG0029453	Cellular Component	organelle(GO:0043226)	1
OG0029454	Cellular Component	cell part(GO:0044464)	1
OG0029454	Cellular Component	cell(GO:0005623)	1
OG0029454	Cellular Component	membrane(GO:0016020)	1
OG0029457	Cellular Component	cell part(GO:0044464)	1
OG0029457	Cellular Component	cell(GO:0005623)	1
OG0029457	Cellular Component	membrane(GO:0016020)	1
OG0029457	Cellular Component	organelle(GO:0043226)	1
OG0029457	Cellular Component	protein-containing complex(GO:0032991)	1
OG0029458	Cellular Component	cell part(GO:0044464)	1
OG0029458	Cellular Component	cell(GO:0005623)	1
OG0029458	Cellular Component	protein-containing complex(GO:0032991)	1
OG0029460	Cellular Component	cell part(GO:0044464)	1
OG0029460	Cellular Component	cell(GO:0005623)	1
OG0029460	Cellular Component	organelle(GO:0043226)	1
OG0029461	Cellular Component	cell part(GO:0044464)	1
OG0029461	Cellular Component	cell(GO:0005623)	1
OG0029463	Cellular Component	cell part(GO:0044464)	1
OG0029463	Cellular Component	cell(GO:0005623)	1
OG0029463	Cellular Component	membrane(GO:0016020)	1
OG0029466	Cellular Component	cell part(GO:0044464)	1
OG0029466	Cellular Component	cell(GO:0005623)	1
OG0029467	Cellular Component	cell part(GO:0044464)	1
OG0029467	Cellular Component	cell(GO:0005623)	1
OG0029468	Cellular Component	nucleoid(GO:0009295)	1
OG0029470	Cellular Component	cell part(GO:0044464)	1
OG0029470	Cellular Component	cell(GO:0005623)	1

OG0029470	Cellular Component	membrane(GO:0016020)	1
OG0029470	Cellular Component	organelle part(GO:0044422)	1
OG0029470	Cellular Component	organelle(GO:0043226)	1
OG0029470	Cellular Component	protein-containing complex(GO:0032991)	1
OG0029471	Cellular Component	cell part(GO:0044464)	1
OG0029471	Cellular Component	cell(GO:0005623)	1
OG0029471	Cellular Component	organelle part(GO:0044422)	1
OG0029471	Cellular Component	organelle(GO:0043226)	1
OG0029471	Cellular Component	protein-containing complex(GO:0032991)	1
OG0029472	Cellular Component	cell part(GO:0044464)	1
OG0029472	Cellular Component	cell(GO:0005623)	1
OG0029472	Cellular Component	membrane(GO:0016020)	1
OG0029473	Cellular Component	cell part(GO:0044464)	1
OG0029473	Cellular Component	cell(GO:0005623)	1
OG0029474	Cellular Component	cell part(GO:0044464)	1
OG0029474	Cellular Component	cell(GO:0005623)	1
OG0029475	Cellular Component	cell part(GO:0044464)	1
OG0029475	Cellular Component	cell(GO:0005623)	1
OG0029478	Cellular Component	cell part(GO:0044464)	1
OG0029478	Cellular Component	cell(GO:0005623)	1
OG0029478	Cellular Component	membrane(GO:0016020)	1
OG0029479	Cellular Component	cell part(GO:0044464)	1
OG0029479	Cellular Component	cell(GO:0005623)	1
OG0029482	Cellular Component	cell part(GO:0044464)	1
OG0029482	Cellular Component	cell(GO:0005623)	1
OG0029482	Cellular Component	membrane(GO:0016020)	1
OG0029484	Cellular Component	cell part(GO:0044464)	1
OG0029484	Cellular Component	cell(GO:0005623)	1
OG0029485	Cellular Component	cell part(GO:0044464)	1
OG0029485	Cellular Component	cell(GO:0005623)	1
OG0029485	Cellular Component	membrane(GO:0016020)	1
OG0029486	Cellular Component	cell part(GO:0044464)	1
OG0029486	Cellular Component	cell(GO:0005623)	1
OG0029486	Cellular Component	protein-containing complex(GO:0032991)	1
OG0029490	Cellular Component	cell part(GO:0044464)	1
OG0029490	Cellular Component	cell(GO:0005623)	1
OG0029490	Cellular Component	membrane(GO:0016020)	1
OG0029491	Cellular Component	cell part(GO:0044464)	1
OG0029491	Cellular Component	cell(GO:0005623)	1
OG0029491	Cellular Component	membrane(GO:0016020)	1
OG0029491	Cellular Component	protein-containing complex(GO:0032991)	1
OG0029492	Cellular Component	cell part(GO:0044464)	1
OG0029492	Cellular Component	cell(GO:0005623)	1
OG0029492	Cellular Component	membrane part(GO:0044425)	1
OG0029492	Cellular Component	membrane(GO:0016020)	1
OG0029492	Cellular Component	organelle part(GO:0044422)	1

OG0029492	Cellular Component	organelle(GO:0043226)	1
OG0029492	Cellular Component	protein-containing complex(GO:0032991)	1
OG0029494	Cellular Component	cell part(GO:0044464)	1
OG0029494	Cellular Component	cell(GO:0005623)	1
OG0029494	Cellular Component	membrane part(GO:0044425)	1
OG0029494	Cellular Component	membrane(GO:0016020)	1
OG0029494	Cellular Component	organelle part(GO:0044422)	1
OG0029494	Cellular Component	organelle(GO:0043226)	1
OG0029494	Cellular Component	protein-containing complex(GO:0032991)	1
OG0029497	Cellular Component	cell part(GO:0044464)	1
OG0029497	Cellular Component	cell(GO:0005623)	1
OG0029497	Cellular Component	membrane(GO:0016020)	1
OG0029500	Cellular Component	cell part(GO:0044464)	1
OG0029500	Cellular Component	cell(GO:0005623)	1
OG0029500	Cellular Component	membrane(GO:0016020)	1
OG0029507	Cellular Component	cell part(GO:0044464)	1
OG0029507	Cellular Component	cell(GO:0005623)	1
OG0029507	Cellular Component	organelle(GO:0043226)	1
OG0029509	Cellular Component	cell part(GO:0044464)	1
OG0029509	Cellular Component	cell(GO:0005623)	1
OG0029509	Cellular Component	membrane part(GO:0044425)	1
OG0029509	Cellular Component	membrane(GO:0016020)	1
OG0029520	Cellular Component	cell part(GO:0044464)	1
OG0029520	Cellular Component	cell(GO:0005623)	1
OG0029522	Cellular Component	cell part(GO:0044464)	1
OG0029522	Cellular Component	cell(GO:0005623)	1
OG0029522	Cellular Component	membrane-enclosed lumen(GO:0031974)	1
OG0029522	Cellular Component	organelle part(GO:0044422)	1
OG0029522	Cellular Component	organelle(GO:0043226)	1
OG0029522	Cellular Component	protein-containing complex(GO:0032991)	1
OG0029523	Cellular Component	cell part(GO:0044464)	1
OG0029523	Cellular Component	cell(GO:0005623)	1
OG0029523	Cellular Component	membrane(GO:0016020)	1
OG0029525	Cellular Component	membrane(GO:0016020)	1
OG0029527	Cellular Component	cell part(GO:0044464)	1
OG0029527	Cellular Component	cell(GO:0005623)	1
OG0029530	Cellular Component	cell part(GO:0044464)	1
OG0029530	Cellular Component	cell(GO:0005623)	1
OG0029530	Cellular Component	membrane(GO:0016020)	1
OG0029534	Cellular Component	cell part(GO:0044464)	1
OG0029534	Cellular Component	cell(GO:0005623)	1
OG0029536	Cellular Component	cell part(GO:0044464)	1
OG0029536	Cellular Component	cell(GO:0005623)	1
OG0029536	Cellular Component	membrane(GO:0016020)	1
OG0029539	Cellular Component	cell part(GO:0044464)	1
OG0029539	Cellular Component	cell(GO:0005623)	1

OG0029539	Cellular Component	membrane(GO:0016020)	1
OG0029540	Cellular Component	cell part(GO:0044464)	1
OG0029540	Cellular Component	cell(GO:0005623)	1
OG0029542	Cellular Component	cell part(GO:0044464)	1
OG0029542	Cellular Component	cell(GO:0005623)	1
OG0029543	Cellular Component	cell part(GO:0044464)	1
OG0029543	Cellular Component	cell(GO:0005623)	1
OG0029543	Cellular Component	membrane(GO:0016020)	1
OG0029548	Cellular Component	cell part(GO:0044464)	1
OG0029548	Cellular Component	cell(GO:0005623)	1
OG0029548	Cellular Component	membrane(GO:0016020)	1
OG0029550	Cellular Component	cell part(GO:0044464)	1
OG0029550	Cellular Component	cell(GO:0005623)	1
OG0029550	Cellular Component	membrane(GO:0016020)	1
OG0029553	Cellular Component	cell part(GO:0044464)	1
OG0029553	Cellular Component	cell(GO:0005623)	1
OG0029554	Cellular Component	cell part(GO:0044464)	1
OG0029554	Cellular Component	cell(GO:0005623)	1
OG0029554	Cellular Component	membrane(GO:0016020)	1
OG0029560	Cellular Component	cell part(GO:0044464)	1
OG0029560	Cellular Component	cell(GO:0005623)	1
OG0029560	Cellular Component	membrane(GO:0016020)	1
OG0029562	Cellular Component	cell part(GO:0044464)	1
OG0029562	Cellular Component	cell(GO:0005623)	1
OG0029567	Cellular Component	cell part(GO:0044464)	1
OG0029567	Cellular Component	cell(GO:0005623)	1
OG0029568	Cellular Component	cell part(GO:0044464)	1
OG0029568	Cellular Component	cell(GO:0005623)	1
OG0029568	Cellular Component	nucleoid(GO:0009295)	1
OG0029569	Cellular Component	cell part(GO:0044464)	1
OG0029569	Cellular Component	cell(GO:0005623)	1
OG0029569	Cellular Component	membrane(GO:0016020)	1
OG0029575	Cellular Component	cell part(GO:0044464)	1
OG0029575	Cellular Component	cell(GO:0005623)	1
OG0029575	Cellular Component	organelle(GO:0043226)	1
OG0029576	Cellular Component	cell part(GO:0044464)	1
OG0029576	Cellular Component	cell(GO:0005623)	1
OG0029577	Cellular Component	cell part(GO:0044464)	1
OG0029577	Cellular Component	cell(GO:0005623)	1
OG0029578	Cellular Component	cell part(GO:0044464)	1
OG0029578	Cellular Component	cell(GO:0005623)	1
OG0029578	Cellular Component	membrane(GO:0016020)	1
OG0029581	Cellular Component	cell junction(GO:0030054)	1
OG0029581	Cellular Component	cell part(GO:0044464)	1
OG0029581	Cellular Component	cell(GO:0005623)	1
OG0029581	Cellular Component	extracellular region(GO:0005576)	1
OG0029581	Cellular Component	symplast(GO:0055044)	1

OG0029584	Cellular Component	cell part(GO:0044464)	1
OG0029584	Cellular Component	cell(GO:0005623)	1
OG0029584	Cellular Component	organelle(GO:0043226)	1
OG0029585	Cellular Component	cell junction(GO:0030054)	1
OG0029585	Cellular Component	cell part(GO:0044464)	1
OG0029585	Cellular Component	cell(GO:0005623)	1
OG0029585	Cellular Component	extracellular region(GO:0005576)	1
OG0029585	Cellular Component	membrane(GO:0016020)	1
OG0029585	Cellular Component	organelle part(GO:0044422)	1
OG0029585	Cellular Component	organelle(GO:0043226)	1
OG0029585	Cellular Component	symplast(GO:0055044)	1
OG0029586	Cellular Component	cell part(GO:0044464)	1
OG0029586	Cellular Component	cell(GO:0005623)	1
OG0029586	Cellular Component	membrane(GO:0016020)	1
OG0029586	Cellular Component	organelle(GO:0043226)	1
OG0029588	Cellular Component	cell part(GO:0044464)	1
OG0029588	Cellular Component	cell(GO:0005623)	1
OG0029588	Cellular Component	protein-containing complex(GO:0032991)	1
OG0029589	Cellular Component	cell junction(GO:0030054)	1
OG0029589	Cellular Component	cell part(GO:0044464)	1
OG0029589	Cellular Component	cell(GO:0005623)	1
OG0029589	Cellular Component	membrane(GO:0016020)	1
OG0029589	Cellular Component	membrane-enclosed lumen(GO:0031974)	1
OG0029589	Cellular Component	organelle part(GO:0044422)	1
OG0029589	Cellular Component	organelle(GO:0043226)	1
OG0029589	Cellular Component	protein-containing complex(GO:0032991)	1
OG0029589	Cellular Component	symplast(GO:0055044)	1
OG0029594	Cellular Component	cell part(GO:0044464)	1
OG0029594	Cellular Component	cell(GO:0005623)	1
OG0029594	Cellular Component	organelle(GO:0043226)	1
OG0029602	Cellular Component	cell part(GO:0044464)	1
OG0029602	Cellular Component	cell(GO:0005623)	1
OG0029602	Cellular Component	membrane(GO:0016020)	1
OG0029602	Cellular Component	organelle part(GO:0044422)	1
OG0029602	Cellular Component	organelle(GO:0043226)	1
OG0029605	Cellular Component	cell part(GO:0044464)	1
OG0029605	Cellular Component	cell(GO:0005623)	1
OG0029605	Cellular Component	organelle(GO:0043226)	1
OG0029606	Cellular Component	cell part(GO:0044464)	1
OG0029606	Cellular Component	cell(GO:0005623)	1
OG0029606	Cellular Component	membrane(GO:0016020)	1
OG0029606	Cellular Component	organelle part(GO:0044422)	1
OG0029606	Cellular Component	organelle(GO:0043226)	1
OG0029610	Cellular Component	cell part(GO:0044464)	1
OG0029610	Cellular Component	cell(GO:0005623)	1
OG0029610	Cellular Component	organelle part(GO:0044422)	1
OG0029610	Cellular Component	organelle(GO:0043226)	1

OG0029610	Cellular Component	protein-containing complex(GO:0032991)	1
OG0029621	Cellular Component	extracellular region(GO:0005576)	1
OG0029626	Cellular Component	cell part(GO:0044464)	1
OG0029626	Cellular Component	cell(GO:0005623)	1
OG0029626	Cellular Component	membrane(GO:0016020)	1
OG0029626	Cellular Component	organelle part(GO:0044422)	1
OG0029626	Cellular Component	organelle(GO:0043226)	1
OG0029629	Cellular Component	cell part(GO:0044464)	1
OG0029629	Cellular Component	cell(GO:0005623)	1
OG0029629	Cellular Component	organelle part(GO:0044422)	1
OG0029629	Cellular Component	organelle(GO:0043226)	1
OG0029629	Cellular Component	protein-containing complex(GO:0032991)	1
OG0029633	Cellular Component	cell part(GO:0044464)	1
OG0029633	Cellular Component	cell(GO:0005623)	1
OG0029633	Cellular Component	membrane(GO:0016020)	1
OG0029636	Cellular Component	cell part(GO:0044464)	1
OG0029636	Cellular Component	cell(GO:0005623)	1
OG0029636	Cellular Component	membrane(GO:0016020)	1
OG0029637	Cellular Component	cell part(GO:0044464)	1
OG0029637	Cellular Component	cell(GO:0005623)	1
OG0029637	Cellular Component	membrane(GO:0016020)	1
OG0029639	Cellular Component	cell part(GO:0044464)	1
OG0029639	Cellular Component	cell(GO:0005623)	1
OG0029639	Cellular Component	extracellular region part(GO:0044421)	1
OG0029639	Cellular Component	extracellular region(GO:0005576)	1
OG0029639	Cellular Component	organelle part(GO:0044422)	1
OG0029639	Cellular Component	organelle(GO:0043226)	1
OG0029639	Cellular Component	protein-containing complex(GO:0032991)	1
OG0029640	Cellular Component	cell part(GO:0044464)	1
OG0029640	Cellular Component	cell(GO:0005623)	1
OG0029642	Cellular Component	cell part(GO:0044464)	1
OG0029642	Cellular Component	cell(GO:0005623)	1
OG0029642	Cellular Component	membrane(GO:0016020)	1
OG0029645	Cellular Component	cell part(GO:0044464)	1
OG0029645	Cellular Component	cell(GO:0005623)	1
OG0029645	Cellular Component	organelle(GO:0043226)	1
OG0029647	Cellular Component	cell part(GO:0044464)	1
OG0029647	Cellular Component	cell(GO:0005623)	1
OG0029647	Cellular Component	membrane-enclosed lumen(GO:0031974)	1
OG0029647	Cellular Component	organelle part(GO:0044422)	1
OG0029647	Cellular Component	organelle(GO:0043226)	1
OG0029650	Cellular Component	cell part(GO:0044464)	1
OG0029650	Cellular Component	cell(GO:0005623)	1
OG0029651	Cellular Component	membrane(GO:0016020)	1
OG0029656	Cellular Component	cell part(GO:0044464)	1
OG0029656	Cellular Component	cell(GO:0005623)	1

OG0029656	Cellular Component	membrane(GO:0016020)	1
OG0029656	Cellular Component	organelle part(GO:0044422)	1
OG0029656	Cellular Component	organelle(GO:0043226)	1
OG0029660	Cellular Component	cell part(GO:0044464)	1
OG0029660	Cellular Component	cell(GO:0005623)	1
OG0029664	Cellular Component	cell part(GO:0044464)	1
OG0029664	Cellular Component	cell(GO:0005623)	1
OG0029664	Cellular Component	membrane(GO:0016020)	1
OG0029665	Cellular Component	extracellular region(GO:0005576)	1
OG0029675	Cellular Component	cell part(GO:0044464)	1
OG0029675	Cellular Component	cell(GO:0005623)	1
OG0029675	Cellular Component	organelle(GO:0043226)	1
OG0029679	Cellular Component	cell part(GO:0044464)	1
OG0029679	Cellular Component	cell(GO:0005623)	1
OG0029679	Cellular Component	organelle(GO:0043226)	1
OG0029683	Cellular Component	membrane(GO:0016020)	1
OG0029686	Cellular Component	cell part(GO:0044464)	1
OG0029686	Cellular Component	cell(GO:0005623)	1
OG0029686	Cellular Component	membrane-enclosed lumen(GO:0031974)	1
OG0029686	Cellular Component	organelle part(GO:0044422)	1
OG0029686	Cellular Component	organelle(GO:0043226)	1
OG0029686	Cellular Component	protein-containing complex(GO:0032991)	1
OG0029691	Cellular Component	cell part(GO:0044464)	1
OG0029691	Cellular Component	cell(GO:0005623)	1
OG0029691	Cellular Component	membrane(GO:0016020)	1
OG0029692	Cellular Component	cell part(GO:0044464)	1
OG0029692	Cellular Component	cell(GO:0005623)	1
OG0029692	Cellular Component	membrane(GO:0016020)	1
OG0029693	Cellular Component	cell part(GO:0044464)	1
OG0029693	Cellular Component	cell(GO:0005623)	1
OG0029701	Cellular Component	membrane(GO:0016020)	1
OG0029743	Cellular Component	membrane(GO:0016020)	1
OG0029744	Cellular Component	cell part(GO:0044464)	1
OG0029744	Cellular Component	cell(GO:0005623)	1
OG0029744	Cellular Component	extracellular region(GO:0005576)	1
OG0029744	Cellular Component	organelle(GO:0043226)	1
OG0029750	Cellular Component	cell part(GO:0044464)	1
OG0029750	Cellular Component	cell(GO:0005623)	1
OG0029750	Cellular Component	membrane-enclosed lumen(GO:0031974)	1
OG0029750	Cellular Component	organelle part(GO:0044422)	1
OG0029750	Cellular Component	organelle(GO:0043226)	1
OG0029753	Cellular Component	cell part(GO:0044464)	1
OG0029753	Cellular Component	cell(GO:0005623)	1
OG0029753	Cellular Component	organelle(GO:0043226)	1
OG0029754	Cellular Component	cell part(GO:0044464)	1
OG0029754	Cellular Component	cell(GO:0005623)	1
OG0029754	Cellular Component	organelle(GO:0043226)	1

OG0029756	Cellular Component	cell part(GO:0044464)	1
OG0029756	Cellular Component	cell(GO:0005623)	1
OG0029756	Cellular Component	organelle(GO:0043226)	1
OG0029757	Cellular Component	cell part(GO:0044464)	1
OG0029757	Cellular Component	cell(GO:0005623)	1
OG0029757	Cellular Component	membrane-enclosed lumen(GO:0031974)	1
OG0029757	Cellular Component	organelle part(GO:0044422)	1
OG0029757	Cellular Component	organelle(GO:0043226)	1
OG0029757	Cellular Component	protein-containing complex(GO:0032991)	1
OG0029758	Cellular Component	cell part(GO:0044464)	1
OG0029758	Cellular Component	cell(GO:0005623)	1
OG0029760	Cellular Component	cell junction(GO:0030054)	1
OG0029760	Cellular Component	cell part(GO:0044464)	1
OG0029760	Cellular Component	cell(GO:0005623)	1
OG0029760	Cellular Component	membrane(GO:0016020)	1
OG0029760	Cellular Component	organelle part(GO:0044422)	1
OG0029760	Cellular Component	organelle(GO:0043226)	1
OG0029760	Cellular Component	symplast(GO:0055044)	1
OG0029768	Cellular Component	cell part(GO:0044464)	1
OG0029768	Cellular Component	cell(GO:0005623)	1
OG0029768	Cellular Component	membrane part(GO:0044425)	1
OG0029768	Cellular Component	membrane(GO:0016020)	1
OG0029768	Cellular Component	organelle part(GO:0044422)	1
OG0029768	Cellular Component	organelle(GO:0043226)	1
OG0029776	Cellular Component	cell part(GO:0044464)	1
OG0029776	Cellular Component	cell(GO:0005623)	1
OG0029776	Cellular Component	organelle part(GO:0044422)	1
OG0029776	Cellular Component	organelle(GO:0043226)	1
OG0029782	Cellular Component	cell part(GO:0044464)	1
OG0029782	Cellular Component	cell(GO:0005623)	1
OG0029782	Cellular Component	membrane(GO:0016020)	1
OG0029782	Cellular Component	organelle part(GO:0044422)	1
OG0029782	Cellular Component	organelle(GO:0043226)	1
OG0029785	Cellular Component	cell part(GO:0044464)	1
OG0029785	Cellular Component	cell(GO:0005623)	1
OG0029785	Cellular Component	organelle(GO:0043226)	1
OG0029788	Cellular Component	cell part(GO:0044464)	1
OG0029788	Cellular Component	cell(GO:0005623)	1
OG0029788	Cellular Component	organelle(GO:0043226)	1
OG0029788	Cellular Component	protein-containing complex(GO:0032991)	1
OG0029789	Cellular Component	cell part(GO:0044464)	1
OG0029789	Cellular Component	cell(GO:0005623)	1
OG0029789	Cellular Component	membrane(GO:0016020)	1
OG0029792	Cellular Component	cell part(GO:0044464)	1
OG0029792	Cellular Component	cell(GO:0005623)	1
OG0029792	Cellular Component	organelle part(GO:0044422)	1
OG0029792	Cellular Component	organelle(GO:0043226)	1

OG0029794	Cellular Component	cell part(GO:0044464)	1
OG0029794	Cellular Component	cell(GO:0005623)	1
OG0029794	Cellular Component	organelle(GO:0043226)	1
OG0029797	Cellular Component	cell part(GO:0044464)	1
OG0029797	Cellular Component	cell(GO:0005623)	1
OG0029797	Cellular Component	membrane(GO:0016020)	1
OG0029797	Cellular Component	organelle part(GO:0044422)	1
OG0029797	Cellular Component	organelle(GO:0043226)	1
OG0029807	Cellular Component	cell part(GO:0044464)	1
OG0029807	Cellular Component	cell(GO:0005623)	1
OG0029807	Cellular Component	membrane part(GO:0044425)	1
OG0029807	Cellular Component	membrane(GO:0016020)	1
OG0029807	Cellular Component	organelle part(GO:0044422)	1
OG0029807	Cellular Component	organelle(GO:0043226)	1
OG0029810	Cellular Component	cell part(GO:0044464)	1
OG0029810	Cellular Component	cell(GO:0005623)	1
OG0029810	Cellular Component	membrane(GO:0016020)	1
OG0029810	Cellular Component	organelle part(GO:0044422)	1
OG0029810	Cellular Component	organelle(GO:0043226)	1
OG0029812	Cellular Component	cell part(GO:0044464)	1
OG0029812	Cellular Component	cell(GO:0005623)	1
OG0029812	Cellular Component	organelle part(GO:0044422)	1
OG0029812	Cellular Component	organelle(GO:0043226)	1
OG0029812	Cellular Component	protein-containing complex(GO:0032991)	1
OG0029813	Cellular Component	cell part(GO:0044464)	1
OG0029813	Cellular Component	cell(GO:0005623)	1
OG0029813	Cellular Component	organelle part(GO:0044422)	1
OG0029813	Cellular Component	organelle(GO:0043226)	1
OG0029820	Cellular Component	cell part(GO:0044464)	1
OG0029820	Cellular Component	cell(GO:0005623)	1
OG0029820	Cellular Component	organelle(GO:0043226)	1
OG0029828	Cellular Component	cell part(GO:0044464)	1
OG0029828	Cellular Component	cell(GO:0005623)	1
OG0029828	Cellular Component	membrane(GO:0016020)	1
OG0029828	Cellular Component	organelle part(GO:0044422)	1
OG0029828	Cellular Component	organelle(GO:0043226)	1
OG0029839	Cellular Component	cell part(GO:0044464)	1
OG0029839	Cellular Component	cell(GO:0005623)	1
OG0029839	Cellular Component	protein-containing complex(GO:0032991)	1
OG0029840	Cellular Component	cell part(GO:0044464)	1
OG0029840	Cellular Component	cell(GO:0005623)	1
OG0029840	Cellular Component	protein-containing complex(GO:0032991)	1
OG0029841	Cellular Component	cell part(GO:0044464)	1
OG0029841	Cellular Component	cell(GO:0005623)	1
OG0029842	Cellular Component	cell part(GO:0044464)	1
OG0029842	Cellular Component	cell(GO:0005623)	1

OG0029842	Cellular Component	membrane part(GO:0044425)	1
OG0029842	Cellular Component	membrane(GO:0016020)	1
OG0029847	Cellular Component	cell part(GO:0044464)	1
OG0029847	Cellular Component	cell(GO:0005623)	1
OG0029847	Cellular Component	membrane(GO:0016020)	1
OG0029848	Cellular Component	cell part(GO:0044464)	1
OG0029848	Cellular Component	cell(GO:0005623)	1
OG0029848	Cellular Component	membrane(GO:0016020)	1
OG0029852	Cellular Component	cell part(GO:0044464)	1
OG0029852	Cellular Component	cell(GO:0005623)	1
OG0029852	Cellular Component	extracellular region(GO:0005576)	1
OG0029853	Cellular Component	cell part(GO:0044464)	1
OG0029853	Cellular Component	cell(GO:0005623)	1
OG0029854	Cellular Component	cell part(GO:0044464)	1
OG0029854	Cellular Component	cell(GO:0005623)	1
OG0029860	Cellular Component	cell part(GO:0044464)	1
OG0029860	Cellular Component	cell(GO:0005623)	1
OG0029861	Cellular Component	cell part(GO:0044464)	1
OG0029861	Cellular Component	cell(GO:0005623)	1
OG0029861	Cellular Component	membrane(GO:0016020)	1
OG0029862	Cellular Component	cell part(GO:0044464)	1
OG0029862	Cellular Component	cell(GO:0005623)	1
OG0029864	Cellular Component	cell part(GO:0044464)	1
OG0029864	Cellular Component	cell(GO:0005623)	1
OG0029869	Cellular Component	cell part(GO:0044464)	1
OG0029869	Cellular Component	cell(GO:0005623)	1
OG0029876	Cellular Component	cell part(GO:0044464)	1
OG0029876	Cellular Component	cell(GO:0005623)	1
OG0029876	Cellular Component	extracellular region(GO:0005576)	1
OG0029877	Cellular Component	cell part(GO:0044464)	1
OG0029877	Cellular Component	cell(GO:0005623)	1
OG0029878	Cellular Component	cell part(GO:0044464)	1
OG0029878	Cellular Component	cell(GO:0005623)	1
OG0029882	Cellular Component	cell part(GO:0044464)	1
OG0029882	Cellular Component	cell(GO:0005623)	1
OG0029882	Cellular Component	extracellular region(GO:0005576)	1
OG0029884	Cellular Component	cell part(GO:0044464)	1
OG0029884	Cellular Component	cell(GO:0005623)	1
OG0029884	Cellular Component	membrane(GO:0016020)	1
OG0029886	Cellular Component	cell part(GO:0044464)	1
OG0029886	Cellular Component	cell(GO:0005623)	1
OG0029886	Cellular Component	membrane(GO:0016020)	1
OG0029893	Cellular Component	cell part(GO:0044464)	1
OG0029893	Cellular Component	cell(GO:0005623)	1
OG0029894	Cellular Component	cell part(GO:0044464)	1
OG0029894	Cellular Component	cell(GO:0005623)	1
OG0029895	Cellular Component	cell part(GO:0044464)	1

OG0029895	Cellular Component	cell(GO:0005623)	1
OG0029895	Cellular Component	extracellular region(GO:0005576)	1
OG0029895	Cellular Component	membrane(GO:0016020)	1
OG0029899	Cellular Component	cell part(GO:0044464)	1
OG0029899	Cellular Component	cell(GO:0005623)	1
OG0029902	Cellular Component	cell part(GO:0044464)	1
OG0029902	Cellular Component	cell(GO:0005623)	1
OG0029902	Cellular Component	membrane(GO:0016020)	1
OG0029906	Cellular Component	cell part(GO:0044464)	1
OG0029906	Cellular Component	cell(GO:0005623)	1
OG0029907	Cellular Component	cell part(GO:0044464)	1
OG0029907	Cellular Component	cell(GO:0005623)	1
OG0029907	Cellular Component	membrane(GO:0016020)	1
OG0029909	Cellular Component	cell part(GO:0044464)	1
OG0029909	Cellular Component	cell(GO:0005623)	1
OG0029909	Cellular Component	membrane part(GO:0044425)	1
OG0029909	Cellular Component	membrane(GO:0016020)	1
OG0029910	Cellular Component	cell part(GO:0044464)	1
OG0029910	Cellular Component	cell(GO:0005623)	1
OG0029910	Cellular Component	membrane(GO:0016020)	1
OG0029913	Cellular Component	cell part(GO:0044464)	1
OG0029913	Cellular Component	cell(GO:0005623)	1
OG0029913	Cellular Component	membrane(GO:0016020)	1
OG0029914	Cellular Component	cell part(GO:0044464)	1
OG0029914	Cellular Component	cell(GO:0005623)	1
OG0029914	Cellular Component	membrane(GO:0016020)	1
OG0029917	Cellular Component	cell part(GO:0044464)	1
OG0029917	Cellular Component	cell(GO:0005623)	1
OG0029921	Cellular Component	cell part(GO:0044464)	1
OG0029921	Cellular Component	cell(GO:0005623)	1
OG0029922	Cellular Component	cell part(GO:0044464)	1
OG0029922	Cellular Component	cell(GO:0005623)	1
OG0029926	Cellular Component	cell part(GO:0044464)	1
OG0029926	Cellular Component	cell(GO:0005623)	1
OG0029926	Cellular Component	membrane(GO:0016020)	1
OG0029928	Cellular Component	cell part(GO:0044464)	1
OG0029928	Cellular Component	cell(GO:0005623)	1
OG0029928	Cellular Component	organelle(GO:0043226)	1
OG0029928	Cellular Component	protein-containing complex(GO:0032991)	1
OG0029941	Cellular Component	cell part(GO:0044464)	1
OG0029941	Cellular Component	cell(GO:0005623)	1
OG0029951	Cellular Component	cell part(GO:0044464)	1
OG0029951	Cellular Component	cell(GO:0005623)	1
OG0029951	Cellular Component	organelle part(GO:0044422)	1
OG0029951	Cellular Component	organelle(GO:0043226)	1
OG0029952	Cellular Component	cell part(GO:0044464)	1
OG0029952	Cellular Component	cell(GO:0005623)	1

OG0029952	Cellular Component	organelle part(GO:0044422)	1
OG0029952	Cellular Component	organelle(GO:0043226)	1
OG0029957	Cellular Component	cell part(GO:0044464)	1
OG0029957	Cellular Component	cell(GO:0005623)	1
OG0029957	Cellular Component	organelle(GO:0043226)	1
OG0029959	Cellular Component	cell part(GO:0044464)	1
OG0029959	Cellular Component	cell(GO:0005623)	1
OG0029959	Cellular Component	extracellular region(GO:0005576)	1
OG0029959	Cellular Component	membrane(GO:0016020)	1
OG0029962	Cellular Component	cell part(GO:0044464)	1
OG0029962	Cellular Component	cell(GO:0005623)	1
OG0029963	Cellular Component	cell part(GO:0044464)	1
OG0029963	Cellular Component	cell(GO:0005623)	1
OG0029964	Cellular Component	cell part(GO:0044464)	1
OG0029964	Cellular Component	cell(GO:0005623)	1
OG0029964	Cellular Component	membrane(GO:0016020)	1
OG0029965	Cellular Component	cell part(GO:0044464)	1
OG0029965	Cellular Component	cell(GO:0005623)	1
OG0029966	Cellular Component	cell part(GO:0044464)	1
OG0029966	Cellular Component	cell(GO:0005623)	1
OG0029966	Cellular Component	extracellular region(GO:0005576)	1
OG0029967	Cellular Component	cell part(GO:0044464)	1
OG0029967	Cellular Component	cell(GO:0005623)	1
OG0029967	Cellular Component	membrane(GO:0016020)	1
OG0029968	Cellular Component	cell part(GO:0044464)	1
OG0029968	Cellular Component	cell(GO:0005623)	1
OG0029968	Cellular Component	membrane(GO:0016020)	1
OG0029970	Cellular Component	cell part(GO:0044464)	1
OG0029970	Cellular Component	cell(GO:0005623)	1
OG0029970	Cellular Component	membrane(GO:0016020)	1
OG0029971	Cellular Component	cell part(GO:0044464)	1
OG0029971	Cellular Component	cell(GO:0005623)	1
OG0029971	Cellular Component	membrane(GO:0016020)	1
OG0029971	Cellular Component	organelle part(GO:0044422)	1
OG0029971	Cellular Component	organelle(GO:0043226)	1
OG0029971	Cellular Component	protein-containing complex(GO:0032991)	1
OG0029972	Cellular Component	cell part(GO:0044464)	1
OG0029972	Cellular Component	cell(GO:0005623)	1
OG0029972	Cellular Component	membrane(GO:0016020)	1
OG0029973	Cellular Component	cell part(GO:0044464)	1
OG0029973	Cellular Component	cell(GO:0005623)	1
OG0029973	Cellular Component	membrane(GO:0016020)	1
OG0029975	Cellular Component	cell part(GO:0044464)	1
OG0029975	Cellular Component	cell(GO:0005623)	1
OG0029975	Cellular Component	membrane(GO:0016020)	1
OG0029983	Cellular Component	cell part(GO:0044464)	1
OG0029983	Cellular Component	cell(GO:0005623)	1

OG0029983	Cellular Component	organelle(GO:0043226)	1
OG0029984	Cellular Component	cell part(GO:0044464)	1
OG0029984	Cellular Component	cell(GO:0005623)	1
OG0029984	Cellular Component	organelle(GO:0043226)	1
OG0029986	Cellular Component	cell part(GO:0044464)	1
OG0029986	Cellular Component	cell(GO:0005623)	1
OG0029986	Cellular Component	organelle part(GO:0044422)	1
OG0029986	Cellular Component	organelle(GO:0043226)	1
OG0029999	Cellular Component	cell part(GO:0044464)	1
OG0029999	Cellular Component	cell(GO:0005623)	1
OG0029999	Cellular Component	membrane(GO:0016020)	1
OG0029999	Cellular Component	organelle(GO:0043226)	1
OG0030001	Cellular Component	cell part(GO:0044464)	1
OG0030001	Cellular Component	cell(GO:0005623)	1
OG0030001	Cellular Component	membrane-enclosed lumen(GO:0031974)	1
OG0030001	Cellular Component	organelle part(GO:0044422)	1
OG0030001	Cellular Component	organelle(GO:0043226)	1
OG0030002	Cellular Component	cell part(GO:0044464)	1
OG0030002	Cellular Component	cell(GO:0005623)	1
OG0030002	Cellular Component	membrane-enclosed lumen(GO:0031974)	1
OG0030002	Cellular Component	organelle part(GO:0044422)	1
OG0030002	Cellular Component	organelle(GO:0043226)	1
OG0030004	Cellular Component	cell part(GO:0044464)	1
OG0030004	Cellular Component	cell(GO:0005623)	1
OG0030004	Cellular Component	membrane-enclosed lumen(GO:0031974)	1
OG0030004	Cellular Component	organelle part(GO:0044422)	1
OG0030004	Cellular Component	organelle(GO:0043226)	1
OG0030006	Cellular Component	cell part(GO:0044464)	1
OG0030006	Cellular Component	cell(GO:0005623)	1
OG0030006	Cellular Component	organelle(GO:0043226)	1
OG0030014	Cellular Component	cell part(GO:0044464)	1
OG0030014	Cellular Component	cell(GO:0005623)	1
OG0030014	Cellular Component	membrane-enclosed lumen(GO:0031974)	1
OG0030014	Cellular Component	organelle part(GO:0044422)	1
OG0030014	Cellular Component	organelle(GO:0043226)	1
OG0030016	Cellular Component	cell part(GO:0044464)	1
OG0030016	Cellular Component	cell(GO:0005623)	1
OG0030016	Cellular Component	membrane(GO:0016020)	1
OG0030016	Cellular Component	organelle part(GO:0044422)	1
OG0030016	Cellular Component	organelle(GO:0043226)	1
OG0030016	Cellular Component	protein-containing complex(GO:0032991)	1
OG0030017	Cellular Component	cell part(GO:0044464)	1
OG0030017	Cellular Component	cell(GO:0005623)	1
OG0030017	Cellular Component	organelle part(GO:0044422)	1
OG0030017	Cellular Component	organelle(GO:0043226)	1
OG0030019	Cellular Component	cell part(GO:0044464)	1
OG0030019	Cellular Component	cell(GO:0005623)	1

OG0030019	Cellular Component	membrane part(GO:0044425)	1
OG0030019	Cellular Component	membrane(GO:0016020)	1
OG0030026	Cellular Component	cell part(GO:0044464)	1
OG0030026	Cellular Component	cell(GO:0005623)	1
OG0030026	Cellular Component	membrane(GO:0016020)	1
OG0030026	Cellular Component	organelle(GO:0043226)	1
OG0030027	Cellular Component	cell part(GO:0044464)	1
OG0030027	Cellular Component	cell(GO:0005623)	1
OG0030027	Cellular Component	organelle part(GO:0044422)	1
OG0030027	Cellular Component	organelle(GO:0043226)	1
OG0030028	Cellular Component	cell part(GO:0044464)	1
OG0030028	Cellular Component	cell(GO:0005623)	1
OG0030028	Cellular Component	extracellular region(GO:0005576)	1
OG0030028	Cellular Component	membrane(GO:0016020)	1
OG0030028	Cellular Component	organelle part(GO:0044422)	1
OG0030028	Cellular Component	organelle(GO:0043226)	1
OG0030029	Cellular Component	cell part(GO:0044464)	1
OG0030029	Cellular Component	cell(GO:0005623)	1
OG0030032	Cellular Component	cell part(GO:0044464)	1
OG0030032	Cellular Component	cell(GO:0005623)	1
OG0030033	Cellular Component	cell part(GO:0044464)	1
OG0030033	Cellular Component	cell(GO:0005623)	1
OG0030035	Cellular Component	cell part(GO:0044464)	1
OG0030035	Cellular Component	cell(GO:0005623)	1
OG0030035	Cellular Component	membrane(GO:0016020)	1
OG0030036	Cellular Component	cell part(GO:0044464)	1
OG0030036	Cellular Component	cell(GO:0005623)	1
OG0030036	Cellular Component	membrane part(GO:0044425)	1
OG0030036	Cellular Component	membrane(GO:0016020)	1
OG0030037	Cellular Component	cell part(GO:0044464)	1
OG0030037	Cellular Component	cell(GO:0005623)	1
OG0030037	Cellular Component	protein-containing complex(GO:0032991)	1
OG0030045	Cellular Component	cell part(GO:0044464)	1
OG0030045	Cellular Component	cell(GO:0005623)	1
OG0030045	Cellular Component	organelle(GO:0043226)	1
OG0030046	Cellular Component	cell part(GO:0044464)	1
OG0030046	Cellular Component	cell(GO:0005623)	1
OG0030046	Cellular Component	organelle(GO:0043226)	1
OG0030050	Cellular Component	cell part(GO:0044464)	1
OG0030050	Cellular Component	cell(GO:0005623)	1
OG0030050	Cellular Component	organelle(GO:0043226)	1
OG0030051	Cellular Component	cell part(GO:0044464)	1
OG0030051	Cellular Component	cell(GO:0005623)	1
OG0030051	Cellular Component	membrane(GO:0016020)	1
OG0030051	Cellular Component	organelle part(GO:0044422)	1
OG0030051	Cellular Component	organelle(GO:0043226)	1
OG0030057	Cellular Component	cell part(GO:0044464)	1

OG0030057	Cellular Component	cell(GO:0005623)	1
OG0030057	Cellular Component	organelle(GO:0043226)	1
OG0030061	Cellular Component	membrane(GO:0016020)	1
OG0030062	Cellular Component	cell part(GO:0044464)	1
OG0030062	Cellular Component	cell(GO:0005623)	1
OG0030062	Cellular Component	membrane-enclosed lumen(GO:0031974)	1
OG0030062	Cellular Component	organelle part(GO:0044422)	1
OG0030062	Cellular Component	organelle(GO:0043226)	1
OG0030062	Cellular Component	protein-containing complex(GO:0032991)	1
OG0030063	Cellular Component	cell part(GO:0044464)	1
OG0030063	Cellular Component	cell(GO:0005623)	1
OG0030063	Cellular Component	membrane part(GO:0044425)	1
OG0030063	Cellular Component	membrane(GO:0016020)	1
OG0030065	Cellular Component	cell part(GO:0044464)	1
OG0030065	Cellular Component	cell(GO:0005623)	1
OG0030065	Cellular Component	membrane(GO:0016020)	1
OG0030065	Cellular Component	organelle part(GO:0044422)	1
OG0030065	Cellular Component	organelle(GO:0043226)	1
OG0030071	Cellular Component	cell part(GO:0044464)	1
OG0030071	Cellular Component	cell(GO:0005623)	1
OG0030074	Cellular Component	cell part(GO:0044464)	1
OG0030074	Cellular Component	cell(GO:0005623)	1
OG0030074	Cellular Component	membrane(GO:0016020)	1
OG0030074	Cellular Component	organelle part(GO:0044422)	1
OG0030074	Cellular Component	organelle(GO:0043226)	1
OG0030075	Cellular Component	cell junction(GO:0030054)	1
OG0030075	Cellular Component	cell part(GO:0044464)	1
OG0030075	Cellular Component	cell(GO:0005623)	1
OG0030075	Cellular Component	membrane(GO:0016020)	1
OG0030075	Cellular Component	organelle part(GO:0044422)	1
OG0030075	Cellular Component	organelle(GO:0043226)	1
OG0030075	Cellular Component	protein-containing complex(GO:0032991)	1
OG0030075	Cellular Component	symplast(GO:0055044)	1
OG0030077	Cellular Component	cell part(GO:0044464)	1
OG0030077	Cellular Component	cell(GO:0005623)	1
OG0030077	Cellular Component	extracellular region(GO:0005576)	1
OG0030077	Cellular Component	membrane(GO:0016020)	1
OG0030077	Cellular Component	membrane-enclosed lumen(GO:0031974)	1
OG0030077	Cellular Component	organelle part(GO:0044422)	1
OG0030077	Cellular Component	organelle(GO:0043226)	1
OG0030083	Cellular Component	cell part(GO:0044464)	1
OG0030083	Cellular Component	cell(GO:0005623)	1
OG0030083	Cellular Component	membrane(GO:0016020)	1
OG0030083	Cellular Component	organelle part(GO:0044422)	1
OG0030083	Cellular Component	organelle(GO:0043226)	1
OG0030086	Cellular Component	cell part(GO:0044464)	1
OG0030086	Cellular Component	cell(GO:0005623)	1

OG0030086	Cellular Component	extracellular region(GO:0005576)	1
OG0030087	Cellular Component	cell part(GO:0044464)	1
OG0030087	Cellular Component	cell(GO:0005623)	1
OG0030087	Cellular Component	organelle(GO:0043226)	1
OG0030088	Cellular Component	cell part(GO:0044464)	1
OG0030088	Cellular Component	cell(GO:0005623)	1
OG0030088	Cellular Component	organelle(GO:0043226)	1
OG0030089	Cellular Component	cell part(GO:0044464)	1
OG0030089	Cellular Component	cell(GO:0005623)	1
OG0030089	Cellular Component	membrane part(GO:0044425)	1
OG0030089	Cellular Component	membrane(GO:0016020)	1
OG0030089	Cellular Component	organelle(GO:0043226)	1
OG0030095	Cellular Component	cell part(GO:0044464)	1
OG0030095	Cellular Component	cell(GO:0005623)	1
OG0030095	Cellular Component	organelle part(GO:0044422)	1
OG0030095	Cellular Component	organelle(GO:0043226)	1
OG0030100	Cellular Component	cell part(GO:0044464)	1
OG0030100	Cellular Component	cell(GO:0005623)	1
OG0030100	Cellular Component	membrane(GO:0016020)	1
OG0030101	Cellular Component	cell part(GO:0044464)	1
OG0030101	Cellular Component	cell(GO:0005623)	1
OG0030106	Cellular Component	cell part(GO:0044464)	1
OG0030106	Cellular Component	cell(GO:0005623)	1
OG0030110	Cellular Component	cell part(GO:0044464)	1
OG0030110	Cellular Component	cell(GO:0005623)	1
OG0030111	Cellular Component	cell part(GO:0044464)	1
OG0030111	Cellular Component	cell(GO:0005623)	1
OG0030112	Cellular Component	cell part(GO:0044464)	1
OG0030112	Cellular Component	cell(GO:0005623)	1
OG0030112	Cellular Component	membrane(GO:0016020)	1
OG0030116	Cellular Component	cell part(GO:0044464)	1
OG0030116	Cellular Component	cell(GO:0005623)	1
OG0030116	Cellular Component	membrane(GO:0016020)	1
OG0030127	Cellular Component	cell part(GO:0044464)	1
OG0030127	Cellular Component	cell(GO:0005623)	1
OG0030127	Cellular Component	membrane(GO:0016020)	1
OG0030127	Cellular Component	organelle part(GO:0044422)	1
OG0030127	Cellular Component	organelle(GO:0043226)	1
OG0030132	Cellular Component	cell part(GO:0044464)	1
OG0030132	Cellular Component	cell(GO:0005623)	1
OG0030132	Cellular Component	extracellular region(GO:0005576)	1
OG0030132	Cellular Component	organelle(GO:0043226)	1
OG0030135	Cellular Component	cell part(GO:0044464)	1
OG0030135	Cellular Component	cell(GO:0005623)	1
OG0030135	Cellular Component	membrane-enclosed lumen(GO:0031974)	1
OG0030135	Cellular Component	nucleoid(GO:0009295)	1
OG0030135	Cellular Component	organelle part(GO:0044422)	1

OG0030135	Cellular Component	organelle(GO:0043226)	1
OG0030139	Cellular Component	cell part(GO:0044464)	1
OG0030139	Cellular Component	cell(GO:0005623)	1
OG0030139	Cellular Component	organelle(GO:0043226)	1
OG0030147	Cellular Component	cell part(GO:0044464)	1
OG0030147	Cellular Component	cell(GO:0005623)	1
OG0030147	Cellular Component	membrane(GO:0016020)	1
OG0030150	Cellular Component	cell part(GO:0044464)	1
OG0030150	Cellular Component	cell(GO:0005623)	1
OG0030150	Cellular Component	organelle part(GO:0044422)	1
OG0030150	Cellular Component	organelle(GO:0043226)	1
OG0030155	Cellular Component	cell junction(GO:0030054)	1
OG0030155	Cellular Component	cell part(GO:0044464)	1
OG0030155	Cellular Component	cell(GO:0005623)	1
OG0030155	Cellular Component	membrane(GO:0016020)	1
OG0030155	Cellular Component	organelle part(GO:0044422)	1
OG0030155	Cellular Component	organelle(GO:0043226)	1
OG0030155	Cellular Component	symplast(GO:0055044)	1
OG0030157	Cellular Component	cell junction(GO:0030054)	1
OG0030157	Cellular Component	cell part(GO:0044464)	1
OG0030157	Cellular Component	cell(GO:0005623)	1
OG0030157	Cellular Component	membrane(GO:0016020)	1
OG0030157	Cellular Component	symplast(GO:0055044)	1
OG0030158	Cellular Component	cell part(GO:0044464)	1
OG0030158	Cellular Component	cell(GO:0005623)	1
OG0030158	Cellular Component	extracellular region(GO:0005576)	1
OG0030158	Cellular Component	membrane(GO:0016020)	1
OG0030158	Cellular Component	organelle part(GO:0044422)	1
OG0030158	Cellular Component	organelle(GO:0043226)	1
OG0030162	Cellular Component	cell part(GO:0044464)	1
OG0030162	Cellular Component	cell(GO:0005623)	1
OG0030162	Cellular Component	membrane-enclosed lumen(GO:0031974)	1
OG0030162	Cellular Component	organelle part(GO:0044422)	1
OG0030162	Cellular Component	organelle(GO:0043226)	1
OG0030162	Cellular Component	protein-containing complex(GO:0032991)	1
OG0030166	Cellular Component	cell part(GO:0044464)	1
OG0030166	Cellular Component	cell(GO:0005623)	1
OG0030168	Cellular Component	cell part(GO:0044464)	1
OG0030168	Cellular Component	cell(GO:0005623)	1
OG0030168	Cellular Component	membrane(GO:0016020)	1
OG0030180	Cellular Component	cell part(GO:0044464)	1
OG0030180	Cellular Component	cell(GO:0005623)	1
OG0030180	Cellular Component	organelle(GO:0043226)	1
OG0030182	Cellular Component	cell part(GO:0044464)	1
OG0030182	Cellular Component	cell(GO:0005623)	1
OG0030182	Cellular Component	extracellular region(GO:0005576)	1
OG0030182	Cellular Component	membrane(GO:0016020)	1

OG0030182	Cellular Component	membrane-enclosed lumen(GO:0031974)	1
OG0030182	Cellular Component	organelle part(GO:0044422)	1
OG0030182	Cellular Component	organelle(GO:0043226)	1
OG0030184	Cellular Component	cell part(GO:0044464)	1
OG0030184	Cellular Component	cell(GO:0005623)	1
OG0030184	Cellular Component	organelle(GO:0043226)	1
OG0030191	Cellular Component	cell part(GO:0044464)	1
OG0030191	Cellular Component	cell(GO:0005623)	1
OG0030191	Cellular Component	organelle(GO:0043226)	1
OG0030193	Cellular Component	cell part(GO:0044464)	1
OG0030193	Cellular Component	cell(GO:0005623)	1
OG0030193	Cellular Component	organelle(GO:0043226)	1
OG0030195	Cellular Component	cell part(GO:0044464)	1
OG0030195	Cellular Component	cell(GO:0005623)	1
OG0030195	Cellular Component	organelle(GO:0043226)	1
OG0030196	Cellular Component	cell part(GO:0044464)	1
OG0030196	Cellular Component	cell(GO:0005623)	1
OG0030196	Cellular Component	organelle(GO:0043226)	1
OG0030200	Cellular Component	cell part(GO:0044464)	1
OG0030200	Cellular Component	cell(GO:0005623)	1
OG0030200	Cellular Component	organelle part(GO:0044422)	1
OG0030200	Cellular Component	organelle(GO:0043226)	1
OG0030202	Cellular Component	cell part(GO:0044464)	1
OG0030202	Cellular Component	cell(GO:0005623)	1
OG0030202	Cellular Component	membrane(GO:0016020)	1
OG0030202	Cellular Component	organelle part(GO:0044422)	1
OG0030202	Cellular Component	organelle(GO:0043226)	1
OG0030203	Cellular Component	cell part(GO:0044464)	1
OG0030203	Cellular Component	cell(GO:0005623)	1
OG0030203	Cellular Component	membrane(GO:0016020)	1
OG0030203	Cellular Component	organelle part(GO:0044422)	1
OG0030203	Cellular Component	organelle(GO:0043226)	1
OG0030204	Cellular Component	cell part(GO:0044464)	1
OG0030204	Cellular Component	cell(GO:0005623)	1
OG0030204	Cellular Component	membrane(GO:0016020)	1
OG0030204	Cellular Component	organelle part(GO:0044422)	1
OG0030204	Cellular Component	organelle(GO:0043226)	1
OG0030205	Cellular Component	cell part(GO:0044464)	1
OG0030205	Cellular Component	cell(GO:0005623)	1
OG0030205	Cellular Component	membrane(GO:0016020)	1
OG0030205	Cellular Component	organelle part(GO:0044422)	1
OG0030205	Cellular Component	organelle(GO:0043226)	1
OG0030209	Cellular Component	cell part(GO:0044464)	1
OG0030209	Cellular Component	cell(GO:0005623)	1
OG0030209	Cellular Component	organelle(GO:0043226)	1
OG0030213	Cellular Component	cell part(GO:0044464)	1
OG0030213	Cellular Component	cell(GO:0005623)	1

OG0030216	Cellular Component	cell part(GO:0044464)	1
OG0030216	Cellular Component	cell(GO:0005623)	1
OG0030218	Cellular Component	cell part(GO:0044464)	1
OG0030218	Cellular Component	cell(GO:0005623)	1
OG0030222	Cellular Component	cell part(GO:0044464)	1
OG0030222	Cellular Component	cell(GO:0005623)	1
OG0030223	Cellular Component	cell part(GO:0044464)	1
OG0030223	Cellular Component	cell(GO:0005623)	1
OG0030223	Cellular Component	membrane(GO:0016020)	1
OG0030225	Cellular Component	cell part(GO:0044464)	1
OG0030225	Cellular Component	cell(GO:0005623)	1
OG0030225	Cellular Component	membrane(GO:0016020)	1
OG0030230	Cellular Component	cell part(GO:0044464)	1
OG0030230	Cellular Component	cell(GO:0005623)	1
OG0030230	Cellular Component	membrane(GO:0016020)	1
OG0030232	Cellular Component	cell part(GO:0044464)	1
OG0030232	Cellular Component	cell(GO:0005623)	1
OG0030232	Cellular Component	membrane(GO:0016020)	1
OG0030233	Cellular Component	cell part(GO:0044464)	1
OG0030233	Cellular Component	cell(GO:0005623)	1
OG0030233	Cellular Component	membrane(GO:0016020)	1
OG0030234	Cellular Component	cell part(GO:0044464)	1
OG0030234	Cellular Component	cell(GO:0005623)	1
OG0030236	Cellular Component	cell part(GO:0044464)	1
OG0030236	Cellular Component	cell(GO:0005623)	1
OG0030236	Cellular Component	membrane(GO:0016020)	1
OG0030238	Cellular Component	cell part(GO:0044464)	1
OG0030238	Cellular Component	cell(GO:0005623)	1
OG0030239	Cellular Component	cell part(GO:0044464)	1
OG0030239	Cellular Component	cell(GO:0005623)	1
OG0030240	Cellular Component	cell part(GO:0044464)	1
OG0030240	Cellular Component	cell(GO:0005623)	1
OG0030241	Cellular Component	cell part(GO:0044464)	1
OG0030241	Cellular Component	cell(GO:0005623)	1
OG0030245	Cellular Component	cell part(GO:0044464)	1
OG0030245	Cellular Component	cell(GO:0005623)	1
OG0030246	Cellular Component	cell part(GO:0044464)	1
OG0030246	Cellular Component	cell(GO:0005623)	1
OG0030246	Cellular Component	membrane(GO:0016020)	1
OG0030247	Cellular Component	cell part(GO:0044464)	1
OG0030247	Cellular Component	cell(GO:0005623)	1
OG0030247	Cellular Component	membrane(GO:0016020)	1
OG0030248	Cellular Component	cell part(GO:0044464)	1
OG0030248	Cellular Component	cell(GO:0005623)	1
OG0030249	Cellular Component	cell part(GO:0044464)	1
OG0030249	Cellular Component	cell(GO:0005623)	1
OG0030250	Cellular Component	cell part(GO:0044464)	1

OG0030250	Cellular Component	cell(GO:0005623)	1
OG0030250	Cellular Component	membrane(GO:0016020)	1
OG0030251	Cellular Component	cell part(GO:0044464)	1
OG0030251	Cellular Component	cell(GO:0005623)	1
OG0030251	Cellular Component	extracellular region(GO:0005576)	1
OG0030251	Cellular Component	membrane part(GO:0044425)	1
OG0030251	Cellular Component	membrane(GO:0016020)	1
OG0030252	Cellular Component	cell part(GO:0044464)	1
OG0030252	Cellular Component	cell(GO:0005623)	1
OG0030252	Cellular Component	membrane(GO:0016020)	1
OG0030252	Cellular Component	organelle part(GO:0044422)	1
OG0030252	Cellular Component	organelle(GO:0043226)	1
OG0030252	Cellular Component	protein-containing complex(GO:0032991)	1
OG0030253	Cellular Component	cell part(GO:0044464)	1
OG0030253	Cellular Component	cell(GO:0005623)	1
OG0030253	Cellular Component	membrane(GO:0016020)	1
OG0030253	Cellular Component	organelle part(GO:0044422)	1
OG0030253	Cellular Component	organelle(GO:0043226)	1
OG0030253	Cellular Component	protein-containing complex(GO:0032991)	1
OG0030254	Cellular Component	cell part(GO:0044464)	1
OG0030254	Cellular Component	cell(GO:0005623)	1
OG0030254	Cellular Component	membrane(GO:0016020)	1
OG0030255	Cellular Component	cell part(GO:0044464)	1
OG0030255	Cellular Component	cell(GO:0005623)	1
OG0030255	Cellular Component	membrane(GO:0016020)	1
OG0030259	Cellular Component	cell part(GO:0044464)	1
OG0030259	Cellular Component	cell(GO:0005623)	1
OG0030259	Cellular Component	membrane(GO:0016020)	1
OG0030260	Cellular Component	cell part(GO:0044464)	1
OG0030260	Cellular Component	cell(GO:0005623)	1
OG0030260	Cellular Component	membrane(GO:0016020)	1
OG0030261	Cellular Component	cell part(GO:0044464)	1
OG0030261	Cellular Component	cell(GO:0005623)	1
OG0030261	Cellular Component	membrane(GO:0016020)	1
OG0030266	Cellular Component	cell part(GO:0044464)	1
OG0030266	Cellular Component	cell(GO:0005623)	1
OG0030266	Cellular Component	membrane(GO:0016020)	1
OG0030267	Cellular Component	cell part(GO:0044464)	1
OG0030267	Cellular Component	cell(GO:0005623)	1
OG0030267	Cellular Component	membrane(GO:0016020)	1
OG0030268	Cellular Component	cell part(GO:0044464)	1
OG0030268	Cellular Component	cell(GO:0005623)	1
OG0030269	Cellular Component	cell part(GO:0044464)	1
OG0030269	Cellular Component	cell(GO:0005623)	1
OG0030269	Cellular Component	extracellular region(GO:0005576)	1
OG0030269	Cellular Component	membrane(GO:0016020)	1
OG0030272	Cellular Component	cell part(GO:0044464)	1

OG0030272	Cellular Component	cell(GO:0005623)	1
OG0030272	Cellular Component	membrane(GO:0016020)	1
OG0030273	Cellular Component	cell part(GO:0044464)	1
OG0030273	Cellular Component	cell(GO:0005623)	1
OG0030273	Cellular Component	membrane(GO:0016020)	1
OG0030275	Cellular Component	cell part(GO:0044464)	1
OG0030275	Cellular Component	cell(GO:0005623)	1
OG0030275	Cellular Component	membrane(GO:0016020)	1
OG0030275	Cellular Component	organelle part(GO:0044422)	1
OG0030275	Cellular Component	organelle(GO:0043226)	1
OG0030275	Cellular Component	protein-containing complex(GO:0032991)	1
OG0030278	Cellular Component	cell part(GO:0044464)	1
OG0030278	Cellular Component	cell(GO:0005623)	1
OG0030278	Cellular Component	membrane(GO:0016020)	1
OG0030280	Cellular Component	cell part(GO:0044464)	1
OG0030280	Cellular Component	cell(GO:0005623)	1
OG0030280	Cellular Component	membrane(GO:0016020)	1
OG0030280	Cellular Component	membrane-enclosed lumen(GO:0031974)	1
OG0030280	Cellular Component	organelle part(GO:0044422)	1
OG0030280	Cellular Component	organelle(GO:0043226)	1
OG0030280	Cellular Component	protein-containing complex(GO:0032991)	1
OG0030281	Cellular Component	cell part(GO:0044464)	1
OG0030281	Cellular Component	cell(GO:0005623)	1
OG0030282	Cellular Component	cell part(GO:0044464)	1
OG0030282	Cellular Component	cell(GO:0005623)	1
OG0030282	Cellular Component	membrane(GO:0016020)	1
OG0030283	Cellular Component	cell part(GO:0044464)	1
OG0030283	Cellular Component	cell(GO:0005623)	1
OG0030283	Cellular Component	membrane part(GO:0044425)	1
OG0030283	Cellular Component	membrane(GO:0016020)	1
OG0030283	Cellular Component	protein-containing complex(GO:0032991)	1
OG0030284	Cellular Component	cell part(GO:0044464)	1
OG0030284	Cellular Component	cell(GO:0005623)	1
OG0030284	Cellular Component	membrane(GO:0016020)	1
OG0030285	Cellular Component	cell part(GO:0044464)	1
OG0030285	Cellular Component	cell(GO:0005623)	1
OG0030285	Cellular Component	membrane part(GO:0044425)	1
OG0030285	Cellular Component	membrane(GO:0016020)	1
OG0030285	Cellular Component	organelle part(GO:0044422)	1
OG0030285	Cellular Component	organelle(GO:0043226)	1
OG0030285	Cellular Component	protein-containing complex(GO:0032991)	1
OG0030286	Cellular Component	cell part(GO:0044464)	1
OG0030286	Cellular Component	cell(GO:0005623)	1
OG0030286	Cellular Component	membrane(GO:0016020)	1
OG0030287	Cellular Component	cell part(GO:0044464)	1
OG0030287	Cellular Component	cell(GO:0005623)	1

OG0030287	Cellular Component	membrane(GO:0016020)	1
OG0030288	Cellular Component	cell part(GO:0044464)	1
OG0030288	Cellular Component	cell(GO:0005623)	1
OG0030288	Cellular Component	membrane part(GO:0044425)	1
OG0030288	Cellular Component	membrane(GO:0016020)	1
OG0030288	Cellular Component	organelle part(GO:0044422)	1
OG0030288	Cellular Component	organelle(GO:0043226)	1
OG0030288	Cellular Component	protein-containing complex(GO:0032991)	1
OG0030291	Cellular Component	cell part(GO:0044464)	1
OG0030291	Cellular Component	cell(GO:0005623)	1
OG0030291	Cellular Component	membrane(GO:0016020)	1
OG0030291	Cellular Component	protein-containing complex(GO:0032991)	1
OG0030293	Cellular Component	cell part(GO:0044464)	1
OG0030293	Cellular Component	cell(GO:0005623)	1
OG0030293	Cellular Component	membrane(GO:0016020)	1
OG0030296	Cellular Component	cell part(GO:0044464)	1
OG0030296	Cellular Component	cell(GO:0005623)	1
OG0030296	Cellular Component	membrane(GO:0016020)	1
OG0030297	Cellular Component	cell part(GO:0044464)	1
OG0030297	Cellular Component	cell(GO:0005623)	1
OG0030298	Cellular Component	cell part(GO:0044464)	1
OG0030298	Cellular Component	cell(GO:0005623)	1
OG0030298	Cellular Component	membrane(GO:0016020)	1
OG0030300	Cellular Component	cell part(GO:0044464)	1
OG0030300	Cellular Component	cell(GO:0005623)	1
OG0030301	Cellular Component	cell part(GO:0044464)	1
OG0030301	Cellular Component	cell(GO:0005623)	1
OG0030302	Cellular Component	cell part(GO:0044464)	1
OG0030302	Cellular Component	cell(GO:0005623)	1
OG0030302	Cellular Component	membrane(GO:0016020)	1
OG0030305	Cellular Component	cell part(GO:0044464)	1
OG0030305	Cellular Component	cell(GO:0005623)	1
OG0030309	Cellular Component	cell part(GO:0044464)	1
OG0030309	Cellular Component	cell(GO:0005623)	1
OG0030310	Cellular Component	cell part(GO:0044464)	1
OG0030310	Cellular Component	cell(GO:0005623)	1
OG0030310	Cellular Component	membrane(GO:0016020)	1
OG0030310	Cellular Component	nucleoid(GO:0009295)	1
OG0030311	Cellular Component	cell part(GO:0044464)	1
OG0030311	Cellular Component	cell(GO:0005623)	1
OG0030311	Cellular Component	membrane(GO:0016020)	1
OG0030313	Cellular Component	membrane(GO:0016020)	1
OG0030314	Cellular Component	cell part(GO:0044464)	1
OG0030314	Cellular Component	cell(GO:0005623)	1
OG0030317	Cellular Component	cell part(GO:0044464)	1
OG0030317	Cellular Component	cell(GO:0005623)	1
OG0030320	Cellular Component	cell part(GO:0044464)	1

OG0030320	Cellular Component	cell(GO:0005623)	1
OG0030320	Cellular Component	extracellular region(GO:0005576)	1
OG0030320	Cellular Component	membrane part(GO:0044425)	1
OG0030320	Cellular Component	membrane(GO:0016020)	1
OG0030321	Cellular Component	cell part(GO:0044464)	1
OG0030321	Cellular Component	cell(GO:0005623)	1
OG0030324	Cellular Component	cell part(GO:0044464)	1
OG0030324	Cellular Component	cell(GO:0005623)	1
OG0030326	Cellular Component	cell part(GO:0044464)	1
OG0030326	Cellular Component	cell(GO:0005623)	1
OG0030326	Cellular Component	membrane(GO:0016020)	1
OG0030330	Cellular Component	cell part(GO:0044464)	1
OG0030330	Cellular Component	cell(GO:0005623)	1
OG0030330	Cellular Component	extracellular region(GO:0005576)	1
OG0030330	Cellular Component	membrane(GO:0016020)	1
OG0030336	Cellular Component	cell part(GO:0044464)	1
OG0030336	Cellular Component	cell(GO:0005623)	1
OG0030336	Cellular Component	membrane part(GO:0044425)	1
OG0030336	Cellular Component	membrane(GO:0016020)	1
OG0030348	Cellular Component	cell part(GO:0044464)	1
OG0030348	Cellular Component	cell(GO:0005623)	1
OG0030348	Cellular Component	organelle(GO:0043226)	1
OG0030350	Cellular Component	cell part(GO:0044464)	1
OG0030350	Cellular Component	cell(GO:0005623)	1
OG0030350	Cellular Component	membrane-enclosed lumen(GO:0031974)	1
OG0030350	Cellular Component	organelle part(GO:0044422)	1
OG0030350	Cellular Component	organelle(GO:0043226)	1
OG0030355	Cellular Component	cell part(GO:0044464)	1
OG0030355	Cellular Component	cell(GO:0005623)	1
OG0030355	Cellular Component	membrane(GO:0016020)	1
OG0030355	Cellular Component	organelle part(GO:0044422)	1
OG0030355	Cellular Component	organelle(GO:0043226)	1
OG0030356	Cellular Component	cell part(GO:0044464)	1
OG0030356	Cellular Component	cell(GO:0005623)	1
OG0030359	Cellular Component	cell part(GO:0044464)	1
OG0030359	Cellular Component	cell(GO:0005623)	1
OG0030359	Cellular Component	membrane(GO:0016020)	1
OG0030359	Cellular Component	organelle part(GO:0044422)	1
OG0030359	Cellular Component	organelle(GO:0043226)	1
OG0030361	Cellular Component	cell part(GO:0044464)	1
OG0030361	Cellular Component	cell(GO:0005623)	1
OG0030361	Cellular Component	membrane part(GO:0044425)	1
OG0030361	Cellular Component	membrane(GO:0016020)	1
OG0030361	Cellular Component	organelle part(GO:0044422)	1
OG0030361	Cellular Component	organelle(GO:0043226)	1
OG0030361	Cellular Component	protein-containing complex(GO:0032991)	1
OG0030367	Cellular Component	cell part(GO:0044464)	1

OG0030367	Cellular Component	cell(GO:0005623)	1
OG0030367	Cellular Component	membrane(GO:0016020)	1
OG0030370	Cellular Component	cell part(GO:0044464)	1
OG0030370	Cellular Component	cell(GO:0005623)	1
OG0030371	Cellular Component	cell part(GO:0044464)	1
OG0030371	Cellular Component	cell(GO:0005623)	1
OG0030372	Cellular Component	cell part(GO:0044464)	1
OG0030372	Cellular Component	cell(GO:0005623)	1
OG0030376	Cellular Component	cell part(GO:0044464)	1
OG0030376	Cellular Component	cell(GO:0005623)	1
OG0030376	Cellular Component	organelle(GO:0043226)	1
OG0030384	Cellular Component	cell part(GO:0044464)	1
OG0030384	Cellular Component	cell(GO:0005623)	1
OG0030384	Cellular Component	organelle(GO:0043226)	1
OG0030386	Cellular Component	cell junction(GO:0030054)	1
OG0030386	Cellular Component	cell part(GO:0044464)	1
OG0030386	Cellular Component	cell(GO:0005623)	1
OG0030386	Cellular Component	membrane part(GO:0044425)	1
OG0030386	Cellular Component	membrane(GO:0016020)	1
OG0030386	Cellular Component	symplast(GO:0055044)	1
OG0030388	Cellular Component	cell part(GO:0044464)	1
OG0030388	Cellular Component	cell(GO:0005623)	1
OG0030388	Cellular Component	organelle(GO:0043226)	1
OG0030392	Cellular Component	cell part(GO:0044464)	1
OG0030392	Cellular Component	cell(GO:0005623)	1
OG0030392	Cellular Component	organelle(GO:0043226)	1
OG0030397	Cellular Component	cell part(GO:0044464)	1
OG0030397	Cellular Component	cell(GO:0005623)	1
OG0030397	Cellular Component	membrane(GO:0016020)	1
OG0030398	Cellular Component	cell part(GO:0044464)	1
OG0030398	Cellular Component	cell(GO:0005623)	1
OG0030398	Cellular Component	membrane(GO:0016020)	1
OG0030398	Cellular Component	organelle part(GO:0044422)	1
OG0030398	Cellular Component	organelle(GO:0043226)	1
OG0030400	Cellular Component	cell part(GO:0044464)	1
OG0030400	Cellular Component	cell(GO:0005623)	1
OG0030400	Cellular Component	membrane(GO:0016020)	1
OG0030408	Cellular Component	cell part(GO:0044464)	1
OG0030408	Cellular Component	cell(GO:0005623)	1
OG0030408	Cellular Component	membrane part(GO:0044425)	1
OG0030408	Cellular Component	membrane(GO:0016020)	1
OG0030408	Cellular Component	organelle part(GO:0044422)	1
OG0030408	Cellular Component	organelle(GO:0043226)	1
OG0030408	Cellular Component	protein-containing complex(GO:0032991)	1
OG0030409	Cellular Component	cell part(GO:0044464)	1
OG0030409	Cellular Component	cell(GO:0005623)	1
OG0030409	Cellular Component	membrane part(GO:0044425)	1

OG0030409	Cellular Component	membrane(GO:0016020)	1
OG0030409	Cellular Component	organelle part(GO:0044422)	1
OG0030409	Cellular Component	organelle(GO:0043226)	1
OG0030409	Cellular Component	protein-containing complex(GO:0032991)	1
OG0030412	Cellular Component	cell part(GO:0044464)	1
OG0030412	Cellular Component	cell(GO:0005623)	1
OG0030412	Cellular Component	nucleoid(GO:0009295)	1
OG0030423	Cellular Component	cell part(GO:0044464)	1
OG0030423	Cellular Component	cell(GO:0005623)	1
OG0030423	Cellular Component	organelle(GO:0043226)	1
OG0030424	Cellular Component	cell part(GO:0044464)	1
OG0030424	Cellular Component	cell(GO:0005623)	1
OG0030424	Cellular Component	membrane(GO:0016020)	1
OG0030424	Cellular Component	organelle part(GO:0044422)	1
OG0030424	Cellular Component	organelle(GO:0043226)	1
OG0030425	Cellular Component	cell part(GO:0044464)	1
OG0030425	Cellular Component	cell(GO:0005623)	1
OG0030425	Cellular Component	membrane(GO:0016020)	1
OG0030425	Cellular Component	organelle part(GO:0044422)	1
OG0030425	Cellular Component	organelle(GO:0043226)	1
OG0030435	Cellular Component	cell part(GO:0044464)	1
OG0030435	Cellular Component	cell(GO:0005623)	1
OG0030435	Cellular Component	membrane(GO:0016020)	1
OG0030436	Cellular Component	cell part(GO:0044464)	1
OG0030436	Cellular Component	cell(GO:0005623)	1
OG0030436	Cellular Component	membrane(GO:0016020)	1
OG0030441	Cellular Component	cell part(GO:0044464)	1
OG0030441	Cellular Component	cell(GO:0005623)	1
OG0030442	Cellular Component	cell part(GO:0044464)	1
OG0030442	Cellular Component	cell(GO:0005623)	1
OG0030446	Cellular Component	cell junction(GO:0030054)	1
OG0030446	Cellular Component	cell part(GO:0044464)	1
OG0030446	Cellular Component	cell(GO:0005623)	1
OG0030446	Cellular Component	organelle(GO:0043226)	1
OG0030446	Cellular Component	symplast(GO:0055044)	1
OG0030450	Cellular Component	cell part(GO:0044464)	1
OG0030450	Cellular Component	cell(GO:0005623)	1
OG0030450	Cellular Component	organelle(GO:0043226)	1
OG0030454	Cellular Component	cell part(GO:0044464)	1
OG0030454	Cellular Component	cell(GO:0005623)	1
OG0030454	Cellular Component	organelle(GO:0043226)	1
OG0030455	Cellular Component	cell part(GO:0044464)	1
OG0030455	Cellular Component	cell(GO:0005623)	1
OG0030458	Cellular Component	cell part(GO:0044464)	1
OG0030458	Cellular Component	cell(GO:0005623)	1
OG0030458	Cellular Component	membrane(GO:0016020)	1
OG0030458	Cellular Component	organelle part(GO:0044422)	1

OG0030458	Cellular Component	organelle(GO:0043226)	1
OG0030459	Cellular Component	cell part(GO:0044464)	1
OG0030459	Cellular Component	cell(GO:0005623)	1
OG0030459	Cellular Component	organelle(GO:0043226)	1
OG0030463	Cellular Component	cell part(GO:0044464)	1
OG0030463	Cellular Component	cell(GO:0005623)	1
OG0030463	Cellular Component	organelle(GO:0043226)	1
OG0030468	Cellular Component	cell part(GO:0044464)	1
OG0030468	Cellular Component	cell(GO:0005623)	1
OG0030468	Cellular Component	organelle(GO:0043226)	1
OG0030476	Cellular Component	cell part(GO:0044464)	1
OG0030476	Cellular Component	cell(GO:0005623)	1
OG0030476	Cellular Component	organelle(GO:0043226)	1
OG0030490	Cellular Component	cell part(GO:0044464)	1
OG0030490	Cellular Component	cell(GO:0005623)	1
OG0030490	Cellular Component	membrane(GO:0016020)	1
OG0030490	Cellular Component	organelle(GO:0043226)	1
OG0030496	Cellular Component	cell junction(GO:0030054)	1
OG0030496	Cellular Component	cell part(GO:0044464)	1
OG0030496	Cellular Component	cell(GO:0005623)	1
OG0030496	Cellular Component	membrane(GO:0016020)	1
OG0030496	Cellular Component	symplast(GO:0055044)	1
OG0030498	Cellular Component	cell part(GO:0044464)	1
OG0030498	Cellular Component	cell(GO:0005623)	1
OG0030498	Cellular Component	organelle part(GO:0044422)	1
OG0030498	Cellular Component	organelle(GO:0043226)	1
OG0030498	Cellular Component	protein-containing complex(GO:0032991)	1
OG0030504	Cellular Component	cell part(GO:0044464)	1
OG0030504	Cellular Component	cell(GO:0005623)	1
OG0030524	Cellular Component	cell part(GO:0044464)	1
OG0030524	Cellular Component	cell(GO:0005623)	1
OG0030524	Cellular Component	organelle(GO:0043226)	1
OG0030526	Cellular Component	cell part(GO:0044464)	1
OG0030526	Cellular Component	cell(GO:0005623)	1
OG0030552	Cellular Component	cell part(GO:0044464)	1
OG0030552	Cellular Component	cell(GO:0005623)	1
OG0030552	Cellular Component	organelle(GO:0043226)	1
OG0030555	Cellular Component	cell part(GO:0044464)	1
OG0030555	Cellular Component	cell(GO:0005623)	1
OG0030555	Cellular Component	organelle(GO:0043226)	1
OG0030559	Cellular Component	cell part(GO:0044464)	1
OG0030559	Cellular Component	cell(GO:0005623)	1
OG0030559	Cellular Component	organelle part(GO:0044422)	1
OG0030559	Cellular Component	organelle(GO:0043226)	1
OG0030559	Cellular Component	protein-containing complex(GO:0032991)	1
OG0030559	Cellular Component	supramolecular complex(GO:0099080)	1
OG0030564	Cellular Component	membrane(GO:0016020)	1

OG0030565	Cellular Component	cell part(GO:0044464)	1
OG0030565	Cellular Component	cell(GO:0005623)	1
OG0030565	Cellular Component	membrane(GO:0016020)	1
OG0030565	Cellular Component	organelle part(GO:0044422)	1
OG0030565	Cellular Component	organelle(GO:0043226)	1
OG0030568	Cellular Component	cell part(GO:0044464)	1
OG0030568	Cellular Component	cell(GO:0005623)	1
OG0030568	Cellular Component	membrane(GO:0016020)	1
OG0030568	Cellular Component	organelle part(GO:0044422)	1
OG0030568	Cellular Component	organelle(GO:0043226)	1
OG0030569	Cellular Component	cell part(GO:0044464)	1
OG0030569	Cellular Component	cell(GO:0005623)	1
OG0030569	Cellular Component	membrane(GO:0016020)	1
OG0030577	Cellular Component	cell part(GO:0044464)	1
OG0030577	Cellular Component	cell(GO:0005623)	1
OG0030577	Cellular Component	membrane(GO:0016020)	1
OG0030577	Cellular Component	organelle part(GO:0044422)	1
OG0030577	Cellular Component	organelle(GO:0043226)	1
OG0030578	Cellular Component	cell part(GO:0044464)	1
OG0030578	Cellular Component	cell(GO:0005623)	1
OG0030578	Cellular Component	membrane part(GO:0044425)	1
OG0030578	Cellular Component	membrane(GO:0016020)	1
OG0030582	Cellular Component	cell part(GO:0044464)	1
OG0030582	Cellular Component	cell(GO:0005623)	1
OG0030582	Cellular Component	membrane-enclosed lumen(GO:0031974)	1
OG0030582	Cellular Component	organelle part(GO:0044422)	1
OG0030582	Cellular Component	organelle(GO:0043226)	1
OG0030583	Cellular Component	cell junction(GO:0030054)	1
OG0030583	Cellular Component	cell part(GO:0044464)	1
OG0030583	Cellular Component	cell(GO:0005623)	1
OG0030583	Cellular Component	organelle(GO:0043226)	1
OG0030583	Cellular Component	symplast(GO:0055044)	1
OG0030584	Cellular Component	cell part(GO:0044464)	1
OG0030584	Cellular Component	cell(GO:0005623)	1
OG0030584	Cellular Component	membrane(GO:0016020)	1
OG0030584	Cellular Component	organelle part(GO:0044422)	1
OG0030584	Cellular Component	organelle(GO:0043226)	1
OG0030585	Cellular Component	cell part(GO:0044464)	1
OG0030585	Cellular Component	cell(GO:0005623)	1
OG0030585	Cellular Component	membrane(GO:0016020)	1
OG0030585	Cellular Component	organelle part(GO:0044422)	1
OG0030585	Cellular Component	organelle(GO:0043226)	1
OG0030586	Cellular Component	cell part(GO:0044464)	1
OG0030586	Cellular Component	cell(GO:0005623)	1
OG0030586	Cellular Component	membrane-enclosed lumen(GO:0031974)	1
OG0030586	Cellular Component	organelle part(GO:0044422)	1
OG0030586	Cellular Component	organelle(GO:0043226)	1

OG0030586	Cellular Component	protein-containing complex(GO:0032991)	1
OG0030587	Cellular Component	cell part(GO:0044464)	1
OG0030587	Cellular Component	cell(GO:0005623)	1
OG0030587	Cellular Component	organelle(GO:0043226)	1
OG0030588	Cellular Component	cell part(GO:0044464)	1
OG0030588	Cellular Component	cell(GO:0005623)	1
OG0030588	Cellular Component	organelle(GO:0043226)	1
OG0030589	Cellular Component	extracellular region(GO:0005576)	1
OG0030590	Cellular Component	cell part(GO:0044464)	1
OG0030590	Cellular Component	cell(GO:0005623)	1
OG0030590	Cellular Component	membrane(GO:0016020)	1
OG0030593	Cellular Component	cell part(GO:0044464)	1
OG0030593	Cellular Component	cell(GO:0005623)	1
OG0030593	Cellular Component	membrane(GO:0016020)	1
OG0030594	Cellular Component	cell part(GO:0044464)	1
OG0030594	Cellular Component	cell(GO:0005623)	1
OG0030594	Cellular Component	membrane(GO:0016020)	1
OG0030596	Cellular Component	cell junction(GO:0030054)	1
OG0030596	Cellular Component	cell part(GO:0044464)	1
OG0030596	Cellular Component	cell(GO:0005623)	1
OG0030596	Cellular Component	extracellular region part(GO:0044421)	1
OG0030596	Cellular Component	extracellular region(GO:0005576)	1
OG0030596	Cellular Component	membrane(GO:0016020)	1
OG0030596	Cellular Component	membrane-enclosed lumen(GO:0031974)	1
OG0030596	Cellular Component	organelle part(GO:0044422)	1
OG0030596	Cellular Component	organelle(GO:0043226)	1
OG0030596	Cellular Component	protein-containing complex(GO:0032991)	1
OG0030596	Cellular Component	symplast(GO:0055044)	1
OG0030604	Cellular Component	cell junction(GO:0030054)	1
OG0030604	Cellular Component	cell part(GO:0044464)	1
OG0030604	Cellular Component	cell(GO:0005623)	1
OG0030604	Cellular Component	extracellular region(GO:0005576)	1
OG0030604	Cellular Component	membrane(GO:0016020)	1
OG0030604	Cellular Component	organelle(GO:0043226)	1
OG0030604	Cellular Component	symplast(GO:0055044)	1
OG0030605	Cellular Component	cell part(GO:0044464)	1
OG0030605	Cellular Component	cell(GO:0005623)	1
OG0030605	Cellular Component	membrane part(GO:0044425)	1
OG0030605	Cellular Component	membrane(GO:0016020)	1
OG0030605	Cellular Component	organelle part(GO:0044422)	1
OG0030605	Cellular Component	organelle(GO:0043226)	1
OG0030605	Cellular Component	protein-containing complex(GO:0032991)	1
OG0030607	Cellular Component	cell part(GO:0044464)	1
OG0030607	Cellular Component	cell(GO:0005623)	1
OG0030607	Cellular Component	membrane(GO:0016020)	1
OG0030608	Cellular Component	cell part(GO:0044464)	1

OG0030608	Cellular Component	cell(GO:0005623)	1
OG0030608	Cellular Component	membrane(GO:0016020)	1
OG0030609	Cellular Component	cell part(GO:0044464)	1
OG0030609	Cellular Component	cell(GO:0005623)	1
OG0030609	Cellular Component	organelle(GO:0043226)	1
OG0030613	Cellular Component	cell part(GO:0044464)	1
OG0030613	Cellular Component	cell(GO:0005623)	1
OG0030613	Cellular Component	organelle(GO:0043226)	1
OG0030620	Cellular Component	cell part(GO:0044464)	1
OG0030620	Cellular Component	cell(GO:0005623)	1
OG0030620	Cellular Component	membrane part(GO:0044425)	1
OG0030620	Cellular Component	membrane(GO:0016020)	1
OG0030621	Cellular Component	cell part(GO:0044464)	1
OG0030621	Cellular Component	cell(GO:0005623)	1
OG0030621	Cellular Component	organelle part(GO:0044422)	1
OG0030621	Cellular Component	organelle(GO:0043226)	1
OG0030622	Cellular Component	cell part(GO:0044464)	1
OG0030622	Cellular Component	cell(GO:0005623)	1
OG0030622	Cellular Component	organelle(GO:0043226)	1
OG0030623	Cellular Component	cell part(GO:0044464)	1
OG0030623	Cellular Component	cell(GO:0005623)	1
OG0030623	Cellular Component	membrane part(GO:0044425)	1
OG0030623	Cellular Component	membrane(GO:0016020)	1
OG0030623	Cellular Component	organelle part(GO:0044422)	1
OG0030623	Cellular Component	organelle(GO:0043226)	1
OG0030624	Cellular Component	cell part(GO:0044464)	1
OG0030624	Cellular Component	cell(GO:0005623)	1
OG0030624	Cellular Component	organelle(GO:0043226)	1
OG0030625	Cellular Component	cell part(GO:0044464)	1
OG0030625	Cellular Component	cell(GO:0005623)	1
OG0030625	Cellular Component	membrane part(GO:0044425)	1
OG0030625	Cellular Component	membrane(GO:0016020)	1
OG0030625	Cellular Component	organelle part(GO:0044422)	1
OG0030625	Cellular Component	organelle(GO:0043226)	1
OG0030629	Cellular Component	cell part(GO:0044464)	1
OG0030629	Cellular Component	cell(GO:0005623)	1
OG0030629	Cellular Component	membrane(GO:0016020)	1
OG0030629	Cellular Component	organelle part(GO:0044422)	1
OG0030629	Cellular Component	organelle(GO:0043226)	1
OG0030630	Cellular Component	cell part(GO:0044464)	1
OG0030630	Cellular Component	cell(GO:0005623)	1
OG0030630	Cellular Component	organelle part(GO:0044422)	1
OG0030630	Cellular Component	organelle(GO:0043226)	1
OG0030635	Cellular Component	cell part(GO:0044464)	1
OG0030635	Cellular Component	cell(GO:0005623)	1
OG0030635	Cellular Component	organelle(GO:0043226)	1
OG0030642	Cellular Component	cell part(GO:0044464)	1

OG0030642	Cellular Component	cell(GO:0005623)	1
OG0030642	Cellular Component	organelle(GO:0043226)	1
OG0030643	Cellular Component	cell part(GO:0044464)	1
OG0030643	Cellular Component	cell(GO:0005623)	1
OG0030643	Cellular Component	organelle(GO:0043226)	1
OG0030647	Cellular Component	cell part(GO:0044464)	1
OG0030647	Cellular Component	cell(GO:0005623)	1
OG0030647	Cellular Component	organelle part(GO:0044422)	1
OG0030647	Cellular Component	organelle(GO:0043226)	1
OG0030658	Cellular Component	cell part(GO:0044464)	1
OG0030658	Cellular Component	cell(GO:0005623)	1
OG0030658	Cellular Component	organelle(GO:0043226)	1
OG0030660	Cellular Component	cell part(GO:0044464)	1
OG0030660	Cellular Component	cell(GO:0005623)	1
OG0030660	Cellular Component	organelle(GO:0043226)	1
OG0030661	Cellular Component	cell part(GO:0044464)	1
OG0030661	Cellular Component	cell(GO:0005623)	1
OG0030661	Cellular Component	organelle(GO:0043226)	1
OG0030665	Cellular Component	cell part(GO:0044464)	1
OG0030665	Cellular Component	cell(GO:0005623)	1
OG0030665	Cellular Component	membrane(GO:0016020)	1
OG0030666	Cellular Component	cell junction(GO:0030054)	1
OG0030666	Cellular Component	cell part(GO:0044464)	1
OG0030666	Cellular Component	cell(GO:0005623)	1
OG0030666	Cellular Component	membrane(GO:0016020)	1
OG0030666	Cellular Component	symplast(GO:0055044)	1
OG0030667	Cellular Component	cell junction(GO:0030054)	1
OG0030667	Cellular Component	cell part(GO:0044464)	1
OG0030667	Cellular Component	cell(GO:0005623)	1
OG0030667	Cellular Component	membrane(GO:0016020)	1
OG0030667	Cellular Component	symplast(GO:0055044)	1
OG0030669	Cellular Component	cell part(GO:0044464)	1
OG0030669	Cellular Component	cell(GO:0005623)	1
OG0030669	Cellular Component	membrane part(GO:0044425)	1
OG0030669	Cellular Component	membrane(GO:0016020)	1
OG0030669	Cellular Component	organelle part(GO:0044422)	1
OG0030669	Cellular Component	organelle(GO:0043226)	1
OG0030670	Cellular Component	cell part(GO:0044464)	1
OG0030670	Cellular Component	cell(GO:0005623)	1
OG0030670	Cellular Component	membrane part(GO:0044425)	1
OG0030670	Cellular Component	membrane(GO:0016020)	1
OG0030670	Cellular Component	organelle part(GO:0044422)	1
OG0030670	Cellular Component	organelle(GO:0043226)	1
OG0030670	Cellular Component	protein-containing complex(GO:0032991)	1
OG0030680	Cellular Component	cell part(GO:0044464)	1
OG0030680	Cellular Component	cell(GO:0005623)	1
OG0030680	Cellular Component	organelle(GO:0043226)	1

OG0030682	Cellular Component	cell part(GO:0044464)	1
OG0030682	Cellular Component	cell(GO:0005623)	1
OG0030682	Cellular Component	membrane(GO:0016020)	1
OG0030687	Cellular Component	cell part(GO:0044464)	1
OG0030687	Cellular Component	cell(GO:0005623)	1
OG0030687	Cellular Component	organelle(GO:0043226)	1
OG0030689	Cellular Component	cell part(GO:0044464)	1
OG0030689	Cellular Component	cell(GO:0005623)	1
OG0030689	Cellular Component	organelle(GO:0043226)	1
OG0030697	Cellular Component	cell part(GO:0044464)	1
OG0030697	Cellular Component	cell(GO:0005623)	1
OG0030697	Cellular Component	membrane(GO:0016020)	1
OG0030697	Cellular Component	organelle part(GO:0044422)	1
OG0030697	Cellular Component	organelle(GO:0043226)	1
OG0030698	Cellular Component	cell part(GO:0044464)	1
OG0030698	Cellular Component	cell(GO:0005623)	1
OG0030698	Cellular Component	organelle(GO:0043226)	1
OG0030700	Cellular Component	cell part(GO:0044464)	1
OG0030700	Cellular Component	cell(GO:0005623)	1
OG0030703	Cellular Component	cell part(GO:0044464)	1
OG0030703	Cellular Component	cell(GO:0005623)	1
OG0030703	Cellular Component	membrane(GO:0016020)	1
OG0030703	Cellular Component	organelle part(GO:0044422)	1
OG0030703	Cellular Component	organelle(GO:0043226)	1
OG0030704	Cellular Component	cell junction(GO:0030054)	1
OG0030704	Cellular Component	cell part(GO:0044464)	1
OG0030704	Cellular Component	cell(GO:0005623)	1
OG0030704	Cellular Component	extracellular region(GO:0005576)	1
OG0030704	Cellular Component	membrane(GO:0016020)	1
OG0030704	Cellular Component	organelle part(GO:0044422)	1
OG0030704	Cellular Component	organelle(GO:0043226)	1
OG0030704	Cellular Component	supramolecular complex(GO:0099080)	1
OG0030704	Cellular Component	symplast(GO:0055044)	1
OG0030706	Cellular Component	cell part(GO:0044464)	1
OG0030706	Cellular Component	cell(GO:0005623)	1
OG0030706	Cellular Component	organelle part(GO:0044422)	1
OG0030706	Cellular Component	organelle(GO:0043226)	1
OG0030707	Cellular Component	cell part(GO:0044464)	1
OG0030707	Cellular Component	cell(GO:0005623)	1
OG0030707	Cellular Component	membrane(GO:0016020)	1
OG0030708	Cellular Component	cell part(GO:0044464)	1
OG0030708	Cellular Component	cell(GO:0005623)	1
OG0030708	Cellular Component	organelle part(GO:0044422)	1
OG0030708	Cellular Component	organelle(GO:0043226)	1
OG0030709	Cellular Component	cell part(GO:0044464)	1
OG0030709	Cellular Component	cell(GO:0005623)	1
OG0030709	Cellular Component	organelle(GO:0043226)	1

OG0030710	Cellular Component	cell part(GO:0044464)	1
OG0030710	Cellular Component	cell(GO:0005623)	1
OG0030710	Cellular Component	organelle(GO:0043226)	1
OG0030712	Cellular Component	cell part(GO:0044464)	1
OG0030712	Cellular Component	cell(GO:0005623)	1
OG0030712	Cellular Component	membrane(GO:0016020)	1
OG0030712	Cellular Component	membrane-enclosed lumen(GO:0031974)	1
OG0030712	Cellular Component	organelle part(GO:0044422)	1
OG0030712	Cellular Component	organelle(GO:0043226)	1
OG0030712	Cellular Component	protein-containing complex(GO:0032991)	1
OG0030719	Cellular Component	cell part(GO:0044464)	1
OG0030719	Cellular Component	cell(GO:0005623)	1
OG0030719	Cellular Component	organelle(GO:0043226)	1
OG0030726	Cellular Component	cell part(GO:0044464)	1
OG0030726	Cellular Component	cell(GO:0005623)	1
OG0030726	Cellular Component	organelle(GO:0043226)	1
OG0030730	Cellular Component	cell part(GO:0044464)	1
OG0030730	Cellular Component	cell(GO:0005623)	1
OG0030730	Cellular Component	extracellular region(GO:0005576)	1
OG0030730	Cellular Component	membrane(GO:0016020)	1
OG0030730	Cellular Component	organelle(GO:0043226)	1
OG0030732	Cellular Component	cell part(GO:0044464)	1
OG0030732	Cellular Component	cell(GO:0005623)	1
OG0030732	Cellular Component	organelle(GO:0043226)	1
OG0030743	Cellular Component	cell part(GO:0044464)	1
OG0030743	Cellular Component	cell(GO:0005623)	1
OG0030743	Cellular Component	membrane part(GO:0044425)	1
OG0030743	Cellular Component	membrane(GO:0016020)	1
OG0030746	Cellular Component	cell part(GO:0044464)	1
OG0030746	Cellular Component	cell(GO:0005623)	1
OG0030746	Cellular Component	membrane-enclosed lumen(GO:0031974)	1
OG0030746	Cellular Component	organelle part(GO:0044422)	1
OG0030746	Cellular Component	organelle(GO:0043226)	1
OG0030753	Cellular Component	cell part(GO:0044464)	1
OG0030753	Cellular Component	cell(GO:0005623)	1
OG0030753	Cellular Component	membrane part(GO:0044425)	1
OG0030753	Cellular Component	membrane(GO:0016020)	1
OG0030753	Cellular Component	organelle part(GO:0044422)	1
OG0030753	Cellular Component	organelle(GO:0043226)	1
OG0030757	Cellular Component	cell part(GO:0044464)	1
OG0030757	Cellular Component	cell(GO:0005623)	1
OG0030757	Cellular Component	extracellular region(GO:0005576)	1
OG0030763	Cellular Component	cell part(GO:0044464)	1
OG0030763	Cellular Component	cell(GO:0005623)	1
OG0030763	Cellular Component	membrane(GO:0016020)	1
OG0030763	Cellular Component	organelle(GO:0043226)	1
OG0030766	Cellular Component	cell part(GO:0044464)	1

OG0030766	Cellular Component	cell(GO:0005623)	1
OG0030766	Cellular Component	organelle(GO:0043226)	1
OG0030767	Cellular Component	cell part(GO:0044464)	1
OG0030767	Cellular Component	cell(GO:0005623)	1
OG0030767	Cellular Component	organelle(GO:0043226)	1
OG0030774	Cellular Component	cell part(GO:0044464)	1
OG0030774	Cellular Component	cell(GO:0005623)	1
OG0030774	Cellular Component	organelle part(GO:0044422)	1
OG0030774	Cellular Component	organelle(GO:0043226)	1
OG0030774	Cellular Component	protein-containing complex(GO:0032991)	1
OG0030776	Cellular Component	cell part(GO:0044464)	1
OG0030776	Cellular Component	cell(GO:0005623)	1
OG0030776	Cellular Component	organelle part(GO:0044422)	1
OG0030776	Cellular Component	organelle(GO:0043226)	1
OG0030776	Cellular Component	protein-containing complex(GO:0032991)	1
OG0030781	Cellular Component	cell part(GO:0044464)	1
OG0030781	Cellular Component	cell(GO:0005623)	1
OG0030781	Cellular Component	membrane(GO:0016020)	1
OG0030781	Cellular Component	organelle part(GO:0044422)	1
OG0030781	Cellular Component	organelle(GO:0043226)	1
OG0030788	Cellular Component	cell junction(GO:0030054)	1
OG0030788	Cellular Component	cell part(GO:0044464)	1
OG0030788	Cellular Component	cell(GO:0005623)	1
OG0030788	Cellular Component	membrane(GO:0016020)	1
OG0030788	Cellular Component	organelle part(GO:0044422)	1
OG0030788	Cellular Component	organelle(GO:0043226)	1
OG0030788	Cellular Component	protein-containing complex(GO:0032991)	1
OG0030788	Cellular Component	symplast(GO:0055044)	1
OG0030789	Cellular Component	cell part(GO:0044464)	1
OG0030789	Cellular Component	cell(GO:0005623)	1
OG0030789	Cellular Component	membrane(GO:0016020)	1
OG0030791	Cellular Component	cell part(GO:0044464)	1
OG0030791	Cellular Component	cell(GO:0005623)	1
OG0030794	Cellular Component	cell part(GO:0044464)	1
OG0030794	Cellular Component	cell(GO:0005623)	1
OG0030796	Cellular Component	cell part(GO:0044464)	1
OG0030796	Cellular Component	cell(GO:0005623)	1
OG0030797	Cellular Component	cell part(GO:0044464)	1
OG0030797	Cellular Component	cell(GO:0005623)	1
OG0030797	Cellular Component	organelle(GO:0043226)	1
OG0030798	Cellular Component	cell part(GO:0044464)	1
OG0030798	Cellular Component	cell(GO:0005623)	1
OG0030798	Cellular Component	membrane(GO:0016020)	1
OG0030798	Cellular Component	organelle(GO:0043226)	1
OG0030806	Cellular Component	membrane(GO:0016020)	1
OG0030817	Cellular Component	cell part(GO:0044464)	1

OG0030817	Cellular Component	cell(GO:0005623)	1
OG0030817	Cellular Component	membrane(GO:0016020)	1
OG0030818	Cellular Component	membrane part(GO:0044425)	1
OG0030818	Cellular Component	membrane(GO:0016020)	1
OG0030820	Cellular Component	cell part(GO:0044464)	1
OG0030820	Cellular Component	cell(GO:0005623)	1
OG0030852	Cellular Component	cell part(GO:0044464)	1
OG0030852	Cellular Component	cell(GO:0005623)	1
OG0030852	Cellular Component	extracellular region part(GO:0044421)	1
OG0030852	Cellular Component	extracellular region(GO:0005576)	1
OG0030852	Cellular Component	membrane-enclosed lumen(GO:0031974)	1
OG0030852	Cellular Component	organelle part(GO:0044422)	1
OG0030852	Cellular Component	organelle(GO:0043226)	1
OG0030852	Cellular Component	protein-containing complex(GO:0032991)	1
OG0030852	Cellular Component	supramolecular complex(GO:0099080)	1
OG0030853	Cellular Component	cell part(GO:0044464)	1
OG0030853	Cellular Component	cell(GO:0005623)	1
OG0030856	Cellular Component	cell part(GO:0044464)	1
OG0030856	Cellular Component	cell(GO:0005623)	1
OG0030859	Cellular Component	cell part(GO:0044464)	1
OG0030859	Cellular Component	cell(GO:0005623)	1
OG0030861	Cellular Component	cell part(GO:0044464)	1
OG0030861	Cellular Component	cell(GO:0005623)	1
OG0030864	Cellular Component	cell part(GO:0044464)	1
OG0030864	Cellular Component	cell(GO:0005623)	1
OG0030864	Cellular Component	protein-containing complex(GO:0032991)	1
OG0030865	Cellular Component	cell part(GO:0044464)	1
OG0030865	Cellular Component	cell(GO:0005623)	1
OG0030865	Cellular Component	membrane(GO:0016020)	1
OG0030868	Cellular Component	cell part(GO:0044464)	1
OG0030868	Cellular Component	cell(GO:0005623)	1
OG0030868	Cellular Component	membrane(GO:0016020)	1
OG0030869	Cellular Component	cell part(GO:0044464)	1
OG0030869	Cellular Component	cell(GO:0005623)	1
OG0030870	Cellular Component	cell part(GO:0044464)	1
OG0030870	Cellular Component	cell(GO:0005623)	1
OG0030870	Cellular Component	membrane(GO:0016020)	1
OG0030873	Cellular Component	cell part(GO:0044464)	1
OG0030873	Cellular Component	cell(GO:0005623)	1
OG0030873	Cellular Component	membrane(GO:0016020)	1
OG0030875	Cellular Component	cell part(GO:0044464)	1
OG0030875	Cellular Component	cell(GO:0005623)	1
OG0030876	Cellular Component	cell part(GO:0044464)	1
OG0030876	Cellular Component	cell(GO:0005623)	1
OG0030876	Cellular Component	membrane(GO:0016020)	1
OG0030879	Cellular Component	cell part(GO:0044464)	1

OG0030879	Cellular Component	cell(GO:0005623)	1
OG0030879	Cellular Component	membrane(GO:0016020)	1
OG0030882	Cellular Component	cell part(GO:0044464)	1
OG0030882	Cellular Component	cell(GO:0005623)	1
OG0030882	Cellular Component	membrane(GO:0016020)	1
OG0030885	Cellular Component	cell part(GO:0044464)	1
OG0030885	Cellular Component	cell(GO:0005623)	1
OG0030885	Cellular Component	membrane(GO:0016020)	1
OG0030887	Cellular Component	cell part(GO:0044464)	1
OG0030887	Cellular Component	cell(GO:0005623)	1
OG0030887	Cellular Component	membrane(GO:0016020)	1
OG0030892	Cellular Component	cell part(GO:0044464)	1
OG0030892	Cellular Component	cell(GO:0005623)	1
OG0030893	Cellular Component	cell part(GO:0044464)	1
OG0030893	Cellular Component	cell(GO:0005623)	1
OG0030893	Cellular Component	membrane(GO:0016020)	1
OG0030896	Cellular Component	cell part(GO:0044464)	1
OG0030896	Cellular Component	cell(GO:0005623)	1
OG0030896	Cellular Component	membrane(GO:0016020)	1
OG0030897	Cellular Component	cell part(GO:0044464)	1
OG0030897	Cellular Component	cell(GO:0005623)	1
OG0030897	Cellular Component	membrane(GO:0016020)	1
OG0030898	Cellular Component	cell part(GO:0044464)	1
OG0030898	Cellular Component	cell(GO:0005623)	1
OG0030898	Cellular Component	membrane(GO:0016020)	1
OG0030905	Cellular Component	cell part(GO:0044464)	1
OG0030905	Cellular Component	cell(GO:0005623)	1
OG0030913	Cellular Component	cell part(GO:0044464)	1
OG0030913	Cellular Component	cell(GO:0005623)	1
OG0030913	Cellular Component	membrane(GO:0016020)	1
OG0030915	Cellular Component	cell part(GO:0044464)	1
OG0030915	Cellular Component	cell(GO:0005623)	1
OG0030915	Cellular Component	membrane(GO:0016020)	1
OG0030926	Cellular Component	cell part(GO:0044464)	1
OG0030926	Cellular Component	cell(GO:0005623)	1
OG0030928	Cellular Component	membrane part(GO:0044425)	1
OG0030928	Cellular Component	membrane(GO:0016020)	1
OG0030931	Cellular Component	cell part(GO:0044464)	1
OG0030931	Cellular Component	cell(GO:0005623)	1
OG0030931	Cellular Component	membrane(GO:0016020)	1
OG0030933	Cellular Component	cell part(GO:0044464)	1
OG0030933	Cellular Component	cell(GO:0005623)	1
OG0030933	Cellular Component	membrane(GO:0016020)	1
OG0030934	Cellular Component	cell part(GO:0044464)	1
OG0030934	Cellular Component	cell(GO:0005623)	1
OG0030934	Cellular Component	membrane(GO:0016020)	1
OG0030936	Cellular Component	cell part(GO:0044464)	1

OG0030936	Cellular Component	cell(GO:0005623)	1
OG0030937	Cellular Component	cell part(GO:0044464)	1
OG0030937	Cellular Component	cell(GO:0005623)	1
OG0030939	Cellular Component	cell part(GO:0044464)	1
OG0030939	Cellular Component	cell(GO:0005623)	1
OG0030940	Cellular Component	cell part(GO:0044464)	1
OG0030940	Cellular Component	cell(GO:0005623)	1
OG0030941	Cellular Component	cell part(GO:0044464)	1
OG0030941	Cellular Component	cell(GO:0005623)	1
OG0030941	Cellular Component	membrane(GO:0016020)	1
OG0030942	Cellular Component	cell part(GO:0044464)	1
OG0030942	Cellular Component	cell(GO:0005623)	1
OG0030942	Cellular Component	membrane(GO:0016020)	1
OG0030944	Cellular Component	cell part(GO:0044464)	1
OG0030944	Cellular Component	cell(GO:0005623)	1
OG0030944	Cellular Component	membrane(GO:0016020)	1
OG0030945	Cellular Component	cell part(GO:0044464)	1
OG0030945	Cellular Component	cell(GO:0005623)	1
OG0030945	Cellular Component	membrane(GO:0016020)	1
OG0030946	Cellular Component	cell part(GO:0044464)	1
OG0030946	Cellular Component	cell(GO:0005623)	1
OG0030946	Cellular Component	membrane(GO:0016020)	1
OG0030950	Cellular Component	cell part(GO:0044464)	1
OG0030950	Cellular Component	cell(GO:0005623)	1
OG0030950	Cellular Component	membrane(GO:0016020)	1
OG0030951	Cellular Component	cell part(GO:0044464)	1
OG0030951	Cellular Component	cell(GO:0005623)	1
OG0030951	Cellular Component	protein-containing complex(GO:0032991)	1
OG0030952	Cellular Component	cell part(GO:0044464)	1
OG0030952	Cellular Component	cell(GO:0005623)	1
OG0030953	Cellular Component	cell part(GO:0044464)	1
OG0030953	Cellular Component	cell(GO:0005623)	1
OG0030953	Cellular Component	membrane(GO:0016020)	1
OG0030955	Cellular Component	cell part(GO:0044464)	1
OG0030955	Cellular Component	cell(GO:0005623)	1
OG0030955	Cellular Component	protein-containing complex(GO:0032991)	1
OG0030958	Cellular Component	cell part(GO:0044464)	1
OG0030958	Cellular Component	cell(GO:0005623)	1
OG0030963	Cellular Component	cell part(GO:0044464)	1
OG0030963	Cellular Component	cell(GO:0005623)	1
OG0030964	Cellular Component	cell part(GO:0044464)	1
OG0030964	Cellular Component	cell(GO:0005623)	1
OG0030964	Cellular Component	membrane(GO:0016020)	1
OG0030969	Cellular Component	cell part(GO:0044464)	1
OG0030969	Cellular Component	cell(GO:0005623)	1
OG0030969	Cellular Component	membrane(GO:0016020)	1
OG0030971	Cellular Component	cell part(GO:0044464)	1

OG0030971	Cellular Component	cell(GO:0005623)	1
OG0030971	Cellular Component	nucleoid(GO:0009295)	1
OG0030972	Cellular Component	cell part(GO:0044464)	1
OG0030972	Cellular Component	cell(GO:0005623)	1
OG0030972	Cellular Component	membrane(GO:0016020)	1
OG0030974	Cellular Component	cell part(GO:0044464)	1
OG0030974	Cellular Component	cell(GO:0005623)	1
OG0030974	Cellular Component	membrane(GO:0016020)	1
OG0030975	Cellular Component	cell part(GO:0044464)	1
OG0030975	Cellular Component	cell(GO:0005623)	1
OG0030975	Cellular Component	membrane part(GO:0044425)	1
OG0030975	Cellular Component	membrane(GO:0016020)	1
OG0030977	Cellular Component	cell part(GO:0044464)	1
OG0030977	Cellular Component	cell(GO:0005623)	1
OG0030977	Cellular Component	membrane part(GO:0044425)	1
OG0030977	Cellular Component	membrane(GO:0016020)	1
OG0030977	Cellular Component	organelle part(GO:0044422)	1
OG0030977	Cellular Component	organelle(GO:0043226)	1
OG0030977	Cellular Component	protein-containing complex(GO:0032991)	1
OG0030979	Cellular Component	cell part(GO:0044464)	1
OG0030979	Cellular Component	cell(GO:0005623)	1
OG0030979	Cellular Component	membrane(GO:0016020)	1
OG0030980	Cellular Component	cell part(GO:0044464)	1
OG0030980	Cellular Component	cell(GO:0005623)	1
OG0030982	Cellular Component	cell part(GO:0044464)	1
OG0030982	Cellular Component	cell(GO:0005623)	1
OG0030982	Cellular Component	membrane(GO:0016020)	1
OG0030984	Cellular Component	cell part(GO:0044464)	1
OG0030984	Cellular Component	cell(GO:0005623)	1
OG0030984	Cellular Component	membrane(GO:0016020)	1
OG0030987	Cellular Component	cell part(GO:0044464)	1
OG0030987	Cellular Component	cell(GO:0005623)	1
OG0030997	Cellular Component	cell part(GO:0044464)	1
OG0030997	Cellular Component	cell(GO:0005623)	1
OG0030997	Cellular Component	organelle(GO:0043226)	1
OG0030998	Cellular Component	cell part(GO:0044464)	1
OG0030998	Cellular Component	cell(GO:0005623)	1
OG0030998	Cellular Component	organelle(GO:0043226)	1
OG0031015	Cellular Component	cell part(GO:0044464)	1
OG0031015	Cellular Component	cell(GO:0005623)	1
OG0031015	Cellular Component	organelle(GO:0043226)	1
OG0031016	Cellular Component	cell part(GO:0044464)	1
OG0031016	Cellular Component	cell(GO:0005623)	1
OG0031017	Cellular Component	cell part(GO:0044464)	1
OG0031017	Cellular Component	cell(GO:0005623)	1
OG0031017	Cellular Component	organelle part(GO:0044422)	1
OG0031017	Cellular Component	organelle(GO:0043226)	1

OG0031017	Cellular Component	protein-containing complex(GO:0032991)	1
OG0031017	Cellular Component	supramolecular complex(GO:0099080)	1
OG0031019	Cellular Component	cell part(GO:0044464)	1
OG0031019	Cellular Component	cell(GO:0005623)	1
OG0031019	Cellular Component	organelle(GO:0043226)	1
OG0031024	Cellular Component	cell part(GO:0044464)	1
OG0031024	Cellular Component	cell(GO:0005623)	1
OG0031024	Cellular Component	organelle part(GO:0044422)	1
OG0031024	Cellular Component	organelle(GO:0043226)	1
OG0031024	Cellular Component	protein-containing complex(GO:0032991)	1
OG0031027	Cellular Component	cell junction(GO:0030054)	1
OG0031027	Cellular Component	cell part(GO:0044464)	1
OG0031027	Cellular Component	cell(GO:0005623)	1
OG0031027	Cellular Component	symplast(GO:0055044)	1
OG0031031	Cellular Component	cell part(GO:0044464)	1
OG0031031	Cellular Component	cell(GO:0005623)	1
OG0031031	Cellular Component	organelle part(GO:0044422)	1
OG0031031	Cellular Component	organelle(GO:0043226)	1
OG0031059	Cellular Component	membrane(GO:0016020)	1
OG0031060	Cellular Component	cell part(GO:0044464)	1
OG0031060	Cellular Component	cell(GO:0005623)	1
OG0031061	Cellular Component	cell part(GO:0044464)	1
OG0031061	Cellular Component	cell(GO:0005623)	1
OG0031061	Cellular Component	membrane-enclosed lumen(GO:0031974)	1
OG0031061	Cellular Component	organelle part(GO:0044422)	1
OG0031061	Cellular Component	organelle(GO:0043226)	1
OG0031061	Cellular Component	protein-containing complex(GO:0032991)	1
OG0031063	Cellular Component	cell part(GO:0044464)	1
OG0031063	Cellular Component	cell(GO:0005623)	1
OG0031063	Cellular Component	extracellular region(GO:0005576)	1
OG0031063	Cellular Component	organelle(GO:0043226)	1
OG0031063	Cellular Component	protein-containing complex(GO:0032991)	1
OG0031067	Cellular Component	cell part(GO:0044464)	1
OG0031067	Cellular Component	cell(GO:0005623)	1
OG0031067	Cellular Component	organelle(GO:0043226)	1
OG0031069	Cellular Component	cell part(GO:0044464)	1
OG0031069	Cellular Component	cell(GO:0005623)	1
OG0031069	Cellular Component	membrane(GO:0016020)	1
OG0031069	Cellular Component	organelle part(GO:0044422)	1
OG0031069	Cellular Component	organelle(GO:0043226)	1
OG0031070	Cellular Component	cell part(GO:0044464)	1
OG0031070	Cellular Component	cell(GO:0005623)	1
OG0031070	Cellular Component	organelle(GO:0043226)	1
OG0031072	Cellular Component	cell part(GO:0044464)	1
OG0031072	Cellular Component	cell(GO:0005623)	1
OG0031072	Cellular Component	extracellular region(GO:0005576)	1

OG0031072	Cellular Component	membrane part(GO:0044425)	1
OG0031072	Cellular Component	membrane(GO:0016020)	1
OG0031077	Cellular Component	cell part(GO:0044464)	1
OG0031077	Cellular Component	cell(GO:0005623)	1
OG0031077	Cellular Component	organelle(GO:0043226)	1
OG0031079	Cellular Component	cell part(GO:0044464)	1
OG0031079	Cellular Component	cell(GO:0005623)	1
OG0031079	Cellular Component	organelle(GO:0043226)	1
OG0031080	Cellular Component	cell part(GO:0044464)	1
OG0031080	Cellular Component	cell(GO:0005623)	1
OG0031080	Cellular Component	organelle(GO:0043226)	1
OG0031084	Cellular Component	cell part(GO:0044464)	1
OG0031084	Cellular Component	cell(GO:0005623)	1
OG0031084	Cellular Component	organelle(GO:0043226)	1
OG0031085	Cellular Component	cell part(GO:0044464)	1
OG0031085	Cellular Component	cell(GO:0005623)	1
OG0031085	Cellular Component	membrane(GO:0016020)	1
OG0031085	Cellular Component	organelle part(GO:0044422)	1
OG0031085	Cellular Component	organelle(GO:0043226)	1
OG0031086	Cellular Component	cell part(GO:0044464)	1
OG0031086	Cellular Component	cell(GO:0005623)	1
OG0031086	Cellular Component	membrane(GO:0016020)	1
OG0031086	Cellular Component	organelle(GO:0043226)	1
OG0031091	Cellular Component	cell part(GO:0044464)	1
OG0031091	Cellular Component	cell(GO:0005623)	1
OG0031092	Cellular Component	cell part(GO:0044464)	1
OG0031092	Cellular Component	cell(GO:0005623)	1
OG0031092	Cellular Component	organelle(GO:0043226)	1
OG0031096	Cellular Component	cell part(GO:0044464)	1
OG0031096	Cellular Component	cell(GO:0005623)	1
OG0031096	Cellular Component	membrane(GO:0016020)	1
OG0031096	Cellular Component	organelle(GO:0043226)	1
OG0031097	Cellular Component	cell part(GO:0044464)	1
OG0031097	Cellular Component	cell(GO:0005623)	1
OG0031097	Cellular Component	organelle part(GO:0044422)	1
OG0031097	Cellular Component	organelle(GO:0043226)	1
OG0031097	Cellular Component	protein-containing complex(GO:0032991)	1
OG0031101	Cellular Component	cell part(GO:0044464)	1
OG0031101	Cellular Component	cell(GO:0005623)	1
OG0031101	Cellular Component	membrane(GO:0016020)	1
OG0031101	Cellular Component	organelle part(GO:0044422)	1
OG0031101	Cellular Component	organelle(GO:0043226)	1
OG0031106	Cellular Component	cell junction(GO:0030054)	1
OG0031106	Cellular Component	cell part(GO:0044464)	1
OG0031106	Cellular Component	cell(GO:0005623)	1
OG0031106	Cellular Component	membrane(GO:0016020)	1
OG0031106	Cellular Component	membrane-enclosed lumen(GO:0031974)	1

OG0031106	Cellular Component	organelle part(GO:0044422)	1
OG0031106	Cellular Component	organelle(GO:0043226)	1
OG0031106	Cellular Component	protein-containing complex(GO:0032991)	1
OG0031106	Cellular Component	symplast(GO:0055044)	1
OG0031107	Cellular Component	cell junction(GO:0030054)	1
OG0031107	Cellular Component	cell part(GO:0044464)	1
OG0031107	Cellular Component	cell(GO:0005623)	1
OG0031107	Cellular Component	membrane(GO:0016020)	1
OG0031107	Cellular Component	symplast(GO:0055044)	1
OG0031111	Cellular Component	cell part(GO:0044464)	1
OG0031111	Cellular Component	cell(GO:0005623)	1
OG0031111	Cellular Component	membrane(GO:0016020)	1
OG0031117	Cellular Component	membrane(GO:0016020)	1
OG0031118	Cellular Component	membrane part(GO:0044425)	1
OG0031118	Cellular Component	membrane(GO:0016020)	1
OG0031120	Cellular Component	cell part(GO:0044464)	1
OG0031120	Cellular Component	cell(GO:0005623)	1
OG0031120	Cellular Component	membrane-enclosed lumen(GO:0031974)	1
OG0031120	Cellular Component	organelle part(GO:0044422)	1
OG0031120	Cellular Component	organelle(GO:0043226)	1
OG0031129	Cellular Component	cell part(GO:0044464)	1
OG0031129	Cellular Component	cell(GO:0005623)	1
OG0031131	Cellular Component	cell part(GO:0044464)	1
OG0031131	Cellular Component	cell(GO:0005623)	1
OG0031131	Cellular Component	membrane(GO:0016020)	1
OG0031135	Cellular Component	cell junction(GO:0030054)	1
OG0031135	Cellular Component	cell part(GO:0044464)	1
OG0031135	Cellular Component	cell(GO:0005623)	1
OG0031135	Cellular Component	membrane part(GO:0044425)	1
OG0031135	Cellular Component	membrane(GO:0016020)	1
OG0031135	Cellular Component	symplast(GO:0055044)	1
OG0031139	Cellular Component	cell part(GO:0044464)	1
OG0031139	Cellular Component	cell(GO:0005623)	1
OG0031139	Cellular Component	membrane(GO:0016020)	1
OG0031139	Cellular Component	membrane-enclosed lumen(GO:0031974)	1
OG0031139	Cellular Component	organelle part(GO:0044422)	1
OG0031139	Cellular Component	organelle(GO:0043226)	1
OG0031139	Cellular Component	protein-containing complex(GO:0032991)	1
OG0031144	Cellular Component	cell part(GO:0044464)	1
OG0031144	Cellular Component	cell(GO:0005623)	1
OG0031150	Cellular Component	cell part(GO:0044464)	1
OG0031150	Cellular Component	cell(GO:0005623)	1
OG0031152	Cellular Component	cell part(GO:0044464)	1
OG0031152	Cellular Component	cell(GO:0005623)	1
OG0031152	Cellular Component	membrane(GO:0016020)	1
OG0031157	Cellular Component	cell part(GO:0044464)	1
OG0031157	Cellular Component	cell(GO:0005623)	1

OG0031157	Cellular Component	membrane(GO:0016020)	1
OG0031158	Cellular Component	cell part(GO:0044464)	1
OG0031158	Cellular Component	cell(GO:0005623)	1
OG0031158	Cellular Component	membrane(GO:0016020)	1
OG0031160	Cellular Component	cell part(GO:0044464)	1
OG0031160	Cellular Component	cell(GO:0005623)	1
OG0031160	Cellular Component	membrane(GO:0016020)	1
OG0031162	Cellular Component	cell part(GO:0044464)	1
OG0031162	Cellular Component	cell(GO:0005623)	1
OG0031162	Cellular Component	membrane part(GO:0044425)	1
OG0031162	Cellular Component	membrane(GO:0016020)	1
OG0031162	Cellular Component	organelle part(GO:0044422)	1
OG0031162	Cellular Component	organelle(GO:0043226)	1
OG0031162	Cellular Component	protein-containing complex(GO:0032991)	1
OG0031169	Cellular Component	cell part(GO:0044464)	1
OG0031169	Cellular Component	cell(GO:0005623)	1
OG0031169	Cellular Component	extracellular region(GO:0005576)	1
OG0031172	Cellular Component	membrane part(GO:0044425)	1
OG0031172	Cellular Component	membrane(GO:0016020)	1
OG0031174	Cellular Component	cell part(GO:0044464)	1
OG0031174	Cellular Component	cell(GO:0005623)	1
OG0031174	Cellular Component	membrane(GO:0016020)	1
OG0031175	Cellular Component	cell part(GO:0044464)	1
OG0031175	Cellular Component	cell(GO:0005623)	1
OG0031175	Cellular Component	extracellular region(GO:0005576)	1
OG0031175	Cellular Component	membrane(GO:0016020)	1
OG0031180	Cellular Component	cell part(GO:0044464)	1
OG0031180	Cellular Component	cell(GO:0005623)	1
OG0031180	Cellular Component	membrane part(GO:0044425)	1
OG0031180	Cellular Component	membrane(GO:0016020)	1
OG0031180	Cellular Component	organelle part(GO:0044422)	1
OG0031180	Cellular Component	organelle(GO:0043226)	1
OG0031180	Cellular Component	protein-containing complex(GO:0032991)	1
OG0031181	Cellular Component	cell part(GO:0044464)	1
OG0031181	Cellular Component	cell(GO:0005623)	1
OG0031189	Cellular Component	cell part(GO:0044464)	1
OG0031189	Cellular Component	cell(GO:0005623)	1
OG0031189	Cellular Component	membrane(GO:0016020)	1
OG0031194	Cellular Component	cell part(GO:0044464)	1
OG0031194	Cellular Component	cell(GO:0005623)	1
OG0031194	Cellular Component	extracellular region(GO:0005576)	1
OG0031194	Cellular Component	membrane part(GO:0044425)	1
OG0031194	Cellular Component	membrane(GO:0016020)	1
OG0031195	Cellular Component	cell part(GO:0044464)	1
OG0031195	Cellular Component	cell(GO:0005623)	1
OG0031196	Cellular Component	cell part(GO:0044464)	1
OG0031196	Cellular Component	cell(GO:0005623)	1

OG0031196	Cellular Component	membrane(GO:0016020)	1
OG0031197	Cellular Component	cell part(GO:0044464)	1
OG0031197	Cellular Component	cell(GO:0005623)	1
OG0031199	Cellular Component	cell part(GO:0044464)	1
OG0031199	Cellular Component	cell(GO:0005623)	1
OG0031199	Cellular Component	membrane(GO:0016020)	1
OG0031200	Cellular Component	cell part(GO:0044464)	1
OG0031200	Cellular Component	cell(GO:0005623)	1
OG0031200	Cellular Component	membrane(GO:0016020)	1
OG0031203	Cellular Component	cell part(GO:0044464)	1
OG0031203	Cellular Component	cell(GO:0005623)	1
OG0031203	Cellular Component	membrane(GO:0016020)	1
OG0031204	Cellular Component	cell part(GO:0044464)	1
OG0031204	Cellular Component	cell(GO:0005623)	1
OG0031204	Cellular Component	membrane(GO:0016020)	1
OG0031209	Cellular Component	cell part(GO:0044464)	1
OG0031209	Cellular Component	cell(GO:0005623)	1
OG0031209	Cellular Component	membrane(GO:0016020)	1
OG0031212	Cellular Component	cell part(GO:0044464)	1
OG0031212	Cellular Component	cell(GO:0005623)	1
OG0031212	Cellular Component	membrane(GO:0016020)	1
OG0031213	Cellular Component	cell part(GO:0044464)	1
OG0031213	Cellular Component	cell(GO:0005623)	1
OG0031213	Cellular Component	membrane(GO:0016020)	1
OG0031214	Cellular Component	cell part(GO:0044464)	1
OG0031214	Cellular Component	cell(GO:0005623)	1
OG0031214	Cellular Component	protein-containing complex(GO:0032991)	1
OG0031217	Cellular Component	cell part(GO:0044464)	1
OG0031217	Cellular Component	cell(GO:0005623)	1
OG0031218	Cellular Component	cell part(GO:0044464)	1
OG0031218	Cellular Component	cell(GO:0005623)	1
OG0031218	Cellular Component	membrane(GO:0016020)	1
OG0031221	Cellular Component	cell part(GO:0044464)	1
OG0031221	Cellular Component	cell(GO:0005623)	1
OG0031221	Cellular Component	membrane(GO:0016020)	1
OG0031222	Cellular Component	cell part(GO:0044464)	1
OG0031222	Cellular Component	cell(GO:0005623)	1
OG0031222	Cellular Component	membrane(GO:0016020)	1
OG0031223	Cellular Component	cell part(GO:0044464)	1
OG0031223	Cellular Component	cell(GO:0005623)	1
OG0031223	Cellular Component	membrane part(GO:0044425)	1
OG0031223	Cellular Component	membrane(GO:0016020)	1
OG0031223	Cellular Component	protein-containing complex(GO:0032991)	1
OG0031224	Cellular Component	cell part(GO:0044464)	1
OG0031224	Cellular Component	cell(GO:0005623)	1
OG0031224	Cellular Component	membrane(GO:0016020)	1
OG0031227	Cellular Component	cell part(GO:0044464)	1

OG0031227	Cellular Component	cell(GO:0005623)	1
OG0031228	Cellular Component	cell part(GO:0044464)	1
OG0031228	Cellular Component	cell(GO:0005623)	1
OG0031228	Cellular Component	extracellular region(GO:0005576)	1
OG0031228	Cellular Component	membrane(GO:0016020)	1
OG0031231	Cellular Component	cell part(GO:0044464)	1
OG0031231	Cellular Component	cell(GO:0005623)	1
OG0031231	Cellular Component	membrane(GO:0016020)	1
OG0031233	Cellular Component	cell part(GO:0044464)	1
OG0031233	Cellular Component	cell(GO:0005623)	1
OG0031234	Cellular Component	cell part(GO:0044464)	1
OG0031234	Cellular Component	cell(GO:0005623)	1
OG0031235	Cellular Component	cell part(GO:0044464)	1
OG0031235	Cellular Component	cell(GO:0005623)	1
OG0031235	Cellular Component	membrane(GO:0016020)	1
OG0031241	Cellular Component	cell part(GO:0044464)	1
OG0031241	Cellular Component	cell(GO:0005623)	1
OG0031241	Cellular Component	protein-containing complex(GO:0032991)	1
OG0031248	Cellular Component	cell part(GO:0044464)	1
OG0031248	Cellular Component	cell(GO:0005623)	1
OG0031248	Cellular Component	protein-containing complex(GO:0032991)	1
OG0031249	Cellular Component	cell part(GO:0044464)	1
OG0031249	Cellular Component	cell(GO:0005623)	1
OG0031249	Cellular Component	membrane(GO:0016020)	1
OG0031250	Cellular Component	membrane part(GO:0044425)	1
OG0031250	Cellular Component	membrane(GO:0016020)	1
OG0031252	Cellular Component	cell part(GO:0044464)	1
OG0031252	Cellular Component	cell(GO:0005623)	1
OG0031254	Cellular Component	cell part(GO:0044464)	1
OG0031254	Cellular Component	cell(GO:0005623)	1
OG0031254	Cellular Component	membrane(GO:0016020)	1
OG0031255	Cellular Component	cell part(GO:0044464)	1
OG0031255	Cellular Component	cell(GO:0005623)	1
OG0031256	Cellular Component	cell part(GO:0044464)	1
OG0031256	Cellular Component	cell(GO:0005623)	1
OG0031257	Cellular Component	cell part(GO:0044464)	1
OG0031257	Cellular Component	cell(GO:0005623)	1
OG0031258	Cellular Component	cell part(GO:0044464)	1
OG0031258	Cellular Component	cell(GO:0005623)	1
OG0031258	Cellular Component	membrane(GO:0016020)	1
OG0031260	Cellular Component	cell part(GO:0044464)	1
OG0031260	Cellular Component	cell(GO:0005623)	1
OG0031263	Cellular Component	cell part(GO:0044464)	1
OG0031263	Cellular Component	cell(GO:0005623)	1
OG0031263	Cellular Component	organelle part(GO:0044422)	1
OG0031263	Cellular Component	organelle(GO:0043226)	1
OG0031267	Cellular Component	cell part(GO:0044464)	1

OG0031267	Cellular Component	cell(GO:0005623)	1
OG0031267	Cellular Component	membrane(GO:0016020)	1
OG0031268	Cellular Component	cell part(GO:0044464)	1
OG0031268	Cellular Component	cell(GO:0005623)	1
OG0031268	Cellular Component	membrane(GO:0016020)	1
OG0031269	Cellular Component	cell part(GO:0044464)	1
OG0031269	Cellular Component	cell(GO:0005623)	1
OG0031269	Cellular Component	membrane(GO:0016020)	1
OG0031270	Cellular Component	cell part(GO:0044464)	1
OG0031270	Cellular Component	cell(GO:0005623)	1
OG0031270	Cellular Component	membrane(GO:0016020)	1
OG0031271	Cellular Component	cell part(GO:0044464)	1
OG0031271	Cellular Component	cell(GO:0005623)	1
OG0031271	Cellular Component	membrane(GO:0016020)	1
OG0031273	Cellular Component	cell part(GO:0044464)	1
OG0031273	Cellular Component	cell(GO:0005623)	1
OG0031273	Cellular Component	membrane(GO:0016020)	1
OG0031277	Cellular Component	cell part(GO:0044464)	1
OG0031277	Cellular Component	cell(GO:0005623)	1
OG0031278	Cellular Component	membrane(GO:0016020)	1
OG0031279	Cellular Component	cell part(GO:0044464)	1
OG0031279	Cellular Component	cell(GO:0005623)	1
OG0031279	Cellular Component	membrane(GO:0016020)	1
OG0031281	Cellular Component	cell part(GO:0044464)	1
OG0031281	Cellular Component	cell(GO:0005623)	1
OG0031281	Cellular Component	organelle part(GO:0044422)	1
OG0031281	Cellular Component	organelle(GO:0043226)	1
OG0031281	Cellular Component	protein-containing complex(GO:0032991)	1
OG0031282	Cellular Component	cell part(GO:0044464)	1
OG0031282	Cellular Component	cell(GO:0005623)	1
OG0031283	Cellular Component	cell part(GO:0044464)	1
OG0031283	Cellular Component	cell(GO:0005623)	1
OG0031283	Cellular Component	membrane(GO:0016020)	1
OG0031292	Cellular Component	cell part(GO:0044464)	1
OG0031292	Cellular Component	cell(GO:0005623)	1
OG0031292	Cellular Component	membrane(GO:0016020)	1
OG0031293	Cellular Component	cell part(GO:0044464)	1
OG0031293	Cellular Component	cell(GO:0005623)	1
OG0031293	Cellular Component	membrane(GO:0016020)	1
OG0031311	Cellular Component	cell part(GO:0044464)	1
OG0031311	Cellular Component	cell(GO:0005623)	1
OG0031311	Cellular Component	organelle(GO:0043226)	1
OG0031313	Cellular Component	cell part(GO:0044464)	1
OG0031313	Cellular Component	cell(GO:0005623)	1
OG0031313	Cellular Component	organelle(GO:0043226)	1
OG0031316	Cellular Component	cell part(GO:0044464)	1
OG0031316	Cellular Component	cell(GO:0005623)	1

OG0031316	Cellular Component	organelle(GO:0043226)	1
OG0031317	Cellular Component	cell junction(GO:0030054)	1
OG0031317	Cellular Component	symplast(GO:0055044)	1
OG0031324	Cellular Component	cell part(GO:0044464)	1
OG0031324	Cellular Component	cell(GO:0005623)	1
OG0031324	Cellular Component	organelle(GO:0043226)	1
OG0031324	Cellular Component	protein-containing complex(GO:0032991)	1
OG0031331	Cellular Component	cell part(GO:0044464)	1
OG0031331	Cellular Component	cell(GO:0005623)	1
OG0031331	Cellular Component	membrane-enclosed lumen(GO:0031974)	1
OG0031331	Cellular Component	organelle part(GO:0044422)	1
OG0031331	Cellular Component	organelle(GO:0043226)	1
OG0031331	Cellular Component	protein-containing complex(GO:0032991)	1
OG0031337	Cellular Component	cell part(GO:0044464)	1
OG0031337	Cellular Component	cell(GO:0005623)	1
OG0031337	Cellular Component	extracellular region(GO:0005576)	1
OG0031337	Cellular Component	membrane(GO:0016020)	1
OG0031338	Cellular Component	cell part(GO:0044464)	1
OG0031338	Cellular Component	cell(GO:0005623)	1
OG0031340	Cellular Component	cell part(GO:0044464)	1
OG0031340	Cellular Component	cell(GO:0005623)	1
OG0031340	Cellular Component	organelle(GO:0043226)	1
OG0031341	Cellular Component	membrane(GO:0016020)	1
OG0031345	Cellular Component	cell part(GO:0044464)	1
OG0031345	Cellular Component	cell(GO:0005623)	1
OG0031345	Cellular Component	organelle(GO:0043226)	1
OG0031349	Cellular Component	cell junction(GO:0030054)	1
OG0031349	Cellular Component	cell part(GO:0044464)	1
OG0031349	Cellular Component	cell(GO:0005623)	1
OG0031349	Cellular Component	organelle(GO:0043226)	1
OG0031349	Cellular Component	symplast(GO:0055044)	1
OG0031352	Cellular Component	cell part(GO:0044464)	1
OG0031352	Cellular Component	cell(GO:0005623)	1
OG0031352	Cellular Component	extracellular region(GO:0005576)	1
OG0031352	Cellular Component	membrane(GO:0016020)	1
OG0031352	Cellular Component	organelle part(GO:0044422)	1
OG0031352	Cellular Component	organelle(GO:0043226)	1
OG0031355	Cellular Component	cell part(GO:0044464)	1
OG0031355	Cellular Component	cell(GO:0005623)	1
OG0031355	Cellular Component	membrane(GO:0016020)	1
OG0031356	Cellular Component	cell part(GO:0044464)	1
OG0031356	Cellular Component	cell(GO:0005623)	1
OG0031356	Cellular Component	membrane(GO:0016020)	1
OG0031363	Cellular Component	cell part(GO:0044464)	1
OG0031363	Cellular Component	cell(GO:0005623)	1
OG0031363	Cellular Component	membrane(GO:0016020)	1
OG0031363	Cellular Component	organelle(GO:0043226)	1

OG0031365	Cellular Component	cell part(GO:0044464)	1
OG0031365	Cellular Component	cell(GO:0005623)	1
OG0031365	Cellular Component	organelle(GO:0043226)	1
OG0031376	Cellular Component	cell part(GO:0044464)	1
OG0031376	Cellular Component	cell(GO:0005623)	1
OG0031376	Cellular Component	organelle(GO:0043226)	1
OG0031377	Cellular Component	cell part(GO:0044464)	1
OG0031377	Cellular Component	cell(GO:0005623)	1
OG0031377	Cellular Component	membrane(GO:0016020)	1
OG0031377	Cellular Component	organelle part(GO:0044422)	1
OG0031377	Cellular Component	organelle(GO:0043226)	1
OG0031379	Cellular Component	cell part(GO:0044464)	1
OG0031379	Cellular Component	cell(GO:0005623)	1
OG0031379	Cellular Component	membrane(GO:0016020)	1
OG0031382	Cellular Component	cell part(GO:0044464)	1
OG0031382	Cellular Component	cell(GO:0005623)	1
OG0031382	Cellular Component	organelle(GO:0043226)	1
OG0031383	Cellular Component	cell part(GO:0044464)	1
OG0031383	Cellular Component	cell(GO:0005623)	1
OG0031383	Cellular Component	extracellular region(GO:0005576)	1
OG0031383	Cellular Component	membrane part(GO:0044425)	1
OG0031383	Cellular Component	membrane(GO:0016020)	1
OG0031384	Cellular Component	cell part(GO:0044464)	1
OG0031384	Cellular Component	cell(GO:0005623)	1
OG0031384	Cellular Component	membrane(GO:0016020)	1
OG0031386	Cellular Component	cell part(GO:0044464)	1
OG0031386	Cellular Component	cell(GO:0005623)	1
OG0031386	Cellular Component	membrane(GO:0016020)	1
OG0031386	Cellular Component	nucleoid(GO:0009295)	1
OG0031387	Cellular Component	cell part(GO:0044464)	1
OG0031387	Cellular Component	cell(GO:0005623)	1
OG0031387	Cellular Component	membrane(GO:0016020)	1
OG0031391	Cellular Component	cell part(GO:0044464)	1
OG0031391	Cellular Component	cell(GO:0005623)	1
OG0031401	Cellular Component	cell part(GO:0044464)	1
OG0031401	Cellular Component	cell(GO:0005623)	1
OG0031401	Cellular Component	membrane(GO:0016020)	1
OG0031402	Cellular Component	cell part(GO:0044464)	1
OG0031402	Cellular Component	cell(GO:0005623)	1
OG0031402	Cellular Component	membrane(GO:0016020)	1
OG0031415	Cellular Component	cell part(GO:0044464)	1
OG0031415	Cellular Component	cell(GO:0005623)	1
OG0031415	Cellular Component	organelle(GO:0043226)	1
OG0031415	Cellular Component	protein-containing complex(GO:0032991)	1
OG0031416	Cellular Component	cell part(GO:0044464)	1
OG0031416	Cellular Component	cell(GO:0005623)	1
OG0031416	Cellular Component	membrane part(GO:0044425)	1

OG0031416	Cellular Component	membrane(GO:0016020)	1
OG0031416	Cellular Component	organelle part(GO:0044422)	1
OG0031416	Cellular Component	organelle(GO:0043226)	1
OG0031417	Cellular Component	membrane(GO:0016020)	1
OG0031419	Cellular Component	cell part(GO:0044464)	1
OG0031419	Cellular Component	cell(GO:0005623)	1
OG0031419	Cellular Component	organelle(GO:0043226)	1
OG0031421	Cellular Component	cell part(GO:0044464)	1
OG0031421	Cellular Component	cell(GO:0005623)	1
OG0031421	Cellular Component	extracellular region(GO:0005576)	1
OG0031423	Cellular Component	cell part(GO:0044464)	1
OG0031423	Cellular Component	cell(GO:0005623)	1
OG0031423	Cellular Component	organelle(GO:0043226)	1
OG0031425	Cellular Component	cell part(GO:0044464)	1
OG0031425	Cellular Component	cell(GO:0005623)	1
OG0031425	Cellular Component	organelle(GO:0043226)	1
OG0031426	Cellular Component	cell part(GO:0044464)	1
OG0031426	Cellular Component	cell(GO:0005623)	1
OG0031426	Cellular Component	organelle(GO:0043226)	1
OG0031428	Cellular Component	cell part(GO:0044464)	1
OG0031428	Cellular Component	cell(GO:0005623)	1
OG0031428	Cellular Component	organelle(GO:0043226)	1
OG0031429	Cellular Component	cell part(GO:0044464)	1
OG0031429	Cellular Component	cell(GO:0005623)	1
OG0031429	Cellular Component	organelle(GO:0043226)	1
OG0031431	Cellular Component	cell part(GO:0044464)	1
OG0031431	Cellular Component	cell(GO:0005623)	1
OG0031431	Cellular Component	membrane(GO:0016020)	1
OG0031431	Cellular Component	organelle part(GO:0044422)	1
OG0031431	Cellular Component	organelle(GO:0043226)	1
OG0031432	Cellular Component	cell part(GO:0044464)	1
OG0031432	Cellular Component	cell(GO:0005623)	1
OG0031432	Cellular Component	organelle(GO:0043226)	1
OG0031433	Cellular Component	cell part(GO:0044464)	1
OG0031433	Cellular Component	cell(GO:0005623)	1
OG0031433	Cellular Component	organelle(GO:0043226)	1
OG0031434	Cellular Component	cell part(GO:0044464)	1
OG0031434	Cellular Component	cell(GO:0005623)	1
OG0031434	Cellular Component	organelle(GO:0043226)	1
OG0031435	Cellular Component	cell part(GO:0044464)	1
OG0031435	Cellular Component	cell(GO:0005623)	1
OG0031435	Cellular Component	organelle(GO:0043226)	1
OG0031437	Cellular Component	cell part(GO:0044464)	1
OG0031437	Cellular Component	cell(GO:0005623)	1
OG0031437	Cellular Component	organelle(GO:0043226)	1
OG0031438	Cellular Component	membrane(GO:0016020)	1
OG0031444	Cellular Component	membrane(GO:0016020)	1

OG0031445	Cellular Component	membrane(GO:0016020)	1
OG0031449	Cellular Component	cell part(GO:0044464)	1
OG0031449	Cellular Component	cell(GO:0005623)	1
OG0031449	Cellular Component	membrane(GO:0016020)	1
OG0031450	Cellular Component	cell part(GO:0044464)	1
OG0031450	Cellular Component	cell(GO:0005623)	1
OG0031450	Cellular Component	membrane(GO:0016020)	1
OG0031451	Cellular Component	cell part(GO:0044464)	1
OG0031451	Cellular Component	cell(GO:0005623)	1
OG0031451	Cellular Component	membrane part(GO:0044425)	1
OG0031451	Cellular Component	membrane(GO:0016020)	1
OG0031451	Cellular Component	organelle part(GO:0044422)	1
OG0031451	Cellular Component	organelle(GO:0043226)	1
OG0031451	Cellular Component	protein-containing complex(GO:0032991)	1
OG0031452	Cellular Component	cell part(GO:0044464)	1
OG0031452	Cellular Component	cell(GO:0005623)	1
OG0031460	Cellular Component	cell part(GO:0044464)	1
OG0031460	Cellular Component	cell(GO:0005623)	1
OG0031460	Cellular Component	membrane part(GO:0044425)	1
OG0031460	Cellular Component	membrane(GO:0016020)	1
OG0031460	Cellular Component	organelle part(GO:0044422)	1
OG0031460	Cellular Component	organelle(GO:0043226)	1
OG0031460	Cellular Component	protein-containing complex(GO:0032991)	1
OG0031466	Cellular Component	cell part(GO:0044464)	1
OG0031466	Cellular Component	cell(GO:0005623)	1
OG0031466	Cellular Component	organelle(GO:0043226)	1
OG0031466	Cellular Component	protein-containing complex(GO:0032991)	1
OG0031467	Cellular Component	cell part(GO:0044464)	1
OG0031467	Cellular Component	cell(GO:0005623)	1
OG0031467	Cellular Component	membrane(GO:0016020)	1
OG0031473	Cellular Component	cell junction(GO:0030054)	1
OG0031473	Cellular Component	cell part(GO:0044464)	1
OG0031473	Cellular Component	cell(GO:0005623)	1
OG0031473	Cellular Component	membrane part(GO:0044425)	1
OG0031473	Cellular Component	membrane(GO:0016020)	1
OG0031473	Cellular Component	symplast(GO:0055044)	1
OG0031474	Cellular Component	cell part(GO:0044464)	1
OG0031474	Cellular Component	cell(GO:0005623)	1
OG0031474	Cellular Component	organelle(GO:0043226)	1
OG0031479	Cellular Component	membrane(GO:0016020)	1
OG0031487	Cellular Component	cell part(GO:0044464)	1
OG0031487	Cellular Component	cell(GO:0005623)	1
OG0031487	Cellular Component	membrane(GO:0016020)	1
OG0031487	Cellular Component	organelle part(GO:0044422)	1
OG0031487	Cellular Component	organelle(GO:0043226)	1
OG0031487	Cellular Component	synapse part(GO:0044456)	1

OG0031487	Cellular Component	synapse(GO:0045202)	1
OG0031495	Cellular Component	cell part(GO:0044464)	1
OG0031495	Cellular Component	cell(GO:0005623)	1
OG0031495	Cellular Component	membrane(GO:0016020)	1
OG0031495	Cellular Component	organelle part(GO:0044422)	1
OG0031495	Cellular Component	organelle(GO:0043226)	1
OG0031502	Cellular Component	cell part(GO:0044464)	1
OG0031502	Cellular Component	cell(GO:0005623)	1
OG0031502	Cellular Component	membrane part(GO:0044425)	1
OG0031502	Cellular Component	membrane(GO:0016020)	1
OG0031502	Cellular Component	protein-containing complex(GO:0032991)	1
OG0031503	Cellular Component	cell part(GO:0044464)	1
OG0031503	Cellular Component	cell(GO:0005623)	1
OG0031504	Cellular Component	cell part(GO:0044464)	1
OG0031504	Cellular Component	cell(GO:0005623)	1
OG0031504	Cellular Component	membrane(GO:0016020)	1
OG0031504	Cellular Component	protein-containing complex(GO:0032991)	1
OG0031505	Cellular Component	cell part(GO:0044464)	1
OG0031505	Cellular Component	cell(GO:0005623)	1
OG0031506	Cellular Component	cell part(GO:0044464)	1
OG0031506	Cellular Component	cell(GO:0005623)	1
OG0031506	Cellular Component	membrane(GO:0016020)	1
OG0031507	Cellular Component	cell part(GO:0044464)	1
OG0031507	Cellular Component	cell(GO:0005623)	1
OG0031507	Cellular Component	organelle(GO:0043226)	1
OG0031509	Cellular Component	cell part(GO:0044464)	1
OG0031509	Cellular Component	cell(GO:0005623)	1
OG0031509	Cellular Component	membrane(GO:0016020)	1
OG0031523	Cellular Component	extracellular region(GO:0005576)	1
OG0031524	Cellular Component	extracellular region(GO:0005576)	1
OG0031526	Cellular Component	cell part(GO:0044464)	1
OG0031526	Cellular Component	cell(GO:0005623)	1
OG0031526	Cellular Component	membrane part(GO:0044425)	1
OG0031526	Cellular Component	membrane(GO:0016020)	1
OG0031526	Cellular Component	nucleoid(GO:0009295)	1
OG0031526	Cellular Component	organelle part(GO:0044422)	1
OG0031526	Cellular Component	organelle(GO:0043226)	1
OG0031526	Cellular Component	protein-containing complex(GO:0032991)	1
OG0031528	Cellular Component	cell part(GO:0044464)	1
OG0031528	Cellular Component	cell(GO:0005623)	1
OG0031528	Cellular Component	organelle part(GO:0044422)	1
OG0031528	Cellular Component	organelle(GO:0043226)	1
OG0031531	Cellular Component	cell part(GO:0044464)	1
OG0031531	Cellular Component	cell(GO:0005623)	1
OG0031532	Cellular Component	cell part(GO:0044464)	1
OG0031532	Cellular Component	cell(GO:0005623)	1

OG0031532	Cellular Component	organelle(GO:0043226)	1
OG0031539	Cellular Component	cell part(GO:0044464)	1
OG0031539	Cellular Component	cell(GO:0005623)	1
OG0031543	Cellular Component	cell part(GO:0044464)	1
OG0031543	Cellular Component	cell(GO:0005623)	1
OG0031549	Cellular Component	cell part(GO:0044464)	1
OG0031549	Cellular Component	cell(GO:0005623)	1
OG0031551	Cellular Component	cell junction(GO:0030054)	1
OG0031551	Cellular Component	cell part(GO:0044464)	1
OG0031551	Cellular Component	cell(GO:0005623)	1
OG0031551	Cellular Component	symplast(GO:0055044)	1
OG0031555	Cellular Component	cell part(GO:0044464)	1
OG0031555	Cellular Component	cell(GO:0005623)	1
OG0031555	Cellular Component	membrane(GO:0016020)	1
OG0031555	Cellular Component	organelle(GO:0043226)	1
OG0031557	Cellular Component	cell part(GO:0044464)	1
OG0031557	Cellular Component	cell(GO:0005623)	1
OG0031557	Cellular Component	membrane(GO:0016020)	1
OG0031557	Cellular Component	organelle(GO:0043226)	1
OG0031560	Cellular Component	cell part(GO:0044464)	1
OG0031560	Cellular Component	cell(GO:0005623)	1
OG0031560	Cellular Component	organelle part(GO:0044422)	1
OG0031560	Cellular Component	organelle(GO:0043226)	1
OG0031564	Cellular Component	cell part(GO:0044464)	1
OG0031564	Cellular Component	cell(GO:0005623)	1
OG0031564	Cellular Component	membrane(GO:0016020)	1
OG0031565	Cellular Component	cell part(GO:0044464)	1
OG0031565	Cellular Component	cell(GO:0005623)	1
OG0031565	Cellular Component	organelle(GO:0043226)	1
OG0031566	Cellular Component	cell part(GO:0044464)	1
OG0031566	Cellular Component	cell(GO:0005623)	1
OG0031566	Cellular Component	organelle(GO:0043226)	1
OG0031580	Cellular Component	cell junction(GO:0030054)	1
OG0031580	Cellular Component	cell part(GO:0044464)	1
OG0031580	Cellular Component	cell(GO:0005623)	1
OG0031580	Cellular Component	membrane(GO:0016020)	1
OG0031580	Cellular Component	symplast(GO:0055044)	1
OG0031582	Cellular Component	cell junction(GO:0030054)	1
OG0031582	Cellular Component	cell part(GO:0044464)	1
OG0031582	Cellular Component	cell(GO:0005623)	1
OG0031582	Cellular Component	membrane(GO:0016020)	1
OG0031582	Cellular Component	symplast(GO:0055044)	1
OG0031587	Cellular Component	cell part(GO:0044464)	1
OG0031587	Cellular Component	cell(GO:0005623)	1
OG0031587	Cellular Component	membrane part(GO:0044425)	1
OG0031587	Cellular Component	membrane(GO:0016020)	1
OG0031587	Cellular Component	membrane-enclosed lumen(GO:0031974)	1

OG0031587	Cellular Component	organelle part(GO:0044422)	1
OG0031587	Cellular Component	organelle(GO:0043226)	1
OG0031587	Cellular Component	protein-containing complex(GO:0032991)	1
OG0031592	Cellular Component	cell part(GO:0044464)	1
OG0031592	Cellular Component	cell(GO:0005623)	1
OG0031592	Cellular Component	membrane(GO:0016020)	1
OG0031593	Cellular Component	membrane part(GO:0044425)	1
OG0031593	Cellular Component	membrane(GO:0016020)	1
OG0031595	Cellular Component	cell part(GO:0044464)	1
OG0031595	Cellular Component	cell(GO:0005623)	1
OG0031595	Cellular Component	membrane-enclosed lumen(GO:0031974)	1
OG0031595	Cellular Component	organelle part(GO:0044422)	1
OG0031595	Cellular Component	organelle(GO:0043226)	1
OG0031595	Cellular Component	protein-containing complex(GO:0032991)	1
OG0031600	Cellular Component	cell part(GO:0044464)	1
OG0031600	Cellular Component	cell(GO:0005623)	1
OG0031600	Cellular Component	organelle(GO:0043226)	1
OG0031603	Cellular Component	extracellular region(GO:0005576)	1
OG0031605	Cellular Component	cell part(GO:0044464)	1
OG0031605	Cellular Component	cell(GO:0005623)	1
OG0031605	Cellular Component	protein-containing complex(GO:0032991)	1
OG0031607	Cellular Component	membrane(GO:0016020)	1
OG0031614	Cellular Component	cell part(GO:0044464)	1
OG0031614	Cellular Component	cell(GO:0005623)	1
OG0031614	Cellular Component	organelle(GO:0043226)	1
OG0031615	Cellular Component	cell part(GO:0044464)	1
OG0031615	Cellular Component	cell(GO:0005623)	1
OG0031615	Cellular Component	organelle(GO:0043226)	1
OG0031617	Cellular Component	cell part(GO:0044464)	1
OG0031617	Cellular Component	cell(GO:0005623)	1
OG0031617	Cellular Component	organelle(GO:0043226)	1
OG0031618	Cellular Component	cell part(GO:0044464)	1
OG0031618	Cellular Component	cell(GO:0005623)	1
OG0031618	Cellular Component	organelle(GO:0043226)	1
OG0031621	Cellular Component	cell part(GO:0044464)	1
OG0031621	Cellular Component	cell(GO:0005623)	1
OG0031621	Cellular Component	organelle(GO:0043226)	1
OG0031630	Cellular Component	cell part(GO:0044464)	1
OG0031630	Cellular Component	cell(GO:0005623)	1
OG0031634	Cellular Component	cell part(GO:0044464)	1
OG0031634	Cellular Component	cell(GO:0005623)	1
OG0031634	Cellular Component	membrane(GO:0016020)	1
OG0031634	Cellular Component	organelle part(GO:0044422)	1
OG0031634	Cellular Component	organelle(GO:0043226)	1
OG0031635	Cellular Component	cell part(GO:0044464)	1
OG0031635	Cellular Component	cell(GO:0005623)	1

OG0031635	Cellular Component	membrane(GO:0016020)	1
OG0031635	Cellular Component	organelle part(GO:0044422)	1
OG0031635	Cellular Component	organelle(GO:0043226)	1
OG0031639	Cellular Component	cell part(GO:0044464)	1
OG0031639	Cellular Component	cell(GO:0005623)	1
OG0031639	Cellular Component	organelle(GO:0043226)	1
OG0031645	Cellular Component	cell part(GO:0044464)	1
OG0031645	Cellular Component	cell(GO:0005623)	1
OG0031648	Cellular Component	cell part(GO:0044464)	1
OG0031648	Cellular Component	cell(GO:0005623)	1
OG0031648	Cellular Component	organelle(GO:0043226)	1
OG0031652	Cellular Component	cell part(GO:0044464)	1
OG0031652	Cellular Component	cell(GO:0005623)	1
OG0031652	Cellular Component	membrane(GO:0016020)	1
OG0031652	Cellular Component	membrane-enclosed lumen(GO:0031974)	1
OG0031652	Cellular Component	organelle part(GO:0044422)	1
OG0031652	Cellular Component	organelle(GO:0043226)	1
OG0031653	Cellular Component	membrane(GO:0016020)	1
OG0031659	Cellular Component	cell part(GO:0044464)	1
OG0031659	Cellular Component	cell(GO:0005623)	1
OG0031659	Cellular Component	membrane(GO:0016020)	1
OG0031665	Cellular Component	cell part(GO:0044464)	1
OG0031665	Cellular Component	cell(GO:0005623)	1
OG0031680	Cellular Component	cell part(GO:0044464)	1
OG0031680	Cellular Component	cell(GO:0005623)	1
OG0031680	Cellular Component	membrane(GO:0016020)	1
OG0031680	Cellular Component	organelle part(GO:0044422)	1
OG0031680	Cellular Component	organelle(GO:0043226)	1
OG0031681	Cellular Component	cell part(GO:0044464)	1
OG0031681	Cellular Component	cell(GO:0005623)	1
OG0031681	Cellular Component	extracellular region(GO:0005576)	1
OG0031681	Cellular Component	membrane(GO:0016020)	1
OG0031681	Cellular Component	organelle part(GO:0044422)	1
OG0031681	Cellular Component	organelle(GO:0043226)	1
OG0031685	Cellular Component	cell part(GO:0044464)	1
OG0031685	Cellular Component	cell(GO:0005623)	1
OG0031685	Cellular Component	organelle(GO:0043226)	1
OG0031689	Cellular Component	cell part(GO:0044464)	1
OG0031689	Cellular Component	cell(GO:0005623)	1
OG0031689	Cellular Component	organelle(GO:0043226)	1
OG0031690	Cellular Component	cell junction(GO:0030054)	1
OG0031690	Cellular Component	cell part(GO:0044464)	1
OG0031690	Cellular Component	cell(GO:0005623)	1
OG0031690	Cellular Component	membrane(GO:0016020)	1
OG0031690	Cellular Component	organelle part(GO:0044422)	1
OG0031690	Cellular Component	organelle(GO:0043226)	1
OG0031690	Cellular Component	protein-containing complex(GO:0032991)	1

OG0031690	Cellular Component	symplast(GO:0055044)	1
OG0031694	Cellular Component	cell part(GO:0044464)	1
OG0031694	Cellular Component	cell(GO:0005623)	1
OG0031694	Cellular Component	membrane(GO:0016020)	1
OG0031694	Cellular Component	organelle(GO:0043226)	1
OG0031695	Cellular Component	cell part(GO:0044464)	1
OG0031695	Cellular Component	cell(GO:0005623)	1
OG0031695	Cellular Component	organelle(GO:0043226)	1
OG0031695	Cellular Component	protein-containing complex(GO:0032991)	1
OG0031701	Cellular Component	cell part(GO:0044464)	1
OG0031701	Cellular Component	cell(GO:0005623)	1
OG0031701	Cellular Component	membrane(GO:0016020)	1
OG0031703	Cellular Component	cell part(GO:0044464)	1
OG0031703	Cellular Component	cell(GO:0005623)	1
OG0031704	Cellular Component	cell part(GO:0044464)	1
OG0031704	Cellular Component	cell(GO:0005623)	1
OG0031704	Cellular Component	membrane(GO:0016020)	1
OG0031705	Cellular Component	cell part(GO:0044464)	1
OG0031705	Cellular Component	cell(GO:0005623)	1
OG0031711	Cellular Component	cell part(GO:0044464)	1
OG0031711	Cellular Component	cell(GO:0005623)	1
OG0031711	Cellular Component	membrane(GO:0016020)	1
OG0031718	Cellular Component	cell part(GO:0044464)	1
OG0031718	Cellular Component	cell(GO:0005623)	1
OG0031719	Cellular Component	cell part(GO:0044464)	1
OG0031719	Cellular Component	cell(GO:0005623)	1
OG0031719	Cellular Component	membrane(GO:0016020)	1
OG0031722	Cellular Component	cell part(GO:0044464)	1
OG0031722	Cellular Component	cell(GO:0005623)	1
OG0031722	Cellular Component	membrane(GO:0016020)	1
OG0031722	Cellular Component	protein-containing complex(GO:0032991)	1
OG0031723	Cellular Component	cell part(GO:0044464)	1
OG0031723	Cellular Component	cell(GO:0005623)	1
OG0031723	Cellular Component	membrane(GO:0016020)	1
OG0031724	Cellular Component	membrane part(GO:0044425)	1
OG0031724	Cellular Component	membrane(GO:0016020)	1
OG0031725	Cellular Component	cell part(GO:0044464)	1
OG0031725	Cellular Component	cell(GO:0005623)	1
OG0031725	Cellular Component	membrane(GO:0016020)	1
OG0031726	Cellular Component	cell part(GO:0044464)	1
OG0031726	Cellular Component	cell(GO:0005623)	1
OG0031726	Cellular Component	membrane(GO:0016020)	1
OG0031727	Cellular Component	cell part(GO:0044464)	1
OG0031727	Cellular Component	cell(GO:0005623)	1
OG0031727	Cellular Component	membrane part(GO:0044425)	1
OG0031727	Cellular Component	membrane(GO:0016020)	1
OG0031727	Cellular Component	organelle part(GO:0044422)	1

OG0031727	Cellular Component	organelle(GO:0043226)	1
OG0031727	Cellular Component	protein-containing complex(GO:0032991)	1
OG0031729	Cellular Component	cell part(GO:0044464)	1
OG0031729	Cellular Component	cell(GO:0005623)	1
OG0031733	Cellular Component	membrane part(GO:0044425)	1
OG0031733	Cellular Component	membrane(GO:0016020)	1
OG0031735	Cellular Component	cell part(GO:0044464)	1
OG0031735	Cellular Component	cell(GO:0005623)	1
OG0031736	Cellular Component	cell part(GO:0044464)	1
OG0031736	Cellular Component	cell(GO:0005623)	1
OG0031736	Cellular Component	membrane part(GO:0044425)	1
OG0031736	Cellular Component	membrane(GO:0016020)	1
OG0031736	Cellular Component	protein-containing complex(GO:0032991)	1
OG0031743	Cellular Component	cell part(GO:0044464)	1
OG0031743	Cellular Component	cell(GO:0005623)	1
OG0031743	Cellular Component	membrane(GO:0016020)	1
OG0031745	Cellular Component	cell part(GO:0044464)	1
OG0031745	Cellular Component	cell(GO:0005623)	1
OG0031745	Cellular Component	membrane(GO:0016020)	1
OG0031747	Cellular Component	membrane part(GO:0044425)	1
OG0031747	Cellular Component	membrane(GO:0016020)	1
OG0031749	Cellular Component	cell part(GO:0044464)	1
OG0031749	Cellular Component	cell(GO:0005623)	1
OG0031749	Cellular Component	membrane(GO:0016020)	1
OG0031751	Cellular Component	cell part(GO:0044464)	1
OG0031751	Cellular Component	cell(GO:0005623)	1
OG0031757	Cellular Component	cell part(GO:0044464)	1
OG0031757	Cellular Component	cell(GO:0005623)	1
OG0031757	Cellular Component	membrane(GO:0016020)	1
OG0031758	Cellular Component	cell part(GO:0044464)	1
OG0031758	Cellular Component	cell(GO:0005623)	1
OG0031758	Cellular Component	membrane(GO:0016020)	1
OG0031764	Cellular Component	cell part(GO:0044464)	1
OG0031764	Cellular Component	cell(GO:0005623)	1
OG0031765	Cellular Component	cell part(GO:0044464)	1
OG0031765	Cellular Component	cell(GO:0005623)	1
OG0031765	Cellular Component	organelle(GO:0043226)	1
OG0031766	Cellular Component	cell part(GO:0044464)	1
OG0031766	Cellular Component	cell(GO:0005623)	1
OG0031766	Cellular Component	membrane(GO:0016020)	1
OG0031767	Cellular Component	cell part(GO:0044464)	1
OG0031767	Cellular Component	cell(GO:0005623)	1
OG0031767	Cellular Component	membrane(GO:0016020)	1
OG0031767	Cellular Component	organelle part(GO:0044422)	1
OG0031767	Cellular Component	organelle(GO:0043226)	1
OG0031769	Cellular Component	cell part(GO:0044464)	1
OG0031769	Cellular Component	cell(GO:0005623)	1

OG0031769	Cellular Component	organelle(GO:0043226)	1
OG0031771	Cellular Component	cell part(GO:0044464)	1
OG0031771	Cellular Component	cell(GO:0005623)	1
OG0031771	Cellular Component	membrane(GO:0016020)	1
OG0031773	Cellular Component	cell part(GO:0044464)	1
OG0031773	Cellular Component	cell(GO:0005623)	1
OG0031773	Cellular Component	membrane(GO:0016020)	1
OG0031776	Cellular Component	cell part(GO:0044464)	1
OG0031776	Cellular Component	cell(GO:0005623)	1
OG0031778	Cellular Component	cell part(GO:0044464)	1
OG0031778	Cellular Component	cell(GO:0005623)	1
OG0031778	Cellular Component	membrane(GO:0016020)	1
OG0031780	Cellular Component	cell part(GO:0044464)	1
OG0031780	Cellular Component	cell(GO:0005623)	1
OG0031780	Cellular Component	membrane(GO:0016020)	1
OG0031783	Cellular Component	cell part(GO:0044464)	1
OG0031783	Cellular Component	cell(GO:0005623)	1
OG0031783	Cellular Component	membrane(GO:0016020)	1
OG0031783	Cellular Component	organelle part(GO:0044422)	1
OG0031783	Cellular Component	organelle(GO:0043226)	1
OG0031784	Cellular Component	cell part(GO:0044464)	1
OG0031784	Cellular Component	cell(GO:0005623)	1
OG0031784	Cellular Component	membrane-enclosed lumen(GO:0031974)	1
OG0031784	Cellular Component	organelle part(GO:0044422)	1
OG0031784	Cellular Component	organelle(GO:0043226)	1
OG0031784	Cellular Component	protein-containing complex(GO:0032991)	1
OG0031787	Cellular Component	cell junction(GO:0030054)	1
OG0031787	Cellular Component	cell part(GO:0044464)	1
OG0031787	Cellular Component	cell(GO:0005623)	1
OG0031787	Cellular Component	membrane part(GO:0044425)	1
OG0031787	Cellular Component	membrane(GO:0016020)	1
OG0031787	Cellular Component	organelle(GO:0043226)	1
OG0031787	Cellular Component	symplast(GO:0055044)	1
OG0031791	Cellular Component	cell part(GO:0044464)	1
OG0031791	Cellular Component	cell(GO:0005623)	1
OG0031793	Cellular Component	cell part(GO:0044464)	1
OG0031793	Cellular Component	cell(GO:0005623)	1
OG0031795	Cellular Component	cell part(GO:0044464)	1
OG0031795	Cellular Component	cell(GO:0005623)	1
OG0031795	Cellular Component	organelle(GO:0043226)	1
OG0031795	Cellular Component	protein-containing complex(GO:0032991)	1
OG0031798	Cellular Component	cell part(GO:0044464)	1
OG0031798	Cellular Component	cell(GO:0005623)	1
OG0031799	Cellular Component	cell part(GO:0044464)	1
OG0031799	Cellular Component	cell(GO:0005623)	1
OG0031799	Cellular Component	organelle(GO:0043226)	1
OG0031801	Cellular Component	cell part(GO:0044464)	1

OG0031801	Cellular Component	cell(GO:0005623)	1
OG0031801	Cellular Component	organelle(GO:0043226)	1
OG0031802	Cellular Component	cell part(GO:0044464)	1
OG0031802	Cellular Component	cell(GO:0005623)	1
OG0031802	Cellular Component	organelle(GO:0043226)	1
OG0031805	Cellular Component	cell part(GO:0044464)	1
OG0031805	Cellular Component	cell(GO:0005623)	1
OG0031805	Cellular Component	organelle(GO:0043226)	1
OG0031806	Cellular Component	cell part(GO:0044464)	1
OG0031806	Cellular Component	cell(GO:0005623)	1
OG0031806	Cellular Component	membrane(GO:0016020)	1
OG0031806	Cellular Component	organelle(GO:0043226)	1
OG0031807	Cellular Component	cell part(GO:0044464)	1
OG0031807	Cellular Component	cell(GO:0005623)	1
OG0031807	Cellular Component	organelle(GO:0043226)	1
OG0031808	Cellular Component	cell part(GO:0044464)	1
OG0031808	Cellular Component	cell(GO:0005623)	1
OG0031810	Cellular Component	cell part(GO:0044464)	1
OG0031810	Cellular Component	cell(GO:0005623)	1
OG0031810	Cellular Component	organelle part(GO:0044422)	1
OG0031810	Cellular Component	organelle(GO:0043226)	1
OG0031810	Cellular Component	protein-containing complex(GO:0032991)	1
OG0031810	Cellular Component	supramolecular complex(GO:0099080)	1
OG0031813	Cellular Component	cell part(GO:0044464)	1
OG0031813	Cellular Component	cell(GO:0005623)	1
OG0031813	Cellular Component	organelle(GO:0043226)	1
OG0031814	Cellular Component	cell part(GO:0044464)	1
OG0031814	Cellular Component	cell(GO:0005623)	1
OG0031814	Cellular Component	organelle(GO:0043226)	1
OG0031816	Cellular Component	cell part(GO:0044464)	1
OG0031816	Cellular Component	cell(GO:0005623)	1
OG0031816	Cellular Component	organelle(GO:0043226)	1
OG0031818	Cellular Component	cell part(GO:0044464)	1
OG0031818	Cellular Component	cell(GO:0005623)	1
OG0031819	Cellular Component	cell part(GO:0044464)	1
OG0031819	Cellular Component	cell(GO:0005623)	1
OG0031819	Cellular Component	organelle(GO:0043226)	1
OG0031822	Cellular Component	cell part(GO:0044464)	1
OG0031822	Cellular Component	cell(GO:0005623)	1
OG0031822	Cellular Component	membrane(GO:0016020)	1
OG0031822	Cellular Component	organelle(GO:0043226)	1
OG0031827	Cellular Component	cell part(GO:0044464)	1
OG0031827	Cellular Component	cell(GO:0005623)	1
OG0031827	Cellular Component	organelle(GO:0043226)	1
OG0031831	Cellular Component	cell part(GO:0044464)	1
OG0031831	Cellular Component	cell(GO:0005623)	1
OG0031831	Cellular Component	organelle(GO:0043226)	1

OG0031835	Cellular Component	cell part(GO:0044464)	1
OG0031835	Cellular Component	cell(GO:0005623)	1
OG0031835	Cellular Component	membrane(GO:0016020)	1
OG0031835	Cellular Component	organelle part(GO:0044422)	1
OG0031835	Cellular Component	organelle(GO:0043226)	1
OG0031837	Cellular Component	cell part(GO:0044464)	1
OG0031837	Cellular Component	cell(GO:0005623)	1
OG0031837	Cellular Component	membrane(GO:0016020)	1
OG0031837	Cellular Component	organelle part(GO:0044422)	1
OG0031837	Cellular Component	organelle(GO:0043226)	1
OG0031840	Cellular Component	cell part(GO:0044464)	1
OG0031840	Cellular Component	cell(GO:0005623)	1
OG0031841	Cellular Component	cell part(GO:0044464)	1
OG0031841	Cellular Component	cell(GO:0005623)	1
OG0031842	Cellular Component	cell part(GO:0044464)	1
OG0031842	Cellular Component	cell(GO:0005623)	1
OG0031842	Cellular Component	membrane(GO:0016020)	1
OG0031843	Cellular Component	cell part(GO:0044464)	1
OG0031843	Cellular Component	cell(GO:0005623)	1
OG0031844	Cellular Component	cell part(GO:0044464)	1
OG0031844	Cellular Component	cell(GO:0005623)	1
OG0031846	Cellular Component	cell part(GO:0044464)	1
OG0031846	Cellular Component	cell(GO:0005623)	1
OG0031847	Cellular Component	cell part(GO:0044464)	1
OG0031847	Cellular Component	cell(GO:0005623)	1
OG0031851	Cellular Component	cell part(GO:0044464)	1
OG0031851	Cellular Component	cell(GO:0005623)	1
OG0031855	Cellular Component	cell part(GO:0044464)	1
OG0031855	Cellular Component	cell(GO:0005623)	1
OG0031855	Cellular Component	membrane(GO:0016020)	1
OG0031862	Cellular Component	cell part(GO:0044464)	1
OG0031862	Cellular Component	cell(GO:0005623)	1
OG0031863	Cellular Component	cell part(GO:0044464)	1
OG0031863	Cellular Component	cell(GO:0005623)	1
OG0031863	Cellular Component	membrane(GO:0016020)	1
OG0031864	Cellular Component	membrane part(GO:0044425)	1
OG0031864	Cellular Component	membrane(GO:0016020)	1
OG0031865	Cellular Component	cell part(GO:0044464)	1
OG0031865	Cellular Component	cell(GO:0005623)	1
OG0031865	Cellular Component	membrane(GO:0016020)	1
OG0031865	Cellular Component	protein-containing complex(GO:0032991)	1
OG0031875	Cellular Component	cell part(GO:0044464)	1
OG0031875	Cellular Component	cell(GO:0005623)	1
OG0031875	Cellular Component	membrane(GO:0016020)	1
OG0031875	Cellular Component	organelle part(GO:0044422)	1
OG0031875	Cellular Component	organelle(GO:0043226)	1
OG0031876	Cellular Component	cell part(GO:0044464)	1

OG0031876	Cellular Component	cell(GO:0005623)	1
OG0031876	Cellular Component	organelle(GO:0043226)	1
OG0031877	Cellular Component	cell part(GO:0044464)	1
OG0031877	Cellular Component	cell(GO:0005623)	1
OG0031877	Cellular Component	organelle(GO:0043226)	1
OG0031878	Cellular Component	cell part(GO:0044464)	1
OG0031878	Cellular Component	cell(GO:0005623)	1
OG0031878	Cellular Component	membrane(GO:0016020)	1
OG0031878	Cellular Component	organelle part(GO:0044422)	1
OG0031878	Cellular Component	organelle(GO:0043226)	1
OG0031880	Cellular Component	cell part(GO:0044464)	1
OG0031880	Cellular Component	cell(GO:0005623)	1
OG0031880	Cellular Component	membrane(GO:0016020)	1
OG0031880	Cellular Component	organelle part(GO:0044422)	1
OG0031880	Cellular Component	organelle(GO:0043226)	1
OG0031881	Cellular Component	cell part(GO:0044464)	1
OG0031881	Cellular Component	cell(GO:0005623)	1
OG0031881	Cellular Component	membrane(GO:0016020)	1
OG0031881	Cellular Component	organelle part(GO:0044422)	1
OG0031881	Cellular Component	organelle(GO:0043226)	1
OG0031885	Cellular Component	cell part(GO:0044464)	1
OG0031885	Cellular Component	cell(GO:0005623)	1
OG0031885	Cellular Component	membrane part(GO:0044425)	1
OG0031885	Cellular Component	membrane(GO:0016020)	1
OG0031885	Cellular Component	organelle part(GO:0044422)	1
OG0031885	Cellular Component	organelle(GO:0043226)	1
OG0031885	Cellular Component	protein-containing complex(GO:0032991)	1
OG0031888	Cellular Component	cell part(GO:0044464)	1
OG0031888	Cellular Component	cell(GO:0005623)	1
OG0031889	Cellular Component	cell part(GO:0044464)	1
OG0031889	Cellular Component	cell(GO:0005623)	1
OG0031889	Cellular Component	organelle part(GO:0044422)	1
OG0031889	Cellular Component	organelle(GO:0043226)	1
OG0031889	Cellular Component	protein-containing complex(GO:0032991)	1
OG0031904	Cellular Component	cell part(GO:0044464)	1
OG0031904	Cellular Component	cell(GO:0005623)	1
OG0031904	Cellular Component	organelle(GO:0043226)	1
OG0031905	Cellular Component	cell part(GO:0044464)	1
OG0031905	Cellular Component	cell(GO:0005623)	1
OG0031905	Cellular Component	organelle(GO:0043226)	1
OG0031922	Cellular Component	cell part(GO:0044464)	1
OG0031922	Cellular Component	cell(GO:0005623)	1
OG0031922	Cellular Component	organelle(GO:0043226)	1
OG0031928	Cellular Component	cell part(GO:0044464)	1
OG0031928	Cellular Component	cell(GO:0005623)	1
OG0031928	Cellular Component	protein-containing complex(GO:0032991)	1

OG0031931	Cellular Component	cell part(GO:0044464)	1
OG0031931	Cellular Component	cell(GO:0005623)	1
OG0031933	Cellular Component	cell junction(GO:0030054)	1
OG0031933	Cellular Component	cell part(GO:0044464)	1
OG0031933	Cellular Component	cell(GO:0005623)	1
OG0031933	Cellular Component	membrane(GO:0016020)	1
OG0031933	Cellular Component	organelle part(GO:0044422)	1
OG0031933	Cellular Component	organelle(GO:0043226)	1
OG0031933	Cellular Component	protein-containing complex(GO:0032991)	1
OG0031933	Cellular Component	symplast(GO:0055044)	1
OG0031937	Cellular Component	cell part(GO:0044464)	1
OG0031937	Cellular Component	cell(GO:0005623)	1
OG0031937	Cellular Component	organelle(GO:0043226)	1
OG0031938	Cellular Component	cell part(GO:0044464)	1
OG0031938	Cellular Component	cell(GO:0005623)	1
OG0031938	Cellular Component	organelle(GO:0043226)	1
OG0031940	Cellular Component	cell part(GO:0044464)	1
OG0031940	Cellular Component	cell(GO:0005623)	1
OG0031940	Cellular Component	organelle(GO:0043226)	1
OG0031943	Cellular Component	cell part(GO:0044464)	1
OG0031943	Cellular Component	cell(GO:0005623)	1
OG0031943	Cellular Component	organelle(GO:0043226)	1
OG0031950	Cellular Component	cell part(GO:0044464)	1
OG0031950	Cellular Component	cell(GO:0005623)	1
OG0031950	Cellular Component	membrane(GO:0016020)	1
OG0031951	Cellular Component	cell part(GO:0044464)	1
OG0031951	Cellular Component	cell(GO:0005623)	1
OG0031951	Cellular Component	membrane(GO:0016020)	1
OG0031952	Cellular Component	cell part(GO:0044464)	1
OG0031952	Cellular Component	cell(GO:0005623)	1
OG0031952	Cellular Component	membrane(GO:0016020)	1
OG0031953	Cellular Component	cell part(GO:0044464)	1
OG0031953	Cellular Component	cell(GO:0005623)	1
OG0031953	Cellular Component	membrane(GO:0016020)	1
OG0031958	Cellular Component	cell part(GO:0044464)	1
OG0031958	Cellular Component	cell(GO:0005623)	1
OG0031958	Cellular Component	membrane part(GO:0044425)	1
OG0031958	Cellular Component	membrane(GO:0016020)	1
OG0031958	Cellular Component	organelle part(GO:0044422)	1
OG0031958	Cellular Component	organelle(GO:0043226)	1
OG0031959	Cellular Component	cell part(GO:0044464)	1
OG0031959	Cellular Component	cell(GO:0005623)	1
OG0031960	Cellular Component	cell part(GO:0044464)	1
OG0031960	Cellular Component	cell(GO:0005623)	1
OG0031962	Cellular Component	cell part(GO:0044464)	1
OG0031962	Cellular Component	cell(GO:0005623)	1
OG0031962	Cellular Component	organelle(GO:0043226)	1

OG0031963	Cellular Component	cell part(GO:0044464)	1
OG0031963	Cellular Component	cell(GO:0005623)	1
OG0031963	Cellular Component	membrane(GO:0016020)	1
OG0031963	Cellular Component	organelle(GO:0043226)	1
OG0031965	Cellular Component	cell part(GO:0044464)	1
OG0031965	Cellular Component	cell(GO:0005623)	1
OG0031965	Cellular Component	organelle part(GO:0044422)	1
OG0031965	Cellular Component	organelle(GO:0043226)	1
OG0031965	Cellular Component	protein-containing complex(GO:0032991)	1
OG0031967	Cellular Component	cell part(GO:0044464)	1
OG0031967	Cellular Component	cell(GO:0005623)	1
OG0031967	Cellular Component	membrane-enclosed lumen(GO:0031974)	1
OG0031967	Cellular Component	organelle part(GO:0044422)	1
OG0031967	Cellular Component	organelle(GO:0043226)	1
OG0031967	Cellular Component	protein-containing complex(GO:0032991)	1
OG0031971	Cellular Component	cell part(GO:0044464)	1
OG0031971	Cellular Component	cell(GO:0005623)	1
OG0031971	Cellular Component	membrane(GO:0016020)	1
OG0031971	Cellular Component	membrane-enclosed lumen(GO:0031974)	1
OG0031971	Cellular Component	organelle part(GO:0044422)	1
OG0031971	Cellular Component	organelle(GO:0043226)	1
OG0031972	Cellular Component	cell part(GO:0044464)	1
OG0031972	Cellular Component	cell(GO:0005623)	1
OG0031972	Cellular Component	membrane(GO:0016020)	1
OG0031973	Cellular Component	cell part(GO:0044464)	1
OG0031973	Cellular Component	cell(GO:0005623)	1
OG0031979	Cellular Component	cell part(GO:0044464)	1
OG0031979	Cellular Component	cell(GO:0005623)	1
OG0031979	Cellular Component	organelle(GO:0043226)	1
OG0031998	Cellular Component	cell part(GO:0044464)	1
OG0031998	Cellular Component	cell(GO:0005623)	1
OG0031998	Cellular Component	membrane(GO:0016020)	1
OG0031998	Cellular Component	organelle(GO:0043226)	1
OG0032001	Cellular Component	cell part(GO:0044464)	1
OG0032001	Cellular Component	cell(GO:0005623)	1
OG0032011	Cellular Component	membrane(GO:0016020)	1
OG0032014	Cellular Component	cell part(GO:0044464)	1
OG0032014	Cellular Component	cell(GO:0005623)	1
OG0032014	Cellular Component	extracellular region(GO:0005576)	1
OG0032021	Cellular Component	cell part(GO:0044464)	1
OG0032021	Cellular Component	cell(GO:0005623)	1
OG0032021	Cellular Component	extracellular region(GO:0005576)	1
OG0032021	Cellular Component	organelle(GO:0043226)	1
OG0032026	Cellular Component	cell part(GO:0044464)	1
OG0032026	Cellular Component	cell(GO:0005623)	1
OG0032026	Cellular Component	organelle(GO:0043226)	1
OG0032028	Cellular Component	cell part(GO:0044464)	1

OG0032028	Cellular Component	cell(GO:0005623)	1
OG0032028	Cellular Component	organelle(GO:0043226)	1
OG0032036	Cellular Component	cell part(GO:0044464)	1
OG0032036	Cellular Component	cell(GO:0005623)	1
OG0032036	Cellular Component	extracellular region(GO:0005576)	1
OG0032036	Cellular Component	membrane(GO:0016020)	1
OG0032036	Cellular Component	organelle part(GO:0044422)	1
OG0032036	Cellular Component	organelle(GO:0043226)	1
OG0032036	Cellular Component	protein-containing complex(GO:0032991)	1
OG0032041	Cellular Component	cell part(GO:0044464)	1
OG0032041	Cellular Component	cell(GO:0005623)	1
OG0032041	Cellular Component	membrane(GO:0016020)	1
OG0032044	Cellular Component	cell part(GO:0044464)	1
OG0032044	Cellular Component	cell(GO:0005623)	1
OG0032045	Cellular Component	cell part(GO:0044464)	1
OG0032045	Cellular Component	cell(GO:0005623)	1
OG0032049	Cellular Component	cell part(GO:0044464)	1
OG0032049	Cellular Component	cell(GO:0005623)	1
OG0032049	Cellular Component	membrane(GO:0016020)	1
OG0032050	Cellular Component	cell part(GO:0044464)	1
OG0032050	Cellular Component	cell(GO:0005623)	1
OG0032050	Cellular Component	membrane(GO:0016020)	1
OG0032051	Cellular Component	cell part(GO:0044464)	1
OG0032051	Cellular Component	cell(GO:0005623)	1
OG0032054	Cellular Component	cell part(GO:0044464)	1
OG0032054	Cellular Component	cell(GO:0005623)	1
OG0032070	Cellular Component	cell part(GO:0044464)	1
OG0032070	Cellular Component	cell(GO:0005623)	1
OG0032070	Cellular Component	membrane(GO:0016020)	1
OG0032073	Cellular Component	cell part(GO:0044464)	1
OG0032073	Cellular Component	cell(GO:0005623)	1
OG0032073	Cellular Component	membrane(GO:0016020)	1
OG0032074	Cellular Component	cell part(GO:0044464)	1
OG0032074	Cellular Component	cell(GO:0005623)	1
OG0032074	Cellular Component	membrane(GO:0016020)	1
OG0032082	Cellular Component	cell part(GO:0044464)	1
OG0032082	Cellular Component	cell(GO:0005623)	1
OG0032086	Cellular Component	cell part(GO:0044464)	1
OG0032086	Cellular Component	cell(GO:0005623)	1
OG0032086	Cellular Component	organelle(GO:0043226)	1
OG0032087	Cellular Component	cell part(GO:0044464)	1
OG0032087	Cellular Component	cell(GO:0005623)	1
OG0032087	Cellular Component	membrane(GO:0016020)	1
OG0032088	Cellular Component	cell part(GO:0044464)	1
OG0032088	Cellular Component	cell(GO:0005623)	1
OG0032088	Cellular Component	membrane(GO:0016020)	1
OG0032089	Cellular Component	cell part(GO:0044464)	1

OG0032089	Cellular Component	cell(GO:0005623)	1
OG0032089	Cellular Component	membrane(GO:0016020)	1
OG0032089	Cellular Component	organelle part(GO:0044422)	1
OG0032089	Cellular Component	organelle(GO:0043226)	1
OG0032090	Cellular Component	cell part(GO:0044464)	1
OG0032090	Cellular Component	cell(GO:0005623)	1
OG0032090	Cellular Component	membrane(GO:0016020)	1
OG0032093	Cellular Component	cell junction(GO:0030054)	1
OG0032093	Cellular Component	cell part(GO:0044464)	1
OG0032093	Cellular Component	cell(GO:0005623)	1
OG0032093	Cellular Component	membrane part(GO:0044425)	1
OG0032093	Cellular Component	membrane(GO:0016020)	1
OG0032093	Cellular Component	organelle part(GO:0044422)	1
OG0032093	Cellular Component	organelle(GO:0043226)	1
OG0032093	Cellular Component	symplast(GO:0055044)	1
OG0032095	Cellular Component	cell part(GO:0044464)	1
OG0032095	Cellular Component	cell(GO:0005623)	1
OG0032095	Cellular Component	membrane(GO:0016020)	1
OG0032095	Cellular Component	organelle part(GO:0044422)	1
OG0032095	Cellular Component	organelle(GO:0043226)	1
OG0032095	Cellular Component	protein-containing complex(GO:0032991)	1
OG0032095	Cellular Component	supramolecular complex(GO:0099080)	1
OG0032096	Cellular Component	cell part(GO:0044464)	1
OG0032096	Cellular Component	cell(GO:0005623)	1
OG0032096	Cellular Component	organelle(GO:0043226)	1
OG0032101	Cellular Component	cell part(GO:0044464)	1
OG0032101	Cellular Component	cell(GO:0005623)	1
OG0032116	Cellular Component	cell junction(GO:0030054)	1
OG0032116	Cellular Component	cell part(GO:0044464)	1
OG0032116	Cellular Component	cell(GO:0005623)	1
OG0032116	Cellular Component	membrane(GO:0016020)	1
OG0032116	Cellular Component	organelle part(GO:0044422)	1
OG0032116	Cellular Component	organelle(GO:0043226)	1
OG0032116	Cellular Component	protein-containing complex(GO:0032991)	1
OG0032116	Cellular Component	symplast(GO:0055044)	1
OG0032119	Cellular Component	cell part(GO:0044464)	1
OG0032119	Cellular Component	cell(GO:0005623)	1
OG0032119	Cellular Component	membrane-enclosed lumen(GO:0031974)	1
OG0032119	Cellular Component	organelle part(GO:0044422)	1
OG0032119	Cellular Component	organelle(GO:0043226)	1
OG0032121	Cellular Component	cell part(GO:0044464)	1
OG0032121	Cellular Component	cell(GO:0005623)	1
OG0032121	Cellular Component	organelle(GO:0043226)	1
OG0032124	Cellular Component	cell junction(GO:0030054)	1
OG0032124	Cellular Component	cell part(GO:0044464)	1
OG0032124	Cellular Component	cell(GO:0005623)	1
OG0032124	Cellular Component	organelle(GO:0043226)	1

OG0032124	Cellular Component	protein-containing complex(GO:0032991)	1
OG0032124	Cellular Component	symplast(GO:0055044)	1
OG0032125	Cellular Component	cell junction(GO:0030054)	1
OG0032125	Cellular Component	cell part(GO:0044464)	1
OG0032125	Cellular Component	cell(GO:0005623)	1
OG0032125	Cellular Component	membrane(GO:0016020)	1
OG0032125	Cellular Component	organelle part(GO:0044422)	1
OG0032125	Cellular Component	organelle(GO:0043226)	1
OG0032125	Cellular Component	protein-containing complex(GO:0032991)	1
OG0032125	Cellular Component	symplast(GO:0055044)	1
OG0032126	Cellular Component	cell part(GO:0044464)	1
OG0032126	Cellular Component	cell(GO:0005623)	1
OG0032126	Cellular Component	organelle(GO:0043226)	1
OG0032127	Cellular Component	cell part(GO:0044464)	1
OG0032127	Cellular Component	cell(GO:0005623)	1
OG0032127	Cellular Component	organelle(GO:0043226)	1
OG0032129	Cellular Component	cell part(GO:0044464)	1
OG0032129	Cellular Component	cell(GO:0005623)	1
OG0032134	Cellular Component	cell part(GO:0044464)	1
OG0032134	Cellular Component	cell(GO:0005623)	1
OG0032134	Cellular Component	extracellular region(GO:0005576)	1
OG0032134	Cellular Component	membrane(GO:0016020)	1
OG0032134	Cellular Component	organelle part(GO:0044422)	1
OG0032134	Cellular Component	organelle(GO:0043226)	1
OG0032139	Cellular Component	cell part(GO:0044464)	1
OG0032139	Cellular Component	cell(GO:0005623)	1
OG0032139	Cellular Component	membrane(GO:0016020)	1
OG0032139	Cellular Component	organelle part(GO:0044422)	1
OG0032139	Cellular Component	organelle(GO:0043226)	1
OG0032141	Cellular Component	cell part(GO:0044464)	1
OG0032141	Cellular Component	cell(GO:0005623)	1
OG0032141	Cellular Component	membrane(GO:0016020)	1
OG0032141	Cellular Component	organelle part(GO:0044422)	1
OG0032141	Cellular Component	organelle(GO:0043226)	1
OG0032143	Cellular Component	cell junction(GO:0030054)	1
OG0032143	Cellular Component	cell part(GO:0044464)	1
OG0032143	Cellular Component	cell(GO:0005623)	1
OG0032143	Cellular Component	symplast(GO:0055044)	1
OG0032148	Cellular Component	cell part(GO:0044464)	1
OG0032148	Cellular Component	cell(GO:0005623)	1
OG0032148	Cellular Component	organelle(GO:0043226)	1
OG0032151	Cellular Component	cell part(GO:0044464)	1
OG0032151	Cellular Component	cell(GO:0005623)	1
OG0032151	Cellular Component	membrane part(GO:0044425)	1
OG0032151	Cellular Component	membrane(GO:0016020)	1
OG0032151	Cellular Component	membrane-enclosed lumen(GO:0031974)	1
OG0032151	Cellular Component	organelle part(GO:0044422)	1

OG0032151	Cellular Component	organelle(GO:0043226)	1
OG0032151	Cellular Component	protein-containing complex(GO:0032991)	1
OG0032158	Cellular Component	cell part(GO:0044464)	1
OG0032158	Cellular Component	cell(GO:0005623)	1
OG0032158	Cellular Component	membrane(GO:0016020)	1
OG0032158	Cellular Component	membrane-enclosed lumen(GO:0031974)	1
OG0032158	Cellular Component	organelle part(GO:0044422)	1
OG0032158	Cellular Component	organelle(GO:0043226)	1
OG0032158	Cellular Component	protein-containing complex(GO:0032991)	1
OG0032161	Cellular Component	cell part(GO:0044464)	1
OG0032161	Cellular Component	cell(GO:0005623)	1
OG0032161	Cellular Component	membrane-enclosed lumen(GO:0031974)	1
OG0032161	Cellular Component	organelle part(GO:0044422)	1
OG0032161	Cellular Component	organelle(GO:0043226)	1
OG0032161	Cellular Component	protein-containing complex(GO:0032991)	1
OG0032165	Cellular Component	cell part(GO:0044464)	1
OG0032165	Cellular Component	cell(GO:0005623)	1
OG0032165	Cellular Component	membrane(GO:0016020)	1
OG0032165	Cellular Component	organelle part(GO:0044422)	1
OG0032165	Cellular Component	organelle(GO:0043226)	1
OG0032168	Cellular Component	cell part(GO:0044464)	1
OG0032168	Cellular Component	cell(GO:0005623)	1
OG0032168	Cellular Component	organelle(GO:0043226)	1
OG0032170	Cellular Component	cell junction(GO:0030054)	1
OG0032170	Cellular Component	cell part(GO:0044464)	1
OG0032170	Cellular Component	cell(GO:0005623)	1
OG0032170	Cellular Component	membrane(GO:0016020)	1
OG0032170	Cellular Component	organelle(GO:0043226)	1
OG0032170	Cellular Component	symplast(GO:0055044)	1
OG0032172	Cellular Component	cell part(GO:0044464)	1
OG0032172	Cellular Component	cell(GO:0005623)	1
OG0032172	Cellular Component	organelle(GO:0043226)	1
OG0032179	Cellular Component	cell part(GO:0044464)	1
OG0032179	Cellular Component	cell(GO:0005623)	1
OG0032179	Cellular Component	membrane(GO:0016020)	1
OG0032180	Cellular Component	cell part(GO:0044464)	1
OG0032180	Cellular Component	cell(GO:0005623)	1
OG0032180	Cellular Component	organelle part(GO:0044422)	1
OG0032180	Cellular Component	organelle(GO:0043226)	1
OG0032180	Cellular Component	protein-containing complex(GO:0032991)	1
OG0032180	Cellular Component	supramolecular complex(GO:0099080)	1
OG0032183	Cellular Component	cell part(GO:0044464)	1
OG0032183	Cellular Component	cell(GO:0005623)	1
OG0032183	Cellular Component	extracellular region(GO:0005576)	1
OG0032183	Cellular Component	membrane(GO:0016020)	1
OG0032183	Cellular Component	organelle part(GO:0044422)	1

OG0032183	Cellular Component	organelle(GO:0043226)	1
OG0032185	Cellular Component	cell part(GO:0044464)	1
OG0032185	Cellular Component	cell(GO:0005623)	1
OG0032185	Cellular Component	membrane(GO:0016020)	1
OG0032186	Cellular Component	cell part(GO:0044464)	1
OG0032186	Cellular Component	cell(GO:0005623)	1
OG0032186	Cellular Component	membrane(GO:0016020)	1
OG0032190	Cellular Component	cell part(GO:0044464)	1
OG0032190	Cellular Component	cell(GO:0005623)	1
OG0032191	Cellular Component	cell part(GO:0044464)	1
OG0032191	Cellular Component	cell(GO:0005623)	1
OG0032191	Cellular Component	membrane part(GO:0044425)	1
OG0032191	Cellular Component	membrane(GO:0016020)	1
OG0032192	Cellular Component	cell part(GO:0044464)	1
OG0032192	Cellular Component	cell(GO:0005623)	1
OG0032192	Cellular Component	membrane(GO:0016020)	1
OG0032201	Cellular Component	cell part(GO:0044464)	1
OG0032201	Cellular Component	cell(GO:0005623)	1
OG0032201	Cellular Component	organelle part(GO:0044422)	1
OG0032201	Cellular Component	organelle(GO:0043226)	1
OG0032202	Cellular Component	cell part(GO:0044464)	1
OG0032202	Cellular Component	cell(GO:0005623)	1
OG0032202	Cellular Component	organelle(GO:0043226)	1
OG0032217	Cellular Component	cell part(GO:0044464)	1
OG0032217	Cellular Component	cell(GO:0005623)	1
OG0032218	Cellular Component	cell part(GO:0044464)	1
OG0032218	Cellular Component	cell(GO:0005623)	1
OG0032218	Cellular Component	organelle(GO:0043226)	1
OG0032220	Cellular Component	cell part(GO:0044464)	1
OG0032220	Cellular Component	cell(GO:0005623)	1
OG0032220	Cellular Component	organelle(GO:0043226)	1
OG0032222	Cellular Component	cell part(GO:0044464)	1
OG0032222	Cellular Component	cell(GO:0005623)	1
OG0032223	Cellular Component	cell junction(GO:0030054)	1
OG0032223	Cellular Component	cell part(GO:0044464)	1
OG0032223	Cellular Component	cell(GO:0005623)	1
OG0032223	Cellular Component	membrane part(GO:0044425)	1
OG0032223	Cellular Component	membrane(GO:0016020)	1
OG0032223	Cellular Component	organelle part(GO:0044422)	1
OG0032223	Cellular Component	organelle(GO:0043226)	1
OG0032223	Cellular Component	symplast(GO:0055044)	1
OG0032224	Cellular Component	cell part(GO:0044464)	1
OG0032224	Cellular Component	cell(GO:0005623)	1
OG0032224	Cellular Component	organelle(GO:0043226)	1
OG0032227	Cellular Component	cell part(GO:0044464)	1
OG0032227	Cellular Component	cell(GO:0005623)	1
OG0032227	Cellular Component	organelle(GO:0043226)	1

OG0032230	Cellular Component	cell part(GO:0044464)	1
OG0032230	Cellular Component	cell(GO:0005623)	1
OG0032230	Cellular Component	organelle(GO:0043226)	1
OG0032231	Cellular Component	cell part(GO:0044464)	1
OG0032231	Cellular Component	cell(GO:0005623)	1
OG0032231	Cellular Component	membrane(GO:0016020)	1
OG0032231	Cellular Component	organelle(GO:0043226)	1
OG0032232	Cellular Component	cell part(GO:0044464)	1
OG0032232	Cellular Component	cell(GO:0005623)	1
OG0032232	Cellular Component	organelle(GO:0043226)	1
OG0032233	Cellular Component	cell part(GO:0044464)	1
OG0032233	Cellular Component	cell(GO:0005623)	1
OG0032233	Cellular Component	organelle(GO:0043226)	1
OG0032234	Cellular Component	cell part(GO:0044464)	1
OG0032234	Cellular Component	cell(GO:0005623)	1
OG0032234	Cellular Component	organelle(GO:0043226)	1
OG0032236	Cellular Component	cell part(GO:0044464)	1
OG0032236	Cellular Component	cell(GO:0005623)	1
OG0032236	Cellular Component	organelle part(GO:0044422)	1
OG0032236	Cellular Component	organelle(GO:0043226)	1
OG0032237	Cellular Component	cell part(GO:0044464)	1
OG0032237	Cellular Component	cell(GO:0005623)	1
OG0032237	Cellular Component	organelle(GO:0043226)	1
OG0032252	Cellular Component	cell part(GO:0044464)	1
OG0032252	Cellular Component	cell(GO:0005623)	1
OG0032252	Cellular Component	organelle(GO:0043226)	1
OG0032253	Cellular Component	cell part(GO:0044464)	1
OG0032253	Cellular Component	cell(GO:0005623)	1
OG0032253	Cellular Component	membrane part(GO:0044425)	1
OG0032253	Cellular Component	membrane(GO:0016020)	1
OG0032253	Cellular Component	organelle part(GO:0044422)	1
OG0032253	Cellular Component	organelle(GO:0043226)	1
OG0032256	Cellular Component	cell part(GO:0044464)	1
OG0032256	Cellular Component	cell(GO:0005623)	1
OG0032256	Cellular Component	membrane(GO:0016020)	1
OG0032256	Cellular Component	membrane-enclosed lumen(GO:0031974)	1
OG0032256	Cellular Component	organelle part(GO:0044422)	1
OG0032256	Cellular Component	organelle(GO:0043226)	1
OG0032256	Cellular Component	protein-containing complex(GO:0032991)	1
OG0032259	Cellular Component	cell part(GO:0044464)	1
OG0032259	Cellular Component	cell(GO:0005623)	1
OG0032259	Cellular Component	organelle(GO:0043226)	1
OG0032262	Cellular Component	cell part(GO:0044464)	1
OG0032262	Cellular Component	cell(GO:0005623)	1
OG0032262	Cellular Component	protein-containing complex(GO:0032991)	1
OG0032264	Cellular Component	cell part(GO:0044464)	1
OG0032264	Cellular Component	cell(GO:0005623)	1

OG0032264	Cellular Component	organelle(GO:0043226)	1
OG0032273	Cellular Component	cell part(GO:0044464)	1
OG0032273	Cellular Component	cell(GO:0005623)	1
OG0032273	Cellular Component	organelle part(GO:0044422)	1
OG0032273	Cellular Component	organelle(GO:0043226)	1
OG0032278	Cellular Component	cell part(GO:0044464)	1
OG0032278	Cellular Component	cell(GO:0005623)	1
OG0032278	Cellular Component	organelle(GO:0043226)	1
OG0032279	Cellular Component	cell part(GO:0044464)	1
OG0032279	Cellular Component	cell(GO:0005623)	1
OG0032279	Cellular Component	organelle(GO:0043226)	1
OG0032289	Cellular Component	cell part(GO:0044464)	1
OG0032289	Cellular Component	cell(GO:0005623)	1
OG0032289	Cellular Component	extracellular region(GO:0005576)	1
OG0032290	Cellular Component	cell part(GO:0044464)	1
OG0032290	Cellular Component	cell(GO:0005623)	1
OG0032290	Cellular Component	extracellular region(GO:0005576)	1
OG0032294	Cellular Component	membrane(GO:0016020)	1
OG0032297	Cellular Component	cell part(GO:0044464)	1
OG0032297	Cellular Component	cell(GO:0005623)	1
OG0032297	Cellular Component	organelle(GO:0043226)	1
OG0032297	Cellular Component	protein-containing complex(GO:0032991)	1
OG0032298	Cellular Component	cell part(GO:0044464)	1
OG0032298	Cellular Component	cell(GO:0005623)	1
OG0032298	Cellular Component	membrane part(GO:0044425)	1
OG0032298	Cellular Component	membrane(GO:0016020)	1
OG0032298	Cellular Component	organelle part(GO:0044422)	1
OG0032298	Cellular Component	organelle(GO:0043226)	1
OG0032298	Cellular Component	protein-containing complex(GO:0032991)	1
OG0032300	Cellular Component	cell part(GO:0044464)	1
OG0032300	Cellular Component	cell(GO:0005623)	1
OG0032300	Cellular Component	membrane(GO:0016020)	1
OG0032300	Cellular Component	organelle part(GO:0044422)	1
OG0032300	Cellular Component	organelle(GO:0043226)	1
OG0032300	Cellular Component	protein-containing complex(GO:0032991)	1
OG0032305	Cellular Component	cell part(GO:0044464)	1
OG0032305	Cellular Component	cell(GO:0005623)	1
OG0032305	Cellular Component	organelle part(GO:0044422)	1
OG0032305	Cellular Component	organelle(GO:0043226)	1
OG0032305	Cellular Component	protein-containing complex(GO:0032991)	1
OG0032305	Cellular Component	supramolecular complex(GO:0099080)	1
OG0032306	Cellular Component	cell part(GO:0044464)	1
OG0032306	Cellular Component	cell(GO:0005623)	1
OG0032306	Cellular Component	organelle(GO:0043226)	1
OG0032315	Cellular Component	cell part(GO:0044464)	1
OG0032315	Cellular Component	cell(GO:0005623)	1

OG0032315	Cellular Component	organelle(GO:0043226)	1
OG0032321	Cellular Component	cell part(GO:0044464)	1
OG0032321	Cellular Component	cell(GO:0005623)	1
OG0032321	Cellular Component	organelle(GO:0043226)	1
OG0032325	Cellular Component	cell part(GO:0044464)	1
OG0032325	Cellular Component	cell(GO:0005623)	1
OG0032325	Cellular Component	membrane(GO:0016020)	1
OG0032327	Cellular Component	extracellular region(GO:0005576)	1
OG0032328	Cellular Component	cell part(GO:0044464)	1
OG0032328	Cellular Component	cell(GO:0005623)	1
OG0032328	Cellular Component	membrane part(GO:0044425)	1
OG0032328	Cellular Component	membrane(GO:0016020)	1
OG0032328	Cellular Component	organelle part(GO:0044422)	1
OG0032328	Cellular Component	organelle(GO:0043226)	1
OG0032328	Cellular Component	protein-containing complex(GO:0032991)	1
OG0032335	Cellular Component	cell junction(GO:0030054)	1
OG0032335	Cellular Component	cell part(GO:0044464)	1
OG0032335	Cellular Component	cell(GO:0005623)	1
OG0032335	Cellular Component	membrane(GO:0016020)	1
OG0032335	Cellular Component	symplast(GO:0055044)	1
OG0032342	Cellular Component	cell junction(GO:0030054)	1
OG0032342	Cellular Component	cell part(GO:0044464)	1
OG0032342	Cellular Component	cell(GO:0005623)	1
OG0032342	Cellular Component	membrane(GO:0016020)	1
OG0032342	Cellular Component	organelle(GO:0043226)	1
OG0032342	Cellular Component	symplast(GO:0055044)	1
OG0032344	Cellular Component	cell part(GO:0044464)	1
OG0032344	Cellular Component	cell(GO:0005623)	1
OG0032344	Cellular Component	membrane part(GO:0044425)	1
OG0032344	Cellular Component	membrane(GO:0016020)	1
OG0032344	Cellular Component	organelle part(GO:0044422)	1
OG0032344	Cellular Component	organelle(GO:0043226)	1
OG0032344	Cellular Component	protein-containing complex(GO:0032991)	1
OG0032345	Cellular Component	cell part(GO:0044464)	1
OG0032345	Cellular Component	cell(GO:0005623)	1
OG0032345	Cellular Component	extracellular region(GO:0005576)	1
OG0032345	Cellular Component	membrane(GO:0016020)	1
OG0032345	Cellular Component	organelle part(GO:0044422)	1
OG0032345	Cellular Component	organelle(GO:0043226)	1
OG0032347	Cellular Component	cell part(GO:0044464)	1
OG0032347	Cellular Component	cell(GO:0005623)	1
OG0032347	Cellular Component	membrane(GO:0016020)	1
OG0032348	Cellular Component	cell part(GO:0044464)	1
OG0032348	Cellular Component	cell(GO:0005623)	1
OG0032348	Cellular Component	membrane(GO:0016020)	1
OG0032349	Cellular Component	cell part(GO:0044464)	1
OG0032349	Cellular Component	cell(GO:0005623)	1

OG0032349	Cellular Component	membrane(GO:0016020)	1
OG0032350	Cellular Component	extracellular region(GO:0005576)	1
OG0032355	Cellular Component	cell part(GO:0044464)	1
OG0032355	Cellular Component	cell(GO:0005623)	1
OG0032355	Cellular Component	membrane(GO:0016020)	1
OG0032355	Cellular Component	organelle part(GO:0044422)	1
OG0032355	Cellular Component	organelle(GO:0043226)	1
OG0032372	Cellular Component	cell part(GO:0044464)	1
OG0032372	Cellular Component	cell(GO:0005623)	1
OG0032372	Cellular Component	membrane(GO:0016020)	1
OG0032377	Cellular Component	cell part(GO:0044464)	1
OG0032377	Cellular Component	cell(GO:0005623)	1
OG0032377	Cellular Component	membrane part(GO:0044425)	1
OG0032377	Cellular Component	membrane(GO:0016020)	1
OG0032377	Cellular Component	organelle part(GO:0044422)	1
OG0032377	Cellular Component	organelle(GO:0043226)	1
OG0032377	Cellular Component	protein-containing complex(GO:0032991)	1
OG0032381	Cellular Component	cell part(GO:0044464)	1
OG0032381	Cellular Component	cell(GO:0005623)	1
OG0032381	Cellular Component	membrane(GO:0016020)	1
OG0032381	Cellular Component	organelle part(GO:0044422)	1
OG0032381	Cellular Component	organelle(GO:0043226)	1
OG0032384	Cellular Component	cell junction(GO:0030054)	1
OG0032384	Cellular Component	cell part(GO:0044464)	1
OG0032384	Cellular Component	cell(GO:0005623)	1
OG0032384	Cellular Component	organelle(GO:0043226)	1
OG0032384	Cellular Component	symplast(GO:0055044)	1
OG0032385	Cellular Component	cell part(GO:0044464)	1
OG0032385	Cellular Component	cell(GO:0005623)	1
OG0032388	Cellular Component	cell part(GO:0044464)	1
OG0032388	Cellular Component	cell(GO:0005623)	1
OG0032388	Cellular Component	extracellular region(GO:0005576)	1
OG0032388	Cellular Component	membrane(GO:0016020)	1
OG0032392	Cellular Component	cell part(GO:0044464)	1
OG0032392	Cellular Component	cell(GO:0005623)	1
OG0032392	Cellular Component	organelle(GO:0043226)	1
OG0032396	Cellular Component	cell part(GO:0044464)	1
OG0032396	Cellular Component	cell(GO:0005623)	1
OG0032396	Cellular Component	organelle(GO:0043226)	1
OG0032397	Cellular Component	cell part(GO:0044464)	1
OG0032397	Cellular Component	cell(GO:0005623)	1
OG0032397	Cellular Component	membrane(GO:0016020)	1
OG0032397	Cellular Component	organelle part(GO:0044422)	1
OG0032397	Cellular Component	organelle(GO:0043226)	1
OG0032399	Cellular Component	cell part(GO:0044464)	1
OG0032399	Cellular Component	cell(GO:0005623)	1
OG0032399	Cellular Component	organelle(GO:0043226)	1

OG0032402	Cellular Component	cell part(GO:0044464)	1
OG0032402	Cellular Component	cell(GO:0005623)	1
OG0032402	Cellular Component	membrane(GO:0016020)	1
OG0032402	Cellular Component	organelle part(GO:0044422)	1
OG0032402	Cellular Component	organelle(GO:0043226)	1
OG0032403	Cellular Component	cell part(GO:0044464)	1
OG0032403	Cellular Component	cell(GO:0005623)	1
OG0032403	Cellular Component	membrane-enclosed lumen(GO:0031974)	1
OG0032403	Cellular Component	organelle part(GO:0044422)	1
OG0032403	Cellular Component	organelle(GO:0043226)	1
OG0032403	Cellular Component	protein-containing complex(GO:0032991)	1
OG0032408	Cellular Component	cell part(GO:0044464)	1
OG0032408	Cellular Component	cell(GO:0005623)	1
OG0032413	Cellular Component	cell part(GO:0044464)	1
OG0032413	Cellular Component	cell(GO:0005623)	1
OG0032413	Cellular Component	organelle(GO:0043226)	1
OG0032414	Cellular Component	cell part(GO:0044464)	1
OG0032414	Cellular Component	cell(GO:0005623)	1
OG0032414	Cellular Component	organelle(GO:0043226)	1
OG0032415	Cellular Component	cell junction(GO:0030054)	1
OG0032415	Cellular Component	cell part(GO:0044464)	1
OG0032415	Cellular Component	cell(GO:0005623)	1
OG0032415	Cellular Component	membrane(GO:0016020)	1
OG0032415	Cellular Component	organelle(GO:0043226)	1
OG0032415	Cellular Component	symplast(GO:0055044)	1
OG0032421	Cellular Component	cell part(GO:0044464)	1
OG0032421	Cellular Component	cell(GO:0005623)	1
OG0032421	Cellular Component	organelle(GO:0043226)	1
OG0032423	Cellular Component	cell part(GO:0044464)	1
OG0032423	Cellular Component	cell(GO:0005623)	1
OG0032423	Cellular Component	membrane(GO:0016020)	1
OG0032426	Cellular Component	cell part(GO:0044464)	1
OG0032426	Cellular Component	cell(GO:0005623)	1
OG0032426	Cellular Component	organelle(GO:0043226)	1
OG0032430	Cellular Component	cell part(GO:0044464)	1
OG0032430	Cellular Component	cell(GO:0005623)	1
OG0032430	Cellular Component	membrane-enclosed lumen(GO:0031974)	1
OG0032430	Cellular Component	organelle part(GO:0044422)	1
OG0032430	Cellular Component	organelle(GO:0043226)	1
OG0032431	Cellular Component	cell part(GO:0044464)	1
OG0032431	Cellular Component	cell(GO:0005623)	1
OG0032431	Cellular Component	extracellular region part(GO:0044421)	1
OG0032431	Cellular Component	extracellular region(GO:0005576)	1
OG0032431	Cellular Component	organelle(GO:0043226)	1
OG0032437	Cellular Component	cell part(GO:0044464)	1
OG0032437	Cellular Component	cell(GO:0005623)	1
OG0032437	Cellular Component	membrane(GO:0016020)	1

OG0032437	Cellular Component	organelle part(GO:0044422)	1
OG0032437	Cellular Component	organelle(GO:0043226)	1
OG0032440	Cellular Component	cell part(GO:0044464)	1
OG0032440	Cellular Component	cell(GO:0005623)	1
OG0032440	Cellular Component	membrane(GO:0016020)	1
OG0032440	Cellular Component	organelle(GO:0043226)	1
OG0032444	Cellular Component	cell part(GO:0044464)	1
OG0032444	Cellular Component	cell(GO:0005623)	1
OG0032444	Cellular Component	extracellular region(GO:0005576)	1
OG0032444	Cellular Component	membrane part(GO:0044425)	1
OG0032444	Cellular Component	membrane(GO:0016020)	1
OG0032445	Cellular Component	cell part(GO:0044464)	1
OG0032445	Cellular Component	cell(GO:0005623)	1
OG0032445	Cellular Component	organelle(GO:0043226)	1
OG0032447	Cellular Component	cell part(GO:0044464)	1
OG0032447	Cellular Component	cell(GO:0005623)	1
OG0032447	Cellular Component	membrane(GO:0016020)	1
OG0032447	Cellular Component	organelle part(GO:0044422)	1
OG0032447	Cellular Component	organelle(GO:0043226)	1
OG0032447	Cellular Component	protein-containing complex(GO:0032991)	1
OG0032452	Cellular Component	cell part(GO:0044464)	1
OG0032452	Cellular Component	cell(GO:0005623)	1
OG0032452	Cellular Component	membrane(GO:0016020)	1
OG0032452	Cellular Component	organelle(GO:0043226)	1
OG0032461	Cellular Component	cell part(GO:0044464)	1
OG0032461	Cellular Component	cell(GO:0005623)	1
OG0032461	Cellular Component	organelle(GO:0043226)	1
OG0032464	Cellular Component	cell part(GO:0044464)	1
OG0032464	Cellular Component	cell(GO:0005623)	1
OG0032464	Cellular Component	membrane part(GO:0044425)	1
OG0032464	Cellular Component	membrane(GO:0016020)	1
OG0032464	Cellular Component	organelle(GO:0043226)	1
OG0032466	Cellular Component	cell part(GO:0044464)	1
OG0032466	Cellular Component	cell(GO:0005623)	1
OG0032468	Cellular Component	cell part(GO:0044464)	1
OG0032468	Cellular Component	cell(GO:0005623)	1
OG0032468	Cellular Component	membrane(GO:0016020)	1
OG0032473	Cellular Component	cell part(GO:0044464)	1
OG0032473	Cellular Component	cell(GO:0005623)	1
OG0032473	Cellular Component	organelle(GO:0043226)	1
OG0032474	Cellular Component	cell part(GO:0044464)	1
OG0032474	Cellular Component	cell(GO:0005623)	1
OG0032474	Cellular Component	extracellular region(GO:0005576)	1
OG0032474	Cellular Component	organelle part(GO:0044422)	1
OG0032474	Cellular Component	organelle(GO:0043226)	1
OG0032476	Cellular Component	cell part(GO:0044464)	1
OG0032476	Cellular Component	cell(GO:0005623)	1

OG0032476	Cellular Component	organelle(GO:0043226)	1
OG0032479	Cellular Component	cell part(GO:0044464)	1
OG0032479	Cellular Component	cell(GO:0005623)	1
OG0032479	Cellular Component	membrane(GO:0016020)	1
OG0032479	Cellular Component	organelle part(GO:0044422)	1
OG0032479	Cellular Component	organelle(GO:0043226)	1
OG0032482	Cellular Component	cell part(GO:0044464)	1
OG0032482	Cellular Component	cell(GO:0005623)	1
OG0032482	Cellular Component	organelle(GO:0043226)	1
OG0032483	Cellular Component	cell part(GO:0044464)	1
OG0032483	Cellular Component	cell(GO:0005623)	1
OG0032483	Cellular Component	organelle(GO:0043226)	1
OG0032491	Cellular Component	cell part(GO:0044464)	1
OG0032491	Cellular Component	cell(GO:0005623)	1
OG0032491	Cellular Component	membrane(GO:0016020)	1
OG0032492	Cellular Component	cell part(GO:0044464)	1
OG0032492	Cellular Component	cell(GO:0005623)	1
OG0032492	Cellular Component	membrane(GO:0016020)	1
OG0032501	Cellular Component	cell part(GO:0044464)	1
OG0032501	Cellular Component	cell(GO:0005623)	1
OG0032501	Cellular Component	membrane(GO:0016020)	1
OG0032503	Cellular Component	cell part(GO:0044464)	1
OG0032503	Cellular Component	cell(GO:0005623)	1
OG0032503	Cellular Component	membrane(GO:0016020)	1
OG0032503	Cellular Component	organelle part(GO:0044422)	1
OG0032503	Cellular Component	organelle(GO:0043226)	1
OG0032506	Cellular Component	cell junction(GO:0030054)	1
OG0032506	Cellular Component	cell part(GO:0044464)	1
OG0032506	Cellular Component	cell(GO:0005623)	1
OG0032506	Cellular Component	symplast(GO:0055044)	1
OG0032507	Cellular Component	cell part(GO:0044464)	1
OG0032507	Cellular Component	cell(GO:0005623)	1
OG0032512	Cellular Component	cell part(GO:0044464)	1
OG0032512	Cellular Component	cell(GO:0005623)	1
OG0032512	Cellular Component	membrane(GO:0016020)	1
OG0032529	Cellular Component	cell part(GO:0044464)	1
OG0032529	Cellular Component	cell(GO:0005623)	1
OG0032529	Cellular Component	organelle(GO:0043226)	1
OG0032530	Cellular Component	cell part(GO:0044464)	1
OG0032530	Cellular Component	cell(GO:0005623)	1
OG0032530	Cellular Component	membrane(GO:0016020)	1
OG0032530	Cellular Component	organelle(GO:0043226)	1
OG0032532	Cellular Component	cell part(GO:0044464)	1
OG0032532	Cellular Component	cell(GO:0005623)	1
OG0032532	Cellular Component	extracellular region(GO:0005576)	1
OG0032532	Cellular Component	organelle part(GO:0044422)	1
OG0032532	Cellular Component	organelle(GO:0043226)	1

OG0032534	Cellular Component	cell part(GO:0044464)	1
OG0032534	Cellular Component	cell(GO:0005623)	1
OG0032534	Cellular Component	membrane(GO:0016020)	1
OG0032534	Cellular Component	organelle part(GO:0044422)	1
OG0032534	Cellular Component	organelle(GO:0043226)	1
OG0032536	Cellular Component	cell part(GO:0044464)	1
OG0032536	Cellular Component	cell(GO:0005623)	1
OG0032536	Cellular Component	extracellular region(GO:0005576)	1
OG0032536	Cellular Component	organelle part(GO:0044422)	1
OG0032536	Cellular Component	organelle(GO:0043226)	1
OG0032538	Cellular Component	cell part(GO:0044464)	1
OG0032538	Cellular Component	cell(GO:0005623)	1
OG0032538	Cellular Component	extracellular region(GO:0005576)	1
OG0032538	Cellular Component	organelle part(GO:0044422)	1
OG0032538	Cellular Component	organelle(GO:0043226)	1
OG0032540	Cellular Component	cell part(GO:0044464)	1
OG0032540	Cellular Component	cell(GO:0005623)	1
OG0032540	Cellular Component	organelle(GO:0043226)	1
OG0032541	Cellular Component	cell part(GO:0044464)	1
OG0032541	Cellular Component	cell(GO:0005623)	1
OG0032541	Cellular Component	protein-containing complex(GO:0032991)	1
OG0032544	Cellular Component	cell part(GO:0044464)	1
OG0032544	Cellular Component	cell(GO:0005623)	1
OG0032544	Cellular Component	protein-containing complex(GO:0032991)	1
OG0032547	Cellular Component	cell part(GO:0044464)	1
OG0032547	Cellular Component	cell(GO:0005623)	1
OG0032547	Cellular Component	membrane(GO:0016020)	1
OG0032547	Cellular Component	organelle part(GO:0044422)	1
OG0032547	Cellular Component	organelle(GO:0043226)	1
OG0032548	Cellular Component	cell part(GO:0044464)	1
OG0032548	Cellular Component	cell(GO:0005623)	1
OG0032548	Cellular Component	membrane(GO:0016020)	1
OG0032551	Cellular Component	cell part(GO:0044464)	1
OG0032551	Cellular Component	cell(GO:0005623)	1
OG0032551	Cellular Component	membrane(GO:0016020)	1
OG0032559	Cellular Component	cell part(GO:0044464)	1
OG0032559	Cellular Component	cell(GO:0005623)	1
OG0032559	Cellular Component	organelle(GO:0043226)	1
OG0032561	Cellular Component	cell part(GO:0044464)	1
OG0032561	Cellular Component	cell(GO:0005623)	1
OG0032561	Cellular Component	organelle part(GO:0044422)	1
OG0032561	Cellular Component	organelle(GO:0043226)	1
OG0032562	Cellular Component	cell part(GO:0044464)	1
OG0032562	Cellular Component	cell(GO:0005623)	1
OG0032562	Cellular Component	extracellular region(GO:0005576)	1
OG0032563	Cellular Component	cell part(GO:0044464)	1
OG0032563	Cellular Component	cell(GO:0005623)	1

OG0032563	Cellular Component	organelle part(GO:0044422)	1
OG0032563	Cellular Component	organelle(GO:0043226)	1
OG0032563	Cellular Component	protein-containing complex(GO:0032991)	1
OG0032572	Cellular Component	cell part(GO:0044464)	1
OG0032572	Cellular Component	cell(GO:0005623)	1
OG0032572	Cellular Component	extracellular region(GO:0005576)	1
OG0032572	Cellular Component	membrane(GO:0016020)	1
OG0032575	Cellular Component	cell part(GO:0044464)	1
OG0032575	Cellular Component	cell(GO:0005623)	1
OG0032576	Cellular Component	cell part(GO:0044464)	1
OG0032576	Cellular Component	cell(GO:0005623)	1
OG0032576	Cellular Component	extracellular region(GO:0005576)	1
OG0032578	Cellular Component	cell part(GO:0044464)	1
OG0032578	Cellular Component	cell(GO:0005623)	1
OG0032578	Cellular Component	membrane(GO:0016020)	1
OG0032579	Cellular Component	cell part(GO:0044464)	1
OG0032579	Cellular Component	cell(GO:0005623)	1
OG0032579	Cellular Component	organelle part(GO:0044422)	1
OG0032579	Cellular Component	organelle(GO:0043226)	1
OG0032579	Cellular Component	protein-containing complex(GO:0032991)	1
OG0032590	Cellular Component	cell part(GO:0044464)	1
OG0032590	Cellular Component	cell(GO:0005623)	1
OG0032590	Cellular Component	membrane part(GO:0044425)	1
OG0032590	Cellular Component	membrane(GO:0016020)	1
OG0032590	Cellular Component	organelle part(GO:0044422)	1
OG0032590	Cellular Component	organelle(GO:0043226)	1
OG0032596	Cellular Component	cell part(GO:0044464)	1
OG0032596	Cellular Component	cell(GO:0005623)	1
OG0032596	Cellular Component	membrane(GO:0016020)	1
OG0032596	Cellular Component	organelle(GO:0043226)	1
OG0032598	Cellular Component	cell part(GO:0044464)	1
OG0032598	Cellular Component	cell(GO:0005623)	1
OG0032598	Cellular Component	membrane-enclosed lumen(GO:0031974)	1
OG0032598	Cellular Component	organelle part(GO:0044422)	1
OG0032598	Cellular Component	organelle(GO:0043226)	1
OG0032598	Cellular Component	protein-containing complex(GO:0032991)	1
OG0032616	Cellular Component	cell part(GO:0044464)	1
OG0032616	Cellular Component	cell(GO:0005623)	1
OG0032616	Cellular Component	organelle(GO:0043226)	1
OG0032626	Cellular Component	cell part(GO:0044464)	1
OG0032626	Cellular Component	cell(GO:0005623)	1
OG0032626	Cellular Component	membrane-enclosed lumen(GO:0031974)	1
OG0032626	Cellular Component	organelle part(GO:0044422)	1
OG0032626	Cellular Component	organelle(GO:0043226)	1
OG0032626	Cellular Component	protein-containing complex(GO:0032991)	1
OG0032627	Cellular Component	cell part(GO:0044464)	1

OG0032627	Cellular Component	cell(GO:0005623)	1
OG0032627	Cellular Component	membrane(GO:0016020)	1
OG0032632	Cellular Component	cell part(GO:0044464)	1
OG0032632	Cellular Component	cell(GO:0005623)	1
OG0032637	Cellular Component	cell part(GO:0044464)	1
OG0032637	Cellular Component	cell(GO:0005623)	1
OG0032637	Cellular Component	membrane(GO:0016020)	1
OG0032639	Cellular Component	cell part(GO:0044464)	1
OG0032639	Cellular Component	cell(GO:0005623)	1
OG0032639	Cellular Component	membrane(GO:0016020)	1
OG0032656	Cellular Component	cell part(GO:0044464)	1
OG0032656	Cellular Component	cell(GO:0005623)	1
OG0032656	Cellular Component	protein-containing complex(GO:0032991)	1
OG0032662	Cellular Component	cell part(GO:0044464)	1
OG0032662	Cellular Component	cell(GO:0005623)	1
OG0032666	Cellular Component	cell part(GO:0044464)	1
OG0032666	Cellular Component	cell(GO:0005623)	1
OG0032667	Cellular Component	cell part(GO:0044464)	1
OG0032667	Cellular Component	cell(GO:0005623)	1
OG0032667	Cellular Component	membrane(GO:0016020)	1
OG0032671	Cellular Component	cell part(GO:0044464)	1
OG0032671	Cellular Component	cell(GO:0005623)	1
OG0032671	Cellular Component	membrane(GO:0016020)	1
OG0032673	Cellular Component	cell part(GO:0044464)	1
OG0032673	Cellular Component	cell(GO:0005623)	1
OG0032673	Cellular Component	protein-containing complex(GO:0032991)	1
OG0032675	Cellular Component	cell part(GO:0044464)	1
OG0032675	Cellular Component	cell(GO:0005623)	1
OG0032675	Cellular Component	membrane part(GO:0044425)	1
OG0032675	Cellular Component	membrane(GO:0016020)	1
OG0032675	Cellular Component	organelle part(GO:0044422)	1
OG0032675	Cellular Component	organelle(GO:0043226)	1
OG0032675	Cellular Component	protein-containing complex(GO:0032991)	1
OG0032677	Cellular Component	cell part(GO:0044464)	1
OG0032677	Cellular Component	cell(GO:0005623)	1
OG0032677	Cellular Component	membrane part(GO:0044425)	1
OG0032677	Cellular Component	membrane(GO:0016020)	1
OG0032677	Cellular Component	organelle part(GO:0044422)	1
OG0032677	Cellular Component	organelle(GO:0043226)	1
OG0032677	Cellular Component	protein-containing complex(GO:0032991)	1
OG0032681	Cellular Component	cell part(GO:0044464)	1
OG0032681	Cellular Component	cell(GO:0005623)	1
OG0032681	Cellular Component	nucleoid(GO:0009295)	1
OG0032693	Cellular Component	cell part(GO:0044464)	1
OG0032693	Cellular Component	cell(GO:0005623)	1
OG0032693	Cellular Component	organelle part(GO:0044422)	1

OG0032693	Cellular Component	organelle(GO:0043226)	1
OG0032694	Cellular Component	cell part(GO:0044464)	1
OG0032694	Cellular Component	cell(GO:0005623)	1
OG0032694	Cellular Component	organelle part(GO:0044422)	1
OG0032694	Cellular Component	organelle(GO:0043226)	1
OG0032700	Cellular Component	cell part(GO:0044464)	1
OG0032700	Cellular Component	cell(GO:0005623)	1
OG0032701	Cellular Component	membrane part(GO:0044425)	1
OG0032701	Cellular Component	membrane(GO:0016020)	1
OG0032702	Cellular Component	cell part(GO:0044464)	1
OG0032702	Cellular Component	cell(GO:0005623)	1
OG0032702	Cellular Component	membrane part(GO:0044425)	1
OG0032702	Cellular Component	membrane(GO:0016020)	1
OG0032704	Cellular Component	cell part(GO:0044464)	1
OG0032704	Cellular Component	cell(GO:0005623)	1
OG0032704	Cellular Component	protein-containing complex(GO:0032991)	1
OG0032707	Cellular Component	cell part(GO:0044464)	1
OG0032707	Cellular Component	cell(GO:0005623)	1
OG0032709	Cellular Component	cell part(GO:0044464)	1
OG0032709	Cellular Component	cell(GO:0005623)	1
OG0032710	Cellular Component	cell part(GO:0044464)	1
OG0032710	Cellular Component	cell(GO:0005623)	1
OG0032710	Cellular Component	protein-containing complex(GO:0032991)	1
OG0032717	Cellular Component	cell part(GO:0044464)	1
OG0032717	Cellular Component	cell(GO:0005623)	1
OG0032719	Cellular Component	cell part(GO:0044464)	1
OG0032719	Cellular Component	cell(GO:0005623)	1
OG0032719	Cellular Component	membrane(GO:0016020)	1
OG0032719	Cellular Component	organelle part(GO:0044422)	1
OG0032719	Cellular Component	organelle(GO:0043226)	1
OG0032721	Cellular Component	cell part(GO:0044464)	1
OG0032721	Cellular Component	cell(GO:0005623)	1
OG0032721	Cellular Component	membrane(GO:0016020)	1
OG0032723	Cellular Component	cell part(GO:0044464)	1
OG0032723	Cellular Component	cell(GO:0005623)	1
OG0032725	Cellular Component	cell part(GO:0044464)	1
OG0032725	Cellular Component	cell(GO:0005623)	1
OG0032725	Cellular Component	extracellular region part(GO:0044421)	1
OG0032725	Cellular Component	extracellular region(GO:0005576)	1
OG0032725	Cellular Component	membrane(GO:0016020)	1
OG0032725	Cellular Component	other organism part(GO:0044217)	1
OG0032725	Cellular Component	other organism(GO:0044215)	1
OG0032727	Cellular Component	cell part(GO:0044464)	1
OG0032727	Cellular Component	cell(GO:0005623)	1
OG0032730	Cellular Component	cell part(GO:0044464)	1
OG0032730	Cellular Component	cell(GO:0005623)	1

OG0032730	Cellular Component	membrane(GO:0016020)	1
OG0032740	Cellular Component	cell part(GO:0044464)	1
OG0032740	Cellular Component	cell(GO:0005623)	1
OG0032745	Cellular Component	cell part(GO:0044464)	1
OG0032745	Cellular Component	cell(GO:0005623)	1
OG0032745	Cellular Component	membrane(GO:0016020)	1
OG0032751	Cellular Component	cell part(GO:0044464)	1
OG0032751	Cellular Component	cell(GO:0005623)	1
OG0032751	Cellular Component	membrane(GO:0016020)	1
OG0032752	Cellular Component	cell part(GO:0044464)	1
OG0032752	Cellular Component	cell(GO:0005623)	1
OG0032752	Cellular Component	membrane(GO:0016020)	1
OG0032755	Cellular Component	cell part(GO:0044464)	1
OG0032755	Cellular Component	cell(GO:0005623)	1
OG0032755	Cellular Component	membrane(GO:0016020)	1
OG0032756	Cellular Component	cell part(GO:0044464)	1
OG0032756	Cellular Component	cell(GO:0005623)	1
OG0032756	Cellular Component	membrane(GO:0016020)	1
OG0032757	Cellular Component	cell part(GO:0044464)	1
OG0032757	Cellular Component	cell(GO:0005623)	1
OG0032757	Cellular Component	membrane(GO:0016020)	1
OG0032759	Cellular Component	cell part(GO:0044464)	1
OG0032759	Cellular Component	cell(GO:0005623)	1
OG0032759	Cellular Component	extracellular region(GO:0005576)	1
OG0032759	Cellular Component	membrane(GO:0016020)	1
OG0032765	Cellular Component	membrane(GO:0016020)	1
OG0032766	Cellular Component	cell part(GO:0044464)	1
OG0032766	Cellular Component	cell(GO:0005623)	1
OG0032766	Cellular Component	membrane(GO:0016020)	1
OG0032768	Cellular Component	membrane(GO:0016020)	1
OG0032770	Cellular Component	cell part(GO:0044464)	1
OG0032770	Cellular Component	cell(GO:0005623)	1
OG0032770	Cellular Component	organelle part(GO:0044422)	1
OG0032770	Cellular Component	organelle(GO:0043226)	1
OG0032771	Cellular Component	cell part(GO:0044464)	1
OG0032771	Cellular Component	cell(GO:0005623)	1
OG0032771	Cellular Component	membrane part(GO:0044425)	1
OG0032771	Cellular Component	membrane(GO:0016020)	1
OG0032771	Cellular Component	organelle part(GO:0044422)	1
OG0032771	Cellular Component	organelle(GO:0043226)	1
OG0032778	Cellular Component	cell part(GO:0044464)	1
OG0032778	Cellular Component	cell(GO:0005623)	1
OG0032778	Cellular Component	organelle(GO:0043226)	1
OG0032779	Cellular Component	cell part(GO:0044464)	1
OG0032779	Cellular Component	cell(GO:0005623)	1
OG0032779	Cellular Component	organelle(GO:0043226)	1
OG0032780	Cellular Component	cell part(GO:0044464)	1

OG0032780	Cellular Component	cell(GO:0005623)	1
OG0032780	Cellular Component	organelle(GO:0043226)	1
OG0032787	Cellular Component	cell junction(GO:0030054)	1
OG0032787	Cellular Component	cell part(GO:0044464)	1
OG0032787	Cellular Component	cell(GO:0005623)	1
OG0032787	Cellular Component	membrane(GO:0016020)	1
OG0032787	Cellular Component	symplast(GO:0055044)	1
OG0032789	Cellular Component	extracellular region(GO:0005576)	1
OG0032794	Cellular Component	cell part(GO:0044464)	1
OG0032794	Cellular Component	cell(GO:0005623)	1
OG0032794	Cellular Component	membrane part(GO:0044425)	1
OG0032794	Cellular Component	membrane(GO:0016020)	1
OG0032794	Cellular Component	organelle part(GO:0044422)	1
OG0032794	Cellular Component	organelle(GO:0043226)	1
OG0032794	Cellular Component	protein-containing complex(GO:0032991)	1
OG0032799	Cellular Component	cell junction(GO:0030054)	1
OG0032799	Cellular Component	cell part(GO:0044464)	1
OG0032799	Cellular Component	cell(GO:0005623)	1
OG0032799	Cellular Component	membrane(GO:0016020)	1
OG0032799	Cellular Component	symplast(GO:0055044)	1
OG0032802	Cellular Component	cell part(GO:0044464)	1
OG0032802	Cellular Component	cell(GO:0005623)	1
OG0032802	Cellular Component	extracellular region(GO:0005576)	1
OG0032802	Cellular Component	membrane(GO:0016020)	1
OG0032803	Cellular Component	cell part(GO:0044464)	1
OG0032803	Cellular Component	cell(GO:0005623)	1
OG0032806	Cellular Component	cell part(GO:0044464)	1
OG0032806	Cellular Component	cell(GO:0005623)	1
OG0032806	Cellular Component	membrane(GO:0016020)	1
OG0032808	Cellular Component	cell part(GO:0044464)	1
OG0032808	Cellular Component	cell(GO:0005623)	1
OG0032808	Cellular Component	extracellular region(GO:0005576)	1
OG0032808	Cellular Component	membrane(GO:0016020)	1
OG0032809	Cellular Component	cell part(GO:0044464)	1
OG0032809	Cellular Component	cell(GO:0005623)	1
OG0032809	Cellular Component	membrane(GO:0016020)	1
OG0032821	Cellular Component	cell part(GO:0044464)	1
OG0032821	Cellular Component	cell(GO:0005623)	1
OG0032821	Cellular Component	membrane(GO:0016020)	1
OG0032822	Cellular Component	cell part(GO:0044464)	1
OG0032822	Cellular Component	cell(GO:0005623)	1
OG0032824	Cellular Component	cell part(GO:0044464)	1
OG0032824	Cellular Component	cell(GO:0005623)	1
OG0032824	Cellular Component	organelle(GO:0043226)	1
OG0032826	Cellular Component	membrane(GO:0016020)	1
OG0032829	Cellular Component	cell part(GO:0044464)	1
OG0032829	Cellular Component	cell(GO:0005623)	1

OG0032829	Cellular Component	organelle(GO:0043226)	1
OG0032830	Cellular Component	cell part(GO:0044464)	1
OG0032830	Cellular Component	cell(GO:0005623)	1
OG0032840	Cellular Component	cell part(GO:0044464)	1
OG0032840	Cellular Component	cell(GO:0005623)	1
OG0032840	Cellular Component	extracellular region(GO:0005576)	1
OG0032847	Cellular Component	cell part(GO:0044464)	1
OG0032847	Cellular Component	cell(GO:0005623)	1
OG0032847	Cellular Component	membrane(GO:0016020)	1
OG0032847	Cellular Component	organelle part(GO:0044422)	1
OG0032847	Cellular Component	organelle(GO:0043226)	1
OG0032847	Cellular Component	protein-containing complex(GO:0032991)	1
OG0032848	Cellular Component	cell part(GO:0044464)	1
OG0032848	Cellular Component	cell(GO:0005623)	1
OG0032848	Cellular Component	extracellular region(GO:0005576)	1
OG0032848	Cellular Component	membrane(GO:0016020)	1
OG0032848	Cellular Component	membrane-enclosed lumen(GO:0031974)	1
OG0032848	Cellular Component	organelle part(GO:0044422)	1
OG0032848	Cellular Component	organelle(GO:0043226)	1
OG0032850	Cellular Component	cell part(GO:0044464)	1
OG0032850	Cellular Component	cell(GO:0005623)	1
OG0032850	Cellular Component	membrane-enclosed lumen(GO:0031974)	1
OG0032850	Cellular Component	organelle part(GO:0044422)	1
OG0032850	Cellular Component	organelle(GO:0043226)	1
OG0032853	Cellular Component	cell part(GO:0044464)	1
OG0032853	Cellular Component	cell(GO:0005623)	1
OG0032853	Cellular Component	membrane(GO:0016020)	1
OG0032853	Cellular Component	organelle part(GO:0044422)	1
OG0032853	Cellular Component	organelle(GO:0043226)	1
OG0032855	Cellular Component	cell part(GO:0044464)	1
OG0032855	Cellular Component	cell(GO:0005623)	1
OG0032855	Cellular Component	organelle(GO:0043226)	1
OG0032855	Cellular Component	protein-containing complex(GO:0032991)	1
OG0032856	Cellular Component	cell part(GO:0044464)	1
OG0032856	Cellular Component	cell(GO:0005623)	1
OG0032856	Cellular Component	membrane(GO:0016020)	1
OG0032856	Cellular Component	organelle part(GO:0044422)	1
OG0032856	Cellular Component	organelle(GO:0043226)	1
OG0032857	Cellular Component	cell part(GO:0044464)	1
OG0032857	Cellular Component	cell(GO:0005623)	1
OG0032863	Cellular Component	cell part(GO:0044464)	1
OG0032863	Cellular Component	cell(GO:0005623)	1
OG0032863	Cellular Component	membrane(GO:0016020)	1
OG0032863	Cellular Component	organelle part(GO:0044422)	1
OG0032863	Cellular Component	organelle(GO:0043226)	1
OG0032868	Cellular Component	cell part(GO:0044464)	1
OG0032868	Cellular Component	cell(GO:0005623)	1

OG0032868	Cellular Component	organelle(GO:0043226)	1
OG0032870	Cellular Component	cell part(GO:0044464)	1
OG0032870	Cellular Component	cell(GO:0005623)	1
OG0032870	Cellular Component	membrane(GO:0016020)	1
OG0032870	Cellular Component	organelle part(GO:0044422)	1
OG0032870	Cellular Component	organelle(GO:0043226)	1
OG0032880	Cellular Component	cell part(GO:0044464)	1
OG0032880	Cellular Component	cell(GO:0005623)	1
OG0032880	Cellular Component	membrane(GO:0016020)	1
OG0032881	Cellular Component	cell part(GO:0044464)	1
OG0032881	Cellular Component	cell(GO:0005623)	1
OG0032881	Cellular Component	organelle(GO:0043226)	1
OG0032885	Cellular Component	cell part(GO:0044464)	1
OG0032885	Cellular Component	cell(GO:0005623)	1
OG0032885	Cellular Component	organelle part(GO:0044422)	1
OG0032885	Cellular Component	organelle(GO:0043226)	1
OG0032886	Cellular Component	cell part(GO:0044464)	1
OG0032886	Cellular Component	cell(GO:0005623)	1
OG0032886	Cellular Component	membrane(GO:0016020)	1
OG0032887	Cellular Component	cell part(GO:0044464)	1
OG0032887	Cellular Component	cell(GO:0005623)	1
OG0032887	Cellular Component	organelle part(GO:0044422)	1
OG0032887	Cellular Component	organelle(GO:0043226)	1
OG0032889	Cellular Component	cell part(GO:0044464)	1
OG0032889	Cellular Component	cell(GO:0005623)	1
OG0032889	Cellular Component	membrane part(GO:0044425)	1
OG0032889	Cellular Component	membrane(GO:0016020)	1
OG0032891	Cellular Component	cell part(GO:0044464)	1
OG0032891	Cellular Component	cell(GO:0005623)	1
OG0032894	Cellular Component	cell part(GO:0044464)	1
OG0032894	Cellular Component	cell(GO:0005623)	1
OG0032896	Cellular Component	cell part(GO:0044464)	1
OG0032896	Cellular Component	cell(GO:0005623)	1
OG0032896	Cellular Component	membrane(GO:0016020)	1
OG0032909	Cellular Component	cell part(GO:0044464)	1
OG0032909	Cellular Component	cell(GO:0005623)	1
OG0032910	Cellular Component	cell junction(GO:0030054)	1
OG0032910	Cellular Component	cell part(GO:0044464)	1
OG0032910	Cellular Component	cell(GO:0005623)	1
OG0032910	Cellular Component	membrane(GO:0016020)	1
OG0032910	Cellular Component	organelle part(GO:0044422)	1
OG0032910	Cellular Component	organelle(GO:0043226)	1
OG0032910	Cellular Component	symplast(GO:0055044)	1
OG0032913	Cellular Component	cell part(GO:0044464)	1
OG0032913	Cellular Component	cell(GO:0005623)	1
OG0032913	Cellular Component	membrane(GO:0016020)	1
OG0032913	Cellular Component	organelle part(GO:0044422)	1

OG0032913	Cellular Component	organelle(GO:0043226)	1
OG0032915	Cellular Component	cell part(GO:0044464)	1
OG0032915	Cellular Component	cell(GO:0005623)	1
OG0032915	Cellular Component	organelle(GO:0043226)	1
OG0032917	Cellular Component	cell junction(GO:0030054)	1
OG0032917	Cellular Component	cell part(GO:0044464)	1
OG0032917	Cellular Component	cell(GO:0005623)	1
OG0032917	Cellular Component	membrane part(GO:0044425)	1
OG0032917	Cellular Component	membrane(GO:0016020)	1
OG0032917	Cellular Component	symplast(GO:0055044)	1
OG0032920	Cellular Component	cell part(GO:0044464)	1
OG0032920	Cellular Component	cell(GO:0005623)	1
OG0032920	Cellular Component	organelle(GO:0043226)	1
OG0032922	Cellular Component	cell part(GO:0044464)	1
OG0032922	Cellular Component	cell(GO:0005623)	1
OG0032922	Cellular Component	organelle(GO:0043226)	1
OG0032926	Cellular Component	cell part(GO:0044464)	1
OG0032926	Cellular Component	cell(GO:0005623)	1
OG0032926	Cellular Component	organelle(GO:0043226)	1
OG0032928	Cellular Component	cell part(GO:0044464)	1
OG0032928	Cellular Component	cell(GO:0005623)	1
OG0032932	Cellular Component	cell part(GO:0044464)	1
OG0032932	Cellular Component	cell(GO:0005623)	1
OG0032932	Cellular Component	membrane(GO:0016020)	1
OG0032939	Cellular Component	membrane(GO:0016020)	1
OG0032942	Cellular Component	cell part(GO:0044464)	1
OG0032942	Cellular Component	cell(GO:0005623)	1
OG0032942	Cellular Component	extracellular region(GO:0005576)	1
OG0032942	Cellular Component	membrane(GO:0016020)	1
OG0032942	Cellular Component	organelle part(GO:0044422)	1
OG0032942	Cellular Component	organelle(GO:0043226)	1
OG0032949	Cellular Component	cell junction(GO:0030054)	1
OG0032949	Cellular Component	cell part(GO:0044464)	1
OG0032949	Cellular Component	cell(GO:0005623)	1
OG0032949	Cellular Component	membrane(GO:0016020)	1
OG0032949	Cellular Component	membrane-enclosed lumen(GO:0031974)	1
OG0032949	Cellular Component	organelle part(GO:0044422)	1
OG0032949	Cellular Component	organelle(GO:0043226)	1
OG0032949	Cellular Component	protein-containing complex(GO:0032991)	1
OG0032949	Cellular Component	symplast(GO:0055044)	1
OG0032950	Cellular Component	cell junction(GO:0030054)	1
OG0032950	Cellular Component	cell part(GO:0044464)	1
OG0032950	Cellular Component	cell(GO:0005623)	1
OG0032950	Cellular Component	membrane(GO:0016020)	1
OG0032950	Cellular Component	membrane-enclosed lumen(GO:0031974)	1
OG0032950	Cellular Component	organelle part(GO:0044422)	1
OG0032950	Cellular Component	organelle(GO:0043226)	1

OG0032950	Cellular Component	protein-containing complex(GO:0032991)	1
OG0032950	Cellular Component	symplast(GO:0055044)	1
OG0032951	Cellular Component	cell part(GO:0044464)	1
OG0032951	Cellular Component	cell(GO:0005623)	1
OG0032951	Cellular Component	membrane(GO:0016020)	1
OG0032951	Cellular Component	organelle(GO:0043226)	1
OG0032954	Cellular Component	cell part(GO:0044464)	1
OG0032954	Cellular Component	cell(GO:0005623)	1
OG0032954	Cellular Component	membrane(GO:0016020)	1
OG0032954	Cellular Component	organelle part(GO:0044422)	1
OG0032954	Cellular Component	organelle(GO:0043226)	1
OG0032956	Cellular Component	cell part(GO:0044464)	1
OG0032956	Cellular Component	cell(GO:0005623)	1
OG0032957	Cellular Component	cell part(GO:0044464)	1
OG0032957	Cellular Component	cell(GO:0005623)	1
OG0032957	Cellular Component	membrane(GO:0016020)	1
OG0032970	Cellular Component	membrane(GO:0016020)	1
OG0032974	Cellular Component	cell part(GO:0044464)	1
OG0032974	Cellular Component	cell(GO:0005623)	1
OG0032975	Cellular Component	cell part(GO:0044464)	1
OG0032975	Cellular Component	cell(GO:0005623)	1
OG0032975	Cellular Component	extracellular region(GO:0005576)	1
OG0032975	Cellular Component	membrane(GO:0016020)	1
OG0032975	Cellular Component	organelle part(GO:0044422)	1
OG0032975	Cellular Component	organelle(GO:0043226)	1
OG0032983	Cellular Component	cell part(GO:0044464)	1
OG0032983	Cellular Component	cell(GO:0005623)	1
OG0032983	Cellular Component	organelle(GO:0043226)	1
OG0032984	Cellular Component	cell part(GO:0044464)	1
OG0032984	Cellular Component	cell(GO:0005623)	1
OG0032984	Cellular Component	organelle(GO:0043226)	1
OG0032986	Cellular Component	cell part(GO:0044464)	1
OG0032986	Cellular Component	cell(GO:0005623)	1
OG0032986	Cellular Component	organelle(GO:0043226)	1
OG0032987	Cellular Component	cell part(GO:0044464)	1
OG0032987	Cellular Component	cell(GO:0005623)	1
OG0032987	Cellular Component	membrane(GO:0016020)	1
OG0032988	Cellular Component	cell part(GO:0044464)	1
OG0032988	Cellular Component	cell(GO:0005623)	1
OG0032988	Cellular Component	membrane part(GO:0044425)	1
OG0032988	Cellular Component	membrane(GO:0016020)	1
OG0032988	Cellular Component	organelle part(GO:0044422)	1
OG0032988	Cellular Component	organelle(GO:0043226)	1
OG0032989	Cellular Component	cell part(GO:0044464)	1
OG0032989	Cellular Component	cell(GO:0005623)	1
OG0032989	Cellular Component	membrane-enclosed lumen(GO:0031974)	1
OG0032989	Cellular Component	organelle part(GO:0044422)	1

OG0032989	Cellular Component	organelle(GO:0043226)	1
OG0032991	Cellular Component	membrane(GO:0016020)	1
OG0032995	Cellular Component	cell part(GO:0044464)	1
OG0032995	Cellular Component	cell(GO:0005623)	1
OG0032995	Cellular Component	organelle(GO:0043226)	1
OG0033002	Cellular Component	cell part(GO:0044464)	1
OG0033002	Cellular Component	cell(GO:0005623)	1
OG0033002	Cellular Component	membrane part(GO:0044425)	1
OG0033002	Cellular Component	membrane(GO:0016020)	1
OG0033003	Cellular Component	cell part(GO:0044464)	1
OG0033003	Cellular Component	cell(GO:0005623)	1
OG0033003	Cellular Component	membrane(GO:0016020)	1
OG0033006	Cellular Component	cell part(GO:0044464)	1
OG0033006	Cellular Component	cell(GO:0005623)	1
OG0033006	Cellular Component	membrane(GO:0016020)	1
OG0033012	Cellular Component	cell part(GO:0044464)	1
OG0033012	Cellular Component	cell(GO:0005623)	1
OG0033012	Cellular Component	membrane part(GO:0044425)	1
OG0033012	Cellular Component	membrane(GO:0016020)	1
OG0033012	Cellular Component	organelle part(GO:0044422)	1
OG0033012	Cellular Component	organelle(GO:0043226)	1
OG0033012	Cellular Component	protein-containing complex(GO:0032991)	1
OG0033013	Cellular Component	cell part(GO:0044464)	1
OG0033013	Cellular Component	cell(GO:0005623)	1
OG0033014	Cellular Component	cell part(GO:0044464)	1
OG0033014	Cellular Component	cell(GO:0005623)	1
OG0033016	Cellular Component	cell part(GO:0044464)	1
OG0033016	Cellular Component	cell(GO:0005623)	1
OG0033016	Cellular Component	membrane(GO:0016020)	1
OG0033017	Cellular Component	cell part(GO:0044464)	1
OG0033017	Cellular Component	cell(GO:0005623)	1
OG0033017	Cellular Component	membrane(GO:0016020)	1
OG0033018	Cellular Component	cell part(GO:0044464)	1
OG0033018	Cellular Component	cell(GO:0005623)	1
OG0033018	Cellular Component	membrane(GO:0016020)	1
OG0033019	Cellular Component	cell part(GO:0044464)	1
OG0033019	Cellular Component	cell(GO:0005623)	1
OG0033019	Cellular Component	membrane part(GO:0044425)	1
OG0033019	Cellular Component	membrane(GO:0016020)	1
OG0033021	Cellular Component	cell part(GO:0044464)	1
OG0033021	Cellular Component	cell(GO:0005623)	1
OG0033021	Cellular Component	membrane(GO:0016020)	1
OG0033022	Cellular Component	cell part(GO:0044464)	1
OG0033022	Cellular Component	cell(GO:0005623)	1
OG0033026	Cellular Component	cell part(GO:0044464)	1
OG0033026	Cellular Component	cell(GO:0005623)	1
OG0033026	Cellular Component	membrane(GO:0016020)	1

OG0033027	Cellular Component	cell part(GO:0044464)	1
OG0033027	Cellular Component	cell(GO:0005623)	1
OG0033027	Cellular Component	membrane(GO:0016020)	1
OG0033030	Cellular Component	cell part(GO:0044464)	1
OG0033030	Cellular Component	cell(GO:0005623)	1
OG0033030	Cellular Component	membrane(GO:0016020)	1
OG0033031	Cellular Component	cell part(GO:0044464)	1
OG0033031	Cellular Component	cell(GO:0005623)	1
OG0033032	Cellular Component	cell part(GO:0044464)	1
OG0033032	Cellular Component	cell(GO:0005623)	1
OG0033032	Cellular Component	membrane part(GO:0044425)	1
OG0033032	Cellular Component	membrane(GO:0016020)	1
OG0033032	Cellular Component	organelle part(GO:0044422)	1
OG0033032	Cellular Component	organelle(GO:0043226)	1
OG0033032	Cellular Component	protein-containing complex(GO:0032991)	1
OG0033033	Cellular Component	cell part(GO:0044464)	1
OG0033033	Cellular Component	cell(GO:0005623)	1
OG0033033	Cellular Component	membrane(GO:0016020)	1
OG0033037	Cellular Component	cell part(GO:0044464)	1
OG0033037	Cellular Component	cell(GO:0005623)	1
OG0033041	Cellular Component	cell part(GO:0044464)	1
OG0033041	Cellular Component	cell(GO:0005623)	1
OG0033041	Cellular Component	membrane(GO:0016020)	1
OG0033044	Cellular Component	cell part(GO:0044464)	1
OG0033044	Cellular Component	cell(GO:0005623)	1
OG0033044	Cellular Component	extracellular region(GO:0005576)	1
OG0033044	Cellular Component	membrane(GO:0016020)	1
OG0033046	Cellular Component	cell part(GO:0044464)	1
OG0033046	Cellular Component	cell(GO:0005623)	1
OG0033046	Cellular Component	membrane(GO:0016020)	1
OG0033047	Cellular Component	cell part(GO:0044464)	1
OG0033047	Cellular Component	cell(GO:0005623)	1
OG0033047	Cellular Component	membrane(GO:0016020)	1
OG0033051	Cellular Component	cell part(GO:0044464)	1
OG0033051	Cellular Component	cell(GO:0005623)	1
OG0033052	Cellular Component	cell part(GO:0044464)	1
OG0033052	Cellular Component	cell(GO:0005623)	1
OG0033052	Cellular Component	membrane(GO:0016020)	1
OG0033054	Cellular Component	cell part(GO:0044464)	1
OG0033054	Cellular Component	cell(GO:0005623)	1
OG0033054	Cellular Component	membrane(GO:0016020)	1
OG0033055	Cellular Component	cell part(GO:0044464)	1
OG0033055	Cellular Component	cell(GO:0005623)	1
OG0033055	Cellular Component	membrane(GO:0016020)	1
OG0033059	Cellular Component	cell part(GO:0044464)	1
OG0033059	Cellular Component	cell(GO:0005623)	1
OG0033059	Cellular Component	membrane(GO:0016020)	1

OG0033060	Cellular Component	cell part(GO:0044464)	1
OG0033060	Cellular Component	cell(GO:0005623)	1
OG0033060	Cellular Component	membrane(GO:0016020)	1
OG0033062	Cellular Component	cell part(GO:0044464)	1
OG0033062	Cellular Component	cell(GO:0005623)	1
OG0033062	Cellular Component	protein-containing complex(GO:0032991)	1
OG0033063	Cellular Component	cell part(GO:0044464)	1
OG0033063	Cellular Component	cell(GO:0005623)	1
OG0033065	Cellular Component	cell part(GO:0044464)	1
OG0033065	Cellular Component	cell(GO:0005623)	1
OG0033065	Cellular Component	membrane(GO:0016020)	1
OG0033066	Cellular Component	cell part(GO:0044464)	1
OG0033066	Cellular Component	cell(GO:0005623)	1
OG0033066	Cellular Component	membrane(GO:0016020)	1
OG0033068	Cellular Component	cell part(GO:0044464)	1
OG0033068	Cellular Component	cell(GO:0005623)	1
OG0033068	Cellular Component	membrane(GO:0016020)	1
OG0033069	Cellular Component	cell part(GO:0044464)	1
OG0033069	Cellular Component	cell(GO:0005623)	1
OG0033069	Cellular Component	membrane(GO:0016020)	1
OG0033073	Cellular Component	cell part(GO:0044464)	1
OG0033073	Cellular Component	cell(GO:0005623)	1
OG0033073	Cellular Component	membrane(GO:0016020)	1
OG0033079	Cellular Component	cell part(GO:0044464)	1
OG0033079	Cellular Component	cell(GO:0005623)	1
OG0033081	Cellular Component	membrane(GO:0016020)	1
OG0033089	Cellular Component	cell part(GO:0044464)	1
OG0033089	Cellular Component	cell(GO:0005623)	1
OG0033089	Cellular Component	organelle(GO:0043226)	1
OG0033095	Cellular Component	cell part(GO:0044464)	1
OG0033095	Cellular Component	cell(GO:0005623)	1
OG0033095	Cellular Component	organelle(GO:0043226)	1
OG0033101	Cellular Component	cell part(GO:0044464)	1
OG0033101	Cellular Component	cell(GO:0005623)	1
OG0033101	Cellular Component	membrane(GO:0016020)	1
OG0033105	Cellular Component	cell part(GO:0044464)	1
OG0033105	Cellular Component	cell(GO:0005623)	1
OG0033107	Cellular Component	cell part(GO:0044464)	1
OG0033107	Cellular Component	cell(GO:0005623)	1
OG0033108	Cellular Component	cell part(GO:0044464)	1
OG0033108	Cellular Component	cell(GO:0005623)	1
OG0033108	Cellular Component	membrane(GO:0016020)	1
OG0033109	Cellular Component	cell part(GO:0044464)	1
OG0033109	Cellular Component	cell(GO:0005623)	1
OG0033109	Cellular Component	organelle part(GO:0044422)	1
OG0033109	Cellular Component	organelle(GO:0043226)	1
OG0033109	Cellular Component	protein-containing complex(GO:0032991)	1

OG0033111	Cellular Component	cell junction(GO:0030054)	1
OG0033111	Cellular Component	cell part(GO:0044464)	1
OG0033111	Cellular Component	cell(GO:0005623)	1
OG0033111	Cellular Component	membrane(GO:0016020)	1
OG0033111	Cellular Component	membrane-enclosed lumen(GO:0031974)	1
OG0033111	Cellular Component	organelle part(GO:0044422)	1
OG0033111	Cellular Component	organelle(GO:0043226)	1
OG0033111	Cellular Component	protein-containing complex(GO:0032991)	1
OG0033111	Cellular Component	symplast(GO:0055044)	1
OG0033114	Cellular Component	cell part(GO:0044464)	1
OG0033114	Cellular Component	cell(GO:0005623)	1
OG0033114	Cellular Component	organelle(GO:0043226)	1
OG0033115	Cellular Component	cell part(GO:0044464)	1
OG0033115	Cellular Component	cell(GO:0005623)	1
OG0033115	Cellular Component	organelle(GO:0043226)	1
OG0033116	Cellular Component	cell junction(GO:0030054)	1
OG0033116	Cellular Component	cell part(GO:0044464)	1
OG0033116	Cellular Component	cell(GO:0005623)	1
OG0033116	Cellular Component	extracellular region(GO:0005576)	1
OG0033116	Cellular Component	membrane(GO:0016020)	1
OG0033116	Cellular Component	organelle(GO:0043226)	1
OG0033116	Cellular Component	symplast(GO:0055044)	1
OG0033122	Cellular Component	cell part(GO:0044464)	1
OG0033122	Cellular Component	cell(GO:0005623)	1
OG0033122	Cellular Component	membrane(GO:0016020)	1
OG0033122	Cellular Component	organelle part(GO:0044422)	1
OG0033122	Cellular Component	organelle(GO:0043226)	1
OG0033123	Cellular Component	cell part(GO:0044464)	1
OG0033123	Cellular Component	cell(GO:0005623)	1
OG0033123	Cellular Component	organelle(GO:0043226)	1
OG0033124	Cellular Component	cell part(GO:0044464)	1
OG0033124	Cellular Component	cell(GO:0005623)	1
OG0033124	Cellular Component	organelle part(GO:0044422)	1
OG0033124	Cellular Component	organelle(GO:0043226)	1
OG0033126	Cellular Component	cell part(GO:0044464)	1
OG0033126	Cellular Component	cell(GO:0005623)	1
OG0033126	Cellular Component	membrane(GO:0016020)	1
OG0033126	Cellular Component	organelle part(GO:0044422)	1
OG0033126	Cellular Component	organelle(GO:0043226)	1
OG0033128	Cellular Component	cell part(GO:0044464)	1
OG0033128	Cellular Component	cell(GO:0005623)	1
OG0033128	Cellular Component	membrane(GO:0016020)	1
OG0033128	Cellular Component	organelle(GO:0043226)	1
OG0033130	Cellular Component	cell part(GO:0044464)	1
OG0033130	Cellular Component	cell(GO:0005623)	1
OG0033130	Cellular Component	membrane-enclosed lumen(GO:0031974)	1
OG0033130	Cellular Component	organelle part(GO:0044422)	1

OG0033130	Cellular Component	organelle(GO:0043226)	1
OG0033132	Cellular Component	cell junction(GO:0030054)	1
OG0033132	Cellular Component	cell part(GO:0044464)	1
OG0033132	Cellular Component	cell(GO:0005623)	1
OG0033132	Cellular Component	membrane part(GO:0044425)	1
OG0033132	Cellular Component	membrane(GO:0016020)	1
OG0033132	Cellular Component	symplast(GO:0055044)	1
OG0033136	Cellular Component	cell part(GO:0044464)	1
OG0033136	Cellular Component	cell(GO:0005623)	1
OG0033136	Cellular Component	membrane(GO:0016020)	1
OG0033137	Cellular Component	cell part(GO:0044464)	1
OG0033137	Cellular Component	cell(GO:0005623)	1
OG0033139	Cellular Component	cell part(GO:0044464)	1
OG0033139	Cellular Component	cell(GO:0005623)	1
OG0033139	Cellular Component	membrane(GO:0016020)	1
OG0033139	Cellular Component	organelle part(GO:0044422)	1
OG0033139	Cellular Component	organelle(GO:0043226)	1
OG0033140	Cellular Component	cell part(GO:0044464)	1
OG0033140	Cellular Component	cell(GO:0005623)	1
OG0033140	Cellular Component	membrane(GO:0016020)	1
OG0033140	Cellular Component	organelle part(GO:0044422)	1
OG0033140	Cellular Component	organelle(GO:0043226)	1
OG0033141	Cellular Component	cell part(GO:0044464)	1
OG0033141	Cellular Component	cell(GO:0005623)	1
OG0033141	Cellular Component	organelle(GO:0043226)	1
OG0033142	Cellular Component	cell part(GO:0044464)	1
OG0033142	Cellular Component	cell(GO:0005623)	1
OG0033142	Cellular Component	membrane(GO:0016020)	1
OG0033142	Cellular Component	organelle part(GO:0044422)	1
OG0033142	Cellular Component	organelle(GO:0043226)	1
OG0033145	Cellular Component	cell part(GO:0044464)	1
OG0033145	Cellular Component	cell(GO:0005623)	1
OG0033145	Cellular Component	organelle(GO:0043226)	1
OG0033147	Cellular Component	cell part(GO:0044464)	1
OG0033147	Cellular Component	cell(GO:0005623)	1
OG0033147	Cellular Component	organelle part(GO:0044422)	1
OG0033147	Cellular Component	organelle(GO:0043226)	1
OG0033147	Cellular Component	protein-containing complex(GO:0032991)	1
OG0033148	Cellular Component	membrane(GO:0016020)	1
OG0033151	Cellular Component	cell part(GO:0044464)	1
OG0033151	Cellular Component	cell(GO:0005623)	1
OG0033151	Cellular Component	organelle(GO:0043226)	1
OG0033152	Cellular Component	cell part(GO:0044464)	1
OG0033152	Cellular Component	cell(GO:0005623)	1
OG0033153	Cellular Component	cell part(GO:0044464)	1
OG0033153	Cellular Component	cell(GO:0005623)	1
OG0033153	Cellular Component	organelle(GO:0043226)	1

OG0033155	Cellular Component	cell part(GO:0044464)	1
OG0033155	Cellular Component	cell(GO:0005623)	1
OG0033155	Cellular Component	extracellular region(GO:0005576)	1
OG0033155	Cellular Component	membrane(GO:0016020)	1
OG0033155	Cellular Component	organelle part(GO:0044422)	1
OG0033155	Cellular Component	organelle(GO:0043226)	1
OG0033157	Cellular Component	cell junction(GO:0030054)	1
OG0033157	Cellular Component	cell part(GO:0044464)	1
OG0033157	Cellular Component	cell(GO:0005623)	1
OG0033157	Cellular Component	membrane(GO:0016020)	1
OG0033157	Cellular Component	organelle(GO:0043226)	1
OG0033157	Cellular Component	symplast(GO:0055044)	1
OG0033176	Cellular Component	cell junction(GO:0030054)	1
OG0033176	Cellular Component	cell part(GO:0044464)	1
OG0033176	Cellular Component	cell(GO:0005623)	1
OG0033176	Cellular Component	extracellular region(GO:0005576)	1
OG0033176	Cellular Component	membrane(GO:0016020)	1
OG0033176	Cellular Component	organelle(GO:0043226)	1
OG0033176	Cellular Component	symplast(GO:0055044)	1
OG0033177	Cellular Component	cell junction(GO:0030054)	1
OG0033177	Cellular Component	cell part(GO:0044464)	1
OG0033177	Cellular Component	cell(GO:0005623)	1
OG0033177	Cellular Component	extracellular region(GO:0005576)	1
OG0033177	Cellular Component	membrane(GO:0016020)	1
OG0033177	Cellular Component	organelle(GO:0043226)	1
OG0033177	Cellular Component	symplast(GO:0055044)	1
OG0033184	Cellular Component	cell part(GO:0044464)	1
OG0033184	Cellular Component	cell(GO:0005623)	1
OG0033184	Cellular Component	membrane(GO:0016020)	1
OG0033184	Cellular Component	organelle part(GO:0044422)	1
OG0033184	Cellular Component	organelle(GO:0043226)	1
OG0033188	Cellular Component	cell part(GO:0044464)	1
OG0033188	Cellular Component	cell(GO:0005623)	1
OG0033188	Cellular Component	organelle(GO:0043226)	1
OG0033191	Cellular Component	cell part(GO:0044464)	1
OG0033191	Cellular Component	cell(GO:0005623)	1
OG0033192	Cellular Component	cell part(GO:0044464)	1
OG0033192	Cellular Component	cell(GO:0005623)	1
OG0033192	Cellular Component	membrane part(GO:0044425)	1
OG0033192	Cellular Component	membrane(GO:0016020)	1
OG0033192	Cellular Component	organelle part(GO:0044422)	1
OG0033192	Cellular Component	organelle(GO:0043226)	1
OG0033192	Cellular Component	protein-containing complex(GO:0032991)	1
OG0033205	Cellular Component	cell part(GO:0044464)	1
OG0033205	Cellular Component	cell(GO:0005623)	1
OG0033205	Cellular Component	organelle(GO:0043226)	1
OG0033209	Cellular Component	cell part(GO:0044464)	1

OG0033209	Cellular Component	cell(GO:0005623)	1
OG0033218	Cellular Component	cell part(GO:0044464)	1
OG0033218	Cellular Component	cell(GO:0005623)	1
OG0033218	Cellular Component	membrane(GO:0016020)	1
OG0033218	Cellular Component	membrane-enclosed lumen(GO:0031974)	1
OG0033218	Cellular Component	organelle part(GO:0044422)	1
OG0033218	Cellular Component	organelle(GO:0043226)	1
OG0033218	Cellular Component	protein-containing complex(GO:0032991)	1
OG0033226	Cellular Component	cell junction(GO:0030054)	1
OG0033226	Cellular Component	cell part(GO:0044464)	1
OG0033226	Cellular Component	cell(GO:0005623)	1
OG0033226	Cellular Component	symplast(GO:0055044)	1
OG0033231	Cellular Component	cell part(GO:0044464)	1
OG0033231	Cellular Component	cell(GO:0005623)	1
OG0033231	Cellular Component	membrane(GO:0016020)	1
OG0033231	Cellular Component	organelle part(GO:0044422)	1
OG0033231	Cellular Component	organelle(GO:0043226)	1
OG0033237	Cellular Component	cell part(GO:0044464)	1
OG0033237	Cellular Component	cell(GO:0005623)	1
OG0033243	Cellular Component	cell part(GO:0044464)	1
OG0033243	Cellular Component	cell(GO:0005623)	1
OG0033244	Cellular Component	cell part(GO:0044464)	1
OG0033244	Cellular Component	cell(GO:0005623)	1
OG0033244	Cellular Component	organelle(GO:0043226)	1
OG0033247	Cellular Component	cell part(GO:0044464)	1
OG0033247	Cellular Component	cell(GO:0005623)	1
OG0033247	Cellular Component	organelle(GO:0043226)	1
OG0033252	Cellular Component	cell part(GO:0044464)	1
OG0033252	Cellular Component	cell(GO:0005623)	1
OG0033252	Cellular Component	organelle(GO:0043226)	1
OG0033264	Cellular Component	cell part(GO:0044464)	1
OG0033264	Cellular Component	cell(GO:0005623)	1
OG0033264	Cellular Component	organelle(GO:0043226)	1
OG0033264	Cellular Component	protein-containing complex(GO:0032991)	1
OG0033265	Cellular Component	cell part(GO:0044464)	1
OG0033265	Cellular Component	cell(GO:0005623)	1
OG0033265	Cellular Component	membrane part(GO:0044425)	1
OG0033265	Cellular Component	membrane(GO:0016020)	1
OG0033265	Cellular Component	membrane-enclosed lumen(GO:0031974)	1
OG0033265	Cellular Component	organelle part(GO:0044422)	1
OG0033265	Cellular Component	organelle(GO:0043226)	1
OG0033265	Cellular Component	protein-containing complex(GO:0032991)	1
OG0033267	Cellular Component	cell part(GO:0044464)	1
OG0033267	Cellular Component	cell(GO:0005623)	1
OG0033267	Cellular Component	organelle(GO:0043226)	1
OG0033268	Cellular Component	cell part(GO:0044464)	1

OG0033268	Cellular Component	cell(GO:0005623)	1
OG0033268	Cellular Component	organelle(GO:0043226)	1
OG0033269	Cellular Component	cell part(GO:0044464)	1
OG0033269	Cellular Component	cell(GO:0005623)	1
OG0033269	Cellular Component	membrane(GO:0016020)	1
OG0033269	Cellular Component	organelle part(GO:0044422)	1
OG0033269	Cellular Component	organelle(GO:0043226)	1
OG0033278	Cellular Component	cell part(GO:0044464)	1
OG0033278	Cellular Component	cell(GO:0005623)	1
OG0033278	Cellular Component	membrane(GO:0016020)	1
OG0033278	Cellular Component	organelle part(GO:0044422)	1
OG0033278	Cellular Component	organelle(GO:0043226)	1
OG0033279	Cellular Component	cell part(GO:0044464)	1
OG0033279	Cellular Component	cell(GO:0005623)	1
OG0033282	Cellular Component	cell part(GO:0044464)	1
OG0033282	Cellular Component	cell(GO:0005623)	1
OG0033282	Cellular Component	organelle(GO:0043226)	1
OG0033283	Cellular Component	extracellular region(GO:0005576)	1
OG0033285	Cellular Component	cell junction(GO:0030054)	1
OG0033285	Cellular Component	cell part(GO:0044464)	1
OG0033285	Cellular Component	cell(GO:0005623)	1
OG0033285	Cellular Component	membrane(GO:0016020)	1
OG0033285	Cellular Component	organelle part(GO:0044422)	1
OG0033285	Cellular Component	organelle(GO:0043226)	1
OG0033285	Cellular Component	protein-containing complex(GO:0032991)	1
OG0033285	Cellular Component	symplast(GO:0055044)	1
OG0033286	Cellular Component	extracellular region(GO:0005576)	1
OG0033287	Cellular Component	extracellular region(GO:0005576)	1
OG0033288	Cellular Component	extracellular region(GO:0005576)	1
OG0033290	Cellular Component	cell part(GO:0044464)	1
OG0033290	Cellular Component	cell(GO:0005623)	1
OG0033290	Cellular Component	organelle(GO:0043226)	1
OG0033291	Cellular Component	cell part(GO:0044464)	1
OG0033291	Cellular Component	cell(GO:0005623)	1
OG0033294	Cellular Component	cell part(GO:0044464)	1
OG0033294	Cellular Component	cell(GO:0005623)	1
OG0033294	Cellular Component	extracellular region(GO:0005576)	1
OG0033294	Cellular Component	membrane(GO:0016020)	1
OG0033294	Cellular Component	membrane-enclosed lumen(GO:0031974)	1
OG0033294	Cellular Component	organelle part(GO:0044422)	1
OG0033294	Cellular Component	organelle(GO:0043226)	1
OG0033299	Cellular Component	cell part(GO:0044464)	1
OG0033299	Cellular Component	cell(GO:0005623)	1
OG0033299	Cellular Component	organelle(GO:0043226)	1
OG0033300	Cellular Component	cell part(GO:0044464)	1
OG0033300	Cellular Component	cell(GO:0005623)	1
OG0033303	Cellular Component	cell part(GO:0044464)	1

OG0033303	Cellular Component	cell(GO:0005623)	1
OG0033303	Cellular Component	organelle(GO:0043226)	1
OG0033307	Cellular Component	cell junction(GO:0030054)	1
OG0033307	Cellular Component	cell part(GO:0044464)	1
OG0033307	Cellular Component	cell(GO:0005623)	1
OG0033307	Cellular Component	extracellular region part(GO:0044421)	1
OG0033307	Cellular Component	extracellular region(GO:0005576)	1
OG0033307	Cellular Component	membrane(GO:0016020)	1
OG0033307	Cellular Component	membrane-enclosed lumen(GO:0031974)	1
OG0033307	Cellular Component	organelle part(GO:0044422)	1
OG0033307	Cellular Component	organelle(GO:0043226)	1
OG0033307	Cellular Component	symplast(GO:0055044)	1
OG0033308	Cellular Component	cell part(GO:0044464)	1
OG0033308	Cellular Component	cell(GO:0005623)	1
OG0033310	Cellular Component	cell part(GO:0044464)	1
OG0033310	Cellular Component	cell(GO:0005623)	1
OG0033310	Cellular Component	extracellular region part(GO:0044421)	1
OG0033310	Cellular Component	extracellular region(GO:0005576)	1
OG0033310	Cellular Component	membrane part(GO:0044425)	1
OG0033310	Cellular Component	membrane(GO:0016020)	1
OG0033310	Cellular Component	organelle(GO:0043226)	1
OG0033311	Cellular Component	cell junction(GO:0030054)	1
OG0033311	Cellular Component	cell part(GO:0044464)	1
OG0033311	Cellular Component	cell(GO:0005623)	1
OG0033311	Cellular Component	organelle(GO:0043226)	1
OG0033311	Cellular Component	symplast(GO:0055044)	1
OG0033318	Cellular Component	cell part(GO:0044464)	1
OG0033318	Cellular Component	cell(GO:0005623)	1
OG0033318	Cellular Component	membrane-enclosed lumen(GO:0031974)	1
OG0033318	Cellular Component	organelle part(GO:0044422)	1
OG0033318	Cellular Component	organelle(GO:0043226)	1
OG0033321	Cellular Component	cell part(GO:0044464)	1
OG0033321	Cellular Component	cell(GO:0005623)	1
OG0033321	Cellular Component	organelle(GO:0043226)	1
OG0033332	Cellular Component	cell part(GO:0044464)	1
OG0033332	Cellular Component	cell(GO:0005623)	1
OG0033332	Cellular Component	organelle(GO:0043226)	1
OG0033335	Cellular Component	cell part(GO:0044464)	1
OG0033335	Cellular Component	cell(GO:0005623)	1
OG0033335	Cellular Component	organelle(GO:0043226)	1
OG0033340	Cellular Component	cell part(GO:0044464)	1
OG0033340	Cellular Component	cell(GO:0005623)	1
OG0033340	Cellular Component	organelle(GO:0043226)	1
OG0033341	Cellular Component	cell part(GO:0044464)	1
OG0033341	Cellular Component	cell(GO:0005623)	1
OG0033342	Cellular Component	cell part(GO:0044464)	1
OG0033342	Cellular Component	cell(GO:0005623)	1

OG0033342	Cellular Component	organelle(GO:0043226)	1
OG0033344	Cellular Component	cell part(GO:0044464)	1
OG0033344	Cellular Component	cell(GO:0005623)	1
OG0033354	Cellular Component	cell part(GO:0044464)	1
OG0033354	Cellular Component	cell(GO:0005623)	1
OG0033358	Cellular Component	cell part(GO:0044464)	1
OG0033358	Cellular Component	cell(GO:0005623)	1
OG0033358	Cellular Component	organelle(GO:0043226)	1
OG0033359	Cellular Component	cell part(GO:0044464)	1
OG0033359	Cellular Component	cell(GO:0005623)	1
OG0033359	Cellular Component	membrane(GO:0016020)	1
OG0033359	Cellular Component	organelle part(GO:0044422)	1
OG0033359	Cellular Component	organelle(GO:0043226)	1
OG0033367	Cellular Component	cell part(GO:0044464)	1
OG0033367	Cellular Component	cell(GO:0005623)	1
OG0033367	Cellular Component	extracellular region(GO:0005576)	1
OG0033367	Cellular Component	organelle(GO:0043226)	1
OG0033378	Cellular Component	membrane(GO:0016020)	1
OG0033391	Cellular Component	cell part(GO:0044464)	1
OG0033391	Cellular Component	cell(GO:0005623)	1
OG0033403	Cellular Component	cell part(GO:0044464)	1
OG0033403	Cellular Component	cell(GO:0005623)	1
OG0033405	Cellular Component	cell part(GO:0044464)	1
OG0033405	Cellular Component	cell(GO:0005623)	1
OG0033406	Cellular Component	cell part(GO:0044464)	1
OG0033406	Cellular Component	cell(GO:0005623)	1
OG0033406	Cellular Component	membrane(GO:0016020)	1
OG0033408	Cellular Component	cell part(GO:0044464)	1
OG0033408	Cellular Component	cell(GO:0005623)	1
OG0033408	Cellular Component	membrane(GO:0016020)	1
OG0033411	Cellular Component	cell part(GO:0044464)	1
OG0033411	Cellular Component	cell(GO:0005623)	1
OG0033411	Cellular Component	membrane(GO:0016020)	1
OG0033414	Cellular Component	membrane part(GO:0044425)	1
OG0033414	Cellular Component	membrane(GO:0016020)	1
OG0033415	Cellular Component	cell part(GO:0044464)	1
OG0033415	Cellular Component	cell(GO:0005623)	1
OG0033415	Cellular Component	membrane(GO:0016020)	1
OG0033419	Cellular Component	cell part(GO:0044464)	1
OG0033419	Cellular Component	cell(GO:0005623)	1
OG0033422	Cellular Component	cell part(GO:0044464)	1
OG0033422	Cellular Component	cell(GO:0005623)	1
OG0033422	Cellular Component	extracellular region(GO:0005576)	1
OG0033422	Cellular Component	membrane(GO:0016020)	1
OG0033423	Cellular Component	cell part(GO:0044464)	1
OG0033423	Cellular Component	cell(GO:0005623)	1
OG0033423	Cellular Component	membrane(GO:0016020)	1

OG0033424	Cellular Component	cell part(GO:0044464)	1
OG0033424	Cellular Component	cell(GO:0005623)	1
OG0033424	Cellular Component	membrane(GO:0016020)	1
OG0033428	Cellular Component	cell part(GO:0044464)	1
OG0033428	Cellular Component	cell(GO:0005623)	1
OG0033435	Cellular Component	cell part(GO:0044464)	1
OG0033435	Cellular Component	cell(GO:0005623)	1
OG0033437	Cellular Component	cell part(GO:0044464)	1
OG0033437	Cellular Component	cell(GO:0005623)	1
OG0033437	Cellular Component	membrane(GO:0016020)	1
OG0033441	Cellular Component	cell part(GO:0044464)	1
OG0033441	Cellular Component	cell(GO:0005623)	1
OG0033441	Cellular Component	membrane(GO:0016020)	1
OG0033444	Cellular Component	cell part(GO:0044464)	1
OG0033444	Cellular Component	cell(GO:0005623)	1
OG0033446	Cellular Component	cell part(GO:0044464)	1
OG0033446	Cellular Component	cell(GO:0005623)	1
OG0033446	Cellular Component	extracellular region(GO:0005576)	1
OG0033446	Cellular Component	membrane(GO:0016020)	1
OG0033450	Cellular Component	cell part(GO:0044464)	1
OG0033450	Cellular Component	cell(GO:0005623)	1
OG0033450	Cellular Component	membrane(GO:0016020)	1
OG0033453	Cellular Component	cell part(GO:0044464)	1
OG0033453	Cellular Component	cell(GO:0005623)	1
OG0033453	Cellular Component	membrane(GO:0016020)	1
OG0033458	Cellular Component	cell part(GO:0044464)	1
OG0033458	Cellular Component	cell(GO:0005623)	1
OG0033458	Cellular Component	extracellular region(GO:0005576)	1
OG0033458	Cellular Component	membrane(GO:0016020)	1
OG0033461	Cellular Component	cell part(GO:0044464)	1
OG0033461	Cellular Component	cell(GO:0005623)	1
OG0033462	Cellular Component	cell part(GO:0044464)	1
OG0033462	Cellular Component	cell(GO:0005623)	1
OG0033462	Cellular Component	membrane(GO:0016020)	1
OG0033463	Cellular Component	cell part(GO:0044464)	1
OG0033463	Cellular Component	cell(GO:0005623)	1
OG0033464	Cellular Component	cell part(GO:0044464)	1
OG0033464	Cellular Component	cell(GO:0005623)	1
OG0033467	Cellular Component	cell part(GO:0044464)	1
OG0033467	Cellular Component	cell(GO:0005623)	1
OG0033467	Cellular Component	membrane(GO:0016020)	1
OG0033470	Cellular Component	cell part(GO:0044464)	1
OG0033470	Cellular Component	cell(GO:0005623)	1
OG0033470	Cellular Component	organelle part(GO:0044422)	1
OG0033470	Cellular Component	organelle(GO:0043226)	1
OG0033470	Cellular Component	protein-containing complex(GO:0032991)	1
OG0033473	Cellular Component	cell part(GO:0044464)	1

OG0033473	Cellular Component	cell(GO:0005623)	1
OG0033473	Cellular Component	membrane(GO:0016020)	1
OG0033475	Cellular Component	cell part(GO:0044464)	1
OG0033475	Cellular Component	cell(GO:0005623)	1
OG0033475	Cellular Component	membrane(GO:0016020)	1
OG0033477	Cellular Component	cell part(GO:0044464)	1
OG0033477	Cellular Component	cell(GO:0005623)	1
OG0033477	Cellular Component	membrane(GO:0016020)	1
OG0033483	Cellular Component	cell part(GO:0044464)	1
OG0033483	Cellular Component	cell(GO:0005623)	1
OG0033483	Cellular Component	membrane(GO:0016020)	1
OG0033484	Cellular Component	cell part(GO:0044464)	1
OG0033484	Cellular Component	cell(GO:0005623)	1
OG0033484	Cellular Component	membrane(GO:0016020)	1
OG0033485	Cellular Component	cell part(GO:0044464)	1
OG0033485	Cellular Component	cell(GO:0005623)	1
OG0033489	Cellular Component	cell part(GO:0044464)	1
OG0033489	Cellular Component	cell(GO:0005623)	1
OG0033489	Cellular Component	membrane(GO:0016020)	1
OG0033489	Cellular Component	organelle part(GO:0044422)	1
OG0033489	Cellular Component	organelle(GO:0043226)	1
OG0033490	Cellular Component	membrane part(GO:0044425)	1
OG0033490	Cellular Component	membrane(GO:0016020)	1
OG0033491	Cellular Component	cell part(GO:0044464)	1
OG0033491	Cellular Component	cell(GO:0005623)	1
OG0033494	Cellular Component	cell part(GO:0044464)	1
OG0033494	Cellular Component	cell(GO:0005623)	1
OG0033494	Cellular Component	membrane(GO:0016020)	1
OG0033499	Cellular Component	cell part(GO:0044464)	1
OG0033499	Cellular Component	cell(GO:0005623)	1
OG0033499	Cellular Component	membrane part(GO:0044425)	1
OG0033499	Cellular Component	membrane(GO:0016020)	1
OG0033499	Cellular Component	organelle part(GO:0044422)	1
OG0033499	Cellular Component	organelle(GO:0043226)	1
OG0033499	Cellular Component	protein-containing complex(GO:0032991)	1
OG0033502	Cellular Component	cell part(GO:0044464)	1
OG0033502	Cellular Component	cell(GO:0005623)	1
OG0033502	Cellular Component	membrane(GO:0016020)	1
OG0033504	Cellular Component	cell part(GO:0044464)	1
OG0033504	Cellular Component	cell(GO:0005623)	1
OG0033504	Cellular Component	membrane(GO:0016020)	1
OG0033505	Cellular Component	cell part(GO:0044464)	1
OG0033505	Cellular Component	cell(GO:0005623)	1
OG0033506	Cellular Component	cell part(GO:0044464)	1
OG0033506	Cellular Component	cell(GO:0005623)	1
OG0033508	Cellular Component	cell part(GO:0044464)	1
OG0033508	Cellular Component	cell(GO:0005623)	1

OG0033508	Cellular Component	membrane(GO:0016020)	1
OG0033510	Cellular Component	cell part(GO:0044464)	1
OG0033510	Cellular Component	cell(GO:0005623)	1
OG0033510	Cellular Component	protein-containing complex(GO:0032991)	1
OG0033514	Cellular Component	cell part(GO:0044464)	1
OG0033514	Cellular Component	cell(GO:0005623)	1
OG0033514	Cellular Component	membrane(GO:0016020)	1
OG0033518	Cellular Component	cell part(GO:0044464)	1
OG0033518	Cellular Component	cell(GO:0005623)	1
OG0033518	Cellular Component	membrane(GO:0016020)	1
OG0033520	Cellular Component	cell part(GO:0044464)	1
OG0033520	Cellular Component	cell(GO:0005623)	1
OG0033520	Cellular Component	membrane(GO:0016020)	1
OG0033521	Cellular Component	cell part(GO:0044464)	1
OG0033521	Cellular Component	cell(GO:0005623)	1
OG0033521	Cellular Component	membrane(GO:0016020)	1
OG0033522	Cellular Component	cell part(GO:0044464)	1
OG0033522	Cellular Component	cell(GO:0005623)	1
OG0033522	Cellular Component	membrane(GO:0016020)	1
OG0033523	Cellular Component	cell part(GO:0044464)	1
OG0033523	Cellular Component	cell(GO:0005623)	1
OG0033523	Cellular Component	membrane(GO:0016020)	1
OG0033528	Cellular Component	cell part(GO:0044464)	1
OG0033528	Cellular Component	cell(GO:0005623)	1
OG0033531	Cellular Component	cell part(GO:0044464)	1
OG0033531	Cellular Component	cell(GO:0005623)	1
OG0033532	Cellular Component	cell part(GO:0044464)	1
OG0033532	Cellular Component	cell(GO:0005623)	1
OG0033533	Cellular Component	cell part(GO:0044464)	1
OG0033533	Cellular Component	cell(GO:0005623)	1
OG0033534	Cellular Component	cell part(GO:0044464)	1
OG0033534	Cellular Component	cell(GO:0005623)	1
OG0033538	Cellular Component	cell part(GO:0044464)	1
OG0033538	Cellular Component	cell(GO:0005623)	1
OG0033538	Cellular Component	organelle(GO:0043226)	1
OG0033539	Cellular Component	cell part(GO:0044464)	1
OG0033539	Cellular Component	cell(GO:0005623)	1
OG0033539	Cellular Component	organelle(GO:0043226)	1
OG0033540	Cellular Component	cell part(GO:0044464)	1
OG0033540	Cellular Component	cell(GO:0005623)	1
OG0033540	Cellular Component	membrane(GO:0016020)	1
OG0033540	Cellular Component	organelle part(GO:0044422)	1
OG0033540	Cellular Component	organelle(GO:0043226)	1
OG0033541	Cellular Component	cell part(GO:0044464)	1
OG0033541	Cellular Component	cell(GO:0005623)	1
OG0033541	Cellular Component	membrane(GO:0016020)	1
OG0033541	Cellular Component	organelle part(GO:0044422)	1

OG0033541	Cellular Component	organelle(GO:0043226)	1
OG0033545	Cellular Component	cell part(GO:0044464)	1
OG0033545	Cellular Component	cell(GO:0005623)	1
OG0033545	Cellular Component	organelle(GO:0043226)	1
OG0033551	Cellular Component	cell part(GO:0044464)	1
OG0033551	Cellular Component	cell(GO:0005623)	1
OG0033551	Cellular Component	organelle(GO:0043226)	1
OG0033556	Cellular Component	cell part(GO:0044464)	1
OG0033556	Cellular Component	cell(GO:0005623)	1
OG0033556	Cellular Component	protein-containing complex(GO:0032991)	1
OG0033558	Cellular Component	cell part(GO:0044464)	1
OG0033558	Cellular Component	cell(GO:0005623)	1
OG0033558	Cellular Component	organelle(GO:0043226)	1
OG0033568	Cellular Component	cell part(GO:0044464)	1
OG0033568	Cellular Component	cell(GO:0005623)	1
OG0033568	Cellular Component	organelle(GO:0043226)	1
OG0033571	Cellular Component	cell junction(GO:0030054)	1
OG0033571	Cellular Component	cell part(GO:0044464)	1
OG0033571	Cellular Component	cell(GO:0005623)	1
OG0033571	Cellular Component	extracellular region(GO:0005576)	1
OG0033571	Cellular Component	membrane(GO:0016020)	1
OG0033571	Cellular Component	organelle part(GO:0044422)	1
OG0033571	Cellular Component	organelle(GO:0043226)	1
OG0033571	Cellular Component	symplast(GO:0055044)	1
OG0033574	Cellular Component	cell part(GO:0044464)	1
OG0033574	Cellular Component	cell(GO:0005623)	1
OG0033574	Cellular Component	membrane(GO:0016020)	1
OG0033574	Cellular Component	organelle part(GO:0044422)	1
OG0033574	Cellular Component	organelle(GO:0043226)	1
OG0033582	Cellular Component	cell part(GO:0044464)	1
OG0033582	Cellular Component	cell(GO:0005623)	1
OG0033582	Cellular Component	organelle(GO:0043226)	1
OG0033596	Cellular Component	cell part(GO:0044464)	1
OG0033596	Cellular Component	cell(GO:0005623)	1
OG0033596	Cellular Component	organelle(GO:0043226)	1
OG0033599	Cellular Component	cell part(GO:0044464)	1
OG0033599	Cellular Component	cell(GO:0005623)	1
OG0033599	Cellular Component	organelle(GO:0043226)	1
OG0033601	Cellular Component	membrane(GO:0016020)	1
OG0033604	Cellular Component	cell part(GO:0044464)	1
OG0033604	Cellular Component	cell(GO:0005623)	1
OG0033604	Cellular Component	organelle(GO:0043226)	1
OG0033609	Cellular Component	cell part(GO:0044464)	1
OG0033609	Cellular Component	cell(GO:0005623)	1
OG0033609	Cellular Component	membrane part(GO:0044425)	1
OG0033609	Cellular Component	membrane(GO:0016020)	1
OG0033609	Cellular Component	organelle part(GO:0044422)	1

OG0033609	Cellular Component	organelle(GO:0043226)	1
OG0033611	Cellular Component	cell part(GO:0044464)	1
OG0033611	Cellular Component	cell(GO:0005623)	1
OG0033611	Cellular Component	membrane part(GO:0044425)	1
OG0033611	Cellular Component	membrane(GO:0016020)	1
OG0033611	Cellular Component	organelle part(GO:0044422)	1
OG0033611	Cellular Component	organelle(GO:0043226)	1
OG0033617	Cellular Component	cell part(GO:0044464)	1
OG0033617	Cellular Component	cell(GO:0005623)	1
OG0033617	Cellular Component	membrane(GO:0016020)	1
OG0033617	Cellular Component	organelle(GO:0043226)	1
OG0033618	Cellular Component	cell part(GO:0044464)	1
OG0033618	Cellular Component	cell(GO:0005623)	1
OG0033618	Cellular Component	organelle(GO:0043226)	1
OG0033624	Cellular Component	cell part(GO:0044464)	1
OG0033624	Cellular Component	cell(GO:0005623)	1
OG0033624	Cellular Component	membrane(GO:0016020)	1
OG0033625	Cellular Component	cell part(GO:0044464)	1
OG0033625	Cellular Component	cell(GO:0005623)	1
OG0033625	Cellular Component	membrane part(GO:0044425)	1
OG0033625	Cellular Component	membrane(GO:0016020)	1
OG0033625	Cellular Component	organelle part(GO:0044422)	1
OG0033625	Cellular Component	organelle(GO:0043226)	1
OG0033627	Cellular Component	cell part(GO:0044464)	1
OG0033627	Cellular Component	cell(GO:0005623)	1
OG0033627	Cellular Component	membrane part(GO:0044425)	1
OG0033627	Cellular Component	membrane(GO:0016020)	1
OG0033627	Cellular Component	organelle part(GO:0044422)	1
OG0033627	Cellular Component	organelle(GO:0043226)	1
OG0033630	Cellular Component	cell part(GO:0044464)	1
OG0033630	Cellular Component	cell(GO:0005623)	1
OG0033630	Cellular Component	organelle part(GO:0044422)	1
OG0033630	Cellular Component	organelle(GO:0043226)	1
OG0033631	Cellular Component	cell part(GO:0044464)	1
OG0033631	Cellular Component	cell(GO:0005623)	1
OG0033631	Cellular Component	organelle(GO:0043226)	1
OG0033642	Cellular Component	cell part(GO:0044464)	1
OG0033642	Cellular Component	cell(GO:0005623)	1
OG0033642	Cellular Component	organelle part(GO:0044422)	1
OG0033642	Cellular Component	organelle(GO:0043226)	1
OG0033643	Cellular Component	cell part(GO:0044464)	1
OG0033643	Cellular Component	cell(GO:0005623)	1
OG0033643	Cellular Component	organelle part(GO:0044422)	1
OG0033643	Cellular Component	organelle(GO:0043226)	1
OG0033644	Cellular Component	cell part(GO:0044464)	1
OG0033644	Cellular Component	cell(GO:0005623)	1
OG0033644	Cellular Component	organelle part(GO:0044422)	1

OG0033644	Cellular Component	organelle(GO:0043226)	1
OG0033648	Cellular Component	cell part(GO:0044464)	1
OG0033648	Cellular Component	cell(GO:0005623)	1
OG0033648	Cellular Component	organelle(GO:0043226)	1
OG0033650	Cellular Component	cell part(GO:0044464)	1
OG0033650	Cellular Component	cell(GO:0005623)	1
OG0033650	Cellular Component	membrane-enclosed lumen(GO:0031974)	1
OG0033650	Cellular Component	organelle part(GO:0044422)	1
OG0033650	Cellular Component	organelle(GO:0043226)	1
OG0033650	Cellular Component	protein-containing complex(GO:0032991)	1
OG0033651	Cellular Component	cell part(GO:0044464)	1
OG0033651	Cellular Component	cell(GO:0005623)	1
OG0033651	Cellular Component	membrane(GO:0016020)	1
OG0033651	Cellular Component	organelle part(GO:0044422)	1
OG0033651	Cellular Component	organelle(GO:0043226)	1
OG0033652	Cellular Component	cell part(GO:0044464)	1
OG0033652	Cellular Component	cell(GO:0005623)	1
OG0033652	Cellular Component	organelle part(GO:0044422)	1
OG0033652	Cellular Component	organelle(GO:0043226)	1
OG0033653	Cellular Component	cell junction(GO:0030054)	1
OG0033653	Cellular Component	cell part(GO:0044464)	1
OG0033653	Cellular Component	cell(GO:0005623)	1
OG0033653	Cellular Component	extracellular region(GO:0005576)	1
OG0033653	Cellular Component	membrane(GO:0016020)	1
OG0033653	Cellular Component	organelle(GO:0043226)	1
OG0033653	Cellular Component	symplast(GO:0055044)	1
OG0033655	Cellular Component	cell part(GO:0044464)	1
OG0033655	Cellular Component	cell(GO:0005623)	1
OG0033655	Cellular Component	organelle(GO:0043226)	1
OG0033657	Cellular Component	cell part(GO:0044464)	1
OG0033657	Cellular Component	cell(GO:0005623)	1
OG0033657	Cellular Component	organelle part(GO:0044422)	1
OG0033657	Cellular Component	organelle(GO:0043226)	1
OG0033661	Cellular Component	cell part(GO:0044464)	1
OG0033661	Cellular Component	cell(GO:0005623)	1
OG0033661	Cellular Component	organelle(GO:0043226)	1
OG0033664	Cellular Component	cell part(GO:0044464)	1
OG0033664	Cellular Component	cell(GO:0005623)	1
OG0033664	Cellular Component	membrane(GO:0016020)	1
OG0033664	Cellular Component	organelle part(GO:0044422)	1
OG0033664	Cellular Component	organelle(GO:0043226)	1
OG0033667	Cellular Component	cell part(GO:0044464)	1
OG0033667	Cellular Component	cell(GO:0005623)	1
OG0033667	Cellular Component	membrane(GO:0016020)	1
OG0033667	Cellular Component	organelle part(GO:0044422)	1
OG0033667	Cellular Component	organelle(GO:0043226)	1
OG0033668	Cellular Component	cell part(GO:0044464)	1

OG0033668	Cellular Component	cell(GO:0005623)	1
OG0033668	Cellular Component	organelle(GO:0043226)	1
OG0033672	Cellular Component	cell part(GO:0044464)	1
OG0033672	Cellular Component	cell(GO:0005623)	1
OG0033672	Cellular Component	membrane(GO:0016020)	1
OG0033673	Cellular Component	cell part(GO:0044464)	1
OG0033673	Cellular Component	cell(GO:0005623)	1
OG0033673	Cellular Component	protein-containing complex(GO:0032991)	1
OG0033678	Cellular Component	cell part(GO:0044464)	1
OG0033678	Cellular Component	cell(GO:0005623)	1
OG0033678	Cellular Component	membrane(GO:0016020)	1
OG0033678	Cellular Component	organelle part(GO:0044422)	1
OG0033678	Cellular Component	organelle(GO:0043226)	1
OG0033679	Cellular Component	cell junction(GO:0030054)	1
OG0033679	Cellular Component	cell part(GO:0044464)	1
OG0033679	Cellular Component	cell(GO:0005623)	1
OG0033679	Cellular Component	membrane-enclosed lumen(GO:0031974)	1
OG0033679	Cellular Component	organelle part(GO:0044422)	1
OG0033679	Cellular Component	organelle(GO:0043226)	1
OG0033679	Cellular Component	symplast(GO:0055044)	1
OG0033680	Cellular Component	cell part(GO:0044464)	1
OG0033680	Cellular Component	cell(GO:0005623)	1
OG0033680	Cellular Component	membrane part(GO:0044425)	1
OG0033680	Cellular Component	membrane(GO:0016020)	1
OG0033682	Cellular Component	cell part(GO:0044464)	1
OG0033682	Cellular Component	cell(GO:0005623)	1
OG0033682	Cellular Component	organelle part(GO:0044422)	1
OG0033682	Cellular Component	organelle(GO:0043226)	1
OG0033682	Cellular Component	protein-containing complex(GO:0032991)	1
OG0033685	Cellular Component	cell part(GO:0044464)	1
OG0033685	Cellular Component	cell(GO:0005623)	1
OG0033685	Cellular Component	organelle(GO:0043226)	1
OG0033686	Cellular Component	cell part(GO:0044464)	1
OG0033686	Cellular Component	cell(GO:0005623)	1
OG0033686	Cellular Component	organelle(GO:0043226)	1
OG0033687	Cellular Component	cell part(GO:0044464)	1
OG0033687	Cellular Component	cell(GO:0005623)	1
OG0033687	Cellular Component	organelle(GO:0043226)	1
OG0033689	Cellular Component	cell part(GO:0044464)	1
OG0033689	Cellular Component	cell(GO:0005623)	1
OG0033689	Cellular Component	membrane(GO:0016020)	1
OG0033689	Cellular Component	organelle part(GO:0044422)	1
OG0033689	Cellular Component	organelle(GO:0043226)	1
OG0033695	Cellular Component	cell part(GO:0044464)	1
OG0033695	Cellular Component	cell(GO:0005623)	1
OG0033695	Cellular Component	organelle(GO:0043226)	1
OG0033699	Cellular Component	cell part(GO:0044464)	1

OG0033699	Cellular Component	cell(GO:0005623)	1
OG0033699	Cellular Component	membrane(GO:0016020)	1
OG0033699	Cellular Component	organelle(GO:0043226)	1
OG0033703	Cellular Component	cell part(GO:0044464)	1
OG0033703	Cellular Component	cell(GO:0005623)	1
OG0033703	Cellular Component	membrane part(GO:0044425)	1
OG0033703	Cellular Component	membrane(GO:0016020)	1
OG0033703	Cellular Component	organelle part(GO:0044422)	1
OG0033703	Cellular Component	organelle(GO:0043226)	1
OG0033709	Cellular Component	cell part(GO:0044464)	1
OG0033709	Cellular Component	cell(GO:0005623)	1
OG0033709	Cellular Component	organelle part(GO:0044422)	1
OG0033709	Cellular Component	organelle(GO:0043226)	1
OG0033710	Cellular Component	cell part(GO:0044464)	1
OG0033710	Cellular Component	cell(GO:0005623)	1
OG0033710	Cellular Component	organelle(GO:0043226)	1
OG0033711	Cellular Component	cell junction(GO:0030054)	1
OG0033711	Cellular Component	symplast(GO:0055044)	1
OG0033715	Cellular Component	cell part(GO:0044464)	1
OG0033715	Cellular Component	cell(GO:0005623)	1
OG0033715	Cellular Component	membrane(GO:0016020)	1
OG0033716	Cellular Component	cell junction(GO:0030054)	1
OG0033716	Cellular Component	cell part(GO:0044464)	1
OG0033716	Cellular Component	cell(GO:0005623)	1
OG0033716	Cellular Component	membrane part(GO:0044425)	1
OG0033716	Cellular Component	membrane(GO:0016020)	1
OG0033716	Cellular Component	organelle part(GO:0044422)	1
OG0033716	Cellular Component	organelle(GO:0043226)	1
OG0033716	Cellular Component	protein-containing complex(GO:0032991)	1
OG0033716	Cellular Component	symplast(GO:0055044)	1
OG0033718	Cellular Component	membrane(GO:0016020)	1
OG0033725	Cellular Component	cell part(GO:0044464)	1
OG0033725	Cellular Component	cell(GO:0005623)	1
OG0033725	Cellular Component	membrane(GO:0016020)	1
OG0033725	Cellular Component	organelle part(GO:0044422)	1
OG0033725	Cellular Component	organelle(GO:0043226)	1
OG0033726	Cellular Component	cell part(GO:0044464)	1
OG0033726	Cellular Component	cell(GO:0005623)	1
OG0033726	Cellular Component	membrane(GO:0016020)	1
OG0033726	Cellular Component	organelle part(GO:0044422)	1
OG0033726	Cellular Component	organelle(GO:0043226)	1
OG0033727	Cellular Component	cell part(GO:0044464)	1
OG0033727	Cellular Component	cell(GO:0005623)	1
OG0033727	Cellular Component	membrane(GO:0016020)	1
OG0033727	Cellular Component	organelle part(GO:0044422)	1
OG0033727	Cellular Component	organelle(GO:0043226)	1
OG0033728	Cellular Component	cell part(GO:0044464)	1

OG0033728	Cellular Component	cell(GO:0005623)	1
OG0033728	Cellular Component	membrane(GO:0016020)	1
OG0033728	Cellular Component	organelle part(GO:0044422)	1
OG0033728	Cellular Component	organelle(GO:0043226)	1
OG0033733	Cellular Component	cell part(GO:0044464)	1
OG0033733	Cellular Component	cell(GO:0005623)	1
OG0033740	Cellular Component	cell part(GO:0044464)	1
OG0033740	Cellular Component	cell(GO:0005623)	1
OG0033740	Cellular Component	membrane(GO:0016020)	1
OG0033740	Cellular Component	organelle part(GO:0044422)	1
OG0033740	Cellular Component	organelle(GO:0043226)	1
OG0033741	Cellular Component	cell part(GO:0044464)	1
OG0033741	Cellular Component	cell(GO:0005623)	1
OG0033741	Cellular Component	membrane(GO:0016020)	1
OG0033741	Cellular Component	organelle part(GO:0044422)	1
OG0033741	Cellular Component	organelle(GO:0043226)	1
OG0033749	Cellular Component	membrane(GO:0016020)	1
OG0033750	Cellular Component	cell part(GO:0044464)	1
OG0033750	Cellular Component	cell(GO:0005623)	1
OG0033750	Cellular Component	membrane(GO:0016020)	1
OG0033750	Cellular Component	organelle part(GO:0044422)	1
OG0033750	Cellular Component	organelle(GO:0043226)	1
OG0033752	Cellular Component	cell part(GO:0044464)	1
OG0033752	Cellular Component	cell(GO:0005623)	1
OG0033752	Cellular Component	organelle(GO:0043226)	1
OG0033753	Cellular Component	cell part(GO:0044464)	1
OG0033753	Cellular Component	cell(GO:0005623)	1
OG0033753	Cellular Component	organelle(GO:0043226)	1
OG0033760	Cellular Component	cell part(GO:0044464)	1
OG0033760	Cellular Component	cell(GO:0005623)	1
OG0033760	Cellular Component	membrane(GO:0016020)	1
OG0033760	Cellular Component	organelle part(GO:0044422)	1
OG0033760	Cellular Component	organelle(GO:0043226)	1
OG0033761	Cellular Component	cell part(GO:0044464)	1
OG0033761	Cellular Component	cell(GO:0005623)	1
OG0033761	Cellular Component	organelle(GO:0043226)	1
OG0033762	Cellular Component	cell part(GO:0044464)	1
OG0033762	Cellular Component	cell(GO:0005623)	1
OG0033762	Cellular Component	membrane(GO:0016020)	1
OG0033762	Cellular Component	organelle part(GO:0044422)	1
OG0033762	Cellular Component	organelle(GO:0043226)	1
OG0033764	Cellular Component	cell junction(GO:0030054)	1
OG0033764	Cellular Component	cell part(GO:0044464)	1
OG0033764	Cellular Component	cell(GO:0005623)	1
OG0033764	Cellular Component	membrane(GO:0016020)	1
OG0033764	Cellular Component	symplast(GO:0055044)	1
OG0033768	Cellular Component	cell part(GO:0044464)	1

OG0033768	Cellular Component	cell(GO:0005623)	1
OG0033768	Cellular Component	membrane(GO:0016020)	1
OG0033768	Cellular Component	organelle part(GO:0044422)	1
OG0033768	Cellular Component	organelle(GO:0043226)	1
OG0033769	Cellular Component	cell part(GO:0044464)	1
OG0033769	Cellular Component	cell(GO:0005623)	1
OG0033769	Cellular Component	organelle(GO:0043226)	1
OG0033770	Cellular Component	cell junction(GO:0030054)	1
OG0033770	Cellular Component	cell part(GO:0044464)	1
OG0033770	Cellular Component	cell(GO:0005623)	1
OG0033770	Cellular Component	membrane(GO:0016020)	1
OG0033770	Cellular Component	organelle(GO:0043226)	1
OG0033770	Cellular Component	symplast(GO:0055044)	1
OG0033772	Cellular Component	cell part(GO:0044464)	1
OG0033772	Cellular Component	cell(GO:0005623)	1
OG0033772	Cellular Component	organelle(GO:0043226)	1
OG0033773	Cellular Component	cell junction(GO:0030054)	1
OG0033773	Cellular Component	cell part(GO:0044464)	1
OG0033773	Cellular Component	cell(GO:0005623)	1
OG0033773	Cellular Component	membrane(GO:0016020)	1
OG0033773	Cellular Component	membrane-enclosed lumen(GO:0031974)	1
OG0033773	Cellular Component	organelle part(GO:0044422)	1
OG0033773	Cellular Component	organelle(GO:0043226)	1
OG0033773	Cellular Component	protein-containing complex(GO:0032991)	1
OG0033773	Cellular Component	symplast(GO:0055044)	1
OG0033775	Cellular Component	cell part(GO:0044464)	1
OG0033775	Cellular Component	cell(GO:0005623)	1
OG0033775	Cellular Component	membrane(GO:0016020)	1
OG0033782	Cellular Component	cell part(GO:0044464)	1
OG0033782	Cellular Component	cell(GO:0005623)	1
OG0033782	Cellular Component	membrane-enclosed lumen(GO:0031974)	1
OG0033782	Cellular Component	organelle part(GO:0044422)	1
OG0033782	Cellular Component	organelle(GO:0043226)	1
OG0033782	Cellular Component	protein-containing complex(GO:0032991)	1
OG0033783	Cellular Component	cell part(GO:0044464)	1
OG0033783	Cellular Component	cell(GO:0005623)	1
OG0033783	Cellular Component	organelle(GO:0043226)	1
OG0033790	Cellular Component	membrane part(GO:0044425)	1
OG0033790	Cellular Component	membrane(GO:0016020)	1
OG0033791	Cellular Component	cell part(GO:0044464)	1
OG0033791	Cellular Component	cell(GO:0005623)	1
OG0033791	Cellular Component	membrane(GO:0016020)	1
OG0033792	Cellular Component	cell part(GO:0044464)	1
OG0033792	Cellular Component	cell(GO:0005623)	1
OG0033792	Cellular Component	membrane(GO:0016020)	1
OG0033793	Cellular Component	cell part(GO:0044464)	1
OG0033793	Cellular Component	cell(GO:0005623)	1

OG0033794	Cellular Component	cell part(GO:0044464)	1
OG0033794	Cellular Component	cell(GO:0005623)	1
OG0033794	Cellular Component	membrane(GO:0016020)	1
OG0033796	Cellular Component	cell part(GO:0044464)	1
OG0033796	Cellular Component	cell(GO:0005623)	1
OG0033796	Cellular Component	membrane(GO:0016020)	1
OG0033800	Cellular Component	cell part(GO:0044464)	1
OG0033800	Cellular Component	cell(GO:0005623)	1
OG0033800	Cellular Component	membrane(GO:0016020)	1
OG0033800	Cellular Component	organelle part(GO:0044422)	1
OG0033800	Cellular Component	organelle(GO:0043226)	1
OG0033802	Cellular Component	cell part(GO:0044464)	1
OG0033802	Cellular Component	cell(GO:0005623)	1
OG0033802	Cellular Component	membrane(GO:0016020)	1
OG0033802	Cellular Component	organelle(GO:0043226)	1
OG0033803	Cellular Component	cell junction(GO:0030054)	1
OG0033803	Cellular Component	cell part(GO:0044464)	1
OG0033803	Cellular Component	cell(GO:0005623)	1
OG0033803	Cellular Component	extracellular region(GO:0005576)	1
OG0033803	Cellular Component	membrane(GO:0016020)	1
OG0033803	Cellular Component	organelle part(GO:0044422)	1
OG0033803	Cellular Component	organelle(GO:0043226)	1
OG0033803	Cellular Component	symplast(GO:0055044)	1
OG0033804	Cellular Component	cell part(GO:0044464)	1
OG0033804	Cellular Component	cell(GO:0005623)	1
OG0033804	Cellular Component	membrane(GO:0016020)	1
OG0033804	Cellular Component	organelle part(GO:0044422)	1
OG0033804	Cellular Component	organelle(GO:0043226)	1
OG0033804	Cellular Component	protein-containing complex(GO:0032991)	1
OG0033806	Cellular Component	cell part(GO:0044464)	1
OG0033806	Cellular Component	cell(GO:0005623)	1
OG0033806	Cellular Component	membrane(GO:0016020)	1
OG0033808	Cellular Component	cell part(GO:0044464)	1
OG0033808	Cellular Component	cell(GO:0005623)	1
OG0033810	Cellular Component	cell part(GO:0044464)	1
OG0033810	Cellular Component	cell(GO:0005623)	1
OG0033810	Cellular Component	extracellular region(GO:0005576)	1
OG0033810	Cellular Component	organelle(GO:0043226)	1
OG0033811	Cellular Component	cell part(GO:0044464)	1
OG0033811	Cellular Component	cell(GO:0005623)	1
OG0033811	Cellular Component	membrane-enclosed lumen(GO:0031974)	1
OG0033811	Cellular Component	organelle part(GO:0044422)	1
OG0033811	Cellular Component	organelle(GO:0043226)	1
OG0033815	Cellular Component	cell part(GO:0044464)	1
OG0033815	Cellular Component	cell(GO:0005623)	1
OG0033815	Cellular Component	membrane(GO:0016020)	1
OG0033816	Cellular Component	cell part(GO:0044464)	1

OG0033816	Cellular Component	cell(GO:0005623)	1
OG0033816	Cellular Component	membrane(GO:0016020)	1
OG0033819	Cellular Component	cell part(GO:0044464)	1
OG0033819	Cellular Component	cell(GO:0005623)	1
OG0033819	Cellular Component	organelle(GO:0043226)	1
OG0033826	Cellular Component	cell part(GO:0044464)	1
OG0033826	Cellular Component	cell(GO:0005623)	1
OG0033826	Cellular Component	membrane(GO:0016020)	1
OG0033830	Cellular Component	cell part(GO:0044464)	1
OG0033830	Cellular Component	cell(GO:0005623)	1
OG0033830	Cellular Component	extracellular region(GO:0005576)	1
OG0033830	Cellular Component	membrane(GO:0016020)	1
OG0033833	Cellular Component	cell part(GO:0044464)	1
OG0033833	Cellular Component	cell(GO:0005623)	1
OG0033833	Cellular Component	organelle(GO:0043226)	1
OG0033835	Cellular Component	cell part(GO:0044464)	1
OG0033835	Cellular Component	cell(GO:0005623)	1
OG0033835	Cellular Component	membrane(GO:0016020)	1
OG0033835	Cellular Component	organelle part(GO:0044422)	1
OG0033835	Cellular Component	organelle(GO:0043226)	1
OG0033842	Cellular Component	cell part(GO:0044464)	1
OG0033842	Cellular Component	cell(GO:0005623)	1
OG0033844	Cellular Component	cell part(GO:0044464)	1
OG0033844	Cellular Component	cell(GO:0005623)	1
OG0033846	Cellular Component	cell part(GO:0044464)	1
OG0033846	Cellular Component	cell(GO:0005623)	1
OG0033847	Cellular Component	cell part(GO:0044464)	1
OG0033847	Cellular Component	cell(GO:0005623)	1
OG0033856	Cellular Component	cell part(GO:0044464)	1
OG0033856	Cellular Component	cell(GO:0005623)	1
OG0033856	Cellular Component	membrane(GO:0016020)	1
OG0033856	Cellular Component	organelle(GO:0043226)	1
OG0033857	Cellular Component	cell part(GO:0044464)	1
OG0033857	Cellular Component	cell(GO:0005623)	1
OG0033857	Cellular Component	membrane(GO:0016020)	1
OG0033857	Cellular Component	organelle part(GO:0044422)	1
OG0033857	Cellular Component	organelle(GO:0043226)	1
OG0033858	Cellular Component	cell part(GO:0044464)	1
OG0033858	Cellular Component	cell(GO:0005623)	1
OG0033858	Cellular Component	membrane(GO:0016020)	1
OG0033863	Cellular Component	membrane(GO:0016020)	1
OG0033865	Cellular Component	cell part(GO:0044464)	1
OG0033865	Cellular Component	cell(GO:0005623)	1
OG0033865	Cellular Component	organelle(GO:0043226)	1
OG0033867	Cellular Component	cell part(GO:0044464)	1
OG0033867	Cellular Component	cell(GO:0005623)	1
OG0033867	Cellular Component	membrane(GO:0016020)	1

OG0033867	Cellular Component	organelle part(GO:0044422)	1
OG0033867	Cellular Component	organelle(GO:0043226)	1
OG0033867	Cellular Component	synapse part(GO:0044456)	1
OG0033867	Cellular Component	synapse(GO:0045202)	1
OG0033868	Cellular Component	cell part(GO:0044464)	1
OG0033868	Cellular Component	cell(GO:0005623)	1
OG0033868	Cellular Component	organelle(GO:0043226)	1
OG0033872	Cellular Component	cell part(GO:0044464)	1
OG0033872	Cellular Component	cell(GO:0005623)	1
OG0033872	Cellular Component	organelle part(GO:0044422)	1
OG0033872	Cellular Component	organelle(GO:0043226)	1
OG0033874	Cellular Component	membrane(GO:0016020)	1
OG0033875	Cellular Component	cell part(GO:0044464)	1
OG0033875	Cellular Component	cell(GO:0005623)	1
OG0033875	Cellular Component	organelle(GO:0043226)	1
OG0033877	Cellular Component	cell part(GO:0044464)	1
OG0033877	Cellular Component	cell(GO:0005623)	1
OG0033877	Cellular Component	membrane(GO:0016020)	1
OG0033877	Cellular Component	organelle part(GO:0044422)	1
OG0033877	Cellular Component	organelle(GO:0043226)	1
OG0033879	Cellular Component	cell part(GO:0044464)	1
OG0033879	Cellular Component	cell(GO:0005623)	1
OG0033879	Cellular Component	membrane(GO:0016020)	1
OG0033879	Cellular Component	organelle(GO:0043226)	1
OG0033880	Cellular Component	cell part(GO:0044464)	1
OG0033880	Cellular Component	cell(GO:0005623)	1
OG0033880	Cellular Component	membrane(GO:0016020)	1
OG0033880	Cellular Component	organelle(GO:0043226)	1
OG0033891	Cellular Component	cell part(GO:0044464)	1
OG0033891	Cellular Component	cell(GO:0005623)	1
OG0033898	Cellular Component	cell part(GO:0044464)	1
OG0033898	Cellular Component	cell(GO:0005623)	1
OG0033898	Cellular Component	membrane(GO:0016020)	1
OG0033898	Cellular Component	organelle part(GO:0044422)	1
OG0033898	Cellular Component	organelle(GO:0043226)	1
OG0033899	Cellular Component	cell part(GO:0044464)	1
OG0033899	Cellular Component	cell(GO:0005623)	1
OG0033899	Cellular Component	membrane(GO:0016020)	1
OG0033899	Cellular Component	organelle part(GO:0044422)	1
OG0033899	Cellular Component	organelle(GO:0043226)	1
OG0033900	Cellular Component	cell part(GO:0044464)	1
OG0033900	Cellular Component	cell(GO:0005623)	1
OG0033900	Cellular Component	membrane(GO:0016020)	1
OG0033900	Cellular Component	organelle part(GO:0044422)	1
OG0033900	Cellular Component	organelle(GO:0043226)	1
OG0033901	Cellular Component	cell part(GO:0044464)	1
OG0033901	Cellular Component	cell(GO:0005623)	1

OG0033901	Cellular Component	membrane(GO:0016020)	1
OG0033904	Cellular Component	cell part(GO:0044464)	1
OG0033904	Cellular Component	cell(GO:0005623)	1
OG0033904	Cellular Component	organelle(GO:0043226)	1
OG0033905	Cellular Component	cell part(GO:0044464)	1
OG0033905	Cellular Component	cell(GO:0005623)	1
OG0033905	Cellular Component	organelle(GO:0043226)	1
OG0033910	Cellular Component	cell part(GO:0044464)	1
OG0033910	Cellular Component	cell(GO:0005623)	1
OG0033910	Cellular Component	organelle(GO:0043226)	1
OG0033918	Cellular Component	cell part(GO:0044464)	1
OG0033918	Cellular Component	cell(GO:0005623)	1
OG0033918	Cellular Component	organelle(GO:0043226)	1
OG0033923	Cellular Component	cell part(GO:0044464)	1
OG0033923	Cellular Component	cell(GO:0005623)	1
OG0033928	Cellular Component	cell part(GO:0044464)	1
OG0033928	Cellular Component	cell(GO:0005623)	1
OG0033928	Cellular Component	extracellular region(GO:0005576)	1
OG0033928	Cellular Component	membrane part(GO:0044425)	1
OG0033928	Cellular Component	membrane(GO:0016020)	1
OG0033928	Cellular Component	organelle part(GO:0044422)	1
OG0033928	Cellular Component	organelle(GO:0043226)	1
OG0033929	Cellular Component	cell part(GO:0044464)	1
OG0033929	Cellular Component	cell(GO:0005623)	1
OG0033929	Cellular Component	extracellular region(GO:0005576)	1
OG0033929	Cellular Component	organelle(GO:0043226)	1
OG0033933	Cellular Component	cell part(GO:0044464)	1
OG0033933	Cellular Component	cell(GO:0005623)	1
OG0033943	Cellular Component	cell part(GO:0044464)	1
OG0033943	Cellular Component	cell(GO:0005623)	1
OG0033943	Cellular Component	membrane(GO:0016020)	1
OG0033944	Cellular Component	cell part(GO:0044464)	1
OG0033944	Cellular Component	cell(GO:0005623)	1
OG0033944	Cellular Component	organelle(GO:0043226)	1
OG0033946	Cellular Component	cell part(GO:0044464)	1
OG0033946	Cellular Component	cell(GO:0005623)	1
OG0033946	Cellular Component	membrane(GO:0016020)	1
OG0033946	Cellular Component	organelle part(GO:0044422)	1
OG0033946	Cellular Component	organelle(GO:0043226)	1
OG0033947	Cellular Component	cell part(GO:0044464)	1
OG0033947	Cellular Component	cell(GO:0005623)	1
OG0033947	Cellular Component	membrane-enclosed lumen(GO:0031974)	1
OG0033947	Cellular Component	organelle part(GO:0044422)	1
OG0033947	Cellular Component	organelle(GO:0043226)	1
OG0033956	Cellular Component	cell part(GO:0044464)	1
OG0033956	Cellular Component	cell(GO:0005623)	1
OG0033957	Cellular Component	cell part(GO:0044464)	1

OG0033957	Cellular Component	cell(GO:0005623)	1
OG0033957	Cellular Component	extracellular region(GO:0005576)	1
OG0033957	Cellular Component	membrane part(GO:0044425)	1
OG0033957	Cellular Component	membrane(GO:0016020)	1
OG0033968	Cellular Component	cell part(GO:0044464)	1
OG0033968	Cellular Component	cell(GO:0005623)	1
OG0033968	Cellular Component	membrane(GO:0016020)	1
OG0033968	Cellular Component	organelle part(GO:0044422)	1
OG0033968	Cellular Component	organelle(GO:0043226)	1
OG0033987	Cellular Component	cell part(GO:0044464)	1
OG0033987	Cellular Component	cell(GO:0005623)	1
OG0033987	Cellular Component	membrane(GO:0016020)	1
OG0033987	Cellular Component	organelle part(GO:0044422)	1
OG0033987	Cellular Component	organelle(GO:0043226)	1
OG0033988	Cellular Component	cell part(GO:0044464)	1
OG0033988	Cellular Component	cell(GO:0005623)	1
OG0033988	Cellular Component	extracellular region(GO:0005576)	1
OG0033988	Cellular Component	membrane(GO:0016020)	1
OG0033988	Cellular Component	organelle part(GO:0044422)	1
OG0033988	Cellular Component	organelle(GO:0043226)	1
OG0033990	Cellular Component	cell part(GO:0044464)	1
OG0033990	Cellular Component	cell(GO:0005623)	1
OG0033990	Cellular Component	organelle(GO:0043226)	1
OG0033991	Cellular Component	cell part(GO:0044464)	1
OG0033991	Cellular Component	cell(GO:0005623)	1
OG0033991	Cellular Component	organelle(GO:0043226)	1
OG0033997	Cellular Component	cell part(GO:0044464)	1
OG0033997	Cellular Component	cell(GO:0005623)	1
OG0033997	Cellular Component	membrane(GO:0016020)	1
OG0034002	Cellular Component	cell part(GO:0044464)	1
OG0034002	Cellular Component	cell(GO:0005623)	1
OG0034002	Cellular Component	organelle(GO:0043226)	1
OG0034003	Cellular Component	cell part(GO:0044464)	1
OG0034003	Cellular Component	cell(GO:0005623)	1
OG0034003	Cellular Component	organelle part(GO:0044422)	1
OG0034003	Cellular Component	organelle(GO:0043226)	1
OG0034003	Cellular Component	protein-containing complex(GO:0032991)	1
OG0034004	Cellular Component	cell part(GO:0044464)	1
OG0034004	Cellular Component	cell(GO:0005623)	1
OG0034004	Cellular Component	organelle(GO:0043226)	1
OG0034006	Cellular Component	cell part(GO:0044464)	1
OG0034006	Cellular Component	cell(GO:0005623)	1
OG0034006	Cellular Component	organelle(GO:0043226)	1
OG0034012	Cellular Component	cell part(GO:0044464)	1
OG0034012	Cellular Component	cell(GO:0005623)	1
OG0034012	Cellular Component	membrane(GO:0016020)	1
OG0034028	Cellular Component	cell part(GO:0044464)	1

OG0034028	Cellular Component	cell(GO:0005623)	1
OG0034028	Cellular Component	membrane(GO:0016020)	1
OG0034029	Cellular Component	cell part(GO:0044464)	1
OG0034029	Cellular Component	cell(GO:0005623)	1
OG0034029	Cellular Component	membrane part(GO:0044425)	1
OG0034029	Cellular Component	membrane(GO:0016020)	1
OG0034029	Cellular Component	membrane-enclosed lumen(GO:0031974)	1
OG0034029	Cellular Component	organelle part(GO:0044422)	1
OG0034029	Cellular Component	organelle(GO:0043226)	1
OG0034029	Cellular Component	protein-containing complex(GO:0032991)	1
OG0034034	Cellular Component	cell part(GO:0044464)	1
OG0034034	Cellular Component	cell(GO:0005623)	1
OG0034034	Cellular Component	membrane(GO:0016020)	1
OG0034034	Cellular Component	membrane-enclosed lumen(GO:0031974)	1
OG0034034	Cellular Component	organelle part(GO:0044422)	1
OG0034034	Cellular Component	organelle(GO:0043226)	1
OG0034034	Cellular Component	protein-containing complex(GO:0032991)	1
OG0034035	Cellular Component	cell part(GO:0044464)	1
OG0034035	Cellular Component	cell(GO:0005623)	1
OG0034035	Cellular Component	membrane(GO:0016020)	1
OG0034036	Cellular Component	membrane part(GO:0044425)	1
OG0034036	Cellular Component	membrane(GO:0016020)	1
OG0034042	Cellular Component	cell part(GO:0044464)	1
OG0034042	Cellular Component	cell(GO:0005623)	1
OG0034042	Cellular Component	organelle(GO:0043226)	1
OG0034043	Cellular Component	cell part(GO:0044464)	1
OG0034043	Cellular Component	cell(GO:0005623)	1
OG0034043	Cellular Component	organelle(GO:0043226)	1
OG0034051	Cellular Component	cell part(GO:0044464)	1
OG0034051	Cellular Component	cell(GO:0005623)	1
OG0034051	Cellular Component	protein-containing complex(GO:0032991)	1
OG0034055	Cellular Component	cell part(GO:0044464)	1
OG0034055	Cellular Component	cell(GO:0005623)	1
OG0034055	Cellular Component	organelle(GO:0043226)	1
OG0034056	Cellular Component	cell part(GO:0044464)	1
OG0034056	Cellular Component	cell(GO:0005623)	1
OG0034056	Cellular Component	organelle(GO:0043226)	1
OG0034060	Cellular Component	cell junction(GO:0030054)	1
OG0034060	Cellular Component	cell part(GO:0044464)	1
OG0034060	Cellular Component	cell(GO:0005623)	1
OG0034060	Cellular Component	membrane(GO:0016020)	1
OG0034060	Cellular Component	organelle(GO:0043226)	1
OG0034060	Cellular Component	symplast(GO:0055044)	1
OG0034063	Cellular Component	cell part(GO:0044464)	1
OG0034063	Cellular Component	cell(GO:0005623)	1
OG0034063	Cellular Component	organelle(GO:0043226)	1

OG0034069	Cellular Component	cell part(GO:0044464)	1
OG0034069	Cellular Component	cell(GO:0005623)	1
OG0034069	Cellular Component	membrane(GO:0016020)	1
OG0034070	Cellular Component	cell part(GO:0044464)	1
OG0034070	Cellular Component	cell(GO:0005623)	1
OG0034070	Cellular Component	membrane(GO:0016020)	1
OG0034072	Cellular Component	cell part(GO:0044464)	1
OG0034072	Cellular Component	cell(GO:0005623)	1
OG0034072	Cellular Component	membrane(GO:0016020)	1
OG0034076	Cellular Component	cell part(GO:0044464)	1
OG0034076	Cellular Component	cell(GO:0005623)	1
OG0034076	Cellular Component	membrane(GO:0016020)	1
OG0034078	Cellular Component	cell part(GO:0044464)	1
OG0034078	Cellular Component	cell(GO:0005623)	1
OG0034078	Cellular Component	membrane(GO:0016020)	1
OG0034078	Cellular Component	organelle(GO:0043226)	1
OG0034079	Cellular Component	cell part(GO:0044464)	1
OG0034079	Cellular Component	cell(GO:0005623)	1
OG0034079	Cellular Component	membrane(GO:0016020)	1
OG0034080	Cellular Component	cell part(GO:0044464)	1
OG0034080	Cellular Component	cell(GO:0005623)	1
OG0034080	Cellular Component	membrane(GO:0016020)	1
OG0034081	Cellular Component	cell part(GO:0044464)	1
OG0034081	Cellular Component	cell(GO:0005623)	1
OG0034081	Cellular Component	membrane(GO:0016020)	1
OG0034084	Cellular Component	cell part(GO:0044464)	1
OG0034084	Cellular Component	cell(GO:0005623)	1
OG0034084	Cellular Component	membrane(GO:0016020)	1
OG0034086	Cellular Component	cell part(GO:0044464)	1
OG0034086	Cellular Component	cell(GO:0005623)	1
OG0034089	Cellular Component	cell part(GO:0044464)	1
OG0034089	Cellular Component	cell(GO:0005623)	1
OG0034089	Cellular Component	extracellular region(GO:0005576)	1
OG0034089	Cellular Component	membrane(GO:0016020)	1
OG0034090	Cellular Component	cell part(GO:0044464)	1
OG0034090	Cellular Component	cell(GO:0005623)	1
OG0034090	Cellular Component	organelle(GO:0043226)	1
OG0034090	Cellular Component	protein-containing complex(GO:0032991)	1
OG0034091	Cellular Component	cell part(GO:0044464)	1
OG0034091	Cellular Component	cell(GO:0005623)	1
OG0034091	Cellular Component	extracellular region(GO:0005576)	1
OG0034091	Cellular Component	membrane(GO:0016020)	1
OG0034092	Cellular Component	cell part(GO:0044464)	1
OG0034092	Cellular Component	cell(GO:0005623)	1
OG0034093	Cellular Component	cell part(GO:0044464)	1
OG0034093	Cellular Component	cell(GO:0005623)	1
OG0034096	Cellular Component	cell part(GO:0044464)	1

OG0034096	Cellular Component	cell(GO:0005623)	1
OG0034097	Cellular Component	cell part(GO:0044464)	1
OG0034097	Cellular Component	cell(GO:0005623)	1
OG0034097	Cellular Component	membrane part(GO:0044425)	1
OG0034097	Cellular Component	membrane(GO:0016020)	1
OG0034098	Cellular Component	cell part(GO:0044464)	1
OG0034098	Cellular Component	cell(GO:0005623)	1
OG0034098	Cellular Component	membrane(GO:0016020)	1
OG0034100	Cellular Component	cell part(GO:0044464)	1
OG0034100	Cellular Component	cell(GO:0005623)	1
OG0034101	Cellular Component	cell part(GO:0044464)	1
OG0034101	Cellular Component	cell(GO:0005623)	1
OG0034102	Cellular Component	cell part(GO:0044464)	1
OG0034102	Cellular Component	cell(GO:0005623)	1
OG0034102	Cellular Component	membrane(GO:0016020)	1
OG0034105	Cellular Component	cell part(GO:0044464)	1
OG0034105	Cellular Component	cell(GO:0005623)	1
OG0034105	Cellular Component	membrane(GO:0016020)	1
OG0034105	Cellular Component	protein-containing complex(GO:0032991)	1
OG0034108	Cellular Component	cell part(GO:0044464)	1
OG0034108	Cellular Component	cell(GO:0005623)	1
OG0034113	Cellular Component	cell part(GO:0044464)	1
OG0034113	Cellular Component	cell(GO:0005623)	1
OG0034113	Cellular Component	membrane(GO:0016020)	1
OG0034114	Cellular Component	cell part(GO:0044464)	1
OG0034114	Cellular Component	cell(GO:0005623)	1
OG0034114	Cellular Component	protein-containing complex(GO:0032991)	1
OG0034115	Cellular Component	cell part(GO:0044464)	1
OG0034115	Cellular Component	cell(GO:0005623)	1
OG0034115	Cellular Component	membrane part(GO:0044425)	1
OG0034115	Cellular Component	membrane(GO:0016020)	1
OG0034118	Cellular Component	cell part(GO:0044464)	1
OG0034118	Cellular Component	cell(GO:0005623)	1
OG0034118	Cellular Component	membrane(GO:0016020)	1
OG0034122	Cellular Component	cell part(GO:0044464)	1
OG0034122	Cellular Component	cell(GO:0005623)	1
OG0034122	Cellular Component	membrane(GO:0016020)	1
OG0034123	Cellular Component	cell part(GO:0044464)	1
OG0034123	Cellular Component	cell(GO:0005623)	1
OG0034131	Cellular Component	cell part(GO:0044464)	1
OG0034131	Cellular Component	cell(GO:0005623)	1
OG0034133	Cellular Component	cell part(GO:0044464)	1
OG0034133	Cellular Component	cell(GO:0005623)	1
OG0034135	Cellular Component	cell part(GO:0044464)	1
OG0034135	Cellular Component	cell(GO:0005623)	1
OG0034135	Cellular Component	membrane(GO:0016020)	1
OG0034136	Cellular Component	cell part(GO:0044464)	1

OG0034136	Cellular Component	cell(GO:0005623)	1
OG0034136	Cellular Component	membrane(GO:0016020)	1
OG0034142	Cellular Component	cell part(GO:0044464)	1
OG0034142	Cellular Component	cell(GO:0005623)	1
OG0034146	Cellular Component	cell part(GO:0044464)	1
OG0034146	Cellular Component	cell(GO:0005623)	1
OG0034146	Cellular Component	membrane part(GO:0044425)	1
OG0034146	Cellular Component	membrane(GO:0016020)	1
OG0034146	Cellular Component	organelle part(GO:0044422)	1
OG0034146	Cellular Component	organelle(GO:0043226)	1
OG0034146	Cellular Component	protein-containing complex(GO:0032991)	1
OG0034147	Cellular Component	cell part(GO:0044464)	1
OG0034147	Cellular Component	cell(GO:0005623)	1
OG0034147	Cellular Component	membrane(GO:0016020)	1
OG0034152	Cellular Component	cell part(GO:0044464)	1
OG0034152	Cellular Component	cell(GO:0005623)	1
OG0034152	Cellular Component	membrane(GO:0016020)	1
OG0034153	Cellular Component	cell part(GO:0044464)	1
OG0034153	Cellular Component	cell(GO:0005623)	1
OG0034155	Cellular Component	cell part(GO:0044464)	1
OG0034155	Cellular Component	cell(GO:0005623)	1
OG0034155	Cellular Component	membrane(GO:0016020)	1
OG0034157	Cellular Component	cell part(GO:0044464)	1
OG0034157	Cellular Component	cell(GO:0005623)	1
OG0034157	Cellular Component	membrane part(GO:0044425)	1
OG0034157	Cellular Component	membrane(GO:0016020)	1
OG0034157	Cellular Component	protein-containing complex(GO:0032991)	1
OG0034158	Cellular Component	cell part(GO:0044464)	1
OG0034158	Cellular Component	cell(GO:0005623)	1
OG0034158	Cellular Component	membrane part(GO:0044425)	1
OG0034158	Cellular Component	membrane(GO:0016020)	1
OG0034158	Cellular Component	protein-containing complex(GO:0032991)	1
OG0034159	Cellular Component	cell part(GO:0044464)	1
OG0034159	Cellular Component	cell(GO:0005623)	1
OG0034160	Cellular Component	cell part(GO:0044464)	1
OG0034160	Cellular Component	cell(GO:0005623)	1
OG0034160	Cellular Component	membrane(GO:0016020)	1
OG0034161	Cellular Component	cell part(GO:0044464)	1
OG0034161	Cellular Component	cell(GO:0005623)	1
OG0034162	Cellular Component	cell part(GO:0044464)	1
OG0034162	Cellular Component	cell(GO:0005623)	1
OG0034163	Cellular Component	cell part(GO:0044464)	1
OG0034163	Cellular Component	cell(GO:0005623)	1
OG0034163	Cellular Component	membrane(GO:0016020)	1
OG0034163	Cellular Component	nucleoid(GO:0009295)	1
OG0034166	Cellular Component	cell part(GO:0044464)	1

OG0034166	Cellular Component	cell(GO:0005623)	1
OG0034169	Cellular Component	cell part(GO:0044464)	1
OG0034169	Cellular Component	cell(GO:0005623)	1
OG0034169	Cellular Component	membrane(GO:0016020)	1
OG0034175	Cellular Component	cell part(GO:0044464)	1
OG0034175	Cellular Component	cell(GO:0005623)	1
OG0034176	Cellular Component	cell part(GO:0044464)	1
OG0034176	Cellular Component	cell(GO:0005623)	1
OG0034179	Cellular Component	cell part(GO:0044464)	1
OG0034179	Cellular Component	cell(GO:0005623)	1
OG0034179	Cellular Component	membrane(GO:0016020)	1
OG0034180	Cellular Component	cell part(GO:0044464)	1
OG0034180	Cellular Component	cell(GO:0005623)	1
OG0034180	Cellular Component	membrane(GO:0016020)	1
OG0034181	Cellular Component	cell part(GO:0044464)	1
OG0034181	Cellular Component	cell(GO:0005623)	1
OG0034182	Cellular Component	cell part(GO:0044464)	1
OG0034182	Cellular Component	cell(GO:0005623)	1
OG0034182	Cellular Component	extracellular region(GO:0005576)	1
OG0034182	Cellular Component	membrane(GO:0016020)	1
OG0034182	Cellular Component	protein-containing complex(GO:0032991)	1
OG0034183	Cellular Component	cell part(GO:0044464)	1
OG0034183	Cellular Component	cell(GO:0005623)	1
OG0034183	Cellular Component	membrane(GO:0016020)	1
OG0034186	Cellular Component	cell part(GO:0044464)	1
OG0034186	Cellular Component	cell(GO:0005623)	1
OG0034186	Cellular Component	membrane(GO:0016020)	1
OG0034186	Cellular Component	organelle part(GO:0044422)	1
OG0034186	Cellular Component	organelle(GO:0043226)	1
OG0034187	Cellular Component	cell part(GO:0044464)	1
OG0034187	Cellular Component	cell(GO:0005623)	1
OG0034188	Cellular Component	cell part(GO:0044464)	1
OG0034188	Cellular Component	cell(GO:0005623)	1
OG0034189	Cellular Component	cell part(GO:0044464)	1
OG0034189	Cellular Component	cell(GO:0005623)	1
OG0034189	Cellular Component	membrane(GO:0016020)	1
OG0034190	Cellular Component	cell part(GO:0044464)	1
OG0034190	Cellular Component	cell(GO:0005623)	1
OG0034190	Cellular Component	membrane(GO:0016020)	1
OG0034191	Cellular Component	cell part(GO:0044464)	1
OG0034191	Cellular Component	cell(GO:0005623)	1
OG0034192	Cellular Component	cell part(GO:0044464)	1
OG0034192	Cellular Component	cell(GO:0005623)	1
OG0034193	Cellular Component	cell part(GO:0044464)	1
OG0034193	Cellular Component	cell(GO:0005623)	1
OG0034193	Cellular Component	membrane(GO:0016020)	1
OG0034195	Cellular Component	cell part(GO:0044464)	1

OG0034195	Cellular Component	cell(GO:0005623)	1
OG0034196	Cellular Component	cell part(GO:0044464)	1
OG0034196	Cellular Component	cell(GO:0005623)	1
OG0034197	Cellular Component	cell part(GO:0044464)	1
OG0034197	Cellular Component	cell(GO:0005623)	1
OG0034197	Cellular Component	membrane(GO:0016020)	1
OG0034198	Cellular Component	cell part(GO:0044464)	1
OG0034198	Cellular Component	cell(GO:0005623)	1
OG0034198	Cellular Component	membrane part(GO:0044425)	1
OG0034198	Cellular Component	membrane(GO:0016020)	1
OG0034200	Cellular Component	cell part(GO:0044464)	1
OG0034200	Cellular Component	cell(GO:0005623)	1
OG0034200	Cellular Component	membrane(GO:0016020)	1
OG0034203	Cellular Component	membrane part(GO:0044425)	1
OG0034203	Cellular Component	membrane(GO:0016020)	1
OG0034206	Cellular Component	cell part(GO:0044464)	1
OG0034206	Cellular Component	cell(GO:0005623)	1
OG0034207	Cellular Component	cell part(GO:0044464)	1
OG0034207	Cellular Component	cell(GO:0005623)	1
OG0034207	Cellular Component	membrane part(GO:0044425)	1
OG0034207	Cellular Component	membrane(GO:0016020)	1
OG0034207	Cellular Component	organelle part(GO:0044422)	1
OG0034207	Cellular Component	organelle(GO:0043226)	1
OG0034207	Cellular Component	protein-containing complex(GO:0032991)	1
OG0034209	Cellular Component	cell part(GO:0044464)	1
OG0034209	Cellular Component	cell(GO:0005623)	1
OG0034209	Cellular Component	membrane(GO:0016020)	1
OG0034212	Cellular Component	cell part(GO:0044464)	1
OG0034212	Cellular Component	cell(GO:0005623)	1
OG0034213	Cellular Component	cell part(GO:0044464)	1
OG0034213	Cellular Component	cell(GO:0005623)	1
OG0034213	Cellular Component	membrane(GO:0016020)	1
OG0034214	Cellular Component	cell part(GO:0044464)	1
OG0034214	Cellular Component	cell(GO:0005623)	1
OG0034214	Cellular Component	membrane part(GO:0044425)	1
OG0034214	Cellular Component	membrane(GO:0016020)	1
OG0034214	Cellular Component	organelle part(GO:0044422)	1
OG0034214	Cellular Component	organelle(GO:0043226)	1
OG0034214	Cellular Component	protein-containing complex(GO:0032991)	1
OG0034215	Cellular Component	cell part(GO:0044464)	1
OG0034215	Cellular Component	cell(GO:0005623)	1
OG0034215	Cellular Component	extracellular region(GO:0005576)	1
OG0034215	Cellular Component	membrane(GO:0016020)	1
OG0034216	Cellular Component	cell part(GO:0044464)	1
OG0034216	Cellular Component	cell(GO:0005623)	1
OG0034220	Cellular Component	cell part(GO:0044464)	1
OG0034220	Cellular Component	cell(GO:0005623)	1

OG0034220	Cellular Component	membrane(GO:0016020)	1
OG0034225	Cellular Component	cell part(GO:0044464)	1
OG0034225	Cellular Component	cell(GO:0005623)	1
OG0034225	Cellular Component	membrane(GO:0016020)	1
OG0034226	Cellular Component	cell part(GO:0044464)	1
OG0034226	Cellular Component	cell(GO:0005623)	1
OG0034227	Cellular Component	cell part(GO:0044464)	1
OG0034227	Cellular Component	cell(GO:0005623)	1
OG0034227	Cellular Component	membrane(GO:0016020)	1
OG0034228	Cellular Component	cell part(GO:0044464)	1
OG0034228	Cellular Component	cell(GO:0005623)	1
OG0034228	Cellular Component	membrane(GO:0016020)	1
OG0034229	Cellular Component	cell part(GO:0044464)	1
OG0034229	Cellular Component	cell(GO:0005623)	1
OG0034232	Cellular Component	cell part(GO:0044464)	1
OG0034232	Cellular Component	cell(GO:0005623)	1
OG0034233	Cellular Component	cell part(GO:0044464)	1
OG0034233	Cellular Component	cell(GO:0005623)	1
OG0034233	Cellular Component	membrane(GO:0016020)	1
OG0034235	Cellular Component	cell part(GO:0044464)	1
OG0034235	Cellular Component	cell(GO:0005623)	1
OG0034235	Cellular Component	membrane(GO:0016020)	1
OG0034238	Cellular Component	cell part(GO:0044464)	1
OG0034238	Cellular Component	cell(GO:0005623)	1
OG0034239	Cellular Component	cell part(GO:0044464)	1
OG0034239	Cellular Component	cell(GO:0005623)	1
OG0034240	Cellular Component	cell part(GO:0044464)	1
OG0034240	Cellular Component	cell(GO:0005623)	1
OG0034240	Cellular Component	membrane(GO:0016020)	1
OG0034241	Cellular Component	cell part(GO:0044464)	1
OG0034241	Cellular Component	cell(GO:0005623)	1
OG0034241	Cellular Component	membrane(GO:0016020)	1
OG0034247	Cellular Component	cell part(GO:0044464)	1
OG0034247	Cellular Component	cell(GO:0005623)	1
OG0034247	Cellular Component	extracellular region(GO:0005576)	1
OG0034247	Cellular Component	membrane part(GO:0044425)	1
OG0034247	Cellular Component	membrane(GO:0016020)	1
OG0034248	Cellular Component	cell part(GO:0044464)	1
OG0034248	Cellular Component	cell(GO:0005623)	1
OG0034249	Cellular Component	cell part(GO:0044464)	1
OG0034249	Cellular Component	cell(GO:0005623)	1
OG0034252	Cellular Component	cell part(GO:0044464)	1
OG0034252	Cellular Component	cell(GO:0005623)	1
OG0034252	Cellular Component	protein-containing complex(GO:0032991)	1
OG0034254	Cellular Component	cell part(GO:0044464)	1
OG0034254	Cellular Component	cell(GO:0005623)	1
OG0034254	Cellular Component	membrane(GO:0016020)	1

OG0034254	Cellular Component	protein-containing complex(GO:0032991)	1
OG0034260	Cellular Component	cell part(GO:0044464)	1
OG0034260	Cellular Component	cell(GO:0005623)	1
OG0034260	Cellular Component	membrane(GO:0016020)	1
OG0034261	Cellular Component	cell part(GO:0044464)	1
OG0034261	Cellular Component	cell(GO:0005623)	1
OG0034261	Cellular Component	membrane(GO:0016020)	1
OG0034263	Cellular Component	cell part(GO:0044464)	1
OG0034263	Cellular Component	cell(GO:0005623)	1
OG0034263	Cellular Component	membrane(GO:0016020)	1
OG0034268	Cellular Component	cell part(GO:0044464)	1
OG0034268	Cellular Component	cell(GO:0005623)	1
OG0034268	Cellular Component	membrane part(GO:0044425)	1
OG0034268	Cellular Component	membrane(GO:0016020)	1
OG0034268	Cellular Component	protein-containing complex(GO:0032991)	1
OG0034269	Cellular Component	cell part(GO:0044464)	1
OG0034269	Cellular Component	cell(GO:0005623)	1
OG0034270	Cellular Component	cell part(GO:0044464)	1
OG0034270	Cellular Component	cell(GO:0005623)	1
OG0034271	Cellular Component	cell part(GO:0044464)	1
OG0034271	Cellular Component	cell(GO:0005623)	1
OG0034271	Cellular Component	membrane(GO:0016020)	1
OG0034274	Cellular Component	cell part(GO:0044464)	1
OG0034274	Cellular Component	cell(GO:0005623)	1
OG0034278	Cellular Component	cell part(GO:0044464)	1
OG0034278	Cellular Component	cell(GO:0005623)	1
OG0034279	Cellular Component	cell part(GO:0044464)	1
OG0034279	Cellular Component	cell(GO:0005623)	1
OG0034283	Cellular Component	cell part(GO:0044464)	1
OG0034283	Cellular Component	cell(GO:0005623)	1
OG0034283	Cellular Component	extracellular region(GO:0005576)	1
OG0034283	Cellular Component	membrane(GO:0016020)	1
OG0034284	Cellular Component	cell part(GO:0044464)	1
OG0034284	Cellular Component	cell(GO:0005623)	1
OG0034284	Cellular Component	membrane(GO:0016020)	1
OG0034285	Cellular Component	cell part(GO:0044464)	1
OG0034285	Cellular Component	cell(GO:0005623)	1
OG0034285	Cellular Component	membrane(GO:0016020)	1
OG0034286	Cellular Component	cell part(GO:0044464)	1
OG0034286	Cellular Component	cell(GO:0005623)	1
OG0034286	Cellular Component	membrane(GO:0016020)	1
OG0034286	Cellular Component	nucleoid(GO:0009295)	1
OG0034290	Cellular Component	cell part(GO:0044464)	1
OG0034290	Cellular Component	cell(GO:0005623)	1
OG0034290	Cellular Component	membrane(GO:0016020)	1
OG0034291	Cellular Component	cell part(GO:0044464)	1
OG0034291	Cellular Component	cell(GO:0005623)	1

OG0034292	Cellular Component	cell part(GO:0044464)	1
OG0034292	Cellular Component	cell(GO:0005623)	1
OG0034295	Cellular Component	cell part(GO:0044464)	1
OG0034295	Cellular Component	cell(GO:0005623)	1
OG0034295	Cellular Component	membrane(GO:0016020)	1
OG0034299	Cellular Component	cell part(GO:0044464)	1
OG0034299	Cellular Component	cell(GO:0005623)	1
OG0034299	Cellular Component	membrane(GO:0016020)	1
OG0034300	Cellular Component	cell part(GO:0044464)	1
OG0034300	Cellular Component	cell(GO:0005623)	1
OG0034300	Cellular Component	membrane(GO:0016020)	1
OG0034301	Cellular Component	cell part(GO:0044464)	1
OG0034301	Cellular Component	cell(GO:0005623)	1
OG0034301	Cellular Component	membrane(GO:0016020)	1
OG0034302	Cellular Component	cell part(GO:0044464)	1
OG0034302	Cellular Component	cell(GO:0005623)	1
OG0034302	Cellular Component	membrane(GO:0016020)	1
OG0034305	Cellular Component	cell part(GO:0044464)	1
OG0034305	Cellular Component	cell(GO:0005623)	1
OG0034305	Cellular Component	membrane(GO:0016020)	1
OG0034306	Cellular Component	cell part(GO:0044464)	1
OG0034306	Cellular Component	cell(GO:0005623)	1
OG0034306	Cellular Component	membrane(GO:0016020)	1
OG0034307	Cellular Component	cell part(GO:0044464)	1
OG0034307	Cellular Component	cell(GO:0005623)	1
OG0034307	Cellular Component	membrane(GO:0016020)	1
OG0034308	Cellular Component	cell part(GO:0044464)	1
OG0034308	Cellular Component	cell(GO:0005623)	1
OG0034308	Cellular Component	membrane(GO:0016020)	1
OG0034308	Cellular Component	organelle part(GO:0044422)	1
OG0034308	Cellular Component	organelle(GO:0043226)	1
OG0034308	Cellular Component	protein-containing complex(GO:0032991)	1
OG0034309	Cellular Component	cell part(GO:0044464)	1
OG0034309	Cellular Component	cell(GO:0005623)	1
OG0034309	Cellular Component	membrane(GO:0016020)	1
OG0034310	Cellular Component	cell part(GO:0044464)	1
OG0034310	Cellular Component	cell(GO:0005623)	1
OG0034310	Cellular Component	membrane(GO:0016020)	1
OG0034311	Cellular Component	cell part(GO:0044464)	1
OG0034311	Cellular Component	cell(GO:0005623)	1
OG0034311	Cellular Component	membrane(GO:0016020)	1
OG0034312	Cellular Component	cell part(GO:0044464)	1
OG0034312	Cellular Component	cell(GO:0005623)	1
OG0034312	Cellular Component	membrane(GO:0016020)	1
OG0034313	Cellular Component	cell part(GO:0044464)	1
OG0034313	Cellular Component	cell(GO:0005623)	1
OG0034313	Cellular Component	membrane(GO:0016020)	1

OG0034315	Cellular Component	cell part(GO:0044464)	1
OG0034315	Cellular Component	cell(GO:0005623)	1
OG0034315	Cellular Component	membrane(GO:0016020)	1
OG0034316	Cellular Component	cell part(GO:0044464)	1
OG0034316	Cellular Component	cell(GO:0005623)	1
OG0034316	Cellular Component	membrane(GO:0016020)	1
OG0034317	Cellular Component	cell part(GO:0044464)	1
OG0034317	Cellular Component	cell(GO:0005623)	1
OG0034317	Cellular Component	membrane(GO:0016020)	1
OG0034320	Cellular Component	cell part(GO:0044464)	1
OG0034320	Cellular Component	cell(GO:0005623)	1
OG0034320	Cellular Component	membrane(GO:0016020)	1
OG0034323	Cellular Component	cell part(GO:0044464)	1
OG0034323	Cellular Component	cell(GO:0005623)	1
OG0034329	Cellular Component	cell part(GO:0044464)	1
OG0034329	Cellular Component	cell(GO:0005623)	1
OG0034330	Cellular Component	cell part(GO:0044464)	1
OG0034330	Cellular Component	cell(GO:0005623)	1
OG0034330	Cellular Component	membrane(GO:0016020)	1
OG0034331	Cellular Component	cell part(GO:0044464)	1
OG0034331	Cellular Component	cell(GO:0005623)	1
OG0034331	Cellular Component	membrane part(GO:0044425)	1
OG0034331	Cellular Component	membrane(GO:0016020)	1
OG0034331	Cellular Component	organelle part(GO:0044422)	1
OG0034331	Cellular Component	organelle(GO:0043226)	1
OG0034331	Cellular Component	protein-containing complex(GO:0032991)	1
OG0034333	Cellular Component	cell part(GO:0044464)	1
OG0034333	Cellular Component	cell(GO:0005623)	1
OG0034333	Cellular Component	membrane(GO:0016020)	1
OG0034339	Cellular Component	cell part(GO:0044464)	1
OG0034339	Cellular Component	cell(GO:0005623)	1
OG0034340	Cellular Component	cell part(GO:0044464)	1
OG0034340	Cellular Component	cell(GO:0005623)	1
OG0034340	Cellular Component	membrane(GO:0016020)	1
OG0034341	Cellular Component	cell part(GO:0044464)	1
OG0034341	Cellular Component	cell(GO:0005623)	1
OG0034341	Cellular Component	membrane part(GO:0044425)	1
OG0034341	Cellular Component	membrane(GO:0016020)	1
OG0034341	Cellular Component	organelle part(GO:0044422)	1
OG0034341	Cellular Component	organelle(GO:0043226)	1
OG0034341	Cellular Component	protein-containing complex(GO:0032991)	1
OG0034347	Cellular Component	cell part(GO:0044464)	1
OG0034347	Cellular Component	cell(GO:0005623)	1
OG0034347	Cellular Component	membrane(GO:0016020)	1
OG0034365	Cellular Component	cell part(GO:0044464)	1
OG0034365	Cellular Component	cell(GO:0005623)	1
OG0034365	Cellular Component	membrane(GO:0016020)	1

OG0034378	Cellular Component	membrane(GO:0016020)	1
OG0034383	Cellular Component	cell part(GO:0044464)	1
OG0034383	Cellular Component	cell(GO:0005623)	1
OG0034386	Cellular Component	cell part(GO:0044464)	1
OG0034386	Cellular Component	cell(GO:0005623)	1
OG0034386	Cellular Component	organelle(GO:0043226)	1
OG0034388	Cellular Component	cell part(GO:0044464)	1
OG0034388	Cellular Component	cell(GO:0005623)	1
OG0034388	Cellular Component	organelle(GO:0043226)	1
OG0034404	Cellular Component	cell part(GO:0044464)	1
OG0034404	Cellular Component	cell(GO:0005623)	1
OG0034404	Cellular Component	membrane(GO:0016020)	1
OG0034407	Cellular Component	cell part(GO:0044464)	1
OG0034407	Cellular Component	cell(GO:0005623)	1
OG0034407	Cellular Component	organelle(GO:0043226)	1
OG0034408	Cellular Component	cell part(GO:0044464)	1
OG0034408	Cellular Component	cell(GO:0005623)	1
OG0034408	Cellular Component	extracellular region(GO:0005576)	1
OG0034408	Cellular Component	organelle(GO:0043226)	1
OG0034425	Cellular Component	cell part(GO:0044464)	1
OG0034425	Cellular Component	cell(GO:0005623)	1
OG0034428	Cellular Component	cell part(GO:0044464)	1
OG0034428	Cellular Component	cell(GO:0005623)	1
OG0034452	Cellular Component	cell part(GO:0044464)	1
OG0034452	Cellular Component	cell(GO:0005623)	1
OG0034467	Cellular Component	cell part(GO:0044464)	1
OG0034467	Cellular Component	cell(GO:0005623)	1
OG0034467	Cellular Component	membrane(GO:0016020)	1
OG0034467	Cellular Component	organelle part(GO:0044422)	1
OG0034467	Cellular Component	organelle(GO:0043226)	1
OG0034468	Cellular Component	cell part(GO:0044464)	1
OG0034468	Cellular Component	cell(GO:0005623)	1
OG0034468	Cellular Component	membrane(GO:0016020)	1
OG0034468	Cellular Component	organelle part(GO:0044422)	1
OG0034468	Cellular Component	organelle(GO:0043226)	1
OG0034476	Cellular Component	cell part(GO:0044464)	1
OG0034476	Cellular Component	cell(GO:0005623)	1
OG0034482	Cellular Component	cell part(GO:0044464)	1
OG0034482	Cellular Component	cell(GO:0005623)	1
OG0034482	Cellular Component	organelle part(GO:0044422)	1
OG0034482	Cellular Component	organelle(GO:0043226)	1
OG0034482	Cellular Component	protein-containing complex(GO:0032991)	1
OG0034484	Cellular Component	cell part(GO:0044464)	1
OG0034484	Cellular Component	cell(GO:0005623)	1
OG0034484	Cellular Component	membrane(GO:0016020)	1
OG0034486	Cellular Component	cell part(GO:0044464)	1
OG0034486	Cellular Component	cell(GO:0005623)	1

OG0034486	Cellular Component	membrane(GO:0016020)	1
OG0034489	Cellular Component	cell part(GO:0044464)	1
OG0034489	Cellular Component	cell(GO:0005623)	1
OG0034489	Cellular Component	membrane(GO:0016020)	1
OG0034492	Cellular Component	cell junction(GO:0030054)	1
OG0034492	Cellular Component	cell part(GO:0044464)	1
OG0034492	Cellular Component	cell(GO:0005623)	1
OG0034492	Cellular Component	membrane(GO:0016020)	1
OG0034492	Cellular Component	symplast(GO:0055044)	1
OG0034493	Cellular Component	cell part(GO:0044464)	1
OG0034493	Cellular Component	cell(GO:0005623)	1
OG0034493	Cellular Component	membrane(GO:0016020)	1
OG0034493	Cellular Component	organelle part(GO:0044422)	1
OG0034493	Cellular Component	organelle(GO:0043226)	1
OG0034497	Cellular Component	cell part(GO:0044464)	1
OG0034497	Cellular Component	cell(GO:0005623)	1
OG0034497	Cellular Component	membrane(GO:0016020)	1
OG0034497	Cellular Component	organelle(GO:0043226)	1
OG0034498	Cellular Component	cell part(GO:0044464)	1
OG0034498	Cellular Component	cell(GO:0005623)	1
OG0034498	Cellular Component	membrane part(GO:0044425)	1
OG0034498	Cellular Component	membrane(GO:0016020)	1
OG0034498	Cellular Component	organelle part(GO:0044422)	1
OG0034498	Cellular Component	organelle(GO:0043226)	1
OG0034499	Cellular Component	cell junction(GO:0030054)	1
OG0034499	Cellular Component	cell part(GO:0044464)	1
OG0034499	Cellular Component	cell(GO:0005623)	1
OG0034499	Cellular Component	membrane(GO:0016020)	1
OG0034499	Cellular Component	organelle(GO:0043226)	1
OG0034499	Cellular Component	symplast(GO:0055044)	1
OG0034504	Cellular Component	cell part(GO:0044464)	1
OG0034504	Cellular Component	cell(GO:0005623)	1
OG0034504	Cellular Component	membrane(GO:0016020)	1
OG0034505	Cellular Component	cell part(GO:0044464)	1
OG0034505	Cellular Component	cell(GO:0005623)	1
OG0034505	Cellular Component	nucleoid(GO:0009295)	1
OG0034507	Cellular Component	cell part(GO:0044464)	1
OG0034507	Cellular Component	cell(GO:0005623)	1
OG0034507	Cellular Component	membrane(GO:0016020)	1
OG0034512	Cellular Component	cell part(GO:0044464)	1
OG0034512	Cellular Component	cell(GO:0005623)	1
OG0034512	Cellular Component	membrane(GO:0016020)	1
OG0034512	Cellular Component	organelle part(GO:0044422)	1
OG0034512	Cellular Component	organelle(GO:0043226)	1
OG0034517	Cellular Component	cell part(GO:0044464)	1
OG0034517	Cellular Component	cell(GO:0005623)	1
OG0034519	Cellular Component	cell junction(GO:0030054)	1

OG0034519	Cellular Component	cell part(GO:0044464)	1
OG0034519	Cellular Component	cell(GO:0005623)	1
OG0034519	Cellular Component	membrane(GO:0016020)	1
OG0034519	Cellular Component	organelle part(GO:0044422)	1
OG0034519	Cellular Component	organelle(GO:0043226)	1
OG0034519	Cellular Component	protein-containing complex(GO:0032991)	1
OG0034519	Cellular Component	symplast(GO:0055044)	1
OG0034523	Cellular Component	cell part(GO:0044464)	1
OG0034523	Cellular Component	cell(GO:0005623)	1
OG0034523	Cellular Component	membrane(GO:0016020)	1
OG0034529	Cellular Component	cell part(GO:0044464)	1
OG0034529	Cellular Component	cell(GO:0005623)	1
OG0034533	Cellular Component	cell part(GO:0044464)	1
OG0034533	Cellular Component	cell(GO:0005623)	1
OG0034538	Cellular Component	cell part(GO:0044464)	1
OG0034538	Cellular Component	cell(GO:0005623)	1
OG0034538	Cellular Component	membrane part(GO:0044425)	1
OG0034538	Cellular Component	membrane(GO:0016020)	1
OG0034538	Cellular Component	organelle part(GO:0044422)	1
OG0034538	Cellular Component	organelle(GO:0043226)	1
OG0034538	Cellular Component	protein-containing complex(GO:0032991)	1
OG0034539	Cellular Component	cell part(GO:0044464)	1
OG0034539	Cellular Component	cell(GO:0005623)	1
OG0034539	Cellular Component	membrane(GO:0016020)	1
OG0034540	Cellular Component	cell part(GO:0044464)	1
OG0034540	Cellular Component	cell(GO:0005623)	1
OG0034540	Cellular Component	membrane part(GO:0044425)	1
OG0034540	Cellular Component	membrane(GO:0016020)	1
OG0034540	Cellular Component	organelle part(GO:0044422)	1
OG0034540	Cellular Component	organelle(GO:0043226)	1
OG0034540	Cellular Component	protein-containing complex(GO:0032991)	1
OG0034543	Cellular Component	cell part(GO:0044464)	1
OG0034543	Cellular Component	cell(GO:0005623)	1
OG0034543	Cellular Component	nucleoid(GO:0009295)	1
OG0034548	Cellular Component	cell part(GO:0044464)	1
OG0034548	Cellular Component	cell(GO:0005623)	1
OG0034555	Cellular Component	cell part(GO:0044464)	1
OG0034555	Cellular Component	cell(GO:0005623)	1
OG0034555	Cellular Component	membrane part(GO:0044425)	1
OG0034555	Cellular Component	membrane(GO:0016020)	1
OG0034556	Cellular Component	cell part(GO:0044464)	1
OG0034556	Cellular Component	cell(GO:0005623)	1
OG0034556	Cellular Component	organelle(GO:0043226)	1
OG0034557	Cellular Component	cell part(GO:0044464)	1
OG0034557	Cellular Component	cell(GO:0005623)	1
OG0034557	Cellular Component	organelle(GO:0043226)	1

OG0034559	Cellular Component	cell part(GO:0044464)	1
OG0034559	Cellular Component	cell(GO:0005623)	1
OG0034560	Cellular Component	cell part(GO:0044464)	1
OG0034560	Cellular Component	cell(GO:0005623)	1
OG0034561	Cellular Component	cell part(GO:0044464)	1
OG0034561	Cellular Component	cell(GO:0005623)	1
OG0034562	Cellular Component	cell part(GO:0044464)	1
OG0034562	Cellular Component	cell(GO:0005623)	1
OG0034564	Cellular Component	cell part(GO:0044464)	1
OG0034564	Cellular Component	cell(GO:0005623)	1
OG0034567	Cellular Component	cell part(GO:0044464)	1
OG0034567	Cellular Component	cell(GO:0005623)	1
OG0034567	Cellular Component	protein-containing complex(GO:0032991)	1
OG0034568	Cellular Component	cell part(GO:0044464)	1
OG0034568	Cellular Component	cell(GO:0005623)	1
OG0034568	Cellular Component	organelle(GO:0043226)	1
OG0034568	Cellular Component	protein-containing complex(GO:0032991)	1
OG0034570	Cellular Component	cell part(GO:0044464)	1
OG0034570	Cellular Component	cell(GO:0005623)	1
OG0034570	Cellular Component	membrane(GO:0016020)	1
OG0034571	Cellular Component	cell part(GO:0044464)	1
OG0034571	Cellular Component	cell(GO:0005623)	1
OG0034572	Cellular Component	cell part(GO:0044464)	1
OG0034572	Cellular Component	cell(GO:0005623)	1
OG0034572	Cellular Component	membrane(GO:0016020)	1
OG0034576	Cellular Component	cell part(GO:0044464)	1
OG0034576	Cellular Component	cell(GO:0005623)	1
OG0034576	Cellular Component	membrane(GO:0016020)	1
OG0034577	Cellular Component	cell part(GO:0044464)	1
OG0034577	Cellular Component	cell(GO:0005623)	1
OG0034577	Cellular Component	membrane(GO:0016020)	1
OG0034577	Cellular Component	organelle part(GO:0044422)	1
OG0034577	Cellular Component	organelle(GO:0043226)	1
OG0034577	Cellular Component	protein-containing complex(GO:0032991)	1
OG0034579	Cellular Component	cell part(GO:0044464)	1
OG0034579	Cellular Component	cell(GO:0005623)	1
OG0034580	Cellular Component	cell part(GO:0044464)	1
OG0034580	Cellular Component	cell(GO:0005623)	1
OG0034580	Cellular Component	membrane(GO:0016020)	1
OG0034581	Cellular Component	cell part(GO:0044464)	1
OG0034581	Cellular Component	cell(GO:0005623)	1
OG0034581	Cellular Component	membrane(GO:0016020)	1
OG0034581	Cellular Component	organelle(GO:0043226)	1
OG0034581	Cellular Component	protein-containing complex(GO:0032991)	1
OG0034582	Cellular Component	cell part(GO:0044464)	1
OG0034582	Cellular Component	cell(GO:0005623)	1

OG0034582	Cellular Component	membrane(GO:0016020)	1
OG0034583	Cellular Component	cell part(GO:0044464)	1
OG0034583	Cellular Component	cell(GO:0005623)	1
OG0034584	Cellular Component	cell part(GO:0044464)	1
OG0034584	Cellular Component	cell(GO:0005623)	1
OG0034584	Cellular Component	membrane(GO:0016020)	1
OG0034585	Cellular Component	cell part(GO:0044464)	1
OG0034585	Cellular Component	cell(GO:0005623)	1
OG0034585	Cellular Component	extracellular region(GO:0005576)	1
OG0034585	Cellular Component	membrane part(GO:0044425)	1
OG0034585	Cellular Component	membrane(GO:0016020)	1
OG0034586	Cellular Component	cell part(GO:0044464)	1
OG0034586	Cellular Component	cell(GO:0005623)	1
OG0034586	Cellular Component	membrane(GO:0016020)	1
OG0034587	Cellular Component	cell part(GO:0044464)	1
OG0034587	Cellular Component	cell(GO:0005623)	1
OG0034587	Cellular Component	membrane(GO:0016020)	1
OG0034588	Cellular Component	cell part(GO:0044464)	1
OG0034588	Cellular Component	cell(GO:0005623)	1
OG0034588	Cellular Component	membrane(GO:0016020)	1
OG0034589	Cellular Component	cell part(GO:0044464)	1
OG0034589	Cellular Component	cell(GO:0005623)	1
OG0034589	Cellular Component	membrane(GO:0016020)	1
OG0034590	Cellular Component	cell part(GO:0044464)	1
OG0034590	Cellular Component	cell(GO:0005623)	1
OG0034590	Cellular Component	membrane(GO:0016020)	1
OG0034591	Cellular Component	cell part(GO:0044464)	1
OG0034591	Cellular Component	cell(GO:0005623)	1
OG0034591	Cellular Component	membrane(GO:0016020)	1
OG0034592	Cellular Component	cell part(GO:0044464)	1
OG0034592	Cellular Component	cell(GO:0005623)	1
OG0034592	Cellular Component	membrane(GO:0016020)	1
OG0034592	Cellular Component	organelle part(GO:0044422)	1
OG0034592	Cellular Component	organelle(GO:0043226)	1
OG0034592	Cellular Component	protein-containing complex(GO:0032991)	1
OG0034595	Cellular Component	cell part(GO:0044464)	1
OG0034595	Cellular Component	cell(GO:0005623)	1
OG0034595	Cellular Component	membrane(GO:0016020)	1
OG0034597	Cellular Component	cell part(GO:0044464)	1
OG0034597	Cellular Component	cell(GO:0005623)	1
OG0034599	Cellular Component	cell part(GO:0044464)	1
OG0034599	Cellular Component	cell(GO:0005623)	1
OG0034599	Cellular Component	membrane part(GO:0044425)	1
OG0034599	Cellular Component	membrane(GO:0016020)	1
OG0034599	Cellular Component	protein-containing complex(GO:0032991)	1
OG0034600	Cellular Component	cell part(GO:0044464)	1
OG0034600	Cellular Component	cell(GO:0005623)	1

OG0034600	Cellular Component	protein-containing complex(GO:0032991)	1
OG0034601	Cellular Component	cell part(GO:0044464)	1
OG0034601	Cellular Component	cell(GO:0005623)	1
OG0034608	Cellular Component	cell part(GO:0044464)	1
OG0034608	Cellular Component	cell(GO:0005623)	1
OG0034608	Cellular Component	membrane(GO:0016020)	1
OG0034609	Cellular Component	cell part(GO:0044464)	1
OG0034609	Cellular Component	cell(GO:0005623)	1
OG0034609	Cellular Component	membrane(GO:0016020)	1
OG0034617	Cellular Component	cell part(GO:0044464)	1
OG0034617	Cellular Component	cell(GO:0005623)	1
OG0034617	Cellular Component	membrane(GO:0016020)	1
OG0034617	Cellular Component	organelle part(GO:0044422)	1
OG0034617	Cellular Component	organelle(GO:0043226)	1
OG0034618	Cellular Component	cell part(GO:0044464)	1
OG0034618	Cellular Component	cell(GO:0005623)	1
OG0034618	Cellular Component	organelle(GO:0043226)	1
OG0034620	Cellular Component	cell part(GO:0044464)	1
OG0034620	Cellular Component	cell(GO:0005623)	1
OG0034620	Cellular Component	membrane(GO:0016020)	1
OG0034620	Cellular Component	organelle(GO:0043226)	1
OG0034623	Cellular Component	cell part(GO:0044464)	1
OG0034623	Cellular Component	cell(GO:0005623)	1
OG0034623	Cellular Component	membrane(GO:0016020)	1
OG0034623	Cellular Component	organelle part(GO:0044422)	1
OG0034623	Cellular Component	organelle(GO:0043226)	1
OG0034625	Cellular Component	cell part(GO:0044464)	1
OG0034625	Cellular Component	cell(GO:0005623)	1
OG0034627	Cellular Component	cell part(GO:0044464)	1
OG0034627	Cellular Component	cell(GO:0005623)	1
OG0034627	Cellular Component	membrane(GO:0016020)	1
OG0034629	Cellular Component	cell part(GO:0044464)	1
OG0034629	Cellular Component	cell(GO:0005623)	1
OG0034629	Cellular Component	membrane-enclosed lumen(GO:0031974)	1
OG0034629	Cellular Component	organelle part(GO:0044422)	1
OG0034629	Cellular Component	organelle(GO:0043226)	1
OG0034629	Cellular Component	protein-containing complex(GO:0032991)	1
OG0034630	Cellular Component	cell part(GO:0044464)	1
OG0034630	Cellular Component	cell(GO:0005623)	1
OG0034630	Cellular Component	membrane(GO:0016020)	1
OG0034630	Cellular Component	organelle part(GO:0044422)	1
OG0034630	Cellular Component	organelle(GO:0043226)	1
OG0034631	Cellular Component	cell part(GO:0044464)	1
OG0034631	Cellular Component	cell(GO:0005623)	1
OG0034631	Cellular Component	membrane part(GO:0044425)	1
OG0034631	Cellular Component	membrane(GO:0016020)	1
OG0034631	Cellular Component	organelle part(GO:0044422)	1

OG0034631	Cellular Component	organelle(GO:0043226)	1
OG0034631	Cellular Component	protein-containing complex(GO:0032991)	1
OG0034632	Cellular Component	cell part(GO:0044464)	1
OG0034632	Cellular Component	cell(GO:0005623)	1
OG0034632	Cellular Component	membrane-enclosed lumen(GO:0031974)	1
OG0034632	Cellular Component	organelle part(GO:0044422)	1
OG0034632	Cellular Component	organelle(GO:0043226)	1
OG0034632	Cellular Component	protein-containing complex(GO:0032991)	1
OG0034641	Cellular Component	cell part(GO:0044464)	1
OG0034641	Cellular Component	cell(GO:0005623)	1
OG0034643	Cellular Component	cell part(GO:0044464)	1
OG0034643	Cellular Component	cell(GO:0005623)	1
OG0034645	Cellular Component	cell part(GO:0044464)	1
OG0034645	Cellular Component	cell(GO:0005623)	1
OG0034645	Cellular Component	membrane(GO:0016020)	1
OG0034646	Cellular Component	membrane part(GO:0044425)	1
OG0034646	Cellular Component	membrane(GO:0016020)	1
OG0034647	Cellular Component	cell part(GO:0044464)	1
OG0034647	Cellular Component	cell(GO:0005623)	1
OG0034647	Cellular Component	membrane(GO:0016020)	1
OG0034648	Cellular Component	cell part(GO:0044464)	1
OG0034648	Cellular Component	cell(GO:0005623)	1
OG0034648	Cellular Component	membrane(GO:0016020)	1
OG0034650	Cellular Component	cell part(GO:0044464)	1
OG0034650	Cellular Component	cell(GO:0005623)	1
OG0034652	Cellular Component	cell part(GO:0044464)	1
OG0034652	Cellular Component	cell(GO:0005623)	1
OG0034652	Cellular Component	extracellular region(GO:0005576)	1
OG0034652	Cellular Component	membrane part(GO:0044425)	1
OG0034652	Cellular Component	membrane(GO:0016020)	1
OG0034654	Cellular Component	cell part(GO:0044464)	1
OG0034654	Cellular Component	cell(GO:0005623)	1
OG0034654	Cellular Component	membrane(GO:0016020)	1
OG0034657	Cellular Component	cell part(GO:0044464)	1
OG0034657	Cellular Component	cell(GO:0005623)	1
OG0034658	Cellular Component	cell part(GO:0044464)	1
OG0034658	Cellular Component	cell(GO:0005623)	1
OG0034659	Cellular Component	cell part(GO:0044464)	1
OG0034659	Cellular Component	cell(GO:0005623)	1
OG0034669	Cellular Component	cell part(GO:0044464)	1
OG0034669	Cellular Component	cell(GO:0005623)	1
OG0034669	Cellular Component	organelle(GO:0043226)	1
OG0034670	Cellular Component	cell part(GO:0044464)	1
OG0034670	Cellular Component	cell(GO:0005623)	1
OG0034670	Cellular Component	organelle(GO:0043226)	1
OG0034672	Cellular Component	cell part(GO:0044464)	1
OG0034672	Cellular Component	cell(GO:0005623)	1

OG0034672	Cellular Component	organelle(GO:0043226)	1
OG0034673	Cellular Component	cell part(GO:0044464)	1
OG0034673	Cellular Component	cell(GO:0005623)	1
OG0034673	Cellular Component	organelle(GO:0043226)	1
OG0034675	Cellular Component	cell part(GO:0044464)	1
OG0034675	Cellular Component	cell(GO:0005623)	1
OG0034675	Cellular Component	organelle(GO:0043226)	1
OG0034676	Cellular Component	cell part(GO:0044464)	1
OG0034676	Cellular Component	cell(GO:0005623)	1
OG0034676	Cellular Component	organelle(GO:0043226)	1
OG0034681	Cellular Component	cell part(GO:0044464)	1
OG0034681	Cellular Component	cell(GO:0005623)	1
OG0034681	Cellular Component	membrane(GO:0016020)	1
OG0034681	Cellular Component	organelle part(GO:0044422)	1
OG0034681	Cellular Component	organelle(GO:0043226)	1
OG0034699	Cellular Component	cell part(GO:0044464)	1
OG0034699	Cellular Component	cell(GO:0005623)	1
OG0034699	Cellular Component	extracellular region(GO:0005576)	1
OG0034699	Cellular Component	membrane(GO:0016020)	1
OG0034699	Cellular Component	organelle part(GO:0044422)	1
OG0034699	Cellular Component	organelle(GO:0043226)	1
OG0034699	Cellular Component	protein-containing complex(GO:0032991)	1
OG0034705	Cellular Component	cell part(GO:0044464)	1
OG0034705	Cellular Component	cell(GO:0005623)	1
OG0034705	Cellular Component	extracellular region part(GO:0044421)	1
OG0034705	Cellular Component	extracellular region(GO:0005576)	1
OG0034705	Cellular Component	membrane part(GO:0044425)	1
OG0034705	Cellular Component	membrane(GO:0016020)	1
OG0034705	Cellular Component	organelle(GO:0043226)	1
OG0034707	Cellular Component	cell part(GO:0044464)	1
OG0034707	Cellular Component	cell(GO:0005623)	1
OG0034707	Cellular Component	extracellular region(GO:0005576)	1
OG0034707	Cellular Component	membrane(GO:0016020)	1
OG0034707	Cellular Component	membrane-enclosed lumen(GO:0031974)	1
OG0034707	Cellular Component	organelle part(GO:0044422)	1
OG0034707	Cellular Component	organelle(GO:0043226)	1
OG0034722	Cellular Component	cell part(GO:0044464)	1
OG0034722	Cellular Component	cell(GO:0005623)	1
OG0034722	Cellular Component	organelle(GO:0043226)	1
OG0034728	Cellular Component	cell part(GO:0044464)	1
OG0034728	Cellular Component	cell(GO:0005623)	1
OG0034728	Cellular Component	membrane(GO:0016020)	1
OG0034729	Cellular Component	cell junction(GO:0030054)	1
OG0034729	Cellular Component	cell part(GO:0044464)	1
OG0034729	Cellular Component	cell(GO:0005623)	1
OG0034729	Cellular Component	membrane(GO:0016020)	1
OG0034729	Cellular Component	membrane-enclosed lumen(GO:0031974)	1

OG0034729	Cellular Component	organelle part(GO:0044422)	1
OG0034729	Cellular Component	organelle(GO:0043226)	1
OG0034729	Cellular Component	protein-containing complex(GO:0032991)	1
OG0034729	Cellular Component	symplast(GO:0055044)	1
OG0034730	Cellular Component	cell part(GO:0044464)	1
OG0034730	Cellular Component	cell(GO:0005623)	1
OG0034730	Cellular Component	membrane(GO:0016020)	1
OG0034732	Cellular Component	cell part(GO:0044464)	1
OG0034732	Cellular Component	cell(GO:0005623)	1
OG0034732	Cellular Component	organelle(GO:0043226)	1
OG0034735	Cellular Component	cell part(GO:0044464)	1
OG0034735	Cellular Component	cell(GO:0005623)	1
OG0034737	Cellular Component	cell part(GO:0044464)	1
OG0034737	Cellular Component	cell(GO:0005623)	1
OG0034737	Cellular Component	membrane(GO:0016020)	1
OG0034737	Cellular Component	organelle part(GO:0044422)	1
OG0034737	Cellular Component	organelle(GO:0043226)	1
OG0034738	Cellular Component	cell part(GO:0044464)	1
OG0034738	Cellular Component	cell(GO:0005623)	1
OG0034738	Cellular Component	organelle(GO:0043226)	1
OG0034740	Cellular Component	cell part(GO:0044464)	1
OG0034740	Cellular Component	cell(GO:0005623)	1
OG0034740	Cellular Component	organelle(GO:0043226)	1
OG0034749	Cellular Component	cell part(GO:0044464)	1
OG0034749	Cellular Component	cell(GO:0005623)	1
OG0034749	Cellular Component	organelle(GO:0043226)	1
OG0034750	Cellular Component	membrane(GO:0016020)	1
OG0034754	Cellular Component	cell part(GO:0044464)	1
OG0034754	Cellular Component	cell(GO:0005623)	1
OG0034754	Cellular Component	organelle(GO:0043226)	1
OG0034755	Cellular Component	cell part(GO:0044464)	1
OG0034755	Cellular Component	cell(GO:0005623)	1
OG0034755	Cellular Component	membrane-enclosed lumen(GO:0031974)	1
OG0034755	Cellular Component	organelle part(GO:0044422)	1
OG0034755	Cellular Component	organelle(GO:0043226)	1
OG0034755	Cellular Component	protein-containing complex(GO:0032991)	1
OG0034767	Cellular Component	cell part(GO:0044464)	1
OG0034767	Cellular Component	cell(GO:0005623)	1
OG0034767	Cellular Component	membrane(GO:0016020)	1
OG0034769	Cellular Component	cell part(GO:0044464)	1
OG0034769	Cellular Component	cell(GO:0005623)	1
OG0034770	Cellular Component	membrane(GO:0016020)	1
OG0034775	Cellular Component	cell part(GO:0044464)	1
OG0034775	Cellular Component	cell(GO:0005623)	1
OG0034775	Cellular Component	organelle(GO:0043226)	1
OG0034776	Cellular Component	cell part(GO:0044464)	1
OG0034776	Cellular Component	cell(GO:0005623)	1

OG0034776	Cellular Component	organelle(GO:0043226)	1
OG0034777	Cellular Component	cell part(GO:0044464)	1
OG0034777	Cellular Component	cell(GO:0005623)	1
OG0034777	Cellular Component	membrane(GO:0016020)	1
OG0034777	Cellular Component	organelle part(GO:0044422)	1
OG0034777	Cellular Component	organelle(GO:0043226)	1
OG0034780	Cellular Component	cell junction(GO:0030054)	1
OG0034780	Cellular Component	cell part(GO:0044464)	1
OG0034780	Cellular Component	cell(GO:0005623)	1
OG0034780	Cellular Component	symplast(GO:0055044)	1
OG0034790	Cellular Component	cell part(GO:0044464)	1
OG0034790	Cellular Component	cell(GO:0005623)	1
OG0034790	Cellular Component	membrane(GO:0016020)	1
OG0034791	Cellular Component	cell junction(GO:0030054)	1
OG0034791	Cellular Component	cell part(GO:0044464)	1
OG0034791	Cellular Component	cell(GO:0005623)	1
OG0034791	Cellular Component	membrane(GO:0016020)	1
OG0034791	Cellular Component	organelle part(GO:0044422)	1
OG0034791	Cellular Component	organelle(GO:0043226)	1
OG0034791	Cellular Component	symplast(GO:0055044)	1
OG0034792	Cellular Component	cell part(GO:0044464)	1
OG0034792	Cellular Component	cell(GO:0005623)	1
OG0034792	Cellular Component	membrane-enclosed lumen(GO:0031974)	1
OG0034792	Cellular Component	organelle part(GO:0044422)	1
OG0034792	Cellular Component	organelle(GO:0043226)	1
OG0034802	Cellular Component	cell part(GO:0044464)	1
OG0034802	Cellular Component	cell(GO:0005623)	1
OG0034802	Cellular Component	organelle(GO:0043226)	1
OG0034803	Cellular Component	cell junction(GO:0030054)	1
OG0034803	Cellular Component	cell part(GO:0044464)	1
OG0034803	Cellular Component	cell(GO:0005623)	1
OG0034803	Cellular Component	membrane part(GO:0044425)	1
OG0034803	Cellular Component	membrane(GO:0016020)	1
OG0034803	Cellular Component	symplast(GO:0055044)	1
OG0034805	Cellular Component	cell part(GO:0044464)	1
OG0034805	Cellular Component	cell(GO:0005623)	1
OG0034805	Cellular Component	organelle(GO:0043226)	1
OG0034811	Cellular Component	cell part(GO:0044464)	1
OG0034811	Cellular Component	cell(GO:0005623)	1
OG0034811	Cellular Component	membrane(GO:0016020)	1
OG0034813	Cellular Component	cell part(GO:0044464)	1
OG0034813	Cellular Component	cell(GO:0005623)	1
OG0034813	Cellular Component	membrane(GO:0016020)	1
OG0034816	Cellular Component	cell part(GO:0044464)	1
OG0034816	Cellular Component	cell(GO:0005623)	1
OG0034816	Cellular Component	membrane(GO:0016020)	1
OG0034825	Cellular Component	cell part(GO:0044464)	1

OG0034825	Cellular Component	cell(GO:0005623)	1
OG0034825	Cellular Component	organelle(GO:0043226)	1
OG0034827	Cellular Component	cell part(GO:0044464)	1
OG0034827	Cellular Component	cell(GO:0005623)	1
OG0034827	Cellular Component	membrane(GO:0016020)	1
OG0034827	Cellular Component	organelle part(GO:0044422)	1
OG0034827	Cellular Component	organelle(GO:0043226)	1
OG0034829	Cellular Component	cell part(GO:0044464)	1
OG0034829	Cellular Component	cell(GO:0005623)	1
OG0034829	Cellular Component	membrane(GO:0016020)	1
OG0034829	Cellular Component	organelle part(GO:0044422)	1
OG0034829	Cellular Component	organelle(GO:0043226)	1
OG0034842	Cellular Component	cell part(GO:0044464)	1
OG0034842	Cellular Component	cell(GO:0005623)	1
OG0034842	Cellular Component	organelle(GO:0043226)	1
OG0034852	Cellular Component	cell part(GO:0044464)	1
OG0034852	Cellular Component	cell(GO:0005623)	1
OG0034852	Cellular Component	organelle(GO:0043226)	1
OG0034853	Cellular Component	cell junction(GO:0030054)	1
OG0034853	Cellular Component	symplast(GO:0055044)	1
OG0034854	Cellular Component	cell part(GO:0044464)	1
OG0034854	Cellular Component	cell(GO:0005623)	1
OG0034854	Cellular Component	organelle part(GO:0044422)	1
OG0034854	Cellular Component	organelle(GO:0043226)	1
OG0034856	Cellular Component	cell part(GO:0044464)	1
OG0034856	Cellular Component	cell(GO:0005623)	1
OG0034856	Cellular Component	membrane-enclosed lumen(GO:0031974)	1
OG0034856	Cellular Component	organelle part(GO:0044422)	1
OG0034856	Cellular Component	organelle(GO:0043226)	1
OG0034873	Cellular Component	cell part(GO:0044464)	1
OG0034873	Cellular Component	cell(GO:0005623)	1
OG0034873	Cellular Component	membrane part(GO:0044425)	1
OG0034873	Cellular Component	membrane(GO:0016020)	1
OG0034877	Cellular Component	cell part(GO:0044464)	1
OG0034877	Cellular Component	cell(GO:0005623)	1
OG0034877	Cellular Component	membrane(GO:0016020)	1
OG0034877	Cellular Component	organelle part(GO:0044422)	1
OG0034877	Cellular Component	organelle(GO:0043226)	1
OG0034878	Cellular Component	membrane(GO:0016020)	1
OG0034888	Cellular Component	cell part(GO:0044464)	1
OG0034888	Cellular Component	cell(GO:0005623)	1
OG0034888	Cellular Component	organelle(GO:0043226)	1
OG0034895	Cellular Component	cell part(GO:0044464)	1
OG0034895	Cellular Component	cell(GO:0005623)	1
OG0034895	Cellular Component	organelle(GO:0043226)	1
OG0034918	Cellular Component	cell part(GO:0044464)	1
OG0034918	Cellular Component	cell(GO:0005623)	1

OG0034918	Cellular Component	membrane part(GO:0044425)	1
OG0034918	Cellular Component	membrane(GO:0016020)	1
OG0034918	Cellular Component	organelle part(GO:0044422)	1
OG0034918	Cellular Component	organelle(GO:0043226)	1
OG0034918	Cellular Component	protein-containing complex(GO:0032991)	1
OG0034921	Cellular Component	cell part(GO:0044464)	1
OG0034921	Cellular Component	cell(GO:0005623)	1
OG0034921	Cellular Component	organelle(GO:0043226)	1
OG0034928	Cellular Component	cell part(GO:0044464)	1
OG0034928	Cellular Component	cell(GO:0005623)	1
OG0034928	Cellular Component	membrane(GO:0016020)	1
OG0034928	Cellular Component	organelle part(GO:0044422)	1
OG0034928	Cellular Component	organelle(GO:0043226)	1
OG0034930	Cellular Component	cell part(GO:0044464)	1
OG0034930	Cellular Component	cell(GO:0005623)	1
OG0034930	Cellular Component	organelle(GO:0043226)	1
OG0034931	Cellular Component	cell part(GO:0044464)	1
OG0034931	Cellular Component	cell(GO:0005623)	1
OG0034931	Cellular Component	organelle(GO:0043226)	1
OG0034934	Cellular Component	cell junction(GO:0030054)	1
OG0034934	Cellular Component	cell part(GO:0044464)	1
OG0034934	Cellular Component	cell(GO:0005623)	1
OG0034934	Cellular Component	membrane(GO:0016020)	1
OG0034934	Cellular Component	organelle part(GO:0044422)	1
OG0034934	Cellular Component	organelle(GO:0043226)	1
OG0034934	Cellular Component	symplast(GO:0055044)	1
OG0034935	Cellular Component	cell part(GO:0044464)	1
OG0034935	Cellular Component	cell(GO:0005623)	1
OG0034935	Cellular Component	organelle(GO:0043226)	1
OG0034943	Cellular Component	cell part(GO:0044464)	1
OG0034943	Cellular Component	cell(GO:0005623)	1
OG0034944	Cellular Component	cell junction(GO:0030054)	1
OG0034944	Cellular Component	cell part(GO:0044464)	1
OG0034944	Cellular Component	cell(GO:0005623)	1
OG0034944	Cellular Component	membrane(GO:0016020)	1
OG0034944	Cellular Component	membrane-enclosed lumen(GO:0031974)	1
OG0034944	Cellular Component	organelle part(GO:0044422)	1
OG0034944	Cellular Component	organelle(GO:0043226)	1
OG0034944	Cellular Component	protein-containing complex(GO:0032991)	1
OG0034944	Cellular Component	symplast(GO:0055044)	1
OG0034945	Cellular Component	cell part(GO:0044464)	1
OG0034945	Cellular Component	cell(GO:0005623)	1
OG0034948	Cellular Component	cell part(GO:0044464)	1
OG0034948	Cellular Component	cell(GO:0005623)	1
OG0034948	Cellular Component	membrane(GO:0016020)	1
OG0034948	Cellular Component	organelle part(GO:0044422)	1
OG0034948	Cellular Component	organelle(GO:0043226)	1

OG0034956	Cellular Component	cell part(GO:0044464)	1
OG0034956	Cellular Component	cell(GO:0005623)	1
OG0034956	Cellular Component	membrane(GO:0016020)	1
OG0034956	Cellular Component	organelle part(GO:0044422)	1
OG0034956	Cellular Component	organelle(GO:0043226)	1
OG0034957	Cellular Component	cell part(GO:0044464)	1
OG0034957	Cellular Component	cell(GO:0005623)	1
OG0034957	Cellular Component	membrane(GO:0016020)	1
OG0034957	Cellular Component	organelle part(GO:0044422)	1
OG0034957	Cellular Component	organelle(GO:0043226)	1
OG0034963	Cellular Component	cell part(GO:0044464)	1
OG0034963	Cellular Component	cell(GO:0005623)	1
OG0034963	Cellular Component	organelle part(GO:0044422)	1
OG0034963	Cellular Component	organelle(GO:0043226)	1
OG0034964	Cellular Component	cell part(GO:0044464)	1
OG0034964	Cellular Component	cell(GO:0005623)	1
OG0034964	Cellular Component	organelle part(GO:0044422)	1
OG0034964	Cellular Component	organelle(GO:0043226)	1
OG0034965	Cellular Component	cell part(GO:0044464)	1
OG0034965	Cellular Component	cell(GO:0005623)	1
OG0034965	Cellular Component	organelle part(GO:0044422)	1
OG0034965	Cellular Component	organelle(GO:0043226)	1
OG0034969	Cellular Component	cell part(GO:0044464)	1
OG0034969	Cellular Component	cell(GO:0005623)	1
OG0034969	Cellular Component	organelle part(GO:0044422)	1
OG0034969	Cellular Component	organelle(GO:0043226)	1
OG0034970	Cellular Component	cell part(GO:0044464)	1
OG0034970	Cellular Component	cell(GO:0005623)	1
OG0034970	Cellular Component	organelle(GO:0043226)	1
OG0034978	Cellular Component	cell part(GO:0044464)	1
OG0034978	Cellular Component	cell(GO:0005623)	1
OG0034978	Cellular Component	organelle part(GO:0044422)	1
OG0034978	Cellular Component	organelle(GO:0043226)	1
OG0034979	Cellular Component	cell junction(GO:0030054)	1
OG0034979	Cellular Component	cell part(GO:0044464)	1
OG0034979	Cellular Component	cell(GO:0005623)	1
OG0034979	Cellular Component	extracellular region(GO:0005576)	1
OG0034979	Cellular Component	membrane(GO:0016020)	1
OG0034979	Cellular Component	organelle(GO:0043226)	1
OG0034979	Cellular Component	symplast(GO:0055044)	1
OG0034980	Cellular Component	cell part(GO:0044464)	1
OG0034980	Cellular Component	cell(GO:0005623)	1
OG0034980	Cellular Component	membrane(GO:0016020)	1
OG0034980	Cellular Component	organelle(GO:0043226)	1
OG0034981	Cellular Component	cell part(GO:0044464)	1
OG0034981	Cellular Component	cell(GO:0005623)	1
OG0034981	Cellular Component	organelle part(GO:0044422)	1

OG0034981	Cellular Component	organelle(GO:0043226)	1
OG0034982	Cellular Component	cell part(GO:0044464)	1
OG0034982	Cellular Component	cell(GO:0005623)	1
OG0034982	Cellular Component	organelle(GO:0043226)	1
OG0034989	Cellular Component	extracellular region part(GO:0044421)	1
OG0034989	Cellular Component	extracellular region(GO:0005576)	1
OG0034998	Cellular Component	cell junction(GO:0030054)	1
OG0034998	Cellular Component	cell part(GO:0044464)	1
OG0034998	Cellular Component	cell(GO:0005623)	1
OG0034998	Cellular Component	extracellular region part(GO:0044421)	1
OG0034998	Cellular Component	extracellular region(GO:0005576)	1
OG0034998	Cellular Component	symplast(GO:0055044)	1
OG0035003	Cellular Component	cell part(GO:0044464)	1
OG0035003	Cellular Component	cell(GO:0005623)	1
OG0035003	Cellular Component	membrane(GO:0016020)	1
OG0035003	Cellular Component	organelle part(GO:0044422)	1
OG0035003	Cellular Component	organelle(GO:0043226)	1
OG0035004	Cellular Component	cell part(GO:0044464)	1
OG0035004	Cellular Component	cell(GO:0005623)	1
OG0035004	Cellular Component	membrane(GO:0016020)	1
OG0035004	Cellular Component	organelle part(GO:0044422)	1
OG0035004	Cellular Component	organelle(GO:0043226)	1
OG0035020	Cellular Component	cell part(GO:0044464)	1
OG0035020	Cellular Component	cell(GO:0005623)	1
OG0035020	Cellular Component	organelle(GO:0043226)	1
OG0035022	Cellular Component	cell part(GO:0044464)	1
OG0035022	Cellular Component	cell(GO:0005623)	1
OG0035022	Cellular Component	extracellular region(GO:0005576)	1
OG0035022	Cellular Component	membrane(GO:0016020)	1
OG0035022	Cellular Component	organelle part(GO:0044422)	1
OG0035022	Cellular Component	organelle(GO:0043226)	1
OG0035025	Cellular Component	cell part(GO:0044464)	1
OG0035025	Cellular Component	cell(GO:0005623)	1
OG0035028	Cellular Component	cell part(GO:0044464)	1
OG0035028	Cellular Component	cell(GO:0005623)	1
OG0035028	Cellular Component	membrane-enclosed lumen(GO:0031974)	1
OG0035028	Cellular Component	nucleoid(GO:0009295)	1
OG0035028	Cellular Component	organelle part(GO:0044422)	1
OG0035028	Cellular Component	organelle(GO:0043226)	1
OG0035031	Cellular Component	cell part(GO:0044464)	1
OG0035031	Cellular Component	cell(GO:0005623)	1
OG0035032	Cellular Component	cell part(GO:0044464)	1
OG0035032	Cellular Component	cell(GO:0005623)	1
OG0035032	Cellular Component	organelle(GO:0043226)	1
OG0035033	Cellular Component	cell part(GO:0044464)	1
OG0035033	Cellular Component	cell(GO:0005623)	1
OG0035033	Cellular Component	membrane(GO:0016020)	1

OG0035033	Cellular Component	organelle part(GO:0044422)	1
OG0035033	Cellular Component	organelle(GO:0043226)	1
OG0035037	Cellular Component	cell part(GO:0044464)	1
OG0035037	Cellular Component	cell(GO:0005623)	1
OG0035037	Cellular Component	organelle part(GO:0044422)	1
OG0035037	Cellular Component	organelle(GO:0043226)	1
OG0035038	Cellular Component	cell part(GO:0044464)	1
OG0035038	Cellular Component	cell(GO:0005623)	1
OG0035038	Cellular Component	membrane(GO:0016020)	1
OG0035038	Cellular Component	organelle part(GO:0044422)	1
OG0035038	Cellular Component	organelle(GO:0043226)	1
OG0035039	Cellular Component	cell part(GO:0044464)	1
OG0035039	Cellular Component	cell(GO:0005623)	1
OG0035039	Cellular Component	organelle part(GO:0044422)	1
OG0035039	Cellular Component	organelle(GO:0043226)	1
OG0035047	Cellular Component	cell part(GO:0044464)	1
OG0035047	Cellular Component	cell(GO:0005623)	1
OG0035047	Cellular Component	membrane(GO:0016020)	1
OG0035063	Cellular Component	cell junction(GO:0030054)	1
OG0035063	Cellular Component	cell part(GO:0044464)	1
OG0035063	Cellular Component	cell(GO:0005623)	1
OG0035063	Cellular Component	membrane part(GO:0044425)	1
OG0035063	Cellular Component	membrane(GO:0016020)	1
OG0035063	Cellular Component	organelle part(GO:0044422)	1
OG0035063	Cellular Component	organelle(GO:0043226)	1
OG0035063	Cellular Component	symplast(GO:0055044)	1
OG0035077	Cellular Component	cell part(GO:0044464)	1
OG0035077	Cellular Component	cell(GO:0005623)	1
OG0035077	Cellular Component	organelle(GO:0043226)	1
OG0035079	Cellular Component	cell junction(GO:0030054)	1
OG0035079	Cellular Component	cell part(GO:0044464)	1
OG0035079	Cellular Component	cell(GO:0005623)	1
OG0035079	Cellular Component	membrane(GO:0016020)	1
OG0035079	Cellular Component	membrane-enclosed lumen(GO:0031974)	1
OG0035079	Cellular Component	organelle part(GO:0044422)	1
OG0035079	Cellular Component	organelle(GO:0043226)	1
OG0035079	Cellular Component	protein-containing complex(GO:0032991)	1
OG0035079	Cellular Component	symplast(GO:0055044)	1
OG0035084	Cellular Component	cell part(GO:0044464)	1
OG0035084	Cellular Component	cell(GO:0005623)	1
OG0035084	Cellular Component	membrane(GO:0016020)	1
OG0035084	Cellular Component	organelle part(GO:0044422)	1
OG0035084	Cellular Component	organelle(GO:0043226)	1
OG0035091	Cellular Component	cell part(GO:0044464)	1
OG0035091	Cellular Component	cell(GO:0005623)	1
OG0035091	Cellular Component	membrane part(GO:0044425)	1
OG0035091	Cellular Component	membrane(GO:0016020)	1

OG0035091	Cellular Component	organelle part(GO:0044422)	1
OG0035091	Cellular Component	organelle(GO:0043226)	1
OG0035091	Cellular Component	protein-containing complex(GO:0032991)	1
OG0035094	Cellular Component	cell junction(GO:0030054)	1
OG0035094	Cellular Component	cell part(GO:0044464)	1
OG0035094	Cellular Component	cell(GO:0005623)	1
OG0035094	Cellular Component	membrane part(GO:0044425)	1
OG0035094	Cellular Component	membrane(GO:0016020)	1
OG0035094	Cellular Component	organelle(GO:0043226)	1
OG0035094	Cellular Component	symplast(GO:0055044)	1
OG0035095	Cellular Component	cell part(GO:0044464)	1
OG0035095	Cellular Component	cell(GO:0005623)	1
OG0035095	Cellular Component	organelle(GO:0043226)	1
OG0035096	Cellular Component	membrane(GO:0016020)	1
OG0035097	Cellular Component	cell part(GO:0044464)	1
OG0035097	Cellular Component	cell(GO:0005623)	1
OG0035097	Cellular Component	membrane(GO:0016020)	1
OG0035113	Cellular Component	cell part(GO:0044464)	1
OG0035113	Cellular Component	cell(GO:0005623)	1
OG0035113	Cellular Component	organelle(GO:0043226)	1
OG0035118	Cellular Component	cell part(GO:0044464)	1
OG0035118	Cellular Component	cell(GO:0005623)	1
OG0035118	Cellular Component	organelle(GO:0043226)	1
OG0035119	Cellular Component	cell part(GO:0044464)	1
OG0035119	Cellular Component	cell(GO:0005623)	1
OG0035119	Cellular Component	organelle(GO:0043226)	1
OG0035122	Cellular Component	cell part(GO:0044464)	1
OG0035122	Cellular Component	cell(GO:0005623)	1
OG0035122	Cellular Component	membrane(GO:0016020)	1
OG0035122	Cellular Component	organelle part(GO:0044422)	1
OG0035122	Cellular Component	organelle(GO:0043226)	1
OG0035150	Cellular Component	cell junction(GO:0030054)	1
OG0035150	Cellular Component	cell part(GO:0044464)	1
OG0035150	Cellular Component	cell(GO:0005623)	1
OG0035150	Cellular Component	organelle(GO:0043226)	1
OG0035150	Cellular Component	symplast(GO:0055044)	1
OG0035156	Cellular Component	cell part(GO:0044464)	1
OG0035156	Cellular Component	cell(GO:0005623)	1
OG0035156	Cellular Component	membrane(GO:0016020)	1
OG0035160	Cellular Component	cell part(GO:0044464)	1
OG0035160	Cellular Component	cell(GO:0005623)	1
OG0035163	Cellular Component	cell part(GO:0044464)	1
OG0035163	Cellular Component	cell(GO:0005623)	1
OG0035163	Cellular Component	organelle part(GO:0044422)	1
OG0035163	Cellular Component	organelle(GO:0043226)	1
OG0035165	Cellular Component	cell part(GO:0044464)	1
OG0035165	Cellular Component	cell(GO:0005623)	1

OG0035165	Cellular Component	organelle(GO:0043226)	1
OG0035167	Cellular Component	cell part(GO:0044464)	1
OG0035167	Cellular Component	cell(GO:0005623)	1
OG0035167	Cellular Component	organelle part(GO:0044422)	1
OG0035167	Cellular Component	organelle(GO:0043226)	1
OG0035167	Cellular Component	protein-containing complex(GO:0032991)	1
OG0035168	Cellular Component	cell part(GO:0044464)	1
OG0035168	Cellular Component	cell(GO:0005623)	1
OG0035168	Cellular Component	extracellular region(GO:0005576)	1
OG0035168	Cellular Component	membrane part(GO:0044425)	1
OG0035168	Cellular Component	membrane(GO:0016020)	1
OG0035168	Cellular Component	protein-containing complex(GO:0032991)	1
OG0035169	Cellular Component	cell part(GO:0044464)	1
OG0035169	Cellular Component	cell(GO:0005623)	1
OG0035169	Cellular Component	membrane(GO:0016020)	1
OG0035169	Cellular Component	organelle part(GO:0044422)	1
OG0035169	Cellular Component	organelle(GO:0043226)	1
OG0035175	Cellular Component	cell part(GO:0044464)	1
OG0035175	Cellular Component	cell(GO:0005623)	1
OG0035175	Cellular Component	organelle(GO:0043226)	1
OG0035176	Cellular Component	cell part(GO:0044464)	1
OG0035176	Cellular Component	cell(GO:0005623)	1
OG0035176	Cellular Component	organelle(GO:0043226)	1
OG0035177	Cellular Component	cell part(GO:0044464)	1
OG0035177	Cellular Component	cell(GO:0005623)	1
OG0035177	Cellular Component	organelle(GO:0043226)	1
OG0035181	Cellular Component	cell part(GO:0044464)	1
OG0035181	Cellular Component	cell(GO:0005623)	1
OG0035181	Cellular Component	organelle(GO:0043226)	1
OG0035186	Cellular Component	cell part(GO:0044464)	1
OG0035186	Cellular Component	cell(GO:0005623)	1
OG0035186	Cellular Component	organelle(GO:0043226)	1
OG0035191	Cellular Component	cell part(GO:0044464)	1
OG0035191	Cellular Component	cell(GO:0005623)	1
OG0035193	Cellular Component	cell part(GO:0044464)	1
OG0035193	Cellular Component	cell(GO:0005623)	1
OG0035193	Cellular Component	membrane(GO:0016020)	1
OG0035194	Cellular Component	cell part(GO:0044464)	1
OG0035194	Cellular Component	cell(GO:0005623)	1
OG0035194	Cellular Component	membrane(GO:0016020)	1
OG0035200	Cellular Component	cell part(GO:0044464)	1
OG0035200	Cellular Component	cell(GO:0005623)	1
OG0035200	Cellular Component	membrane(GO:0016020)	1
OG0035202	Cellular Component	cell part(GO:0044464)	1
OG0035202	Cellular Component	cell(GO:0005623)	1
OG0035205	Cellular Component	cell part(GO:0044464)	1
OG0035205	Cellular Component	cell(GO:0005623)	1

OG0035205	Cellular Component	membrane(GO:0016020)	1
OG0035206	Cellular Component	cell part(GO:0044464)	1
OG0035206	Cellular Component	cell(GO:0005623)	1
OG0035206	Cellular Component	extracellular region(GO:0005576)	1
OG0035206	Cellular Component	organelle part(GO:0044422)	1
OG0035206	Cellular Component	organelle(GO:0043226)	1
OG0035207	Cellular Component	cell part(GO:0044464)	1
OG0035207	Cellular Component	cell(GO:0005623)	1
OG0035207	Cellular Component	organelle(GO:0043226)	1
OG0035208	Cellular Component	cell part(GO:0044464)	1
OG0035208	Cellular Component	cell(GO:0005623)	1
OG0035208	Cellular Component	extracellular region(GO:0005576)	1
OG0035208	Cellular Component	organelle part(GO:0044422)	1
OG0035208	Cellular Component	organelle(GO:0043226)	1
OG0035210	Cellular Component	cell part(GO:0044464)	1
OG0035210	Cellular Component	cell(GO:0005623)	1
OG0035210	Cellular Component	organelle(GO:0043226)	1
OG0035212	Cellular Component	cell part(GO:0044464)	1
OG0035212	Cellular Component	cell(GO:0005623)	1
OG0035212	Cellular Component	membrane(GO:0016020)	1
OG0035212	Cellular Component	organelle part(GO:0044422)	1
OG0035212	Cellular Component	organelle(GO:0043226)	1
OG0035214	Cellular Component	cell part(GO:0044464)	1
OG0035214	Cellular Component	cell(GO:0005623)	1
OG0035214	Cellular Component	organelle(GO:0043226)	1
OG0035216	Cellular Component	cell part(GO:0044464)	1
OG0035216	Cellular Component	cell(GO:0005623)	1
OG0035216	Cellular Component	extracellular region(GO:0005576)	1
OG0035216	Cellular Component	organelle part(GO:0044422)	1
OG0035216	Cellular Component	organelle(GO:0043226)	1
OG0035217	Cellular Component	cell part(GO:0044464)	1
OG0035217	Cellular Component	cell(GO:0005623)	1
OG0035217	Cellular Component	membrane(GO:0016020)	1
OG0035218	Cellular Component	cell part(GO:0044464)	1
OG0035218	Cellular Component	cell(GO:0005623)	1
OG0035218	Cellular Component	membrane(GO:0016020)	1
OG0035220	Cellular Component	cell part(GO:0044464)	1
OG0035220	Cellular Component	cell(GO:0005623)	1
OG0035220	Cellular Component	membrane(GO:0016020)	1
OG0035220	Cellular Component	organelle part(GO:0044422)	1
OG0035220	Cellular Component	organelle(GO:0043226)	1
OG0035225	Cellular Component	cell junction(GO:0030054)	1
OG0035225	Cellular Component	cell part(GO:0044464)	1
OG0035225	Cellular Component	cell(GO:0005623)	1
OG0035225	Cellular Component	membrane-enclosed lumen(GO:0031974)	1
OG0035225	Cellular Component	organelle part(GO:0044422)	1
OG0035225	Cellular Component	organelle(GO:0043226)	1

OG0035225	Cellular Component	protein-containing complex(GO:0032991)	1
OG0035225	Cellular Component	symplast(GO:0055044)	1
OG0035226	Cellular Component	cell junction(GO:0030054)	1
OG0035226	Cellular Component	cell part(GO:0044464)	1
OG0035226	Cellular Component	cell(GO:0005623)	1
OG0035226	Cellular Component	membrane(GO:0016020)	1
OG0035226	Cellular Component	membrane-enclosed lumen(GO:0031974)	1
OG0035226	Cellular Component	organelle part(GO:0044422)	1
OG0035226	Cellular Component	organelle(GO:0043226)	1
OG0035226	Cellular Component	protein-containing complex(GO:0032991)	1
OG0035226	Cellular Component	symplast(GO:0055044)	1
OG0035227	Cellular Component	cell part(GO:0044464)	1
OG0035227	Cellular Component	cell(GO:0005623)	1
OG0035227	Cellular Component	organelle(GO:0043226)	1
OG0035228	Cellular Component	cell part(GO:0044464)	1
OG0035228	Cellular Component	cell(GO:0005623)	1
OG0035228	Cellular Component	organelle(GO:0043226)	1
OG0035232	Cellular Component	cell part(GO:0044464)	1
OG0035232	Cellular Component	cell(GO:0005623)	1
OG0035232	Cellular Component	membrane part(GO:0044425)	1
OG0035232	Cellular Component	membrane(GO:0016020)	1
OG0035232	Cellular Component	organelle part(GO:0044422)	1
OG0035232	Cellular Component	organelle(GO:0043226)	1
OG0035232	Cellular Component	protein-containing complex(GO:0032991)	1
OG0035233	Cellular Component	cell junction(GO:0030054)	1
OG0035233	Cellular Component	membrane(GO:0016020)	1
OG0035233	Cellular Component	symplast(GO:0055044)	1
OG0035247	Cellular Component	cell part(GO:0044464)	1
OG0035247	Cellular Component	cell(GO:0005623)	1
OG0035247	Cellular Component	organelle(GO:0043226)	1
OG0035251	Cellular Component	cell part(GO:0044464)	1
OG0035251	Cellular Component	cell(GO:0005623)	1
OG0035251	Cellular Component	organelle(GO:0043226)	1
OG0035261	Cellular Component	cell part(GO:0044464)	1
OG0035261	Cellular Component	cell(GO:0005623)	1
OG0035261	Cellular Component	organelle(GO:0043226)	1
OG0005149	Molecular Function	binding(GO:0005488)	1
OG0005149	Molecular Function	molecular transducer activity(GO:0060089)	1
OG0011836	Molecular Function	binding(GO:0005488)	5
OG0011855	Molecular Function	catalytic activity(GO:0003824)	1
OG0012548	Molecular Function	transcription regulator activity(GO:0140110)	1
OG0013086	Molecular Function	transcription regulator activity(GO:0140110)	3
OG0013092	Molecular Function	transcription regulator activity(GO:0140110)	3
OG0013101	Molecular Function	binding(GO:0005488)	2
OG0013101	Molecular Function	catalytic activity(GO:0003824)	2

OG0013101	Molecular Function	molecular transducer activity(GO:0060089)	2
OG0013110	Molecular Function	catalytic activity(GO:0003824)	2
OG0013567	Molecular Function	transcription regulator activity(GO:0140110)	2
OG0013576	Molecular Function	transcription regulator activity(GO:0140110)	1
OG0013579	Molecular Function	binding(GO:0005488)	2
OG0013579	Molecular Function	transcription regulator activity(GO:0140110)	2
OG0013600	Molecular Function	transcription regulator activity(GO:0140110)	1
OG0013612	Molecular Function	binding(GO:0005488)	2
OG0013612	Molecular Function	transcription regulator activity(GO:0140110)	2
OG0013622	Molecular Function	transcription regulator activity(GO:0140110)	2
OG0013632	Molecular Function	transcription regulator activity(GO:0140110)	2
OG0013633	Molecular Function	binding(GO:0005488)	2
OG0013636	Molecular Function	binding(GO:0005488)	1
OG0013636	Molecular Function	catalytic activity(GO:0003824)	1
OG0013638	Molecular Function	binding(GO:0005488)	1
OG0013638	Molecular Function	catalytic activity(GO:0003824)	1
OG0027162	Molecular Function	binding(GO:0005488)	1
OG0027162	Molecular Function	catalytic activity(GO:0003824)	1
OG0027163	Molecular Function	catalytic activity(GO:0003824)	1
OG0027176	Molecular Function	binding(GO:0005488)	1
OG0027176	Molecular Function	catalytic activity(GO:0003824)	1
OG0027184	Molecular Function	catalytic activity(GO:0003824)	1
OG0027184	Molecular Function	molecular transducer activity(GO:0060089)	1
OG0027185	Molecular Function	transcription regulator activity(GO:0140110)	1
OG0027199	Molecular Function	transporter activity(GO:0005215)	1
OG0027200	Molecular Function	transporter activity(GO:0005215)	1
OG0027201	Molecular Function	catalytic activity(GO:0003824)	1
OG0027210	Molecular Function	catalytic activity(GO:0003824)	1
OG0027214	Molecular Function	transporter activity(GO:0005215)	1
OG0027218	Molecular Function	binding(GO:0005488)	1
OG0027218	Molecular Function	structural molecule activity(GO:0005198)	1
OG0027225	Molecular Function	catalytic activity(GO:0003824)	1
OG0027226	Molecular Function	binding(GO:0005488)	1
OG0027226	Molecular Function	catalytic activity(GO:0003824)	1
OG0027227	Molecular Function	catalytic activity(GO:0003824)	1
OG0027237	Molecular Function	binding(GO:0005488)	1
OG0027237	Molecular Function	catalytic activity(GO:0003824)	1
OG0027238	Molecular Function	catalytic activity(GO:0003824)	1
OG0027240	Molecular Function	binding(GO:0005488)	1
OG0027240	Molecular Function	catalytic activity(GO:0003824)	1
OG0027242	Molecular Function	binding(GO:0005488)	1
OG0027242	Molecular Function	catalytic activity(GO:0003824)	1
OG0027246	Molecular Function	binding(GO:0005488)	1
OG0027246	Molecular Function	catalytic activity(GO:0003824)	1
OG0027251	Molecular Function	binding(GO:0005488)	1

OG0027260	Molecular Function	catalytic activity(GO:0003824)	1
OG0027262	Molecular Function	catalytic activity(GO:0003824)	1
OG0027303	Molecular Function	catalytic activity(GO:0003824)	1
OG0027312	Molecular Function	transcription regulator activity(GO:0140110)	1
OG0027316	Molecular Function	transporter activity(GO:0005215)	1
OG0027318	Molecular Function	binding(GO:0005488)	1
OG0027322	Molecular Function	binding(GO:0005488)	1
OG0027322	Molecular Function	catalytic activity(GO:0003824)	1
OG0027323	Molecular Function	binding(GO:0005488)	1
OG0027323	Molecular Function	catalytic activity(GO:0003824)	1
OG0027328	Molecular Function	binding(GO:0005488)	1
OG0027328	Molecular Function	structural molecule activity(GO:0005198)	1
OG0027329	Molecular Function	catalytic activity(GO:0003824)	1
OG0027330	Molecular Function	binding(GO:0005488)	1
OG0027330	Molecular Function	structural molecule activity(GO:0005198)	1
OG0027337	Molecular Function	binding(GO:0005488)	1
OG0027340	Molecular Function	catalytic activity(GO:0003824)	1
OG0027348	Molecular Function	catalytic activity(GO:0003824)	1
OG0027352	Molecular Function	transcription regulator activity(GO:0140110)	1
OG0027376	Molecular Function	transporter activity(GO:0005215)	1
OG0027379	Molecular Function	binding(GO:0005488)	1
OG0027379	Molecular Function	transcription regulator activity(GO:0140110)	1
OG0027380	Molecular Function	binding(GO:0005488)	1
OG0027380	Molecular Function	molecular function regulator(GO:0098772)	1
OG0027389	Molecular Function	binding(GO:0005488)	1
OG0027396	Molecular Function	transcription regulator activity(GO:0140110)	1
OG0027400	Molecular Function	binding(GO:0005488)	1
OG0027400	Molecular Function	catalytic activity(GO:0003824)	1
OG0027402	Molecular Function	binding(GO:0005488)	1
OG0027402	Molecular Function	catalytic activity(GO:0003824)	1
OG0027410	Molecular Function	binding(GO:0005488)	1
OG0027411	Molecular Function	binding(GO:0005488)	1
OG0027411	Molecular Function	catalytic activity(GO:0003824)	1
OG0027417	Molecular Function	catalytic activity(GO:0003824)	1
OG0027418	Molecular Function	binding(GO:0005488)	1
OG0027418	Molecular Function	catalytic activity(GO:0003824)	1
OG0027419	Molecular Function	catalytic activity(GO:0003824)	1
OG0027441	Molecular Function	binding(GO:0005488)	1
OG0027442	Molecular Function	catalytic activity(GO:0003824)	1
OG0027452	Molecular Function	transcription regulator activity(GO:0140110)	1
OG0027455	Molecular Function	catalytic activity(GO:0003824)	1
OG0027458	Molecular Function	binding(GO:0005488)	1
OG0027461	Molecular Function	catalytic activity(GO:0003824)	1
OG0027474	Molecular Function	catalytic activity(GO:0003824)	1
OG0027475	Molecular Function	binding(GO:0005488)	1

OG0027479	Molecular Function	binding(GO:0005488)	1
OG0027479	Molecular Function	transcription regulator activity(GO:0140110)	1
OG0027486	Molecular Function	binding(GO:0005488)	1
OG0027487	Molecular Function	binding(GO:0005488)	1
OG0027488	Molecular Function	transcription regulator activity(GO:0140110)	1
OG0027492	Molecular Function	catalytic activity(GO:0003824)	1
OG0027507	Molecular Function	catalytic activity(GO:0003824)	1
OG0027510	Molecular Function	structural molecule activity(GO:0005198)	1
OG0027516	Molecular Function	transcription regulator activity(GO:0140110)	1
OG0027523	Molecular Function	binding(GO:0005488)	1
OG0027529	Molecular Function	catalytic activity(GO:0003824)	1
OG0027530	Molecular Function	binding(GO:0005488)	1
OG0027532	Molecular Function	catalytic activity(GO:0003824)	1
OG0027538	Molecular Function	catalytic activity(GO:0003824)	1
OG0027540	Molecular Function	catalytic activity(GO:0003824)	1
OG0027565	Molecular Function	catalytic activity(GO:0003824)	1
OG0027567	Molecular Function	catalytic activity(GO:0003824)	1
OG0027569	Molecular Function	binding(GO:0005488)	1
OG0027569	Molecular Function	catalytic activity(GO:0003824)	1
OG0027570	Molecular Function	binding(GO:0005488)	1
OG0027570	Molecular Function	catalytic activity(GO:0003824)	1
OG0027572	Molecular Function	catalytic activity(GO:0003824)	1
OG0027573	Molecular Function	catalytic activity(GO:0003824)	1
OG0027605	Molecular Function	catalytic activity(GO:0003824)	1
OG0027606	Molecular Function	binding(GO:0005488)	1
OG0027609	Molecular Function	binding(GO:0005488)	1
OG0027609	Molecular Function	catalytic activity(GO:0003824)	1
OG0027613	Molecular Function	catalytic activity(GO:0003824)	1
OG0027615	Molecular Function	transcription regulator activity(GO:0140110)	1
OG0027626	Molecular Function	transporter activity(GO:0005215)	1
OG0027634	Molecular Function	binding(GO:0005488)	1
OG0027634	Molecular Function	catalytic activity(GO:0003824)	1
OG0027636	Molecular Function	catalytic activity(GO:0003824)	1
OG0027640	Molecular Function	catalytic activity(GO:0003824)	1
OG0027643	Molecular Function	binding(GO:0005488)	1
OG0027643	Molecular Function	catalytic activity(GO:0003824)	1
OG0027644	Molecular Function	binding(GO:0005488)	1
OG0027644	Molecular Function	catalytic activity(GO:0003824)	1
OG0027669	Molecular Function	binding(GO:0005488)	1
OG0027669	Molecular Function	catalytic activity(GO:0003824)	1
OG0027704	Molecular Function	transcription regulator activity(GO:0140110)	1
OG0027708	Molecular Function	catalytic activity(GO:0003824)	1
OG0027711	Molecular Function	binding(GO:0005488)	1
OG0027711	Molecular Function	transcription regulator activity(GO:0140110)	1
OG0027714	Molecular Function	catalytic activity(GO:0003824)	1
OG0027718	Molecular Function	catalytic activity(GO:0003824)	1

OG0027738	Molecular Function	catalytic activity(GO:0003824)	1
OG0027738	Molecular Function	transporter activity(GO:0005215)	1
OG0027761	Molecular Function	transcription regulator activity(GO:0140110)	1
OG0027773	Molecular Function	catalytic activity(GO:0003824)	1
OG0027774	Molecular Function	catalytic activity(GO:0003824)	1
OG0027775	Molecular Function	catalytic activity(GO:0003824)	1
OG0027775	Molecular Function	molecular transducer activity(GO:0060089)	1
OG0027783	Molecular Function	binding(GO:0005488)	1
OG0027793	Molecular Function	catalytic activity(GO:0003824)	1
OG0027794	Molecular Function	binding(GO:0005488)	1
OG0027794	Molecular Function	catalytic activity(GO:0003824)	1
OG0027794	Molecular Function	molecular function regulator(GO:0098772)	1
OG0027795	Molecular Function	catalytic activity(GO:0003824)	1
OG0027800	Molecular Function	binding(GO:0005488)	1
OG0027800	Molecular Function	catalytic activity(GO:0003824)	1
OG0027803	Molecular Function	catalytic activity(GO:0003824)	1
OG0027811	Molecular Function	binding(GO:0005488)	1
OG0027811	Molecular Function	catalytic activity(GO:0003824)	1
OG0027811	Molecular Function	transporter activity(GO:0005215)	1
OG0027812	Molecular Function	binding(GO:0005488)	1
OG0027814	Molecular Function	catalytic activity(GO:0003824)	1
OG0027818	Molecular Function	transcription regulator activity(GO:0140110)	1
OG0027820	Molecular Function	binding(GO:0005488)	1
OG0027821	Molecular Function	binding(GO:0005488)	1
OG0027828	Molecular Function	binding(GO:0005488)	1
OG0027837	Molecular Function	transcription regulator activity(GO:0140110)	1
OG0027840	Molecular Function	binding(GO:0005488)	1
OG0027843	Molecular Function	binding(GO:0005488)	1
OG0027844	Molecular Function	binding(GO:0005488)	1
OG0027844	Molecular Function	catalytic activity(GO:0003824)	1
OG0027850	Molecular Function	binding(GO:0005488)	1
OG0027850	Molecular Function	catalytic activity(GO:0003824)	1
OG0027852	Molecular Function	binding(GO:0005488)	1
OG0027852	Molecular Function	catalytic activity(GO:0003824)	1
OG0027854	Molecular Function	catalytic activity(GO:0003824)	1
OG0027876	Molecular Function	catalytic activity(GO:0003824)	1
OG0027883	Molecular Function	binding(GO:0005488)	1
OG0027883	Molecular Function	catalytic activity(GO:0003824)	1
OG0027884	Molecular Function	transporter activity(GO:0005215)	1
OG0027887	Molecular Function	binding(GO:0005488)	1
OG0027887	Molecular Function	structural molecule activity(GO:0005198)	1
OG0027898	Molecular Function	catalytic activity(GO:0003824)	1
OG0027899	Molecular Function	binding(GO:0005488)	1
OG0027905	Molecular Function	catalytic activity(GO:0003824)	1
OG0027907	Molecular Function	binding(GO:0005488)	1
OG0027907	Molecular Function	catalytic activity(GO:0003824)	1

OG0027908	Molecular Function	binding(GO:0005488)	1
OG0027908	Molecular Function	catalytic activity(GO:0003824)	1
OG0027916	Molecular Function	catalytic activity(GO:0003824)	1
OG0027938	Molecular Function	catalytic activity(GO:0003824)	1
OG0027940	Molecular Function	transcription regulator activity(GO:0140110)	1
OG0027941	Molecular Function	catalytic activity(GO:0003824)	1
OG0027943	Molecular Function	binding(GO:0005488)	1
OG0027943	Molecular Function	catalytic activity(GO:0003824)	1
OG0027943	Molecular Function	transporter activity(GO:0005215)	1
OG0027952	Molecular Function	transcription regulator activity(GO:0140110)	1
OG0027953	Molecular Function	transcription regulator activity(GO:0140110)	1
OG0027962	Molecular Function	catalytic activity(GO:0003824)	1
OG0027964	Molecular Function	catalytic activity(GO:0003824)	1
OG0027966	Molecular Function	catalytic activity(GO:0003824)	1
OG0027968	Molecular Function	catalytic activity(GO:0003824)	1
OG0027981	Molecular Function	catalytic activity(GO:0003824)	1
OG0027984	Molecular Function	binding(GO:0005488)	1
OG0027984	Molecular Function	catalytic activity(GO:0003824)	1
OG0027986	Molecular Function	binding(GO:0005488)	1
OG0027987	Molecular Function	binding(GO:0005488)	1
OG0027993	Molecular Function	transcription regulator activity(GO:0140110)	1
OG0028002	Molecular Function	catalytic activity(GO:0003824)	1
OG0028003	Molecular Function	catalytic activity(GO:0003824)	1
OG0028016	Molecular Function	catalytic activity(GO:0003824)	1
OG0028018	Molecular Function	structural molecule activity(GO:0005198)	1
OG0028019	Molecular Function	structural molecule activity(GO:0005198)	1
OG0028038	Molecular Function	catalytic activity(GO:0003824)	1
OG0028043	Molecular Function	catalytic activity(GO:0003824)	1
OG0028048	Molecular Function	catalytic activity(GO:0003824)	1
OG0028052	Molecular Function	binding(GO:0005488)	1
OG0028052	Molecular Function	structural molecule activity(GO:0005198)	1
OG0028052	Molecular Function	transcription regulator activity(GO:0140110)	1
OG0028057	Molecular Function	structural molecule activity(GO:0005198)	1
OG0028059	Molecular Function	catalytic activity(GO:0003824)	1
OG0028063	Molecular Function	catalytic activity(GO:0003824)	1
OG0028065	Molecular Function	binding(GO:0005488)	1
OG0028065	Molecular Function	catalytic activity(GO:0003824)	1
OG0028068	Molecular Function	catalytic activity(GO:0003824)	1
OG0028071	Molecular Function	binding(GO:0005488)	1
OG0028071	Molecular Function	catalytic activity(GO:0003824)	1
OG0028073	Molecular Function	catalytic activity(GO:0003824)	1
OG0028074	Molecular Function	binding(GO:0005488)	1
OG0028074	Molecular Function	catalytic activity(GO:0003824)	1
OG0028076	Molecular Function	binding(GO:0005488)	1
OG0028076	Molecular Function	catalytic activity(GO:0003824)	1

OG0028078	Molecular Function	binding(GO:0005488)	1
OG0028078	Molecular Function	catalytic activity(GO:0003824)	1
OG0028078	Molecular Function	molecular function regulator(GO:0098772)	1
OG0028081	Molecular Function	binding(GO:0005488)	1
OG0028081	Molecular Function	catalytic activity(GO:0003824)	1
OG0028083	Molecular Function	catalytic activity(GO:0003824)	1
OG0028083	Molecular Function	transporter activity(GO:0005215)	1
OG0028087	Molecular Function	binding(GO:0005488)	1
OG0028087	Molecular Function	catalytic activity(GO:0003824)	1
OG0028089	Molecular Function	catalytic activity(GO:0003824)	1
OG0028091	Molecular Function	binding(GO:0005488)	1
OG0028091	Molecular Function	catalytic activity(GO:0003824)	1
OG0028098	Molecular Function	catalytic activity(GO:0003824)	1
OG0028099	Molecular Function	binding(GO:0005488)	1
OG0028099	Molecular Function	molecular transducer activity(GO:0060089)	1
OG0028116	Molecular Function	transcription regulator activity(GO:0140110)	1
OG0028119	Molecular Function	binding(GO:0005488)	1
OG0028124	Molecular Function	catalytic activity(GO:0003824)	1
OG0028128	Molecular Function	binding(GO:0005488)	1
OG0028128	Molecular Function	catalytic activity(GO:0003824)	1
OG0028132	Molecular Function	catalytic activity(GO:0003824)	1
OG0028139	Molecular Function	catalytic activity(GO:0003824)	1
OG0028145	Molecular Function	binding(GO:0005488)	1
OG0028152	Molecular Function	catalytic activity(GO:0003824)	1
OG0028153	Molecular Function	binding(GO:0005488)	1
OG0028153	Molecular Function	catalytic activity(GO:0003824)	1
OG0028160	Molecular Function	binding(GO:0005488)	1
OG0028160	Molecular Function	catalytic activity(GO:0003824)	1
OG0028171	Molecular Function	binding(GO:0005488)	1
OG0028171	Molecular Function	catalytic activity(GO:0003824)	1
OG0028175	Molecular Function	catalytic activity(GO:0003824)	1
OG0028176	Molecular Function	catalytic activity(GO:0003824)	1
OG0028177	Molecular Function	catalytic activity(GO:0003824)	1
OG0028180	Molecular Function	catalytic activity(GO:0003824)	1
OG0028181	Molecular Function	catalytic activity(GO:0003824)	1
OG0028184	Molecular Function	catalytic activity(GO:0003824)	1
OG0028185	Molecular Function	transcription regulator activity(GO:0140110)	1
OG0028186	Molecular Function	transcription regulator activity(GO:0140110)	1
OG0028193	Molecular Function	catalytic activity(GO:0003824)	1
OG0028196	Molecular Function	transcription regulator activity(GO:0140110)	1
OG0028200	Molecular Function	binding(GO:0005488)	1
OG0028201	Molecular Function	structural molecule activity(GO:0005198)	1
OG0028202	Molecular Function	binding(GO:0005488)	1
OG0028209	Molecular Function	binding(GO:0005488)	1
OG0028210	Molecular Function	binding(GO:0005488)	1
OG0028216	Molecular Function	binding(GO:0005488)	1

OG0028231	Molecular Function	catalytic activity(GO:0003824)	1
OG0028232	Molecular Function	catalytic activity(GO:0003824)	1
OG0028233	Molecular Function	catalytic activity(GO:0003824)	1
OG0028241	Molecular Function	binding(GO:0005488)	1
OG0028249	Molecular Function	catalytic activity(GO:0003824)	1
OG0028255	Molecular Function	binding(GO:0005488)	1
OG0028255	Molecular Function	molecular function regulator(GO:0098772)	1
OG0028259	Molecular Function	binding(GO:0005488)	1
OG0028259	Molecular Function	transcription regulator activity(GO:0140110)	1
OG0028299	Molecular Function	transporter activity(GO:0005215)	1
OG0028305	Molecular Function	binding(GO:0005488)	1
OG0028305	Molecular Function	transcription regulator activity(GO:0140110)	1
OG0028305	Molecular Function	translation regulator activity(GO:0045182)	1
OG0028308	Molecular Function	catalytic activity(GO:0003824)	1
OG0028323	Molecular Function	transcription regulator activity(GO:0140110)	1
OG0028334	Molecular Function	catalytic activity(GO:0003824)	1
OG0028334	Molecular Function	molecular transducer activity(GO:0060089)	1
OG0028345	Molecular Function	transcription regulator activity(GO:0140110)	1
OG0028373	Molecular Function	binding(GO:0005488)	1
OG0028373	Molecular Function	catalytic activity(GO:0003824)	1
OG0028388	Molecular Function	binding(GO:0005488)	1
OG0028388	Molecular Function	transcription regulator activity(GO:0140110)	1
OG0028390	Molecular Function	binding(GO:0005488)	1
OG0028399	Molecular Function	catalytic activity(GO:0003824)	1
OG0028405	Molecular Function	binding(GO:0005488)	1
OG0028405	Molecular Function	catalytic activity(GO:0003824)	1
OG0028410	Molecular Function	binding(GO:0005488)	1
OG0028410	Molecular Function	catalytic activity(GO:0003824)	1
OG0028415	Molecular Function	catalytic activity(GO:0003824)	1
OG0028416	Molecular Function	catalytic activity(GO:0003824)	1
OG0028417	Molecular Function	transcription regulator activity(GO:0140110)	1
OG0028419	Molecular Function	catalytic activity(GO:0003824)	1
OG0028419	Molecular Function	molecular transducer activity(GO:0060089)	1
OG0028420	Molecular Function	structural molecule activity(GO:0005198)	1
OG0028433	Molecular Function	transcription regulator activity(GO:0140110)	1
OG0028440	Molecular Function	binding(GO:0005488)	1
OG0028440	Molecular Function	catalytic activity(GO:0003824)	1
OG0028446	Molecular Function	catalytic activity(GO:0003824)	1
OG0028447	Molecular Function	catalytic activity(GO:0003824)	1
OG0028448	Molecular Function	catalytic activity(GO:0003824)	1
OG0028468	Molecular Function	binding(GO:0005488)	1
OG0028500	Molecular Function	binding(GO:0005488)	1
OG0028504	Molecular Function	transcription regulator activity(GO:0140110)	1
OG0028505	Molecular Function	binding(GO:0005488)	1

OG0028506	Molecular Function	catalytic activity(GO:0003824)	1
OG0028528	Molecular Function	transcription regulator activity(GO:0140110)	1
OG0028530	Molecular Function	transcription regulator activity(GO:0140110)	1
OG0028538	Molecular Function	transcription regulator activity(GO:0140110)	1
OG0028539	Molecular Function	catalytic activity(GO:0003824)	1
OG0028545	Molecular Function	binding(GO:0005488)	1
OG0028548	Molecular Function	catalytic activity(GO:0003824)	1
OG0028549	Molecular Function	catalytic activity(GO:0003824)	1
OG0028549	Molecular Function	molecular function regulator(GO:0098772)	1
OG0028551	Molecular Function	catalytic activity(GO:0003824)	1
OG0028551	Molecular Function	molecular function regulator(GO:0098772)	1
OG0028556	Molecular Function	transcription regulator activity(GO:0140110)	1
OG0028562	Molecular Function	structural molecule activity(GO:0005198)	1
OG0028566	Molecular Function	binding(GO:0005488)	1
OG0028566	Molecular Function	catalytic activity(GO:0003824)	1
OG0028571	Molecular Function	binding(GO:0005488)	1
OG0028571	Molecular Function	catalytic activity(GO:0003824)	1
OG0028572	Molecular Function	binding(GO:0005488)	1
OG0028572	Molecular Function	catalytic activity(GO:0003824)	1
OG0028593	Molecular Function	catalytic activity(GO:0003824)	1
OG0028595	Molecular Function	transcription regulator activity(GO:0140110)	1
OG0028597	Molecular Function	binding(GO:0005488)	1
OG0028597	Molecular Function	catalytic activity(GO:0003824)	1
OG0028610	Molecular Function	binding(GO:0005488)	1
OG0028610	Molecular Function	transcription regulator activity(GO:0140110)	1
OG0028624	Molecular Function	transporter activity(GO:0005215)	1
OG0028627	Molecular Function	transcription regulator activity(GO:0140110)	1
OG0028630	Molecular Function	catalytic activity(GO:0003824)	1
OG0028656	Molecular Function	binding(GO:0005488)	1
OG0028656	Molecular Function	catalytic activity(GO:0003824)	1
OG0028657	Molecular Function	catalytic activity(GO:0003824)	1
OG0028661	Molecular Function	binding(GO:0005488)	1
OG0028661	Molecular Function	catalytic activity(GO:0003824)	1
OG0028662	Molecular Function	catalytic activity(GO:0003824)	1
OG0028663	Molecular Function	catalytic activity(GO:0003824)	1
OG0028664	Molecular Function	catalytic activity(GO:0003824)	1
OG0028666	Molecular Function	catalytic activity(GO:0003824)	1
OG0028672	Molecular Function	binding(GO:0005488)	1
OG0028672	Molecular Function	structural molecule activity(GO:0005198)	1
OG0028672	Molecular Function	transcription regulator activity(GO:0140110)	1
OG0028713	Molecular Function	transcription regulator activity(GO:0140110)	1
OG0028719	Molecular Function	catalytic activity(GO:0003824)	1
OG0028722	Molecular Function	transporter activity(GO:0005215)	1
OG0028732	Molecular Function	catalytic activity(GO:0003824)	1

OG0028745	Molecular Function	catalytic activity(GO:0003824)	1
OG0028746	Molecular Function	catalytic activity(GO:0003824)	1
OG0028747	Molecular Function	binding(GO:0005488)	1
OG0028747	Molecular Function	catalytic activity(GO:0003824)	1
OG0028749	Molecular Function	binding(GO:0005488)	1
OG0028749	Molecular Function	catalytic activity(GO:0003824)	1
OG0028769	Molecular Function	transcription regulator activity(GO:0140110)	1
OG0028771	Molecular Function	catalytic activity(GO:0003824)	1
OG0028822	Molecular Function	protein tag(GO:0031386)	1
OG0028843	Molecular Function	transporter activity(GO:0005215)	1
OG0028849	Molecular Function	transcription regulator activity(GO:0140110)	1
OG0028859	Molecular Function	catalytic activity(GO:0003824)	1
OG0028859	Molecular Function	molecular transducer activity(GO:0060089)	1
OG0028867	Molecular Function	binding(GO:0005488)	1
OG0028874	Molecular Function	catalytic activity(GO:0003824)	1
OG0028878	Molecular Function	catalytic activity(GO:0003824)	1
OG0028879	Molecular Function	binding(GO:0005488)	1
OG0028903	Molecular Function	binding(GO:0005488)	1
OG0028903	Molecular Function	catalytic activity(GO:0003824)	1
OG0028907	Molecular Function	catalytic activity(GO:0003824)	1
OG0028907	Molecular Function	transporter activity(GO:0005215)	1
OG0028908	Molecular Function	binding(GO:0005488)	1
OG0028908	Molecular Function	catalytic activity(GO:0003824)	1
OG0028910	Molecular Function	binding(GO:0005488)	1
OG0028913	Molecular Function	binding(GO:0005488)	1
OG0028913	Molecular Function	catalytic activity(GO:0003824)	1
OG0028925	Molecular Function	transporter activity(GO:0005215)	1
OG0028941	Molecular Function	transcription regulator activity(GO:0140110)	1
OG0028946	Molecular Function	transcription regulator activity(GO:0140110)	1
OG0028991	Molecular Function	molecular transducer activity(GO:0060089)	1
OG0028991	Molecular Function	transporter activity(GO:0005215)	1
OG0029012	Molecular Function	catalytic activity(GO:0003824)	1
OG0029015	Molecular Function	catalytic activity(GO:0003824)	1
OG0029015	Molecular Function	molecular transducer activity(GO:0060089)	1
OG0029029	Molecular Function	binding(GO:0005488)	1
OG0029029	Molecular Function	catalytic activity(GO:0003824)	1
OG0029033	Molecular Function	binding(GO:0005488)	1
OG0029033	Molecular Function	catalytic activity(GO:0003824)	1
OG0029035	Molecular Function	catalytic activity(GO:0003824)	1
OG0029037	Molecular Function	binding(GO:0005488)	1
OG0029047	Molecular Function	catalytic activity(GO:0003824)	1
OG0029058	Molecular Function	binding(GO:0005488)	1
OG0029060	Molecular Function	transcription regulator activity(GO:0140110)	1
OG0029072	Molecular Function	binding(GO:0005488)	1
OG0029072	Molecular Function	structural molecule activity(GO:0005198)	1

OG0029099	Molecular Function	binding(GO:0005488)	1
OG0029112	Molecular Function	catalytic activity(GO:0003824)	1
OG0029117	Molecular Function	catalytic activity(GO:0003824)	1
OG0029118	Molecular Function	binding(GO:0005488)	1
OG0029122	Molecular Function	catalytic activity(GO:0003824)	1
OG0029124	Molecular Function	catalytic activity(GO:0003824)	1
OG0029124	Molecular Function	transporter activity(GO:0005215)	1
OG0029125	Molecular Function	binding(GO:0005488)	1
OG0029131	Molecular Function	structural molecule activity(GO:0005198)	1
OG0029141	Molecular Function	binding(GO:0005488)	1
OG0029141	Molecular Function	catalytic activity(GO:0003824)	1
OG0029143	Molecular Function	transcription regulator activity(GO:0140110)	1
OG0029161	Molecular Function	catalytic activity(GO:0003824)	1
OG0029165	Molecular Function	catalytic activity(GO:0003824)	1
OG0029172	Molecular Function	transporter activity(GO:0005215)	1
OG0029194	Molecular Function	transcription regulator activity(GO:0140110)	1
OG0029203	Molecular Function	binding(GO:0005488)	1
OG0029204	Molecular Function	binding(GO:0005488)	1
OG0029230	Molecular Function	catalytic activity(GO:0003824)	1
OG0029254	Molecular Function	transporter activity(GO:0005215)	1
OG0029294	Molecular Function	catalytic activity(GO:0003824)	1
OG0029295	Molecular Function	binding(GO:0005488)	1
OG0029295	Molecular Function	catalytic activity(GO:0003824)	1
OG0029315	Molecular Function	transcription regulator activity(GO:0140110)	1
OG0029316	Molecular Function	binding(GO:0005488)	1
OG0029316	Molecular Function	transcription regulator activity(GO:0140110)	1
OG0029316	Molecular Function	translation regulator activity(GO:0045182)	1
OG0029319	Molecular Function	catalytic activity(GO:0003824)	1
OG0029319	Molecular Function	transporter activity(GO:0005215)	1
OG0029323	Molecular Function	binding(GO:0005488)	1
OG0029327	Molecular Function	catalytic activity(GO:0003824)	1
OG0029337	Molecular Function	binding(GO:0005488)	1
OG0029337	Molecular Function	catalytic activity(GO:0003824)	1
OG0029338	Molecular Function	catalytic activity(GO:0003824)	1
OG0029341	Molecular Function	binding(GO:0005488)	1
OG0029341	Molecular Function	catalytic activity(GO:0003824)	1
OG0029348	Molecular Function	binding(GO:0005488)	1
OG0029348	Molecular Function	catalytic activity(GO:0003824)	1
OG0029355	Molecular Function	binding(GO:0005488)	1
OG0029355	Molecular Function	catalytic activity(GO:0003824)	1
OG0029356	Molecular Function	catalytic activity(GO:0003824)	1
OG0029359	Molecular Function	antioxidant activity(GO:0016209)	1
OG0029359	Molecular Function	catalytic activity(GO:0003824)	1
OG0029362	Molecular Function	binding(GO:0005488)	1
OG0029362	Molecular Function	catalytic activity(GO:0003824)	1
OG0029366	Molecular Function	catalytic activity(GO:0003824)	1

OG0029375	Molecular Function	catalytic activity(GO:0003824)	1
OG0029376	Molecular Function	transcription regulator activity(GO:0140110)	1
OG0029384	Molecular Function	molecular function regulator(GO:0098772)	1
OG0029387	Molecular Function	binding(GO:0005488)	1
OG0029389	Molecular Function	catalytic activity(GO:0003824)	1
OG0029391	Molecular Function	transcription regulator activity(GO:0140110)	1
OG0029392	Molecular Function	binding(GO:0005488)	1
OG0029414	Molecular Function	structural molecule activity(GO:0005198)	1
OG0029421	Molecular Function	catalytic activity(GO:0003824)	1
OG0029424	Molecular Function	binding(GO:0005488)	1
OG0029426	Molecular Function	transcription regulator activity(GO:0140110)	1
OG0029429	Molecular Function	molecular function regulator(GO:0098772)	1
OG0029430	Molecular Function	molecular function regulator(GO:0098772)	1
OG0029431	Molecular Function	molecular function regulator(GO:0098772)	1
OG0029453	Molecular Function	binding(GO:0005488)	1
OG0029453	Molecular Function	transcription regulator activity(GO:0140110)	1
OG0029457	Molecular Function	binding(GO:0005488)	1
OG0029457	Molecular Function	catalytic activity(GO:0003824)	1
OG0029458	Molecular Function	binding(GO:0005488)	1
OG0029458	Molecular Function	catalytic activity(GO:0003824)	1
OG0029459	Molecular Function	binding(GO:0005488)	1
OG0029461	Molecular Function	catalytic activity(GO:0003824)	1
OG0029466	Molecular Function	binding(GO:0005488)	1
OG0029467	Molecular Function	binding(GO:0005488)	1
OG0029469	Molecular Function	catalytic activity(GO:0003824)	1
OG0029470	Molecular Function	binding(GO:0005488)	1
OG0029470	Molecular Function	structural molecule activity(GO:0005198)	1
OG0029471	Molecular Function	binding(GO:0005488)	1
OG0029471	Molecular Function	structural molecule activity(GO:0005198)	1
OG0029472	Molecular Function	catalytic activity(GO:0003824)	1
OG0029479	Molecular Function	catalytic activity(GO:0003824)	1
OG0029482	Molecular Function	binding(GO:0005488)	1
OG0029482	Molecular Function	catalytic activity(GO:0003824)	1
OG0029486	Molecular Function	binding(GO:0005488)	1
OG0029486	Molecular Function	molecular transducer activity(GO:0060089)	1
OG0029489	Molecular Function	catalytic activity(GO:0003824)	1
OG0029490	Molecular Function	binding(GO:0005488)	1
OG0029490	Molecular Function	catalytic activity(GO:0003824)	1
OG0029493	Molecular Function	catalytic activity(GO:0003824)	1
OG0029497	Molecular Function	catalytic activity(GO:0003824)	1
OG0029502	Molecular Function	catalytic activity(GO:0003824)	1
OG0029504	Molecular Function	catalytic activity(GO:0003824)	1
OG0029509	Molecular Function	transporter activity(GO:0005215)	1
OG0029517	Molecular Function	catalytic activity(GO:0003824)	1

OG0029522	Molecular Function	binding(GO:0005488)	1
OG0029522	Molecular Function	catalytic activity(GO:0003824)	1
OG0029527	Molecular Function	binding(GO:0005488)	1
OG0029527	Molecular Function	catalytic activity(GO:0003824)	1
OG0029528	Molecular Function	catalytic activity(GO:0003824)	1
OG0029539	Molecular Function	catalytic activity(GO:0003824)	1
OG0029540	Molecular Function	catalytic activity(GO:0003824)	1
OG0029542	Molecular Function	binding(GO:0005488)	1
OG0029542	Molecular Function	catalytic activity(GO:0003824)	1
OG0029543	Molecular Function	binding(GO:0005488)	1
OG0029543	Molecular Function	catalytic activity(GO:0003824)	1
OG0029549	Molecular Function	antioxidant activity(GO:0016209)	1
OG0029549	Molecular Function	catalytic activity(GO:0003824)	1
OG0029550	Molecular Function	binding(GO:0005488)	1
OG0029550	Molecular Function	catalytic activity(GO:0003824)	1
OG0029553	Molecular Function	catalytic activity(GO:0003824)	1
OG0029554	Molecular Function	catalytic activity(GO:0003824)	1
OG0029554	Molecular Function	transporter activity(GO:0005215)	1
OG0029567	Molecular Function	antioxidant activity(GO:0016209)	1
OG0029567	Molecular Function	catalytic activity(GO:0003824)	1
OG0029569	Molecular Function	catalytic activity(GO:0003824)	1
OG0029571	Molecular Function	binding(GO:0005488)	1
OG0029571	Molecular Function	catalytic activity(GO:0003824)	1
OG0029576	Molecular Function	binding(GO:0005488)	1
OG0029576	Molecular Function	catalytic activity(GO:0003824)	1
OG0029578	Molecular Function	catalytic activity(GO:0003824)	1
OG0029581	Molecular Function	catalytic activity(GO:0003824)	1
OG0029585	Molecular Function	catalytic activity(GO:0003824)	1
OG0029588	Molecular Function	molecular function regulator(GO:0098772)	1
OG0029590	Molecular Function	catalytic activity(GO:0003824)	1
OG0029610	Molecular Function	catalytic activity(GO:0003824)	1
OG0029626	Molecular Function	transporter activity(GO:0005215)	1
OG0029632	Molecular Function	transcription regulator activity(GO:0140110)	1
OG0029636	Molecular Function	structural molecule activity(GO:0005198)	1
OG0029637	Molecular Function	structural molecule activity(GO:0005198)	1
OG0029640	Molecular Function	catalytic activity(GO:0003824)	1
OG0029645	Molecular Function	molecular transducer activity(GO:0060089)	1
OG0029648	Molecular Function	transcription regulator activity(GO:0140110)	1
OG0029650	Molecular Function	catalytic activity(GO:0003824)	1
OG0029651	Molecular Function	catalytic activity(GO:0003824)	1
OG0029656	Molecular Function	catalytic activity(GO:0003824)	1
OG0029660	Molecular Function	binding(GO:0005488)	1
OG0029660	Molecular Function	catalytic activity(GO:0003824)	1
OG0029665	Molecular Function	molecular function regulator(GO:0098772)	1
OG0029679	Molecular Function	transcription regulator activity(GO:0140110)	1

OG0029690	Molecular Function	catalytic activity(GO:0003824)	1
OG0029691	Molecular Function	binding(GO:0005488)	1
OG0029691	Molecular Function	catalytic activity(GO:0003824)	1
OG0029692	Molecular Function	catalytic activity(GO:0003824)	1
OG0029744	Molecular Function	antioxidant activity(GO:0016209)	1
OG0029744	Molecular Function	binding(GO:0005488)	1
OG0029744	Molecular Function	catalytic activity(GO:0003824)	1
OG0029753	Molecular Function	transcription regulator activity(GO:0140110)	1
OG0029757	Molecular Function	structural molecule activity(GO:0005198)	1
OG0029758	Molecular Function	catalytic activity(GO:0003824)	1
OG0029768	Molecular Function	catalytic activity(GO:0003824)	1
OG0029777	Molecular Function	catalytic activity(GO:0003824)	1
OG0029791	Molecular Function	catalytic activity(GO:0003824)	1
OG0029793	Molecular Function	binding(GO:0005488)	1
OG0029807	Molecular Function	catalytic activity(GO:0003824)	1
OG0029813	Molecular Function	binding(GO:0005488)	1
OG0029813	Molecular Function	catalytic activity(GO:0003824)	1
OG0029820	Molecular Function	binding(GO:0005488)	1
OG0029820	Molecular Function	transcription regulator activity(GO:0140110)	1
OG0029820	Molecular Function	translation regulator activity(GO:0045182)	1
OG0029837	Molecular Function	catalytic activity(GO:0003824)	1
OG0029837	Molecular Function	molecular transducer activity(GO:0060089)	1
OG0029839	Molecular Function	catalytic activity(GO:0003824)	1
OG0029840	Molecular Function	catalytic activity(GO:0003824)	1
OG0029842	Molecular Function	binding(GO:0005488)	1
OG0029842	Molecular Function	catalytic activity(GO:0003824)	1
OG0029845	Molecular Function	catalytic activity(GO:0003824)	1
OG0029847	Molecular Function	binding(GO:0005488)	1
OG0029847	Molecular Function	catalytic activity(GO:0003824)	1
OG0029847	Molecular Function	transporter activity(GO:0005215)	1
OG0029848	Molecular Function	binding(GO:0005488)	1
OG0029848	Molecular Function	catalytic activity(GO:0003824)	1
OG0029853	Molecular Function	binding(GO:0005488)	1
OG0029853	Molecular Function	catalytic activity(GO:0003824)	1
OG0029854	Molecular Function	catalytic activity(GO:0003824)	1
OG0029860	Molecular Function	binding(GO:0005488)	1
OG0029862	Molecular Function	catalytic activity(GO:0003824)	1
OG0029865	Molecular Function	catalytic activity(GO:0003824)	1
OG0029869	Molecular Function	catalytic activity(GO:0003824)	1
OG0029876	Molecular Function	binding(GO:0005488)	1
OG0029876	Molecular Function	catalytic activity(GO:0003824)	1
OG0029878	Molecular Function	catalytic activity(GO:0003824)	1
OG0029881	Molecular Function	binding(GO:0005488)	1
OG0029881	Molecular Function	catalytic activity(GO:0003824)	1
OG0029882	Molecular Function	binding(GO:0005488)	1
OG0029882	Molecular Function	catalytic activity(GO:0003824)	1

OG0029884	Molecular Function	catalytic activity(GO:0003824)	1
OG0029886	Molecular Function	catalytic activity(GO:0003824)	1
OG0029893	Molecular Function	catalytic activity(GO:0003824)	1
OG0029894	Molecular Function	binding(GO:0005488)	1
OG0029894	Molecular Function	catalytic activity(GO:0003824)	1
OG0029895	Molecular Function	binding(GO:0005488)	1
OG0029895	Molecular Function	catalytic activity(GO:0003824)	1
OG0029904	Molecular Function	binding(GO:0005488)	1
OG0029904	Molecular Function	catalytic activity(GO:0003824)	1
OG0029906	Molecular Function	catalytic activity(GO:0003824)	1
OG0029907	Molecular Function	transporter activity(GO:0005215)	1
OG0029908	Molecular Function	catalytic activity(GO:0003824)	1
OG0029909	Molecular Function	transporter activity(GO:0005215)	1
OG0029910	Molecular Function	catalytic activity(GO:0003824)	1
OG0029913	Molecular Function	binding(GO:0005488)	1
OG0029913	Molecular Function	catalytic activity(GO:0003824)	1
OG0029914	Molecular Function	binding(GO:0005488)	1
OG0029914	Molecular Function	catalytic activity(GO:0003824)	1
OG0029921	Molecular Function	antioxidant activity(GO:0016209)	1
OG0029921	Molecular Function	catalytic activity(GO:0003824)	1
OG0029928	Molecular Function	binding(GO:0005488)	1
OG0029928	Molecular Function	catalytic activity(GO:0003824)	1
OG0029932	Molecular Function	catalytic activity(GO:0003824)	1
OG0029936	Molecular Function	catalytic activity(GO:0003824)	1
OG0029937	Molecular Function	catalytic activity(GO:0003824)	1
OG0029937	Molecular Function	transporter activity(GO:0005215)	1
OG0029941	Molecular Function	binding(GO:0005488)	1
OG0029941	Molecular Function	catalytic activity(GO:0003824)	1
OG0029951	Molecular Function	catalytic activity(GO:0003824)	1
OG0029952	Molecular Function	catalytic activity(GO:0003824)	1
OG0029957	Molecular Function	transcription regulator activity(GO:0140110)	1
OG0029962	Molecular Function	binding(GO:0005488)	1
OG0029964	Molecular Function	binding(GO:0005488)	1
OG0029965	Molecular Function	binding(GO:0005488)	1
OG0029967	Molecular Function	structural molecule activity(GO:0005198)	1
OG0029968	Molecular Function	structural molecule activity(GO:0005198)	1
OG0029971	Molecular Function	binding(GO:0005488)	1
OG0029971	Molecular Function	structural molecule activity(GO:0005198)	1
OG0029972	Molecular Function	structural molecule activity(GO:0005198)	1
OG0029973	Molecular Function	structural molecule activity(GO:0005198)	1
OG0029974	Molecular Function	catalytic activity(GO:0003824)	1
OG0029975	Molecular Function	structural molecule activity(GO:0005198)	1
OG0029983	Molecular Function	transcription regulator activity(GO:0140110)	1
OG0030006	Molecular Function	catalytic activity(GO:0003824)	1
OG0030014	Molecular Function	binding(GO:0005488)	1

OG0030016	Molecular Function	binding(GO:0005488)	1
OG0030019	Molecular Function	catalytic activity(GO:0003824)	1
OG0030021	Molecular Function	catalytic activity(GO:0003824)	1
OG0030026	Molecular Function	binding(GO:0005488)	1
OG0030028	Molecular Function	binding(GO:0005488)	1
OG0030032	Molecular Function	binding(GO:0005488)	1
OG0030032	Molecular Function	catalytic activity(GO:0003824)	1
OG0030033	Molecular Function	binding(GO:0005488)	1
OG0030033	Molecular Function	catalytic activity(GO:0003824)	1
OG0030035	Molecular Function	catalytic activity(GO:0003824)	1
OG0030036	Molecular Function	binding(GO:0005488)	1
OG0030036	Molecular Function	catalytic activity(GO:0003824)	1
OG0030037	Molecular Function	catalytic activity(GO:0003824)	1
OG0030045	Molecular Function	transcription regulator activity(GO:0140110)	1
OG0030046	Molecular Function	transcription regulator activity(GO:0140110)	1
OG0030049	Molecular Function	catalytic activity(GO:0003824)	1
OG0030057	Molecular Function	transcription regulator activity(GO:0140110)	1
OG0030061	Molecular Function	transporter activity(GO:0005215)	1
OG0030062	Molecular Function	catalytic activity(GO:0003824)	1
OG0030063	Molecular Function	binding(GO:0005488)	1
OG0030063	Molecular Function	catalytic activity(GO:0003824)	1
OG0030065	Molecular Function	catalytic activity(GO:0003824)	1
OG0030071	Molecular Function	catalytic activity(GO:0003824)	1
OG0030072	Molecular Function	catalytic activity(GO:0003824)	1
OG0030072	Molecular Function	molecular transducer activity(GO:0060089)	1
OG0030077	Molecular Function	binding(GO:0005488)	1
OG0030077	Molecular Function	catalytic activity(GO:0003824)	1
OG0030080	Molecular Function	catalytic activity(GO:0003824)	1
OG0030083	Molecular Function	catalytic activity(GO:0003824)	1
OG0030087	Molecular Function	molecular function regulator(GO:0098772)	1
OG0030088	Molecular Function	transcription regulator activity(GO:0140110)	1
OG0030089	Molecular Function	binding(GO:0005488)	1
OG0030089	Molecular Function	transporter activity(GO:0005215)	1
OG0030095	Molecular Function	catalytic activity(GO:0003824)	1
OG0030100	Molecular Function	catalytic activity(GO:0003824)	1
OG0030110	Molecular Function	catalytic activity(GO:0003824)	1
OG0030111	Molecular Function	antioxidant activity(GO:0016209)	1
OG0030111	Molecular Function	catalytic activity(GO:0003824)	1
OG0030112	Molecular Function	transporter activity(GO:0005215)	1
OG0030116	Molecular Function	binding(GO:0005488)	1
OG0030116	Molecular Function	catalytic activity(GO:0003824)	1
OG0030135	Molecular Function	binding(GO:0005488)	1
OG0030135	Molecular Function	catalytic activity(GO:0003824)	1
OG0030141	Molecular Function	catalytic activity(GO:0003824)	1
OG0030147	Molecular Function	catalytic activity(GO:0003824)	1
OG0030150	Molecular Function	binding(GO:0005488)	1

OG0030150	Molecular Function	structural molecule activity(GO:0005198)	1
OG0030150	Molecular Function	transcription regulator activity(GO:0140110)	1
OG0030155	Molecular Function	transporter activity(GO:0005215)	1
OG0030162	Molecular Function	structural molecule activity(GO:0005198)	1
OG0030162	Molecular Function	translation regulator activity(GO:0045182)	1
OG0030166	Molecular Function	catalytic activity(GO:0003824)	1
OG0030168	Molecular Function	binding(GO:0005488)	1
OG0030175	Molecular Function	binding(GO:0005488)	1
OG0030180	Molecular Function	catalytic activity(GO:0003824)	1
OG0030182	Molecular Function	catalytic activity(GO:0003824)	1
OG0030184	Molecular Function	catalytic activity(GO:0003824)	1
OG0030191	Molecular Function	transcription regulator activity(GO:0140110)	1
OG0030195	Molecular Function	antioxidant activity(GO:0016209)	1
OG0030195	Molecular Function	catalytic activity(GO:0003824)	1
OG0030196	Molecular Function	protein tag(GO:0031386)	1
OG0030200	Molecular Function	catalytic activity(GO:0003824)	1
OG0030209	Molecular Function	transcription regulator activity(GO:0140110)	1
OG0030213	Molecular Function	binding(GO:0005488)	1
OG0030213	Molecular Function	catalytic activity(GO:0003824)	1
OG0030216	Molecular Function	catalytic activity(GO:0003824)	1
OG0030218	Molecular Function	catalytic activity(GO:0003824)	1
OG0030219	Molecular Function	catalytic activity(GO:0003824)	1
OG0030224	Molecular Function	catalytic activity(GO:0003824)	1
OG0030225	Molecular Function	binding(GO:0005488)	1
OG0030225	Molecular Function	catalytic activity(GO:0003824)	1
OG0030230	Molecular Function	binding(GO:0005488)	1
OG0030230	Molecular Function	catalytic activity(GO:0003824)	1
OG0030236	Molecular Function	catalytic activity(GO:0003824)	1
OG0030239	Molecular Function	binding(GO:0005488)	1
OG0030239	Molecular Function	catalytic activity(GO:0003824)	1
OG0030241	Molecular Function	binding(GO:0005488)	1
OG0030241	Molecular Function	catalytic activity(GO:0003824)	1
OG0030241	Molecular Function	molecular carrier activity(GO:0140104)	1
OG0030241	Molecular Function	transporter activity(GO:0005215)	1
OG0030244	Molecular Function	binding(GO:0005488)	1
OG0030244	Molecular Function	catalytic activity(GO:0003824)	1
OG0030246	Molecular Function	structural molecule activity(GO:0005198)	1
OG0030247	Molecular Function	binding(GO:0005488)	1
OG0030247	Molecular Function	catalytic activity(GO:0003824)	1
OG0030248	Molecular Function	binding(GO:0005488)	1
OG0030249	Molecular Function	binding(GO:0005488)	1
OG0030250	Molecular Function	structural molecule activity(GO:0005198)	1
OG0030252	Molecular Function	binding(GO:0005488)	1
OG0030252	Molecular Function	structural molecule activity(GO:0005198)	1
OG0030253	Molecular Function	binding(GO:0005488)	1

OG0030253	Molecular Function	structural molecule activity(GO:0005198)	1
OG0030254	Molecular Function	structural molecule activity(GO:0005198)	1
OG0030255	Molecular Function	structural molecule activity(GO:0005198)	1
OG0030257	Molecular Function	binding(GO:0005488)	1
OG0030257	Molecular Function	catalytic activity(GO:0003824)	1
OG0030259	Molecular Function	structural molecule activity(GO:0005198)	1
OG0030260	Molecular Function	structural molecule activity(GO:0005198)	1
OG0030261	Molecular Function	structural molecule activity(GO:0005198)	1
OG0030263	Molecular Function	binding(GO:0005488)	1
OG0030263	Molecular Function	catalytic activity(GO:0003824)	1
OG0030266	Molecular Function	binding(GO:0005488)	1
OG0030266	Molecular Function	catalytic activity(GO:0003824)	1
OG0030267	Molecular Function	binding(GO:0005488)	1
OG0030267	Molecular Function	catalytic activity(GO:0003824)	1
OG0030269	Molecular Function	catalytic activity(GO:0003824)	1
OG0030272	Molecular Function	binding(GO:0005488)	1
OG0030272	Molecular Function	catalytic activity(GO:0003824)	1
OG0030273	Molecular Function	catalytic activity(GO:0003824)	1
OG0030275	Molecular Function	structural molecule activity(GO:0005198)	1
OG0030278	Molecular Function	structural molecule activity(GO:0005198)	1
OG0030281	Molecular Function	catalytic activity(GO:0003824)	1
OG0030282	Molecular Function	binding(GO:0005488)	1
OG0030282	Molecular Function	catalytic activity(GO:0003824)	1
OG0030287	Molecular Function	transcription regulator activity(GO:0140110)	1
OG0030289	Molecular Function	catalytic activity(GO:0003824)	1
OG0030289	Molecular Function	transporter activity(GO:0005215)	1
OG0030291	Molecular Function	catalytic activity(GO:0003824)	1
OG0030298	Molecular Function	binding(GO:0005488)	1
OG0030298	Molecular Function	catalytic activity(GO:0003824)	1
OG0030299	Molecular Function	binding(GO:0005488)	1
OG0030300	Molecular Function	catalytic activity(GO:0003824)	1
OG0030301	Molecular Function	binding(GO:0005488)	1
OG0030302	Molecular Function	binding(GO:0005488)	1
OG0030302	Molecular Function	catalytic activity(GO:0003824)	1
OG0030305	Molecular Function	catalytic activity(GO:0003824)	1
OG0030309	Molecular Function	catalytic activity(GO:0003824)	1
OG0030310	Molecular Function	binding(GO:0005488)	1
OG0030310	Molecular Function	catalytic activity(GO:0003824)	1
OG0030314	Molecular Function	binding(GO:0005488)	1
OG0030314	Molecular Function	molecular carrier activity(GO:0140104)	1
OG0030315	Molecular Function	catalytic activity(GO:0003824)	1
OG0030317	Molecular Function	catalytic activity(GO:0003824)	1
OG0030318	Molecular Function	binding(GO:0005488)	1
OG0030318	Molecular Function	catalytic activity(GO:0003824)	1
OG0030320	Molecular Function	catalytic activity(GO:0003824)	1

OG0030322	Molecular Function	catalytic activity(GO:0003824)	1
OG0030326	Molecular Function	binding(GO:0005488)	1
OG0030326	Molecular Function	catalytic activity(GO:0003824)	1
OG0030327	Molecular Function	catalytic activity(GO:0003824)	1
OG0030330	Molecular Function	binding(GO:0005488)	1
OG0030336	Molecular Function	catalytic activity(GO:0003824)	1
OG0030348	Molecular Function	antioxidant activity(GO:0016209)	1
OG0030348	Molecular Function	catalytic activity(GO:0003824)	1
OG0030361	Molecular Function	catalytic activity(GO:0003824)	1
OG0030361	Molecular Function	molecular transducer activity(GO:0060089)	1
OG0030367	Molecular Function	transporter activity(GO:0005215)	1
OG0030371	Molecular Function	catalytic activity(GO:0003824)	1
OG0030372	Molecular Function	molecular carrier activity(GO:0140104)	1
OG0030376	Molecular Function	transcription regulator activity(GO:0140110)	1
OG0030384	Molecular Function	binding(GO:0005488)	1
OG0030388	Molecular Function	transcription regulator activity(GO:0140110)	1
OG0030389	Molecular Function	transcription regulator activity(GO:0140110)	1
OG0030398	Molecular Function	binding(GO:0005488)	1
OG0030423	Molecular Function	catalytic activity(GO:0003824)	1
OG0030435	Molecular Function	binding(GO:0005488)	1
OG0030435	Molecular Function	catalytic activity(GO:0003824)	1
OG0030436	Molecular Function	catalytic activity(GO:0003824)	1
OG0030441	Molecular Function	binding(GO:0005488)	1
OG0030441	Molecular Function	catalytic activity(GO:0003824)	1
OG0030446	Molecular Function	catalytic activity(GO:0003824)	1
OG0030458	Molecular Function	catalytic activity(GO:0003824)	1
OG0030498	Molecular Function	binding(GO:0005488)	1
OG0030524	Molecular Function	transcription regulator activity(GO:0140110)	1
OG0030542	Molecular Function	binding(GO:0005488)	1
OG0030551	Molecular Function	catalytic activity(GO:0003824)	1
OG0030552	Molecular Function	transcription regulator activity(GO:0140110)	1
OG0030555	Molecular Function	catalytic activity(GO:0003824)	1
OG0030578	Molecular Function	transporter activity(GO:0005215)	1
OG0030584	Molecular Function	binding(GO:0005488)	1
OG0030584	Molecular Function	molecular function regulator(GO:0098772)	1
OG0030586	Molecular Function	protein tag(GO:0031386)	1
OG0030587	Molecular Function	binding(GO:0005488)	1
OG0030587	Molecular Function	transcription regulator activity(GO:0140110)	1
OG0030590	Molecular Function	catalytic activity(GO:0003824)	1
OG0030593	Molecular Function	catalytic activity(GO:0003824)	1
OG0030594	Molecular Function	catalytic activity(GO:0003824)	1
OG0030596	Molecular Function	binding(GO:0005488)	1
OG0030596	Molecular Function	structural molecule activity(GO:0005198)	1
OG0030605	Molecular Function	transporter activity(GO:0005215)	1
OG0030621	Molecular Function	binding(GO:0005488)	1

OG0030621	Molecular Function	catalytic activity(GO:0003824)	1
OG0030623	Molecular Function	catalytic activity(GO:0003824)	1
OG0030624	Molecular Function	transcription regulator activity(GO:0140110)	1
OG0030625	Molecular Function	catalytic activity(GO:0003824)	1
OG0030630	Molecular Function	binding(GO:0005488)	1
OG0030630	Molecular Function	catalytic activity(GO:0003824)	1
OG0030642	Molecular Function	binding(GO:0005488)	1
OG0030642	Molecular Function	transcription regulator activity(GO:0140110)	1
OG0030642	Molecular Function	translation regulator activity(GO:0045182)	1
OG0030643	Molecular Function	transcription regulator activity(GO:0140110)	1
OG0030658	Molecular Function	transcription regulator activity(GO:0140110)	1
OG0030660	Molecular Function	binding(GO:0005488)	1
OG0030661	Molecular Function	binding(GO:0005488)	1
OG0030669	Molecular Function	catalytic activity(GO:0003824)	1
OG0030682	Molecular Function	catalytic activity(GO:0003824)	1
OG0030687	Molecular Function	binding(GO:0005488)	1
OG0030700	Molecular Function	catalytic activity(GO:0003824)	1
OG0030702	Molecular Function	catalytic activity(GO:0003824)	1
OG0030704	Molecular Function	binding(GO:0005488)	1
OG0030704	Molecular Function	catalytic activity(GO:0003824)	1
OG0030706	Molecular Function	binding(GO:0005488)	1
OG0030706	Molecular Function	catalytic activity(GO:0003824)	1
OG0030708	Molecular Function	binding(GO:0005488)	1
OG0030708	Molecular Function	catalytic activity(GO:0003824)	1
OG0030709	Molecular Function	transcription regulator activity(GO:0140110)	1
OG0030710	Molecular Function	transcription regulator activity(GO:0140110)	1
OG0030715	Molecular Function	transcription regulator activity(GO:0140110)	1
OG0030732	Molecular Function	catalytic activity(GO:0003824)	1
OG0030746	Molecular Function	catalytic activity(GO:0003824)	1
OG0030753	Molecular Function	catalytic activity(GO:0003824)	1
OG0030757	Molecular Function	catalytic activity(GO:0003824)	1
OG0030766	Molecular Function	binding(GO:0005488)	1
OG0030767	Molecular Function	binding(GO:0005488)	1
OG0030767	Molecular Function	transcription regulator activity(GO:0140110)	1
OG0030776	Molecular Function	transporter activity(GO:0005215)	1
OG0030778	Molecular Function	catalytic activity(GO:0003824)	1
OG0030815	Molecular Function	transcription regulator activity(GO:0140110)	1
OG0030816	Molecular Function	transcription regulator activity(GO:0140110)	1
OG0030818	Molecular Function	catalytic activity(GO:0003824)	1
OG0030818	Molecular Function	transporter activity(GO:0005215)	1
OG0030820	Molecular Function	binding(GO:0005488)	1
OG0030852	Molecular Function	binding(GO:0005488)	1
OG0030852	Molecular Function	structural molecule activity(GO:0005198)	1
OG0030853	Molecular Function	binding(GO:0005488)	1

OG0030856	Molecular Function	catalytic activity(GO:0003824)	1
OG0030859	Molecular Function	molecular carrier activity(GO:0140104)	1
OG0030861	Molecular Function	binding(GO:0005488)	1
OG0030861	Molecular Function	catalytic activity(GO:0003824)	1
OG0030864	Molecular Function	catalytic activity(GO:0003824)	1
OG0030868	Molecular Function	catalytic activity(GO:0003824)	1
OG0030869	Molecular Function	binding(GO:0005488)	1
OG0030869	Molecular Function	catalytic activity(GO:0003824)	1
OG0030879	Molecular Function	binding(GO:0005488)	1
OG0030879	Molecular Function	catalytic activity(GO:0003824)	1
OG0030882	Molecular Function	catalytic activity(GO:0003824)	1
OG0030884	Molecular Function	catalytic activity(GO:0003824)	1
OG0030887	Molecular Function	structural molecule activity(GO:0005198)	1
OG0030895	Molecular Function	binding(GO:0005488)	1
OG0030895	Molecular Function	catalytic activity(GO:0003824)	1
OG0030896	Molecular Function	binding(GO:0005488)	1
OG0030896	Molecular Function	catalytic activity(GO:0003824)	1
OG0030907	Molecular Function	catalytic activity(GO:0003824)	1
OG0030908	Molecular Function	catalytic activity(GO:0003824)	1
OG0030909	Molecular Function	catalytic activity(GO:0003824)	1
OG0030913	Molecular Function	catalytic activity(GO:0003824)	1
OG0030927	Molecular Function	catalytic activity(GO:0003824)	1
OG0030928	Molecular Function	catalytic activity(GO:0003824)	1
OG0030928	Molecular Function	transporter activity(GO:0005215)	1
OG0030929	Molecular Function	binding(GO:0005488)	1
OG0030929	Molecular Function	catalytic activity(GO:0003824)	1
OG0030931	Molecular Function	catalytic activity(GO:0003824)	1
OG0030936	Molecular Function	antioxidant activity(GO:0016209)	1
OG0030936	Molecular Function	catalytic activity(GO:0003824)	1
OG0030937	Molecular Function	binding(GO:0005488)	1
OG0030937	Molecular Function	catalytic activity(GO:0003824)	1
OG0030940	Molecular Function	catalytic activity(GO:0003824)	1
OG0030941	Molecular Function	catalytic activity(GO:0003824)	1
OG0030945	Molecular Function	catalytic activity(GO:0003824)	1
OG0030950	Molecular Function	binding(GO:0005488)	1
OG0030950	Molecular Function	catalytic activity(GO:0003824)	1
OG0030951	Molecular Function	catalytic activity(GO:0003824)	1
OG0030952	Molecular Function	catalytic activity(GO:0003824)	1
OG0030955	Molecular Function	catalytic activity(GO:0003824)	1
OG0030962	Molecular Function	catalytic activity(GO:0003824)	1
OG0030969	Molecular Function	catalytic activity(GO:0003824)	1
OG0030972	Molecular Function	catalytic activity(GO:0003824)	1
OG0030973	Molecular Function	binding(GO:0005488)	1
OG0030975	Molecular Function	binding(GO:0005488)	1
OG0030975	Molecular Function	catalytic activity(GO:0003824)	1
OG0030980	Molecular Function	catalytic activity(GO:0003824)	1
OG0030987	Molecular Function	binding(GO:0005488)	1

OG0031011	Molecular Function	catalytic activity(GO:0003824)	1
OG0031012	Molecular Function	catalytic activity(GO:0003824)	1
OG0031015	Molecular Function	binding(GO:0005488)	1
OG0031015	Molecular Function	catalytic activity(GO:0003824)	1
OG0031016	Molecular Function	catalytic activity(GO:0003824)	1
OG0031017	Molecular Function	binding(GO:0005488)	1
OG0031019	Molecular Function	binding(GO:0005488)	1
OG0031031	Molecular Function	catalytic activity(GO:0003824)	1
OG0031059	Molecular Function	transporter activity(GO:0005215)	1
OG0031060	Molecular Function	catalytic activity(GO:0003824)	1
OG0031063	Molecular Function	catalytic activity(GO:0003824)	1
OG0031080	Molecular Function	catalytic activity(GO:0003824)	1
OG0031080	Molecular Function	molecular function regulator(GO:0098772)	1
OG0031088	Molecular Function	catalytic activity(GO:0003824)	1
OG0031101	Molecular Function	binding(GO:0005488)	1
OG0031107	Molecular Function	transporter activity(GO:0005215)	1
OG0031120	Molecular Function	catalytic activity(GO:0003824)	1
OG0031144	Molecular Function	catalytic activity(GO:0003824)	1
OG0031145	Molecular Function	catalytic activity(GO:0003824)	1
OG0031148	Molecular Function	binding(GO:0005488)	1
OG0031148	Molecular Function	catalytic activity(GO:0003824)	1
OG0031149	Molecular Function	catalytic activity(GO:0003824)	1
OG0031152	Molecular Function	binding(GO:0005488)	1
OG0031152	Molecular Function	catalytic activity(GO:0003824)	1
OG0031155	Molecular Function	catalytic activity(GO:0003824)	1
OG0031157	Molecular Function	catalytic activity(GO:0003824)	1
OG0031172	Molecular Function	catalytic activity(GO:0003824)	1
OG0031172	Molecular Function	transporter activity(GO:0005215)	1
OG0031174	Molecular Function	binding(GO:0005488)	1
OG0031174	Molecular Function	catalytic activity(GO:0003824)	1
OG0031177	Molecular Function	catalytic activity(GO:0003824)	1
OG0031189	Molecular Function	binding(GO:0005488)	1
OG0031189	Molecular Function	catalytic activity(GO:0003824)	1
OG0031190	Molecular Function	binding(GO:0005488)	1
OG0031190	Molecular Function	catalytic activity(GO:0003824)	1
OG0031191	Molecular Function	binding(GO:0005488)	1
OG0031191	Molecular Function	catalytic activity(GO:0003824)	1
OG0031194	Molecular Function	catalytic activity(GO:0003824)	1
OG0031195	Molecular Function	binding(GO:0005488)	1
OG0031195	Molecular Function	catalytic activity(GO:0003824)	1
OG0031197	Molecular Function	binding(GO:0005488)	1
OG0031199	Molecular Function	binding(GO:0005488)	1
OG0031199	Molecular Function	catalytic activity(GO:0003824)	1
OG0031199	Molecular Function	transporter activity(GO:0005215)	1
OG0031200	Molecular Function	catalytic activity(GO:0003824)	1
OG0031204	Molecular Function	catalytic activity(GO:0003824)	1
OG0031208	Molecular Function	catalytic activity(GO:0003824)	1

OG0031209	Molecular Function	catalytic activity(GO:0003824)	1
OG0031212	Molecular Function	catalytic activity(GO:0003824)	1
OG0031212	Molecular Function	transporter activity(GO:0005215)	1
OG0031214	Molecular Function	binding(GO:0005488)	1
OG0031214	Molecular Function	catalytic activity(GO:0003824)	1
OG0031217	Molecular Function	antioxidant activity(GO:0016209)	1
OG0031217	Molecular Function	binding(GO:0005488)	1
OG0031217	Molecular Function	catalytic activity(GO:0003824)	1
OG0031223	Molecular Function	catalytic activity(GO:0003824)	1
OG0031223	Molecular Function	transporter activity(GO:0005215)	1
OG0031228	Molecular Function	binding(GO:0005488)	1
OG0031228	Molecular Function	catalytic activity(GO:0003824)	1
OG0031232	Molecular Function	catalytic activity(GO:0003824)	1
OG0031233	Molecular Function	binding(GO:0005488)	1
OG0031235	Molecular Function	binding(GO:0005488)	1
OG0031241	Molecular Function	binding(GO:0005488)	1
OG0031241	Molecular Function	catalytic activity(GO:0003824)	1
OG0031248	Molecular Function	catalytic activity(GO:0003824)	1
OG0031249	Molecular Function	binding(GO:0005488)	1
OG0031249	Molecular Function	catalytic activity(GO:0003824)	1
OG0031250	Molecular Function	catalytic activity(GO:0003824)	1
OG0031250	Molecular Function	transporter activity(GO:0005215)	1
OG0031254	Molecular Function	catalytic activity(GO:0003824)	1
OG0031256	Molecular Function	catalytic activity(GO:0003824)	1
OG0031258	Molecular Function	catalytic activity(GO:0003824)	1
OG0031259	Molecular Function	antioxidant activity(GO:0016209)	1
OG0031259	Molecular Function	catalytic activity(GO:0003824)	1
OG0031273	Molecular Function	structural molecule activity(GO:0005198)	1
OG0031277	Molecular Function	catalytic activity(GO:0003824)	1
OG0031282	Molecular Function	catalytic activity(GO:0003824)	1
OG0031292	Molecular Function	structural molecule activity(GO:0005198)	1
OG0031316	Molecular Function	binding(GO:0005488)	1
OG0031318	Molecular Function	transcription regulator activity(GO:0140110)	1
OG0031319	Molecular Function	catalytic activity(GO:0003824)	1
OG0031331	Molecular Function	binding(GO:0005488)	1
OG0031331	Molecular Function	structural molecule activity(GO:0005198)	1
OG0031337	Molecular Function	catalytic activity(GO:0003824)	1
OG0031340	Molecular Function	transcription regulator activity(GO:0140110)	1
OG0031349	Molecular Function	catalytic activity(GO:0003824)	1
OG0031352	Molecular Function	binding(GO:0005488)	1
OG0031356	Molecular Function	catalytic activity(GO:0003824)	1
OG0031377	Molecular Function	binding(GO:0005488)	1
OG0031377	Molecular Function	catalytic activity(GO:0003824)	1
OG0031380	Molecular Function	binding(GO:0005488)	1
OG0031382	Molecular Function	catalytic activity(GO:0003824)	1
OG0031383	Molecular Function	catalytic activity(GO:0003824)	1

OG0031384	Molecular Function	catalytic activity(GO:0003824)	1
OG0031386	Molecular Function	binding(GO:0005488)	1
OG0031386	Molecular Function	catalytic activity(GO:0003824)	1
OG0031396	Molecular Function	catalytic activity(GO:0003824)	1
OG0031401	Molecular Function	catalytic activity(GO:0003824)	1
OG0031402	Molecular Function	binding(GO:0005488)	1
OG0031402	Molecular Function	catalytic activity(GO:0003824)	1
OG0031415	Molecular Function	binding(GO:0005488)	1
OG0031415	Molecular Function	catalytic activity(GO:0003824)	1
OG0031416	Molecular Function	catalytic activity(GO:0003824)	1
OG0031419	Molecular Function	binding(GO:0005488)	1
OG0031421	Molecular Function	catalytic activity(GO:0003824)	1
OG0031423	Molecular Function	catalytic activity(GO:0003824)	1
OG0031425	Molecular Function	transcription regulator activity(GO:0140110)	1
OG0031437	Molecular Function	transcription regulator activity(GO:0140110)	1
OG0031442	Molecular Function	catalytic activity(GO:0003824)	1
OG0031448	Molecular Function	catalytic activity(GO:0003824)	1
OG0031460	Molecular Function	catalytic activity(GO:0003824)	1
OG0031460	Molecular Function	transporter activity(GO:0005215)	1
OG0031466	Molecular Function	catalytic activity(GO:0003824)	1
OG0031489	Molecular Function	catalytic activity(GO:0003824)	1
OG0031502	Molecular Function	transporter activity(GO:0005215)	1
OG0031503	Molecular Function	binding(GO:0005488)	1
OG0031503	Molecular Function	catalytic activity(GO:0003824)	1
OG0031505	Molecular Function	binding(GO:0005488)	1
OG0031524	Molecular Function	catalytic activity(GO:0003824)	1
OG0031526	Molecular Function	binding(GO:0005488)	1
OG0031528	Molecular Function	binding(GO:0005488)	1
OG0031532	Molecular Function	transcription regulator activity(GO:0140110)	1
OG0031551	Molecular Function	structural molecule activity(GO:0005198)	1
OG0031555	Molecular Function	transporter activity(GO:0005215)	1
OG0031557	Molecular Function	catalytic activity(GO:0003824)	1
OG0031560	Molecular Function	catalytic activity(GO:0003824)	1
OG0031564	Molecular Function	catalytic activity(GO:0003824)	1
OG0031565	Molecular Function	transcription regulator activity(GO:0140110)	1
OG0031588	Molecular Function	catalytic activity(GO:0003824)	1
OG0031595	Molecular Function	binding(GO:0005488)	1
OG0031602	Molecular Function	catalytic activity(GO:0003824)	1
OG0031607	Molecular Function	transporter activity(GO:0005215)	1
OG0031630	Molecular Function	catalytic activity(GO:0003824)	1
OG0031646	Molecular Function	transcription regulator activity(GO:0140110)	1
OG0031648	Molecular Function	catalytic activity(GO:0003824)	1
OG0031665	Molecular Function	catalytic activity(GO:0003824)	1
OG0031680	Molecular Function	binding(GO:0005488)	1
OG0031681	Molecular Function	binding(GO:0005488)	1
OG0031689	Molecular Function	transcription regulator activity(GO:0140110)	1

OG0031703	Molecular Function	catalytic activity(GO:0003824)	1
OG0031704	Molecular Function	catalytic activity(GO:0003824)	1
OG0031710	Molecular Function	binding(GO:0005488)	1
OG0031711	Molecular Function	binding(GO:0005488)	1
OG0031711	Molecular Function	catalytic activity(GO:0003824)	1
OG0031711	Molecular Function	transporter activity(GO:0005215)	1
OG0031718	Molecular Function	catalytic activity(GO:0003824)	1
OG0031722	Molecular Function	binding(GO:0005488)	1
OG0031722	Molecular Function	catalytic activity(GO:0003824)	1
OG0031724	Molecular Function	catalytic activity(GO:0003824)	1
OG0031724	Molecular Function	transporter activity(GO:0005215)	1
OG0031726	Molecular Function	catalytic activity(GO:0003824)	1
OG0031733	Molecular Function	catalytic activity(GO:0003824)	1
OG0031733	Molecular Function	transporter activity(GO:0005215)	1
OG0031736	Molecular Function	catalytic activity(GO:0003824)	1
OG0031736	Molecular Function	transporter activity(GO:0005215)	1
OG0031739	Molecular Function	catalytic activity(GO:0003824)	1
OG0031745	Molecular Function	binding(GO:0005488)	1
OG0031745	Molecular Function	catalytic activity(GO:0003824)	1
OG0031747	Molecular Function	catalytic activity(GO:0003824)	1
OG0031747	Molecular Function	transporter activity(GO:0005215)	1
OG0031750	Molecular Function	binding(GO:0005488)	1
OG0031750	Molecular Function	catalytic activity(GO:0003824)	1
OG0031757	Molecular Function	binding(GO:0005488)	1
OG0031757	Molecular Function	catalytic activity(GO:0003824)	1
OG0031759	Molecular Function	binding(GO:0005488)	1
OG0031759	Molecular Function	catalytic activity(GO:0003824)	1
OG0031762	Molecular Function	catalytic activity(GO:0003824)	1
OG0031776	Molecular Function	catalytic activity(GO:0003824)	1
OG0031777	Molecular Function	catalytic activity(GO:0003824)	1
OG0031780	Molecular Function	transporter activity(GO:0005215)	1
OG0031784	Molecular Function	structural molecule activity(GO:0005198)	1
OG0031784	Molecular Function	translation regulator activity(GO:0045182)	1
OG0031785	Molecular Function	catalytic activity(GO:0003824)	1
OG0031785	Molecular Function	molecular function regulator(GO:0098772)	1
OG0031787	Molecular Function	binding(GO:0005488)	1
OG0031787	Molecular Function	catalytic activity(GO:0003824)	1
OG0031787	Molecular Function	transporter activity(GO:0005215)	1
OG0031789	Molecular Function	binding(GO:0005488)	1
OG0031789	Molecular Function	catalytic activity(GO:0003824)	1
OG0031790	Molecular Function	binding(GO:0005488)	1
OG0031790	Molecular Function	catalytic activity(GO:0003824)	1
OG0031793	Molecular Function	binding(GO:0005488)	1
OG0031793	Molecular Function	molecular carrier activity(GO:0140104)	1
OG0031795	Molecular Function	binding(GO:0005488)	1
OG0031795	Molecular Function	catalytic activity(GO:0003824)	1

OG0031805	Molecular Function	transcription regulator activity(GO:0140110)	1
OG0031806	Molecular Function	catalytic activity(GO:0003824)	1
OG0031807	Molecular Function	catalytic activity(GO:0003824)	1
OG0031813	Molecular Function	binding(GO:0005488)	1
OG0031813	Molecular Function	catalytic activity(GO:0003824)	1
OG0031816	Molecular Function	binding(GO:0005488)	1
OG0031822	Molecular Function	catalytic activity(GO:0003824)	1
OG0031822	Molecular Function	transporter activity(GO:0005215)	1
OG0031827	Molecular Function	binding(GO:0005488)	1
OG0031842	Molecular Function	binding(GO:0005488)	1
OG0031842	Molecular Function	catalytic activity(GO:0003824)	1
OG0031843	Molecular Function	catalytic activity(GO:0003824)	1
OG0031844	Molecular Function	catalytic activity(GO:0003824)	1
OG0031849	Molecular Function	catalytic activity(GO:0003824)	1
OG0031851	Molecular Function	catalytic activity(GO:0003824)	1
OG0031855	Molecular Function	binding(GO:0005488)	1
OG0031855	Molecular Function	catalytic activity(GO:0003824)	1
OG0031862	Molecular Function	binding(GO:0005488)	1
OG0031862	Molecular Function	catalytic activity(GO:0003824)	1
OG0031863	Molecular Function	binding(GO:0005488)	1
OG0031863	Molecular Function	catalytic activity(GO:0003824)	1
OG0031864	Molecular Function	catalytic activity(GO:0003824)	1
OG0031864	Molecular Function	transporter activity(GO:0005215)	1
OG0031865	Molecular Function	catalytic activity(GO:0003824)	1
OG0031870	Molecular Function	binding(GO:0005488)	1
OG0031870	Molecular Function	catalytic activity(GO:0003824)	1
OG0031888	Molecular Function	catalytic activity(GO:0003824)	1
OG0031889	Molecular Function	catalytic activity(GO:0003824)	1
OG0031919	Molecular Function	binding(GO:0005488)	1
OG0031925	Molecular Function	catalytic activity(GO:0003824)	1
OG0031928	Molecular Function	catalytic activity(GO:0003824)	1
OG0031929	Molecular Function	catalytic activity(GO:0003824)	1
OG0031929	Molecular Function	molecular transducer activity(GO:0060089)	1
OG0031931	Molecular Function	catalytic activity(GO:0003824)	1
OG0031937	Molecular Function	catalytic activity(GO:0003824)	1
OG0031943	Molecular Function	transcription regulator activity(GO:0140110)	1
OG0031950	Molecular Function	binding(GO:0005488)	1
OG0031950	Molecular Function	catalytic activity(GO:0003824)	1
OG0031952	Molecular Function	catalytic activity(GO:0003824)	1
OG0031952	Molecular Function	transporter activity(GO:0005215)	1
OG0031953	Molecular Function	binding(GO:0005488)	1
OG0031953	Molecular Function	catalytic activity(GO:0003824)	1
OG0031958	Molecular Function	binding(GO:0005488)	1
OG0031958	Molecular Function	catalytic activity(GO:0003824)	1
OG0031959	Molecular Function	catalytic activity(GO:0003824)	1
OG0031960	Molecular Function	catalytic activity(GO:0003824)	1

OG0031962	Molecular Function	catalytic activity(GO:0003824)	1
OG0031967	Molecular Function	binding(GO:0005488)	1
OG0031971	Molecular Function	binding(GO:0005488)	1
OG0031972	Molecular Function	catalytic activity(GO:0003824)	1
OG0032001	Molecular Function	catalytic activity(GO:0003824)	1
OG0032021	Molecular Function	catalytic activity(GO:0003824)	1
OG0032031	Molecular Function	catalytic activity(GO:0003824)	1
OG0032036	Molecular Function	catalytic activity(GO:0003824)	1
OG0032044	Molecular Function	catalytic activity(GO:0003824)	1
OG0032045	Molecular Function	catalytic activity(GO:0003824)	1
OG0032046	Molecular Function	catalytic activity(GO:0003824)	1
OG0032050	Molecular Function	binding(GO:0005488)	1
OG0032050	Molecular Function	catalytic activity(GO:0003824)	1
OG0032051	Molecular Function	catalytic activity(GO:0003824)	1
OG0032070	Molecular Function	catalytic activity(GO:0003824)	1
OG0032073	Molecular Function	catalytic activity(GO:0003824)	1
OG0032074	Molecular Function	catalytic activity(GO:0003824)	1
OG0032092	Molecular Function	catalytic activity(GO:0003824)	1
OG0032095	Molecular Function	catalytic activity(GO:0003824)	1
OG0032095	Molecular Function	molecular transducer activity(GO:0060089)	1
OG0032096	Molecular Function	transcription regulator activity(GO:0140110)	1
OG0032101	Molecular Function	catalytic activity(GO:0003824)	1
OG0032126	Molecular Function	transcription regulator activity(GO:0140110)	1
OG0032134	Molecular Function	binding(GO:0005488)	1
OG0032142	Molecular Function	catalytic activity(GO:0003824)	1
OG0032143	Molecular Function	structural molecule activity(GO:0005198)	1
OG0032148	Molecular Function	transcription regulator activity(GO:0140110)	1
OG0032151	Molecular Function	binding(GO:0005488)	1
OG0032159	Molecular Function	catalytic activity(GO:0003824)	1
OG0032161	Molecular Function	binding(GO:0005488)	1
OG0032170	Molecular Function	binding(GO:0005488)	1
OG0032180	Molecular Function	binding(GO:0005488)	1
OG0032183	Molecular Function	catalytic activity(GO:0003824)	1
OG0032185	Molecular Function	catalytic activity(GO:0003824)	1
OG0032186	Molecular Function	binding(GO:0005488)	1
OG0032186	Molecular Function	catalytic activity(GO:0003824)	1
OG0032191	Molecular Function	catalytic activity(GO:0003824)	1
OG0032191	Molecular Function	transporter activity(GO:0005215)	1
OG0032196	Molecular Function	catalytic activity(GO:0003824)	1
OG0032218	Molecular Function	binding(GO:0005488)	1
OG0032220	Molecular Function	transcription regulator activity(GO:0140110)	1
OG0032222	Molecular Function	binding(GO:0005488)	1
OG0032223	Molecular Function	transporter activity(GO:0005215)	1
OG0032224	Molecular Function	binding(GO:0005488)	1
OG0032224	Molecular Function	transcription regulator activity(GO:0140110)	1

OG0032224	Molecular Function	translation regulator activity(GO:0045182)	1
OG0032230	Molecular Function	binding(GO:0005488)	1
OG0032230	Molecular Function	transcription regulator activity(GO:0140110)	1
OG0032232	Molecular Function	catalytic activity(GO:0003824)	1
OG0032232	Molecular Function	molecular function regulator(GO:0098772)	1
OG0032234	Molecular Function	catalytic activity(GO:0003824)	1
OG0032234	Molecular Function	molecular function regulator(GO:0098772)	1
OG0032236	Molecular Function	binding(GO:0005488)	1
OG0032236	Molecular Function	structural molecule activity(GO:0005198)	1
OG0032236	Molecular Function	transcription regulator activity(GO:0140110)	1
OG0032245	Molecular Function	catalytic activity(GO:0003824)	1
OG0032252	Molecular Function	binding(GO:0005488)	1
OG0032252	Molecular Function	transcription regulator activity(GO:0140110)	1
OG0032254	Molecular Function	catalytic activity(GO:0003824)	1
OG0032262	Molecular Function	catalytic activity(GO:0003824)	1
OG0032264	Molecular Function	catalytic activity(GO:0003824)	1
OG0032273	Molecular Function	catalytic activity(GO:0003824)	1
OG0032278	Molecular Function	binding(GO:0005488)	1
OG0032278	Molecular Function	transcription regulator activity(GO:0140110)	1
OG0032289	Molecular Function	catalytic activity(GO:0003824)	1
OG0032290	Molecular Function	catalytic activity(GO:0003824)	1
OG0032297	Molecular Function	binding(GO:0005488)	1
OG0032297	Molecular Function	catalytic activity(GO:0003824)	1
OG0032315	Molecular Function	transcription regulator activity(GO:0140110)	1
OG0032321	Molecular Function	binding(GO:0005488)	1
OG0032328	Molecular Function	binding(GO:0005488)	1
OG0032342	Molecular Function	binding(GO:0005488)	1
OG0032342	Molecular Function	catalytic activity(GO:0003824)	1
OG0032342	Molecular Function	molecular function regulator(GO:0098772)	1
OG0032345	Molecular Function	binding(GO:0005488)	1
OG0032350	Molecular Function	molecular function regulator(GO:0098772)	1
OG0032372	Molecular Function	transporter activity(GO:0005215)	1
OG0032384	Molecular Function	catalytic activity(GO:0003824)	1
OG0032388	Molecular Function	catalytic activity(GO:0003824)	1
OG0032403	Molecular Function	binding(GO:0005488)	1
OG0032403	Molecular Function	structural molecule activity(GO:0005198)	1
OG0032408	Molecular Function	catalytic activity(GO:0003824)	1
OG0032413	Molecular Function	catalytic activity(GO:0003824)	1
OG0032414	Molecular Function	catalytic activity(GO:0003824)	1
OG0032424	Molecular Function	binding(GO:0005488)	1
OG0032426	Molecular Function	catalytic activity(GO:0003824)	1
OG0032431	Molecular Function	catalytic activity(GO:0003824)	1
OG0032445	Molecular Function	binding(GO:0005488)	1
OG0032447	Molecular Function	structural molecule activity(GO:0005198)	1

OG0032449	Molecular Function	catalytic activity(GO:0003824)	1
OG0032452	Molecular Function	catalytic activity(GO:0003824)	1
OG0032452	Molecular Function	transporter activity(GO:0005215)	1
OG0032453	Molecular Function	binding(GO:0005488)	1
OG0032453	Molecular Function	catalytic activity(GO:0003824)	1
OG0032464	Molecular Function	binding(GO:0005488)	1
OG0032464	Molecular Function	catalytic activity(GO:0003824)	1
OG0032464	Molecular Function	molecular transducer activity(GO:0060089)	1
OG0032466	Molecular Function	catalytic activity(GO:0003824)	1
OG0032473	Molecular Function	binding(GO:0005488)	1
OG0032473	Molecular Function	transcription regulator activity(GO:0140110)	1
OG0032474	Molecular Function	catalytic activity(GO:0003824)	1
OG0032491	Molecular Function	binding(GO:0005488)	1
OG0032491	Molecular Function	catalytic activity(GO:0003824)	1
OG0032492	Molecular Function	catalytic activity(GO:0003824)	1
OG0032495	Molecular Function	binding(GO:0005488)	1
OG0032498	Molecular Function	catalytic activity(GO:0003824)	1
OG0032501	Molecular Function	binding(GO:0005488)	1
OG0032501	Molecular Function	catalytic activity(GO:0003824)	1
OG0032506	Molecular Function	structural molecule activity(GO:0005198)	1
OG0032507	Molecular Function	catalytic activity(GO:0003824)	1
OG0032530	Molecular Function	catalytic activity(GO:0003824)	1
OG0032530	Molecular Function	transporter activity(GO:0005215)	1
OG0032537	Molecular Function	binding(GO:0005488)	1
OG0032551	Molecular Function	structural molecule activity(GO:0005198)	1
OG0032561	Molecular Function	catalytic activity(GO:0003824)	1
OG0032562	Molecular Function	catalytic activity(GO:0003824)	1
OG0032626	Molecular Function	binding(GO:0005488)	1
OG0032627	Molecular Function	binding(GO:0005488)	1
OG0032627	Molecular Function	catalytic activity(GO:0003824)	1
OG0032638	Molecular Function	catalytic activity(GO:0003824)	1
OG0032656	Molecular Function	catalytic activity(GO:0003824)	1
OG0032662	Molecular Function	catalytic activity(GO:0003824)	1
OG0032666	Molecular Function	binding(GO:0005488)	1
OG0032671	Molecular Function	catalytic activity(GO:0003824)	1
OG0032673	Molecular Function	catalytic activity(GO:0003824)	1
OG0032674	Molecular Function	catalytic activity(GO:0003824)	1
OG0032693	Molecular Function	catalytic activity(GO:0003824)	1
OG0032694	Molecular Function	catalytic activity(GO:0003824)	1
OG0032700	Molecular Function	catalytic activity(GO:0003824)	1
OG0032701	Molecular Function	catalytic activity(GO:0003824)	1
OG0032701	Molecular Function	transporter activity(GO:0005215)	1
OG0032702	Molecular Function	catalytic activity(GO:0003824)	1
OG0032702	Molecular Function	transporter activity(GO:0005215)	1
OG0032704	Molecular Function	catalytic activity(GO:0003824)	1
OG0032709	Molecular Function	catalytic activity(GO:0003824)	1

OG0032710	Molecular Function	binding(GO:0005488)	1
OG0032710	Molecular Function	catalytic activity(GO:0003824)	1
OG0032711	Molecular Function	catalytic activity(GO:0003824)	1
OG0032714	Molecular Function	binding(GO:0005488)	1
OG0032719	Molecular Function	catalytic activity(GO:0003824)	1
OG0032722	Molecular Function	catalytic activity(GO:0003824)	1
OG0032725	Molecular Function	catalytic activity(GO:0003824)	1
OG0032727	Molecular Function	catalytic activity(GO:0003824)	1
OG0032745	Molecular Function	binding(GO:0005488)	1
OG0032745	Molecular Function	catalytic activity(GO:0003824)	1
OG0032751	Molecular Function	binding(GO:0005488)	1
OG0032751	Molecular Function	catalytic activity(GO:0003824)	1
OG0032757	Molecular Function	binding(GO:0005488)	1
OG0032759	Molecular Function	binding(GO:0005488)	1
OG0032759	Molecular Function	catalytic activity(GO:0003824)	1
OG0032766	Molecular Function	binding(GO:0005488)	1
OG0032779	Molecular Function	transcription regulator activity(GO:0140110)	1
OG0032780	Molecular Function	transcription regulator activity(GO:0140110)	1
OG0032794	Molecular Function	binding(GO:0005488)	1
OG0032794	Molecular Function	catalytic activity(GO:0003824)	1
OG0032802	Molecular Function	binding(GO:0005488)	1
OG0032802	Molecular Function	catalytic activity(GO:0003824)	1
OG0032803	Molecular Function	antioxidant activity(GO:0016209)	1
OG0032803	Molecular Function	catalytic activity(GO:0003824)	1
OG0032806	Molecular Function	binding(GO:0005488)	1
OG0032806	Molecular Function	catalytic activity(GO:0003824)	1
OG0032807	Molecular Function	antioxidant activity(GO:0016209)	1
OG0032807	Molecular Function	catalytic activity(GO:0003824)	1
OG0032808	Molecular Function	binding(GO:0005488)	1
OG0032808	Molecular Function	catalytic activity(GO:0003824)	1
OG0032819	Molecular Function	catalytic activity(GO:0003824)	1
OG0032822	Molecular Function	catalytic activity(GO:0003824)	1
OG0032824	Molecular Function	binding(GO:0005488)	1
OG0032824	Molecular Function	transcription regulator activity(GO:0140110)	1
OG0032829	Molecular Function	catalytic activity(GO:0003824)	1
OG0032830	Molecular Function	catalytic activity(GO:0003824)	1
OG0032850	Molecular Function	binding(GO:0005488)	1
OG0032852	Molecular Function	transcription regulator activity(GO:0140110)	1
OG0032857	Molecular Function	catalytic activity(GO:0003824)	1
OG0032863	Molecular Function	binding(GO:0005488)	1
OG0032868	Molecular Function	binding(GO:0005488)	1
OG0032868	Molecular Function	transcription regulator activity(GO:0140110)	1
OG0032881	Molecular Function	binding(GO:0005488)	1
OG0032887	Molecular Function	antioxidant activity(GO:0016209)	1
OG0032887	Molecular Function	catalytic activity(GO:0003824)	1
OG0032889	Molecular Function	transporter activity(GO:0005215)	1

OG0032891	Molecular Function	catalytic activity(GO:0003824)	1
OG0032896	Molecular Function	binding(GO:0005488)	1
OG0032896	Molecular Function	catalytic activity(GO:0003824)	1
OG0032909	Molecular Function	catalytic activity(GO:0003824)	1
OG0032910	Molecular Function	transporter activity(GO:0005215)	1
OG0032920	Molecular Function	transcription regulator activity(GO:0140110)	1
OG0032922	Molecular Function	transcription regulator activity(GO:0140110)	1
OG0032926	Molecular Function	binding(GO:0005488)	1
OG0032928	Molecular Function	catalytic activity(GO:0003824)	1
OG0032932	Molecular Function	binding(GO:0005488)	1
OG0032942	Molecular Function	binding(GO:0005488)	1
OG0032949	Molecular Function	structural molecule activity(GO:0005198)	1
OG0032950	Molecular Function	structural molecule activity(GO:0005198)	1
OG0032956	Molecular Function	catalytic activity(GO:0003824)	1
OG0032970	Molecular Function	catalytic activity(GO:0003824)	1
OG0032974	Molecular Function	binding(GO:0005488)	1
OG0032974	Molecular Function	catalytic activity(GO:0003824)	1
OG0032975	Molecular Function	catalytic activity(GO:0003824)	1
OG0032983	Molecular Function	transcription regulator activity(GO:0140110)	1
OG0032986	Molecular Function	binding(GO:0005488)	1
OG0032986	Molecular Function	catalytic activity(GO:0003824)	1
OG0032986	Molecular Function	molecular function regulator(GO:0098772)	1
OG0033002	Molecular Function	catalytic activity(GO:0003824)	1
OG0033002	Molecular Function	transporter activity(GO:0005215)	1
OG0033003	Molecular Function	catalytic activity(GO:0003824)	1
OG0033006	Molecular Function	catalytic activity(GO:0003824)	1
OG0033014	Molecular Function	binding(GO:0005488)	1
OG0033016	Molecular Function	catalytic activity(GO:0003824)	1
OG0033017	Molecular Function	catalytic activity(GO:0003824)	1
OG0033018	Molecular Function	transcription regulator activity(GO:0140110)	1
OG0033019	Molecular Function	binding(GO:0005488)	1
OG0033019	Molecular Function	catalytic activity(GO:0003824)	1
OG0033021	Molecular Function	catalytic activity(GO:0003824)	1
OG0033022	Molecular Function	catalytic activity(GO:0003824)	1
OG0033024	Molecular Function	catalytic activity(GO:0003824)	1
OG0033026	Molecular Function	catalytic activity(GO:0003824)	1
OG0033027	Molecular Function	catalytic activity(GO:0003824)	1
OG0033028	Molecular Function	catalytic activity(GO:0003824)	1
OG0033031	Molecular Function	catalytic activity(GO:0003824)	1
OG0033034	Molecular Function	catalytic activity(GO:0003824)	1
OG0033037	Molecular Function	catalytic activity(GO:0003824)	1
OG0033040	Molecular Function	catalytic activity(GO:0003824)	1
OG0033042	Molecular Function	catalytic activity(GO:0003824)	1
OG0033042	Molecular Function	transporter activity(GO:0005215)	1
OG0033048	Molecular Function	catalytic activity(GO:0003824)	1
OG0033051	Molecular Function	binding(GO:0005488)	1

OG0033051	Molecular Function	catalytic activity(GO:0003824)	1
OG0033052	Molecular Function	catalytic activity(GO:0003824)	1
OG0033053	Molecular Function	catalytic activity(GO:0003824)	1
OG0033054	Molecular Function	catalytic activity(GO:0003824)	1
OG0033055	Molecular Function	catalytic activity(GO:0003824)	1
OG0033062	Molecular Function	binding(GO:0005488)	1
OG0033062	Molecular Function	catalytic activity(GO:0003824)	1
OG0033068	Molecular Function	binding(GO:0005488)	1
OG0033069	Molecular Function	catalytic activity(GO:0003824)	1
OG0033070	Molecular Function	binding(GO:0005488)	1
OG0033070	Molecular Function	catalytic activity(GO:0003824)	1
OG0033085	Molecular Function	binding(GO:0005488)	1
OG0033095	Molecular Function	binding(GO:0005488)	1
OG0033095	Molecular Function	transcription regulator activity(GO:0140110)	1
OG0033095	Molecular Function	translation regulator activity(GO:0045182)	1
OG0033101	Molecular Function	binding(GO:0005488)	1
OG0033101	Molecular Function	catalytic activity(GO:0003824)	1
OG0033105	Molecular Function	catalytic activity(GO:0003824)	1
OG0033109	Molecular Function	structural molecule activity(GO:0005198)	1
OG0033123	Molecular Function	transcription regulator activity(GO:0140110)	1
OG0033124	Molecular Function	catalytic activity(GO:0003824)	1
OG0033128	Molecular Function	catalytic activity(GO:0003824)	1
OG0033132	Molecular Function	binding(GO:0005488)	1
OG0033136	Molecular Function	binding(GO:0005488)	1
OG0033136	Molecular Function	catalytic activity(GO:0003824)	1
OG0033141	Molecular Function	binding(GO:0005488)	1
OG0033145	Molecular Function	catalytic activity(GO:0003824)	1
OG0033147	Molecular Function	binding(GO:0005488)	1
OG0033147	Molecular Function	catalytic activity(GO:0003824)	1
OG0033153	Molecular Function	catalytic activity(GO:0003824)	1
OG0033155	Molecular Function	binding(GO:0005488)	1
OG0033155	Molecular Function	catalytic activity(GO:0003824)	1
OG0033157	Molecular Function	transporter activity(GO:0005215)	1
OG0033184	Molecular Function	catalytic activity(GO:0003824)	1
OG0033226	Molecular Function	structural molecule activity(GO:0005198)	1
OG0033243	Molecular Function	catalytic activity(GO:0003824)	1
OG0033247	Molecular Function	binding(GO:0005488)	1
OG0033252	Molecular Function	binding(GO:0005488)	1
OG0033267	Molecular Function	transcription regulator activity(GO:0140110)	1
OG0033268	Molecular Function	catalytic activity(GO:0003824)	1
OG0033291	Molecular Function	catalytic activity(GO:0003824)	1
OG0033294	Molecular Function	catalytic activity(GO:0003824)	1
OG0033299	Molecular Function	transcription regulator activity(GO:0140110)	1
OG0033300	Molecular Function	binding(GO:0005488)	1
OG0033303	Molecular Function	binding(GO:0005488)	1

OG0033303	Molecular Function	transcription regulator activity(GO:0140110)	1
OG0033307	Molecular Function	binding(GO:0005488)	1
OG0033307	Molecular Function	catalytic activity(GO:0003824)	1
OG0033308	Molecular Function	catalytic activity(GO:0003824)	1
OG0033310	Molecular Function	transporter activity(GO:0005215)	1
OG0033311	Molecular Function	binding(GO:0005488)	1
OG0033311	Molecular Function	molecular function regulator(GO:0098772)	1
OG0033324	Molecular Function	catalytic activity(GO:0003824)	1
OG0033327	Molecular Function	catalytic activity(GO:0003824)	1
OG0033332	Molecular Function	transcription regulator activity(GO:0140110)	1
OG0033335	Molecular Function	binding(GO:0005488)	1
OG0033335	Molecular Function	catalytic activity(GO:0003824)	1
OG0033341	Molecular Function	catalytic activity(GO:0003824)	1
OG0033343	Molecular Function	transcription regulator activity(GO:0140110)	1
OG0033344	Molecular Function	catalytic activity(GO:0003824)	1
OG0033347	Molecular Function	catalytic activity(GO:0003824)	1
OG0033348	Molecular Function	binding(GO:0005488)	1
OG0033354	Molecular Function	catalytic activity(GO:0003824)	1
OG0033355	Molecular Function	catalytic activity(GO:0003824)	1
OG0033359	Molecular Function	binding(GO:0005488)	1
OG0033361	Molecular Function	catalytic activity(GO:0003824)	1
OG0033388	Molecular Function	catalytic activity(GO:0003824)	1
OG0033388	Molecular Function	transporter activity(GO:0005215)	1
OG0033413	Molecular Function	binding(GO:0005488)	1
OG0033413	Molecular Function	catalytic activity(GO:0003824)	1
OG0033414	Molecular Function	catalytic activity(GO:0003824)	1
OG0033414	Molecular Function	transporter activity(GO:0005215)	1
OG0033419	Molecular Function	catalytic activity(GO:0003824)	1
OG0033422	Molecular Function	catalytic activity(GO:0003824)	1
OG0033428	Molecular Function	binding(GO:0005488)	1
OG0033428	Molecular Function	catalytic activity(GO:0003824)	1
OG0033428	Molecular Function	molecular carrier activity(GO:0140104)	1
OG0033428	Molecular Function	transporter activity(GO:0005215)	1
OG0033429	Molecular Function	catalytic activity(GO:0003824)	1
OG0033433	Molecular Function	catalytic activity(GO:0003824)	1
OG0033435	Molecular Function	catalytic activity(GO:0003824)	1
OG0033441	Molecular Function	catalytic activity(GO:0003824)	1
OG0033444	Molecular Function	catalytic activity(GO:0003824)	1
OG0033446	Molecular Function	binding(GO:0005488)	1
OG0033453	Molecular Function	catalytic activity(GO:0003824)	1
OG0033458	Molecular Function	binding(GO:0005488)	1
OG0033458	Molecular Function	catalytic activity(GO:0003824)	1
OG0033459	Molecular Function	binding(GO:0005488)	1
OG0033459	Molecular Function	catalytic activity(GO:0003824)	1
OG0033460	Molecular Function	binding(GO:0005488)	1
OG0033460	Molecular Function	catalytic activity(GO:0003824)	1

OG0033462	Molecular Function	catalytic activity(GO:0003824)	1
OG0033463	Molecular Function	binding(GO:0005488)	1
OG0033463	Molecular Function	catalytic activity(GO:0003824)	1
OG0033464	Molecular Function	catalytic activity(GO:0003824)	1
OG0033469	Molecular Function	catalytic activity(GO:0003824)	1
OG0033470	Molecular Function	catalytic activity(GO:0003824)	1
OG0033484	Molecular Function	catalytic activity(GO:0003824)	1
OG0033489	Molecular Function	catalytic activity(GO:0003824)	1
OG0033490	Molecular Function	catalytic activity(GO:0003824)	1
OG0033490	Molecular Function	transporter activity(GO:0005215)	1
OG0033491	Molecular Function	binding(GO:0005488)	1
OG0033504	Molecular Function	catalytic activity(GO:0003824)	1
OG0033510	Molecular Function	catalytic activity(GO:0003824)	1
OG0033514	Molecular Function	catalytic activity(GO:0003824)	1
OG0033517	Molecular Function	binding(GO:0005488)	1
OG0033517	Molecular Function	catalytic activity(GO:0003824)	1
OG0033518	Molecular Function	catalytic activity(GO:0003824)	1
OG0033520	Molecular Function	catalytic activity(GO:0003824)	1
OG0033521	Molecular Function	catalytic activity(GO:0003824)	1
OG0033523	Molecular Function	binding(GO:0005488)	1
OG0033523	Molecular Function	catalytic activity(GO:0003824)	1
OG0033533	Molecular Function	catalytic activity(GO:0003824)	1
OG0033534	Molecular Function	catalytic activity(GO:0003824)	1
OG0033552	Molecular Function	transcription regulator activity(GO:0140110)	1
OG0033554	Molecular Function	catalytic activity(GO:0003824)	1
OG0033556	Molecular Function	catalytic activity(GO:0003824)	1
OG0033571	Molecular Function	binding(GO:0005488)	1
OG0033571	Molecular Function	catalytic activity(GO:0003824)	1
OG0033582	Molecular Function	binding(GO:0005488)	1
OG0033582	Molecular Function	transcription regulator activity(GO:0140110)	1
OG0033599	Molecular Function	transcription regulator activity(GO:0140110)	1
OG0033604	Molecular Function	transcription regulator activity(GO:0140110)	1
OG0033618	Molecular Function	transcription regulator activity(GO:0140110)	1
OG0033624	Molecular Function	catalytic activity(GO:0003824)	1
OG0033625	Molecular Function	catalytic activity(GO:0003824)	1
OG0033650	Molecular Function	structural molecule activity(GO:0005198)	1
OG0033655	Molecular Function	binding(GO:0005488)	1
OG0033657	Molecular Function	antioxidant activity(GO:0016209)	1
OG0033657	Molecular Function	catalytic activity(GO:0003824)	1
OG0033657	Molecular Function	molecular function regulator(GO:0098772)	1
OG0033661	Molecular Function	catalytic activity(GO:0003824)	1
OG0033667	Molecular Function	binding(GO:0005488)	1
OG0033667	Molecular Function	molecular function regulator(GO:0098772)	1
OG0033673	Molecular Function	binding(GO:0005488)	1
OG0033673	Molecular Function	catalytic activity(GO:0003824)	1

OG0033675	Molecular Function	binding(GO:0005488)	1
OG0033678	Molecular Function	catalytic activity(GO:0003824)	1
OG0033680	Molecular Function	binding(GO:0005488)	1
OG0033680	Molecular Function	catalytic activity(GO:0003824)	1
OG0033699	Molecular Function	catalytic activity(GO:0003824)	1
OG0033703	Molecular Function	catalytic activity(GO:0003824)	1
OG0033715	Molecular Function	transporter activity(GO:0005215)	1
OG0033716	Molecular Function	binding(GO:0005488)	1
OG0033716	Molecular Function	catalytic activity(GO:0003824)	1
OG0033716	Molecular Function	molecular function regulator(GO:0098772)	1
OG0033716	Molecular Function	molecular transducer activity(GO:0060089)	1
OG0033725	Molecular Function	binding(GO:0005488)	1
OG0033726	Molecular Function	catalytic activity(GO:0003824)	1
OG0033727	Molecular Function	catalytic activity(GO:0003824)	1
OG0033728	Molecular Function	catalytic activity(GO:0003824)	1
OG0033733	Molecular Function	catalytic activity(GO:0003824)	1
OG0033738	Molecular Function	catalytic activity(GO:0003824)	1
OG0033740	Molecular Function	catalytic activity(GO:0003824)	1
OG0033741	Molecular Function	catalytic activity(GO:0003824)	1
OG0033749	Molecular Function	catalytic activity(GO:0003824)	1
OG0033761	Molecular Function	catalytic activity(GO:0003824)	1
OG0033764	Molecular Function	catalytic activity(GO:0003824)	1
OG0033768	Molecular Function	catalytic activity(GO:0003824)	1
OG0033769	Molecular Function	catalytic activity(GO:0003824)	1
OG0033772	Molecular Function	transcription regulator activity(GO:0140110)	1
OG0033782	Molecular Function	binding(GO:0005488)	1
OG0033782	Molecular Function	structural molecule activity(GO:0005198)	1
OG0033783	Molecular Function	transcription regulator activity(GO:0140110)	1
OG0033790	Molecular Function	catalytic activity(GO:0003824)	1
OG0033790	Molecular Function	transporter activity(GO:0005215)	1
OG0033791	Molecular Function	catalytic activity(GO:0003824)	1
OG0033792	Molecular Function	catalytic activity(GO:0003824)	1
OG0033803	Molecular Function	catalytic activity(GO:0003824)	1
OG0033804	Molecular Function	catalytic activity(GO:0003824)	1
OG0033810	Molecular Function	catalytic activity(GO:0003824)	1
OG0033826	Molecular Function	binding(GO:0005488)	1
OG0033826	Molecular Function	catalytic activity(GO:0003824)	1
OG0033856	Molecular Function	molecular transducer activity(GO:0060089)	1
OG0033856	Molecular Function	transporter activity(GO:0005215)	1
OG0033865	Molecular Function	catalytic activity(GO:0003824)	1
OG0033875	Molecular Function	binding(GO:0005488)	1
OG0033875	Molecular Function	catalytic activity(GO:0003824)	1
OG0033877	Molecular Function	catalytic activity(GO:0003824)	1
OG0033910	Molecular Function	transcription regulator activity(GO:0140110)	1
OG0033918	Molecular Function	catalytic activity(GO:0003824)	1
OG0033933	Molecular Function	catalytic activity(GO:0003824)	1

OG0033944	Molecular Function	binding(GO:0005488)	1
OG0033944	Molecular Function	transcription regulator activity(GO:0140110)	1
OG0033947	Molecular Function	molecular transducer activity(GO:0060089)	1
OG0033956	Molecular Function	binding(GO:0005488)	1
OG0033956	Molecular Function	catalytic activity(GO:0003824)	1
OG0033957	Molecular Function	catalytic activity(GO:0003824)	1
OG0033979	Molecular Function	catalytic activity(GO:0003824)	1
OG0033989	Molecular Function	catalytic activity(GO:0003824)	1
OG0033991	Molecular Function	binding(GO:0005488)	1
OG0034004	Molecular Function	molecular transducer activity(GO:0060089)	1
OG0034030	Molecular Function	catalytic activity(GO:0003824)	1
OG0034045	Molecular Function	catalytic activity(GO:0003824)	1
OG0034055	Molecular Function	transcription regulator activity(GO:0140110)	1
OG0034056	Molecular Function	binding(GO:0005488)	1
OG0034056	Molecular Function	transcription regulator activity(GO:0140110)	1
OG0034069	Molecular Function	catalytic activity(GO:0003824)	1
OG0034072	Molecular Function	catalytic activity(GO:0003824)	1
OG0034076	Molecular Function	catalytic activity(GO:0003824)	1
OG0034078	Molecular Function	binding(GO:0005488)	1
OG0034078	Molecular Function	catalytic activity(GO:0003824)	1
OG0034079	Molecular Function	binding(GO:0005488)	1
OG0034081	Molecular Function	binding(GO:0005488)	1
OG0034081	Molecular Function	catalytic activity(GO:0003824)	1
OG0034090	Molecular Function	binding(GO:0005488)	1
OG0034090	Molecular Function	catalytic activity(GO:0003824)	1
OG0034091	Molecular Function	binding(GO:0005488)	1
OG0034092	Molecular Function	binding(GO:0005488)	1
OG0034093	Molecular Function	catalytic activity(GO:0003824)	1
OG0034096	Molecular Function	binding(GO:0005488)	1
OG0034096	Molecular Function	catalytic activity(GO:0003824)	1
OG0034097	Molecular Function	binding(GO:0005488)	1
OG0034097	Molecular Function	catalytic activity(GO:0003824)	1
OG0034098	Molecular Function	catalytic activity(GO:0003824)	1
OG0034100	Molecular Function	binding(GO:0005488)	1
OG0034100	Molecular Function	catalytic activity(GO:0003824)	1
OG0034101	Molecular Function	binding(GO:0005488)	1
OG0034101	Molecular Function	catalytic activity(GO:0003824)	1
OG0034102	Molecular Function	binding(GO:0005488)	1
OG0034102	Molecular Function	catalytic activity(GO:0003824)	1
OG0034103	Molecular Function	catalytic activity(GO:0003824)	1
OG0034105	Molecular Function	binding(GO:0005488)	1
OG0034105	Molecular Function	catalytic activity(GO:0003824)	1
OG0034108	Molecular Function	catalytic activity(GO:0003824)	1
OG0034113	Molecular Function	binding(GO:0005488)	1
OG0034113	Molecular Function	catalytic activity(GO:0003824)	1
OG0034114	Molecular Function	catalytic activity(GO:0003824)	1

OG0034115	Molecular Function	binding(GO:0005488)	1
OG0034115	Molecular Function	catalytic activity(GO:0003824)	1
OG0034118	Molecular Function	binding(GO:0005488)	1
OG0034118	Molecular Function	catalytic activity(GO:0003824)	1
OG0034123	Molecular Function	catalytic activity(GO:0003824)	1
OG0034131	Molecular Function	catalytic activity(GO:0003824)	1
OG0034136	Molecular Function	catalytic activity(GO:0003824)	1
OG0034137	Molecular Function	catalytic activity(GO:0003824)	1
OG0034141	Molecular Function	binding(GO:0005488)	1
OG0034141	Molecular Function	catalytic activity(GO:0003824)	1
OG0034142	Molecular Function	binding(GO:0005488)	1
OG0034143	Molecular Function	catalytic activity(GO:0003824)	1
OG0034144	Molecular Function	catalytic activity(GO:0003824)	1
OG0034147	Molecular Function	catalytic activity(GO:0003824)	1
OG0034153	Molecular Function	catalytic activity(GO:0003824)	1
OG0034155	Molecular Function	binding(GO:0005488)	1
OG0034155	Molecular Function	catalytic activity(GO:0003824)	1
OG0034155	Molecular Function	transcription regulator activity(GO:0140110)	1
OG0034157	Molecular Function	catalytic activity(GO:0003824)	1
OG0034157	Molecular Function	transporter activity(GO:0005215)	1
OG0034158	Molecular Function	catalytic activity(GO:0003824)	1
OG0034158	Molecular Function	transporter activity(GO:0005215)	1
OG0034159	Molecular Function	binding(GO:0005488)	1
OG0034159	Molecular Function	catalytic activity(GO:0003824)	1
OG0034161	Molecular Function	catalytic activity(GO:0003824)	1
OG0034162	Molecular Function	catalytic activity(GO:0003824)	1
OG0034163	Molecular Function	binding(GO:0005488)	1
OG0034163	Molecular Function	catalytic activity(GO:0003824)	1
OG0034166	Molecular Function	catalytic activity(GO:0003824)	1
OG0034172	Molecular Function	binding(GO:0005488)	1
OG0034172	Molecular Function	catalytic activity(GO:0003824)	1
OG0034173	Molecular Function	catalytic activity(GO:0003824)	1
OG0034182	Molecular Function	binding(GO:0005488)	1
OG0034182	Molecular Function	catalytic activity(GO:0003824)	1
OG0034183	Molecular Function	binding(GO:0005488)	1
OG0034183	Molecular Function	catalytic activity(GO:0003824)	1
OG0034188	Molecular Function	catalytic activity(GO:0003824)	1
OG0034189	Molecular Function	catalytic activity(GO:0003824)	1
OG0034190	Molecular Function	catalytic activity(GO:0003824)	1
OG0034192	Molecular Function	catalytic activity(GO:0003824)	1
OG0034195	Molecular Function	binding(GO:0005488)	1
OG0034195	Molecular Function	catalytic activity(GO:0003824)	1
OG0034197	Molecular Function	binding(GO:0005488)	1
OG0034197	Molecular Function	catalytic activity(GO:0003824)	1
OG0034198	Molecular Function	binding(GO:0005488)	1
OG0034198	Molecular Function	catalytic activity(GO:0003824)	1
OG0034200	Molecular Function	catalytic activity(GO:0003824)	1

OG0034201	Molecular Function	binding(GO:0005488)	1
OG0034201	Molecular Function	catalytic activity(GO:0003824)	1
OG0034203	Molecular Function	catalytic activity(GO:0003824)	1
OG0034203	Molecular Function	transporter activity(GO:0005215)	1
OG0034209	Molecular Function	binding(GO:0005488)	1
OG0034209	Molecular Function	catalytic activity(GO:0003824)	1
OG0034212	Molecular Function	binding(GO:0005488)	1
OG0034215	Molecular Function	catalytic activity(GO:0003824)	1
OG0034216	Molecular Function	binding(GO:0005488)	1
OG0034218	Molecular Function	catalytic activity(GO:0003824)	1
OG0034223	Molecular Function	catalytic activity(GO:0003824)	1
OG0034225	Molecular Function	catalytic activity(GO:0003824)	1
OG0034227	Molecular Function	binding(GO:0005488)	1
OG0034227	Molecular Function	catalytic activity(GO:0003824)	1
OG0034228	Molecular Function	binding(GO:0005488)	1
OG0034228	Molecular Function	catalytic activity(GO:0003824)	1
OG0034229	Molecular Function	catalytic activity(GO:0003824)	1
OG0034233	Molecular Function	catalytic activity(GO:0003824)	1
OG0034238	Molecular Function	catalytic activity(GO:0003824)	1
OG0034239	Molecular Function	catalytic activity(GO:0003824)	1
OG0034247	Molecular Function	catalytic activity(GO:0003824)	1
OG0034250	Molecular Function	binding(GO:0005488)	1
OG0034250	Molecular Function	catalytic activity(GO:0003824)	1
OG0034251	Molecular Function	binding(GO:0005488)	1
OG0034251	Molecular Function	catalytic activity(GO:0003824)	1
OG0034252	Molecular Function	binding(GO:0005488)	1
OG0034252	Molecular Function	catalytic activity(GO:0003824)	1
OG0034254	Molecular Function	catalytic activity(GO:0003824)	1
OG0034258	Molecular Function	catalytic activity(GO:0003824)	1
OG0034261	Molecular Function	catalytic activity(GO:0003824)	1
OG0034262	Molecular Function	catalytic activity(GO:0003824)	1
OG0034264	Molecular Function	catalytic activity(GO:0003824)	1
OG0034268	Molecular Function	transporter activity(GO:0005215)	1
OG0034269	Molecular Function	catalytic activity(GO:0003824)	1
OG0034278	Molecular Function	binding(GO:0005488)	1
OG0034279	Molecular Function	binding(GO:0005488)	1
OG0034279	Molecular Function	catalytic activity(GO:0003824)	1
OG0034281	Molecular Function	catalytic activity(GO:0003824)	1
OG0034283	Molecular Function	catalytic activity(GO:0003824)	1
OG0034284	Molecular Function	catalytic activity(GO:0003824)	1
OG0034285	Molecular Function	structural molecule activity(GO:0005198)	1
OG0034286	Molecular Function	binding(GO:0005488)	1
OG0034286	Molecular Function	catalytic activity(GO:0003824)	1
OG0034291	Molecular Function	binding(GO:0005488)	1
OG0034295	Molecular Function	structural molecule activity(GO:0005198)	1
OG0034298	Molecular Function	binding(GO:0005488)	1
OG0034298	Molecular Function	catalytic activity(GO:0003824)	1

OG0034299	Molecular Function	transporter activity(GO:0005215)	1
OG0034306	Molecular Function	catalytic activity(GO:0003824)	1
OG0034307	Molecular Function	structural molecule activity(GO:0005198)	1
OG0034308	Molecular Function	binding(GO:0005488)	1
OG0034308	Molecular Function	structural molecule activity(GO:0005198)	1
OG0034309	Molecular Function	structural molecule activity(GO:0005198)	1
OG0034310	Molecular Function	structural molecule activity(GO:0005198)	1
OG0034311	Molecular Function	structural molecule activity(GO:0005198)	1
OG0034312	Molecular Function	structural molecule activity(GO:0005198)	1
OG0034313	Molecular Function	structural molecule activity(GO:0005198)	1
OG0034315	Molecular Function	structural molecule activity(GO:0005198)	1
OG0034316	Molecular Function	binding(GO:0005488)	1
OG0034316	Molecular Function	catalytic activity(GO:0003824)	1
OG0034317	Molecular Function	catalytic activity(GO:0003824)	1
OG0034323	Molecular Function	binding(GO:0005488)	1
OG0034323	Molecular Function	catalytic activity(GO:0003824)	1
OG0034326	Molecular Function	catalytic activity(GO:0003824)	1
OG0034333	Molecular Function	binding(GO:0005488)	1
OG0034333	Molecular Function	catalytic activity(GO:0003824)	1
OG0034336	Molecular Function	catalytic activity(GO:0003824)	1
OG0034348	Molecular Function	catalytic activity(GO:0003824)	1
OG0034350	Molecular Function	catalytic activity(GO:0003824)	1
OG0034356	Molecular Function	catalytic activity(GO:0003824)	1
OG0034383	Molecular Function	catalytic activity(GO:0003824)	1
OG0034407	Molecular Function	transcription regulator activity(GO:0140110)	1
OG0034410	Molecular Function	transcription regulator activity(GO:0140110)	1
OG0034482	Molecular Function	binding(GO:0005488)	1
OG0034487	Molecular Function	catalytic activity(GO:0003824)	1
OG0034489	Molecular Function	transporter activity(GO:0005215)	1
OG0034497	Molecular Function	catalytic activity(GO:0003824)	1
OG0034498	Molecular Function	catalytic activity(GO:0003824)	1
OG0034499	Molecular Function	binding(GO:0005488)	1
OG0034504	Molecular Function	catalytic activity(GO:0003824)	1
OG0034517	Molecular Function	catalytic activity(GO:0003824)	1
OG0034522	Molecular Function	binding(GO:0005488)	1
OG0034525	Molecular Function	transcription regulator activity(GO:0140110)	1
OG0034529	Molecular Function	catalytic activity(GO:0003824)	1
OG0034533	Molecular Function	catalytic activity(GO:0003824)	1
OG0034539	Molecular Function	binding(GO:0005488)	1
OG0034539	Molecular Function	catalytic activity(GO:0003824)	1
OG0034548	Molecular Function	catalytic activity(GO:0003824)	1
OG0034549	Molecular Function	catalytic activity(GO:0003824)	1
OG0034553	Molecular Function	binding(GO:0005488)	1
OG0034553	Molecular Function	catalytic activity(GO:0003824)	1

OG0034557	Molecular Function	transcription regulator activity(GO:0140110)	1
OG0034561	Molecular Function	catalytic activity(GO:0003824)	1
OG0034562	Molecular Function	catalytic activity(GO:0003824)	1
OG0034567	Molecular Function	catalytic activity(GO:0003824)	1
OG0034568	Molecular Function	binding(GO:0005488)	1
OG0034568	Molecular Function	catalytic activity(GO:0003824)	1
OG0034572	Molecular Function	binding(GO:0005488)	1
OG0034572	Molecular Function	catalytic activity(GO:0003824)	1
OG0034572	Molecular Function	molecular function regulator(GO:0098772)	1
OG0034574	Molecular Function	binding(GO:0005488)	1
OG0034574	Molecular Function	catalytic activity(GO:0003824)	1
OG0034576	Molecular Function	structural molecule activity(GO:0005198)	1
OG0034577	Molecular Function	structural molecule activity(GO:0005198)	1
OG0034581	Molecular Function	binding(GO:0005488)	1
OG0034581	Molecular Function	catalytic activity(GO:0003824)	1
OG0034582	Molecular Function	structural molecule activity(GO:0005198)	1
OG0034583	Molecular Function	catalytic activity(GO:0003824)	1
OG0034586	Molecular Function	catalytic activity(GO:0003824)	1
OG0034587	Molecular Function	structural molecule activity(GO:0005198)	1
OG0034588	Molecular Function	structural molecule activity(GO:0005198)	1
OG0034589	Molecular Function	structural molecule activity(GO:0005198)	1
OG0034590	Molecular Function	structural molecule activity(GO:0005198)	1
OG0034591	Molecular Function	structural molecule activity(GO:0005198)	1
OG0034592	Molecular Function	binding(GO:0005488)	1
OG0034592	Molecular Function	structural molecule activity(GO:0005198)	1
OG0034599	Molecular Function	catalytic activity(GO:0003824)	1
OG0034599	Molecular Function	transporter activity(GO:0005215)	1
OG0034600	Molecular Function	binding(GO:0005488)	1
OG0034600	Molecular Function	catalytic activity(GO:0003824)	1
OG0034601	Molecular Function	catalytic activity(GO:0003824)	1
OG0034608	Molecular Function	structural molecule activity(GO:0005198)	1
OG0034614	Molecular Function	catalytic activity(GO:0003824)	1
OG0034623	Molecular Function	catalytic activity(GO:0003824)	1
OG0034626	Molecular Function	catalytic activity(GO:0003824)	1
OG0034627	Molecular Function	catalytic activity(GO:0003824)	1
OG0034629	Molecular Function	binding(GO:0005488)	1
OG0034632	Molecular Function	binding(GO:0005488)	1
OG0034638	Molecular Function	catalytic activity(GO:0003824)	1
OG0034643	Molecular Function	catalytic activity(GO:0003824)	1
OG0034645	Molecular Function	catalytic activity(GO:0003824)	1
OG0034645	Molecular Function	transporter activity(GO:0005215)	1
OG0034646	Molecular Function	catalytic activity(GO:0003824)	1
OG0034646	Molecular Function	transporter activity(GO:0005215)	1
OG0034647	Molecular Function	catalytic activity(GO:0003824)	1

OG0034648	Molecular Function	catalytic activity(GO:0003824)	1
OG0034650	Molecular Function	catalytic activity(GO:0003824)	1
OG0034652	Molecular Function	catalytic activity(GO:0003824)	1
OG0034658	Molecular Function	catalytic activity(GO:0003824)	1
OG0034659	Molecular Function	catalytic activity(GO:0003824)	1
OG0034672	Molecular Function	transcription regulator activity(GO:0140110)	1
OG0034673	Molecular Function	transcription regulator activity(GO:0140110)	1
OG0034675	Molecular Function	transcription regulator activity(GO:0140110)	1
OG0034676	Molecular Function	transcription regulator activity(GO:0140110)	1
OG0034699	Molecular Function	catalytic activity(GO:0003824)	1
OG0034705	Molecular Function	transporter activity(GO:0005215)	1
OG0034707	Molecular Function	binding(GO:0005488)	1
OG0034707	Molecular Function	catalytic activity(GO:0003824)	1
OG0034722	Molecular Function	binding(GO:0005488)	1
OG0034722	Molecular Function	transcription regulator activity(GO:0140110)	1
OG0034730	Molecular Function	binding(GO:0005488)	1
OG0034732	Molecular Function	binding(GO:0005488)	1
OG0034732	Molecular Function	transcription regulator activity(GO:0140110)	1
OG0034735	Molecular Function	catalytic activity(GO:0003824)	1
OG0034737	Molecular Function	transporter activity(GO:0005215)	1
OG0034750	Molecular Function	catalytic activity(GO:0003824)	1
OG0034769	Molecular Function	catalytic activity(GO:0003824)	1
OG0034770	Molecular Function	transporter activity(GO:0005215)	1
OG0034785	Molecular Function	binding(GO:0005488)	1
OG0034785	Molecular Function	catalytic activity(GO:0003824)	1
OG0034805	Molecular Function	binding(GO:0005488)	1
OG0034812	Molecular Function	binding(GO:0005488)	1
OG0034825	Molecular Function	transcription regulator activity(GO:0140110)	1
OG0034842	Molecular Function	binding(GO:0005488)	1
OG0034852	Molecular Function	catalytic activity(GO:0003824)	1
OG0034852	Molecular Function	molecular transducer activity(GO:0060089)	1
OG0034853	Molecular Function	binding(GO:0005488)	1
OG0034888	Molecular Function	catalytic activity(GO:0003824)	1
OG0034909	Molecular Function	catalytic activity(GO:0003824)	1
OG0034926	Molecular Function	catalytic activity(GO:0003824)	1
OG0034930	Molecular Function	binding(GO:0005488)	1
OG0034935	Molecular Function	transcription regulator activity(GO:0140110)	1
OG0034981	Molecular Function	antioxidant activity(GO:0016209)	1
OG0034981	Molecular Function	catalytic activity(GO:0003824)	1
OG0034981	Molecular Function	molecular function regulator(GO:0098772)	1
OG0034982	Molecular Function	binding(GO:0005488)	1
OG0034989	Molecular Function	catalytic activity(GO:0003824)	1
OG0034998	Molecular Function	binding(GO:0005488)	1
OG0034998	Molecular Function	catalytic activity(GO:0003824)	1
OG0035020	Molecular Function	transcription regulator activity(GO:0140110)	1

OG0035022	Molecular Function	catalytic activity(GO:0003824)	1
OG0035025	Molecular Function	binding(GO:0005488)	1
OG0035028	Molecular Function	binding(GO:0005488)	1
OG0035028	Molecular Function	catalytic activity(GO:0003824)	1
OG0035031	Molecular Function	catalytic activity(GO:0003824)	1
OG0035032	Molecular Function	binding(GO:0005488)	1
OG0035047	Molecular Function	catalytic activity(GO:0003824)	1
OG0035063	Molecular Function	catalytic activity(GO:0003824)	1
OG0035077	Molecular Function	transcription regulator activity(GO:0140110)	1
OG0035083	Molecular Function	catalytic activity(GO:0003824)	1
OG0035094	Molecular Function	transporter activity(GO:0005215)	1
OG0035095	Molecular Function	transcription regulator activity(GO:0140110)	1
OG0035099	Molecular Function	catalytic activity(GO:0003824)	1
OG0035150	Molecular Function	binding(GO:0005488)	1
OG0035168	Molecular Function	catalytic activity(GO:0003824)	1
OG0035168	Molecular Function	transporter activity(GO:0005215)	1
OG0035175	Molecular Function	binding(GO:0005488)	1
OG0035175	Molecular Function	transcription regulator activity(GO:0140110)	1
OG0035175	Molecular Function	translation regulator activity(GO:0045182)	1
OG0035177	Molecular Function	binding(GO:0005488)	1
OG0035177	Molecular Function	transcription regulator activity(GO:0140110)	1
OG0035177	Molecular Function	translation regulator activity(GO:0045182)	1
OG0035181	Molecular Function	binding(GO:0005488)	1
OG0035181	Molecular Function	transcription regulator activity(GO:0140110)	1
OG0035181	Molecular Function	translation regulator activity(GO:0045182)	1
OG0035194	Molecular Function	binding(GO:0005488)	1
OG0035194	Molecular Function	catalytic activity(GO:0003824)	1
OG0035200	Molecular Function	binding(GO:0005488)	1
OG0035200	Molecular Function	catalytic activity(GO:0003824)	1
OG0035205	Molecular Function	catalytic activity(GO:0003824)	1
OG0035214	Molecular Function	binding(GO:0005488)	1
