# Supplementary material for: A high-quality genome provides insights into the new taxonomic status and genomic characteristics of Cladopus chinensis (Podostemaceae)
Source: Hortic Res. 2020 Apr 1;7:46. doi: 10.1038/s41438-020-0269-5 (PMC7109043; doi:10.1038/s41438-020-0269-5)
Supplement: Supplementary file 5 — Supplementary Table S1-S7,S26-27 [file 41438_2020_269_MOESM5_ESM.docx]

**Supplementary Tables**

Table S1 Resequencing alignment rate of the *C. chinensis* genome by Bowtie2 software

| Mapping reads | 342,007632 |
| --- | --- |
| Mapping rate (%) | 99.78 |
| Unmapping reads | 754,076 |
| Unmapping rate (%) | 0.22 |
| Total reads | 342,761,708 |

Table S2 Assessment of the completeness of the *C. chinensis* genome assembly by BUSCO

| Type | Number | Percent (%) |
| --- | --- | --- |
| Complete BUSCOs (C) | 1,247 | 90.7 |
| Complete and single-copy BUSCOs (S) | 352 | 25.6 |
| Complete and duplicated BUSCOs (D) | 895 | 65.1 |
| Fragmented BUSCOs (F) | 23 | 1.7 |
| Missing BUSCOs (M) | 105 | 7.6 |
| Total BUSCO groups searched | 1,375 | 100 |

Table S3 Pseudomolecule length statistics after Hi-C assisted assembly

| Pseudomolecule | Scaffold Num | Length |
| --- | --- | --- |
| chr1 | 22 | 33,494,499 |
| chr2 | 28 | 27,810,442 |
| chr3 | 19 | 26,329,586 |
| chr4 | 22 | 31,969,939 |
| chr5 | 17 | 30,432,471 |
| chr6 | 17 | 32,871,161 |
| chr7 | 27 | 22,380,268 |
| chr8 | 18 | 30,192,955 |
| chr9 | 23 | 25,425,217 |
| chr10 | 20 | 27,323,330 |
| chr11 | 22 | 24,023,441 |
| chr12 | 30 | 21,225,508 |
| chr13 | 22 | 21,701,559 |
| chr14 | 18 | 19,621,026 |
| chr15 | 16 | 21,785,505 |
| chr16 | 23 | 21,030,367 |
| chr17 | 23 | 24,828,186 |
| chr18 | 18 | 18,583,880 |
| chr19 | 10 | 21,174,227 |
| chr20 | 21 | 24,067,307 |
| chr21 | 14 | 16,285,696 |
| chr22 | 14 | 18,286,461 |
| chr23 | 21 | 21,976,262 |
| chr24 | 11 | 22,041,959 |
| chr25 | 17 | 16,142,721 |
| chr26 | 13 | 18,872,796 |
| chr27 | 15 | 20,588,600 |
| chr28 | 14 | 16,772,361 |
| chr29 | 3 | 2,187,051 |
| Total anchored | 538 | 659,424,781 |
| Unanchored | 4391 | 168,493,579 |

Table S4 Transcription factors

| Number | Classify |
| --- | --- |
| 705 | protein kinase family protein |
| 459 | pentatricopeptide (PPR) repeat-containing protein |
| 155 | MYB family protein |
| 123 | basic helix-loop-helix (bHLH) DNA-binding superfamily protein |
| 110 | Integrase-type DNA-binding superfamily protein |
| 89 | C2H2-type zinc finger family protein |
| 81 | NAC domain containing protein 2 |
| 79 | basic region/leucine zipper transcription factor 68 |
| 75 | WRKY family transcription factor |
| 45 | WD-40 repeat family protein / zfwd4 protein (ZFWD4) |
| 43 | M-type_MADS family protein |
| 39 | GATA transcription factor 1 |
| 38 | AP2 transcription factor family protein |
| 29 | GRAS family transcription factor |
| 15 | Dof family protein |
| 12 | B3 transcription factor family protein |
| 10 | CO-like family protein |
| 9 | TCP family transcription factor |
| 8 | FAR1-related sequence 4 |
| 8 | plant-specific transcription factor YABBY family protein |
| 4 | C3H family protein |
| 4 | E2F transcription factor 3 |
| 4 | LSD family protein |
| 4 | SBP transcription factor family protein |
| 3 | BES1-interacting Myc-like protein 2 |
| 2 | HD-ZIP family protein |
| 2 | LFY family protein |
| 1 | HB family protein |

Table S5 Statistical analysis of non-coding RNAs in *C. chinensis* genome

| Class | number | totalLen | meanLen |
| --- | --- | --- | --- |
| rRNAs | 397 | 196,795 | 495.7052897 |
| miRNAs | 79 | 7,886 | 99.82278481 |
| tRNAs | 1,997 | 149,597 | 74.9108663 |
| Intron | 384 | 39,486 | 102.828125 |
| sRNAs | 116 | 10,530 | 90.77586207 |
| snRNAs | 128 | 39,323 | 122.5836094 |

Table S6 Repetitive element annotations in the *C. chinensis*

|  | No. of TEs | Length (bp) | % of genome |
| --- | --- | --- | --- |
| Total repeat fraction | 163,492 | 490,128,971 | 59.20 |
| Class I: Retroelement | 95,288 | 386,955,019 | 46.63 |
| LTR Retrotransposon | 49,766 | 239,848,761 | 28.97 |
| Ty1/Copia | 38,419 | 184,626,512 | 22.30 |
| Ty3/gypsy | 1,425 | 5,968,938 | 0.72 |
| Non-LTR Retrotransposon | 37,381 | 135,199,143 | 16.33 |
| LINE | 35,540 | 134,123,355 | 16.20 |
| SINE | 1,841 | 1,076,788 | 0.13 |
| Class II: DNA Transposon | 16,415 | 63,170,623 | 7.63 |
| Other | 16,415 | 63,170,623 | 7.63 |
| Tandem Repeats | 50,071 | 61,266,580 | 7.40 |

Table S7 Size and location of telomere satellite repeat arrays

| Contig_Name | Contig_Size | Start_of_telo | End_of_telo | Size_of_telo | posi_of_telo | No_of_tel | Teleomeric_repeat_sequence |
| --- | --- | --- | --- | --- | --- | --- | --- |
| tig00000051_pilon | 5126398 | 4380982 | 4849614 | 468632 | end | 9249.7 | AAATTAATAAATTTACTTAAAATAATATTATAAGTTATATAATTTTATTA |
| tig00000051_pilon | 5126398 | 4440083 | 4849578 | 409495 | end | 44575.1 | ATTAAATTA |
| tig00000051_pilon | 5126398 | 4753563 | 4849612 | 96049 | end | 5035.7 | GCTAATTTTTAATATTATA |
| tig00000051_pilon | 5126398 | 4806678 | 4849645 | 42967 | end | 248.3 | AGTACCTTATTATAAATTATAATAGAATAATTATAAAATCAAATAACACTTAATAGAATAATATAAATTTAAAAAATATAAAAATAAATAAAGATTTTTTCTATTAGAAAAAAAAATAATATCTTAAATCCAAATATTACGAATATAAGTATAGATGAATATAAAACTAAAT |
| tig00000051_pilon | 5126398 | 4823244 | 4849583 | 26339 | end | 13489.5 | TA |
| tig00000051_pilon | 5126398 | 4823237 | 4849657 | 26420 | end | 353.4 | TTAATTTTTTTTAAATATAATTTTATTGAAATTAAATTAATATTTTTAATTTAAATTCTAAAACAATGAAAATA |
| tig00000255_pilon | 3375573 | 2690839 | 3188691 | 497852 | end | 41012.6 | ATATATATTTAA |
| tig00000255_pilon | 3375573 | 2734587 | 2791035 | 56448 | end | 2676.9 | ATTATAAATAATTGTCATGTT |

Table S26 Percent identity of NAC gene paris

| Gene pairs | Percent identity score |
| --- | --- |
| Cladopus_020264-Cladopus_004681 | 97.8 |
| Cladopus_004734-Cladopus_025202 | 97.0 |
| Cladopus_004681_Cladopus_020264 | 97.8 |

Table S27 Primers for genes validated by quantitative real-time PCR (qRT-PCR)

| Genes | Sequences |
| --- | --- |
| GDAPH-F | TCACCGCTACCCAGAAAACT |
| GDAPH-R | AGTAGGAACACGGAATGCCA |
| CcALS1-F | GCAGTGAAAGCTAGGCATCC |
| CcALS1-R | GGTGGCGTACATATCCTCGA |
| CcALS2-F | CAATACGGCGAAGGGAAGTG |
| CcALS2-R | TCCCAGCAATCAGTGACCAT |
| CcALS4-F | AGTTCGTAAAGGCCCATGGA |
| CcALS4-R | CAAACTTCTCGTGGAGCTCG |
| CcALS7-F | ACGAGCAGGGTTGAGAAGAA |
| CcALS7-R | TCTTCCCGCTATCAGTGACC |
| CcALS9-F | AAGAGGCTTGGCAAATGGTG |
| CcALS9-R | AGGGAGGAGCGTTAGATGTG |
| CcSTM3-F | ATCCAGCCCTCGATCAGTTC |
| CcSTM3-R | ACGCATTCCTCTCATTTGCC |
| CcSTM4-F | CGAACAACCGCACTCTTCTT |
| CcSTM4-R | CACCTGCCAGACACCTTTTC |
| CcWUS1-F | ACTTCGCTCCCTCCAATGAA |
| CcWUS1-R | TCTTGCTCCTTGTACTGGCA |
| CcWUS10-F | AGAGCTCTTCAAATCTGGGCT |
| CcWUS10-R | CGATTACCGCCTTCTTGTACG |
