# Supplementary material for: A high-quality genome provides insights into the new taxonomic status and genomic characteristics of Cladopus chinensis (Podostemaceae)
Source: Hortic Res. 2020 Apr 1;7:46. doi: 10.1038/s41438-020-0269-5 (PMC7109043; doi:10.1038/s41438-020-0269-5)
Supplement: Supplementary file 6 — Table S8 Size and location of centromere satellite repeat arrays [file 41438_2020_269_MOESM6_ESM.pdf]

seqid	start	end	period	copynum	consensus	pctmatch	pctindel	score
tig00000C	5704	46053	175	231.4	174	48	15	5294
tig00000C	6439	38418	175	184.9	173	51	13	6636
tig00000C	34346	47703	176	76	176	45	15	1190
tig00000C	35365	47707	175	70.9	175	59	10	4614
tig00000C	53067	55781	175	15.6	175	64	4	1497
tig00000C	84351	99332	175	86	175	50	14	2419
tig00000C	2542372	2679880	176	781.7	175	47	15	13137
tig00000C	2544408	2563336	175	107.6	176	55	11	4406
tig00000C	2563315	2582746	175	110.1	176	48	14	1996
tig00000C	2582714	2597658	175	85.3	176	49	15	2456
tig00000C	2673157	2681694	176	48.8	174	61	7	3989
tig00000C	2698006	2729346	175	179.2	175	50	11	7691
tig00000C	2771129	2774553	176	19.6	175	62	8	1426
tig00000C	2771084	2773455	176	13.6	176	72	3	1543
tig00000C	2774793	2777322	176	14.5	176	74	2	1775
tig00000C	4640586	4647035	174	37.5	173	55	11	1544
tig00000C	4635681	4648956	175	75.2	175	47	15	1704
tig00000C	4674787	4681790	175	39.7	174	48	14	1249
tig00000C	4693238	4698107	175	28.1	174	51	12	1002
tig00000C	4720823	4726786	175	34.3	175	57	9	2302
tig00000C	4738676	4748105	174	54.8	170	47	16	1452
tig00000C	5418001	5447944	176	170.5	174	42	16	1809
tig00000C	5416925	5426978	176	57.2	176	51	13	2476
tig00000C	5444776	5463616	176	106.9	176	46	15	2459
tig00000C	5458378	5463620	174	30.4	173	58	10	1940
tig00000C	5529833	5533109	174	18.9	172	60	8	1528
tig00000C	5623226	5648321	176	142.9	175	50	15	3949
tig00000C	5646227	5661104	175	84.8	174	48	15	2133
tig00000C	5657758	5661109	174	19.3	174	65	8	1741
tig00000C	5788548	5810452	176	122.6	176	38	18	1136
tig00000C	5854080	5939610	176	487.1	174	44	16	6137
tig00000C	5853839	5953027	176	561.6	176	43	17	4882
tig00000C	5947222	5955290	176	46.2	174	57	12	2668
tig00000C	5954353	6012417	175	330.5	175	43	16	2497
tig00000C	5969633	6062546	175	531.6	174	45	15	8399
tig00000C	6051421	6067265	176	89.6	175	49	14	2773
tig00000C	6070883	6133725	175	358.4	174	45	15	5734
tig00000C	6095290	6138888	175	248.1	175	46	15	5657
tig00000C	6137204	6139155	174	11.3	175	64	6	1020
tig00000C	6152967	6158927	175	34.6	170	52	13	1342
tig00000C	6153035	6156460	176	19.6	175	54	10	1098
tig00000C	6156460	6277332	175	683.4	176	42	16	5345
tig00000C	6186897	6225853	175	221.9	174	47	14	6322
tig00000C	6240638	6327229	175	493.6	174	46	15	9672
tig00000C	6289986	6333710	175	246.4	176	46	16	3729
tig00000C	7212687	7231781	175	108.9	175	45	16	1980

tig00000C	7232554	7240225	176	43.6	176	46	15	1405
tig00000C	7236881	7245472	176	48.5	175	50	15	1515
tig00000C	7239822	7242717	175	16.6	175	70	3	1804
tig00000C	7242690	7245461	176	15.8	175	70	4	1711
tig00000C	7708273	7716775	175	49.1	173	46	15	1156
tig00000C	7761948	7769928	176	45.5	176	50	12	1842
tig00000C	8691922	8752755	175	347.7	174	42	16	1380
tig00000C	8700779	8814882	175	646.5	175	40	18	1389
tig00000C	8707368	8712104	176	27.4	174	67	6	2464
tig00000C	8707218	8751835	175	253.6	176	45	15	3518
tig00000C	8750698	8752892	176	12.5	176	69	5	1290
tig00000C	319289	321526	176	12.9	176	75	4	1571
tig00000C	1117571	1123374	176	32.8	176	55	12	1193
tig00000C	1117545	1123374	175	33	176	58	11	1504
tig00000C	1137408	1145326	176	45	176	65	7	3722
tig00000C	1146491	1161581	176	86.9	174	76	4	10415
tig00000C	1168297	1198448	176	168.4	179	39	18	1396
tig00000C	3323962	3334571	175	60.5	175	53	13	2243
tig00000C	3323962	3333337	175	53.3	176	64	7	4267
tig00000C	6562527	6711266	175	867.6	169	42	16	7300
tig00000C	6621168	6671668	174	289.2	174	43	15	3770
tig00000C	6755031	6769784	175	84.9	174	43	16	1433
tig00000C	6774569	6779597	176	28.6	175	48	12	1056
tig00000C	6817252	6820596	174	19.2	174	54	9	1156
tig00000C	6842333	6921027	175	447	175	45	15	8070
tig00000C	314941	342073	175	154.9	175	44	16	2157
tig00000C	327075	342114	174	86.4	174	48	14	2022
tig00000C	1200482	1237795	174	214.2	174	45	14	4897
tig00000C	1237590	1240869	174	19	173	63	6	1686
tig00000C	1245548	1255171	174	55.6	172	45	14	1196
tig00000C	1252017	1254972	174	17.1	175	61	7	1440
tig00000C	1265277	1275470	174	58.5	174	49	14	1865
tig00000C	1332716	1344257	175	65.9	175	49	14	2211
tig00000C	1341779	1344934	176	18.3	176	55	11	1040
tig00000C	1752977	1759246	175	35.8	176	77	2	4462
tig00000C	1934072	1952664	175	106.3	173	46	15	1927
tig00000C	1935307	1951307	175	91.4	175	56	11	4956
tig00000C	2517304	2552676	175	202.1	174	43	16	2384
tig00000C	2538214	2601920	174	361.5	175	41	16	2603
tig00000C	2553463	2556334	176	16.6	174	63	9	1160
tig00000C	2632704	2643842	174	63.9	172	43	15	1285
tig00000C	2641698	2643842	176	12.3	176	68	2	1262
tig00000C	2656502	2745512	175	508	174	44	15	6091
tig00000C	2659513	2679574	174	115.6	173	44	15	2105
tig00000C	2719962	2741752	175	124.1	176	54	9	6715
tig00000C	2753362	2756284	175	16.9	175	59	9	1198
tig00000C	2923587	2926214	174	15.1	175	58	7	1149

tig00000C	2957807	2969674	174	67.9	174	45	16	1506
tig00000C	2988932	2992363	175	19.8	176	64	7	1778
tig00000C	3002417	3020327	174	101	176	47	13	2768
tig00000C	3009509	3020412	174	62.9	173	55	11	3239
tig00000C	3186568	3191996	175	31.1	173	58	11	1950
tig00000C	3186601	3190933	175	25	175	61	8	2017
tig00000C	3218942	3229582	174	61.7	173	47	16	1607
tig00000C	3364421	3677433	175	1783	175	44	17	11785
tig00000C	3625631	3642242	174	95.2	174	52	12	2880
tig00000C	3612478	3716638	175	596.7	174	45	15	6148
tig00000C	3730672	3744919	176	81.2	175	50	13	2722
tig00000C	3730672	3744794	175	80.5	174	49	13	2410
tig00000C	3745960	3749353	175	19.7	174	64	8	1780
tig00000C	3760357	3767246	175	39.4	175	55	11	2118
tig00000C	3761160	3763836	176	15.4	174	68	6	1527
tig00000C	3788336	3878248	175	510.9	176	46	14	10302
tig00000C	3895512	3897640	174	12.2	171	66	7	1150
tig00000C	3899999	3932384	175	184.8	175	43	16	1247
tig00000C	3902938	3929998	175	153.2	176	43	15	1709
tig00000C	3922114	3932248	174	58.2	174	53	11	2547
tig00000C	3984644	4011457	175	152.6	175	47	13	4452
tig00000C	3996588	4012297	175	91.1	174	60	8	6731
tig00000C	4032583	4126718	175	538.2	174	44	15	9251
tig00000C	4122082	4126718	174	26.7	174	60	9	2013
tig00000C	4174327	4207267	174	189.7	173	41	17	1184
tig00000C	4216359	4231997	174	89.8	174	44	16	1470
tig00000C	4235438	4237561	174	12.3	173	64	6	1118
tig00000C	4252737	4262547	175	56.1	175	53	12	2883
tig00000C	4264047	4266314	174	13.1	172	65	8	1191
tig00000C	4282069	4291208	175	52.9	175	49	15	1053
tig00000C	4302107	4384222	175	470.5	174	45	15	7549
tig00000C	4347086	4384199	174	212.8	174	49	14	5828
tig00000C	4389666	4423540	174	193.5	173	46	15	4282
tig00000C	1237	4750	174	20.2	173	66	9	1834
tig00000C	4727	18376	175	78.2	175	52	13	3145
tig00000C	365	19069	174	106.2	175	44	15	2191
tig00000C	3632	18690	175	85.9	174	43	15	1272
tig00000C	29721	34712	174	29	174	58	11	1865
tig00000C	32355	34694	175	13.5	173	64	6	1253
tig00000C	59294	78007	174	107.3	173	43	15	1594
tig00000C	71010	78231	174	40.9	175	52	12	2011
tig00000C	100541	106368	175	33.4	174	51	12	1509
tig00000C	104913	120885	174	91.4	173	45	15	2190
tig00000C	128076	136134	174	45.8	174	43	15	1050
tig00000C	156692	229542	175	412.6	175	44	15	5626
tig00000C	1529001	1541886	175	73.7	174	58	10	4724
tig00000C	1547510	1590452	174	244.6	174	44	16	2189

tig00000C	1558926	1656871	175	555.6	175	42	16	2901
tig00000C	1591588	1669201	174	442.6	174	43	15	6099
tig00000C	1667091	1675640	175	49.2	175	49	14	1633
tig00000C	1669156	1671357	174	12.7	171	63	7	1076
tig00000C	1700105	1702173	174	12	174	62	7	1027
tig00000C	1725033	1757957	175	187.7	174	46	13	4650
tig00000C	1734796	1741883	174	40.8	174	56	10	2362
tig00000C	1797889	1803341	175	31.4	175	53	14	1312
tig00000C	2143672	2148283	174	26.6	172	68	7	2618
tig00000C	2150956	2153306	174	13.5	174	67	5	1138
tig00000C	2160631	2182589	176	127.4	171	46	15	2843
tig00000C	2162948	2186350	176	134.6	175	50	13	4464
tig00000C	2206683	2209573	176	16.6	176	68	5	1602
tig00000C	3829609	3832179	175	14.7	174	70	4	1526
tig00000C	4043241	4081642	176	217.7	176	45	16	3086
tig00000C	4045746	4081193	175	201.3	175	45	16	3058
tig00000C	4042733	4066618	175	136.5	174	51	12	5552
tig00000C	4277091	4279982	175	16.5	176	66	3	1643
tig00000C	51182	53380	174	12.8	173	66	7	1263
tig00000C	57931	68662	174	61.7	174	54	12	1733
tig00000C	59335	80038	175	117.9	175	48	14	2309
tig00000C	73443	110821	175	214.3	174	43	16	1947
tig00000C	80146	108720	175	162.5	175	45	15	3005
tig00000C	93327	95570	174	13	173	64	8	1003
tig00000C	174983	177336	176	13.5	176	63	6	1193
tig00000C	209134	213509	174	25.3	173	61	6	2089
tig00000C	277713	286828	176	52.3	174	55	11	2940
tig00000C	277207	287002	174	56.7	174	48	16	1504
tig00000C	294005	304492	175	61	171	56	11	3277
tig00000C	294005	307219	174	75.6	174	48	14	2001
tig00000C	334754	347890	174	75.3	173	46	14	2180
tig00000C	349166	352233	175	17.5	173	59	11	1308
tig00000C	353687	376596	175	131.6	175	49	14	3594
tig00000C	368761	371415	174	15.3	173	64	7	1374
tig00000C	371382	376571	174	29.8	174	67	5	2989
tig00000C	388193	437658	175	281.7	175	46	15	5003
tig00000C	1754669	1786937	175	186	170	58	11	10893
tig00000C	1751644	2033438	175	1619.3	173	44	16	9622
tig00000C	1757896	1797542	174	227.1	175	57	11	11795
tig00000C	1797521	2034153	175	1349.1	175	45	15	14085
tig00000C	1964836	2000784	174	206.6	173	49	13	6878
tig00000C	2038763	2040872	175	12.2	173	65	6	1153
tig00000C	2067719	2077819	174	57.7	174	50	14	1293
tig00000C	2084232	2096608	174	70.5	174	43	14	1613
tig00000C	2096827	2104779	175	45.7	173	52	11	1919
tig00000C	4263503	4291348	175	159.3	175	49	14	5230
tig00000C	4281489	4294966	174	77.2	174	53	11	3034

tig00000C	4470217	4491272	174	120.1	175	47	14	2944
tig00000C	4477306	4488059	174	61.5	174	55	11	3307
tig00000C	5469656	5626030	175	890.3	176	49	14	24394
tig00000C	5625993	5632545	176	37.5	173	73	4	4180
tig00000C	5687067	5691742	174	26.6	175	59	10	1587
tig00000C	5691635	5694464	174	16.5	174	67	6	1585
tig00000C	1	5304	174	30.8	171	61	8	2509
tig00000C	21381	190028	175	962.2	174	45	14	15212
tig00000C	239006	260358	175	121.6	174	51	14	3123
tig00000C	754010	756720	175	15.7	175	76	5	1884
tig00000C	129251	131664	176	13.7	176	73	2	1610
tig00000C	129251	164559	175	201.1	174	47	14	4590
tig00000C	142945	164545	175	123.5	175	55	11	6856
tig00000C	142945	202071	175	336	176	46	15	4526
tig00000C	164357	200745	176	207.6	176	53	11	10039
tig00000C	167515	178924	175	65.6	174	69	7	5568
tig00000C	193574	201244	174	44.3	173	61	9	3381
tig00000C	205721	213367	174	43.6	175	52	11	2255
tig00000C	216527	325719	175	624.6	174	47	14	17236
tig00000C	335620	352260	174	96.3	173	62	8	7735
tig00000C	350711	353044	175	13.6	173	60	7	1052
tig00000C	362189	368242	174	34.9	174	49	13	1043
tig00000C	362189	391495	175	167	175	43	15	2091
tig00000C	381447	391493	174	57.6	174	52	12	2800
tig00000C	379029	391444	175	71.8	174	52	12	2883
tig00000C	414693	479114	175	365.6	175	43	16	3801
tig00000C	428744	435225	176	36.9	176	50	12	1333
tig00000C	456803	465550	176	50	176	53	12	2075
tig00000C	483119	493700	175	59.9	174	42	14	1288
tig00000C	499494	604266	175	620.5	165	42	15	5452
tig00000C	508285	512948	174	27	174	63	6	1939
tig00000C	517295	541911	176	141.8	172	44	14	2611
tig00000C	598305	605024	175	38.1	176	47	13	1369
tig00000C	607020	628438	175	122.7	173	46	15	2191
tig00000C	635415	638111	175	15.5	173	65	6	1471
tig00000C	682228	687224	175	28.5	175	49	12	1017
tig00000C	690809	693315	175	14.4	173	62	7	1157
tig00000C	707874	712386	174	26.3	174	59	9	1869
tig00000C	723703	728270	175	26.4	174	58	10	1787
tig00000C	732682	743229	174	61.1	174	46	13	1695
tig00000C	732682	734867	175	12.7	173	67	5	1229
tig00000C	739352	791242	174	297.9	173	44	15	4297
tig00000C	751128	832023	174	461.8	175	44	15	6223
tig00000C	813287	815414	175	12.4	172	67	6	1170
tig00000C	863576	866888	175	19	173	65	5	1804
tig00000C	899312	973574	175	425.8	173	42	15	3696
tig00000C	921638	1029816	175	619.4	174	42	16	5326

tig00000C	990134	993594	174	19.9	174	58	10	1352
tig00000C	1037582	1161220	175	710.1	174	45	14	12829
tig00000C	1152526	1158621	174	35.5	172	63	8	2917
tig00000C	1158659	1213545	175	312.6	175	44	16	3164
tig00000C	1175250	1213723	176	218.6	175	44	15	2344
tig00000C	1213726	1225909	175	70.1	173	59	11	3844
tig00000C	1213726	1224345	175	61	174	63	9	4467
tig00000C	1224328	1226393	176	11.9	175	75	6	1409
tig00000C	2191666	2236910	175	259.5	174	42	17	1902
tig00000C	2530060	2626958	175	551.2	176	51	13	18520
tig00000C	2622049	2626979	174	28.4	175	67	4	2834
tig00000C	2621908	2628742	175	39.5	173	67	6	3902
tig00000C	2635429	2677527	175	240.1	174	47	15	4252
tig00000C	2638162	2671583	175	190.3	176	50	13	5978
tig00000C	4956773	4980401	174	136	175	50	14	4578
tig00000C	4960583	4982938	174	129.5	170	45	16	1876
tig00000C	2294565	2304920	176	58.6	176	44	16	1006
tig00000C	2295498	2298478	174	16.9	173	62	10	1291
tig00000C	2303217	2400009	175	544.5	176	44	17	5849
tig00000C	2351625	2400512	175	274.3	176	47	15	7401
tig00000C	2407384	2418664	176	63.4	175	47	14	1830
tig00000C	2407384	2415429	176	45.9	174	57	11	3013
tig00000C	2429911	2431712	175	10.3	175	66	5	1017
tig00000C	2429911	2441141	176	63.9	175	44	14	1822
tig00000C	2449405	2453873	174	25.5	176	50	13	1155
tig00000C	2461880	2470666	175	49.9	175	46	13	1740
tig00000C	2482563	2488964	175	36.6	175	55	10	2144
tig00000C	2572383	2577132	174	27.2	172	55	9	1691
tig00000C	2595988	2600018	175	23.1	176	71	4	2520
tig00000C	2600372	2602162	175	10.3	176	70	5	1139
tig00000C	2615333	2619789	176	25.7	176	62	6	2139
tig00000C	2648210	2650325	176	12.1	176	68	2	1265
tig00000C	2739349	2756591	175	97.7	174	49	16	2485
tig00000C	2765223	2774419	174	52.7	174	53	11	2952
tig00000C	3067801	3112245	176	250.4	176	46	16	4728
tig00000C	3116900	3159117	175	251.1	165	41	18	2041
tig00000C	3136107	3159951	175	135.1	175	57	10	8318
tig00000C	3888613	4290780	175	2294.7	175	49	15	37591
tig00000C	5014229	5049065	175	199.4	175	52	11	9339
tig00000C	5014889	5047510	176	185.6	176	46	15	3679
tig00000C	5089610	5093615	175	23	175	64	9	1870
tig00000C	107812	465585	175	2013.7	176	38	17	10967
tig00000C	337804	452332	175	651	175	46	15	12106
tig00000C	444019	448058	174	23.2	174	68	4	2243
tig00000C	369864	464496	176	539.8	174	46	16	6660
tig00000C	646573	746689	176	564.9	176	43	16	5627
tig00000C	649405	712224	176	357.6	175	46	15	6541

tig00000C	718246	739428	176	119.9	175	53	12	5890
tig00000C	736313	760068	175	134.9	175	40	14	1203
tig00000C	956754	1332767	175	2146.1	175	47	16	22857
tig00000C	1325679	1349169	175	134.1	175	51	13	5328
tig00000C	1354571	1366327	175	67.7	173	42	15	1232
tig00000C	1437475	1440205	176	15.8	176	73	4	1811
tig00000C	2867591	3090408	175	1269.6	175	38	19	3898
tig00000C	3725121	3748691	175	134.9	174	44	16	1768
tig00000C	3731086	4228217	175	2825.1	176	47	17	35329
tig00000C	3731079	3745482	176	82.4	175	46	15	1190
tig00000C	4163527	4251391	175	501.3	175	55	13	13896
tig00000C	4137073	4251960	175	658	173	52	15	8800
tig00000C	1506	4678	174	18.3	175	77	2	2256
tig00000C	4675	9177	174	25.9	175	76	1	3260
tig00000C	9529	23745	174	82	175	57	11	4311
tig000001	799542	837899	175	218.6	175	44	14	4215
tig000001	823036	840244	175	98.3	174	51	14	2806
tig000001	823034	837899	174	84.9	175	48	14	1533
tig000001	956364	985227	175	164.7	173	42	17	1934
tig000001	1013249	1015875	174	15.3	173	62	8	1315
tig000001	1029311	1073971	175	255	174	44	16	2692
tig000001	1033437	1066270	174	194.1	166	42	18	1009
tig000001	2024181	2046836	175	128.8	175	48	14	3352
tig000001	2296461	2299882	175	19.7	176	68	4	1802
tig000001	2300096	2335358	175	200.9	174	44	15	4013
tig000001	2317643	2335359	176	102.8	172	44	15	1982
tig000001	2354368	2356961	175	15	171	66	7	1399
tig000001	2354367	2356145	176	10.2	175	68	4	1044
tig000001	2368137	2391303	175	133.3	172	47	13	3171
tig000001	2384127	2438653	175	311.4	175	43	15	5060
tig000001	2388537	2438620	174	285.9	174	44	15	4245
tig000001	2473046	2514169	174	234.8	175	41	17	1733
tig000001	4337400	4348909	174	65.5	175	50	14	2097
tig000001	4341871	4346179	175	24.5	174	50	14	1041
tig000001	4359553	4365266	175	33	173	55	10	1823
tig000001	655	262277	175	1508.4	171	52	15	24653
tig000001	1041	218030	175	1243.6	174	51	15	27669
tig000001	265246	268398	175	18	175	78	2	2276
tig000001	270914	273424	175	14.4	176	71	2	1568
tig000001	303000	306347	174	19.2	173	56	9	1310
tig000001	307209	309104	175	10.9	173	68	5	1112
tig000001	807117	819966	175	74.6	171	44	15	1208
tig000001	816860	826728	175	56.6	175	57	9	3553
tig000001	819931	826733	175	39.2	174	67	5	3875
tig000001	3127182	3207276	175	457.9	174	52	14	12193
tig000001	16520	30483	175	80.3	175	58	11	4533
tig000001	30449	35976	175	31.8	175	59	10	2103

tig000001	27509	35976	175	48.5	174	56	13	1811
tig000001	1261	4135	175	16.5	174	62	7	1401
tig000001	10275	12714	174	14	174	60	6	1149
tig000001	17594	21554	174	22.7	174	55	10	1384
tig000001	84163	100667	174	94.8	174	55	11	4310
tig000001	85269	110046	174	141.2	175	50	14	3987
tig000001	114933	118468	174	20.3	175	60	9	1448
tig000001	87894	108048	175	114.7	176	51	13	3342
tig000001	563147	623608	174	344.2	175	42	16	4219
tig000001	635668	642148	175	37.1	175	47	15	1060
tig000001	672523	678341	174	33.4	174	48	13	1092
tig000001	733729	764135	175	174.5	174	45	15	3586
tig000001	778352	788472	174	58.1	173	46	14	1625
tig000001	801413	806671	175	30.3	173	63	8	2639
tig000001	2453034	2459221	174	35.7	174	48	13	1160
tig000001	2474125	2491908	175	102.4	174	48	13	3020
tig000001	2475621	2491909	174	94	173	50	14	3594
tig000001	2498970	2504437	174	31.3	172	49	14	1172
tig000001	2545346	2556765	174	64.9	175	45	16	1112
tig000001	2549745	2556766	174	40.5	173	58	11	2527
tig000001	2580241	2596044	175	90.2	174	46	15	1551
tig000001	2581587	2607243	176	145.2	176	44	16	1447
tig000001	2600662	2606707	175	34.5	175	61	9	2437
tig000001	2602440	2624349	175	125.7	174	45	14	3306
tig000001	2631099	2636803	174	32.8	174	69	5	3225
tig000001	2631313	2646408	175	86.1	175	57	10	5465
tig000001	2642332	2647569	175	30	174	63	9	2299
tig000001	2700525	2710705	174	57.9	174	49	13	1892
tig000001	2732211	2738764	174	38	172	50	12	1166
tig000001	2732169	2738764	175	37.8	175	55	9	2408
tig000001	2764798	2773675	175	50.5	174	45	13	1362
tig000001	2764400	2766794	174	13.9	174	63	9	1219
tig000001	2794115	2850414	175	319.7	175	44	15	6054
tig000001	2930368	2949564	175	109.8	174	52	12	5196
tig000001	2954411	2966591	175	70	175	49	15	1350
tig000001	2954409	2959346	176	28	176	57	9	1694
tig000001	2964827	3011751	175	267.2	175	44	15	4531
tig000001	2981710	3016041	174	197.5	174	46	14	3973
tig000001	2992241	3018409	175	149.6	176	43	15	1707
tig000001	3009308	3031932	175	130.5	174	47	14	2794
tig000001	3020538	3072880	174	301.1	174	48	13	7227
tig000001	3020530	3098634	175	444.7	175	44	15	5066
tig000001	3040209	3132326	174	523.2	175	43	16	5700
tig000001	3122484	3137659	174	87.3	174	54	12	4057
tig000001	3135109	3200582	175	372.8	175	50	13	12425
tig000001	3139771	3209920	175	401.9	174	48	14	9301
tig000001	3497269	3508579	174	65.1	174	73	5	7336

tig000001	3504768	3508583	175	21.8	176	77	2	2710
tig000001	1102915	1114615	175	66.4	176	52	13	2713
tig000001	1102915	1114615	176	66.4	176	51	13	2833
tig000001	1108898	1118854	175	57	176	62	9	3929
tig000001	1130627	1141069	175	59.4	176	48	13	2209
tig000001	1138883	1141074	176	12.6	176	62	7	1106
tig000001	1141066	1181565	175	230.3	175	44	15	5183
tig000001	1141777	1143836	176	11.8	175	70	5	1249
tig000001	1146204	1188829	175	243.6	174	43	16	3551
tig000001	1188233	1198295	174	56.9	175	44	14	1258
tig000001	1196004	1245688	176	282.3	175	42	16	3127
tig000001	1266875	1268988	175	12.1	174	66	5	1208
tig000001	1278158	1286022	174	45	174	62	7	3764
tig000001	1278206	1349251	175	401.3	176	43	15	7345
tig000001	1393329	1402480	175	51.4	176	44	16	1047
tig000001	1400450	1402204	175	10	174	73	3	1186
tig000001	1783805	1786870	175	17.6	175	74	3	2131
tig000001	2768694	2777484	176	50.2	175	67	6	4742
tig000001	2781587	3281454	175	2857.1	174	46	18	15256
tig000001	2781587	2865925	176	476.4	176	43	16	5699
tig000001	2795012	2798568	174	20.6	174	62	8	1650
tig000001	2888557	2896117	176	43.4	175	65	5	4069
tig000001	2916870	3416869	175	2860.3	174	57	11	120897
tig000001	3267321	3759161	175	2810	175	54	13	68160
tig000001	3759127	3768518	175	53.9	175	68	5	5122
tig000001	3769734	3774394	174	26.8	173	73	3	3148
tig000001	1	24774	175	141.9	175	78	1	18181
tig000001	769463	783243	176	78	176	52	13	3119
tig000001	1041964	1068940	176	153.1	176	50	13	5801
tig000001	1041950	1056653	176	84.1	175	60	9	6239
tig000001	1045198	1062889	175	101.1	174	50	13	3931
tig000001	1079507	1081402	175	10.8	175	69	2	1164
tig000001	1086109	1088544	175	14	174	70	3	1485
tig000001	1086600	1090154	175	20.5	173	55	10	1334
tig000001	1103035	1107533	176	25.7	176	59	7	1952
tig000001	1123239	1125084	175	10.6	176	67	4	1083
tig000001	1126688	1128507	174	10.5	174	67	4	1049
tig000001	1126688	1136624	175	56.4	176	42	14	1166
tig000001	1134363	1147673	175	75.9	175	48	14	2264
tig000001	1139700	1147691	175	46	175	53	10	2329
tig000001	1142330	1172370	175	171	175	43	16	2979
tig000001	1159139	1172242	176	74.4	176	43	17	1054
tig000001	1186309	1189263	176	17	175	64	4	1641
tig000001	1219223	1226556	175	42.3	173	65	5	3919
tig000001	1232434	1256244	175	134.7	176	41	16	1557
tig000001	1252240	1293846	175	237.6	174	43	16	2862
tig000001	1252217	1288109	175	203.6	175	43	15	3454

tig000001	1275348	1288109	176	72.5	174	49	13	2529
tig000001	1270955	1293846	175	129.6	176	47	14	3966
tig000001	1291864	1293846	174	11.4	173	70	5	1198
tig000001	1306916	1320899	175	79.7	175	45	16	1821
tig000001	1306951	1318309	176	64.4	176	43	17	1122
tig000001	1508165	1509978	176	10.4	175	66	4	1042
tig000001	1537317	1540058	174	15.8	174	60	7	1113
tig000001	1540011	1564036	175	136.5	174	44	16	2326
tig000001	1537854	1549577	175	66.9	175	47	14	2144
tig000001	1619044	1621510	175	14.2	173	64	8	1221
tig000001	1713875	1721329	174	42.2	176	45	16	1047
tig000001	1713842	1728848	175	85.5	174	45	14	1914
tig000001	1713890	1720166	175	35.8	175	54	10	2046
tig000001	1728151	1732827	175	26.8	175	64	5	2480
tig000001	1808534	1811157	175	15.2	174	64	6	1423
tig000001	1848913	1850872	176	11.3	176	63	7	1001
tig000001	1850806	1853205	174	13.9	174	67	6	1405
tig000001	2089890	2150828	176	344.4	176	38	17	1255
tig000001	3741386	3748216	175	39.2	175	80	1	5228
tig000001	3748174	3758239	175	57.8	174	81	2	7749
tig000001	1481774	1486582	174	27.6	174	70	4	2948
tig000001	1496539	1499761	175	18.6	174	68	5	1817
tig000001	1591278	1623908	174	186.8	173	43	16	3518
tig000001	1666480	1669503	176	17.6	175	58	8	1294
tig000001	1705985	1709357	174	19.5	174	56	10	1206
tig000001	1725077	1746843	175	123.9	175	43	15	2102
tig000001	1834613	1849177	175	83.7	175	48	15	2111
tig000001	1839724	1871952	174	184.8	173	41	16	1801
tig000001	1866233	1879614	175	76.7	174	51	13	2343
tig000001	1892919	1932702	174	227.4	175	44	16	2752
tig000001	1922913	1934295	176	64.2	176	52	13	1915
tig000001	1927291	1934280	175	39.9	173	55	12	1755
tig000001	2481154	2484523	176	19.4	176	74	4	2261
tig000001	36753	54463	174	101.2	174	44	16	1907
tig000001	43754	63193	175	110.6	175	44	14	1431
tig000001	43809	58862	174	86	174	47	14	2042
tig000001	63947	251209	175	1074	174	45	15	15440
tig000001	63919	250737	174	1064.4	175	43	15	8262
tig000001	226596	293817	175	386	175	44	16	3189
tig000001	248834	286883	176	217.1	176	50	15	6035
tig000001	567399	753523	174	1064	173	38	17	3600
tig000001	1	177124	175	1009.3	175	56	13	37362
tig000001	178737	226434	175	273.1	175	67	7	20007
tig000001	226346	229659	174	19.1	175	76	3	2359
tig000001	352547	360757	175	46.3	176	52	12	2278
tig000001	355859	360698	176	27.8	175	61	8	2205
tig000001	387810	402586	175	83.9	176	42	16	1211

tig000001	399629	402586	175	17	175	69	3	1764
tig000001	1575714	1786190	175	1200.9	174	39	17	4851
tig000001	1811697	1814765	176	17.6	176	68	3	1816
tig000002	103769	106020	175	12.9	176	82	1	1790
tig000002	1458102	1673936	175	1234.6	174	52	15	17716
tig000002	1655701	1684196	175	163.2	175	76	3	19227
tig000002	2071867	2199196	175	719.9	176	44	16	11061
tig000002	2161840	2208485	175	265.9	175	51	13	9131
tig000002	2280119	2323586	175	250.3	172	45	16	2877
tig000002	2299406	2306341	175	39.9	175	65	6	3457
tig000002	2306186	2310730	174	25.7	175	58	9	1838
tig000002	2310792	2323622	175	72.9	175	55	12	3374
tig000002	2336776	2358892	175	127.2	172	56	13	4176
tig000002	2336267	2361009	175	142.2	175	53	14	4160
tig000002	2358865	2361406	175	14.6	175	69	4	1264
tig000002	2366821	2374535	175	44.1	175	55	11	2133
tig000002	2366350	2373886	174	43.5	173	52	14	1299
tig000002	2369528	2375450	174	34	174	49	15	1004
tig000002	2391347	2409881	175	106	175	57	12	5724
tig000002	2410114	2413634	175	20.3	175	71	3	2290
tig000002	2423087	2436339	174	76.6	173	44	16	1039
tig000002	2491083	2522155	175	177.7	174	50	14	4772
tig000002	2491088	2502756	175	67	174	67	7	6001
tig000002	2530179	2533801	176	20.9	176	67	5	2091
tig000002	2543587	2547264	175	21.1	173	70	5	2203
tig000002	2543587	2549297	175	32.3	175	56	11	2048
tig000002	3447377	3512010	175	367.8	175	55	13	12493
tig000002	391553	400230	176	49.7	175	61	9	3613
tig000002	2931477	3136277	175	1168.1	175	54	13	35896
tig000002	1	19209	175	109.6	175	57	13	3702
tig000002	3300616	3510138	176	1178	176	39	15	9432
tig000002	3300983	3510202	175	1182.3	175	39	16	10317
tig000002	3525183	3623938	175	559	176	44	16	9889
tig000002	3548459	3609912	176	350.9	174	43	17	3916
tig000002	3619220	3626079	176	38.9	173	55	12	1795
tig000002	3633299	3676832	176	246.6	176	43	16	3562
tig000002	3649361	3654780	175	30.7	176	65	7	2397
tig000002	3649653	3676879	176	154.3	176	48	13	5038
tig000002	3672576	3676881	174	24.6	175	72	3	2799
tig000002	1351045	1364736	175	78.9	174	45	17	1483
tig000002	2335526	2401550	174	375.4	175	42	16	3046
tig000002	2335573	2397354	175	352.9	174	45	15	5532
tig000002	2437621	2439737	175	12.3	174	64	6	1123
tig000002	2452470	2459931	175	43	175	57	9	2903
tig000002	2449628	2479020	174	167.7	173	44	15	2804
tig000002	2476616	2747390	175	1543	175	44	15	18287
tig000002	2592917	2747251	174	922.8	162	46	15	19349

tig000002	3278008	3336988	176	338.2	175	39	18	1504
tig000002	3298040	3300852	175	16.2	174	66	4	1565
tig000002	3551425	3553286	175	10.7	175	72	3	1223
tig000002	3557781	3563658	175	33.3	175	55	12	1740
tig000002	3602755	3621296	175	105.3	174	44	15	1412
tig000002	3597731	3615397	175	100.2	175	41	16	1015
tig000002	3613822	3620621	176	39	176	63	6	3316
tig000002	3620624	3636268	175	90	175	90	1	13998
tig000002	3621065	3636268	174	87.4	175	90	1	13615
tig000002	1418050	1445200	175	155.5	173	42	16	1029
tig000002	1419360	1428028	175	49.9	174	63	8	4118
tig000002	1753394	1757217	175	21.9	175	58	9	1442
tig000002	3363848	3367342	175	20	175	78	1	2548
tig000002	3367268	3375048	175	44.7	173	74	3	4928
tig000002	978016	1008346	175	171.2	176	43	17	1790
tig000002	990835	1068278	175	440	175	41	17	2736
tig000002	1144819	1150904	174	35.1	174	64	7	3178
tig000002	1141721	1158196	175	94.5	175	49	13	2490
tig000002	1164052	1176950	175	73.8	173	45	15	1943
tig000002	1176859	1188060	175	64.3	174	49	14	1833
tig000002	1185261	1188059	176	16	173	65	8	1416
tig000002	1202731	1207158	175	25.7	172	58	9	1865
tig000002	1205376	1272804	175	385.7	174	41	17	1556
tig000002	1220119	1223798	174	21.5	167	57	12	1184
tig000002	1231545	1239443	174	45.2	173	47	15	1185
tig000002	1240737	1290070	175	281.1	175	43	16	2891
tig000002	1247721	1290070	176	240.5	176	43	15	3649
tig000002	1251017	1290070	175	227.6	170	45	15	4312
tig000002	1317584	1462797	175	829.7	174	43	17	7030
tig000002	1339500	1387908	174	276.6	175	48	14	6384
tig000002	2489428	2493009	175	20.1	178	56	13	1273
tig000002	132551	280658	175	847.9	174	43	16	6017
tig000002	132747	261688	175	736.2	174	43	16	7476
tig000002	229647	277869	175	275.2	174	50	13	8487
tig000002	521847	621447	175	568.8	174	43	16	5558
tig000002	520634	545472	176	141.1	176	53	12	5331
tig000002	556446	562041	174	32.1	174	53	12	1247
tig000002	592888	617909	174	143.4	173	45	14	3509
tig000002	671507	687538	174	92.3	174	46	15	1953
tig000002	683875	704888	175	120.4	174	44	15	2987
tig000002	716567	718795	175	12.8	173	62	7	1082
tig000002	767003	806170	174	225.8	174	43	17	2819
tig000002	826662	834032	174	42.3	175	49	12	1956
tig000002	859548	877066	174	100.4	175	46	15	2108
tig000002	899935	902728	175	16	175	66	4	1580
tig000002	919993	990905	174	403.9	175	48	15	10527
tig000002	920014	949417	176	167.2	176	51	13	6684

tig000002	913626	999207	175	488.2	174	44	15	6075
tig000002	996158	999570	175	19.6	175	69	5	2018
tig000002	1479190	1493303	175	79.6	176	45	15	1829
tig000002	1486454	1493215	175	38.6	175	52	12	1876
tig000002	1519293	1526867	174	44.2	171	46	15	1038
tig000002	2853118	2900027	175	267.3	175	45	16	2987
tig000002	2855141	2884037	175	163.2	176	47	14	3774
tig000003	1288077	1290627	175	14.9	175	66	7	1423
