# Supplementary material for: A high-quality genome provides insights into the new taxonomic status and genomic characteristics of Cladopus chinensis (Podostemaceae)
Source: Hortic Res. 2020 Apr 1;7:46. doi: 10.1038/s41438-020-0269-5 (PMC7109043; doi:10.1038/s41438-020-0269-5)
Supplement: Supplementary file 7 — Table S9. KEGG enrichment of the expansion familes genes identified in the C. chinensis [file 41438_2020_269_MOESM7_ESM.pdf]

OG	KEGG class	KEGG sub class	Description	KEGG ID	Number of
OG0000113	Metabolism	Biosynthesis of other secondary	Phenylpropanoid biosynthesis	ko00940	32
OG0000212	Metabolism	Energy metabolism	Photosynthesis - antenna proteins	ko00196	69
OG0000396	Cellular Processes	Transport and catabolism	Peroxisome	ko04146	15
OG0000396	Metabolism	Lipid metabolism	Cutin, suberine and wax	ko00073	15
OG0000396	Organismal Systems	Aging	Longevity regulating pathway -	ko04212	15
OG0002529	Metabolism	Metabolism of terpenoids and	Carotenoid biosynthesis	ko00906	8
OG0002619	Metabolism	Carbohydrate metabolism	Pentose and glucuronate	ko00040	8
OG0003225	Environmental Information	Signal transduction	Two-component system	ko02020	1
OG0003225	Metabolism	Carbohydrate metabolism	Starch and sucrose metabolism	ko00500	2
OG0003546	Environmental Information	Signal transduction	Two-component system	ko02020	7
OG0003546	Human Diseases	Cancers: Overview	Pathways in cancer	ko05200	10
OG0003546	Human Diseases	Cancers: Specific types	Renal cell carcinoma	ko05211	10
OG0003546	Human Diseases	Endocrine and metabolic diseases	Cushing syndrome	ko04934	10
OG0003546	Metabolism	Amino acid metabolism	Alanine, aspartate and glutamate	ko00250	1
OG0003546	Metabolism	Carbohydrate metabolism	Citrate cycle (TCA cycle)	ko00020	10
OG0003546	Metabolism	Carbohydrate metabolism	Pyruvate metabolism	ko00620	17
OG0003546	Metabolism	Energy metabolism	Carbon fixation pathways in	ko00720	10
OG0003576	Cellular Processes	Cellular community - prokaryotes	Quorum sensing	ko02024	6
OG0003576	Metabolism	Carbohydrate metabolism	Pentose and glucuronate	ko00040	6
OG0003741	Metabolism	Metabolism of terpenoids and	Diterpenoid biosynthesis	ko00904	8
OG0003795	Metabolism	Carbohydrate metabolism	Galactose metabolism	ko00052	8
OG0003795	Metabolism	Carbohydrate metabolism	Glycolysis / Gluconeogenesis	ko00010	8
OG0003956	Organismal Systems	Aging	Longevity regulating pathway -	ko04213	5
OG0004759	Metabolism	Amino acid metabolism	Lysine biosynthesis	ko00300	6
OG0005302	Human Diseases	Cancers: Overview	Transcriptional misregulation in	ko05202	1
OG0005302	Metabolism	Glycan biosynthesis and metabolism	Glycosaminoglycan biosynthesis -	ko00533	1
OG0005302	Metabolism	Glycan biosynthesis and metabolism	N-Glycan biosynthesis	ko00510	1
OG0005302	Metabolism	Glycan biosynthesis and metabolism	Various types of N-glycan	ko00513	1
OG0005521	Human Diseases	Infectious diseases: Parasitic	Amoebiasis	ko05146	7
OG0005788	Human Diseases	Neurodegenerative diseases	Parkinson disease	ko05012	1
OG0005788	Metabolism	Carbohydrate metabolism	Starch and sucrose metabolism	ko00500	1
OG0005788	Metabolism	Energy metabolism	Oxidative phosphorylation	ko00190	15
OG0005788	Organismal Systems	Environmental adaptation	Thermogenesis	ko04714	1

OG0005788	Organismal Systems	Nervous system	Retrograde endocannabinoid	ko04723	1
OG0005830	Metabolism	Energy metabolism	Nitrogen metabolism	ko00910	7
OG0005865	Metabolism	Amino acid metabolism	Glycine, serine and threonine	ko00260	6
OG0005865	Metabolism	Amino acid metabolism	Valine, leucine and isoleucine	ko00290	6
OG0006103	Cellular Processes	Cell growth and death	Apoptosis - fly	ko04214	5
OG0006185	Metabolism	Lipid metabolism	Cutin, suberine and wax	ko00073	1
OG0006333	Genetic Information	Translation	Ribosome	ko03010	12
OG0006634	Human Diseases	Neurodegenerative diseases	Parkinson disease	ko05012	11
OG0006634	Metabolism	Energy metabolism	Oxidative phosphorylation	ko00190	27
OG0006634	Organismal Systems	Environmental adaptation	Thermogenesis	ko04714	11
OG0006634	Organismal Systems	Nervous system	Retrograde endocannabinoid	ko04723	11
OG0006916	Human Diseases	Neurodegenerative diseases	Parkinson disease	ko05012	5
OG0006916	Metabolism	Energy metabolism	Oxidative phosphorylation	ko00190	24
OG0006916	Organismal Systems	Environmental adaptation	Thermogenesis	ko04714	5
OG0006916	Organismal Systems	Nervous system	Retrograde endocannabinoid	ko04723	5
OG0007170	Cellular Processes	Cellular community - prokaryotes	Biofilm formation - Escherichia	ko02026	3
OG0007170	Cellular Processes	Cellular community - prokaryotes	Biofilm formation - Vibrio	ko05111	3
OG0007170	Environmental Information	Signal transduction	Two-component system	ko02020	1
OG0007170	Genetic Information	Replication and repair	Base excision repair	ko03410	2
OG0007170	Human Diseases	Drug resistance: Antimicrobial	Vancomycin resistance	ko01502	1
OG0007170	Metabolism	Glycan biosynthesis and metabolism	Peptidoglycan biosynthesis	ko00550	1
OG0007216	Metabolism	Carbohydrate metabolism	Pentose and glucuronate	ko00040	6
OG0007476	Genetic Information	Translation	Ribosome	ko03010	8
OG0007590	Human Diseases	Drug resistance: Antimicrobial	Vancomycin resistance	ko01502	6
OG0007590	Metabolism	Glycan biosynthesis and metabolism	Peptidoglycan biosynthesis	ko00550	6
OG0007749	Environmental Information	Membrane transport	ABC transporters	ko02010	3
OG0007839	Human Diseases	Neurodegenerative diseases	Alzheimer disease	ko05010	1
OG0007839	Human Diseases	Neurodegenerative diseases	Huntington disease	ko05016	1
OG0007839	Human Diseases	Neurodegenerative diseases	Parkinson disease	ko05012	1
OG0007839	Metabolism	Energy metabolism	Oxidative phosphorylation	ko00190	5
OG0007839	Metabolism	Energy metabolism	Photosynthesis	ko00195	4
OG0007839	Organismal Systems	Environmental adaptation	Thermogenesis	ko04714	1
OG0007841	Genetic Information	Translation	Ribosome	ko03010	9
OG0007841	Metabolism	Carbohydrate metabolism	Glyoxylate and dicarboxylate	ko00630	1

OG0007841	Metabolism	Energy metabolism	Carbon fixation in photosynthetic	ko00710	1
OG0007870	Cellular Processes	Cell growth and death	Apoptosis - fly	ko04214	6
OG0007880	Cellular Processes	Cellular community - prokaryotes	Quorum sensing	ko02024	4
OG0007880	Cellular Processes	Transport and catabolism	Phagosome	ko04145	2
OG0007880	Environmental Information	Membrane transport	Bacterial secretion system	ko03070	4
OG0007880	Genetic Information	Folding, sorting and degradation	Protein export	ko03060	6
OG0007880	Genetic Information	Folding, sorting and degradation	Protein processing in endoplasmic	ko04141	2
OG0007880	Human Diseases	Infectious diseases: Bacterial	Vibrio cholerae infection	ko05110	2
OG0008175	Metabolism	Carbohydrate metabolism	Glyoxylate and dicarboxylate	ko00630	10
OG0008175	Metabolism	Energy metabolism	Carbon fixation in photosynthetic	ko00710	10
OG0008350	Cellular Processes	Cell growth and death	Cell cycle - Caulobacter	ko04112	5
OG0008350	Organismal Systems	Aging	Longevity regulating pathway -	ko04212	5
OG0008460	Human Diseases	Cancers: Overview	Chemical carcinogenesis	ko05204	4
OG0008460	Metabolism	Amino acid metabolism	Tyrosine metabolism	ko00350	4
OG0008460	Metabolism	Carbohydrate metabolism	Glycolysis / Gluconeogenesis	ko00010	4
OG0008460	Metabolism	Energy metabolism	Methane metabolism	ko00680	4
OG0008460	Metabolism	Lipid metabolism	Fatty acid degradation	ko00071	4
OG0008460	Metabolism	Metabolism of cofactors and vitamins	Retinol metabolism	ko00830	4
OG0008460	Metabolism	Xenobiotics biodegradation and	Chloroalkane and chloroalkene	ko00625	4
OG0008460	Metabolism	Xenobiotics biodegradation and	Drug metabolism - cytochrome P450	ko00982	4
OG0008460	Metabolism	Xenobiotics biodegradation and	Metabolism of xenobiotics by	ko00980	4
OG0008460	Metabolism	Xenobiotics biodegradation and	Naphthalene degradation	ko00626	4
OG0008548	Human Diseases	Endocrine and metabolic diseases	Non-alcoholic fatty liver disease	ko04932	2
OG0008548	Human Diseases	Neurodegenerative diseases	Alzheimer disease	ko05010	2
OG0008548	Human Diseases	Neurodegenerative diseases	Huntington disease	ko05016	2
OG0008548	Human Diseases	Neurodegenerative diseases	Parkinson disease	ko05012	2
OG0008548	Metabolism	Energy metabolism	Oxidative phosphorylation	ko00190	17
OG0008548	Organismal Systems	Environmental adaptation	Thermogenesis	ko04714	2
OG0008548	Organismal Systems	Nervous system	Retrograde endocannabinoid	ko04723	2
OG0008710	Genetic Information	Folding, sorting and degradation	Protein processing in endoplasmic	ko04141	4
OG0008747	Cellular Processes	Cell growth and death	Cell cycle - Caulobacter	ko04112	1
OG0008747	Organismal Systems	Aging	Longevity regulating pathway -	ko04212	1
OG0008915	Metabolism	Lipid metabolism	Cutin, suberine and wax	ko00073	4
OG0009240	Metabolism	Lipid metabolism	Glycerophospholipid metabolism	ko00564	2

OG0009240	Metabolism	Metabolism of cofactors and vitamins	Lipoic acid metabolism	ko00785	1
OG0009331	Metabolism	Metabolism of terpenoids and	Brassinosteroid biosynthesis	ko00905	4
OG0009384	Genetic Information	Translation	Aminoacyl-tRNA biosynthesis	ko00970	8
OG0009664	Genetic Information	Translation	Ribosome	ko03010	5
OG0009687	Human Diseases	Infectious diseases: Bacterial	Epithelial cell signaling in	ko05120	3
OG0009687	Metabolism	Amino acid metabolism	Arginine biosynthesis	ko00220	5
OG0009687	Metabolism	Nucleotide metabolism	Purine metabolism	ko00230	5
OG0009687	Metabolism	Xenobiotics biodegradation and	Atrazine degradation	ko00791	5
OG0009747	Genetic Information	Translation	Ribosome	ko03010	4
OG0010112	Metabolism	Energy metabolism	Photosynthesis	ko00195	4
OG0010161	Metabolism	Carbohydrate metabolism	Starch and sucrose metabolism	ko00500	2
OG0010188	Cellular Processes	Cell growth and death	p53 signaling pathway	ko04115	4
OG0010188	Genetic Information	Folding, sorting and degradation	Ubiquitin mediated proteolysis	ko04120	4
OG0010188	Organismal Systems	Environmental adaptation	Circadian rhythm - plant	ko04712	4
OG0010196	Genetic Information	Replication and repair	Base excision repair	ko03410	4
OG0010196	Genetic Information	Replication and repair	DNA replication	ko03030	4
OG0010196	Genetic Information	Replication and repair	Homologous recombination	ko03440	4
OG0010196	Genetic Information	Replication and repair	Mismatch repair	ko03430	4
OG0010196	Genetic Information	Replication and repair	Nucleotide excision repair	ko03420	4
OG0010196	Metabolism	Nucleotide metabolism	Purine metabolism	ko00230	4
OG0010196	Metabolism	Nucleotide metabolism	Pyrimidine metabolism	ko00240	4
OG0010322	Metabolism	Metabolism of cofactors and vitamins	Porphyrin and chlorophyll	ko00860	4
OG0010426	Human Diseases	Neurodegenerative diseases	Parkinson disease	ko05012	5
OG0010426	Metabolism	Energy metabolism	Oxidative phosphorylation	ko00190	13
OG0010426	Organismal Systems	Environmental adaptation	Thermogenesis	ko04714	5
OG0010426	Organismal Systems	Nervous system	Retrograde endocannabinoid	ko04723	5
OG0010457	Metabolism	Amino acid metabolism	Arginine biosynthesis	ko00220	4
OG0010483	Human Diseases	Cancers: Overview	Choline metabolism in cancer	ko05231	5
OG0010488	Genetic Information	Translation	Aminoacyl-tRNA biosynthesis	ko00970	4
OG0010565	Metabolism	Energy metabolism	Photosynthesis	ko00195	3
OG0010725	Metabolism	Energy metabolism	Photosynthesis	ko00195	3
OG0010810	Metabolism	Amino acid metabolism	Phenylalanine, tyrosine and	ko00400	6
OG0010844	Genetic Information	Translation	RNA transport	ko03013	3
OG0011023	Cellular Processes	Cellular community - prokaryotes	Biofilm formation - Escherichia	ko02026	4

OG0011023	Cellular Processes	Cellular community - prokaryotes	Biofilm formation - Vibrio	ko05111	1
OG0011023	Cellular Processes	Cellular community - prokaryotes	Quorum sensing	ko02024	6
OG0011023	Environmental Information	Signal transduction	Two-component system	ko02020	47
OG0011023	Human Diseases	Drug resistance: Antimicrobial	Cationic antimicrobial peptide	ko01503	9
OG0011023	Human Diseases	Infectious diseases: Bacterial	Epithelial cell signaling in	ko05120	1
OG0011145	Environmental Information	Signal transduction	Two-component system	ko02020	12
OG0011145	Genetic Information	Translation	RNA transport	ko03013	4
OG0011145	Metabolism	Energy metabolism	Photosynthesis	ko00195	12
OG0011146	Metabolism	Amino acid metabolism	Glycine, serine and threonine	ko00260	3
OG0011146	Metabolism	Amino acid metabolism	Phenylalanine, tyrosine and	ko00400	3
OG0011146	Metabolism	Carbohydrate metabolism	Propanoate metabolism	ko00640	11
OG0011146	Metabolism	Carbohydrate metabolism	Pyruvate metabolism	ko00620	11
OG0011146	Metabolism	Energy metabolism	Carbon fixation pathways in	ko00720	11
OG0011146	Metabolism	Energy metabolism	Photosynthesis	ko00195	1
OG0011146	Metabolism	Lipid metabolism	Fatty acid biosynthesis	ko00061	11
OG0011152	Metabolism	Amino acid metabolism	Tyrosine metabolism	ko00350	8
OG0011152	Metabolism	Xenobiotics biodegradation and	Styrene degradation	ko00643	8
OG0011232	Metabolism	Energy metabolism	Oxidative phosphorylation	ko00190	4
OG0011232	Metabolism	Energy metabolism	Photosynthesis	ko00195	4
OG0011438	Genetic Information	Translation	Aminoacyl-tRNA biosynthesis	ko00970	3
OG0011438	Human Diseases	Infectious diseases: Bacterial	Bacterial invasion of epithelial	ko05100	1
OG0011528	Metabolism	Carbohydrate metabolism	Pentose and glucuronate	ko00040	4
OG0011729	Metabolism	Amino acid metabolism	Cysteine and methionine metabolism	ko00270	5
OG0011729	Metabolism	Amino acid metabolism	Glycine, serine and threonine	ko00260	5
OG0011729	Metabolism	Metabolism of other amino acids	Selenocompound metabolism	ko00450	5
OG0011730	Organismal Systems	Endocrine system	Aldosterone synthesis and	ko04925	4
OG0011730	Organismal Systems	Endocrine system	Oxytocin signaling pathway	ko04921	4
OG0011731	Genetic Information	Folding, sorting and degradation	RNA degradation	ko03018	5
OG0011855	Cellular Processes	Cell growth and death	p53 signaling pathway	ko04115	3
OG0011855	Genetic Information	Folding, sorting and degradation	Ubiquitin mediated proteolysis	ko04120	3
OG0011855	Genetic Information	Replication and repair	Nucleotide excision repair	ko03420	3
OG0011855	Human Diseases	Cancers: Overview	Pathways in cancer	ko05200	3
OG0011855	Human Diseases	Cancers: Overview	Transcriptional misregulation in	ko05202	3
OG0011855	Human Diseases	Cancers: Specific types	Basal cell carcinoma	ko05217	3

OG0011855	Human Diseases	Cancers: Specific types	Breast cancer	ko05224	3
OG0011855	Human Diseases	Cancers: Specific types	Chronic myeloid leukemia	ko05220	3
OG0011855	Human Diseases	Cancers: Specific types	Colorectal cancer	ko05210	3
OG0011855	Human Diseases	Cancers: Specific types	Endometrial cancer	ko05213	3
OG0011855	Human Diseases	Cancers: Specific types	Gastric cancer	ko05226	3
OG0011855	Human Diseases	Cancers: Specific types	Glioma	ko05214	3
OG0011855	Human Diseases	Cancers: Specific types	Hepatocellular carcinoma	ko05225	3
OG0011855	Human Diseases	Cancers: Specific types	Melanoma	ko05218	3
OG0011855	Human Diseases	Cancers: Specific types	Non-small cell lung cancer	ko05223	3
OG0011855	Human Diseases	Cancers: Specific types	Pancreatic cancer	ko05212	3
OG0011855	Human Diseases	Cancers: Specific types	Small cell lung cancer	ko05222	3
OG0011855	Human Diseases	Cancers: Specific types	Thyroid cancer	ko05216	3
OG0011855	Human Diseases	Infectious diseases: Viral	Epstein-Barr virus infection	ko05169	3
OG0011855	Human Diseases	Infectious diseases: Viral	Hepatitis B	ko05161	3
OG0012085	Genetic Information	Translation	RNA transport	ko03013	1
OG0012504	Environmental Information	Signal transduction	Two-component system	ko02020	6
OG0012504	Metabolism	Energy metabolism	Photosynthesis	ko00195	9
OG0012663	Genetic Information	Transcription	Basal transcription factors	ko03022	3
OG0012663	Human Diseases	Cancers: Overview	Transcriptional misregulation in	ko05202	3
OG0012761	Genetic Information	Translation	Ribosome	ko03010	8
OG0013268	Metabolism	Energy metabolism	Photosynthesis	ko00195	5
OG0013270	Genetic Information	Translation	Ribosome	ko03010	7
OG0013295	Genetic Information	Translation	RNA transport	ko03013	2
OG0013329	Cellular Processes	Transport and catabolism	Endocytosis	ko04144	2
OG0013338	Metabolism	Metabolism of cofactors and vitamins	Porphyrin and chlorophyll	ko00860	2
OG0013359	Human Diseases	Neurodegenerative diseases	Parkinson disease	ko05012	1
OG0013359	Metabolism	Energy metabolism	Oxidative phosphorylation	ko00190	1
OG0013359	Organismal Systems	Environmental adaptation	Thermogenesis	ko04714	1
OG0013359	Organismal Systems	Nervous system	Retrograde endocannabinoid	ko04723	1
OG0013486	Metabolism	Energy metabolism	Oxidative phosphorylation	ko00190	5
OG0013487	Metabolism	Energy metabolism	Photosynthesis	ko00195	15
OG0013564	Human Diseases	Neurodegenerative diseases	Parkinson disease	ko05012	10
OG0013564	Metabolism	Energy metabolism	Oxidative phosphorylation	ko00190	10
OG0013564	Organismal Systems	Environmental adaptation	Thermogenesis	ko04714	10

OG0013564	Organismal Systems	Nervous system	Retrograde endocannabinoid	ko04723	10
OG0013698	Environmental Information	Signal transduction	Two-component system	ko02020	3
OG0013698	Human Diseases	Endocrine and metabolic diseases	Non-alcoholic fatty liver disease	ko04932	3
OG0013698	Human Diseases	Neurodegenerative diseases	Alzheimer disease	ko05010	3
OG0013698	Human Diseases	Neurodegenerative diseases	Huntington disease	ko05016	3
OG0013698	Human Diseases	Neurodegenerative diseases	Parkinson disease	ko05012	3
OG0013698	Metabolism	Energy metabolism	Oxidative phosphorylation	ko00190	9
OG0013698	Metabolism	Energy metabolism	Photosynthesis	ko00195	4
OG0013698	Organismal Systems	Circulatory system	Cardiac muscle contraction	ko04260	3
OG0013698	Organismal Systems	Environmental adaptation	Thermogenesis	ko04714	3
OG0013763	Metabolism	Biosynthesis of other secondary	Aflatoxin biosynthesis	ko00254	12
OG0013763	Metabolism	Biosynthesis of other secondary	Flavone and flavonol biosynthesis	ko00944	6
OG0013763	Metabolism	Biosynthesis of other secondary	Isoflavonoid biosynthesis	ko00943	6
OG0013763	Metabolism	Biosynthesis of other secondary	Phenylpropanoid biosynthesis	ko00940	6
OG0013763	Metabolism	Biosynthesis of other secondary	Staurosporine biosynthesis	ko00404	6
OG0013907	Metabolism	Energy metabolism	Photosynthesis	ko00195	6
OG0013908	Genetic Information	Translation	Ribosome	ko03010	3
OG0013909	Genetic Information	Transcription	RNA polymerase	ko03020	3
OG0013909	Metabolism	Nucleotide metabolism	Purine metabolism	ko00230	3
OG0013909	Metabolism	Nucleotide metabolism	Pyrimidine metabolism	ko00240	3
OG0013910	Metabolism	Energy metabolism	Oxidative phosphorylation	ko00190	2
OG0013910	Metabolism	Energy metabolism	Photosynthesis	ko00195	2
OG0013912	Genetic Information	Translation	Ribosome	ko03010	6
OG0013913	Genetic Information	Translation	Ribosome	ko03010	4
OG0013914	Metabolism	Amino acid metabolism	Alanine, aspartate and glutamate	ko00250	4
OG0013914	Metabolism	Amino acid metabolism	Arginine biosynthesis	ko00220	4
OG0013971	Metabolism	Biosynthesis of other secondary	Monobactam biosynthesis	ko00261	2
OG0013971	Metabolism	Carbohydrate metabolism	Pentose phosphate pathway	ko00030	12
OG0013971	Metabolism	Energy metabolism	Carbon fixation in photosynthetic	ko00710	6
OG0013971	Metabolism	Energy metabolism	Sulfur metabolism	ko00920	2
OG0013971	Metabolism	Metabolism of other amino acids	Selenocompound metabolism	ko00450	2
OG0013971	Metabolism	Nucleotide metabolism	Purine metabolism	ko00230	2
OG0013974	Metabolism	Metabolism of cofactors and vitamins	Porphyrin and chlorophyll	ko00860	3
OG0013974	Metabolism	Metabolism of terpenoids and	Terpenoid backbone biosynthesis	ko00900	3

OG0014100	Metabolism	Energy metabolism	Photosynthesis	ko00195	3
OG0014102	Genetic Information	Translation	Ribosome	ko03010	3
OG0014327	Metabolism	Energy metabolism	Oxidative phosphorylation	ko00190	3
OG0014327	Metabolism	Energy metabolism	Photosynthesis	ko00195	3
OG0014328	Metabolism	Energy metabolism	Oxidative phosphorylation	ko00190	4
OG0014328	Metabolism	Energy metabolism	Photosynthesis	ko00195	4
OG0014329	Genetic Information	Translation	Ribosome	ko03010	4
OG0014330	Environmental Information	Signal transduction	Two-component system	ko02020	4
OG0014375	Metabolism	Energy metabolism	Oxidative phosphorylation	ko00190	6
OG0014543	Environmental Information	Signal transduction	Two-component system	ko02020	3
OG0014543	Human Diseases	Endocrine and metabolic diseases	Non-alcoholic fatty liver disease	ko04932	3
OG0014543	Human Diseases	Neurodegenerative diseases	Alzheimer disease	ko05010	3
OG0014543	Human Diseases	Neurodegenerative diseases	Huntington disease	ko05016	3
OG0014543	Human Diseases	Neurodegenerative diseases	Parkinson disease	ko05012	3
OG0014543	Metabolism	Energy metabolism	Oxidative phosphorylation	ko00190	3
OG0014543	Metabolism	Energy metabolism	Photosynthesis	ko00195	1
OG0014543	Organismal Systems	Circulatory system	Cardiac muscle contraction	ko04260	3
OG0014543	Organismal Systems	Environmental adaptation	Thermogenesis	ko04714	3
OG0014587	Metabolism	Energy metabolism	Oxidative phosphorylation	ko00190	5
OG0014612	Environmental Information	Signal transduction	Ras signaling pathway	ko04014	1
OG0014781	Metabolism	Energy metabolism	Photosynthesis	ko00195	2
OG0014782	Genetic Information	Translation	Ribosome	ko03010	2
OG0014783	Genetic Information	Translation	Ribosome	ko03010	2
OG0014785	Metabolism	Energy metabolism	Oxidative phosphorylation	ko00190	3
OG0014826	Genetic Information	Translation	Ribosome	ko03010	5
OG0014827	Human Diseases	Endocrine and metabolic diseases	Non-alcoholic fatty liver disease	ko04932	5
OG0014827	Human Diseases	Neurodegenerative diseases	Alzheimer disease	ko05010	5
OG0014827	Human Diseases	Neurodegenerative diseases	Huntington disease	ko05016	5
OG0014827	Human Diseases	Neurodegenerative diseases	Parkinson disease	ko05012	5
OG0014827	Metabolism	Energy metabolism	Oxidative phosphorylation	ko00190	5
OG0014827	Organismal Systems	Environmental adaptation	Thermogenesis	ko04714	5
OG0014827	Organismal Systems	Nervous system	Retrograde endocannabinoid	ko04723	5
OG0015130	Metabolism	Biosynthesis of other secondary	Phenylpropanoid biosynthesis	ko00940	7
OG0015130	Metabolism	Carbohydrate metabolism	Starch and sucrose metabolism	ko00500	7

OG0015130	Metabolism	Metabolism of other amino acids	Cyanoamino acid metabolism	ko00460	7
OG0015453	Metabolism	Amino acid metabolism	Valine, leucine and isoleucine	ko00290	5
OG0015453	Metabolism	Carbohydrate metabolism	Butanoate metabolism	ko00650	5
OG0015453	Metabolism	Carbohydrate metabolism	C5-Branched dibasic acid	ko00660	5
OG0015453	Metabolism	Metabolism of cofactors and vitamins	Pantothenate and CoA biosynthesis	ko00770	5
OG0015901	Genetic Information	Translation	Aminoacyl-tRNA biosynthesis	ko00970	6
OG0015903	Metabolism	Biosynthesis of other secondary	Phenylpropanoid biosynthesis	ko00940	4
OG0015903	Metabolism	Glycan biosynthesis and metabolism	Peptidoglycan biosynthesis	ko00550	4
OG0015903	Metabolism	Metabolism of cofactors and vitamins	Ubiquinone and other terpenoid-	ko00130	4
OG0015903	Metabolism	Xenobiotics biodegradation and	Aminobenzoate degradation	ko00627	4
OG0016357	Cellular Processes	Cell growth and death	Apoptosis	ko04210	2
OG0016357	Cellular Processes	Cellular community - eukaryotes	Gap junction	ko04540	2
OG0016357	Cellular Processes	Cellular community - eukaryotes	Tight junction	ko04530	2
OG0016357	Cellular Processes	Transport and catabolism	Phagosome	ko04145	2
OG0016357	Human Diseases	Infectious diseases: Bacterial	Pathogenic Escherichia coli	ko05130	2
OG0016362	Metabolism	Carbohydrate metabolism	Starch and sucrose metabolism	ko00500	4
OG0016390	Cellular Processes	Transport and catabolism	Endocytosis	ko04144	2
OG0016390	Environmental Information	Signal transduction	MAPK signaling pathway	ko04010	2
OG0016390	Genetic Information	Folding, sorting and degradation	Protein processing in endoplasmic	ko04141	2
OG0016390	Genetic Information	Transcription	Spliceosome	ko03040	2
OG0016390	Human Diseases	Infectious diseases: Bacterial	Legionellosis	ko05134	2
OG0016390	Human Diseases	Infectious diseases: Parasitic	Toxoplasmosis	ko05145	2
OG0016390	Human Diseases	Infectious diseases: Viral	Influenza A	ko05164	2
OG0016390	Human Diseases	Infectious diseases: Viral	Measles	ko05162	2
OG0016390	Organismal Systems	Aging	Longevity regulating pathway -	ko04213	2
OG0016390	Organismal Systems	Endocrine system	Estrogen signaling pathway	ko04915	2
OG0016390	Organismal Systems	Immune system	Antigen processing and	ko04612	2
OG0016914	Cellular Processes	Cellular community - eukaryotes	Gap junction	ko04540	3
OG0016914	Cellular Processes	Transport and catabolism	Phagosome	ko04145	3
OG0016914	Human Diseases	Infectious diseases: Bacterial	Pathogenic Escherichia coli	ko05130	3
OG0017007	Environmental Information	Signal transduction	MAPK signaling pathway	ko04010	3
OG0017007	Environmental Information	Signal transduction	NF-kappa B signaling pathway	ko04064	3
OG0017007	Human Diseases	Infectious diseases: Bacterial	Pertussis	ko05133	3
OG0017007	Human Diseases	Infectious diseases: Bacterial	Tuberculosis	ko05152	3

OG0017007	Human Diseases	Infectious diseases: Parasitic	Chagas disease (American	ko05142	3
OG0017007	Human Diseases	Infectious diseases: Parasitic	Leishmaniasis	ko05140	3
OG0017007	Human Diseases	Infectious diseases: Parasitic	Toxoplasmosis	ko05145	3
OG0017007	Human Diseases	Infectious diseases: Viral	Epstein-Barr virus infection	ko05169	3
OG0017007	Human Diseases	Infectious diseases: Viral	Human immunodeficiency virus 1	ko05170	3
OG0017007	Human Diseases	Infectious diseases: Viral	Measles	ko05162	3
OG0017007	Organismal Systems	Immune system	Toll and Imd signaling pathway	ko04624	3
OG0017007	Organismal Systems	Immune system	Toll-like receptor signaling	ko04620	3
OG0017007	Organismal Systems	Nervous system	Neurotrophin signaling pathway	ko04722	3
OG0017009	Cellular Processes	Cell growth and death	Apoptosis	ko04210	5
OG0017009	Cellular Processes	Transport and catabolism	Autophagy - animal	ko04140	3
OG0017009	Cellular Processes	Transport and catabolism	Lysosome	ko04142	5
OG0017009	Cellular Processes	Transport and catabolism	Phagosome	ko04145	5
OG0017009	Human Diseases	Cancers: Overview	Proteoglycans in cancer	ko05205	3
OG0017009	Human Diseases	Cardiovascular diseases	Fluid shear stress and	ko05418	3
OG0017009	Human Diseases	Immune diseases	Rheumatoid arthritis	ko05323	3
OG0017009	Human Diseases	Infectious diseases: Bacterial	Tuberculosis	ko05152	2
OG0017009	Organismal Systems	Immune system	Antigen processing and	ko04612	5
OG0017733	Metabolism	Amino acid metabolism	Glycine, serine and threonine	ko00260	5
OG0017733	Metabolism	Energy metabolism	Methane metabolism	ko00680	5
OG0017733	Metabolism	Metabolism of cofactors and vitamins	Vitamin B6 metabolism	ko00750	5
OG0017734	Metabolism	Amino acid metabolism	Alanine, aspartate and glutamate	ko00250	4
OG0017734	Metabolism	Nucleotide metabolism	Purine metabolism	ko00230	4
OG0018773	Metabolism	Glycan biosynthesis and metabolism	Glycosylphosphatidylinositol	ko00563	2
OG0018790	Environmental Information	Membrane transport	ABC transporters	ko02010	2
OG0018792	Metabolism	Amino acid metabolism	Alanine, aspartate and glutamate	ko00250	6
OG0018792	Metabolism	Amino acid metabolism	Lysine degradation	ko00310	3
OG0018792	Metabolism	Amino acid metabolism	Tyrosine metabolism	ko00350	3
OG0018792	Metabolism	Amino acid metabolism	Valine, leucine and isoleucine	ko00290	1
OG0018792	Metabolism	Carbohydrate metabolism	Butanoate metabolism	ko00650	7
OG0018792	Metabolism	Carbohydrate metabolism	C5-Branched dibasic acid	ko00660	1
OG0018792	Metabolism	Metabolism of cofactors and vitamins	Nicotinate and nicotinamide	ko00760	6
OG0018792	Metabolism	Metabolism of cofactors and vitamins	Pantothenate and CoA biosynthesis	ko00770	1
OG0018801	Human Diseases	Drug resistance: Antimicrobial	Cationic antimicrobial peptide	ko01503	4

OG0018801	Human Diseases	Drug resistance: Antimicrobial	beta-Lactam resistance	ko01501	4
OG0018802	Human Diseases	Cancers: Overview	Chemical carcinogenesis	ko05204	3
OG0018802	Human Diseases	Cancers: Overview	MicroRNAs in cancer	ko05206	3
OG0018802	Human Diseases	Endocrine and metabolic diseases	Cushing syndrome	ko04934	3
OG0018802	Metabolism	Amino acid metabolism	Tryptophan metabolism	ko00380	6
OG0018802	Metabolism	Lipid metabolism	Steroid hormone biosynthesis	ko00140	6
OG0018802	Metabolism	Xenobiotics biodegradation and	Metabolism of xenobiotics by	ko00980	3
OG0018802	Organismal Systems	Endocrine system	Cortisol synthesis and secretion	ko04927	3
OG0018802	Organismal Systems	Endocrine system	Ovarian steroidogenesis	ko04913	6
OG0018802	Organismal Systems	Endocrine system	Prolactin signaling pathway	ko04917	3
OG0018803	Metabolism	Carbohydrate metabolism	Citrate cycle (TCA cycle)	ko00020	4
OG0018803	Metabolism	Carbohydrate metabolism	Glyoxylate and dicarboxylate	ko00630	4
OG0018803	Metabolism	Carbohydrate metabolism	Propanoate metabolism	ko00640	4
OG0018803	Metabolism	Energy metabolism	Carbon fixation pathways in	ko00720	4
OG0018804	Metabolism	Carbohydrate metabolism	Amino sugar and nucleotide sugar	ko00520	4
OG0018804	Metabolism	Carbohydrate metabolism	Ascorbate and aldarate metabolism	ko00053	4
OG0018804	Metabolism	Carbohydrate metabolism	Pentose and glucuronate	ko00040	4
OG0018807	Metabolism	Glycan biosynthesis and metabolism	Other glycan degradation	ko00511	2
OG0018809	Genetic Information	Replication and repair	DNA replication	ko03030	1
OG0018809	Metabolism	Carbohydrate metabolism	Starch and sucrose metabolism	ko00500	2
OG0018816	Metabolism	Metabolism of other amino acids	Selenocompound metabolism	ko00450	2
OG0020308	Genetic Information	Replication and repair	DNA replication	ko03030	3
OG0020316	Metabolism	Metabolism of cofactors and vitamins	Folate biosynthesis	ko00790	4
OG0020316	Metabolism	Nucleotide metabolism	Purine metabolism	ko00230	1
OG0020322	Metabolism	Metabolism of cofactors and vitamins	Pantothenate and CoA biosynthesis	ko00770	3
OG0020324	Metabolism	Metabolism of other amino acids	Glutathione metabolism	ko00480	3
OG0020326	Human Diseases	Endocrine and metabolic diseases	Insulin resistance	ko04931	3
OG0020326	Metabolism	Amino acid metabolism	Alanine, aspartate and glutamate	ko00250	3
OG0020326	Metabolism	Carbohydrate metabolism	Amino sugar and nucleotide sugar	ko00520	3
OG0020329	Metabolism	Amino acid metabolism	Cysteine and methionine metabolism	ko00270	2
OG0022053	Environmental Information	Membrane transport	ABC transporters	ko02010	4
OG0022054	Metabolism	Amino acid metabolism	Phenylalanine, tyrosine and	ko00400	1
OG0022057	Cellular Processes	Cell growth and death	Apoptosis - fly	ko04214	2
OG0022059	Genetic Information	Translation	Aminoacyl-tRNA biosynthesis	ko00970	2

OG0022061	Metabolism	Metabolism of cofactors and vitamins	Pantothenate and CoA biosynthesis	ko00770	6
OG0022065	Cellular Processes	Cell growth and death	Cell cycle - Caulobacter	ko04112	1
OG0022065	Genetic Information	Replication and repair	DNA replication	ko03030	1
OG0022065	Metabolism	Amino acid metabolism	Cysteine and methionine metabolism	ko00270	1
OG0022072	Metabolism	Glycan biosynthesis and metabolism	N-Glycan biosynthesis	ko00510	2
OG0022073	Cellular Processes	Cellular community - prokaryotes	Biofilm formation - Escherichia	ko02026	3
OG0022073	Metabolism	Carbohydrate metabolism	Starch and sucrose metabolism	ko00500	3
OG0022075	Metabolism	Amino acid metabolism	Histidine metabolism	ko00340	2
OG0022075	Metabolism	Amino acid metabolism	Phenylalanine metabolism	ko00360	2
OG0022075	Metabolism	Amino acid metabolism	Phenylalanine, tyrosine and	ko00400	2
OG0022075	Metabolism	Amino acid metabolism	Tyrosine metabolism	ko00350	2
OG0022075	Metabolism	Biosynthesis of other secondary	Novobiocin biosynthesis	ko00401	2
OG0022075	Metabolism	Biosynthesis of other secondary	Tropane, piperidine and pyridine	ko00960	2
OG0022075	Metabolism	Metabolism of cofactors and vitamins	Porphyrin and chlorophyll	ko00860	2
OG0022086	Cellular Processes	Cellular community - prokaryotes	Quorum sensing	ko02024	2
OG0022086	Environmental Information	Membrane transport	ABC transporters	ko02010	2
OG0024184	Human Diseases	Infectious diseases: Bacterial	Staphylococcus aureus infection	ko05150	1
OG0024190	Environmental Information	Signal transduction	MAPK signaling pathway - plant	ko04016	2
OG0024190	Organismal Systems	Environmental adaptation	Plant-pathogen interaction	ko04626	2
OG0024196	Metabolism	Metabolism of cofactors and vitamins	Porphyrin and chlorophyll	ko00860	3
OG0024231	Environmental Information	Signal transduction	Plant hormone signal transduction	ko04075	2
