# Supplementary material for: A high-quality genome provides insights into the new taxonomic status and genomic characteristics of Cladopus chinensis (Podostemaceae)
Source: Hortic Res. 2020 Apr 1;7:46. doi: 10.1038/s41438-020-0269-5 (PMC7109043; doi:10.1038/s41438-020-0269-5)
Supplement: Supplementary file 8 — Table S10. GO enrichment of the expansion familes genes identified in the C. chinensis [file 41438_2020_269_MOESM8_ESM.pdf]

OG	GO class	GO description	Number of genes
OG0000212	Biological Process	cellular process(GO:0009987)	34
OG0000212	Biological Process	metabolic process(GO:0008152)	34
OG0000212	Biological Process	response to stimulus(GO:0050896)	34
OG0000212	Cellular Component	cell part(GO:0044464)	34
OG0000212	Cellular Component	cell(GO:0005623)	34
OG0000212	Cellular Component	extracellular region(GO:0005576)	34
OG0000212	Cellular Component	membrane(GO:0016020)	34
OG0000212	Cellular Component	organelle part(GO:0044422)	34
OG0000212	Cellular Component	organelle(GO:0043226)	34
OG0000212	Molecular Function	binding(GO:0005488)	34
OG0000396	Biological Process	metabolic process(GO:0008152)	15
OG0000396	Cellular Component	cell part(GO:0044464)	15
OG0000396	Cellular Component	cell(GO:0005623)	15
OG0000396	Cellular Component	organelle(GO:0043226)	15
OG0000396	Molecular Function	catalytic activity(GO:0003824)	15
OG0000567	Biological Process	biological	3
OG0000567	Biological Process	cellular process(GO:0009987)	3
OG0000567	Biological Process	metabolic process(GO:0008152)	3
OG0000567	Biological Process	multi-organism	3
OG0000567	Biological Process	regulation of biological	3
OG0000567	Biological Process	response to stimulus(GO:0050896)	3
OG0000567	Cellular Component	cell part(GO:0044464)	3
OG0000567	Cellular Component	cell(GO:0005623)	3
OG0000567	Cellular Component	organelle(GO:0043226)	3
OG0000567	Molecular Function	transcription regulator	3
OG0000981	Cellular Component	extracellular region(GO:0005576)	5
OG0001284	Cellular Component	cell part(GO:0044464)	14
OG0001284	Cellular Component	cell(GO:0005623)	14
OG0001284	Cellular Component	organelle(GO:0043226)	14
OG0001319	Biological Process	biological phase(GO:0044848)	4
OG0001319	Biological Process	biological	7
OG0001319	Biological Process	cellular process(GO:0009987)	7
OG0001319	Biological Process	developmental	4

OG0001319	Biological Process	metabolic process(GO:0008152)	7
OG0001319	Biological Process	multicellular organismal	4
OG0001319	Biological Process	negative regulation of	7
OG0001319	Biological Process	positive regulation of	4
OG0001319	Biological Process	regulation of biological	7
OG0001319	Biological Process	response to stimulus(GO:0050896)	4
OG0001319	Cellular Component	cell part(GO:0044464)	7
OG0001319	Cellular Component	cell(GO:0005623)	7
OG0001319	Cellular Component	membrane-enclosed	7
OG0001319	Cellular Component	organelle part(GO:0044422)	7
OG0001319	Cellular Component	organelle(GO:0043226)	7
OG0001319	Molecular Function	molecular function	4
OG0002016	Biological Process	biological	2
OG0002016	Biological Process	cellular process(GO:0009987)	2
OG0002016	Biological Process	immune system	2
OG0002016	Biological Process	positive regulation of	2
OG0002016	Biological Process	regulation of biological	2
OG0002016	Biological Process	response to stimulus(GO:0050896)	2
OG0002016	Biological Process	signaling(GO:0023052)	2
OG0002016	Cellular Component	cell part(GO:0044464)	2
OG0002016	Cellular Component	cell(GO:0005623)	2
OG0002016	Cellular Component	organelle(GO:0043226)	2
OG0002460	Cellular Component	extracellular region(GO:0005576)	1
OG0002529	Biological Process	cellular process(GO:0009987)	8
OG0002529	Biological Process	developmental	8
OG0002529	Biological Process	metabolic process(GO:0008152)	8
OG0002529	Biological Process	multicellular organismal	8
OG0002529	Biological Process	reproduction(GO:0000003)	8
OG0002529	Biological Process	reproductive process(GO:0022414)	8
OG0002529	Biological Process	response to stimulus(GO:0050896)	8
OG0002529	Cellular Component	cell part(GO:0044464)	8
OG0002529	Cellular Component	cell(GO:0005623)	8
OG0002529	Cellular Component	membrane(GO:0016020)	8
OG0002529	Cellular Component	organelle part(GO:0044422)	8

OG0002529	Cellular Component	organelle(GO:0043226)	8
OG0002529	Molecular Function	catalytic activity(GO:0003824)	8
OG0002619	Cellular Component	cell part(GO:0044464)	6
OG0002619	Cellular Component	cell(GO:0005623)	6
OG0002619	Cellular Component	membrane(GO:0016020)	6
OG0002633	Cellular Component	membrane(GO:0016020)	7
OG0003097	Biological Process	developmental	6
OG0003097	Biological Process	multicellular organismal	6
OG0003097	Cellular Component	cell part(GO:0044464)	8
OG0003097	Cellular Component	cell(GO:0005623)	8
OG0003097	Cellular Component	membrane part(GO:0044425)	8
OG0003097	Cellular Component	membrane(GO:0016020)	8
OG0003097	Cellular Component	organelle part(GO:0044422)	2
OG0003097	Cellular Component	organelle(GO:0043226)	2
OG0003147	Biological Process	cellular process(GO:0009987)	8
OG0003147	Biological Process	metabolic process(GO:0008152)	8
OG0003147	Cellular Component	cell part(GO:0044464)	8
OG0003147	Cellular Component	cell(GO:0005623)	8
OG0003147	Molecular Function	catalytic activity(GO:0003824)	8
OG0003367	Cellular Component	cell part(GO:0044464)	6
OG0003367	Cellular Component	cell(GO:0005623)	6
OG0003367	Cellular Component	membrane(GO:0016020)	6
OG0003546	Biological Process	cellular process(GO:0009987)	7
OG0003546	Biological Process	developmental	2
OG0003546	Biological Process	growth(GO:0040007)	7
OG0003546	Biological Process	metabolic process(GO:0008152)	7
OG0003546	Biological Process	multi-organism	2
OG0003546	Biological Process	multicellular organismal	2
OG0003546	Biological Process	reproduction(GO:0000003)	2
OG0003546	Biological Process	reproductive process(GO:0022414)	2
OG0003546	Biological Process	response to stimulus(GO:0050896)	2
OG0003546	Cellular Component	cell part(GO:0044464)	10
OG0003546	Cellular Component	cell(GO:0005623)	10
OG0003546	Cellular Component	extracellular region(GO:0005576)	7

OG0003546	Cellular Component	membrane(GO:0016020)	7
OG0003546	Cellular Component	organelle(GO:0043226)	2
OG0003546	Molecular Function	catalytic activity(GO:0003824)	7
OG0003576	Biological Process	cellular component organization	3
OG0003576	Biological Process	cellular process(GO:0009987)	3
OG0003576	Biological Process	growth(GO:0040007)	3
OG0003576	Biological Process	immune system	3
OG0003576	Biological Process	metabolic process(GO:0008152)	3
OG0003576	Biological Process	multi-organism	3
OG0003576	Biological Process	response to stimulus(GO:0050896)	3
OG0003576	Cellular Component	cell part(GO:0044464)	3
OG0003576	Cellular Component	cell(GO:0005623)	3
OG0003576	Cellular Component	membrane part(GO:0044425)	3
OG0003576	Cellular Component	membrane(GO:0016020)	5
OG0003576	Molecular Function	catalytic activity(GO:0003824)	3
OG0003741	Biological Process	biological	8
OG0003741	Biological Process	cellular process(GO:0009987)	8
OG0003741	Biological Process	metabolic process(GO:0008152)	8
OG0003772	Biological Process	biological	12
OG0003772	Biological Process	cellular process(GO:0009987)	12
OG0003772	Biological Process	metabolic process(GO:0008152)	12
OG0003772	Biological Process	regulation of biological	12
OG0003772	Cellular Component	cell part(GO:0044464)	12
OG0003772	Cellular Component	cell(GO:0005623)	12
OG0003772	Cellular Component	organelle(GO:0043226)	12
OG0003772	Molecular Function	transcription regulator	12
OG0003956	Biological Process	cellular component organization	2
OG0003956	Biological Process	cellular process(GO:0009987)	2
OG0003956	Biological Process	response to stimulus(GO:0050896)	2
OG0003956	Cellular Component	cell part(GO:0044464)	5
OG0003956	Cellular Component	cell(GO:0005623)	5
OG0003956	Cellular Component	organelle part(GO:0044422)	2
OG0003956	Cellular Component	organelle(GO:0043226)	2
OG0003995	Biological Process	cellular process(GO:0009987)	11

OG0003995	Biological Process	developmental	11
OG0003995	Biological Process	metabolic process(GO:0008152)	11
OG0003995	Biological Process	multi-organism	11
OG0003995	Biological Process	multicellular organismal	11
OG0003995	Biological Process	reproduction(GO:0000003)	11
OG0003995	Biological Process	reproductive process(GO:0022414)	11
OG0003995	Biological Process	response to stimulus(GO:0050896)	11
OG0004759	Biological Process	metabolic process(GO:0008152)	2
OG0004759	Cellular Component	cell part(GO:0044464)	6
OG0004759	Cellular Component	cell(GO:0005623)	6
OG0004759	Cellular Component	organelle part(GO:0044422)	2
OG0004759	Cellular Component	organelle(GO:0043226)	2
OG0004759	Molecular Function	catalytic activity(GO:0003824)	2
OG0005302	Biological Process	biological	8
OG0005302	Biological Process	cellular process(GO:0009987)	8
OG0005302	Biological Process	developmental	8
OG0005302	Biological Process	growth(GO:0040007)	2
OG0005302	Biological Process	localization(GO:0051179)	1
OG0005302	Biological Process	locomotion(GO:0040011)	1
OG0005302	Biological Process	metabolic process(GO:0008152)	8
OG0005302	Biological Process	multicellular organismal	8
OG0005302	Biological Process	regulation of biological	8
OG0005302	Biological Process	reproduction(GO:0000003)	2
OG0005302	Biological Process	reproductive process(GO:0022414)	2
OG0005302	Biological Process	response to stimulus(GO:0050896)	8
OG0005302	Biological Process	signaling(GO:0023052)	5
OG0005302	Cellular Component	cell part(GO:0044464)	8
OG0005302	Cellular Component	cell(GO:0005623)	8
OG0005302	Cellular Component	membrane part(GO:0044425)	1
OG0005302	Cellular Component	membrane(GO:0016020)	1
OG0005302	Cellular Component	organelle part(GO:0044422)	1
OG0005302	Cellular Component	organelle(GO:0043226)	8
OG0005302	Molecular Function	binding(GO:0005488)	5
OG0005302	Molecular Function	catalytic activity(GO:0003824)	1

OG0005302	Molecular Function	transcription regulator	7
OG0005521	Biological Process	biological	7
OG0005521	Biological Process	cellular process(GO:0009987)	7
OG0005521	Biological Process	metabolic process(GO:0008152)	7
OG0005521	Biological Process	negative regulation of	7
OG0005521	Biological Process	regulation of biological	7
OG0005521	Cellular Component	extracellular region(GO:0005576)	7
OG0005521	Molecular Function	molecular function	7
OG0005788	Cellular Component	cell part(GO:0044464)	14
OG0005788	Cellular Component	cell(GO:0005623)	14
OG0005788	Cellular Component	membrane(GO:0016020)	14
OG0005788	Cellular Component	organelle part(GO:0044422)	3
OG0005788	Cellular Component	organelle(GO:0043226)	3
OG0005830	Biological Process	biological	7
OG0005830	Biological Process	cellular process(GO:0009987)	7
OG0005830	Biological Process	metabolic process(GO:0008152)	7
OG0005830	Biological Process	multi-organism	7
OG0005830	Biological Process	response to stimulus(GO:0050896)	7
OG0005830	Cellular Component	cell part(GO:0044464)	7
OG0005830	Cellular Component	cell(GO:0005623)	7
OG0005830	Cellular Component	membrane(GO:0016020)	7
OG0005830	Cellular Component	organelle(GO:0043226)	7
OG0005830	Molecular Function	catalytic activity(GO:0003824)	7
OG0005844	Biological Process	response to stimulus(GO:0050896)	6
OG0005844	Cellular Component	cell part(GO:0044464)	6
OG0005844	Cellular Component	cell(GO:0005623)	6
OG0005844	Cellular Component	organelle part(GO:0044422)	6
OG0005844	Cellular Component	organelle(GO:0043226)	6
OG0005865	Biological Process	cellular process(GO:0009987)	6
OG0005865	Biological Process	metabolic process(GO:0008152)	6
OG0005865	Cellular Component	cell part(GO:0044464)	6
OG0005865	Cellular Component	cell(GO:0005623)	6
OG0005865	Cellular Component	membrane(GO:0016020)	3
OG0005865	Cellular Component	organelle(GO:0043226)	3

OG0005865	Molecular Function	binding(GO:0005488)	3
OG0005865	Molecular Function	catalytic activity(GO:0003824)	6
OG0006103	Biological Process	cellular process(GO:0009987)	2
OG0006103	Biological Process	metabolic process(GO:0008152)	2
OG0006103	Biological Process	multi-organism	2
OG0006103	Biological Process	response to stimulus(GO:0050896)	2
OG0006103	Cellular Component	cell part(GO:0044464)	5
OG0006103	Cellular Component	cell(GO:0005623)	5
OG0006103	Cellular Component	extracellular region(GO:0005576)	2
OG0006103	Cellular Component	organelle part(GO:0044422)	2
OG0006103	Cellular Component	organelle(GO:0043226)	2
OG0006103	Molecular Function	antioxidant activity(GO:0016209)	5
OG0006103	Molecular Function	catalytic activity(GO:0003824)	2
OG0006185	Biological Process	cellular process(GO:0009987)	1
OG0006185	Biological Process	metabolic process(GO:0008152)	1
OG0006185	Cellular Component	cell part(GO:0044464)	2
OG0006185	Cellular Component	cell(GO:0005623)	2
OG0006185	Cellular Component	membrane part(GO:0044425)	2
OG0006185	Cellular Component	membrane(GO:0016020)	2
OG0006185	Cellular Component	organelle part(GO:0044422)	1
OG0006185	Cellular Component	organelle(GO:0043226)	1
OG0006298	Biological Process	biological	6
OG0006298	Biological Process	cell proliferation(GO:0008283)	6
OG0006298	Biological Process	cellular process(GO:0009987)	6
OG0006298	Biological Process	developmental	6
OG0006298	Biological Process	metabolic process(GO:0008152)	6
OG0006298	Biological Process	multicellular organismal	6
OG0006298	Biological Process	negative regulation of	6
OG0006298	Biological Process	regulation of biological	6
OG0006298	Biological Process	reproduction(GO:0000003)	6
OG0006298	Biological Process	reproductive process(GO:0022414)	6
OG0006298	Cellular Component	cell part(GO:0044464)	6
OG0006298	Cellular Component	cell(GO:0005623)	6
OG0006298	Cellular Component	organelle(GO:0043226)	6

OG0006298	Molecular Function	transcription regulator	6
OG0006333	Biological Process	biological	1
OG0006333	Biological Process	cellular component organization	3
OG0006333	Biological Process	cellular process(GO:0009987)	4
OG0006333	Biological Process	growth(GO:0040007)	1
OG0006333	Biological Process	metabolic process(GO:0008152)	4
OG0006333	Biological Process	negative regulation of	1
OG0006333	Biological Process	regulation of biological	1
OG0006333	Cellular Component	cell part(GO:0044464)	11
OG0006333	Cellular Component	cell(GO:0005623)	11
OG0006333	Cellular Component	membrane(GO:0016020)	1
OG0006333	Cellular Component	membrane-enclosed	2
OG0006333	Cellular Component	organelle part(GO:0044422)	3
OG0006333	Cellular Component	organelle(GO:0043226)	10
OG0006333	Cellular Component	protein-containing	3
OG0006333	Molecular Function	binding(GO:0005488)	2
OG0006333	Molecular Function	structural molecule	3
OG0006352	Biological Process	metabolic process(GO:0008152)	6
OG0006352	Molecular Function	catalytic activity(GO:0003824)	6
OG0006458	Cellular Component	cell part(GO:0044464)	5
OG0006458	Cellular Component	cell(GO:0005623)	5
OG0006458	Cellular Component	membrane part(GO:0044425)	5
OG0006458	Cellular Component	membrane(GO:0016020)	5
OG0006553	Biological Process	cellular process(GO:0009987)	6
OG0006553	Biological Process	metabolic process(GO:0008152)	6
OG0006553	Biological Process	response to stimulus(GO:0050896)	6
OG0006553	Cellular Component	cell part(GO:0044464)	6
OG0006553	Cellular Component	cell(GO:0005623)	6
OG0006553	Cellular Component	membrane(GO:0016020)	6
OG0006553	Cellular Component	organelle part(GO:0044422)	6
OG0006553	Cellular Component	organelle(GO:0043226)	6
OG0006553	Molecular Function	catalytic activity(GO:0003824)	6
OG0006634	Cellular Component	cell part(GO:0044464)	15
OG0006634	Cellular Component	cell(GO:0005623)	15

OG0006634	Cellular Component	membrane(GO:0016020)	15
OG0006634	Cellular Component	organelle part(GO:0044422)	13
OG0006634	Cellular Component	organelle(GO:0043226)	13
OG0006916	Biological Process	cellular process(GO:0009987)	5
OG0006916	Biological Process	metabolic process(GO:0008152)	5
OG0006916	Cellular Component	cell part(GO:0044464)	14
OG0006916	Cellular Component	cell(GO:0005623)	14
OG0006916	Cellular Component	membrane(GO:0016020)	14
OG0006916	Cellular Component	organelle part(GO:0044422)	9
OG0006916	Cellular Component	organelle(GO:0043226)	9
OG0006916	Cellular Component	synapse part(GO:0044456)	5
OG0006916	Cellular Component	synapse(GO:0045202)	5
OG0006962	Biological Process	biological	7
OG0006962	Biological Process	cellular process(GO:0009987)	7
OG0006962	Biological Process	metabolic process(GO:0008152)	7
OG0006962	Biological Process	regulation of biological	7
OG0006962	Biological Process	response to stimulus(GO:0050896)	4
OG0006962	Biological Process	rhythmic process(GO:0048511)	6
OG0006962	Cellular Component	cell part(GO:0044464)	8
OG0006962	Cellular Component	cell(GO:0005623)	8
OG0006962	Cellular Component	organelle(GO:0043226)	8
OG0006962	Molecular Function	binding(GO:0005488)	2
OG0006962	Molecular Function	transcription regulator	7
OG0007027	Biological Process	cellular process(GO:0009987)	4
OG0007027	Biological Process	developmental	4
OG0007027	Biological Process	multi-organism	4
OG0007027	Biological Process	multicellular organismal	4
OG0007027	Biological Process	reproduction(GO:0000003)	4
OG0007027	Biological Process	reproductive process(GO:0022414)	4
OG0007027	Cellular Component	cell part(GO:0044464)	4
OG0007027	Cellular Component	cell(GO:0005623)	4
OG0007027	Cellular Component	organelle(GO:0043226)	4
OG0007027	Molecular Function	binding(GO:0005488)	4
OG0007170	Biological Process	cellular process(GO:0009987)	2

OG0007170	Biological Process	metabolic process(GO:0008152)	2
OG0007170	Biological Process	response to stimulus(GO:0050896)	2
OG0007170	Molecular Function	catalytic activity(GO:0003824)	2
OG0007201	Cellular Component	cell part(GO:0044464)	4
OG0007201	Cellular Component	cell(GO:0005623)	4
OG0007201	Cellular Component	membrane(GO:0016020)	4
OG0007201	Cellular Component	organelle part(GO:0044422)	4
OG0007201	Cellular Component	organelle(GO:0043226)	4
OG0007216	Biological Process	metabolic process(GO:0008152)	6
OG0007216	Cellular Component	cell junction(GO:0030054)	6
OG0007216	Cellular Component	cell part(GO:0044464)	6
OG0007216	Cellular Component	cell(GO:0005623)	6
OG0007216	Cellular Component	extracellular region(GO:0005576)	6
OG0007216	Cellular Component	membrane(GO:0016020)	6
OG0007216	Cellular Component	organelle part(GO:0044422)	6
OG0007216	Cellular Component	organelle(GO:0043226)	6
OG0007216	Cellular Component	symplast(GO:0055044)	6
OG0007216	Molecular Function	catalytic activity(GO:0003824)	6
OG0007476	Biological Process	cellular process(GO:0009987)	5
OG0007476	Biological Process	growth(GO:0040007)	5
OG0007476	Biological Process	metabolic process(GO:0008152)	5
OG0007476	Cellular Component	cell part(GO:0044464)	8
OG0007476	Cellular Component	cell(GO:0005623)	8
OG0007476	Cellular Component	membrane(GO:0016020)	5
OG0007476	Cellular Component	organelle part(GO:0044422)	1
OG0007476	Cellular Component	organelle(GO:0043226)	4
OG0007476	Cellular Component	protein-containing	1
OG0007476	Molecular Function	structural molecule	5
OG0007590	Biological Process	cellular component organization	4
OG0007590	Biological Process	cellular process(GO:0009987)	4
OG0007590	Biological Process	metabolic process(GO:0008152)	4
OG0007590	Cellular Component	cell part(GO:0044464)	4
OG0007590	Cellular Component	cell(GO:0005623)	4
OG0007590	Cellular Component	membrane(GO:0016020)	6

OG0007590	Molecular Function	catalytic activity(GO:0003824)	4
OG0007749	Biological Process	metabolic process(GO:0008152)	1
OG0007749	Cellular Component	cell part(GO:0044464)	3
OG0007749	Cellular Component	cell(GO:0005623)	3
OG0007749	Cellular Component	membrane(GO:0016020)	3
OG0007749	Molecular Function	catalytic activity(GO:0003824)	1
OG0007839	Biological Process	cellular process(GO:0009987)	1
OG0007839	Biological Process	growth(GO:0040007)	1
OG0007839	Biological Process	localization(GO:0051179)	1
OG0007839	Biological Process	metabolic process(GO:0008152)	1
OG0007839	Cellular Component	cell part(GO:0044464)	5
OG0007839	Cellular Component	cell(GO:0005623)	5
OG0007839	Cellular Component	membrane part(GO:0044425)	1
OG0007839	Cellular Component	membrane(GO:0016020)	5
OG0007839	Cellular Component	membrane-enclosed	1
OG0007839	Cellular Component	organelle part(GO:0044422)	4
OG0007839	Cellular Component	organelle(GO:0043226)	4
OG0007839	Cellular Component	protein-containing	1
OG0007839	Molecular Function	binding(GO:0005488)	1
OG0007839	Molecular Function	catalytic activity(GO:0003824)	1
OG0007839	Molecular Function	transporter activity(GO:0005215)	1
OG0007841	Biological Process	cellular process(GO:0009987)	1
OG0007841	Biological Process	growth(GO:0040007)	1
OG0007841	Biological Process	metabolic process(GO:0008152)	1
OG0007841	Cellular Component	cell part(GO:0044464)	9
OG0007841	Cellular Component	cell(GO:0005623)	9
OG0007841	Cellular Component	membrane(GO:0016020)	1
OG0007841	Cellular Component	organelle part(GO:0044422)	1
OG0007841	Cellular Component	organelle(GO:0043226)	9
OG0007841	Cellular Component	protein-containing	1
OG0007841	Molecular Function	binding(GO:0005488)	1
OG0007841	Molecular Function	structural molecule	1
OG0007870	Biological Process	response to stimulus(GO:0050896)	6
OG0007870	Cellular Component	cell part(GO:0044464)	6

OG0007870	Cellular Component	cell(GO:0005623)	6
OG0007870	Cellular Component	membrane(GO:0016020)	6
OG0007870	Cellular Component	organelle(GO:0043226)	6
OG0007880	Biological Process	cellular component organization	2
OG0007880	Biological Process	cellular process(GO:0009987)	6
OG0007880	Biological Process	growth(GO:0040007)	4
OG0007880	Biological Process	localization(GO:0051179)	4
OG0007880	Biological Process	multi-organism	4
OG0007880	Biological Process	response to stimulus(GO:0050896)	4
OG0007880	Cellular Component	cell part(GO:0044464)	6
OG0007880	Cellular Component	cell(GO:0005623)	6
OG0007880	Cellular Component	extracellular region(GO:0005576)	4
OG0007880	Cellular Component	membrane part(GO:0044425)	4
OG0007880	Cellular Component	membrane(GO:0016020)	6
OG0007880	Cellular Component	organelle part(GO:0044422)	2
OG0007880	Cellular Component	organelle(GO:0043226)	2
OG0007927	Biological Process	cellular process(GO:0009987)	4
OG0007927	Biological Process	localization(GO:0051179)	4
OG0007927	Cellular Component	cell part(GO:0044464)	4
OG0007927	Cellular Component	cell(GO:0005623)	4
OG0007927	Cellular Component	membrane(GO:0016020)	4
OG0007927	Cellular Component	organelle part(GO:0044422)	4
OG0007927	Cellular Component	organelle(GO:0043226)	4
OG0007927	Molecular Function	transporter activity(GO:0005215)	4
OG0008062	Biological Process	cellular component organization	4
OG0008062	Biological Process	cellular process(GO:0009987)	4
OG0008062	Biological Process	metabolic process(GO:0008152)	4
OG0008062	Cellular Component	cell part(GO:0044464)	4
OG0008062	Cellular Component	cell(GO:0005623)	4
OG0008062	Cellular Component	organelle(GO:0043226)	4
OG0008120	Cellular Component	cell part(GO:0044464)	4
OG0008120	Cellular Component	cell(GO:0005623)	4
OG0008120	Cellular Component	membrane(GO:0016020)	4
OG0008120	Cellular Component	organelle part(GO:0044422)	4

OG0008120	Cellular Component	organelle(GO:0043226)	4
OG0008175	Cellular Component	cell part(GO:0044464)	5
OG0008175	Cellular Component	cell(GO:0005623)	5
OG0008175	Cellular Component	organelle(GO:0043226)	5
OG0008185	Biological Process	cellular component organization	5
OG0008185	Biological Process	cellular process(GO:0009987)	5
OG0008185	Biological Process	metabolic process(GO:0008152)	5
OG0008185	Cellular Component	cell part(GO:0044464)	2
OG0008185	Cellular Component	cell(GO:0005623)	2
OG0008185	Cellular Component	organelle part(GO:0044422)	2
OG0008185	Cellular Component	organelle(GO:0043226)	2
OG0008185	Molecular Function	binding(GO:0005488)	3
OG0008185	Molecular Function	molecular carrier	3
OG0008185	Molecular Function	structural molecule	2
OG0008209	Biological Process	biological	4
OG0008209	Biological Process	cellular component organization	4
OG0008209	Biological Process	cellular process(GO:0009987)	4
OG0008209	Biological Process	developmental	4
OG0008209	Biological Process	metabolic process(GO:0008152)	4
OG0008209	Biological Process	multicellular organismal	4
OG0008209	Biological Process	regulation of biological	4
OG0008209	Cellular Component	cell part(GO:0044464)	4
OG0008209	Cellular Component	cell(GO:0005623)	4
OG0008209	Cellular Component	organelle(GO:0043226)	4
OG0008209	Molecular Function	transcription regulator	4
OG0008309	Biological Process	cellular component organization	4
OG0008309	Biological Process	cellular process(GO:0009987)	4
OG0008309	Biological Process	multicellular organismal	4
OG0008309	Biological Process	response to stimulus(GO:0050896)	4
OG0008309	Cellular Component	cell part(GO:0044464)	4
OG0008309	Cellular Component	cell(GO:0005623)	4
OG0008309	Cellular Component	membrane-enclosed	4
OG0008309	Cellular Component	organelle part(GO:0044422)	4
OG0008309	Cellular Component	organelle(GO:0043226)	4

OG0008309	Cellular Component	protein-containing	4
OG0008309	Molecular Function	binding(GO:0005488)	4
OG0008350	Cellular Component	cell part(GO:0044464)	5
OG0008350	Cellular Component	cell(GO:0005623)	5
OG0008350	Cellular Component	membrane(GO:0016020)	4
OG0008350	Cellular Component	organelle part(GO:0044422)	4
OG0008350	Cellular Component	organelle(GO:0043226)	4
OG0008350	Cellular Component	protein-containing	4
OG0008350	Molecular Function	binding(GO:0005488)	4
OG0008363	Biological Process	biological	2
OG0008363	Biological Process	cellular process(GO:0009987)	2
OG0008363	Biological Process	immune system	2
OG0008363	Biological Process	metabolic process(GO:0008152)	2
OG0008363	Biological Process	multi-organism	4
OG0008363	Biological Process	regulation of biological	2
OG0008363	Biological Process	response to stimulus(GO:0050896)	4
OG0008363	Biological Process	signaling(GO:0023052)	2
OG0008363	Cellular Component	cell junction(GO:0030054)	2
OG0008363	Cellular Component	cell part(GO:0044464)	15
OG0008363	Cellular Component	cell(GO:0005623)	15
OG0008363	Cellular Component	membrane(GO:0016020)	12
OG0008363	Cellular Component	organelle(GO:0043226)	4
OG0008363	Cellular Component	symplast(GO:0055044)	2
OG0008363	Molecular Function	binding(GO:0005488)	2
OG0008363	Molecular Function	catalytic activity(GO:0003824)	2
OG0008363	Molecular Function	molecular transducer	2
OG0008460	Cellular Component	cell part(GO:0044464)	4
OG0008460	Cellular Component	cell(GO:0005623)	4
OG0008548	Cellular Component	cell part(GO:0044464)	11
OG0008548	Cellular Component	cell(GO:0005623)	11
OG0008548	Cellular Component	membrane(GO:0016020)	9
OG0008548	Cellular Component	organelle part(GO:0044422)	3
OG0008548	Cellular Component	organelle(GO:0043226)	5
OG0008625	Biological Process	biological	2

OG0008625	Biological Process	cellular component organization	6
OG0008625	Biological Process	cellular process(GO:0009987)	6
OG0008625	Biological Process	growth(GO:0040007)	4
OG0008625	Biological Process	localization(GO:0051179)	2
OG0008625	Biological Process	metabolic process(GO:0008152)	6
OG0008625	Biological Process	regulation of biological	2
OG0008625	Biological Process	response to stimulus(GO:0050896)	2
OG0008625	Cellular Component	cell part(GO:0044464)	6
OG0008625	Cellular Component	cell(GO:0005623)	6
OG0008625	Cellular Component	organelle part(GO:0044422)	2
OG0008625	Cellular Component	organelle(GO:0043226)	2
OG0008625	Molecular Function	binding(GO:0005488)	4
OG0008625	Molecular Function	catalytic activity(GO:0003824)	6
OG0008625	Molecular Function	transporter activity(GO:0005215)	2
OG0008668	Cellular Component	cell part(GO:0044464)	4
OG0008668	Cellular Component	cell(GO:0005623)	4
OG0008668	Cellular Component	membrane(GO:0016020)	4
OG0008668	Cellular Component	organelle(GO:0043226)	4
OG0008747	Biological Process	biological	1
OG0008747	Biological Process	cellular component organization	1
OG0008747	Biological Process	cellular process(GO:0009987)	1
OG0008747	Biological Process	metabolic process(GO:0008152)	1
OG0008747	Cellular Component	cell part(GO:0044464)	2
OG0008747	Cellular Component	cell(GO:0005623)	2
OG0008747	Cellular Component	organelle part(GO:0044422)	2
OG0008747	Cellular Component	organelle(GO:0043226)	2
OG0008915	Biological Process	cellular process(GO:0009987)	4
OG0008915	Biological Process	metabolic process(GO:0008152)	4
OG0008915	Cellular Component	cell part(GO:0044464)	4
OG0008915	Cellular Component	cell(GO:0005623)	4
OG0008915	Cellular Component	membrane part(GO:0044425)	4
OG0008915	Cellular Component	membrane(GO:0016020)	4
OG0008915	Cellular Component	organelle part(GO:0044422)	4
OG0008915	Cellular Component	organelle(GO:0043226)	4

OG0008975	Biological Process	cellular process(GO:0009987)	2
OG0008975	Biological Process	metabolic process(GO:0008152)	2
OG0008975	Cellular Component	cell part(GO:0044464)	2
OG0008975	Cellular Component	cell(GO:0005623)	2
OG0008975	Molecular Function	binding(GO:0005488)	2
OG0009240	Biological Process	cellular component organization	1
OG0009240	Biological Process	cellular process(GO:0009987)	2
OG0009240	Biological Process	metabolic process(GO:0008152)	2
OG0009240	Cellular Component	cell part(GO:0044464)	4
OG0009240	Cellular Component	cell(GO:0005623)	4
OG0009240	Cellular Component	membrane part(GO:0044425)	1
OG0009240	Cellular Component	membrane(GO:0016020)	3
OG0009240	Molecular Function	binding(GO:0005488)	1
OG0009240	Molecular Function	catalytic activity(GO:0003824)	2
OG0009302	Cellular Component	cell part(GO:0044464)	4
OG0009302	Cellular Component	cell(GO:0005623)	4
OG0009302	Cellular Component	membrane(GO:0016020)	4
OG0009331	Biological Process	biological	4
OG0009331	Biological Process	metabolic process(GO:0008152)	4
OG0009331	Biological Process	response to stimulus(GO:0050896)	4
OG0009331	Cellular Component	membrane(GO:0016020)	4
OG0009331	Molecular Function	catalytic activity(GO:0003824)	4
OG0009384	Biological Process	cellular process(GO:0009987)	6
OG0009384	Biological Process	developmental	2
OG0009384	Biological Process	metabolic process(GO:0008152)	6
OG0009384	Biological Process	multicellular organismal	2
OG0009384	Biological Process	reproduction(GO:0000003)	2
OG0009384	Biological Process	reproductive process(GO:0022414)	2
OG0009384	Cellular Component	cell part(GO:0044464)	8
OG0009384	Cellular Component	cell(GO:0005623)	8
OG0009384	Cellular Component	organelle part(GO:0044422)	2
OG0009384	Cellular Component	organelle(GO:0043226)	2
OG0009384	Molecular Function	catalytic activity(GO:0003824)	6
OG0009395	Biological Process	cellular process(GO:0009987)	2

OG0009395	Biological Process	metabolic process(GO:0008152)	2
OG0009395	Cellular Component	cell part(GO:0044464)	2
OG0009395	Cellular Component	cell(GO:0005623)	2
OG0009395	Cellular Component	organelle(GO:0043226)	2
OG0009423	Cellular Component	cell part(GO:0044464)	4
OG0009423	Cellular Component	cell(GO:0005623)	4
OG0009423	Cellular Component	membrane(GO:0016020)	4
OG0009423	Cellular Component	organelle part(GO:0044422)	4
OG0009423	Cellular Component	organelle(GO:0043226)	4
OG0009664	Biological Process	cellular process(GO:0009987)	3
OG0009664	Biological Process	growth(GO:0040007)	3
OG0009664	Biological Process	metabolic process(GO:0008152)	3
OG0009664	Cellular Component	cell part(GO:0044464)	5
OG0009664	Cellular Component	cell(GO:0005623)	5
OG0009664	Cellular Component	membrane(GO:0016020)	5
OG0009664	Cellular Component	organelle part(GO:0044422)	2
OG0009664	Cellular Component	organelle(GO:0043226)	2
OG0009664	Molecular Function	structural molecule	3
OG0009687	Biological Process	cellular process(GO:0009987)	3
OG0009687	Biological Process	growth(GO:0040007)	3
OG0009687	Biological Process	metabolic process(GO:0008152)	5
OG0009687	Biological Process	response to stimulus(GO:0050896)	3
OG0009687	Cellular Component	cell part(GO:0044464)	3
OG0009687	Cellular Component	cell(GO:0005623)	3
OG0009687	Cellular Component	protein-containing	3
OG0009687	Molecular Function	binding(GO:0005488)	3
OG0009687	Molecular Function	catalytic activity(GO:0003824)	5
OG0009747	Biological Process	cellular process(GO:0009987)	2
OG0009747	Biological Process	metabolic process(GO:0008152)	2
OG0009747	Cellular Component	cell part(GO:0044464)	4
OG0009747	Cellular Component	cell(GO:0005623)	4
OG0009747	Cellular Component	membrane(GO:0016020)	2
OG0009747	Cellular Component	organelle part(GO:0044422)	2
OG0009747	Cellular Component	organelle(GO:0043226)	2

OG0009747	Molecular Function	structural molecule	2
OG0009866	Biological Process	cellular process(GO:0009987)	3
OG0009866	Biological Process	metabolic process(GO:0008152)	3
OG0009866	Molecular Function	catalytic activity(GO:0003824)	3
OG0010038	Cellular Component	cell part(GO:0044464)	6
OG0010038	Cellular Component	cell(GO:0005623)	6
OG0010038	Cellular Component	extracellular region(GO:0005576)	6
OG0010038	Cellular Component	membrane(GO:0016020)	6
OG0010112	Cellular Component	cell part(GO:0044464)	4
OG0010112	Cellular Component	cell(GO:0005623)	4
OG0010112	Cellular Component	membrane(GO:0016020)	4
OG0010112	Cellular Component	organelle part(GO:0044422)	4
OG0010112	Cellular Component	organelle(GO:0043226)	4
OG0010161	Cellular Component	extracellular region(GO:0005576)	5
OG0010188	Biological Process	cellular component organization	4
OG0010188	Biological Process	cellular process(GO:0009987)	4
OG0010188	Biological Process	response to stimulus(GO:0050896)	4
OG0010188	Cellular Component	cell part(GO:0044464)	4
OG0010188	Cellular Component	cell(GO:0005623)	4
OG0010188	Cellular Component	organelle(GO:0043226)	4
OG0010188	Cellular Component	protein-containing	4
OG0010322	Biological Process	metabolic process(GO:0008152)	1
OG0010322	Cellular Component	cell part(GO:0044464)	1
OG0010322	Cellular Component	cell(GO:0005623)	1
OG0010322	Cellular Component	membrane(GO:0016020)	1
OG0010322	Cellular Component	organelle part(GO:0044422)	1
OG0010322	Cellular Component	organelle(GO:0043226)	1
OG0010322	Cellular Component	protein-containing	1
OG0010322	Molecular Function	catalytic activity(GO:0003824)	1
OG0010426	Cellular Component	cell part(GO:0044464)	9
OG0010426	Cellular Component	cell(GO:0005623)	9
OG0010426	Cellular Component	membrane(GO:0016020)	9
OG0010426	Cellular Component	organelle part(GO:0044422)	5
OG0010426	Cellular Component	organelle(GO:0043226)	5

OG0010457	Cellular Component	cell part(GO:0044464)	4
OG0010457	Cellular Component	cell(GO:0005623)	4
OG0010457	Cellular Component	organelle part(GO:0044422)	1
OG0010457	Cellular Component	organelle(GO:0043226)	1
OG0010483	Biological Process	cellular process(GO:0009987)	5
OG0010483	Biological Process	response to stimulus(GO:0050896)	5
OG0010483	Cellular Component	cell part(GO:0044464)	5
OG0010483	Cellular Component	cell(GO:0005623)	5
OG0010483	Cellular Component	membrane(GO:0016020)	5
OG0010483	Cellular Component	organelle part(GO:0044422)	5
OG0010483	Cellular Component	organelle(GO:0043226)	5
OG0010488	Biological Process	growth(GO:0040007)	3
OG0010488	Cellular Component	cell part(GO:0044464)	1
OG0010488	Cellular Component	cell(GO:0005623)	1
OG0010488	Cellular Component	organelle part(GO:0044422)	1
OG0010488	Cellular Component	organelle(GO:0043226)	1
OG0010511	Biological Process	response to stimulus(GO:0050896)	5
OG0010565	Biological Process	cellular process(GO:0009987)	3
OG0010565	Biological Process	metabolic process(GO:0008152)	3
OG0010565	Cellular Component	cell part(GO:0044464)	3
OG0010565	Cellular Component	cell(GO:0005623)	3
OG0010565	Cellular Component	membrane(GO:0016020)	3
OG0010565	Cellular Component	organelle part(GO:0044422)	3
OG0010565	Cellular Component	organelle(GO:0043226)	3
OG0010635	Biological Process	biological	2
OG0010635	Biological Process	cellular process(GO:0009987)	2
OG0010635	Biological Process	developmental	2
OG0010635	Biological Process	metabolic process(GO:0008152)	2
OG0010635	Biological Process	multicellular organismal	2
OG0010635	Biological Process	regulation of biological	2
OG0010635	Biological Process	reproduction(GO:0000003)	2
OG0010635	Biological Process	reproductive process(GO:0022414)	2
OG0010635	Cellular Component	cell part(GO:0044464)	2
OG0010635	Cellular Component	cell(GO:0005623)	2

OG0010635	Cellular Component	organelle(GO:0043226)	2
OG0010635	Molecular Function	transcription regulator	2
OG0010725	Biological Process	cellular component organization	3
OG0010725	Biological Process	cellular process(GO:0009987)	3
OG0010725	Biological Process	response to stimulus(GO:0050896)	3
OG0010725	Cellular Component	cell part(GO:0044464)	3
OG0010725	Cellular Component	cell(GO:0005623)	3
OG0010725	Cellular Component	membrane part(GO:0044425)	3
OG0010725	Cellular Component	membrane(GO:0016020)	3
OG0010725	Cellular Component	organelle part(GO:0044422)	3
OG0010725	Cellular Component	organelle(GO:0043226)	3
OG0010725	Cellular Component	protein-containing	3
OG0010725	Molecular Function	binding(GO:0005488)	3
OG0010810	Cellular Component	cell part(GO:0044464)	6
OG0010810	Cellular Component	cell(GO:0005623)	6
OG0010810	Cellular Component	organelle(GO:0043226)	6
OG0010844	Biological Process	developmental	3
OG0010844	Biological Process	multi-organism	3
OG0010844	Biological Process	multicellular organismal	3
OG0010844	Biological Process	reproduction(GO:0000003)	3
OG0010844	Biological Process	reproductive process(GO:0022414)	3
OG0010844	Cellular Component	cell part(GO:0044464)	3
OG0010844	Cellular Component	cell(GO:0005623)	3
OG0010844	Cellular Component	membrane(GO:0016020)	3
OG0010844	Cellular Component	organelle(GO:0043226)	3
OG0011023	Biological Process	biological	3
OG0011023	Biological Process	cellular process(GO:0009987)	3
OG0011023	Biological Process	metabolic process(GO:0008152)	3
OG0011023	Biological Process	multi-organism	3
OG0011023	Biological Process	positive regulation of	3
OG0011023	Biological Process	regulation of biological	3
OG0011023	Biological Process	response to stimulus(GO:0050896)	3
OG0011023	Biological Process	signaling(GO:0023052)	3
OG0011023	Cellular Component	cell part(GO:0044464)	3

OG0011023	Cellular Component	cell(GO:0005623)	3
OG0011023	Cellular Component	protein-containing	3
OG0011023	Molecular Function	binding(GO:0005488)	3
OG0011023	Molecular Function	molecular transducer	3
OG0011145	Cellular Component	cell part(GO:0044464)	11
OG0011145	Cellular Component	cell(GO:0005623)	11
OG0011145	Cellular Component	membrane part(GO:0044425)	6
OG0011145	Cellular Component	membrane(GO:0016020)	11
OG0011145	Cellular Component	organelle part(GO:0044422)	11
OG0011145	Cellular Component	organelle(GO:0043226)	11
OG0011145	Cellular Component	protein-containing	6
OG0011146	Biological Process	cellular process(GO:0009987)	3
OG0011146	Biological Process	metabolic process(GO:0008152)	3
OG0011146	Cellular Component	cell part(GO:0044464)	9
OG0011146	Cellular Component	cell(GO:0005623)	9
OG0011146	Cellular Component	membrane(GO:0016020)	6
OG0011146	Cellular Component	organelle part(GO:0044422)	6
OG0011146	Cellular Component	organelle(GO:0043226)	6
OG0011146	Cellular Component	protein-containing	3
OG0011146	Molecular Function	catalytic activity(GO:0003824)	3
OG0011152	Biological Process	cellular process(GO:0009987)	8
OG0011152	Biological Process	metabolic process(GO:0008152)	8
OG0011152	Cellular Component	cell part(GO:0044464)	8
OG0011152	Cellular Component	cell(GO:0005623)	8
OG0011152	Molecular Function	catalytic activity(GO:0003824)	8
OG0011172	Biological Process	cellular process(GO:0009987)	2
OG0011172	Biological Process	metabolic process(GO:0008152)	2
OG0011172	Biological Process	response to stimulus(GO:0050896)	2
OG0011172	Cellular Component	cell part(GO:0044464)	2
OG0011172	Cellular Component	cell(GO:0005623)	2
OG0011172	Molecular Function	binding(GO:0005488)	2
OG0011172	Molecular Function	catalytic activity(GO:0003824)	2
OG0011181	Biological Process	biological	3
OG0011181	Biological Process	cellular process(GO:0009987)	3

OG0011181	Biological Process	metabolic process(GO:0008152)	3
OG0011181	Biological Process	regulation of biological	3
OG0011181	Biological Process	response to stimulus(GO:0050896)	1
OG0011181	Cellular Component	cell part(GO:0044464)	1
OG0011181	Cellular Component	cell(GO:0005623)	1
OG0011181	Cellular Component	organelle(GO:0043226)	1
OG0011181	Molecular Function	transcription regulator	3
OG0011190	Biological Process	cellular process(GO:0009987)	4
OG0011190	Biological Process	metabolic process(GO:0008152)	4
OG0011190	Biological Process	response to stimulus(GO:0050896)	4
OG0011190	Cellular Component	cell part(GO:0044464)	4
OG0011190	Cellular Component	cell(GO:0005623)	4
OG0011190	Cellular Component	organelle(GO:0043226)	4
OG0011190	Molecular Function	catalytic activity(GO:0003824)	4
OG0011232	Biological Process	multi-organism	4
OG0011232	Biological Process	response to stimulus(GO:0050896)	4
OG0011232	Cellular Component	cell part(GO:0044464)	4
OG0011232	Cellular Component	cell(GO:0005623)	4
OG0011232	Cellular Component	membrane(GO:0016020)	4
OG0011232	Cellular Component	organelle part(GO:0044422)	4
OG0011232	Cellular Component	organelle(GO:0043226)	4
OG0011409	Cellular Component	cell part(GO:0044464)	4
OG0011409	Cellular Component	cell(GO:0005623)	4
OG0011409	Cellular Component	organelle(GO:0043226)	4
OG0011412	Biological Process	metabolic process(GO:0008152)	3
OG0011412	Biological Process	response to stimulus(GO:0050896)	3
OG0011412	Cellular Component	cell part(GO:0044464)	3
OG0011412	Cellular Component	cell(GO:0005623)	3
OG0011412	Cellular Component	organelle part(GO:0044422)	3
OG0011412	Cellular Component	organelle(GO:0043226)	3
OG0011412	Molecular Function	catalytic activity(GO:0003824)	3
OG0011438	Cellular Component	cell part(GO:0044464)	3
OG0011438	Cellular Component	cell(GO:0005623)	3
OG0011528	Cellular Component	cell part(GO:0044464)	4

OG0011528	Cellular Component	cell(GO:0005623)	4
OG0011629	Cellular Component	cell part(GO:0044464)	4
OG0011629	Cellular Component	cell(GO:0005623)	4
OG0011629	Cellular Component	membrane(GO:0016020)	4
OG0011855	Biological Process	cellular process(GO:0009987)	3
OG0011855	Biological Process	metabolic process(GO:0008152)	3
OG0011855	Biological Process	response to stimulus(GO:0050896)	3
OG0011855	Cellular Component	cell part(GO:0044464)	3
OG0011855	Cellular Component	cell(GO:0005623)	3
OG0011855	Cellular Component	organelle(GO:0043226)	3
OG0011855	Cellular Component	protein-containing	3
OG0012085	Biological Process	response to stimulus(GO:0050896)	1
OG0012085	Cellular Component	cell part(GO:0044464)	1
OG0012085	Cellular Component	cell(GO:0005623)	1
OG0012085	Cellular Component	membrane(GO:0016020)	6
OG0012085	Cellular Component	organelle(GO:0043226)	1
OG0012166	Biological Process	cellular process(GO:0009987)	3
OG0012166	Biological Process	metabolic process(GO:0008152)	3
OG0012166	Biological Process	response to stimulus(GO:0050896)	3
OG0012166	Cellular Component	cell part(GO:0044464)	3
OG0012166	Cellular Component	cell(GO:0005623)	3
OG0012166	Cellular Component	organelle(GO:0043226)	3
OG0012504	Cellular Component	cell part(GO:0044464)	9
OG0012504	Cellular Component	cell(GO:0005623)	9
OG0012504	Cellular Component	membrane part(GO:0044425)	6
OG0012504	Cellular Component	membrane(GO:0016020)	9
OG0012504	Cellular Component	organelle part(GO:0044422)	9
OG0012504	Cellular Component	organelle(GO:0043226)	9
OG0012504	Cellular Component	protein-containing	6
OG0012514	Biological Process	cellular process(GO:0009987)	1
OG0012514	Biological Process	metabolic process(GO:0008152)	1
OG0012514	Cellular Component	cell part(GO:0044464)	3
OG0012514	Cellular Component	cell(GO:0005623)	3
OG0012514	Cellular Component	membrane(GO:0016020)	1

OG0012514	Cellular Component	organelle part(GO:0044422)	2
OG0012514	Cellular Component	organelle(GO:0043226)	2
OG0012514	Molecular Function	binding(GO:0005488)	1
OG0012514	Molecular Function	catalytic activity(GO:0003824)	1
OG0012663	Biological Process	biological	3
OG0012663	Biological Process	cellular process(GO:0009987)	3
OG0012663	Biological Process	metabolic process(GO:0008152)	3
OG0012663	Biological Process	positive regulation of	3
OG0012663	Biological Process	regulation of biological	3
OG0012663	Cellular Component	cell part(GO:0044464)	3
OG0012663	Cellular Component	cell(GO:0005623)	3
OG0012663	Cellular Component	organelle(GO:0043226)	3
OG0012761	Cellular Component	cell part(GO:0044464)	1
OG0012761	Cellular Component	cell(GO:0005623)	1
OG0012761	Cellular Component	organelle(GO:0043226)	1
OG0013047	Cellular Component	cell part(GO:0044464)	3
OG0013047	Cellular Component	cell(GO:0005623)	3
OG0013047	Cellular Component	organelle(GO:0043226)	3
OG0013114	Cellular Component	cell part(GO:0044464)	3
OG0013114	Cellular Component	cell(GO:0005623)	3
OG0013114	Cellular Component	extracellular region(GO:0005576)	3
OG0013114	Cellular Component	organelle(GO:0043226)	3
OG0013143	Cellular Component	cell part(GO:0044464)	1
OG0013143	Cellular Component	cell(GO:0005623)	1
OG0013143	Cellular Component	organelle(GO:0043226)	1
OG0013149	Biological Process	cellular process(GO:0009987)	5
OG0013149	Biological Process	metabolic process(GO:0008152)	5
OG0013149	Molecular Function	binding(GO:0005488)	5
OG0013268	Cellular Component	cell part(GO:0044464)	5
OG0013268	Cellular Component	cell(GO:0005623)	5
OG0013268	Cellular Component	membrane(GO:0016020)	5
OG0013268	Cellular Component	organelle part(GO:0044422)	5
OG0013268	Cellular Component	organelle(GO:0043226)	5
OG0013270	Biological Process	cellular process(GO:0009987)	2

OG0013270	Biological Process	localization(GO:0051179)	1
OG0013270	Biological Process	metabolic process(GO:0008152)	2
OG0013270	Cellular Component	cell part(GO:0044464)	7
OG0013270	Cellular Component	cell(GO:0005623)	7
OG0013270	Cellular Component	membrane(GO:0016020)	1
OG0013270	Cellular Component	organelle part(GO:0044422)	1
OG0013270	Cellular Component	organelle(GO:0043226)	7
OG0013270	Cellular Component	protein-containing	1
OG0013270	Molecular Function	binding(GO:0005488)	1
OG0013270	Molecular Function	catalytic activity(GO:0003824)	1
OG0013270	Molecular Function	structural molecule	1
OG0013270	Molecular Function	transporter activity(GO:0005215)	1
OG0013295	Biological Process	cellular process(GO:0009987)	2
OG0013295	Biological Process	metabolic process(GO:0008152)	2
OG0013295	Cellular Component	cell part(GO:0044464)	2
OG0013295	Cellular Component	cell(GO:0005623)	2
OG0013295	Cellular Component	organelle(GO:0043226)	2
OG0013295	Molecular Function	catalytic activity(GO:0003824)	2
OG0013311	Biological Process	cellular component organization	2
OG0013311	Biological Process	cellular process(GO:0009987)	2
OG0013311	Biological Process	metabolic process(GO:0008152)	2
OG0013311	Cellular Component	cell part(GO:0044464)	2
OG0013311	Cellular Component	cell(GO:0005623)	2
OG0013311	Cellular Component	nucleoid(GO:0009295)	2
OG0013311	Cellular Component	organelle part(GO:0044422)	2
OG0013311	Cellular Component	organelle(GO:0043226)	2
OG0013329	Biological Process	biological	2
OG0013329	Biological Process	metabolic process(GO:0008152)	2
OG0013329	Biological Process	multi-organism	2
OG0013329	Biological Process	multicellular organismal	2
OG0013329	Biological Process	regulation of biological	2
OG0013329	Biological Process	reproduction(GO:0000003)	2
OG0013329	Biological Process	reproductive process(GO:0022414)	2
OG0013329	Cellular Component	cell part(GO:0044464)	2

OG0013329	Cellular Component	cell(GO:0005623)	2
OG0013329	Cellular Component	membrane(GO:0016020)	2
OG0013329	Molecular Function	binding(GO:0005488)	2
OG0013329	Molecular Function	molecular function	2
OG0013336	Biological Process	cellular process(GO:0009987)	2
OG0013336	Biological Process	metabolic process(GO:0008152)	2
OG0013336	Cellular Component	cell part(GO:0044464)	2
OG0013336	Cellular Component	cell(GO:0005623)	2
OG0013336	Cellular Component	organelle(GO:0043226)	2
OG0013359	Biological Process	developmental	1
OG0013359	Biological Process	multicellular organismal	1
OG0013359	Biological Process	reproduction(GO:0000003)	1
OG0013359	Biological Process	reproductive process(GO:0022414)	1
OG0013359	Cellular Component	cell part(GO:0044464)	2
OG0013359	Cellular Component	cell(GO:0005623)	2
OG0013359	Cellular Component	membrane(GO:0016020)	1
OG0013359	Cellular Component	organelle part(GO:0044422)	2
OG0013359	Cellular Component	organelle(GO:0043226)	2
OG0013486	Biological Process	metabolic process(GO:0008152)	2
OG0013486	Cellular Component	cell part(GO:0044464)	3
OG0013486	Cellular Component	cell(GO:0005623)	3
OG0013486	Cellular Component	membrane(GO:0016020)	3
OG0013486	Cellular Component	organelle part(GO:0044422)	1
OG0013486	Cellular Component	organelle(GO:0043226)	1
OG0013486	Molecular Function	catalytic activity(GO:0003824)	2
OG0013487	Cellular Component	cell part(GO:0044464)	8
OG0013487	Cellular Component	cell(GO:0005623)	8
OG0013487	Cellular Component	membrane(GO:0016020)	8
OG0013487	Cellular Component	organelle part(GO:0044422)	8
OG0013487	Cellular Component	organelle(GO:0043226)	8
OG0013494	Biological Process	cellular component organization	2
OG0013494	Biological Process	cellular process(GO:0009987)	2
OG0013494	Biological Process	growth(GO:0040007)	2
OG0013494	Biological Process	metabolic process(GO:0008152)	2

OG0013494	Biological Process	response to stimulus(GO:0050896)	2
OG0013494	Cellular Component	cell part(GO:0044464)	2
OG0013494	Cellular Component	cell(GO:0005623)	2
OG0013494	Cellular Component	organelle(GO:0043226)	2
OG0013541	Cellular Component	cell part(GO:0044464)	2
OG0013541	Cellular Component	cell(GO:0005623)	2
OG0013541	Cellular Component	organelle(GO:0043226)	2
OG0013564	Cellular Component	cell part(GO:0044464)	10
OG0013564	Cellular Component	cell(GO:0005623)	10
OG0013564	Cellular Component	membrane(GO:0016020)	10
OG0013564	Cellular Component	organelle part(GO:0044422)	10
OG0013564	Cellular Component	organelle(GO:0043226)	10
OG0013669	Cellular Component	cell part(GO:0044464)	3
OG0013669	Cellular Component	cell(GO:0005623)	3
OG0013669	Cellular Component	membrane(GO:0016020)	3
OG0013669	Cellular Component	organelle part(GO:0044422)	3
OG0013669	Cellular Component	organelle(GO:0043226)	3
OG0013698	Biological Process	growth(GO:0040007)	3
OG0013698	Cellular Component	cell part(GO:0044464)	4
OG0013698	Cellular Component	cell(GO:0005623)	4
OG0013698	Cellular Component	membrane part(GO:0044425)	3
OG0013698	Cellular Component	membrane(GO:0016020)	4
OG0013698	Cellular Component	organelle part(GO:0044422)	1
OG0013698	Cellular Component	organelle(GO:0043226)	1
OG0013698	Cellular Component	protein-containing	3
OG0013725	Cellular Component	cell part(GO:0044464)	2
OG0013725	Cellular Component	cell(GO:0005623)	2
OG0013725	Cellular Component	organelle(GO:0043226)	2
OG0013733	Biological Process	cellular process(GO:0009987)	4
OG0013733	Biological Process	metabolic process(GO:0008152)	4
OG0013733	Cellular Component	cell part(GO:0044464)	4
OG0013733	Cellular Component	cell(GO:0005623)	4
OG0013747	Cellular Component	cell part(GO:0044464)	11
OG0013747	Cellular Component	cell(GO:0005623)	11

OG0013747	Cellular Component	organelle(GO:0043226)	11
OG0013763	Biological Process	biological	6
OG0013763	Biological Process	cellular process(GO:0009987)	6
OG0013763	Biological Process	developmental	6
OG0013763	Biological Process	metabolic process(GO:0008152)	6
OG0013763	Biological Process	multi-organism	6
OG0013763	Biological Process	multicellular organismal	6
OG0013763	Biological Process	regulation of biological	6
OG0013763	Biological Process	reproduction(GO:0000003)	6
OG0013763	Biological Process	reproductive process(GO:0022414)	6
OG0013763	Biological Process	response to stimulus(GO:0050896)	6
OG0013763	Cellular Component	cell part(GO:0044464)	6
OG0013763	Cellular Component	cell(GO:0005623)	6
OG0013763	Molecular Function	catalytic activity(GO:0003824)	6
OG0013907	Cellular Component	cell part(GO:0044464)	6
OG0013907	Cellular Component	cell(GO:0005623)	6
OG0013907	Cellular Component	organelle(GO:0043226)	6
OG0013908	Biological Process	cellular process(GO:0009987)	2
OG0013908	Biological Process	growth(GO:0040007)	2
OG0013908	Biological Process	metabolic process(GO:0008152)	2
OG0013908	Cellular Component	cell part(GO:0044464)	3
OG0013908	Cellular Component	cell(GO:0005623)	3
OG0013908	Cellular Component	membrane(GO:0016020)	2
OG0013908	Cellular Component	organelle part(GO:0044422)	2
OG0013908	Cellular Component	organelle(GO:0043226)	3
OG0013908	Cellular Component	protein-containing	2
OG0013908	Molecular Function	binding(GO:0005488)	2
OG0013908	Molecular Function	structural molecule	2
OG0013909	Cellular Component	cell part(GO:0044464)	3
OG0013909	Cellular Component	cell(GO:0005623)	3
OG0013909	Cellular Component	organelle(GO:0043226)	3
OG0013910	Biological Process	growth(GO:0040007)	1
OG0013910	Cellular Component	cell part(GO:0044464)	3
OG0013910	Cellular Component	cell(GO:0005623)	3

OG0013910	Cellular Component	membrane(GO:0016020)	3
OG0013910	Cellular Component	organelle part(GO:0044422)	2
OG0013910	Cellular Component	organelle(GO:0043226)	2
OG0013912	Biological Process	biological	4
OG0013912	Biological Process	cellular component organization	4
OG0013912	Biological Process	cellular process(GO:0009987)	4
OG0013912	Biological Process	metabolic process(GO:0008152)	4
OG0013912	Biological Process	negative regulation of	4
OG0013912	Biological Process	regulation of biological	4
OG0013912	Cellular Component	cell part(GO:0044464)	6
OG0013912	Cellular Component	cell(GO:0005623)	6
OG0013912	Cellular Component	membrane(GO:0016020)	4
OG0013912	Cellular Component	organelle part(GO:0044422)	4
OG0013912	Cellular Component	organelle(GO:0043226)	6
OG0013912	Cellular Component	protein-containing	4
OG0013912	Molecular Function	binding(GO:0005488)	4
OG0013912	Molecular Function	structural molecule	4
OG0013912	Molecular Function	translation regulator	4
OG0013913	Biological Process	cellular process(GO:0009987)	4
OG0013913	Biological Process	growth(GO:0040007)	4
OG0013913	Biological Process	metabolic process(GO:0008152)	4
OG0013913	Cellular Component	cell part(GO:0044464)	4
OG0013913	Cellular Component	cell(GO:0005623)	4
OG0013913	Cellular Component	membrane(GO:0016020)	4
OG0013913	Cellular Component	organelle part(GO:0044422)	4
OG0013913	Cellular Component	organelle(GO:0043226)	4
OG0013913	Cellular Component	protein-containing	4
OG0013913	Molecular Function	binding(GO:0005488)	1
OG0013913	Molecular Function	structural molecule	4
OG0013914	Biological Process	cellular process(GO:0009987)	2
OG0013914	Biological Process	metabolic process(GO:0008152)	2
OG0013914	Cellular Component	cell part(GO:0044464)	6
OG0013914	Cellular Component	cell(GO:0005623)	6
OG0013914	Cellular Component	membrane(GO:0016020)	4

OG0013914	Molecular Function	catalytic activity(GO:0003824)	2
OG0013971	Biological Process	cellular process(GO:0009987)	6
OG0013971	Biological Process	metabolic process(GO:0008152)	6
OG0013971	Cellular Component	cell part(GO:0044464)	6
OG0013971	Cellular Component	cell(GO:0005623)	6
OG0013971	Molecular Function	catalytic activity(GO:0003824)	6
OG0014100	Cellular Component	cell part(GO:0044464)	3
OG0014100	Cellular Component	cell(GO:0005623)	3
OG0014100	Cellular Component	membrane part(GO:0044425)	1
OG0014100	Cellular Component	membrane(GO:0016020)	3
OG0014100	Cellular Component	organelle part(GO:0044422)	3
OG0014100	Cellular Component	organelle(GO:0043226)	3
OG0014100	Cellular Component	protein-containing	1
OG0014101	Cellular Component	cell part(GO:0044464)	5
OG0014101	Cellular Component	cell(GO:0005623)	5
OG0014101	Cellular Component	membrane(GO:0016020)	5
OG0014101	Cellular Component	organelle part(GO:0044422)	5
OG0014101	Cellular Component	organelle(GO:0043226)	5
OG0014102	Biological Process	cellular process(GO:0009987)	3
OG0014102	Biological Process	growth(GO:0040007)	3
OG0014102	Biological Process	metabolic process(GO:0008152)	3
OG0014102	Cellular Component	cell part(GO:0044464)	3
OG0014102	Cellular Component	cell(GO:0005623)	3
OG0014102	Cellular Component	membrane(GO:0016020)	3
OG0014102	Molecular Function	structural molecule	3
OG0014110	Biological Process	response to stimulus(GO:0050896)	1
OG0014115	Cellular Component	cell part(GO:0044464)	3
OG0014115	Cellular Component	cell(GO:0005623)	3
OG0014115	Cellular Component	organelle(GO:0043226)	3
OG0014327	Cellular Component	cell part(GO:0044464)	3
OG0014327	Cellular Component	cell(GO:0005623)	3
OG0014327	Cellular Component	membrane(GO:0016020)	3
OG0014327	Cellular Component	organelle part(GO:0044422)	3
OG0014327	Cellular Component	organelle(GO:0043226)	3

OG0014328	Cellular Component	cell part(GO:0044464)	4
OG0014328	Cellular Component	cell(GO:0005623)	4
OG0014328	Cellular Component	membrane(GO:0016020)	4
OG0014328	Cellular Component	organelle part(GO:0044422)	4
OG0014328	Cellular Component	organelle(GO:0043226)	4
OG0014329	Cellular Component	cell part(GO:0044464)	1
OG0014329	Cellular Component	cell(GO:0005623)	1
OG0014329	Cellular Component	organelle(GO:0043226)	1
OG0014330	Cellular Component	cell part(GO:0044464)	3
OG0014330	Cellular Component	cell(GO:0005623)	3
OG0014330	Cellular Component	membrane(GO:0016020)	2
OG0014375	Biological Process	biological	3
OG0014375	Biological Process	cellular process(GO:0009987)	3
OG0014375	Biological Process	localization(GO:0051179)	3
OG0014375	Biological Process	metabolic process(GO:0008152)	3
OG0014375	Biological Process	regulation of biological	3
OG0014375	Biological Process	response to stimulus(GO:0050896)	3
OG0014375	Cellular Component	cell junction(GO:0030054)	3
OG0014375	Cellular Component	cell part(GO:0044464)	6
OG0014375	Cellular Component	cell(GO:0005623)	6
OG0014375	Cellular Component	membrane part(GO:0044425)	3
OG0014375	Cellular Component	membrane(GO:0016020)	6
OG0014375	Cellular Component	organelle(GO:0043226)	3
OG0014375	Cellular Component	symplast(GO:0055044)	3
OG0014375	Molecular Function	binding(GO:0005488)	3
OG0014375	Molecular Function	catalytic activity(GO:0003824)	3
OG0014375	Molecular Function	transporter activity(GO:0005215)	3
OG0014392	Biological Process	biological	5
OG0014392	Biological Process	cellular process(GO:0009987)	5
OG0014392	Biological Process	metabolic process(GO:0008152)	5
OG0014392	Biological Process	regulation of biological	5
OG0014392	Molecular Function	transcription regulator	5
OG0014543	Cellular Component	cell part(GO:0044464)	4
OG0014543	Cellular Component	cell(GO:0005623)	4

OG0014543	Cellular Component	membrane(GO:0016020)	4
OG0014543	Cellular Component	organelle part(GO:0044422)	4
OG0014543	Cellular Component	organelle(GO:0043226)	4
OG0014587	Cellular Component	cell part(GO:0044464)	1
OG0014587	Cellular Component	cell(GO:0005623)	1
OG0014587	Cellular Component	membrane(GO:0016020)	1
OG0014776	Cellular Component	cell part(GO:0044464)	2
OG0014776	Cellular Component	cell(GO:0005623)	2
OG0014776	Cellular Component	organelle(GO:0043226)	2
OG0014781	Cellular Component	cell part(GO:0044464)	2
OG0014781	Cellular Component	cell(GO:0005623)	2
OG0014781	Cellular Component	membrane(GO:0016020)	2
OG0014781	Cellular Component	organelle part(GO:0044422)	2
OG0014781	Cellular Component	organelle(GO:0043226)	2
OG0014782	Cellular Component	cell part(GO:0044464)	2
OG0014782	Cellular Component	cell(GO:0005623)	2
OG0014782	Cellular Component	organelle(GO:0043226)	2
OG0014783	Cellular Component	cell part(GO:0044464)	2
OG0014783	Cellular Component	cell(GO:0005623)	2
OG0014783	Cellular Component	organelle part(GO:0044422)	1
OG0014783	Cellular Component	organelle(GO:0043226)	2
OG0014785	Cellular Component	cell part(GO:0044464)	1
OG0014785	Cellular Component	cell(GO:0005623)	1
OG0014785	Cellular Component	membrane(GO:0016020)	2
OG0014785	Cellular Component	organelle part(GO:0044422)	1
OG0014785	Cellular Component	organelle(GO:0043226)	1
OG0014826	Cellular Component	cell part(GO:0044464)	5
OG0014826	Cellular Component	cell(GO:0005623)	5
OG0014826	Cellular Component	membrane(GO:0016020)	1
OG0014826	Cellular Component	organelle part(GO:0044422)	1
OG0014826	Cellular Component	organelle(GO:0043226)	5
OG0014827	Cellular Component	cell part(GO:0044464)	5
OG0014827	Cellular Component	cell(GO:0005623)	5
OG0014827	Cellular Component	membrane(GO:0016020)	5

OG0014827	Cellular Component	organelle part(GO:0044422)	5
OG0014827	Cellular Component	organelle(GO:0043226)	5
OG0014854	Cellular Component	cell part(GO:0044464)	4
OG0014854	Cellular Component	cell(GO:0005623)	4
OG0014854	Cellular Component	organelle(GO:0043226)	4
OG0015129	Biological Process	multi-organism	1
OG0015129	Biological Process	response to stimulus(GO:0050896)	1
OG0015453	Biological Process	cellular process(GO:0009987)	5
OG0015453	Biological Process	growth(GO:0040007)	5
OG0015453	Biological Process	metabolic process(GO:0008152)	5
OG0015453	Cellular Component	cell part(GO:0044464)	5
OG0015453	Cellular Component	cell(GO:0005623)	5
OG0015453	Cellular Component	membrane(GO:0016020)	5
OG0015453	Molecular Function	catalytic activity(GO:0003824)	5
OG0015754	Cellular Component	cell part(GO:0044464)	2
OG0015754	Cellular Component	cell(GO:0005623)	2
OG0015754	Cellular Component	membrane(GO:0016020)	1
OG0015754	Cellular Component	organelle(GO:0043226)	1
OG0015901	Biological Process	cellular process(GO:0009987)	6
OG0015901	Biological Process	metabolic process(GO:0008152)	6
OG0015901	Cellular Component	cell part(GO:0044464)	6
OG0015901	Cellular Component	cell(GO:0005623)	6
OG0015901	Cellular Component	membrane(GO:0016020)	6
OG0015901	Molecular Function	catalytic activity(GO:0003824)	6
OG0015903	Biological Process	cellular process(GO:0009987)	4
OG0015903	Biological Process	metabolic process(GO:0008152)	4
OG0015903	Molecular Function	catalytic activity(GO:0003824)	4
OG0016357	Cellular Component	cell part(GO:0044464)	2
OG0016357	Cellular Component	cell(GO:0005623)	2
OG0016357	Cellular Component	organelle(GO:0043226)	2
OG0016362	Biological Process	cellular process(GO:0009987)	1
OG0016362	Biological Process	metabolic process(GO:0008152)	1
OG0016362	Molecular Function	catalytic activity(GO:0003824)	1
OG0016390	Biological Process	biological	2

OG0016390	Biological Process	cellular process(GO:0009987)	2
OG0016390	Biological Process	developmental	2
OG0016390	Biological Process	multi-organism	2
OG0016390	Biological Process	multicellular organismal	2
OG0016390	Biological Process	negative regulation of	2
OG0016390	Biological Process	regulation of biological	2
OG0016390	Biological Process	response to stimulus(GO:0050896)	2
OG0016390	Cellular Component	cell junction(GO:0030054)	2
OG0016390	Cellular Component	cell part(GO:0044464)	2
OG0016390	Cellular Component	cell(GO:0005623)	2
OG0016390	Cellular Component	extracellular region(GO:0005576)	2
OG0016390	Cellular Component	membrane(GO:0016020)	2
OG0016390	Cellular Component	membrane-enclosed	2
OG0016390	Cellular Component	organelle part(GO:0044422)	2
OG0016390	Cellular Component	organelle(GO:0043226)	2
OG0016390	Cellular Component	protein-containing	2
OG0016390	Cellular Component	symplast(GO:0055044)	2
OG0016914	Cellular Component	cell part(GO:0044464)	3
OG0016914	Cellular Component	cell(GO:0005623)	3
OG0016914	Cellular Component	organelle(GO:0043226)	3
OG0017009	Biological Process	metabolic process(GO:0008152)	2
OG0017009	Molecular Function	catalytic activity(GO:0003824)	2
OG0017011	Biological Process	developmental	3
OG0017011	Biological Process	multi-organism	3
OG0017011	Biological Process	multicellular organismal	3
OG0017011	Biological Process	response to stimulus(GO:0050896)	3
OG0017011	Cellular Component	cell part(GO:0044464)	3
OG0017011	Cellular Component	cell(GO:0005623)	3
OG0017011	Molecular Function	binding(GO:0005488)	3
OG0017733	Cellular Component	cell part(GO:0044464)	5
OG0017733	Cellular Component	cell(GO:0005623)	5
OG0017734	Cellular Component	cell part(GO:0044464)	4
OG0017734	Cellular Component	cell(GO:0005623)	4
OG0017735	Biological Process	cellular process(GO:0009987)	1

OG0017735	Biological Process	metabolic process(GO:0008152)	1
OG0017735	Biological Process	response to stimulus(GO:0050896)	2
OG0017735	Cellular Component	membrane(GO:0016020)	3
OG0017735	Molecular Function	catalytic activity(GO:0003824)	1
OG0017741	Biological Process	metabolic process(GO:0008152)	6
OG0017741	Cellular Component	cell part(GO:0044464)	6
OG0017741	Cellular Component	cell(GO:0005623)	6
OG0017741	Cellular Component	membrane(GO:0016020)	6
OG0017741	Molecular Function	catalytic activity(GO:0003824)	6
OG0018790	Biological Process	cellular process(GO:0009987)	1
OG0018790	Biological Process	localization(GO:0051179)	1
OG0018790	Biological Process	metabolic process(GO:0008152)	1
OG0018790	Cellular Component	cell part(GO:0044464)	3
OG0018790	Cellular Component	cell(GO:0005623)	3
OG0018790	Cellular Component	membrane(GO:0016020)	1
OG0018790	Molecular Function	catalytic activity(GO:0003824)	1
OG0018790	Molecular Function	transporter activity(GO:0005215)	1
OG0018792	Biological Process	cellular process(GO:0009987)	3
OG0018792	Biological Process	metabolic process(GO:0008152)	3
OG0018792	Cellular Component	cell part(GO:0044464)	3
OG0018792	Cellular Component	cell(GO:0005623)	3
OG0018792	Cellular Component	membrane(GO:0016020)	3
OG0018792	Molecular Function	catalytic activity(GO:0003824)	3
OG0018793	Biological Process	cellular process(GO:0009987)	2
OG0018793	Biological Process	metabolic process(GO:0008152)	2
OG0018793	Molecular Function	catalytic activity(GO:0003824)	2
OG0018795	Biological Process	cellular component organization	4
OG0018795	Biological Process	cellular process(GO:0009987)	4
OG0018795	Biological Process	metabolic process(GO:0008152)	4
OG0018795	Cellular Component	cell part(GO:0044464)	4
OG0018795	Cellular Component	cell(GO:0005623)	4
OG0018795	Cellular Component	organelle(GO:0043226)	4
OG0018795	Cellular Component	protein-containing	4
OG0018795	Molecular Function	binding(GO:0005488)	4

OG0018795	Molecular Function	catalytic activity(GO:0003824)	4
OG0018799	Biological Process	cellular component organization	5
OG0018799	Biological Process	cellular process(GO:0009987)	5
OG0018799	Biological Process	metabolic process(GO:0008152)	5
OG0018799	Molecular Function	binding(GO:0005488)	5
OG0018802	Cellular Component	cell part(GO:0044464)	3
OG0018802	Cellular Component	cell(GO:0005623)	3
OG0018802	Cellular Component	membrane(GO:0016020)	3
OG0018802	Cellular Component	organelle(GO:0043226)	3
OG0018803	Biological Process	biological	4
OG0018803	Biological Process	cellular process(GO:0009987)	4
OG0018803	Biological Process	metabolic process(GO:0008152)	4
OG0018803	Biological Process	regulation of biological	4
OG0018803	Molecular Function	binding(GO:0005488)	4
OG0018803	Molecular Function	catalytic activity(GO:0003824)	4
OG0018807	Cellular Component	cell part(GO:0044464)	1
OG0018807	Cellular Component	cell(GO:0005623)	1
OG0018807	Cellular Component	extracellular region(GO:0005576)	1
OG0018807	Cellular Component	membrane(GO:0016020)	1
OG0018807	Cellular Component	organelle part(GO:0044422)	1
OG0018807	Cellular Component	organelle(GO:0043226)	1
OG0018809	Biological Process	cellular process(GO:0009987)	1
OG0018809	Biological Process	metabolic process(GO:0008152)	1
OG0018809	Cellular Component	cell part(GO:0044464)	1
OG0018809	Cellular Component	cell(GO:0005623)	1
OG0018809	Molecular Function	catalytic activity(GO:0003824)	1
OG0018816	Biological Process	biological	2
OG0018816	Biological Process	cellular process(GO:0009987)	2
OG0018816	Biological Process	metabolic process(GO:0008152)	2
OG0018816	Biological Process	regulation of biological	2
OG0018816	Biological Process	response to stimulus(GO:0050896)	2
OG0018816	Cellular Component	cell part(GO:0044464)	2
OG0018816	Cellular Component	cell(GO:0005623)	2
OG0018816	Cellular Component	organelle part(GO:0044422)	2

OG0018816	Cellular Component	organelle(GO:0043226)	2
OG0018816	Molecular Function	antioxidant activity(GO:0016209)	2
OG0018816	Molecular Function	catalytic activity(GO:0003824)	2
OG0018816	Molecular Function	molecular function	2
OG0020308	Cellular Component	cell part(GO:0044464)	3
OG0020308	Cellular Component	cell(GO:0005623)	3
OG0020309	Cellular Component	membrane(GO:0016020)	3
OG0020316	Biological Process	cellular process(GO:0009987)	1
OG0020316	Biological Process	metabolic process(GO:0008152)	1
OG0020316	Biological Process	response to stimulus(GO:0050896)	1
OG0020316	Cellular Component	cell part(GO:0044464)	2
OG0020316	Cellular Component	cell(GO:0005623)	2
OG0020316	Molecular Function	catalytic activity(GO:0003824)	1
OG0020317	Biological Process	cellular component organization	3
OG0020317	Biological Process	cellular process(GO:0009987)	3
OG0020317	Biological Process	growth(GO:0040007)	3
OG0020317	Biological Process	metabolic process(GO:0008152)	3
OG0020317	Cellular Component	cell part(GO:0044464)	3
OG0020317	Cellular Component	cell(GO:0005623)	3
OG0020317	Cellular Component	membrane(GO:0016020)	3
OG0020317	Molecular Function	catalytic activity(GO:0003824)	3
OG0020318	Biological Process	cellular process(GO:0009987)	3
OG0020318	Biological Process	growth(GO:0040007)	3
OG0020318	Biological Process	metabolic process(GO:0008152)	3
OG0020318	Biological Process	multi-organism	3
OG0020318	Biological Process	response to stimulus(GO:0050896)	3
OG0020318	Cellular Component	cell part(GO:0044464)	3
OG0020318	Cellular Component	cell(GO:0005623)	3
OG0020318	Cellular Component	membrane(GO:0016020)	3
OG0020318	Cellular Component	nucleoid(GO:0009295)	3
OG0020318	Molecular Function	binding(GO:0005488)	3
OG0020318	Molecular Function	catalytic activity(GO:0003824)	3
OG0020322	Biological Process	cellular component organization	3
OG0020322	Biological Process	cellular process(GO:0009987)	3

OG0020322	Biological Process	metabolic process(GO:0008152)	3
OG0020322	Cellular Component	cell part(GO:0044464)	3
OG0020322	Cellular Component	cell(GO:0005623)	3
OG0020322	Molecular Function	catalytic activity(GO:0003824)	3
OG0020326	Biological Process	growth(GO:0040007)	3
OG0020326	Cellular Component	cell part(GO:0044464)	3
OG0020326	Cellular Component	cell(GO:0005623)	3
OG0020326	Cellular Component	membrane(GO:0016020)	3
OG0020329	Biological Process	cellular process(GO:0009987)	1
OG0020329	Biological Process	metabolic process(GO:0008152)	1
OG0020329	Biological Process	response to stimulus(GO:0050896)	1
OG0020329	Cellular Component	cell junction(GO:0030054)	1
OG0020329	Cellular Component	cell part(GO:0044464)	2
OG0020329	Cellular Component	cell(GO:0005623)	2
OG0020329	Cellular Component	membrane(GO:0016020)	1
OG0020329	Cellular Component	membrane-enclosed	1
OG0020329	Cellular Component	organelle part(GO:0044422)	1
OG0020329	Cellular Component	organelle(GO:0043226)	1
OG0020329	Cellular Component	symplast(GO:0055044)	1
OG0020329	Molecular Function	catalytic activity(GO:0003824)	1
OG0020331	Cellular Component	cell part(GO:0044464)	3
OG0020331	Cellular Component	cell(GO:0005623)	3
OG0022052	Cellular Component	cell part(GO:0044464)	1
OG0022052	Cellular Component	cell(GO:0005623)	1
OG0022053	Cellular Component	membrane(GO:0016020)	2
OG0022054	Biological Process	biological	1
OG0022054	Biological Process	cellular process(GO:0009987)	1
OG0022054	Biological Process	metabolic process(GO:0008152)	1
OG0022054	Biological Process	negative regulation of	1
OG0022054	Biological Process	regulation of biological	1
OG0022054	Cellular Component	cell part(GO:0044464)	1
OG0022054	Cellular Component	cell(GO:0005623)	1
OG0022054	Cellular Component	membrane(GO:0016020)	1
OG0022058	Cellular Component	cell part(GO:0044464)	3

OG0022058	Cellular Component	cell(GO:0005623)	3
OG0022058	Cellular Component	organelle(GO:0043226)	3
OG0022059	Cellular Component	cell part(GO:0044464)	2
OG0022059	Cellular Component	cell(GO:0005623)	2
OG0022059	Cellular Component	membrane(GO:0016020)	2
OG0022059	Cellular Component	membrane-enclosed	2
OG0022059	Cellular Component	organelle part(GO:0044422)	2
OG0022059	Cellular Component	organelle(GO:0043226)	2
OG0022060	Biological Process	cellular process(GO:0009987)	2
OG0022060	Biological Process	metabolic process(GO:0008152)	2
OG0022060	Cellular Component	cell part(GO:0044464)	2
OG0022060	Cellular Component	cell(GO:0005623)	2
OG0022060	Cellular Component	membrane(GO:0016020)	2
OG0022060	Molecular Function	binding(GO:0005488)	2
OG0022060	Molecular Function	catalytic activity(GO:0003824)	2
OG0022061	Biological Process	cellular process(GO:0009987)	2
OG0022061	Biological Process	growth(GO:0040007)	2
OG0022061	Biological Process	metabolic process(GO:0008152)	2
OG0022061	Cellular Component	cell part(GO:0044464)	2
OG0022061	Cellular Component	cell(GO:0005623)	2
OG0022061	Cellular Component	membrane(GO:0016020)	2
OG0022061	Molecular Function	binding(GO:0005488)	2
OG0022061	Molecular Function	catalytic activity(GO:0003824)	2
OG0022064	Biological Process	biological	2
OG0022064	Biological Process	cellular process(GO:0009987)	2
OG0022064	Biological Process	metabolic process(GO:0008152)	2
OG0022064	Biological Process	regulation of biological	2
OG0022064	Biological Process	response to stimulus(GO:0050896)	2
OG0022064	Cellular Component	cell part(GO:0044464)	2
OG0022064	Cellular Component	cell(GO:0005623)	2
OG0022064	Cellular Component	organelle(GO:0043226)	2
OG0022064	Molecular Function	transcription regulator	2
OG0022065	Biological Process	cellular component organization	1
OG0022065	Biological Process	cellular process(GO:0009987)	1

OG0022065	Biological Process	growth(GO:0040007)	1
OG0022065	Biological Process	metabolic process(GO:0008152)	1
OG0022065	Biological Process	response to stimulus(GO:0050896)	1
OG0022065	Cellular Component	cell part(GO:0044464)	1
OG0022065	Cellular Component	cell(GO:0005623)	1
OG0022065	Cellular Component	membrane-enclosed	1
OG0022065	Cellular Component	organelle part(GO:0044422)	1
OG0022065	Cellular Component	organelle(GO:0043226)	1
OG0022065	Cellular Component	protein-containing	1
OG0022065	Molecular Function	binding(GO:0005488)	1
OG0022065	Molecular Function	catalytic activity(GO:0003824)	1
OG0022068	Biological Process	biological	2
OG0022068	Biological Process	cellular process(GO:0009987)	2
OG0022068	Biological Process	developmental	2
OG0022068	Biological Process	metabolic process(GO:0008152)	2
OG0022068	Biological Process	multicellular organismal	2
OG0022068	Biological Process	regulation of biological	2
OG0022068	Biological Process	reproduction(GO:0000003)	2
OG0022068	Biological Process	reproductive process(GO:0022414)	2
OG0022068	Cellular Component	cell part(GO:0044464)	2
OG0022068	Cellular Component	cell(GO:0005623)	2
OG0022068	Cellular Component	organelle(GO:0043226)	2
OG0022068	Molecular Function	transcription regulator	2
OG0022072	Cellular Component	cell part(GO:0044464)	2
OG0022072	Cellular Component	cell(GO:0005623)	2
OG0022072	Cellular Component	membrane(GO:0016020)	2
OG0022075	Biological Process	cellular process(GO:0009987)	2
OG0022075	Biological Process	metabolic process(GO:0008152)	2
OG0022075	Molecular Function	catalytic activity(GO:0003824)	2
OG0022081	Cellular Component	cell part(GO:0044464)	2
OG0022081	Cellular Component	cell(GO:0005623)	2
OG0022085	Cellular Component	cell part(GO:0044464)	3
OG0022085	Cellular Component	cell(GO:0005623)	3
OG0022085	Cellular Component	organelle(GO:0043226)	3

OG0022086	Cellular Component	cell part(GO:0044464)	2
OG0022086	Cellular Component	cell(GO:0005623)	2
OG0022086	Cellular Component	membrane(GO:0016020)	2
OG0022089	Biological Process	cellular process(GO:0009987)	2
OG0022089	Biological Process	growth(GO:0040007)	2
OG0022089	Biological Process	metabolic process(GO:0008152)	2
OG0022089	Cellular Component	cell part(GO:0044464)	2
OG0022089	Cellular Component	cell(GO:0005623)	2
OG0022089	Cellular Component	membrane(GO:0016020)	2
OG0022089	Molecular Function	binding(GO:0005488)	2
OG0024184	Cellular Component	cell part(GO:0044464)	1
OG0024184	Cellular Component	cell(GO:0005623)	1
OG0024188	Biological Process	cellular component organization	2
OG0024188	Biological Process	cellular process(GO:0009987)	2
OG0024188	Cellular Component	cell part(GO:0044464)	2
OG0024188	Cellular Component	cell(GO:0005623)	2
OG0024188	Cellular Component	organelle(GO:0043226)	2
OG0024191	Cellular Component	cell part(GO:0044464)	2
OG0024191	Cellular Component	cell(GO:0005623)	2
OG0024191	Cellular Component	membrane(GO:0016020)	2
OG0024196	Biological Process	cellular process(GO:0009987)	2
OG0024196	Biological Process	metabolic process(GO:0008152)	2
OG0024196	Cellular Component	cell part(GO:0044464)	2
OG0024196	Cellular Component	cell(GO:0005623)	2
OG0024196	Cellular Component	organelle part(GO:0044422)	2
OG0024196	Cellular Component	organelle(GO:0043226)	2
OG0024196	Cellular Component	protein-containing	2
OG0024196	Molecular Function	catalytic activity(GO:0003824)	2
OG0024213	Biological Process	developmental	2
OG0024213	Biological Process	multicellular organismal	2
OG0024213	Biological Process	reproduction(GO:0000003)	2
OG0024213	Biological Process	reproductive process(GO:0022414)	2
OG0024219	Cellular Component	cell part(GO:0044464)	2
OG0024219	Cellular Component	cell(GO:0005623)	2

OG0024219	Cellular Component	membrane(GO:0016020)	2
OG0024219	Cellular Component	organelle part(GO:0044422)	2
OG0024219	Cellular Component	organelle(GO:0043226)	2
OG0024223	Biological Process	biological	2
OG0024223	Biological Process	cellular process(GO:0009987)	2
OG0024223	Biological Process	metabolic process(GO:0008152)	2
OG0024223	Biological Process	regulation of biological	2
OG0024223	Cellular Component	cell part(GO:0044464)	2
OG0024223	Cellular Component	cell(GO:0005623)	2
OG0024223	Cellular Component	organelle(GO:0043226)	2
OG0024223	Molecular Function	transcription regulator	2
OG0024224	Biological Process	biological	1
OG0024224	Biological Process	cellular process(GO:0009987)	1
OG0024224	Biological Process	metabolic process(GO:0008152)	1
OG0024224	Biological Process	regulation of biological	1
OG0024224	Molecular Function	transcription regulator	1
OG0024231	Biological Process	biological	2
OG0024231	Biological Process	cellular process(GO:0009987)	2
OG0024231	Biological Process	regulation of biological	2
OG0024231	Biological Process	response to stimulus(GO:0050896)	2
OG0024231	Biological Process	signaling(GO:0023052)	2
OG0024231	Cellular Component	cell part(GO:0044464)	2
OG0024231	Cellular Component	cell(GO:0005623)	2
OG0024231	Cellular Component	organelle(GO:0043226)	2
OG0024237	Cellular Component	cell part(GO:0044464)	1
OG0024237	Cellular Component	cell(GO:0005623)	1
OG0024237	Cellular Component	organelle(GO:0043226)	1
OG0024237	Cellular Component	protein-containing	1
OG0024242	Biological Process	biological	2
OG0024242	Biological Process	cellular process(GO:0009987)	2
OG0024242	Biological Process	developmental	2
OG0024242	Biological Process	metabolic process(GO:0008152)	2
OG0024242	Biological Process	multicellular organismal	2
OG0024242	Biological Process	regulation of biological	2

OG0024242	Cellular Component	cell part(GO:0044464)	2
OG0024242	Cellular Component	cell(GO:0005623)	2
OG0024242	Cellular Component	organelle(GO:0043226)	2
OG0024242	Molecular Function	transcription regulator	2
OG0024249	Biological Process	response to stimulus(GO:0050896)	2
OG0024249	Cellular Component	cell part(GO:0044464)	2
OG0024249	Cellular Component	cell(GO:0005623)	2
OG0024249	Cellular Component	membrane(GO:0016020)	2
OG0024250	Cellular Component	cell part(GO:0044464)	2
OG0024250	Cellular Component	cell(GO:0005623)	2
OG0024250	Cellular Component	membrane(GO:0016020)	2
OG0024250	Cellular Component	organelle(GO:0043226)	2
OG0024261	Biological Process	biological	2
OG0024261	Biological Process	cellular process(GO:0009987)	2
OG0024261	Biological Process	metabolic process(GO:0008152)	2
OG0024261	Biological Process	regulation of biological	2
OG0024261	Biological Process	response to stimulus(GO:0050896)	2
OG0024261	Cellular Component	cell part(GO:0044464)	2
OG0024261	Cellular Component	cell(GO:0005623)	2
OG0024261	Cellular Component	organelle(GO:0043226)	2
OG0024261	Molecular Function	binding(GO:0005488)	2
OG0024261	Molecular Function	transcription regulator	2
OG0024264	Biological Process	cellular process(GO:0009987)	2
OG0024264	Biological Process	metabolic process(GO:0008152)	2
OG0024264	Cellular Component	cell part(GO:0044464)	2
OG0024264	Cellular Component	cell(GO:0005623)	2
OG0024264	Molecular Function	catalytic activity(GO:0003824)	2
OG0024265	Cellular Component	cell part(GO:0044464)	2
OG0024265	Cellular Component	cell(GO:0005623)	2
OG0024265	Cellular Component	membrane(GO:0016020)	2
OG0024273	Biological Process	metabolic process(GO:0008152)	2
OG0024273	Cellular Component	cell part(GO:0044464)	2
OG0024273	Cellular Component	cell(GO:0005623)	2
OG0024273	Molecular Function	catalytic activity(GO:0003824)	2

OG0024286	Cellular Component	cell part(GO:0044464)	2
OG0024286	Cellular Component	cell(GO:0005623)	2
OG0024286	Cellular Component	organelle(GO:0043226)	2
