# Supplementary material for: A high-quality genome provides insights into the new taxonomic status and genomic characteristics of Cladopus chinensis (Podostemaceae)
Source: Hortic Res. 2020 Apr 1;7:46. doi: 10.1038/s41438-020-0269-5 (PMC7109043; doi:10.1038/s41438-020-0269-5)
Supplement: Supplementary file 9 — Table S11. KEGG enrichment of the contraction familes genes identified in the C. chinensis [file 41438_2020_269_MOESM9_ESM.pdf]

OG	KEGG class	KEGG sub class	Description	KEGG ID	Number of genes
OG0000029	Metabolism	Biosynthesis of	Phenylpropanoid	ko00940	5
OG0000029	Metabolism	Carbohydrate	Starch and sucrose	ko00500	5
OG0000029	Metabolism	Metabolism of other	Cyanoamino acid	ko00460	5
OG0000036	Metabolism	Biosynthesis of	Isoflavonoid	ko00943	2
OG0000046	Organismal Systems	Environmental	Plant-pathogen	ko04626	2
OG0000051	Metabolism	Energy metabolism	Oxidative	ko00190	2
OG0000074	Human Diseases	Cancers: Overview	Transcriptional	ko05202	3
OG0000074	Human Diseases	Immune diseases	Systemic lupus	ko05322	3
OG0000074	Human Diseases	Substance dependence	Alcoholism	ko05034	3
OG0000083	Cellular Processes	Cell motility	Regulation of actin	ko04810	2
OG0000083	Cellular Processes	Cellular community -	Adherens junction	ko04520	2
OG0000083	Cellular Processes	Cellular community -	Focal adhesion	ko04510	2
OG0000083	Cellular Processes	Cellular community -	Tight junction	ko04530	2
OG0000083	Cellular Processes	Transport and	Phagosome	ko04145	2
OG0000083	Environmental	Signal transduction	MAPK signaling	ko04013	2
OG0000083	Environmental	Signal transduction	MAPK signaling	ko04010	2
OG0000083	Environmental	Signal transduction	PI3K-Akt signaling	ko04151	2
OG0000083	Environmental	Signal transduction	Rap1 signaling	ko04015	2
OG0000083	Environmental	Signal transduction	Ras signaling	ko04014	2
OG0000083	Environmental	Signal transduction	Sphingolipid	ko04071	2
OG0000083	Environmental	Signal transduction	VEGF signaling	ko04370	2
OG0000083	Environmental	Signal transduction	Wnt signaling	ko04310	2
OG0000083	Environmental	Signal transduction	cAMP signaling	ko04024	2
OG0000083	Human Diseases	Cancers: Overview	Choline metabolism	ko05231	2
OG0000083	Human Diseases	Cancers: Overview	Pathways in cancer	ko05200	2
OG0000083	Human Diseases	Cancers: Overview	Proteoglycans in	ko05205	2
OG0000083	Human Diseases	Cancers: Overview	Viral carcinogenesis	ko05203	2
OG0000083	Human Diseases	Cancers: Specific	Colorectal cancer	ko05210	2
OG0000083	Human Diseases	Cancers: Specific	Pancreatic cancer	ko05212	2
OG0000083	Human Diseases	Cancers: Specific	Renal cell carcinoma	ko05211	2
OG0000083	Human Diseases	Cardiovascular	Fluid shear stress	ko05418	2
OG0000083	Human Diseases	Cardiovascular	Viral myocarditis	ko05416	2
OG0000083	Human Diseases	Endocrine and	AGE-RAGE signaling	ko04933	2

OG0000083	Human Diseases	Endocrine and	Non-alcoholic fatty	ko04932	2
OG0000083	Human Diseases	Infectious diseases:	Bacterial invasion	ko05100	2
OG0000083	Human Diseases	Infectious diseases:	Epithelial cell	ko05120	2
OG0000083	Human Diseases	Infectious diseases:	Salmonella infection	ko05132	2
OG0000083	Human Diseases	Infectious diseases:	Shigellosis	ko05131	2
OG0000083	Human Diseases	Infectious diseases:	Epstein-Barr virus	ko05169	2
OG0000083	Human Diseases	Infectious diseases:	Human	ko05163	2
OG0000083	Human Diseases	Infectious diseases:	Human	ko05170	2
OG0000083	Human Diseases	Infectious diseases:	Kaposi sarcoma-	ko05167	2
OG0000083	Human Diseases	Neurodegenerative	Amyotrophic lateral	ko05014	2
OG0000083	Organismal Systems	Development	Axon guidance	ko04360	2
OG0000083	Organismal Systems	Development	Osteoclast	ko04380	2
OG0000083	Organismal Systems	Digestive system	Pancreatic secretion	ko04972	2
OG0000083	Organismal Systems	Immune system	B cell receptor	ko04662	2
OG0000083	Organismal Systems	Immune system	Chemokine signaling	ko04062	2
OG0000083	Organismal Systems	Immune system	Fc epsilon RI	ko04664	2
OG0000083	Organismal Systems	Immune system	Fc gamma R-mediated	ko04666	2
OG0000083	Organismal Systems	Immune system	Leukocyte	ko04670	2
OG0000083	Organismal Systems	Immune system	Natural killer cell	ko04650	2
OG0000083	Organismal Systems	Immune system	Toll-like receptor	ko04620	2
OG0000083	Organismal Systems	Nervous system	Neurotrophin	ko04722	2
OG0000129	Environmental	Signal transduction	FoxO signaling	ko04068	2
OG0000129	Human Diseases	Cancers: Overview	Viral carcinogenesis	ko05203	2
OG0000129	Human Diseases	Infectious diseases:	Epstein-Barr virus	ko05169	2
OG0000129	Human Diseases	Infectious diseases:	Herpes simplex	ko05168	2
OG0000144	Environmental	Signal transduction	FoxO signaling	ko04068	1
OG0000144	Environmental	Signal transduction	Hedgehog signaling	ko04341	1
OG0000144	Environmental	Signal transduction	Hedgehog signaling	ko04340	1
OG0000144	Environmental	Signal transduction	Hippo signaling	ko04391	1
OG0000144	Environmental	Signal transduction	Hippo signaling	ko04392	1
OG0000144	Environmental	Signal transduction	Hippo signaling	ko04390	1
OG0000144	Environmental	Signal transduction	Wnt signaling	ko04310	1
OG0000144	Organismal Systems	Environmental	Circadian rhythm -	ko04711	1
OG0000144	Organismal Systems	Environmental	Circadian rhythm	ko04710	1

OG0000166	Environmental	Signal transduction	MAPK signaling	ko04016	2
OG0000166	Environmental	Signal transduction	Plant hormone signal	ko04075	2
OG0000166	Organismal Systems	Environmental	Plant-pathogen	ko04626	2
OG0000207	Environmental	Signal transduction	MAPK signaling	ko04016	1
OG0000207	Environmental	Signal transduction	Plant hormone signal	ko04075	1
OG0000207	Human Diseases	Endocrine and	Insulin resistance	ko04931	1
OG0000209	Environmental	Signal transduction	MAPK signaling	ko04016	2
OG0000209	Environmental	Signal transduction	Plant hormone signal	ko04075	2
OG0000238	Environmental	Signal transduction	Plant hormone signal	ko04075	2
OG0000254	Cellular Processes	Cell growth and	Cellular senescence	ko04218	2
OG0000254	Cellular Processes	Cell growth and	Oocyte meiosis	ko04114	2
OG0000254	Environmental	Signal transduction	Apelin signaling	ko04371	2
OG0000254	Environmental	Signal transduction	Calcium signaling	ko04020	2
OG0000254	Environmental	Signal transduction	MAPK signaling	ko04016	2
OG0000254	Environmental	Signal transduction	Phosphatidylinositol	ko04070	2
OG0000254	Environmental	Signal transduction	Rap1 signaling	ko04015	2
OG0000254	Environmental	Signal transduction	Ras signaling	ko04014	2
OG0000254	Environmental	Signal transduction	cAMP signaling	ko04024	2
OG0000254	Environmental	Signal transduction	cGMP-PKG signaling	ko04022	2
OG0000254	Human Diseases	Cancers: Overview	Pathways in cancer	ko05200	2
OG0000254	Human Diseases	Cancers: Specific	Glioma	ko05214	2
OG0000254	Human Diseases	Cardiovascular	Fluid shear stress	ko05418	2
OG0000254	Human Diseases	Infectious diseases:	Pertussis	ko05133	2
OG0000254	Human Diseases	Infectious diseases:	Tuberculosis	ko05152	2
OG0000254	Human Diseases	Infectious diseases:	Human	ko05163	2
OG0000254	Human Diseases	Infectious diseases:	Human	ko05170	2
OG0000254	Human Diseases	Infectious diseases:	Kaposi sarcoma-	ko05167	2
OG0000254	Human Diseases	Neurodegenerative	Alzheimer disease	ko05010	2
OG0000254	Human Diseases	Substance dependence	Alcoholism	ko05034	2
OG0000254	Human Diseases	Substance dependence	Amphetamine	ko05031	2
OG0000254	Organismal Systems	Circulatory system	Adrenergic signaling	ko04261	2
OG0000254	Organismal Systems	Circulatory system	Vascular smooth	ko04270	2
OG0000254	Organismal Systems	Digestive system	Gastric acid	ko04971	2
OG0000254	Organismal Systems	Digestive system	Salivary secretion	ko04970	2

OG0000254	Organismal Systems	Endocrine system	Aldosterone	ko04925	2
OG0000254	Organismal Systems	Endocrine system	Estrogen signaling	ko04915	2
OG0000254	Organismal Systems	Endocrine system	Glucagon signaling	ko04922	2
OG0000254	Organismal Systems	Endocrine system	GnRH signaling	ko04912	2
OG0000254	Organismal Systems	Endocrine system	Insulin signaling	ko04910	2
OG0000254	Organismal Systems	Endocrine system	Melanogenesis	ko04916	2
OG0000254	Organismal Systems	Endocrine system	Oxytocin signaling	ko04921	2
OG0000254	Organismal Systems	Endocrine system	Renin secretion	ko04924	2
OG0000254	Organismal Systems	Environmental	Circadian	ko04713	2
OG0000254	Organismal Systems	Environmental	Plant-pathogen	ko04626	2
OG0000254	Organismal Systems	Immune system	C-type lectin	ko04625	2
OG0000254	Organismal Systems	Nervous system	Dopaminergic synapse	ko04728	2
OG0000254	Organismal Systems	Nervous system	Long-term	ko04720	2
OG0000254	Organismal Systems	Nervous system	Neurotrophin	ko04722	2
OG0000254	Organismal Systems	Sensory system	Inflammatory	ko04750	2
OG0000254	Organismal Systems	Sensory system	Olfactory	ko04740	2
OG0000254	Organismal Systems	Sensory system	Phototransduction -	ko04745	2
OG0000254	Organismal Systems	Sensory system	Phototransduction	ko04744	2
OG0000311	Genetic Information	Transcription	Spliceosome	ko03040	1
OG0000311	Human Diseases	Infectious diseases:	Shigellosis	ko05131	1
OG0000324	Cellular Processes	Cellular community -	Signaling pathways	ko04550	2
OG0000324	Human Diseases	Cancers: Overview	Transcriptional	ko05202	2
OG0000346	Metabolism	Nucleotide	Purine metabolism	ko00230	1
OG0000346	Metabolism	Nucleotide	Pyrimidine	ko00240	1
OG0000356	Environmental	Signal transduction	MAPK signaling	ko04010	1
OG0000356	Environmental	Signal transduction	NF-kappa B signaling	ko04064	1
OG0000356	Human Diseases	Infectious diseases:	Pertussis	ko05133	1
OG0000356	Human Diseases	Infectious diseases:	Tuberculosis	ko05152	1
OG0000356	Human Diseases	Infectious diseases:	Chagas disease	ko05142	1
OG0000356	Human Diseases	Infectious diseases:	Leishmaniasis	ko05140	1
OG0000356	Human Diseases	Infectious diseases:	Toxoplasmosis	ko05145	1
OG0000356	Human Diseases	Infectious diseases:	Epstein-Barr virus	ko05169	1
OG0000356	Human Diseases	Infectious diseases:	Human	ko05170	1
OG0000356	Human Diseases	Infectious diseases:	Measles	ko05162	1

OG0000356	Organismal Systems	Immune system	Toll and Imd	ko04624	1
OG0000356	Organismal Systems	Immune system	Toll-like receptor	ko04620	1
OG0000356	Organismal Systems	Nervous system	Neurotrophin	ko04722	1
OG0000358	Cellular Processes	Cell growth and	Apoptosis	ko04210	1
OG0000358	Cellular Processes	Cellular community -	Gap junction	ko04540	1
OG0000358	Cellular Processes	Cellular community -	Tight junction	ko04530	1
OG0000358	Cellular Processes	Transport and	Phagosome	ko04145	1
OG0000358	Human Diseases	Infectious diseases:	Pathogenic	ko05130	1
OG0000365	Cellular Processes	Cell growth and	Cellular senescence	ko04218	1
OG0000365	Cellular Processes	Cell growth and	Oocyte meiosis	ko04114	1
OG0000365	Environmental	Signal transduction	Calcium signaling	ko04020	1
OG0000365	Environmental	Signal transduction	MAPK signaling	ko04010	1
OG0000365	Environmental	Signal transduction	VEGF signaling	ko04370	1
OG0000365	Environmental	Signal transduction	Wnt signaling	ko04310	1
OG0000365	Environmental	Signal transduction	cGMP-PKG signaling	ko04022	1
OG0000365	Human Diseases	Infectious diseases:	Tuberculosis	ko05152	1
OG0000365	Human Diseases	Infectious diseases:	Human T-cell	ko05166	1
OG0000365	Human Diseases	Infectious diseases:	Human	ko05163	1
OG0000365	Human Diseases	Infectious diseases:	Human	ko05170	1
OG0000365	Human Diseases	Infectious diseases:	Kaposi sarcoma-	ko05167	1
OG0000365	Human Diseases	Neurodegenerative	Alzheimer disease	ko05010	1
OG0000365	Human Diseases	Neurodegenerative	Amyotrophic lateral	ko05014	1
OG0000365	Human Diseases	Substance dependence	Amphetamine	ko05031	1
OG0000365	Organismal Systems	Development	Axon guidance	ko04360	1
OG0000365	Organismal Systems	Development	Osteoclast	ko04380	1
OG0000365	Organismal Systems	Endocrine system	Glucagon signaling	ko04922	1
OG0000365	Organismal Systems	Endocrine system	Oxytocin signaling	ko04921	1
OG0000365	Organismal Systems	Endocrine system	Renin secretion	ko04924	1
OG0000365	Organismal Systems	Immune system	B cell receptor	ko04662	1
OG0000365	Organismal Systems	Immune system	C-type lectin	ko04625	1
OG0000365	Organismal Systems	Immune system	Natural killer cell	ko04650	1
OG0000365	Organismal Systems	Immune system	T cell receptor	ko04660	1
OG0000365	Organismal Systems	Immune system	Th1 and Th2 cell	ko04658	1
OG0000365	Organismal Systems	Immune system	Th17 cell	ko04659	1

OG0000365	Organismal Systems	Nervous system	Glutamatergic	ko04724	1
OG0000365	Organismal Systems	Nervous system	Long-term	ko04720	1
OG0000374	Metabolism	Metabolism of	Carotenoid	ko00906	1
OG0000376	Cellular Processes	Cell growth and	Cell cycle - yeast	ko04111	1
OG0000376	Cellular Processes	Cell growth and	Cell cycle	ko04110	1
OG0000376	Cellular Processes	Cell growth and	Oocyte meiosis	ko04114	1
OG0000376	Environmental	Signal transduction	Hedgehog signaling	ko04341	1
OG0000376	Environmental	Signal transduction	Hedgehog signaling	ko04340	1
OG0000376	Environmental	Signal transduction	TGF-beta signaling	ko04350	1
OG0000376	Environmental	Signal transduction	Wnt signaling	ko04310	1
OG0000376	Genetic Information	Folding, sorting and	Protein processing	ko04141	1
OG0000376	Genetic Information	Folding, sorting and	Ubiquitin mediated	ko04120	1
OG0000376	Human Diseases	Cancers: Overview	Pathways in cancer	ko05200	1
OG0000376	Human Diseases	Endocrine and	Non-alcoholic fatty	ko04932	1
OG0000376	Human Diseases	Infectious diseases:	Herpes simplex	ko05168	1
OG0000376	Human Diseases	Infectious diseases:	Human	ko05170	1
OG0000376	Human Diseases	Neurodegenerative	Alzheimer disease	ko05010	1
OG0000376	Human Diseases	Neurodegenerative	Huntington disease	ko05016	1
OG0000376	Human Diseases	Neurodegenerative	Parkinson disease	ko05012	1
OG0000376	Metabolism	Energy metabolism	Oxidative	ko00190	1
OG0000376	Organismal Systems	Environmental	Circadian rhythm	ko04710	1
OG0000376	Organismal Systems	Environmental	Thermogenesis	ko04714	1
OG0000376	Organismal Systems	Nervous system	Retrograde	ko04723	1
OG0000395	Human Diseases	Cancers: Overview	Central carbon	ko05230	1
OG0000395	Human Diseases	Cancers: Overview	Viral carcinogenesis	ko05203	1
OG0000395	Human Diseases	Endocrine and	Type II diabetes	ko04930	1
OG0000395	Human Diseases	Infectious diseases:	Human papillomavirus	ko05165	1
OG0000395	Metabolism	Carbohydrate	Glycolysis /	ko00010	1
OG0000395	Metabolism	Carbohydrate	Pyruvate metabolism	ko00620	1
OG0000395	Metabolism	Nucleotide	Purine metabolism	ko00230	1
OG0000395	Organismal Systems	Endocrine system	Glucagon signaling	ko04922	1
OG0000462	Metabolism	Energy metabolism	Nitrogen metabolism	ko00910	1
OG0000510	Cellular Processes	Transport and	Phagosome	ko04145	1
OG0000510	Environmental	Signal transduction	mTOR signaling	ko04150	1

OG0000510	Human Diseases	Immune diseases	Rheumatoid arthritis	ko05323	1
OG0000510	Human Diseases	Infectious diseases:	Epithelial cell	ko05120	1
OG0000510	Human Diseases	Infectious diseases:	Vibrio cholerae	ko05110	1
OG0000510	Human Diseases	Infectious diseases:	Human papillomavirus	ko05165	1
OG0000510	Metabolism	Energy metabolism	Oxidative	ko00190	1
OG0000510	Organismal Systems	Excretory system	Collecting duct acid	ko04966	1
OG0000510	Organismal Systems	Nervous system	Synaptic vesicle	ko04721	1
OG0000526	Metabolism	Carbohydrate	Starch and sucrose	ko00500	2
OG0000532	Cellular Processes	Cell motility	Regulation of actin	ko04810	2
OG0000532	Environmental	Signal transduction	MAPK signaling	ko04013	2
OG0000532	Environmental	Signal transduction	Rap1 signaling	ko04015	2
OG0000532	Human Diseases	Infectious diseases:	Salmonella infection	ko05132	2
OG0000532	Human Diseases	Infectious diseases:	Shigellosis	ko05131	2
OG0000550	Human Diseases	Infectious diseases:	Toxoplasmosis	ko05145	1
OG0000550	Organismal Systems	Environmental	Plant-pathogen	ko04626	1
OG0000555	Cellular Processes	Cell growth and	p53 signaling	ko04115	1
OG0000610	Genetic Information	Transcription	Spliceosome	ko03040	1
OG0000610	Human Diseases	Infectious diseases:	Herpes simplex	ko05168	1
OG0000676	Metabolism	Nucleotide	Pyrimidine	ko00240	1
OG0000726	Environmental	Signal transduction	MAPK signaling	ko04016	1
OG0000726	Environmental	Signal transduction	Plant hormone signal	ko04075	1
OG0000734	Metabolism	Biosynthesis of	Phenylpropanoid	ko00940	2
OG0000751	Metabolism	Amino acid	Lysine degradation	ko00310	1
OG0000751	Organismal Systems	Aging	Longevity regulating	ko04211	1
OG0000755	Cellular Processes	Transport and	Peroxisome	ko04146	1
OG0000755	Human Diseases	Neurodegenerative	Amyotrophic lateral	ko05014	1
OG0000755	Human Diseases	Neurodegenerative	Huntington disease	ko05016	1
OG0000755	Human Diseases	Neurodegenerative	Prion diseases	ko05020	1
OG0000755	Organismal Systems	Aging	Longevity regulating	ko04213	1
OG0000785	Genetic Information	Folding, sorting and	RNA degradation	ko03018	2
OG0000785	Genetic Information	Translation	RNA transport	ko03013	2
OG0000785	Genetic Information	Translation	mRNA surveillance	ko03015	2
OG0000838	Metabolism	Metabolism of	Terpenoid backbone	ko00900	1
OG0000866	Metabolism	Lipid metabolism	Linoleic acid	ko00591	2

OG0000866	Metabolism	Lipid metabolism	alpha-Linolenic acid	ko00592	1
OG0000871	Metabolism	Nucleotide	Pyrimidine	ko00240	1
OG0000871	Metabolism	Xenobiotics	Drug metabolism -	ko00983	1
OG0000881	Environmental	Signal transduction	Hippo signaling	ko04391	2
OG0000926	Metabolism	Lipid metabolism	Arachidonic acid	ko00590	2
OG0000926	Metabolism	Metabolism of other	Glutathione	ko00480	2
OG0000926	Organismal Systems	Endocrine system	Thyroid hormone	ko04918	2
OG0000933	Metabolism	Lipid metabolism	Biosynthesis of	ko01040	1
OG0000933	Metabolism	Lipid metabolism	Fatty acid	ko00061	1
OG0000948	Metabolism	Carbohydrate	Pyruvate metabolism	ko00620	1
OG0000948	Metabolism	Energy metabolism	Carbon fixation in	ko00710	1
OG0000948	Metabolism	Energy metabolism	Carbon fixation	ko00720	1
OG0000948	Metabolism	Energy metabolism	Methane metabolism	ko00680	1
OG0000953	Genetic Information	Translation	Ribosome	ko03010	1
OG0000972	Human Diseases	Infectious diseases:	Legionellosis	ko05134	1
OG0001014	Metabolism	Carbohydrate	Fructose and mannose	ko00051	1
OG0001014	Metabolism	Carbohydrate	Glycolysis /	ko00010	1
OG0001014	Metabolism	Carbohydrate	Pentose phosphate	ko00030	1
OG0001014	Metabolism	Energy metabolism	Carbon fixation in	ko00710	1
OG0001014	Metabolism	Energy metabolism	Methane metabolism	ko00680	1
OG0001025	Cellular Processes	Cell growth and	Necroptosis	ko04217	1
OG0001025	Environmental	Signal transduction	Two-component system	ko02020	1
OG0001025	Metabolism	Amino acid	Alanine, aspartate	ko00250	1
OG0001025	Metabolism	Amino acid	Arginine	ko00220	1
OG0001025	Metabolism	Carbohydrate	Glyoxylate and	ko00630	1
OG0001025	Metabolism	Energy metabolism	Nitrogen metabolism	ko00910	1
OG0001025	Organismal Systems	Nervous system	GABAergic synapse	ko04727	1
OG0001025	Organismal Systems	Nervous system	Glutamatergic	ko04724	1
OG0001058	Cellular Processes	Transport and	Endocytosis	ko04144	2
OG0001058	Environmental	Signal transduction	Phospholipase D	ko04072	2
OG0001058	Environmental	Signal transduction	Ras signaling	ko04014	2
OG0001058	Environmental	Signal transduction	Sphingolipid	ko04071	2
OG0001058	Environmental	Signal transduction	cAMP signaling	ko04024	2
OG0001058	Human Diseases	Cancers: Overview	Choline metabolism	ko05231	2

OG0001058	Metabolism	Lipid metabolism	Ether lipid	ko00565	2
OG0001058	Metabolism	Lipid metabolism	Glycerophospholipid	ko00564	2
OG0001058	Organismal Systems	Endocrine system	GnRH signaling	ko04912	2
OG0001058	Organismal Systems	Endocrine system	Parathyroid hormone	ko04928	2
OG0001058	Organismal Systems	Immune system	Fc gamma R-mediated	ko04666	2
OG0001058	Organismal Systems	Nervous system	Glutamatergic	ko04724	2
OG0001080	Metabolism	Amino acid	Arginine and proline	ko00330	1
OG0001080	Metabolism	Amino acid	Cysteine and	ko00270	1
OG0001091	Cellular Processes	Cell growth and	Necroptosis	ko04217	1
OG0001091	Environmental	Signal transduction	PI3K-Akt signaling	ko04151	1
OG0001091	Genetic Information	Folding, sorting and	Protein processing	ko04141	1
OG0001091	Human Diseases	Cancers: Overview	Pathways in cancer	ko05200	1
OG0001091	Human Diseases	Cancers: Specific	Prostate cancer	ko05215	1
OG0001091	Human Diseases	Cardiovascular	Fluid shear stress	ko05418	1
OG0001091	Organismal Systems	Endocrine system	Estrogen signaling	ko04915	1
OG0001091	Organismal Systems	Endocrine system	Progesterone-	ko04914	1
OG0001091	Organismal Systems	Environmental	Plant-pathogen	ko04626	1
OG0001091	Organismal Systems	Immune system	Antigen processing	ko04612	1
OG0001091	Organismal Systems	Immune system	IL-17 signaling	ko04657	1
OG0001091	Organismal Systems	Immune system	NOD-like receptor	ko04621	1
OG0001091	Organismal Systems	Immune system	Th17 cell	ko04659	1
OG0001120	Environmental	Signal transduction	MAPK signaling	ko04010	2
OG0001120	Environmental	Signal transduction	NF-kappa B signaling	ko04064	2
OG0001120	Human Diseases	Infectious diseases:	Pertussis	ko05133	2
OG0001120	Human Diseases	Infectious diseases:	Tuberculosis	ko05152	2
OG0001120	Human Diseases	Infectious diseases:	Chagas disease	ko05142	2
OG0001120	Human Diseases	Infectious diseases:	Leishmaniasis	ko05140	2
OG0001120	Human Diseases	Infectious diseases:	Toxoplasmosis	ko05145	2
OG0001120	Human Diseases	Infectious diseases:	Epstein-Barr virus	ko05169	2
OG0001120	Human Diseases	Infectious diseases:	Human	ko05170	2
OG0001120	Human Diseases	Infectious diseases:	Influenza A	ko05164	2
OG0001120	Human Diseases	Infectious diseases:	Measles	ko05162	2
OG0001120	Organismal Systems	Immune system	NOD-like receptor	ko04621	2
OG0001120	Organismal Systems	Immune system	Toll and Imd	ko04624	2

OG0001120	Organismal Systems	Immune system	Toll-like receptor	ko04620	2
OG0001120	Organismal Systems	Nervous system	Neurotrophin	ko04722	2
OG0001129	Metabolism	Metabolism of	Thiamine metabolism	ko00730	1
OG0001156	Environmental	Signal transduction	MAPK signaling	ko04011	2
OG0001156	Metabolism	Lipid metabolism	Glycerophospholipid	ko00564	2
OG0001183	Genetic Information	Folding, sorting and	Protein processing	ko04141	1
OG0001210	Environmental	Signal transduction	Hippo signaling	ko04392	1
OG0001210	Environmental	Signal transduction	MAPK signaling	ko04011	1
OG0001259	Environmental	Signal transduction	mTOR signaling	ko04150	1
OG0001259	Genetic Information	Folding, sorting and	Protein processing	ko04141	1
OG0001259	Genetic Information	Translation	RNA transport	ko03013	1
OG0001273	Genetic Information	Translation	Ribosome	ko03010	1
OG0001280	Metabolism	Carbohydrate	Starch and sucrose	ko00500	1
OG0001282	Environmental	Membrane transport	ABC transporters	ko02010	2
OG0001282	Human Diseases	Cancers: Overview	MicroRNAs in cancer	ko05206	2
OG0001282	Human Diseases	Cancers: Specific	Gastric cancer	ko05226	2
OG0001282	Organismal Systems	Digestive system	Bile secretion	ko04976	2
OG0001399	Metabolism	Amino acid	Cysteine and	ko00270	1
OG0001399	Metabolism	Carbohydrate	Citrate cycle (TCA	ko00020	1
OG0001399	Metabolism	Carbohydrate	Glyoxylate and	ko00630	1
OG0001399	Metabolism	Carbohydrate	Pyruvate metabolism	ko00620	1
OG0001399	Metabolism	Energy metabolism	Carbon fixation in	ko00710	1
OG0001399	Organismal Systems	Excretory system	Proximal tubule	ko04964	1
OG0001437	Environmental	Signal transduction	MAPK signaling	ko04016	1
OG0001437	Environmental	Signal transduction	Plant hormone signal	ko04075	1
OG0001490	Environmental	Signal transduction	Plant hormone signal	ko04075	1
OG0001557	Metabolism	Carbohydrate	Citrate cycle (TCA	ko00020	1
OG0001583	Genetic Information	Translation	Ribosome	ko03010	1
OG0001590	Genetic Information	Folding, sorting and	RNA degradation	ko03018	1
OG0001590	Human Diseases	Endocrine and	Type I diabetes	ko04940	1
OG0001590	Human Diseases	Infectious diseases:	Legionellosis	ko05134	1
OG0001590	Human Diseases	Infectious diseases:	Tuberculosis	ko05152	1
OG0001590	Organismal Systems	Aging	Longevity regulating	ko04212	1
OG0001629	Genetic Information	Transcription	Spliceosome	ko03040	1

OG0001721	Genetic Information	Translation	Ribosome	ko03010	1
OG0001740	Metabolism	Amino acid	Cysteine and	ko00270	1
OG0001740	Metabolism	Amino acid	Glycine, serine and	ko00260	1
OG0001740	Metabolism	Amino acid	Lysine biosynthesis	ko00300	1
OG0001740	Metabolism	Biosynthesis of	Monobactam	ko00261	1
OG0001770	Metabolism	Amino acid	Tryptophan	ko00380	1
OG0001804	Genetic Information	Folding, sorting and	Ubiquitin mediated	ko04120	1
OG0001813	Genetic Information	Transcription	RNA polymerase	ko03020	1
OG0001813	Metabolism	Nucleotide	Purine metabolism	ko00230	1
OG0001813	Metabolism	Nucleotide	Pyrimidine	ko00240	1
OG0001813	Organismal Systems	Immune system	Cytosolic DNA-	ko04623	1
OG0001829	Genetic Information	Transcription	Spliceosome	ko03040	1
OG0001838	Genetic Information	Translation	Ribosome	ko03010	1
OG0001946	Cellular Processes	Transport and	Autophagy - animal	ko04140	1
OG0001946	Environmental	Signal transduction	AMPK signaling	ko04152	1
OG0001946	Environmental	Signal transduction	Apelin signaling	ko04371	1
OG0001946	Environmental	Signal transduction	ErbB signaling	ko04012	1
OG0001946	Environmental	Signal transduction	HIF-1 signaling	ko04066	1
OG0001946	Environmental	Signal transduction	PI3K-Akt signaling	ko04151	1
OG0001946	Environmental	Signal transduction	TGF-beta signaling	ko04350	1
OG0001946	Environmental	Signal transduction	mTOR signaling	ko04150	1
OG0001946	Human Diseases	Cancers: Overview	Choline metabolism	ko05231	1
OG0001946	Human Diseases	Cancers: Overview	Pathways in cancer	ko05200	1
OG0001946	Human Diseases	Cancers: Overview	Proteoglycans in	ko05205	1
OG0001946	Human Diseases	Cancers: Specific	Acute myeloid	ko05221	1
OG0001946	Human Diseases	Cancers: Specific	Breast cancer	ko05224	1
OG0001946	Human Diseases	Cancers: Specific	Colorectal cancer	ko05210	1
OG0001946	Human Diseases	Cancers: Specific	Gastric cancer	ko05226	1
OG0001946	Human Diseases	Cancers: Specific	Hepatocellular	ko05225	1
OG0001946	Human Diseases	Cancers: Specific	Pancreatic cancer	ko05212	1
OG0001946	Human Diseases	Drug resistance:	EGFR tyrosine kinase	ko01521	1
OG0001946	Human Diseases	Drug resistance:	Endocrine resistance	ko01522	1
OG0001946	Human Diseases	Endocrine and	Insulin resistance	ko04931	1
OG0001946	Human Diseases	Infectious diseases:	Human	ko05163	1

OG0001946	Human Diseases	Infectious diseases:	Human	ko05170	1
OG0001946	Human Diseases	Infectious diseases:	Human papillomavirus	ko05165	1
OG0001946	Organismal Systems	Aging	Longevity regulating	ko04213	1
OG0001946	Organismal Systems	Aging	Longevity regulating	ko04212	1
OG0001946	Organismal Systems	Aging	Longevity regulating	ko04211	1
OG0001946	Organismal Systems	Endocrine system	Insulin signaling	ko04910	1
OG0001946	Organismal Systems	Environmental	Thermogenesis	ko04714	1
OG0001946	Organismal Systems	Immune system	Fc gamma R-mediated	ko04666	1
OG0001986	Genetic Information	Translation	Ribosome	ko03010	1
OG0002003	Organismal Systems	Endocrine system	PPAR signaling	ko03320	1
OG0002038	Metabolism	Metabolism of	Ubiquinone and other	ko00130	1
OG0002134	Genetic Information	Translation	Ribosome	ko03010	1
OG0002225	Genetic Information	Translation	Ribosome	ko03010	1
OG0002310	Genetic Information	Translation	Ribosome	ko03010	1
OG0002337	Cellular Processes	Transport and	Autophagy - animal	ko04140	1
OG0002337	Cellular Processes	Transport and	Autophagy - yeast	ko04138	1
OG0002337	Cellular Processes	Transport and	Endocytosis	ko04144	1
OG0002337	Cellular Processes	Transport and	Mitophagy - animal	ko04137	1
OG0002337	Cellular Processes	Transport and	Phagosome	ko04145	1
OG0002337	Human Diseases	Infectious diseases:	Salmonella infection	ko05132	1
OG0002337	Human Diseases	Infectious diseases:	Tuberculosis	ko05152	1
OG0002337	Human Diseases	Infectious diseases:	Amoebiasis	ko05146	1
OG0002348	Metabolism	Metabolism of	Zeatin biosynthesis	ko00908	1
OG0002355	Metabolism	Amino acid	Cysteine and	ko00270	1
OG0002415	Cellular Processes	Transport and	Autophagy - yeast	ko04138	1
OG0002415	Genetic Information	Folding, sorting and	SNARE interactions	ko04130	1
OG0002455	Genetic Information	Translation	Ribosome	ko03010	1
OG0002964	Genetic Information	Transcription	Basal transcription	ko03022	1
OG0002964	Human Diseases	Cancers: Overview	Viral carcinogenesis	ko05203	1
OG0002964	Human Diseases	Infectious diseases:	Herpes simplex	ko05168	1
OG0002964	Human Diseases	Infectious diseases:	Human T-cell	ko05166	1
OG0002964	Human Diseases	Infectious diseases:	Human papillomavirus	ko05165	1
OG0002964	Human Diseases	Neurodegenerative	Huntington disease	ko05016	1
OG0003012	Cellular Processes	Transport and	Mitophagy - animal	ko04137	1

OG0003012	Cellular Processes	Transport and	Mitophagy - yeast	ko04139	1
OG0003027	Cellular Processes	Cell growth and	Necroptosis	ko04217	1
OG0003027	Human Diseases	Immune diseases	Systemic lupus	ko05322	1
OG0003027	Human Diseases	Substance dependence	Alcoholism	ko05034	1
OG0003164	Genetic Information	Folding, sorting and	Ubiquitin mediated	ko04120	1
OG0003428	Organismal Systems	Endocrine system	Insulin signaling	ko04910	1
OG0003503	Cellular Processes	Cell growth and	Ferroptosis	ko04216	1
OG0003503	Cellular Processes	Cellular community -	Quorum sensing	ko02024	1
OG0003503	Cellular Processes	Transport and	Peroxisome	ko04146	1
OG0003503	Metabolism	Lipid metabolism	Fatty acid	ko00061	1
OG0003503	Metabolism	Lipid metabolism	Fatty acid	ko00071	1
OG0003503	Organismal Systems	Endocrine system	Adipocytokine	ko04920	1
OG0003503	Organismal Systems	Endocrine system	PPAR signaling	ko03320	1
OG0003503	Organismal Systems	Environmental	Thermogenesis	ko04714	1
OG0003549	Metabolism	Carbohydrate	Ascorbate and	ko00053	1
OG0003549	Metabolism	Metabolism of other	Glutathione	ko00480	1
OG0003776	Human Diseases	Cardiovascular	Fluid shear stress	ko05418	1
OG0003776	Organismal Systems	Immune system	NOD-like receptor	ko04621	1
OG0003784	Metabolism	Carbohydrate	Starch and sucrose	ko00500	1
OG0003784	Organismal Systems	Digestive system	Carbohydrate	ko04973	1
OG0003888	Environmental	Signal transduction	HIF-1 signaling	ko04066	1
OG0003888	Genetic Information	Folding, sorting and	RNA degradation	ko03018	1
OG0003888	Metabolism	Carbohydrate	Glycolysis /	ko00010	1
OG0003888	Metabolism	Energy metabolism	Methane metabolism	ko00680	1
OG0004289	Metabolism	Amino acid	Cysteine and	ko00270	1
OG0004289	Metabolism	Carbohydrate	Citrate cycle (TCA	ko00020	1
OG0004289	Metabolism	Carbohydrate	Glyoxylate and	ko00630	1
OG0004289	Metabolism	Carbohydrate	Pyruvate metabolism	ko00620	1
OG0004289	Metabolism	Energy metabolism	Carbon fixation in	ko00710	1
OG0004753	Organismal Systems	Endocrine system	Estrogen signaling	ko04915	1
OG0004917	Metabolism	Metabolism of	Vitamin B6	ko00750	1
