# Supplementary material for: A high-quality genome provides insights into the new taxonomic status and genomic characteristics of Cladopus chinensis (Podostemaceae)
Source: Hortic Res. 2020 Apr 1;7:46. doi: 10.1038/s41438-020-0269-5 (PMC7109043; doi:10.1038/s41438-020-0269-5)
Supplement: Supplementary file 10 — Table S12. GO enrichment of the contraction familes genes identified in the C. chinensis [file 41438_2020_269_MOESM10_ESM.pdf]

OG	GO class	GO description	Number of genes
OG0000005	Cellular Component	cell part(GO:0044464)	3
OG0000005	Cellular Component	cell(GO:0005623)	3
OG0000005	Cellular Component	membrane(GO:0016020)	3
OG0000011	Biological Process	biological adhesion(GO:0022610)	1
OG0000011	Biological Process	biological	1
OG0000011	Biological Process	cellular process(GO:0009987)	1
OG0000011	Biological Process	metabolic process(GO:0008152)	1
OG0000011	Biological Process	negative regulation of	1
OG0000011	Biological Process	positive regulation of	1
OG0000011	Biological Process	regulation of biological	1
OG0000011	Biological Process	response to stimulus(GO:0050896)	1
OG0000011	Cellular Component	cell part(GO:0044464)	1
OG0000011	Cellular Component	cell(GO:0005623)	1
OG0000011	Cellular Component	membrane-enclosed	1
OG0000011	Cellular Component	organelle part(GO:0044422)	1
OG0000011	Cellular Component	organelle(GO:0043226)	1
OG0000011	Cellular Component	protein-containing	1
OG0000011	Molecular Function	transcription regulator	1
OG0000016	Biological Process	biological	3
OG0000016	Biological Process	cellular component organization	3
OG0000016	Biological Process	cellular process(GO:0009987)	7
OG0000016	Biological Process	developmental	7
OG0000016	Biological Process	growth(GO:0040007)	3
OG0000016	Biological Process	metabolic process(GO:0008152)	7
OG0000016	Biological Process	multi-organism	4
OG0000016	Biological Process	multicellular organismal	7
OG0000016	Biological Process	regulation of biological	3
OG0000016	Biological Process	reproduction(GO:0000003)	4
OG0000016	Biological Process	reproductive process(GO:0022414)	4
OG0000016	Biological Process	response to stimulus(GO:0050896)	7
OG0000016	Biological Process	signaling(GO:0023052)	3
OG0000016	Cellular Component	cell junction(GO:0030054)	7
OG0000016	Cellular Component	cell part(GO:0044464)	11

OG0000016	Cellular Component	cell(GO:0005623)	11
OG0000016	Cellular Component	membrane(GO:0016020)	11
OG0000016	Cellular Component	organelle(GO:0043226)	4
OG0000016	Cellular Component	symplast(GO:0055044)	7
OG0000016	Molecular Function	catalytic activity(GO:0003824)	7
OG0000020	Biological Process	cellular component organization	1
OG0000020	Biological Process	cellular process(GO:0009987)	1
OG0000020	Biological Process	metabolic process(GO:0008152)	1
OG0000020	Cellular Component	extracellular region(GO:0005576)	1
OG0000020	Molecular Function	catalytic activity(GO:0003824)	1
OG0000031	Biological Process	biological	7
OG0000031	Biological Process	cellular component organization	6
OG0000031	Biological Process	cellular process(GO:0009987)	7
OG0000031	Biological Process	developmental	6
OG0000031	Biological Process	metabolic process(GO:0008152)	7
OG0000031	Biological Process	multicellular organismal	6
OG0000031	Biological Process	positive regulation of	6
OG0000031	Biological Process	regulation of biological	7
OG0000031	Biological Process	reproduction(GO:0000003)	3
OG0000031	Biological Process	reproductive process(GO:0022414)	3
OG0000031	Cellular Component	cell part(GO:0044464)	7
OG0000031	Cellular Component	cell(GO:0005623)	7
OG0000031	Cellular Component	organelle(GO:0043226)	7
OG0000031	Molecular Function	transcription regulator	7
OG0000036	Biological Process	response to stimulus(GO:0050896)	2
OG0000036	Cellular Component	cell part(GO:0044464)	2
OG0000036	Cellular Component	cell(GO:0005623)	2
OG0000036	Cellular Component	membrane(GO:0016020)	2
OG0000036	Cellular Component	organelle(GO:0043226)	2
OG0000039	Cellular Component	extracellular region(GO:0005576)	4
OG0000040	Biological Process	biological	3
OG0000040	Biological Process	cellular component organization	8
OG0000040	Biological Process	cellular process(GO:0009987)	8
OG0000040	Biological Process	growth(GO:0040007)	2

OG0000040	Biological Process	metabolic process(GO:0008152)	8
OG0000040	Biological Process	multi-organism	2
OG0000040	Biological Process	positive regulation of	2
OG0000040	Biological Process	regulation of biological	3
OG0000040	Biological Process	response to stimulus(GO:0050896)	5
OG0000040	Cellular Component	cell junction(GO:0030054)	1
OG0000040	Cellular Component	cell part(GO:0044464)	8
OG0000040	Cellular Component	cell(GO:0005623)	8
OG0000040	Cellular Component	membrane(GO:0016020)	8
OG0000040	Cellular Component	organelle part(GO:0044422)	3
OG0000040	Cellular Component	organelle(GO:0043226)	6
OG0000040	Cellular Component	protein-containing	2
OG0000040	Cellular Component	symplast(GO:0055044)	1
OG0000046	Cellular Component	cell junction(GO:0030054)	2
OG0000046	Cellular Component	cell part(GO:0044464)	2
OG0000046	Cellular Component	cell(GO:0005623)	2
OG0000046	Cellular Component	membrane(GO:0016020)	2
OG0000046	Cellular Component	symplast(GO:0055044)	2
OG0000051	Biological Process	biological	1
OG0000051	Biological Process	cellular component organization	1
OG0000051	Biological Process	cellular process(GO:0009987)	1
OG0000051	Biological Process	localization(GO:0051179)	1
OG0000051	Biological Process	metabolic process(GO:0008152)	1
OG0000051	Cellular Component	cell junction(GO:0030054)	1
OG0000051	Cellular Component	cell part(GO:0044464)	1
OG0000051	Cellular Component	cell(GO:0005623)	1
OG0000051	Cellular Component	membrane(GO:0016020)	1
OG0000051	Cellular Component	organelle(GO:0043226)	1
OG0000051	Cellular Component	symplast(GO:0055044)	1
OG0000051	Molecular Function	catalytic activity(GO:0003824)	1
OG0000051	Molecular Function	transporter activity(GO:0005215)	1
OG0000056	Cellular Component	cell part(GO:0044464)	5
OG0000056	Cellular Component	cell(GO:0005623)	5
OG0000056	Cellular Component	organelle(GO:0043226)	5

OG0000074	Cellular Component	cell part(GO:0044464)	2
OG0000074	Cellular Component	cell(GO:0005623)	2
OG0000074	Cellular Component	organelle(GO:0043226)	2
OG0000083	Biological Process	biological	1
OG0000083	Biological Process	cellular component organization	1
OG0000083	Biological Process	cellular process(GO:0009987)	1
OG0000083	Biological Process	metabolic process(GO:0008152)	1
OG0000083	Biological Process	negative regulation of	1
OG0000083	Biological Process	regulation of biological	1
OG0000083	Biological Process	response to stimulus(GO:0050896)	1
OG0000083	Biological Process	signaling(GO:0023052)	1
OG0000083	Cellular Component	cell part(GO:0044464)	1
OG0000083	Cellular Component	cell(GO:0005623)	1
OG0000083	Cellular Component	membrane(GO:0016020)	1
OG0000083	Cellular Component	organelle(GO:0043226)	1
OG0000083	Molecular Function	catalytic activity(GO:0003824)	1
OG0000129	Cellular Component	cell part(GO:0044464)	1
OG0000129	Cellular Component	cell(GO:0005623)	1
OG0000131	Biological Process	cellular process(GO:0009987)	5
OG0000131	Biological Process	immune system	5
OG0000131	Biological Process	metabolic process(GO:0008152)	5
OG0000131	Biological Process	multi-organism	5
OG0000131	Biological Process	response to stimulus(GO:0050896)	5
OG0000131	Cellular Component	cell part(GO:0044464)	5
OG0000131	Cellular Component	cell(GO:0005623)	5
OG0000131	Cellular Component	membrane(GO:0016020)	5
OG0000131	Molecular Function	catalytic activity(GO:0003824)	5
OG0000144	Biological Process	biological	1
OG0000144	Biological Process	cellular component organization	1
OG0000144	Biological Process	cellular process(GO:0009987)	1
OG0000144	Biological Process	developmental	1
OG0000144	Biological Process	growth(GO:0040007)	1
OG0000144	Biological Process	metabolic process(GO:0008152)	1
OG0000144	Biological Process	multicellular organismal	1

OG0000144	Biological Process	response to stimulus(GO:0050896)	1
OG0000144	Cellular Component	cell part(GO:0044464)	1
OG0000144	Cellular Component	cell(GO:0005623)	1
OG0000144	Cellular Component	organelle(GO:0043226)	1
OG0000157	Biological Process	biological	2
OG0000157	Biological Process	cellular process(GO:0009987)	2
OG0000157	Biological Process	metabolic process(GO:0008152)	2
OG0000157	Biological Process	positive regulation of	1
OG0000157	Biological Process	regulation of biological	2
OG0000157	Biological Process	response to stimulus(GO:0050896)	1
OG0000157	Cellular Component	cell part(GO:0044464)	2
OG0000157	Cellular Component	cell(GO:0005623)	2
OG0000157	Cellular Component	organelle(GO:0043226)	2
OG0000157	Molecular Function	transcription regulator	2
OG0000172	Biological Process	cellular component organization	2
OG0000172	Biological Process	cellular process(GO:0009987)	2
OG0000172	Biological Process	metabolic process(GO:0008152)	2
OG0000172	Cellular Component	cell part(GO:0044464)	2
OG0000172	Cellular Component	cell(GO:0005623)	2
OG0000172	Molecular Function	catalytic activity(GO:0003824)	2
OG0000173	Biological Process	cellular process(GO:0009987)	3
OG0000173	Biological Process	metabolic process(GO:0008152)	3
OG0000173	Cellular Component	membrane(GO:0016020)	3
OG0000173	Molecular Function	catalytic activity(GO:0003824)	3
OG0000201	Cellular Component	cell junction(GO:0030054)	3
OG0000201	Cellular Component	cell part(GO:0044464)	3
OG0000201	Cellular Component	cell(GO:0005623)	3
OG0000201	Cellular Component	membrane(GO:0016020)	3
OG0000201	Cellular Component	organelle part(GO:0044422)	3
OG0000201	Cellular Component	organelle(GO:0043226)	3
OG0000201	Cellular Component	symplast(GO:0055044)	3
OG0000209	Biological Process	cellular process(GO:0009987)	2
OG0000209	Biological Process	developmental	2
OG0000209	Biological Process	metabolic process(GO:0008152)	2

OG0000209	Biological Process	multicellular organismal	2
OG0000209	Biological Process	response to stimulus(GO:0050896)	2
OG0000209	Cellular Component	cell part(GO:0044464)	2
OG0000209	Cellular Component	cell(GO:0005623)	2
OG0000209	Molecular Function	binding(GO:0005488)	2
OG0000209	Molecular Function	catalytic activity(GO:0003824)	2
OG0000229	Biological Process	cellular process(GO:0009987)	1
OG0000229	Biological Process	developmental	1
OG0000229	Biological Process	localization(GO:0051179)	1
OG0000229	Biological Process	metabolic process(GO:0008152)	1
OG0000229	Biological Process	multi-organism	1
OG0000229	Biological Process	multicellular organismal	1
OG0000229	Biological Process	reproduction(GO:0000003)	1
OG0000229	Biological Process	reproductive process(GO:0022414)	1
OG0000229	Cellular Component	cell part(GO:0044464)	1
OG0000229	Cellular Component	cell(GO:0005623)	1
OG0000229	Cellular Component	membrane(GO:0016020)	1
OG0000229	Molecular Function	binding(GO:0005488)	1
OG0000229	Molecular Function	catalytic activity(GO:0003824)	1
OG0000229	Molecular Function	transporter activity(GO:0005215)	1
OG0000252	Biological Process	developmental	1
OG0000252	Biological Process	multicellular organismal	1
OG0000252	Biological Process	reproduction(GO:0000003)	1
OG0000252	Biological Process	reproductive process(GO:0022414)	1
OG0000254	Biological Process	biological	2
OG0000254	Biological Process	cellular process(GO:0009987)	2
OG0000254	Biological Process	developmental	1
OG0000254	Biological Process	multicellular organismal	1
OG0000254	Biological Process	regulation of biological	2
OG0000254	Biological Process	response to stimulus(GO:0050896)	2
OG0000254	Biological Process	signaling(GO:0023052)	2
OG0000254	Cellular Component	cell part(GO:0044464)	1
OG0000254	Cellular Component	cell(GO:0005623)	1
OG0000254	Cellular Component	membrane(GO:0016020)	2

OG0000254	Cellular Component	organelle part(GO:0044422)	1
OG0000254	Cellular Component	organelle(GO:0043226)	1
OG0000254	Molecular Function	binding(GO:0005488)	2
OG0000255	Biological Process	developmental	2
OG0000255	Biological Process	multicellular organismal	2
OG0000255	Biological Process	reproduction(GO:0000003)	2
OG0000255	Biological Process	reproductive process(GO:0022414)	2
OG0000255	Cellular Component	cell part(GO:0044464)	2
OG0000255	Cellular Component	cell(GO:0005623)	2
OG0000255	Cellular Component	organelle(GO:0043226)	2
OG0000255	Molecular Function	binding(GO:0005488)	2
OG0000280	Biological Process	biological	2
OG0000280	Biological Process	cellular process(GO:0009987)	2
OG0000280	Biological Process	developmental	2
OG0000280	Biological Process	localization(GO:0051179)	2
OG0000280	Biological Process	metabolic process(GO:0008152)	2
OG0000280	Biological Process	multi-organism	2
OG0000280	Biological Process	positive regulation of	2
OG0000280	Biological Process	regulation of biological	2
OG0000280	Biological Process	reproduction(GO:0000003)	2
OG0000280	Biological Process	reproductive process(GO:0022414)	2
OG0000280	Cellular Component	cell part(GO:0044464)	2
OG0000280	Cellular Component	cell(GO:0005623)	2
OG0000280	Cellular Component	membrane part(GO:0044425)	2
OG0000280	Cellular Component	membrane(GO:0016020)	2
OG0000282	Cellular Component	cell part(GO:0044464)	1
OG0000282	Cellular Component	cell(GO:0005623)	1
OG0000282	Cellular Component	organelle(GO:0043226)	1
OG0000302	Cellular Component	cell part(GO:0044464)	1
OG0000302	Cellular Component	cell(GO:0005623)	1
OG0000302	Cellular Component	membrane(GO:0016020)	1
OG0000311	Biological Process	developmental	1
OG0000311	Biological Process	multicellular organismal	1
OG0000311	Biological Process	reproduction(GO:0000003)	1

OG0000311	Biological Process	reproductive process(GO:0022414)	1
OG0000311	Biological Process	response to stimulus(GO:0050896)	1
OG0000311	Cellular Component	cell part(GO:0044464)	1
OG0000311	Cellular Component	cell(GO:0005623)	1
OG0000311	Cellular Component	organelle(GO:0043226)	1
OG0000324	Biological Process	biological	2
OG0000324	Biological Process	cellular process(GO:0009987)	2
OG0000324	Biological Process	metabolic process(GO:0008152)	2
OG0000324	Biological Process	regulation of biological	2
OG0000324	Biological Process	response to stimulus(GO:0050896)	2
OG0000324	Cellular Component	cell part(GO:0044464)	2
OG0000324	Cellular Component	cell(GO:0005623)	2
OG0000324	Cellular Component	organelle(GO:0043226)	2
OG0000324	Molecular Function	transcription regulator	2
OG0000337	Biological Process	cellular component organization	1
OG0000337	Biological Process	cellular process(GO:0009987)	1
OG0000337	Biological Process	metabolic process(GO:0008152)	1
OG0000337	Cellular Component	cell part(GO:0044464)	1
OG0000337	Cellular Component	cell(GO:0005623)	1
OG0000337	Cellular Component	extracellular region(GO:0005576)	1
OG0000337	Molecular Function	catalytic activity(GO:0003824)	1
OG0000346	Cellular Component	cell part(GO:0044464)	1
OG0000346	Cellular Component	cell(GO:0005623)	1
OG0000346	Cellular Component	organelle(GO:0043226)	1
OG0000349	Cellular Component	cell part(GO:0044464)	1
OG0000349	Cellular Component	cell(GO:0005623)	1
OG0000349	Cellular Component	membrane(GO:0016020)	1
OG0000349	Cellular Component	organelle part(GO:0044422)	1
OG0000349	Cellular Component	organelle(GO:0043226)	1
OG0000352	Biological Process	multi-organism	1
OG0000352	Biological Process	response to stimulus(GO:0050896)	1
OG0000352	Cellular Component	cell part(GO:0044464)	1
OG0000352	Cellular Component	cell(GO:0005623)	1
OG0000352	Cellular Component	membrane(GO:0016020)	1

OG0000352	Cellular Component	organelle(GO:0043226)	1
OG0000355	Biological Process	developmental	1
OG0000355	Biological Process	multicellular organismal	1
OG0000355	Cellular Component	cell part(GO:0044464)	1
OG0000355	Cellular Component	cell(GO:0005623)	1
OG0000355	Cellular Component	membrane(GO:0016020)	1
OG0000356	Cellular Component	cell part(GO:0044464)	1
OG0000356	Cellular Component	cell(GO:0005623)	1
OG0000356	Cellular Component	membrane(GO:0016020)	1
OG0000358	Biological Process	cellular component organization	1
OG0000358	Biological Process	cellular process(GO:0009987)	1
OG0000358	Biological Process	response to stimulus(GO:0050896)	1
OG0000358	Cellular Component	cell junction(GO:0030054)	1
OG0000358	Cellular Component	cell part(GO:0044464)	1
OG0000358	Cellular Component	cell(GO:0005623)	1
OG0000358	Cellular Component	membrane(GO:0016020)	1
OG0000358	Cellular Component	membrane-enclosed	1
OG0000358	Cellular Component	organelle part(GO:0044422)	1
OG0000358	Cellular Component	organelle(GO:0043226)	1
OG0000358	Cellular Component	protein-containing	1
OG0000358	Cellular Component	supramolecular	1
OG0000358	Cellular Component	symplast(GO:0055044)	1
OG0000358	Molecular Function	structural molecule	1
OG0000365	Biological Process	biological	1
OG0000365	Biological Process	cellular process(GO:0009987)	1
OG0000365	Biological Process	metabolic process(GO:0008152)	1
OG0000365	Biological Process	response to stimulus(GO:0050896)	1
OG0000365	Cellular Component	cell part(GO:0044464)	1
OG0000365	Cellular Component	cell(GO:0005623)	1
OG0000365	Cellular Component	membrane(GO:0016020)	1
OG0000365	Cellular Component	protein-containing	1
OG0000365	Molecular Function	binding(GO:0005488)	1
OG0000365	Molecular Function	catalytic activity(GO:0003824)	1
OG0000376	Biological Process	biological	1

OG0000376	Biological Process	cellular component organization	1
OG0000376	Biological Process	cellular process(GO:0009987)	1
OG0000376	Biological Process	developmental	1
OG0000376	Biological Process	multicellular organismal	1
OG0000376	Biological Process	regulation of biological	1
OG0000376	Biological Process	reproduction(GO:0000003)	1
OG0000376	Biological Process	reproductive process(GO:0022414)	1
OG0000376	Biological Process	response to stimulus(GO:0050896)	1
OG0000376	Biological Process	rhythmic process(GO:0048511)	1
OG0000376	Biological Process	signaling(GO:0023052)	1
OG0000376	Cellular Component	cell part(GO:0044464)	1
OG0000376	Cellular Component	cell(GO:0005623)	1
OG0000376	Cellular Component	membrane-enclosed	1
OG0000376	Cellular Component	organelle part(GO:0044422)	1
OG0000376	Cellular Component	organelle(GO:0043226)	1
OG0000376	Cellular Component	protein-containing	1
OG0000386	Biological Process	cellular process(GO:0009987)	1
OG0000386	Biological Process	localization(GO:0051179)	1
OG0000386	Biological Process	metabolic process(GO:0008152)	1
OG0000386	Biological Process	multi-organism	1
OG0000386	Biological Process	response to stimulus(GO:0050896)	1
OG0000386	Cellular Component	cell part(GO:0044464)	1
OG0000386	Cellular Component	cell(GO:0005623)	1
OG0000386	Molecular Function	binding(GO:0005488)	1
OG0000386	Molecular Function	catalytic activity(GO:0003824)	1
OG0000393	Biological Process	cellular process(GO:0009987)	1
OG0000393	Biological Process	localization(GO:0051179)	1
OG0000393	Cellular Component	cell part(GO:0044464)	1
OG0000393	Cellular Component	cell(GO:0005623)	1
OG0000393	Cellular Component	membrane part(GO:0044425)	1
OG0000393	Cellular Component	membrane(GO:0016020)	1
OG0000393	Cellular Component	organelle part(GO:0044422)	1
OG0000393	Cellular Component	organelle(GO:0043226)	1
OG0000395	Cellular Component	cell part(GO:0044464)	1

OG0000395	Cellular Component	cell(GO:0005623)	1
OG0000419	Biological Process	cellular process(GO:0009987)	1
OG0000419	Biological Process	developmental	1
OG0000419	Biological Process	metabolic process(GO:0008152)	1
OG0000419	Biological Process	multicellular organismal	1
OG0000419	Biological Process	reproduction(GO:0000003)	1
OG0000419	Biological Process	reproductive process(GO:0022414)	1
OG0000419	Biological Process	response to stimulus(GO:0050896)	1
OG0000419	Cellular Component	cell part(GO:0044464)	1
OG0000419	Cellular Component	cell(GO:0005623)	1
OG0000419	Cellular Component	organelle(GO:0043226)	1
OG0000419	Molecular Function	catalytic activity(GO:0003824)	1
OG0000424	Biological Process	cellular process(GO:0009987)	1
OG0000424	Biological Process	metabolic process(GO:0008152)	1
OG0000424	Cellular Component	membrane(GO:0016020)	1
OG0000424	Molecular Function	catalytic activity(GO:0003824)	1
OG0000453	Cellular Component	cell part(GO:0044464)	1
OG0000453	Cellular Component	cell(GO:0005623)	1
OG0000462	Biological Process	response to stimulus(GO:0050896)	1
OG0000462	Cellular Component	membrane(GO:0016020)	1
OG0000463	Cellular Component	membrane part(GO:0044425)	2
OG0000463	Cellular Component	membrane(GO:0016020)	2
OG0000470	Biological Process	biological	4
OG0000470	Biological Process	cell proliferation(GO:0008283)	4
OG0000470	Biological Process	cellular process(GO:0009987)	4
OG0000470	Biological Process	negative regulation of	4
OG0000470	Biological Process	regulation of biological	4
OG0000470	Biological Process	response to stimulus(GO:0050896)	3
OG0000470	Cellular Component	cell part(GO:0044464)	3
OG0000470	Cellular Component	cell(GO:0005623)	3
OG0000470	Cellular Component	membrane(GO:0016020)	4
OG0000510	Biological Process	cellular process(GO:0009987)	1
OG0000510	Biological Process	localization(GO:0051179)	1
OG0000510	Biological Process	metabolic process(GO:0008152)	1

OG0000510	Cellular Component	cell part(GO:0044464)	1
OG0000510	Cellular Component	cell(GO:0005623)	1
OG0000510	Cellular Component	membrane part(GO:0044425)	1
OG0000510	Cellular Component	membrane(GO:0016020)	1
OG0000510	Cellular Component	organelle part(GO:0044422)	1
OG0000510	Cellular Component	organelle(GO:0043226)	1
OG0000510	Cellular Component	protein-containing	1
OG0000510	Molecular Function	catalytic activity(GO:0003824)	1
OG0000510	Molecular Function	transporter activity(GO:0005215)	1
OG0000518	Cellular Component	cell part(GO:0044464)	1
OG0000518	Cellular Component	cell(GO:0005623)	1
OG0000518	Cellular Component	organelle(GO:0043226)	1
OG0000524	Biological Process	biological	1
OG0000524	Biological Process	cellular process(GO:0009987)	1
OG0000524	Biological Process	metabolic process(GO:0008152)	1
OG0000524	Biological Process	multi-organism	1
OG0000524	Biological Process	regulation of biological	1
OG0000524	Biological Process	response to stimulus(GO:0050896)	1
OG0000524	Cellular Component	cell part(GO:0044464)	1
OG0000524	Cellular Component	cell(GO:0005623)	1
OG0000524	Cellular Component	membrane(GO:0016020)	1
OG0000524	Cellular Component	organelle(GO:0043226)	1
OG0000524	Molecular Function	transcription regulator	1
OG0000532	Cellular Component	cell part(GO:0044464)	2
OG0000532	Cellular Component	cell(GO:0005623)	2
OG0000532	Cellular Component	organelle(GO:0043226)	2
OG0000550	Biological Process	biological	1
OG0000550	Biological Process	cellular process(GO:0009987)	1
OG0000550	Biological Process	metabolic process(GO:0008152)	1
OG0000550	Biological Process	regulation of biological	1
OG0000550	Biological Process	response to stimulus(GO:0050896)	1
OG0000550	Biological Process	signaling(GO:0023052)	1
OG0000550	Cellular Component	cell junction(GO:0030054)	1
OG0000550	Cellular Component	cell part(GO:0044464)	1

OG0000550	Cellular Component	cell(GO:0005623)	1
OG0000550	Cellular Component	membrane(GO:0016020)	1
OG0000550	Cellular Component	organelle(GO:0043226)	1
OG0000550	Cellular Component	symplast(GO:0055044)	1
OG0000550	Molecular Function	catalytic activity(GO:0003824)	1
OG0000553	Cellular Component	cell part(GO:0044464)	1
OG0000553	Cellular Component	cell(GO:0005623)	1
OG0000553	Cellular Component	membrane(GO:0016020)	1
OG0000553	Cellular Component	organelle part(GO:0044422)	1
OG0000553	Cellular Component	organelle(GO:0043226)	1
OG0000555	Cellular Component	cell part(GO:0044464)	1
OG0000555	Cellular Component	cell(GO:0005623)	1
OG0000555	Cellular Component	membrane(GO:0016020)	1
OG0000606	Biological Process	biological	1
OG0000606	Biological Process	cellular component organization	1
OG0000606	Biological Process	cellular process(GO:0009987)	1
OG0000606	Cellular Component	cell junction(GO:0030054)	1
OG0000606	Cellular Component	cell part(GO:0044464)	1
OG0000606	Cellular Component	cell(GO:0005623)	1
OG0000606	Cellular Component	membrane part(GO:0044425)	1
OG0000606	Cellular Component	membrane(GO:0016020)	1
OG0000606	Cellular Component	organelle part(GO:0044422)	1
OG0000606	Cellular Component	organelle(GO:0043226)	1
OG0000606	Cellular Component	symplast(GO:0055044)	1
OG0000606	Molecular Function	binding(GO:0005488)	1
OG0000610	Biological Process	cellular process(GO:0009987)	1
OG0000610	Biological Process	metabolic process(GO:0008152)	1
OG0000610	Cellular Component	cell part(GO:0044464)	1
OG0000610	Cellular Component	cell(GO:0005623)	1
OG0000610	Cellular Component	membrane-enclosed	1
OG0000610	Cellular Component	organelle part(GO:0044422)	1
OG0000610	Cellular Component	organelle(GO:0043226)	1
OG0000616	Biological Process	biological	1
OG0000616	Biological Process	cellular component organization	1

OG0000616	Biological Process	cellular process(GO:0009987)	1
OG0000616	Biological Process	negative regulation of	1
OG0000616	Biological Process	regulation of biological	1
OG0000616	Biological Process	response to stimulus(GO:0050896)	1
OG0000616	Cellular Component	cell junction(GO:0030054)	1
OG0000616	Cellular Component	cell part(GO:0044464)	1
OG0000616	Cellular Component	cell(GO:0005623)	1
OG0000616	Cellular Component	membrane(GO:0016020)	1
OG0000616	Cellular Component	symplast(GO:0055044)	1
OG0000616	Molecular Function	binding(GO:0005488)	1
OG0000656	Cellular Component	cell part(GO:0044464)	1
OG0000656	Cellular Component	cell(GO:0005623)	1
OG0000656	Cellular Component	membrane(GO:0016020)	1
OG0000662	Cellular Component	cell part(GO:0044464)	1
OG0000662	Cellular Component	cell(GO:0005623)	1
OG0000662	Cellular Component	membrane(GO:0016020)	1
OG0000666	Biological Process	cellular component organization	1
OG0000666	Biological Process	cellular process(GO:0009987)	1
OG0000666	Biological Process	developmental	1
OG0000666	Biological Process	growth(GO:0040007)	1
OG0000666	Biological Process	localization(GO:0051179)	1
OG0000666	Biological Process	metabolic process(GO:0008152)	1
OG0000666	Biological Process	multi-organism	1
OG0000666	Biological Process	multicellular organismal	1
OG0000666	Biological Process	reproduction(GO:0000003)	1
OG0000666	Biological Process	reproductive process(GO:0022414)	1
OG0000666	Cellular Component	cell junction(GO:0030054)	1
OG0000666	Cellular Component	cell part(GO:0044464)	1
OG0000666	Cellular Component	cell(GO:0005623)	1
OG0000666	Cellular Component	membrane(GO:0016020)	1
OG0000666	Cellular Component	symplast(GO:0055044)	1
OG0000666	Molecular Function	transporter activity(GO:0005215)	1
OG0000676	Biological Process	response to stimulus(GO:0050896)	1
OG0000676	Cellular Component	cell part(GO:0044464)	1

OG0000676	Cellular Component	cell(GO:0005623)	1
OG0000686	Biological Process	cellular process(GO:0009987)	1
OG0000686	Biological Process	metabolic process(GO:0008152)	1
OG0000686	Cellular Component	cell part(GO:0044464)	1
OG0000686	Cellular Component	cell(GO:0005623)	1
OG0000686	Cellular Component	organelle part(GO:0044422)	1
OG0000686	Cellular Component	organelle(GO:0043226)	1
OG0000686	Molecular Function	binding(GO:0005488)	1
OG0000686	Molecular Function	molecular carrier	1
OG0000689	Biological Process	biological	1
OG0000689	Biological Process	developmental	1
OG0000689	Biological Process	multicellular organismal	1
OG0000689	Biological Process	regulation of biological	1
OG0000689	Biological Process	reproduction(GO:0000003)	1
OG0000689	Biological Process	reproductive process(GO:0022414)	1
OG0000689	Cellular Component	cell part(GO:0044464)	1
OG0000689	Cellular Component	cell(GO:0005623)	1
OG0000689	Cellular Component	organelle(GO:0043226)	1
OG0000691	Biological Process	response to stimulus(GO:0050896)	1
OG0000707	Biological Process	cellular process(GO:0009987)	2
OG0000707	Biological Process	localization(GO:0051179)	2
OG0000707	Cellular Component	cell part(GO:0044464)	2
OG0000707	Cellular Component	cell(GO:0005623)	2
OG0000707	Cellular Component	membrane(GO:0016020)	2
OG0000707	Cellular Component	organelle part(GO:0044422)	2
OG0000707	Cellular Component	organelle(GO:0043226)	2
OG0000707	Molecular Function	transporter activity(GO:0005215)	2
OG0000734	Cellular Component	extracellular region(GO:0005576)	2
OG0000738	Cellular Component	cell part(GO:0044464)	1
OG0000738	Cellular Component	cell(GO:0005623)	1
OG0000738	Cellular Component	organelle(GO:0043226)	1
OG0000751	Biological Process	biological	1
OG0000751	Biological Process	cellular component organization	1
OG0000751	Biological Process	cellular process(GO:0009987)	1

OG0000751	Biological Process	metabolic process(GO:0008152)	1
OG0000751	Biological Process	regulation of biological	1
OG0000751	Cellular Component	cell part(GO:0044464)	1
OG0000751	Cellular Component	cell(GO:0005623)	1
OG0000751	Cellular Component	organelle part(GO:0044422)	1
OG0000751	Cellular Component	organelle(GO:0043226)	1
OG0000751	Molecular Function	catalytic activity(GO:0003824)	1
OG0000755	Biological Process	biological	1
OG0000755	Biological Process	cellular process(GO:0009987)	1
OG0000755	Biological Process	detoxification(GO:0098754)	1
OG0000755	Biological Process	metabolic process(GO:0008152)	1
OG0000755	Biological Process	negative regulation of	1
OG0000755	Biological Process	regulation of biological	1
OG0000755	Biological Process	response to stimulus(GO:0050896)	1
OG0000755	Cellular Component	cell part(GO:0044464)	1
OG0000755	Cellular Component	cell(GO:0005623)	1
OG0000755	Cellular Component	extracellular region(GO:0005576)	1
OG0000755	Cellular Component	organelle part(GO:0044422)	1
OG0000755	Cellular Component	organelle(GO:0043226)	1
OG0000755	Molecular Function	antioxidant activity(GO:0016209)	1
OG0000755	Molecular Function	binding(GO:0005488)	1
OG0000755	Molecular Function	catalytic activity(GO:0003824)	1
OG0000808	Cellular Component	cell part(GO:0044464)	2
OG0000808	Cellular Component	cell(GO:0005623)	2
OG0000808	Cellular Component	membrane(GO:0016020)	2
OG0000838	Biological Process	metabolic process(GO:0008152)	1
OG0000838	Biological Process	response to stimulus(GO:0050896)	1
OG0000838	Cellular Component	cell part(GO:0044464)	1
OG0000838	Cellular Component	cell(GO:0005623)	1
OG0000838	Cellular Component	membrane part(GO:0044425)	1
OG0000838	Cellular Component	membrane(GO:0016020)	1
OG0000838	Cellular Component	organelle part(GO:0044422)	1
OG0000838	Cellular Component	organelle(GO:0043226)	1
OG0000838	Molecular Function	catalytic activity(GO:0003824)	1

OG0000866	Biological Process	cellular process(GO:0009987)	1
OG0000866	Biological Process	developmental	1
OG0000866	Biological Process	metabolic process(GO:0008152)	1
OG0000866	Biological Process	multi-organism	1
OG0000866	Biological Process	multicellular organismal	1
OG0000866	Biological Process	response to stimulus(GO:0050896)	1
OG0000866	Cellular Component	cell part(GO:0044464)	1
OG0000866	Cellular Component	cell(GO:0005623)	1
OG0000866	Cellular Component	organelle(GO:0043226)	1
OG0000866	Molecular Function	catalytic activity(GO:0003824)	1
OG0000871	Biological Process	cellular process(GO:0009987)	1
OG0000871	Biological Process	metabolic process(GO:0008152)	1
OG0000871	Cellular Component	cell part(GO:0044464)	1
OG0000871	Cellular Component	cell(GO:0005623)	1
OG0000871	Cellular Component	extracellular region(GO:0005576)	1
OG0000871	Molecular Function	catalytic activity(GO:0003824)	1
OG0000877	Cellular Component	cell part(GO:0044464)	1
OG0000877	Cellular Component	cell(GO:0005623)	1
OG0000877	Cellular Component	organelle(GO:0043226)	1
OG0000878	Cellular Component	cell part(GO:0044464)	1
OG0000878	Cellular Component	cell(GO:0005623)	1
OG0000881	Cellular Component	membrane(GO:0016020)	2
OG0000896	Cellular Component	cell part(GO:0044464)	1
OG0000896	Cellular Component	cell(GO:0005623)	1
OG0000896	Cellular Component	membrane(GO:0016020)	1
OG0000897	Cellular Component	cell part(GO:0044464)	1
OG0000897	Cellular Component	cell(GO:0005623)	1
OG0000897	Cellular Component	organelle(GO:0043226)	1
OG0000926	Biological Process	metabolic process(GO:0008152)	2
OG0000926	Biological Process	response to stimulus(GO:0050896)	2
OG0000926	Cellular Component	cell part(GO:0044464)	2
OG0000926	Cellular Component	cell(GO:0005623)	2
OG0000926	Cellular Component	extracellular region(GO:0005576)	1
OG0000926	Cellular Component	membrane(GO:0016020)	1

OG0000926	Cellular Component	organelle(GO:0043226)	2
OG0000926	Molecular Function	antioxidant activity(GO:0016209)	2
OG0000926	Molecular Function	catalytic activity(GO:0003824)	2
OG0000933	Biological Process	biological	1
OG0000933	Biological Process	cellular process(GO:0009987)	1
OG0000933	Biological Process	immune system	1
OG0000933	Biological Process	metabolic process(GO:0008152)	1
OG0000933	Biological Process	multi-organism	1
OG0000933	Biological Process	regulation of biological	1
OG0000933	Biological Process	response to stimulus(GO:0050896)	1
OG0000933	Biological Process	signaling(GO:0023052)	1
OG0000933	Cellular Component	cell part(GO:0044464)	1
OG0000933	Cellular Component	cell(GO:0005623)	1
OG0000933	Cellular Component	organelle part(GO:0044422)	1
OG0000933	Cellular Component	organelle(GO:0043226)	1
OG0000933	Molecular Function	catalytic activity(GO:0003824)	1
OG0000948	Biological Process	cellular component organization	1
OG0000948	Biological Process	cellular process(GO:0009987)	1
OG0000948	Biological Process	metabolic process(GO:0008152)	1
OG0000948	Biological Process	response to stimulus(GO:0050896)	1
OG0000948	Cellular Component	cell part(GO:0044464)	1
OG0000948	Cellular Component	cell(GO:0005623)	1
OG0000948	Cellular Component	extracellular region(GO:0005576)	1
OG0000948	Molecular Function	catalytic activity(GO:0003824)	1
OG0000953	Cellular Component	cell junction(GO:0030054)	1
OG0000953	Cellular Component	cell part(GO:0044464)	1
OG0000953	Cellular Component	cell(GO:0005623)	1
OG0000953	Cellular Component	membrane(GO:0016020)	1
OG0000953	Cellular Component	membrane-enclosed	1
OG0000953	Cellular Component	organelle part(GO:0044422)	1
OG0000953	Cellular Component	organelle(GO:0043226)	1
OG0000953	Cellular Component	protein-containing	1
OG0000953	Cellular Component	symplast(GO:0055044)	1
OG0000960	Biological Process	metabolic process(GO:0008152)	1

OG0000960	Cellular Component	cell part(GO:0044464)	2
OG0000960	Cellular Component	cell(GO:0005623)	2
OG0000960	Cellular Component	membrane(GO:0016020)	2
OG0000960	Cellular Component	organelle part(GO:0044422)	2
OG0000960	Cellular Component	organelle(GO:0043226)	2
OG0000960	Molecular Function	catalytic activity(GO:0003824)	1
OG0000972	Cellular Component	cell part(GO:0044464)	1
OG0000972	Cellular Component	cell(GO:0005623)	1
OG0000972	Cellular Component	membrane(GO:0016020)	1
OG0001007	Cellular Component	cell part(GO:0044464)	1
OG0001007	Cellular Component	cell(GO:0005623)	1
OG0001007	Cellular Component	organelle(GO:0043226)	1
OG0001014	Biological Process	cellular process(GO:0009987)	1
OG0001014	Biological Process	metabolic process(GO:0008152)	1
OG0001014	Biological Process	response to stimulus(GO:0050896)	1
OG0001014	Cellular Component	cell junction(GO:0030054)	1
OG0001014	Cellular Component	cell part(GO:0044464)	1
OG0001014	Cellular Component	cell(GO:0005623)	1
OG0001014	Cellular Component	extracellular region(GO:0005576)	1
OG0001014	Cellular Component	membrane(GO:0016020)	1
OG0001014	Cellular Component	membrane-enclosed	1
OG0001014	Cellular Component	organelle part(GO:0044422)	1
OG0001014	Cellular Component	organelle(GO:0043226)	1
OG0001014	Cellular Component	symplast(GO:0055044)	1
OG0001014	Molecular Function	binding(GO:0005488)	1
OG0001014	Molecular Function	catalytic activity(GO:0003824)	1
OG0001020	Cellular Component	cell part(GO:0044464)	1
OG0001020	Cellular Component	cell(GO:0005623)	1
OG0001020	Cellular Component	membrane part(GO:0044425)	1
OG0001020	Cellular Component	membrane(GO:0016020)	1
OG0001025	Biological Process	cellular process(GO:0009987)	1
OG0001025	Biological Process	developmental	1
OG0001025	Biological Process	metabolic process(GO:0008152)	1
OG0001025	Biological Process	multicellular organismal	1

OG0001025	Cellular Component	cell junction(GO:0030054)	1
OG0001025	Cellular Component	cell part(GO:0044464)	1
OG0001025	Cellular Component	cell(GO:0005623)	1
OG0001025	Cellular Component	membrane(GO:0016020)	1
OG0001025	Cellular Component	organelle(GO:0043226)	1
OG0001025	Cellular Component	protein-containing	1
OG0001025	Cellular Component	symplast(GO:0055044)	1
OG0001025	Molecular Function	binding(GO:0005488)	1
OG0001025	Molecular Function	catalytic activity(GO:0003824)	1
OG0001032	Cellular Component	extracellular region(GO:0005576)	2
OG0001058	Biological Process	biological	2
OG0001058	Biological Process	cellular process(GO:0009987)	2
OG0001058	Biological Process	developmental	2
OG0001058	Biological Process	metabolic process(GO:0008152)	2
OG0001058	Biological Process	multicellular organismal	2
OG0001058	Biological Process	positive regulation of	2
OG0001058	Biological Process	regulation of biological	2
OG0001058	Biological Process	response to stimulus(GO:0050896)	2
OG0001058	Biological Process	signaling(GO:0023052)	2
OG0001058	Cellular Component	cell junction(GO:0030054)	2
OG0001058	Cellular Component	cell part(GO:0044464)	2
OG0001058	Cellular Component	cell(GO:0005623)	2
OG0001058	Cellular Component	membrane(GO:0016020)	2
OG0001058	Cellular Component	organelle part(GO:0044422)	2
OG0001058	Cellular Component	organelle(GO:0043226)	2
OG0001058	Cellular Component	symplast(GO:0055044)	2
OG0001058	Molecular Function	binding(GO:0005488)	2
OG0001058	Molecular Function	catalytic activity(GO:0003824)	2
OG0001062	Biological Process	biological	1
OG0001062	Biological Process	cellular process(GO:0009987)	1
OG0001062	Biological Process	developmental	1
OG0001062	Biological Process	immune system	1
OG0001062	Biological Process	localization(GO:0051179)	1
OG0001062	Biological Process	metabolic process(GO:0008152)	1

OG0001062	Biological Process	multi-organism	1
OG0001062	Biological Process	multicellular organismal	1
OG0001062	Biological Process	response to stimulus(GO:0050896)	1
OG0001062	Cellular Component	cell part(GO:0044464)	1
OG0001062	Cellular Component	cell(GO:0005623)	1
OG0001062	Cellular Component	membrane part(GO:0044425)	1
OG0001062	Cellular Component	membrane(GO:0016020)	1
OG0001062	Cellular Component	organelle part(GO:0044422)	1
OG0001062	Cellular Component	organelle(GO:0043226)	1
OG0001062	Cellular Component	protein-containing	1
OG0001062	Molecular Function	binding(GO:0005488)	1
OG0001091	Biological Process	biological	1
OG0001091	Biological Process	cellular component organization	1
OG0001091	Biological Process	cellular process(GO:0009987)	1
OG0001091	Biological Process	developmental	1
OG0001091	Biological Process	localization(GO:0051179)	1
OG0001091	Biological Process	metabolic process(GO:0008152)	1
OG0001091	Biological Process	multicellular organismal	1
OG0001091	Biological Process	positive regulation of	1
OG0001091	Biological Process	regulation of biological	1
OG0001091	Biological Process	reproduction(GO:0000003)	1
OG0001091	Biological Process	reproductive process(GO:0022414)	1
OG0001091	Biological Process	response to stimulus(GO:0050896)	1
OG0001091	Cellular Component	cell part(GO:0044464)	1
OG0001091	Cellular Component	cell(GO:0005623)	1
OG0001091	Molecular Function	binding(GO:0005488)	1
OG0001091	Molecular Function	catalytic activity(GO:0003824)	1
OG0001120	Biological Process	cellular process(GO:0009987)	2
OG0001120	Biological Process	metabolic process(GO:0008152)	2
OG0001120	Biological Process	multi-organism	2
OG0001120	Biological Process	response to stimulus(GO:0050896)	2
OG0001120	Molecular Function	catalytic activity(GO:0003824)	2
OG0001124	Biological Process	biological	1
OG0001124	Biological Process	cellular process(GO:0009987)	1

OG0001124	Biological Process	metabolic process(GO:0008152)	1
OG0001124	Biological Process	multicellular organismal	1
OG0001124	Biological Process	regulation of biological	1
OG0001124	Biological Process	response to stimulus(GO:0050896)	1
OG0001124	Cellular Component	cell part(GO:0044464)	1
OG0001124	Cellular Component	cell(GO:0005623)	1
OG0001124	Cellular Component	organelle(GO:0043226)	1
OG0001124	Molecular Function	binding(GO:0005488)	1
OG0001124	Molecular Function	transcription regulator	1
OG0001129	Biological Process	cellular process(GO:0009987)	1
OG0001129	Biological Process	metabolic process(GO:0008152)	1
OG0001129	Cellular Component	cell part(GO:0044464)	1
OG0001129	Cellular Component	cell(GO:0005623)	1
OG0001129	Molecular Function	catalytic activity(GO:0003824)	1
OG0001156	Biological Process	immune system	2
OG0001156	Biological Process	multi-organism	2
OG0001156	Biological Process	response to stimulus(GO:0050896)	2
OG0001156	Cellular Component	cell part(GO:0044464)	2
OG0001156	Cellular Component	cell(GO:0005623)	2
OG0001158	Biological Process	biological	1
OG0001158	Biological Process	cellular process(GO:0009987)	1
OG0001158	Biological Process	metabolic process(GO:0008152)	1
OG0001158	Biological Process	negative regulation of	1
OG0001158	Biological Process	positive regulation of	1
OG0001158	Biological Process	regulation of biological	1
OG0001158	Biological Process	response to stimulus(GO:0050896)	1
OG0001158	Biological Process	signaling(GO:0023052)	1
OG0001158	Cellular Component	cell part(GO:0044464)	1
OG0001158	Cellular Component	cell(GO:0005623)	1
OG0001158	Cellular Component	organelle(GO:0043226)	1
OG0001158	Molecular Function	binding(GO:0005488)	1
OG0001158	Molecular Function	transcription regulator	1
OG0001182	Biological Process	cellular process(GO:0009987)	1
OG0001182	Biological Process	metabolic process(GO:0008152)	1

OG0001182	Biological Process	response to stimulus(GO:0050896)	1
OG0001182	Cellular Component	cell part(GO:0044464)	1
OG0001182	Cellular Component	cell(GO:0005623)	1
OG0001182	Cellular Component	membrane(GO:0016020)	1
OG0001182	Cellular Component	organelle part(GO:0044422)	1
OG0001182	Cellular Component	organelle(GO:0043226)	1
OG0001182	Molecular Function	catalytic activity(GO:0003824)	1
OG0001183	Biological Process	cellular process(GO:0009987)	1
OG0001183	Biological Process	metabolic process(GO:0008152)	1
OG0001183	Cellular Component	cell junction(GO:0030054)	1
OG0001183	Cellular Component	cell part(GO:0044464)	1
OG0001183	Cellular Component	cell(GO:0005623)	1
OG0001183	Cellular Component	membrane(GO:0016020)	1
OG0001183	Cellular Component	symplast(GO:0055044)	1
OG0001210	Cellular Component	cell part(GO:0044464)	1
OG0001210	Cellular Component	cell(GO:0005623)	1
OG0001210	Cellular Component	membrane(GO:0016020)	1
OG0001210	Cellular Component	organelle(GO:0043226)	1
OG0001215	Cellular Component	cell part(GO:0044464)	1
OG0001215	Cellular Component	cell(GO:0005623)	1
OG0001215	Cellular Component	membrane(GO:0016020)	1
OG0001241	Biological Process	multi-organism	1
OG0001241	Biological Process	response to stimulus(GO:0050896)	1
OG0001241	Cellular Component	cell part(GO:0044464)	1
OG0001241	Cellular Component	cell(GO:0005623)	1
OG0001259	Cellular Component	cell part(GO:0044464)	1
OG0001259	Cellular Component	cell(GO:0005623)	1
OG0001259	Cellular Component	membrane part(GO:0044425)	1
OG0001259	Cellular Component	membrane(GO:0016020)	1
OG0001259	Cellular Component	membrane-enclosed	1
OG0001259	Cellular Component	organelle part(GO:0044422)	1
OG0001259	Cellular Component	organelle(GO:0043226)	1
OG0001259	Cellular Component	protein-containing	1
OG0001273	Cellular Component	cell part(GO:0044464)	1

OG0001273	Cellular Component	cell(GO:0005623)	1
OG0001273	Cellular Component	organelle(GO:0043226)	1
OG0001273	Cellular Component	protein-containing	1
OG0001280	Biological Process	cellular process(GO:0009987)	1
OG0001280	Biological Process	metabolic process(GO:0008152)	1
OG0001280	Molecular Function	catalytic activity(GO:0003824)	1
OG0001282	Biological Process	biological	2
OG0001282	Biological Process	cellular process(GO:0009987)	2
OG0001282	Biological Process	localization(GO:0051179)	2
OG0001282	Biological Process	metabolic process(GO:0008152)	2
OG0001282	Biological Process	regulation of biological	2
OG0001282	Biological Process	response to stimulus(GO:0050896)	2
OG0001282	Biological Process	signaling(GO:0023052)	2
OG0001282	Cellular Component	cell part(GO:0044464)	2
OG0001282	Cellular Component	cell(GO:0005623)	2
OG0001282	Cellular Component	membrane part(GO:0044425)	2
OG0001282	Cellular Component	membrane(GO:0016020)	2
OG0001282	Molecular Function	catalytic activity(GO:0003824)	2
OG0001282	Molecular Function	transporter activity(GO:0005215)	2
OG0001287	Biological Process	biological	1
OG0001287	Biological Process	cellular process(GO:0009987)	1
OG0001287	Biological Process	developmental	1
OG0001287	Biological Process	immune system	1
OG0001287	Biological Process	metabolic process(GO:0008152)	1
OG0001287	Biological Process	multi-organism	1
OG0001287	Biological Process	multicellular organismal	1
OG0001287	Biological Process	positive regulation of	1
OG0001287	Biological Process	regulation of biological	1
OG0001287	Biological Process	response to stimulus(GO:0050896)	1
OG0001287	Cellular Component	cell part(GO:0044464)	1
OG0001287	Cellular Component	cell(GO:0005623)	1
OG0001287	Cellular Component	organelle(GO:0043226)	1
OG0001287	Molecular Function	binding(GO:0005488)	1
OG0001287	Molecular Function	transcription regulator	1

OG0001311	Cellular Component	cell part(GO:0044464)	1
OG0001311	Cellular Component	cell(GO:0005623)	1
OG0001311	Cellular Component	membrane(GO:0016020)	1
OG0001332	Biological Process	biological	1
OG0001332	Biological Process	cellular process(GO:0009987)	1
OG0001332	Biological Process	metabolic process(GO:0008152)	1
OG0001332	Biological Process	regulation of biological	1
OG0001332	Cellular Component	cell part(GO:0044464)	1
OG0001332	Cellular Component	cell(GO:0005623)	1
OG0001332	Cellular Component	organelle(GO:0043226)	1
OG0001332	Molecular Function	binding(GO:0005488)	1
OG0001332	Molecular Function	transcription regulator	1
OG0001367	Cellular Component	cell part(GO:0044464)	1
OG0001367	Cellular Component	cell(GO:0005623)	1
OG0001367	Cellular Component	membrane(GO:0016020)	1
OG0001399	Biological Process	response to stimulus(GO:0050896)	1
OG0001399	Cellular Component	cell junction(GO:0030054)	1
OG0001399	Cellular Component	cell part(GO:0044464)	1
OG0001399	Cellular Component	cell(GO:0005623)	1
OG0001399	Cellular Component	extracellular region(GO:0005576)	1
OG0001399	Cellular Component	membrane(GO:0016020)	1
OG0001399	Cellular Component	organelle part(GO:0044422)	1
OG0001399	Cellular Component	organelle(GO:0043226)	1
OG0001399	Cellular Component	symplast(GO:0055044)	1
OG0001437	Biological Process	biological	1
OG0001437	Biological Process	cellular process(GO:0009987)	1
OG0001437	Biological Process	developmental	1
OG0001437	Biological Process	metabolic process(GO:0008152)	1
OG0001437	Biological Process	multicellular organismal	1
OG0001437	Biological Process	positive regulation of	1
OG0001437	Biological Process	regulation of biological	1
OG0001437	Biological Process	response to stimulus(GO:0050896)	1
OG0001437	Biological Process	signaling(GO:0023052)	1
OG0001437	Cellular Component	cell part(GO:0044464)	1

OG0001437	Cellular Component	cell(GO:0005623)	1
OG0001437	Cellular Component	organelle(GO:0043226)	1
OG0001437	Molecular Function	catalytic activity(GO:0003824)	1
OG0001490	Biological Process	biological	1
OG0001490	Biological Process	cellular process(GO:0009987)	1
OG0001490	Biological Process	developmental	1
OG0001490	Biological Process	multi-organism	1
OG0001490	Biological Process	multicellular organismal	1
OG0001490	Biological Process	regulation of biological	1
OG0001490	Biological Process	reproduction(GO:0000003)	1
OG0001490	Biological Process	reproductive process(GO:0022414)	1
OG0001490	Biological Process	response to stimulus(GO:0050896)	1
OG0001490	Biological Process	signaling(GO:0023052)	1
OG0001490	Cellular Component	cell part(GO:0044464)	1
OG0001490	Cellular Component	cell(GO:0005623)	1
OG0001490	Cellular Component	organelle(GO:0043226)	1
OG0001583	Biological Process	response to stimulus(GO:0050896)	1
OG0001583	Cellular Component	cell part(GO:0044464)	1
OG0001583	Cellular Component	cell(GO:0005623)	1
OG0001583	Cellular Component	membrane(GO:0016020)	1
OG0001583	Cellular Component	organelle part(GO:0044422)	1
OG0001583	Cellular Component	organelle(GO:0043226)	1
OG0001583	Cellular Component	protein-containing	1
OG0001590	Biological Process	cellular component organization	1
OG0001590	Biological Process	cellular process(GO:0009987)	1
OG0001590	Biological Process	response to stimulus(GO:0050896)	1
OG0001590	Cellular Component	cell part(GO:0044464)	1
OG0001590	Cellular Component	cell(GO:0005623)	1
OG0001590	Cellular Component	membrane(GO:0016020)	1
OG0001590	Cellular Component	membrane-enclosed	1
OG0001590	Cellular Component	organelle part(GO:0044422)	1
OG0001590	Cellular Component	organelle(GO:0043226)	1
OG0001590	Cellular Component	protein-containing	1
OG0001590	Molecular Function	binding(GO:0005488)	1

OG0001605	Cellular Component	cell junction(GO:0030054)	1
OG0001605	Cellular Component	cell part(GO:0044464)	1
OG0001605	Cellular Component	cell(GO:0005623)	1
OG0001605	Cellular Component	membrane(GO:0016020)	1
OG0001605	Cellular Component	organelle part(GO:0044422)	1
OG0001605	Cellular Component	organelle(GO:0043226)	1
OG0001605	Cellular Component	symplast(GO:0055044)	1
OG0001629	Biological Process	cellular process(GO:0009987)	1
OG0001629	Biological Process	metabolic process(GO:0008152)	1
OG0001629	Cellular Component	cell part(GO:0044464)	1
OG0001629	Cellular Component	cell(GO:0005623)	1
OG0001629	Cellular Component	organelle part(GO:0044422)	1
OG0001629	Cellular Component	organelle(GO:0043226)	1
OG0001629	Cellular Component	protein-containing	1
OG0001630	Biological Process	cellular component organization	1
OG0001630	Biological Process	cellular process(GO:0009987)	1
OG0001630	Biological Process	metabolic process(GO:0008152)	1
OG0001630	Cellular Component	cell part(GO:0044464)	1
OG0001630	Cellular Component	cell(GO:0005623)	1
OG0001630	Cellular Component	membrane(GO:0016020)	1
OG0001630	Cellular Component	organelle part(GO:0044422)	1
OG0001630	Cellular Component	organelle(GO:0043226)	1
OG0001654	Biological Process	biological	1
OG0001654	Biological Process	developmental	1
OG0001654	Biological Process	multicellular organismal	1
OG0001654	Biological Process	negative regulation of	1
OG0001654	Biological Process	regulation of biological	1
OG0001654	Biological Process	reproduction(GO:0000003)	1
OG0001654	Biological Process	reproductive process(GO:0022414)	1
OG0001654	Biological Process	response to stimulus(GO:0050896)	1
OG0001654	Cellular Component	cell part(GO:0044464)	1
OG0001654	Cellular Component	cell(GO:0005623)	1
OG0001654	Cellular Component	organelle(GO:0043226)	1
OG0001654	Molecular Function	binding(GO:0005488)	1

OG0001711	Cellular Component	membrane part(GO:0044425)	1
OG0001711	Cellular Component	membrane(GO:0016020)	1
OG0001721	Biological Process	biological	1
OG0001721	Biological Process	cellular component organization	1
OG0001721	Biological Process	cellular process(GO:0009987)	1
OG0001721	Biological Process	developmental	1
OG0001721	Biological Process	growth(GO:0040007)	1
OG0001721	Biological Process	localization(GO:0051179)	1
OG0001721	Biological Process	metabolic process(GO:0008152)	1
OG0001721	Biological Process	multi-organism	1
OG0001721	Biological Process	multicellular organismal	1
OG0001721	Biological Process	negative regulation of	1
OG0001721	Biological Process	regulation of biological	1
OG0001721	Biological Process	reproduction(GO:0000003)	1
OG0001721	Biological Process	reproductive process(GO:0022414)	1
OG0001721	Cellular Component	cell part(GO:0044464)	1
OG0001721	Cellular Component	cell(GO:0005623)	1
OG0001721	Cellular Component	membrane-enclosed	1
OG0001721	Cellular Component	organelle part(GO:0044422)	1
OG0001721	Cellular Component	organelle(GO:0043226)	1
OG0001721	Cellular Component	protein-containing	1
OG0001721	Molecular Function	structural molecule	1
OG0001721	Molecular Function	translation regulator	1
OG0001752	Biological Process	cellular component organization	1
OG0001752	Biological Process	cellular process(GO:0009987)	1
OG0001752	Biological Process	localization(GO:0051179)	1
OG0001752	Biological Process	response to stimulus(GO:0050896)	1
OG0001752	Cellular Component	cell part(GO:0044464)	1
OG0001752	Cellular Component	cell(GO:0005623)	1
OG0001752	Cellular Component	organelle part(GO:0044422)	1
OG0001752	Cellular Component	organelle(GO:0043226)	1
OG0001752	Molecular Function	transporter activity(GO:0005215)	1
OG0001804	Biological Process	metabolic process(GO:0008152)	1
OG0001804	Biological Process	response to stimulus(GO:0050896)	1

OG0001804	Molecular Function	catalytic activity(GO:0003824)	1
OG0001814	Cellular Component	cell part(GO:0044464)	1
OG0001814	Cellular Component	cell(GO:0005623)	1
OG0001814	Cellular Component	organelle(GO:0043226)	1
OG0001829	Cellular Component	cell part(GO:0044464)	1
OG0001829	Cellular Component	cell(GO:0005623)	1
OG0001829	Cellular Component	organelle(GO:0043226)	1
OG0001835	Biological Process	cellular process(GO:0009987)	1
OG0001835	Biological Process	localization(GO:0051179)	1
OG0001835	Cellular Component	cell part(GO:0044464)	1
OG0001835	Cellular Component	cell(GO:0005623)	1
OG0001835	Cellular Component	membrane(GO:0016020)	1
OG0001835	Molecular Function	transporter activity(GO:0005215)	1
OG0001838	Cellular Component	cell junction(GO:0030054)	1
OG0001838	Cellular Component	cell part(GO:0044464)	1
OG0001838	Cellular Component	cell(GO:0005623)	1
OG0001838	Cellular Component	membrane(GO:0016020)	1
OG0001838	Cellular Component	membrane-enclosed	1
OG0001838	Cellular Component	organelle part(GO:0044422)	1
OG0001838	Cellular Component	organelle(GO:0043226)	1
OG0001838	Cellular Component	protein-containing	1
OG0001838	Cellular Component	symplast(GO:0055044)	1
OG0001873	Cellular Component	cell part(GO:0044464)	1
OG0001873	Cellular Component	cell(GO:0005623)	1
OG0001873	Cellular Component	organelle(GO:0043226)	1
OG0001880	Cellular Component	cell part(GO:0044464)	1
OG0001880	Cellular Component	cell(GO:0005623)	1
OG0001880	Cellular Component	extracellular region(GO:0005576)	1
OG0001880	Cellular Component	organelle(GO:0043226)	1
OG0001927	Biological Process	cellular component organization	1
OG0001927	Biological Process	cellular process(GO:0009987)	1
OG0001927	Biological Process	metabolic process(GO:0008152)	1
OG0001927	Cellular Component	cell part(GO:0044464)	1
OG0001927	Cellular Component	cell(GO:0005623)	1

OG0001927	Cellular Component	membrane part(GO:0044425)	1
OG0001927	Cellular Component	membrane(GO:0016020)	1
OG0001927	Cellular Component	organelle part(GO:0044422)	1
OG0001927	Cellular Component	organelle(GO:0043226)	1
OG0001927	Cellular Component	protein-containing	1
OG0001927	Molecular Function	catalytic activity(GO:0003824)	1
OG0001946	Biological Process	biological	1
OG0001946	Biological Process	cellular process(GO:0009987)	1
OG0001946	Biological Process	metabolic process(GO:0008152)	1
OG0001946	Biological Process	positive regulation of	1
OG0001946	Biological Process	regulation of biological	1
OG0001946	Biological Process	response to stimulus(GO:0050896)	1
OG0001946	Cellular Component	cell part(GO:0044464)	1
OG0001946	Cellular Component	cell(GO:0005623)	1
OG0001946	Cellular Component	organelle(GO:0043226)	1
OG0001946	Molecular Function	catalytic activity(GO:0003824)	1
OG0001983	Biological Process	biological	1
OG0001983	Biological Process	cellular process(GO:0009987)	1
OG0001983	Biological Process	developmental	1
OG0001983	Biological Process	growth(GO:0040007)	1
OG0001983	Biological Process	metabolic process(GO:0008152)	1
OG0001983	Biological Process	multicellular organismal	1
OG0001983	Biological Process	negative regulation of	1
OG0001983	Biological Process	regulation of biological	1
OG0001983	Biological Process	reproduction(GO:0000003)	1
OG0001983	Biological Process	reproductive process(GO:0022414)	1
OG0001983	Molecular Function	catalytic activity(GO:0003824)	1
OG0001986	Cellular Component	cell junction(GO:0030054)	1
OG0001986	Cellular Component	cell part(GO:0044464)	1
OG0001986	Cellular Component	cell(GO:0005623)	1
OG0001986	Cellular Component	membrane(GO:0016020)	1
OG0001986	Cellular Component	organelle part(GO:0044422)	1
OG0001986	Cellular Component	organelle(GO:0043226)	1
OG0001986	Cellular Component	protein-containing	1

OG0001986	Cellular Component	symplast(GO:0055044)	1
OG0002003	Cellular Component	cell part(GO:0044464)	1
OG0002003	Cellular Component	cell(GO:0005623)	1
OG0002003	Cellular Component	organelle(GO:0043226)	1
OG0002019	Biological Process	cellular process(GO:0009987)	1
OG0002019	Biological Process	localization(GO:0051179)	1
OG0002019	Biological Process	metabolic process(GO:0008152)	1
OG0002019	Cellular Component	cell part(GO:0044464)	1
OG0002019	Cellular Component	cell(GO:0005623)	1
OG0002019	Cellular Component	membrane part(GO:0044425)	1
OG0002019	Cellular Component	membrane(GO:0016020)	1
OG0002019	Cellular Component	organelle part(GO:0044422)	1
OG0002019	Cellular Component	organelle(GO:0043226)	1
OG0002019	Molecular Function	binding(GO:0005488)	1
OG0002019	Molecular Function	catalytic activity(GO:0003824)	1
OG0002019	Molecular Function	transporter activity(GO:0005215)	1
OG0002027	Cellular Component	cell part(GO:0044464)	1
OG0002027	Cellular Component	cell(GO:0005623)	1
OG0002027	Cellular Component	membrane(GO:0016020)	1
OG0002040	Cellular Component	cell part(GO:0044464)	1
OG0002040	Cellular Component	cell(GO:0005623)	1
OG0002040	Cellular Component	membrane(GO:0016020)	1
OG0002040	Cellular Component	organelle part(GO:0044422)	1
OG0002040	Cellular Component	organelle(GO:0043226)	1
OG0002134	Cellular Component	cell junction(GO:0030054)	1
OG0002134	Cellular Component	cell part(GO:0044464)	1
OG0002134	Cellular Component	cell(GO:0005623)	1
OG0002134	Cellular Component	membrane(GO:0016020)	1
OG0002134	Cellular Component	organelle part(GO:0044422)	1
OG0002134	Cellular Component	organelle(GO:0043226)	1
OG0002134	Cellular Component	protein-containing	1
OG0002134	Cellular Component	symplast(GO:0055044)	1
OG0002147	Biological Process	cellular process(GO:0009987)	1
OG0002147	Biological Process	metabolic process(GO:0008152)	1

OG0002147	Molecular Function	catalytic activity(GO:0003824)	1
OG0002161	Cellular Component	cell part(GO:0044464)	1
OG0002161	Cellular Component	cell(GO:0005623)	1
OG0002161	Cellular Component	organelle(GO:0043226)	1
OG0002204	Cellular Component	cell part(GO:0044464)	1
OG0002204	Cellular Component	cell(GO:0005623)	1
OG0002204	Cellular Component	membrane(GO:0016020)	1
OG0002204	Cellular Component	organelle part(GO:0044422)	1
OG0002204	Cellular Component	organelle(GO:0043226)	1
OG0002224	Cellular Component	membrane(GO:0016020)	1
OG0002225	Cellular Component	cell junction(GO:0030054)	1
OG0002225	Cellular Component	cell part(GO:0044464)	1
OG0002225	Cellular Component	cell(GO:0005623)	1
OG0002225	Cellular Component	membrane(GO:0016020)	1
OG0002225	Cellular Component	membrane-enclosed	1
OG0002225	Cellular Component	organelle part(GO:0044422)	1
OG0002225	Cellular Component	organelle(GO:0043226)	1
OG0002225	Cellular Component	protein-containing	1
OG0002225	Cellular Component	symplast(GO:0055044)	1
OG0002303	Biological Process	biological	1
OG0002303	Biological Process	cellular process(GO:0009987)	1
OG0002303	Biological Process	metabolic process(GO:0008152)	1
OG0002303	Biological Process	regulation of biological	1
OG0002303	Cellular Component	cell part(GO:0044464)	1
OG0002303	Cellular Component	cell(GO:0005623)	1
OG0002303	Cellular Component	membrane-enclosed	1
OG0002303	Cellular Component	organelle part(GO:0044422)	1
OG0002303	Cellular Component	organelle(GO:0043226)	1
OG0002303	Cellular Component	protein-containing	1
OG0002303	Molecular Function	transcription regulator	1
OG0002310	Biological Process	biological	1
OG0002310	Biological Process	cellular process(GO:0009987)	1
OG0002310	Biological Process	developmental	1
OG0002310	Biological Process	multicellular organismal	1

OG0002310	Biological Process	regulation of biological	1
OG0002310	Biological Process	reproduction(GO:0000003)	1
OG0002310	Biological Process	reproductive process(GO:0022414)	1
OG0002310	Biological Process	response to stimulus(GO:0050896)	1
OG0002310	Biological Process	signaling(GO:0023052)	1
OG0002310	Cellular Component	cell part(GO:0044464)	1
OG0002310	Cellular Component	cell(GO:0005623)	1
OG0002310	Cellular Component	membrane(GO:0016020)	1
OG0002310	Cellular Component	membrane-enclosed	1
OG0002310	Cellular Component	organelle part(GO:0044422)	1
OG0002310	Cellular Component	organelle(GO:0043226)	1
OG0002310	Cellular Component	protein-containing	1
OG0002337	Cellular Component	cell part(GO:0044464)	1
OG0002337	Cellular Component	cell(GO:0005623)	1
OG0002337	Cellular Component	membrane(GO:0016020)	1
OG0002337	Cellular Component	organelle part(GO:0044422)	1
OG0002337	Cellular Component	organelle(GO:0043226)	1
OG0002345	Cellular Component	cell junction(GO:0030054)	1
OG0002345	Cellular Component	cell part(GO:0044464)	1
OG0002345	Cellular Component	cell(GO:0005623)	1
OG0002345	Cellular Component	membrane part(GO:0044425)	1
OG0002345	Cellular Component	membrane(GO:0016020)	1
OG0002345	Cellular Component	symplast(GO:0055044)	1
OG0002348	Biological Process	biological	1
OG0002348	Biological Process	cellular process(GO:0009987)	1
OG0002348	Biological Process	developmental	1
OG0002348	Biological Process	metabolic process(GO:0008152)	1
OG0002348	Biological Process	multicellular organismal	1
OG0002348	Cellular Component	cell part(GO:0044464)	1
OG0002348	Cellular Component	cell(GO:0005623)	1
OG0002348	Cellular Component	extracellular region	1
OG0002348	Cellular Component	extracellular region(GO:0005576)	1
OG0002348	Cellular Component	organelle(GO:0043226)	1
OG0002348	Molecular Function	catalytic activity(GO:0003824)	1

OG0002355	Biological Process	cellular process(GO:0009987)	1
OG0002355	Biological Process	metabolic process(GO:0008152)	1
OG0002355	Molecular Function	catalytic activity(GO:0003824)	1
OG0002415	Biological Process	cellular process(GO:0009987)	1
OG0002415	Biological Process	localization(GO:0051179)	1
OG0002415	Biological Process	multicellular organismal	1
OG0002415	Biological Process	response to stimulus(GO:0050896)	1
OG0002415	Cellular Component	cell part(GO:0044464)	1
OG0002415	Cellular Component	cell(GO:0005623)	1
OG0002415	Cellular Component	membrane(GO:0016020)	1
OG0002415	Cellular Component	organelle part(GO:0044422)	1
OG0002415	Cellular Component	organelle(GO:0043226)	1
OG0002431	Biological Process	biological	1
OG0002431	Biological Process	cellular process(GO:0009987)	1
OG0002431	Biological Process	developmental	1
OG0002431	Biological Process	growth(GO:0040007)	1
OG0002431	Biological Process	metabolic process(GO:0008152)	1
OG0002431	Biological Process	multicellular organismal	1
OG0002431	Biological Process	regulation of biological	1
OG0002431	Biological Process	reproduction(GO:0000003)	1
OG0002431	Biological Process	reproductive process(GO:0022414)	1
OG0002431	Cellular Component	cell part(GO:0044464)	1
OG0002431	Cellular Component	cell(GO:0005623)	1
OG0002431	Cellular Component	organelle(GO:0043226)	1
OG0002431	Molecular Function	transcription regulator	1
OG0002452	Biological Process	response to stimulus(GO:0050896)	1
OG0002455	Biological Process	cellular component organization	1
OG0002455	Biological Process	cellular process(GO:0009987)	1
OG0002455	Biological Process	metabolic process(GO:0008152)	1
OG0002455	Cellular Component	cell part(GO:0044464)	1
OG0002455	Cellular Component	cell(GO:0005623)	1
OG0002455	Cellular Component	membrane(GO:0016020)	1
OG0002455	Cellular Component	organelle part(GO:0044422)	1
OG0002455	Cellular Component	organelle(GO:0043226)	1

OG0002455	Cellular Component	protein-containing	1
OG0002455	Molecular Function	structural molecule	1
OG0002464	Biological Process	response to stimulus(GO:0050896)	1
OG0002464	Cellular Component	membrane(GO:0016020)	1
OG0002538	Biological Process	cellular process(GO:0009987)	1
OG0002538	Biological Process	metabolic process(GO:0008152)	1
OG0002538	Molecular Function	catalytic activity(GO:0003824)	1
OG0002649	Biological Process	developmental	1
OG0002649	Biological Process	multi-organism	1
OG0002649	Biological Process	multicellular organismal	1
OG0002649	Biological Process	reproduction(GO:0000003)	1
OG0002649	Biological Process	reproductive process(GO:0022414)	1
OG0002741	Cellular Component	cell part(GO:0044464)	1
OG0002741	Cellular Component	cell(GO:0005623)	1
OG0002741	Cellular Component	organelle(GO:0043226)	1
OG0002741	Molecular Function	binding(GO:0005488)	1
OG0002956	Biological Process	response to stimulus(GO:0050896)	1
OG0002956	Cellular Component	cell part(GO:0044464)	1
OG0002956	Cellular Component	cell(GO:0005623)	1
OG0002956	Cellular Component	membrane-enclosed	1
OG0002956	Cellular Component	organelle part(GO:0044422)	1
OG0002956	Cellular Component	organelle(GO:0043226)	1
OG0002956	Molecular Function	binding(GO:0005488)	1
OG0002957	Cellular Component	cell part(GO:0044464)	1
OG0002957	Cellular Component	cell(GO:0005623)	1
OG0002957	Cellular Component	organelle(GO:0043226)	1
OG0002964	Biological Process	cellular process(GO:0009987)	1
OG0002964	Biological Process	metabolic process(GO:0008152)	1
OG0002964	Cellular Component	cell part(GO:0044464)	1
OG0002964	Cellular Component	cell(GO:0005623)	1
OG0002964	Cellular Component	organelle(GO:0043226)	1
OG0002964	Molecular Function	binding(GO:0005488)	1
OG0002984	Biological Process	biological	1
OG0002984	Biological Process	cellular component organization	1

OG0002984	Biological Process	cellular process(GO:0009987)	1
OG0002984	Biological Process	metabolic process(GO:0008152)	1
OG0002984	Biological Process	negative regulation of	1
OG0002984	Biological Process	positive regulation of	1
OG0002984	Biological Process	regulation of biological	1
OG0002984	Cellular Component	cell part(GO:0044464)	1
OG0002984	Cellular Component	cell(GO:0005623)	1
OG0002984	Cellular Component	organelle(GO:0043226)	1
OG0002984	Cellular Component	protein-containing	1
OG0002984	Molecular Function	binding(GO:0005488)	1
OG0003027	Cellular Component	cell part(GO:0044464)	1
OG0003027	Cellular Component	cell(GO:0005623)	1
OG0003027	Cellular Component	membrane-enclosed	1
OG0003027	Cellular Component	organelle part(GO:0044422)	1
OG0003027	Cellular Component	organelle(GO:0043226)	1
OG0003151	Biological Process	response to stimulus(GO:0050896)	1
OG0003223	Cellular Component	cell part(GO:0044464)	1
OG0003223	Cellular Component	cell(GO:0005623)	1
OG0003223	Cellular Component	organelle(GO:0043226)	1
OG0003257	Cellular Component	extracellular region(GO:0005576)	1
OG0003389	Cellular Component	cell part(GO:0044464)	1
OG0003389	Cellular Component	cell(GO:0005623)	1
OG0003389	Cellular Component	organelle(GO:0043226)	1
OG0003472	Biological Process	biological	1
OG0003472	Biological Process	localization(GO:0051179)	1
OG0003472	Biological Process	regulation of biological	1
OG0003472	Cellular Component	cell junction(GO:0030054)	1
OG0003472	Cellular Component	cell part(GO:0044464)	1
OG0003472	Cellular Component	cell(GO:0005623)	1
OG0003472	Cellular Component	membrane part(GO:0044425)	1
OG0003472	Cellular Component	membrane(GO:0016020)	1
OG0003472	Cellular Component	organelle part(GO:0044422)	1
OG0003472	Cellular Component	organelle(GO:0043226)	1
OG0003472	Cellular Component	symplast(GO:0055044)	1

OG0003503	Biological Process	cellular process(GO:0009987)	1
OG0003503	Biological Process	metabolic process(GO:0008152)	1
OG0003503	Biological Process	response to stimulus(GO:0050896)	1
OG0003503	Cellular Component	cell part(GO:0044464)	1
OG0003503	Cellular Component	cell(GO:0005623)	1
OG0003503	Cellular Component	membrane(GO:0016020)	1
OG0003503	Cellular Component	organelle part(GO:0044422)	1
OG0003503	Cellular Component	organelle(GO:0043226)	1
OG0003503	Molecular Function	catalytic activity(GO:0003824)	1
OG0003549	Cellular Component	cell part(GO:0044464)	1
OG0003549	Cellular Component	cell(GO:0005623)	1
OG0003549	Cellular Component	membrane(GO:0016020)	1
OG0003549	Cellular Component	organelle part(GO:0044422)	1
OG0003549	Cellular Component	organelle(GO:0043226)	1
OG0003569	Biological Process	cellular process(GO:0009987)	1
OG0003569	Biological Process	developmental	1
OG0003569	Biological Process	localization(GO:0051179)	1
OG0003569	Biological Process	multi-organism	1
OG0003569	Biological Process	multicellular organismal	1
OG0003569	Biological Process	reproduction(GO:0000003)	1
OG0003569	Biological Process	reproductive process(GO:0022414)	1
OG0003569	Biological Process	response to stimulus(GO:0050896)	1
OG0003569	Cellular Component	cell part(GO:0044464)	1
OG0003569	Cellular Component	cell(GO:0005623)	1
OG0003569	Cellular Component	membrane(GO:0016020)	1
OG0003569	Molecular Function	transporter activity(GO:0005215)	1
OG0003776	Biological Process	biological	1
OG0003776	Biological Process	metabolic process(GO:0008152)	1
OG0003776	Biological Process	regulation of biological	1
OG0003776	Cellular Component	cell part(GO:0044464)	1
OG0003776	Cellular Component	cell(GO:0005623)	1
OG0003776	Cellular Component	extracellular region(GO:0005576)	1
OG0003776	Molecular Function	catalytic activity(GO:0003824)	1
OG0003776	Molecular Function	molecular function	1

OG0003784	Biological Process	cellular process(GO:0009987)	1
OG0003784	Biological Process	metabolic process(GO:0008152)	1
OG0003784	Biological Process	response to stimulus(GO:0050896)	1
OG0003784	Cellular Component	extracellular region	1
OG0003784	Cellular Component	extracellular region(GO:0005576)	1
OG0003784	Molecular Function	catalytic activity(GO:0003824)	1
OG0003888	Biological Process	metabolic process(GO:0008152)	1
OG0003888	Biological Process	response to stimulus(GO:0050896)	1
OG0003888	Cellular Component	cell junction(GO:0030054)	1
OG0003888	Cellular Component	cell part(GO:0044464)	1
OG0003888	Cellular Component	cell(GO:0005623)	1
OG0003888	Cellular Component	extracellular region(GO:0005576)	1
OG0003888	Cellular Component	membrane(GO:0016020)	1
OG0003888	Cellular Component	organelle part(GO:0044422)	1
OG0003888	Cellular Component	organelle(GO:0043226)	1
OG0003888	Cellular Component	symplast(GO:0055044)	1
OG0003888	Molecular Function	binding(GO:0005488)	1
OG0003888	Molecular Function	catalytic activity(GO:0003824)	1
OG0003927	Biological Process	developmental	1
OG0003927	Biological Process	multicellular organismal	1
OG0003927	Cellular Component	cell part(GO:0044464)	1
OG0003927	Cellular Component	cell(GO:0005623)	1
OG0003927	Cellular Component	membrane(GO:0016020)	1
OG0003927	Cellular Component	organelle(GO:0043226)	1
OG0004102	Biological Process	cellular component organization	1
OG0004102	Biological Process	cellular process(GO:0009987)	1
OG0004102	Biological Process	developmental	1
OG0004102	Biological Process	growth(GO:0040007)	1
OG0004102	Biological Process	multi-organism	1
OG0004102	Biological Process	multicellular organismal	1
OG0004102	Biological Process	reproduction(GO:0000003)	1
OG0004102	Biological Process	reproductive process(GO:0022414)	1
OG0004102	Cellular Component	cell part(GO:0044464)	1
OG0004102	Cellular Component	cell(GO:0005623)	1

OG0004102	Cellular Component	organelle(GO:0043226)	1
OG0004361	Cellular Component	cell part(GO:0044464)	1
OG0004361	Cellular Component	cell(GO:0005623)	1
OG0004361	Cellular Component	organelle(GO:0043226)	1
OG0004753	Biological Process	cellular process(GO:0009987)	1
OG0004753	Biological Process	developmental	1
OG0004753	Biological Process	metabolic process(GO:0008152)	1
OG0004753	Biological Process	multicellular organismal	1
OG0004753	Biological Process	response to stimulus(GO:0050896)	1
OG0004753	Cellular Component	cell part(GO:0044464)	1
OG0004753	Cellular Component	cell(GO:0005623)	1
OG0004753	Cellular Component	membrane(GO:0016020)	1
OG0004753	Cellular Component	organelle(GO:0043226)	1
OG0004753	Molecular Function	binding(GO:0005488)	1
OG0004753	Molecular Function	catalytic activity(GO:0003824)	1
OG0004917	Biological Process	cellular process(GO:0009987)	1
OG0004917	Biological Process	metabolic process(GO:0008152)	1
OG0004917	Biological Process	response to stimulus(GO:0050896)	1
OG0004917	Cellular Component	cell part(GO:0044464)	1
OG0004917	Cellular Component	cell(GO:0005623)	1
OG0004917	Cellular Component	membrane(GO:0016020)	1
