# Supplementary material for: A high-quality genome provides insights into the new taxonomic status and genomic characteristics of Cladopus chinensis (Podostemaceae)
Source: Hortic Res. 2020 Apr 1;7:46. doi: 10.1038/s41438-020-0269-5 (PMC7109043; doi:10.1038/s41438-020-0269-5)
Supplement: Supplementary file 11 — Table S13 expand_annotation [file 41438_2020_269_MOESM11_ESM.pdf]

Cladopus_000015	2.30E-83	313.9	EMB2394		J	50S ribosomal protein L6
Cladopus_000059	6.30E-48	195.3	RPS7		J	ribosomal protein S7
Cladopus_000060	3.30E-111	406.8	ND2		C	Core subunit of the mitochondrial membrane respiratory chain NADH
Cladopus_000061	6.70E-32	142.9	RPL2		J	50S ribosomal protein L2
Cladopus_000103	3.80E-26	122.9	OSI_00989		Q	cytochrome P450
Cladopus_000284	2.20E-144	517.3	OSI_31806		A	Metallo-beta-lactamase superfamily
Cladopus_000535	5.20E-12	77			O	regulation of cellular process
Cladopus_000555	4.60E-188	662.9	DDB2		L	damaged DNA-binding
Cladopus_000570	2.20E-24	118.2	MRPS12		J	Ribosomal protein s12
Cladopus_000574	1.20E-86	325.5	FG04596.1		D	o-methyltransferase
Cladopus_000622	9.40E-175	618.6	OSI_33884		G	Pectate lyase
Cladopus_000664	1.10E-264	917.5	URE		F	Urea amidohydrolase
Cladopus_000728	4.90E-21	105.1	RPL20		J	Binds directly to 23S ribosomal RNA and is necessary for the in vitro
Cladopus_000730	4.00E-91	339.7	ACCD		H	Component of the acetyl coenzyme A carboxylase (ACC) complex. Biotin
Cladopus_000851	7.50E-159	565.8			G	pectinesterase
Cladopus_000957	9.10E-38	161.8	PSAE		C	photosystem I reaction center subunit IV
Cladopus_000978	7.00E-102	375.9	GA2OX1		Q	gibberellin
Cladopus_001001	4.80E-103	379.8	GA2OX1		Q	gibberellin
Cladopus_001003	1.00E-97	362.1	GA2OX1		Q	gibberellin
Cladopus_001121	9.10E-14	83.2			G	cellulose 1,4-beta-cellobiosidase
Cladopus_001166	2.20E-200	703.4		LALDO	Q	dehydrogenase
Cladopus_001222	9.70E-35	152.1	RPS7		J	One of the primary rRNA binding proteins, it binds directly to 16S
Cladopus_001367	4.00E-165	586.6			G	organic cation
Cladopus_001427	7.70E-106	389	OSI_34838		G	peroxidase
Cladopus_001617	7.70E-264	914.8	HA1	ATPS	P	Plasma membrane
Cladopus_001637	7.40E-65	251.9	TPX1		O	thioredoxin-dependent peroxidase 1
Cladopus_001749	4.10E-245	852.8	PSBC		O	One of the components of the core antenna complex of photosystem II.
Cladopus_001873	3.80E-88	330.5	FG04596.1		D	o-methyltransferase
Cladopus_001956	1.20E-108	398.3	CHLI		H	magnesium chelatase
Cladopus_002037	0	1419.4	EMB2369		J	leucyl-tRNA
Cladopus_002110	4.40E-124	449.9			G	Converts alpha-aldose to the beta-anomer. It is active on D-glucose,
Cladopus_002169	6.50E-21	105.5			L	DNA polymerase
Cladopus_002175	2.50E-22	110.2			L	DNA polymerase
Cladopus_002274	6.80E-93	346.3	FG04596.1		D	o-methyltransferase

Cladopus_002316	1.50E-117	427.6	NDHB	C	NDH shuttles electrons from NAD(P)H plastoquinone, via FMN and iron-
Cladopus_002326	5.30E-17	92.8			
Cladopus_002395	2.80E-81	306.6	PETA	C	Component of the cytochrome b6-f complex, which mediates electron
Cladopus_002396	4.80E-37	158.7	PSBE	C	This b-type cytochrome is tightly associated with the reaction center
Cladopus_002441	1.20E-108	399.4	GA2OX1	Q	gibberellin
Cladopus_002536	2.40E-09	65.9	NDHE	C	NDH shuttles electrons from NAD(P)H plastoquinone, via FMN and iron-
Cladopus_002827	2.70E-211	740.3	NCED3	Q	9-cis-epoxy-carotenoid dioxygenase
Cladopus_002828	4.30E-217	759.6	NCED3	Q	9-cis-epoxy-carotenoid dioxygenase
Cladopus_002938	1.50E-15	86.7	RPS14	J	Binds 16S rRNA, required for the assembly of 30S particles (By
Cladopus_003014	0	1501.5	CLPB3	O	chaperone protein
Cladopus_003132	1.00E-227	794.7	GATA	J	Allows the formation of correctly charged Gln-tRNA(Gln) through the
Cladopus_003189	3.80E-61	239.6	TPX1	O	Reduces hydrogen peroxide and alkyl hydroperoxides with reducing
Cladopus_003196	3.80E-61	239.6	TPX1	O	Reduces hydrogen peroxide and alkyl hydroperoxides with reducing
Cladopus_003444	6.00E-81	305.4	NTRC	O	Thioredoxin reductase
Cladopus_003465	2.10E-165	587.4	OSI_33884	G	Pectate lyase
Cladopus_003548	7.60E-91	339		O	ATP-dependent Clp protease proteolytic subunit
Cladopus_003552	8.90E-112	408.3	COB	C	Component of the ubiquinol-cytochrome c reductase complex (complex III
Cladopus_003738	4.40E-232	809.7	NCED3	Q	9-cis-epoxy-carotenoid dioxygenase
Cladopus_003767	1.70E-88	331.6	FG04596.1	D	o-methyltransferase
Cladopus_003771	1.80E-51	207.2	ATPF	C	F(1)F(0) ATP synthase produces ATP from ADP in the presence of a
Cladopus_003814	2.20E-64	250.8		H	atp synthase
Cladopus_004206	2.20E-35	154.8		T	receptor-like protein kinase
Cladopus_004221	1.60E-15	89		T	receptor-like protein kinase
Cladopus_004350	1.80E-108	397.5	RBCL	C	RuBisCO catalyzes two reactions the carboxylation of D- ribulose 1,5-
Cladopus_004390	4.90E-211	739.2		G	organic cation
Cladopus_004391	4.90E-171	606.3		G	organic cation
Cladopus_004566	2.80E-147	527.3		H	Oxygen-independent coproporphyrinogen-III oxidase-like protein
Cladopus_004569	6.40E-156	555.8		G	Pectate lyase
Cladopus_004579	1.30E-127	461.1	LHCB1-1	O	Chlorophyll a-b binding protein
Cladopus_004687	6.40E-56	222.2		Z	Tubulin is the major constituent of microtubules. It binds two moles
Cladopus_004821	2.40E-21	106.7	RPL2	J	50S ribosomal protein L2
Cladopus_004822	7.70E-93	345.5	PSBA	P	This is one of the two reaction center proteins of photosystem II
Cladopus_004847	3.10E-44	183.7	RPL16	J	60S ribosomal protein L16
Cladopus_004886	5.00E-158	562.8		G	Pectate lyase

Cladopus_004897	1.00E-127	461.5	LHCB1-1		O	Chlorophyll a-b binding protein
Cladopus_005026	4.80E-222	775.8			U	SecY translocase
Cladopus_005073	2.40E-60	237.3			H	ATP synthase
Cladopus_005166	1.60E-23	114	PSBA		C	This is one of the two reaction center proteins of photosystem II
Cladopus_005175	2.90E-133	480.3		DAPE	E	Diaminopimelate epimerase
Cladopus_005178	1.70E-111	407.9			S	peroxidase
Cladopus_005180	2.20E-113	414.1	OSI_34838		G	peroxidase
Cladopus_005191	2.60E-114	417.2	OSI_34838		G	peroxidase
Cladopus_005198	3.80E-113	413.3	OSI_34838		G	peroxidase
Cladopus_005226	1.40E-112	411.4	OSI_34838		G	peroxidase
Cladopus_005229	1.40E-105	388.3	OSI_34838		G	peroxidase
Cladopus_005238	6.90E-105	386	OSI_34838		G	peroxidase
Cladopus_005244	1.60E-108	398.3	OSI_34838		G	peroxidase
Cladopus_005245	1.60E-108	398.3	OSI_34838		G	peroxidase
Cladopus_005246	3.10E-115	420.2	OSI_34838		G	peroxidase
Cladopus_005251	2.80E-106	390.6	OSI_34838		G	peroxidase
Cladopus_005660	0	1419.8	EMB2369		J	leucyl-tRNA
Cladopus_005690	2.60E-14	82.4	NAD1		C	NADH dehydrogenase subunit 1
Cladopus_005691	1.40E-10	70.1	NAD1		C	NADH dehydrogenase
Cladopus_005692	4.80E-40	169.1	NAD1		C	NADH dehydrogenase subunit 1
Cladopus_005718	2.00E-98	364.8			G	glycosyl hydrolase
Cladopus_005834	4.50E-36	156	PSAE		C	photosystem I reaction center subunit IV
Cladopus_005851	7.10E-94	348.6	SAM2	METAT	H	Catalyzes the formation of S-adenosylmethionine from methionine and
Cladopus_005914	1.10E-239	834.3	OSI_11478		C	Fumarate hydratase
Cladopus_006039	2.00E-92	344.7	FG04596.1		D	o-methyltransferase
Cladopus_006068	8.50E-109	400.6	RPOC2		K	DNA-dependent RNA polymerase catalyzes the transcription of DNA into
Cladopus_006105	8.50E-23	111.7			L	DNA polymerase
Cladopus_006221	2.50E-21	105.9	PSBK		S	Photosystem II reaction center protein K
Cladopus_006380	1.80E-36	156.8	PSBE		C	This b-type cytochrome is tightly associated with the reaction center
Cladopus_006465	7.70E-109	399.1	GA2OX1		Q	gibberellin
Cladopus_006497	1.70E-35	154.1	PSAE		C	photosystem I reaction center subunit IV
Cladopus_006522	5.80E-241	838.6	OSI_11478		C	Fumarate hydratase
Cladopus_006703	2.20E-152	544.3			G	pectinesterase
Cladopus_006894	1.90E-207	727.2			G	pectinesterase

Cladopus_006921	4.80E-65	252.3	TUB2		Z	Tubulin is the major constituent of microtubules. It binds two moles
Cladopus_006927	2.30E-182	643.7			G	fumarylacetoacetase EC 3.7.1.2
Cladopus_006934	2.30E-177	627.1			G	fumarylacetoacetase EC 3.7.1.2
Cladopus_006935	1.00E-193	681.4			G	fumarylacetoacetase EC 3.7.1.2
Cladopus_006939	2.50E-173	613.6			G	Pfam:DUF1969
Cladopus_006955	4.60E-83	313.2	OSI_24417		E	prephenate dehydrogenase family protein
Cladopus_006972	9.50E-09	66.2			J	ribosomal protein S3
Cladopus_006976	3.00E-29	132.9	NAD1		C	NADH dehydrogenase subunit 1
Cladopus_007108	3.70E-141	507.7			G	beta-glucosidase
Cladopus_007114	1.20E-38	165.6			G	beta-glucosidase
Cladopus_007119	7.50E-156	556.2			G	beta-glucosidase
Cladopus_007175	3.50E-192	676.8	CYP86B1		Q	cytochrome P450
Cladopus_007178	3.50E-192	676.8	CYP86B1		Q	cytochrome P450
Cladopus_007181	4.50E-94	349.7			O	ATP-dependent Clp protease proteolytic subunit
Cladopus_007235	4.00E-122	443			J	Component of the eukaryotic translation initiation factor 3 (eIF-3)
Cladopus_007299	1.60E-257	894			P	plasma membrane
Cladopus_007327	4.40E-23	113.6			K	Transcription factor
Cladopus_007521	4.80E-37	158.7	PSBE		C	This b-type cytochrome is tightly associated with the reaction center
Cladopus_007524	1.90E-21	106.3	PSBK		S	Photosystem II reaction center protein K
Cladopus_007560	1.80E-29	135.2	NAD4L		C	NADH dehydrogenase subunit 4L
Cladopus_007680	8.90E-41	171.4	RPS7		J	ribosomal protein S7
Cladopus_007898	6.20E-73	278.5	TUB2		Z	Tubulin is the major constituent of microtubules. It binds two moles
Cladopus_008315	2.70E-251	873.2		ATPS	P	plasma membrane ATPase
Cladopus_008427	1.70E-21	107.5	OSI_00989		Q	cytochrome P450
Cladopus_008439	1.40E-25	120.9	OSI_00989		Q	cytochrome P450
Cladopus_008484	3.90E-32	142.5	NAD4L		C	NADH dehydrogenase subunit 4L
Cladopus_008528	1.00E-83	315.1	EMB2394		J	50S ribosomal protein L6
Cladopus_008561	5.30E-100	369.4	PSBS		I	Photosystem II 22 kDa protein
Cladopus_008703	1.40E-68	266.2			C	Mitochondrial membrane ATP synthase (F(1)F(0) ATP synthase or Complex
Cladopus_008742	3.80E-187	659.8			Q	cytochrome P450
Cladopus_008819	2.50E-110	404.1	OSI_34838		G	peroxidase
Cladopus_008821	5.00E-113	412.9	OSI_34838		G	peroxidase
Cladopus_008824	2.30E-43	182.2			J	Ribosomal protein S3
Cladopus_008825	1.30E-20	105.1	NAD4L		C	NADH dehydrogenase subunit 4L

Cladopus_008831	2.60E-114	417.2	OSI_34838		G	peroxidase
Cladopus_008836	6.20E-108	396	OSI_34838		G	peroxidase
Cladopus_008853	8.90E-115	418.7	OSI_34838		G	peroxidase
Cladopus_008854	4.60E-114	416.8	OSI_34838		G	peroxidase
Cladopus_008855	4.00E-115	419.9	OSI_34838		G	peroxidase
Cladopus_008870	2.30E-110	404.1			S	peroxidase
Cladopus_008875	4.90E-133	479.6		DAPE	E	Diaminopimelate epimerase
Cladopus_008916	1.90E-47	194.1			G	Glycosyl hydrolase family 38 protein
Cladopus_009142	8.20E-61	239.2			H	atp synthase
Cladopus_009250	1.20E-202	711.4			Q	cytochrome P450
Cladopus_009307	1.90E-87	328.2	FG04596.1		D	o-methyltransferase
Cladopus_009477	7.40E-46	188.3	RPS7		J	ribosomal protein S7
Cladopus_009722	3.80E-96	357.1	OSI_05571		Z	WD domain, G-beta repeat
Cladopus_009739	3.30E-100	370.5	OSI_05571		S	WD40
Cladopus_009859	0	1497.3			U	pattern formation protein
Cladopus_009899	5.40E-122	443	OSI_24417		E	Arogenate dehydrogenase
Cladopus_009900	1.80E-123	448	OSI_24417		E	Arogenate dehydrogenase
Cladopus_009902	2.10E-142	511.1			G	pectinesterase
Cladopus_009933	1.30E-71	274.2	PHES		J	Phenylalanyl-tRNA synthetase
Cladopus_009989	4.20E-112	409.8	OSI_34838		G	peroxidase
Cladopus_009990	4.20E-112	409.8	OSI_34838		G	peroxidase
Cladopus_009993	8.70E-110	402.1	OSI_34838		G	peroxidase
Cladopus_009996	2.50E-109	400.6	OSI_34838		G	peroxidase
Cladopus_009998	1.30E-110	404.8	OSI_34838		G	peroxidase
Cladopus_010007	4.40E-148	530			G	beta-glucosidase
Cladopus_010109	4.10E-28	129	RPS7		J	ribosomal protein S7
Cladopus_010176	6.30E-108	396	MRPS12		J	Ribosomal protein s12
Cladopus_010177	3.90E-37	159.5	ND2		C	Core subunit of the mitochondrial membrane respiratory chain NADH
Cladopus_010238	4.50E-128	462.6	LHCB1-1		O	Chlorophyll a-b binding protein
Cladopus_010306	9.10E-129	464.9	LHCB1-1		O	Chlorophyll a-b binding protein
Cladopus_010307	4.80E-127	459.5	LHCB1-1		O	Chlorophyll a-b binding protein
Cladopus_010308	9.80E-131	471.5	LHCB1-1		O	Chlorophyll a-b binding protein
Cladopus_010309	3.70E-130	469.5	LHCB1-1		O	Chlorophyll a-b binding protein
Cladopus_010320	9.80E-131	471.5	LHCB1-1		O	Chlorophyll a-b binding protein

Cladopus_010321	9.80E-131	471.5	LHCB1-1		O	Chlorophyll a-b binding protein
Cladopus_010341	2.00E-119	434.1		LALDO	Q	sensitive to hot temperatures 5
Cladopus_010500	7.70E-167	591.7	PSBA		P	This is one of the two reaction center proteins of photosystem II
Cladopus_010501	2.50E-227	793.1	RBCL		C	RuBisCO catalyzes two reactions the carboxylation of D- ribulose 1,5-
Cladopus_010580	1.30E-257	894.4			P	plasma membrane
Cladopus_010634	6.90E-44	182.6	RPL16		J	60S ribosomal protein L16
Cladopus_010676	1.10E-77	294.7	ILVH	ACHBS,ACL	E	Acetolactate synthase, small subunit
Cladopus_010684	1.30E-112	411.8	FBPC	FE3abcpp	P	ABC transporter
Cladopus_010687	7.20E-100	368.6	LSFA		O	Alkyl hydroperoxide reductase Thiol specific antioxidant
Cladopus_010694	2.30E-229	800.8	GLGX		G	Glycogen debranching enzyme
Cladopus_010703	0	1232.2			P	Transporter, hydrophobe amphiphile efflux-1 (HAE1) family
Cladopus_010741	2.50E-176	623.6	YNBB		P	aluminum resistance protein
Cladopus_010754	7.00E-247	859	ATPA	ATPS1,ATP	C	Produces ATP from ADP in the presence of a proton gradient across the
Cladopus_010762	1.80E-175	621.7	GABD1	OXPTNDH,S	C	Aldehyde dehydrogenase
Cladopus_010779	4.00E-85	319.7	UBIX	OPHBDC	H	aromatic acid decarboxylase
Cladopus_010790	1.30E-247	860.5	PSBC		S	Photosystem II 44 kDa subunit reaction center protein
Cladopus_010791	1.90E-193	680.2	PSBD		C	One of the two reaction center proteins of photosystem II (PSII), D2
Cladopus_010801	3.30E-306	1056.6	PHET		J	phenylalanyl-tRNA synthetase beta subunit
Cladopus_010805	5.20E-92	342.8	RPIA	RPI	G	Phosphoriboisomerase A
Cladopus_010807	2.00E-265	920.6	RECQ		L	ATP-dependent DNA helicase
Cladopus_010813	0	1290	CLPB		O	Part of a stress-induced multi-chaperone system, it is involved in the
Cladopus_010825	3.40E-109	400.2	GPSA	G3PD1,G3P	C	NAD(P)H-dependent glycerol-3-phosphate dehydrogenase
Cladopus_010830	4.60E-167	592.8	FUMC	FUM	C	fumarate hydratase, class II
Cladopus_010850	3.90E-200	703.4	PURA	ADSS	F	Plays an important role in the de novo pathway of purine nucleotide
Cladopus_010865	4.90E-40	168.7	RPSN		J	Binds 16S rRNA, required for the assembly of 30S particles and may
Cladopus_010869	0	1369	BCHH		H	Magnesium chelatase subunit H
Cladopus_010884	3.00E-278	963	UREC	UREA	E	Urea amidohydrolase subunit alpha
Cladopus_010891	2.20E-124	450.7			M	glycosyl transferase family
Cladopus_010904	5.90E-43	180.6			T	Two component transcriptional regulator, winged helix family
Cladopus_010907	0	1102	NDHF		C	NAD(P)H-quinone oxidoreductase subunit F
Cladopus_010908	1.60E-244	850.5	NDHD		C	NDH-1 shuttles electrons from NAD(P)H, via FMN and iron- sulfur (Fe-S)
Cladopus_010933	1.20E-48	197.6	RPLQ		J	50S ribosomal protein L17
Cladopus_010934	6.40E-61	238.8	RPSK		J	Located on the platform of the 30S subunit, it bridges several
Cladopus_010935	9.90E-177	625.9	SECY		U	The central subunit of the protein translocation channel SecYEG.

Cladopus_010936	7.10E-70	270.4	RPLF		J	This protein binds to the 23S rRNA, and is important in its secondary
Cladopus_010939	1.80E-83	315.5	RPSC		J	Binds the lower part of the 30S subunit head. Binds mRNA in the 70S
Cladopus_010946	9.70E-153	546.2	MUTY		L	A G-specific adenine glycosylase
Cladopus_010956	0	1386.7	ACNB	ACONT,ACO	C	Aconitate hydratase 2
Cladopus_010967	6.20E-140	503.1	SIGB		K	Sigma factors are initiation factors that promote the attachment of
Cladopus_010973	1.30E-197	694.1	PSBA		C	This is one of the two reaction center proteins of photosystem II
Cladopus_010980	6.60E-145	520	PSBA		C	This is one of the two reaction center proteins of photosystem II
Cladopus_010981	6.10E-191	673.3	NDHF3	NDH1_1p,N	C	dehydrogenase subunit
Cladopus_010982	0	1446.4	CLPB		O	Part of a stress-induced multi-chaperone system, it is involved in the
Cladopus_011053	1.50E-75	287.3	PSBD		C	One of the two reaction center proteins of photosystem II (PSII), D2
Cladopus_011145	1.00E-119	435.3	OSI_24417		E	Arogenate dehydrogenase
Cladopus_011146	1.30E-122	444.9	OSI_24417		E	Arogenate dehydrogenase
Cladopus_011149	4.20E-143	513.5			G	pectinesterase
Cladopus_011237	1.50E-189	667.9			G	pectinesterase
Cladopus_011262	8.40E-130	468.4	LHCB1-1		O	Chlorophyll a-b binding protein
Cladopus_011276	4.60E-129	466.1	LHCB1-1		O	Chlorophyll a-b binding protein
Cladopus_011294	2.40E-129	466.8	LHCB1-1		O	Chlorophyll a-b binding protein
Cladopus_011636	5.30E-175	619.8			G	pectinesterase
Cladopus_011637	1.20E-196	691.4			G	pectinesterase
Cladopus_011638	1.70E-21	106.7	RPS15		J	30S ribosomal protein S15, chloroplastic
Cladopus_011639	1.60E-75	287.3	NDHH		C	respiratory chain. The immediate electron acceptor for the enzyme
Cladopus_011640	1.80E-49	201.1	NDHH		C	respiratory chain. The immediate electron acceptor for the enzyme
Cladopus_011801	5.30E-115	419.5	OSI_34838		G	peroxidase
Cladopus_011923	3.30E-23	112.8	NAD4L		C	NADH dehydrogenase subunit 4L
Cladopus_012173	2.40E-102	377.5	GA2OX1		Q	gibberellin
Cladopus_012176	1.20E-94	352.1	GA2OX1		Q	gibberellin
Cladopus_012381	5.90E-21	104.8	ATPH		C	F(1)F(0) ATP synthase produces ATP from ADP in the presence of a
Cladopus_012818	4.10E-107	393.3	OSI_34838		G	peroxidase
Cladopus_012820	8.80E-110	402.1			S	peroxidase
Cladopus_012900	5.70E-11	72			O	cysteine proteinase
Cladopus_012937	7.30E-74	283.5	OMR1		E	threonine dehydratase biosynthetic
Cladopus_013026	2.30E-48	197.2			H	Photosystem II
Cladopus_013096	4.40E-232	809.3	NCED3		Q	9-cis-epoxy-carotenoid dioxygenase
Cladopus_013164	1.40E-66	257.7	ACCD		H	Component of the acetyl coenzyme A carboxylase (ACC) complex. Biotin

Cladopus_013231	2.30E-96	357.8	OSI_05571	Z	WD domain, G-beta repeat
Cladopus_013283	3.20E-126	456.4	LHCB1-1	O	Chlorophyll a-b binding protein
Cladopus_013346	2.60E-74	283.1	RBCL	C	RuBisCO catalyzes two reactions the carboxylation of D- ribulose 1,5-
Cladopus_013347	9.40E-81	304.7	RBCL	C	RuBisCO catalyzes two reactions the carboxylation of D- ribulose 1,5-
Cladopus_013348	1.60E-164	583.9	PSBA	P	This is one of the two reaction center proteins of photosystem II
Cladopus_013350	3.50E-67	259.6	RPL2	J	50S ribosomal protein L2
Cladopus_013494	3.50E-13	79.3	RPS15	J	30S ribosomal protein S15, chloroplastic
Cladopus_013496	1.80E-163	580.9	OSI_33884	G	Pectate lyase
Cladopus_013808	4.50E-14	83.2	NDHB1	C	NAD(P)H-quinone oxidoreductase subunit 2 B, chloroplastic (EC
Cladopus_013952	2.40E-35	152.9	ATPH	C	F(1)F(0) ATP synthase produces ATP from ADP in the presence of a
Cladopus_013953	2.20E-76	290.4	ATPF	C	F(1)F(0) ATP synthase produces ATP from ADP in the presence of a
Cladopus_013954	3.10E-250	869.4	ATPA	C	Produces ATP from ADP in the presence of a proton gradient across the
Cladopus_014025	1.30E-127	461.1	LHCB1-1	O	Chlorophyll a-b binding protein
Cladopus_014026	1.50E-122	444.1	LHCB1-1	O	Chlorophyll a-b binding protein
Cladopus_014028	4.80E-125	452.6	LHCB1-1	O	Chlorophyll a-b binding protein
Cladopus_014029	7.70E-128	461.8	LHCB1-1	O	Chlorophyll a-b binding protein
Cladopus_014041	3.50E-128	463	LHCB1-1	O	Chlorophyll a-b binding protein
Cladopus_014043	2.20E-127	460.3	LHCB1-1	O	Chlorophyll a-b binding protein
Cladopus_014242	8.00E-76	288.9	RPL2	J	50S ribosomal protein L2
Cladopus_014254	3.50E-128	463	LHCB1-1	O	Chlorophyll a-b binding protein
Cladopus_014283	4.00E-25	119	NAD4L	C	NADH dehydrogenase subunit 4L
Cladopus_014284	8.70E-11	73.2	RPL16	J	ribosomal protein L16
Cladopus_014285	1.30E-09	67.8	NAD4L	C	NADH dehydrogenase subunit 4L
Cladopus_014307	8.10E-133	480.7	RPOC1	K	DNA-dependent RNA polymerase catalyzes the transcription of DNA into
Cladopus_014308	2.40E-35	152.9	ATPH	C	F(1)F(0) ATP synthase produces ATP from ADP in the presence of a
Cladopus_014406	6.70E-53	213.4		G	beta-glucosidase
Cladopus_014408	4.80E-20	103.6		G	Glycosyl hydrolase family 1
Cladopus_014422	6.70E-110	402.5	OSI_34838	G	peroxidase
Cladopus_014424	6.70E-110	402.5	OSI_34838	G	peroxidase
Cladopus_014426	2.90E-91	340.5	OSI_34838	G	peroxidase
Cladopus_014506	2.40E-79	300.8	PPCK1	T	phosphoenolpyruvate carboxylase kinase
Cladopus_014509	8.90E-87	325.5	PPCK1	T	phosphoenolpyruvate carboxylase kinase
Cladopus_014609	1.40E-187	661.8		Q	cytochrome P450
Cladopus_014764	1.80E-20	105.1	TAF15	K	TAF15 RNA polymerase II, TATA box binding protein (TBP)-associated

Cladopus_014822	8.40E-37	158.3	NAD5	C	NADH-Ubiquinone oxidoreductase (complex I), chain 5 N-terminus
Cladopus_014839	1.40E-174	618.2		G	pectinesterase
Cladopus_014846	6.70E-177	625.9		G	pectinesterase
Cladopus_015510	3.40E-91	340.1		O	ATP-dependent Clp protease proteolytic subunit
Cladopus_015635	2.20E-31	140.6	NDHB1	C	NDH shuttles electrons from NAD(P)H plastoquinone, via FMN and iron-
Cladopus_015714	3.99999999	1068.9	OSI_20132	U	sec23 sec24 transport family protein
Cladopus_015716	0	1163.3	OSI_20132	U	sec23 sec24 transport family protein
Cladopus_015718	2.00E-169	600.9		I	fatty acyl-CoA reductase
Cladopus_015719	5.10E-102	376.7		I	fatty acyl-CoA reductase
Cladopus_015721	4.10E-183	646.4		I	fatty acyl-CoA reductase
Cladopus_015722	7.60E-176	622.5		I	fatty acyl-CoA reductase
Cladopus_015724	3.60E-179	633.3		I	fatty acyl-CoA reductase
Cladopus_015825	0	1271.1	URE	F	Urea amidohydrolase
Cladopus_015845	3.60E-147	526.6	PSBA	C	This is one of the two reaction center proteins of photosystem II
Cladopus_015846	4.80E-118	429.1	RBCL	C	RuBisCO catalyzes two reactions the carboxylation of D- ribulose 1,5-
Cladopus_015877	4.60E-123	446.4		G	Converts alpha-aldose to the beta-anomer. It is active on D-glucose,
Cladopus_015878	3.40E-103	380.6		G	Converts alpha-aldose to the beta-anomer. It is active on D-glucose,
Cladopus_015879	3.00E-114	417.2		G	Converts alpha-aldose to the beta-anomer. It is active on D-glucose,
Cladopus_015949	1.30E-61	241.1	TPX1	O	Reduces hydrogen peroxide and alkyl hydroperoxides with reducing
Cladopus_015950	1.30E-61	241.1	TPX1	O	Reduces hydrogen peroxide and alkyl hydroperoxides with reducing
Cladopus_016119	2.80E-144	517.3	PSBD	C	Inherit from lilNOG: One of the two reaction center proteins of
Cladopus_016172	3.30E-21	108.2	NAD9	C	Core subunit of the mitochondrial membrane respiratory chain NADH
Cladopus_016191	5.50E-197	692.6	CYP86B1	Q	cytochrome P450
Cladopus_016197	5.90E-94	349.4		O	ATP-dependent Clp protease proteolytic subunit
Cladopus_016221	1.80E-14	84.7	RPL14	J	ribosomal protein L14
Cladopus_016254	2.80E-33	146.4	PETB	C	Component of the ubiquinol-cytochrome c reductase complex (complex III
Cladopus_016286	1.40E-138	497.7	ATPA	C	Produces ATP from ADP in the presence of a proton gradient across the
Cladopus_016504	3.30E-65	253.4	PRPL17	J	ribosomal protein L17
Cladopus_016505	5.90E-107	392.5		O	2-Cys peroxiredoxin
Cladopus_016577	1.80E-197	694.1		G	pectinesterase
Cladopus_016578	1.60E-176	624.8		G	pectinesterase
Cladopus_016594	3.10E-29	133.7	NDHB	C	NDH shuttles electrons from NAD(P)H plastoquinone, via FMN and iron-
Cladopus_016885	2.10E-206	723.8		G	pectinesterase
Cladopus_016913	5.60E-21	104.8	PSBK	S	Photosystem II reaction center protein K

Cladopus_016921	1.60E-09	66.6	NDHD		C	NADH dehydrogenase subunit 4
Cladopus_016923	3.30E-80	304.7	NAD7		C	NADH dehydrogenase subunit 7
Cladopus_016959	2.70E-213	746.9	NCED3		Q	9-cis-epoxy-carotenoid dioxygenase
Cladopus_016961	3.10E-217	760	NCED3		Q	9-cis-epoxy-carotenoid dioxygenase
Cladopus_016962	5.80E-214	749.2	NCED3		Q	9-cis-epoxy-carotenoid dioxygenase
Cladopus_017012	5.40E-187	659.4			G	pectinesterase
Cladopus_017296	2.20E-67	260.4	ACCD		H	Component of the acetyl coenzyme A carboxylase (ACC) complex. Biotin
Cladopus_017298	1.70E-91	340.9	PETA		C	Component of the cytochrome b6-f complex, which mediates electron
Cladopus_017326	2.20E-24	116.3	ATPE		C	Produces ATP from ADP in the presence of a proton gradient across the
Cladopus_017492	1.60E-167	594.3	OMR1		E	threonine dehydratase biosynthetic
Cladopus_017528	5.60E-51	207.2			G	Glycosyl transferase family 4
Cladopus_017580	6.20E-20	101.7			C	This is one of the two reaction center proteins of photosystem II
Cladopus_017582	2.90E-23	113.2	RPL2		J	50S ribosomal protein L2
Cladopus_017689	5.50E-126	455.7	LHCB1-1		O	Chlorophyll a-b binding protein
Cladopus_017807	5.60E-21	104.8	PSBK		S	Photosystem II reaction center protein K
Cladopus_017808	2.30E-19	99.4	PSBK		S	Photosystem II reaction center protein K
Cladopus_017938	5.30E-30	135.6			H	Photosystem II 11 kD protein
Cladopus_017957	9.00E-108	395.2	PSBS		I	Photosystem II 22 kDa protein
Cladopus_017976	8.40E-130	468.4	LHCB1-1		O	Chlorophyll a-b binding protein
Cladopus_017988	4.50E-128	462.6	LHCB1-1		O	Chlorophyll a-b binding protein
Cladopus_018002	4.50E-128	462.6	LHCB1-1		O	Chlorophyll a-b binding protein
Cladopus_018003	7.00E-129	465.3	LHCB1-1		O	Chlorophyll a-b binding protein
Cladopus_018100	4.10E-90	335.9	NAD9		C	Core subunit of the mitochondrial membrane respiratory chain NADH
Cladopus_018102	5.00E-104	382.5	NAD9		C	Core subunit of the mitochondrial membrane respiratory chain NADH
Cladopus_018176	1.00E-50	206.5			G	Phospho-N-acetylmuramoyl-pentapeptide-transferase homolog
Cladopus_018213	1.90E-168	597.4	OMR1		E	threonine dehydratase biosynthetic
Cladopus_018248	5.10E-23	111.7	RPS15		J	30S ribosomal protein S15, chloroplastic
Cladopus_018255	0	1080.5		NITR	C	Nitrate reductase is a key enzyme involved in the first step of
Cladopus_018315	9.10E-129	464.9	LHCB1-1		O	Chlorophyll a-b binding protein
Cladopus_018316	1.70E-130	470.7	LHCB1-1		O	Chlorophyll a-b binding protein
Cladopus_018317	2.80E-130	469.9	LHCB1-1		O	Chlorophyll a-b binding protein
Cladopus_018485	1.80E-27	128.3	ACCD		H	Component of the acetyl coenzyme A carboxylase (ACC) complex. Biotin
Cladopus_018624	1.80E-30	137.9	HSC70-1		O	heat shock
Cladopus_018704	1.20E-16	91.7	RPL2		J	ribosomal protein L2

Cladopus_018720	0	1370.1		NITR	C	Nitrate reductase is a key enzyme involved in the first step of
Cladopus_019085	1.00E-115	422.2			V	K13963 serpin B
Cladopus_019256	4.80E-109	399.4			O	2-Cys peroxiredoxin
Cladopus_019264	5.00E-65	252.7	PRPL17		J	ribosomal protein L17
Cladopus_019368	2.80E-180	637.1			I	fatty acyl-CoA reductase
Cladopus_019370	5.30E-168	596.3			I	fatty acyl-CoA reductase
Cladopus_019371	5.30E-168	596.3			I	fatty acyl-CoA reductase
Cladopus_019372	7.90E-169	599			I	fatty acyl-CoA reductase
Cladopus_019374	0	1167.9	OSI_20132		U	sec23 sec24 transport family protein
Cladopus_019410	3.00E-182	643.3			G	fumarylacetoacetase EC 3.7.1.2
Cladopus_019418	6.50E-185	652.1			G	fumarylacetoacetase EC 3.7.1.2
Cladopus_019419	1.10E-192	677.9			G	fumarylacetoacetase EC 3.7.1.2
Cladopus_019496	1.80E-08	63.5			O	cathepsin
Cladopus_019496-RB	1.80E-08	63.5			O	cathepsin
Cladopus_019614	5.50E-199	699.1	NUON	NADH5,NDH	C	electron donor, via FMN and iron-sulfur (Fe-S) centers, to quinones in
Cladopus_019618	4.40E-178	630.2	UGD		M	Udp-glucose
Cladopus_019622	2.80E-92	345.1	COBD	THRPD	E	decarboxylase
Cladopus_019659	3.60E-100	369.8	ACCD	ACCOAC	I	Component of the acetyl coenzyme A carboxylase (ACC) complex. Biotin
Cladopus_019667	0	1388.2	ACNB	ACONT,ACO	C	Aconitate hydratase 2
Cladopus_019676	6.00E-96	355.5	RPSD		J	One of the primary rRNA binding proteins, it binds directly to 16S
Cladopus_019701	3.10E-259	899.4	CBBL	RBCh,RBPC	G	RuBisCO catalyzes two reactions the carboxylation of D- ribulose 1,5-
Cladopus_019702	0	2094.3	BCHH		H	Magnesium chelatase subunit H
Cladopus_019721	2.50E-183	647.5	SAT	SADT	P	Sulfate adenylate transferase
Cladopus_019728	0	1212.2			P	Transporter, hydrophobe amphiphile efflux-1 (HAE1) family
Cladopus_019742	2.70E-208	729.9	ASPA	ASPT	C	aspartate ammonia-lyase (EC
Cladopus_019744	2.20E-204	716.8	PURA	ADSS	F	Plays an important role in the de novo pathway of purine nucleotide
Cladopus_019860	1.60E-66	257.7	ND2		C	Core subunit of the mitochondrial membrane respiratory chain NADH
Cladopus_019862	7.20E-73	278.9	PETA		C	Component of the cytochrome b6-f complex, which mediates electron
Cladopus_019895	2.80E-268	929.9		ATPS	P	Plasma membrane
Cladopus_020046	2.30E-53	214.9	RPS7		J	ribosomal protein S7
Cladopus_020220	1.80E-149	534.3		LALDO	Q	dehydrogenase
Cladopus_020240	7.80E-128	461.8	LHCB1-1		O	Chlorophyll a-b binding protein
Cladopus_020241	1.80E-129	467.2	LHCB1-1		O	Chlorophyll a-b binding protein
Cladopus_020261	5.30E-25	118.6	RPS18		J	Ribosomal protein S18

Cladopus_020330	1.70E-127	460.7			S	Chlorophyll A-B binding protein
Cladopus_020574	2.30E-76	290	RPL2		J	50S ribosomal protein L2
Cladopus_020586	0	1290.8	LEUS		J	Leucyl-tRNA synthetase
Cladopus_020592	5.90E-207	726.9	NDHH	NDH1_1p,N	C	electron donor, via FMN and iron-sulfur (Fe-S) centers, to quinones in
Cladopus_020602	1.10E-180	637.9	CHLP		H	geranylgeranyl reductase
Cladopus_020624	9.30E-126	455.7	GABD1	OXPTNDH,S	C	Aldehyde dehydrogenase
Cladopus_020630	3.80E-69	266.5	NDHD		C	NDH-1 shuttles electrons from NAD(P)H, via FMN and iron- sulfur (Fe-S)
Cladopus_020631	2.10E-159	567	NDHD		C	NDH-1 shuttles electrons from NAD(P)H, via FMN and iron- sulfur (Fe-S)
Cladopus_020632	5.50E-173	612.5	NDHF		C	NAD(P)H-quinone oxidoreductase subunit F
Cladopus_020633	1.40E-104	384.4	NDHF		C	NAD(P)H-quinone oxidoreductase subunit F
Cladopus_020646	1.20E-211	741.1	FUMC	FUM	C	fumarate hydratase, class II
Cladopus_020649	8.60E-124	448.7	RPSB		J	30S ribosomal protein S2
Cladopus_020661	3.00E-95	353.2	RPSD		J	One of the primary rRNA binding proteins, it binds directly to 16S
Cladopus_020681	6.30E-232	808.9	PHET		J	phenylalanyl-tRNA synthetase beta subunit
Cladopus_020696	6.30E-102	376.7			T	Two component transcriptional regulator, winged helix family
Cladopus_020750	3.20E-31	139.8	NDHK		C	NDH shuttles electrons from NAD(P)H plastoquinone, via FMN and iron-
Cladopus_020777	3.70E-41	172.9	RPSG		J	One of the primary rRNA binding proteins, it binds directly to 16S
Cladopus_020786	4.80E-38	162.5	SUSA		G	synthase
Cladopus_020795	1.90E-84	318.9	PHOR		T	Histidine kinase
Cladopus_020797	8.70E-196	688.3	CHLP		H	geranylgeranyl reductase
Cladopus_020809	1.30E-175	620.9	PSBD		C	One of the two reaction center proteins of photosystem II (PSII), D2
Cladopus_020816	8.70E-169	600.1	PHOP		T	transcriptional regulator, winged helix family
Cladopus_020832	3.10E-48	196.8	RPLT		J	Binds directly to 23S ribosomal RNA and is necessary for the in vitro
Cladopus_020847	4.80E-93	345.9	RPSD		J	One of the primary rRNA binding proteins, it binds directly to 16S
Cladopus_020853	2.70E-70	270.8	NDHG	NDH1_1p,N	C	NADH dehydrogenase subunit J
Cladopus_020854	1.50E-184	651.4	NUOH	NDH1_1p,N	C	electron donor, via FMN and iron-sulfur (Fe-S) centers, to quinones in
Cladopus_020872	5.40E-297	1025	THRS		J	Threonyl-tRNA synthetase
Cladopus_020881	2.10E-118	430.6	DAPF	DAPE	E	Catalyzes the stereoinversion of LL-2,6- diaminoheptanedioate (L,L-
Cladopus_020884	4.00E-106	391	AAPM	ARGabcpp,	E	amino acid ABC transporter
Cladopus_020909	1.20E-258	897.5	CTAD		C	Cytochrome C oxidase subunit I
Cladopus_020911	1.20E-124	451.4	ARGF	OCBT,OCBT	E	ornithine carbamoyltransferase
Cladopus_021081	3.40E-60	236.1	RPSK		J	Located on the platform of the 30S subunit, it bridges several
Cladopus_021083	2.30E-148	531.2	SECY		U	The central subunit of the protein translocation channel SecYEG.
Cladopus_021086	1.10E-69	268.9	RPLF		J	This protein binds to the 23S rRNA, and is important in its secondary

Cladopus_021089	3.20E-59	233	RPLP		J	Binds 23S rRNA and is also seen to make contacts with the A and
Cladopus_021090	3.10E-105	387.5	RPSC		J	Binds the lower part of the 30S subunit head. Binds mRNA in the 70S
Cladopus_021091	5.50E-135	485.7	RPLB		J	One of the primary rRNA binding proteins. Required for association of
Cladopus_021107	8.20E-110	402.9	DAPF	DAPE	E	Catalyzes the stereoinversion of LL-2,6- diaminoheptanedioate (L,L-
Cladopus_021134	2.80E-120	437.2	MRAY	PAPPT3	M	First step of the lipid cycle reactions in the biosynthesis of the
Cladopus_021136	1.70E-36	157.1	PSBE		C	This b-type cytochrome is tightly associated with the reaction center
Cladopus_021149	6.20E-60	235.7	COAD	PTPATi	H	Reversibly transfers an adenylyl group from ATP to 4'-
Cladopus_021162	2.30E-205	721.5	GATA		J	Allows the formation of correctly charged Gln-tRNA(Gln) through the
Cladopus_021169	3.10E-88	330.5	UBIX	OPHBDC	H	aromatic acid decarboxylase
Cladopus_021183	2.10E-51	206.8	GLGA	GLCS1	G	Synthesizes alpha-1,4-glucan chains using ADP-glucose (By similarity)
Cladopus_021213	3.90E-22	109	NDHD		C	NADH dehydrogenase subunit 4
Cladopus_021243	4.50E-128	462.6	LHCB1-1		O	Chlorophyll a-b binding protein
Cladopus_021260	4.30E-119	433.3			V	K13963 serpin B
Cladopus_021262	5.00E-123	446.4			V	K13963 serpin B
Cladopus_021263	4.20E-122	443.4			V	K13963 serpin B
Cladopus_021280	4.10E-90	335.9	NAD9		C	Core subunit of the mitochondrial membrane respiratory chain NADH
Cladopus_021329	5.90E-83	312.8	PPCK1		T	phosphoenolpyruvate carboxylase kinase
Cladopus_021367	7.20E-126	455.3	LHCB1-1		O	Chlorophyll a-b binding protein
Cladopus_021368	4.50E-128	462.6	LHCB1-1		O	Chlorophyll a-b binding protein
Cladopus_021369	3.50E-128	463	LHCB1-1		O	Chlorophyll a-b binding protein
Cladopus_021388	1.70E-120	438			V	K13963 serpin B
Cladopus_021389	7.20E-122	442.6			V	K13963 serpin B
Cladopus_021553	2.40E-92	344	DDB2		L	damaged DNA-binding
Cladopus_021925	0	1501.1	CLPB3		O	chaperone protein
Cladopus_021993	0	1364.7		NITR	C	Nitrate reductase is a key enzyme involved in the first step of
Cladopus_022014	1.10E-56	226.5	NAD7		C	NADH dehydrogenase subunit 7
Cladopus_022023	5.40E-67	261.5	NAD4		C	NADH-Ubiquinone/plastoquinone (complex I), various chains
Cladopus_022024	2.90E-24	117.5			J	ribosomal protein S3
Cladopus_022026	3.80E-23	114	NAD1		C	NADH dehydrogenase subunit 1
Cladopus_022251	1.20E-41	174.5	COAD	PTPATi	H	Reversibly transfers an adenylyl group from ATP to 4'-
Cladopus_022252	6.50E-43	178.7	RPSN		J	Binds 16S rRNA, required for the assembly of 30S particles and may
Cladopus_022269	4.90E-255	887.1	COXA	CYO1b2pp_	C	Cytochrome C oxidase subunit I
Cladopus_022274	1.60E-108	397.5	UGD		M	Udp-glucose
Cladopus_022300	1.70E-71	274.2	HRTA		V	ABC transporter

Cladopus_022307	4.10E-64	249.6	ILVH	ACHBS,ACL	E	Acetolactate synthase, small subunit
Cladopus_022314	6.80E-83	312.4	HRTA		V	ABC transporter
Cladopus_022319	1.70E-184	650.6	SERC		E	Catalyzes the reversible conversion of 3- phosphohydroxypyruvate to
Cladopus_022332	6.10E-129	466.8	ARGF	OCBT,OCBT	E	ornithine carbamoyltransferase
Cladopus_022333	8.90E-136	490	MRAY	PAPPT3	M	First step of the lipid cycle reactions in the biosynthesis of the
Cladopus_022341	1.20E-270	938.3	PBPC		M	penicillin-binding protein
Cladopus_022560	0	2490.3	CHLH		H	chelatase subunit
Cladopus_022581	1.30E-23	114	NAD4L		C	NADH dehydrogenase subunit 4L
Cladopus_022743	2.60E-30	136.3	RPS2		J	ribosomal protein S2
Cladopus_022912	1.20E-150	538.1	OTC1	OCT	E	ornithine carbamoyltransferase
Cladopus_022952	0	1145.6		NITR	C	Nitrate reductase is a key enzyme involved in the first step of
Cladopus_023126	7.40E-176	622.5			G	pectinesterase
Cladopus_023192	3.50E-08	62	RPS15		J	30S ribosomal protein S15, chloroplastic
Cladopus_023260	2.60E-121	440.3			J	Component of the eukaryotic translation initiation factor 3 (eIF-3)
Cladopus_023399	1.10E-114	418.7			G	Converts alpha-aldose to the beta-anomer. It is active on D-glucose,
Cladopus_023400	6.40E-102	376.3			G	Converts alpha-aldose to the beta-anomer. It is active on D-glucose,
Cladopus_023401	4.20E-124	449.9			G	Converts alpha-aldose to the beta-anomer. It is active on D-glucose,
Cladopus_023714	6.60E-20	101.3	RPL33		J	Ribosomal protein L33
Cladopus_023715	0	1183.7	LEUS		J	Leucyl-tRNA synthetase
Cladopus_023744	4.30E-205	720.7	NDHH	NDH1_1p,N	C	electron donor, via FMN and iron-sulfur (Fe-S) centers, to quinones in
Cladopus_023750	2.20E-162	578.2	RECQ		L	ATP-dependent DNA helicase
Cladopus_023752	3.50E-223	779.6	ILVA	SERD_L,TH	E	Threonine dehydratase
Cladopus_023784	3.70E-261	906	GLMS	GF6PTA	M	Catalyzes the first step in hexosamine metabolism, converting
Cladopus_023801	4.60E-233	813.5	NDHD2	NDH1_1p,N	C	subunit m
Cladopus_023846	1.50E-20	103.6	RPS15		J	30S ribosomal protein S15, chloroplastic
Cladopus_023847	2.70E-86	323.6	NDHH		C	NDH shuttles electrons from NAD(P)H plastoquinone, via FMN and iron-
Cladopus_023848	3.10E-68	263.5	NDHA		C	NDH shuttles electrons from NAD(P)H plastoquinone, via FMN and iron-
Cladopus_023872	1.90E-16	90.5	RPS4		J	One of the primary rRNA binding proteins, it binds directly to 16S
Cladopus_023874	3.40E-35	152.5	PSBE		C	This b-type cytochrome is tightly associated with the reaction center
Cladopus_024023	2.50E-61	240.7	PSBD		C	One of the two reaction center proteins of photosystem II (PSII), D2
Cladopus_024053	7.20E-219	765.4			U	SecY translocase
Cladopus_024122	0	1118.2		NITR	C	Nitrate reductase is a key enzyme involved in the first step of
Cladopus_024123	0	1152.5		NITR	C	Nitrate reductase is a key enzyme involved in the first step of
Cladopus_024158	2.20E-163	581.3	SERC		E	Catalyzes the reversible conversion of 3- phosphohydroxypyruvate to

Cladopus_024195	8.90E-204	714.9	UGD		M	Udp-glucose
Cladopus_024197	2.20E-227	793.5	NUON	NADH5,NDH	C	electron donor, via FMN and iron-sulfur (Fe-S) centers, to quinones in
Cladopus_024222	6.40E-190	668.7	NDHH	NDH1_1p,N	C	electron donor, via FMN and iron-sulfur (Fe-S) centers, to quinones in
Cladopus_024233	1.60E-119	434.9	ACCD	ACCOAC	I	Component of the acetyl coenzyme A carboxylase (ACC) complex. Biotin
Cladopus_024234	1.30E-282	977.2	GLMS	GF6PTA	M	Catalyzes the first step in hexosamine metabolism, converting
Cladopus_024283	2.00E-274	950.3	UREC	UREA	E	Urea amidohydrolase subunit alpha
Cladopus_024306	2.30E-246	856.7	NDHD		C	NDH-1 shuttles electrons from NAD(P)H, via FMN and iron- sulfur (Fe-S)
Cladopus_024315	2.50E-26	122.9	RPLT		J	Binds directly to 23S ribosomal RNA and is necessary for the in vitro
Cladopus_024325	2.30E-246	856.7	CYOB		C	Cytochrome C oxidase subunit I
Cladopus_024333	2.60E-112	411.8	PHOP		T	Two component transcriptional regulator (Winged helix family
Cladopus_024432	1.90E-168	597.8			G	organic cation
Cladopus_024513	1.10E-148	531.9	COABC	PPCDC,PPN	H	Phosphopantothenoylcysteine decarboxylase
Cladopus_024515	4.10E-160	570.5	PSBD		C	One of the two reaction center proteins of photosystem II (PSII), D2
Cladopus_024536	2.50E-166	590.5	GABD1	OXPTNDH,S	C	Aldehyde dehydrogenase
Cladopus_024563	9.20E-253	877.9	NDHD		C	NDH-1 shuttles electrons from NAD(P)H, via FMN and iron- sulfur (Fe-S)
Cladopus_024564	3.80E-302	1042.3	NDHF		C	NAD(P)H-quinone oxidoreductase subunit F
Cladopus_024570	5.80E-66	256.9	ILVH	ACHBS,ACL	E	Acetolactate synthase, small subunit
Cladopus_024573	4.70E-50	203	RPLT		J	Binds directly to 23S ribosomal RNA and is necessary for the in vitro
Cladopus_024640	3.10E-20	104.4	TAF15		K	TAF15 RNA polymerase II, TATA box binding protein (TBP)-associated
Cladopus_024747	7.80E-40	168.3	RPL20		J	Binds directly to 23S ribosomal RNA and is necessary for the in vitro
Cladopus_024848	1.20E-270	938.3	PBPC		M	penicillin-binding protein
Cladopus_024874	4.00E-230	803.1	RECQ		L	ATP-dependent DNA helicase
Cladopus_024888	6.80E-183	646	YNBB		P	aluminum resistance protein
Cladopus_025011	8.50E-35	151.4	NAD4L		C	NADH dehydrogenase subunit 4L
Cladopus_025275	4.60E-118	429.5	DAPF	DAPE	E	Catalyzes the stereoinversion of LL-2,6- diaminoheptanedioate (L,L-
Cladopus_025292	6.40E-171	606.3	PHOP		T	transcriptional regulator, winged helix family
Cladopus_025311	7.00E-13	80.9	NAD4L		C	NADH dehydrogenase subunit 4L
Cladopus_025316	2.20E-163	581.3	SERC		E	Catalyzes the reversible conversion of 3- phosphohydroxypyruvate to
Cladopus_025338	2.10E-211	740.3	ILVA	SERD_L,TH	E	Threonine dehydratase
Cladopus_025342	3.10E-155	553.5	SIGC		K	Sigma factors are initiation factors that promote the attachment of
Cladopus_025353	3.00E-134	483.8	MRAY	PAPPT3	M	First step of the lipid cycle reactions in the biosynthesis of the
Cladopus_025360	0	1248.8	LEUS		J	Leucyl-tRNA synthetase
Cladopus_025521	2.40E-20	102.8	PSBE		C	This b-type cytochrome is tightly associated with the reaction center
Cladopus_025683	7.40E-145	520	NUOH	NDH1_1p,N	C	electron donor, via FMN and iron-sulfur (Fe-S) centers, to quinones in

Cladopus_025701	4.50E-106	391	RPLB		J	One of the primary rRNA binding proteins. Required for association of
Cladopus_025702	8.10E-18	94.7	RPLP		J	Binds 23S rRNA and is also seen to make contacts with the A and
Cladopus_025708	3.80E-170	603.6	SECY		U	The central subunit of the protein translocation channel SecYEG.
Cladopus_025716	2.00E-222	776.9	NUON	NADH5,NDH	C	electron donor, via FMN and iron-sulfur (Fe-S) centers, to quinones in
Cladopus_025717	4.70E-34	149.4	RPSO		J	One of the primary rRNA binding proteins, it binds directly to 16S
Cladopus_025754	0	1231.9	ACNB	ACONT,ACO	C	Aconitate hydratase 2
Cladopus_025761	1.70E-214	750.7	NDHD1		C	NDH-1 shuttles electrons from NAD(P)H, via FMN and iron- sulfur (Fe-S)
Cladopus_025762	1.60E-191	675.6	FUMC	FUM	C	fumarate hydratase, class II
Cladopus_025766	6.60E-86	322.4			T	Two component transcriptional regulator (Winged helix family
Cladopus_025769	2.70E-144	516.9	FUMC	FUM	C	fumarate hydratase, class II
Cladopus_025835	6.90E-120	436	ACCD	ACCOAC	I	Component of the acetyl coenzyme A carboxylase (ACC) complex. Biotin
Cladopus_025854	3.20E-194	684.1	ARGH	ARGSL,ARG	E	Arginosuccinase
Cladopus_025857	1.90E-47	193.7	LEUS		J	Leucyl-tRNA synthetase
Cladopus_025858	3.40E-298	1029.2	LEUS		J	Leucyl-tRNA synthetase
Cladopus_025911	3.20E-109	399.8	RPIA	RPI	G	Phosphoriboisomerase A
Cladopus_025913	0	1080.5	PHET		J	phenylalanyl-tRNA synthetase beta subunit
Cladopus_025918	9.20E-104	381.7	NDHK	NADHDH,ND	C	electron donor, via FMN and iron-sulfur (Fe-S) centers, to quinones in
Cladopus_025926	4.70E-192	675.6	PSBA		C	This is one of the two reaction center proteins of photosystem II
Cladopus_025928	6.10E-63	246.9	PEPE		E	peptidase S51, dipeptidase E
Cladopus_025942	3.10E-186	656.8	NAD5		C	NADH-Ubiquinone oxidoreductase (complex I), chain 5 N-terminus
Cladopus_025946	3.10E-186	656.8	NAD5		C	NADH-Ubiquinone oxidoreductase (complex I), chain 5 N-terminus
Cladopus_025950	3.10E-186	656.8	NAD5		C	NADH-Ubiquinone oxidoreductase (complex I), chain 5 N-terminus
Cladopus_025952	2.20E-211	740	COB		C	Component of the ubiquinol-cytochrome c reductase complex (complex III
Cladopus_025962	2.80E-18	95.9	NAD1		C	NADH dehydrogenase subunit 1
Cladopus_025966	1.40E-173	616.3			T	Histidine kinase
Cladopus_025988	3.80E-234	816.6	CBBL	RBCh,RBPC	G	RuBisCO catalyzes two reactions the carboxylation of D- ribulose 1,5-
Cladopus_025993	4.70E-208	729.9	CTAD		C	Cytochrome C oxidase subunit I
Cladopus_026012	3.70E-216	756.1	CBBL	RBCh,RBPC	G	RuBisCO catalyzes two reactions the carboxylation of D- ribulose 1,5-
Cladopus_026016	4.20E-94	349.4	AHPC	NTRARz	O	Thioredoxin peroxidase
Cladopus_026023	6.80E-52	209.5	PEPE		E	peptidase S51, dipeptidase E
Cladopus_026025	4.70E-192	675.6	PSBA		C	This is one of the two reaction center proteins of photosystem II
Cladopus_026030	2.40E-175	620.9	SIGA		K	Sigma factors are initiation factors that promote the attachment of
Cladopus_026057	1.50E-212	745.3	ARGH	ARGSL,ARG	E	Arginosuccinase
Cladopus_026064	3.70E-189	666.4	YNBB		P	aluminum resistance protein

Cladopus_026086	3.80E-234	816.6	CBBL	RBCh,RBPC	G	RuBisCO catalyzes two reactions the carboxylation of D- ribulose 1,5-
Cladopus_026098	4.20E-94	349.4	AHPC	NTRARz	O	Thioredoxin peroxidase
Cladopus_026104	2.10E-49	200.7	FOLK	DHNPA_1,F	H	2-amino-4-hydroxy-6- hydroxymethyldihydropteridine pyrophosphokinase
Cladopus_026106	9.70E-269	931.4	RECQ		L	ATP-dependent DNA helicase
Cladopus_026137	5.60E-77	292.7	PETA		C	Component of the cytochrome b6-f complex, which mediates electron
Cladopus_026144	9.00E-15	84	NAD1		C	NADH dehydrogenase subunit 1
Cladopus_026169	2.80E-18	95.9	NAD1		C	NADH dehydrogenase subunit 1
Cladopus_026180	3.40E-191	674.5	NDHH	NDH1_1p,N	C	electron donor, via FMN and iron-sulfur (Fe-S) centers, to quinones in
Cladopus_026190	2.10E-88	330.5	NDHK	NADHDH,ND	C	electron donor, via FMN and iron-sulfur (Fe-S) centers, to quinones in
Cladopus_026206	1.70E-214	750.7	NDHD1		C	NDH-1 shuttles electrons from NAD(P)H, via FMN and iron- sulfur (Fe-S)
Cladopus_026216	2.00E-102	377.9	YNBB		P	aluminum resistance protein
Cladopus_026217	6.00E-214	748.8	PURA	ADSS	F	Plays an important role in the de novo pathway of purine nucleotide
Cladopus_026220	6.20E-240	835.1	GATA		J	Allows the formation of correctly charged Gln-tRNA(Gln) through the
Cladopus_026247	7.40E-94	348.6	RPSD		J	One of the primary rRNA binding proteins, it binds directly to 16S
Cladopus_026282	2.99999999	1072.8	PHET		J	phenylalanyl-tRNA synthetase beta subunit
Cladopus_026287	3.80E-113	412.9	RPIA	RPI	G	Phosphoriboisomerase A
Cladopus_026300	7.90E-71	271.9	ILVH	ACHBS,ACL	E	Acetolactate synthase, small subunit
Cladopus_026302	5.50E-106	389.8	BGTB		E	amino acid ABC transporter
Cladopus_026321	4.80E-226	790	NUON	NADH5,NDH	C	electron donor, via FMN and iron-sulfur (Fe-S) centers, to quinones in
Cladopus_026327	1.60E-105	388.3	BGTB		E	amino acid ABC transporter
Cladopus_026343	1.70E-63	247.7	RPLP		J	Binds 23S rRNA and is also seen to make contacts with the A and
Cladopus_026344	1.10E-110	405.6	RPSC		J	Binds the lower part of the 30S subunit head. Binds mRNA in the 70S
Cladopus_026347	1.80E-85	321.2	RNHA		L	Endonuclease that specifically degrades the RNA of RNA- DNA hybrids
Cladopus_026349	2.90E-85	320.9	QCRB	CBFC2pp,C	C	Component of the cytochrome b6-f complex, which mediates electron
Cladopus_026358	7.90E-169	599			I	fatty acyl-CoA reductase
Cladopus_026359	9.40E-169	598.6			I	fatty acyl-CoA reductase
Cladopus_026360	5.00E-168	596.3			I	fatty acyl-CoA reductase
Cladopus_026361	7.90E-169	599			I	fatty acyl-CoA reductase
Cladopus_026362	9.40E-169	598.6			I	fatty acyl-CoA reductase
Cladopus_026376	4.70E-192	675.6	PSBA		C	This is one of the two reaction center proteins of photosystem II
Cladopus_026383	6.00E-232	808.5	GLGA	GLCS1	G	Synthesizes alpha-1,4-glucan chains using ADP-glucose (By similarity)
Cladopus_026432	2.10E-49	200.7	FOLK	DHNPA_1,F	H	2-amino-4-hydroxy-6- hydroxymethyldihydropteridine pyrophosphokinase
Cladopus_026434	0	1232.2			P	Transporter, hydrophobe amphiphile efflux-1 (HAE1) family
Cladopus_026459	1.80E-85	321.2	RNHA		L	Endonuclease that specifically degrades the RNA of RNA- DNA hybrids

Cladopus_026461	2.90E-85	320.9	QCRB	CBFC2pp,C	C	Component of the cytochrome b6-f complex, which mediates electron
Cladopus_026569	9.10E-210	734.6	NDHH	NDH1_1p,N	C	electron donor, via FMN and iron-sulfur (Fe-S) centers, to quinones in
Cladopus_026575	1.70E-145	521.5	PSBA		C	This is one of the two reaction center proteins of photosystem II
Cladopus_026596	2.80E-18	95.9	NAD1		C	NADH dehydrogenase subunit 1
Cladopus_026649	2.30E-134	483.8	NDHD1		C	NDH-1 shuttles electrons from NAD(P)H, via FMN and iron- sulfur (Fe-S)
Cladopus_026650	1.90E-33	146.7	NDHD1		C	NDH-1 shuttles electrons from NAD(P)H, via FMN and iron- sulfur (Fe-S)
Cladopus_026658	7.80E-291	1005.7	THRS		J	Threonyl-tRNA synthetase
Cladopus_026664	2.60E-275	953.4	SUSB		H	sucrose synthase
Cladopus_026689	1.50E-170	604.4	SERC		E	Catalyzes the reversible conversion of 3- phosphohydroxypyruvate to
Cladopus_026692	1.20E-193	681	PSBD		C	One of the two reaction center proteins of photosystem II (PSII), D2
Cladopus_026705	7.40E-94	348.6	RPSD		J	One of the primary rRNA binding proteins, it binds directly to 16S
Cladopus_026710	6.30E-77	292.4	UBIX	OPHBDC	H	aromatic acid decarboxylase
Cladopus_026727	2.80E-18	95.9	NAD1		C	NADH dehydrogenase subunit 1
Cladopus_026739	1.10E-14	84.3	PSAJ		D	May help in the organization of the PsaE and PsaF subunits (By
Cladopus_026740	1.40E-36	157.1	PSBE		C	This b-type cytochrome is tightly associated with the reaction center
Cladopus_026747	1.40E-36	157.1	PSBE		C	This b-type cytochrome is tightly associated with the reaction center
Cladopus_026766	1.10E-193	681	PSBD		C	One of the two reaction center proteins of photosystem II (PSII), D2
Cladopus_026778	5.00E-227	792.3	NUON	NADH5,NDH	C	electron donor, via FMN and iron-sulfur (Fe-S) centers, to quinones in
Cladopus_026808	7.40E-142	509.2	COABC	PPCDC,PPN	H	Phosphopantothenoylcysteine decarboxylase
Cladopus_026809	3.30E-85	320.1			T	Two component transcriptional regulator (Winged helix family
Cladopus_026894	2.60E-250	870.2	PHET		J	phenylalanyl-tRNA synthetase beta subunit
Cladopus_026896	9.90E-109	399.8	RPIA	RPI	G	Phosphoriboisomerase A
Cladopus_026905	9.10E-101	371.7	RPIA	RPI	G	Phosphoriboisomerase A
Cladopus_026928	2.10E-182	644	FUMC	FUM	C	fumarate hydratase, class II
Cladopus_026965	8.30E-68	261.9	RPSD		J	One of the primary rRNA binding proteins, it binds directly to 16S
Cladopus_027017	1.10E-102	380.2	RPIA	RPI	G	Phosphoriboisomerase A
Cladopus_027094	6.60E-86	322.4			T	Two component transcriptional regulator (Winged helix family
Cladopus_027098	1.60E-191	675.6	FUMC	FUM	C	fumarate hydratase, class II
Cladopus_027188	2.90E-85	320.9	QCRB	CBFC2pp,C	C	Component of the cytochrome b6-f complex, which mediates electron
Cladopus_027201	4.60E-167	592.8	FUMC	FUM	C	fumarate hydratase, class II
Cladopus_027218	9.10E-210	734.6	NDHH	NDH1_1p,N	C	electron donor, via FMN and iron-sulfur (Fe-S) centers, to quinones in
Cladopus_027244	1.60E-56	224.6	ND2		C	Core subunit of the mitochondrial membrane respiratory chain NADH
Cladopus_027246	1.60E-18	96.7	NAD1		C	NADH dehydrogenase subunit 1
Cladopus_027261	2.90E-55	219.9	ND2		C	Core subunit of the mitochondrial membrane respiratory chain NADH
