# Supplementary material for: A high-quality genome provides insights into the new taxonomic status and genomic characteristics of Cladopus chinensis (Podostemaceae)
Source: Hortic Res. 2020 Apr 1;7:46. doi: 10.1038/s41438-020-0269-5 (PMC7109043; doi:10.1038/s41438-020-0269-5)
Supplement: Supplementary file 12 — Table S16 GO enrichment of the positively genes identified in the C. chinensis [file 41438_2020_269_MOESM12_ESM.pdf]

ID	ID	Description	Count	GeneRatio	GO_Class
GO:0000003	GO:0000003	reproduction	26	26/413	Biological
GO:0008152	GO:0008152	metabolic	199	199/413	Biological
GO:0001906	GO:0001906	cell killing	0	0/413	Biological
GO:0002376	GO:0002376	immune system	1	1/413	Biological
GO:0006791	GO:0006791	sulfur	0	0/413	Biological
GO:0006794	GO:0006794	phosphorus	0	0/413	Biological
GO:0040007	GO:0040007	growth	83	83/413	Biological
GO:0007610	GO:0007610	behavior	0	0/413	Biological
GO:0008283	GO:0008283	cell	2	2/413	Biological
GO:0009758	GO:0009758	carbohydrate	0	0/413	Biological
GO:0009987	GO:0009987	cellular	211	211/413	Biological
GO:0015976	GO:0015976	carbon	0	0/413	Biological
GO:0019740	GO:0019740	nitrogen	0	0/413	Biological
GO:0022414	GO:0022414	reproductive	23	23/413	Biological
GO:0022610	GO:0022610	biological	2	2/413	Biological
GO:0023052	GO:0023052	signaling	11	11/413	Biological
GO:0032501	GO:0032501	multicellular	30	30/413	Biological
GO:0032502	GO:0032502	developmental	45	45/413	Biological
GO:0040011	GO:0040011	locomotion	2	2/413	Biological
GO:0043473	GO:0043473	pigmentation	0	0/413	Biological
GO:0044848	GO:0044848	biological	0	0/413	Biological
GO:0048511	GO:0048511	rhythmic	3	3/413	Biological
GO:0048518	GO:0048518	positive	12	12/413	Biological
GO:0048519	GO:0048519	negative	12	12/413	Biological
GO:0050789	GO:0050789	regulation of	57	57/413	Biological
GO:0050896	GO:0050896	response to	64	64/413	Biological
GO:0051179	GO:0051179	localization	29	29/413	Biological
GO:0051704	GO:0051704	multi-	13	13/413	Biological
GO:0065007	GO:0065007	biological	61	61/413	Biological
GO:0071840	GO:0071840	cellular	28	28/413	Biological
GO:0098743	GO:0098743	cell	0	0/413	Biological
GO:0098754	GO:0098754	detoxificatio	0	0/413	Biological
GO:0016020	GO:0016020	membrane	187	187/413	Cellular
GO:0005576	GO:0005576	extracellular	16	16/413	Cellular
GO:0005623	GO:0005623	cell	260	260/413	Cellular
GO:0009295	GO:0009295	nucleoid	2	2/413	Cellular
GO:0019012	GO:0019012	virion	0	0/413	Cellular
GO:0030054	GO:0030054	cell junction	11	11/413	Cellular
GO:0031974	GO:0031974	membrane-	8	8/413	Cellular
GO:0032991	GO:0032991	protein-	40	40/413	Cellular
GO:0043226	GO:0043226	organelle	106	106/413	Cellular
GO:0044215	GO:0044215	other	0	0/413	Cellular
GO:0044217	GO:0044217	other	0	0/413	Cellular
GO:0044421	GO:0044421	extracellular	1	1/413	Cellular
GO:0044422	GO:0044422	organelle	62	62/413	Cellular
GO:0044423	GO:0044423	virion part	0	0/413	Cellular
GO:0044425	GO:0044425	membrane part	30	30/413	Cellular
GO:0044456	GO:0044456	synapse part	0	0/413	Cellular
GO:0044464	GO:0044464	cell part	260	260/413	Cellular
GO:0045202	GO:0045202	synapse	0	0/413	Cellular
GO:0055044	GO:0055044	symplast	11	11/413	Cellular
GO:0097423	GO:0097423	mitochondrion	0	0/413	Cellular

GO:0099080	GO:0099080	supramolecula	0	0/413	Cellular
GO:0140110	GO:0140110	transcription	27	27/413	Molecular
GO:0003824	GO:0003824	catalytic	109	109/413	Molecular
GO:0038024	GO:0038024	cargo	0	0/413	Molecular
GO:0045735	GO:0045735	nutrient	0	0/413	Molecular
GO:0005198	GO:0005198	structural	36	36/413	Molecular
GO:0005215	GO:0005215	transporter	14	14/413	Molecular
GO:0005488	GO:0005488	binding	54	54/413	Molecular
GO:0031386	GO:0031386	protein tag	0	0/413	Molecular
GO:0090729	GO:0090729	toxin	0	0/413	Molecular
GO:0016209	GO:0016209	antioxidant	0	0/413	Molecular
GO:0098772	GO:0098772	molecular	3	3/413	Molecular
GO:0045182	GO:0045182	translation	2	2/413	Molecular
GO:0060089	GO:0060089	molecular	3	3/413	Molecular
GO:0104005	GO:0104005	hijacked	0	0/413	Molecular
GO:0140104	GO:0140104	molecular	0	0/413	Molecular
