# Supplementary material for: A high-quality genome provides insights into the new taxonomic status and genomic characteristics of Cladopus chinensis (Podostemaceae)
Source: Hortic Res. 2020 Apr 1;7:46. doi: 10.1038/s41438-020-0269-5 (PMC7109043; doi:10.1038/s41438-020-0269-5)
Supplement: Supplementary file 13 — Table S16 GO enrichment of the positively genes identified in the C. chinensis [file 41438_2020_269_MOESM13_ESM.pdf]

GID	Ko	Pathway	Pathway_Name	Pathway_Class	Pathway_Subclass
Cladopus_013411	K12821	ko03040	Spliceosome	Genetic Informatio	Transcription
Cladopus_007408	K11251	ko04217	Necroptosis	Cellular Processes	Cell growth and death
Cladopus_007408	K11251	ko05322	Systemic lupus ery	Human Diseases	Immune diseases
Cladopus_007408	K11251	ko05034	Alcoholism	Human Diseases	Substance dependence
Cladopus_006596	K03083	ko04012	ErbB signaling pat	Environmental Info	Signal transduction
Cladopus_006596	K03083	ko04310	Wnt signaling path	Environmental Info	Signal transduction
Cladopus_006596	K03083	ko04340	Hedgehog signaling	Environmental Info	Signal transduction
Cladopus_006596	K03083	ko04341	Hedgehog signaling	Environmental Info	Signal transduction
Cladopus_006596	K03083	ko04390	Hippo signaling pa	Environmental Info	Signal transduction
Cladopus_006596	K03083	ko04151	PI3K-Akt signaling	Environmental Info	Signal transduction
Cladopus_006596	K03083	ko04150	mTOR signaling pat	Environmental Info	Signal transduction
Cladopus_006596	K03083	ko04110	Cell cycle	Cellular Processes	Cell growth and death
Cladopus_006596	K03083	ko04510	Focal adhesion	Cellular Processes	Cellular community - eukaryotes
Cladopus_006596	K03083	ko04550	Signaling pathways	Cellular Processes	Cellular community - eukaryotes
Cladopus_006596	K03083	ko04660	T cell receptor si	Organismal Systems	Immune system
Cladopus_006596	K03083	ko04657	IL-17 signaling pa	Organismal Systems	Immune system
Cladopus_006596	K03083	ko04662	B cell receptor si	Organismal Systems	Immune system
Cladopus_006596	K03083	ko04062	Chemokine signalin	Organismal Systems	Immune system
Cladopus_006596	K03083	ko04910	Insulin signaling	Organismal Systems	Endocrine system
Cladopus_006596	K03083	ko04917	Prolactin signalin	Organismal Systems	Endocrine system
Cladopus_006596	K03083	ko04919	Thyroid hormone si	Organismal Systems	Endocrine system
Cladopus_006596	K03083	ko04916	Melanogenesis	Organismal Systems	Endocrine system
Cladopus_006596	K03083	ko04728	Dopaminergic synap	Organismal Systems	Nervous system
Cladopus_006596	K03083	ko04722	Neurotrophin signa	Organismal Systems	Nervous system
Cladopus_006596	K03083	ko04360	Axon guidance	Organismal Systems	Development
Cladopus_006596	K03083	ko04711	Circadian rhythm -	Organismal Systems	Environmental adaptation
Cladopus_006596	K03083	ko05200	Pathways in cancer	Human Diseases	Cancers: Overview
Cladopus_006596	K03083	ko05210	Colorectal cancer	Human Diseases	Cancers: Specific types
Cladopus_006596	K03083	ko05225	Hepatocellular car	Human Diseases	Cancers: Specific types

Cladopus_006596	K03083	ko05226	Gastric cancer	Human Diseases	Cancers: Specific types
Cladopus_006596	K03083	ko05217	Basal cell carcino	Human Diseases	Cancers: Specific types
Cladopus_006596	K03083	ko05215	Prostate cancer	Human Diseases	Cancers: Specific types
Cladopus_006596	K03083	ko05213	Endometrial cancer	Human Diseases	Cancers: Specific types
Cladopus_006596	K03083	ko05224	Breast cancer	Human Diseases	Cancers: Specific types
Cladopus_006596	K03083	ko05010	Alzheimer disease	Human Diseases	Neurodegenerative diseases
Cladopus_006596	K03083	ko04932	Non-alcoholic fatt	Human Diseases	Endocrine and metabolic diseases
Cladopus_006596	K03083	ko04931	Insulin resistance	Human Diseases	Endocrine and metabolic diseases
Cladopus_006596	K03083	ko04934	Cushing syndrome	Human Diseases	Endocrine and metabolic diseases
Cladopus_006596	K03083	ko05162	Measles	Human Diseases	Infectious diseases: Viral
Cladopus_006596	K03083	ko05164	Influenza A	Human Diseases	Infectious diseases: Viral
Cladopus_006596	K03083	ko05160	Hepatitis C	Human Diseases	Infectious diseases: Viral
Cladopus_006596	K03083	ko05163	Human cytomegalovi	Human Diseases	Infectious diseases: Viral
Cladopus_006596	K03083	ko05167	Kaposi sarcoma-ass	Human Diseases	Infectious diseases: Viral
Cladopus_006596	K03083	ko05165	Human papillomavir	Human Diseases	Infectious diseases: Viral
Cladopus_006596	K03083	ko01521	EGFR tyrosine kina	Human Diseases	Drug resistance: Antineoplastic
Cladopus_006642	K01213	ko00040	Pentose and glucur	Metabolism	Carbohydrate metabolism
Cladopus_025285	K00514	ko00906	Carotenoid biosynt	Metabolism	Metabolism of terpenoids and polyketides
Cladopus_025285	K02293	ko00906	Carotenoid biosynt	Metabolism	Metabolism of terpenoids and polyketides
Cladopus_010276	K11644	ko04139	Mitophagy - yeast	Cellular Processes	Transport and catabolism
Cladopus_010276	K11644	ko04919	Thyroid hormone si	Organismal Systems	Endocrine system
Cladopus_010276	K11644	ko05202	Transcriptional mi	Human Diseases	Cancers: Overview
Cladopus_010276	K11644	ko05016	Huntington disease	Human Diseases	Neurodegenerative diseases
Cladopus_010276	K11644	ko05169	Epstein-Barr virus	Human Diseases	Infectious diseases: Viral
Cladopus_005675	K08917	ko00196	Photosynthesis - a	Metabolism	Energy metabolism
Cladopus_005731	K05284	ko00563	Glycosylphosphatid	Metabolism	Glycan biosynthesis and metabolism
Cladopus_005743	K14488	ko04075	Plant hormone sign	Environmental Info	Signal transduction
Cladopus_005757	K03767	ko04217	Necroptosis	Cellular Processes	Cell growth and death
Cladopus_005757	K03767	ko01503	Cationic antimicro	Human Diseases	Drug resistance: Antimicrobial
Cladopus_005774	K01803	ko00010	Glycolysis / Gluco	Metabolism	Carbohydrate metabolism

Cladopus_005774	K01803	ko00051	Fructose and manno	Metabolism	Carbohydrate metabolism
Cladopus_005774	K01803	ko00562	Inositol phosphate	Metabolism	Carbohydrate metabolism
Cladopus_005774	K01803	ko00710	Carbon fixation in	Metabolism	Energy metabolism
Cladopus_005788	K06966	ko00230	Purine metabolism	Metabolism	Nucleotide metabolism
Cladopus_005788	K06966	ko00240	Pyrimidine metabol	Metabolism	Nucleotide metabolism
Cladopus_005795	K13412	ko04626	Plant-pathogen int	Organismal Systems	Environmental adaptation
Cladopus_005795	K13412	ko05145	Toxoplasmosis	Human Diseases	Infectious diseases: Parasitic
Cladopus_024276	K00794	ko00740	Riboflavin metabol	Metabolism	Metabolism of cofactors and vitamins
Cladopus_021526	K14575	ko03008	Ribosome biogenesi	Genetic Informatio	Translation
Cladopus_002827	K09840	ko00906	Carotenoid biosynt	Metabolism	Metabolism of terpenoids and polyketides
Cladopus_002926	K00218	ko00860	Porphyrin and chlo	Metabolism	Metabolism of cofactors and vitamins
Cladopus_005242	K00430	ko00940	Phenylpropanoid bi	Metabolism	Biosynthesis of other secondary metabolites
Cladopus_003101	K01580	ko00650	Butanoate metaboli	Metabolism	Carbohydrate metabolism
Cladopus_003101	K01580	ko00250	Alanine, aspartate	Metabolism	Amino acid metabolism
Cladopus_003101	K01580	ko00410	beta-Alanine metab	Metabolism	Metabolism of other amino acids
Cladopus_003101	K01580	ko00430	Taurine and hypota	Metabolism	Metabolism of other amino acids
Cladopus_003101	K01580	ko02024	Quorum sensing	Cellular Processes	Cellular community - prokaryotes
Cladopus_003101	K01580	ko04727	GABAergic synapse	Organismal Systems	Nervous system
Cladopus_003101	K01580	ko04940	Type I diabetes me	Human Diseases	Endocrine and metabolic diseases
Cladopus_020806	K00003	ko00260	Glycine, serine an	Metabolism	Amino acid metabolism
Cladopus_020806	K00003	ko00270	Cysteine and methi	Metabolism	Amino acid metabolism
Cladopus_020806	K00003	ko00300	Lysine biosynthesi	Metabolism	Amino acid metabolism
Cladopus_020806	K12524	ko00260	Glycine, serine an	Metabolism	Amino acid metabolism
Cladopus_020806	K12524	ko00270	Cysteine and methi	Metabolism	Amino acid metabolism
Cladopus_020806	K12524	ko00300	Lysine biosynthesi	Metabolism	Amino acid metabolism
Cladopus_020806	K12524	ko00261	Monobactam biosynt	Metabolism	Biosynthesis of other secondary metabolites
Cladopus_020816	K07657	ko02020	Two-component syst	Environmental Info	Signal transduction
Cladopus_020816	K07658	ko02020	Two-component syst	Environmental Info	Signal transduction
Cladopus_020816	K07660	ko02020	Two-component syst	Environmental Info	Signal transduction
Cladopus_020816	K07660	ko01503	Cationic antimicro	Human Diseases	Drug resistance: Antimicrobial

Cladopus_020816	K07665	ko02020	Two-component syst	Environmental Info	Signal transduction
Cladopus_020816	K07666	ko02020	Two-component syst	Environmental Info	Signal transduction
Cladopus_020816	K07666	ko02024	Quorum sensing	Cellular Processes	Cellular community - prokaryotes
Cladopus_020816	K07771	ko02020	Two-component syst	Environmental Info	Signal transduction
Cladopus_020816	K07771	ko01503	Cationic antimicro	Human Diseases	Drug resistance: Antimicrobial
Cladopus_020816	K07774	ko02020	Two-component syst	Environmental Info	Signal transduction
Cladopus_020816	K14983	ko02020	Two-component syst	Environmental Info	Signal transduction
Cladopus_020816	K14983	ko02024	Quorum sensing	Cellular Processes	Cellular community - prokaryotes
Cladopus_020810	K03404	ko00860	Porphyrin and chlo	Metabolism	Metabolism of cofactors and vitamins
Cladopus_020809	K02706	ko00195	Photosynthesis	Metabolism	Energy metabolism
Cladopus_020809	K08929	ko02020	Two-component syst	Environmental Info	Signal transduction
Cladopus_020843	K01515	ko00230	Purine metabolism	Metabolism	Nucleotide metabolism
Cladopus_020861	K00805	ko00900	Terpenoid backbone	Metabolism	Metabolism of terpenoids and polyketides
Cladopus_020861	K02523	ko00900	Terpenoid backbone	Metabolism	Metabolism of terpenoids and polyketides
Cladopus_020861	K05356	ko00900	Terpenoid backbone	Metabolism	Metabolism of terpenoids and polyketides
Cladopus_020862	K03100	ko03060	Protein export	Genetic Informatio	Folding, sorting and degradation
Cladopus_020862	K03100	ko02024	Quorum sensing	Cellular Processes	Cellular community - prokaryotes
Cladopus_020860	K00855	ko00710	Carbon fixation in	Metabolism	Energy metabolism
Cladopus_020875	K00228	ko00860	Porphyrin and chlo	Metabolism	Metabolism of cofactors and vitamins
Cladopus_020894	K01696	ko00260	Glycine, serine an	Metabolism	Amino acid metabolism
Cladopus_020894	K01696	ko00400	Phenylalanine, tyr	Metabolism	Amino acid metabolism
Cladopus_021260	K13963	ko05146	Amoebiasis	Human Diseases	Infectious diseases: Parasitic
Cladopus_022236	K09811	ko02010	ABC transporters	Environmental Info	Membrane transport
Cladopus_022236	K09812	ko02010	ABC transporters	Environmental Info	Membrane transport
Cladopus_022249	K02536	ko00540	Lipopolysaccharide	Metabolism	Glycan biosynthesis and metabolism
Cladopus_022248	K00161	ko00010	Glycolysis / Gluco	Metabolism	Carbohydrate metabolism
Cladopus_022248	K00161	ko00020	Citrate cycle (TCA	Metabolism	Carbohydrate metabolism
Cladopus_022248	K00161	ko00620	Pyruvate metabolis	Metabolism	Carbohydrate metabolism
Cladopus_022248	K00161	ko04066	HIF-1 signaling pa	Environmental Info	Signal transduction
Cladopus_022248	K00161	ko04922	Glucagon signaling	Organismal Systems	Endocrine system

Cladopus_022248	K00161	ko05230	Central carbon met	Human Diseases	Cancers: Overview
Cladopus_022260	K02888	ko03010	Ribosome	Genetic Informatio	Translation
Cladopus_022254	K00036	ko00030	Pentose phosphate	Metabolism	Carbohydrate metabolism
Cladopus_022254	K00036	ko00480	Glutathione metabo	Metabolism	Metabolism of other amino acids
Cladopus_022254	K00036	ko05230	Central carbon met	Human Diseases	Cancers: Overview
Cladopus_022255	K03841	ko00010	Glycolysis / Gluco	Metabolism	Carbohydrate metabolism
Cladopus_022255	K03841	ko00030	Pentose phosphate	Metabolism	Carbohydrate metabolism
Cladopus_022255	K03841	ko00051	Fructose and manno	Metabolism	Carbohydrate metabolism
Cladopus_022255	K03841	ko00710	Carbon fixation in	Metabolism	Energy metabolism
Cladopus_022255	K03841	ko00680	Methane metabolism	Metabolism	Energy metabolism
Cladopus_022255	K03841	ko04152	AMPK signaling pat	Environmental Info	Signal transduction
Cladopus_022255	K03841	ko04910	Insulin signaling	Organismal Systems	Endocrine system
Cladopus_022276	K01251	ko00270	Cysteine and methi	Metabolism	Amino acid metabolism
Cladopus_022272	K01710	ko00523	Polyketide sugar u	Metabolism	Metabolism of terpenoids and polyketides
Cladopus_022272	K01710	ko01055	Biosynthesis of va	Metabolism	Metabolism of terpenoids and polyketides
Cladopus_022272	K01710	ko00521	Streptomycin biosy	Metabolism	Biosynthesis of other secondary metabolites
Cladopus_022272	K01710	ko00525	Acarbose and valid	Metabolism	Biosynthesis of other secondary metabolites
Cladopus_022272	K08678	ko00520	Amino sugar and nu	Metabolism	Carbohydrate metabolism
Cladopus_022273	K00012	ko00040	Pentose and glucur	Metabolism	Carbohydrate metabolism
Cladopus_022273	K00012	ko00053	Ascorbate and alda	Metabolism	Carbohydrate metabolism
Cladopus_022273	K00012	ko00520	Amino sugar and nu	Metabolism	Carbohydrate metabolism
Cladopus_022265	K09835	ko00906	Carotenoid biosynt	Metabolism	Metabolism of terpenoids and polyketides
Cladopus_022283	K00366	ko00910	Nitrogen metabolis	Metabolism	Energy metabolism
Cladopus_022294	K00703	ko00500	Starch and sucrose	Metabolism	Carbohydrate metabolism
Cladopus_022294	K00703	ko02026	Biofilm formation	Cellular Processes	Cellular community - prokaryotes
Cladopus_022294	K01176	ko00500	Starch and sucrose	Metabolism	Carbohydrate metabolism
Cladopus_022294	K01176	ko04973	Carbohydrate diges	Organismal Systems	Digestive system
Cladopus_022319	K00831	ko00680	Methane metabolism	Metabolism	Energy metabolism
Cladopus_022319	K00831	ko00260	Glycine, serine an	Metabolism	Amino acid metabolism
Cladopus_022319	K00831	ko00750	Vitamin B6 metabol	Metabolism	Metabolism of cofactors and vitamins

Cladopus_022330	K01703	ko00660	C5-Branched dibasi	Metabolism	Carbohydrate metabolism
Cladopus_022330	K01703	ko00290	Valine, leucine an	Metabolism	Amino acid metabolism
Cladopus_022330	K01703	ko00966	Glucosinolate bios	Metabolism	Biosynthesis of other secondary metabolites
Cladopus_026337	K02879	ko03010	Ribosome	Genetic Informatio	Translation
Cladopus_026344	K02982	ko03010	Ribosome	Genetic Informatio	Translation
Cladopus_026340	K02933	ko03010	Ribosome	Genetic Informatio	Translation
Cladopus_026339	K03076	ko03060	Protein export	Genetic Informatio	Folding, sorting and degradation
Cladopus_026339	K03076	ko03070	Bacterial secretio	Environmental Info	Membrane transport
Cladopus_026339	K03076	ko02024	Quorum sensing	Cellular Processes	Cellular community - prokaryotes
Cladopus_026342	K02874	ko03010	Ribosome	Genetic Informatio	Translation
Cladopus_026338	K02948	ko03010	Ribosome	Genetic Informatio	Translation
Cladopus_026346	K00259	ko00250	Alanine, aspartate	Metabolism	Amino acid metabolism
Cladopus_026346	K00259	ko00430	Taurine and hypota	Metabolism	Metabolism of other amino acids
Cladopus_026343	K02878	ko03010	Ribosome	Genetic Informatio	Translation
Cladopus_004390	K08202	ko05231	Choline metabolism	Human Diseases	Cancers: Overview
Cladopus_004441	K08901	ko00195	Photosynthesis	Metabolism	Energy metabolism
Cladopus_004450	K04730	ko04010	MAPK signaling pat	Environmental Info	Signal transduction
Cladopus_004450	K04730	ko04064	NF-kappa B signali	Environmental Info	Signal transduction
Cladopus_004450	K04730	ko04620	Toll-like receptor	Organismal Systems	Immune system
Cladopus_004450	K04730	ko04624	Toll and Imd signa	Organismal Systems	Immune system
Cladopus_004450	K04730	ko04722	Neurotrophin signa	Organismal Systems	Nervous system
Cladopus_004450	K04730	ko05133	Pertussis	Human Diseases	Infectious diseases: Bacterial
Cladopus_004450	K04730	ko05152	Tuberculosis	Human Diseases	Infectious diseases: Bacterial
Cladopus_004450	K04730	ko05170	Human immunodefici	Human Diseases	Infectious diseases: Viral
Cladopus_004450	K04730	ko05162	Measles	Human Diseases	Infectious diseases: Viral
Cladopus_004450	K04730	ko05169	Epstein-Barr virus	Human Diseases	Infectious diseases: Viral
Cladopus_004450	K04730	ko05145	Toxoplasmosis	Human Diseases	Infectious diseases: Parasitic
Cladopus_004450	K04730	ko05140	Leishmaniasis	Human Diseases	Infectious diseases: Parasitic
Cladopus_004450	K04730	ko05142	Chagas disease (Am	Human Diseases	Infectious diseases: Parasitic
Cladopus_021094	K01945	ko00230	Purine metabolism	Metabolism	Nucleotide metabolism

Cladopus_021094	K01952	ko00230	Purine metabolism	Metabolism	Nucleotide metabolism
Cladopus_021079	K02871	ko03010	Ribosome	Genetic Informatio	Translation
Cladopus_021090	K02982	ko03010	Ribosome	Genetic Informatio	Translation
Cladopus_021086	K02933	ko03010	Ribosome	Genetic Informatio	Translation
Cladopus_021088	K02874	ko03010	Ribosome	Genetic Informatio	Translation
Cladopus_021091	K02886	ko03010	Ribosome	Genetic Informatio	Translation
Cladopus_021085	K02988	ko03010	Ribosome	Genetic Informatio	Translation
Cladopus_021084	K02876	ko03010	Ribosome	Genetic Informatio	Translation
Cladopus_021089	K02878	ko03010	Ribosome	Genetic Informatio	Translation
Cladopus_021092	K02926	ko03010	Ribosome	Genetic Informatio	Translation
Cladopus_021082	K02952	ko03010	Ribosome	Genetic Informatio	Translation
Cladopus_021120	K02935	ko03010	Ribosome	Genetic Informatio	Translation
Cladopus_021136	K02707	ko00195	Photosynthesis	Metabolism	Energy metabolism
Cladopus_021136	K02713	ko00195	Photosynthesis	Metabolism	Energy metabolism
Cladopus_021144	K01895	ko00010	Glycolysis / Gluco	Metabolism	Carbohydrate metabolism
Cladopus_021144	K01895	ko00620	Pyruvate metabolis	Metabolism	Carbohydrate metabolism
Cladopus_021144	K01895	ko00630	Glyoxylate and dic	Metabolism	Carbohydrate metabolism
Cladopus_021144	K01895	ko00640	Propanoate metabol	Metabolism	Carbohydrate metabolism
Cladopus_021144	K01895	ko00720	Carbon fixation pa	Metabolism	Energy metabolism
Cladopus_021144	K01895	ko00680	Methane metabolism	Metabolism	Energy metabolism
Cladopus_021148	K00806	ko00900	Terpenoid backbone	Metabolism	Metabolism of terpenoids and polyketides
Cladopus_021148	K12503	ko00900	Terpenoid backbone	Metabolism	Metabolism of terpenoids and polyketides
Cladopus_021146	K01469	ko00480	Glutathione metabo	Metabolism	Metabolism of other amino acids
Cladopus_021146	K01474	ko00330	Arginine and proli	Metabolism	Amino acid metabolism
Cladopus_021162	K02433	ko00970	Aminoacyl-tRNA bio	Genetic Informatio	Translation
Cladopus_021168	K09458	ko00061	Fatty acid biosynt	Metabolism	Lipid metabolism
Cladopus_021168	K09458	ko00780	Biotin metabolism	Metabolism	Metabolism of cofactors and vitamins
Cladopus_021164	K02704	ko00195	Photosynthesis	Metabolism	Energy metabolism
Cladopus_021177	K03671	ko04621	NOD-like receptor	Organismal Systems	Immune system
Cladopus_021177	K03671	ko05418	Fluid shear stress	Human Diseases	Cardiovascular diseases

Cladopus_021186	K01961	ko00620	Pyruvate metabolis	Metabolism	Carbohydrate metabolism
Cladopus_021186	K01961	ko00640	Propanoate metabol	Metabolism	Carbohydrate metabolism
Cladopus_021186	K01961	ko00720	Carbon fixation pa	Metabolism	Energy metabolism
Cladopus_021186	K01961	ko00061	Fatty acid biosynt	Metabolism	Lipid metabolism
Cladopus_001288	K12761	ko04138	Autophagy - yeast	Cellular Processes	Transport and catabolism
Cladopus_001288	K12761	ko04113	Meiosis - yeast	Cellular Processes	Cell growth and death
Cladopus_001297	K02976	ko03010	Ribosome	Genetic Informatio	Translation
Cladopus_001298	K02976	ko03010	Ribosome	Genetic Informatio	Translation
Cladopus_001299	K02976	ko03010	Ribosome	Genetic Informatio	Translation
Cladopus_001347	K02973	ko03010	Ribosome	Genetic Informatio	Translation
Cladopus_001368	K01087	ko00500	Starch and sucrose	Metabolism	Carbohydrate metabolism
Cladopus_001390	K04730	ko04010	MAPK signaling pat	Environmental Info	Signal transduction
Cladopus_001390	K04730	ko04064	NF-kappa B signali	Environmental Info	Signal transduction
Cladopus_001390	K04730	ko04620	Toll-like receptor	Organismal Systems	Immune system
Cladopus_001390	K04730	ko04624	Toll and Imd signa	Organismal Systems	Immune system
Cladopus_001390	K04730	ko04722	Neurotrophin signa	Organismal Systems	Nervous system
Cladopus_001390	K04730	ko05133	Pertussis	Human Diseases	Infectious diseases: Bacterial
Cladopus_001390	K04730	ko05152	Tuberculosis	Human Diseases	Infectious diseases: Bacterial
Cladopus_001390	K04730	ko05170	Human immunodefici	Human Diseases	Infectious diseases: Viral
Cladopus_001390	K04730	ko05162	Measles	Human Diseases	Infectious diseases: Viral
Cladopus_001390	K04730	ko05169	Epstein-Barr virus	Human Diseases	Infectious diseases: Viral
Cladopus_001390	K04730	ko05145	Toxoplasmosis	Human Diseases	Infectious diseases: Parasitic
Cladopus_001390	K04730	ko05140	Leishmaniasis	Human Diseases	Infectious diseases: Parasitic
Cladopus_001390	K04730	ko05142	Chagas disease (Am	Human Diseases	Infectious diseases: Parasitic
Cladopus_001395	K07198	ko04371	Apelin signaling p	Environmental Info	Signal transduction
Cladopus_001395	K07198	ko04068	FoxO signaling pat	Environmental Info	Signal transduction
Cladopus_001395	K07198	ko04151	PI3K-Akt signaling	Environmental Info	Signal transduction
Cladopus_001395	K07198	ko04152	AMPK signaling pat	Environmental Info	Signal transduction
Cladopus_001395	K07198	ko04150	mTOR signaling pat	Environmental Info	Signal transduction
Cladopus_001395	K07198	ko04140	Autophagy - animal	Cellular Processes	Transport and catabolism

Cladopus_001395	K07198	ko04530	Tight junction	Cellular Processes	Cellular community - eukaryotes
Cladopus_001395	K07198	ko04910	Insulin signaling	Organismal Systems	Endocrine system
Cladopus_001395	K07198	ko04922	Glucagon signaling	Organismal Systems	Endocrine system
Cladopus_001395	K07198	ko04920	Adipocytokine sign	Organismal Systems	Endocrine system
Cladopus_001395	K07198	ko04921	Oxytocin signaling	Organismal Systems	Endocrine system
Cladopus_001395	K07198	ko04211	Longevity regulati	Organismal Systems	Aging
Cladopus_001395	K07198	ko04213	Longevity regulati	Organismal Systems	Aging
Cladopus_001395	K07198	ko04710	Circadian rhythm	Organismal Systems	Environmental adaptation
Cladopus_001395	K07198	ko04714	Thermogenesis	Organismal Systems	Environmental adaptation
Cladopus_001395	K07198	ko05418	Fluid shear stress	Human Diseases	Cardiovascular diseases
Cladopus_001395	K07198	ko05410	Hypertrophic cardi	Human Diseases	Cardiovascular diseases
Cladopus_001395	K07198	ko04932	Non-alcoholic fatt	Human Diseases	Endocrine and metabolic diseases
Cladopus_001395	K07198	ko04931	Insulin resistance	Human Diseases	Endocrine and metabolic diseases
Cladopus_023734	K10255	ko02020	Two-component syst	Environmental Info	Signal transduction
Cladopus_023746	K00602	ko00230	Purine metabolism	Metabolism	Nucleotide metabolism
Cladopus_023746	K00602	ko00670	One carbon pool by	Metabolism	Metabolism of cofactors and vitamins
Cladopus_023746	K00602	ko01523	Antifolate resista	Human Diseases	Drug resistance: Antineoplastic
Cladopus_023786	K00981	ko00564	Glycerophospholipi	Metabolism	Lipid metabolism
Cladopus_023786	K00981	ko04070	Phosphatidylinosit	Environmental Info	Signal transduction
Cladopus_023798	K02660	ko02020	Two-component syst	Environmental Info	Signal transduction
Cladopus_023798	K02660	ko02025	Biofilm formation	Cellular Processes	Cellular community - prokaryotes
Cladopus_023798	K03406	ko02020	Two-component syst	Environmental Info	Signal transduction
Cladopus_023798	K03406	ko02030	Bacterial chemotax	Cellular Processes	Cell motility
Cladopus_023798	K11525	ko02020	Two-component syst	Environmental Info	Signal transduction
Cladopus_023798	K13487	ko02020	Two-component syst	Environmental Info	Signal transduction
Cladopus_023798	K13487	ko02025	Biofilm formation	Cellular Processes	Cellular community - prokaryotes
Cladopus_002228	K19613	ko04014	Ras signaling path	Environmental Info	Signal transduction
Cladopus_019615	K03404	ko00860	Porphyrin and chlo	Metabolism	Metabolism of cofactors and vitamins
Cladopus_019614	K00343	ko00190	Oxidative phosphor	Metabolism	Energy metabolism
Cladopus_019614	K05573	ko00190	Oxidative phosphor	Metabolism	Energy metabolism

Cladopus_019644	K03644	ko00785	Lipoic acid metabo	Metabolism	Metabolism of cofactors and vitamins
Cladopus_019653	K01695	ko00260	Glycine, serine an	Metabolism	Amino acid metabolism
Cladopus_019653	K01695	ko00400	Phenylalanine, tyr	Metabolism	Amino acid metabolism
Cladopus_019658	K00034	ko00030	Pentose phosphate	Metabolism	Carbohydrate metabolism
Cladopus_019658	K00059	ko00061	Fatty acid biosynt	Metabolism	Lipid metabolism
Cladopus_019658	K00059	ko00780	Biotin metabolism	Metabolism	Metabolism of cofactors and vitamins
Cladopus_019658	K00059	ko00333	Prodigiosin biosyn	Metabolism	Biosynthesis of other secondary metabolites
Cladopus_019674	K00278	ko00250	Alanine, aspartate	Metabolism	Amino acid metabolism
Cladopus_019674	K00278	ko00760	Nicotinate and nic	Metabolism	Metabolism of cofactors and vitamins
Cladopus_019687	K02492	ko00860	Porphyrin and chlo	Metabolism	Metabolism of cofactors and vitamins
Cladopus_019693	K01749	ko00860	Porphyrin and chlo	Metabolism	Metabolism of cofactors and vitamins
Cladopus_019690	K02045	ko00920	Sulfur metabolism	Metabolism	Energy metabolism
Cladopus_019690	K02045	ko02010	ABC transporters	Environmental Info	Membrane transport
Cladopus_019711	K00162	ko00010	Glycolysis / Gluco	Metabolism	Carbohydrate metabolism
Cladopus_019711	K00162	ko00020	Citrate cycle (TCA	Metabolism	Carbohydrate metabolism
Cladopus_019711	K00162	ko00620	Pyruvate metabolis	Metabolism	Carbohydrate metabolism
Cladopus_019711	K00162	ko04066	HIF-1 signaling pa	Environmental Info	Signal transduction
Cladopus_019711	K00162	ko04922	Glucagon signaling	Organismal Systems	Endocrine system
Cladopus_019711	K00162	ko05230	Central carbon met	Human Diseases	Cancers: Overview
Cladopus_019711	K11381	ko00640	Propanoate metabol	Metabolism	Carbohydrate metabolism
Cladopus_019711	K11381	ko00280	Valine, leucine an	Metabolism	Amino acid metabolism
Cladopus_010511	K02976	ko03010	Ribosome	Genetic Informatio	Translation
Cladopus_010516	K12761	ko04138	Autophagy - yeast	Cellular Processes	Transport and catabolism
Cladopus_010516	K12761	ko04113	Meiosis - yeast	Cellular Processes	Cell growth and death
Cladopus_007689	K02868	ko03010	Ribosome	Genetic Informatio	Translation
Cladopus_005099	K00850	ko00010	Glycolysis / Gluco	Metabolism	Carbohydrate metabolism
Cladopus_005099	K00850	ko00030	Pentose phosphate	Metabolism	Carbohydrate metabolism
Cladopus_005099	K00850	ko00051	Fructose and manno	Metabolism	Carbohydrate metabolism
Cladopus_005099	K00850	ko00052	Galactose metaboli	Metabolism	Carbohydrate metabolism
Cladopus_005099	K00850	ko00680	Methane metabolism	Metabolism	Energy metabolism

Cladopus_005099	K00850	ko03018	RNA degradation	Genetic Informatio	Folding, sorting and degradation
Cladopus_005099	K00850	ko04152	AMPK signaling pat	Environmental Info	Signal transduction
Cladopus_005099	K00850	ko05230	Central carbon met	Human Diseases	Cancers: Overview
Cladopus_005137	K16241	ko04712	Circadian rhythm -	Organismal Systems	Environmental adaptation
Cladopus_005147	K14515	ko04016	MAPK signaling pat	Environmental Info	Signal transduction
Cladopus_005147	K14515	ko04075	Plant hormone sign	Environmental Info	Signal transduction
Cladopus_009405	K13412	ko04626	Plant-pathogen int	Organismal Systems	Environmental adaptation
Cladopus_009405	K13412	ko05145	Toxoplasmosis	Human Diseases	Infectious diseases: Parasitic
Cladopus_009419	K06966	ko00230	Purine metabolism	Metabolism	Nucleotide metabolism
Cladopus_009419	K06966	ko00240	Pyrimidine metabol	Metabolism	Nucleotide metabolism
Cladopus_009451	K12741	ko03040	Spliceosome	Genetic Informatio	Transcription
Cladopus_009451	K14411	ko03015	mRNA surveillance	Genetic Informatio	Translation
Cladopus_009473	K14488	ko04075	Plant hormone sign	Environmental Info	Signal transduction
Cladopus_025829	K00854	ko00040	Pentose and glucur	Metabolism	Carbohydrate metabolism
Cladopus_025835	K01695	ko00260	Glycine, serine an	Metabolism	Amino acid metabolism
Cladopus_025835	K01695	ko00400	Phenylalanine, tyr	Metabolism	Amino acid metabolism
Cladopus_025835	K01962	ko00620	Pyruvate metabolis	Metabolism	Carbohydrate metabolism
Cladopus_025835	K01962	ko00640	Propanoate metabol	Metabolism	Carbohydrate metabolism
Cladopus_025835	K01962	ko00720	Carbon fixation pa	Metabolism	Energy metabolism
Cladopus_025835	K01962	ko00061	Fatty acid biosynt	Metabolism	Lipid metabolism
Cladopus_025835	K01963	ko00620	Pyruvate metabolis	Metabolism	Carbohydrate metabolism
Cladopus_025835	K01963	ko00640	Propanoate metabol	Metabolism	Carbohydrate metabolism
Cladopus_025835	K01963	ko00720	Carbon fixation pa	Metabolism	Energy metabolism
Cladopus_025835	K01963	ko00061	Fatty acid biosynt	Metabolism	Lipid metabolism
Cladopus_025838	K00962	ko00230	Purine metabolism	Metabolism	Nucleotide metabolism
Cladopus_025838	K00962	ko00240	Pyrimidine metabol	Metabolism	Nucleotide metabolism
Cladopus_025838	K00962	ko03018	RNA degradation	Genetic Informatio	Folding, sorting and degradation
Cladopus_025831	K05371	ko00860	Porphyrin and chlo	Metabolism	Metabolism of cofactors and vitamins
Cladopus_025834	K00381	ko00920	Sulfur metabolism	Metabolism	Energy metabolism
Cladopus_025834	K00392	ko00920	Sulfur metabolism	Metabolism	Energy metabolism

Cladopus_025836	K00820	ko00520	Amino sugar and nu	Metabolism	Carbohydrate metabolism
Cladopus_025836	K00820	ko00250	Alanine, aspartate	Metabolism	Amino acid metabolism
Cladopus_025836	K00820	ko04931	Insulin resistance	Human Diseases	Endocrine and metabolic diseases
Cladopus_020619	K00812	ko00250	Alanine, aspartate	Metabolism	Amino acid metabolism
Cladopus_020619	K00812	ko00270	Cysteine and methi	Metabolism	Amino acid metabolism
Cladopus_020619	K00812	ko00220	Arginine biosynthe	Metabolism	Amino acid metabolism
Cladopus_020619	K00812	ko00330	Arginine and proli	Metabolism	Amino acid metabolism
Cladopus_020619	K00812	ko00350	Tyrosine metabolis	Metabolism	Amino acid metabolism
Cladopus_020619	K00812	ko00360	Phenylalanine meta	Metabolism	Amino acid metabolism
Cladopus_020619	K00812	ko00400	Phenylalanine, tyr	Metabolism	Amino acid metabolism
Cladopus_020619	K00812	ko00950	Isoquinoline alkal	Metabolism	Biosynthesis of other secondary metabolites
Cladopus_020619	K00812	ko00960	Tropane, piperidin	Metabolism	Biosynthesis of other secondary metabolites
Cladopus_020619	K00812	ko00401	Novobiocin biosynt	Metabolism	Biosynthesis of other secondary metabolites
Cladopus_020624	K00135	ko00650	Butanoate metaboli	Metabolism	Carbohydrate metabolism
Cladopus_020624	K00135	ko00250	Alanine, aspartate	Metabolism	Amino acid metabolism
Cladopus_020624	K00135	ko00310	Lysine degradation	Metabolism	Amino acid metabolism
Cladopus_020624	K00135	ko00350	Tyrosine metabolis	Metabolism	Amino acid metabolism
Cladopus_020624	K00135	ko00760	Nicotinate and nic	Metabolism	Metabolism of cofactors and vitamins
Cladopus_020624	K08324	ko00650	Butanoate metaboli	Metabolism	Carbohydrate metabolism
Cladopus_020624	K08324	ko00250	Alanine, aspartate	Metabolism	Amino acid metabolism
Cladopus_020624	K08324	ko00760	Nicotinate and nic	Metabolism	Metabolism of cofactors and vitamins
Cladopus_020638	K00805	ko00900	Terpenoid backbone	Metabolism	Metabolism of terpenoids and polyketides
Cladopus_020638	K02523	ko00900	Terpenoid backbone	Metabolism	Metabolism of terpenoids and polyketides
Cladopus_020638	K05356	ko00900	Terpenoid backbone	Metabolism	Metabolism of terpenoids and polyketides
Cladopus_020637	K03404	ko00860	Porphyrin and chlo	Metabolism	Metabolism of cofactors and vitamins
Cladopus_020637	K03405	ko00860	Porphyrin and chlo	Metabolism	Metabolism of cofactors and vitamins
Cladopus_020628	K00059	ko00061	Fatty acid biosynt	Metabolism	Lipid metabolism
Cladopus_020628	K00059	ko00780	Biotin metabolism	Metabolism	Metabolism of cofactors and vitamins
Cladopus_020628	K00059	ko00333	Prodigiosin biosyn	Metabolism	Biosynthesis of other secondary metabolites
Cladopus_020671	K03575	ko03410	Base excision repa	Genetic Informatio	Replication and repair

Cladopus_009175	K00850	ko00010	Glycolysis / Gluco	Metabolism	Carbohydrate metabolism
Cladopus_009175	K00850	ko00030	Pentose phosphate	Metabolism	Carbohydrate metabolism
Cladopus_009175	K00850	ko00051	Fructose and manno	Metabolism	Carbohydrate metabolism
Cladopus_009175	K00850	ko00052	Galactose metaboli	Metabolism	Carbohydrate metabolism
Cladopus_009175	K00850	ko00680	Methane metabolism	Metabolism	Energy metabolism
Cladopus_009175	K00850	ko03018	RNA degradation	Genetic Informatio	Folding, sorting and degradation
Cladopus_009175	K00850	ko04152	AMPK signaling pat	Environmental Info	Signal transduction
Cladopus_009175	K00850	ko05230	Central carbon met	Human Diseases	Cancers: Overview
Cladopus_009219	K14515	ko04016	MAPK signaling pat	Environmental Info	Signal transduction
Cladopus_009219	K14515	ko04075	Plant hormone sign	Environmental Info	Signal transduction
Cladopus_009232	K04124	ko00904	Diterpenoid biosyn	Metabolism	Metabolism of terpenoids and polyketides
Cladopus_009240	K11816	ko00380	Tryptophan metabol	Metabolism	Amino acid metabolism
Cladopus_009250	K15639	ko00905	Brassinosteroid bi	Metabolism	Metabolism of terpenoids and polyketides
Cladopus_014839	K01051	ko00040	Pentose and glucur	Metabolism	Carbohydrate metabolism
Cladopus_014838	K02730	ko03050	Proteasome	Genetic Informatio	Folding, sorting and degradation
Cladopus_014849	K02729	ko03050	Proteasome	Genetic Informatio	Folding, sorting and degradation
Cladopus_003996	K01673	ko00910	Nitrogen metabolis	Metabolism	Energy metabolism
Cladopus_010676	K01653	ko00650	Butanoate metaboli	Metabolism	Carbohydrate metabolism
Cladopus_010676	K01653	ko00660	C5-Branched dibasi	Metabolism	Carbohydrate metabolism
Cladopus_010676	K01653	ko00290	Valine, leucine an	Metabolism	Amino acid metabolism
Cladopus_010676	K01653	ko00770	Pantothenate and C	Metabolism	Metabolism of cofactors and vitamins
Cladopus_010680	K05592	ko03018	RNA degradation	Genetic Informatio	Folding, sorting and degradation
Cladopus_010684	K02010	ko02010	ABC transporters	Environmental Info	Membrane transport
Cladopus_010684	K02052	ko02024	Quorum sensing	Cellular Processes	Cellular community - prokaryotes
Cladopus_010672	K11356	ko02020	Two-component syst	Environmental Info	Signal transduction
Cladopus_010682	K01563	ko00625	Chloroalkane and c	Metabolism	Xenobiotics biodegradation and metabolism
Cladopus_010682	K01563	ko00361	Chlorocyclohexane	Metabolism	Xenobiotics biodegradation and metabolism
Cladopus_010677	K01448	ko01503	Cationic antimicro	Human Diseases	Drug resistance: Antimicrobial
Cladopus_010686	K00058	ko00680	Methane metabolism	Metabolism	Energy metabolism
Cladopus_010686	K00058	ko00260	Glycine, serine an	Metabolism	Amino acid metabolism

Cladopus_010686	K16843	ko00270	Cysteine and methi	Metabolism	Amino acid metabolism
Cladopus_010695	K04042	ko00520	Amino sugar and nu	Metabolism	Carbohydrate metabolism
Cladopus_010695	K11528	ko00520	Amino sugar and nu	Metabolism	Carbohydrate metabolism
Cladopus_010705	K01582	ko00310	Lysine degradation	Metabolism	Amino acid metabolism
Cladopus_010705	K01582	ko00960	Tropane, piperidin	Metabolism	Biosynthesis of other secondary metabolites
Cladopus_010705	K01583	ko00330	Arginine and proli	Metabolism	Amino acid metabolism
Cladopus_010703	K18138	ko01501	beta-Lactam resist	Human Diseases	Drug resistance: Antimicrobial
Cladopus_010703	K18138	ko01503	Cationic antimicro	Human Diseases	Drug resistance: Antimicrobial
Cladopus_010719	K03405	ko00860	Porphyrin and chlo	Metabolism	Metabolism of cofactors and vitamins
Cladopus_010718	K04077	ko03018	RNA degradation	Genetic Informatio	Folding, sorting and degradation
Cladopus_010718	K04077	ko04212	Longevity regulati	Organismal Systems	Aging
Cladopus_010718	K04077	ko04940	Type I diabetes me	Human Diseases	Endocrine and metabolic diseases
Cladopus_010718	K04077	ko05134	Legionellosis	Human Diseases	Infectious diseases: Bacterial
Cladopus_010718	K04077	ko05152	Tuberculosis	Human Diseases	Infectious diseases: Bacterial
Cladopus_010727	K01895	ko00010	Glycolysis / Gluco	Metabolism	Carbohydrate metabolism
Cladopus_010727	K01895	ko00620	Pyruvate metabolis	Metabolism	Carbohydrate metabolism
Cladopus_010727	K01895	ko00630	Glyoxylate and dic	Metabolism	Carbohydrate metabolism
Cladopus_010727	K01895	ko00640	Propanoate metabol	Metabolism	Carbohydrate metabolism
Cladopus_010727	K01895	ko00720	Carbon fixation pa	Metabolism	Energy metabolism
Cladopus_010727	K01895	ko00680	Methane metabolism	Metabolism	Energy metabolism
Cladopus_010725	K01424	ko00250	Alanine, aspartate	Metabolism	Amino acid metabolism
Cladopus_010725	K01424	ko00460	Cyanoamino acid me	Metabolism	Metabolism of other amino acids
Cladopus_010732	K00239	ko00020	Citrate cycle (TCA	Metabolism	Carbohydrate metabolism
Cladopus_010732	K00239	ko00650	Butanoate metaboli	Metabolism	Carbohydrate metabolism
Cladopus_010732	K00239	ko00190	Oxidative phosphor	Metabolism	Energy metabolism
Cladopus_010732	K00239	ko00720	Carbon fixation pa	Metabolism	Energy metabolism
Cladopus_010732	K00239	ko05134	Legionellosis	Human Diseases	Infectious diseases: Bacterial
Cladopus_010732	K00242	ko00020	Citrate cycle (TCA	Metabolism	Carbohydrate metabolism
Cladopus_010732	K00242	ko00650	Butanoate metaboli	Metabolism	Carbohydrate metabolism
Cladopus_010732	K00242	ko00190	Oxidative phosphor	Metabolism	Energy metabolism

Cladopus_010732	K00242	ko00720	Carbon fixation pa	Metabolism	Energy metabolism
Cladopus_010750	K17686	ko04016	MAPK signaling pat	Environmental Info	Signal transduction
Cladopus_010750	K17686	ko01524	Platinum drug resi	Human Diseases	Drug resistance: Antineoplastic
Cladopus_010741	K01758	ko00260	Glycine, serine an	Metabolism	Amino acid metabolism
Cladopus_010741	K01758	ko00270	Cysteine and methi	Metabolism	Amino acid metabolism
Cladopus_010741	K01758	ko00450	Selenocompound met	Metabolism	Metabolism of other amino acids
Cladopus_010738	K00265	ko00910	Nitrogen metabolis	Metabolism	Energy metabolism
Cladopus_010738	K00265	ko00250	Alanine, aspartate	Metabolism	Amino acid metabolism
Cladopus_010738	K00284	ko00630	Glyoxylate and dic	Metabolism	Carbohydrate metabolism
Cladopus_010738	K00284	ko00910	Nitrogen metabolis	Metabolism	Energy metabolism
Cladopus_010743	K02437	ko00630	Glyoxylate and dic	Metabolism	Carbohydrate metabolism
Cladopus_010743	K02437	ko00260	Glycine, serine an	Metabolism	Amino acid metabolism
Cladopus_010740	K00507	ko01040	Biosynthesis of un	Metabolism	Lipid metabolism
Cladopus_010740	K00507	ko04152	AMPK signaling pat	Environmental Info	Signal transduction
Cladopus_010740	K00507	ko03320	PPAR signaling pat	Organismal Systems	Endocrine system
Cladopus_010740	K00507	ko04212	Longevity regulati	Organismal Systems	Aging
Cladopus_010764	K03671	ko04621	NOD-like receptor	Organismal Systems	Immune system
Cladopus_010764	K03671	ko05418	Fluid shear stress	Human Diseases	Cardiovascular diseases
Cladopus_010759	K03148	ko00730	Thiamine metabolis	Metabolism	Metabolism of cofactors and vitamins
Cladopus_010759	K03148	ko04122	Sulfur relay syste	Genetic Informatio	Folding, sorting and degradation
Cladopus_010759	K11996	ko04122	Sulfur relay syste	Genetic Informatio	Folding, sorting and degradation
Cladopus_010757	K02010	ko02010	ABC transporters	Environmental Info	Membrane transport
Cladopus_010757	K05816	ko02010	ABC transporters	Environmental Info	Membrane transport
Cladopus_010757	K10112	ko02010	ABC transporters	Environmental Info	Membrane transport
Cladopus_010762	K00135	ko00650	Butanoate metaboli	Metabolism	Carbohydrate metabolism
Cladopus_010762	K00135	ko00250	Alanine, aspartate	Metabolism	Amino acid metabolism
Cladopus_010762	K00135	ko00310	Lysine degradation	Metabolism	Amino acid metabolism
Cladopus_010762	K00135	ko00350	Tyrosine metabolis	Metabolism	Amino acid metabolism
Cladopus_010762	K00135	ko00760	Nicotinate and nic	Metabolism	Metabolism of cofactors and vitamins
Cladopus_010762	K08324	ko00650	Butanoate metaboli	Metabolism	Carbohydrate metabolism

Cladopus_010762	K08324	ko00250	Alanine, aspartate	Metabolism	Amino acid metabolism
Cladopus_010762	K08324	ko00760	Nicotinate and nic	Metabolism	Metabolism of cofactors and vitamins
Cladopus_010761	K05585	ko00190	Oxidative phosphor	Metabolism	Energy metabolism
Cladopus_010768	K01873	ko00970	Aminoacyl-tRNA bio	Genetic Informatio	Translation
Cladopus_010773	K01253	ko00980	Metabolism of xeno	Metabolism	Xenobiotics biodegradation and metabolism
Cladopus_010773	K01253	ko04976	Bile secretion	Organismal Systems	Digestive system
Cladopus_010773	K01253	ko05204	Chemical carcinoge	Human Diseases	Cancers: Overview
Cladopus_010775	K00162	ko00010	Glycolysis / Gluco	Metabolism	Carbohydrate metabolism
Cladopus_010775	K00162	ko00020	Citrate cycle (TCA	Metabolism	Carbohydrate metabolism
Cladopus_010775	K00162	ko00620	Pyruvate metabolis	Metabolism	Carbohydrate metabolism
Cladopus_010775	K00162	ko04066	HIF-1 signaling pa	Environmental Info	Signal transduction
Cladopus_010775	K00162	ko04922	Glucagon signaling	Organismal Systems	Endocrine system
Cladopus_010775	K00162	ko05230	Central carbon met	Human Diseases	Cancers: Overview
Cladopus_010775	K11381	ko00640	Propanoate metabol	Metabolism	Carbohydrate metabolism
Cladopus_010775	K11381	ko00280	Valine, leucine an	Metabolism	Amino acid metabolism
Cladopus_010779	K03186	ko00130	Ubiquinone and oth	Metabolism	Metabolism of cofactors and vitamins
Cladopus_010779	K03186	ko00940	Phenylpropanoid bi	Metabolism	Biosynthesis of other secondary metabolites
Cladopus_010779	K03186	ko00627	Aminobenzoate degr	Metabolism	Xenobiotics biodegradation and metabolism
Cladopus_010783	K09458	ko00061	Fatty acid biosynt	Metabolism	Lipid metabolism
Cladopus_010783	K09458	ko00780	Biotin metabolism	Metabolism	Metabolism of cofactors and vitamins
Cladopus_010785	K03273	ko00540	Lipopolysaccharide	Metabolism	Glycan biosynthesis and metabolism
Cladopus_010785	K16881	ko00051	Fructose and manno	Metabolism	Carbohydrate metabolism
Cladopus_010785	K16881	ko00520	Amino sugar and nu	Metabolism	Carbohydrate metabolism
Cladopus_010788	K00275	ko00750	Vitamin B6 metabol	Metabolism	Metabolism of cofactors and vitamins
Cladopus_010791	K02706	ko00195	Photosynthesis	Metabolism	Energy metabolism
Cladopus_010791	K08929	ko02020	Two-component syst	Environmental Info	Signal transduction
Cladopus_010801	K01890	ko00970	Aminoacyl-tRNA bio	Genetic Informatio	Translation
Cladopus_010808	K19221	ko00860	Porphyrin and chlo	Metabolism	Metabolism of cofactors and vitamins
Cladopus_010825	K00057	ko00564	Glycerophospholipi	Metabolism	Lipid metabolism
Cladopus_010830	K00027	ko00620	Pyruvate metabolis	Metabolism	Carbohydrate metabolism

Cladopus_010830	K00027	ko02020	Two-component syst	Environmental Info	Signal transduction
Cladopus_010830	K01679	ko00020	Citrate cycle (TCA	Metabolism	Carbohydrate metabolism
Cladopus_010830	K01679	ko00620	Pyruvate metabolis	Metabolism	Carbohydrate metabolism
Cladopus_010830	K01679	ko00720	Carbon fixation pa	Metabolism	Energy metabolism
Cladopus_010830	K01679	ko05200	Pathways in cancer	Human Diseases	Cancers: Overview
Cladopus_010830	K01679	ko05211	Renal cell carcino	Human Diseases	Cancers: Specific types
Cladopus_010830	K01679	ko04934	Cushing syndrome	Human Diseases	Endocrine and metabolic diseases
Cladopus_010841	K01657	ko00400	Phenylalanine, tyr	Metabolism	Amino acid metabolism
Cladopus_010841	K01657	ko00405	Phenazine biosynth	Metabolism	Biosynthesis of other secondary metabolites
Cladopus_010841	K01657	ko02024	Quorum sensing	Cellular Processes	Cellular community - prokaryotes
Cladopus_010841	K01657	ko02025	Biofilm formation	Cellular Processes	Cellular community - prokaryotes
Cladopus_010838	K16786	ko02010	ABC transporters	Environmental Info	Membrane transport
Cladopus_010839	K03072	ko03060	Protein export	Genetic Informatio	Folding, sorting and degradation
Cladopus_010839	K03072	ko03070	Bacterial secretio	Environmental Info	Membrane transport
Cladopus_010839	K12257	ko03060	Protein export	Genetic Informatio	Folding, sorting and degradation
Cladopus_010839	K12257	ko03070	Bacterial secretio	Environmental Info	Membrane transport
Cladopus_010839	K12257	ko02024	Quorum sensing	Cellular Processes	Cellular community - prokaryotes
Cladopus_010840	K02692	ko00195	Photosynthesis	Metabolism	Energy metabolism
Cladopus_010836	K00588	ko00360	Phenylalanine meta	Metabolism	Amino acid metabolism
Cladopus_010836	K00588	ko00940	Phenylpropanoid bi	Metabolism	Biosynthesis of other secondary metabolites
Cladopus_010836	K00588	ko00945	Stilbenoid, diaryl	Metabolism	Biosynthesis of other secondary metabolites
Cladopus_010836	K00588	ko00941	Flavonoid biosynth	Metabolism	Biosynthesis of other secondary metabolites
Cladopus_010836	K16028	ko01052	Type I polyketide	Metabolism	Metabolism of terpenoids and polyketides
Cladopus_010836	K16028	ko01051	Biosynthesis of an	Metabolism	Metabolism of terpenoids and polyketides
Cladopus_010852	K04043	ko03018	RNA degradation	Genetic Informatio	Folding, sorting and degradation
Cladopus_010852	K04043	ko04212	Longevity regulati	Organismal Systems	Aging
Cladopus_010852	K04043	ko05152	Tuberculosis	Human Diseases	Infectious diseases: Bacterial
Cladopus_010863	K04567	ko00970	Aminoacyl-tRNA bio	Genetic Informatio	Translation
Cladopus_010862	K00945	ko00240	Pyrimidine metabol	Metabolism	Nucleotide metabolism
Cladopus_010862	K13799	ko00240	Pyrimidine metabol	Metabolism	Nucleotide metabolism

Cladopus_010862	K13799	ko00410	beta-Alanine metab	Metabolism	Metabolism of other amino acids
Cladopus_010862	K13799	ko00770	Pantothenate and C	Metabolism	Metabolism of cofactors and vitamins
Cladopus_010865	K02954	ko03010	Ribosome	Genetic Informatio	Translation
Cladopus_010869	K03403	ko00860	Porphyrin and chlo	Metabolism	Metabolism of cofactors and vitamins
Cladopus_010876	K04771	ko02020	Two-component syst	Environmental Info	Signal transduction
Cladopus_010876	K04771	ko01503	Cationic antimicro	Human Diseases	Drug resistance: Antimicrobial
Cladopus_010876	K08372	ko02020	Two-component syst	Environmental Info	Signal transduction
Cladopus_010872	K03778	ko00620	Pyruvate metabolis	Metabolism	Carbohydrate metabolism
Cladopus_010879	K02031	ko02024	Quorum sensing	Cellular Processes	Cellular community - prokaryotes
Cladopus_010879	K02032	ko02024	Quorum sensing	Cellular Processes	Cellular community - prokaryotes
Cladopus_010879	K10823	ko02010	ABC transporters	Environmental Info	Membrane transport
Cladopus_010879	K10823	ko02024	Quorum sensing	Cellular Processes	Cellular community - prokaryotes
Cladopus_010879	K10823	ko01501	beta-Lactam resist	Human Diseases	Drug resistance: Antimicrobial
Cladopus_010879	K10824	ko02010	ABC transporters	Environmental Info	Membrane transport
Cladopus_010879	K15587	ko02010	ABC transporters	Environmental Info	Membrane transport
Cladopus_010896	K00981	ko00564	Glycerophospholipi	Metabolism	Lipid metabolism
Cladopus_010896	K00981	ko04070	Phosphatidylinosit	Environmental Info	Signal transduction
Cladopus_010898	K00794	ko00740	Riboflavin metabol	Metabolism	Metabolism of cofactors and vitamins
Cladopus_010930	K01520	ko00240	Pyrimidine metabol	Metabolism	Nucleotide metabolism
Cladopus_010930	K01520	ko00983	Drug metabolism -	Metabolism	Xenobiotics biodegradation and metabolism
Cladopus_010908	K05575	ko00190	Oxidative phosphor	Metabolism	Energy metabolism
Cladopus_010947	K07769	ko02020	Two-component syst	Environmental Info	Signal transduction
Cladopus_010941	K02886	ko03010	Ribosome	Genetic Informatio	Translation
Cladopus_010936	K02933	ko03010	Ribosome	Genetic Informatio	Translation
Cladopus_010937	K02931	ko03010	Ribosome	Genetic Informatio	Translation
Cladopus_010939	K02982	ko03010	Ribosome	Genetic Informatio	Translation
Cladopus_010934	K02948	ko03010	Ribosome	Genetic Informatio	Translation
Cladopus_010935	K03076	ko03060	Protein export	Genetic Informatio	Folding, sorting and degradation
Cladopus_010935	K03076	ko03070	Bacterial secretio	Environmental Info	Membrane transport
Cladopus_010935	K03076	ko02024	Quorum sensing	Cellular Processes	Cellular community - prokaryotes

Cladopus_010942	K02926	ko03010	Ribosome	Genetic Informatio	Translation
Cladopus_010933	K02879	ko03010	Ribosome	Genetic Informatio	Translation
Cladopus_010963	K01495	ko00790	Folate biosynthesi	Metabolism	Metabolism of cofactors and vitamins
Cladopus_010964	K00278	ko00250	Alanine, aspartate	Metabolism	Amino acid metabolism
Cladopus_010964	K00278	ko00760	Nicotinate and nic	Metabolism	Metabolism of cofactors and vitamins
Cladopus_010984	K04035	ko00860	Porphyrin and chlo	Metabolism	Metabolism of cofactors and vitamins
Cladopus_007065	K01188	ko00500	Starch and sucrose	Metabolism	Carbohydrate metabolism
Cladopus_007065	K01188	ko00460	Cyanoamino acid me	Metabolism	Metabolism of other amino acids
Cladopus_007065	K01188	ko00940	Phenylpropanoid bi	Metabolism	Biosynthesis of other secondary metabolites
Cladopus_005492	K02976	ko03010	Ribosome	Genetic Informatio	Translation
Cladopus_005494	K02976	ko03010	Ribosome	Genetic Informatio	Translation
Cladopus_000957	K02693	ko00195	Photosynthesis	Metabolism	Energy metabolism
Cladopus_001001	K04125	ko00904	Diterpenoid biosyn	Metabolism	Metabolism of terpenoids and polyketides
Cladopus_001019	K05658	ko02010	ABC transporters	Environmental Info	Membrane transport
Cladopus_001019	K05658	ko04976	Bile secretion	Organismal Systems	Digestive system
Cladopus_001019	K05658	ko05206	MicroRNAs in cance	Human Diseases	Cancers: Overview
Cladopus_001019	K05658	ko05226	Gastric cancer	Human Diseases	Cancers: Specific types
Cladopus_001051	K12128	ko04712	Circadian rhythm -	Organismal Systems	Environmental adaptation
Cladopus_001051	K12130	ko04712	Circadian rhythm -	Organismal Systems	Environmental adaptation
Cladopus_001086	K02997	ko03010	Ribosome	Genetic Informatio	Translation
Cladopus_001121	K01179	ko00500	Starch and sucrose	Metabolism	Carbohydrate metabolism
Cladopus_001121	K19668	ko00500	Starch and sucrose	Metabolism	Carbohydrate metabolism
Cladopus_001121	K19668	ko02020	Two-component syst	Environmental Info	Signal transduction
Cladopus_001167	K14484	ko04075	Plant hormone sign	Environmental Info	Signal transduction
Cladopus_011636	K01051	ko00040	Pentose and glucur	Metabolism	Carbohydrate metabolism
Cladopus_016441	K14488	ko04075	Plant hormone sign	Environmental Info	Signal transduction
