# Supplementary material for: A high-quality genome provides insights into the new taxonomic status and genomic characteristics of Cladopus chinensis (Podostemaceae)
Source: Hortic Res. 2020 Apr 1;7:46. doi: 10.1038/s41438-020-0269-5 (PMC7109043; doi:10.1038/s41438-020-0269-5)
Supplement: Supplementary file 14 — Table S18 my.gene.counts.matrix.root_vs_shoot.DESeq2 [file 41438_2020_269_MOESM14_ESM.pdf]

geneid	sampleA	sampleB	baseMeanA	baseMeanB	baseMean	log2FoldChange	pvalue	padj
Cladopus_005856	root	shoot	10801.21826	821.4931039	5811.355681	3.718679888	0	0
Cladopus_019790	root	shoot	34830.52877	9357.843008	22094.18589	1.895971081	0	0
Cladopus_004188	root	shoot	14391.46025	46538.09777	30464.77901	-1.693151803	0	0
Cladopus_003559	root	shoot	10576.12311	1294.904161	5935.513635	3.030001531	0	0
Cladopus_010308	root	shoot	4815.714237	559.1668551	2687.440546	3.104775281	0	0
Cladopus_011914	root	shoot	4417.289459	601.8193965	2509.554428	2.87631463	0	0
Cladopus_018002	root	shoot	3640.917324	189.9211548	1915.41924	4.260640934	0	0
Cladopus_002028	root	shoot	22027.80648	5241.61514	13634.71081	2.071151903	0	0
Cladopus_004901	root	shoot	11752.98491	2369.61592	7061.300414	2.309894802	0	0
Cladopus_009592	root	shoot	8235.893271	1027.780425	4631.836848	3.001932993	0	0
Cladopus_001246	root	shoot	28313.9018	4083.06045	16198.48112	2.79347245	0	0
Cladopus_010320	root	shoot	5494.601181	647.8259298	3071.213555	3.083159822	0	0
Cladopus_020020	root	shoot	459.4225076	4977.92049	2718.671499	-3.437901171	0	0
Cladopus_001123	root	shoot	6892.763955	1284.336578	4088.550266	2.424181835	0	0
Cladopus_016322	root	shoot	7931.835629	1290.212656	4611.024142	2.621486481	0	0
Cladopus_016058	root	shoot	18092.81915	4786.729177	11439.77416	1.91855812	0	0
Cladopus_018296	root	shoot	11220.36704	1986.798975	6603.583009	2.497541305	0	0
Cladopus_015772	root	shoot	11392.97841	45616.13399	28504.5562	-2.001537839	0	0
Cladopus_022658	root	shoot	8021.580359	1883.849334	4952.714846	2.091307463	0	0
Cladopus_009915	root	shoot	2708.820481	174.6256314	1441.723056	3.951678474	0	0
Cladopus_014025	root	shoot	20792.46999	1595.783793	11194.12689	3.703597332	0	0
Cladopus_022537	root	shoot	6242.100121	802.4082784	3522.2542	2.959542794	0	0
Cladopus_001827	root	shoot	1434.45707	10676.7294	6055.593233	-2.896953938	0	0
Cladopus_004196	root	shoot	22345.41746	3897.872781	13121.64512	2.519089879	0	0
Cladopus_016709	root	shoot	64703.10445	16624.14535	40663.6249	1.960494622	0	0
Cladopus_014029	root	shoot	3678.986953	264.3233176	1971.655135	3.796789144	0	0
Cladopus_023930	root	shoot	16650.79635	3684.277992	10167.53717	2.175861301	0	0
Cladopus_014024	root	shoot	13112.05788	1997.373167	7554.715524	2.714968785	0	0
Cladopus_012830	root	shoot	64727.52709	378.2924506	32552.90977	7.416827799	0	0

Cladopus_009902	root	shoot	6634.125324	1297.246182	3965.685753	2.353597165	0	0
Cladopus_025103	root	shoot	58226.40528	10335.77123	34281.08826	2.494059457	0	0
Cladopus_007299	root	shoot	5886.324266	46174.93137	26030.62782	-2.972064686	0	0
Cladopus_015472	root	shoot	33044.37529	7840.564257	20442.46977	2.075406736	0	0
Cladopus_003018	root	shoot	5137.029745	519.2964545	2828.1631	3.305320742	0	0
Cladopus_000856	root	shoot	17823.41656	5299.284789	11561.35068	1.749869251	0	0
Cladopus_001362	root	shoot	10366.15459	1928.087906	6147.121247	2.42686685	0	0
Cladopus_001810	root	shoot	7241.192659	1810.612694	4525.902677	1.999246593	0	0
Cladopus_002023	root	shoot	11451.1509	1378.3616	6414.756249	3.055050296	0	0
Cladopus_009290	root	shoot	1022.88028	10279.72303	5651.301657	-3.329751556	0	0
Cladopus_011181	root	shoot	5992.283001	999.9345919	3496.108796	2.581097558	0	0
Cladopus_020179	root	shoot	32151.65238	6772.91367	19462.28303	2.247172795	0	0
Cladopus_021243	root	shoot	2000.666297	130.8717935	1065.769046	3.936344418	0	0
Cladopus_024913	root	shoot	41906.99651	1640.898894	21773.9477	4.674028955	0	0
Cladopus_009570	root	shoot	17033.86406	2699.830835	9866.847448	2.657322832	0	0
Cladopus_003706	root	shoot	53.2663862	2288.090654	1170.67852	-5.428708155	0	0
Cladopus_019903	root	shoot	34744.73755	6021.224838	20382.98119	2.52872255	0	0
Cladopus_011122	root	shoot	8281.083222	995.4284724	4638.255847	3.057406731	0	0
Cladopus_002634	root	shoot	12781.70792	863.7294236	6822.718671	3.886438305	0	0
Cladopus_021837	root	shoot	10194.4521	1218.12735	5706.289725	3.064932535	0	0
Cladopus_013506	root	shoot	3711.986046	76.25352168	1894.119784	5.61363708	0	0
Cladopus_015929	root	shoot	24110.14748	1146.671105	12628.40929	4.394809172	0	0
Cladopus_002365	root	shoot	9190.568479	1984.515203	5587.541841	2.21145124	0	0
Cladopus_011276	root	shoot	2634.588874	62.72535683	1348.657116	5.406558969	0	0
Cladopus_015012	root	shoot	7832.289878	1035.724854	4434.007366	2.919336319	0	0
Cladopus_010082	root	shoot	93.82894616	2387.453069	1240.641008	-4.662397775	0	0
Cladopus_023017	root	shoot	3623.980258	552.6708255	2088.325542	2.713740046	0	0
Cladopus_020138	root	shoot	3651.538182	342.3331665	1996.935674	3.412717714	0	0
Cladopus_004584	root	shoot	19399.84389	876.2226007	10138.03324	4.46822122	0	0
Cladopus_003424	root	shoot	3057.928143	114.1246246	1586.026384	4.734289984	0	0

Cladopus_002366	root	shoot	1087.291225	6947.216631	4017.253928	-2.675496817	0	0
Cladopus_022481	root	shoot	13308.27785	1266.069603	7287.173728	3.39406087	0	0
Cladopus_010228	root	shoot	6534.811604	516.6079377	3525.709771	3.667277848	0	0
Cladopus_001833	root	shoot	5958.915939	649.9746459	3304.445293	3.19668043	0	0
Cladopus_014072	root	shoot	24727.65801	5942.102239	15334.88013	2.057259708	0	0
Cladopus_010444	root	shoot	12459.16271	1767.495693	7113.329203	2.817847068	0	0
Cladopus_005565	root	shoot	82447.30567	11948.56809	47197.93688	2.786641985	0	0
Cladopus_023469	root	shoot	30856.15939	2941.249979	16898.70468	3.391006482	0	0
Cladopus_017249	root	shoot	7623.1682	1747.132619	4685.15041	2.125647624	0	0
Cladopus_001534	root	shoot	11841.60421	1577.746385	6709.675299	2.907511289	0	0
Cladopus_000462	root	shoot	5491.545433	869.6627762	3180.604104	2.657992322	0	0
Cladopus_007253	root	shoot	6332.927053	991.2280558	3662.077554	2.676445311	0	0
Cladopus_012836	root	shoot	63537.83406	346.0153825	31941.92472	7.517475757	0	0
Cladopus_004574	root	shoot	10153.04664	759.3706786	5456.20866	3.741319627	0	0
Cladopus_025633	root	shoot	17356.33719	2317.865574	9837.101381	2.904732764	0	0
Cladopus_006634	root	shoot	22762.76091	3109.916862	12936.33888	2.871266188	0	0
Cladopus_004301	root	shoot	46010.38407	3022.357996	24516.37103	3.927960841	0	0
Cladopus_016456	root	shoot	22887.57083	5731.280341	14309.42558	1.997615144	0	0
Cladopus_004161	root	shoot	281.9461784	3043.666187	1662.806183	-3.432087304	0	0
Cladopus_023117	root	shoot	9802.643084	1840.799447	5821.721266	2.412928779	0	0
Cladopus_005883	root	shoot	18796.18816	4334.7053	11565.44673	2.11672635	0	0
Cladopus_010085	root	shoot	8404.322268	1556.130666	4980.226467	2.433227819	0	0
Cladopus_025104	root	shoot	8024.234685	1724.965767	4874.600226	2.217490385	0	0
Cladopus_000075	root	shoot	8050.536755	1638.93839	4844.737572	2.296288014	0	0
Cladopus_005124	root	shoot	15991.98231	3213.564351	9602.773331	2.315668273	0	0
Cladopus_004229	root	shoot	6844.921074	507.3172581	3676.119166	3.758443762	0	0
Cladopus_000371	root	shoot	10978.21181	2847.048293	6912.630051	1.946336233	0	0
Cladopus_004038	root	shoot	4296.985083	399.6250134	2348.305048	3.421869492	0	0
Cladopus_004903	root	shoot	27859.12422	4444.641113	16151.88267	2.647689265	0	0
Cladopus_006721	root	shoot	1667.964753	71.54853256	869.756643	4.54555833	0	0

Cladopus_008492	root	shoot	3467.402373	280.6003217	1874.001348	3.632308929	0	0
Cladopus_009868	root	shoot	32771.50912	9114.827773	20943.16845	1.846020724	0	0
Cladopus_010289	root	shoot	15375.80537	3422.498671	9399.152022	2.167519061	0	0
Cladopus_017656	root	shoot	10850.21374	1530.035543	6190.124642	2.826858909	0	0
Cladopus_001874	root	shoot	34950.71185	7503.22258	21226.96722	2.219839965	0	0
Cladopus_023400	root	shoot	9919.175378	2721.153185	6320.164282	1.866346142	0	0
Cladopus_003692	root	shoot	2943.140794	16024.75268	9483.946736	-2.445267351	0	0
Cladopus_014052	root	shoot	20321.43634	5407.866891	12864.65162	1.909915826	0	0
Cladopus_005054	root	shoot	6973.577174	1047.918804	4010.747989	2.732665301	0	0
Cladopus_010402	root	shoot	5224.506494	697.3236648	2960.915079	2.907475993	0	0
Cladopus_001896	root	shoot	7264.380858	1490.540652	4377.460755	2.2856905	0	0
Cladopus_013075	root	shoot	14102.65821	3614.166894	8858.412554	1.963996687	0	0
Cladopus_008477	root	shoot	3083.611892	198.8954551	1641.253673	3.959935341	0	0
Cladopus_024011	root	shoot	72701.58305	8553.288589	40627.43582	3.087530893	0	0
Cladopus_010455	root	shoot	15157.82044	4028.524167	9593.172302	1.911466069	0	0
Cladopus_023940	root	shoot	2493.842412	160.0908243	1326.966618	3.955350093	0	0
Cladopus_020719	root	shoot	5262.174229	384.8738149	2823.524022	3.774023796	0	0
Cladopus_002649	root	shoot	531.556262	3816.72885	2174.142556	-2.844491422	0	0
Cladopus_004902	root	shoot	6038.332708	154.8660115	3096.59936	5.291125116	0	0
Cladopus_012851	root	shoot	32866.86601	860.7855102	16863.82576	5.257531672	0	0
Cladopus_007477	root	shoot	3938.578415	338.2191246	2138.39877	3.545102926	0	0
Cladopus_004302	root	shoot	51161.15902	3105.082526	27133.12077	4.042566599	0	0
Cladopus_002364	root	shoot	9130.681568	1271.253044	5200.967306	2.845381504	0	0
Cladopus_015347	root	shoot	813.7255416	6824.749376	3819.237459	-3.068877239	0	0
Cladopus_001936	root	shoot	139466.0835	21159.94738	80313.01544	2.720537253	0	0
Cladopus_008489	root	shoot	23521.97575	2644.623622	13083.29969	3.153404921	0	0
Cladopus_014860	root	shoot	8527.698617	1040.739483	4784.21905	3.036212026	0	0
Cladopus_006475	root	shoot	10549.82106	688.4714091	5619.146237	3.939151446	0	0
Cladopus_024338	root	shoot	10756.73071	2777.948432	6767.339572	1.952609009	0	0
Cladopus_018315	root	shoot	1928.62344	134.2405877	1031.432014	3.850593209	0	0

Cladopus_025107	root	shoot	20566.98185	3872.577456	12219.77965	2.409056466	0	0
Cladopus_010580	root	shoot	9562.148164	50851.68499	30206.91658	-2.411133247	0	0
Cladopus_019845	root	shoot	17495.70396	2336.154591	9915.929275	2.904274459	0	0
Cladopus_005271	root	shoot	4371.674335	70.05055737	2220.862446	5.948781912	0	0
Cladopus_022486	root	shoot	41185.098	7104.340679	24144.71934	2.535315916	0	0
Cladopus_009962	root	shoot	31709.02847	3986.273499	17847.65099	2.991717768	0	0
Cladopus_003590	root	shoot	62700.99115	14750.88881	38725.93998	2.087601477	0	0
Cladopus_018985	root	shoot	15946.78435	2315.582076	9131.183213	2.783178796	0	0
Cladopus_011010	root	shoot	4549.640204	374.4546748	2462.047439	3.596999384	0	0
Cladopus_025568	root	shoot	6559.434464	865.9229935	3712.678729	2.923547153	0	0
Cladopus_010610	root	shoot	1884.343834	139.223472	1011.783653	3.762566663	0	0
Cladopus_011015	root	shoot	18874.99243	2199.894145	10537.44329	3.101420362	0	0
Cladopus_005648	root	shoot	12762.38033	1585.151808	7173.766072	3.01020761	0	0
Cladopus_002979	root	shoot	6235.106364	531.6527263	3383.379545	3.556358861	0	0
Cladopus_020964	root	shoot	4298.579388	331.1326688	2314.856029	3.70209041	0	0
Cladopus_008856	root	shoot	4593.073281	940.1579008	2766.615591	2.289504429	0	0
Cladopus_008324	root	shoot	3065.137161	270.9156302	1668.026396	3.498172859	0	0
Cladopus_004897	root	shoot	12663.48223	2743.155727	7703.31898	2.207329205	0	0
Cladopus_017293	root	shoot	19471.23067	4551.501586	12011.36613	2.096774515	0	0
Cladopus_009195	root	shoot	18277.84705	4490.018687	11383.93287	2.025733514	0	0
Cladopus_019054	root	shoot	32824.994	5325.956884	19075.47544	2.623601986	0	0
Cladopus_019901	root	shoot	12651.0218	2718.117792	7684.569798	2.218442542	0	0
Cladopus_021964	root	shoot	996.0523907	6250.84248	3623.447435	-2.651487975	0	0
Cladopus_017060	root	shoot	5910.824323	946.5156719	3428.669997	2.640603025	0	0
Cladopus_014763	root	shoot	12368.50763	3410.12835	7889.317991	1.858607519	0	0
Cladopus_018003	root	shoot	2882.909252	151.5533911	1517.231322	4.256506974	0	0
Cladopus_021924	root	shoot	6834.995106	916.9889209	3875.992013	2.896791684	0	0
Cladopus_004228	root	shoot	57060.94824	12665.06922	34863.00873	2.171621732	0	0
Cladopus_004891	root	shoot	8484.230407	741.0477565	4612.639082	3.517133007	0	0
Cladopus_011669	root	shoot	25828.95695	5271.799454	15550.3782	2.292655847	0	0

Cladopus_011294	root	shoot	2919.439021	70.67451162	1495.056766	5.3844552	0	0
Cladopus_012820	root	shoot	4615.443016	940.0290466	2777.736032	2.294681353	0	0
Cladopus_003618	root	shoot	42039.07997	8232.824369	25135.95217	2.352367772	0	0
Cladopus_000052	root	shoot	16403.90075	1986.384916	9195.142834	3.0456395	0	0
Cladopus_009593	root	shoot	16212.74617	1507.566235	8860.1562	3.427548416	0	0
Cladopus_017988	root	shoot	3817.211147	196.9837796	2007.097463	4.274861432	0	0
Cladopus_021872	root	shoot	8630.871152	975.8950051	4803.383079	3.143803067	0	0
Cladopus_014026	root	shoot	10966.72814	846.3933871	5906.560765	3.694209602	0	0
Cladopus_019550	root	shoot	160061.338	15202.11788	87631.72794	3.396391922	0	0
Cladopus_011262	root	shoot	4772.441349	214.0200203	2493.230685	4.483668229	0	0
Cladopus_014028	root	shoot	7450.436506	588.0950436	4019.265775	3.662162156	0	0
Cladopus_023939	root	shoot	8663.832429	420.4428541	4542.137641	4.36071412	0	0
Cladopus_008668	root	shoot	19954.60558	1916.3764	10935.49099	3.380499818	0	0
Cladopus_001847	root	shoot	7937.684728	1807.528897	4872.606813	2.134603077	0	0
Cladopus_014276	root	shoot	17596.44893	2834.903796	10215.67636	2.63430847	0	0
Cladopus_021369	root	shoot	3287.882588	276.1127658	1781.997677	3.579171199	0	0
Cladopus_021814	root	shoot	26158.36288	4401.218022	15279.79045	2.571567398	0	0
Cladopus_008467	root	shoot	6363.10504	1201.709505	3782.407272	2.403027733	0	0
Cladopus_014865	root	shoot	32971.4736	7835.440045	20403.45682	2.073010647	0	0
Cladopus_010208	root	shoot	19207.0325	4656.792292	11931.9124	2.043852688	0	0
Cladopus_012852	root	shoot	34615.71106	855.9400543	17735.82556	5.34000149	0	0
Cladopus_014027	root	shoot	11838.93308	1818.998193	6828.965637	2.702044046	0	0
Cladopus_003767	root	shoot	5472.189639	332.5308844	2902.360262	4.043253138	0	0
Cladopus_010238	root	shoot	5610.318251	488.1203131	3049.219282	3.523870812	0	0
Cladopus_006234	root	shoot	1700.865664	55.55260935	878.2091367	4.933408452	5.33E-307	5.96E-305
Cladopus_007478	root	shoot	11492.28416	1751.849524	6622.066844	2.713069346	2.17E-306	2.41E-304
Cladopus_004583	root	shoot	4836.631862	808.2278904	2822.429876	2.583568835	2.26E-306	2.50E-304
Cladopus_002325	root	shoot	12901.35575	3907.154803	8404.255275	1.723458199	2.57E-305	2.83E-303
Cladopus_011489	root	shoot	8498.003801	44498.16089	26498.08234	-2.388774043	3.12E-305	3.41E-303
Cladopus_009812	root	shoot	4293.869757	853.4779164	2573.673837	2.332242751	3.44E-305	3.74E-303

Cladopus_017976	root	shoot	5464.29549	1020.894983	3242.595237	2.421816722	5.20E-305	5.62E-303
Cladopus_011665	root	shoot	37374.34608	7329.499495	22351.92279	2.350410955	8.70E-305	9.35E-303
Cladopus_016047	root	shoot	47617.03095	7731.519234	27674.27509	2.622778707	1.34E-304	1.43E-302
Cladopus_008426	root	shoot	2387.418593	357.0202833	1372.219438	2.741805626	5.77E-303	6.13E-301
Cladopus_009876	root	shoot	6019.460458	831.6119166	3425.536187	2.85880908	1.51E-301	1.60E-299
Cladopus_017444	root	shoot	12041.95538	2424.035639	7232.99551	2.313051321	4.27E-300	4.50E-298
Cladopus_021983	root	shoot	6174.980867	824.9143692	3499.947618	2.906261792	3.27E-299	3.43E-297
Cladopus_013584	root	shoot	2472.192634	10911.58828	6691.890458	-2.142401715	1.22E-298	1.27E-296
Cladopus_000034	root	shoot	2536.605789	14565.13698	8550.871382	-2.522400937	2.44E-297	2.53E-295
Cladopus_009955	root	shoot	2666.989968	411.6831788	1539.336573	2.694438221	5.56E-296	5.72E-294
Cladopus_018122	root	shoot	4965.140152	1064.030778	3014.585465	2.220834092	1.57E-295	1.61E-293
Cladopus_007545	root	shoot	6231.571275	1366.75768	3799.164478	2.188424626	4.62E-294	4.71E-292
Cladopus_011666	root	shoot	4392.341468	744.8363943	2568.588931	2.562469507	1.44E-293	1.46E-291
Cladopus_018094	root	shoot	31846.31441	6794.445997	19320.38021	2.228889209	2.49E-291	2.51E-289
Cladopus_014041	root	shoot	3580.823764	623.0756592	2101.949712	2.522408249	3.97E-291	3.98E-289
Cladopus_007381	root	shoot	17565.89726	5122.839398	11344.36833	1.777895316	9.15E-288	9.13E-286
Cladopus_009780	root	shoot	5656.033282	1296.689871	3476.361577	2.124192478	1.67E-287	1.66E-285
Cladopus_002691	root	shoot	4463.604751	921.7311466	2692.667949	2.278305846	4.92E-287	4.86E-285
Cladopus_003090	root	shoot	12085.04416	2610.030732	7347.537446	2.21160671	1.43E-286	1.40E-284
Cladopus_024733	root	shoot	4692.603807	16281.96158	10487.28269	-1.795099855	6.44E-286	6.30E-284
Cladopus_000752	root	shoot	1858.07085	7358.934755	4608.502803	-1.986071839	2.05E-283	2.00E-281
Cladopus_007349	root	shoot	6364.792004	1704.233924	4034.512964	1.900444772	3.73E-283	3.61E-281
Cladopus_015380	root	shoot	6093.427383	1525.510686	3809.469034	1.997580306	5.71E-283	5.50E-281
Cladopus_011812	root	shoot	4852.738048	1029.46919	2941.103619	2.23549857	1.17E-282	1.12E-280
Cladopus_002926	root	shoot	1406.380195	96.34898296	751.3645892	3.871226315	1.22E-281	1.17E-279
Cladopus_004140	root	shoot	16160.74157	4132.671487	10146.70653	1.967215107	8.10E-281	7.69E-279
Cladopus_018884	root	shoot	2983.091144	402.4500095	1692.770577	2.895598637	8.15E-280	7.70E-278
Cladopus_024097	root	shoot	910.759445	4288.754419	2599.756932	-2.235242902	1.60E-279	1.51E-277
Cladopus_024247	root	shoot	3919.818882	606.9302405	2263.374561	2.693296751	4.33E-279	4.05E-277
Cladopus_021744	root	shoot	6623.328379	1267.531093	3945.429736	2.386169676	4.36E-278	4.06E-276

Cladopus_008960	root	shoot	15170.84782	4460.640792	9815.744308	1.765839844	4.97E-277	4.61E-275
Cladopus_006614	root	shoot	2937.428064	562.0824269	1749.755245	2.384250937	1.65E-275	1.53E-273
Cladopus_023866	root	shoot	3082.722095	536.2910272	1809.506561	2.523082861	1.59E-271	1.46E-269
Cladopus_010093	root	shoot	2136.25245	7678.5794	4907.415925	-1.846229672	2.14E-271	1.95E-269
Cladopus_021319	root	shoot	354.3780588	2424.869991	1389.624025	-2.775575907	1.09E-270	9.91E-269
Cladopus_016819	root	shoot	2729.40199	431.480245	1580.441118	2.662241978	1.43E-268	1.29E-266
Cladopus_003432	root	shoot	1432.045369	5976.007733	3704.026551	-2.06219431	1.52E-268	1.37E-266
Cladopus_014043	root	shoot	4381.413388	750.0517366	2565.732562	2.548341934	2.01E-268	1.81E-266
Cladopus_002217	root	shoot	49738.30868	11565.54846	30651.92857	2.104441103	3.56E-268	3.18E-266
Cladopus_005345	root	shoot	6323.696728	28542.14978	17432.92326	-2.174597344	3.01E-267	2.68E-265
Cladopus_009810	root	shoot	3862.885447	857.5191366	2360.202292	2.172982908	7.75E-267	6.86E-265
Cladopus_014125	root	shoot	1193.652392	5524.243536	3358.947964	-2.211505499	1.41E-266	1.24E-264
Cladopus_013124	root	shoot	1606.031487	166.7709064	886.4011969	3.259938375	2.77E-266	2.43E-264
Cladopus_018391	root	shoot	1204.039604	5131.791054	3167.915329	-2.092488464	1.47E-265	1.28E-263
Cladopus_010321	root	shoot	3153.350196	494.5524687	1823.951332	2.669703694	2.37E-265	2.06E-263
Cladopus_007692	root	shoot	75840.09835	17994.96003	46917.52919	2.075392995	4.43E-263	3.83E-261
Cladopus_020076	root	shoot	3922.165591	586.343299	2254.254445	2.742694957	2.95E-262	2.54E-260
Cladopus_001265	root	shoot	30282.60621	9283.340114	19782.97316	1.705641548	4.11E-262	3.52E-260
Cladopus_018665	root	shoot	4549.9585	1156.617996	2853.288248	1.976005475	1.01E-261	8.64E-260
Cladopus_023363	root	shoot	3267.968019	698.4482733	1983.208146	2.227149948	1.50E-261	1.27E-259
Cladopus_011210	root	shoot	6797.330803	1670.663455	4233.997129	2.026068493	5.25E-261	4.44E-259
Cladopus_008301	root	shoot	6860.929671	1873.569911	4367.249791	1.872505306	1.47E-259	1.24E-257
Cladopus_022873	root	shoot	6047.865542	1464.330076	3756.097809	2.047156141	3.92E-258	3.29E-256
Cladopus_007901	root	shoot	3588.713217	783.7225683	2186.217892	2.195052403	9.31E-256	7.78E-254
Cladopus_013143	root	shoot	7565.224386	2297.177673	4931.20103	1.719058057	9.58E-256	7.97E-254
Cladopus_010042	root	shoot	62972.23259	20594.28851	41783.26055	1.61254132	3.50E-255	2.90E-253
Cladopus_016166	root	shoot	853.5262985	3871.777741	2362.65202	-2.180232892	9.59E-255	7.91E-253
Cladopus_022085	root	shoot	4388.289362	612.5799302	2500.434646	2.84231962	3.97E-253	3.26E-251
Cladopus_023455	root	shoot	1306.807933	68.40634953	687.6071413	4.253108162	8.85E-253	7.24E-251
Cladopus_004346	root	shoot	6989.21493	1917.245911	4453.23042	1.867253767	1.86E-251	1.52E-249

Cladopus_006657	root	shoot	16077.42257	5478.686827	10778.0547	1.553358284	5.17E-250	4.19E-248
Cladopus_012649	root	shoot	4728.905064	1243.546198	2986.225631	1.926613113	1.13E-247	9.12E-246
Cladopus_006948	root	shoot	3584.765892	598.6684755	2091.717184	2.583130869	4.51E-247	3.63E-245
Cladopus_016877	root	shoot	1505.356708	36.6920266	771.0243673	5.380917157	6.27E-247	5.02E-245
Cladopus_005377	root	shoot	7068.436906	1574.086221	4321.261563	2.168154475	6.76E-247	5.39E-245
Cladopus_000246	root	shoot	16122.37021	5269.035733	10695.70297	1.613229968	7.41E-247	5.89E-245
Cladopus_019973	root	shoot	1778.527166	261.8313565	1020.179261	2.764141435	1.69E-245	1.33E-243
Cladopus_008915	root	shoot	3570.466617	834.4861862	2202.476402	2.094735272	3.24E-245	2.55E-243
Cladopus_007696	root	shoot	6320.079012	1014.830047	3667.454529	2.639298044	3.31E-245	2.60E-243
Cladopus_012208	root	shoot	9506.890787	2742.960799	6124.925793	1.793001797	3.70E-245	2.90E-243
Cladopus_019329	root	shoot	1984.281493	175.8633201	1080.072407	3.494130676	4.73E-245	3.69E-243
Cladopus_000380	root	shoot	39284.11804	12633.54613	25958.83208	1.636620644	1.57E-244	1.22E-242
Cladopus_021273	root	shoot	15024.83165	4948.178902	9986.505278	1.602229158	2.19E-243	1.69E-241
Cladopus_000666	root	shoot	1260.741993	32.5023468	646.6221699	5.275862403	3.06E-243	2.35E-241
Cladopus_007127	root	shoot	8301.997933	2421.519471	5361.758702	1.77750059	5.31E-243	4.07E-241
Cladopus_013568	root	shoot	1761.859953	115.7090368	938.7844949	3.923633234	8.70E-243	6.64E-241
Cladopus_015538	root	shoot	9876.373802	3098.750709	6487.562256	1.672203572	1.15E-241	8.77E-240
Cladopus_019282	root	shoot	10122.57626	3369.067321	6745.82179	1.58690114	9.68E-241	7.33E-239
Cladopus_024951	root	shoot	3176.670929	327.8545352	1752.262732	3.279217055	2.22E-239	1.68E-237
Cladopus_006079	root	shoot	46462.24743	11886.16062	29174.20402	1.966668128	2.42E-239	1.82E-237
Cladopus_019995	root	shoot	5979.63839	1507.648087	3743.643238	1.987868362	4.89E-239	3.66E-237
Cladopus_019972	root	shoot	5256.438212	1299.832361	3278.135287	2.014858027	7.04E-238	5.25E-236
Cladopus_019513	root	shoot	3817.759603	710.0365021	2263.898053	2.430993318	2.36E-237	1.76E-235
Cladopus_016885	root	shoot	1376.779936	5228.434729	3302.607333	-1.925696187	1.87E-235	1.38E-233
Cladopus_016027	root	shoot	2509.213866	504.0184754	1506.616171	2.31578468	3.36E-235	2.48E-233
Cladopus_023515	root	shoot	475.2081028	2451.32678	1463.267442	-2.367495319	1.61E-233	1.18E-231
Cladopus_018881	root	shoot	6457.563681	1753.18779	4105.375736	1.88105151	1.26E-232	9.23E-231
Cladopus_024061	root	shoot	680.3482852	3366.206966	2023.277626	-2.308865632	1.72E-232	1.25E-230
Cladopus_003148	root	shoot	5655.903161	1621.347316	3638.625238	1.802266375	3.05E-231	2.21E-229
Cladopus_004348	root	shoot	58284.89515	20052.43966	39168.6674	1.539369872	7.06E-230	5.11E-228

Cladopus_021457	root	shoot	13038.65088	45390.45691	29214.5539	-1.799761733	1.16E-228	8.36E-227
Cladopus_016114	root	shoot	152.6328836	1641.683394	897.1581386	-3.4229757	2.18E-227	1.56E-225
Cladopus_016029	root	shoot	2769.341172	545.2273622	1657.284267	2.344131374	3.55E-227	2.54E-225
Cladopus_002484	root	shoot	856.0880679	3914.090425	2385.089246	-2.191199907	4.41E-227	3.14E-225
Cladopus_024622	root	shoot	4909.3164	942.0485555	2925.682478	2.382975275	1.20E-226	8.56E-225
Cladopus_011074	root	shoot	6367.73295	1152.624953	3760.178951	2.465026361	1.23E-226	8.69E-225
Cladopus_002677	root	shoot	16752.43024	6078.685873	11415.55806	1.462572729	1.45E-226	1.02E-224
Cladopus_025042	root	shoot	1754.815224	281.0846188	1017.949921	2.640100304	4.18E-226	2.94E-224
Cladopus_018270	root	shoot	6651.753412	1687.96854	4169.860976	1.978876001	1.47E-225	1.03E-223
Cladopus_013059	root	shoot	1597.747942	5899.914947	3748.831445	-1.885625519	1.82E-225	1.27E-223
Cladopus_006425	root	shoot	856.8268291	3791.58735	2324.20709	-2.146966704	1.99E-225	1.38E-223
Cladopus_012842	root	shoot	3033.280054	10257.17636	6645.228205	-1.758061653	3.04E-225	2.10E-223
Cladopus_001454	root	shoot	8205.065251	2547.245351	5376.155301	1.688040517	2.22E-224	1.53E-222
Cladopus_003421	root	shoot	385.1287638	2411.520714	1398.324739	-2.643063074	2.38E-224	1.64E-222
Cladopus_019804	root	shoot	3648.008947	649.9788716	2148.99391	2.491489452	3.31E-224	2.27E-222
Cladopus_006315	root	shoot	3594.462946	849.1050619	2221.784004	2.081199735	1.81E-222	1.24E-220
Cladopus_001305	root	shoot	3633.653011	526.7004768	2080.176744	2.788588528	1.35E-221	9.18E-220
Cladopus_018885	root	shoot	5992.525684	1301.081163	3646.803423	2.203949239	2.63E-221	1.79E-219
Cladopus_018334	root	shoot	408.5984079	2181.977891	1295.28815	-2.417323156	6.74E-221	4.56E-219
Cladopus_009484	root	shoot	5284.372204	18089.15217	11686.76218	-1.775662244	1.09E-220	7.36E-219
Cladopus_025091	root	shoot	1294.173173	5478.584315	3386.378744	-2.082237046	1.15E-220	7.69E-219
Cladopus_004287	root	shoot	23879.86929	5460.426677	14670.14798	2.128955971	5.19E-220	3.47E-218
Cladopus_020773	root	shoot	1087.630461	46.77058077	567.2005207	4.536597852	6.18E-220	4.12E-218
Cladopus_013687	root	shoot	2838.287017	600.2454118	1719.266214	2.238415442	2.16E-217	1.44E-215
Cladopus_011310	root	shoot	266.5913149	1840.924061	1053.757688	-2.78859013	2.30E-217	1.53E-215
Cladopus_012895	root	shoot	1677.884159	85.31906589	881.6016126	4.286350149	4.00E-217	2.64E-215
Cladopus_012831	root	shoot	1757.673909	218.2511399	987.9625243	3.013053923	1.04E-214	6.85E-213
Cladopus_018876	root	shoot	2493.532744	455.0347895	1474.283767	2.457591899	3.86E-214	2.53E-212
Cladopus_007909	root	shoot	3966.090918	1140.666844	2553.378881	1.797840871	1.52E-213	9.92E-212
Cladopus_021905	root	shoot	809.2779006	3252.179662	2030.728781	-2.005769229	3.80E-213	2.47E-211

Cladopus_006617	root	shoot	15640.58542	4745.40403	10192.99472	1.721053465	5.22E-213	3.38E-211
Cladopus_004286	root	shoot	23884.06474	5468.627233	14676.34599	2.1270485	5.43E-212	3.51E-210
Cladopus_001968	root	shoot	1306.737454	63.73732696	685.2373905	4.366150861	1.14E-211	7.34E-210
Cladopus_022825	root	shoot	7220.997566	2042.114557	4631.556061	1.821764763	2.01E-211	1.29E-209
Cladopus_013924	root	shoot	5260.823497	1634.891985	3447.857741	1.685674056	2.36E-210	1.51E-208
Cladopus_010306	root	shoot	1203.933892	87.50982166	645.7218566	3.780894399	7.68E-210	4.90E-208
Cladopus_019479	root	shoot	2217.273476	329.6317361	1273.452606	2.750482844	1.18E-209	7.53E-208
Cladopus_001473	root	shoot	1067.403177	3929.307357	2498.355267	-1.879395877	1.30E-209	8.21E-208
Cladopus_016115	root	shoot	7069.4196	1762.848209	4416.133904	2.003967354	1.55E-209	9.80E-208
Cladopus_021418	root	shoot	4011.273214	1152.012618	2581.642916	1.798840272	2.85E-209	1.80E-207
Cladopus_016394	root	shoot	1110.160021	4291.117788	2700.638904	-1.951281583	1.62E-208	1.02E-206
Cladopus_015480	root	shoot	2353.279572	502.5434388	1427.911505	2.227184226	2.23E-208	1.40E-206
Cladopus_002274	root	shoot	16876.53626	6006.318811	11441.42753	1.490552023	2.74E-208	1.71E-206
Cladopus_002547	root	shoot	959.7058419	52.0491971	505.8775195	4.205794615	2.77E-206	1.72E-204
Cladopus_019594	root	shoot	20166.31365	5335.995243	12751.15445	1.918339242	1.65E-205	1.02E-203
Cladopus_025150	root	shoot	4032.545744	1144.691265	2588.618504	1.815788587	2.82E-205	1.74E-203
Cladopus_019432	root	shoot	4645.346156	758.9802943	2702.163225	2.616497387	3.25E-205	2.00E-203
Cladopus_016049	root	shoot	13991.46496	3308.544644	8650.004802	2.080375608	3.62E-204	2.23E-202
Cladopus_016853	root	shoot	624.0279893	2974.764381	1799.396185	-2.253237319	4.22E-204	2.59E-202
Cladopus_004043	root	shoot	901.3754686	3700.496475	2300.935972	-2.038698633	5.13E-204	3.13E-202
Cladopus_020492	root	shoot	1372.857752	210.9969097	791.9273307	2.700504944	9.58E-204	5.83E-202
Cladopus_007102	root	shoot	1747.87912	330.2146885	1039.046904	2.406072204	1.28E-202	7.79E-201
Cladopus_008583	root	shoot	93.97553613	1315.489716	704.7326261	-3.815288065	2.57E-202	1.55E-200
Cladopus_025624	root	shoot	248.629506	1690.0864	969.3579529	-2.762683257	3.11E-201	1.88E-199
Cladopus_004596	root	shoot	2405.410872	551.7966573	1478.603765	2.124566246	7.99E-201	4.80E-199
Cladopus_011149	root	shoot	1467.042158	216.8599562	841.9510572	2.756088832	8.40E-201	5.03E-199
Cladopus_015185	root	shoot	15383.49583	6027.637096	10705.56646	1.351733281	3.48E-200	2.08E-198
Cladopus_012938	root	shoot	1983.977998	405.934543	1194.95627	2.287152074	4.51E-200	2.68E-198
Cladopus_018341	root	shoot	10204.68174	3618.66496	6911.673348	1.495616969	5.50E-200	3.27E-198
Cladopus_017078	root	shoot	13188.79324	4340.457695	8764.625465	1.603327433	1.04E-199	6.16E-198

Cladopus_006385	root	shoot	1097.471309	120.7747362	609.1230228	3.183498747	1.76E-199	1.04E-197
Cladopus_002247	root	shoot	15759.01326	5386.980801	10572.99703	1.548524164	1.94E-199	1.14E-197
Cladopus_001466	root	shoot	1421.221374	196.8194817	809.0204277	2.845885518	3.23E-199	1.90E-197
Cladopus_009306	root	shoot	35416.38806	10313.94062	22865.16434	1.779955829	6.52E-199	3.81E-197
Cladopus_011811	root	shoot	25784.58991	10163.80564	17974.19777	1.343095116	2.04E-198	1.19E-196
Cladopus_009916	root	shoot	4948.902221	1064.655627	3006.778924	2.217363173	2.65E-198	1.54E-196
Cladopus_025376	root	shoot	182.6207852	1391.206017	786.9134011	-2.928678177	3.12E-198	1.81E-196
Cladopus_017210	root	shoot	4629.147357	1417.064165	3023.105761	1.708361819	5.93E-198	3.42E-196
Cladopus_003589	root	shoot	56183.28375	20130.11201	38156.69788	1.480772156	1.16E-196	6.70E-195
Cladopus_025811	root	shoot	1001.486696	77.68333286	539.5850146	3.670187377	1.72E-195	9.90E-194
Cladopus_025632	root	shoot	4636.914525	837.2585659	2737.086545	2.470727556	2.95E-195	1.69E-193
Cladopus_009370	root	shoot	3840.118125	857.073907	2348.596016	2.164502216	3.67E-195	2.10E-193
Cladopus_020746	root	shoot	485.3571097	2565.480566	1525.418838	-2.399798937	6.48E-195	3.69E-193
Cladopus_013247	root	shoot	7645.399484	20464.44404	14054.92176	-1.420628256	9.08E-195	5.15E-193
Cladopus_015564	root	shoot	2622.54421	650.3675537	1636.455882	2.013592955	1.59E-194	8.99E-193
Cladopus_023069	root	shoot	8587.509597	30130.23636	19358.87298	-1.810642638	1.88E-194	1.06E-192
Cladopus_022062	root	shoot	885.1675636	56.01159949	470.5895816	3.988240165	3.94E-194	2.22E-192
Cladopus_002538	root	shoot	98.54327696	1328.580854	713.5620654	-3.756853304	6.83E-194	3.83E-192
Cladopus_016391	root	shoot	837.9307493	3379.05971	2108.49523	-2.011729434	8.81E-194	4.93E-192
Cladopus_021836	root	shoot	4056.585738	463.1959024	2259.89082	3.132909387	9.07E-194	5.06E-192
Cladopus_001996	root	shoot	1653.786314	5977.074009	3815.430161	-1.85480566	1.62E-193	9.01E-192
Cladopus_012964	root	shoot	7444.690383	2470.884081	4957.787232	1.591895385	6.94E-193	3.85E-191
Cladopus_012282	root	shoot	1041.569995	25.89538103	533.7326879	5.300040981	9.90E-193	5.47E-191
Cladopus_004633	root	shoot	1245.735164	15.06410333	630.3996339	6.388795711	1.71E-192	9.42E-191
Cladopus_019921	root	shoot	47580.01865	8729.9309	28154.97478	2.446385518	1.96E-192	1.08E-190
Cladopus_009118	root	shoot	22094.64049	7872.654641	14983.64756	1.488719009	6.24E-192	3.42E-190
Cladopus_008630	root	shoot	3009.528037	692.4083554	1850.968196	2.120953179	1.07E-190	5.86E-189
Cladopus_004106	root	shoot	3645.053559	857.684167	2251.368863	2.089367098	5.76E-190	3.14E-188
Cladopus_006259	root	shoot	285.4615298	1815.962156	1050.711843	-2.666936214	7.86E-190	4.28E-188
Cladopus_019592	root	shoot	12761.24086	3426.293905	8093.767384	1.897447099	2.02E-189	1.09E-187

Cladopus_015175	root	shoot	706.468969	2770.289021	1738.378995	-1.97147285	2.14E-189	1.16E-187
Cladopus_024067	root	shoot	1674.139697	131.7138453	902.9267709	3.668251278	6.02E-189	3.25E-187
Cladopus_001505	root	shoot	638.2958037	3116.673416	1877.48461	-2.287300777	9.02E-189	4.85E-187
Cladopus_023853	root	shoot	2712.298134	10756.98279	6734.640464	-1.988292804	1.82E-188	9.78E-187
Cladopus_009722	root	shoot	228.913667	1796.694808	1012.804237	-2.968974343	6.34E-188	3.39E-186
Cladopus_000106	root	shoot	1087.289991	16.80948224	552.0497364	6.033954758	3.00E-187	1.60E-185
Cladopus_013796	root	shoot	3554.789999	950.5631974	2252.676598	1.903668025	3.09E-187	1.64E-185
Cladopus_013780	root	shoot	1616.151751	232.2604199	924.2060855	2.799680264	2.33E-186	1.24E-184
Cladopus_005254	root	shoot	19215.25249	7136.934944	13176.09371	1.428849842	1.51E-185	7.97E-184
Cladopus_020721	root	shoot	1033.477588	5299.821482	3166.649535	-2.360308418	1.54E-185	8.12E-184
Cladopus_001043	root	shoot	1249.023452	4131.351803	2690.187627	-1.725279706	2.08E-185	1.09E-183
Cladopus_001787	root	shoot	2324.386519	594.0998085	1459.243164	1.966357116	3.41E-185	1.79E-183
Cladopus_004635	root	shoot	1171.616977	14.48980989	593.0533934	6.356669813	1.39E-184	7.30E-183
Cladopus_019965	root	shoot	1677.992763	5473.961531	3575.977147	-1.706383471	1.98E-184	1.03E-182
Cladopus_003707	root	shoot	4535.434163	1400.000908	2967.717535	1.695059378	8.24E-184	4.29E-182
Cladopus_016623	root	shoot	794.0720945	3594.487877	2194.279986	-2.180729155	2.67E-183	1.39E-181
Cladopus_002976	root	shoot	483.2320177	2104.221353	1293.726686	-2.122719021	4.30E-183	2.22E-181
Cladopus_023168	root	shoot	1467.998092	4693.64944	3080.823766	-1.677522732	8.94E-183	4.62E-181
Cladopus_025142	root	shoot	9610.262519	2128.580424	5869.421472	2.174684281	1.57E-182	8.07E-181
Cladopus_003577	root	shoot	4776.788168	12563.50889	8670.14853	-1.395431235	1.67E-182	8.57E-181
Cladopus_005899	root	shoot	3228.254555	979.9006363	2104.077595	1.720757082	3.98E-182	2.04E-180
Cladopus_001389	root	shoot	6024.665763	2082.647234	4053.656498	1.532181282	6.75E-182	3.45E-180
Cladopus_007919	root	shoot	5252.622402	1634.309259	3443.46583	1.683584296	2.30E-181	1.17E-179
Cladopus_016437	root	shoot	5676.797855	17459.47385	11568.13585	-1.621160171	9.12E-181	4.63E-179
Cladopus_024592	root	shoot	1388.174214	4972.701088	3180.437651	-1.841585376	2.86E-180	1.45E-178
Cladopus_004579	root	shoot	10388.78696	2921.412222	6655.099591	1.83063316	7.65E-180	3.87E-178
Cladopus_013447	root	shoot	1160.973475	3752.861605	2456.91754	-1.693043179	1.28E-179	6.44E-178
Cladopus_017518	root	shoot	936.6611076	84.94207936	510.8015935	3.469977177	1.96E-179	9.85E-178
Cladopus_020233	root	shoot	8262.417787	2387.064453	5324.74112	1.792035561	5.48E-179	2.75E-177
Cladopus_002932	root	shoot	54159.90612	20618.84475	37389.37544	1.393325327	1.29E-178	6.44E-177

Cladopus_000660	root	shoot	3525.477955	1005.255541	2265.366748	1.807904631	1.42E-178	7.07E-177
Cladopus_011667	root	shoot	1517.594616	312.0828509	914.8387336	2.281360108	1.15E-176	5.74E-175
Cladopus_014483	root	shoot	1759.479058	302.4533764	1030.966217	2.542240079	1.37E-176	6.78E-175
Cladopus_025185	root	shoot	12217.57834	4403.89055	8310.734443	1.471998657	4.94E-176	2.45E-174
Cladopus_023629	root	shoot	1342.275426	4326.310162	2834.292794	-1.689311394	5.77E-175	2.85E-173
Cladopus_016046	root	shoot	55776.78111	13503.205	34639.99305	2.046474848	5.97E-175	2.94E-173
Cladopus_021262	root	shoot	3204.31391	9487.152271	6345.73309	-1.566363081	1.63E-174	8.03E-173
Cladopus_006039	root	shoot	13184.13198	5191.02935	9187.580667	1.34472682	2.38E-174	1.17E-172
Cladopus_013821	root	shoot	1692.704065	288.1326714	990.418368	2.553303077	4.39E-174	2.15E-172
Cladopus_009764	root	shoot	6257.048463	2304.989107	4281.018785	1.440428458	4.04E-173	1.97E-171
Cladopus_003375	root	shoot	82912.61669	34695.24564	58803.93116	1.25688466	1.48E-172	7.22E-171
Cladopus_005441	root	shoot	8307.358712	3256.690153	5782.024432	1.350745824	1.52E-172	7.38E-171
Cladopus_023880	root	shoot	1913.624759	378.3366382	1145.980699	2.342736743	1.56E-172	7.56E-171
Cladopus_013187	root	shoot	867.4918794	55.83701947	461.6644494	3.975166829	7.60E-172	3.67E-170
Cladopus_007553	root	shoot	3121.26863	979.2569936	2050.262812	1.67181977	4.31E-171	2.08E-169
Cladopus_018307	root	shoot	14651.48938	5566.590754	10109.04007	1.396387551	1.30E-170	6.26E-169
Cladopus_002772	root	shoot	11073.584	3754.652052	7414.118025	1.560748575	1.60E-170	7.68E-169
Cladopus_000271	root	shoot	2898.087157	8158.740056	5528.413606	-1.492722128	1.05E-169	5.04E-168
Cladopus_017254	root	shoot	5932.074628	1671.60331	3801.838969	1.82633174	1.23E-169	5.87E-168
Cladopus_011200	root	shoot	172.3758363	1350.281767	761.3288014	-2.969176141	3.33E-169	1.58E-167
Cladopus_021413	root	shoot	1396.10232	242.7333576	819.4178387	2.51834376	3.93E-169	1.87E-167
Cladopus_006135	root	shoot	802.1659709	40.98198297	421.5739769	4.279371061	4.87E-169	2.31E-167
Cladopus_005323	root	shoot	249.7312971	1471.309297	860.5202969	-2.555903982	1.89E-168	8.92E-167
Cladopus_011110	root	shoot	3544.307175	9583.265921	6563.786548	-1.434909044	2.32E-168	1.09E-166
Cladopus_013132	root	shoot	5699.676464	1854.648196	3777.16233	1.618773501	7.57E-168	3.56E-166
Cladopus_022048	root	shoot	1381.929112	226.0395542	803.9843333	2.614904383	2.88E-167	1.35E-165
Cladopus_019091	root	shoot	17188.673	7245.279096	12216.97605	1.246417079	3.80E-167	1.78E-165
Cladopus_025380	root	shoot	3343.177489	1005.864991	2174.52124	1.732978069	4.58E-167	2.14E-165
Cladopus_018183	root	shoot	9076.680201	2617.585794	5847.132997	1.794397307	9.87E-167	4.60E-165
Cladopus_009875	root	shoot	18734.66963	7579.442151	13157.05589	1.305427563	1.99E-166	9.24E-165

Cladopus_000854	root	shoot	2476.169562	585.8929639	1531.031263	2.075493197	2.30E-166	1.07E-164
Cladopus_020540	root	shoot	19952.9538	4616.398491	12284.67615	2.112059047	4.20E-166	1.94E-164
Cladopus_016467	root	shoot	741.0774849	41.598756	391.3381205	4.144703511	2.57E-165	1.19E-163
Cladopus_001543	root	shoot	18120.67724	6179.245769	12149.96151	1.552556825	7.53E-165	3.46E-163
Cladopus_008839	root	shoot	3423.158833	1030.008773	2226.583803	1.732513665	1.19E-164	5.48E-163
Cladopus_027202	root	shoot	5805.720457	1638.015801	3721.868129	1.825969854	1.46E-164	6.70E-163
Cladopus_023837	root	shoot	20432.72549	8807.838982	14620.28224	1.214134653	2.59E-164	1.18E-162
Cladopus_018086	root	shoot	1490.98243	230.9510926	860.9667614	2.691751668	4.95E-164	2.26E-162
Cladopus_000769	root	shoot	20821.809	4811.130443	12816.46972	2.113735982	4.98E-164	2.27E-162
Cladopus_007378	root	shoot	13424.94295	30291.50703	21858.22499	-1.173958104	5.13E-164	2.33E-162
Cladopus_018493	root	shoot	349.2202437	1755.194998	1052.207621	-2.32783014	3.68E-163	1.67E-161
Cladopus_003756	root	shoot	1486.606954	4555.786148	3021.196551	-1.615272022	4.56E-162	2.06E-160
Cladopus_004604	root	shoot	2024.162702	517.4752944	1270.818998	1.967281494	6.68E-162	3.01E-160
Cladopus_006369	root	shoot	93.09537038	988.9117917	541.003581	-3.403547618	2.93E-161	1.32E-159
Cladopus_013457	root	shoot	2183.403852	6574.424503	4378.914177	-1.590320381	6.63E-161	2.97E-159
Cladopus_006159	root	shoot	588.0389872	2747.793426	1667.916207	-2.227012489	1.07E-160	4.80E-159
Cladopus_011870	root	shoot	2335.211294	562.5702834	1448.890789	2.05365743	1.12E-160	5.01E-159
Cladopus_009302	root	shoot	2607.960081	698.4896467	1653.224864	1.901080473	3.10E-160	1.38E-158
Cladopus_022152	root	shoot	37314.18534	14975.45827	26144.8218	1.317129849	1.94E-159	8.61E-158
Cladopus_023460	root	shoot	6987.945254	2670.514026	4829.22964	1.387149225	2.35E-159	1.04E-157
Cladopus_011408	root	shoot	16044.91183	6619.524254	11332.21804	1.277360799	2.41E-159	1.06E-157
Cladopus_015569	root	shoot	2553.86948	629.2100235	1591.539752	2.023008249	4.82E-159	2.13E-157
Cladopus_022405	root	shoot	2025.925625	509.9283726	1267.926999	1.992640396	7.42E-159	3.27E-157
Cladopus_008561	root	shoot	686.1952432	2477.899776	1582.04751	-1.852947789	9.10E-159	4.00E-157
Cladopus_009332	root	shoot	3952.179328	1317.094763	2634.637045	1.586192791	1.04E-158	4.57E-157
Cladopus_017267	root	shoot	5700.233422	1817.265844	3758.749633	1.649528806	2.06E-158	9.01E-157
Cladopus_002377	root	shoot	29853.2486	9129.113595	19491.1811	1.709307809	2.26E-158	9.85E-157
Cladopus_003268	root	shoot	2828.040443	664.9878389	1746.514141	2.089150443	1.25E-157	5.44E-156
Cladopus_018131	root	shoot	11153.91573	4114.286569	7634.101148	1.439233518	1.39E-157	6.04E-156
Cladopus_020508	root	shoot	3239.70517	970.5027291	2105.10395	1.740819676	1.71E-157	7.42E-156

Cladopus_018208	root	shoot	47.06496345	932.0408224	489.5528929	-4.310011919	3.98E-157	1.72E-155
Cladopus_021757	root	shoot	3408.177145	1127.794829	2267.985987	1.596036731	1.00E-156	4.33E-155
Cladopus_003511	root	shoot	663.251296	2697.235394	1680.243345	-2.022894897	1.11E-156	4.79E-155
Cladopus_011013	root	shoot	1008.584878	144.0412468	576.3130624	2.811756034	1.21E-156	5.20E-155
Cladopus_021459	root	shoot	7075.322921	2756.198404	4915.760662	1.360127895	1.82E-156	7.79E-155
Cladopus_001660	root	shoot	4388.286331	1525.729163	2957.007747	1.525451764	1.84E-156	7.87E-155
Cladopus_016813	root	shoot	249.0794349	1428.700262	838.8898485	-2.516376682	2.53E-156	1.08E-154
Cladopus_005592	root	shoot	164.8355265	1178.970228	671.902877	-2.837852171	4.48E-156	1.91E-154
Cladopus_004632	root	shoot	786.2471675	24.1903377	405.2187526	5.032658147	4.50E-156	1.91E-154
Cladopus_020309	root	shoot	2751.531511	697.6380474	1724.584779	1.98292241	5.39E-156	2.29E-154
Cladopus_022952	root	shoot	1272.767812	5022.710949	3147.73938	-1.982192897	3.45E-155	1.46E-153
Cladopus_015140	root	shoot	2655.967351	769.4769139	1712.722133	1.787582052	6.55E-155	2.77E-153
Cladopus_025561	root	shoot	9217.467102	3237.377553	6227.422328	1.510010381	8.45E-155	3.56E-153
Cladopus_016095	root	shoot	7078.907974	16767.85043	11923.3792	-1.244243463	1.57E-154	6.61E-153
Cladopus_014507	root	shoot	2057.434291	5913.154175	3985.294233	-1.523169106	4.23E-154	1.77E-152
Cladopus_014508	root	shoot	2057.597845	5902.110126	3979.853985	-1.520385918	5.30E-154	2.22E-152
Cladopus_018448	root	shoot	2455.983392	659.2637004	1557.623546	1.89938614	1.04E-153	4.33E-152
Cladopus_001486	root	shoot	389.8132564	1657.470084	1023.64167	-2.088488625	1.51E-153	6.28E-152
Cladopus_012115	root	shoot	10559.54733	3292.022012	6925.78467	1.681854616	3.31E-153	1.37E-151
Cladopus_021433	root	shoot	1338.166783	275.6547345	806.9107587	2.282032217	5.11E-153	2.12E-151
Cladopus_020979	root	shoot	1386.172092	288.3963553	837.2842234	2.269201859	8.45E-153	3.50E-151
Cladopus_002426	root	shoot	189.2793142	1379.0261	784.1527069	-2.870268931	1.32E-152	5.45E-151
Cladopus_026296	root	shoot	2434.976434	699.215648	1567.096041	1.79888354	3.12E-152	1.29E-150
Cladopus_019591	root	shoot	12491.90714	3252.394098	7872.150621	1.94166299	4.13E-152	1.70E-150
Cladopus_001427	root	shoot	703.5155973	49.39131307	376.4534552	3.825564518	8.66E-152	3.56E-150
Cladopus_012161	root	shoot	5644.691346	2102.061529	3873.376437	1.424433881	1.69E-151	6.93E-150
Cladopus_015878	root	shoot	4030.633746	1325.255498	2677.944622	1.604174579	2.42E-151	9.90E-150
Cladopus_023399	root	shoot	4646.970877	1637.50473	3142.237804	1.505939468	3.43E-151	1.40E-149
Cladopus_021998	root	shoot	3435.450244	9187.563882	6311.507063	-1.419573931	3.62E-151	1.47E-149
Cladopus_009917	root	shoot	3780.59024	843.3322361	2311.961238	2.164441292	3.94E-151	1.60E-149

Cladopus_016216	root	shoot	4931.835534	1582.884979	3257.360256	1.640367497	4.36E-151	1.77E-149
Cladopus_024641	root	shoot	6942.522411	2443.369116	4692.945763	1.506805506	4.77E-151	1.93E-149
Cladopus_017583	root	shoot	3118.855303	960.289553	2039.572428	1.699107979	7.46E-151	3.01E-149
Cladopus_020745	root	shoot	809.4074009	14.66824	412.0378204	5.79499913	2.84E-150	1.15E-148
Cladopus_007447	root	shoot	28261.12442	12078.62919	20169.87681	1.226212006	1.26E-149	5.08E-148
Cladopus_007431	root	shoot	1698.051199	401.7066073	1049.878903	2.079318344	1.41E-149	5.65E-148
Cladopus_016967	root	shoot	71834.00933	25962.40226	48898.2058	1.468190792	4.73E-149	1.89E-147
Cladopus_012015	root	shoot	4133.252128	1198.655182	2665.953655	1.788317645	5.10E-149	2.04E-147
Cladopus_009448	root	shoot	10594.19537	2818.087788	6706.141581	1.911011221	9.45E-149	3.77E-147
Cladopus_015141	root	shoot	2657.520802	793.8503433	1725.685573	1.742974455	3.16E-148	1.26E-146
Cladopus_008434	root	shoot	674.2705563	42.92429519	358.5974257	3.990148367	4.74E-148	1.88E-146
Cladopus_005448	root	shoot	4585.547713	1748.909154	3167.228433	1.390504491	5.80E-148	2.30E-146
Cladopus_020921	root	shoot	2451.963163	448.3369515	1450.150057	2.455024435	6.22E-148	2.46E-146
Cladopus_016305	root	shoot	115.3360749	1032.426204	573.8811394	-3.165011422	7.45E-148	2.94E-146
Cladopus_010148	root	shoot	1165.8625	3859.981034	2512.921767	-1.727476083	1.39E-147	5.47E-146
Cladopus_008680	root	shoot	2690.577121	686.9373864	1688.757254	1.96924071	2.43E-147	9.55E-146
Cladopus_007159	root	shoot	698.6530656	43.5503915	371.1017286	3.979431352	6.81E-147	2.67E-145
Cladopus_021420	root	shoot	8333.076193	24278.28557	16305.68088	-1.542998073	1.72E-146	6.72E-145
Cladopus_005633	root	shoot	8429.137004	3532.808167	5980.972586	1.254460936	5.10E-146	1.99E-144
Cladopus_005904	root	shoot	1975.130732	483.8736479	1229.50219	2.029464599	5.15E-146	2.01E-144
Cladopus_000725	root	shoot	1500.712687	265.8759098	883.2942985	2.505563629	6.36E-146	2.47E-144
Cladopus_004449	root	shoot	2445.183611	609.996495	1527.590053	2.003372629	8.76E-146	3.40E-144
Cladopus_001921	root	shoot	2542.429988	779.9720449	1661.201016	1.704645025	1.70E-145	6.59E-144
Cladopus_017294	root	shoot	680.834615	31.17066818	356.0026416	4.481877455	4.16E-145	1.61E-143
Cladopus_006134	root	shoot	1347.702426	3869.843133	2608.772779	-1.520993824	4.85E-145	1.87E-143
Cladopus_022549	root	shoot	3635.655954	1213.459871	2424.557912	1.582800726	6.01E-145	2.32E-143
Cladopus_003063	root	shoot	6550.607814	1448.653874	3999.630844	2.177208591	1.29E-144	4.98E-143
Cladopus_000152	root	shoot	728.7277571	54.16024057	391.4439989	3.733159147	2.25E-144	8.65E-143
Cladopus_016595	root	shoot	2721.3397	751.6827671	1736.511233	1.855546133	3.99E-144	1.53E-142
Cladopus_005757	root	shoot	11310.29048	3270.283083	7290.286782	1.790855952	4.69E-144	1.79E-142

Cladopus_009541	root	shoot	1016.860599	74.22787536	545.5442373	3.769602052	5.68E-144	2.17E-142
Cladopus_000949	root	shoot	7030.320549	2837.835563	4934.078056	1.309150706	7.25E-143	2.76E-141
Cladopus_010549	root	shoot	965.7892706	3170.691927	2068.240599	-1.713605241	1.27E-142	4.82E-141
Cladopus_023503	root	shoot	188.1616757	1142.905115	665.5333952	-2.606792156	1.29E-142	4.89E-141
Cladopus_005212	root	shoot	2491.364241	651.4809065	1571.422574	1.936543215	1.42E-142	5.38E-141
Cladopus_020330	root	shoot	690.0155462	61.88449025	375.9500182	3.486993713	1.22E-141	4.60E-140
Cladopus_010309	root	shoot	1459.669135	245.132514	852.4008245	2.568500148	1.85E-141	6.98E-140
Cladopus_014090	root	shoot	695.9746706	65.16166493	380.5681678	3.430666491	2.19E-141	8.24E-140
Cladopus_006367	root	shoot	65.81458594	806.2706949	436.0426404	-3.610296717	2.42E-141	9.09E-140
Cladopus_006969	root	shoot	3411.654742	1143.251093	2277.452917	1.578206464	7.33E-141	2.75E-139
Cladopus_016458	root	shoot	10681.37047	3368.390526	7024.880499	1.665088863	8.55E-141	3.20E-139
Cladopus_020047	root	shoot	1163.023417	3416.042759	2289.533088	-1.554139187	1.08E-140	4.03E-139
Cladopus_021279	root	shoot	1581.704216	320.5133472	951.1087817	2.302761168	1.50E-140	5.61E-139
Cladopus_012029	root	shoot	1220.730785	104.5010632	662.615924	3.53773672	1.64E-140	6.12E-139
Cladopus_025037	root	shoot	16495.01503	7268.495962	11881.75549	1.182324437	4.62E-140	1.72E-138
Cladopus_001839	root	shoot	654.3665322	2369.244927	1511.80573	-1.856462026	2.48E-139	9.21E-138
Cladopus_012095	root	shoot	1319.289252	4244.47607	2781.882661	-1.687220619	2.75E-139	1.02E-137
Cladopus_009760	root	shoot	100024.1395	46282.17531	73153.15742	1.111853393	4.91E-139	1.81E-137
Cladopus_015731	root	shoot	1244.956137	3685.818611	2465.387374	-1.566475553	5.02E-139	1.85E-137
Cladopus_019258	root	shoot	930.4569562	2887.3677	1908.912328	-1.634796708	5.67E-139	2.09E-137
Cladopus_009606	root	shoot	3140.472724	872.5152232	2006.493974	1.847542489	1.14E-138	4.20E-137
Cladopus_024256	root	shoot	2005.743905	528.4333446	1267.088625	1.92331477	2.82E-138	1.03E-136
Cladopus_002724	root	shoot	5978.422136	2132.554364	4055.48825	1.487026264	2.96E-138	1.08E-136
Cladopus_021002	root	shoot	4243.689991	1545.76601	2894.728	1.457003708	9.33E-138	3.41E-136
Cladopus_008848	root	shoot	1925.072158	5134.733052	3529.902605	-1.41574774	1.54E-137	5.60E-136
Cladopus_015137	root	shoot	6335.519568	2512.634916	4424.077242	1.334297955	1.72E-137	6.25E-136
Cladopus_001877	root	shoot	10901.87291	4193.828567	7547.850737	1.378344571	3.27E-137	1.19E-135
Cladopus_015820	root	shoot	692.4471731	54.28938542	373.3682793	3.682996569	9.29E-137	3.37E-135
Cladopus_014625	root	shoot	4640.02442	1395.326121	3017.67527	1.733523017	1.25E-136	4.52E-135
Cladopus_009256	root	shoot	1158.220201	146.448396	652.3342986	2.987385957	2.15E-136	7.75E-135

Cladopus_018471	root	shoot	2326.662282	679.7511769	1503.20673	1.778362978	2.44E-136	8.79E-135
Cladopus_005423	root	shoot	2424.167893	652.7724759	1538.470184	1.892329745	4.43E-136	1.60E-134
Cladopus_023607	root	shoot	868.4314773	140.7587442	504.5951107	2.630090821	6.47E-136	2.32E-134
Cladopus_017841	root	shoot	9081.923991	20267.94247	14674.93323	-1.158183197	1.92E-135	6.89E-134
Cladopus_006959	root	shoot	6492.25598	2237.829182	4365.042581	1.536449976	2.99E-135	1.07E-133
Cladopus_005252	root	shoot	2790.137007	825.588273	1807.86264	1.758693672	3.57E-135	1.27E-133
Cladopus_012140	root	shoot	14656.2892	6742.709971	10699.49959	1.12007087	3.82E-135	1.36E-133
Cladopus_004612	root	shoot	1077.574943	3247.127431	2162.351187	-1.59212981	9.62E-135	3.42E-133
Cladopus_009635	root	shoot	3258.499596	1125.599557	2192.049576	1.533327128	1.22E-134	4.34E-133
Cladopus_011603	root	shoot	976.8221358	124.4878815	550.6550086	2.964069507	5.70E-134	2.02E-132
Cladopus_009935	root	shoot	13192.69591	5916.384219	9554.540065	1.156585134	1.22E-133	4.30E-132
Cladopus_019466	root	shoot	11061.12027	3265.534173	7163.327223	1.759763361	1.24E-133	4.39E-132
Cladopus_003369	root	shoot	14935.941	6315.427309	10625.68416	1.241522808	1.29E-133	4.56E-132
Cladopus_020032	root	shoot	739.3584955	91.481402	415.4199487	3.015317811	1.39E-133	4.91E-132
Cladopus_007152	root	shoot	2166.463103	637.6692221	1402.066163	1.762844109	1.40E-133	4.93E-132
Cladopus_011539	root	shoot	90.58820508	816.9273945	453.7577998	-3.177158853	1.42E-133	4.96E-132
Cladopus_000184	root	shoot	7406.061913	2731.766739	5068.914326	1.43945549	7.66E-133	2.68E-131
Cladopus_009589	root	shoot	5409.207656	2089.182565	3749.19511	1.372670866	7.80E-133	2.72E-131
Cladopus_021300	root	shoot	858.9333515	2717.046464	1787.989908	-1.660307808	9.69E-133	3.38E-131
Cladopus_017957	root	shoot	14508.35173	37945.13475	26226.74324	-1.386895299	1.64E-132	5.69E-131
Cladopus_013112	root	shoot	1503.18381	4024.887548	2764.035679	-1.421148163	1.75E-132	6.08E-131
Cladopus_007143	root	shoot	664.8243101	62.89779284	363.8610515	3.416803194	2.58E-132	8.94E-131
Cladopus_018505	root	shoot	3685.532798	1352.724943	2519.12887	1.445598176	6.31E-132	2.19E-130
Cladopus_012866	root	shoot	27559.11591	12573.64675	20066.38133	1.13216225	8.64E-132	2.99E-130
Cladopus_018432	root	shoot	2379.137393	6377.031934	4378.084664	-1.422861056	1.01E-131	3.50E-130
Cladopus_015486	root	shoot	763.0521272	2542.47634	1652.764234	-1.73525577	1.20E-131	4.12E-130
Cladopus_022771	root	shoot	2075.504449	594.3353733	1334.919911	1.805829216	1.37E-131	4.71E-130
Cladopus_012463	root	shoot	1375.641506	354.2505725	864.9460394	1.957269992	1.88E-131	6.46E-130
Cladopus_010012	root	shoot	1972.869474	4992.171418	3482.520446	-1.339785816	2.56E-131	8.78E-130
Cladopus_014809	root	shoot	682.9270181	13.04549094	347.9862545	5.700436477	3.15E-131	1.08E-129

Cladopus_002141	root	shoot	1486.777816	288.437874	887.6078451	2.365069714	7.00E-131	2.39E-129
Cladopus_019920	root	shoot	4946.150273	1538.386265	3242.268269	1.686244625	2.35E-130	8.01E-129
Cladopus_010168	root	shoot	7444.755684	2710.403679	5077.579682	1.457851075	6.61E-130	2.25E-128
Cladopus_009677	root	shoot	625.3937522	32.39355654	328.8936544	4.247172375	6.73E-130	2.29E-128
Cladopus_005018	root	shoot	220.4742467	1122.471591	671.4729188	-2.345494834	8.50E-130	2.88E-128
Cladopus_017398	root	shoot	1302.573352	3599.007425	2450.790388	-1.466241881	1.25E-129	4.22E-128
Cladopus_000851	root	shoot	3335.620321	7672.745948	5504.183134	-1.202013098	2.05E-129	6.93E-128
Cladopus_003694	root	shoot	4881.624645	1799.063176	3340.343911	1.43912679	3.52E-129	1.19E-127
Cladopus_018237	root	shoot	13794.7889	5192.418399	9493.603647	1.410029548	5.13E-129	1.73E-127
Cladopus_017422	root	shoot	1148.520601	3774.394436	2461.457519	-1.717627343	6.51E-129	2.19E-127
Cladopus_005217	root	shoot	2372.215769	774.7766154	1573.496192	1.614075618	7.65E-129	2.57E-127
Cladopus_013236	root	shoot	320.8172394	1415.702288	868.2597636	-2.138529865	1.33E-128	4.47E-127
Cladopus_002883	root	shoot	4557.514112	1119.353301	2838.433707	2.025827084	7.45E-128	2.49E-126
Cladopus_002080	root	shoot	558.4795983	1986.026061	1272.25283	-1.830683082	9.83E-128	3.28E-126
Cladopus_006480	root	shoot	6261.058844	2632.550426	4446.804635	1.249746251	3.89E-127	1.30E-125
Cladopus_006584	root	shoot	46085.61668	19494.64469	32790.13068	1.241148682	6.61E-127	2.20E-125
Cladopus_003493	root	shoot	38950.59697	12342.75377	25646.67537	1.658043581	1.33E-126	4.43E-125
Cladopus_006933	root	shoot	12417.65686	5828.645812	9123.151335	1.091167865	1.46E-126	4.85E-125
Cladopus_001173	root	shoot	1146.075746	3927.422491	2536.749118	-1.775278807	2.20E-126	7.29E-125
Cladopus_008473	root	shoot	1806.400143	553.2604364	1179.83029	1.705524506	4.89E-126	1.62E-124
Cladopus_015761	root	shoot	3365.804398	810.9039664	2088.354182	2.05398453	7.06E-126	2.33E-124
Cladopus_017176	root	shoot	15166.20948	5678.09152	10422.1505	1.417700352	1.47E-125	4.86E-124
Cladopus_019359	root	shoot	1874.083775	4838.465988	3356.274881	-1.367805252	4.70E-125	1.55E-123
Cladopus_010123	root	shoot	852.656405	144.7789436	498.7176743	2.553724998	1.98E-124	6.51E-123
Cladopus_004616	root	shoot	2598.434839	817.6553301	1708.045085	1.669974085	3.56E-124	1.17E-122
Cladopus_018476	root	shoot	5380.07389	1739.84153	3559.95771	1.628183949	4.24E-124	1.39E-122
Cladopus_011779	root	shoot	5356.868641	2159.60389	3758.236266	1.311166313	4.74E-124	1.55E-122
Cladopus_003095	root	shoot	658.4604369	49.49492079	353.9776789	3.717832016	7.02E-124	2.29E-122
Cladopus_021312	root	shoot	1491.016209	371.3426135	931.1794113	2.007710816	1.04E-123	3.39E-122
Cladopus_000273	root	shoot	1032.703782	2911.865612	1972.284697	-1.496260113	2.44E-123	7.94E-122

Cladopus_024718	root	shoot	5127.035255	1494.847337	3310.941296	1.778478467	2.87E-123	9.32E-122
Cladopus_026680	root	shoot	854.1932649	144.110513	499.151889	2.562536759	3.87E-123	1.25E-121
Cladopus_002085	root	shoot	853.7076614	3584.110322	2218.908992	-2.072193498	5.61E-123	1.81E-121
Cladopus_002822	root	shoot	57915.94411	22511.21397	40213.57904	1.363303047	5.73E-123	1.85E-121
Cladopus_013231	root	shoot	95.71408506	824.8857955	460.2999403	-3.112006073	7.96E-123	2.56E-121
Cladopus_009642	root	shoot	1039.65475	210.9432534	625.2990017	2.296103763	9.08E-123	2.92E-121
Cladopus_020488	root	shoot	21924.37928	6963.786667	14444.08297	1.654435071	1.32E-122	4.24E-121
Cladopus_005178	root	shoot	768.2482064	101.0632954	434.6557509	2.919709686	2.95E-122	9.47E-121
Cladopus_011508	root	shoot	34438.33106	16400.15119	25419.24112	1.070288343	4.80E-122	1.54E-120
Cladopus_009347	root	shoot	2742.450497	903.6403752	1823.045436	1.602622125	6.15E-122	1.97E-120
Cladopus_004532	root	shoot	1420.117486	236.6014466	828.3594661	2.580091428	3.67E-121	1.17E-119
Cladopus_002957	root	shoot	11047.15032	4660.362711	7853.756516	1.245521762	4.02E-121	1.28E-119
Cladopus_000293	root	shoot	23297.12979	7939.440468	15618.28513	1.553202347	4.33E-121	1.38E-119
Cladopus_011209	root	shoot	1506.493897	420.0475117	963.2707044	1.841890639	7.09E-121	2.25E-119
Cladopus_012108	root	shoot	581.4805038	44.9449043	313.212704	3.669094252	8.04E-121	2.55E-119
Cladopus_016079	root	shoot	145.1361561	886.5270208	515.8315885	-2.607807269	8.05E-121	2.55E-119
Cladopus_003789	root	shoot	4395.038341	1781.360802	3088.199572	1.302934711	1.39E-120	4.40E-119
Cladopus_000111	root	shoot	1153.503733	217.03387	685.2688015	2.405338635	1.66E-120	5.23E-119
Cladopus_001878	root	shoot	4170.469935	1526.593332	2848.531634	1.448869918	5.21E-120	1.64E-118
Cladopus_003483	root	shoot	590.7245685	26.37620356	308.550386	4.466363342	6.86E-120	2.16E-118
Cladopus_017611	root	shoot	1766.075425	539.0940417	1152.584733	1.714613623	1.01E-119	3.16E-118
Cladopus_024064	root	shoot	4670.146198	1981.273555	3325.709876	1.236916361	1.52E-119	4.76E-118
Cladopus_015161	root	shoot	1194.061966	297.06922	745.5655932	2.007401197	1.74E-119	5.46E-118
Cladopus_008286	root	shoot	3134.805371	884.5242467	2009.664809	1.825932991	2.08E-119	6.51E-118
Cladopus_018316	root	shoot	1446.187271	375.6790448	910.9331578	1.944610194	4.17E-119	1.30E-117
Cladopus_019064	root	shoot	160.3527164	1065.058428	612.7055723	-2.73468175	4.54E-119	1.41E-117
Cladopus_011468	root	shoot	5710.205587	2517.579088	4113.892338	1.181501493	1.05E-118	3.26E-117
Cladopus_020009	root	shoot	10955.702	4813.267445	7884.484723	1.18634958	1.11E-118	3.44E-117
Cladopus_023567	root	shoot	2258.207173	5850.423895	4054.315534	-1.374107614	1.76E-118	5.45E-117
Cladopus_017738	root	shoot	5896.203699	2347.675911	4121.939805	1.329105937	4.74E-118	1.47E-116

Cladopus_024744	root	shoot	851.802993	86.00703949	468.9050163	3.318987599	4.82E-118	1.49E-116
Cladopus_023881	root	shoot	17986.28139	8143.895532	13065.08846	1.142999894	2.98E-117	9.20E-116
Cladopus_009942	root	shoot	5389.505207	2241.889646	3815.697427	1.266186105	3.26E-117	1.00E-115
Cladopus_006055	root	shoot	1459.093638	398.576707	928.8351727	1.872819371	3.44E-117	1.06E-115
Cladopus_009274	root	shoot	1480.081627	252.9323956	866.5070114	2.539154202	3.53E-117	1.08E-115
Cladopus_016042	root	shoot	2193.303385	567.1066885	1380.205037	1.954163267	4.49E-117	1.38E-115
Cladopus_002207	root	shoot	4858.623738	1818.186405	3338.405072	1.417694815	4.97E-117	1.52E-115
Cladopus_015556	root	shoot	1651.787779	496.7863722	1074.287076	1.734526956	6.38E-117	1.95E-115
Cladopus_022632	root	shoot	135.3241685	879.1458727	507.2350206	-2.699928224	1.19E-116	3.64E-115
Cladopus_011525	root	shoot	34866.05174	14549.35853	24707.70514	1.260915824	4.93E-116	1.50E-114
Cladopus_006658	root	shoot	4217.778681	1724.627786	2971.203233	1.290948785	5.76E-116	1.75E-114
Cladopus_004776	root	shoot	1061.522176	224.0606112	642.7913936	2.247659367	7.95E-116	2.41E-114
Cladopus_004913	root	shoot	1043.768968	3193.834034	2118.801501	-1.614410271	1.16E-115	3.53E-114
Cladopus_011946	root	shoot	16079.15478	7750.257675	11914.70623	1.052710844	1.17E-115	3.53E-114
Cladopus_007925	root	shoot	8992.452991	3979.368956	6485.910974	1.176168106	1.23E-115	3.73E-114
Cladopus_015860	root	shoot	2484.3381	863.0780827	1673.708092	1.524661537	1.47E-115	4.44E-114
Cladopus_014254	root	shoot	505.6614104	27.09163004	266.3765202	4.245197514	1.70E-115	5.13E-114
Cladopus_011273	root	shoot	90.0902388	769.5117577	429.8009982	-3.102583895	2.18E-115	6.54E-114
Cladopus_008870	root	shoot	756.9581674	126.4002232	441.6791953	2.590939381	2.36E-115	7.08E-114
Cladopus_019535	root	shoot	744.4328744	2473.385569	1608.909222	-1.733538545	3.33E-115	9.98E-114
Cladopus_019905	root	shoot	2166.811736	5680.692113	3923.751924	-1.391215755	8.54E-115	2.56E-113
Cladopus_020434	root	shoot	323.6626491	1284.838793	804.2507208	-1.990080138	1.33E-114	3.99E-113
Cladopus_010024	root	shoot	2077.138498	723.0173036	1400.077901	1.523265159	1.67E-114	4.98E-113
Cladopus_008077	root	shoot	7378.516917	2774.342233	5076.429575	1.411832796	2.18E-114	6.51E-113
Cladopus_008880	root	shoot	80862.51513	38824.58828	59843.55171	1.058488676	5.28E-114	1.57E-112
Cladopus_003126	root	shoot	558.9802932	50.57852945	304.7794113	3.450319572	6.15E-114	1.83E-112
Cladopus_003484	root	shoot	1890.829773	4795.460937	3343.145355	-1.342121238	7.23E-114	2.15E-112
Cladopus_005528	root	shoot	1127.815531	3102.534466	2115.174999	-1.459721391	7.26E-114	2.15E-112
Cladopus_001545	root	shoot	15923.34505	4796.358663	10359.85186	1.731524232	9.58E-114	2.83E-112
Cladopus_005014	root	shoot	10596.80962	5067.677191	7832.243404	1.064294865	2.17E-113	6.41E-112

Cladopus_016949	root	shoot	830.9130432	150.4805855	490.6968144	2.471588916	3.98E-113	1.17E-111
Cladopus_017605	root	shoot	1611.371938	490.0826063	1050.727272	1.71958243	4.89E-113	1.44E-111
Cladopus_025072	root	shoot	1678.962454	516.8283226	1097.895388	1.698025384	7.16E-113	2.11E-111
Cladopus_019178	root	shoot	2481.550998	916.1914304	1698.871214	1.438772904	1.57E-112	4.59E-111
Cladopus_008357	root	shoot	8151.350005	3606.644353	5878.997179	1.176490094	1.75E-112	5.14E-111
Cladopus_003792	root	shoot	1384.292646	3639.409764	2511.851205	-1.39398815	1.79E-112	5.23E-111
Cladopus_004014	root	shoot	124.9074948	778.7723194	451.8399071	-2.643072622	2.89E-112	8.44E-111
Cladopus_006331	root	shoot	1282.753132	4083.120231	2682.936681	-1.672082432	3.29E-112	9.61E-111
Cladopus_002122	root	shoot	1404.107515	3913.109461	2658.608488	-1.479654193	5.65E-112	1.65E-110
Cladopus_025060	root	shoot	1900.880099	581.0388548	1240.959477	1.712119015	7.25E-112	2.11E-110
Cladopus_007242	root	shoot	4497.979632	1865.342799	3181.661215	1.270732481	8.57E-112	2.49E-110
Cladopus_021925	root	shoot	21766.63533	8361.053418	15063.84437	1.380170482	1.07E-111	3.10E-110
Cladopus_002958	root	shoot	10796.55273	4649.606694	7723.079711	1.21575691	1.43E-111	4.15E-110
Cladopus_001794	root	shoot	527.7854787	27.74821775	277.7668482	4.228060907	2.02E-111	5.84E-110
Cladopus_013564	root	shoot	9505.467762	4200.552814	6853.010288	1.177692837	2.14E-111	6.18E-110
Cladopus_007306	root	shoot	697.2385287	84.15605417	390.6972914	3.0571104	2.47E-111	7.13E-110
Cladopus_023409	root	shoot	3158.104128	1136.384299	2147.244213	1.477145356	2.89E-111	8.33E-110
Cladopus_021042	root	shoot	388.4253382	1600.010168	994.2177532	-2.045438279	4.74E-111	1.36E-109
Cladopus_017168	root	shoot	908.6001777	119.0373481	513.8187629	2.918153177	5.68E-111	1.63E-109
Cladopus_014451	root	shoot	37877.81067	15814.91522	26846.36295	1.260160399	8.80E-111	2.52E-109
Cladopus_014291	root	shoot	1434.480596	412.6426798	923.5616378	1.797577344	9.65E-111	2.76E-109
Cladopus_005957	root	shoot	3123.534488	1242.327081	2182.930784	1.331023756	1.24E-110	3.55E-109
Cladopus_026095	root	shoot	983.9332261	137.9177685	560.9254973	2.842050972	1.30E-110	3.71E-109
Cladopus_003096	root	shoot	3204.989667	1193.329709	2199.159688	1.424536267	1.69E-110	4.81E-109
Cladopus_017497	root	shoot	59.23826313	641.5662727	350.4022679	-3.438339635	2.12E-110	6.03E-109
Cladopus_009377	root	shoot	6522.509774	13853.73955	10188.12466	-1.08693518	2.35E-110	6.67E-109
Cladopus_007557	root	shoot	1021.880387	2741.660639	1881.770513	-1.424258281	3.95E-110	1.12E-108
Cladopus_011216	root	shoot	1655.380414	391.7693315	1023.574873	2.079237416	4.71E-110	1.33E-108
Cladopus_012030	root	shoot	5049.378794	1611.222081	3330.300438	1.649364393	1.86E-109	5.26E-108
Cladopus_019244	root	shoot	2141.015446	778.0351416	1459.525294	1.460356449	4.07E-109	1.15E-107

Cladopus_023512	root	shoot	3100.984998	1162.645941	2131.815469	1.415588086	5.15E-109	1.45E-107
Cladopus_013056	root	shoot	1430.4803	345.9312368	888.2057684	2.047937632	5.20E-109	1.47E-107
Cladopus_016856	root	shoot	2546.860183	917.7759196	1732.318051	1.471507105	5.55E-109	1.56E-107
Cladopus_018039	root	shoot	1396.696344	379.8119655	888.2541547	1.8784014	6.48E-109	1.82E-107
Cladopus_000264	root	shoot	936.5498089	200.88187	568.7158394	2.222045184	8.95E-109	2.51E-107
Cladopus_021782	root	shoot	2759.720696	1038.230198	1898.975447	1.408287686	1.07E-108	3.01E-107
Cladopus_002441	root	shoot	142.0167506	838.7055498	490.3611502	-2.560026643	1.29E-108	3.60E-107
Cladopus_011344	root	shoot	1859.330497	4509.420677	3184.375587	-1.278895353	2.69E-108	7.51E-107
Cladopus_006205	root	shoot	1231.921746	341.8002485	786.8609974	1.849907964	3.07E-108	8.57E-107
Cladopus_017342	root	shoot	2943.759254	1112.035278	2027.897266	1.403975833	5.33E-108	1.48E-106
Cladopus_017138	root	shoot	1112.57804	2941.151259	2026.86465	-1.402992384	8.12E-108	2.26E-106
Cladopus_000202	root	shoot	3784.654913	1270.800874	2527.727894	1.575082797	1.07E-107	2.97E-106
Cladopus_013283	root	shoot	8970.826502	3750.311183	6360.568842	1.258567581	1.09E-107	3.03E-106
Cladopus_004424	root	shoot	4855.067926	1871.430828	3363.249377	1.375514383	1.30E-107	3.61E-106
Cladopus_005046	root	shoot	2352.041114	673.0311308	1512.536122	1.806778348	1.68E-107	4.64E-106
Cladopus_005950	root	shoot	361.6732235	1347.803336	854.7382797	-1.900176427	1.87E-107	5.16E-106
Cladopus_018225	root	shoot	4719.697874	1877.027517	3298.362695	1.33037067	4.94E-107	1.36E-105
Cladopus_001340	root	shoot	1396.939398	3404.755891	2400.847645	-1.284782732	6.39E-107	1.76E-105
Cladopus_001064	root	shoot	1788.580336	4355.591865	3072.086101	-1.28479477	7.38E-107	2.03E-105
Cladopus_010245	root	shoot	24.93630729	593.3279175	309.1321124	-4.596825336	8.77E-107	2.41E-105
Cladopus_000639	root	shoot	645.3441897	32.1131438	338.7286668	4.323594698	9.89E-107	2.71E-105
Cladopus_005305	root	shoot	1068.550912	210.605189	639.5780506	2.34258701	1.02E-106	2.79E-105
Cladopus_019161	root	shoot	1527.00308	4770.445736	3148.724408	-1.644726985	1.10E-106	3.00E-105
Cladopus_008593	root	shoot	2103.044489	4948.723442	3525.883966	-1.234171054	1.33E-106	3.62E-105
Cladopus_016004	root	shoot	3084.061559	1061.195424	2072.628492	1.538611295	2.49E-106	6.78E-105
Cladopus_004834	root	shoot	1061.078433	2936.163041	1998.620737	-1.468709035	2.92E-106	7.94E-105
Cladopus_021444	root	shoot	1228.995964	287.2239373	758.1099505	2.098653771	3.42E-106	9.31E-105
Cladopus_022562	root	shoot	584.2440802	1890.751442	1237.497761	-1.692962054	4.77E-106	1.30E-104
Cladopus_017035	root	shoot	4020.329105	1608.926736	2814.627921	1.320513345	5.57E-106	1.51E-104
Cladopus_008552	root	shoot	700.8219629	2550.852423	1625.837193	-1.865778939	5.64E-106	1.53E-104

Cladopus_013768	root	shoot	5989.488024	1829.223407	3909.355716	1.710790758	1.12E-105	3.04E-104
Cladopus_002007	root	shoot	7911.709475	3467.121868	5689.415671	1.189978056	1.18E-105	3.19E-104
Cladopus_016747	root	shoot	642.0986401	101.9911198	372.04488	2.65710768	1.31E-105	3.52E-104
Cladopus_021422	root	shoot	2224.73439	5011.675413	3618.204902	-1.172195093	2.01E-105	5.42E-104
Cladopus_014108	root	shoot	2166.310313	802.0226991	1484.166506	1.434129885	2.21E-105	5.94E-104
Cladopus_001436	root	shoot	5338.600869	2189.98114	3764.291005	1.28571984	2.24E-105	6.01E-104
Cladopus_001844	root	shoot	3217.64571	1314.603919	2266.124814	1.291293031	2.27E-105	6.09E-104
Cladopus_006175	root	shoot	594.1416172	1959.59019	1276.865904	-1.723048918	3.90E-105	1.04E-103
Cladopus_000913	root	shoot	7131.714994	2862.76389	4997.239442	1.316633863	4.52E-105	1.21E-103
Cladopus_019509	root	shoot	4022.658324	9221.355416	6622.00687	-1.197165985	5.61E-105	1.50E-103
Cladopus_020486	root	shoot	4165.170309	1680.319192	2922.74475	1.309059436	7.32E-105	1.95E-103
Cladopus_010622	root	shoot	647.1586499	1868.101101	1257.629875	-1.530057229	1.67E-104	4.45E-103
Cladopus_017799	root	shoot	1027.061261	183.7136762	605.3874686	2.485303822	1.69E-104	4.50E-103
Cladopus_014659	root	shoot	3160.801204	6884.237912	5022.519558	-1.12302616	3.06E-104	8.13E-103
Cladopus_004942	root	shoot	677.3678064	106.1536374	391.7607219	2.667162221	3.08E-104	8.16E-103
Cladopus_012937	root	shoot	2502.889859	958.8719397	1730.880899	1.38265753	4.86E-104	1.29E-102
Cladopus_001461	root	shoot	3298.147161	662.9427325	1980.544947	2.315971747	1.06E-103	2.79E-102
Cladopus_014316	root	shoot	1133.183726	234.4875138	683.8356201	2.274971787	1.08E-103	2.84E-102
Cladopus_003603	root	shoot	1055.840889	3004.574575	2030.207732	-1.508133569	1.11E-103	2.94E-102
Cladopus_022944	root	shoot	1888.695638	4385.279955	3136.987796	-1.215565346	1.18E-103	3.12E-102
Cladopus_004361	root	shoot	3261.482465	1333.414616	2297.44854	1.290950847	1.35E-103	3.55E-102
Cladopus_021025	root	shoot	121.812725	802.0633273	461.9380262	-2.718264185	1.44E-103	3.78E-102
Cladopus_017047	root	shoot	3956.38555	1661.881713	2809.133632	1.251347231	1.70E-103	4.45E-102
Cladopus_002715	root	shoot	4649.610815	1542.486156	3096.048486	1.591168799	1.97E-103	5.17E-102
Cladopus_018656	root	shoot	1361.471272	391.7713282	876.6212999	1.793570186	2.10E-103	5.49E-102
Cladopus_025372	root	shoot	12268.88445	5510.397991	8889.641219	1.154469461	5.19E-103	1.35E-101
Cladopus_000373	root	shoot	532.2872378	1766.759101	1149.523169	-1.729002467	1.17E-102	3.06E-101
Cladopus_007070	root	shoot	567.8493398	1812.84193	1190.345635	-1.6738424	1.26E-102	3.29E-101
Cladopus_008909	root	shoot	1831.388071	4329.749852	3080.568961	-1.241861824	1.90E-102	4.95E-101
Cladopus_001298	root	shoot	89.27247147	651.1811127	370.2267921	-2.86429071	2.45E-102	6.37E-101

Cladopus_022719	root	shoot	1439.29845	458.6461766	948.9723135	1.649435008	2.69E-102	6.98E-101
Cladopus_009728	root	shoot	530.0367384	49.87213559	289.954437	3.408790789	5.84E-102	1.51E-100
Cladopus_023454	root	shoot	2034.173148	715.3948932	1374.784021	1.509638536	6.45E-102	1.67E-100
Cladopus_021216	root	shoot	13544.64603	6718.075167	10131.3606	1.011575842	6.99E-102	1.81E-100
Cladopus_001488	root	shoot	2519.731166	6411.57449	4465.652828	-1.348226348	7.28E-102	1.88E-100
Cladopus_022101	root	shoot	28.50873665	539.9240223	284.2163795	-4.253863347	8.50E-102	2.19E-100
Cladopus_024929	root	shoot	10804.63294	5188.36236	7996.497652	1.058533997	9.92E-102	2.55E-100
Cladopus_019904	root	shoot	2121.509463	759.8175995	1440.663531	1.481666602	1.02E-101	2.61E-100
Cladopus_016291	root	shoot	2282.938975	857.990113	1570.464544	1.412050009	3.57E-101	9.17E-100
Cladopus_016962	root	shoot	521.3100708	54.11110065	287.7105857	3.249899179	4.01E-101	1.03E-99
Cladopus_014681	root	shoot	1758.618313	507.0074729	1132.812893	1.796440225	5.35E-101	1.37E-99
Cladopus_011912	root	shoot	2619.947916	902.3699847	1761.15895	1.539056573	5.87E-101	1.50E-99
Cladopus_010538	root	shoot	4406.804803	1936.606687	3171.705745	1.185335828	6.65E-101	1.70E-99
Cladopus_019169	root	shoot	4004.62691	1359.077394	2681.852152	1.559165156	9.61E-101	2.45E-99
Cladopus_018854	root	shoot	2645.785904	993.7868485	1819.786376	1.413412924	1.15E-100	2.94E-99
Cladopus_017872	root	shoot	490.7825956	1745.05968	1117.921138	-1.832577878	1.27E-100	3.24E-99
Cladopus_022803	root	shoot	1475.089578	3912.496607	2693.793092	-1.408341606	1.38E-100	3.51E-99
Cladopus_012121	root	shoot	1187.289785	324.5330276	755.9114061	1.869250395	1.71E-100	4.33E-99
Cladopus_014330	root	shoot	685.0283186	2393.014159	1539.021239	-1.807146092	2.92E-100	7.40E-99
Cladopus_003939	root	shoot	1795.994629	4995.417728	3395.706178	-1.474851392	1.01E-99	2.54E-98
Cladopus_011746	root	shoot	1712.462248	4035.722597	2874.092422	-1.237116083	1.35E-99	3.41E-98
Cladopus_010213	root	shoot	1967.791254	4461.491142	3214.641198	-1.181663314	1.46E-99	3.67E-98
Cladopus_025031	root	shoot	688.6942268	2438.080249	1563.387238	-1.825204613	1.65E-99	4.15E-98
Cladopus_013545	root	shoot	1007.707308	2692.914822	1850.311065	-1.416768051	1.66E-99	4.18E-98
Cladopus_024138	root	shoot	1165.116064	291.0169422	728.0665031	2.007881332	3.18E-99	7.98E-98
Cladopus_005398	root	shoot	436.4911884	29.90477933	233.1979838	3.885732327	5.96E-99	1.49E-97
Cladopus_017340	root	shoot	1047.771912	3313.01712	2180.394516	-1.658762072	7.69E-99	1.93E-97
Cladopus_020504	root	shoot	6239.609654	2959.59563	4599.602642	1.076551447	7.79E-99	1.95E-97
Cladopus_013805	root	shoot	5687.135845	2586.897214	4137.01653	1.136982268	8.64E-99	2.16E-97
Cladopus_004681	root	shoot	292.4850529	1138.102655	715.2938541	-1.959531626	9.94E-99	2.48E-97

Cladopus_003433	root	shoot	197.3013723	919.4656436	558.383508	-2.218707575	1.07E-98	2.68E-97
Cladopus_000532	root	shoot	295.7598407	1123.191675	709.4757579	-1.924375086	1.48E-98	3.68E-97
Cladopus_024645	root	shoot	1614.31741	426.22398	1020.270695	1.921168766	1.51E-98	3.75E-97
Cladopus_009964	root	shoot	3254.772306	1326.592816	2290.682561	1.295250325	1.72E-98	4.27E-97
Cladopus_011830	root	shoot	1586.065292	4211.689836	2898.877564	-1.410103191	5.07E-98	1.26E-96
Cladopus_025365	root	shoot	1763.045315	429.67203	1096.358672	2.034570124	7.03E-98	1.74E-96
Cladopus_003106	root	shoot	1813.015229	590.6049119	1201.81007	1.621398141	7.99E-98	1.98E-96
Cladopus_013554	root	shoot	3573.272281	1534.454124	2553.863202	1.220001047	8.71E-98	2.15E-96
Cladopus_004836	root	shoot	932.750179	2471.701106	1702.225643	-1.406738957	9.15E-98	2.26E-96
Cladopus_010257	root	shoot	1892.609677	4525.320032	3208.964854	-1.25739515	9.22E-98	2.27E-96
Cladopus_011770	root	shoot	644.4828613	6.475010669	325.478936	6.559298904	1.11E-97	2.74E-96
Cladopus_000104	root	shoot	471.0025054	12.39356487	241.6980351	5.259007368	1.62E-97	3.99E-96
Cladopus_019021	root	shoot	223.9054042	986.4139009	605.1596525	-2.14392196	2.69E-97	6.59E-96
Cladopus_011321	root	shoot	315.4429909	1177.637808	746.5403992	-1.901840357	3.14E-97	7.70E-96
Cladopus_015535	root	shoot	1777.563929	4066.444075	2922.004002	-1.193356805	3.59E-97	8.78E-96
Cladopus_023636	root	shoot	716.9766622	90.66769567	403.8221789	2.975780928	3.68E-97	8.99E-96
Cladopus_010047	root	shoot	12052.23753	5355.8529	8704.045216	1.170018701	1.12E-96	2.74E-95
Cladopus_020121	root	shoot	7694.899534	3173.237354	5434.068444	1.277230736	1.52E-96	3.70E-95
Cladopus_008288	root	shoot	59208.02696	25494.01943	42351.0232	1.215707104	1.59E-96	3.86E-95
Cladopus_003068	root	shoot	411.7613956	19.52116784	215.6412817	4.386749924	1.84E-96	4.46E-95
Cladopus_018600	root	shoot	2556.460437	949.7529695	1753.106703	1.429057802	2.52E-96	6.12E-95
Cladopus_011620	root	shoot	2050.801149	785.0772664	1417.939208	1.385829383	9.68E-96	2.35E-94
Cladopus_022711	root	shoot	909.7591859	232.3185904	571.0388881	1.967599738	1.62E-95	3.93E-94
Cladopus_004491	root	shoot	4838.068693	10052.95673	7445.512712	-1.055396971	1.91E-95	4.63E-94
Cladopus_007790	root	shoot	421.3695908	1544.654058	983.0118245	-1.874997427	2.02E-95	4.87E-94
Cladopus_020183	root	shoot	2610.662071	7091.361477	4851.011774	-1.442360765	2.04E-95	4.92E-94
Cladopus_007963	root	shoot	24028.45419	8083.426533	16055.94036	1.571988317	2.21E-95	5.32E-94
Cladopus_010260	root	shoot	481.44233	1606.880006	1044.161168	-1.741562648	2.23E-95	5.36E-94
Cladopus_009664	root	shoot	819.5646971	181.246584	500.4056406	2.177968814	5.04E-95	1.21E-93
Cladopus_012737	root	shoot	4960.553383	2132.303272	3546.428328	1.218639441	5.87E-95	1.41E-93

Cladopus_003352	root	shoot	3535.670919	1534.061152	2534.866035	1.205447245	6.49E-95	1.56E-93
Cladopus_009232	root	shoot	502.7628764	46.93850046	274.8506885	3.405779437	6.50E-95	1.56E-93
Cladopus_013700	root	shoot	3891.35186	9146.359194	6518.855527	-1.232372317	6.57E-95	1.57E-93
Cladopus_005875	root	shoot	3188.101291	1309.929566	2249.015428	1.28259516	7.31E-95	1.75E-93
Cladopus_017996	root	shoot	7505.299866	3531.015209	5518.157538	1.088134037	8.04E-95	1.92E-93
Cladopus_017993	root	shoot	416.6292584	1546.945964	981.7876114	-1.889992297	9.33E-95	2.22E-93
Cladopus_010296	root	shoot	6666.085925	3246.547351	4956.316638	1.037610385	9.76E-95	2.32E-93
Cladopus_014996	root	shoot	6768.341006	2335.347702	4551.844354	1.535968488	1.87E-94	4.45E-93
Cladopus_013544	root	shoot	4488.845597	1883.230192	3186.037895	1.254393696	2.18E-94	5.18E-93
Cladopus_009835	root	shoot	929.1880453	160.2392839	544.7136646	2.537529965	2.52E-94	5.97E-93
Cladopus_018645	root	shoot	1105.342933	309.642921	707.492927	1.839546953	2.61E-94	6.19E-93
Cladopus_020276	root	shoot	845.9290306	2373.461964	1609.695497	-1.489212378	2.88E-94	6.81E-93
Cladopus_003280	root	shoot	608.8951256	97.71448215	353.3048039	2.627044397	2.94E-94	6.95E-93
Cladopus_004924	root	shoot	2264.541321	5858.224653	4061.382987	-1.372225971	3.28E-94	7.74E-93
Cladopus_023548	root	shoot	2495.336315	1017.440333	1756.388324	1.293704715	6.18E-94	1.46E-92
Cladopus_021912	root	shoot	581.3266185	69.04776519	325.1871918	3.063368227	6.69E-94	1.57E-92
Cladopus_010307	root	shoot	883.7815151	181.2893563	532.5354357	2.285035347	8.33E-94	1.96E-92
Cladopus_008336	root	shoot	9664.287082	4688.623941	7176.455512	1.043350407	9.68E-94	2.27E-92
Cladopus_007780	root	shoot	942.8767506	3129.622377	2036.249564	-1.732013619	1.22E-93	2.87E-92
Cladopus_017969	root	shoot	1337.815525	404.7323809	871.2739528	1.723254883	1.47E-93	3.45E-92
Cladopus_010196	root	shoot	5608.094777	2411.014708	4009.554743	1.217569938	2.16E-93	5.06E-92
Cladopus_001341	root	shoot	254.5918474	995.347135	624.9694912	-1.966090183	2.66E-93	6.22E-92
Cladopus_001216	root	shoot	7382.749195	3630.431183	5506.590189	1.024395941	4.52E-93	1.05E-91
Cladopus_006465	root	shoot	326.8230303	1309.257661	818.0403457	-2.005045463	5.22E-93	1.22E-91
Cladopus_010656	root	shoot	49.41848118	549.6195978	299.5190395	-3.468501665	7.08E-93	1.65E-91
Cladopus_018814	root	shoot	5782.023361	11988.56731	8885.295333	-1.052255053	1.27E-92	2.95E-91
Cladopus_015698	root	shoot	2246.252305	5143.888205	3695.070255	-1.195127909	1.72E-92	3.99E-91
Cladopus_015122	root	shoot	5877.988567	2681.767605	4279.878086	1.132831446	2.72E-92	6.29E-91
Cladopus_015821	root	shoot	3097.150569	1175.51455	2136.332559	1.398676016	3.25E-92	7.51E-91
Cladopus_007698	root	shoot	2233.694707	789.0786739	1511.38669	1.503479877	5.26E-92	1.21E-90

Cladopus_019259	root	shoot	1204.569137	3447.673694	2326.121415	-1.518259332	5.56E-92	1.28E-90
Cladopus_001359	root	shoot	505.0492923	65.40655103	285.2279217	2.945784342	5.68E-92	1.31E-90
Cladopus_016044	root	shoot	8978.479144	3402.461467	6190.470305	1.40035234	5.82E-92	1.34E-90
Cladopus_003744	root	shoot	4064.501201	1678.827505	2871.664353	1.275168991	7.16E-92	1.64E-90
Cladopus_006303	root	shoot	3671.001399	1578.938639	2624.970019	1.217357698	7.36E-92	1.69E-90
Cladopus_011191	root	shoot	516.5427444	1538.036416	1027.28958	-1.575603977	7.75E-92	1.77E-90
Cladopus_003027	root	shoot	620.0416696	105.5951823	362.8184259	2.546886573	8.93E-92	2.04E-90
Cladopus_013965	root	shoot	8712.785668	4145.085103	6428.935386	1.07153819	1.31E-91	2.99E-90
Cladopus_010028	root	shoot	2593.500451	6479.534273	4536.517362	-1.321868525	1.46E-91	3.34E-90
Cladopus_018712	root	shoot	2631.520466	1047.299154	1839.40981	1.327538217	2.49E-91	5.68E-90
Cladopus_006267	root	shoot	3287.655355	8043.334586	5665.494971	-1.291290385	2.85E-91	6.49E-90
Cladopus_006703	root	shoot	166.5661485	803.6189542	485.0925513	-2.273460808	2.91E-91	6.62E-90
Cladopus_001299	root	shoot	95.80607741	637.6481389	366.7271082	-2.729033495	3.22E-91	7.31E-90
Cladopus_023164	root	shoot	1669.260795	586.0566638	1127.658729	1.509773843	6.12E-91	1.39E-89
Cladopus_023924	root	shoot	3447.962569	1201.050113	2324.506341	1.522888779	6.14E-91	1.39E-89
Cladopus_019806	root	shoot	30.84355618	467.1696248	249.0065905	-3.916997528	9.69E-91	2.19E-89
Cladopus_019193	root	shoot	2029.544233	4388.798914	3209.171574	-1.113099768	1.52E-90	3.43E-89
Cladopus_014779	root	shoot	1842.599749	664.1777583	1253.388753	1.475369008	2.20E-90	4.98E-89
Cladopus_002066	root	shoot	825.5085821	2264.074771	1544.791677	-1.456579723	2.66E-90	6.01E-89
Cladopus_009342	root	shoot	663.241141	2197.924297	1430.582719	-1.730424089	4.59E-90	1.03E-88
Cladopus_006191	root	shoot	532.8516848	63.20551312	298.028599	3.0848476	4.70E-90	1.06E-88
Cladopus_025182	root	shoot	1494.674671	3917.226159	2705.950415	-1.390704315	6.71E-90	1.51E-88
Cladopus_000109	root	shoot	678.1527563	94.1562285	386.1544924	2.836773289	7.49E-90	1.68E-88
Cladopus_019343	root	shoot	2585.102178	1020.031388	1802.566783	1.341596256	9.75E-90	2.19E-88
Cladopus_015879	root	shoot	6085.29295	2946.717762	4516.005356	1.046340061	9.87E-90	2.21E-88
Cladopus_018076	root	shoot	386.5221222	1244.494294	815.5082082	-1.687851939	1.21E-89	2.71E-88
Cladopus_004429	root	shoot	175371.912	80274.0291	127822.9706	1.127426979	1.35E-89	3.01E-88
Cladopus_009062	root	shoot	42278.96413	17292.22927	29785.5967	1.28991153	1.35E-89	3.02E-88
Cladopus_005723	root	shoot	1784.452255	614.2117119	1199.331983	1.541666589	1.44E-89	3.21E-88
Cladopus_016484	root	shoot	1236.178459	3329.442995	2282.810727	-1.430732425	1.50E-89	3.35E-88

Cladopus_013020	root	shoot	557.738588	56.70585786	307.2222229	3.281439765	1.76E-89	3.90E-88
Cladopus_018057	root	shoot	320.1435817	1153.578527	736.8610541	-1.852329715	1.89E-89	4.20E-88
Cladopus_010061	root	shoot	1602.696045	493.3982652	1048.047155	1.70339899	1.90E-89	4.21E-88
Cladopus_012141	root	shoot	3453.958449	1487.670658	2470.814554	1.215015374	3.30E-89	7.30E-88
Cladopus_015959	root	shoot	1404.499769	405.312961	904.9063649	1.792046317	3.96E-89	8.75E-88
Cladopus_013105	root	shoot	756.9079069	134.8361927	445.8720498	2.483068987	4.02E-89	8.88E-88
Cladopus_008918	root	shoot	823.5910091	2145.139689	1484.365349	-1.382310234	6.17E-89	1.36E-87
Cladopus_020564	root	shoot	607.0156882	1704.868162	1155.941925	-1.489299719	7.85E-89	1.73E-87
Cladopus_012668	root	shoot	460.6737393	1475.630996	968.1523677	-1.681247495	1.49E-88	3.28E-87
Cladopus_003669	root	shoot	14792.98801	5680.028696	10236.50835	1.381104837	1.63E-88	3.59E-87
Cladopus_001437	root	shoot	2375.703503	954.1760662	1664.939784	1.316468266	2.21E-88	4.86E-87
Cladopus_005429	root	shoot	767.5209361	1992.09114	1379.806038	-1.37656775	3.30E-88	7.25E-87
Cladopus_018007	root	shoot	764.8893362	2073.793714	1419.341525	-1.440314774	4.31E-88	9.46E-87
Cladopus_014909	root	shoot	670.2579818	1769.625531	1219.941756	-1.401107314	5.20E-88	1.14E-86
Cladopus_003841	root	shoot	1980.476631	610.8117581	1295.644195	1.696996859	7.04E-88	1.54E-86
Cladopus_023016	root	shoot	3931.821498	1543.681316	2737.751407	1.348738026	8.68E-88	1.90E-86
Cladopus_014618	root	shoot	1587.179587	3949.534094	2768.356841	-1.316153997	9.20E-88	2.01E-86
Cladopus_025880	root	shoot	375.1615136	1302.539761	838.8506373	-1.798587383	1.14E-87	2.50E-86
Cladopus_009069	root	shoot	2519.950324	996.5348683	1758.242596	1.336693441	1.33E-87	2.89E-86
Cladopus_005326	root	shoot	2191.23438	752.6202114	1471.927296	1.543823644	1.61E-87	3.51E-86
Cladopus_013370	root	shoot	3290.852294	1469.993896	2380.423095	1.162345216	1.76E-87	3.83E-86
Cladopus_003337	root	shoot	327.3299216	1162.457293	744.8936072	-1.825614405	2.89E-87	6.27E-86
Cladopus_010244	root	shoot	1255.291973	416.8422038	836.0670882	1.59233128	3.74E-87	8.11E-86
Cladopus_017034	root	shoot	799.9180715	205.340267	502.6291692	1.963089843	4.11E-87	8.89E-86
Cladopus_000884	root	shoot	2743.566394	1145.523396	1944.544895	1.259998662	4.74E-87	1.03E-85
Cladopus_013609	root	shoot	407.8788228	1304.315337	856.0970798	-1.676181112	8.64E-87	1.87E-85
Cladopus_024012	root	shoot	486.0683974	69.33883563	277.7036165	2.81265415	9.41E-87	2.03E-85
Cladopus_003376	root	shoot	2308.821541	907.2514716	1608.036506	1.34806844	1.37E-86	2.95E-85
Cladopus_022458	root	shoot	1340.461381	378.1450308	859.3032057	1.824757533	1.57E-86	3.37E-85
Cladopus_021593	root	shoot	1688.352526	423.4741048	1055.913315	1.996817459	1.58E-86	3.40E-85

Cladopus_018551	root	shoot	1021.725371	2727.232474	1874.478923	-1.417259743	1.95E-86	4.18E-85
Cladopus_007538	root	shoot	693.5883419	1808.529915	1251.059129	-1.383619352	2.53E-86	5.43E-85
Cladopus_021522	root	shoot	1652.986872	4154.287271	2903.637071	-1.330834831	2.89E-86	6.19E-85
Cladopus_019122	root	shoot	834.9506344	2113.165828	1474.058231	-1.339218611	3.14E-86	6.72E-85
Cladopus_013945	root	shoot	4523.619908	2149.958652	3336.78928	1.072764923	4.04E-86	8.62E-85
Cladopus_020240	root	shoot	517.5261792	67.14860076	292.33739	2.962151679	6.30E-86	1.35E-84
Cladopus_012728	root	shoot	769.6420468	2262.510108	1516.076077	-1.557587821	1.56E-85	3.32E-84
Cladopus_011941	root	shoot	10533.45776	4241.563433	7387.510595	1.311797097	1.60E-85	3.41E-84
Cladopus_014450	root	shoot	4342.692222	1946.450585	3144.571404	1.159056517	1.65E-85	3.51E-84
Cladopus_018742	root	shoot	2269.671648	5589.972479	3929.822063	-1.300936222	1.89E-85	4.01E-84
Cladopus_002823	root	shoot	1265.408168	416.1540849	840.7811267	1.605829529	2.09E-85	4.42E-84
Cladopus_004422	root	shoot	3430.401071	921.2039925	2175.802532	1.897619272	2.23E-85	4.71E-84
Cladopus_008455	root	shoot	2772.500455	934.7906225	1853.645539	1.569848391	6.15E-85	1.30E-83
Cladopus_015008	root	shoot	6549.316209	3149.70504	4849.510625	1.055949934	1.03E-84	2.17E-83
Cladopus_022210	root	shoot	1305.52105	3208.3664	2256.943725	-1.296025671	1.29E-84	2.72E-83
Cladopus_008230	root	shoot	234.33706	976.0112653	605.1741626	-2.062385228	2.27E-84	4.78E-83
Cladopus_019080	root	shoot	392.9758737	28.59507843	210.7854761	3.762220279	2.33E-84	4.90E-83
Cladopus_016507	root	shoot	1660.464038	3758.634809	2709.549423	-1.178832043	4.65E-84	9.78E-83
Cladopus_002827	root	shoot	390.5619365	28.87363978	209.7177881	3.727855744	5.60E-84	1.18E-82
Cladopus_002315	root	shoot	15.48473656	476.182179	245.8334578	-4.928776642	6.23E-84	1.31E-82
Cladopus_011875	root	shoot	369.7939628	21.89915806	195.8465604	4.073888405	8.76E-84	1.84E-82
Cladopus_011379	root	shoot	393.7654179	12.06267975	202.9140488	5.036790397	1.10E-83	2.29E-82
Cladopus_003604	root	shoot	689.7171508	1929.943551	1309.830351	-1.486399136	1.45E-83	3.04E-82
Cladopus_024344	root	shoot	1038.292234	276.7342005	657.5132173	1.91078766	1.66E-83	3.46E-82
Cladopus_013562	root	shoot	1019.981007	289.0497591	654.5153829	1.819502323	2.39E-83	4.99E-82
Cladopus_017359	root	shoot	1859.759648	703.1171505	1281.438399	1.404958307	2.60E-83	5.42E-82
Cladopus_019601	root	shoot	4305.404356	1877.302888	3091.353622	1.197102068	4.19E-83	8.72E-82
Cladopus_011546	root	shoot	370.7423505	1174.390747	772.5665486	-1.663317935	5.09E-83	1.06E-81
Cladopus_018806	root	shoot	383.7039522	1242.677067	813.1905096	-1.694524316	6.26E-83	1.30E-81
Cladopus_015704	root	shoot	1448.311851	3166.058131	2307.184991	-1.128708314	8.46E-83	1.75E-81

Cladopus_007617	root	shoot	3619.687012	1690.63768	2655.162346	1.098696515	1.14E-82	2.36E-81
Cladopus_015383	root	shoot	929.4994058	2387.771233	1658.63532	-1.360748758	1.44E-82	2.98E-81
Cladopus_011474	root	shoot	1066.300038	3232.538677	2149.419357	-1.598201151	1.63E-82	3.38E-81
Cladopus_013397	root	shoot	7097.080252	3477.345549	5287.2129	1.028770945	1.75E-82	3.62E-81
Cladopus_013613	root	shoot	198.2595398	804.1159056	501.1877227	-2.023298748	2.03E-82	4.18E-81
Cladopus_000159	root	shoot	2159.33892	823.2141348	1491.276527	1.394413808	3.89E-82	8.02E-81
Cladopus_014127	root	shoot	258.0116167	937.366065	597.6888408	-1.862923562	3.94E-82	8.12E-81
Cladopus_015453	root	shoot	104.9128345	626.386949	365.6498918	-2.585758406	4.71E-82	9.67E-81
Cladopus_017855	root	shoot	203.6191765	805.4134711	504.5163238	-1.983916606	5.14E-82	1.06E-80
Cladopus_011135	root	shoot	833.417615	184.0598787	508.7387468	2.174788093	6.19E-82	1.27E-80
Cladopus_012301	root	shoot	374.4961622	21.32752954	197.9118459	4.190674242	9.13E-82	1.87E-80
Cladopus_001590	root	shoot	39.68300883	435.6487567	237.6658828	-3.466613366	1.05E-81	2.14E-80
Cladopus_027151	root	shoot	1068.659722	337.6313632	703.1455428	1.663223282	1.23E-81	2.52E-80
Cladopus_005815	root	shoot	3115.519871	6297.493767	4706.506819	-1.015207656	1.26E-81	2.57E-80
Cladopus_002107	root	shoot	1849.167409	686.826973	1267.997191	1.429081463	1.80E-81	3.66E-80
Cladopus_014992	root	shoot	5609.101491	1939.188652	3774.145072	1.53308556	2.04E-81	4.14E-80
Cladopus_018384	root	shoot	1373.215427	3272.741501	2322.978464	-1.254291471	2.44E-81	4.96E-80
Cladopus_022856	root	shoot	1216.544312	21.27986741	618.9120899	5.833253329	3.82E-81	7.75E-80
Cladopus_025469	root	shoot	1637.60727	610.4101143	1124.008692	1.424950539	4.07E-81	8.26E-80
Cladopus_023610	root	shoot	972.6236001	2522.862734	1747.743167	-1.376888856	4.42E-81	8.96E-80
Cladopus_015006	root	shoot	6545.834299	2296.057068	4420.945684	1.512116813	4.77E-81	9.65E-80
Cladopus_024610	root	shoot	458.4697868	66.11013465	262.2899607	2.788459088	5.63E-81	1.14E-79
Cladopus_001387	root	shoot	1343.38874	449.0671744	896.2279573	1.582304663	7.75E-81	1.56E-79
Cladopus_008736	root	shoot	22.0447129	427.296931	224.6708219	-4.271844154	8.09E-81	1.63E-79
Cladopus_014235	root	shoot	2961.15883	1319.569487	2140.364159	1.166250762	1.21E-80	2.44E-79
Cladopus_022898	root	shoot	470.8867491	73.95183164	272.4192904	2.667543954	1.27E-80	2.55E-79
Cladopus_001319	root	shoot	617.9076802	94.37218393	356.1399321	2.711362194	1.67E-80	3.35E-79
Cladopus_008351	root	shoot	12556.71127	5128.733797	8842.722533	1.291614193	1.89E-80	3.81E-79
Cladopus_005767	root	shoot	772.0931297	172.3387417	472.2159357	2.162788511	2.16E-80	4.34E-79
Cladopus_004671	root	shoot	654.7856274	1761.387367	1208.086497	-1.427625677	2.30E-80	4.60E-79

Cladopus_025588	root	shoot	390.2748219	32.79075233	211.5327871	3.570178826	2.69E-80	5.37E-79
Cladopus_025366	root	shoot	1741.444596	435.6859085	1088.565252	1.999347998	2.92E-80	5.83E-79
Cladopus_017630	root	shoot	4202.339386	2038.445503	3120.392445	1.043905256	2.99E-80	5.97E-79
Cladopus_014498	root	shoot	566.7974429	1573.944705	1070.371074	-1.474809348	3.15E-80	6.28E-79
Cladopus_010275	root	shoot	643.0003402	1863.848738	1253.424539	-1.537216191	3.17E-80	6.31E-79
Cladopus_024364	root	shoot	49104.17854	21513.61868	35308.89861	1.190705369	6.17E-80	1.23E-78
Cladopus_012313	root	shoot	418.6648254	51.13350813	234.8991667	3.022526516	6.23E-80	1.24E-78
Cladopus_002730	root	shoot	1373.93229	461.1702541	917.551272	1.573266909	6.66E-80	1.32E-78
Cladopus_013121	root	shoot	612.5798541	1595.923141	1104.251498	-1.38176897	7.96E-80	1.58E-78
Cladopus_023468	root	shoot	393.5380952	1170.40008	781.9690876	-1.573019452	8.18E-80	1.62E-78
Cladopus_018165	root	shoot	561.5387129	1560.294435	1060.916574	-1.473908371	8.61E-80	1.70E-78
Cladopus_020436	root	shoot	1298.836121	3304.421407	2301.628764	-1.348383846	9.11E-80	1.80E-78
Cladopus_015679	root	shoot	3393.553516	1559.577317	2476.565417	1.121488204	1.10E-79	2.18E-78
Cladopus_006534	root	shoot	7284.849044	3275.025459	5279.937252	1.154023783	1.52E-79	3.00E-78
Cladopus_016080	root	shoot	279.6642242	1231.300487	755.4823556	-2.136268714	1.52E-79	3.00E-78
Cladopus_005805	root	shoot	9493.884424	4332.391825	6913.138125	1.131919062	1.57E-79	3.09E-78
Cladopus_017947	root	shoot	516.1594354	85.873233	301.0163342	2.593278287	2.30E-79	4.53E-78
Cladopus_013466	root	shoot	47167.42889	20245.38484	33706.40686	1.22022038	2.65E-79	5.21E-78
Cladopus_008476	root	shoot	476.2638313	55.34531293	265.8045721	3.095231582	6.57E-79	1.29E-77
Cladopus_021594	root	shoot	843.060579	2364.138817	1603.599698	-1.488511861	1.03E-78	2.01E-77
Cladopus_007219	root	shoot	3138.231398	1331.324818	2234.778108	1.236576713	1.08E-78	2.12E-77
Cladopus_022914	root	shoot	1474.887414	515.3894144	995.1384141	1.518473283	1.11E-78	2.16E-77
Cladopus_019916	root	shoot	1526.57264	399.9478287	963.2602342	1.934206689	1.22E-78	2.38E-77
Cladopus_027120	root	shoot	335.9699148	14.08662001	175.0282674	4.559564891	1.26E-78	2.46E-77
Cladopus_011446	root	shoot	5271.345571	2257.050925	3764.198248	1.224759331	1.45E-78	2.83E-77
Cladopus_004801	root	shoot	4167.221023	2057.525078	3112.37305	1.018318034	1.58E-78	3.07E-77
Cladopus_025562	root	shoot	1448.178037	345.8573109	897.0176739	2.072298468	1.71E-78	3.33E-77
Cladopus_009644	root	shoot	335.182356	13.78489382	174.4836249	4.585691611	2.71E-78	5.26E-77
Cladopus_011968	root	shoot	422.8351339	1229.932993	826.3840634	-1.541709971	3.08E-78	5.97E-77
Cladopus_012332	root	shoot	383.7332849	32.08050839	207.9068966	3.580971567	4.48E-78	8.67E-77

Cladopus_000263	root	shoot	2497.839347	6342.99859	4420.418968	-1.345327872	6.25E-78	1.21E-76
Cladopus_003385	root	shoot	2887.116638	6429.940998	4658.528818	-1.155880627	9.11E-78	1.76E-76
Cladopus_025617	root	shoot	775.8845803	2110.117783	1443.001181	-1.443582378	1.05E-77	2.02E-76
Cladopus_020156	root	shoot	50.19415274	443.0515919	246.6228723	-3.144898596	1.09E-77	2.10E-76
Cladopus_018934	root	shoot	1272.186048	451.3936524	861.78985	1.494119417	1.19E-77	2.30E-76
Cladopus_015373	root	shoot	6670.578929	2952.4889	4811.533915	1.176420834	1.43E-77	2.75E-76
Cladopus_008167	root	shoot	1170.430748	2663.545929	1916.988338	-1.186671912	1.54E-77	2.96E-76
Cladopus_025598	root	shoot	795.4021916	2225.172736	1510.287464	-1.485180195	2.02E-77	3.87E-76
Cladopus_013081	root	shoot	1731.686182	3716.338015	2724.012099	-1.101511197	2.38E-77	4.56E-76
Cladopus_008115	root	shoot	1876.861354	723.4110209	1300.136187	1.375360966	2.77E-77	5.29E-76
Cladopus_003146	root	shoot	2142.066366	808.5389399	1475.302653	1.407622128	3.03E-77	5.79E-76
Cladopus_010084	root	shoot	581.2230987	1745.612141	1163.41762	-1.589301826	4.88E-77	9.31E-76
Cladopus_010276	root	shoot	696.0140207	2536.8208	1616.41741	-1.868395237	5.18E-77	9.88E-76
Cladopus_005010	root	shoot	772.5108882	150.7266567	461.6187725	2.357617864	7.37E-77	1.40E-75
Cladopus_021934	root	shoot	2903.940371	7611.088112	5257.514241	-1.390859336	1.23E-76	2.34E-75
Cladopus_002376	root	shoot	48047.06559	23266.90677	35656.98618	1.046241997	1.25E-76	2.36E-75
Cladopus_003641	root	shoot	51.84120423	438.3561563	245.0986802	-3.075435871	1.52E-76	2.88E-75
Cladopus_014166	root	shoot	970.5317106	2345.008262	1657.769986	-1.272994148	1.73E-76	3.27E-75
Cladopus_009957	root	shoot	2874.484038	1208.112213	2041.298126	1.249636037	1.83E-76	3.46E-75
Cladopus_015950	root	shoot	1131.848301	337.3161711	734.5822359	1.751165442	2.37E-76	4.48E-75
Cladopus_001118	root	shoot	354.9176218	1200.533386	777.725504	-1.761050122	2.75E-76	5.19E-75
Cladopus_009960	root	shoot	119.4441071	580.0368742	349.7404907	-2.281205542	3.07E-76	5.80E-75
Cladopus_006778	root	shoot	839.2828943	203.5012698	521.3920821	2.04312987	3.10E-76	5.85E-75
Cladopus_019589	root	shoot	3055.243496	1047.298347	2051.270921	1.545683801	3.33E-76	6.27E-75
Cladopus_020241	root	shoot	636.483959	115.7105145	376.0972368	2.449429613	3.89E-76	7.31E-75
Cladopus_018860	root	shoot	403.1107638	8.961124922	206.0359444	5.507778909	3.95E-76	7.43E-75
Cladopus_017025	root	shoot	169.8826616	717.2122842	443.5474729	-2.07551766	4.09E-76	7.68E-75
Cladopus_007107	root	shoot	4828.631162	2185.55315	3507.092156	1.14369975	5.29E-76	9.91E-75
Cladopus_024254	root	shoot	1568.692825	526.1580013	1047.425413	1.571513054	6.86E-76	1.28E-74
Cladopus_010262	root	shoot	215.920989	834.203924	525.0624565	-1.95272777	1.09E-75	2.04E-74

Cladopus_000594	root	shoot	1407.606537	458.6626811	933.1346089	1.616646644	1.15E-75	2.15E-74
Cladopus_019969	root	shoot	589.2783382	118.5950098	353.936674	2.303334661	1.17E-75	2.18E-74
Cladopus_003566	root	shoot	1189.856613	392.2790203	791.0678168	1.600602654	1.57E-75	2.93E-74
Cladopus_003780	root	shoot	4228.621359	2085.101007	3156.861183	1.019866634	2.06E-75	3.84E-74
Cladopus_005446	root	shoot	698.915538	145.7341566	422.3248473	2.269657895	2.45E-75	4.56E-74
Cladopus_007234	root	shoot	576.712689	123.7913318	350.2520104	2.219387843	2.61E-75	4.87E-74
Cladopus_000364	root	shoot	1202.539783	3017.543043	2110.041413	-1.325746523	3.12E-75	5.80E-74
Cladopus_024044	root	shoot	10781.57327	5336.364818	8058.969045	1.014774976	3.34E-75	6.21E-74
Cladopus_022171	root	shoot	1562.689626	3290.818353	2426.75399	-1.075018052	4.91E-75	9.11E-74
Cladopus_008012	root	shoot	530.8063503	78.08852143	304.4474359	2.764217694	8.95E-75	1.66E-73
Cladopus_020427	root	shoot	7723.68423	3616.884204	5670.284217	1.094375107	9.19E-75	1.70E-73
Cladopus_012450	root	shoot	761.035374	208.0632745	484.5493243	1.868336443	1.09E-74	2.02E-73
Cladopus_006715	root	shoot	1088.448744	312.9952068	700.7219754	1.792252635	1.29E-74	2.38E-73
Cladopus_006441	root	shoot	72.81792854	541.040638	306.9292833	-2.897323427	1.56E-74	2.88E-73
Cladopus_016061	root	shoot	16596.29289	7193.662483	11894.97769	1.206160496	3.03E-74	5.58E-73
Cladopus_010197	root	shoot	3769.804135	1551.044255	2660.424195	1.281157673	3.13E-74	5.77E-73
Cladopus_017050	root	shoot	601.008638	1650.798729	1125.903684	-1.45941259	3.22E-74	5.92E-73
Cladopus_012444	root	shoot	1313.679287	441.1140223	877.3966546	1.573640837	3.23E-74	5.93E-73
Cladopus_008846	root	shoot	679.0862904	1742.771466	1210.928878	-1.359953859	3.27E-74	6.00E-73
Cladopus_020414	root	shoot	2165.261271	924.6487113	1544.954991	1.226701349	4.36E-74	8.00E-73
Cladopus_023123	root	shoot	107.7575076	545.9912624	326.874385	-2.341140956	4.38E-74	8.03E-73
Cladopus_012854	root	shoot	282.4764567	915.6050014	599.0407291	-1.695216581	4.38E-74	8.03E-73
Cladopus_018775	root	shoot	3398.592599	1357.44577	2378.019185	1.32411756	5.86E-74	1.07E-72
Cladopus_023261	root	shoot	1023.082108	298.0328617	660.5574849	1.778157689	6.45E-74	1.18E-72
Cladopus_002102	root	shoot	1275.440878	3384.819028	2330.129953	-1.409263368	7.45E-74	1.36E-72
Cladopus_011987	root	shoot	1538.613439	621.3557364	1079.984588	1.308470481	8.56E-74	1.56E-72
Cladopus_015372	root	shoot	1728.762267	3627.794023	2678.278145	-1.069864824	8.60E-74	1.57E-72
Cladopus_006861	root	shoot	1415.230038	3150.037791	2282.633915	-1.15525718	9.39E-74	1.71E-72
Cladopus_009890	root	shoot	3328.776618	1204.327286	2266.551952	1.467673486	9.72E-74	1.77E-72
Cladopus_013546	root	shoot	281.2852757	966.3934942	623.8393849	-1.777444198	1.06E-73	1.93E-72

Cladopus_022904	root	shoot	4561.595554	2254.250715	3407.923134	1.016349012	1.13E-73	2.06E-72
Cladopus_009387	root	shoot	891.3197173	261.8830141	576.6013657	1.76810063	1.14E-73	2.06E-72
Cladopus_003191	root	shoot	1058.026894	288.3200551	673.1734743	1.88088993	1.21E-73	2.18E-72
Cladopus_015949	root	shoot	1134.582161	342.435672	738.5089165	1.73275933	1.49E-73	2.70E-72
Cladopus_019020	root	shoot	556.0102022	1458.023498	1007.01685	-1.391873266	1.78E-73	3.22E-72
Cladopus_019832	root	shoot	2781.899741	1241.352254	2011.625998	1.162053093	2.12E-73	3.84E-72
Cladopus_018648	root	shoot	1930.361621	4602.601669	3266.481645	-1.252486271	2.30E-73	4.15E-72
Cladopus_023011	root	shoot	431.7842896	59.43352903	245.6089093	2.857962859	3.40E-73	6.14E-72
Cladopus_009000	root	shoot	2487.345573	1057.575161	1772.460367	1.232433288	3.98E-73	7.16E-72
Cladopus_009016	root	shoot	1196.536704	2779.247643	1987.892174	-1.214666566	5.68E-73	1.02E-71
Cladopus_020143	root	shoot	5755.462015	2006.559946	3881.01098	1.520781597	7.70E-73	1.38E-71
Cladopus_004300	root	shoot	505.6043365	100.4015251	303.0029308	2.324655942	8.11E-73	1.46E-71
Cladopus_003151	root	shoot	848.7470784	2033.777599	1441.262339	-1.261446563	9.85E-73	1.77E-71
Cladopus_003142	root	shoot	2614.404768	1227.144567	1920.774668	1.090716416	9.96E-73	1.79E-71
Cladopus_014783	root	shoot	1265.49112	434.2242754	849.8576978	1.547391691	1.24E-72	2.23E-71
Cladopus_021325	root	shoot	309.5503537	17.83943467	163.6948942	4.071216194	3.52E-72	6.29E-71
Cladopus_014519	root	shoot	194.1890179	833.5112225	513.8501202	-2.103898838	3.58E-72	6.39E-71
Cladopus_018562	root	shoot	845.137518	235.9403445	540.5389313	1.842678056	3.77E-72	6.73E-71
Cladopus_007147	root	shoot	1768.656884	655.1412257	1211.899055	1.435752667	4.16E-72	7.42E-71
Cladopus_000070	root	shoot	1182.009518	374.2036492	778.1065836	1.658863943	5.21E-72	9.28E-71
Cladopus_014070	root	shoot	2206.648666	917.1692853	1561.908976	1.267415521	5.23E-72	9.32E-71
Cladopus_008087	root	shoot	3267.321485	1621.256883	2444.289184	1.011057498	8.06E-72	1.43E-70
Cladopus_010259	root	shoot	4494.977671	9410.119912	6952.548791	-1.066344423	8.51E-72	1.51E-70
Cladopus_017908	root	shoot	1330.362257	2941.172184	2135.767221	-1.145429079	9.58E-72	1.70E-70
Cladopus_007218	root	shoot	2538.412577	1091.552173	1814.982375	1.216293397	1.25E-71	2.22E-70
Cladopus_001512	root	shoot	1496.821783	4018.55388	2757.687831	-1.42609391	2.82E-71	4.99E-70
Cladopus_006659	root	shoot	1660.451928	622.4984784	1141.475203	1.418696889	3.56E-71	6.31E-70
Cladopus_000959	root	shoot	1793.992971	736.687853	1265.340412	1.284818713	4.14E-71	7.32E-70
Cladopus_003765	root	shoot	546.4312281	1405.789507	976.1103678	-1.362982067	4.24E-71	7.49E-70
Cladopus_025381	root	shoot	766.2381421	1916.644746	1341.441444	-1.3207785	4.41E-71	7.79E-70

Cladopus_021932	root	shoot	2642.583148	6749.859441	4696.221295	-1.353674263	5.21E-71	9.20E-70
Cladopus_005422	root	shoot	615.165021	153.9208709	384.542946	2.00266347	6.38E-71	1.12E-69
Cladopus_018855	root	shoot	7725.523511	2616.407763	5170.965637	1.562803984	7.69E-71	1.35E-69
Cladopus_021251	root	shoot	8176.702621	3883.005969	6029.854295	1.074088569	9.03E-71	1.59E-69
Cladopus_005035	root	shoot	7615.945533	3756.950484	5686.448008	1.019447423	9.52E-71	1.67E-69
Cladopus_012652	root	shoot	3598.934286	1572.854751	2585.894519	1.195074792	1.16E-70	2.03E-69
Cladopus_025149	root	shoot	686.1685024	189.4704501	437.8194763	1.855233585	1.16E-70	2.04E-69
Cladopus_023615	root	shoot	1842.270894	784.1674262	1313.21916	1.232698871	1.42E-70	2.48E-69
Cladopus_025407	root	shoot	944.0097482	2234.790383	1589.400065	-1.244473951	1.78E-70	3.11E-69
Cladopus_024754	root	shoot	2593.113715	1146.03945	1869.576582	1.178110123	1.81E-70	3.16E-69
Cladopus_019117	root	shoot	323.3764023	9.268845208	166.3226237	5.104582899	2.70E-70	4.72E-69
Cladopus_014083	root	shoot	7194.849688	3365.034174	5279.941931	1.096010731	3.06E-70	5.33E-69
Cladopus_003016	root	shoot	680.6702003	1638.368239	1159.51922	-1.267491338	3.15E-70	5.48E-69
Cladopus_019422	root	shoot	953.9830596	2341.946945	1647.965002	-1.296633113	3.85E-70	6.70E-69
Cladopus_019280	root	shoot	354.5301019	46.58164025	200.5558711	2.916776518	4.91E-70	8.53E-69
Cladopus_003338	root	shoot	1150.044104	2494.397825	1822.220965	-1.117397109	5.75E-70	9.99E-69
Cladopus_003503	root	shoot	839.6097505	1983.781273	1411.695512	-1.239581043	7.90E-70	1.37E-68
Cladopus_025573	root	shoot	1065.097944	2329.561016	1697.32948	-1.128915978	9.26E-70	1.60E-68
Cladopus_007063	root	shoot	318.2983089	8.428644854	163.3634769	5.216944274	1.05E-69	1.83E-68
Cladopus_005058	root	shoot	498.7088273	1304.712891	901.7108594	-1.388475805	1.34E-69	2.31E-68
Cladopus_010075	root	shoot	488.7500055	99.5380146	294.14401	2.299469126	1.37E-69	2.37E-68
Cladopus_011797	root	shoot	4311.972165	11397.68548	7854.82882	-1.402860806	1.40E-69	2.42E-68
Cladopus_011359	root	shoot	6527.169921	3215.404007	4871.286964	1.021631384	1.74E-69	3.00E-68
Cladopus_008481	root	shoot	2890.257025	1223.241444	2056.749234	1.241258188	1.85E-69	3.18E-68
Cladopus_002321	root	shoot	900.2287416	292.1522065	596.1904741	1.62413689	1.89E-69	3.24E-68
Cladopus_021294	root	shoot	968.2898708	2237.560079	1602.924975	-1.209021448	2.46E-69	4.23E-68
Cladopus_000088	root	shoot	3332.030619	1169.33941	2250.685014	1.511371982	2.55E-69	4.37E-68
Cladopus_011136	root	shoot	5480.98492	2384.942916	3932.963918	1.201424426	3.47E-69	5.95E-68
Cladopus_021922	root	shoot	435.9812127	1219.253274	827.6172433	-1.485409471	3.48E-69	5.96E-68
Cladopus_003022	root	shoot	1187.630636	426.0530218	806.8418287	1.476391613	5.66E-69	9.66E-68

Cladopus_013644	root	shoot	22.75810815	361.5651173	192.1616127	-4.001537011	8.06E-69	1.37E-67
Cladopus_014067	root	shoot	1145.083393	2572.879337	1858.981365	-1.167033176	8.17E-69	1.39E-67
Cladopus_024914	root	shoot	1513.836135	398.8822813	956.3592083	1.923863138	8.61E-69	1.47E-67
Cladopus_011942	root	shoot	2461.580981	4950.415789	3705.998385	-1.008032119	9.38E-69	1.59E-67
Cladopus_008090	root	shoot	2359.447918	1005.231794	1682.339856	1.230935057	9.76E-69	1.66E-67
Cladopus_014393	root	shoot	1239.557133	2669.85161	1954.704372	-1.107122247	1.04E-68	1.76E-67
Cladopus_018386	root	shoot	2141.663232	892.4723763	1517.067804	1.2632561	1.17E-68	1.98E-67
Cladopus_011106	root	shoot	525.6039404	99.07369658	312.3388185	2.410804486	1.27E-68	2.16E-67
Cladopus_005696	root	shoot	3130.20858	7339.359517	5234.784048	-1.230159533	1.33E-68	2.24E-67
Cladopus_026989	root	shoot	2053.938891	888.799631	1471.369261	1.208527838	1.36E-68	2.30E-67
Cladopus_025187	root	shoot	2149.094873	585.7912759	1367.443074	1.875324713	1.86E-68	3.14E-67
Cladopus_002891	root	shoot	2061.448371	892.3083711	1476.878371	1.20779749	2.18E-68	3.68E-67
Cladopus_005205	root	shoot	1528.726908	3208.051048	2368.388978	-1.070008491	2.90E-68	4.88E-67
Cladopus_009531	root	shoot	1729.27422	3557.84351	2643.558865	-1.041348189	2.95E-68	4.97E-67
Cladopus_010590	root	shoot	557.4173071	1462.723907	1010.070607	-1.392333919	2.99E-68	5.03E-67
Cladopus_007999	root	shoot	1505.048246	4299.494436	2902.271341	-1.515543311	3.19E-68	5.37E-67
Cladopus_008842	root	shoot	1645.841662	671.5691222	1158.705392	1.295093744	3.63E-68	6.10E-67
Cladopus_010281	root	shoot	638.6677245	1835.312432	1236.990078	-1.523942284	4.46E-68	7.49E-67
Cladopus_015586	root	shoot	1379.809493	352.7391313	866.2743121	1.969383623	5.42E-68	9.09E-67
Cladopus_017670	root	shoot	1598.114418	653.4274385	1125.770928	1.288558895	5.72E-68	9.58E-67
Cladopus_003574	root	shoot	487.6619479	1559.716665	1023.689307	-1.679998472	6.22E-68	1.04E-66
Cladopus_002918	root	shoot	3770.548204	1520.743154	2645.645679	1.309775196	7.09E-68	1.19E-66
Cladopus_006222	root	shoot	353.2611634	1099.54972	726.4054418	-1.639189539	9.55E-68	1.60E-66
Cladopus_018851	root	shoot	901.263557	2114.99269	1508.128124	-1.230767927	1.03E-67	1.71E-66
Cladopus_002810	root	shoot	1527.268796	637.457785	1082.36329	1.260366686	1.32E-67	2.19E-66
Cladopus_012968	root	shoot	1081.557456	2803.267241	1942.412349	-1.373265694	1.52E-67	2.53E-66
Cladopus_007384	root	shoot	867.2339819	2061.183988	1464.208985	-1.248645857	1.71E-67	2.84E-66
Cladopus_017101	root	shoot	1294.915057	511.6260084	903.2705326	1.339714687	1.75E-67	2.92E-66
Cladopus_001795	root	shoot	308.4868203	31.72113055	170.1039754	3.291570348	1.85E-67	3.08E-66
Cladopus_003079	root	shoot	3188.42122	1532.289339	2360.35528	1.055978286	2.17E-67	3.60E-66

Cladopus_015270	root	shoot	1130.984205	3227.435226	2179.209715	-1.514268617	2.47E-67	4.10E-66
Cladopus_010210	root	shoot	1871.178309	820.8856542	1346.031982	1.18915875	3.33E-67	5.51E-66
Cladopus_022715	root	shoot	2852.690029	1353.811881	2103.250955	1.07455729	4.98E-67	8.23E-66
Cladopus_005296	root	shoot	4761.68531	2270.704086	3516.194698	1.068872642	8.44E-67	1.39E-65
Cladopus_016292	root	shoot	3919.852904	1929.845789	2924.849346	1.021792588	1.18E-66	1.96E-65
Cladopus_005395	root	shoot	1122.677018	398.1563445	760.4166813	1.491119162	1.61E-66	2.65E-65
Cladopus_022775	root	shoot	361.5249029	5.736274016	183.6305884	5.984197557	1.89E-66	3.11E-65
Cladopus_001224	root	shoot	1053.813858	378.5221003	716.1679789	1.479009618	2.02E-66	3.32E-65
Cladopus_013824	root	shoot	19279.53953	7710.880501	13495.21001	1.322078289	2.26E-66	3.71E-65
Cladopus_006107	root	shoot	2041.561295	892.976509	1467.268902	1.192745117	2.35E-66	3.85E-65
Cladopus_009524	root	shoot	85.5674404	523.3774914	304.4724659	-2.61988461	2.42E-66	3.97E-65
Cladopus_008838	root	shoot	1117.932717	2613.656236	1865.794476	-1.226621217	2.43E-66	3.98E-65
Cladopus_015595	root	shoot	37.18216199	377.551568	207.366865	-3.337990983	3.06E-66	5.01E-65
Cladopus_008706	root	shoot	3610.498128	7261.043048	5435.770588	-1.008610427	3.15E-66	5.15E-65
Cladopus_009788	root	shoot	1315.532094	3376.215722	2345.873908	-1.361120339	3.19E-66	5.22E-65
Cladopus_009383	root	shoot	1002.229553	2199.978066	1601.10381	-1.133782333	5.46E-66	8.91E-65
Cladopus_027048	root	shoot	212.6830946	791.6923742	502.1877344	-1.897862138	5.87E-66	9.57E-65
Cladopus_008318	root	shoot	1121.826682	316.0784096	718.9525457	1.829025023	6.27E-66	1.02E-64
Cladopus_000134	root	shoot	276.3058636	871.4717218	573.8887927	-1.657474602	6.93E-66	1.13E-64
Cladopus_016954	root	shoot	495.5475702	1316.524168	906.0358692	-1.411986545	7.93E-66	1.29E-64
Cladopus_002108	root	shoot	2394.706405	6399.699488	4397.202947	-1.419042043	8.93E-66	1.45E-64
Cladopus_003465	root	shoot	39.56885702	361.4931861	200.5310216	-3.186967682	9.41E-66	1.53E-64
Cladopus_006358	root	shoot	1808.815822	769.9020835	1289.358953	1.233699983	9.57E-66	1.55E-64
Cladopus_003054	root	shoot	353.4853888	51.06386828	202.2746285	2.794900072	1.14E-65	1.85E-64
Cladopus_015278	root	shoot	283.7171614	10.39493347	147.0560474	4.750248883	1.40E-65	2.26E-64
Cladopus_002904	root	shoot	846.9186552	267.522488	557.2205716	1.666816454	1.42E-65	2.29E-64
Cladopus_015977	root	shoot	354.3728669	5.93720273	180.1550348	5.887370345	1.75E-65	2.82E-64
Cladopus_016963	root	shoot	2317.483972	963.4266325	1640.455302	1.265762249	1.77E-65	2.86E-64
Cladopus_005143	root	shoot	165.0733657	626.5645656	395.8189656	-1.924529901	2.15E-65	3.47E-64
Cladopus_020167	root	shoot	2573.268358	1266.963605	1920.115982	1.022371818	2.44E-65	3.93E-64

Cladopus_000919	root	shoot	1331.91734	439.388479	885.6529095	1.601241699	2.49E-65	4.00E-64
Cladopus_005635	root	shoot	1381.27872	2924.68599	2152.982355	-1.082466217	2.57E-65	4.14E-64
Cladopus_017609	root	shoot	1832.684736	755.029127	1293.856932	1.27695776	3.13E-65	5.02E-64
Cladopus_012265	root	shoot	302.0101767	1145.829929	723.9200527	-1.920143906	3.38E-65	5.43E-64
Cladopus_022582	root	shoot	5527.211446	2433.832155	3980.521801	1.183898609	3.45E-65	5.53E-64
Cladopus_008248	root	shoot	2902.71144	5940.27721	4421.494325	-1.033741017	4.34E-65	6.94E-64
Cladopus_020108	root	shoot	378.1847222	1124.881609	751.5331655	-1.575602229	4.55E-65	7.28E-64
Cladopus_023579	root	shoot	352.0166181	1135.53521	743.7759138	-1.69317828	4.59E-65	7.33E-64
Cladopus_014147	root	shoot	379.2844123	59.24377696	219.2640946	2.68989977	1.05E-64	1.67E-63
Cladopus_020585	root	shoot	352.4973832	5.852909768	179.1751465	5.943969144	1.23E-64	1.96E-63
Cladopus_012983	root	shoot	304.8703098	14.20458822	159.537449	4.461774365	1.48E-64	2.36E-63
Cladopus_003487	root	shoot	1717.014002	3576.868623	2646.941313	-1.0595956	1.59E-64	2.52E-63
Cladopus_008352	root	shoot	1604.703635	3715.805854	2660.254745	-1.210000646	1.75E-64	2.78E-63
Cladopus_013563	root	shoot	748.4411223	208.5529824	478.4970523	1.843055586	1.92E-64	3.04E-63
Cladopus_017481	root	shoot	4834.528657	2152.672862	3493.600759	1.167282126	3.00E-64	4.76E-63
Cladopus_015166	root	shoot	4765.691867	2186.556093	3476.12398	1.12406818	4.08E-64	6.44E-63
Cladopus_005751	root	shoot	1108.6009	315.7251049	712.1630027	1.813096658	4.62E-64	7.30E-63
Cladopus_006811	root	shoot	4977.272057	2254.854008	3616.063032	1.1418885	4.78E-64	7.55E-63
Cladopus_001855	root	shoot	20.18544949	323.8947958	172.0401226	-4.02364577	5.48E-64	8.64E-63
Cladopus_003626	root	shoot	435.1929148	64.70267479	249.9477948	2.753737967	7.10E-64	1.12E-62
Cladopus_001090	root	shoot	815.6592127	269.7935393	542.726376	1.597682746	7.16E-64	1.13E-62
Cladopus_005070	root	shoot	4536.31589	9250.586653	6893.451271	-1.028486986	1.20E-63	1.88E-62
Cladopus_015754	root	shoot	418.051343	80.8525308	249.4519369	2.366748247	1.38E-63	2.16E-62
Cladopus_008731	root	shoot	1578.939372	3678.87972	2628.909546	-1.221546609	1.57E-63	2.46E-62
Cladopus_014120	root	shoot	1773.280942	774.7840892	1274.032516	1.19430259	1.57E-63	2.47E-62
Cladopus_014950	root	shoot	176.6888966	700.6325101	438.6607034	-1.993108536	1.72E-63	2.70E-62
Cladopus_010041	root	shoot	1310.169875	540.4707175	925.3202961	1.27863573	1.88E-63	2.94E-62
Cladopus_017826	root	shoot	916.1858173	2044.45493	1480.320374	-1.157760428	2.39E-63	3.73E-62
Cladopus_008175	root	shoot	199.9296225	711.1377946	455.5337086	-1.830211469	2.95E-63	4.60E-62
Cladopus_025105	root	shoot	959.5717432	302.378475	630.9751091	1.669282787	3.22E-63	5.02E-62

Cladopus_001645	root	shoot	188.9960402	716.4835995	452.7398198	-1.921202949	3.32E-63	5.17E-62
Cladopus_007592	root	shoot	1037.058184	2768.114985	1902.586585	-1.417728253	3.40E-63	5.29E-62
Cladopus_020403	root	shoot	69.38936703	420.2123285	244.8008478	-2.596420875	3.86E-63	6.01E-62
Cladopus_025721	root	shoot	267.0397575	11.68931503	139.3645362	4.511075307	3.91E-63	6.07E-62
Cladopus_023616	root	shoot	3114.53776	6743.468085	4929.002923	-1.115294246	4.78E-63	7.42E-62
Cladopus_019549	root	shoot	188911.1782	12216.86507	100564.0216	3.950757693	5.10E-63	7.91E-62
Cladopus_015366	root	shoot	629.3377295	1502.199619	1065.768674	-1.256340187	6.38E-63	9.87E-62
Cladopus_011356	root	shoot	46.6045116	387.8438526	217.2241821	-3.066659224	8.37E-63	1.29E-61
Cladopus_011579	root	shoot	1595.75917	689.2370797	1142.498125	1.211594873	1.08E-62	1.66E-61
Cladopus_008754	root	shoot	5193.180139	2506.986202	3850.08317	1.051321313	1.20E-62	1.86E-61
Cladopus_001208	root	shoot	202.8437492	688.0147103	445.4292297	-1.762588275	1.62E-62	2.50E-61
Cladopus_009443	root	shoot	881.165525	249.4163329	565.2909289	1.824411838	1.91E-62	2.94E-61
Cladopus_017521	root	shoot	6412.269212	2772.682408	4592.47581	1.209797751	2.01E-62	3.10E-61
Cladopus_008784	root	shoot	1591.39646	658.0707806	1124.73362	1.273858853	2.35E-62	3.62E-61
Cladopus_015043	root	shoot	1799.109701	705.7933075	1252.451504	1.349913053	2.43E-62	3.74E-61
Cladopus_023569	root	shoot	919.218555	1971.151562	1445.185059	-1.100802287	3.28E-62	5.03E-61
Cladopus_011386	root	shoot	1603.909349	4217.997873	2910.953611	-1.396140885	4.09E-62	6.27E-61
Cladopus_006595	root	shoot	479.7362257	1218.541639	849.1389323	-1.344589037	4.67E-62	7.15E-61
Cladopus_002378	root	shoot	2308.497433	972.5405734	1640.519003	1.247807783	6.24E-62	9.52E-61
Cladopus_016966	root	shoot	1759.366753	794.8139061	1277.090329	1.146225763	9.21E-62	1.40E-60
Cladopus_021844	root	shoot	779.5252292	1912.958171	1346.2417	-1.29681764	9.86E-62	1.50E-60
Cladopus_023059	root	shoot	1415.024023	603.7148582	1009.36944	1.227743032	1.09E-61	1.65E-60
Cladopus_023668	root	shoot	853.9887293	297.52925	575.7589896	1.524930012	1.18E-61	1.78E-60
Cladopus_014911	root	shoot	597.942723	1417.256952	1007.599837	-1.246347977	1.56E-61	2.37E-60
Cladopus_011744	root	shoot	5973.25364	2893.833566	4433.543603	1.045628904	2.07E-61	3.13E-60
Cladopus_000205	root	shoot	1079.53414	390.2520435	734.8930919	1.470456518	2.53E-61	3.82E-60
Cladopus_016735	root	shoot	1506.327122	641.2626308	1073.794877	1.232696533	2.78E-61	4.20E-60
Cladopus_024738	root	shoot	28605.10069	12224.59212	20414.8464	1.226562685	2.80E-61	4.23E-60
Cladopus_014692	root	shoot	578.2886613	123.6390221	350.9638417	2.214568331	2.98E-61	4.49E-60
Cladopus_012943	root	shoot	1133.691967	320.8560771	727.2740222	1.82635355	5.74E-61	8.64E-60

Cladopus_014917	root	shoot	3985.418545	1498.541383	2741.979964	1.41107322	5.98E-61	9.00E-60
Cladopus_016053	root	shoot	292.1011164	855.7913644	573.9462404	-1.550888384	6.16E-61	9.25E-60
Cladopus_008176	root	shoot	1313.35937	490.0674323	901.7134009	1.421497399	7.54E-61	1.13E-59
Cladopus_023343	root	shoot	142.7540424	575.6692852	359.2116638	-2.009764971	8.24E-61	1.24E-59
Cladopus_004304	root	shoot	2263.783858	1083.756058	1673.769958	1.061162481	9.81E-61	1.47E-59
Cladopus_001556	root	shoot	364.3911143	67.06164288	215.7263786	2.444420215	1.01E-60	1.52E-59
Cladopus_009983	root	shoot	545.9829183	1443.581276	994.7820969	-1.400524978	1.30E-60	1.95E-59
Cladopus_016604	root	shoot	1296.557887	2776.254943	2036.406415	-1.099377111	1.57E-60	2.34E-59
Cladopus_007165	root	shoot	132.7830827	565.3941713	349.088627	-2.092600204	2.24E-60	3.35E-59
Cladopus_005905	root	shoot	568.7640471	119.9474829	344.355765	2.241532358	2.26E-60	3.37E-59
Cladopus_016522	root	shoot	2234.064762	1024.054333	1629.059548	1.125587389	2.76E-60	4.10E-59
Cladopus_016729	root	shoot	660.8576549	1791.94726	1226.402457	-1.440958353	2.86E-60	4.25E-59
Cladopus_004391	root	shoot	11.09775691	324.5897204	167.8437387	-4.873460521	2.99E-60	4.45E-59
Cladopus_006816	root	shoot	1224.274353	413.7079306	818.9911418	1.563873125	3.42E-60	5.09E-59
Cladopus_021200	root	shoot	190.9481548	688.1835889	439.5658718	-1.853349605	3.43E-60	5.10E-59
Cladopus_016383	root	shoot	433.1234208	85.43274606	259.2780834	2.351946259	3.48E-60	5.16E-59
Cladopus_019296	root	shoot	760.9836166	1820.440291	1290.711954	-1.257095614	3.66E-60	5.43E-59
Cladopus_001119	root	shoot	2825.87926	1255.683619	2040.78144	1.170519061	4.48E-60	6.63E-59
Cladopus_012413	root	shoot	396.0176479	1055.432735	725.7251917	-1.414245979	5.21E-60	7.70E-59
Cladopus_006843	root	shoot	795.4523405	266.8220863	531.1372134	1.57365932	5.41E-60	7.99E-59
Cladopus_023581	root	shoot	1502.214235	3396.711044	2449.462639	-1.178057899	8.62E-60	1.27E-58
Cladopus_011929	root	shoot	258.7059474	782.3635821	520.5347647	-1.596572161	9.05E-60	1.33E-58
Cladopus_021417	root	shoot	269.0862267	8.363666649	138.7249467	4.979242384	1.48E-59	2.18E-58
Cladopus_005515	root	shoot	2253.804127	1066.232191	1660.018159	1.07957762	2.02E-59	2.98E-58
Cladopus_006707	root	shoot	310.0995744	884.7043829	597.4019786	-1.51346413	2.61E-59	3.83E-58
Cladopus_008749	root	shoot	924.1792794	324.9823999	624.5808397	1.506113441	2.75E-59	4.04E-58
Cladopus_017251	root	shoot	1101.42657	382.8561613	742.1413656	1.525244056	3.08E-59	4.52E-58
Cladopus_022464	root	shoot	562.4970065	1410.735616	986.616311	-1.327694473	3.70E-59	5.42E-58
Cladopus_013259	root	shoot	23.47212448	297.3388297	160.4054771	-3.67175919	4.59E-59	6.72E-58
Cladopus_012694	root	shoot	501.8594352	123.4797614	312.6695983	2.020445012	4.69E-59	6.87E-58

Cladopus_026124	root	shoot	296.4603613	875.5470699	586.0037156	-1.561790481	4.96E-59	7.25E-58
Cladopus_017435	root	shoot	2021.881219	895.3843907	1458.632805	1.174675311	5.51E-59	8.06E-58
Cladopus_011477	root	shoot	1509.211914	620.830064	1065.020989	1.282567824	6.28E-59	9.16E-58
Cladopus_022802	root	shoot	1811.359285	556.1911159	1183.7752	1.704896468	1.01E-58	1.47E-57
Cladopus_011796	root	shoot	240.0477002	12.19263616	126.1201682	4.318071627	1.54E-58	2.23E-57
Cladopus_008460	root	shoot	4153.540376	1725.493827	2939.517101	1.267779302	1.70E-58	2.47E-57
Cladopus_005066	root	shoot	1237.814645	427.0564159	832.4355303	1.536794607	1.94E-58	2.81E-57
Cladopus_014673	root	shoot	1402.272061	476.5668033	939.4194319	1.559676068	2.68E-58	3.88E-57
Cladopus_003608	root	shoot	1993.74269	947.9030884	1470.822889	1.073111714	3.04E-58	4.39E-57
Cladopus_023964	root	shoot	669.3347328	203.2506823	436.2927076	1.721015564	3.42E-58	4.94E-57
Cladopus_014983	root	shoot	169.3920012	634.4890797	401.9405405	-1.900849504	3.58E-58	5.17E-57
Cladopus_001155	root	shoot	577.4944177	1447.586316	1012.540367	-1.327413455	3.82E-58	5.51E-57
Cladopus_002608	root	shoot	1136.588745	2326.539315	1731.56403	-1.034570774	4.48E-58	6.46E-57
Cladopus_004636	root	shoot	299.2472173	5.960367569	152.6037924	5.706535031	4.62E-58	6.66E-57
Cladopus_008323	root	shoot	3858.692	1810.787359	2834.73968	1.092308927	6.47E-58	9.31E-57
Cladopus_005622	root	shoot	1301.986234	2871.99098	2086.988607	-1.142879728	6.52E-58	9.37E-57
Cladopus_002470	root	shoot	31.74588787	306.005036	168.875462	-3.269012662	6.88E-58	9.89E-57
Cladopus_002168	root	shoot	1549.425679	666.4334113	1107.929545	1.219527069	8.03E-58	1.15E-56
Cladopus_009245	root	shoot	202.0012199	697.0513902	449.5263051	-1.784023811	1.28E-57	1.83E-56
Cladopus_022742	root	shoot	261.5018399	18.87124046	140.1865402	3.844434076	1.65E-57	2.37E-56
Cladopus_006463	root	shoot	463.9684529	1277.76402	870.8662362	-1.464108696	1.84E-57	2.64E-56
Cladopus_022444	root	shoot	4390.246906	2168.59818	3279.422543	1.017775635	1.92E-57	2.75E-56
Cladopus_015627	root	shoot	1219.022282	2659.033193	1939.027737	-1.126654521	2.87E-57	4.11E-56
Cladopus_024719	root	shoot	1771.230166	771.5402142	1271.38519	1.198442233	6.47E-57	9.25E-56
Cladopus_008022	root	shoot	716.68632	1724.641032	1220.663676	-1.266812665	7.97E-57	1.14E-55
Cladopus_014432	root	shoot	633.7383335	1493.255162	1063.496748	-1.234746931	8.30E-57	1.18E-55
Cladopus_018458	root	shoot	299.6120723	7.150101575	153.3810869	5.341493157	9.65E-57	1.37E-55
Cladopus_007988	root	shoot	1023.740635	2317.186116	1670.463376	-1.179871217	9.92E-57	1.41E-55
Cladopus_004255	root	shoot	2561.684786	1162.188787	1861.936787	1.140719789	1.09E-56	1.55E-55
Cladopus_020148	root	shoot	341.3312781	47.70491826	194.5180982	2.839418903	1.16E-56	1.65E-55

Cladopus_017041	root	shoot	1359.09598	422.9642044	891.0300923	1.686309681	1.27E-56	1.80E-55
Cladopus_013258	root	shoot	286.3769804	844.624538	565.5007592	-1.562726247	1.83E-56	2.59E-55
Cladopus_021228	root	shoot	1745.626519	3535.670696	2640.648608	-1.017558592	2.39E-56	3.39E-55
Cladopus_006730	root	shoot	4908.069765	9946.759489	7427.414627	-1.019460869	2.50E-56	3.53E-55
Cladopus_012911	root	shoot	263.2775263	801.245252	532.2613892	-1.605573493	2.58E-56	3.64E-55
Cladopus_008510	root	shoot	1434.477621	3070.350111	2252.413866	-1.099075957	2.76E-56	3.90E-55
Cladopus_000238	root	shoot	538.7468128	1494.363997	1016.555405	-1.468982877	3.57E-56	5.04E-55
Cladopus_005144	root	shoot	401.6639084	1064.648153	733.1560305	-1.405874471	5.11E-56	7.21E-55
Cladopus_020279	root	shoot	2177.903821	1020.279831	1599.091826	1.093906076	5.89E-56	8.30E-55
Cladopus_009007	root	shoot	4012.6041	2001.036572	3006.820336	1.004091299	7.80E-56	1.10E-54
Cladopus_017705	root	shoot	1614.323583	716.2380511	1165.280817	1.174988237	7.90E-56	1.11E-54
Cladopus_001170	root	shoot	558.2668979	1290.689251	924.4780745	-1.20837527	1.31E-55	1.84E-54
Cladopus_010193	root	shoot	43.84576721	330.2411825	187.0434749	-2.91991942	1.34E-55	1.88E-54
Cladopus_002956	root	shoot	2169.336389	890.8842633	1530.110326	1.285654166	1.57E-55	2.20E-54
Cladopus_012720	root	shoot	1384.659124	629.2007626	1006.929943	1.137035489	2.07E-55	2.90E-54
Cladopus_003962	root	shoot	1079.240333	394.474942	736.8576374	1.450480517	2.18E-55	3.05E-54
Cladopus_007952	root	shoot	632.8475056	169.8433042	401.3454049	1.901441787	2.69E-55	3.76E-54
Cladopus_012266	root	shoot	969.8787348	380.8969067	675.3878207	1.350007241	3.46E-55	4.83E-54
Cladopus_005998	root	shoot	7.711291176	335.2866727	171.4989819	-5.455845374	3.53E-55	4.92E-54
Cladopus_012322	root	shoot	378.9969167	1055.30336	717.1501385	-1.479622753	4.96E-55	6.92E-54
Cladopus_009238	root	shoot	2015.634716	4546.500429	3281.067572	-1.174564148	4.98E-55	6.93E-54
Cladopus_009923	root	shoot	289.8066579	35.94529716	162.8759775	3.025086798	4.99E-55	6.95E-54
Cladopus_001045	root	shoot	39.39403742	329.3298645	184.361951	-3.053056737	6.11E-55	8.49E-54
Cladopus_007451	root	shoot	78.65039279	403.6976839	241.1740383	-2.365736518	6.87E-55	9.54E-54
Cladopus_018489	root	shoot	1406.840765	515.7042993	961.2725323	1.44713706	7.83E-55	1.09E-53
Cladopus_021994	root	shoot	296.1678	860.6353445	578.4015723	-1.537228917	8.46E-55	1.17E-53
Cladopus_024943	root	shoot	79.66526777	411.0268837	245.3460757	-2.373900339	8.51E-55	1.18E-53
Cladopus_014375	root	shoot	1390.618096	3566.826324	2478.72221	-1.360353651	8.98E-55	1.24E-53
Cladopus_012758	root	shoot	1300.550923	533.1645391	916.857731	1.289907113	9.32E-55	1.29E-53
Cladopus_006977	root	shoot	90.70966277	421.4466861	256.0781745	-2.21880393	1.13E-54	1.56E-53

Cladopus_006868	root	shoot	809.9140309	1765.102089	1287.50806	-1.125114853	1.39E-54	1.92E-53
Cladopus_018502	root	shoot	1189.213419	2442.011981	1815.6127	-1.039071659	1.70E-54	2.35E-53
Cladopus_002548	root	shoot	5481.139535	2646.264069	4063.701802	1.051429691	2.19E-54	3.02E-53
Cladopus_016597	root	shoot	786.4781851	286.4480489	536.463117	1.45947169	2.26E-54	3.10E-53
Cladopus_000255	root	shoot	1537.125479	676.0185675	1106.572023	1.185824915	2.35E-54	3.23E-53
Cladopus_013693	root	shoot	694.4174974	1585.461227	1139.939362	-1.193033703	2.38E-54	3.26E-53
Cladopus_012546	root	shoot	281.2811933	948.5482937	614.9147435	-1.748448929	2.56E-54	3.51E-53
Cladopus_002586	root	shoot	47.62731356	356.5526381	202.0899758	-2.890671619	3.36E-54	4.61E-53
Cladopus_010629	root	shoot	811.2187555	283.1029385	547.160847	1.519511097	3.38E-54	4.63E-53
Cladopus_016667	root	shoot	772.2538325	1848.380258	1310.317045	-1.261258109	3.79E-54	5.18E-53
Cladopus_021036	root	shoot	561.4817676	1432.275101	996.8784344	-1.348536931	4.06E-54	5.55E-53
Cladopus_002308	root	shoot	1030.571767	433.0950013	731.8333843	1.250582438	1.00E-53	1.37E-52
Cladopus_013183	root	shoot	6339.171929	13239.67494	9789.423436	-1.062860784	1.13E-53	1.54E-52
Cladopus_015356	root	shoot	267.2671958	30.24484241	148.7560191	3.159373356	1.22E-53	1.66E-52
Cladopus_005489	root	shoot	100.6850818	489.2658615	294.9754716	-2.276577484	1.55E-53	2.10E-52
Cladopus_014614	root	shoot	1196.784323	500.520831	848.6525769	1.258746195	1.72E-53	2.33E-52
Cladopus_008973	root	shoot	3386.539937	8357.032588	5871.786262	-1.303672745	1.78E-53	2.42E-52
Cladopus_022692	root	shoot	22.97019795	279.5027262	151.2364621	-3.586416221	1.87E-53	2.54E-52
Cladopus_012765	root	shoot	403.8433392	1052.170737	728.0070379	-1.382930208	1.98E-53	2.68E-52
Cladopus_023896	root	shoot	1635.857785	4484.990449	3060.424117	-1.456440572	2.01E-53	2.72E-52
Cladopus_004839	root	shoot	1439.175615	2892.398325	2165.78697	-1.008121312	2.31E-53	3.12E-52
Cladopus_003251	root	shoot	262.7049081	770.2422109	516.4735595	-1.549518985	2.47E-53	3.34E-52
Cladopus_013192	root	shoot	549.1574163	1276.918278	913.037847	-1.21622666	3.57E-53	4.81E-52
Cladopus_006904	root	shoot	478.6906473	1149.523323	814.1069853	-1.26416155	3.98E-53	5.35E-52
Cladopus_008058	root	shoot	2570.847379	1191.920104	1881.383741	1.108644559	4.09E-53	5.50E-52
Cladopus_004634	root	shoot	262.2412392	5.996186836	134.118713	5.514769309	4.52E-53	6.07E-52
Cladopus_018604	root	shoot	957.3871035	2062.752329	1510.069716	-1.108732975	4.58E-53	6.15E-52
Cladopus_015337	root	shoot	410.9033893	1043.092017	726.9977032	-1.344509444	4.71E-53	6.31E-52
Cladopus_018853	root	shoot	262.7206334	28.91464158	145.8176375	3.19667357	4.71E-53	6.31E-52
Cladopus_016815	root	shoot	19.90935274	262.5369388	141.2231458	-3.722324616	4.92E-53	6.60E-52

Cladopus_005207	root	shoot	254.6074402	12.50035644	133.5538983	4.340204001	6.47E-53	8.64E-52
Cladopus_001965	root	shoot	81.87380405	426.4602091	254.1670066	-2.373806309	9.43E-53	1.26E-51
Cladopus_010189	root	shoot	770.0205473	257.1270356	513.5737914	1.588854999	1.43E-52	1.90E-51
Cladopus_017520	root	shoot	511.8825252	108.9795884	310.4310568	2.232855366	1.56E-52	2.08E-51
Cladopus_015955	root	shoot	857.0042511	306.0857072	581.5449792	1.489585542	1.84E-52	2.45E-51
Cladopus_004352	root	shoot	225.3777582	10.96256657	118.1701624	4.341595152	1.98E-52	2.63E-51
Cladopus_005624	root	shoot	1672.565174	779.5669373	1226.066056	1.101532285	2.10E-52	2.78E-51
Cladopus_003937	root	shoot	1175.471455	440.6125565	808.0420058	1.420227919	2.11E-52	2.80E-51
Cladopus_021233	root	shoot	676.0233377	1500.211058	1088.117198	-1.151786828	2.58E-52	3.42E-51
Cladopus_003882	root	shoot	1556.57502	619.8494797	1088.21225	1.331615872	2.76E-52	3.65E-51
Cladopus_001135	root	shoot	1829.873889	838.6729085	1334.273399	1.124295604	4.03E-52	5.32E-51
Cladopus_003705	root	shoot	2551.771339	5500.516614	4026.143976	-1.108933748	4.32E-52	5.69E-51
Cladopus_013082	root	shoot	179.3467407	587.1413123	383.2440265	-1.709748615	5.27E-52	6.93E-51
Cladopus_001104	root	shoot	2865.173262	1254.908403	2060.040833	1.19280131	6.92E-52	9.10E-51
Cladopus_024342	root	shoot	872.9681313	1875.381308	1374.17472	-1.104675586	7.66E-52	1.01E-50
Cladopus_004314	root	shoot	373.4764551	953.2181863	663.3473207	-1.353434371	7.83E-52	1.03E-50
Cladopus_008905	root	shoot	540.8617353	1300.237174	920.5494549	-1.267521991	7.87E-52	1.03E-50
Cladopus_016722	root	shoot	33.77105648	284.5648662	159.1679613	-3.080912518	9.87E-52	1.29E-50
Cladopus_019197	root	shoot	2065.11758	4333.06335	3199.090465	-1.069778513	1.03E-51	1.36E-50
Cladopus_015751	root	shoot	967.2045313	2361.914901	1664.559716	-1.290087802	1.08E-51	1.42E-50
Cladopus_000269	root	shoot	4766.296666	1973.409988	3369.853327	1.273686912	1.30E-51	1.70E-50
Cladopus_014303	root	shoot	563.5931002	1513.764828	1038.678964	-1.427037875	1.33E-51	1.73E-50
Cladopus_017940	root	shoot	385.6046797	1136.981735	761.2932073	-1.563608073	1.37E-51	1.80E-50
Cladopus_015544	root	shoot	349.7449467	896.7196288	623.2322877	-1.35855338	1.40E-51	1.83E-50
Cladopus_014448	root	shoot	1043.514922	401.1282375	722.3215795	1.379050931	2.15E-51	2.81E-50
Cladopus_005411	root	shoot	386.8926892	967.5723403	677.2325147	-1.323946649	2.78E-51	3.62E-50
Cladopus_015676	root	shoot	2529.370473	1251.490318	1890.430395	1.015015701	3.73E-51	4.84E-50
Cladopus_023190	root	shoot	1348.019654	434.7037635	891.3617088	1.633729481	3.74E-51	4.86E-50
Cladopus_006825	root	shoot	1064.665722	452.5217394	758.5937308	1.234909716	4.91E-51	6.36E-50
Cladopus_015031	root	shoot	138.4814588	567.7576538	353.1195563	-2.042432002	5.58E-51	7.22E-50

Cladopus_015557	root	shoot	273.7916431	754.1040504	513.9478468	-1.461308529	5.65E-51	7.31E-50
Cladopus_020568	root	shoot	1597.178899	3195.835083	2396.506991	-1.001400986	6.40E-51	8.28E-50
Cladopus_013432	root	shoot	1505.72567	558.3100068	1032.017839	1.433691097	7.89E-51	1.02E-49
Cladopus_020264	root	shoot	204.6924896	629.971842	417.3321658	-1.622100866	8.78E-51	1.13E-49
Cladopus_002705	root	shoot	5314.888524	2401.552653	3858.220588	1.146788202	1.15E-50	1.48E-49
Cladopus_020177	root	shoot	1281.993182	2780.436838	2031.21501	-1.118444731	1.20E-50	1.54E-49
Cladopus_001399	root	shoot	923.009281	2319.674371	1621.341826	-1.328155915	1.56E-50	2.00E-49
Cladopus_014228	root	shoot	53.15693784	341.3122487	197.2345933	-2.687675059	1.83E-50	2.34E-49
Cladopus_022783	root	shoot	268.3583418	33.90603755	151.1321897	2.980880109	2.15E-50	2.76E-49
Cladopus_008967	root	shoot	288.6887792	848.6665093	568.6776443	-1.55181047	2.47E-50	3.17E-49
Cladopus_006542	root	shoot	8662.37099	3405.149259	6033.760124	1.347479657	3.96E-50	5.05E-49
Cladopus_007623	root	shoot	132.1782705	515.3572209	323.7677457	-1.957914539	4.22E-50	5.39E-49
Cladopus_012314	root	shoot	251.0095163	28.79534092	139.9024286	3.094570287	4.34E-50	5.53E-49
Cladopus_014331	root	shoot	1659.023685	476.1420717	1067.582878	1.801150226	4.86E-50	6.19E-49
Cladopus_024431	root	shoot	9460.33331	4262.1069	6861.220105	1.150697313	5.05E-50	6.43E-49
Cladopus_023992	root	shoot	150.5614083	517.6478777	334.104643	-1.781303211	5.10E-50	6.49E-49
Cladopus_018015	root	shoot	823.5609227	1704.434707	1263.997815	-1.049875462	6.75E-50	8.56E-49
Cladopus_022116	root	shoot	2843.125971	7444.278348	5143.702159	-1.389380524	6.82E-50	8.65E-49
Cladopus_019443	root	shoot	404.2495638	1005.266136	704.7578498	-1.314421013	7.62E-50	9.66E-49
Cladopus_022355	root	shoot	1768.328987	4684.443246	3226.386116	-1.406667713	7.80E-50	9.88E-49
Cladopus_012475	root	shoot	454.4230435	1131.905623	793.1643331	-1.31538082	8.22E-50	1.04E-48
Cladopus_008140	root	shoot	939.3919846	2048.819939	1494.105962	-1.126043172	8.33E-50	1.05E-48
Cladopus_001564	root	shoot	927.7312735	381.8717251	654.8014993	1.281059589	9.15E-50	1.16E-48
Cladopus_020926	root	shoot	243.953555	740.8682056	492.4108803	-1.599596383	1.28E-49	1.61E-48
Cladopus_024782	root	shoot	1233.179787	540.5376924	886.8587399	1.190422857	1.32E-49	1.67E-48
Cladopus_004853	root	shoot	160.0638671	543.0155876	351.5397273	-1.763954947	1.47E-49	1.85E-48
Cladopus_016323	root	shoot	1107.833139	2235.545788	1671.689463	-1.012142364	1.58E-49	1.99E-48
Cladopus_014085	root	shoot	1585.770263	745.9208106	1165.845537	1.086427816	1.60E-49	2.02E-48
Cladopus_014999	root	shoot	16194.38307	7002.081304	11598.23219	1.209808661	1.71E-49	2.15E-48
Cladopus_022075	root	shoot	183.4354576	589.1614006	386.2984291	-1.686855062	1.72E-49	2.16E-48

Cladopus_010062	root	shoot	794.7499672	1628.602295	1211.676131	-1.035080078	1.87E-49	2.35E-48
Cladopus_011454	root	shoot	1373.298657	544.6305721	958.9646146	1.336707977	2.66E-49	3.33E-48
Cladopus_001998	root	shoot	1420.310121	639.1272393	1029.71868	1.154513315	3.74E-49	4.69E-48
Cladopus_003735	root	shoot	711.4733848	1638.466892	1174.970139	-1.205392677	3.75E-49	4.69E-48
Cladopus_009020	root	shoot	728.9777253	1686.667203	1207.822464	-1.211831238	4.87E-49	6.09E-48
Cladopus_012182	root	shoot	54.48889175	320.9203871	187.7046394	-2.555180599	5.35E-49	6.69E-48
Cladopus_004683	root	shoot	196.7345671	617.5394194	407.1369932	-1.653133407	5.90E-49	7.37E-48
Cladopus_009535	root	shoot	1171.031229	535.2807631	853.1559962	1.130468903	6.28E-49	7.84E-48
Cladopus_012458	root	shoot	566.4947169	1502.303681	1034.399199	-1.407464333	6.60E-49	8.23E-48
Cladopus_002587	root	shoot	40.89263972	303.2063945	172.0495171	-2.878264437	7.97E-49	9.93E-48
Cladopus_010424	root	shoot	1281.042837	564.016603	922.5297198	1.186834291	8.45E-49	1.05E-47
Cladopus_023110	root	shoot	211.9605367	13.74441291	112.8524748	3.996707158	9.54E-49	1.19E-47
Cladopus_009365	root	shoot	841.7028798	1807.624384	1324.663632	-1.103646214	1.01E-48	1.25E-47
Cladopus_008885	root	shoot	1673.412009	3582.481508	2627.946758	-1.09906748	1.13E-48	1.41E-47
Cladopus_013724	root	shoot	234.1861369	29.28134598	131.7337415	3.013466913	1.13E-48	1.41E-47
Cladopus_012473	root	shoot	81.42313192	371.6726851	226.5479085	-2.186253455	1.34E-48	1.66E-47
Cladopus_008507	root	shoot	871.4370843	2043.435569	1457.436327	-1.231333527	1.40E-48	1.74E-47
Cladopus_001713	root	shoot	4069.261195	1692.317204	2880.7892	1.266572578	1.63E-48	2.01E-47
Cladopus_021849	root	shoot	486.1642214	139.0445229	312.6043722	1.813284284	1.75E-48	2.17E-47
Cladopus_018798	root	shoot	184.9376498	629.5833053	407.2604775	-1.7664533	1.95E-48	2.41E-47
Cladopus_007654	root	shoot	103.4550923	423.1670488	263.3110705	-2.029315529	2.55E-48	3.15E-47
Cladopus_018367	root	shoot	628.3103567	220.5773252	424.443841	1.509734064	2.72E-48	3.35E-47
Cladopus_014318	root	shoot	846.7190761	309.9939325	578.3565043	1.451043199	3.42E-48	4.22E-47
Cladopus_001829	root	shoot	284.4454126	39.74310904	162.0942608	2.839577665	3.64E-48	4.49E-47
Cladopus_015632	root	shoot	4776.839837	661.6980575	2719.268947	2.85127774	4.29E-48	5.28E-47
Cladopus_017191	root	shoot	816.5099196	327.2024619	571.8561908	1.320937593	5.88E-48	7.24E-47
Cladopus_009027	root	shoot	285.3003281	42.57542776	163.9378779	2.773648117	6.21E-48	7.64E-47
Cladopus_001492	root	shoot	2070.562695	962.4239086	1516.493302	1.106059383	7.74E-48	9.50E-47
Cladopus_025736	root	shoot	3919.351001	1903.397224	2911.374112	1.043175096	8.02E-48	9.85E-47
Cladopus_010233	root	shoot	977.3799175	400.2375004	688.8087089	1.290483606	9.35E-48	1.14E-46

Cladopus_006164	root	shoot	567.3563317	164.3178011	365.8370664	1.783854018	1.09E-47	1.34E-46
Cladopus_016395	root	shoot	120.375283	533.2258125	326.8005478	-2.155677461	1.15E-47	1.41E-46
Cladopus_017439	root	shoot	1092.667446	2207.35078	1650.009113	-1.015083214	1.19E-47	1.45E-46
Cladopus_023441	root	shoot	626.9333277	1412.120141	1019.526734	-1.172304176	1.23E-47	1.50E-46
Cladopus_000352	root	shoot	721.387151	1525.075905	1123.231528	-1.0786453	1.49E-47	1.82E-46
Cladopus_003358	root	shoot	1096.910696	464.6556821	780.7831893	1.236672134	1.61E-47	1.96E-46
Cladopus_022448	root	shoot	594.6792014	187.8097397	391.2444705	1.671924384	1.71E-47	2.08E-46
Cladopus_009426	root	shoot	367.622941	89.83082646	228.7268837	2.036743005	1.84E-47	2.24E-46
Cladopus_008055	root	shoot	1099.687524	433.0951467	766.3913355	1.343899674	1.93E-47	2.35E-46
Cladopus_010508	root	shoot	28.15761104	257.1232645	142.6404378	-3.185460101	2.57E-47	3.12E-46
Cladopus_021455	root	shoot	338.5709228	949.117467	643.8441949	-1.489179643	2.68E-47	3.25E-46
Cladopus_006014	root	shoot	1955.246267	968.7680684	1462.007168	1.01171543	3.23E-47	3.92E-46
Cladopus_007749	root	shoot	772.2746471	291.9204128	532.0975299	1.402895792	3.84E-47	4.65E-46
Cladopus_018293	root	shoot	1236.266251	541.0739416	888.6700962	1.190896489	4.66E-47	5.63E-46
Cladopus_007486	root	shoot	2100.826653	1017.39571	1559.111181	1.04490519	4.91E-47	5.93E-46
Cladopus_009973	root	shoot	822.0155187	1770.872754	1296.444137	-1.108838029	5.15E-47	6.22E-46
Cladopus_018613	root	shoot	16.71033704	234.6827411	125.6965391	-3.804184942	6.96E-47	8.38E-46
Cladopus_024989	root	shoot	513.9308407	162.630742	338.2807914	1.666255871	7.21E-47	8.67E-46
Cladopus_024432	root	shoot	17.63371458	236.5104143	127.0720644	-3.750318319	8.16E-47	9.81E-46
Cladopus_000855	root	shoot	1065.233774	458.658831	761.9463024	1.215458792	9.49E-47	1.14E-45
Cladopus_024035	root	shoot	1955.599783	913.9963866	1434.798085	1.099053539	1.57E-46	1.88E-45
Cladopus_001600	root	shoot	329.3770013	843.3568787	586.36694	-1.355547403	1.59E-46	1.90E-45
Cladopus_011538	root	shoot	873.5997143	291.962747	582.7812306	1.583682963	1.70E-46	2.03E-45
Cladopus_021801	root	shoot	193.357881	584.7827179	389.0702995	-1.597700685	1.77E-46	2.11E-45
Cladopus_016811	root	shoot	21.8603578	250.466121	136.1632394	-3.540227176	3.05E-46	3.64E-45
Cladopus_004037	root	shoot	228.6334814	676.8922149	452.7628481	-1.56625822	3.18E-46	3.79E-45
Cladopus_022514	root	shoot	342.5501979	906.2663711	624.4082845	-1.402033605	3.19E-46	3.80E-45
Cladopus_003901	root	shoot	103.457444	401.8100697	252.6337568	-1.956111377	3.39E-46	4.04E-45
Cladopus_014386	root	shoot	405.4973302	107.5687974	256.5330638	1.91026666	4.12E-46	4.91E-45
Cladopus_007322	root	shoot	233.9681056	680.5098925	457.238999	-1.543528272	4.32E-46	5.13E-45

Cladopus_018317	root	shoot	499.7223444	150.8712663	325.2968054	1.730370848	5.13E-46	6.10E-45
Cladopus_003700	root	shoot	275.7799183	51.56652318	163.6732208	2.42234425	7.02E-46	8.33E-45
Cladopus_013479	root	shoot	455.8888348	128.9482771	292.418556	1.821676816	7.03E-46	8.33E-45
Cladopus_008534	root	shoot	274.4524196	729.8906972	502.1715584	-1.408990523	7.72E-46	9.13E-45
Cladopus_009858	root	shoot	2912.659473	6809.50281	4861.081141	-1.225993043	8.23E-46	9.74E-45
Cladopus_011286	root	shoot	501.6842493	1183.017595	842.3509221	-1.234622161	8.30E-46	9.81E-45
Cladopus_011885	root	shoot	24.30920704	240.6292671	132.4692371	-3.320061738	8.57E-46	1.01E-44
Cladopus_000865	root	shoot	367.6307394	912.6039475	640.1173434	-1.314066889	8.71E-46	1.03E-44
Cladopus_015773	root	shoot	853.4255074	1759.603817	1306.514662	-1.045303654	8.80E-46	1.04E-44
Cladopus_010030	root	shoot	79.18810312	359.5475467	219.3678249	-2.181286892	8.96E-46	1.06E-44
Cladopus_017195	root	shoot	36.33158368	278.2086353	157.2701095	-2.928503554	9.30E-46	1.10E-44
Cladopus_004798	root	shoot	512.5087496	1160.017801	836.2632753	-1.178213484	9.65E-46	1.14E-44
Cladopus_021476	root	shoot	114.695601	437.1723334	275.9339672	-1.934216197	1.04E-45	1.22E-44
Cladopus_014487	root	shoot	505.1603492	161.1764316	333.1683904	1.647611506	1.10E-45	1.29E-44
Cladopus_011199	root	shoot	192.3812637	15.39565468	103.8884592	3.682241251	1.10E-45	1.30E-44
Cladopus_005971	root	shoot	1892.395706	894.2133862	1393.304546	1.080970269	1.11E-45	1.30E-44
Cladopus_008539	root	shoot	104.1963443	403.3824917	253.789418	-1.956257003	1.15E-45	1.34E-44
Cladopus_013726	root	shoot	613.8686027	215.5957734	414.7321881	1.511951761	1.16E-45	1.36E-44
Cladopus_007172	root	shoot	444.4582801	114.294543	279.3764115	1.956333609	1.80E-45	2.11E-44
Cladopus_011984	root	shoot	2412.170623	5255.862108	3834.016365	-1.124459924	1.88E-45	2.19E-44
Cladopus_011861	root	shoot	651.5020514	1389.822451	1020.662251	-1.093143515	2.12E-45	2.47E-44
Cladopus_019947	root	shoot	1448.288096	618.2326399	1033.260368	1.227429271	2.73E-45	3.18E-44
Cladopus_018246	root	shoot	873.2693668	350.257388	611.7633774	1.320824367	2.91E-45	3.39E-44
Cladopus_008337	root	shoot	546.0616591	1261.063929	903.5627943	-1.204637022	2.93E-45	3.41E-44
Cladopus_007232	root	shoot	423.3623213	105.1306379	264.2464796	2.006395417	3.07E-45	3.57E-44
Cladopus_016179	root	shoot	186.371007	567.9289027	377.1499548	-1.608101915	3.21E-45	3.73E-44
Cladopus_021208	root	shoot	377.9566628	916.5164628	647.2365628	-1.2806109	3.48E-45	4.04E-44
Cladopus_005034	root	shoot	257.7221463	728.8923403	493.3072433	-1.501219455	3.99E-45	4.63E-44
Cladopus_014201	root	shoot	1484.584712	3967.098	2725.841356	-1.419426847	4.45E-45	5.16E-44
Cladopus_025152	root	shoot	946.4522765	2310.844667	1628.648472	-1.289757237	4.55E-45	5.28E-44

Cladopus_023592	root	shoot	401.0337132	101.1529182	251.0933157	1.996468625	6.21E-45	7.18E-44
Cladopus_005157	root	shoot	1368.492934	566.5363242	967.514629	1.273770869	8.94E-45	1.03E-43
Cladopus_002427	root	shoot	385.6602883	1096.472742	741.066515	-1.505429171	8.95E-45	1.03E-43
Cladopus_003724	root	shoot	473.4873867	1087.653626	780.5705062	-1.20165532	1.19E-44	1.38E-43
Cladopus_016124	root	shoot	442.7992193	1090.945538	766.8723788	-1.298415631	1.31E-44	1.51E-43
Cladopus_012778	root	shoot	1131.720762	525.8954295	828.8080959	1.107434014	1.32E-44	1.52E-43
Cladopus_013475	root	shoot	73.04078549	343.4311377	208.2359616	-2.236947525	1.40E-44	1.61E-43
Cladopus_013058	root	shoot	865.3221022	355.5867674	610.4544348	1.283967103	1.43E-44	1.65E-43
Cladopus_014747	root	shoot	627.073486	1392.009859	1009.541672	-1.150339013	1.57E-44	1.81E-43
Cladopus_018470	root	shoot	1906.089766	920.5850735	1413.33742	1.049235956	1.68E-44	1.92E-43
Cladopus_011395	root	shoot	975.1763265	2064.847014	1520.01167	-1.084132991	1.72E-44	1.97E-43
Cladopus_019902	root	shoot	329.3339157	64.61068167	196.9722987	2.342600109	1.91E-44	2.19E-43
Cladopus_018328	root	shoot	4033.342996	9222.179708	6627.761352	-1.193706224	2.04E-44	2.33E-43
Cladopus_012206	root	shoot	503.6347489	1164.565298	834.1000233	-1.211909032	2.06E-44	2.36E-43
Cladopus_023169	root	shoot	591.5059414	1313.242338	952.3741396	-1.148341929	2.32E-44	2.66E-43
Cladopus_001922	root	shoot	519.5634858	138.1698338	328.8666598	1.90999017	2.68E-44	3.06E-43
Cladopus_014229	root	shoot	98.03305712	388.5587601	243.2959086	-1.985789843	3.01E-44	3.43E-43
Cladopus_010452	root	shoot	717.0938879	1556.232875	1136.663381	-1.11900539	3.38E-44	3.86E-43
Cladopus_009620	root	shoot	1501.715782	717.3238847	1109.519834	1.067933994	3.40E-44	3.88E-43
Cladopus_002049	root	shoot	473.3473529	125.9404215	299.6438872	1.920147045	3.66E-44	4.16E-43
Cladopus_015953	root	shoot	195.5475905	11.43606254	103.4918265	4.067643421	3.98E-44	4.52E-43
Cladopus_021771	root	shoot	357.7159987	892.8546098	625.2853042	-1.318620498	4.21E-44	4.78E-43
Cladopus_024740	root	shoot	46799.97809	23170.41869	34985.19839	1.014252503	4.21E-44	4.78E-43
Cladopus_013679	root	shoot	476.1147716	1063.555804	769.8352878	-1.16007826	4.36E-44	4.95E-43
Cladopus_023925	root	shoot	996.4229496	2197.563962	1596.993456	-1.142035105	4.48E-44	5.08E-43
Cladopus_012871	root	shoot	290.3872072	734.9135414	512.6503743	-1.338402651	5.32E-44	6.02E-43
Cladopus_024627	root	shoot	1433.824532	653.0293521	1043.426942	1.13531827	5.72E-44	6.47E-43
Cladopus_024647	root	shoot	341.3045349	84.15101893	212.7277769	2.026776574	5.88E-44	6.65E-43
Cladopus_018459	root	shoot	345.9439695	73.66513217	209.8045508	2.242882041	6.50E-44	7.34E-43
Cladopus_013617	root	shoot	918.7634366	2046.033524	1482.39848	-1.15322613	6.53E-44	7.37E-43

Cladopus_014176	root	shoot	959.2611113	417.1938834	688.2274974	1.205120031	7.29E-44	8.22E-43
Cladopus_008353	root	shoot	120.5187845	443.0116279	281.7652062	-1.878654155	7.50E-44	8.45E-43
Cladopus_013831	root	shoot	833.9260888	1878.681652	1356.30387	-1.173125291	7.56E-44	8.52E-43
Cladopus_000083	root	shoot	3081.190579	1441.78513	2261.487854	1.096140921	9.32E-44	1.05E-42
Cladopus_024078	root	shoot	215.1110201	4.753462824	109.9322415	5.549522925	9.85E-44	1.11E-42
Cladopus_014590	root	shoot	18.14975796	238.8284635	128.4891107	-3.727769425	1.15E-43	1.29E-42
Cladopus_001495	root	shoot	901.1316879	1817.867595	1359.499641	-1.013321202	1.22E-43	1.37E-42
Cladopus_006540	root	shoot	1003.903855	2050.676487	1527.290171	-1.030258282	1.45E-43	1.62E-42
Cladopus_007404	root	shoot	724.3311029	1794.299858	1259.315481	-1.310996323	1.59E-43	1.78E-42
Cladopus_007254	root	shoot	389.7140868	102.7078768	246.2109818	1.917182366	1.89E-43	2.12E-42
Cladopus_019420	root	shoot	522.8773965	153.9946535	338.436025	1.765578454	2.19E-43	2.45E-42
Cladopus_018055	root	shoot	2898.114904	1422.358997	2160.236951	1.028539661	2.32E-43	2.59E-42
Cladopus_007674	root	shoot	82.5770427	401.5630415	242.0700421	-2.286667726	2.73E-43	3.04E-42
Cladopus_017140	root	shoot	575.6727909	1332.214889	953.9438398	-1.212957272	3.26E-43	3.63E-42
Cladopus_023105	root	shoot	241.8039558	29.65722832	135.7305921	3.043154384	3.29E-43	3.66E-42
Cladopus_015202	root	shoot	290.9263975	834.0799597	562.5031786	-1.524340512	3.34E-43	3.72E-42
Cladopus_017473	root	shoot	191.9180938	14.46065096	103.1893724	3.745559016	3.43E-43	3.82E-42
Cladopus_016434	root	shoot	1227.951015	540.3365394	884.1437773	1.186912172	3.95E-43	4.39E-42
Cladopus_003565	root	shoot	513.9480484	1111.641123	812.7945856	-1.112236493	4.68E-43	5.19E-42
Cladopus_000951	root	shoot	496.1458262	1080.351528	788.2486769	-1.123364684	4.92E-43	5.46E-42
Cladopus_017860	root	shoot	403.990919	104.95073	254.4708245	1.948699329	4.93E-43	5.46E-42
Cladopus_019438	root	shoot	288.9768788	797.9821452	543.479512	-1.468368982	5.58E-43	6.18E-42
Cladopus_018720	root	shoot	950.8707093	1916.404357	1433.637533	-1.012656195	5.68E-43	6.29E-42
Cladopus_019336	root	shoot	1532.078554	3905.003993	2718.541273	-1.351097482	1.05E-42	1.15E-41
Cladopus_022872	root	shoot	220.4449035	650.3426788	435.3937912	-1.560334413	1.45E-42	1.60E-41
Cladopus_014396	root	shoot	232.5499853	34.96396376	133.7569745	2.723053197	1.60E-42	1.76E-41
Cladopus_015117	root	shoot	1256.16227	405.4364044	830.7993374	1.632422344	1.78E-42	1.96E-41
Cladopus_005413	root	shoot	186.1223902	12.30675429	99.21457225	3.945023434	1.93E-42	2.12E-41
Cladopus_003193	root	shoot	525.9870052	158.2419849	342.1144951	1.73371974	2.65E-42	2.91E-41
Cladopus_020041	root	shoot	1054.291528	435.2122652	744.7518965	1.275972583	2.73E-42	3.00E-41

Cladopus_014532	root	shoot	1026.702555	2120.537195	1573.619875	-1.048498748	2.80E-42	3.07E-41
Cladopus_003425	root	shoot	103.4694533	385.6388192	244.5541363	-1.897766564	3.09E-42	3.39E-41
Cladopus_011028	root	shoot	244.9257131	690.6144218	467.7700674	-1.492301969	3.27E-42	3.58E-41
Cladopus_018555	root	shoot	75.60452974	352.5155587	214.0600442	-2.227559331	3.29E-42	3.60E-41
Cladopus_008211	root	shoot	377.1730708	99.66611961	238.4195952	1.927347039	3.42E-42	3.73E-41
Cladopus_005837	root	shoot	2230.338647	4600.389582	3415.364114	-1.045446367	3.62E-42	3.95E-41
Cladopus_007775	root	shoot	1247.43719	604.1022098	925.7697001	1.045961382	4.64E-42	5.06E-41
Cladopus_025408	root	shoot	1657.917777	753.0954924	1205.506635	1.140020267	4.92E-42	5.36E-41
Cladopus_000710	root	shoot	606.8085396	1515.987993	1061.398266	-1.323130015	5.09E-42	5.55E-41
Cladopus_023989	root	shoot	1058.754871	2302.365693	1680.560282	-1.122681758	5.36E-42	5.83E-41
Cladopus_014633	root	shoot	45.12101357	298.2943353	171.7076744	-2.739727775	5.70E-42	6.19E-41
Cladopus_022798	root	shoot	429.5375022	1093.22429	761.3808961	-1.345148656	5.93E-42	6.44E-41
Cladopus_012658	root	shoot	144.5708372	540.1888913	342.3798643	-1.903720118	6.25E-42	6.79E-41
Cladopus_012775	root	shoot	411.6510844	96.47627445	254.0636794	2.090564372	6.92E-42	7.49E-41
Cladopus_005750	root	shoot	586.5312328	216.9213126	401.7262727	1.439195494	7.24E-42	7.84E-41
Cladopus_013782	root	shoot	466.3065065	129.2238829	297.7651947	1.861323416	8.75E-42	9.47E-41
Cladopus_012499	root	shoot	243.3629752	638.8974403	441.1302078	-1.394429787	9.15E-42	9.89E-41
Cladopus_004947	root	shoot	294.5523173	720.4217328	507.4870251	-1.290438183	1.18E-41	1.28E-40
Cladopus_018366	root	shoot	1869.39814	868.6245423	1369.011341	1.106403357	1.25E-41	1.34E-40
Cladopus_002881	root	shoot	576.0426382	137.9047404	356.9736893	2.067155054	1.32E-41	1.42E-40
Cladopus_007311	root	shoot	332.6980934	918.5362604	625.6171769	-1.46882925	1.36E-41	1.46E-40
Cladopus_024832	root	shoot	855.8916929	376.1297496	616.0107213	1.186043419	1.37E-41	1.47E-40
Cladopus_018155	root	shoot	431.6447508	997.0601731	714.3524619	-1.205324455	1.45E-41	1.56E-40
Cladopus_017539	root	shoot	306.9000662	824.7636807	565.8318734	-1.424060808	1.83E-41	1.96E-40
Cladopus_010101	root	shoot	773.9851935	1822.226593	1298.105893	-1.236888137	1.94E-41	2.08E-40
Cladopus_013093	root	shoot	648.7884831	1404.675407	1026.731945	-1.116530843	2.00E-41	2.15E-40
Cladopus_000223	root	shoot	1328.31096	535.6900115	932.0004858	1.308294671	2.10E-41	2.25E-40
Cladopus_007121	root	shoot	550.712587	166.2192588	358.4659229	1.726098596	2.70E-41	2.89E-40
Cladopus_003911	root	shoot	455.5624711	1050.669798	753.1161346	-1.206348273	2.91E-41	3.12E-40
Cladopus_013849	root	shoot	16.57080225	206.4411917	111.505997	-3.623217689	3.16E-41	3.38E-40

Cladopus_012016	root	shoot	1049.715477	487.2866258	768.5010512	1.106264039	3.25E-41	3.47E-40
Cladopus_017569	root	shoot	1935.548604	682.4946677	1309.021636	1.502908887	3.48E-41	3.71E-40
Cladopus_004867	root	shoot	18.6965048	210.4548137	114.5756592	-3.489042528	4.66E-41	4.96E-40
Cladopus_001438	root	shoot	2214.88827	990.8726734	1602.880472	1.161436244	5.32E-41	5.66E-40
Cladopus_013694	root	shoot	531.5550303	163.5903884	347.5727094	1.70476456	5.81E-41	6.17E-40
Cladopus_004796	root	shoot	217.3729183	25.17181643	121.2723674	3.090059616	5.85E-41	6.20E-40
Cladopus_015118	root	shoot	50.29245707	271.7829928	161.0377249	-2.437338917	6.83E-41	7.23E-40
Cladopus_016899	root	shoot	278.6273095	717.9419033	498.2846064	-1.367505192	7.04E-41	7.45E-40
Cladopus_018544	root	shoot	2061.236223	4890.299122	3475.767673	-1.247509256	7.51E-41	7.94E-40
Cladopus_002737	root	shoot	11.44962573	209.6707042	110.560165	-4.236819859	7.67E-41	8.10E-40
Cladopus_014929	root	shoot	171.7601242	505.8781178	338.819121	-1.560307834	9.47E-41	9.98E-40
Cladopus_021451	root	shoot	48.50426227	262.7503747	155.6273185	-2.437878085	9.60E-41	1.01E-39
Cladopus_017760	root	shoot	1941.935193	865.6262157	1403.780705	1.164949937	9.70E-41	1.02E-39
Cladopus_017685	root	shoot	1554.330338	707.6636886	1130.997013	1.135681247	9.75E-41	1.03E-39
Cladopus_024048	root	shoot	823.5722059	296.7447006	560.1584532	1.470935763	1.04E-40	1.10E-39
Cladopus_020188	root	shoot	654.2688449	1346.040562	1000.154703	-1.039406377	1.10E-40	1.15E-39
Cladopus_000384	root	shoot	256.4173125	666.296498	461.3569052	-1.376163171	1.22E-40	1.28E-39
Cladopus_016927	root	shoot	739.7978909	268.1259404	503.9619156	1.46698571	1.68E-40	1.77E-39
Cladopus_011632	root	shoot	230.2867228	3.094894496	116.6908086	6.195145841	1.73E-40	1.81E-39
Cladopus_003820	root	shoot	162.2373628	9.807985605	86.0226742	4.059952707	1.91E-40	2.00E-39
Cladopus_005090	root	shoot	315.0443228	761.9565524	538.5004376	-1.27178491	2.19E-40	2.30E-39
Cladopus_001226	root	shoot	20.69777684	225.7184323	123.2081046	-3.470714011	2.31E-40	2.42E-39
Cladopus_016993	root	shoot	930.6801795	1887.669854	1409.175017	-1.021477549	2.41E-40	2.52E-39
Cladopus_010639	root	shoot	193.4554422	565.2369737	379.3462079	-1.54767697	2.43E-40	2.54E-39
Cladopus_002443	root	shoot	458.0505549	1164.553897	811.3022258	-1.348339507	2.47E-40	2.59E-39
Cladopus_004549	root	shoot	455.9441716	1025.373944	740.6590579	-1.171110578	2.50E-40	2.61E-39
Cladopus_018186	root	shoot	192.9413842	4.244147594	98.59276589	5.585976053	2.71E-40	2.82E-39
Cladopus_008387	root	shoot	2311.125745	5132.138419	3721.632082	-1.151820844	2.81E-40	2.94E-39
Cladopus_016835	root	shoot	688.6218044	273.8645847	481.2431946	1.330709686	2.99E-40	3.12E-39
Cladopus_012894	root	shoot	176.6666085	9.7430074	93.20480796	4.181318429	3.35E-40	3.49E-39

Cladopus_022379	root	shoot	348.7841833	85.29694088	217.0405621	2.030437466	3.58E-40	3.72E-39
Cladopus_015574	root	shoot	116.9531621	415.8340008	266.3935815	-1.83174856	4.79E-40	4.97E-39
Cladopus_013490	root	shoot	496.3423128	1083.787137	790.0647248	-1.127271695	5.16E-40	5.35E-39
Cladopus_022622	root	shoot	156.7932903	501.0731447	328.9332175	-1.671614817	5.68E-40	5.88E-39
Cladopus_014549	root	shoot	523.658382	1122.363091	823.0107367	-1.099876375	6.07E-40	6.28E-39
Cladopus_006007	root	shoot	813.8337542	358.8572818	586.345518	1.182350714	6.53E-40	6.75E-39
Cladopus_017939	root	shoot	309.817913	59.18331506	184.500614	2.406087147	6.73E-40	6.96E-39
Cladopus_021919	root	shoot	882.6041792	1785.381813	1333.992996	-1.01833128	8.48E-40	8.74E-39
Cladopus_023684	root	shoot	372.8240981	916.1613067	644.4927024	-1.300074394	8.80E-40	9.07E-39
Cladopus_018607	root	shoot	212.2389852	597.4659378	404.8524615	-1.496155882	9.19E-40	9.46E-39
Cladopus_021916	root	shoot	828.6983301	332.7927939	580.745562	1.316588208	9.63E-40	9.92E-39
Cladopus_012553	root	shoot	144.163998	469.6775714	306.9207847	-1.700550151	9.78E-40	1.01E-38
Cladopus_013710	root	shoot	1361.537135	663.1463905	1012.341763	1.037910735	1.11E-39	1.14E-38
Cladopus_002444	root	shoot	455.3128563	1158.807112	807.0599844	-1.349854146	1.21E-39	1.24E-38
Cladopus_007998	root	shoot	383.2505515	112.759937	248.0052442	1.759982385	1.21E-39	1.25E-38
Cladopus_016083	root	shoot	423.3811375	927.7125311	675.5468343	-1.132067623	1.31E-39	1.34E-38
Cladopus_002839	root	shoot	782.7084039	308.0648766	545.3866402	1.348204129	1.42E-39	1.45E-38
Cladopus_007426	root	shoot	3203.008884	1535.45048	2369.229682	1.061219124	1.50E-39	1.53E-38
Cladopus_015509	root	shoot	1750.604657	3516.163191	2633.383924	-1.007418615	2.03E-39	2.08E-38
Cladopus_003313	root	shoot	292.2295071	716.0869226	504.1582149	-1.295391877	2.21E-39	2.26E-38
Cladopus_019931	root	shoot	206.5366423	3.474253316	105.0054478	5.803234748	2.46E-39	2.51E-38
Cladopus_007644	root	shoot	761.5650182	1669.83641	1215.700714	-1.134696977	2.54E-39	2.59E-38
Cladopus_018835	root	shoot	2875.673022	1422.635037	2149.15403	1.016134652	2.56E-39	2.60E-38
Cladopus_021335	root	shoot	253.2239941	40.27773313	146.7508636	2.643566318	2.69E-39	2.74E-38
Cladopus_026978	root	shoot	293.7543577	717.8747811	505.8145694	-1.291430878	2.71E-39	2.76E-38
Cladopus_018892	root	shoot	253.1386865	48.08560954	150.612148	2.404693506	2.98E-39	3.03E-38
Cladopus_023116	root	shoot	224.1368092	39.84205512	131.9894322	2.502075246	3.10E-39	3.15E-38
Cladopus_006550	root	shoot	2342.410686	5215.089326	3778.750006	-1.155568759	3.58E-39	3.63E-38
Cladopus_025804	root	shoot	412.7705692	986.4422483	699.6064087	-1.259385787	4.01E-39	4.06E-38
Cladopus_021302	root	shoot	261.0598314	661.1481307	461.1039811	-1.343601084	5.11E-39	5.17E-38

Cladopus_013513	root	shoot	965.4569483	1932.391331	1448.92414	-1.002190687	5.28E-39	5.34E-38
Cladopus_010271	root	shoot	360.7316467	835.5804506	598.1560487	-1.214366	5.53E-39	5.60E-38
Cladopus_021203	root	shoot	941.9548846	403.6378902	672.7963874	1.224056122	6.82E-39	6.89E-38
Cladopus_011469	root	shoot	62.11388793	292.8592685	177.4865782	-2.231609838	7.05E-39	7.12E-38
Cladopus_009227	root	shoot	1064.300959	2317.105719	1690.703339	-1.1242726	8.36E-39	8.42E-38
Cladopus_009689	root	shoot	340.8424745	784.0035019	562.4229882	-1.201445324	8.45E-39	8.52E-38
Cladopus_002695	root	shoot	267.881055	678.6045848	473.2428199	-1.339170048	8.94E-39	9.00E-38
Cladopus_023276	root	shoot	882.4611937	369.771518	626.1163559	1.249896968	9.03E-39	9.08E-38
Cladopus_013468	root	shoot	136.1790839	444.2325235	290.2058037	-1.70242217	9.59E-39	9.63E-38
Cladopus_004660	root	shoot	853.2939061	390.4391307	621.8665184	1.127513128	1.06E-38	1.07E-37
Cladopus_011645	root	shoot	939.5167749	2253.842932	1596.679853	-1.264172112	1.09E-38	1.10E-37
Cladopus_015119	root	shoot	192.591745	541.3778928	366.9848189	-1.488298634	1.17E-38	1.17E-37
Cladopus_022090	root	shoot	9062.204232	4172.710031	6617.457131	1.119172253	1.23E-38	1.24E-37
Cladopus_023489	root	shoot	1180.863524	587.3785793	884.1210514	1.006992001	1.24E-38	1.24E-37
Cladopus_012897	root	shoot	1333.22007	626.0354167	979.6277435	1.091487959	1.54E-38	1.54E-37
Cladopus_020740	root	shoot	305.218351	68.12008802	186.6692195	2.154773801	1.59E-38	1.59E-37
Cladopus_007217	root	shoot	831.1331886	352.0977157	591.6154521	1.241857228	1.64E-38	1.64E-37
Cladopus_000835	root	shoot	1017.417408	2101.723192	1559.5703	-1.044684111	2.32E-38	2.31E-37
Cladopus_003941	root	shoot	1223.019625	2728.102128	1975.560877	-1.159047311	2.49E-38	2.47E-37
Cladopus_009359	root	shoot	62.45201678	315.2698858	188.8609513	-2.325957502	2.73E-38	2.72E-37
Cladopus_010265	root	shoot	238.2916953	633.9517078	436.1217015	-1.4106394	2.92E-38	2.90E-37
Cladopus_017618	root	shoot	1018.285924	483.5927972	750.9393608	1.077333713	3.02E-38	2.99E-37
Cladopus_024635	root	shoot	938.5598667	450.0807696	694.3203182	1.061502336	3.20E-38	3.17E-37
Cladopus_009995	root	shoot	1218.982936	571.2233973	895.1031665	1.096175971	3.34E-38	3.30E-37
Cladopus_007594	root	shoot	494.9222006	1111.80282	803.3625104	-1.169962503	3.34E-38	3.31E-37
Cladopus_005211	root	shoot	183.8705265	519.5213616	351.6959441	-1.499712573	3.43E-38	3.39E-37
Cladopus_020182	root	shoot	1410.147008	701.7956731	1055.97134	1.006422742	3.49E-38	3.44E-37
Cladopus_004108	root	shoot	1209.025682	2862.6474	2035.836541	-1.245102197	4.61E-38	4.55E-37
Cladopus_008280	root	shoot	1267.774559	579.8569663	923.8157628	1.130653608	4.87E-38	4.80E-37
Cladopus_005354	root	shoot	339.5195572	781.5181992	560.5188782	-1.204350235	5.11E-38	5.04E-37

Cladopus_001582	root	shoot	277.5652624	709.6828901	493.6240763	-1.352899268	6.91E-38	6.79E-37
Cladopus_008390	root	shoot	697.5287423	286.7542914	492.1415169	1.286297488	1.17E-37	1.14E-36
Cladopus_006467	root	shoot	13.64949182	193.3362787	103.4928853	-3.846896606	1.18E-37	1.16E-36
Cladopus_025226	root	shoot	416.6104356	908.7287166	662.6695761	-1.126238981	1.34E-37	1.31E-36
Cladopus_006640	root	shoot	229.0639665	24.90590951	126.984938	3.180875754	1.45E-37	1.42E-36
Cladopus_012287	root	shoot	213.3629422	35.22587526	124.2944087	2.616840084	1.65E-37	1.61E-36
Cladopus_009122	root	shoot	442.1683965	143.8661478	293.0172722	1.628952972	1.73E-37	1.68E-36
Cladopus_003996	root	shoot	266.7777776	60.84269495	163.8102363	2.13758183	1.78E-37	1.73E-36
Cladopus_006368	root	shoot	70.58289974	1003.966717	537.2748084	-3.833266723	1.84E-37	1.79E-36
Cladopus_022943	root	shoot	621.4569432	1244.087773	932.7723582	-1.003396237	1.97E-37	1.91E-36
Cladopus_005075	root	shoot	1370.265506	2891.750071	2131.007788	-1.078761864	1.97E-37	1.92E-36
Cladopus_017421	root	shoot	249.0472451	48.07509913	148.5611721	2.371365752	1.98E-37	1.93E-36
Cladopus_002397	root	shoot	631.4321075	1288.96866	960.2003837	-1.031706473	2.04E-37	1.98E-36
Cladopus_021868	root	shoot	437.5578167	946.7442857	692.1510512	-1.113319022	2.05E-37	2.00E-36
Cladopus_008295	root	shoot	1356.352578	652.8419723	1004.597275	1.057441176	2.23E-37	2.16E-36
Cladopus_015109	root	shoot	209.6359959	33.9088478	121.7724218	2.611907696	2.54E-37	2.46E-36
Cladopus_023132	root	shoot	1445.76038	683.8083787	1064.784379	1.080951341	2.80E-37	2.71E-36
Cladopus_003582	root	shoot	275.2190547	667.7824021	471.5007284	-1.280656121	3.52E-37	3.40E-36
Cladopus_020923	root	shoot	57.65571757	277.8806414	167.7681795	-2.275494374	4.36E-37	4.21E-36
Cladopus_018815	root	shoot	31.25609279	219.9535202	125.6048065	-2.810071375	4.59E-37	4.43E-36
Cladopus_009609	root	shoot	72.7526794	302.4934837	187.6230816	-2.058434486	4.76E-37	4.59E-36
Cladopus_025419	root	shoot	473.3291578	1007.695101	740.5121293	-1.091116205	4.77E-37	4.59E-36
Cladopus_000037	root	shoot	705.8095398	1764.813215	1235.311377	-1.324416147	4.77E-37	4.59E-36
Cladopus_004541	root	shoot	150.3275294	457.570787	303.9491582	-1.609072285	5.19E-37	4.99E-36
Cladopus_005412	root	shoot	169.2602648	17.38843732	93.32435107	3.24663524	5.32E-37	5.11E-36
Cladopus_021357	root	shoot	475.4367833	175.585425	325.5111041	1.436634883	5.63E-37	5.41E-36
Cladopus_008669	root	shoot	1221.891862	586.0469006	903.9693815	1.061072638	5.72E-37	5.50E-36
Cladopus_020368	root	shoot	193.0743563	543.7425643	368.4084603	-1.494166886	6.29E-37	6.03E-36
Cladopus_020753	root	shoot	1572.533475	576.9441277	1074.738801	1.449480065	6.68E-37	6.41E-36
Cladopus_022057	root	shoot	992.9285195	2280.969404	1636.948962	-1.2017502	7.20E-37	6.90E-36

Cladopus_011564	root	shoot	336.286122	765.5236085	550.9048652	-1.188651239	7.25E-37	6.94E-36
Cladopus_007960	root	shoot	1174.139735	568.9394633	871.5395991	1.046694779	9.19E-37	8.79E-36
Cladopus_019032	root	shoot	364.2499645	913.0736637	638.6618141	-1.328727237	9.55E-37	9.12E-36
Cladopus_015462	root	shoot	804.6473845	371.6866719	588.1670282	1.115211713	1.20E-36	1.14E-35
Cladopus_009829	root	shoot	59.71158964	793.9277497	426.8196696	-3.733711693	1.40E-36	1.33E-35
Cladopus_014392	root	shoot	73.06331776	313.9830572	193.5231875	-2.107566486	2.59E-36	2.45E-35
Cladopus_006004	root	shoot	398.1396385	126.9093911	262.5245148	1.649072404	2.70E-36	2.55E-35
Cladopus_009757	root	shoot	70.33887076	313.1226456	191.7307582	-2.153194385	2.82E-36	2.67E-35
Cladopus_011739	root	shoot	167.1577156	3.646023092	85.40186936	5.397751408	2.85E-36	2.69E-35
Cladopus_011719	root	shoot	378.4255301	113.3508822	245.8882062	1.743208127	3.20E-36	3.02E-35
Cladopus_006414	root	shoot	3510.142588	1697.796227	2603.969408	1.048379031	3.28E-36	3.09E-35
Cladopus_015097	root	shoot	733.9807857	321.1000024	527.5403941	1.191806459	3.38E-36	3.18E-35
Cladopus_019802	root	shoot	155.5259644	15.93346262	85.72971352	3.301485743	3.68E-36	3.46E-35
Cladopus_015248	root	shoot	1058.012644	527.4996753	792.7561597	1.00574973	3.78E-36	3.55E-35
Cladopus_018573	root	shoot	1389.234878	3321.467643	2355.35126	-1.258737656	3.97E-36	3.73E-35
Cladopus_002748	root	shoot	433.5327404	141.6657742	287.5992573	1.616187813	4.90E-36	4.60E-35
Cladopus_024368	root	shoot	428.2128477	131.1395519	279.6761998	1.706566112	5.26E-36	4.93E-35
Cladopus_014232	root	shoot	1073.609191	483.4794907	778.5443409	1.153254377	5.58E-36	5.23E-35
Cladopus_023945	root	shoot	154.0589309	488.3433025	321.2011167	-1.663427572	5.66E-36	5.30E-35
Cladopus_012474	root	shoot	1259.860225	552.3568185	906.1085219	1.191599702	5.82E-36	5.45E-35
Cladopus_002593	root	shoot	526.5889773	1078.006692	802.2978345	-1.034819687	5.88E-36	5.51E-35
Cladopus_006099	root	shoot	517.2074877	178.8856173	348.0465525	1.530472471	5.93E-36	5.54E-35
Cladopus_022453	root	shoot	3461.604898	1673.401193	2567.503046	1.049416516	7.95E-36	7.44E-35
Cladopus_025609	root	shoot	1083.648236	534.9991633	809.3236997	1.018610352	8.50E-36	7.95E-35
Cladopus_006905	root	shoot	102.5598158	356.8129869	229.6864013	-1.803312025	9.14E-36	8.53E-35
Cladopus_010124	root	shoot	176.7851039	19.24260649	98.01385522	3.206481244	1.02E-35	9.54E-35
Cladopus_018480	root	shoot	315.6319338	799.9676013	557.7997675	-1.338317796	1.05E-35	9.76E-35
Cladopus_023033	root	shoot	391.5422763	113.3327526	252.4375145	1.792747273	1.06E-35	9.87E-35
Cladopus_019269	root	shoot	994.442252	455.7847009	725.1134764	1.127372089	1.20E-35	1.11E-34
Cladopus_001487	root	shoot	533.0074375	194.2187304	363.6130839	1.45100134	1.20E-35	1.11E-34

Cladopus_004483	root	shoot	407.644445	888.6489483	648.1466966	-1.125169466	1.40E-35	1.30E-34
Cladopus_006364	root	shoot	410.6141737	927.7009809	669.1575773	-1.175284977	1.61E-35	1.49E-34
Cladopus_015571	root	shoot	312.5696022	752.8701308	532.7198665	-1.265583133	2.02E-35	1.87E-34
Cladopus_014366	root	shoot	240.9028599	597.5114559	419.2071579	-1.312404929	2.17E-35	2.01E-34
Cladopus_020045	root	shoot	221.6368277	41.30235769	131.4695927	2.405109681	2.30E-35	2.13E-34
Cladopus_003210	root	shoot	310.2756362	84.02091523	197.1482757	1.879545767	2.54E-35	2.35E-34
Cladopus_021427	root	shoot	56.28748737	273.8742026	165.080845	-2.276975477	2.79E-35	2.58E-34
Cladopus_012907	root	shoot	923.0589058	407.8824737	665.4706898	1.173976161	2.84E-35	2.62E-34
Cladopus_023680	root	shoot	1224.713003	579.4522118	902.0826073	1.075205727	3.09E-35	2.84E-34
Cladopus_013191	root	shoot	254.7904415	2.089584695	128.4400131	6.992278367	4.76E-35	4.37E-34
Cladopus_013857	root	shoot	38.13116673	227.9119983	133.0215825	-2.586897648	4.86E-35	4.45E-34
Cladopus_012173	root	shoot	263.5504101	667.6181871	465.5842986	-1.340910262	5.29E-35	4.84E-34
Cladopus_005948	root	shoot	762.4936152	327.2734362	544.8835257	1.225432611	5.57E-35	5.10E-34
Cladopus_024483	root	shoot	884.5334017	424.0342661	654.2838339	1.062725164	6.10E-35	5.58E-34
Cladopus_016192	root	shoot	615.3728894	1257.076218	936.2245538	-1.031729601	6.13E-35	5.61E-34
Cladopus_005581	root	shoot	123.3324994	394.4533379	258.8929186	-1.681002517	7.16E-35	6.53E-34
Cladopus_018610	root	shoot	169.6359081	24.05438719	96.84514765	2.831855607	8.06E-35	7.34E-34
Cladopus_026368	root	shoot	260.8915745	58.26229822	159.5769364	2.181576483	8.86E-35	8.06E-34
Cladopus_010343	root	shoot	7.161449399	174.0030115	90.58223047	-4.584009531	1.16E-34	1.05E-33
Cladopus_024386	root	shoot	99.24825678	341.9034826	220.5758697	-1.787910233	1.25E-34	1.13E-33
Cladopus_010114	root	shoot	1019.879991	495.6607239	757.7703574	1.04293527	1.58E-34	1.43E-33
Cladopus_006066	root	shoot	181.1913883	3.101554825	92.14647154	5.850284148	1.60E-34	1.45E-33
Cladopus_019299	root	shoot	1549.725775	3239.411012	2394.568393	-1.065007582	1.85E-34	1.67E-33
Cladopus_011699	root	shoot	921.4296872	455.843685	688.6366861	1.016317605	1.87E-34	1.68E-33
Cladopus_019326	root	shoot	578.0472429	230.5525663	404.2999046	1.330746573	2.28E-34	2.05E-33
Cladopus_009108	root	shoot	175.9438064	480.9389086	328.4413575	-1.448682616	2.41E-34	2.17E-33
Cladopus_023377	root	shoot	624.5517195	251.0087378	437.7802287	1.319761894	2.41E-34	2.17E-33
Cladopus_020272	root	shoot	850.0407958	349.5717867	599.8062913	1.285369986	2.56E-34	2.30E-33
Cladopus_020359	root	shoot	1001.63874	464.9053791	733.2720593	1.106846411	2.63E-34	2.36E-33
Cladopus_023644	root	shoot	155.3990601	17.49656135	86.44781071	3.139899881	2.68E-34	2.41E-33

Cladopus_010420	root	shoot	974.9263584	487.2081007	731.0672295	1.000459781	3.30E-34	2.96E-33
Cladopus_024975	root	shoot	312.9017789	710.0854909	511.4936349	-1.183711503	3.35E-34	3.01E-33
Cladopus_010186	root	shoot	439.0410705	917.0038003	678.0224354	-1.062281104	3.94E-34	3.53E-33
Cladopus_009329	root	shoot	662.9515485	1400.378934	1031.665241	-1.081023948	4.13E-34	3.70E-33
Cladopus_015199	root	shoot	126.0294664	382.6034278	254.3164471	-1.602599894	4.45E-34	3.98E-33
Cladopus_003197	root	shoot	294.5847555	700.03187	497.3083127	-1.251497402	4.73E-34	4.22E-33
Cladopus_004176	root	shoot	426.557126	138.6333403	282.5952331	1.62288436	5.05E-34	4.51E-33
Cladopus_002409	root	shoot	285.4496426	686.9804659	486.2150542	-1.266865045	5.23E-34	4.67E-33
Cladopus_012547	root	shoot	839.0886203	401.1774584	620.1330393	1.064825738	5.35E-34	4.77E-33
Cladopus_011493	root	shoot	6.187183786	176.0416049	91.11439435	-4.844589339	5.54E-34	4.94E-33
Cladopus_006329	root	shoot	581.6966695	1265.267071	923.4818704	-1.124047804	6.49E-34	5.78E-33
Cladopus_009765	root	shoot	923.9352545	403.9063127	663.9207836	1.192573417	7.71E-34	6.86E-33
Cladopus_002851	root	shoot	315.249351	707.1485246	511.1989378	-1.167150243	8.18E-34	7.26E-33
Cladopus_010174	root	shoot	623.0627643	265.4595426	444.2611535	1.228067675	8.53E-34	7.57E-33
Cladopus_017225	root	shoot	1051.555054	516.5598961	784.0574752	1.021580234	1.01E-33	8.96E-33
Cladopus_000877	root	shoot	204.189942	516.5489497	360.3694458	-1.33827528	1.06E-33	9.39E-33
Cladopus_021848	root	shoot	494.1825571	1046.002923	770.0927403	-1.083811184	1.13E-33	1.00E-32
Cladopus_019480	root	shoot	354.0263056	967.8975219	660.9619138	-1.455490141	1.25E-33	1.10E-32
Cladopus_014259	root	shoot	705.2615783	303.5712436	504.416411	1.21320132	1.26E-33	1.11E-32
Cladopus_010146	root	shoot	511.643067	1068.682858	790.1629624	-1.062437644	1.28E-33	1.13E-32
Cladopus_016194	root	shoot	39.46832307	224.9770307	132.2226769	-2.505377566	1.33E-33	1.18E-32
Cladopus_011496	root	shoot	1158.425063	2481.277578	1819.85132	-1.100552215	1.40E-33	1.24E-32
Cladopus_000946	root	shoot	754.645741	1601.481714	1178.063727	-1.087755134	1.45E-33	1.28E-32
Cladopus_001353	root	shoot	787.9049862	350.5006491	569.2028176	1.171109236	1.58E-33	1.40E-32
Cladopus_002522	root	shoot	403.8712025	120.0158712	261.9435369	1.746603641	1.62E-33	1.42E-32
Cladopus_008254	root	shoot	1431.601535	698.5437409	1065.072638	1.035264921	1.65E-33	1.46E-32
Cladopus_000297	root	shoot	719.7319389	289.7372099	504.7345744	1.310595202	1.70E-33	1.49E-32
Cladopus_018395	root	shoot	361.2622954	844.6260138	602.9441546	-1.229693159	1.72E-33	1.52E-32
Cladopus_011994	root	shoot	531.2261822	220.0243452	375.6252637	1.272849583	1.73E-33	1.52E-32
Cladopus_004830	root	shoot	34.16848881	210.7893226	122.4789057	-2.614912371	1.90E-33	1.67E-32

Cladopus_016670	root	shoot	498.6699485	1022.361797	760.5158726	-1.037448475	2.05E-33	1.80E-32
Cladopus_011409	root	shoot	1061.111346	2308.861581	1684.986464	-1.12331254	2.09E-33	1.84E-32
Cladopus_014984	root	shoot	982.8484971	488.8350734	735.8417853	1.008595373	2.10E-33	1.84E-32
Cladopus_007779	root	shoot	300.295762	709.0489405	504.6723513	-1.243459611	2.12E-33	1.86E-32
Cladopus_019580	root	shoot	106.1630748	365.2256462	235.6943605	-1.788867471	2.17E-33	1.91E-32
Cladopus_008757	root	shoot	9.661430924	164.6991606	87.18029576	-4.101589456	2.32E-33	2.04E-32
Cladopus_011249	root	shoot	540.7558727	1098.305518	819.5306955	-1.023295285	2.40E-33	2.10E-32
Cladopus_021351	root	shoot	1143.289881	547.1223879	845.2061346	1.065750471	2.42E-33	2.12E-32
Cladopus_006429	root	shoot	99.52138071	346.2614557	222.8914182	-1.803632194	2.65E-33	2.31E-32
Cladopus_003084	root	shoot	356.2654273	102.8121508	229.538789	1.784190586	2.67E-33	2.33E-32
Cladopus_010258	root	shoot	530.196207	1115.389565	822.7928858	-1.073250851	2.86E-33	2.50E-32
Cladopus_008632	root	shoot	6.448919472	168.5627622	87.50584085	-4.697886605	2.99E-33	2.61E-32
Cladopus_017183	root	shoot	753.1602787	350.124912	551.6425954	1.104194967	3.03E-33	2.64E-32
Cladopus_025559	root	shoot	336.2266923	759.4047918	547.815742	-1.175732332	3.14E-33	2.74E-32
Cladopus_014112	root	shoot	214.1044384	568.8806225	391.4925305	-1.413834379	3.26E-33	2.84E-32
Cladopus_002255	root	shoot	249.2168663	698.4223772	473.8196217	-1.486108616	3.42E-33	2.97E-32
Cladopus_000028	root	shoot	607.9072446	231.162679	419.5349618	1.399800649	3.50E-33	3.04E-32
Cladopus_026699	root	shoot	15.62352814	200.8964421	108.2599851	-3.681915389	3.71E-33	3.23E-32
Cladopus_014086	root	shoot	50.25729437	248.9799867	149.6186405	-2.32046871	3.89E-33	3.38E-32
Cladopus_008644	root	shoot	49.18076418	240.1516284	144.6661963	-2.287532876	4.24E-33	3.68E-32
Cladopus_019493	root	shoot	1059.032366	428.612626	743.8224958	1.304382224	4.28E-33	3.72E-32
Cladopus_015000	root	shoot	492.1519459	169.2727116	330.7123287	1.531755257	4.37E-33	3.79E-32
Cladopus_010086	root	shoot	116.0297845	362.9411289	239.4854567	-1.64328617	4.88E-33	4.23E-32
Cladopus_003876	root	shoot	598.348832	223.5722358	410.9605339	1.424327256	5.58E-33	4.83E-32
Cladopus_019919	root	shoot	240.6211943	611.9096502	426.2654223	-1.341814918	5.92E-33	5.11E-32
Cladopus_005251	root	shoot	138.7089036	5.226958786	71.96793118	4.752510832	5.97E-33	5.16E-32
Cladopus_019774	root	shoot	134.9200513	5.664635482	70.29234342	4.561483598	7.06E-33	6.09E-32
Cladopus_011016	root	shoot	462.7023757	173.2174243	317.9599	1.411904756	7.17E-33	6.18E-32
Cladopus_021209	root	shoot	79.42111668	318.1022006	198.7616587	-1.995578045	7.94E-33	6.83E-32
Cladopus_008512	root	shoot	473.2569691	123.5126895	298.3848293	1.932888901	9.77E-33	8.40E-32

Cladopus_016191	root	shoot	42.23078349	225.4856797	133.8582316	-2.414005557	1.09E-32	9.38E-32
Cladopus_002553	root	shoot	145.055552	13.64228298	79.34891748	3.401980173	1.13E-32	9.68E-32
Cladopus_015321	root	shoot	1179.249636	2920.643413	2049.946525	-1.309468	1.13E-32	9.71E-32
Cladopus_006093	root	shoot	167.96496	22.50460912	95.23478458	2.931694751	1.16E-32	9.96E-32
Cladopus_022137	root	shoot	640.0139092	1298.515029	969.2644689	-1.021439032	1.17E-32	1.00E-31
Cladopus_023887	root	shoot	382.8905114	825.807618	604.3490647	-1.112026022	1.19E-32	1.02E-31
Cladopus_021587	root	shoot	14.68442523	161.3488676	88.01664642	-3.440756045	1.29E-32	1.10E-31
Cladopus_014149	root	shoot	217.8934168	46.96618161	132.4297992	2.220590503	1.29E-32	1.11E-31
Cladopus_009396	root	shoot	338.0796478	757.5617992	547.8207235	-1.161997573	1.52E-32	1.30E-31
Cladopus_023326	root	shoot	446.2397901	153.929009	300.0843996	1.535727669	1.90E-32	1.62E-31
Cladopus_023934	root	shoot	1119.920512	559.4182581	839.6693852	1.003628741	2.22E-32	1.89E-31
Cladopus_004071	root	shoot	230.2061251	577.3110544	403.7585898	-1.32499856	3.18E-32	2.70E-31
Cladopus_012578	root	shoot	177.7464844	489.7244137	333.735449	-1.464662645	3.19E-32	2.71E-31
Cladopus_013035	root	shoot	3609.565911	7356.296444	5482.931177	-1.027752968	3.25E-32	2.76E-31
Cladopus_004968	root	shoot	368.3906961	821.2772919	594.833994	-1.155695215	3.48E-32	2.95E-31
Cladopus_002216	root	shoot	275.5653547	637.2292561	456.3973054	-1.207687616	3.50E-32	2.96E-31
Cladopus_007229	root	shoot	184.7055056	25.65330518	105.1794054	2.84414282	3.67E-32	3.10E-31
Cladopus_020937	root	shoot	1098.541883	542.0720078	820.3069456	1.020604722	3.87E-32	3.27E-31
Cladopus_023462	root	shoot	207.1542135	510.7590194	358.9566165	-1.302920619	3.97E-32	3.36E-31
Cladopus_001776	root	shoot	787.639515	1776.372733	1282.006124	-1.175815823	4.20E-32	3.55E-31
Cladopus_006669	root	shoot	235.3027881	55.4604709	145.3816295	2.093729993	4.23E-32	3.57E-31
Cladopus_015454	root	shoot	160.32263	21.05429607	90.68846301	2.963293217	4.36E-32	3.68E-31
Cladopus_001511	root	shoot	2511.408688	1192.566336	1851.987512	1.076205178	4.83E-32	4.07E-31
Cladopus_024839	root	shoot	1995.234016	969.4720256	1482.353021	1.041669053	4.91E-32	4.14E-31
Cladopus_001301	root	shoot	10.04920565	155.2165849	82.6328953	-3.960353051	5.97E-32	5.02E-31
Cladopus_001444	root	shoot	38.53392362	212.3914225	125.462673	-2.47134163	6.69E-32	5.62E-31
Cladopus_018980	root	shoot	3022.703007	1380.977003	2201.840005	1.130649652	6.99E-32	5.87E-31
Cladopus_020166	root	shoot	620.4982874	255.3253355	437.9118114	1.283798632	7.95E-32	6.67E-31
Cladopus_001081	root	shoot	1032.33707	484.8233857	758.5802279	1.09329255	9.04E-32	7.58E-31
Cladopus_023001	root	shoot	608.048392	237.2036328	422.6260124	1.352066264	9.15E-32	7.67E-31

Cladopus_001044	root	shoot	388.4429228	836.603535	612.5232289	-1.106848715	9.77E-32	8.18E-31
Cladopus_008425	root	shoot	481.5483212	171.0035866	326.2759539	1.497160043	9.83E-32	8.23E-31
Cladopus_020286	root	shoot	393.6180823	129.2153692	261.4167258	1.603765637	1.12E-31	9.35E-31
Cladopus_021327	root	shoot	18.48429288	164.4750651	91.47967898	-3.163889797	1.17E-31	9.75E-31
Cladopus_016018	root	shoot	480.5325833	160.5094808	320.521032	1.588475364	1.32E-31	1.10E-30
Cladopus_015703	root	shoot	715.2028115	324.214357	519.7085842	1.136898435	1.50E-31	1.25E-30
Cladopus_008369	root	shoot	61.50102664	256.0619262	158.7814764	-2.053930428	1.89E-31	1.57E-30
Cladopus_018695	root	shoot	674.3982103	282.7640626	478.5811364	1.258906309	1.98E-31	1.64E-30
Cladopus_000391	root	shoot	349.4698439	109.2233703	229.3466071	1.683707525	2.16E-31	1.79E-30
Cladopus_006541	root	shoot	173.4236443	28.53594899	100.9797967	2.62960706	2.75E-31	2.27E-30
Cladopus_009814	root	shoot	689.3458512	326.646817	507.9963341	1.077876993	3.32E-31	2.73E-30
Cladopus_006521	root	shoot	532.5349933	1186.919455	859.7272239	-1.153444108	3.69E-31	3.04E-30
Cladopus_000066	root	shoot	135.9001364	3.575050788	69.73759361	5.199309483	4.01E-31	3.29E-30
Cladopus_009268	root	shoot	911.9215286	1833.048624	1372.485077	-1.008675261	4.01E-31	3.30E-30
Cladopus_004715	root	shoot	431.8083042	162.0319513	296.9201278	1.417613292	4.30E-31	3.53E-30
Cladopus_019170	root	shoot	45.45679069	226.5157151	135.9862529	-2.31419444	4.79E-31	3.93E-30
Cladopus_000850	root	shoot	1232.39682	612.4043103	922.4005654	1.010957087	4.90E-31	4.02E-30
Cladopus_022071	root	shoot	395.1042984	816.1138355	605.6090669	-1.047291679	5.83E-31	4.77E-30
Cladopus_021809	root	shoot	372.143259	800.9878568	586.5655579	-1.108309403	5.85E-31	4.79E-30
Cladopus_025455	root	shoot	877.2111234	428.3617458	652.7864346	1.033450921	6.96E-31	5.68E-30
Cladopus_004815	root	shoot	245.3796022	64.00256765	154.6910849	1.943739426	8.33E-31	6.78E-30
Cladopus_019605	root	shoot	547.9487793	1101.545981	824.7473803	-1.005753012	1.07E-30	8.71E-30
Cladopus_009143	root	shoot	1317.1805	2704.200557	2010.690528	-1.039141871	1.15E-30	9.32E-30
Cladopus_014453	root	shoot	160.142612	455.8936365	308.0181242	-1.511181438	1.26E-30	1.02E-29
Cladopus_016386	root	shoot	368.1100139	99.46237869	233.7861963	1.881469501	1.31E-30	1.06E-29
Cladopus_010134	root	shoot	172.5881703	30.93192152	101.7600459	2.481655999	1.33E-30	1.08E-29
Cladopus_024695	root	shoot	348.0130931	103.7378312	225.8754622	1.753635805	1.38E-30	1.12E-29
Cladopus_024684	root	shoot	467.093804	173.0681531	320.0809786	1.425915393	1.42E-30	1.14E-29
Cladopus_016036	root	shoot	494.5214332	180.4440543	337.4827438	1.452742739	1.43E-30	1.16E-29
Cladopus_000707	root	shoot	560.5253219	223.1655694	391.8454457	1.329254468	1.44E-30	1.16E-29

Cladopus_018727	root	shoot	1475.004879	2993.576537	2234.290708	-1.022436652	1.44E-30	1.16E-29
Cladopus_009703	root	shoot	371.8934106	126.5064939	249.1999522	1.549940869	1.50E-30	1.21E-29
Cladopus_001019	root	shoot	896.8234491	416.8904473	656.8569482	1.100116904	1.53E-30	1.24E-29
Cladopus_024968	root	shoot	371.2099756	839.0365159	605.1232457	-1.175930977	1.55E-30	1.25E-29
Cladopus_001264	root	shoot	604.9403747	258.5440431	431.7422089	1.220641304	1.56E-30	1.26E-29
Cladopus_000143	root	shoot	553.4676194	1138.186431	845.8270254	-1.042617459	2.15E-30	1.73E-29
Cladopus_006229	root	shoot	711.9906899	263.9791138	487.9849018	1.435939166	2.50E-30	2.00E-29
Cladopus_022184	root	shoot	246.9802184	635.6187898	441.2995041	-1.367924547	2.65E-30	2.12E-29
Cladopus_014329	root	shoot	378.1764249	802.2220008	590.1992128	-1.086868357	2.71E-30	2.17E-29
Cladopus_005463	root	shoot	54.08910768	235.6036106	144.8463591	-2.121498515	2.81E-30	2.24E-29
Cladopus_013567	root	shoot	470.1882204	138.5842004	304.3862104	1.766331962	2.86E-30	2.29E-29
Cladopus_023155	root	shoot	1872.020629	798.1817414	1335.101185	1.230878195	2.90E-30	2.32E-29
Cladopus_022216	root	shoot	657.3527213	286.8596033	472.1061623	1.197097937	3.50E-30	2.79E-29
Cladopus_007516	root	shoot	637.0032194	292.0808606	464.54204	1.125589954	3.57E-30	2.84E-29
Cladopus_013881	root	shoot	45.21758727	211.3098125	128.2636999	-2.226499465	3.63E-30	2.89E-29
Cladopus_006573	root	shoot	149.5023341	19.06099253	84.28166332	2.968438682	4.18E-30	3.32E-29
Cladopus_009110	root	shoot	138.6889673	12.72578245	75.70737485	3.503501536	4.92E-30	3.90E-29
Cladopus_020115	root	shoot	328.752996	102.9446268	215.8488114	1.687418085	5.70E-30	4.52E-29
Cladopus_010155	root	shoot	219.2038259	534.3772057	376.7905158	-1.289046756	6.02E-30	4.77E-29
Cladopus_019323	root	shoot	7.112047741	160.0792097	83.59562871	-4.533426267	7.01E-30	5.54E-29
Cladopus_022066	root	shoot	646.0493257	222.5242991	434.2868124	1.539083207	7.67E-30	6.06E-29
Cladopus_005258	root	shoot	89.27395788	301.96552	195.6197389	-1.756862828	9.11E-30	7.18E-29
Cladopus_026992	root	shoot	256.4772306	52.98982132	154.7335259	2.265288736	9.18E-30	7.23E-29
Cladopus_001741	root	shoot	279.676726	631.31884	455.497783	-1.176204495	9.43E-30	7.43E-29
Cladopus_005370	root	shoot	276.9891724	81.07277427	179.0309733	1.767251262	9.56E-30	7.53E-29
Cladopus_009242	root	shoot	312.8521289	737.3963245	525.1242267	-1.237001009	1.10E-29	8.67E-29
Cladopus_000577	root	shoot	746.3560015	1749.2466	1247.801301	-1.231185275	1.12E-29	8.78E-29
Cladopus_016848	root	shoot	154.8890845	22.6578757	88.77348008	2.754648108	1.15E-29	9.03E-29
Cladopus_008937	root	shoot	1293.835408	615.6432954	954.7393518	1.071982764	1.21E-29	9.49E-29
Cladopus_017563	root	shoot	397.6920613	137.7498488	267.720955	1.537147512	1.26E-29	9.85E-29

Cladopus_013043	root	shoot	48.07513497	238.9305914	143.5028632	-2.305517582	1.27E-29	9.93E-29
Cladopus_009992	root	shoot	106.402152	359.5695244	232.9858382	-1.754680384	1.33E-29	1.04E-28
Cladopus_012862	root	shoot	899.0469676	440.093397	669.5701823	1.033961149	1.39E-29	1.08E-28
Cladopus_005255	root	shoot	931.6653279	2048.08157	1489.873449	-1.138317323	1.40E-29	1.09E-28
Cladopus_013318	root	shoot	106.358696	323.3583203	214.8585082	-1.604596876	1.50E-29	1.17E-28
Cladopus_020382	root	shoot	698.1190672	333.4123792	515.7657232	1.069449727	1.73E-29	1.35E-28
Cladopus_022594	root	shoot	865.7748859	389.8763065	627.8255962	1.15160208	1.90E-29	1.47E-28
Cladopus_016241	root	shoot	805.3154669	389.9420319	597.6287494	1.048147775	1.96E-29	1.52E-28
Cladopus_020082	root	shoot	26.35975862	174.8280398	100.5938992	-2.727702334	2.10E-29	1.63E-28
Cladopus_024423	root	shoot	293.7097881	89.50208536	191.6059367	1.712220313	2.13E-29	1.65E-28
Cladopus_000518	root	shoot	1072.704019	477.0206109	774.8623151	1.171347654	2.24E-29	1.73E-28
Cladopus_011108	root	shoot	1345.339888	667.6783564	1006.509122	1.011234818	2.45E-29	1.89E-28
Cladopus_011818	root	shoot	1763.070012	3541.576566	2652.323289	-1.007486194	2.65E-29	2.05E-28
Cladopus_027260	root	shoot	123.3581266	8.22172204	65.7899243	3.934690636	3.31E-29	2.55E-28
Cladopus_000763	root	shoot	965.1923635	2003.346627	1484.269495	-1.055405724	3.94E-29	3.03E-28
Cladopus_016054	root	shoot	13.45969006	145.7556154	79.60765272	-3.4423843	4.25E-29	3.27E-28
Cladopus_005570	root	shoot	1404.89462	650.5294793	1027.71205	1.112599146	4.53E-29	3.47E-28
Cladopus_002825	root	shoot	111.2228403	337.2281733	224.2255068	-1.602736052	4.66E-29	3.57E-28
Cladopus_002899	root	shoot	393.545778	828.1176764	610.8317272	-1.071258869	5.06E-29	3.88E-28
Cladopus_010156	root	shoot	478.8165537	1010.218448	744.5175007	-1.079878409	5.41E-29	4.14E-28
Cladopus_011465	root	shoot	119.8056335	3.948415508	61.87702451	4.90673281	5.49E-29	4.19E-28
Cladopus_015641	root	shoot	196.6559443	484.0567397	340.356342	-1.302116238	6.78E-29	5.17E-28
Cladopus_019428	root	shoot	248.0204829	567.3646007	407.6925418	-1.191979552	8.30E-29	6.31E-28
Cladopus_015954	root	shoot	141.6090396	15.1119108	78.36047518	3.206897681	8.43E-29	6.41E-28
Cladopus_006193	root	shoot	51.47979992	220.603155	136.0414775	-2.102694961	9.01E-29	6.84E-28
Cladopus_024467	root	shoot	1071.953967	493.6994705	782.8267187	1.120842177	9.28E-29	7.04E-28
Cladopus_021555	root	shoot	17.89842309	156.6465434	87.27248325	-3.124509713	1.15E-28	8.67E-28
Cladopus_023482	root	shoot	693.336508	330.3186679	511.8275879	1.066494432	1.24E-28	9.38E-28
Cladopus_024087	root	shoot	585.5916414	250.1430813	417.8673614	1.230687746	1.42E-28	1.07E-27
Cladopus_005008	root	shoot	322.1485762	102.5745912	212.3615837	1.654231606	1.44E-28	1.08E-27

Cladopus_017868	root	shoot	196.4053526	479.0888802	337.7471164	-1.289129844	1.54E-28	1.16E-27
Cladopus_014210	root	shoot	293.5094634	644.4278313	468.9686474	-1.132413695	1.58E-28	1.19E-27
Cladopus_016000	root	shoot	345.7336103	704.9469677	525.340289	-1.028379652	1.68E-28	1.26E-27
Cladopus_004802	root	shoot	600.5052291	256.4331489	428.469189	1.230545011	1.70E-28	1.28E-27
Cladopus_007801	root	shoot	666.6017313	317.750004	492.1758677	1.06772269	1.98E-28	1.49E-27
Cladopus_009586	root	shoot	299.4636337	632.3374724	465.900553	-1.07736145	2.47E-28	1.85E-27
Cladopus_019849	root	shoot	468.4754143	192.1818431	330.3286287	1.284415489	2.62E-28	1.96E-27
Cladopus_016328	root	shoot	22.11082735	161.3648532	91.73784028	-2.881130694	2.65E-28	1.99E-27
Cladopus_023547	root	shoot	611.8241188	280.7982119	446.3111654	1.124435601	2.83E-28	2.12E-27
Cladopus_003588	root	shoot	323.4508141	101.6853963	212.5681052	1.661169325	2.94E-28	2.20E-27
Cladopus_020158	root	shoot	234.8737829	531.3479576	383.1108703	-1.178275195	2.94E-28	2.20E-27
Cladopus_021271	root	shoot	470.0317051	190.4292849	330.230495	1.303161625	3.03E-28	2.26E-27
Cladopus_008927	root	shoot	298.3983696	95.46911325	196.9337414	1.643957971	3.05E-28	2.28E-27
Cladopus_024628	root	shoot	426.6220047	172.998368	299.8101864	1.305633639	3.12E-28	2.33E-27
Cladopus_022809	root	shoot	598.7779487	266.7565891	432.7672689	1.167467734	3.19E-28	2.38E-27
Cladopus_000733	root	shoot	207.3528076	50.15003071	128.7514192	2.037730531	3.31E-28	2.47E-27
Cladopus_010115	root	shoot	146.7781313	390.2891933	268.5336623	-1.411902948	3.50E-28	2.61E-27
Cladopus_003668	root	shoot	572.7825673	243.3342943	408.0584308	1.237673633	3.50E-28	2.61E-27
Cladopus_010429	root	shoot	713.3132133	334.7914253	524.0523193	1.090458785	3.99E-28	2.97E-27
Cladopus_023978	root	shoot	241.6702381	62.13641028	151.9033242	1.959609916	4.21E-28	3.13E-27
Cladopus_017024	root	shoot	508.9463377	1163.398502	836.17242	-1.195329604	4.37E-28	3.25E-27
Cladopus_019775	root	shoot	119.2258274	4.717643557	61.97173549	4.697779511	5.09E-28	3.77E-27
Cladopus_000379	root	shoot	2790.754739	5587.189255	4188.971997	-1.002256241	5.22E-28	3.86E-27
Cladopus_019577	root	shoot	627.7974083	301.4061723	464.6017903	1.05899489	5.31E-28	3.93E-27
Cladopus_011711	root	shoot	157.7320204	22.66971857	90.20086948	2.822858095	5.55E-28	4.11E-27
Cladopus_015779	root	shoot	112.4032501	5.972355768	59.18780292	4.17563163	5.85E-28	4.32E-27
Cladopus_009745	root	shoot	323.1302634	690.9901588	507.0602111	-1.097598302	6.27E-28	4.63E-27
Cladopus_011694	root	shoot	116.9698749	352.8287522	234.8993135	-1.598277369	6.74E-28	4.97E-27
Cladopus_019975	root	shoot	126.136563	13.35387745	69.74522025	3.236878535	7.58E-28	5.58E-27
Cladopus_016002	root	shoot	90.06906677	304.6748352	197.371951	-1.756513946	7.79E-28	5.73E-27

Cladopus_013209	root	shoot	1252.674958	2629.559369	1941.117164	-1.071230562	8.93E-28	6.57E-27
Cladopus_022467	root	shoot	227.2681019	59.32074336	143.2944226	1.941826557	1.22E-27	8.92E-27
Cladopus_002785	root	shoot	706.5380604	331.6408779	519.0894691	1.091658462	1.23E-27	9.04E-27
Cladopus_002310	root	shoot	402.7174138	153.8247351	278.2710745	1.389290835	1.41E-27	1.03E-26
Cladopus_007110	root	shoot	290.7122131	73.91786376	182.3150384	1.972331859	1.45E-27	1.06E-26
Cladopus_015260	root	shoot	563.6302507	230.9409538	397.2856022	1.282687404	1.50E-27	1.10E-26
Cladopus_001395	root	shoot	608.2135539	276.0872267	442.1503903	1.134219899	1.70E-27	1.24E-26
Cladopus_003458	root	shoot	140.1453517	18.13649922	79.14092544	2.95863648	1.77E-27	1.29E-26
Cladopus_003993	root	shoot	261.3453416	83.20269455	172.2740181	1.65446573	1.94E-27	1.41E-26
Cladopus_023604	root	shoot	313.5176089	725.1717211	519.344665	-1.209990593	1.95E-27	1.42E-26
Cladopus_020325	root	shoot	652.9784902	300.5359994	476.7572448	1.118188187	1.97E-27	1.43E-26
Cladopus_017512	root	shoot	482.1239147	184.3155054	333.21971	1.396630889	2.01E-27	1.46E-26
Cladopus_004850	root	shoot	377.8530405	128.6537302	253.2533853	1.551984378	2.18E-27	1.58E-26
Cladopus_009510	root	shoot	310.9034797	103.1209109	207.0121953	1.589448764	2.45E-27	1.77E-26
Cladopus_026367	root	shoot	182.4192184	37.56138589	109.9903021	2.293753304	2.48E-27	1.80E-26
Cladopus_015785	root	shoot	584.4811867	244.7538233	414.617505	1.259091447	2.69E-27	1.95E-26
Cladopus_007738	root	shoot	178.2801123	438.4937299	308.3869211	-1.296086248	2.90E-27	2.10E-26
Cladopus_023346	root	shoot	227.3841105	55.22520071	141.3046556	2.034082677	3.08E-27	2.22E-26
Cladopus_000010	root	shoot	320.664453	684.9756161	502.8200346	-1.092897577	3.56E-27	2.57E-26
Cladopus_006433	root	shoot	2797.01856	1368.534964	2082.776762	1.031809197	4.48E-27	3.23E-26
Cladopus_012650	root	shoot	174.3134677	35.23505322	104.7742604	2.320054973	4.62E-27	3.33E-26
Cladopus_020342	root	shoot	640.948079	268.5476315	454.7478553	1.257104695	6.28E-27	4.51E-26
Cladopus_016600	root	shoot	1056.48358	0	528.24179	12.69357731	6.83E-27	4.90E-26
Cladopus_015184	root	shoot	34.68304578	183.2970203	108.990033	-2.396729603	7.10E-27	5.09E-26
Cladopus_018487	root	shoot	166.9492197	1.687061037	84.31814039	6.606969053	8.25E-27	5.90E-26
Cladopus_011217	root	shoot	697.2582208	313.0200797	505.1391502	1.157291286	8.31E-27	5.94E-26
Cladopus_012795	root	shoot	975.1113175	463.7553954	719.4333565	1.071819601	9.83E-27	7.02E-26
Cladopus_022833	root	shoot	67.96653679	260.7578827	164.3622097	-1.942073873	1.00E-26	7.16E-26
Cladopus_000945	root	shoot	291.6574994	626.2764527	458.9669761	-1.105638156	1.12E-26	7.98E-26
Cladopus_011009	root	shoot	152.6574013	424.4057774	288.5315894	-1.469488082	1.24E-26	8.85E-26

Cladopus_001099	root	shoot	185.5219151	449.9891501	317.7555326	-1.276942043	1.25E-26	8.89E-26
Cladopus_023284	root	shoot	306.7799663	653.9618382	480.3709023	-1.093838708	1.45E-26	1.03E-25
Cladopus_023154	root	shoot	1608.566916	734.8697274	1171.718322	1.130387319	1.50E-26	1.06E-25
Cladopus_016958	root	shoot	114.1825305	12.77292369	63.47772708	3.152198534	1.57E-26	1.11E-25
Cladopus_001869	root	shoot	616.0048281	296.1697429	456.0872855	1.058340926	1.60E-26	1.14E-25
Cladopus_004890	root	shoot	398.1442158	153.6094459	275.8768308	1.36909702	1.79E-26	1.27E-25
Cladopus_015010	root	shoot	140.5161629	393.9731777	267.2446703	-1.494066174	1.80E-26	1.28E-25
Cladopus_006597	root	shoot	604.172508	260.5819702	432.3772391	1.209396145	1.80E-26	1.28E-25
Cladopus_004510	root	shoot	989.9959388	0	494.9979694	12.59922924	1.88E-26	1.33E-25
Cladopus_007605	root	shoot	330.5939316	120.680599	225.6372653	1.452334929	2.19E-26	1.55E-25
Cladopus_018269	root	shoot	704.5970857	341.3017344	522.94941	1.042517773	2.28E-26	1.61E-25
Cladopus_018051	root	shoot	139.8161413	19.65578589	79.73596359	2.789183375	2.33E-26	1.65E-25
Cladopus_022800	root	shoot	106.0944865	321.8242352	213.9593608	-1.594573908	2.64E-26	1.85E-25
Cladopus_009215	root	shoot	75.52652806	273.5600483	174.5432882	-1.862388708	2.68E-26	1.88E-25
Cladopus_007541	root	shoot	766.2712013	376.5638045	571.4175029	1.027877549	2.75E-26	1.93E-25
Cladopus_018454	root	shoot	242.5636513	544.3349854	393.4493183	-1.169023051	2.84E-26	1.99E-25
Cladopus_021863	root	shoot	324.7251911	106.0407064	215.3829487	1.621820578	3.03E-26	2.12E-25
Cladopus_010267	root	shoot	223.4113981	578.3058538	400.858626	-1.377814507	3.08E-26	2.16E-25
Cladopus_007966	root	shoot	651.1431339	272.487388	461.8152609	1.257995815	3.34E-26	2.33E-25
Cladopus_011943	root	shoot	74.47624616	252.9125659	163.694406	-1.760857097	3.80E-26	2.65E-25
Cladopus_011171	root	shoot	109.5042277	3.575717017	56.53997235	4.992805893	4.30E-26	2.99E-25
Cladopus_004724	root	shoot	1140.675877	567.1558265	853.9158516	1.009421247	6.43E-26	4.45E-25
Cladopus_017258	root	shoot	768.5432221	1599.231683	1183.887453	-1.059536325	6.43E-26	4.45E-25
Cladopus_000974	root	shoot	61.82516074	231.6281821	146.7266714	-1.902934367	6.47E-26	4.48E-25
Cladopus_007642	root	shoot	17.70936453	140.640776	79.17507028	-2.973953455	6.95E-26	4.81E-25
Cladopus_001023	root	shoot	660.6059578	319.6924616	490.1492097	1.044750816	8.56E-26	5.89E-25
Cladopus_015565	root	shoot	361.2676159	777.2109287	569.2392723	-1.106527353	9.64E-26	6.63E-25
Cladopus_020737	root	shoot	187.8599517	444.4279751	316.1439634	-1.243810181	1.04E-25	7.15E-25
Cladopus_024722	root	shoot	117.6853776	342.1562162	229.9207969	-1.544396494	1.08E-25	7.44E-25
Cladopus_024950	root	shoot	234.395875	62.1099143	148.2528947	1.899106108	1.14E-25	7.78E-25

Cladopus_003381	root	shoot	210.7033674	58.84524871	134.7743081	1.833132843	1.23E-25	8.40E-25
Cladopus_020541	root	shoot	151.7717824	28.31585085	90.04381663	2.407898436	1.34E-25	9.14E-25
Cladopus_022056	root	shoot	990.6765596	493.2314477	741.9540037	1.007069345	1.39E-25	9.47E-25
Cladopus_009015	root	shoot	2786.823265	1363.105372	2074.964319	1.032362985	1.41E-25	9.61E-25
Cladopus_011254	root	shoot	36.5694228	174.5741211	105.571772	-2.25137136	1.44E-25	9.82E-25
Cladopus_002302	root	shoot	257.7154615	571.1718832	414.4436723	-1.152104471	1.61E-25	1.10E-24
Cladopus_006242	root	shoot	148.3055779	397.1275792	272.7165785	-1.426498581	1.69E-25	1.15E-24
Cladopus_027187	root	shoot	310.6663797	662.0085403	486.33746	-1.090487125	1.88E-25	1.28E-24
Cladopus_014037	root	shoot	448.3914821	905.3549038	676.873193	-1.012982281	1.98E-25	1.35E-24
Cladopus_008150	root	shoot	173.5584756	34.32454679	103.9415112	2.366345589	2.15E-25	1.46E-24
Cladopus_017090	root	shoot	1641.178787	3290.188264	2465.683525	-1.004549783	2.19E-25	1.49E-24
Cladopus_012904	root	shoot	539.1984659	253.8497136	396.5240897	1.090162351	2.31E-25	1.57E-24
Cladopus_016614	root	shoot	245.7438573	64.90893138	155.3263943	1.919813736	2.34E-25	1.59E-24
Cladopus_001411	root	shoot	16.38483863	130.2378498	73.3113442	-2.988476566	2.35E-25	1.60E-24
Cladopus_024752	root	shoot	113.3999258	341.9720183	227.6859721	-1.587228636	2.38E-25	1.61E-24
Cladopus_024723	root	shoot	215.2147776	498.8126038	357.0136907	-1.209916867	2.67E-25	1.81E-24
Cladopus_014588	root	shoot	303.9437152	659.0514458	481.4975805	-1.117234194	2.93E-25	1.98E-24
Cladopus_010256	root	shoot	221.2692375	545.6580879	383.4636627	-1.300203164	2.99E-25	2.02E-24
Cladopus_000526	root	shoot	517.4911386	232.7504203	375.1207795	1.154694367	3.39E-25	2.29E-24
Cladopus_024700	root	shoot	394.4412987	146.978651	270.7099749	1.427815608	3.40E-25	2.29E-24
Cladopus_024119	root	shoot	221.5065777	63.90776428	142.707171	1.791258438	3.58E-25	2.41E-24
Cladopus_003785	root	shoot	253.3109037	79.96052757	166.6357156	1.675256178	3.58E-25	2.41E-24
Cladopus_003143	root	shoot	87.85632602	283.9391435	185.8977347	-1.68766688	3.60E-25	2.42E-24
Cladopus_023398	root	shoot	703.1570348	1474.725008	1088.941021	-1.070389599	3.81E-25	2.56E-24
Cladopus_017270	root	shoot	611.7284104	301.6944305	456.7114205	1.017687182	3.99E-25	2.68E-24
Cladopus_006078	root	shoot	276.4541906	584.6825866	430.5683886	-1.07859192	4.02E-25	2.69E-24
Cladopus_008746	root	shoot	299.7570578	771.5662723	535.661665	-1.36812075	4.19E-25	2.80E-24
Cladopus_015388	root	shoot	38.69586856	184.3967598	111.5463142	-2.253131603	4.46E-25	2.98E-24
Cladopus_005643	root	shoot	124.0822995	13.91351777	68.99790863	3.112447478	4.51E-25	3.02E-24
Cladopus_001477	root	shoot	269.4717718	615.7469841	442.609378	-1.18753337	4.81E-25	3.21E-24

Cladopus_002372	root	shoot	132.4965851	1.952967956	67.22477653	6.050064726	5.33E-25	3.55E-24
Cladopus_004607	root	shoot	40.34304216	194.3032496	117.3231459	-2.275825481	5.35E-25	3.57E-24
Cladopus_013941	root	shoot	442.4844945	153.9540253	298.2192599	1.524336455	5.39E-25	3.60E-24
Cladopus_005589	root	shoot	590.74042	288.8893113	439.8148657	1.032059533	5.45E-25	3.63E-24
Cladopus_018726	root	shoot	99.90655949	6.160630054	53.03359477	3.997625852	5.86E-25	3.90E-24
Cladopus_002919	root	shoot	184.436959	443.9498175	314.1933882	-1.271030892	6.62E-25	4.41E-24
Cladopus_014802	root	shoot	102.5782657	3.841623936	53.20994481	4.783118564	6.67E-25	4.44E-24
Cladopus_007778	root	shoot	166.7529774	418.1874938	292.4702356	-1.332305982	7.24E-25	4.80E-24
Cladopus_018333	root	shoot	243.1346716	527.499528	385.3170998	-1.11545683	7.24E-25	4.80E-24
Cladopus_012094	root	shoot	97.29415679	4.716977328	51.00556706	4.332823764	8.09E-25	5.36E-24
Cladopus_026877	root	shoot	900.0102129	1859.506311	1379.758262	-1.048266168	8.95E-25	5.92E-24
Cladopus_013421	root	shoot	644.9239979	314.6376481	479.780823	1.039787507	9.55E-25	6.31E-24
Cladopus_026991	root	shoot	186.2579647	42.35747364	114.3077192	2.122299613	9.87E-25	6.52E-24
Cladopus_009547	root	shoot	292.6998608	614.5627877	453.6313242	-1.071137644	1.17E-24	7.71E-24
Cladopus_000852	root	shoot	122.4224955	14.32270176	68.37259861	3.060310594	1.39E-24	9.16E-24
Cladopus_020953	root	shoot	94.14478696	278.8807706	186.5127788	-1.563887675	1.47E-24	9.68E-24
Cladopus_007608	root	shoot	207.7892302	39.04818444	123.4187073	2.427362471	1.53E-24	1.00E-23
Cladopus_016154	root	shoot	99.30805926	7.049304104	53.17868168	3.759738644	1.77E-24	1.16E-23
Cladopus_025474	root	shoot	412.2369517	177.8959194	295.0664356	1.211573728	2.11E-24	1.38E-23
Cladopus_006624	root	shoot	261.5896213	86.38920856	173.9894149	1.603337018	2.12E-24	1.39E-23
Cladopus_017124	root	shoot	380.9061964	773.6209926	577.2635945	-1.026264327	2.16E-24	1.41E-23
Cladopus_025557	root	shoot	384.841024	148.9066837	266.8738539	1.376975982	2.23E-24	1.46E-23
Cladopus_005406	root	shoot	202.1977065	471.0455902	336.6216484	-1.217252449	2.31E-24	1.51E-23
Cladopus_017571	root	shoot	764.0792322	41.40596541	402.7425988	4.198716407	2.33E-24	1.52E-23
Cladopus_024901	root	shoot	676.6457239	0	338.322862	12.05015408	2.60E-24	1.69E-23
Cladopus_011006	root	shoot	656.8406382	327.0401607	491.9403995	1.002746753	2.75E-24	1.79E-23
Cladopus_016188	root	shoot	257.2353305	75.87097901	166.5531547	1.76806751	2.81E-24	1.83E-23
Cladopus_023044	root	shoot	210.4777819	62.27302702	136.3754044	1.759643364	2.81E-24	1.83E-23
Cladopus_001770	root	shoot	445.2412511	201.023003	323.1321271	1.15012935	2.81E-24	1.83E-23
Cladopus_016084	root	shoot	178.7317719	419.0430135	298.8873927	-1.230516011	2.87E-24	1.87E-23

Cladopus_017196	root	shoot	298.6846165	631.2992346	464.9919255	-1.080855484	2.99E-24	1.95E-23
Cladopus_024045	root	shoot	155.301	379.5471628	267.4240814	-1.29132759	3.24E-24	2.10E-23
Cladopus_007755	root	shoot	133.9966738	364.9670678	249.4818708	-1.447546081	3.31E-24	2.15E-23
Cladopus_004477	root	shoot	222.033144	503.1719093	362.6025267	-1.181147961	3.39E-24	2.20E-23
Cladopus_002414	root	shoot	107.8002204	7.244238718	57.52222954	3.869358739	3.46E-24	2.24E-23
Cladopus_006373	root	shoot	168.8925485	412.9565415	290.924545	-1.287455921	3.78E-24	2.45E-23
Cladopus_021291	root	shoot	254.402412	630.5822454	442.4923287	-1.312336069	3.83E-24	2.48E-23
Cladopus_008283	root	shoot	560.5446436	267.4302003	413.9874219	1.065643846	3.90E-24	2.53E-23
Cladopus_021487	root	shoot	294.0672322	95.45231612	194.7597741	1.625757929	5.05E-24	3.26E-23
Cladopus_008525	root	shoot	306.3973941	642.4225416	474.4099678	-1.069267824	5.45E-24	3.51E-23
Cladopus_023586	root	shoot	123.1122448	16.37047309	69.74135896	2.902149311	6.27E-24	4.04E-23
Cladopus_006207	root	shoot	326.6953828	693.7415781	510.2184804	-1.088544613	6.35E-24	4.09E-23
Cladopus_008139	root	shoot	428.7307397	878.9929651	653.8618524	-1.036615527	6.46E-24	4.15E-23
Cladopus_003523	root	shoot	161.3231439	384.7551804	273.0391622	-1.254742819	7.50E-24	4.81E-23
Cladopus_004469	root	shoot	610.7997043	281.7410592	446.2703817	1.111772333	8.18E-24	5.24E-23
Cladopus_020461	root	shoot	436.919684	993.0337513	714.9767177	-1.186788896	9.04E-24	5.78E-23
Cladopus_013984	root	shoot	643.7950868	295.0089376	469.4020122	1.118666822	9.20E-24	5.87E-23
Cladopus_023296	root	shoot	528.5908874	195.6458786	362.118383	1.432426706	1.02E-23	6.53E-23
Cladopus_014334	root	shoot	394.1378271	792.4022406	593.2700339	-1.01003848	1.06E-23	6.75E-23
Cladopus_008148	root	shoot	349.3363663	772.8139525	561.0751594	-1.14593241	1.08E-23	6.86E-23
Cladopus_007209	root	shoot	196.9709198	460.2898655	328.6303926	-1.226776669	1.30E-23	8.28E-23
Cladopus_007525	root	shoot	276.6297534	96.15989711	186.3948253	1.535959554	1.31E-23	8.32E-23
Cladopus_015376	root	shoot	248.4028044	77.54657079	162.9746876	1.678231219	1.34E-23	8.50E-23
Cladopus_001149	root	shoot	178.7086185	46.18644315	112.4475308	1.945281586	1.36E-23	8.62E-23
Cladopus_006962	root	shoot	276.747255	646.656843	461.702049	-1.222685566	1.45E-23	9.21E-23
Cladopus_020248	root	shoot	578.7670617	251.3184548	415.0427582	1.200941916	1.58E-23	1.00E-22
Cladopus_010007	root	shoot	258.5997161	544.8184708	401.7090935	-1.078556557	1.87E-23	1.18E-22
Cladopus_027049	root	shoot	85.24850467	258.0179327	171.6332187	-1.597843906	2.04E-23	1.29E-22
Cladopus_001892	root	shoot	151.9227054	28.55126638	90.2369859	2.37968287	2.22E-23	1.40E-22
Cladopus_006149	root	shoot	519.3508881	1086.409953	802.8804204	-1.066294239	2.43E-23	1.53E-22

Cladopus_007034	root	shoot	518.1940174	219.0165178	368.6052676	1.245456825	2.44E-23	1.54E-22
Cladopus_023879	root	shoot	94.23863612	5.730279916	49.98445802	4.101371303	2.68E-23	1.68E-22
Cladopus_010104	root	shoot	185.8061831	48.29704866	117.0516159	1.957532126	2.68E-23	1.68E-22
Cladopus_001453	root	shoot	319.0812904	653.1183877	486.0998391	-1.033907426	2.83E-23	1.77E-22
Cladopus_015067	root	shoot	263.0333752	588.952483	425.9929291	-1.159208896	2.95E-23	1.85E-22
Cladopus_008659	root	shoot	145.4648716	31.82059557	88.64273356	2.187281649	3.01E-23	1.88E-22
Cladopus_018723	root	shoot	344.8566616	137.4031254	241.1298935	1.336623208	3.15E-23	1.97E-22
Cladopus_001225	root	shoot	10.07322432	118.7205971	64.39691074	-3.576476138	3.26E-23	2.04E-22
Cladopus_002018	root	shoot	194.1061948	52.77742334	123.4418091	1.866483106	3.48E-23	2.18E-22
Cladopus_005287	root	shoot	139.2560208	27.93049793	83.59325937	2.313725657	3.52E-23	2.20E-22
Cladopus_004889	root	shoot	636.5586085	306.6809366	471.6197725	1.047671786	4.03E-23	2.52E-22
Cladopus_021625	root	shoot	95.97507754	8.516787897	52.24593272	3.521264596	4.14E-23	2.58E-22
Cladopus_016416	root	shoot	200.961461	462.7965606	331.8790108	-1.199445449	4.50E-23	2.80E-22
Cladopus_006432	root	shoot	7.497470737	109.4369535	58.46721211	-3.843512946	4.91E-23	3.05E-22
Cladopus_015086	root	shoot	132.8276588	20.14245718	76.48505797	2.753561065	5.34E-23	3.32E-22
Cladopus_026697	root	shoot	145.146679	1.586929794	73.36680441	6.661775	5.64E-23	3.51E-22
Cladopus_024974	root	shoot	101.4075135	282.5949557	192.0012346	-1.476035471	6.49E-23	4.02E-22
Cladopus_010067	root	shoot	327.0405733	129.4614425	228.2510079	1.339860723	7.07E-23	4.37E-22
Cladopus_017446	root	shoot	180.2621912	45.8249213	113.0435563	1.984795751	7.57E-23	4.68E-22
Cladopus_000804	root	shoot	76.43146218	262.7132229	169.5723426	-1.775287411	8.85E-23	5.46E-22
Cladopus_002629	root	shoot	13.49943411	118.6188408	66.05913746	-3.147492467	9.25E-23	5.70E-22
Cladopus_003479	root	shoot	322.0456906	117.5213926	219.7835416	1.456872492	9.41E-23	5.79E-22
Cladopus_001596	root	shoot	328.147194	709.2957629	518.7214784	-1.112714736	1.00E-22	6.16E-22
Cladopus_025420	root	shoot	184.8933285	51.8204419	118.3568852	1.846228842	1.02E-22	6.28E-22
Cladopus_009291	root	shoot	93.64521216	8.11359801	50.87940508	3.506561316	1.09E-22	6.69E-22
Cladopus_024006	root	shoot	207.0018041	62.43347483	134.7176395	1.74207568	1.13E-22	6.92E-22
Cladopus_023960	root	shoot	135.3663864	361.2933616	248.329874	-1.422196365	1.18E-22	7.24E-22
Cladopus_001061	root	shoot	111.845115	311.8236043	211.8343597	-1.475902304	1.21E-22	7.39E-22
Cladopus_006883	root	shoot	261.0894294	583.2222011	422.1558153	-1.159437238	1.25E-22	7.63E-22
Cladopus_005488	root	shoot	47.87877053	186.1373298	117.0080502	-1.95989736	1.31E-22	8.03E-22

Cladopus_026679	root	shoot	89.9061344	6.096318078	48.00122624	3.902952709	1.33E-22	8.11E-22
Cladopus_007788	root	shoot	294.89292	594.5291188	444.7110194	-1.013822443	1.47E-22	8.98E-22
Cladopus_019104	root	shoot	86.39598136	5.256117724	45.82604954	4.066267602	1.68E-22	1.02E-21
Cladopus_022897	root	shoot	89.53111222	6.096318078	47.81371515	3.896875059	1.70E-22	1.04E-21
Cladopus_013997	root	shoot	1018.709877	471.9014816	745.3056792	1.111081324	1.71E-22	1.04E-21
Cladopus_013260	root	shoot	205.3857044	465.8797576	335.632731	-1.185090446	1.82E-22	1.10E-21
Cladopus_002127	root	shoot	198.9246534	466.4130512	332.6688523	-1.230526702	2.12E-22	1.29E-21
Cladopus_017650	root	shoot	255.1031809	565.6566681	410.3799245	-1.151596125	2.31E-22	1.40E-21
Cladopus_020395	root	shoot	159.3848914	39.68479117	99.53484126	2.005840451	2.42E-22	1.46E-21
Cladopus_010284	root	shoot	99.18139912	11.43073467	55.30606689	3.144461407	2.45E-22	1.48E-21
Cladopus_001311	root	shoot	0.362269633	1180.083656	590.2229631	-11.48508953	2.51E-22	1.52E-21
Cladopus_013686	root	shoot	104.2555257	12.98717306	58.62134939	2.994559176	3.11E-22	1.88E-21
Cladopus_013061	root	shoot	331.1530647	134.3387203	232.7458925	1.301562214	3.17E-22	1.91E-21
Cladopus_003447	root	shoot	198.3666364	59.42938633	128.8980114	1.734866424	3.64E-22	2.19E-21
Cladopus_003468	root	shoot	520.682478	251.8672941	386.2748861	1.052589701	4.41E-22	2.64E-21
Cladopus_020217	root	shoot	3.637800604	116.3236658	59.98073319	-5.073404282	4.42E-22	2.65E-21
Cladopus_002841	root	shoot	84.09793304	5.119500986	44.60871701	4.032069648	4.52E-22	2.71E-21
Cladopus_018991	root	shoot	83.71238794	254.1079994	168.9101937	-1.604064675	4.56E-22	2.73E-21
Cladopus_007470	root	shoot	532.8752191	259.4657281	396.1704736	1.039887991	4.71E-22	2.82E-21
Cladopus_009630	root	shoot	17.52352302	122.8988077	70.21116535	-2.831326114	4.74E-22	2.83E-21
Cladopus_003810	root	shoot	229.2706097	512.7688294	371.0197195	-1.163857055	5.02E-22	3.00E-21
Cladopus_002907	root	shoot	108.1117281	15.33001025	61.72086918	2.826931524	5.05E-22	3.01E-21
Cladopus_013683	root	shoot	237.8686357	76.12371061	156.9961732	1.633716149	5.08E-22	3.03E-21
Cladopus_021404	root	shoot	320.1388782	129.7889964	224.9639373	1.296896313	5.18E-22	3.09E-21
Cladopus_008428	root	shoot	97.5852357	8.811853755	53.19854473	3.496412415	5.48E-22	3.26E-21
Cladopus_021304	root	shoot	209.5930283	483.6203954	346.6067119	-1.210054932	5.51E-22	3.28E-21
Cladopus_014995	root	shoot	75.48889152	231.9930332	153.7409624	-1.624166917	5.53E-22	3.29E-21
Cladopus_015015	root	shoot	16.1103504	117.1036969	66.60702362	-2.864862095	6.64E-22	3.94E-21
Cladopus_004626	root	shoot	19.19905243	133.1837657	76.19140908	-2.800358878	6.83E-22	4.05E-21
Cladopus_003913	root	shoot	435.6210571	178.9860411	307.3035491	1.28175494	7.36E-22	4.36E-21

Cladopus_024900	root	shoot	429.0141359	0	214.5070679	11.39217497	8.43E-22	4.98E-21
Cladopus_004093	root	shoot	200.050337	59.41887592	129.7346065	1.742058694	8.61E-22	5.08E-21
Cladopus_015176	root	shoot	34.10719992	170.1748536	102.1410268	-2.333053286	8.74E-22	5.15E-21
Cladopus_021867	root	shoot	176.3362846	49.25602879	112.7961567	1.840024202	9.78E-22	5.76E-21
Cladopus_018461	root	shoot	105.3586811	14.22323675	59.79095891	2.886871446	1.06E-21	6.25E-21
Cladopus_016374	root	shoot	115.8911151	315.4929396	215.6920273	-1.444492009	1.13E-21	6.63E-21
Cladopus_025952	root	shoot	291.5641492	115.1732275	203.3686884	1.342124448	1.17E-21	6.89E-21
Cladopus_016837	root	shoot	103.3869965	1.852170484	52.61958349	5.699141324	1.19E-21	6.98E-21
Cladopus_003171	root	shoot	164.4689238	1.148586869	82.80875536	7.168347463	1.28E-21	7.49E-21
Cladopus_006650	root	shoot	30.37035179	144.3166243	87.34348804	-2.254277505	1.37E-21	8.05E-21
Cladopus_002094	root	shoot	334.7032101	126.903397	230.8033036	1.400328355	1.41E-21	8.24E-21
Cladopus_000863	root	shoot	318.1644756	116.0347393	217.0996075	1.446622496	1.41E-21	8.25E-21
Cladopus_001300	root	shoot	151.2058424	370.0789476	260.642395	-1.29528332	1.41E-21	8.25E-21
Cladopus_009496	root	shoot	926.6920241	462.2169413	694.4544827	1.007173026	1.42E-21	8.31E-21
Cladopus_018420	root	shoot	8.011284497	99.32058137	53.66593293	-3.629270822	1.45E-21	8.50E-21
Cladopus_013572	root	shoot	613.1194042	1369.11665	991.1180272	-1.161679596	1.51E-21	8.78E-21
Cladopus_000065	root	shoot	109.2681192	17.51373209	63.39092564	2.660886969	1.59E-21	9.30E-21
Cladopus_003812	root	shoot	67.36209092	222.222054	144.7920724	-1.722197248	1.61E-21	9.36E-21
Cladopus_017289	root	shoot	268.885403	93.52931866	181.2073608	1.516013162	1.67E-21	9.70E-21
Cladopus_006065	root	shoot	241.094643	83.80148528	162.4480641	1.535342024	1.74E-21	1.01E-20
Cladopus_019031	root	shoot	245.6479006	76.07257396	160.8602373	1.698887337	1.75E-21	1.02E-20
Cladopus_005373	root	shoot	453.5782887	192.2074445	322.8928666	1.236363091	1.79E-21	1.04E-20
Cladopus_011850	root	shoot	488.5480722	233.8064288	361.1772505	1.065689977	1.95E-21	1.13E-20
Cladopus_011180	root	shoot	183.0189608	53.93785308	118.4784069	1.766526493	1.99E-21	1.15E-20
Cladopus_008843	root	shoot	245.1330929	521.5415991	383.337346	-1.088827203	2.08E-21	1.21E-20
Cladopus_002908	root	shoot	145.5921528	369.9444769	257.7683148	-1.342929993	2.08E-21	1.21E-20
Cladopus_025946	root	shoot	295.6427201	103.0527508	199.3477355	1.533415358	2.21E-21	1.28E-20
Cladopus_024965	root	shoot	321.4699603	661.9362999	491.7031301	-1.046181152	2.38E-21	1.38E-20
Cladopus_003291	root	shoot	178.7191414	407.4550148	293.0870781	-1.192234745	2.41E-21	1.39E-20
Cladopus_008240	root	shoot	104.0573085	13.80872489	58.93301671	2.942000135	2.50E-21	1.44E-20

Cladopus_008298	root	shoot	46.62184549	210.6626973	128.6422714	-2.184298871	3.05E-21	1.76E-20
Cladopus_004556	root	shoot	305.768936	616.4222847	461.0956103	-1.008744116	3.13E-21	1.80E-20
Cladopus_015385	root	shoot	348.3799441	706.0620266	527.2209854	-1.016466421	3.13E-21	1.80E-20
Cladopus_004259	root	shoot	295.498604	110.9632741	203.2309391	1.414607347	3.57E-21	2.05E-20
Cladopus_025210	root	shoot	588.125055	277.3604441	432.7427496	1.086828711	3.83E-21	2.19E-20
Cladopus_015917	root	shoot	102.4951877	272.4694057	187.4822967	-1.410678063	4.00E-21	2.29E-20
Cladopus_023425	root	shoot	484.1717417	240.9435024	362.557622	1.006854182	4.02E-21	2.30E-20
Cladopus_020944	root	shoot	447.3945727	171.4810761	309.4378244	1.3780229	4.05E-21	2.31E-20
Cladopus_012822	root	shoot	218.5513427	458.9612234	338.756283	-1.072055632	4.06E-21	2.32E-20
Cladopus_004178	root	shoot	233.4396865	81.03347853	157.2365825	1.522952022	4.63E-21	2.64E-20
Cladopus_020711	root	shoot	224.8432714	71.15452062	147.998896	1.658039838	4.64E-21	2.64E-20
Cladopus_006720	root	shoot	657.8767903	1314.71307	986.29493	-1.00084461	4.76E-21	2.71E-20
Cladopus_003844	root	shoot	179.4148365	48.8773362	114.1460863	1.88659892	4.95E-21	2.82E-20
Cladopus_018839	root	shoot	117.0946823	18.99919818	68.04694022	2.631802818	5.80E-21	3.29E-20
Cladopus_025238	root	shoot	9849.805345	26915.57053	18382.68794	-1.450352224	5.94E-21	3.36E-20
Cladopus_004293	root	shoot	458.1356182	0.538474168	229.3370462	9.647030192	6.09E-21	3.45E-20
Cladopus_018856	root	shoot	174.3425626	45.53651577	109.9395392	1.947847562	6.49E-21	3.68E-20
Cladopus_026924	root	shoot	88.20955914	6.713757338	47.46165824	3.789493761	7.44E-21	4.20E-20
Cladopus_002873	root	shoot	597.6706035	265.38191	431.5262568	1.171812667	8.10E-21	4.57E-20
Cladopus_015085	root	shoot	147.1491933	29.8754731	88.51233318	2.283613512	8.69E-21	4.89E-20
Cladopus_009175	root	shoot	188.5085968	412.6360195	300.5723082	-1.128939336	8.76E-21	4.93E-20
Cladopus_023830	root	shoot	127.1778084	312.3010957	219.7394521	-1.297210299	9.09E-21	5.11E-20
Cladopus_004349	root	shoot	245.8485957	491.7875024	368.818049	-1.000863548	9.57E-21	5.37E-20
Cladopus_008290	root	shoot	307.3937035	110.6132215	209.0034625	1.482469215	9.85E-21	5.53E-20
Cladopus_012686	root	shoot	152.0420597	32.4128713	92.22746549	2.206606303	1.01E-20	5.68E-20
Cladopus_005992	root	shoot	477.3039607	981.6927018	729.4983313	-1.043460961	1.07E-20	6.00E-20
Cladopus_025950	root	shoot	300.0500024	113.1266433	206.5883229	1.420066814	1.09E-20	6.10E-20
Cladopus_004055	root	shoot	144.7485035	33.2337569	88.99113019	2.104795604	1.16E-20	6.49E-20
Cladopus_012101	root	shoot	336.0236431	0	168.0118215	11.04058007	1.25E-20	7.01E-20
Cladopus_023040	root	shoot	411.8090666	188.9306454	300.369856	1.130154851	1.37E-20	7.67E-20

Cladopus_001749	root	shoot	102.7073996	307.8934804	205.30044	-1.581424611	1.39E-20	7.76E-20
Cladopus_010581	root	shoot	100.3311054	286.9279146	193.62951	-1.507698496	1.60E-20	8.87E-20
Cladopus_018242	root	shoot	40.30466242	160.9430148	100.6238386	-1.990244849	1.60E-20	8.91E-20
Cladopus_012453	root	shoot	91.71091989	256.5230604	174.1169901	-1.486400775	1.72E-20	9.55E-20
Cladopus_005021	root	shoot	184.7999718	411.4044721	298.102222	-1.157470003	1.89E-20	1.05E-19
Cladopus_024902	root	shoot	335.7589281	0	167.879464	11.03803297	1.90E-20	1.05E-19
Cladopus_023095	root	shoot	811.471217	384.9621052	598.2166611	1.079144557	1.91E-20	1.06E-19
Cladopus_023484	root	shoot	253.4546495	96.31849156	174.8865705	1.395346067	2.03E-20	1.12E-19
Cladopus_010663	root	shoot	90.37537207	2.296507509	46.33593979	5.152263891	2.03E-20	1.12E-19
Cladopus_001416	root	shoot	369.8941199	159.8460855	264.8701027	1.215215987	2.24E-20	1.23E-19
Cladopus_024051	root	shoot	43.91695793	165.3530834	104.6350207	-1.912183734	2.48E-20	1.37E-19
Cladopus_005695	root	shoot	368.9318677	163.7435077	266.3376877	1.166615406	2.58E-20	1.42E-19
Cladopus_023590	root	shoot	363.1034923	150.9181149	257.0108036	1.268220963	2.63E-20	1.45E-19
Cladopus_024711	root	shoot	262.256954	548.0297728	405.1433634	-1.067095589	2.68E-20	1.48E-19
Cladopus_005434	root	shoot	83.0335343	242.2181956	162.6258649	-1.542678591	2.75E-20	1.52E-19
Cladopus_014339	root	shoot	140.8175205	333.6119735	237.214747	-1.247218995	2.85E-20	1.57E-19
Cladopus_021720	root	shoot	79.06417159	251.2414884	165.15283	-1.663844773	2.85E-20	1.57E-19
Cladopus_014440	root	shoot	69.46130095	220.2325986	144.8469498	-1.662743274	2.91E-20	1.60E-19
Cladopus_002728	root	shoot	508.3084849	228.6432631	368.475874	1.154727837	3.16E-20	1.74E-19
Cladopus_004490	root	shoot	720.4508753	1478.633966	1099.542421	-1.039514892	3.16E-20	1.74E-19
Cladopus_004941	root	shoot	288.4163879	115.8130181	202.114703	1.312506593	3.21E-20	1.76E-19
Cladopus_027259	root	shoot	79.70922277	3.130713763	41.41996826	4.667345218	3.42E-20	1.87E-19
Cladopus_009804	root	shoot	221.1667182	458.0015751	339.5841466	-1.053433399	3.81E-20	2.08E-19
Cladopus_020299	root	shoot	180.1096597	393.3296219	286.7196408	-1.12521212	5.07E-20	2.76E-19
Cladopus_007353	root	shoot	216.7101628	63.01761244	139.8638876	1.796426649	5.40E-20	2.94E-19
Cladopus_024270	root	shoot	189.0359063	0.876019621	94.95596297	7.784581106	6.64E-20	3.60E-19
Cladopus_022894	root	shoot	107.9583312	300.37518	204.1667556	-1.472582785	7.05E-20	3.82E-19
Cladopus_025729	root	shoot	291.2724492	0	145.6362246	10.83517051	7.14E-20	3.87E-19
Cladopus_013119	root	shoot	257.657274	98.07452621	177.8659001	1.384939966	7.51E-20	4.06E-19
Cladopus_024915	root	shoot	538.8305054	268.8693367	403.849921	1.000157236	8.34E-20	4.50E-19

Cladopus_016215	root	shoot	286.9899678	0	143.4949839	10.81239333	9.35E-20	5.03E-19
Cladopus_018083	root	shoot	193.7164346	419.1284935	306.4224641	-1.1146734	1.01E-19	5.42E-19
Cladopus_014215	root	shoot	126.9506143	321.890023	224.4203187	-1.344312467	1.15E-19	6.16E-19
Cladopus_012723	root	shoot	227.947943	472.2413203	350.0946316	-1.049464231	1.16E-19	6.24E-19
Cladopus_001089	root	shoot	150.2396259	41.34402572	95.79182582	1.870711619	1.47E-19	7.84E-19
Cladopus_010617	root	shoot	18.48342757	114.4456655	66.46454655	-2.620629653	1.49E-19	7.93E-19
Cladopus_018394	root	shoot	82.24894834	232.1746471	157.2117977	-1.496095559	1.51E-19	8.02E-19
Cladopus_021324	root	shoot	122.1919622	21.33352364	71.76274293	2.549688786	1.51E-19	8.03E-19
Cladopus_016137	root	shoot	261.7189995	530.843744	396.2813717	-1.021823657	1.54E-19	8.20E-19
Cladopus_002991	root	shoot	460.3757273	225.9712449	343.1734861	1.025039126	1.66E-19	8.83E-19
Cladopus_021293	root	shoot	30.01773976	140.4570181	85.23737891	-2.219396518	1.70E-19	9.06E-19
Cladopus_022221	root	shoot	265.3813177	0	132.6906589	10.70127428	1.96E-19	1.04E-18
Cladopus_008421	root	shoot	326.9115614	129.5272322	228.2193968	1.332778565	1.99E-19	1.05E-18
Cladopus_022031	root	shoot	236.907371	518.7385866	377.8229788	-1.131706802	2.08E-19	1.10E-18
Cladopus_006818	root	shoot	178.5465515	49.6003799	114.0734657	1.826733656	2.10E-19	1.11E-18
Cladopus_009417	root	shoot	446.0085031	219.424743	332.716623	1.025697146	2.11E-19	1.12E-18
Cladopus_017997	root	shoot	139.0789716	322.117593	230.5982823	-1.21546831	2.14E-19	1.13E-18
Cladopus_019177	root	shoot	87.62108285	6.547315432	47.08419914	3.738333749	2.15E-19	1.14E-18
Cladopus_027154	root	shoot	232.3709506	477.7335198	355.0522352	-1.038145292	2.19E-19	1.15E-18
Cladopus_024794	root	shoot	284.8882905	112.8494125	198.8688515	1.339404596	2.19E-19	1.16E-18
Cladopus_021275	root	shoot	110.0158118	19.41304381	64.71442782	2.477933476	2.24E-19	1.18E-18
Cladopus_019018	root	shoot	200.7477626	57.21731907	128.9825409	1.823611708	2.34E-19	1.23E-18
Cladopus_008821	root	shoot	101.6587263	14.73787985	58.19830306	2.746517227	2.34E-19	1.23E-18
Cladopus_016056	root	shoot	113.3215513	291.2899474	202.3057494	-1.364589701	2.35E-19	1.24E-18
Cladopus_008511	root	shoot	87.27503757	9.849798971	48.56241827	3.13437212	2.38E-19	1.25E-18
Cladopus_002014	root	shoot	18.19779531	113.0243661	65.6110807	-2.647308137	2.40E-19	1.26E-18
Cladopus_021474	root	shoot	73.11494903	4.243481365	38.6792152	4.09393893	2.52E-19	1.32E-18
Cladopus_012549	root	shoot	128.147242	328.3483838	228.2478129	-1.36457941	2.70E-19	1.42E-18
Cladopus_019224	root	shoot	644.7450879	306.2015294	475.4733086	1.076625623	2.80E-19	1.47E-18
Cladopus_023213	root	shoot	183.2735127	392.0791958	287.6763542	-1.100982287	2.81E-19	1.47E-18

Cladopus_013862	root	shoot	486.9074484	0.301726186	243.6045873	10.61316442	3.46E-19	1.80E-18
Cladopus_018142	root	shoot	41.08256359	157.7899477	99.43625567	-1.946461699	3.62E-19	1.89E-18
Cladopus_012878	root	shoot	391.0189271	180.3063978	285.6626624	1.114042678	3.94E-19	2.05E-18
Cladopus_017847	root	shoot	248.7713925	0	124.3856963	10.60830924	4.17E-19	2.17E-18
Cladopus_017981	root	shoot	74.08549862	5.019369743	39.55243418	3.921405994	4.19E-19	2.18E-18
Cladopus_004050	root	shoot	4.312072896	89.19821515	46.75514402	-4.402225602	4.24E-19	2.20E-18
Cladopus_008160	root	shoot	19.73539845	113.444872	66.59013525	-2.536878318	5.13E-19	2.66E-18
Cladopus_012748	root	shoot	223.6130936	77.87841476	150.7457542	1.522567617	5.20E-19	2.70E-18
Cladopus_016259	root	shoot	205.7464876	67.61676689	136.6816272	1.592158129	5.23E-19	2.71E-18
Cladopus_020930	root	shoot	156.5595335	353.2050081	254.8822708	-1.172861264	5.45E-19	2.82E-18
Cladopus_000866	root	shoot	190.017722	398.4856065	294.2516642	-1.067515174	5.64E-19	2.91E-18
Cladopus_019606	root	shoot	557.677072	273.5570117	415.6170418	1.031844629	5.64E-19	2.91E-18
Cladopus_008592	root	shoot	8.711061879	90.25599406	49.48352797	-3.366801289	5.70E-19	2.95E-18
Cladopus_000654	root	shoot	651.0798661	297.6763751	474.3781206	1.13060197	6.16E-19	3.18E-18
Cladopus_009512	root	shoot	304.4608746	111.8704513	208.165663	1.450394164	6.22E-19	3.21E-18
Cladopus_005879	root	shoot	401.0268893	845.0017528	623.014321	-1.079359124	6.86E-19	3.54E-18
Cladopus_002675	root	shoot	20.64813096	122.2037378	71.42593436	-2.560705761	6.92E-19	3.56E-18
Cladopus_001024	root	shoot	105.1826193	20.82220973	63.00241451	2.334541399	7.91E-19	4.06E-18
Cladopus_018473	root	shoot	143.9900437	340.6176148	242.3038292	-1.238693498	8.19E-19	4.21E-18
Cladopus_009759	root	shoot	244.3684433	91.36876166	167.8686025	1.412589013	8.21E-19	4.22E-18
Cladopus_023263	root	shoot	191.2012267	60.98582472	126.0935257	1.641387241	8.54E-19	4.38E-18
Cladopus_011084	root	shoot	1716.311244	446.1846652	1081.247954	1.944129057	8.69E-19	4.45E-18
Cladopus_016832	root	shoot	228.765344	0	114.382672	10.48702677	1.13E-18	5.74E-18
Cladopus_008340	root	shoot	772.1066449	377.064604	574.5856245	1.032155954	1.17E-18	5.96E-18
Cladopus_008429	root	shoot	173.7987886	55.5494255	114.674107	1.638938591	1.30E-18	6.59E-18
Cladopus_021740	root	shoot	91.76267327	248.8370042	170.2998387	-1.437940845	1.36E-18	6.91E-18
Cladopus_014675	root	shoot	683.8031226	298.2533335	491.0282281	1.199101931	1.37E-18	6.95E-18
Cladopus_015305	root	shoot	180.8637929	54.02880637	117.4462996	1.746784077	1.46E-18	7.42E-18
Cladopus_014444	root	shoot	197.1296477	428.2714568	312.7005522	-1.120127934	1.62E-18	8.16E-18
Cladopus_016240	root	shoot	435.4550233	213.6576039	324.5563136	1.026776602	1.81E-18	9.12E-18

Cladopus_018586	root	shoot	178.3605878	374.1581331	276.2593605	-1.069597625	1.91E-18	9.64E-18
Cladopus_003553	root	shoot	248.6504233	88.12141215	168.3859177	1.485998448	2.00E-18	1.01E-17
Cladopus_005359	root	shoot	188.0070366	403.0232631	295.5151499	-1.099130331	2.06E-18	1.04E-17
Cladopus_003414	root	shoot	133.487071	33.4426784	83.4648747	1.993045418	2.18E-18	1.10E-17
Cladopus_023376	root	shoot	205.3351891	63.70668827	134.5209387	1.677819199	2.26E-18	1.14E-17
Cladopus_011760	root	shoot	130.707026	24.45705618	77.58204108	2.379567065	2.29E-18	1.15E-17
Cladopus_023238	root	shoot	148.4277788	38.34726378	93.38752127	1.940015882	2.33E-18	1.17E-17
Cladopus_017332	root	shoot	214.4794606	0	107.2397303	10.39236677	2.46E-18	1.23E-17
Cladopus_015583	root	shoot	390.3739874	167.1702481	278.7721178	1.226137964	2.53E-18	1.27E-17
Cladopus_022788	root	shoot	78.82113003	226.8813796	152.8512548	-1.517392398	2.55E-18	1.27E-17
Cladopus_002210	root	shoot	449.8567891	221.3253871	335.5910881	1.025001304	2.65E-18	1.33E-17
Cladopus_023104	root	shoot	209.8703672	0	104.9351836	10.36102671	3.10E-18	1.54E-17
Cladopus_019340	root	shoot	99.01128298	253.256917	176.1341	-1.357958468	3.31E-18	1.65E-17
Cladopus_024121	root	shoot	392.8754619	172.9282092	282.9018355	1.192521893	3.32E-18	1.65E-17
Cladopus_007996	root	shoot	367.1617459	167.553974	267.35786	1.124617949	3.35E-18	1.66E-17
Cladopus_014711	root	shoot	75.3124634	213.2237773	144.2681204	-1.497382264	3.66E-18	1.81E-17
Cladopus_022668	root	shoot	23.18488371	116.4511046	69.81799413	-2.338837839	3.74E-18	1.86E-17
Cladopus_002885	root	shoot	103.0351277	20.08280684	61.55896726	2.363336257	4.19E-18	2.08E-17
Cladopus_008760	root	shoot	169.1182562	367.0083241	268.0632902	-1.115249236	4.37E-18	2.16E-17
Cladopus_000243	root	shoot	43.3184577	157.7634518	100.5409547	-1.871998276	4.70E-18	2.33E-17
Cladopus_003245	root	shoot	203.1256654	73.29990557	138.2127855	1.469690819	4.91E-18	2.43E-17
Cladopus_003137	root	shoot	525.2737532	237.8384691	381.5561111	1.140048885	5.05E-18	2.49E-17
Cladopus_005030	root	shoot	661.4148301	298.5812611	479.9980456	1.149078129	5.11E-18	2.52E-17
Cladopus_016393	root	shoot	143.8299515	330.6881845	237.259068	-1.20482504	5.17E-18	2.55E-17
Cladopus_015765	root	shoot	401.3292514	183.2791833	292.3042173	1.133304888	5.31E-18	2.62E-17
Cladopus_018346	root	shoot	332.7377178	145.6496354	239.1936766	1.192811437	5.47E-18	2.69E-17
Cladopus_024411	root	shoot	283.8523631	568.7477086	426.3000359	-1.006271355	5.49E-18	2.70E-17
Cladopus_009344	root	shoot	402.4588952	191.1358241	296.7973596	1.069657411	5.50E-18	2.71E-17
Cladopus_024365	root	shoot	199.2080561	0	99.60402803	10.28769579	5.68E-18	2.79E-17
Cladopus_009390	root	shoot	297.7332665	104.5315546	201.1324105	1.508429352	6.05E-18	2.97E-17

Cladopus_019779	root	shoot	89.1081748	13.75706734	51.43262107	2.72019953	6.21E-18	3.05E-17
Cladopus_017846	root	shoot	301.8870999	133.042342	217.464721	1.189651375	6.26E-18	3.07E-17
Cladopus_025062	root	shoot	365.9588039	176.0164414	270.9876226	1.054291346	6.33E-18	3.11E-17
Cladopus_010127	root	shoot	161.5330041	351.5456926	256.5393483	-1.120889201	6.80E-18	3.34E-17
Cladopus_000221	root	shoot	220.5136139	70.94389502	145.7287544	1.641094959	7.22E-18	3.54E-17
Cladopus_016369	root	shoot	54.8871894	195.9977865	125.4424879	-1.831523285	7.82E-18	3.83E-17
Cladopus_021511	root	shoot	219.4315084	469.0404458	344.2359771	-1.099218939	8.08E-18	3.95E-17
Cladopus_004922	root	shoot	349.4543668	165.4803749	257.4673708	1.075351513	8.17E-18	3.99E-17
Cladopus_019227	root	shoot	233.5763706	89.76917744	161.672774	1.370583637	8.49E-18	4.15E-17
Cladopus_017132	root	shoot	121.178441	16.63919026	68.90881563	2.83022779	8.61E-18	4.21E-17
Cladopus_016413	root	shoot	121.4707621	27.70240897	74.58658554	2.15628718	8.84E-18	4.31E-17
Cladopus_018038	root	shoot	203.1005307	61.27119369	132.1858622	1.728833856	9.31E-18	4.54E-17
Cladopus_022033	root	shoot	63.57014376	231.587035	147.5785894	-1.876776558	9.76E-18	4.75E-17
Cladopus_018017	root	shoot	416.4730084	203.1482597	309.810634	1.035428049	9.94E-18	4.84E-17
Cladopus_008129	root	shoot	504.5390725	250.0034241	377.2712483	1.007213352	1.10E-17	5.33E-17
Cladopus_018672	root	shoot	132.417097	305.6748152	219.0459561	-1.212107181	1.13E-17	5.47E-17
Cladopus_021258	root	shoot	31.25844452	131.0092218	81.13383318	-2.079016287	1.13E-17	5.50E-17
Cladopus_006856	root	shoot	74.22738513	4.557195751	39.39229044	3.891611061	1.23E-17	5.98E-17
Cladopus_001976	root	shoot	142.4615992	38.25912074	90.36035997	1.879752772	1.26E-17	6.09E-17
Cladopus_012145	root	shoot	178.019863	57.3213004	117.6705817	1.652075936	1.33E-17	6.44E-17
Cladopus_005335	root	shoot	89.70976996	231.717803	160.7137865	-1.367593841	1.46E-17	7.06E-17
Cladopus_009811	root	shoot	320.8651481	138.763961	229.8145545	1.200564358	1.48E-17	7.17E-17
Cladopus_022968	root	shoot	80.42026628	8.937293855	44.67878007	3.120286267	1.84E-17	8.87E-17
Cladopus_004606	root	shoot	187.9336163	63.36781232	125.6507143	1.578171724	2.10E-17	1.01E-16
Cladopus_006343	root	shoot	371.4299965	168.917852	270.1739242	1.142726486	2.11E-17	1.01E-16
Cladopus_008822	root	shoot	422.7750872	204.6226984	313.6988928	1.053773287	2.12E-17	1.02E-16
Cladopus_010442	root	shoot	165.4095091	366.2572237	265.8333664	-1.150076375	2.18E-17	1.04E-16
Cladopus_004200	root	shoot	354.8444522	156.4601205	255.6522864	1.182052037	2.23E-17	1.07E-16
Cladopus_003593	root	shoot	305.3520663	133.7897357	219.570901	1.18814635	2.24E-17	1.08E-16
Cladopus_002499	root	shoot	257.012467	105.1888105	181.1006388	1.295175136	2.29E-17	1.10E-16

Cladopus_016897	root	shoot	273.7621777	114.0383349	193.9002563	1.270891175	2.32E-17	1.11E-16
Cladopus_025374	root	shoot	70.74026334	6.908025723	38.82414453	3.40930741	2.32E-17	1.11E-16
Cladopus_009661	root	shoot	8878.798828	23469.09664	16173.94774	-1.402408801	2.34E-17	1.12E-16
Cladopus_006307	root	shoot	75.15608723	6.232268588	40.69417791	3.578341799	2.56E-17	1.23E-16
Cladopus_004972	root	shoot	44.34212497	165.4107331	104.876429	-1.897970963	2.59E-17	1.24E-16
Cladopus_017617	root	shoot	203.614473	411.2879817	307.4512274	-1.012090435	2.63E-17	1.26E-16
Cladopus_006110	root	shoot	418.7318116	203.3251311	311.0284713	1.042740526	2.85E-17	1.36E-16
Cladopus_020377	root	shoot	137.8366583	308.1847605	223.0107094	-1.158984011	2.90E-17	1.38E-16
Cladopus_020561	root	shoot	155.4779271	340.0266695	247.7522983	-1.128835858	2.98E-17	1.42E-16
Cladopus_005548	root	shoot	151.7974096	45.66647218	98.73194089	1.747215135	3.06E-17	1.46E-16
Cladopus_026486	root	shoot	212.6114114	432.2581282	322.4347698	-1.024955711	3.12E-17	1.49E-16
Cladopus_007471	root	shoot	99.9074248	246.8559152	173.38167	-1.304334334	3.13E-17	1.49E-16
Cladopus_022564	root	shoot	165.8926194	49.38946168	107.6410405	1.746048319	3.18E-17	1.51E-16
Cladopus_019335	root	shoot	241.6400296	91.11136647	166.375698	1.397073007	3.42E-17	1.63E-16
Cladopus_002343	root	shoot	174.2578761	57.55671592	115.907296	1.605800432	3.56E-17	1.69E-16
Cladopus_023859	root	shoot	123.1179357	285.959379	204.5386573	-1.220351729	3.66E-17	1.74E-16
Cladopus_022041	root	shoot	277.1767486	110.1172269	193.6469878	1.338907782	3.86E-17	1.83E-16
Cladopus_022759	root	shoot	200.9953856	71.81443948	136.4049125	1.486682308	3.88E-17	1.84E-16
Cladopus_001742	root	shoot	3.723973485	78.5135437	41.11875859	-4.365162768	4.17E-17	1.98E-16
Cladopus_013523	root	shoot	178.7603719	369.2528795	274.0066257	-1.050186602	4.25E-17	2.02E-16
Cladopus_017963	root	shoot	110.2071	261.0063282	185.6067141	-1.242400155	4.47E-17	2.12E-16
Cladopus_005242	root	shoot	1.474583622	120.7137534	61.0941685	-6.308176467	4.62E-17	2.18E-16
Cladopus_011542	root	shoot	291.8600537	125.3830705	208.6215621	1.218213734	4.69E-17	2.22E-16
Cladopus_009605	root	shoot	135.0771707	36.27618228	85.67667651	1.909771954	4.98E-17	2.35E-16
Cladopus_017688	root	shoot	84.49462217	232.2758182	158.3852202	-1.45659797	5.00E-17	2.36E-16
Cladopus_010249	root	shoot	115.545314	271.029603	193.2874585	-1.229679482	7.09E-17	3.32E-16
Cladopus_019538	root	shoot	158.9609665	0	79.48048325	9.963265981	7.29E-17	3.42E-16
Cladopus_009113	root	shoot	397.9435248	194.4503788	296.1969518	1.038198947	7.29E-17	3.42E-16
Cladopus_015940	root	shoot	158.8425997	0	79.42129984	9.961530676	7.60E-17	3.56E-16
Cladopus_009507	root	shoot	146.5196127	40.71540982	93.61751125	1.832441676	8.52E-17	3.98E-16

Cladopus_001793	root	shoot	63.40325114	2.728190105	33.06572062	4.481041905	8.95E-17	4.18E-16
Cladopus_012817	root	shoot	58.26697034	174.9731683	116.6200693	-1.587745048	8.96E-17	4.18E-16
Cladopus_025093	root	shoot	71.71972733	5.966361669	38.8430445	3.584155008	9.10E-17	4.24E-16
Cladopus_005140	root	shoot	317.6769036	640.2347414	478.9558225	-1.014149051	9.30E-17	4.33E-16
Cladopus_020199	root	shoot	169.7963666	58.27761561	114.0369911	1.536455275	9.61E-17	4.47E-16
Cladopus_003935	root	shoot	376.4732934	187.6845903	282.0789418	1.006442893	9.82E-17	4.56E-16
Cladopus_011439	root	shoot	191.079769	62.78182135	126.9307952	1.588926562	1.03E-16	4.76E-16
Cladopus_004452	root	shoot	105.8719959	252.8355975	179.3537967	-1.259803224	1.08E-16	5.00E-16
Cladopus_018898	root	shoot	200.8871753	70.76198844	135.8245819	1.517320152	1.09E-16	5.06E-16
Cladopus_012300	root	shoot	12.01061153	93.09001885	52.55031519	-2.964263254	1.19E-16	5.50E-16
Cladopus_004975	root	shoot	373.4512048	166.5716857	270.0114452	1.174100175	1.19E-16	5.51E-16
Cladopus_024147	root	shoot	141.0297324	41.27971375	91.15472309	1.78547607	1.21E-16	5.57E-16
Cladopus_005512	root	shoot	342.4133876	152.4090542	247.4112209	1.161193253	1.25E-16	5.79E-16
Cladopus_025874	root	shoot	79.11307427	11.51687902	45.31497664	2.762909909	1.34E-16	6.16E-16
Cladopus_014473	root	shoot	149.9422285	0	74.97111427	9.874625015	1.58E-16	7.27E-16
Cladopus_018782	root	shoot	63.89155981	5.392068234	34.64181402	3.55881054	1.59E-16	7.31E-16
Cladopus_005559	root	shoot	253.4107009	94.71890734	174.0648041	1.421906956	1.60E-16	7.35E-16
Cladopus_012411	root	shoot	39.24335861	138.6400006	88.94167961	-1.816342034	1.62E-16	7.42E-16
Cladopus_014702	root	shoot	153.224577	336.9542736	245.0894253	-1.133537681	1.78E-16	8.16E-16
Cladopus_004039	root	shoot	153.1483059	50.1520294	101.6501677	1.613321522	1.85E-16	8.46E-16
Cladopus_023491	root	shoot	232.536982	494.2675416	363.4022618	-1.090999095	1.87E-16	8.54E-16
Cladopus_017430	root	shoot	206.0531657	0.473495963	103.2633308	8.497037398	2.13E-16	9.72E-16
Cladopus_022969	root	shoot	63.91793021	4.185163489	34.05154685	3.904237703	2.14E-16	9.79E-16
Cladopus_019099	root	shoot	332.7947957	151.8406115	242.3177036	1.140088808	2.15E-16	9.83E-16
Cladopus_001115	root	shoot	1826.044614	5119.636402	3472.840508	-1.487691849	2.54E-16	1.16E-15
Cladopus_000789	root	shoot	66.20260489	192.6873102	129.4449575	-1.54391107	3.05E-16	1.39E-15
Cladopus_017008	root	shoot	285.4271103	121.5640925	203.4956014	1.238387817	3.08E-16	1.40E-15
Cladopus_006649	root	shoot	40.87914397	145.1534935	93.01631873	-1.823122941	3.31E-16	1.50E-15
Cladopus_008526	root	shoot	422.6174858	197.651025	310.1342554	1.098760512	3.55E-16	1.61E-15
Cladopus_024694	root	shoot	62.19201172	179.322775	120.7573934	-1.531364933	3.89E-16	1.76E-15

Cladopus_017570	root	shoot	249.903266	0.337545453	125.1204057	9.653006923	4.56E-16	2.06E-15
Cladopus_020956	root	shoot	284.8536162	122.8150356	203.8343259	1.220193952	4.57E-16	2.06E-15
Cladopus_016915	root	shoot	101.2442043	20.61528691	60.92974559	2.30730647	4.59E-16	2.07E-15
Cladopus_005664	root	shoot	135.1768394	308.9175031	222.0471712	-1.188840912	4.63E-16	2.09E-15
Cladopus_019924	root	shoot	237.7370215	95.57028433	166.6536529	1.317410145	4.81E-16	2.17E-15
Cladopus_004501	root	shoot	83.51949124	13.00900544	48.26424834	2.656322477	5.17E-16	2.33E-15
Cladopus_024760	root	shoot	435.1501939	216.3820269	325.7661104	1.013879533	5.20E-16	2.34E-15
Cladopus_004713	root	shoot	348.7262377	167.3882003	258.057219	1.060470606	5.63E-16	2.53E-15
Cladopus_025370	root	shoot	58.54071537	169.2025528	113.8716341	-1.533555027	5.67E-16	2.55E-15
Cladopus_018440	root	shoot	69.30628908	203.3533311	136.3298101	-1.558636796	5.79E-16	2.60E-15
Cladopus_010463	root	shoot	25.36964553	112.9298553	69.14975043	-2.146822093	6.00E-16	2.69E-15
Cladopus_001469	root	shoot	142.2892534	319.5952879	230.9422707	-1.169755687	6.91E-16	3.09E-15
Cladopus_011514	root	shoot	204.1194945	81.3431975	142.731346	1.328810365	6.97E-16	3.11E-15
Cladopus_020448	root	shoot	352.6048502	150.1730086	251.3889294	1.222160433	8.39E-16	3.74E-15
Cladopus_009391	root	shoot	11.92357333	84.86940099	48.39648716	-2.839836127	9.56E-16	4.24E-15
Cladopus_011614	root	shoot	6.84944674	74.42332891	40.63638783	-3.441746454	1.01E-15	4.48E-15
Cladopus_023463	root	shoot	47.34489835	161.9829567	104.6639275	-1.788677666	1.03E-15	4.57E-15
Cladopus_003186	root	shoot	22.43471724	106.2206143	64.32766578	-2.238610215	1.06E-15	4.71E-15
Cladopus_009372	root	shoot	409.5449453	200.1118304	304.8283879	1.038206534	1.06E-15	4.71E-15
Cladopus_027150	root	shoot	321.0340326	150.5480813	235.791057	1.099086666	1.11E-15	4.90E-15
Cladopus_007125	root	shoot	61.138757	5.026030072	33.08239354	3.645840374	1.14E-15	5.02E-15
Cladopus_008038	root	shoot	415.7301413	204.9552876	310.3427145	1.022237578	1.14E-15	5.04E-15
Cladopus_016884	root	shoot	385.3048256	186.9491828	286.1270042	1.043593903	1.15E-15	5.09E-15
Cladopus_009294	root	shoot	463.2253586	231.7035235	347.464441	1.001687637	1.21E-15	5.32E-15
Cladopus_024726	root	shoot	157.4169228	49.89996207	103.6584424	1.647033902	1.24E-15	5.46E-15
Cladopus_004194	root	shoot	361.4745138	169.4472916	265.4609027	1.085834659	1.33E-15	5.85E-15
Cladopus_009459	root	shoot	123.460768	284.2058619	203.833315	-1.207812837	1.40E-15	6.16E-15
Cladopus_006897	root	shoot	232.3713275	94.97415852	163.672743	1.298885841	1.70E-15	7.44E-15
Cladopus_017000	root	shoot	98.35124964	240.556817	169.4540333	-1.294659198	1.78E-15	7.79E-15
Cladopus_023030	root	shoot	124.070168	29.33366975	76.7019189	2.099594816	2.06E-15	8.96E-15

Cladopus_023502	root	shoot	226.3453455	90.74133289	158.5433392	1.320390793	2.09E-15	9.10E-15
Cladopus_006451	root	shoot	373.1393202	180.8605649	276.9999425	1.046785341	2.13E-15	9.25E-15
Cladopus_000272	root	shoot	40.60787278	140.7887147	90.69829376	-1.805323617	2.19E-15	9.54E-15
Cladopus_026682	root	shoot	113.658937	0	56.82946849	9.477124153	2.60E-15	1.12E-14
Cladopus_008446	root	shoot	83.73702771	13.96465638	48.85084205	2.602604804	2.72E-15	1.18E-14
Cladopus_002703	root	shoot	170.2311913	356.9611519	263.5961716	-1.065910418	2.74E-15	1.19E-14
Cladopus_026734	root	shoot	354.9238117	161.205217	258.0645143	1.133602997	2.84E-15	1.23E-14
Cladopus_011083	root	shoot	1007.527888	254.0713519	630.7996199	1.985773965	2.91E-15	1.26E-14
Cladopus_006562	root	shoot	191.5194193	73.41321214	132.4663157	1.395834816	2.95E-15	1.27E-14
Cladopus_013496	root	shoot	28.88437992	123.3378168	76.11109838	-2.089594454	2.96E-15	1.28E-14
Cladopus_004934	root	shoot	194.9135572	409.7412422	302.3273997	-1.072117237	3.16E-15	1.36E-14
Cladopus_019222	root	shoot	81.69180068	13.07464988	47.38322528	2.637478165	3.25E-15	1.40E-14
Cladopus_015133	root	shoot	161.2757276	55.08577372	108.1807507	1.549828083	3.50E-15	1.51E-14
Cladopus_011303	root	shoot	110.5943757	0	55.29718787	9.436483423	3.90E-15	1.67E-14
Cladopus_012391	root	shoot	49.3756422	167.830246	108.6029441	-1.754914702	4.13E-15	1.77E-14
Cladopus_006855	root	shoot	61.60552083	4.165848732	32.88568478	3.925585696	4.54E-15	1.94E-14
Cladopus_012990	root	shoot	231.5343606	462.356219	346.9452898	-1.002713271	4.56E-15	1.95E-14
Cladopus_009643	root	shoot	295.3660088	114.9808105	205.1734096	1.364592357	4.62E-15	1.97E-14
Cladopus_022972	root	shoot	109.4513583	22.93776951	66.19456389	2.246338784	4.83E-15	2.06E-14
Cladopus_014401	root	shoot	106.9482818	0	53.47414091	9.388724313	5.10E-15	2.17E-14
Cladopus_013311	root	shoot	120.9212931	305.1516623	213.0364777	-1.327458623	6.01E-15	2.56E-14
Cladopus_002065	root	shoot	330.4532873	164.8137977	247.6335425	1.009616735	6.07E-15	2.58E-14
Cladopus_014042	root	shoot	273.1458487	126.4080687	199.7769587	1.114793352	6.21E-15	2.64E-14
Cladopus_021248	root	shoot	108.305985	25.17248266	66.73923385	2.095359134	6.26E-15	2.66E-14
Cladopus_000832	root	shoot	331.1292902	155.5009951	243.3151426	1.099112925	6.46E-15	2.74E-14
Cladopus_022370	root	shoot	12.77179991	81.62109262	47.19644626	-2.659577311	7.20E-15	3.04E-14
Cladopus_026686	root	shoot	103.0975261	0	51.54876305	9.335827014	7.23E-15	3.05E-14
Cladopus_015868	root	shoot	111.5295184	254.3852302	182.9573743	-1.188505024	7.31E-15	3.08E-14
Cladopus_009767	root	shoot	87.12894011	228.6725673	157.9007537	-1.395542766	7.60E-15	3.21E-14
Cladopus_003233	root	shoot	121.2919823	280.0711728	200.6815775	-1.200259454	7.77E-15	3.28E-14

Cladopus_006280	root	shoot	126.5306497	277.4460675	201.9883586	-1.137486916	8.49E-15	3.57E-14
Cladopus_005418	root	shoot	187.5532695	68.84417352	128.1987215	1.452910475	8.60E-15	3.62E-14
Cladopus_021715	root	shoot	20.36014698	101.6134671	60.98680703	-2.318987041	8.86E-15	3.72E-14
Cladopus_001462	root	shoot	226.4889626	98.85108082	162.6700217	1.190764666	9.73E-15	4.08E-14
Cladopus_001687	root	shoot	72.93789982	186.3927263	129.6653131	-1.352810072	9.77E-15	4.09E-14
Cladopus_008497	root	shoot	155.7898036	47.85552189	101.8226627	1.710632259	1.06E-14	4.44E-14
Cladopus_017619	root	shoot	59.75727933	3.870782874	31.8140311	3.992142286	1.21E-14	5.07E-14
Cladopus_023673	root	shoot	99.64643232	24.97954673	62.31298953	2.01050174	1.22E-14	5.11E-14
Cladopus_016101	root	shoot	81.43353274	14.66824	48.05088637	2.483019735	1.27E-14	5.31E-14
Cladopus_016492	root	shoot	175.2664391	65.22878715	120.2476132	1.428567531	1.38E-14	5.75E-14
Cladopus_014803	root	shoot	58.99386134	5.421227172	32.20754425	3.438252321	1.42E-14	5.90E-14
Cladopus_013016	root	shoot	72.47373194	1.177745807	36.82573887	5.982145662	1.43E-14	5.97E-14
Cladopus_025954	root	shoot	142.5732772	48.35817679	95.46572698	1.566476125	1.46E-14	6.05E-14
Cladopus_008624	root	shoot	96.04465974	0	48.02232987	9.23568175	1.52E-14	6.30E-14
Cladopus_013789	root	shoot	12.98562035	78.67265905	45.8291397	-2.59809488	1.64E-14	6.81E-14
Cladopus_014264	root	shoot	103.1605456	240.0762871	171.6184164	-1.222524869	1.65E-14	6.85E-14
Cladopus_023485	root	shoot	88.67644508	0.811041416	44.74374325	6.693493549	1.82E-14	7.54E-14
Cladopus_017689	root	shoot	256.6166497	112.9018816	184.7592656	1.181184375	1.84E-14	7.61E-14
Cladopus_009091	root	shoot	174.5738391	62.62189444	118.5978668	1.471201855	1.84E-14	7.62E-14
Cladopus_016209	root	shoot	94.62356013	0	47.31178006	9.210855988	1.99E-14	8.22E-14
Cladopus_014009	root	shoot	39.57789354	139.2316101	89.40475182	-1.812440682	2.04E-14	8.42E-14
Cladopus_008565	root	shoot	65.66848848	8.026787425	36.84763795	3.034716451	2.28E-14	9.36E-14
Cladopus_010002	root	shoot	30.76852733	115.4628182	73.11567276	-1.903521589	2.35E-14	9.64E-14
Cladopus_020490	root	shoot	308.8914299	150.3458201	229.618625	1.040309463	2.40E-14	9.83E-14
Cladopus_006549	root	shoot	123.6181316	270.7369719	197.1775517	-1.136105108	2.60E-14	1.06E-13
Cladopus_007648	root	shoot	215.1225369	89.73269391	152.4276154	1.272973636	2.78E-14	1.14E-13
Cladopus_004425	root	shoot	138.5428698	297.4755937	218.0092318	-1.106215692	2.91E-14	1.19E-13
Cladopus_001664	root	shoot	77.98875094	196.8245189	137.4066349	-1.334040006	3.07E-14	1.25E-13
Cladopus_016808	root	shoot	90.18309647	0	45.09154823	9.143815574	3.12E-14	1.27E-13
Cladopus_015865	root	shoot	111.2700123	244.7345086	178.0022605	-1.136083091	3.12E-14	1.27E-13

Cladopus_001890	root	shoot	40.24089969	140.7949371	90.5179184	-1.798551999	3.18E-14	1.29E-13
Cladopus_016990	root	shoot	352.4196363	158.0303985	255.2250174	1.155513634	3.37E-14	1.37E-13
Cladopus_001004	root	shoot	68.15039291	0.946991925	34.54869242	5.9139462	3.52E-14	1.43E-13
Cladopus_019464	root	shoot	96.88706684	229.3571288	163.1220978	-1.245888023	3.92E-14	1.58E-13
Cladopus_008268	root	shoot	28.89576816	110.0762251	69.48599665	-1.93585587	3.98E-14	1.61E-13
Cladopus_015412	root	shoot	392.9850323	189.1798986	291.0824655	1.049548256	4.11E-14	1.66E-13
Cladopus_007144	root	shoot	326.9007943	135.9774569	231.4391256	1.279109179	4.28E-14	1.73E-13
Cladopus_004489	root	shoot	222.275693	94.4914846	158.3835888	1.242695647	5.23E-14	2.11E-13
Cladopus_005197	root	shoot	5.349358028	63.73118557	34.5402718	-3.576112483	5.27E-14	2.12E-13
Cladopus_007552	root	shoot	429.1273003	210.6190306	319.8731654	1.027625572	5.49E-14	2.21E-13
Cladopus_017363	root	shoot	42.28179367	137.3814383	89.83161599	-1.695845681	5.69E-14	2.28E-13
Cladopus_008542	root	shoot	52.05428147	2.059759528	27.0570205	4.529608217	5.87E-14	2.35E-13
Cladopus_013205	root	shoot	228.4902347	104.3399511	166.4150929	1.132311289	6.17E-14	2.47E-13
Cladopus_005232	root	shoot	3.574781085	62.8684866	33.22163384	-4.186387846	6.18E-14	2.48E-13
Cladopus_018657	root	shoot	315.1639213	150.4129404	232.7884308	1.056774577	6.27E-14	2.51E-13
Cladopus_017800	root	shoot	119.5962723	35.25236929	77.4243208	1.752858785	6.61E-14	2.64E-13
Cladopus_015157	root	shoot	86.13090004	236.1124069	161.1216535	-1.464152864	6.70E-14	2.68E-13
Cladopus_011573	root	shoot	111.7656269	31.84908827	71.80735758	1.803098928	6.72E-14	2.69E-13
Cladopus_017547	root	shoot	93.68099596	19.81623369	56.74861483	2.23827442	6.79E-14	2.71E-13
Cladopus_000811	root	shoot	189.5923108	71.27648424	130.4343975	1.406200412	6.88E-14	2.75E-13
Cladopus_019070	root	shoot	66.14689125	8.315192954	37.2310421	2.995774703	7.40E-14	2.95E-13
Cladopus_009471	root	shoot	76.83743611	13.63310503	45.23527057	2.491070439	7.56E-14	3.01E-13
Cladopus_009085	root	shoot	6.124164268	62.28434899	34.20425663	-3.35122804	7.65E-14	3.05E-13
Cladopus_021405	root	shoot	141.1780595	299.774098	220.4760787	-1.084942041	8.25E-14	3.28E-13
Cladopus_010304	root	shoot	76.45771047	13.74308045	45.10039546	2.48100903	8.58E-14	3.41E-13
Cladopus_001499	root	shoot	94.84765928	218.0277106	156.4376849	-1.204821456	8.84E-14	3.51E-13
Cladopus_005399	root	shoot	104.7070632	29.6302134	67.16863828	1.811577397	9.80E-14	3.88E-13
Cladopus_017645	root	shoot	233.3282592	95.53808687	164.4331731	1.283799366	1.11E-13	4.39E-13
Cladopus_008717	root	shoot	21.88190264	96.49692167	59.18941215	-2.12993685	1.22E-13	4.79E-13
Cladopus_009970	root	shoot	94.69462874	221.5875893	158.141109	-1.228742724	1.25E-13	4.91E-13

Cladopus_020767	root	shoot	65.60386044	9.629555499	37.61670797	2.787820246	1.25E-13	4.92E-13
Cladopus_006556	root	shoot	117.9037859	29.31649901	73.61014245	2.015477098	1.33E-13	5.21E-13
Cladopus_015617	root	shoot	49.63985173	148.5151084	99.07748008	-1.580432828	1.37E-13	5.37E-13
Cladopus_017917	root	shoot	73.3488279	192.0305732	132.6897006	-1.386589704	1.37E-13	5.40E-13
Cladopus_010211	root	shoot	137.4200329	296.9460692	217.1830511	-1.117671619	1.43E-13	5.61E-13
Cladopus_011145	root	shoot	217.8448805	96.13340113	156.9891408	1.177346177	1.53E-13	6.01E-13
Cladopus_017798	root	shoot	117.0742534	29.44778788	73.26102066	2.014891617	1.67E-13	6.53E-13
Cladopus_019912	root	shoot	268.4447694	107.3265762	187.8856728	1.330985392	1.78E-13	6.94E-13
Cladopus_000659	root	shoot	197.9149768	84.84275968	141.3788683	1.218779449	1.78E-13	6.95E-13
Cladopus_002490	root	shoot	298.9789084	140.685107	219.8320077	1.084626195	1.81E-13	7.07E-13
Cladopus_016007	root	shoot	56.51170862	5.82974493	31.17072678	3.250210904	1.94E-13	7.56E-13
Cladopus_012734	root	shoot	32.11942364	112.5836509	72.35153725	-1.814449373	2.03E-13	7.92E-13
Cladopus_016483	root	shoot	78.83858603	194.6416106	136.7400983	-1.300620133	2.05E-13	7.99E-13
Cladopus_009366	root	shoot	132.3643562	46.40387637	89.38411628	1.510381993	2.07E-13	8.07E-13
Cladopus_014274	root	shoot	54.94042919	6.741583818	30.84100651	3.009950374	2.29E-13	8.87E-13
Cladopus_011150	root	shoot	58.47397982	178.6160075	118.5449937	-1.611161813	2.37E-13	9.20E-13
Cladopus_019012	root	shoot	182.0422213	71.17968415	126.6109527	1.364463007	2.39E-13	9.27E-13
Cladopus_008957	root	shoot	293.5830059	146.7097866	220.1463962	1.001700271	2.40E-13	9.29E-13
Cladopus_004072	root	shoot	42.66894729	133.0604696	87.86470847	-1.647981301	2.46E-13	9.52E-13
Cladopus_000791	root	shoot	83.92397876	16.52892221	50.22645048	2.325774022	2.55E-13	9.85E-13
Cladopus_013721	root	shoot	63.02736364	8.902140817	35.96475223	2.80172441	2.66E-13	1.03E-12
Cladopus_006127	root	shoot	175.7216926	67.63260517	121.6771489	1.367011824	2.67E-13	1.03E-12
Cladopus_015084	root	shoot	72.83427094	0.976817093	36.90554401	6.401914422	2.87E-13	1.11E-12
Cladopus_007359	root	shoot	21.70943477	91.94638624	56.82791051	-2.078162331	3.40E-13	1.31E-12
Cladopus_005329	root	shoot	5.074126591	63.24799076	34.16105867	-3.672620129	3.64E-13	1.40E-12
Cladopus_007716	root	shoot	124.1549766	263.4518143	193.8033954	-1.083089858	3.87E-13	1.49E-12
Cladopus_015441	root	shoot	17.35910424	83.99656522	50.67783473	-2.263318725	3.96E-13	1.52E-12
Cladopus_002375	root	shoot	77.0661166	13.31073163	45.18842411	2.50933352	4.17E-13	1.60E-12
Cladopus_015804	root	shoot	285.3820417	139.8595598	212.6208007	1.031972539	4.33E-13	1.66E-12
Cladopus_013750	root	shoot	36.8042891	131.2286538	84.01647142	-1.836664929	4.41E-13	1.69E-12

Cladopus_007703	root	shoot	334.0059065	165.8344268	249.9201667	1.015634732	4.74E-13	1.81E-12
Cladopus_022612	root	shoot	78.43769244	10.18001787	44.30885515	2.919829207	4.75E-13	1.81E-12
Cladopus_020061	root	shoot	142.3807509	51.17450994	96.7776304	1.482224399	4.79E-13	1.83E-12
Cladopus_010637	root	shoot	85.97378714	22.0357748	54.00478097	1.965296062	4.84E-13	1.84E-12
Cladopus_007374	root	shoot	128.8278327	45.22613057	87.02698165	1.508224032	5.12E-13	1.95E-12
Cladopus_001531	root	shoot	30.9949782	115.0127797	73.00387895	-1.883264199	5.59E-13	2.12E-12
Cladopus_012927	root	shoot	90.80574155	240.8837067	165.8447241	-1.410515695	5.67E-13	2.15E-12
Cladopus_018514	root	shoot	51.17894128	4.996204905	28.08757309	3.327507055	5.91E-13	2.24E-12
Cladopus_005494	root	shoot	62.66831105	166.463707	114.566009	-1.412588056	5.94E-13	2.25E-12
Cladopus_002185	root	shoot	62.82320082	7.683247872	35.25322434	3.067485007	6.06E-13	2.29E-12
Cladopus_009355	root	shoot	68.81166843	0	34.40583422	8.757200375	6.06E-13	2.29E-12
Cladopus_015432	root	shoot	154.4975937	57.3936032	105.9455984	1.415682506	6.18E-13	2.34E-12
Cladopus_012684	root	shoot	266.0571985	124.5786875	195.317943	1.083329587	6.34E-13	2.40E-12
Cladopus_015074	root	shoot	295.3019848	146.1932921	220.7476384	1.015681348	6.44E-13	2.44E-12
Cladopus_022748	root	shoot	106.0440974	250.403951	178.2240242	-1.235574186	6.73E-13	2.54E-12
Cladopus_009712	root	shoot	111.0809538	34.10977652	72.59536515	1.68752077	7.13E-13	2.69E-12
Cladopus_000252	root	shoot	64.55072783	10.89092804	37.72082793	2.549508131	7.49E-13	2.82E-12
Cladopus_026497	root	shoot	171.8048224	69.4941009	120.6494617	1.306319604	7.61E-13	2.87E-12
Cladopus_026192	root	shoot	67.00032027	0	33.50016013	8.71839179	7.74E-13	2.91E-12
Cladopus_011673	root	shoot	2.91090961	60.83285077	31.87188019	-4.32456041	7.82E-13	2.94E-12
Cladopus_000827	root	shoot	103.2690065	28.72888492	65.99894573	1.851973903	8.39E-13	3.15E-12
Cladopus_016542	root	shoot	52.95203181	6.605633308	29.77883256	3.009722243	8.61E-13	3.23E-12
Cladopus_002200	root	shoot	164.0160221	328.5526457	246.2843339	-1.002019945	8.88E-13	3.33E-12
Cladopus_027275	root	shoot	75.61493056	184.7932894	130.20411	-1.289761936	8.95E-13	3.36E-12
Cladopus_014514	root	shoot	54.75372236	6.848375389	30.80104888	2.956703839	9.35E-13	3.50E-12
Cladopus_017762	root	shoot	70.70361423	174.3651996	122.5344069	-1.307089865	9.36E-13	3.51E-12
Cladopus_024468	root	shoot	118.2183845	38.71396817	78.46617634	1.603081875	9.79E-13	3.66E-12
Cladopus_020772	root	shoot	47.15262275	2.420469819	24.78654628	4.353202704	9.83E-13	3.68E-12
Cladopus_020399	root	shoot	169.9424641	69.08558314	119.5140236	1.300487333	9.93E-13	3.71E-12
Cladopus_021768	root	shoot	47.15720409	2.124737732	24.64097091	4.382132415	1.01E-12	3.77E-12

Cladopus_004493	root	shoot	79.62082027	193.8705292	136.7456747	-1.282264245	1.02E-12	3.83E-12
Cladopus_013606	root	shoot	268.7458723	126.187159	197.4665156	1.094449404	1.03E-12	3.84E-12
Cladopus_019072	root	shoot	57.01190452	1.076948335	29.04442643	5.651075926	1.07E-12	3.97E-12
Cladopus_011990	root	shoot	162.2104934	64.51639919	113.3634463	1.340588337	1.17E-12	4.35E-12
Cladopus_017389	root	shoot	55.33365058	163.3947876	109.3642191	-1.569925754	1.21E-12	4.50E-12
Cladopus_003828	root	shoot	62.53992029	0	31.26996014	8.616604004	1.22E-12	4.55E-12
Cladopus_012872	root	shoot	50.38085957	5.226958786	27.80390918	3.287262093	1.28E-12	4.76E-12
Cladopus_021375	root	shoot	97.68131041	24.60803341	61.14467191	1.98921964	1.39E-12	5.16E-12
Cladopus_016721	root	shoot	63.24440113	0	31.62220056	8.629013195	1.52E-12	5.60E-12
Cladopus_001441	root	shoot	67.83839025	13.16945325	40.50392175	2.384292108	1.61E-12	5.93E-12
Cladopus_001321	root	shoot	239.9716839	115.2971898	177.6344369	1.06301418	1.62E-12	5.96E-12
Cladopus_017584	root	shoot	5.29983426	59.1229985	32.21141638	-3.466330102	1.62E-12	5.96E-12
Cladopus_026868	root	shoot	76.6355029	17.22466033	46.93008161	2.161113416	1.68E-12	6.20E-12
Cladopus_015266	root	shoot	48.65048184	2.6632119	25.65684687	4.112557875	1.72E-12	6.33E-12
Cladopus_008828	root	shoot	61.65405717	0	30.82702859	8.596670803	1.76E-12	6.48E-12
Cladopus_006896	root	shoot	1.912625319	59.88704401	30.89983466	-4.971854997	1.94E-12	7.13E-12
Cladopus_010449	root	shoot	157.4241066	319.7590005	238.5915535	-1.01727216	2.13E-12	7.80E-12
Cladopus_008651	root	shoot	70.77715667	177.4560967	124.1166267	-1.334908344	2.17E-12	7.93E-12
Cladopus_011411	root	shoot	55.81490406	150.0732529	102.9440785	-1.41956463	2.29E-12	8.38E-12
Cladopus_013493	root	shoot	140.7112892	302.431982	221.5716356	-1.109751793	2.40E-12	8.75E-12
Cladopus_019547	root	shoot	79.63654564	19.81623369	49.72638967	2.000380257	2.52E-12	9.18E-12
Cladopus_002537	root	shoot	88.74664837	200.8097125	144.7781805	-1.17386221	2.64E-12	9.62E-12
Cladopus_013325	root	shoot	24.63321904	94.95099368	59.79210636	-1.950928378	2.77E-12	1.01E-11
Cladopus_001266	root	shoot	298.1178177	148.438142	223.2779799	1.008791251	2.77E-12	1.01E-11
Cladopus_000117	root	shoot	201.3418012	414.4321595	307.8869804	-1.048326331	2.84E-12	1.03E-11
Cladopus_012092	root	shoot	83.9373524	192.6786512	138.3080018	-1.195688425	2.97E-12	1.08E-11
Cladopus_013212	root	shoot	48.3150816	3.834963607	26.0750226	3.692396298	3.09E-12	1.12E-11
Cladopus_002882	root	shoot	30.42297049	111.8269319	71.1249512	-1.895012816	3.10E-12	1.12E-11
Cladopus_025542	root	shoot	103.4703187	221.9648041	162.7175614	-1.105219923	3.44E-12	1.25E-11
Cladopus_002129	root	shoot	99.82657647	27.16726398	63.49692023	1.861292378	3.55E-12	1.28E-11

Cladopus_001085	root	shoot	30.67108831	103.0674019	66.86924512	-1.751712522	3.65E-12	1.32E-11
Cladopus_006370	root	shoot	120.9465475	258.5697255	189.7581365	-1.102549168	4.20E-12	1.51E-11
Cladopus_010330	root	shoot	108.0702534	234.1537355	171.1119945	-1.119483198	4.28E-12	1.54E-11
Cladopus_017437	root	shoot	94.41853199	27.12012275	60.76932737	1.808561675	4.31E-12	1.55E-11
Cladopus_018291	root	shoot	51.04398784	1.076948335	26.06046809	5.485643266	4.50E-12	1.61E-11
Cladopus_026234	root	shoot	167.0579249	59.94484099	113.5013829	1.457781878	4.65E-12	1.67E-11
Cladopus_000598	root	shoot	120.1295234	42.6405513	81.38503735	1.491866471	4.70E-12	1.68E-11
Cladopus_002567	root	shoot	282.0247947	571.6645505	426.8446726	-1.021454639	4.75E-12	1.70E-11
Cladopus_022910	root	shoot	237.7425967	114.2200942	175.9813455	1.056880073	4.86E-12	1.74E-11
Cladopus_000571	root	shoot	35.8936641	120.0164565	77.9550603	-1.74533163	4.91E-12	1.75E-11
Cladopus_023692	root	shoot	90.35630755	206.8985568	148.6274322	-1.194609153	5.23E-12	1.87E-11
Cladopus_017651	root	shoot	42.86914987	2.254694142	22.56192201	4.230802964	5.46E-12	1.95E-11
Cladopus_012764	root	shoot	222.006159	95.79267182	158.8994154	1.213365569	5.55E-12	1.98E-11
Cladopus_010163	root	shoot	103.0967829	221.66693	162.3818564	-1.102816445	5.98E-12	2.13E-11
Cladopus_013858	root	shoot	87.14676892	24.18967147	55.66822019	1.847509249	6.30E-12	2.24E-11
Cladopus_008217	root	shoot	45.66528658	131.3671219	88.51620423	-1.521460874	6.34E-12	2.25E-11
Cladopus_024725	root	shoot	36.16976085	112.879383	74.5245719	-1.65143177	6.59E-12	2.34E-11
Cladopus_001822	root	shoot	67.14468062	10.8922605	39.01847056	2.646402737	6.98E-12	2.47E-11
Cladopus_017508	root	shoot	221.8242777	107.6217874	164.7230326	1.044046626	7.35E-12	2.60E-11
Cladopus_007358	root	shoot	240.9663743	115.3350058	178.1506901	1.057983794	7.90E-12	2.79E-11
Cladopus_012580	root	shoot	61.91987522	165.4757132	113.6977942	-1.427500846	8.42E-12	2.97E-11
Cladopus_021774	root	shoot	37.20704598	113.7255774	75.46631169	-1.619648863	8.55E-12	3.01E-11
Cladopus_021608	root	shoot	125.7755291	39.69278395	82.73415654	1.669103085	8.72E-12	3.07E-11
Cladopus_002479	root	shoot	12.18456581	66.89520098	39.53988339	-2.454600066	8.87E-12	3.12E-11
Cladopus_026687	root	shoot	52.55596378	0	26.27798189	8.366090476	9.19E-12	3.23E-11
Cladopus_014304	root	shoot	114.6947357	40.22459779	77.45966675	1.520512134	9.38E-12	3.30E-11
Cladopus_010466	root	shoot	58.70736377	0.911838888	29.80960133	6.089287289	9.43E-12	3.31E-11
Cladopus_001420	root	shoot	57.55951668	7.138113376	32.34881503	3.049274239	9.83E-12	3.45E-11
Cladopus_014495	root	shoot	107.4415447	230.8961058	169.1688252	-1.096800084	1.05E-11	3.67E-11
Cladopus_008091	root	shoot	54.3531951	8.399485916	31.37634051	2.674897078	1.10E-11	3.84E-11

Cladopus_023024	root	shoot	151.2925102	306.9919899	229.1422501	-1.016950237	1.13E-11	3.93E-11
Cladopus_017817	root	shoot	124.7183142	250.1095514	187.4139328	-1.004372311	1.27E-11	4.41E-11
Cladopus_013126	root	shoot	215.5704845	97.22766553	156.399075	1.140891572	1.27E-11	4.42E-11
Cladopus_004734	root	shoot	44.48983096	132.0250421	88.25743651	-1.560132184	1.27E-11	4.43E-11
Cladopus_015353	root	shoot	142.9994316	54.34851486	98.67397324	1.386345342	1.32E-11	4.59E-11
Cladopus_003104	root	shoot	24.95797424	95.28483438	60.12140431	-1.941742134	1.33E-11	4.63E-11
Cladopus_016071	root	shoot	266.7556181	121.985491	194.3705546	1.133421062	1.36E-11	4.74E-11
Cladopus_020367	root	shoot	56.54179506	144.3929245	100.4673598	-1.354233474	1.44E-11	5.00E-11
Cladopus_014643	root	shoot	17.22117797	78.02672708	47.62395253	-2.1814649	1.52E-11	5.28E-11
Cladopus_015681	root	shoot	69.32795603	166.1989852	117.7634706	-1.262996707	1.56E-11	5.41E-11
Cladopus_014746	root	shoot	2.362676354	51.54868622	26.95568129	-4.489851453	1.62E-11	5.62E-11
Cladopus_010588	root	shoot	48.38280457	4.148677993	26.26574128	3.442835679	1.66E-11	5.74E-11
Cladopus_000705	root	shoot	87.13413848	191.6263454	139.380242	-1.136641268	1.95E-11	6.70E-11
Cladopus_019836	root	shoot	74.84086753	17.10388187	45.9723747	2.124797709	1.97E-11	6.79E-11
Cladopus_026683	root	shoot	48.74420889	0	24.37210445	8.257479023	2.03E-11	6.97E-11
Cladopus_009532	root	shoot	50.63145123	137.2725027	93.95197698	-1.437919189	2.03E-11	6.98E-11
Cladopus_000600	root	shoot	199.7264471	85.1603261	142.4433866	1.244757423	2.04E-11	7.02E-11
Cladopus_025904	root	shoot	84.44893654	184.970906	134.7099213	-1.131543156	2.06E-11	7.08E-11
Cladopus_002526	root	shoot	3.735982823	54.77368442	29.25483362	-3.845417448	2.20E-11	7.57E-11
Cladopus_009946	root	shoot	41.45362552	125.0898561	83.2717408	-1.582464793	2.34E-11	8.02E-11
Cladopus_011011	root	shoot	46.22936731	5.463040539	25.84620393	3.035101728	2.38E-11	8.17E-11
Cladopus_006876	root	shoot	247.1351082	122.5764343	184.8557712	1.006197104	2.45E-11	8.38E-11
Cladopus_006008	root	shoot	214.3622139	104.4765679	159.4193909	1.039852712	2.48E-11	8.47E-11
Cladopus_016995	root	shoot	28.68504266	106.0271594	67.35610105	-1.893215683	2.73E-11	9.34E-11
Cladopus_015451	root	shoot	4.97433585	50.12686587	27.55060086	-3.340882432	2.87E-11	9.79E-11
Cladopus_023474	root	shoot	195.6093784	90.50140106	143.0553897	1.109941666	3.20E-11	1.09E-10
Cladopus_009260	root	shoot	69.92880799	165.3954157	117.6621118	-1.248816585	3.32E-11	1.13E-10
Cladopus_000168	root	shoot	102.1396029	27.14409915	64.64185102	1.893025074	3.71E-11	1.26E-10
Cladopus_008397	root	shoot	235.2519	112.0055074	173.6287037	1.061092898	3.97E-11	1.34E-10
Cladopus_022979	root	shoot	103.6024255	29.89545409	66.74893977	1.775186302	3.98E-11	1.34E-10

Cladopus_009980	root	shoot	101.0108244	213.0643693	157.0375969	-1.077042686	4.06E-11	1.37E-10
Cladopus_026046	root	shoot	43.63046036	0	21.81523018	8.095720621	4.21E-11	1.42E-10
Cladopus_010465	root	shoot	41.67140621	1.722880303	21.69714326	4.600579273	4.48E-11	1.51E-10
Cladopus_020744	root	shoot	43.65447904	0	21.82723952	8.096292246	4.64E-11	1.56E-10
Cladopus_010620	root	shoot	345.3093256	1087.936039	716.6226824	-1.65452235	4.71E-11	1.58E-10
Cladopus_009361	root	shoot	47.88681962	4.487555904	26.18718776	3.466539967	4.74E-11	1.60E-10
Cladopus_016964	root	shoot	123.2693577	253.6299061	188.4496319	-1.04606977	5.07E-11	1.70E-10
Cladopus_024363	root	shoot	207.6687705	102.5102792	155.0895249	1.021283122	5.12E-11	1.72E-10
Cladopus_006610	root	shoot	13.78518847	67.23792897	40.51155872	-2.283598657	5.40E-11	1.81E-10
Cladopus_015543	root	shoot	34.39431859	109.0272506	71.71078458	-1.66094791	5.50E-11	1.84E-10
Cladopus_007563	root	shoot	22.96945475	94.49110904	58.73028189	-2.035437729	5.50E-11	1.84E-10
Cladopus_018477	root	shoot	105.0736659	248.7400568	176.9068613	-1.237878532	5.51E-11	1.85E-10
Cladopus_026867	root	shoot	47.03933625	133.1990851	90.11921066	-1.495651604	6.38E-11	2.13E-10
Cladopus_005182	root	shoot	134.242562	51.99368947	93.11812574	1.361351186	6.38E-11	2.13E-10
Cladopus_008383	root	shoot	66.02691997	154.3255386	110.1762293	-1.223615098	7.16E-11	2.38E-10
Cladopus_014904	root	shoot	14.64777611	67.16843445	40.90810528	-2.205556349	7.77E-11	2.58E-10
Cladopus_006300	root	shoot	40.80584574	1.616088732	21.21096724	4.808719318	8.81E-11	2.92E-10
Cladopus_001940	root	shoot	21.78297722	100.7488338	61.2659055	-2.213043262	8.93E-11	2.95E-10
Cladopus_008541	root	shoot	91.57608856	196.8771353	144.2266119	-1.107598348	9.37E-11	3.10E-10
Cladopus_010662	root	shoot	72.11654263	0.236747981	36.17664531	7.860108806	9.90E-11	3.27E-10
Cladopus_015103	root	shoot	18.94858288	78.00023306	48.47440797	-2.047424989	1.00E-10	3.31E-10
Cladopus_017649	root	shoot	242.1547046	118.6918118	180.4232582	1.027492764	1.07E-10	3.51E-10
Cladopus_018703	root	shoot	39.5666274	0	19.7833137	7.956894974	1.18E-10	3.86E-10
Cladopus_021810	root	shoot	10.12188278	60.10795371	35.11491824	-2.553029548	1.19E-10	3.91E-10
Cladopus_001953	root	shoot	80.7895911	177.8552912	129.3224412	-1.144511591	1.43E-10	4.66E-10
Cladopus_009697	root	shoot	41.89178933	4.43841598	23.16510265	3.273772572	1.64E-10	5.35E-10
Cladopus_002571	root	shoot	40.84014313	3.474253316	22.15719822	3.488279976	1.69E-10	5.51E-10
Cladopus_018937	root	shoot	61.82739036	14.39500652	38.11119844	2.093126222	1.72E-10	5.60E-10
Cladopus_025221	root	shoot	166.3746161	72.25426019	119.3144381	1.197089241	1.78E-10	5.79E-10
Cladopus_004124	root	shoot	181.1130243	77.88626026	129.4996423	1.213623018	1.80E-10	5.84E-10

Cladopus_014524	root	shoot	12.02336407	62.44013515	37.23174961	-2.386035978	1.82E-10	5.90E-10
Cladopus_006844	root	shoot	111.2254362	256.8214593	184.0234478	-1.212476535	1.95E-10	6.31E-10
Cladopus_002893	root	shoot	81.14938689	176.0621048	128.6057459	-1.115040222	2.08E-10	6.72E-10
Cladopus_022204	root	shoot	1651.766116	280.3028094	966.0344627	2.557466099	2.14E-10	6.91E-10
Cladopus_009792	root	shoot	93.66774442	209.9898294	151.8287869	-1.160872266	2.16E-10	6.96E-10
Cladopus_019040	root	shoot	81.72250414	24.10152843	52.91201628	1.747133555	2.18E-10	7.02E-10
Cladopus_015792	root	shoot	38.04252001	2.498768681	20.27064435	4.033718209	2.24E-10	7.22E-10
Cladopus_020931	root	shoot	124.3627293	48.20438931	86.28355931	1.364753108	2.25E-10	7.24E-10
Cladopus_004525	root	shoot	42.74534045	4.853594066	23.79946726	3.138742744	2.42E-10	7.78E-10
Cladopus_009225	root	shoot	74.39898769	20.06696856	47.23297813	1.894804904	2.61E-10	8.36E-10
Cladopus_018740	root	shoot	208.2539036	86.14498869	147.1994461	1.286181604	2.61E-10	8.38E-10
Cladopus_018958	root	shoot	109.3613473	225.3622363	167.3617918	-1.051822046	2.94E-10	9.39E-10
Cladopus_006525	root	shoot	35.14436295	0	17.57218147	7.784436651	3.27E-10	1.04E-09
Cladopus_022722	root	shoot	156.0983385	65.417873	110.7581057	1.244355745	3.34E-10	1.06E-09
Cladopus_025884	root	shoot	171.021346	78.52871575	124.7750309	1.119269005	3.37E-10	1.07E-09
Cladopus_020546	root	shoot	79.54888634	171.2298242	125.3893553	-1.10510054	3.38E-10	1.07E-09
Cladopus_003747	root	shoot	58.75453581	12.87971526	35.81712553	2.170363185	3.40E-10	1.08E-09
Cladopus_011504	root	shoot	126.5811609	44.57487073	85.57801583	1.505699561	3.52E-10	1.12E-09
Cladopus_022617	root	shoot	50.36352568	136.5310992	93.44731243	-1.436930146	3.57E-10	1.13E-09
Cladopus_017764	root	shoot	70.72738869	16.98657989	43.85698429	2.04021007	3.70E-10	1.17E-09
Cladopus_020558	root	shoot	241.1518495	115.1532465	178.152548	1.070998119	3.90E-10	1.23E-09
Cladopus_018697	root	shoot	47.46697713	8.191896873	27.829437	2.523389245	3.98E-10	1.26E-09
Cladopus_006583	root	shoot	111.8379312	232.9922016	172.4150664	-1.065526283	4.03E-10	1.27E-09
Cladopus_024115	root	shoot	22.64853768	83.98990489	53.31922129	-1.895528619	4.12E-10	1.30E-09
Cladopus_000096	root	shoot	78.4581172	22.51593109	50.48702414	1.803009982	4.28E-10	1.35E-09
Cladopus_018759	root	shoot	94.78142272	30.97306865	62.87724568	1.606823649	4.54E-10	1.43E-09
Cladopus_008393	root	shoot	199.62777	89.7101953	144.6689826	1.159843106	4.68E-10	1.47E-09
Cladopus_023618	root	shoot	36.11639894	2.02460649	19.07050272	4.174253355	4.94E-10	1.55E-09
Cladopus_007419	root	shoot	34.17975495	0	17.08987747	7.746363849	4.95E-10	1.56E-09
Cladopus_000419	root	shoot	132.5405401	54.51495676	93.52774843	1.27970579	5.10E-10	1.60E-09

Cladopus_017876	root	shoot	64.21878885	156.6755551	110.447172	-1.296378152	5.11E-10	1.60E-09
Cladopus_010666	root	shoot	33.49421652	0	16.74710826	7.716484031	5.25E-10	1.65E-09
Cladopus_000203	root	shoot	64.73607035	152.7691002	108.7525853	-1.234984919	5.39E-10	1.69E-09
Cladopus_019827	root	shoot	70.11477161	155.9316359	113.0232037	-1.158275907	5.53E-10	1.73E-09
Cladopus_017900	root	shoot	3.499752227	43.04507365	23.27241294	-3.635538025	5.56E-10	1.74E-09
Cladopus_011450	root	shoot	38.08399469	111.6133488	74.84867173	-1.563770391	5.65E-10	1.77E-09
Cladopus_014418	root	shoot	62.0412108	145.2013029	103.6212569	-1.225828438	5.83E-10	1.82E-09
Cladopus_002012	root	shoot	53.30464383	130.2305232	91.76758352	-1.293655047	5.88E-10	1.84E-09
Cladopus_018678	root	shoot	82.78603757	25.49485606	54.14044682	1.702844819	6.03E-10	1.88E-09
Cladopus_010621	root	shoot	209.1260202	104.0022603	156.5641403	1.019060352	6.22E-10	1.94E-09
Cladopus_012247	root	shoot	40.49136925	111.76647	76.12891963	-1.454874428	6.84E-10	2.13E-09
Cladopus_022727	root	shoot	7.585995345	49.41662193	28.50130864	-2.701494987	6.84E-10	2.13E-09
Cladopus_015073	root	shoot	126.8762066	52.84373401	89.85997029	1.261251361	7.01E-10	2.18E-09
Cladopus_005028	root	shoot	283.5754076	135.5379268	209.5566672	1.072166279	7.13E-10	2.21E-09
Cladopus_012751	root	shoot	193.9838718	92.6611465	143.3225091	1.072744293	8.36E-10	2.58E-09
Cladopus_006341	root	shoot	70.32772673	19.12848836	44.72810755	1.876898214	8.64E-10	2.67E-09
Cladopus_020250	root	shoot	55.12564961	0.301726186	27.7136879	7.47377337	9.04E-10	2.79E-09
Cladopus_016028	root	shoot	43.84106376	5.923882073	24.88247292	2.87844326	9.13E-10	2.81E-09
Cladopus_011392	root	shoot	76.51057339	19.40838217	47.95947778	1.986238073	9.26E-10	2.85E-09
Cladopus_003184	root	shoot	163.9817312	74.88683536	119.4342833	1.134190967	9.89E-10	3.04E-09
Cladopus_023427	root	shoot	1059.167264	2378.421777	1718.794521	-1.167683469	1.05E-09	3.22E-09
Cladopus_016845	root	shoot	0	37.95206668	18.97603334	-7.492121043	1.19E-09	3.64E-09
Cladopus_025546	root	shoot	134.6175842	59.24311073	96.93034746	1.188248209	1.20E-09	3.67E-09
Cladopus_013291	root	shoot	42.83101435	117.9947432	80.41287877	-1.464889286	1.22E-09	3.71E-09
Cladopus_001751	root	shoot	73.42930342	164.139371	118.7843372	-1.167616308	1.22E-09	3.73E-09
Cladopus_006817	root	shoot	58.3682475	13.61978437	35.99401593	2.091997862	1.34E-09	4.07E-09
Cladopus_019076	root	shoot	153.6415687	65.93488839	109.7882286	1.222592978	1.36E-09	4.14E-09
Cladopus_012142	root	shoot	116.1715489	46.50534007	81.33844451	1.324096877	1.45E-09	4.41E-09
Cladopus_014734	root	shoot	104.6321564	39.06483428	71.84849535	1.413648226	1.47E-09	4.47E-09
Cladopus_025730	root	shoot	33.41844446	1.680400708	17.54942258	4.29021005	1.48E-09	4.48E-09

Cladopus_016555	root	shoot	134.2768594	56.33863454	95.30774697	1.253117078	1.57E-09	4.75E-09
Cladopus_015196	root	shoot	93.80864351	32.26878267	63.03871309	1.54766317	1.59E-09	4.81E-09
Cladopus_015988	root	shoot	156.4378316	70.91592125	113.6768764	1.144015614	1.66E-09	5.03E-09
Cladopus_019608	root	shoot	179.5457011	88.77452534	134.1601132	1.020845636	1.71E-09	5.17E-09
Cladopus_017259	root	shoot	129.1832914	49.75483361	89.46906251	1.372746645	1.73E-09	5.21E-09
Cladopus_026814	root	shoot	12.48530234	58.55336671	35.51933452	-2.221828198	1.75E-09	5.26E-09
Cladopus_014717	root	shoot	71.40029668	21.50329473	46.4517957	1.724094145	1.79E-09	5.38E-09
Cladopus_008035	root	shoot	85.01846394	23.1365542	54.07750907	1.888998405	1.91E-09	5.74E-09
Cladopus_019201	root	shoot	3.250025881	41.66373421	22.45688004	-3.754138755	1.94E-09	5.83E-09
Cladopus_015389	root	shoot	70.93526348	160.0499698	115.4926166	-1.174707713	2.10E-09	6.28E-09
Cladopus_008199	root	shoot	41.97882752	6.001514706	23.99017112	2.762824012	2.21E-09	6.62E-09
Cladopus_026845	root	shoot	50.76887851	0.473495963	25.62118724	6.481697528	2.27E-09	6.78E-09
Cladopus_001552	root	shoot	62.11611755	15.73786178	38.92698966	1.968643436	2.31E-09	6.90E-09
Cladopus_026987	root	shoot	75.51204488	160.3460754	117.9290602	-1.084918613	2.38E-09	7.10E-09
Cladopus_016017	root	shoot	159.6441532	70.71232761	115.1782404	1.170128574	2.39E-09	7.12E-09
Cladopus_006031	root	shoot	144.6831322	63.53891587	104.1110241	1.19504647	2.41E-09	7.18E-09
Cladopus_018679	root	shoot	75.82330434	173.8794892	124.8513967	-1.193780849	2.42E-09	7.22E-09
Cladopus_009878	root	shoot	113.3787579	38.73328293	76.0560204	1.543439459	2.50E-09	7.44E-09
Cladopus_003968	root	shoot	66.83044834	16.881787	41.85611767	2.007492074	2.64E-09	7.86E-09
Cladopus_022616	root	shoot	9.722098715	57.26616441	33.49413156	-2.544528162	2.87E-09	8.53E-09
Cladopus_021288	root	shoot	68.33858615	19.35924224	43.84891419	1.827689359	2.92E-09	8.67E-09
Cladopus_018247	root	shoot	67.95898262	15.00711791	41.48305027	2.215828672	2.93E-09	8.68E-09
Cladopus_016820	root	shoot	179.2033678	4.795942419	91.99965509	5.245013981	3.04E-09	9.02E-09
Cladopus_022970	root	shoot	98.57324128	37.08337362	67.82830745	1.407956766	3.06E-09	9.08E-09
Cladopus_002710	root	shoot	228.2098113	111.0602215	169.6350164	1.040222006	3.27E-09	9.66E-09
Cladopus_014402	root	shoot	46.89163027	0.301726186	23.59667823	7.239463867	3.28E-09	9.70E-09
Cladopus_020929	root	shoot	15.68506124	62.01378043	38.84942084	-1.982296493	3.35E-09	9.90E-09
Cladopus_022476	root	shoot	65.55458089	16.18153257	40.86805673	1.983771589	3.46E-09	1.02E-08
Cladopus_022752	root	shoot	63.42801302	17.39709633	40.41255468	1.878839198	3.47E-09	1.03E-08
Cladopus_001875	root	shoot	11.69700035	55.39482646	33.5459134	-2.22965786	3.52E-09	1.04E-08

Cladopus_015473	root	shoot	161.7020042	72.40257446	117.0522893	1.174259077	3.54E-09	1.05E-08
Cladopus_017948	root	shoot	93.54863846	34.46449271	64.00656558	1.436343044	3.62E-09	1.07E-08
Cladopus_017727	root	shoot	32.14332021	2.261354471	17.20233734	3.822476285	3.85E-09	1.13E-08
Cladopus_017438	root	shoot	36.01574289	3.231511235	19.62362706	3.512254702	4.06E-09	1.19E-08
Cladopus_027010	root	shoot	45.85360193	0.574293434	23.21394768	6.326564329	4.14E-09	1.22E-08
Cladopus_021513	root	shoot	4.212282155	40.01115998	22.11172107	-3.25652671	4.23E-09	1.24E-08
Cladopus_006020	root	shoot	46.05764265	116.9007695	81.47920605	-1.348462579	4.70E-09	1.37E-08
Cladopus_009201	root	shoot	83.97721856	27.75806193	55.86764024	1.588858636	4.77E-09	1.39E-08
Cladopus_003625	root	shoot	40.15695643	6.498841736	23.32789908	2.658152857	4.82E-09	1.41E-08
Cladopus_021995	root	shoot	34.26753635	3.705007197	18.98627177	3.207626637	4.92E-09	1.44E-08
Cladopus_011264	root	shoot	67.10332805	19.64579638	43.37456221	1.790817814	4.99E-09	1.45E-08
Cladopus_013153	root	shoot	134.7151453	58.73660575	96.72587553	1.196271264	5.00E-09	1.46E-08
Cladopus_019843	root	shoot	107.6657659	39.02102223	73.34339407	1.451744297	5.11E-09	1.49E-08
Cladopus_018072	root	shoot	32.62122806	91.83093566	62.22608186	-1.498712905	5.12E-09	1.49E-08
Cladopus_012245	root	shoot	6.161678696	47.0159858	26.58883225	-2.95138577	5.13E-09	1.50E-08
Cladopus_016891	root	shoot	189.8225957	92.62147715	141.2220364	1.0392889	5.21E-09	1.52E-08
Cladopus_024040	root	shoot	41.04591448	111.6458389	76.34587667	-1.448788798	5.29E-09	1.54E-08
Cladopus_010000	root	shoot	94.43450157	193.3003785	143.86744	-1.03697143	5.39E-09	1.57E-08
Cladopus_006273	root	shoot	60.71284675	16.00510115	38.35897395	1.938264865	5.45E-09	1.58E-08
Cladopus_013837	root	shoot	43.37838229	115.0867886	79.23258543	-1.409030927	5.66E-09	1.64E-08
Cladopus_020441	root	shoot	66.239254	19.12982082	42.68453741	1.813205906	5.70E-09	1.65E-08
Cladopus_016182	root	shoot	95.06606106	32.48303204	63.77454655	1.55027609	5.76E-09	1.67E-08
Cladopus_026815	root	shoot	18.57195218	71.32947425	44.95071321	-1.935271445	6.05E-09	1.75E-08
Cladopus_007052	root	shoot	186.1407221	82.42266346	134.2816928	1.175524296	6.09E-09	1.77E-08
Cladopus_002525	root	shoot	18.87206761	67.19692716	43.03449738	-1.840777013	6.20E-09	1.80E-08
Cladopus_024759	root	shoot	99.02180591	37.04622189	68.0340139	1.404312995	6.53E-09	1.89E-08
Cladopus_004552	root	shoot	31.09563426	1.586929794	16.34128203	4.419825135	6.68E-09	1.93E-08
Cladopus_023892	root	shoot	88.91181036	29.94725893	59.42953464	1.581436636	7.38E-09	2.13E-08
Cladopus_017487	root	shoot	35.70460554	98.21166385	66.9581347	-1.452166588	7.40E-09	2.13E-08
Cladopus_022022	root	shoot	50.52943087	11.58252346	31.05597716	2.134850456	7.56E-09	2.18E-08

Cladopus_023490	root	shoot	104.9925733	40.30289665	72.64773498	1.394474254	7.63E-09	2.20E-08
Cladopus_014505	root	shoot	41.84152235	107.3660173	74.60376984	-1.3567782	7.64E-09	2.20E-08
Cladopus_024954	root	shoot	51.76456685	11.38825507	31.57641096	2.206886797	8.37E-09	2.40E-08
Cladopus_012940	root	shoot	6.099402384	43.16318719	24.63129479	-2.821758823	8.57E-09	2.46E-08
Cladopus_021794	root	shoot	3.761487913	37.87310159	20.81729475	-3.319709389	8.67E-09	2.49E-08
Cladopus_000003	root	shoot	69.96632242	152.8673781	111.4168503	-1.136847828	8.70E-09	2.49E-08
Cladopus_006517	root	shoot	43.80367144	109.0682504	76.43596093	-1.315719935	9.02E-09	2.58E-08
Cladopus_000636	root	shoot	53.69316176	12.52418751	33.10867464	2.13171398	1.07E-08	3.05E-08
Cladopus_006492	root	shoot	12.40953027	55.31201129	33.86077078	-2.144928804	1.07E-08	3.06E-08
Cladopus_017901	root	shoot	13.05829748	140.1513608	76.60482914	-3.413843661	1.12E-08	3.18E-08
Cladopus_018619	root	shoot	10.39872274	49.89596666	30.1473447	-2.267174129	1.15E-08	3.27E-08
Cladopus_001694	root	shoot	151.3235841	69.83630799	110.579946	1.105881639	1.16E-08	3.29E-08
Cladopus_004363	root	shoot	128.080018	57.23715277	92.65858538	1.161447337	1.17E-08	3.32E-08
Cladopus_025803	root	shoot	83.04938178	28.22837404	55.63887791	1.541328491	1.18E-08	3.34E-08
Cladopus_015132	root	shoot	34.57953901	100.4541415	67.51684026	-1.537730199	1.19E-08	3.38E-08
Cladopus_023029	root	shoot	123.0786907	55.06260888	89.07064977	1.157352928	1.24E-08	3.51E-08
Cladopus_021851	root	shoot	11.13527134	52.84825032	31.99176083	-2.24834671	1.24E-08	3.51E-08
Cladopus_004570	root	shoot	130.66097	57.83527727	94.24812362	1.181800496	1.27E-08	3.59E-08
Cladopus_013378	root	shoot	64.68568127	18.03503552	41.36035839	1.836976318	1.27E-08	3.61E-08
Cladopus_024962	root	shoot	53.05244365	129.2760594	91.16425152	-1.276790608	1.27E-08	3.61E-08
Cladopus_020348	root	shoot	4.435760206	39.3552385	21.89549935	-3.108646676	1.31E-08	3.70E-08
Cladopus_020180	root	shoot	87.03657736	30.26149225	58.64903481	1.504504336	1.34E-08	3.78E-08
Cladopus_006198	root	shoot	149.4437738	68.11609261	108.7799332	1.134703006	1.36E-08	3.86E-08
Cladopus_006137	root	shoot	17.09588214	63.80763304	40.45175759	-1.894118991	1.37E-08	3.86E-08
Cladopus_012213	root	shoot	26.29364417	84.78577426	55.53970921	-1.675305431	1.39E-08	3.93E-08
Cladopus_025905	root	shoot	86.88429003	174.4893092	130.6867996	-1.000810227	1.42E-08	4.01E-08
Cladopus_006524	root	shoot	40.3212531	0.301726186	20.31148964	7.019099304	1.43E-08	4.02E-08
Cladopus_015131	root	shoot	9.333580786	60.00582574	34.66970326	-2.659566924	1.45E-08	4.08E-08
Cladopus_015836	root	shoot	41.31879419	7.949154792	24.63397449	2.404531123	1.45E-08	4.09E-08
Cladopus_003637	root	shoot	106.9157216	42.80566075	74.86069115	1.319276267	1.46E-08	4.12E-08

Cladopus_017252	root	shoot	116.4594108	45.66447349	81.06194215	1.351804907	1.47E-08	4.14E-08
Cladopus_009863	root	shoot	211.3825915	658.6140052	434.9982983	-1.639970261	1.51E-08	4.25E-08
Cladopus_001809	root	shoot	23.11059806	74.24090339	48.67575073	-1.690683165	1.56E-08	4.37E-08
Cladopus_022832	root	shoot	1.861615139	38.16735589	20.01448551	-4.322148119	1.60E-08	4.50E-08
Cladopus_001845	root	shoot	2.749707871	34.89299145	18.82134966	-3.70096779	1.68E-08	4.71E-08
Cladopus_010341	root	shoot	97.10608565	198.460732	147.7834088	-1.036501989	1.72E-08	4.81E-08
Cladopus_011229	root	shoot	25.02099376	77.31714936	51.16907156	-1.632122507	1.76E-08	4.94E-08
Cladopus_005599	root	shoot	87.41890542	30.28066168	58.84978355	1.545434441	1.79E-08	5.01E-08
Cladopus_000121	root	shoot	91.76267327	35.11775123	63.44021225	1.390874455	1.83E-08	5.12E-08
Cladopus_021736	root	shoot	41.89178933	105.240468	73.56612868	-1.321667817	1.87E-08	5.21E-08
Cladopus_013645	root	shoot	126.7500454	56.31865355	91.53434949	1.170896727	1.93E-08	5.39E-08
Cladopus_012251	root	shoot	49.2783253	119.0246937	84.15150948	-1.278308061	2.03E-08	5.64E-08
Cladopus_000743	root	shoot	124.2535252	51.79912846	88.02632682	1.260995565	2.04E-08	5.67E-08
Cladopus_026425	root	shoot	88.92592721	31.35242747	60.13917734	1.491410499	2.33E-08	6.44E-08
Cladopus_025446	root	shoot	42.43915727	6.978998028	24.70907765	2.626294487	2.34E-08	6.47E-08
Cladopus_025478	root	shoot	34.91618144	3.071729658	18.99395555	3.404601534	2.43E-08	6.71E-08
Cladopus_014856	root	shoot	43.72938579	107.5084828	75.61893431	-1.289334994	2.68E-08	7.38E-08
Cladopus_023106	root	shoot	32.44343564	3.811798769	18.1276172	3.041237512	2.72E-08	7.50E-08
Cladopus_025220	root	shoot	63.3116316	18.00720904	40.65942032	1.830248472	2.79E-08	7.69E-08
Cladopus_011761	root	shoot	54.96283935	125.8889093	90.42587432	-1.188240609	2.91E-08	8.01E-08
Cladopus_025453	root	shoot	90.75819912	34.80203816	62.78011864	1.384445847	2.92E-08	8.04E-08
Cladopus_001214	root	shoot	130.4036935	61.24773623	95.82571487	1.095226296	3.04E-08	8.37E-08
Cladopus_013244	root	shoot	46.88122945	10.88560017	28.88341481	2.129158976	3.16E-08	8.68E-08
Cladopus_020713	root	shoot	81.52453118	29.94245	55.73349059	1.449129872	3.17E-08	8.70E-08
Cladopus_008368	root	shoot	135.7043931	5.810430173	70.75741163	4.54857544	3.29E-08	9.01E-08
Cladopus_018213	root	shoot	8.247515093	45.10216826	26.67484167	-2.436346964	3.39E-08	9.28E-08
Cladopus_001537	root	shoot	18.09565285	64.74330299	41.41947792	-1.822882883	3.40E-08	9.29E-08
Cladopus_007361	root	shoot	109.5200752	48.07828298	78.79917907	1.189331726	3.52E-08	9.63E-08
Cladopus_026505	root	shoot	108.7325164	47.50398955	78.11825296	1.196295835	3.58E-08	9.78E-08
Cladopus_002432	root	shoot	17.1446627	73.12880202	45.13673236	-2.089961962	3.69E-08	1.01E-07

Cladopus_024671	root	shoot	28.45846967	79.96319052	54.2108301	-1.497699263	3.80E-08	1.04E-07
Cladopus_011879	root	shoot	18.95898369	66.0358312	42.49740744	-1.802062341	4.06E-08	1.11E-07
Cladopus_008373	root	shoot	42.13334447	108.6019337	75.36763909	-1.371826517	4.10E-08	1.12E-07
Cladopus_014558	root	shoot	63.69061402	136.8406728	100.2656434	-1.105973727	4.30E-08	1.17E-07
Cladopus_006386	root	shoot	140.5286712	68.57160627	104.5501387	1.043451341	4.39E-08	1.19E-07
Cladopus_014601	root	shoot	5.961354008	40.16494746	23.06315073	-2.726629712	4.67E-08	1.27E-07
Cladopus_024425	root	shoot	104.3956816	40.87933411	72.63750785	1.351036652	4.76E-08	1.29E-07
Cladopus_011384	root	shoot	30.01613124	90.9967294	60.50643032	-1.588856703	4.78E-08	1.29E-07
Cladopus_006075	root	shoot	72.85705149	21.70488967	47.28097058	1.743717437	4.80E-08	1.30E-07
Cladopus_003098	root	shoot	115.3402859	48.55525542	81.94777064	1.248259647	4.96E-08	1.34E-07
Cladopus_009623	root	shoot	62.67549483	16.65569476	39.6655948	1.896645018	5.04E-08	1.36E-07
Cladopus_014525	root	shoot	7.310763909	42.55026424	24.93051407	-2.546525703	5.16E-08	1.40E-07
Cladopus_022785	root	shoot	39.98139363	98.33710395	69.15924879	-1.295187497	5.58E-08	1.50E-07
Cladopus_021554	root	shoot	1.23760982	35.44863636	18.34312309	-4.938968124	5.82E-08	1.57E-07
Cladopus_003115	root	shoot	146.4456998	71.55837674	109.0020383	1.038517862	6.03E-08	1.62E-07
Cladopus_015046	root	shoot	62.39964229	134.4979829	98.44881262	-1.101685193	6.03E-08	1.62E-07
Cladopus_022951	root	shoot	45.91501293	113.2707319	79.59287243	-1.29438995	6.09E-08	1.64E-07
Cladopus_015245	root	shoot	105.9477679	44.79711289	75.3724404	1.251686878	6.58E-08	1.76E-07
Cladopus_011109	root	shoot	147.7901491	662.3109308	405.0505399	-2.166200768	6.69E-08	1.79E-07
Cladopus_001766	root	shoot	31.22006477	4.380098104	17.80008144	2.856972985	6.76E-08	1.81E-07
Cladopus_003077	root	shoot	43.83078505	9.127566828	26.47917594	2.312257058	7.04E-08	1.88E-07
Cladopus_003956	root	shoot	38.64485838	6.747577917	22.69621815	2.467140984	7.04E-08	1.88E-07
Cladopus_013222	root	shoot	106.5575343	46.67777608	76.61765518	1.195077533	7.40E-08	1.97E-07
Cladopus_012700	root	shoot	65.86955637	18.18948922	42.0295228	1.881460111	7.57E-08	2.02E-07
Cladopus_011463	root	shoot	80.11222387	30.52939786	55.32081087	1.38913386	7.70E-08	2.05E-07
Cladopus_023315	root	shoot	88.98213576	32.17612332	60.57912954	1.451516541	7.81E-08	2.08E-07
Cladopus_025222	root	shoot	30.20828473	81.5051231	55.85670392	-1.433243579	7.97E-08	2.12E-07
Cladopus_021653	root	shoot	17.57292468	61.22257271	39.39774869	-1.805065093	8.07E-08	2.14E-07
Cladopus_016799	root	shoot	90.07501242	34.92947695	62.50224468	1.369323593	8.25E-08	2.19E-07
Cladopus_010294	root	shoot	158.8250151	77.42527339	118.1251442	1.039669547	8.87E-08	2.35E-07

Cladopus_005839	root	shoot	13.92323685	55.23837603	34.58080644	-1.991396984	8.96E-08	2.37E-07
Cladopus_010510	root	shoot	17.42224587	64.98286122	41.20255354	-1.908800496	9.32E-08	2.46E-07
Cladopus_022923	root	shoot	58.92849009	16.34664202	37.63756606	1.813842493	9.75E-08	2.57E-07
Cladopus_019543	root	shoot	4.810782384	37.15567838	20.98323038	-2.912367442	9.98E-08	2.63E-07
Cladopus_011840	root	shoot	18.07175628	63.48244939	40.77710283	-1.816849467	1.00E-07	2.63E-07
Cladopus_002447	root	shoot	10.74823982	46.79307937	28.7706596	-2.127522108	1.02E-07	2.68E-07
Cladopus_015585	root	shoot	31.04388087	3.107548925	17.0757149	3.222692044	1.04E-07	2.73E-07
Cladopus_016643	root	shoot	97.75114089	38.33941829	68.04527959	1.350683266	1.06E-07	2.79E-07
Cladopus_015461	root	shoot	205.2820795	88.19172019	146.7368999	1.222993421	1.10E-07	2.90E-07
Cladopus_003655	root	shoot	5.348614822	36.42345476	20.88603479	-2.771707615	1.11E-07	2.91E-07
Cladopus_001428	root	shoot	47.81735954	10.23833574	29.02784764	2.194177422	1.11E-07	2.92E-07
Cladopus_009070	root	shoot	20.94738108	66.2797604	43.61357074	-1.657283266	1.14E-07	3.00E-07
Cladopus_010149	root	shoot	59.04982567	17.45408175	38.25195371	1.751290677	1.15E-07	3.03E-07
Cladopus_005662	root	shoot	2.736955327	33.27675739	18.00685636	-3.62037518	1.17E-07	3.06E-07
Cladopus_013190	root	shoot	76.21107906	28.09094574	52.1510124	1.457479792	1.18E-07	3.08E-07
Cladopus_010503	root	shoot	56.25467639	124.8079655	90.53132097	-1.151018405	1.20E-07	3.14E-07
Cladopus_004018	root	shoot	78.19650362	27.57245256	52.88447809	1.52230727	1.21E-07	3.16E-07
Cladopus_017301	root	shoot	9.186618005	45.93370959	27.5601638	-2.333340785	1.24E-07	3.23E-07
Cladopus_003597	root	shoot	34.98390441	4.517381071	19.75064274	3.05900599	1.24E-07	3.23E-07
Cladopus_010326	root	shoot	164.4639721	78.80276079	121.6333664	1.055342267	1.34E-07	3.49E-07
Cladopus_015255	root	shoot	109.1802157	49.29118183	79.23569876	1.147453944	1.38E-07	3.60E-07
Cladopus_017253	root	shoot	54.17676698	119.8808796	87.02882328	-1.142342885	1.38E-07	3.60E-07
Cladopus_027264	root	shoot	111.4423581	50.90727056	81.17481433	1.133067788	1.44E-07	3.75E-07
Cladopus_023659	root	shoot	75.32397375	24.71363981	50.01880678	1.617412161	1.45E-07	3.77E-07
Cladopus_007006	root	shoot	92.35584895	37.68749222	65.02167059	1.300611462	1.55E-07	4.04E-07
Cladopus_008222	root	shoot	35.99333273	6.806562022	21.39994738	2.399769137	1.66E-07	4.29E-07
Cladopus_010642	root	shoot	3.11272071	31.9936978	17.55320926	-3.378914964	1.68E-07	4.35E-07
Cladopus_014994	root	shoot	24.45988585	71.96371064	48.21179825	-1.556820395	1.75E-07	4.51E-07
Cladopus_010035	root	shoot	109.1428234	50.28864613	79.71573475	1.12270772	1.76E-07	4.54E-07
Cladopus_013619	root	shoot	97.62522397	41.60860018	69.61691207	1.225595103	2.07E-07	5.30E-07

Cladopus_022141	root	shoot	46.54149208	10.97907108	28.76028158	2.089003653	2.11E-07	5.40E-07
Cladopus_006214	root	shoot	59.22934873	128.517927	93.87363787	-1.121124564	2.21E-07	5.65E-07
Cladopus_003372	root	shoot	53.12908103	113.6889466	83.4090138	-1.097927349	2.24E-07	5.73E-07
Cladopus_004790	root	shoot	75.07462428	155.36904	115.2218321	-1.040945967	2.37E-07	6.05E-07
Cladopus_006702	root	shoot	122.9780346	52.81576023	87.89689741	1.212250509	2.41E-07	6.15E-07
Cladopus_021688	root	shoot	97.90763918	41.79435685	69.85099801	1.232907708	2.44E-07	6.23E-07
Cladopus_016024	root	shoot	87.93507091	34.29753187	61.11630139	1.366545761	2.45E-07	6.24E-07
Cladopus_014789	root	shoot	52.6535249	116.1050474	84.37928614	-1.138002832	2.47E-07	6.29E-07
Cladopus_015593	root	shoot	85.87486171	35.49711005	60.68598588	1.274692424	2.53E-07	6.45E-07
Cladopus_003492	root	shoot	49.11700145	109.4462768	79.28163911	-1.157926089	2.54E-07	6.47E-07
Cladopus_005929	root	shoot	12.68711344	52.08183251	32.38447298	-2.043467668	2.55E-07	6.50E-07
Cladopus_020349	root	shoot	57.15230463	119.9605109	88.55640777	-1.068310088	2.57E-07	6.54E-07
Cladopus_003689	root	shoot	3.487742889	32.47118918	17.97946603	-3.241770598	2.71E-07	6.88E-07
Cladopus_013221	root	shoot	151.147655	74.97949472	113.0635748	1.022660473	2.79E-07	7.07E-07
Cladopus_022596	root	shoot	70.92845658	148.2932399	109.6108482	-1.062214913	2.80E-07	7.10E-07
Cladopus_023314	root	shoot	76.26295456	29.09906579	52.68101017	1.388273074	2.80E-07	7.12E-07
Cladopus_015186	root	shoot	45.45307466	11.06336405	28.25821935	2.034138773	2.94E-07	7.45E-07
Cladopus_001031	root	shoot	73.49888562	23.04988895	48.27438728	1.65070033	2.95E-07	7.47E-07
Cladopus_005639	root	shoot	84.7096848	31.74296293	58.22632387	1.42095049	2.99E-07	7.57E-07
Cladopus_018196	root	shoot	125.7798622	61.31619091	93.54802655	1.036944743	3.08E-07	7.78E-07
Cladopus_017822	root	shoot	28.66597814	91.29831026	59.9821442	-1.652157375	3.24E-07	8.18E-07
Cladopus_003970	root	shoot	3.786249797	32.94801628	18.36713304	-3.107682581	3.30E-07	8.32E-07
Cladopus_023963	root	shoot	36.80491019	94.51916575	65.66203797	-1.347966391	3.38E-07	8.52E-07
Cladopus_007731	root	shoot	87.79788785	37.11852666	62.45820725	1.237310016	3.40E-07	8.56E-07
Cladopus_008823	root	shoot	73.46669574	22.87412376	48.17040975	1.692416001	3.58E-07	9.00E-07
Cladopus_001840	root	shoot	42.10474445	9.174041836	25.63939314	2.155529352	3.63E-07	9.13E-07
Cladopus_014515	root	shoot	23.5081525	70.61005235	47.05910243	-1.593792474	3.70E-07	9.28E-07
Cladopus_018666	root	shoot	22.74449028	68.68024924	45.71236976	-1.586304728	3.77E-07	9.46E-07
Cladopus_004484	root	shoot	2.223884772	29.17255572	15.69822024	-3.680456719	3.97E-07	9.94E-07
Cladopus_006258	root	shoot	55.83966595	17.95740288	36.89853442	1.637530389	4.05E-07	1.01E-06

Cladopus_009929	root	shoot	44.64384893	112.5249594	78.58440416	-1.342967797	4.07E-07	1.02E-06
Cladopus_013225	root	shoot	3.512504772	30.2038406	16.85817269	-3.134565873	4.11E-07	1.03E-06
Cladopus_016763	root	shoot	49.35409736	14.0641214	31.70910938	1.799135134	4.15E-07	1.04E-06
Cladopus_018252	root	shoot	72.1114623	148.5034938	110.3074781	-1.042041471	4.28E-07	1.07E-06
Cladopus_015587	root	shoot	10.57267702	44.70549337	27.63908519	-2.063208606	4.31E-07	1.08E-06
Cladopus_000900	root	shoot	53.93991528	114.7374022	84.33865874	-1.08933353	4.36E-07	1.09E-06
Cladopus_022475	root	shoot	20.03452646	63.6650222	41.84977433	-1.657372604	4.43E-07	1.11E-06
Cladopus_009937	root	shoot	75.85809423	167.6680921	121.7630932	-1.15344392	4.72E-07	1.18E-06
Cladopus_004906	root	shoot	125.3055482	61.44614732	93.37584778	1.033016374	4.78E-07	1.19E-06
Cladopus_003238	root	shoot	47.85153481	12.04018114	29.94585798	1.999817071	4.81E-07	1.20E-06
Cladopus_022115	root	shoot	36.48324992	6.153969725	21.31860982	2.545566195	4.82E-07	1.20E-06
Cladopus_017129	root	shoot	136.8777412	19.34554798	78.11164459	2.815071384	4.88E-07	1.21E-06
Cladopus_006971	root	shoot	54.1445771	13.87237064	34.00847387	1.958139539	4.95E-07	1.23E-06
Cladopus_000920	root	shoot	75.41436165	24.51004618	49.96220392	1.609717862	5.01E-07	1.24E-06
Cladopus_008959	root	shoot	35.9551972	88.46880178	62.21199949	-1.303779617	5.19E-07	1.29E-06
Cladopus_006873	root	shoot	36.9812162	89.56876962	63.27499291	-1.270447839	5.21E-07	1.29E-06
Cladopus_001748	root	shoot	72.67789476	154.6548006	113.6663477	-1.091416206	5.24E-07	1.30E-06
Cladopus_005206	root	shoot	41.85278849	8.937293855	25.39504117	2.187151057	5.24E-07	1.30E-06
Cladopus_025942	root	shoot	56.19525079	14.06545386	35.13035232	2.015798589	5.29E-07	1.31E-06
Cladopus_004048	root	shoot	51.29606591	115.8681521	83.58210901	-1.183745317	5.34E-07	1.32E-06
Cladopus_017079	root	shoot	66.1770998	135.0915911	100.6343455	-1.032053248	5.75E-07	1.42E-06
Cladopus_012472	root	shoot	15.96029268	54.07061974	35.01545621	-1.756432738	5.81E-07	1.43E-06
Cladopus_023580	root	shoot	24.48625625	71.55519288	48.02072457	-1.559595454	5.84E-07	1.44E-06
Cladopus_021265	root	shoot	41.7139969	102.6389032	72.17645005	-1.293700716	6.03E-07	1.49E-06
Cladopus_020549	root	shoot	1.57511757	28.48014874	15.02763316	-4.206799484	6.10E-07	1.50E-06
Cladopus_002038	root	shoot	5.210566446	33.97382601	19.59219623	-2.683215946	6.24E-07	1.54E-06
Cladopus_003959	root	shoot	69.22432715	26.22826485	47.726296	1.409204268	6.76E-07	1.66E-06
Cladopus_016105	root	shoot	25.74701944	69.32965768	47.53833856	-1.432571231	7.61E-07	1.86E-06
Cladopus_011925	root	shoot	47.10545071	13.67943471	30.39244271	1.813923106	7.96E-07	1.94E-06
Cladopus_016090	root	shoot	45.48167468	100.1249625	72.80331857	-1.144647945	8.66E-07	2.11E-06

Cladopus_015750	root	shoot	12.10953695	47.88749107	29.99851401	-1.975009913	8.75E-07	2.13E-06
Cladopus_008897	root	shoot	38.80841185	92.52933673	65.66887429	-1.264075688	8.78E-07	2.14E-06
Cladopus_004920	root	shoot	30.01922618	5.392734463	17.70598032	2.523504559	8.90E-07	2.16E-06
Cladopus_011473	root	shoot	0.412536607	28.87682363	14.64468012	-6.139366631	9.05E-07	2.20E-06
Cladopus_003847	root	shoot	10.11148196	41.85933505	25.98540851	-2.065014283	9.06E-07	2.20E-06
Cladopus_001790	root	shoot	7.072924791	37.67816698	22.37554588	-2.395355398	9.18E-07	2.23E-06
Cladopus_001172	root	shoot	91.38393507	40.45401922	65.91897714	1.17751879	9.27E-07	2.25E-06
Cladopus_023862	root	shoot	13.19708906	50.07787324	31.63748115	-1.911119819	9.74E-07	2.36E-06
Cladopus_012643	root	shoot	12.07202252	47.12344556	29.59773404	-1.946759145	9.82E-07	2.38E-06
Cladopus_024646	root	shoot	33.70729375	7.150767804	20.42903078	2.237906894	9.95E-07	2.41E-06
Cladopus_014613	root	shoot	2.01241606	27.53063919	14.77152763	-3.826020592	1.01E-06	2.43E-06
Cladopus_007611	root	shoot	11.17278577	45.89670516	28.53474547	-2.051849255	1.01E-06	2.45E-06
Cladopus_016290	root	shoot	108.5220351	50.90860302	79.71531905	1.0959915	1.03E-06	2.49E-06
Cladopus_002095	root	shoot	104.3842934	42.50127161	73.44278248	1.315354935	1.14E-06	2.74E-06
Cladopus_012492	root	shoot	8.612136453	39.30091604	23.95652624	-2.218822343	1.16E-06	2.78E-06
Cladopus_017128	root	shoot	31.33186485	5.333084129	18.33247449	2.493889525	1.16E-06	2.80E-06
Cladopus_014446	root	shoot	28.30915516	77.32432863	52.8167419	-1.452586082	1.24E-06	2.97E-06
Cladopus_004404	root	shoot	3.250025881	29.78466711	16.51734649	-3.25647732	1.24E-06	2.98E-06
Cladopus_002134	root	shoot	48.54016817	14.5242967	31.53223244	1.72676458	1.33E-06	3.17E-06
Cladopus_006510	root	shoot	58.33816106	20.41369197	39.37592651	1.520006342	1.41E-06	3.35E-06
Cladopus_003772	root	shoot	105.9176815	47.92982338	76.92375243	1.12890843	1.44E-06	3.44E-06
Cladopus_019084	root	shoot	1.974901632	25.33759211	13.65624687	-3.726543907	1.45E-06	3.45E-06
Cladopus_022949	root	shoot	1.57511757	25.17980922	13.37746339	-4.035534132	1.46E-06	3.48E-06
Cladopus_005387	root	shoot	63.38678256	127.6237778	95.50528018	-1.009456267	1.48E-06	3.52E-06
Cladopus_001289	root	shoot	29.85802444	5.284610433	17.57131744	2.471443063	1.55E-06	3.68E-06
Cladopus_015747	root	shoot	77.38195333	157.1028686	117.242411	-1.020255901	1.55E-06	3.69E-06
Cladopus_015062	root	shoot	54.20548911	17.06421252	35.63485082	1.638335336	1.59E-06	3.77E-06
Cladopus_012629	root	shoot	7.54773771	37.38228956	22.46501363	-2.292368839	1.65E-06	3.91E-06
Cladopus_001078	root	shoot	87.00302318	32.69276511	59.84789414	1.398543988	1.66E-06	3.95E-06
Cladopus_015683	root	shoot	111.5219683	51.00155303	81.26176067	1.111983125	1.77E-06	4.19E-06

Cladopus_004655	root	shoot	103.8195915	49.44911201	76.63435177	1.080245836	1.88E-06	4.44E-06
Cladopus_017877	root	shoot	39.00403308	90.48489655	64.74446481	-1.209493598	1.95E-06	4.61E-06
Cladopus_014405	root	shoot	97.52147298	45.84608745	71.68378021	1.091878351	1.97E-06	4.65E-06
Cladopus_014774	root	shoot	5.762637841	34.5314696	20.14705372	-2.581386832	2.00E-06	4.72E-06
Cladopus_011502	root	shoot	55.6695498	115.9331303	85.80134006	-1.06348315	2.01E-06	4.73E-06
Cladopus_005656	root	shoot	32.88073413	79.53284039	56.20678726	-1.274307063	2.05E-06	4.83E-06
Cladopus_004546	root	shoot	104.2974994	49.55642252	76.92696095	1.067727546	2.06E-06	4.84E-06
Cladopus_017145	root	shoot	102.0736146	46.63810673	74.35586067	1.12795903	2.14E-06	5.02E-06
Cladopus_018439	root	shoot	13.74767404	47.51930889	30.63349147	-1.781114699	2.14E-06	5.04E-06
Cladopus_024071	root	shoot	46.10332828	14.25905601	30.18119214	1.693309227	2.15E-06	5.05E-06
Cladopus_008479	root	shoot	80.6581054	34.3285422	57.4933238	1.234887771	2.18E-06	5.12E-06
Cladopus_007231	root	shoot	50.67862327	16.55689598	33.61775963	1.628522635	2.23E-06	5.23E-06
Cladopus_018527	root	shoot	70.51666318	26.02600367	48.27133343	1.43335325	2.27E-06	5.32E-06
Cladopus_005511	root	shoot	26.29525269	69.63338059	47.96431664	-1.411993903	2.28E-06	5.34E-06
Cladopus_025590	root	shoot	115.5300876	55.76966897	85.6498783	1.045603888	2.30E-06	5.39E-06
Cladopus_023479	root	shoot	75.75966372	29.13421883	52.44694128	1.372612895	2.32E-06	5.43E-06
Cladopus_016738	root	shoot	38.89148979	89.57091364	64.23120172	-1.193812773	2.40E-06	5.62E-06
Cladopus_004502	root	shoot	70.7620524	28.59892851	49.68049046	1.306639409	2.48E-06	5.80E-06
Cladopus_004637	root	shoot	107.5586693	52.87222671	80.215448	1.018815469	2.55E-06	5.96E-06
Cladopus_007133	root	shoot	100.5329165	46.08364699	73.30828176	1.109807469	2.59E-06	6.05E-06
Cladopus_007547	root	shoot	26.69652317	69.85628898	48.27640607	-1.390903778	2.60E-06	6.07E-06
Cladopus_023264	root	shoot	129.259556	60.60513345	94.93234471	1.079705749	2.63E-06	6.12E-06
Cladopus_004492	root	shoot	41.77850284	93.00083597	67.3896694	-1.148977298	2.64E-06	6.15E-06
Cladopus_022688	root	shoot	44.50357093	11.6826547	28.09311282	1.924722896	2.66E-06	6.20E-06
Cladopus_000944	root	shoot	47.51959584	105.0430158	76.28130581	-1.148323264	2.76E-06	6.42E-06
Cladopus_005333	root	shoot	145.2219521	71.56836625	108.3951592	1.012914735	2.81E-06	6.53E-06
Cladopus_017972	root	shoot	87.21065375	40.53964464	63.87514919	1.113754669	2.94E-06	6.83E-06
Cladopus_005438	root	shoot	28.81987399	73.77991653	51.29989526	-1.3429458	3.23E-06	7.46E-06
Cladopus_024490	root	shoot	17.23467372	51.45173883	34.34320628	-1.582176813	3.55E-06	8.17E-06
Cladopus_017879	root	shoot	66.82351527	134.3447164	100.5841158	-1.001560398	3.74E-06	8.59E-06

Cladopus_001698	root	shoot	106.9113885	48.79304324	77.85221586	1.139913962	3.78E-06	8.68E-06
Cladopus_006268	root	shoot	2.236637317	24.34626919	13.29145326	-3.414215667	3.85E-06	8.83E-06
Cladopus_000327	root	shoot	94.54965136	40.02833072	67.28899104	1.238874954	3.90E-06	8.94E-06
Cladopus_019585	root	shoot	54.95627667	109.9674349	82.46185577	-1.006035054	4.04E-06	9.25E-06
Cladopus_017190	root	shoot	43.20269737	10.91342665	27.05806201	1.975927652	4.16E-06	9.52E-06
Cladopus_018100	root	shoot	60.40233051	22.62871676	41.51552363	1.401131824	4.25E-06	9.70E-06
Cladopus_019986	root	shoot	66.64881778	24.79379007	45.72130393	1.430767062	4.51E-06	1.03E-05
Cladopus_018084	root	shoot	48.41648086	100.1076464	74.26206363	-1.04109873	4.71E-06	1.07E-05
Cladopus_008019	root	shoot	50.1558951	104.7993149	77.47760498	-1.058901224	4.93E-06	1.12E-05
Cladopus_020232	root	shoot	53.06767003	18.69081166	35.87924085	1.506306274	4.99E-06	1.13E-05
Cladopus_008688	root	shoot	37.31872395	82.78366638	60.05119516	-1.146757531	5.07E-06	1.15E-05
Cladopus_000451	root	shoot	67.84136307	25.26329062	46.55232685	1.454178794	5.11E-06	1.16E-05
Cladopus_016510	root	shoot	28.23499162	70.58089342	49.40794252	-1.331499094	5.15E-06	1.17E-05
Cladopus_024005	root	shoot	93.33704764	45.07819186	69.20761975	1.05657285	5.28E-06	1.20E-05
Cladopus_012060	root	shoot	51.68161102	106.1403168	78.91096389	-1.048532591	5.34E-06	1.21E-05
Cladopus_019926	root	shoot	60.02941584	3.04257072	31.53599328	4.242038463	5.37E-06	1.21E-05
Cladopus_001867	root	shoot	101.1345117	49.71834811	75.4264299	1.014550217	5.49E-06	1.24E-05
Cladopus_025953	root	shoot	97.24945858	47.27523435	72.26234647	1.047045493	5.52E-06	1.25E-05
Cladopus_012959	root	shoot	4.312072896	27.20441571	15.7582443	-2.683414978	5.57E-06	1.26E-05
Cladopus_000641	root	shoot	8.323287156	34.70856725	21.5159272	-2.048787752	6.05E-06	1.36E-05
Cladopus_025958	root	shoot	41.71696973	11.4069036	26.56193666	1.855989581	6.24E-06	1.40E-05
Cladopus_010467	root	shoot	63.55491737	24.92374648	44.23933193	1.355732323	6.27E-06	1.41E-05
Cladopus_007583	root	shoot	132.0994035	63.4439652	97.77168433	1.053420342	6.43E-06	1.44E-05
Cladopus_010419	root	shoot	5.836923492	29.6242193	17.7305714	-2.36314136	6.43E-06	1.44E-05
Cladopus_025219	root	shoot	31.13228337	6.439191402	18.78573739	2.22344717	6.62E-06	1.48E-05
Cladopus_000947	root	shoot	66.83899642	23.54573819	45.1923673	1.525674317	7.20E-06	1.61E-05
Cladopus_016281	root	shoot	91.2378376	43.36130565	67.29957163	1.072502345	7.20E-06	1.61E-05
Cladopus_012883	root	shoot	32.92122138	8.185902773	20.55356208	2.025792123	7.26E-06	1.62E-05
Cladopus_008921	root	shoot	29.49426839	6.469682798	17.9819756	2.212365292	7.44E-06	1.66E-05
Cladopus_023357	root	shoot	9.561640183	37.03489992	23.29827005	-1.952445979	7.51E-06	1.67E-05

Cladopus_008156	root	shoot	50.90668267	103.562064	77.23437336	-1.028327278	7.57E-06	1.69E-05
Cladopus_004935	root	shoot	4.986345189	27.97963786	16.48299152	-2.492328154	7.83E-06	1.74E-05
Cladopus_011820	root	shoot	50.23018076	18.08802552	34.15910314	1.486756031	7.96E-06	1.77E-05
Cladopus_022499	root	shoot	60.42622708	23.90059971	42.16341339	1.329930774	8.02E-06	1.78E-05
Cladopus_003059	root	shoot	19.42327369	57.54620551	38.4847396	-1.563097368	8.10E-06	1.80E-05
Cladopus_020291	root	shoot	58.51446707	23.24933987	40.88190347	1.334304051	8.35E-06	1.85E-05
Cladopus_019818	root	shoot	59.6650387	23.49141573	41.57822721	1.329006273	8.36E-06	1.85E-05
Cladopus_009649	root	shoot	99.26249574	48.71955331	73.99102452	1.037705246	8.69E-06	1.92E-05
Cladopus_021564	root	shoot	133.5222378	60.88584078	97.20403927	1.138756259	8.77E-06	1.94E-05
Cladopus_003716	root	shoot	5.074869797	28.03662328	16.55574654	-2.494058212	9.22E-06	2.04E-05
Cladopus_022931	root	shoot	5.824914153	29.7376712	17.78129268	-2.370326165	9.59E-06	2.11E-05
Cladopus_001792	root	shoot	5.611093714	29.38214345	17.49661858	-2.36084917	9.83E-06	2.16E-05
Cladopus_000587	root	shoot	22.66054702	61.73270146	42.19662424	-1.445977275	1.12E-05	2.45E-05
Cladopus_000623	root	shoot	16.4351056	47.76071852	32.09791206	-1.538605189	1.13E-05	2.48E-05
Cladopus_004530	root	shoot	110.4123788	52.41005272	81.41121578	1.066533509	1.21E-05	2.64E-05
Cladopus_001297	root	shoot	29.78064385	73.67408185	51.72736285	-1.302954494	1.21E-05	2.65E-05
Cladopus_025014	root	shoot	33.28039608	8.46513035	20.87276322	1.997757437	1.21E-05	2.65E-05
Cladopus_006991	root	shoot	81.2812454	35.85582166	58.56853353	1.178224323	1.25E-05	2.71E-05
Cladopus_007622	root	shoot	20.45907241	57.42209787	38.94058514	-1.473222763	1.25E-05	2.71E-05
Cladopus_021323	root	shoot	27.02053516	65.29028888	46.15541202	-1.266980788	1.25E-05	2.72E-05
Cladopus_007714	root	shoot	15.05721779	51.77211354	33.41466566	-1.774732909	1.29E-05	2.81E-05
Cladopus_001072	root	shoot	44.41492421	14.76237714	29.58865068	1.602004095	1.29E-05	2.82E-05
Cladopus_010201	root	shoot	89.27160616	43.56822847	66.41991731	1.035671844	1.30E-05	2.82E-05
Cladopus_006791	root	shoot	31.49777005	75.30201345	53.39989175	-1.270433619	1.31E-05	2.84E-05
Cladopus_008043	root	shoot	40.68191421	12.50102267	26.59146844	1.717628008	1.34E-05	2.91E-05
Cladopus_026903	root	shoot	81.68102947	35.60256917	58.64179932	1.191811699	1.35E-05	2.94E-05
Cladopus_021368	root	shoot	48.80351238	18.15899783	33.4812551	1.433721716	1.44E-05	3.13E-05
Cladopus_017137	root	shoot	4.098995663	25.45237647	14.77568607	-2.607232247	1.45E-05	3.13E-05
Cladopus_025124	root	shoot	80.89656561	38.44339961	59.66998261	1.08375684	1.56E-05	3.37E-05
Cladopus_019041	root	shoot	5.499415743	28.4809603	16.99018802	-2.407205554	1.56E-05	3.37E-05

Cladopus_009826	root	shoot	41.93227658	92.11763512	67.02495585	-1.139768014	1.57E-05	3.40E-05
Cladopus_017680	root	shoot	36.15713041	91.36395273	63.76054157	-1.351213857	1.67E-05	3.60E-05
Cladopus_011315	root	shoot	45.80568668	92.28326547	69.04447608	-1.015946074	1.90E-05	4.07E-05
Cladopus_011570	root	shoot	16.44860135	48.09693151	32.27276643	-1.552135991	2.07E-05	4.42E-05
Cladopus_016804	root	shoot	69.19288048	27.61545109	48.40416578	1.322226784	2.13E-05	4.56E-05
Cladopus_015399	root	shoot	14.3492692	45.10069047	29.72497984	-1.660814588	2.13E-05	4.56E-05
Cladopus_016243	root	shoot	10.49764816	39.14513183	24.82139	-1.87702066	2.14E-05	4.57E-05
Cladopus_024632	root	shoot	13.28412726	43.86410589	28.57411657	-1.728954081	2.23E-05	4.75E-05
Cladopus_001678	root	shoot	32.44417884	72.36409028	52.40413456	-1.151415868	2.26E-05	4.81E-05
Cladopus_019978	root	shoot	30.4326281	70.43828258	50.43545534	-1.216571784	2.29E-05	4.87E-05
Cladopus_024670	root	shoot	21.922512	55.42931327	38.67591264	-1.345835888	2.31E-05	4.92E-05
Cladopus_006382	root	shoot	21.12220067	54.99378059	38.05799063	-1.391006205	2.33E-05	4.95E-05
Cladopus_023255	root	shoot	55.93363722	20.39119336	38.16241529	1.455529521	2.46E-05	5.22E-05
Cladopus_004939	root	shoot	45.68187726	16.24717701	30.96452714	1.474848718	2.50E-05	5.30E-05
Cladopus_017589	root	shoot	50.86384369	19.51517374	35.18950871	1.388684364	2.55E-05	5.41E-05
Cladopus_016092	root	shoot	38.22997005	12.13431828	25.18214416	1.668979471	2.56E-05	5.42E-05
Cladopus_017382	root	shoot	86.59927887	41.79835226	64.19881556	1.036498448	2.57E-05	5.45E-05
Cladopus_002458	root	shoot	63.27349607	25.85356767	44.56353187	1.282001579	2.72E-05	5.74E-05
Cladopus_014948	root	shoot	41.30913658	87.14844514	64.22879086	-1.087664807	2.80E-05	5.92E-05
Cladopus_003616	root	shoot	16.6369167	49.14923722	32.89307696	-1.570829592	2.92E-05	6.16E-05
Cladopus_023184	root	shoot	88.31467443	42.4256357	65.37015507	1.051581287	3.03E-05	6.37E-05
Cladopus_024749	root	shoot	32.39242546	73.88818589	53.14030567	-1.186720398	3.11E-05	6.53E-05
Cladopus_001291	root	shoot	1300.852691	618.5562794	959.7044854	1.072625924	3.12E-05	6.54E-05
Cladopus_021938	root	shoot	58.91895459	21.62340696	40.27118077	1.431649824	3.22E-05	6.74E-05
Cladopus_020292	root	shoot	70.27374373	32.3179226	51.29583316	1.127393458	3.25E-05	6.81E-05
Cladopus_014191	root	shoot	12.63461685	40.34271133	26.48866409	-1.681320246	3.36E-05	7.03E-05
Cladopus_004420	root	shoot	25.89386011	4.823768899	15.3588145	2.37935419	3.59E-05	7.49E-05
Cladopus_014814	root	shoot	49.99234164	19.66762876	34.8299852	1.354509929	3.70E-05	7.70E-05
Cladopus_019337	root	shoot	10.86078311	36.60921143	23.73499727	-1.768497043	4.42E-05	9.13E-05
Cladopus_000027	root	shoot	93.85234781	46.66312296	70.25773539	1.004491098	4.51E-05	9.31E-05

Cladopus_022187	root	shoot	35.37985034	10.44592479	22.91288756	1.732612719	4.70E-05	9.69E-05
Cladopus_012050	root	shoot	75.8031238	30.14670789	52.97491585	1.308103384	4.76E-05	9.81E-05
Cladopus_019956	root	shoot	63.67934789	28.60492261	46.14213525	1.146417086	5.09E-05	0.00010449
Cladopus_007820	root	shoot	11.14876709	36.79363563	23.97120136	-1.733311915	5.19E-05	0.00010642
Cladopus_005041	root	shoot	37.92849031	80.26839319	59.09844175	-1.078571468	5.40E-05	0.000110586
Cladopus_009408	root	shoot	14.0596767	40.84736492	27.45352081	-1.530840661	5.45E-05	0.000111473
Cladopus_014724	root	shoot	37.34137833	9.067916493	23.20464741	2.034761951	5.53E-05	0.000113118
Cladopus_015578	root	shoot	46.43860641	15.81682687	31.12771664	1.560389212	5.61E-05	0.000114726
Cladopus_016208	root	shoot	4.612066217	24.01471784	14.31339203	-2.389197003	6.07E-05	0.000123738
Cladopus_013401	root	shoot	76.12466196	35.42480529	55.77473363	1.103107793	6.17E-05	0.000125684
Cladopus_009565	root	shoot	99.10104572	49.51209153	74.30656862	1.001688771	6.23E-05	0.000126789
Cladopus_018143	root	shoot	37.38100026	77.34830699	57.36465363	-1.040937521	6.83E-05	0.000138594
Cladopus_024424	root	shoot	95.46906865	44.71496394	70.0920163	1.100897115	7.49E-05	0.000151405
Cladopus_014337	root	shoot	41.80338683	90.35198456	66.07768569	-1.109850735	7.65E-05	0.000154449
Cladopus_004712	root	shoot	36.68493891	82.44478847	59.56486369	-1.17025926	8.03E-05	0.000161906
Cladopus_007930	root	shoot	8.273020183	30.76481338	19.51891678	-1.888013682	8.31E-05	0.000167181
Cladopus_001054	root	shoot	37.42074431	77.61968711	57.52021571	-1.056389679	8.46E-05	0.000170018
Cladopus_018943	root	shoot	17.97110022	52.55570208	35.26340115	-1.531620756	8.59E-05	0.000172589
Cladopus_003619	root	shoot	60.73686543	26.05516261	43.39601402	1.215855069	8.65E-05	0.000173745
Cladopus_005472	root	shoot	60.06395744	27.63010421	43.84703082	1.13480309	9.00E-05	0.000180668
Cladopus_018175	root	shoot	19.99564773	56.54356063	38.26960418	-1.497180648	9.11E-05	0.000182596
Cladopus_006989	root	shoot	16.33531486	44.08420403	30.20975944	-1.428860116	9.24E-05	0.000185141
Cladopus_025519	root	shoot	27.59588203	61.30538788	44.45063495	-1.153329114	9.52E-05	0.000190365
Cladopus_009473	root	shoot	35.89515051	11.19465291	23.54490171	1.721236015	9.62E-05	0.000192456
Cladopus_000082	root	shoot	73.16868375	32.99101481	53.07984928	1.147081347	0.000101588	0.000202781
Cladopus_024402	root	shoot	30.05822702	65.93355593	47.99589148	-1.133845028	0.000102641	0.000204756
Cladopus_024031	root	shoot	39.2688637	14.69073861	26.97980115	1.425705344	0.000102717	0.000204865
Cladopus_001941	root	shoot	7.387279178	31.14698245	19.26713081	-2.10122641	0.000108028	0.000214973
Cladopus_003462	root	shoot	16.43659201	47.36019354	31.89839278	-1.52144171	0.000114658	0.000227749
Cladopus_018053	root	shoot	13.53694854	39.89171398	26.71433126	-1.572564188	0.00011771	0.000233549

Cladopus_004125	root	shoot	33.17020452	10.26216681	21.71618567	1.71023346	0.000122172	0.000242056
Cladopus_003690	root	shoot	9.410096056	32.90620292	21.15814949	-1.785960329	0.000123288	0.000244167
Cladopus_001750	root	shoot	28.23424842	62.72121412	45.47773127	-1.156697156	0.000126063	0.000249537
Cladopus_023187	root	shoot	69.8506842	33.94799821	51.89934121	1.056779372	0.000126523	0.000250395
Cladopus_019977	root	shoot	9.135607825	30.74231477	19.9389613	-1.75551694	0.00013251	0.00026158
Cladopus_010507	root	shoot	26.46029257	60.37171661	43.41600459	-1.190561614	0.000139145	0.000274038
Cladopus_022404	root	shoot	5.910343828	26.04916851	15.97975617	-2.107947707	0.000144878	0.000284868
Cladopus_007874	root	shoot	50.05474006	20.81688185	35.43581096	1.278531245	0.000145538	0.000286078
Cladopus_023560	root	shoot	6.149669358	25.38739826	15.76853381	-2.051003779	0.000147499	0.000289729
Cladopus_001137	root	shoot	34.54574061	74.38551292	54.46562676	-1.098682432	0.000147877	0.000290441
Cladopus_000033	root	shoot	69.39171876	34.23440506	51.81306191	1.016600266	0.000148974	0.000292537
Cladopus_022693	root	shoot	8.095970966	83.95009021	46.02303059	-3.361685417	0.000150189	0.000294786
Cladopus_015333	root	shoot	77.7647739	37.71598493	57.74037941	1.038769619	0.000151669	0.00029753
Cladopus_019073	root	shoot	49.50019483	19.33541117	34.417803	1.348219591	0.00015302	0.000299999
Cladopus_006130	root	shoot	29.79723454	65.83409092	47.81566273	-1.144374942	0.000153332	0.00030058
Cladopus_001898	root	shoot	21.20763035	50.57571921	35.89167478	-1.249012254	0.000155456	0.000304651
Cladopus_008332	root	shoot	55.28103188	24.43241355	39.85672271	1.164233087	0.000155692	0.000305083
Cladopus_000492	root	shoot	34.84523494	76.5250491	55.68514202	-1.144982239	0.000155932	0.000305491
Cladopus_004952	root	shoot	9.261646862	30.39278112	19.82721399	-1.725786984	0.000158606	0.000310449
Cladopus_007511	root	shoot	29.33294454	62.10791757	45.72043106	-1.076973396	0.000160622	0.000314111
Cladopus_005074	root	shoot	16.32342763	42.91311855	29.61827309	-1.410057701	0.000165491	0.000322919
Cladopus_006932	root	shoot	61.13145112	27.07164905	44.10155009	1.182523438	0.000165865	0.000323584
Cladopus_016451	root	shoot	28.36994506	7.824526253	18.09723566	1.813323404	0.000166647	0.000325044
Cladopus_002756	root	shoot	68.30119383	33.28008657	50.7906402	1.03178102	0.000170972	0.00033298
Cladopus_014808	root	shoot	37.49193503	13.43469394	25.46331448	1.484075631	0.00017627	0.000342852
Cladopus_013747	root	shoot	47.31766262	20.35470786	33.83618524	1.21024579	0.000188243	0.000364936
Cladopus_001296	root	shoot	8.659308494	33.4606607	21.0599846	-1.946751462	0.000188349	0.000365106
Cladopus_020435	root	shoot	36.45786694	75.51626282	55.98706488	-1.061218747	0.000189452	0.000367134
Cladopus_024729	root	shoot	8.798100076	30.79130936	19.79470472	-1.796344864	0.000190663	0.000369224
Cladopus_016247	root	shoot	32.09453965	68.57760037	50.33607001	-1.083751597	0.000216185	0.000416146

Cladopus_012841	root	shoot	51.01538781	20.97614253	35.99576517	1.254406664	0.000221702	0.000426346
Cladopus_022456	root	shoot	9.14836037	31.99954461	20.57395249	-1.815770882	0.000222234	0.000427258
Cladopus_011014	root	shoot	62.17702956	29.60105446	45.88904201	1.06043732	0.000225697	0.000433703
Cladopus_024480	root	shoot	16.38483863	41.6292474	29.00704301	-1.340478249	0.000228723	0.000439126
Cladopus_023823	root	shoot	5.386872457	24.56037127	14.97362187	-2.20893601	0.000229667	0.000440896
Cladopus_003272	root	shoot	24.75925807	55.64807895	40.20366851	-1.174758366	0.000241789	0.000463207
Cladopus_007424	root	shoot	19.37238562	46.73609396	33.05423979	-1.277968622	0.000276061	0.000525347
Cladopus_009820	root	shoot	53.60005581	24.03122235	38.81563908	1.160746367	0.0002801	0.000532305
Cladopus_013970	root	shoot	40.64613042	13.85971621	27.25292331	1.551652378	0.000282706	0.000536785
Cladopus_002754	root	shoot	11.13452813	37.54688007	24.3407041	-1.748593259	0.000298377	0.000564997
Cladopus_019225	root	shoot	50.90370984	23.30832398	37.10601691	1.138608001	0.000307818	0.000581629
Cladopus_020974	root	shoot	34.00493535	11.02288314	22.51390924	1.673760812	0.000317886	0.000599257
Cladopus_018653	root	shoot	35.60630121	13.11712947	24.36171534	1.444358947	0.000324607	0.000611691
Cladopus_007267	root	shoot	40.21997595	16.48459122	28.35228358	1.282305516	0.000354625	0.00066523
Cladopus_016543	root	shoot	19.15695665	48.10159315	33.6292749	-1.324556957	0.000366496	0.000686307
Cladopus_009726	root	shoot	38.28828611	88.81034461	63.54931536	-1.198725687	0.000371054	0.000694308
Cladopus_025154	root	shoot	26.57048413	56.12371894	41.34710153	-1.076528329	0.000385231	0.000719729
Cladopus_023530	root	shoot	16.42160985	40.4799943	28.45080208	-1.301960931	0.000398333	0.000743137
Cladopus_025195	root	shoot	15.49761121	39.9063671	27.70198915	-1.373839394	0.000403673	0.000752596
Cladopus_016237	root	shoot	49.25504983	23.01259189	36.13382086	1.102837331	0.000406786	0.000758036
Cladopus_013704	root	shoot	34.26753635	11.88957751	23.07855693	1.505391624	0.000410064	0.000763706
Cladopus_022976	root	shoot	23.5087736	52.34907189	37.92892274	-1.145934585	0.000414273	0.000771102
Cladopus_011815	root	shoot	17.99672742	42.67570434	30.33621588	-1.251713834	0.000419917	0.000781234
Cladopus_003250	root	shoot	12.71274064	39.89704185	26.30489124	-1.681841744	0.000421232	0.00078338
Cladopus_008374	root	shoot	41.4343103	83.69498437	62.56464733	-1.02278414	0.000424073	0.000788211
Cladopus_016557	root	shoot	58.67864163	29.34565795	44.01214979	1.005564342	0.000453645	0.000840528
Cladopus_025783	root	shoot	200.4689414	477.3641864	338.9165639	-1.25293785	0.000475542	0.00087809
Cladopus_001589	root	shoot	8.811595827	27.73689578	18.2742458	-1.651993728	0.000478691	0.000883403
Cladopus_018153	root	shoot	59.88913784	29.5757456	44.73244172	1.024528909	0.000481247	0.000887951
Cladopus_016642	root	shoot	17.12373896	44.8379674	30.98085318	-1.396886739	0.000500559	0.000921836

Cladopus_008645	root	shoot	11.16003322	32.45387311	21.80695316	-1.544181682	0.000540408	0.000990536
Cladopus_012452	root	shoot	41.53187142	17.76979482	29.65083312	1.224496588	0.000543998	0.000996835
Cladopus_009090	root	shoot	7.837330213	26.249431	17.04338061	-1.770214931	0.000556694	0.001018853
Cladopus_007461	root	shoot	13.57211124	36.3678018	24.96995652	-1.403783105	0.000558096	0.001020746
Cladopus_017337	root	shoot	14.93266516	39.97667317	27.45466917	-1.405457285	0.000572384	0.001045405
Cladopus_000989	root	shoot	8.486097416	26.65195466	17.56902604	-1.662811992	0.000592107	0.001079503
Cladopus_024693	root	shoot	57.87981672	28.0147909	42.94730381	1.043242087	0.000595386	0.001085176
Cladopus_004298	root	shoot	47.16834812	21.31953676	34.24394244	1.16097485	0.000683905	0.001237016
Cladopus_000645	root	shoot	46.26453001	21.12393591	33.69423296	1.133902856	0.000698683	0.001262455
Cladopus_016131	root	shoot	48.48841479	22.5809093	35.53466204	1.107137715	0.00079936	0.001436249
Cladopus_004887	root	shoot	32.22379573	66.01984758	49.12182165	-1.044603423	0.000873338	0.001561659
Cladopus_008073	root	shoot	34.4565949	14.25905601	24.35782546	1.270896513	0.000887378	0.001585745
Cladopus_016485	root	shoot	51.63283045	23.94307931	37.78795488	1.097335751	0.000931563	0.001661043
Cladopus_011594	root	shoot	46.56687506	22.51593109	34.54140308	1.047552405	0.000933994	0.001665073
Cladopus_008094	root	shoot	18.82019211	44.13852649	31.4793593	-1.210193587	0.000959921	0.001709102
Cladopus_004675	root	shoot	30.04485338	10.3591142	20.20198379	1.5206375	0.000992217	0.001764183
Cladopus_019059	root	shoot	45.13042697	20.49265706	32.81154201	1.157856672	0.001019511	0.001811058
Cladopus_002179	root	shoot	10.13389212	30.9537539	20.54382301	-1.599677036	0.001038157	0.001843002
Cladopus_016660	root	shoot	43.39422977	20.4853305	31.93978013	1.086787102	0.001101244	0.001950374
Cladopus_021734	root	shoot	196.5750919	422.4401404	309.5076161	-1.104236559	0.001147447	0.002028143
Cladopus_008224	root	shoot	13.77392234	35.01761999	24.39577116	-1.342010802	0.00115716	0.002044568
Cladopus_000228	root	shoot	36.32786765	73.52961962	54.92874364	-1.014948176	0.001164831	0.002056442
Cladopus_004110	root	shoot	46.98164129	21.85667846	34.41915988	1.099411395	0.001184355	0.002089016
Cladopus_009375	root	shoot	52.87997578	25.46088819	39.17043198	1.049932344	0.001214344	0.002138617
Cladopus_001474	root	shoot	35.09558239	14.31670766	24.70614502	1.282061491	0.001222389	0.002151033
Cladopus_006016	root	shoot	34.26667103	13.12312357	23.6948973	1.377644875	0.001261321	0.002214337
Cladopus_026730	root	shoot	38.24581752	16.24051668	27.2431671	1.221905873	0.0012827	0.002250043
Cladopus_006717	root	shoot	8.962396748	27.69123233	18.32681454	-1.642278842	0.001336569	0.002340319
Cladopus_022491	root	shoot	15.33715268	38.39107584	26.86411426	-1.343327349	0.001351771	0.002365873
Cladopus_004111	root	shoot	15.78410878	38.35392411	27.06901645	-1.291170848	0.001356498	0.002373506

Cladopus_020739	root	shoot	11.34674005	31.28012271	21.31343138	-1.465851407	0.001368704	0.002392496
Cladopus_020039	root	shoot	56.32425859	26.42704954	41.37565407	1.111243286	0.001395046	0.002436354
Cladopus_014307	root	shoot	17.29855856	43.74251587	30.52053721	-1.358681738	0.001408211	0.002458024
Cladopus_004307	root	shoot	55.91618122	27.23875719	41.5774692	1.062942572	0.001479658	0.002575113
Cladopus_018159	root	shoot	46.18937905	20.49065837	33.34001871	1.169853949	0.001581479	0.002740799
Cladopus_021630	root	shoot	11.78787668	38.89743352	25.3426551	-1.740905786	0.001635758	0.002829074
Cladopus_004888	root	shoot	11.67384698	29.99691779	20.83538239	-1.380284288	0.001681648	0.002902766
Cladopus_024103	root	shoot	39.41978673	17.001233	28.21050987	1.21475372	0.001848131	0.003173256
Cladopus_014509	root	shoot	12.36235823	33.35386912	22.85811368	-1.447249999	0.001868967	0.00320592
Cladopus_025955	root	shoot	43.0200729	19.39187766	31.20597528	1.143085738	0.001870655	0.003208533
Cladopus_004932	root	shoot	51.4614786	24.07902982	37.77025421	1.092637385	0.002150087	0.003661996
Cladopus_014878	root	shoot	35.81950056	15.72653981	25.77302018	1.206911294	0.002293775	0.003892912
Cladopus_004413	root	shoot	11.59881813	29.57855585	20.58868699	-1.364548004	0.002363157	0.004000042
Cladopus_004264	root	shoot	13.32399341	37.32959218	25.32679279	-1.504663424	0.002398051	0.004054522
Cladopus_021629	root	shoot	20.81168443	44.92440438	32.8680444	-1.117456	0.00244147	0.004123083
Cladopus_010088	root	shoot	13.26097389	32.35374186	22.80735788	-1.298648084	0.002514805	0.004241609
Cladopus_019554	root	shoot	41.11475347	18.54501697	29.82988522	1.14998537	0.002652882	0.004462509
Cladopus_008316	root	shoot	15.4623264	38.14966621	26.80599631	-1.302547036	0.002681215	0.004508221
Cladopus_018276	root	shoot	10.92380263	27.97297753	19.44839008	-1.373854651	0.00277421	0.004655736
Cladopus_020083	root	shoot	6.912466258	22.97358877	14.94302752	-1.737560732	0.002872429	0.004810614
Cladopus_015724	root	shoot	16.98569058	36.59722323	26.79145691	-1.111929076	0.002988071	0.004992259
Cladopus_001574	root	shoot	23.27328621	48.31503096	35.79415859	-1.052490544	0.003089143	0.005147866
Cladopus_022102	root	shoot	20.62188267	43.18835071	31.90511669	-1.057399779	0.003137064	0.005223252
Cladopus_010661	root	shoot	10.98521363	28.33516561	19.66018962	-1.36955843	0.003149171	0.00524072
Cladopus_012877	root	shoot	37.41764938	17.72731523	27.5724823	1.0839679	0.003268235	0.005430041
Cladopus_023311	root	shoot	18.02297571	38.68932554	28.35615063	-1.101431735	0.003670811	0.00605908
Cladopus_000817	root	shoot	41.15833566	19.15046803	30.15440184	1.127214267	0.003730534	0.006151399
Cladopus_017172	root	shoot	39.33262642	19.30825092	29.32043867	1.041925497	0.003731475	0.006151908
Cladopus_025134	root	shoot	36.21606757	15.60057881	25.90832319	1.195018173	0.003890953	0.006398573
Cladopus_016796	root	shoot	47.3512168	22.74468629	35.04795154	1.048854946	0.004233945	0.006931575

Cladopus_027079	root	shoot	74.60278419	36.84677097	55.72477758	1.004869973	0.004322443	0.007071108
Cladopus_000924	root	shoot	9.809136912	26.48617898	18.14765795	-1.42830626	0.004649608	0.00757639
Cladopus_004158	root	shoot	15.97539695	37.73781731	26.85660713	-1.2539959	0.005289327	0.008555761
Cladopus_010398	root	shoot	35.53139446	17.12919073	26.3302926	1.031203059	0.005576436	0.008994759
Cladopus_017529	root	shoot	16.260286	35.66355196	25.96191898	-1.119621848	0.005647876	0.009102447
Cladopus_025127	root	shoot	34.49708216	15.66888816	25.08298516	1.166620161	0.006565845	0.010489831
Cladopus_021732	root	shoot	14.5854998	32.65332403	23.61941191	-1.153722385	0.007762389	0.012271535
Cladopus_009429	root	shoot	16.29631402	33.73308261	25.01469832	-1.035774379	0.007779969	0.012298329
Cladopus_020997	root	shoot	12.25885146	54.72957974	33.4942156	-2.159043753	0.010127797	0.015733692
Cladopus_022546	root	shoot	15.50801203	32.64214739	24.07507971	-1.062767272	0.010706326	0.016572923
Cladopus_015351	root	shoot	13.79719781	28.90731503	21.35225642	-1.069032984	0.010949818	0.016929642
Cladopus_017033	root	shoot	15.79760453	31.47705601	23.63733027	-1.00048666	0.010954907	0.016936165
Cladopus_014188	root	shoot	27.63191005	11.74097257	19.68644131	1.23214339	0.011362889	0.017519588
Cladopus_023031	root	shoot	14.93427368	29.98426336	22.45926852	-1.007842769	0.014790145	0.022390556
Cladopus_015969	root	shoot	20.81329295	44.31177406	32.5625335	-1.103655032	0.016939645	0.025436911
Cladopus_008576	root	shoot	12.97138139	28.193221	20.5823012	-1.122925128	0.017761859	0.026562952
Cladopus_014315	root	shoot	8.784604325	39.99251145	24.38855789	-2.18712385	0.035010727	0.049777122
