# Supplementary material for: A high-quality genome provides insights into the new taxonomic status and genomic characteristics of Cladopus chinensis (Podostemaceae)
Source: Hortic Res. 2020 Apr 1;7:46. doi: 10.1038/s41438-020-0269-5 (PMC7109043; doi:10.1038/s41438-020-0269-5)
Supplement: Supplementary file 15 — Table S19 CcWUS_sequence [file 41438_2020_269_MOESM15_ESM.pdf]

>Cladopus_011815-RA protein (CcWUS1)
METTNEDNVGSYSTKGTFLCRQSSTRWTPTTDQIRILKDLYYNNGIRSPNADQIQRISAS
LRQYGKIEGKNVFYWFQNHKARERQKKRFTTDPHHPMQKLRASTNFAPSNEPSSALVPSS
SPALPYFGYNSRDCSASPRYGWATENQFRAPYGLCQYKEQDTEEQDDENMSAKETLPLFP
MHNEDFKSSTASNYYCHSNSPVPYTSLELTLSSYFSQPHDAH
>Cladopus_002745-RA protein (CcWUS2)
MSPATSTRWCPTPEQLMILEELYRNGVRTPNATQIQRITAHLAMYGRIEGKNVFYWFQNH
KARDRQKLRKKFMKQLQQHQQQFLPSFDLLHHQHLLMQNTNPNRHLLFNPQNNPFSLAFP
PLQMGDESGSKVIAPIPRRYVEDASFVNDLQAPTTTKNNNDDEGSSPSCCTEHSPRQGIV
TLELFPTTATNLKEECNPSGSVSGSNVLYEGLSFL
>Cladopus_000528-RA protein (CcWUS3)
MWLKGCTDAADYIKHDSFAATRLRPLVPRPMEKPHVEFAPQSPSVVGSSRWNPTPEQLRI
LEELYRRGTRTPSADQIQNITAQLCRYGKIEGKNVFYWFQNHKARERQKRRRQMDYSLHS
QNMPQFLRFSVSLEDVPIAPAKTGVAEVNEGDQSHLPILSKDLSVIEATALNAAKRRVEV
QGAAMNIDGEGEDFRIRTLQLFPVKNSINCMEKDTSANGTEASDGAETNSNLAAQDLIIF
VKLLHSYALMDQANKF
>Cladopus_000847-RA protein (CcWUS4)
MIPLNAKDAFALVSRQLLHSNRNDRQQTHGFFFISTLHSVSPMTPQTELYKPLSISGHRT
CLPEINGSTTSRWNPTPEQIETLEEMYSKGVRTPSADQIQHIATELRKYGKIEGKNVFYW
FQNHKARERQKRRRECDDPVGKQILGVRMSTSSSSQCKKIAEGLASMQRAERAERRTWPH
FPTKGLQLHCNNNNVNLSIPDTCQDGYSSSLLALELKNNHESEGKEGQIQTLELFPLSGE
NEEKDKKATLATIMTPLQFFEFLPLKNKQI
>Cladopus_003833-RA protein (CcWUS5)
MERIGVGVSKTRKKKPTGGARSNPRLLSLYKGFDMESNENANTENPGMYAESCGSGSSVQ
SNSRWNPTKEQISVLESLYRQGIKTPNADQIQQITARLRTFGHIEGKNVFYWFQNHKARQ
RQKQKQEKVALFHRFVPHHHHHQPLNVVCSPYYLPQHEPSFYQHHHPKMMLPPVSFKRVQ
RSPASFLRESKTVKTTDPETLQLFPLSPTGILEEKEKEKSLTTNSERFVSLCDQTAPSMN
WPLCHANTINPRLSTTHDGLLNSQRYFLLFYLLLREENQGAPLMAFKTFIASPLLSFSAS
TSSHLPHRTSFFHCSPSIQRLSNFRVRLNLHEADFSDLDLATLISRTESLLYSLADAAVT
ADSAAPVVKKNGGWFGFISEGMEIVLKVLKDGLSAVHVPYAYGFAIILLTLFVKFATLPL
TKKQVESTLAMQNLQPKIKAIQQRYAGNQERIQLETSRLYKQAGVTPLAGCFPTLATIPV
WIGLYQALSNVANEGLFTEGFFWIPSLGGPTTIAARQSGSGISWLIPFVDGHPPLGWNDT
AAYLVLPVLLVISQYVSMEIMKPPQTDDPSQKNTLLVLKFLPLLIGYFSLSVPSGLSIYW
FTNNVLSTAQQLWLRKMGGAKPVVAENANGIITAGQAKRSGSAPGERFNKFKEEEKRKAL
VKAVQEDDSDFISGEIIGEEAEKADQIKEEVYVSKTKKSKRSKRKKAI
>Cladopus_018846-RA protein (CcWUS6)
MIPLNAKDAFALVSRQLLHSNRNDRQQTYGFFFISTLHSESPMTPQTELYKPVSISGQRT
CLPEINGSTTSRWNPTPEQIETLEEMYSKGVRTPSADQIQHIATELRKYGKIEGKNVFYW
FQNHKARERQKRRRECDDPVGKQIHGVRMSTSSSSQCKKIAEGLQSMERAERAQRRTWPH
FPTKGLQLHCNNNNVKLGIPDTCQDGYSSSRLALQLKNNHESEGKKAQIQTLELFPLSGE
NEEKDKKAALATIMTPLQKFHEVGEEKRRSEVVYSTDVKESDVEKTFMDSSFVI
>Cladopus_022049-RA protein (CcWUS7)
GGARSNPKLLSLYKGFDMECNENANTENPGMYAESCGSGSSVQSNSRWNPTKEQISVLES
LYRQGIKTPNADQIQQITARLRTFGHIEGKNVFYWFQNHKARQRQKQKQEKWFANCLHVS
VVCSPYYLPQHEPSFYQHHHPKMMLPPVSFKRVQRSPASLLRESNTVKTTDPETLQLFPL
SPTGILEEKEKEKSATTNSERFRYFLLFYLLLREENQGAPLMAFKTFIASPPLSFSASTS
SHLPHRTSFFHCSPSIQRLSNFRVRLNLHEADFSDVDLATLISRTESLLYSLADAAVITA

DSAAPVVKKNGGWFGFISEGMEIVLKVLKDGLSAVHVPYAYGFAIILLTLFVKFATLPLT
KKQVESTLAMQNLQPKIKAIQQRYAGNQERIQLETSRLYKQAGVNPLAGCFPTLATIPVW
IGLYQALSNVANEGLFTEGFFWIPSLGGPTTIAARQSGSGISWLIPFVDGHPPLGWNDTA
AYLVLPVLLVISQYVSMEIMKPPQTDDPSQKNTLLVLKFLPLMIGYFSLSVPSGLSIYWF
TNNVLSTAQQLWLRKMGGAKPVVAENANGIITAGQAKRSGSAPGERFNKFKEEEKRKALV
KAVQEDDSDFVSGEIIGEEEDKADQSKEEIYVSKAKKSKRSKRKKAI
>Cladopus_023688-RA protein (CcWUS8)
MWLMGCTDAADYIKHDSFAATRLRPLVPRPMEKPHVEFSSQSPSVVGSSRWNPTPEQLRI
LEELYRRGTRTPSADQIQNITAQLCRYGKIEGKNVFYWFQNHKARERQKRRRQMDYSLHS
QNMPQFLRFSVSLEDVPIAPAKTGGAEVNEGDQSHLPILSKDLSVIEATALNAAKRRVEV
QGGAMNIDGEGEDFRIRTLQLFPVKNSINCMEKDRSANGTEASDGAETNSNLAAQDLIIF
VKLLHSYAVMDEANKFVLLL
>Cladopus_015470-RA protein (CcWUS9)
MTFSYKLCYLLGKIISLYIYMCADTSLRYPELYRSDRLKMSTFLLPRPSNAGSKNNERWI
PTAQQVKVLKELFKSGLKTPNIDQICKISTHLSSYGKIESKNVFYWFQNHKARERLKRRK
ISVQEGGNRKEIETLQLFPMHSSVESEASSTNGKHHISSEMTHPHLDLRL
>Cladopus_003625-RA protein (CcWUS10)
MSTFLLQRPSNAGSKNNNERWIPTAQQVKVLKELFKSGLKTPNIDQICKISTHLSSYGKI
ESKNVFYWFQNHKARERLKRRKISVQEGGNRKEIETLQLFPMHSSVESEASSTNGKHHIS
REMNHPQLDLRL
