# Supplementary material for: A high-quality genome provides insights into the new taxonomic status and genomic characteristics of Cladopus chinensis (Podostemaceae)
Source: Hortic Res. 2020 Apr 1;7:46. doi: 10.1038/s41438-020-0269-5 (PMC7109043; doi:10.1038/s41438-020-0269-5)
Supplement: Supplementary file 16 — Table S20 CcALS1_sequence [file 41438_2020_269_MOESM16_ESM.pdf]

>Cladopus_003059-RA protein CcALS1
MGKARERGHSLVIQYDGQCSNKRLEIKRPSLHHLVGSALYSGPQAEVAVKARHPLKKAAL
TWSDFLIRRNLSWMKTGGLLVRSLKDMDASLHESRICTPPRASAKRRRLAKLPYCTPLED
TNCLSLSKRNKVASSASVECILQRGMPRPPEQQWVVVSPPIWILVEGMKVGRMHGEGIEG
VAGGLFEKCFPFKEGNGCIKLSRTRASGRSRERSWRIIVVGLGKIDERSLGRRGRPRRRL
VYRLMLEGWAIQGIVRAAVSRIYGDFGVVQAFVGRGYGRSWVKPASLHAVGLLPFAITIA
KFHIFSNFFSAPINGWRNTKKKDINELSMLLTPRKTGKSLRPVKESTAFYDLKRLVLFIH
ENIACFLSSLVLFNSSHGVSCLPSSITSNVHHQANTDRSFEFLCFDISHANEISAESTEK
LIKFYSTEEDKLLRAYVEQYGEGKWHQVPEGAGLNRCRKSCRMRWFNYLNPGIKKGKFME
DEVDLICRLHKLLGNRWSLIAGRLPGRTANSVKNYWNTYHKNPRRVLHRQANSQNEEFRV
IKPRPWNFKNSLVTSDSTEANRVDNLGPPCTEKPGFEECVARVEYSNNEEDDIFLWENLL
SDVNMDEVEMAPERNPNGESRLPGVGESFGQFGEGLHLSGTDFLSFLLDR
>Cladopus_003104-RA protein CcALS2
MNDRLNHATHVNAGEIGYVSFYISQIDLLRPAKKMEGVRKGSWTAKEDELLTAYMEQYGE
GKWHLVPVGAGLNRCRKSCRMRWLNYLKPSIKKGKFMDDEVDLICRLHKLLGNRWSLIAG
RLPGRTANGVKNYWNTNHSKRVRGVNNVKSQNEISRVIKPQPWNFSNSLLLPRCKEVKRV
DNLGAFCKEKRECQEFVLKQECSYIGEDEDLWWENLLSDVKLDDHDMATERNPIGQNMPQ
SDEQNIGELGLNFVDTDFLNYFVDG
>Cladopus_023643-RA protein CcALS3
MVRSPCCDKIGVKKGPWSPEEDIILVSYIQEHGPGNWRTVPCNTGLVRCSKSCRLRWTNY
LRPGIKRGNFSDHEEKMIIHLQALLGNRWAAIASYLPQRTDNDIKNYWNTHLKKKLRKAY
EGESGSSSKCRPKGQWESRLQTDIVQAKQALCEALSLDKQVELSPSVSVPENFSTGVSRP
PASSIYASSAENIAKLLKNWMNSTPKIDESEDQGNFNLLVSNWSSSTQQLIDKPMLSFNS
SISDGSFSGDERNPNANVAQDTCFLEEQSKQISLKNHQVPLSLIEKWLLDENGSQVQEDM
SLVDLGVPFF
>Cladopus_003882-RA protein CcALS4
MMGALSQSEVRKGPWTEEEDTYLTYFVNMFGDRRWDFIAKVSGLNRNGKSCRLRWVNYLH
PDLKRGRMTPQEEKLVVELHEKFGNSSYMCLTCRWSRIARKLPGRTDNEIKNYWRTHMRK
KAQERKRIPRTSPCSSVIMVENPLPPVVKGNDAHSDEQGTYSMDDIWNVIETNSAVGNLP
GEECCVPVGSPLGLMIGDSQRRAEEHEGFV
>Cladopus_014267-RA protein CcALS5
MGRIPYCDNGVKKGPWTPEEDRLMKEYIEKHGHGSWRVLPKLAGLNRCGKSCRLRWTNYL
RPGIKRGKFSEPEEQTILHLHSIIGNKWSVIANHLPGRTDNEVKNFWNTHLKKKLIQMGL
DPITHEPHEDIFACFPHQLIAMLQSQVIDPVNVQLLLQSWAQTINAGEKYNASMNFQKAL
AGSLASSQTLAATLSNFSNDELQYPSAYSVAPFTCQGGDTHANSLHSGENHNPFTNSMEC
DMSYDSSTLSSLPNQSSIYGEASPFFDYMVLP
>Cladopus_019131-RA protein CcALS6
MVNKQGIKSRCEALDKLGWCEGPWAYPTWECRVRPARLHAVGLLPFAIPIAKNLWDLEKN
RLPSMISSERFKSHPAKAEISRKTALPLHSRKHCMFFIFFSPLQLQPWSFLLLVIQFHQR
HSSITSIVHHQANTDRSFEFLCFDISHANEISSESTQKLIKFYSTKEDELLRAYVEQYGE
GKWHPVPEGAGLNRCRKSCRMRWFNHLNPSIKKGKFMEDEVDLICRLHKLLGNRWSLIAG
RLPGRTANSVKNYWNTYHKNTRRVLHRQANSQNEEVRVIKPRPWNFKNSSVTSDSTEANR
VDNLGPRCTEKPGFEECVAKVEYSNNEEDDIFWWENLLSDVKVDEVEMETERNPNGESRF
PGVGESFGQFGEGLNLSGTDFLSFLLDQ
>Cladopus_009992-RA protein CcALS7
MVRKGRCSKQGLNKGGWSTKEDKILVSYIAAYGDGKWTELPQRAGLRRSGKSCRSRWMNY
LKPGIKRGNITPQEEDLIVRLHNLLGNRWSLIAGRIPGRTDNEIKNYWKTVISKKMGKSE

KTAGSPAEKFCSKKDQLSRELDVKKNDDGAAFPDQSFVGDSRDSLSSIFLDFELFLTSAS
VDHGLENHSLFGNGFEDCVDFLDDNLSDNYDVST
>Cladopus_014784-RA protein CcALS8
MERNITSRKGAWTREEDDLLKSYIEEYGEGKWHLVPKKTGINRCRRSCRQRWLNYLKPNI
KRGKFKDDEVDLILKMHRLLGNRWSLIAGRIPGRTANDIKNYWNTHQKVKAGVKVDLFSP
NETPKMQNNIIQPRPWTFRKFLNSEGNQLHAVPRECNLLNDGKIKDVLGSFQSDEEGNFQ
WENFLRDLAFDDEKVNEVTGEALAFDCMESLALDEFGQGNHMKINDKSCEGTEKEGNGGF
WEPDFWSFMHAQ
>Cladopus_016555-RA protein CcALS9
MKERQRWRAEEDALLRAYVKQYGPREWSLVSQRMNASLNRDAKSCLERWKNYLKPGIKKG
SLTEEEQRLVIRLQAKHGNKWKKIAAEVPGRTAKRLGKWWEVFKEKQHRQHNQITKAHQP
IENTKYDRILENFAEKLVKVRPPHPLMMPTSNAPPSAVLPPWLCSSIAGATAPSPSVTLS
LSPSTVAIPTAASPVPWLQSNAGNLAMVELLDCSRDLEEGHRAWVAHKKEAAWRLSRVEL
QLESEKSYRKREKMEEIEMKIKALREEEKACMEMIETEYREQLSQLRTDADVKERKLAEQ
WAANHLRLTTFLQSLQNKPTPPEASGR
>Cladopus_018880-RA protein CcALS10
MGRPPCCDKIGVKKGPWTPEEDIILVSYIQDHGPGNWRAVPTKTGLQRCSKSCRLRWTNY
LRPGIKRGNFTDHEEKMIVHLQALLGNRWAAIASYLPQRTDNDIKNYWNTHLKKKLQKTD
DQSSSSSSSHAKGQWERRLQTDIRQAKQALCEALSLDKESPSSMPESKPTVAHPATSFYA
SSADNIAKLLKNWMKTTPKEAKSEIHEKTLIAEVNSNSNSNSNSSSSTEGLLSFNSNETA
EEHDDRKPMTNVDHMPLSLLEKWLFDENSTQVHEDLIDMPLEESVPLF
