# Supplementary material for: A high-quality genome provides insights into the new taxonomic status and genomic characteristics of Cladopus chinensis (Podostemaceae)
Source: Hortic Res. 2020 Apr 1;7:46. doi: 10.1038/s41438-020-0269-5 (PMC7109043; doi:10.1038/s41438-020-0269-5)
Supplement: Supplementary file 17 — Table S21 CcSTM4_sequence [file 41438_2020_269_MOESM17_ESM.pdf]

>Cladopus_005714-RA protein CcSTM1
MSEDNFDSTVYLPNLPVICCLCTRTTTLSFLLCNGRASVAVEGKRGEIEFSSGSARIYTV
RPPSLDTCFLRKKVEISVPFDSDMNSDYIMGSSTVETSICATKARIMSHPHYTRLLSAFA
NCQKVCSCDLIFLLLAIRVGAPPDAVARLEAARVSALETARVAAATVFGPSSICIGEDPA
LDRFMEAYCVMLNNLKLSLIHPIHFLARQGKGKVCFRGAVEGEVNENNASQEEEEVDVYD
DFIDPHAKDHELRGQLMQRYRGYLGSLKQVFTKKRKKGKLPNEARQKLLDWWTDHHKWPY
PSESQKLALAESTGLDHQQINNWFINQRKRHWKPSEDVQYYYVDNVIVSPCGVDVQENML
Q
>Cladopus_017219-RA protein CcSTM2
MSEDNFDSTVYLTKLLPSLFFSAMAEHQWQWKEREERLSFHREAVRPPSLDTCFLRKKGE
ISVPFDSDMNSDYIMGSSTVETSICATKARIMSHPHYTRLLSAFANCQKGYCFPTCENLK
MGLLIISYLNHVFLMEVGAPPDAVARLEAARVSALEGARVAAATVFGPSSICIGEDPALD
RFMEAYCVMLNNELNSSLKLSLIHPILFLARQGKGKVCFRGAGDDEVNENNASQEEEEVD
VYGDFIDPQAKDHELRWQLMQRYRGYLGSLKQVFMKKRKKGKLPNEARQKLLDWWTDHNK
WPYPSESQKLALAESTGLDHQQINNWFINQRKRHWKPSEDVQYYYVDNVIVSPCGVDVQE
NMLQ
>Cladopus_009507-RA protein CcSTM3
MRLFPKMSFGGKESPPTKHERPDCYSYTSENKPNTLTRSWDKIVREIMEGGSNSNCSSLL
AFGDITNGLVGPMMIIPQSPNALFPPSNTSFHHHQNKSKTHHDTLSSPCMAFGSQMNVNA
TANSTGAGCFFVENNVHEGINTTCSIKAKIMSHPHYNRLLSAYVNCQKIGAPPEVVARLE
EARVAAAAALGPSDSCLGQDPALDQFMEAYCEMLTKYEQELSKPLKEAMVFLQRVEYQFK
ALTVSSPNSGYSGEANERNASSDEEGDGNNVFIDPQAEDQELKGQLLRRYSGYLGSLKQE
FMKKRKKGKLPKEARQQLLEWWSRHYKWPYPSESQKLALAESTGLDQKQINNWFINQRKR
HWKPSEDMQFAVMDGAHPHYYMDNVIGNPFPMDVSPTML
>Cladopus_016413-RA protein CcSTM4
MESLAWNLQDREKSQRFCTWVKLNDNRKRSSKATLDKIVRCILSISGLIACAHEQPHSSL
PVMAEHQWQCKKGERVSFHREGKSYHPSHLSLSTRPIPYTFSPLRFRKLISLTINTRNAS
LSKDVHRKGVWQVKGFLWYLHYLEFSLSKSLLRGGKSRHQQKHERADCYSYTSESKPNTK
TRSWDKIVREIMEGGSNSNCSSLLAFGDITNGLVGPMMIIPQTPNALFPPSNTTFHHHQN
KSKTHHDTLSSPCMAFGSQMNVNATANSTGAGCFFVENNVHEGINTTCSIKAKIMSHPHY
NRLLLAYVNCQKIGAPPEVVARLEEARVAAAAALGPSDSCLGQDPALDQFMEAYCEMLTK
YEQELSKPLKEAMVFLQRVEYQFKALTVSSPNSGYSGEANERNASSDEEGDGNNVFIDPQ
AEDQELKGQLLRRYSGYLGSLKQEFMKKRKKGKLPKEARQQLLDWWSRHYKWPYPSESQK
LALAESTGLDQKQINNWFINQRKRHWKPSEDMQFAVMDGAHPHYYMDNVIGNPFPMDVSP
TML
