# Supplementary material for: A high-quality genome provides insights into the new taxonomic status and genomic characteristics of Cladopus chinensis (Podostemaceae)
Source: Hortic Res. 2020 Apr 1;7:46. doi: 10.1038/s41438-020-0269-5 (PMC7109043; doi:10.1038/s41438-020-0269-5)
Supplement: Supplementary file 18 — Table S22 609 specific expressed genes in shoots [file 41438_2020_269_MOESM18_ESM.pdf]

GeneID	root_1	root_2	root_3	shoot_1	shoot_2	shoot_3
Cladopus_	12899	13375	11414	51349	45763	65883
Cladopus_	390	434	378	5360	4982	7092
Cladopus_	9964	10226	9579	50424	45095	64120
Cladopus_	1231	1263	1249	11362	10863	15129
Cladopus_	5039	5065	5240	50332	45761	65648
Cladopus_	858	956	860	11045	10222	14770
Cladopus_	43	49	47	2431	2277	3320
Cladopus_	69	109	69	2645	2392	3303
Cladopus_	880	1078	889	7632	6874	9817
Cladopus_	277	241	220	3287	3051	4317
Cladopus_	2548	2684	2461	17477	15824	22852
Cladopus_	500	468	423	4020	3756	5643
Cladopus_	737	718	672	7639	6695	9546
Cladopus_	8190	8226	8510	55608	50454	71987
Cladopus_	851	863	883	6887	6193	8796
Cladopus_	6954	7659	7552	48248	44529	62978
Cladopus_	2101	2251	2106	11614	11168	15365
Cladopus_	2093	2223	2294	15820	14322	20940
Cladopus_	4061	4257	3945	17755	16186	23068
Cladopus_	1577	1720	1560	8158	7289	10294
Cladopus_	778	858	747	4840	4258	5876
Cladopus_	1902	1918	1765	8546	7462	10903
Cladopus_	289	334	303	2686	2415	3376
Cladopus_	1258	1258	1223	6582	5845	8520
Cladopus_	5286	5593	5612	31001	28318	40675
Cladopus_	1008	1066	1041	6011	5562	7743
Cladopus_	1046	1069	1029	5646	5131	7165
Cladopus_	756	823	659	4351	3821	5361
Cladopus_	1175	1254	1168	5727	5239	7316
Cladopus_	404	446	393	2683	2414	3493
Cladopus_	578	593	603	3763	3297	4722
Cladopus_	11114	11648	11274	48766	44956	65478
Cladopus_	128	159	114	1938	1584	2206
Cladopus_	795	825	628	4304	3853	5554
Cladopus_	1434	1392	1347	6482	5730	8490
Cladopus_	720	776	741	4312	3673	5283
Cladopus_	2548	2803	2575	11287	10150	14469
Cladopus_	344	387	281	2729	2431	3242
Cladopus_	361	367	340	2449	2178	2990
Cladopus_	4484	4750	4562	19517	18088	25744
Cladopus_	1062	1209	1110	5882	5622	7629
Cladopus_	214	257	226	1958	1859	2630
Cladopus_	740	755	626	3643	3192	4543
Cladopus_	945	1023	830	4355	3856	5549
Cladopus_	954	1011	936	4539	4351	6137
Cladopus_	499	607	526	3324	2966	4100

Cladopus_	773	794	785	4057	3744	5122
Cladopus_	79	74	91	1563	1239	1798
Cladopus_	221	248	184	1908	1595	2433
Cladopus_	166	166	146	1583	1376	1897
Cladopus_	480	450	345	2814	2539	3630
Cladopus_	7910	8341	6301	33550	29627	42268
Cladopus_	60	104	93	1460	1292	1909
Cladopus_	727	802	666	3580	3317	4981
Cladopus_	1416	1456	1443	6461	5896	8606
Cladopus_	252	280	217	2151	1750	2434
Cladopus_	599	665	584	3026	2776	3887
Cladopus_	640	538	493	3513	3080	4296
Cladopus_	2197	2450	2426	11676	10832	15112
Cladopus_	175	256	171	1964	1771	2561
Cladopus_	905	825	958	5804	5301	7431
Cladopus_	1158	1141	973	4564	4091	5801
Cladopus_	1436	1534	1415	5839	5524	7804
Cladopus_	663	680	725	3919	3554	5121
Cladopus_	424	452	389	2348	2026	3007
Cladopus_	1291	1306	1238	5115	4699	6607
Cladopus_	4732	5203	4890	19056	17235	24888
Cladopus_	1143	1281	1201	5384	5004	7008
Cladopus_	1005	1070	961	4183	3682	5271
Cladopus_	1149	1213	1144	4812	4247	6086
Cladopus_	2810	2816	2740	10546	9485	13109
Cladopus_	173	143	135	1600	1309	1798
Cladopus_	234	241	181	1672	1404	2082
Cladopus_	346	312	258	1908	1743	2497
Cladopus_	1380	1370	1146	5104	4400	6465
Cladopus_	88	93	64	1145	932	1389
Cladopus_	2049	1970	1700	7279	6278	9542
Cladopus_	483	506	542	2904	2763	3966
Cladopus_	615	605	573	2700	2514	3441
Cladopus_	27	53	43	1080	863	1330
Cladopus_	554	672	514	2874	2673	3919
Cladopus_	231	248	176	1524	1429	2055
Cladopus_	153	152	127	1380	1083	1677
Cladopus_	1070	1079	1165	5566	4852	7204
Cladopus_	1749	1967	1670	6343	5857	8542
Cladopus_	1750	1963	1673	6327	5847	8530
Cladopus_	354	352	314	1860	1603	2345
Cladopus_	184	135	173	1494	1383	1949
Cladopus_	119	87	95	1071	1045	1506
Cladopus_	945	1116	987	4264	3854	5375
Cladopus_	7131	7320	7289	25857	24416	34784
Cladopus_	1216	1252	1062	4356	3862	5288
Cladopus_	836	951	746	3460	3232	4375

Cladopus_	165	155	170	1301	1112	1584
Cladopus_	69	59	45	923	764	1140
Cladopus_	1074	1068	905	3818	3305	4851
Cladopus_	633	560	519	2644	2313	3340
Cladopus_	1115	1154	1171	4674	4170	6026
Cladopus_	1039	1163	1052	4102	3614	5188
Cladopus_	814	825	791	3129	2887	4092
Cladopus_	901	993	921	3605	3211	4543
Cladopus_	84	72	80	940	793	1122
Cladopus_	746	846	661	3004	2673	3837
Cladopus_	733	695	573	2810	2513	3575
Cladopus_	195	212	171	1259	1136	1517
Cladopus_	937	1032	1026	4222	3746	5221
Cladopus_	295	316	232	1528	1429	1995
Cladopus_	454	533	473	2218	1973	2749
Cladopus_	1098	1100	814	4420	3887	5414
Cladopus_	699	706	814	3848	3525	5209
Cladopus_	93	70	86	879	869	1125
Cladopus_	128	144	109	940	901	1262
Cladopus_	144	133	141	1038	1134	1559
Cladopus_	98	140	116	961	880	1234
Cladopus_	847	959	918	3543	3192	4424
Cladopus_	86	64	84	808	760	1137
Cladopus_	714	620	611	2745	2358	3587
Cladopus_	291	287	268	1481	1225	1793
Cladopus_	116	104	106	845	774	1109
Cladopus_	1086	1090	1165	4407	4012	5910
Cladopus_	366	305	341	1803	1523	2289
Cladopus_	41	63	51	676	628	953
Cladopus_	143	121	108	923	865	1133
Cladopus_	319	306	318	1494	1341	1877
Cladopus_	23	12	29	637	595	846
Cladopus_	1266	1308	1402	5314	4682	6702
Cladopus_	496	580	456	2176	1837	2594
Cladopus_	577	605	643	2627	2653	3644
Cladopus_	555	495	501	2071	1984	2809
Cladopus_	576	590	527	2079	1804	2669
Cladopus_	1008	968	793	3422	2858	4255
Cladopus_	84	132	103	880	804	1120
Cladopus_	446	543	408	1971	1749	2457
Cladopus_	506	554	430	2051	1706	2611
Cladopus_	81	84	69	759	637	875
Cladopus_	23	23	28	561	544	790
Cladopus_	385	454	441	1950	1660	2519
Cladopus_	564	576	642	2604	2382	3393
Cladopus_	561	612	622	2930	2323	3252
Cladopus_	974	1039	742	3726	3378	4429

Cladopus_	252	288	227	1300	1065	1632
Cladopus_	173	197	148	1054	856	1320
Cladopus_	250	290	235	1283	1076	1575
Cladopus_	188	190	205	1058	968	1438
Cladopus_	288	264	271	1307	1192	1609
Cladopus_	414	336	350	1759	1533	2097
Cladopus_	403	413	438	1772	1590	2262
Cladopus_	353	443	300	1706	1503	2217
Cladopus_	711	901	848	3387	3083	4507
Cladopus_	212	251	204	1133	966	1383
Cladopus_	247	298	306	1462	1301	1812
Cladopus_	60	39	31	596	573	745
Cladopus_	1053	1004	1081	3879	3455	4693
Cladopus_	448	469	433	1645	1515	2240
Cladopus_	157	131	146	892	807	1107
Cladopus_	82	101	69	753	617	854
Cladopus_	23	34	24	507	463	667
Cladopus_	630	517	581	2449	2153	3093
Cladopus_	324	366	321	1354	1219	1793
Cladopus_	296	263	276	1253	1122	1676
Cladopus_	440	380	383	1616	1436	2126
Cladopus_	332	307	338	1476	1277	1800
Cladopus_	307	317	236	1336	1119	1612
Cladopus_	355	394	320	1517	1240	1808
Cladopus_	615	693	698	2488	2219	3222
Cladopus_	183	216	212	1036	933	1472
Cladopus_	18	15	8	486	472	719
Cladopus_	353	330	288	1279	1152	1688
Cladopus_	372	337	296	1430	1206	1707
Cladopus_	997	1080	730	3597	3210	4493
Cladopus_	178	166	173	874	793	1152
Cladopus_	223	224	226	1056	936	1279
Cladopus_	90	78	104	700	624	864
Cladopus_	195	178	160	897	784	1141
Cladopus_	36	29	38	479	422	628
Cladopus_	14	27	17	486	391	628
Cladopus_	520	575	581	2019	1878	2622
Cladopus_	351	354	324	1320	1140	1636
Cladopus_	214	317	205	1331	1164	1845
Cladopus_	379	374	352	1322	1222	1768
Cladopus_	53	37	41	475	444	633
Cladopus_	530	467	517	1944	1667	2519
Cladopus_	530	588	689	2766	2497	3630
Cladopus_	48	49	39	497	449	578
Cladopus_	273	327	325	1312	1174	1725
Cladopus_	105	106	101	641	578	809
Cladopus_	144	165	136	821	726	948

Cladopus_	201	175	187	878	847	1197
Cladopus_	84	48	58	581	514	812
Cladopus_	96	99	87	624	526	761
Cladopus_	246	270	224	1015	918	1265
Cladopus_	237	284	217	1076	995	1292
Cladopus_	135	197	175	919	809	1196
Cladopus_	10	24	25	386	360	522
Cladopus_	1204	1274	1435	4755	4316	5947
Cladopus_	622	503	541	2083	1812	2514
Cladopus_	370	437	462	1670	1566	2227
Cladopus_	274	351	299	1197	1066	1599
Cladopus_	1032	875	1035	3512	3240	4537
Cladopus_	88	54	80	568	528	734
Cladopus_	45	31	22	418	350	563
Cladopus_	187	176	191	853	843	1055
Cladopus_	249	251	223	907	888	1259
Cladopus_	42	35	27	417	344	505
Cladopus_	148	151	133	715	602	877
Cladopus_	317	288	191	1209	1151	1658
Cladopus_	352	314	321	1208	1073	1682
Cladopus_	302	281	332	1255	1139	1573
Cladopus_	15	14	23	356	318	461
Cladopus_	157	143	160	796	634	1041
Cladopus_	185	185	154	833	658	1004
Cladopus_	194	166	136	756	709	1052
Cladopus_	70	61	51	491	395	586
Cladopus_	28	45	48	408	374	585
Cladopus_	180	180	170	771	687	944
Cladopus_	259	274	232	912	849	1242
Cladopus_	144	122	108	642	581	785
Cladopus_	126	104	116	658	558	754
Cladopus_	7	12	10	365	305	471
Cladopus_	161	160	176	728	707	971
Cladopus_	219	246	212	884	761	1093
Cladopus_	258	286	266	967	889	1237
Cladopus_	21	18	22	342	282	418
Cladopus_	256	288	233	1019	821	1229
Cladopus_	141	177	127	727	640	841
Cladopus_	33	25	25	331	314	423
Cladopus_	177	199	154	717	741	974
Cladopus_	230	270	248	908	823	1237
Cladopus_	209	261	219	855	812	1137
Cladopus_	46	29	39	358	328	471
Cladopus_	5	7	8	342	332	507
Cladopus_	48	34	22	368	336	443
Cladopus_	64	70	71	435	380	609
Cladopus_	293	258	225	939	882	1181

Cladopus_	71	61	75	429	419	592
Cladopus_	73	86	78	471	399	611
Cladopus_	250	299	192	1101	978	1209
Cladopus_	45	53	28	409	380	443
Cladopus_	115	86	64	523	484	710
Cladopus_	25	25	11	321	283	368
Cladopus_	250	250	190	838	762	1099
Cladopus_	17	18	17	281	272	363
Cladopus_	91	76	49	461	427	605
Cladopus_	172	161	137	638	587	830
Cladopus_	34	25	29	301	278	422
Cladopus_	298	338	367	1183	1134	1678
Cladopus_	105	113	141	664	540	782
Cladopus_	190	185	161	720	595	895
Cladopus_	51	37	50	383	363	436
Cladopus_	288	271	200	957	855	1146
Cladopus_	129	122	96	563	555	668
Cladopus_	125	145	124	562	522	726
Cladopus_	222	250	170	789	730	1083
Cladopus_	145	138	135	565	558	778
Cladopus_	166	158	155	635	562	878
Cladopus_	41	59	43	370	310	442
Cladopus_	187	165	162	680	582	912
Cladopus_	44	40	24	338	324	388
Cladopus_	77	79	58	411	369	520
Cladopus_	137	199	149	712	601	895
Cladopus_	90	107	75	457	411	619
Cladopus_	100	83	128	588	540	733
Cladopus_	27	27	20	315	241	341
Cladopus_	21	13	10	245	242	334
Cladopus_	9	20	17	246	236	349
Cladopus_	170	171	164	678	566	799
Cladopus_	13	15	28	283	259	328
Cladopus_	176	228	194	778	652	938
Cladopus_	91	98	82	454	396	554
Cladopus_	203	192	214	759	679	939
Cladopus_	18	20	25	249	235	364
Cladopus_	73	69	65	403	376	469
Cladopus_	43	33	20	289	272	419
Cladopus_	98	99	102	447	446	641
Cladopus_	89	93	90	451	383	583
Cladopus_	176	166	146	653	530	811
Cladopus_	209	232	231	768	768	1005
Cladopus_	399	360	256	1260	1016	1577
Cladopus_	66	57	67	376	358	461
Cladopus_	87	94	76	401	395	567
Cladopus_	119	107	90	500	404	658

Cladopus_	25	6	16	275	245	309
Cladopus_	65	65	84	477	421	488
Cladopus_	257	225	274	881	818	1234
Cladopus_	184	229	166	729	594	971
Cladopus_	88	100	83	407	388	557
Cladopus_	69	53	74	392	365	469
Cladopus_	34	29	53	347	300	390
Cladopus_	150	106	121	529	579	782
Cladopus_	14	21	9	217	203	306
Cladopus_	15	19	15	259	204	268
Cladopus_	41	44	46	315	269	363
Cladopus_	7	5	17	225	197	318
Cladopus_	150	156	143	573	478	725
Cladopus_	42	46	39	295	251	376
Cladopus_	11	15	27	221	239	331
Cladopus_	154	181	170	594	593	785
Cladopus_	88	119	99	444	403	616
Cladopus_	137	168	108	544	496	716
Cladopus_	148	132	99	518	462	665
Cladopus_	55	61	47	328	310	377
Cladopus_	135	120	102	483	467	595
Cladopus_	72	58	35	340	332	425
Cladopus_	13	6	16	241	182	250
Cladopus_	80	38	65	1150	956	1412
Cladopus_	55	43	52	328	253	395
Cladopus_	23	34	25	226	242	296
Cladopus_	71	59	60	349	284	428
Cladopus_	128	131	133	539	427	637
Cladopus_	30	70	56	879	785	1114
Cladopus_	52	67	71	372	308	413
Cladopus_	75	63	47	347	278	484
Cladopus_	116	156	131	593	472	634
Cladopus_	83	89	95	380	359	511
Cladopus_	40	65	43	286	292	376
Cladopus_	24	36	39	236	237	324
Cladopus_	103	104	114	420	406	552
Cladopus_	10	6	3	201	171	235
Cladopus_	90	84	85	357	341	503
Cladopus_	114	116	100	421	370	552
Cladopus_	7	3	6	195	181	237
Cladopus_	42	37	25	222	231	338
Cladopus_	32	35	23	228	232	269
Cladopus_	102	77	97	398	351	535
Cladopus_	8	7	10	173	186	210
Cladopus_	72	92	95	385	338	490
Cladopus_	7	6	4	172	178	239
Cladopus_	21	10	10	158	233	315

Cladopus_	43	35	52	290	228	357
Cladopus_	54	40	35	266	224	356
Cladopus_	100	112	92	386	376	505
Cladopus_	77	86	47	353	293	476
Cladopus_	44	38	29	222	229	343
Cladopus_	16	16	7	181	157	227
Cladopus_	10	6	10	185	157	196
Cladopus_	39	25	36	244	200	301
Cladopus_	11	18	19	175	159	245
Cladopus_	59	62	41	282	240	380
Cladopus_	50	35	34	267	229	290
Cladopus_	163	123	132	473	467	657
Cladopus_	42	58	42	274	216	338
Cladopus_	36	42	40	242	207	289
Cladopus_	3	4	11	219	134	206
Cladopus_	89	78	67	357	283	417
Cladopus_	32	62	33	280	224	333
Cladopus_	71	120	88	398	363	494
Cladopus_	96	94	88	352	327	451
Cladopus_	32	18	19	194	179	236
Cladopus_	6	15	14	157	154	196
Cladopus_	105	89	96	359	345	475
Cladopus_	41	45	48	254	221	293
Cladopus_	25	11	11	167	164	215
Cladopus_	20	13	24	171	173	217
Cladopus_	87	103	114	379	353	504
Cladopus_	63	98	75	322	316	426
Cladopus_	40	27	24	212	188	236
Cladopus_	76	52	50	270	245	408
Cladopus_	106	100	73	368	332	417
Cladopus_	55	65	76	336	259	358
Cladopus_	72	65	58	277	266	336
Cladopus_	45	66	51	249	242	316
Cladopus_	22	17	8	150	143	199
Cladopus_	106	91	109	347	357	494
Cladopus_	38	32	26	188	178	244
Cladopus_	16	15	12	150	122	185
Cladopus_	91	130	78	399	324	474
Cladopus_	67	98	66	333	277	380
Cladopus_	43	27	31	190	199	253
Cladopus_	27	39	39	239	160	288
Cladopus_	78	96	73	315	276	383
Cladopus_	74	79	70	309	242	351
Cladopus_	4	10	12	105	126	188
Cladopus_	4	12	4	114	115	153
Cladopus_	58	94	50	298	244	382
Cladopus_	17	6	12	135	108	175

Cladopus_	34	48	43	213	185	251
Cladopus_	0	1	0	1213	1298	1588
Cladopus_	2	0	7	132	113	162
Cladopus_	79	73	67	309	222	363
Cladopus_	13	11	21	139	115	178
Cladopus_	67	65	65	243	226	348
Cladopus_	15	13	14	115	127	167
Cladopus_	26	9	15	133	134	202
Cladopus_	17	31	40	180	163	257
Cladopus_	23	27	29	153	147	205
Cladopus_	8	7	6	106	102	139
Cladopus_	46	70	60	241	229	305
Cladopus_	42	26	52	221	247	256
Cladopus_	89	98	82	418	279	370
Cladopus_	92	102	70	333	309	347
Cladopus_	35	42	29	178	169	212
Cladopus_	35	44	36	173	169	237
Cladopus_	66	88	64	246	248	356
Cladopus_	94	64	50	273	252	354
Cladopus_	63	73	47	247	196	336
Cladopus_	18	21	10	129	101	175
Cladopus_	19	37	23	155	145	189
Cladopus_	13	14	20	141	105	148
Cladopus_	40	31	36	157	163	234
Cladopus_	2	3	6	100	90	121
Cladopus_	16	14	21	136	103	159
Cladopus_	10	8	5	99	80	141
Cladopus_	26	13	15	141	135	144
Cladopus_	71	86	51	242	237	312
Cladopus_	19	17	24	120	122	165
Cladopus_	37	38	38	158	148	254
Cladopus_	32	66	46	211	216	251
Cladopus_	47	38	78	251	245	309
Cladopus_	24	25	32	139	125	198
Cladopus_	47	43	27	185	136	269
Cladopus_	4	5	1	90	75	110
Cladopus_	2	2	0	136	107	184
Cladopus_	54	48	50	205	171	234
Cladopus_	6	11	14	124	92	104
Cladopus_	37	37	29	152	143	188
Cladopus_	49	58	65	212	206	250
Cladopus_	39	44	25	160	132	221
Cladopus_	58	49	55	210	171	246
Cladopus_	51	53	49	190	161	243
Cladopus_	73	44	63	236	193	283
Cladopus_	23	28	16	110	117	170
Cladopus_	11	9	11	74	92	133

Cladopus_	10	4	4	79	65	121
Cladopus_	34	34	54	172	155	244
Cladopus_	28	17	14	118	104	150
Cladopus_	32	30	43	146	140	209
Cladopus_	39	20	17	138	120	174
Cladopus_	44	60	27	213	149	225
Cladopus_	12	16	6	91	77	119
Cladopus_	20	15	18	134	102	113
Cladopus_	13	11	10	94	74	107
Cladopus_	23	49	32	147	138	204
Cladopus_	25	34	22	140	104	161
Cladopus_	30	47	29	183	135	169
Cladopus_	21	25	29	115	116	153
Cladopus_	6	4	4	84	52	88
Cladopus_	48	34	29	141	147	191
Cladopus_	1	2	6	77	55	89
Cladopus_	6	5	5	67	58	95
Cladopus_	17	28	13	101	96	142
Cladopus_	34	54	42	149	156	215
Cladopus_	26	31	27	116	105	178
Cladopus_	30	70	53	194	179	252
Cladopus_	35	36	40	128	136	205
Cladopus_	24	18	15	95	97	129
Cladopus_	1	5	7	63	49	117
Cladopus_	19	18	9	86	85	124
Cladopus_	17	43	36	144	131	184
Cladopus_	38	28	16	115	118	171
Cladopus_	1	7	0	50	71	92
Cladopus_	56	31	56	171	172	227
Cladopus_	10	2	2	73	58	74
Cladopus_	4	0	1	66	56	89
Cladopus_	15	24	25	110	96	124
Cladopus_	24	17	37	119	112	161
Cladopus_	30	24	26	108	105	148
Cladopus_	30	35	29	102	122	203
Cladopus_	34	51	35	158	121	181
Cladopus_	28	31	35	119	106	174
Cladopus_	32	32	33	119	105	179
Cladopus_	7	15	10	74	64	97
Cladopus_	42	52	24	152	122	190
Cladopus_	16	25	24	87	93	159
Cladopus_	9	20	16	96	71	106
Cladopus_	3	0	3	49	50	84
Cladopus_	1	7	2	39	72	79
Cladopus_	43	46	21	140	122	176
Cladopus_	25	18	31	95	131	140
Cladopus_	5	4	4	51	44	84

Cladopus_	376	351	187	1181	1048	1596
Cladopus_	11	13	12	84	68	80
Cladopus_	30	32	28	99	130	149
Cladopus_	21	28	12	82	86	172
Cladopus_	10	13	15	72	68	95
Cladopus_	14	24	19	62	114	184
Cladopus_	19	11	19	90	84	95
Cladopus_	7	15	5	58	61	93
Cladopus_	7	10	14	69	70	76
Cladopus_	29	12	18	89	83	123
Cladopus_	3	2	4	41	53	54
Cladopus_	29	25	44	123	112	155
Cladopus_	7	8	5	51	44	81
Cladopus_	0	0	0	40	36	58
Cladopus_	14	12	7	73	50	83
Cladopus_	0	1	7	42	41	64
Cladopus_	8	14	4	77	37	91
Cladopus_	14	14	13	65	66	85
Cladopus_	9	15	7	64	55	74
Cladopus_	6	2	3	42	34	67
Cladopus_	5	5	6	55	27	90
Cladopus_	21	17	11	100	56	94
Cladopus_	13	17	19	75	64	97
Cladopus_	8	4	4	36	48	68
Cladopus_	3	5	2	46	29	60
Cladopus_	12	15	6	58	51	87
Cladopus_	10	20	5	85	173	237
Cladopus_	9	8	10	60	50	63
Cladopus_	24	41	26	86	105	165
Cladopus_	6	12	11	69	50	64
Cladopus_	3	8	1	45	42	49
Cladopus_	11	21	13	64	68	91
Cladopus_	26	32	12	80	92	125
Cladopus_	2	18	5	53	82	69
Cladopus_	231	128	190	781	713	770
Cladopus_	25	14	21	82	73	105
Cladopus_	0	4	1	62	31	38
Cladopus_	1	2	4	33	36	54
Cladopus_	17	23	25	95	74	100
Cladopus_	6	12	4	46	42	72
Cladopus_	16	22	10	67	73	84
Cladopus_	3	26	16	80	75	100
Cladopus_	19	19	12	69	54	114
Cladopus_	9	6	1	47	37	57
Cladopus_	22	43	15	99	85	137
Cladopus_	2	9	8	52	41	55
Cladopus_	0	0	3	40	37	46

Cladopus_	106	83	189	762	621	941
Cladopus_	20	13	13	74	50	93
Cladopus_	10	11	15	56	68	65
Cladopus_	9	16	20	52	78	97
Cladopus_	4	8	1	32	45	52
Cladopus_	11	18	18	88	54	79
Cladopus_	8	10	10	59	41	64
Cladopus_	2	7	5	44	30	55
Cladopus_	25	16	14	76	66	89
Cladopus_	0	3	4	42	40	30
Cladopus_	11	6	7	54	31	81
Cladopus_	5	0	3	29	38	44
Cladopus_	29	17	18	73	75	104
Cladopus_	18	5	10	51	54	78
Cladopus_	6	0	3	30	28	59
Cladopus_	25	43	9	111	92	113
Cladopus_	1	6	3	25	43	46
Cladopus_	12	24	25	89	63	95
Cladopus_	16	28	16	90	62	87
Cladopus_	0	5	1	38	23	42
Cladopus_	4	1	4	31	26	51
Cladopus_	10	12	6	54	47	53
Cladopus_	26	17	10	67	67	88
Cladopus_	19	13	10	53	56	81
Cladopus_	24	12	27	74	75	101
Cladopus_	2	0	2	43	27	27
Cladopus_	3	9	2	37	29	55
Cladopus_	9	15	8	38	56	74
Cladopus_	0	0	1	35	22	46
Cladopus_	7	7	12	44	44	58
Cladopus_	7	10	2	43	29	63
Cladopus_	13	15	7	40	60	75
Cladopus_	10	15	7	45	58	59
Cladopus_	0	1	4	29	36	28
Cladopus_	5	12	12	39	45	80
Cladopus_	7	4	11	42	34	64
Cladopus_	0	1	7	34	34	34
Cladopus_	1	1	3	31	20	39
Cladopus_	2	0	2	25	27	36
Cladopus_	4	11	5	51	35	43
Cladopus_	10	1	4	41	39	38
Cladopus_	12	13	11	49	57	57
Cladopus_	14	16	15	61	46	74
Cladopus_	1	4	1	20	29	36
Cladopus_	2	3	6	26	30	39
Cladopus_	8	9	5	39	35	47
Cladopus_	4	4	7	32	29	43

Cladopus_	12	6	7	41	38	50
Cladopus_	2	6	5	27	30	41
Cladopus_	27	12	12	54	64	83
Cladopus_	5	2	6	33	22	45
Cladopus_	7	2	6	27	31	47
Cladopus_	6	7	2	33	33	35
Cladopus_	16	14	13	49	50	68
Cladopus_	5	25	10	55	46	83
Cladopus_	5	5	1	24	28	37
Cladopus_	2	4	8	31	23	48
Cladopus_	21	10	12	51	45	74
Cladopus_	15	7	15	59	43	54
Cladopus_	12	12	4	33	50	52
Cladopus_	8	17	10	43	34	82
Cladopus_	20	7	16	62	46	63
Cladopus_	6	15	12	44	36	63
Cladopus_	5	10	13	36	37	56
Cladopus_	11	8	10	30	38	63
Cladopus_	12	15	10	43	44	55
Cladopus_	5	3	4	18	27	40
Cladopus_	8	10	4	37	23	50
Cladopus_	22	20	6	79	43	60
Cladopus_	8	3	8	19	29	66
Cladopus_	24	8	11	45	58	60
Cladopus_	16	6	13	49	33	59
Cladopus_	8	12	5	28	43	42
Cladopus_	7	10	7	32	26	52
Cladopus_	5	10	1	29	26	36
Cladopus_	8	3	5	23	28	38
Cladopus_	2	18	2	89	92	110
Cladopus_	9	6	9	36	27	44
Cladopus_	2	15	6	26	31	64
Cladopus_	5	10	8	36	38	30
Cladopus_	8	9	7	45	16	55
Cladopus_	5	4	5	33	11	46
Cladopus_	2	15	12	36	51	40
Cladopus_	9	3	20	48	29	66
Cladopus_	10	6	7	29	31	36
Cladopus_	4	13	12	35	34	44
Cladopus_	7	3	10	31	22	40
Cladopus_	5	8	9	32	23	39
Cladopus_	4	17	6	42	24	43
Cladopus_	10	3	10	20	34	43
Cladopus_	13	2	15	71	16	51
Cladopus_	8	8	18	17	47	69
Cladopus_	11	2	5	15	28	38
