# Supplementary material for: A high-quality genome provides insights into the new taxonomic status and genomic characteristics of Cladopus chinensis (Podostemaceae)
Source: Hortic Res. 2020 Apr 1;7:46. doi: 10.1038/s41438-020-0269-5 (PMC7109043; doi:10.1038/s41438-020-0269-5)
Supplement: Supplementary file 19 — Table S23 GO enrichment of the 609 specific expressed genes in the shoot of C. chinensis [file 41438_2020_269_MOESM19_ESM.pdf]

GO ID (level1)	GO Term (level1)	GO ID (level2)	GO Term (level2)	number_of _out (All)
GO:0008150	Biological Process	GO:0050896	response to stimulus	145
GO:0008150	Biological Process	GO:0032502	developmental process	91
GO:0008150	Biological Process	GO:0065007	biological regulation	112
GO:0008150	Biological Process	GO:0050789	regulation of biological process	103
GO:0008150	Biological Process	GO:0032501	multicellular organismal process	80
GO:0008150	Biological Process	GO:0048511	rhythmic process	5
GO:0008150	Biological Process	GO:0044699	single-organism process	185
GO:0008150	Biological Process	GO:0022414	reproductive process	38
GO:0008150	Biological Process	GO:0000003	reproduction	38
GO:0008150	Biological Process	GO:0051179	localization	31
GO:0008150	Biological Process	GO:0023052	signaling	15
GO:0008150	Biological Process	GO:0048518	positive regulation of biological process	12
GO:0008150	Biological Process	GO:0051704	multi-organism process	24
GO:0008150	Biological Process	GO:0009987	cellular process	203
GO:0008150	Biological Process	GO:0002376	immune system process	4
GO:0008150	Biological Process	GO:0048519	negative regulation of biological process	12
GO:0008150	Biological Process	GO:0008152	metabolic process	179
GO:0008150	Biological Process	GO:0040007	growth	7
GO:0008150	Biological Process	GO:0071840	cellular component organization or biogenesis	22
GO:0003674	Molecular Function	GO:0001071	nucleic acid binding transcription factor activity	75
GO:0003674	Molecular Function	GO:0009055	electron carrier activity	6
GO:0003674	Molecular Function	GO:0005215	transporter activity	22
GO:0003674	Molecular Function	GO:0060089	molecular transducer activity	4
GO:0003674	Molecular Function	GO:0098772	molecular function regulator	3
GO:0003674	Molecular Function	GO:0004871	signal transducer activity	2
GO:0003674	Molecular Function	GO:0005198	structural molecule activity	3
GO:0003674	Molecular Function	GO:0005488	binding	41
GO:0003674	Molecular Function	GO:0003824	catalytic activity	76
GO:0005575	Cellular Component	GO:0016020	membrane	155
GO:0005575	Cellular Component	GO:0044420	extracellular matrix component	1
GO:0005575	Cellular Component	GO:0005576	extracellular region	21
GO:0005575	Cellular Component	GO:0031012	extracellular matrix	1
GO:0005575	Cellular Component	GO:0055044	symplast	21
GO:0005575	Cellular Component	GO:0030054	cell junction	21
GO:0005575	Cellular Component	GO:0044421	extracellular region part	1
GO:0005575	Cellular Component	GO:0044425	membrane part	21

GO:0005575	Cellular Component	GO:0005623	cell	286
GO:0005575	Cellular Component	GO:0044464	cell part	286
GO:0005575	Cellular Component	GO:0043226	organelle	190
GO:0005575	Cellular Component	GO:0032991	macromolecular complex	8
GO:0005575	Cellular Component	GO:0044422	organelle part	54
