# Supplementary material for: A high-quality genome provides insights into the new taxonomic status and genomic characteristics of Cladopus chinensis (Podostemaceae)
Source: Hortic Res. 2020 Apr 1;7:46. doi: 10.1038/s41438-020-0269-5 (PMC7109043; doi:10.1038/s41438-020-0269-5)
Supplement: Supplementary file 20 — Table S24 KEGG enrichment of the 609 specific expressed genes in the shoot of C. chinensis [file 41438_2020_269_MOESM20_ESM.pdf]

KEGG_A_class	KEGG_B_class	Pathway	out (148)	All (7219	Pvalue	Qvalue	Pathway ID
Metabolism	Carbohydrate metabolism	Pentose and glucuronate interconversions	15	147	2.71E-07	4.27E-05	ko00040
Metabolism	Lipid metabolism	Fatty acid elongation	5	28	0.000227329	1.46E-02	ko00062
Cellular Processes	Cellular community - prokaryotes	Quorum sensing	10	128	0.000277802	1.46E-02	ko02024
Organismal Systems	Environmental adaptation	Plant-pathogen interaction	15	282	0.000631297	2.49E-02	ko04626
Environmental Information Processing	Signal transduction	Plant hormone signal transduction	12	205	0.000980484	2.79E-02	ko04075
Organismal Systems	Environmental adaptation	Circadian rhythm - plant	6	59	0.001235264	2.79E-02	ko04712
Environmental Information Processing	Signaling molecules and interaction	Neuroactive ligand-receptor interaction	2	3	0.001235689	2.79E-02	ko04080
Human Diseases	Cancers	Choline metabolism in cancer	6	66	0.002215283	4.38E-02	ko05231
Cellular Processes	Cell growth and death	Ferroptosis	4	33	0.004371677	7.67E-02	ko04216
Metabolism	Metabolism of terpenoids and polyketides	Diterpenoid biosynthesis	4	35	0.005419775	7.90E-02	ko00904
Human Diseases	Substance dependence	Cocaine addiction	2	6	0.005932606	7.90E-02	ko05030
Cellular Processes	Transport and catabolism	Mitophagy - yeast	4	36	0.006001713	7.90E-02	ko04139
Metabolism	Energy metabolism	Nitrogen metabolism	5	61	0.008011056	9.25E-02	ko00910

Human Diseases	Substance dependence	Nicotine addiction	2	7	0.008194357	9.25E-02	ko05033
Organismal Systems	Immune system	Toll and Imd signaling pathway	5	64	0.009778354	1.03E-01	ko04624
Metabolism	Lipid metabolism	Linoleic acid metabolism	2	9	0.01367444	1.32E-01	ko00591
Cellular Processes	Cellular community - eukaryotes	Focal adhesion	4	46	0.01420602	1.32E-01	ko04510
Environmental Information Processing	Signal transduction	Phosphatidylinositol signaling system	5	72	0.01576127	1.38E-01	ko04070
Organismal Systems	Endocrine system	Adipocytokine signaling pathway	4	53	0.02284817	1.90E-01	ko04920
Human Diseases	Substance dependence	Amphetamine addiction	3	31	0.02495313	1.97E-01	ko05031
Metabolism	Lipid metabolism	alpha-Linolenic acid metabolism	3	33	0.02940662	2.21E-01	ko00592
Environmental Information Processing	Signal transduction	MAPK signaling pathway - plant	5	86	0.03130574	2.22E-01	ko04016
Environmental Information Processing	Signal transduction	cAMP signaling pathway	4	59	0.03234525	2.22E-01	ko04024
Metabolism	Global and overview maps	Biosynthesis of secondary metabolites	37	1358	0.03615787	2.38E-01	ko01110
Metabolism	Amino acid metabolism	Arginine and proline metabolism	4	72	0.05980615	3.53E-01	ko00330
Organismal Systems	Nervous system	Long-term potentiation	3	44	0.06075033	3.53E-01	ko04720

Metabolism	Carbohydrate metabolism	Inositol phosphate metabolism	4	73	0.06230803	3.53E-01	ko00562
Metabolism	Lipid metabolism	Fatty acid degradation	3	45	0.06414851	3.53E-01	ko00071
Environmenta l Information Processing	Signal transduction	Phospholipase D signaling pathway	4	74	0.06486456	3.53E-01	ko04072
Human Diseases	Infectious diseases	Measles	4	77	0.07285863	3.65E-01	ko05162
Metabolism	Metabolism of cofactors and vitamins	Retinol metabolism	2	22	0.07382498	3.65E-01	ko00830
Organismal Systems	Nervous system	Glutamatergic synapse	3	48	0.07485434	3.65E-01	ko04724
Cellular Processes	Cell motility	Regulation of actin cytoskeleton	4	79	0.07845451	3.65E-01	ko04810
Organismal Systems	Endocrine system	PPAR signaling pathway	3	49	0.07858844	3.65E-01	ko03320
Metabolism	Biosynthesis of other secondary metabolites	Isoquinoline alkaloid biosynthesis	2	24	0.08592304	3.88E-01	ko00950
Organismal Systems	Environmental adaptation	Circadian entrainment	2	25	0.09218105	4.05E-01	ko04713
Environmenta l Information Processing	Signal transduction	MAPK signaling pathway - yeast	3	54	0.09842893	4.20E-01	ko04011
Organismal Systems	Immune system	Fc gamma R-mediated phagocytosis	4	87	0.1028873	4.26E-01	ko04666
Metabolism	Lipid metabolism	Glycerophosphol ipid metabolism	5	122	0.1051647	4.26E-01	ko00564

Metabolism	Lipid metabolism	Fatty acid biosynthesis	3	58	0.1156044	4.35E-01	ko00061
Environmental Information Processing	Signal transduction	Ras signaling pathway	3	58	0.1156044	4.35E-01	ko04014
Environmental Information Processing	Signal transduction	NF-kappa B signaling pathway	3	58	0.1156044	4.35E-01	ko04064
Organismal Systems	Excretory system	Vasopressin-regulated water reabsorption	2	29	0.1184208	4.35E-01	ko04962
Human Diseases	Infectious diseases	Leishmaniasis	3	60	0.124588	4.40E-01	ko05140
Human Diseases	Neurodegenerative diseases	Amyotrophic lateral sclerosis (ALS)	2	30	0.1252456	4.40E-01	ko05014
Metabolism	Global and overview maps	Fatty acid metabolism	4	98	0.141394	4.78E-01	ko01212
Metabolism	Amino acid metabolism	Glycine, serine and threonine metabolism	4	99	0.1451487	4.78E-01	ko00260
Environmental Information Processing	Signal transduction	MAPK signaling pathway	4	99	0.1451487	4.78E-01	ko04010
Environmental Information Processing	Signal transduction	Calcium signaling pathway	2	35	0.1606207	5.10E-01	ko04020
Organismal Systems	Immune system	Toll-like receptor signaling pathway	3	68	0.1628254	5.10E-01	ko04620
Human Diseases	Infectious diseases	Toxoplasmosis	4	104	0.1644914	5.10E-01	ko05145

Metabolism	Lipid metabolism	Cutin, suberine and wax biosynthesis	2	36	0.1679031	5.10E-01	ko00073
Environmental Information Processing	Signal transduction	Rap1 signaling pathway	2	38	0.1826301	5.44E-01	ko04015
Metabolism	Lipid metabolism	Steroid hormone biosynthesis	1	10	0.1872046	5.48E-01	ko00140
Human Diseases	Infectious diseases	Chagas disease (American trypanosomiasis)	3	74	0.1935164	5.56E-01	ko05142
Metabolism	Amino acid metabolism	Phenylalanine metabolism	2	42	0.2125908	6.00E-01	ko00360
Human Diseases	Infectious diseases	Pertussis	3	81	0.230941	6.40E-01	ko05133
Metabolism	Global and overview maps	Metabolic pathways	62	2797	0.2383355	6.49E-01	ko01100
Organismal Systems	Sensory system	Inflammatory mediator regulation of TRP channels	1	14	0.2519366	6.63E-01	ko04750
Human Diseases	Infectious diseases	Amoebiasis	1	14	0.2519366	6.63E-01	ko05146
Metabolism	Amino acid metabolism	Tyrosine metabolism	2	48	0.25826	6.69E-01	ko00350
Organismal Systems	Digestive system	Mineral absorption	1	15	0.2673028	6.73E-01	ko04978
Metabolism	Amino acid metabolism	Cysteine and methionine metabolism	4	129	0.2721762	6.73E-01	ko00270
Cellular Processes	Cell growth and death	p53 signaling pathway	2	50	0.2735516	6.73E-01	ko04115

Metabolism	Biosynthesis of other secondary metabolites	Phenylpropanoid biosynthesis	4	130	0.2767497	6.73E-01	ko00940
Cellular Processes	Transport and catabolism	Endocytosis	5	174	0.2853666	6.83E-01	ko04144
Human Diseases	Infectious diseases	Tuberculosis	4	137	0.3090814	7.29E-01	ko05152
Organismal Systems	Environmental adaptation	Thermogenesis	6	227	0.3218692	7.46E-01	ko04714
Metabolism	Biosynthesis of other secondary metabolites	Tropane, piperidine and pyridine alkaloid biosynthesis	1	19	0.3256949	7.46E-01	ko00960
Organismal Systems	Nervous system	Neurotrophin signaling pathway	3	103	0.3539929	7.99E-01	ko04722
Human Diseases	Substance dependence	Alcoholism	3	104	0.3596217	8.00E-01	ko05034
Cellular Processes	Transport and catabolism	Peroxisome	3	107	0.3764642	8.21E-01	ko04146
Organismal Systems	Immune system	Platelet activation	1	23	0.3794625	8.21E-01	ko04611
Organismal Systems	Digestive system	Bile secretion	1	24	0.3922251	8.37E-01	ko04976
Metabolism	Lipid metabolism	Steroid biosynthesis	1	27	0.4289694	9.04E-01	ko00100
Human Diseases	Infectious diseases	Influenza A	2	74	0.4505264	9.27E-01	ko05164
Human Diseases	Drug resistance	Antifolate resistance	1	29	0.452231	9.27E-01	ko01523
Organismal Systems	Aging	Longevity regulating pathway - multiple species	2	75	0.4574239	9.27E-01	ko04213
Human Diseases	Endocrine and metabolic diseases	Insulin resistance	2	76	0.4642717	9.29E-01	ko04931

Metabolism	Lipid metabolism	Ether lipid metabolism	1	31	0.4745511	9.37E-01	ko00565
Human Diseases	Cardiovascular diseases	Hypertrophic cardiomyopathy (HCM)	1	32	0.48537	9.42E-01	ko05410
Metabolism	Lipid metabolism	Biosynthesis of unsaturated fatty acids	1	33	0.4959677	9.42E-01	ko01040
Human Diseases	Infectious diseases	Epstein-Barr virus infection	4	180	0.5070955	9.42E-01	ko05169
Cellular Processes	Transport and catabolism	Autophagy - other eukaryotes	1	35	0.516517	9.42E-01	ko04136
Organismal Systems	Circulatory system	Vascular smooth muscle contraction	1	35	0.516517	9.42E-01	ko04270
Metabolism	Amino acid metabolism	Alanine, aspartate and glutamate metabolism	2	84	0.5171735	9.42E-01	ko00250
Metabolism	Lipid metabolism	Glycerolipid metabolism	2	85	0.5235415	9.42E-01	ko00561
Environmental Information Processing	Signal transduction	Apelin signaling pathway	2	86	0.5298535	9.42E-01	ko04371
Human Diseases	Neurodegenerative diseases	Prion diseases	1	37	0.536234	9.42E-01	ko05020
Organismal Systems	Endocrine system	Oxytocin signaling pathway	2	90	0.5545335	9.42E-01	ko04921
Environmental Information Processing	Signal transduction	Hippo signaling pathway	1	39	0.555152	9.42E-01	ko04390
Metabolism	Metabolism of other amino acids	beta-Alanine metabolism	1	40	0.5643216	9.42E-01	ko00410

Metabolism	Metabolism of cofactors and vitamins	One carbon pool by folate	1	41	0.5733034	9.42E-01	ko00670
Organismal Systems	Nervous system	Dopaminergic synapse	1	41	0.5733034	9.42E-01	ko04728
Organismal Systems	Endocrine system	GnRH signaling pathway	1	41	0.5733034	9.42E-01	ko04912
Organismal Systems	Endocrine system	Parathyroid hormone synthesis, secretion and action	1	42	0.5821013	9.42E-01	ko04928
Cellular Processes	Transport and catabolism	Autophagy - animal	2	96	0.5898221	9.42E-01	ko04140
Human Diseases	Cancers	Pancreatic cancer	1	43	0.590719	9.42E-01	ko05212
Organismal Systems	Circulatory system	Adrenergic signaling in cardiomyocytes	1	46	0.6155271	9.42E-01	ko04261
Human Diseases	Cancers	Proteoglycans in cancer	1	46	0.6155271	9.42E-01	ko05205
Metabolism	Carbohydrate metabolism	Ascorbate and aldarate metabolism	1	47	0.6234599	9.42E-01	ko00053
Metabolism	Metabolism of other amino acids	Cyanoamino acid metabolism	1	47	0.6234599	9.42E-01	ko00460
Metabolism	Metabolism of terpenoids and polyketides	Carotenoid biosynthesis	1	47	0.6234599	9.42E-01	ko00906
Organismal Systems	Environmental adaptation	Circadian rhythm	1	47	0.6234599	9.42E-01	ko04710
Human Diseases	Cancers	Gastric cancer	1	48	0.6312301	9.42E-01	ko05226
Organismal Systems	Endocrine system	Estrogen signaling pathway	1	51	0.6535979	9.42E-01	ko04915

Environmental Information Processing	Signal transduction	cGMP - PKG signaling pathway	1	53	0.6677558	9.42E-01	ko04022
Human Diseases	Neurodegenerative diseases	Huntington disease	3	168	0.6747508	9.42E-01	ko05016
Metabolism	Amino acid metabolism	Valine, leucine and isoleucine biosynthesis	1	55	0.6813387	9.42E-01	ko00290
Human Diseases	Cancers	Transcriptional misregulation in cancers	1	56	0.6879219	9.42E-01	ko05202
Environmental Information Processing	Membrane transport	ABC transporters	1	57	0.69437	9.42E-01	ko02010
Human Diseases	Infectious diseases	Kaposi sarcoma-associated herpesvirus infection	1	57	0.69437	9.42E-01	ko05167
Organismal Systems	Endocrine system	Insulin signaling pathway	2	117	0.6968931	9.42E-01	ko04910
Organismal Systems	Immune system	Antigen processing and presentation	1	60	0.7129309	9.42E-01	ko04612
Metabolism	Carbohydrate metabolism	Glyoxylate and dicarboxylate metabolism	2	122	0.7187311	9.42E-01	ko00630
Organismal Systems	Endocrine system	Thyroid hormone signaling pathway	1	61	0.7188655	9.42E-01	ko04919
Cellular Processes	Transport and catabolism	Autophagy - yeast	1	62	0.7246783	9.42E-01	ko04138
Metabolism	Energy metabolism	Oxidative phosphorylation	4	238	0.7251057	9.42E-01	ko00190

Cellular Processes	Transport and catabolism	Phagosome	1	63	0.7303717	9.42E-01	ko04145
Metabolism	Energy metabolism	Photosynthesis	3	186	0.740025	9.42E-01	ko00195
Metabolism	Carbohydrate metabolism	Starch and sucrose metabolism	3	186	0.740025	9.42E-01	ko00500
Cellular Processes	Cell growth and death	Necroptosis	1	65	0.74141	9.42E-01	ko04217
Genetic Information Processing	Folding, sorting and degradation	Ubiquitin mediated proteolysis	2	128	0.7431873	9.42E-01	ko04120
Human Diseases	Infectious diseases	Legionellosis	1	67	0.7519993	9.42E-01	ko05134
Human Diseases	Cancers	MicroRNAs in cancer	1	67	0.7519993	9.42E-01	ko05206
Organismal Systems	Aging	Longevity regulating pathway - mammal	1	68	0.7571313	9.42E-01	ko04211
Human Diseases	Neurodegenerative diseases	Alzheimer disease	2	135	0.7694139	9.42E-01	ko05010
Metabolism	Amino acid metabolism	Phenylalanine, tyrosine and tryptophan biosynthesis	1	71	0.7719029	9.42E-01	ko00400
Genetic Information Processing	Translation	Ribosome	9	535	0.7781375	9.42E-01	ko03010
Metabolism	Metabolism of terpenoids and polyketides	Terpenoid backbone biosynthesis	1	73	0.7812514	9.42E-01	ko00900
Cellular Processes	Cellular community - eukaryotes	Tight junction	1	73	0.7812514	9.42E-01	ko04530

Environmental Information Processing	Signal transduction	Sphingolipid signaling pathway	1	80	0.8110726	9.65E-01	ko04071
Human Diseases	Infectious diseases	Herpes simplex infection	1	81	0.8149893	9.65E-01	ko05168
Cellular Processes	Cell growth and death	Meiosis - yeast	1	82	0.8188254	9.65E-01	ko04113
Environmental Information Processing	Signal transduction	mTOR signaling pathway	1	85	0.8298658	9.68E-01	ko04150
Metabolism	Energy metabolism	Methane metabolism	1	86	0.8333954	9.68E-01	ko00680
Metabolism	Carbohydrate metabolism	Fructose and mannose metabolism	1	89	0.8435535	9.73E-01	ko00051
Metabolism	Carbohydrate metabolism	Amino sugar and nucleotide sugar metabolism	2	168	0.8639865	9.89E-01	ko00520
Environmental Information Processing	Signal transduction	FoxO signaling pathway	1	98	0.8704814	9.89E-01	ko04068
Organismal Systems	Endocrine system	Glucagon signaling pathway	1	109	0.8972158	9.94E-01	ko04922
Cellular Processes	Cell growth and death	Cellular senescence	1	110	0.8993554	9.94E-01	ko04218
Human Diseases	Cardiovascular diseases	Fluid shear stress and atherosclerosis	1	111	0.9014506	9.94E-01	ko05418
Environmental Information Processing	Signal transduction	PI3K-Akt signaling pathway	1	113	0.9055121	9.94E-01	ko04151

Environmental Information Processing	Signal transduction	AMPK signaling pathway	1	118	0.914953	9.94E-01	ko04152
Human Diseases	Endocrine and metabolic diseases	Non-alcoholic fatty liver disease (NAFLD)	1	120	0.9184614	9.94E-01	ko04932
Cellular Processes	Cell growth and death	Oocyte meiosis	1	121	0.9201613	9.94E-01	ko04114
Genetic Information Processing	Transcription	Spliceosome	2	207	0.9295612	9.94E-01	ko03040
Genetic Information Processing	Translation	mRNA surveillance pathway	1	128	0.9311145	9.94E-01	ko03015
Genetic Information Processing	Folding, sorting and degradation	RNA degradation	1	158	0.9634617	9.99E-01	ko03018
Human Diseases	Cancers	Pathways in cancer	1	159	0.9642275	9.99E-01	ko05200
Metabolism	Nucleotide metabolism	Purine metabolism	2	249	0.9663954	9.99E-01	ko00230
Metabolism	Nucleotide metabolism	Pyrimidine metabolism	1	177	0.9755809	9.99E-01	ko00240
Genetic Information Processing	Translation	RNA transport	1	201	0.9853455	9.99E-01	ko03013
Metabolism	Global and overview maps	Biosynthesis of amino acids	3	379	0.9861326	9.99E-01	ko01230
Metabolism	Global and overview maps	Carbon metabolism	3	402	0.9906422	9.99E-01	ko01200
Metabolism	Global and overview maps	Microbial metabolism in diverse environments	5	554	0.9908733	9.99E-01	ko01120

Genetic Information Processing	Folding, sorting and degradation	Protein processing in endoplasmic reticulum	1	248	0.9946363	9.99E-01	ko04141
Metabolism	Global and overview maps	Biosynthesis of antibiotics	5	667	0.9985107	9.99E-01	ko01130
